# Genome-Wide Identification of LRR-RLK Family in *Saccharum* and Expression Analysis in Response to Biotic and Abiotic Stress

**DOI:** 10.3390/cimb43030116

**Published:** 2021-10-18

**Authors:** Wei Cheng, Zhoutao Wang, Fu Xu, Waqar Ahmad, Guilong Lu, Yachun Su, Liping Xu

**Affiliations:** Research Centre of National Sugarcane Engineering Technology, Key Laboratory of Sugarcane Biology and Genetic Breeding, Ministry of Agriculture, Fujian Agriculture and Forestry University, Fuzhou 350002, China; 2150101004@fafu.edu.cn (W.C.); 2170102010@fafu.edu.cn (Z.W.); 2200101003@fafu.edu.cn (F.X.); wahmadhu@gmail.com (W.A.); 2190102004@fafu.edu.cn.com (G.L.); 000q814030@fafu.edu.cn (Y.S.)

**Keywords:** LRR-RLK gene family, bioinformatics, molecular evolution, stress response, gene expression analysis, *Saccharum*

## Abstract

The leucine-rich repeat receptor-like protein kinase (LRR-RLK) gene family is the largest family of the receptor-like protein kinases (RLKs) superfamily in higher plants, which is involved in regulating the plant growth and development, stress responses, signal transduction and so on. However, no comprehensive analyses of LRR-RLKs have been reported in sugarcane. Here, we performed a comprehensive analysis of the LRR-RLK gene family in sugarcane ancestor species *Saccharum spontaneum*. A total of 437 *LRR-RLK* genes were identified and categorized into 14 groups based on a maximum likelihood phylogenetic tree. The chromosome location showed an uneven distribution on all 32 chromosomes in sugarcane. Subsequently, the exon–intron organization structure and conserved motif arrangement were relatively conserved among the same groups or subgroups and between *Arabidopsis* and *S. spontaneum* genomes. Furthermore, the promoter sequences analyses showed that sugarcane LRR-RLK genes (*SsLRR-RLKs*) were strongly regulated by various environmental stimuli, phytohormonal factors and transcription factors (TFs). Eventually, the expression profiles of *SsLRR-RLK* genes at different stresses were analyzed based on RNA-seq data, suggesting their potential roles in the regulation of sugarcane responses to diverse abiotic and biotic stress. Overall, the findings provide insight into the potential functional roles and lay the foundation for further functional study.

## 1. Introduction

During plant growth and development, plant cells are capable of sensing the stimulation of biotic and abiotic factors through extracellular cell surface receptors. These receptors mediate cell-to-cell and cell-to-environment interactions by interacting with polysaccharides, proteins, lipids and other ligands, which trigger a series of signal transductions [1,2,3]. Receptor-like protein kinases (RLKs) are a class of protein kinases that are widespread in plants, which are the key components in a variety of signal recognition pathways [1]. In 1990, the first *RLK* gene of higher plant species was identified in maize (*Zea mays*), and, since then, more *RLK* genes have been isolated from other plant species [4,5]. According to the classification based on the N-terminal extracellular domains (ECD) amino acid sequences, the RLKs superfamily can be further classified into 17 subfamilies, including leucine-rich repeat receptor-like protein kinases (LRR-RLKs), pathogenesis-related protein 5-like receptor kinases (PR5K-RLKs), S-domain RLKs (S-RLKs) and epidermal growth factor-like RLKs (EGF-RLKs) [4,5,6]. Among all of these subfamilies, the major and largest group of plant RLKs is the LRR-RLKs subfamily. The structural features of the LRR-RLK gene family include an intracellular protein kinase catalytic domain (KD), a single-pass transmembrane domain (TM) and a typical leucine-rich repeat (LRR) domain [6,7,8]. In terms of their structural features, previous evidence shows that plant LRR-RLKs play an essential role in various important processes [9,10,11,12,13,14].

In plants, previous studies have testified that *LRR-RLKs* genes play an essential role in diverse processes during both the plant growth cycle and responses to multiple stresses [10,11,12,13]. In the previous work, the best characterized *LRR-RLKs* are *CLAVATA1* (*CLV1*) and *BRASSINOSTEROID INSENSITIVE1* (*BRI1*) in *Arabidopsis thaliana* [15,16,17]. BRs are a class of plant growth regulator substances in plants that promote plant growth, plant meristems, flowering time and vascular differentiation [18,19]. In addition, many other aspects are regulated by *LRR-RLKs*, such as extra sporogenous cells (EXS) promoting the germination of plant seeds [20], *HAESA (HAE)* promoting plant floral organ abscission [21], *CRINKLY4* (*cr4*) participating in epidermal cell differentiation [22], *PXY* (*phloem intercalated with xylem*) regulating the activity of procambium in plant vascular tissues [23], *Xa21* being an effective bacterial blight resistance gene [24] and receptor-like kinase1 (*RPK1*) regulating ABA-induced sensitivity but being unchanged in jasmonic acid- and ethylene-induced sensitivity [25,26]. In addition, *ERECTA* has been confirmed not only to regulate the *Arabidopsis* ovule development process but also to be involved in resistance to bacterial wilt disease in *Arabidopsis* [27,28,29]. 

With the rapid development of genome sequencing technology, it has become possible to identify a whole gene family using bioinformatics tools at the plant genome level. Previous reports suggested that the members, structural features and expression profiles at the transcriptional level of the LRR-RLK gene family have been isolated in plants, including *A. thaliana*, *Solanum lycopersicum*, *Glycine max* and other species [9,11,13,14], but no information is available in sugarcane species. In most of these species, it is well known that LRR-RLKs are the largest group of the transmembrane receptor-like kinase subfamily and perform a variety of biochemical functions. For LRR-RLKs, according to the results of the phylogenic tree and gene structure, approximately 225 identified *LRR-RLK* genes were grouped into 15 groups in *Arabidopsis* [14], 234 identified were classified into 10 groups in *S. lycopersicum* [13], 309 identified were grouped into five groups in *Oryza sativa* [30], 467 identified were grouped into 14 groups in *G. max* [11], 303 identified were categorized into 15 groups in *Brassica rapa* [31], 379 identified in *Populus trichocarpa* were categorized into 14 groups [10] and 298, 511, 515 and 317 identified in *Gossypium arboreum, G. barbadense, G. hirsutum* and *G. raimondii* were classified into 15 groups [12], respectively. Apparently, there are a large number of LRR-RLKs in plant species, but detailed studies on their biochemical functions have only been performed for a few genes. Therefore, comprehensive analyses of the evolution, gene structure and expression patterns of the LRR-RLK gene family are necessary.

Sugarcane (*Saccharum* spp. hybrids) is now an important sugar and biofuel crop in the world, particularly in tropical and subtropical countries, producing 80% of the world’s sugar and 40% of the world’s biofuel. Considering the important resource value of sugarcane *Saccharum* genus, genome sequencing has been widely concerned. However, the complexity of the genetic background in sugarcane blocks the decoding of the genome sequence of modern sugarcane hybrids. Fortunately, Ray Ming’s research team, for the first time, deciphered the genome of one accession of the sugarcane ancestor species (*S. spontaneum* L.) [32]. Genomic deciphering is a milestone in the basic biological research of sugarcane, which can also provide the genome data for identification of the interesting family genes. In the present study, we first performed genome-wide identification, characterization and a phylogenetic and gene structure analysis of *LRR-RLK* genes. Moreover, the expression pattern of sugarcane *LRR-RLK* genes in several stress response processes was carried out. Our findings provide a foundation for the further functional characterization of the SsLRR-RLK gene family in regulating networks during plant growth and development and stress defense. 

## 2. Materials and Methods

### 2.1. Arabidopsis LRR-RLKs and Saccharum spontaneum Genome Resources

All *Arabidopsis* LRR-RLKs sequences were obtained from TAIR database v10.0 (http://www.arabidopsis.org/, accessed on 21 April 2021) [33]. The classification of different AtLRR-RLKs and nomenclature of groups were known from the PlantsP server v.2011 *Arabidopsis* 2010 project (http://plantsp.genomics.purdue.edu/projects/lrr/Clouse2010.htm, accessed on 21 April 2021) [14]. Meanwhile, the detailed genome information of *S. spontaneum* was based on public database (http://www.life.illinois.edu/ming/downloads/Spontaneum_genome/, accessed on 21 April 2021) [32].

### 2.2. Identification of LRR-RLKs in Saccharum spontaneum Genome

Plant LRR-RLK gene family is featured with LRR domain, KD domain and TM domain, and the corresponding hidden Markov models (HMM) of LRR and HMM models of KD were downloaded from Pfam database version 32.0 (http://pfam.xfam.org/, accessed on 23 April 2021) [34] and used to run a blast search with an E-value less than 10^−10^ against the proteome in *S. spontaneum* by using HMMER v3.1 software [35]. After initial screening, the resulting hits were collected for further filtering. In order to ensure we got as close as possible to the overall LRR-RLKs, all *Arabidopsis* LRR-RLKs members (AtLRR-RLKs) reported by Shiu et al. [6] were obtained from TAIR database v10.0 and served as the query to run a similarity search (E-value < 1 × 10^−5^, identity > 50%) against the proteome in *S. spontaneum* by using BLAST+ v.2.6.0 [36]. These resulting hit sequences were then analyzed by Pfam database [34] and SMART (http://smart.embl-heidelberg.de, accessed on 23 April 2021) [37] to confirm the presence of LRR domain and KD domain and other characteristic domains. Furthermore, TMHMM server v.2.0 (http://www.cbs.dtu.dk/services/TMHMM/, accessed on 24 April 2021) [38] was devoted for TM domain prediction. Protein sequences that contained both LRR domain, KD domain and TM domain were considered as LRR-RLK genes.

LRR-RLK proteins in *S. spontaneum* and their key features, such as length of amino acids, isoelectric point (*p*I) and molecular weights, were analyzed using ProtParam online tool (http://au.expasy.org/tools/protparam.html, accessed on 24 April 2021) [39], the subcellular localization of SsLRR-RLKs was carried out by ProtComp version 9.0 software on SoftBerry online database (http://linux1.softberry.com/berry.phtml?topic=protcomppl&group=programs&subgroup=proloc, accessed on 24 April 2021) [40] and the result of signal peptides was calculated using Signalp v5.0 (http://www.cbs.dtu.dk/services/SignalP/, accessed on 24 April 2021) [41], respectively.

### 2.3. Multiple Sequence Alignments and Phylogenetic Tree Construction

The sequence of each SsLRR-RLK and previously reported AtLRR-RLK protein was selected for phylogenetic analysis. Multiple sequence comparison by log-expectation (MUSCLE) [42] was carried out a multiple sequence alignment of the complete amino acid sequences of identified SsLRR-RLKs and AtLRR-RLKs. Finally, an unrooted phylogenetic tree was generated for sugarcane/*Arabidopsis* together by FastTree 2 tool with default arguments using the maximum likelihood (ML) method [43].

### 2.4. The Analysis of the Chromosome Distribution, Gene Structure, and Conserved Motif of the Sugarcane LRR-RLK Genes

Chromosome locations of all *SsLRR-RLK* genes were mapped based on the physical position of sugarcane chromosomes. The figure of chromosomal distribution of the *SsLRR-RLK* genes was drawn at MG2C v2.0 (http://mg2c.iask.in/mg2c_v2.0/, accessed on 26 April 2021) [44]. The exon–intron structure distribution patterns of each *SsLRR-RLK* were determined using GSDS 2.0 (http://gsds.cbi.pku.edu.cn/, accessed on 26 April 2021) [45]. To reveal the structural divergence of individual *SsLRR-RLKs*, the conserved motifs in their encoded proteins were predicted with Multiple Em for Motif Elicitation (MEME) online program version 5.0.5 (http://meme-suite.org/tools/meme, accessed on 27 April 2021) [46]. The maximum number of motifs was set as 20. 

### 2.5. Promoter and Regulatory Analysis of the Sugarcane LRR-RLK Genes

The upstream 2000 bp sequence of each *SsLRR-RLK* gene translation start codon (ATG) was extracted from their genome sequence as a promoter region (Supplementary Material S3). In order to predict potential *cis*-acting regulatory elements, all promoter sequences of each *SsLRR-RLK* genes were submitted to PlantCARE online tool (http://bioinformatics.psb.ugent.be/webtools/plantcare/html/, accessed on 28 April 2021) [47]. In addition, transcription factor (TF) binding sites were predicted using the PlantTFDB v4.0 online tool (http://plantregmap.cbi.pku.edu.cn/binding_site_prediction.php, accessed on 28 April 2021) [48].

### 2.6. Expression Pattern Analysis of the Sugarcane LRR-RLK Genes

For a better understanding of the potential roles of sugarcane *LRR-RLK* expression pattern during different stress responses, the expression profiles of all *LRR-RLK* genes were carried out based on the RNA-seq data from five different datasets of sugarcane transcriptomes in response to different stress treatments, except for low nitrogen stress, which contained two different tissues i.e. leaves and roots, and all the other RNA-seq raw data were obtained from sugarcane leaves. The above five sets of transcriptomes were provided by our laboratory research team. Detailed information of the transcriptomes prepared are as follows: (1) leaf and root transcriptome in two sugarcane varieties, ROC22 (resistant) and Badila (susceptible), under low nitrogen condition [49], respectively; (2) leaf transcriptome in two sugarcane varieties, ROC22 (susceptible) and FN39 (resistant), under low temperature treatment (unpublished); (3) leaf transcriptome of two sugarcane varieties, FN12-047 (susceptible) and ROC22 (resistant), subjected to infection by sugarcane leaf blight disease (*Leptosphaeria taiwanensis*) at different stages (unpublished); (4) transcriptome data of two sugarcane varieties, YC05-179 (resistant) and ROC22 (susceptible), inoculated with sugarcane smut pathogen (*Sporisorium scitaminea* Syd.) at different time points (24, 48 and 120 h post-inoculation (hpi)) [50]. These transcriptome datasets were selected for further analysis. Quality assessment of raw RNA-Seq reads were processed using FastQC, and then the high-quality cleaned reads were aligned to the *S. spontaneum* genome using HISAT2. Following alignments, raw counts for each gene were normalized and derived into FPKM (fragments per kilobase of transcript per million fragments mapped) value [51]. The transcript abundance of each sugarcane *LRR-RLK* genes were calculated as FPKM. Finally, we used 1.0 FPKM threshold, which created a full list of *SsLRR-RLK* genes with significant expression level. In addition, the resulting expression data based on the log-transformed data using log2 (FPKM+offset) with an offset=1.0 were then used for generation of heatmap [51,52] by pheatmap one package of R [53]. Here, in order to better and more easily understand the gene family analysis process, a diagram (Supplementary Material S4) showing all of the steps developed (from the use of *Arabidopsis* and *S. spontaneum* genome resources to the qRT-PCR), and summarizing all of the tools used in the manuscript, was made

### 2.7. Plant Materials, RNA Extraction and qRT-PCR Analysis

The sugarcane variety FN12-047 (*Saccharum* spp. hybrids) was provided by the Key Laboratory of Sugarcane Biology and Genetic Breeding, Fujian Agriculture and Forestry University (Fuzhou, China). In this study, we selected the sugarcane maturity stage for sampling. The field was watched throughout the plant life cycle, especially after appearance of sugarcane leaf blight disease (SLB) symptoms, and five different disease development stages were defined according to the previous report [54]. The leaf samples that had no visible sign of the SLB symptom and without pathogenic spores or hyphae observed under the microscope were represented as control [54]; Supplementary Material S5 shows the details. Three biological replicates were performed for each infection stage and for control (healthy), and a total of 18 samples were obtained. For each sugarcane accession, three plants with similar growth vigor and disease severity were selected for sampling, and the leaf located at the same leaf position was collected [54]. The collected samples were immediately put into liquid nitrogen and stored at −80 °C for later use.

Total RNA of each sample was extracted using TRIzol Reagent (Invitrogen, Waltham, Massachusetts, USA) and RNase-free DNase (TaKaRa, Shimogyo-ku, Kyoto, Japan) was treated according to the manufacturer’s protocol. The quality of the RNA was tested by using NanoDrop-1000 and RNA integrity was checked by electrophoresis. First strand cDNA synthesis was performed using the Prime-ScriptTM RT Reagent Kit (TaKaRa, Shimogyo-ku, Kyoto, Japan). Plant LRR-RLK gene family is the largest family in the plant RLK superfamily. In this study, a total of 437 *LRR-RLK* genes were identified in sugarcane. According to the results of the expression pattern analysis of the *SsLRR-RLK* genes in the transcriptome dataset (leaf transcriptome of two sugarcane varieties, FN12-047 (susceptible) and ROC22 (resistant), subjected to infection by sugarcane leaf blight disease), on the one hand, based on the transcriptome data (leaf transcriptome of sugarcane varieties FN12-047 (susceptible) subjected to infection by sugarcane leaf blight disease) FPKM values of 437 *SsLRR-RLK* genes, we found that approximately 24% of *SsLRR-RLK* genes (106 out of 437) were not expressed, approximately 49% of *SsLRR-RLK* genes (213 out of 437) were down-regulated and the remaining 27% of *SsLRR-RLK* genes (118 out of 437) were up-regulated. On the other hand, based on the FPKM value of 437 *SsLRR-RLK* genes depending on transcriptome data (leaf transcriptome of ROC22 (resistant) subjected to infection by sugarcane leaf blight disease), we found that approximately 22% of *SsLRR-RLK* genes (95 out of 437) were not expressed, approximately 29% of *SsLRR-RLK* genes (126 out of 437) were down-regulated and the remaining 41% of *SsLRR-RLK* genes (216 out of 437) were up-regulated. The detailed data are shown in Supplementary Material Table S8. Among the up-regulated *SsLRR-RLK* genes, we selected nine SsLRR-RLK genes with significant expression levels (FPKM values ranging from 5.4 to 36.9) to both verify the accuracy of the transcription dataset through RT-qPCR experimental results and show the transcriptional response of *SsLRR-RLK* gene in different periods. These qRT-PCR primers designed by Primer Premier 6 are listed in Supplementary Material Table S1. Furthermore, as an internal control primer, GAPDH (glyceraldehyde-3-phosphate dehydrogenase) is unanimously recognized for its stability in academic research, such as in different sugarcane tissues and various adversity stress, and the primer sequence of GAPDH is universal [54]. Therefore, this housekeeping gene was used as the only internal control gene. The qRT-PCR amplification was performed using SYBR Green Master Mix Reagent (TaKaRa, Shimogyo-ku, Kyoto, Japan), according to the manufacturer’s instructions on an Applied Biosystems 7500 Real-Time PCR system. The standard qRT-PCR program refers to reports by Wang et al. [54]. Three technical replicates for each sample were performed and the relative expression level of each gene was calculated from the 2^−ΔΔCt^ value [55].

## 3. Results

### 3.1. Identification and Distribution of LRR-RLK Genes in Saccharum spontaneum

**Figure 1:**
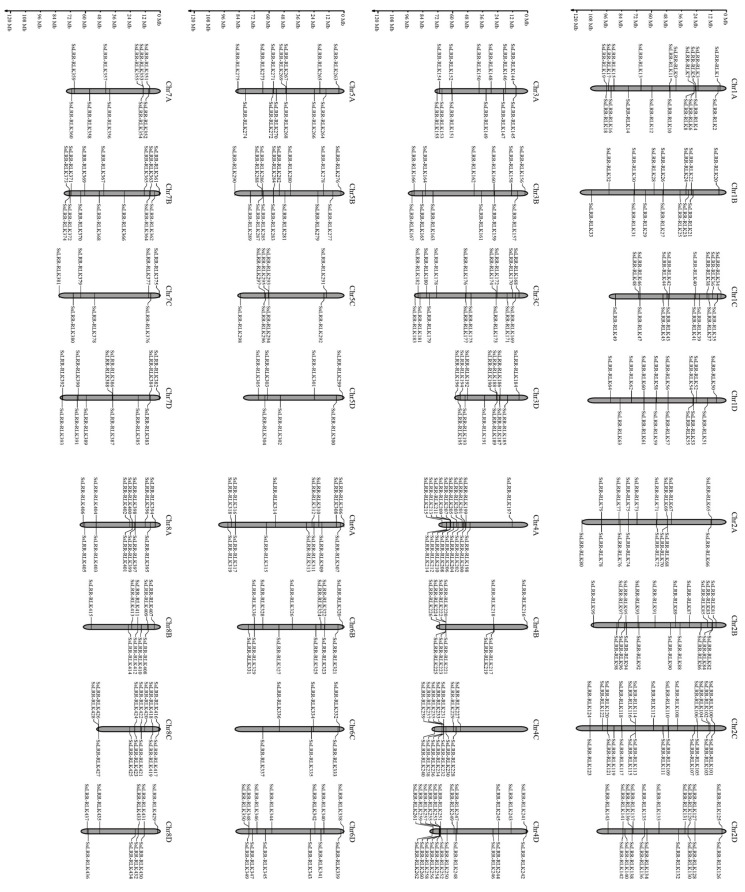
Chromosome distribution of LRR-RLK family in *S. spontaneum*. 437 SsLRR-RLK alleles were located in all 32 chromosomes. The figure was drawn using MG2C v2.0.

The LRR-RLK amino acid sequences are composed of 437 members, which were identified from the whole genome of one accession of sugarcane ancestor species *S. spontaneum*, and all contain LRR-, TM- and KD-domains simultaneously. They accounted for 1.23% of the whole coding genes in *S. spontaneum*. Detailed information of the sugarcane LRR-RLK family is listed in Supplementary Material Table S2 and the summarized information of each group or subgroup is shown in Table 1. The results showed that the encoded protein molecular weight (MW) of all predicted *LRR-RLKs* ranged from 39.00 to 349.15 kDa and that their *p*Is varied from 5.06 to 10.63. In addition, the number of amino acids (AA) in encoded proteins varies from 450 to 1750 AA for approximately 97.7% of the aforementioned proteins, except for ten with different lengths, including the SsLRR-RLK305 encoding protein with 436 AA, SsLRR-RLK353 encoding protein with 358 AA and SsLRR-RLK72/92/25/244/348/355/407/427 encoding proteins with 1775, 2069, 2678, 3171, 1829, 1930, 1830 and 2005 AA, respectively. Supplementary Material Table S2 provides the results of the number of TM by TMHMM server v.2.0. A total of 422 SsLRR-RLKs had at least one TM domain, while 15 proteins had more than three TM domains. Meanwhile, physical positions of SsLRR-RLKs were used to map them onto corresponding chromosomes of this species. Results indicated that 437 *LRR-RLK* genes could be mapped onto all 32 chromosomes, from chromosome 1A (Chr1A) to Chr8D (Figure 1). In spite of every chromosome containing a certain number of *SsLRR-RLK* genes, their distribution appeared to be uneven across different chromosomes. The distribution ratio for each chromosome ranged from 1.37% to 5.72%. The largest number of *SsLRR-RLK* genes was found on Chr2C (25 genes); on the contrary, just six members were found on Chr6C. In addition, 22 *SsLRR-RLK* genes were located on Chr4D, followed by 19 on Chr1A, 2B, 2D and 4A, and the least (seven) on Chr5D and Chr7C. The results indicate that the distribution pattern of *LRR-RLK* in *S. spontaneum* is unlike the other gene family, such as the potato PLD gene family [56] and the maize JHDM gene family [34], while it is similar with *LRR-RLK* gene families in other plant species, such as soybeans [11].

### 3.2. Signal Peptide and Subcellular Localization Analysis of the LRR-RLK Family Proteins

The signal peptide analysis of the 437 LRR-RLK proteins was carried out using SignalP 5.0 Server. A total of 117 (26.8%) of LRR-RLK proteins do not contain a signal peptide sequence. Subsequently, in order to predict the location of the above proteins, we performed a subcellular localization prediction. The results show that 89.5% (391/437) of LRR-RLK proteins are likely to be located at the plasma membrane, whereas the other 10.5% (46/437) are more likely to be located in the extracellular domain (shown in Supplementary Material Tables S3 and S4).

### 3.3. Phylogenetic Analysis of Sugarcane LRR-RLKs

**Figure 2:**
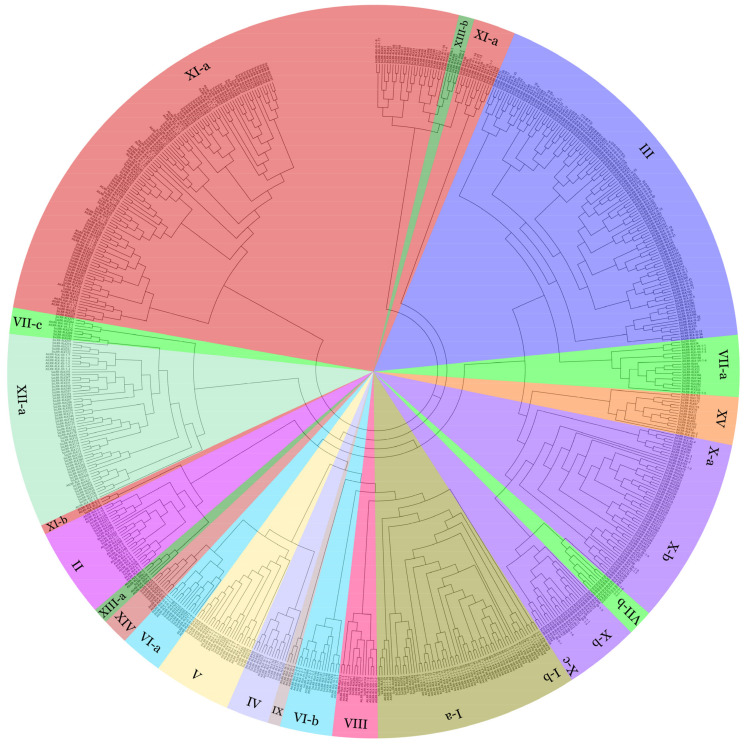
Phylogenetic tree of the LRR-RLK genes between *Arabidopsis* and *S. spontaneum*. The phylogenetic tree was generated using FastTree 2.0 using the maximum likelihood method. All sugarcane LRR-RLKs were classified into 14 distinct groups and different groups were shown by different color.

The 437 LRR-RLKs identified in this work and the previously reported 225 *A. thaliana* [57] LRR-RLKs were aligned (Additional data S1) for the maximum likelihood (ML) phylogenetic tree construction. The results revealed that sugarcane LRR-RLKs were classified into 14 groups and 21 distinct clades (Figure 2 and Additional data S2). It is noteworthy that not a single SsLRR-RLK protein is placed into the I-b and IX subfamily, which might be the result of gene loss during the evolutionary process of LRR-RLK family genes in *S. spontaneum*. In summary, group III, X-b, XI-a and XII show the largest number (75, 59, 124 and 51, respectively) of SsLRR-RLKs, whereas group X-c, XI-b, XIII-a and XIII-b contain only three, one, one and two in the phylogeny, respectively. Meanwhile, the majority of LRR-RLKs in *A. thaliana* is distributed in group I, III and XI-a, which contain 48, 46 and 33 LRR-RLKs, respectively. The phylogenetic analysis suggested that the 437 SsLRR-RLK proteins may have similar functions to their *Arabidopsis* orthologs, and provided insight into the evolution of gene family members and gene family diversity in sugarcane. The detailed classification of *Arabidopsis* and sugarcane LRR-RLKs is described in Supplementary Material Table S5.

### 3.4. Exon–Intron Organization and Conserved Motifs of SsLRR-RLKs

**Figure 3:**
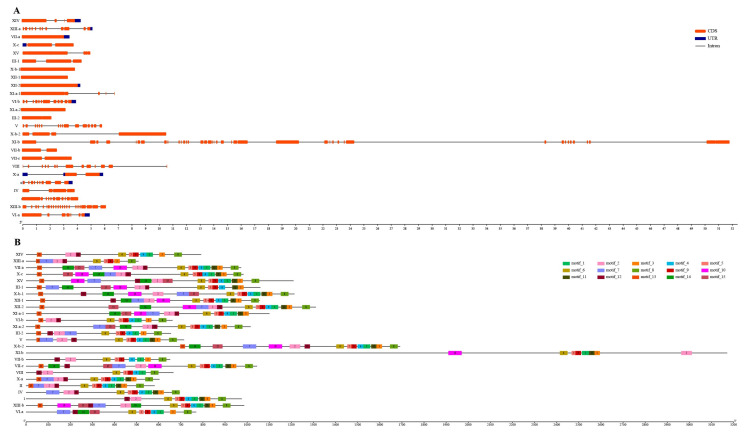
Representative exon–intron (**A**) and conserved motifs structure (**B**) of each *S. spontaneum* LRR-RLK subfamily. (**A**) Exon–intron structures of *SsLRR-RLKs*. Untranslated regions (UTRs), exons and introns are represented by blue boxes, red boxes and black lines, respectively. (**B**) The motif distribution of SsLRR-RLK proteins. The 15 predicted motifs are represented by distinct color boxes using the MEME program.

To identify the diversification of the *SsLRR-RLK* genes, the exon–intron organizations and conserved motifs were analyzed. Supplementary Material Figure S1 reveals the detailed information of each *SsLRR-RLK* gene and Figure 3 describes the representative exon–intron distributions in sugarcane. Among the *LRR-RLKs*, nearly one third (157 out of 437) contained only one intron, while 53 genes were without any introns. Two, three, four, five and six introns were found in 64, 48, 18, 11 and 9 genes, respectively. Furthermore, a total of 77 *SsLRR-RLKs* contained more than six introns, and, out of them, 19 had more than 15 introns. In terms of the results of exon–intron number and distribution, the same groups or subgroups have a very close organization, which strongly supports their evolutionary relationship. For instance, a large number of *SsLRR-RLKs* in groups VII-b, VII-c, X-c and XV contain zero, one, two and three introns, except for the gene *SsLRR-RLK82*, which contains six introns. Nevertheless, the members of groups I, III and XI exhibited a high changeability in the number of introns. Interestingly, the members of group XIII-b contained 24 introns, which was approximately twice as much as group XIII-a.

Meanwhile, the conserved motifs and diversification of all SsLRR-RLK proteins were generated from the results of the MEME motif analysis (Supplementary Material Figure S2). As exhibited in Figure 3, a total of 15 distinct conserved motifs were found: motifs 1 to 15, which are widely distributed across all subfamilies, except for SsLRR-RLK386/265/338. This specific motifs arrangement might contribute to the functional divergence of LRR-RLK genes in sugarcane. SsLRR-RLKs members within the same groups or subgroups were usually found to have shared a composition. For instance, the clustered SsLRR-RLK pairs, i.e. SsLRR-RLK81/101, SsLRR-RLK398/433 and SsLRR-RLK179/314, were demonstrated to have a highly similar motif distribution. It is noteworthy that differences within the group and subgroups were found for not only the number of motifs but also the types of motifs in one SsLRR-RLK protein (Supplementary Material Table S6).

### 3.5. A cis-Acting Regulatory Elements and TF Binding Sites Analysis of SsLRR-RLKs

The *cis*-acting regulatory elements of the promoter region (2000 bp sequence upstream of translation start site) among 437 *LRR-RLK* members were detected using PlantCARE, and the results indicated that a total of 128 putative *cis*-acting elements were widespread in *SsLRR-RLKs*. Detailed information of all genes is shown in Supplementary Material Table S7 and summarized in Table 2. Briefly, *cis*-acting regulatory elements can be mainly classified into seven major categories. By contrast, four types of *cis*-acting regulatory elements related to phytohormones, environmental stress, photoreactions and development were particularly abundant. The first type of photoreaction-related elements included G-Box, TCCC-motif, ACA-motif, Box 4, GT1-motif, LS7 and Sp1 etc., of which, SP1 and G-Box appeared to be the most widespread, with more than five copies. The phytohormone regulation-related *cis*-acting elements of the second type responds to auxin, abscisic acid (ABA), jasmonic acid (JA), gibberellins (GA), salicylic acid (SA) and ethylene (ETH). AuXRR-core and AuxRE are involved in auxin responsiveness, GARE-motif and P-box are considered to be gibberellin responsive elements involved in the regulation of gibberellin and other *cis*-elements participated in a specific stress response. The third type of widely distributed regulatory elements is associated with environmental stimuli, including abiotic and biotic stresses; of which, ARE, MYB, MYC, GC-motif, STRE, LTR and MBS were the most abundant. In particular, MYB and MYC were the most abundant, both having more than 15 copies in all subfamilies, except for X-c and VII-b. Finally, *cis*-acting elements of the fourth type, including CAT-box, O_2_-site, ACII, Myb-binding site, CCGTCC-box and CCGTCC-motif etc., are generally related to plant growth and development, which is either related to the cell cycle and cell proliferation response or required for tissue-specific expression. Overall, the *cis*-acting regulatory elements SP1, G-BOX, CCGTCC-box/motif, ABRE, CGTCA-motif, MYB, MYC and STRE are prominently distributed in these four categories. Captivatingly, two very specific *cis*-elements, OCT and JERE, could be prominently found in the X-c subfamily (containing *SsLRR-RLK66/84/104* genes), with 8.0 and 10.7 copies, respectively. Thus, considering the distribution of *cis*-acting regulatory elements in the promoter regions, we speculate that the expression of *SsLRR-RLKs* was extensively regulated, implying the important roles in plant meristem maintenance and organogenesis, as well as in mediating defense responses to environmental stresses.

In addition, transcription factors (TFs) play an essential role in many biological processes by regulating and mediating related target gene expression. To explore the potential regulation relationship among TFs and *SsLRR-RLKs*, TFs binding sites were predicted using PlantTFDB. As shown in Supplementary Material Figure S3, NAC, TALE, G2-like, WRKY, ERF and MYB were the most widely functioning TF family and could regulate the majority of *SsLRR-RLKs*. Meanwhile, the abundant TF families were concerned in various aspects of plant growth and development, stress defense and signal transduction, suggesting that *SsLRR-RLKs* with TF binding sites were considered to participate in these key processes by TF-mediated regulation.

### 3.6. Expression Profiling of SsLRR-RLK Genes Based on RNA-seq Datasets

**Figure 4:**
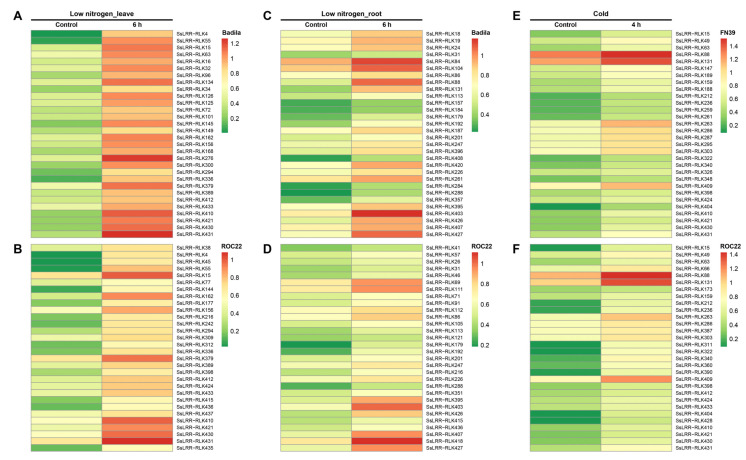
Heatmaps of the expression analysis of *SsLRR-RLKs* in response to diverse abiotic stress. The differential expression profiles in two different sugarcane varieties is shown by heatmap under (**A**,**B**) low nitrogen (leaf) stress, (**C**,**D**) low nitrogen (root) stress and (**E**,**F**) cold stress. Heatmap was generated based on FPKM values. The color scale represents expression values of each sample, with red showing high levels and green showing low levels of transcript abundance.

For a better understanding of the *SsLRR-RLKs* functions, the expression patterns of all *SsLRR-RLKs* in different abiotic stresses (i.e., cold and low nitrogen treatments) and biotic stresses (i.e., challenged by sugarcane leaf blight disease and sugarcane smut) were investigated based on RNA-seq datasets. The expression profiles of *SsLRR-RLK* genes under a low temperature are presented in Table S8 (Supplementary Material Table S8) and Figure 4E,F. Our results showed that 27.0% (118 out of 437) of genes were expressed in the relatively tolerant variety FN39 under cold stress, and 28.0% (33/118) genes among them were induced and shown to be highly expressed, such as *SsLRR-RLK88/131*/*263.* However, 72.0% (85/118) of genes were suppressed after cold stress, and *SsLRR-RLK169/197/246/266*/*420* among them was shown to be more strongly suppressed. By contrast, the expression level of 27.9% (122 out of 437) of *LRR-RLK* genes in the variety ROC22 was expressed, and 51.6% (63 out of 122) with a high expression, such as *SsLRR-RLK88/131*/263/*409*. In contrast, 48.4% of genes showed a low expression, such as *SsLRR-RLK169/197/246/266/396*/*420*. 

Subsequently, we further investigated the expression profiles of *SsLRR-RLKs* in root and leaf transcriptome in two sugarcane varieties, ROC22 and Badila, under low nitrogen stress, respectively (Supplementary Material Table S8). The results indicated that 51.5% (225 out of 437) and 53.3% (233 out of 437) of genes were expressed, while 33.3% (75 out of 225) and 62.7% (146 out of 233) of genes were shown to be highly expressed in the roots in the varieties Badila and ROC22 under a low nitrogen stress. Besides, 66.7% (150 out of 225) and 37.3% (87 out of 233) of genes were shown to have a low expression in Badila and ROC22, respectively. Among these genes, *SsLRR-RLK84/88/104*/*403* and *SsLRR-RLK69/111/403/407/418*/*427* had the highest expression level, whereas *SsLRR-RLK161/286/394/417* and *SsLRR-RLK12/161/295* had the weakest expression in roots in Badila and ROC22, respectively (Figure 4CD). In addition, The RNA-seq datasets of the leaves transcriptome in ROC22 and Badila under low nitrogen stress were used to generate a heatmap (Supplementary Material Table S8 and Figure 4AB). It suggested that 52.9% (231 out of 437) and 46.9% (205 out of 437) of genes were expressed, and 77.9% (180 out of 231) and 73.7% (151 out of 205) of genes had a high expression in Badila and ROC22, respectively. However, 22.1% (51 out of 231) and 26.3% (54 out of 205) of genes were suppressed in Badila and ROC22, respectively. Among these genes, *SsLRR-RLK15/276/379/410/431* and *SsLRR-RLK15/379/410/431* were strongly expressed, whereas *SsLRR-RLK141/169/396/420* and *SsLRR-RLK169/420* showed the weakest expression in Badila and ROC22, respectively.

**Figure 5:**
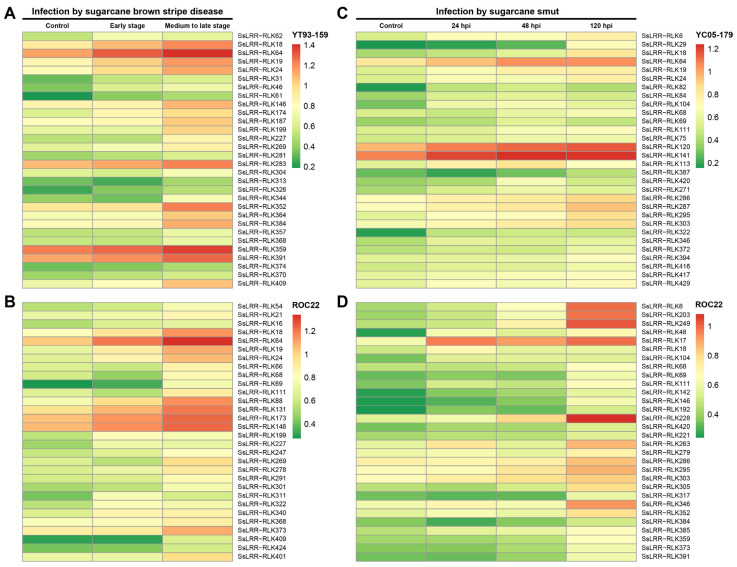
Heatmaps of the expression analysis of *SsLRR-RLKs* under different biotic stresses. The differential expression patterns in two different sugarcane varieties were shown by heatmap under (**A**,**B**) infection by sugarcane leaf blight disease and (**C**,**D**) infection by sugarcane smut. Heatmap was generated based on FPKM values. The color scale represents expression values of each sample, with red showing high levels and green showing low levels of transcript abundance.

Additionally, the datasets obtained from leaves of FN12-047 and ROC22 subjected to pathogenic infection of sugarcane leaf blight disease at two disease-developed stages were used for analysis of the expression profiles. As shown in Figure 5AB, expression heatmaps of the *SsLRR-RLK* genes responsive to sugarcane leaf blight disease in susceptible variety FN12-047 and resistant variety ROC22 revealed that 34.1% (149 out of 437) and 34.6% (151 out of 437) of genes were expressed, and 64 out of 149 genes and 105 out of 151 genes were shown to be highly expressed at early stages of disease development in FN12-047 and ROC22, respectively. Among them, *SsLRR-RLK64/359/391* and *SsLRR-RLK64/146/173* showed significant expression, particularly in FN12-047 and ROC22, respectively. In contrast, 34.3% (150 out of 437) and 35.7% (156 out of 437) of genes showed the weakest expression, whereas 86 out of 150 and 114 out of 156 genes were highly expressed at medium to late stages of the disease development. Moreover, five (*SsLRR-RLK18/64/352/359/391*) and six *LRR-RLKs* (*SsLRR-RLK18/64/88/131/146/173*) were strongly expressed at medium to late stages in FN12-047 and ROC22, respectively. Furthermore, expression profiles of the *SsLRR-RLKs* response to the varieties YC05-179 (resistant) and ROC22 (susceptible) challenged by smut pathogen *Sporisorium scitaminea* in the samples collected at different time points (24, 48 and 120 h post-inoculation (hpi)) were investigated, and the heatmap was constructed (Figure 5CD). Generally, for both investigated varieties, approximately half of the *LRR-RLK* genes appeared to be expressed across all three time points, and those significantly induced in variety YC05-179 were *SsLRR-RLK120/141* at 24 hpi, *SsLRR-RLK64/120/141/* at 48 hpi and *SsLRR-RLK64/120/141* at 120 hpi. By contrast, those strongly induced in variety ROC22 were *SsLRR-RLK17* at 24 hpi, *SsLRR-RLK17/228* at 48 hpi and *SsLRR-RLK6/17/203/228/246/249/346* at 120 hpi.

Overall, many *SsLRR-RLK* genes were strongly induced or suppressed by different stress treatments. For example, *SsLRR-RLK88* significantly responded to the stresses of the cold, low nitrogen and sugarcane leaf blight disease infection. *SsLRR-RLK141* was obviously induced by all of the investigated stress treatments. In contrast, some *LRR-RLK* genes were simultaneously induced in both varieties with a different tolerance/resistance by one treatment. For example, *SsLRR-RLK88* and *SsLRR-RLK131* were induced by the cold treatment and *SsLRR-RLK18/64/146* was induced by the infection of sugarcane leaf blight disease. Most interestingly, the expression profiles for some *SsLRR-RLK* genes, such as *SsLRR-RLK131*, was significantly expressed in response to the infection of sugarcane leaf blight disease, whereas it was strongly repressed under cold stress. This indicates the potential roles in multiple biotic and abiotic stress responses of these differentially expressed *SsLRR-RLK* genes.

### 3.7. Expression Analysis of SsLRR-RLK Genes by Quantitative Real-Time PCR

**Figure 6:**
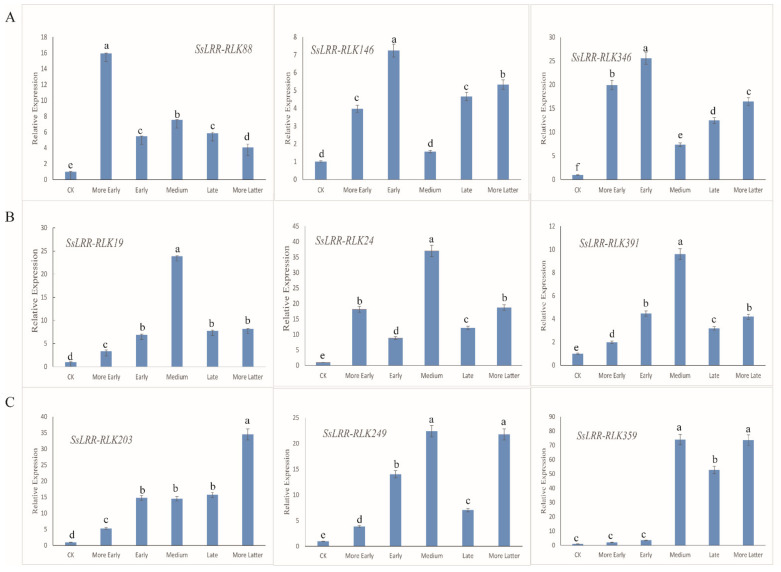
Relative expression levels of nine selected *LRR-RLK* genes were analyzed using quantitative real-time PCR (qRT-PCR). Among them, three obvious periods were discovered, namely the early, medium and late periods. (**A**) contains three highly expressed genes that respond to early period (*SsLRR-RLK88/146/346*) that were identified. (**B**) contains three highly expressed genes that respond to medium period (*SsLRR-RLK19/24/391*) that were identified. (**C**) contains three highly expressed genes that respond to late period (*SsLRR-RLK203/249/359*) that were identified. X-axes show different stages and y-axes show relative expression level of each *LRR-RLK* gene. Bars represent the standard deviation (SD) (*n* = 3) of three technical replicates. Different lowercase letters above the bars in the figure indicate a significant difference (*p* < 0.05). Lowercase letters reflect the 5% significance level. As shown in the chart, a, b, c, d, e and f are different from each other, and they are all significantly different from each other. In addition, the same lowercase letter means no significantly different.

According to the expression profiling of *SsLRR-RLK genes*, an overview of expressed *SsLRR-RLKs* under sugarcane leaf blight disease stress (SLB) is known. To further investigate their physiological functions, we first selected and analyzed nine *SsLRR-RLK* genes by qRT-PCR based on the expression analysis of *SsLRR-RLK* genes. As shown in Figure 6, the results showed that the expression pattern of each *LRR-RLK* gene is differentially expressed in the leaves. Meanwhile, on the basis of their expression levels, these nine *SsLRR-RLKs* were clearly divided into three groups (A–C). Among them, *SsLRR-RLK88/146* and *SsLRR-RLK346* genes of group A were significantly expressed in the more early and early stage. The genes of group B were specifically expressed in the medium stage, such as *SsLRR-RLK19/24* and *SsLRR-RLK391*. Compared with the group A and B analyzed genes, the C group of genes (*SsLRR-RLK203/249* and *SsLRR-RLK359*) was significantly expressed in the late and more latter stage. The qRT-PCR results were highly consistent with that of the RNA-seq results, and revealed that the identified *LRR-RLK* genes in sugarcane exhibited significantly up-regulated expression levels. In summary, the LRR-RLK family may be involved in the disease defense process of sugarcane.

## 4. Discussion

Plant LRR-RLKs are the largest family of plant RLKs, which play various essential roles in plant life activities. The genome-wide identification and functional analysis of LRR-RLK gene families have been extensively carried out in many plant species whose genomes have been sequenced, such as *Arabidopsis*, *P. trichocarpa*, *G. hirsutum, S. lycopersicum*, *G. max* and other plants [9,10,11,12,13,14,30,31]. Previous studies identified 225, 234 and 467 *LRR-RLK* genes in soybean [11], tomato [13] and *Arabidopsis* [14], respectively. Sugarcane is subjected to various abiotic and biotic stresses during growth and development [54,58]. It is worth noting that the members of *LRR-RLK* genes in all of these species mentioned are larger than those in *Arabidopsis*, which might be due to their small genome. However, very little information is known about the *LRR-RLK* gene and its gene family in sugarcane because the modern sugarcane genome has been undeciphered until now. Fortunately, the genome-wide sequence of *S. spontaneum* AP85-441, one accession in the sugarcane ancestor wild species, has recently been published [32]. In order to better understand the characteristics and biological functions of *SsLRR-RLK* genes, we investigated the *LRR-RLK* gene family in sugarcane by bioinformatics means, together with a gene structure assay, and an expression pattern analysis was based on the several transcriptomic datasets. In the current study, a total of 437 *SsLRR-RLKs* members are successfully identified based on the genome of *S. spontaneum*. Compared with the *LRR-RLK* genes in other plants, the members in our study are smaller than those in soybean, but larger than those in tomato and *Arabidopsis*; this might be because the genome size of *S. spontaneum* AP85-441 is larger than that of tomato and *Arabidopsis*. When talking about the number of their categorization, 437 *SsLRR-RLK* genes are categorized into 14 groups (I, II, III, IV, V, VI, VII, VIII, X, XI, XII, XIII, XIV and XV) (Figure 2) and 10 subgroups (VI-a, VI-b, VII-a, VII-b, VII-c, X-a, X-b, X-c, XI-a, XI-b, XIII-a and XIII-b), of which, the group IX and the subgroup I-b are not found in sugarcane wild species accession AP85-441, indicating that the evolution of the *LRR-RLKs* in these two subfamilies may occur after the divergence of dicots and monocots or gene loss during evolution, according to the opinion of previous report [33], which also occurred in *G. max* [11] and *G. hirsutum* [12]. Previous reports showed that the *LRR-RLKs* in *Arabidopsis*, *S. lycopersicum* and *P. trichocarpa* were mainly categorized into 15, 10 and 14 groups, suggesting the same number of groups to that in *P. trichocarpa* [10], but less than that in *Arabidopsis* [14] and more than that in *S. lycopersicum* [13]. Furthermore, the *LRR-RLK* genes in *G. max*, *P. trichocarpa* and *G. arboretum* were mainly categorized into 26, 9 and 11 subgroups, suggesting a similar number of subgroups to that in *P. trichocarpa* and *G. arboretum* [12], but less than that in *G. max.* In contrast, the quantity of group II in *Arabidopsis*, *G. max*, *P. trichocarpa*, and strawberry is 14, 26, 29 and 8, respectively. Group V LRR-RLKs amount to 9, 18, 11 and 7, in the aforementioned four plants, respectively. Moreover, there are 33, 3, 28 and 56 members in group XI-a in the mentioned four species, respectively. By contrast, a total of 437 sugarcane *LRR-RLK* genes are identified in this study, and among them, 13 belonged to group II, 15 to group V and 124 to group XI-a, which is similar to *G. max*, but more than strawberry, and less than *P. trichocarpa*. Thus, group XI-a is relatively expanded in sugarcane in contrast to the other plant genomes mentioned. It was notable that LRR-RLKs were more closely related to the members of the same type than those of the different types in the same species, revealing a relatively high degree of homology similarity within the same type of SsLRR-RLKs. Furthermore, the diversification of exon–intron organizations and conserved motifs among the *LRR-RLK* gene members always plays an essential role in the evolution of this gene family. For instance, a previous study reported that the presence of multiple introns has been shown to be essential for *ERECTA* gene expression in *Arabidopsis* [59]. In addition, the findings further confirmed the classifications of the *SsLRR-RLK* genes, while the structure of SsLRR-RLKs in different groups or subgroups showed relatively lower identities [10,11]. Our research also identified the similar motif arrangements among the SsLRR-RLK proteins within groups or subgroups, which suggested that the protein structure is highly conserved within a specific subfamily. Indisputably, the roles of the vast majority of these conserved motifs remain to be clarified. Overall, the exon–intron structure and conserved motifs organization of the SsLRR-RLK members in the same group or subgroup, combined with the results of the phylogenetic analysis, could strongly support the consistency of the group or subgroup categories. As identified, *cis*-acting regulatory elements serve as an important molecular switch involved in the transcriptional regulation of the gene activities under various environmental factors, photoreactions and phytohormones, including the cold, drought, light, wounding, ethylene (ETH) and abscisic acid (ABA), etc. [10]. We found that the promoters of the *SsLRR-RLK* genes contain a large number of *cis*-acting regulatory elements related to environmental stress and hormonal signal responses, such as AAAC-motif, AACA-motif, ACA-motif, DRE, ABRE, LTR, MBS, I-box, WUN-motif, ERE, TGA-element and CGTCA-motif, etc. Two very interesting elements, OCT (participating in the cell proliferation response) and JERE (participating in the MeJA response), were significantly found in the X-c subfamily, with more than eight copies, implying that *SsLRR-RLK* genes within the X-c subfamily are mainly involved in the cell proliferation and response to MeJA (methyl jasmonate). In a previous study, the AACA-motif was shown to be an enhancer element that is required for the endosperm-specific expression of the glutelin gene from rice [60], and DRE and ABRE regulatory elements were shown to be interdependent in the ABA-responsive expression in response to ABA in *Arabidopsis* [61]. Hence, our results suggested that these *cis*-acting regulatory elements also play essential roles in the regulation of sugarcane *LRR-RLKs* expression in response to corresponding stress.

In recent years, although many *LRR-RLK* genes have been reported based on the whole-genome in the plant kingdom, their biological functions have been verified only in a small quantity of LRR-RLKs until now [14,16,19]. As the research progresses, increasing evidence suggests that *LRR-RLKs* are involved in the immune response, signal transduction and response to abiotic and biotic stresses [11,12,62]. In addition, many plant LRR-RLKs have been elucidated to play key roles in plant disease resistance. For example, FLAGELLIN-SENSING 2 (*FLS2*) and EF-Tu RECEPTOR (*EFR*) recognize the corresponding antigens and mediate the defense against pathogens in the processes of the plant immune response in *Arabidopsis* [63,64,65]. Besides, recent research indicates that somatic embryogenesis receptor-like kinases (SERKs) are essential in steroidal hormones BRs signaling, including the rice *OsSERK1* against fungal pathogen infection by mediating defense signaling transduction [66,67,68].

Abiotic and biotic factors, such as the cold, low nitrogen and pathogens, pose a serious threat to the yield of sugarcane. LRR-RLKs are essential for plants to perceive and receive environmental signal factors as transmembrane receptor-like kinase proteins to perform a series of biological functions [1,3,6]. In *Arabidopsis*, *LRR-RLK* genes were confirmed to regulate plant growth and development, including to mediate the defense against pathogens [27], signal transduction [16,18], adjust the meristem size [20] and promote stem cell differentiation [17,21]. Recently, many plant *LRR-RLKs* have also been reported to respond to many environmental stresses, including dehydration [12], heat [12], pathogens [12,27], the cold [12], high salinity [12] and ABA treatments [26]. Additionally, the expression difference in *SsLRR-RLK* genes based on the genome-wide identification and expression analysis between one pair of varieties with a different tolerance or resistance may play an important role in stress response or disease resistance. In summary *LRR-RLK* genes in sugarcane are also likely to have diverse biological functions. However, there are no reports about *LRR-RLK* genes observed in sugarcane. Thus, to investigate the potential roles and expression levels of *LRR-RLK* genes in sugarcane, we comprehensively analyzed the expression patterns of *SsLRR-RLKs* in the sugarcane’s response to biotic and abiotic stress. Five different datasets of RNA-seq used in our study were carried out, and the value could be significantly changed due to highly expressed *SsLRR-RLK* genes. The results showed that the expression of the vast majority of *SsLRR-RLK* genes was distinctively regulated in response to a given stress, which forcefully reveals that they may be vital stress response genes. According to the heat map, we also found that the *SsLRR-RLK* genes in the same subfamily have no consistent expression patterns depending on the analyses of gene expression data. Interestingly, some *SsLRR-RLK* genes might be highly expressed in a subfamily, whereas other genes exhibit a low expression or no expression at all. In a previous study, Sun et al. [12] revealed that the expression pattern of *Gossypium LRR-RLK* genes was widely involved in stress defense, including dehydration, the cold and salt, which provides insight into potential functional diversity within every one subfamily. The expression profiling of the *SsLRR-RLK* genes indicated that the *SsLRR-RLKs* could be responsive not only to abiotic stresses but also to biotic stresses. Therefore, these *SsLRR-RLK* genes stand as strong functional candidates for follow-up research into environmental stress in sugarcane. Overall, these above findings first provide a part of extremely valuable biological information that will accelerate our understanding of the regulatory roles of *LRR-RLK* genes in sugarcane stress defense response processes. Meanwhile, our analyses were also helpful in selecting candidate *LRR-RLK* genes for the further functional verification and disease resistance breeding of sugarcane.

## 5. Conclusions

Here, we performed the first comprehensive analysis of the identification and characterization of the *LRR-RLKs* gene family in sugarcane. Using the latest available *S. spontaneum* genome sequence as a reference, a total of 437 *SsLRR-RLK* genes were identified and were classified into 14 groups based on a phylogenetic tree analysis. The distribution of *SsLRR-RLKs* was found to be a random but unbalanced distribution across all chromosomes. The result of the exon–intron structures and conserved motifs were considerably conserved among members within the same groups and the same subgroups. The *cis*-acting regulatory elements and TF binding sites analysis of *SsLRR-RLKs* also indicated that *SsLRR-RLK* genes were multi-functional. Subsequently, further expression profiles and a qRT-PCR expression analysis of these *SsLRR-RLK* genes that were responsive to stresses in sugarcane were carried out based on the transcriptome datasets. In summary, all of these findings provide more valuable tools and information for future research of the potential biological functions of the *SsLRR-RLK* genes in sugarcane.

## Figures and Tables

**Table 1:** LRR-RLK proteins in *Saccharum spontaneum* and their key features.

Groups	Subgroups	No. of Genes	Amino Acid Length	With Signal PEPTIDE (%)	Molecular Weight (kDa)	Isoelectric Point
I	a	12	521–1081	58.3	53.79–117.29	5.70–8.57
	b	0	-	-	-	-
II		13	473–982	69.2	52.04–105.55	5.45–10.63
III		75	358–1775	84.0	39.00–189.90	5.55–10.49
IV		10	529–977	80.0	55.54–108.99	6.83–9.07
V		15	553–1403	80.0	59.12–153.82	5.22–8.69
VI	a	8	692–771	100	75.81–82.38	6.40–8.87
	b	8	572–1070	87.5	63.67–118.29	5.16–6.66
VII	a	14	925–1930	92.9	98.09–209.91	5.21–7.14
	b	5	649–714	100	69.08–76.10	7.25–9.56
	c	6	436–1095	33.3	48.33–115.15	5.54–8.97
VIII		6	549–1000	50.0	60.55–110.28	5.67–8.77
IX		0	-	-	-	-
X	a	5	560–849	80.0	61.52–91.70	6.44–9.90
	b	59	474–1830	59.3	52.62–197.90	5.06–7.25
	c	3	973–983	100	104.62–106.10	5.74–6.18
XI	a	124	471–2678	74.2	51.03–292.02	5.17–9.50
	b	1	3171	100	349.15	5.78
XII		51	566–2005	56.9	61.30–213.89	5.26–9.56
XIII	a	1	508	100	56.19	5.60
	b	2	958–986	100	103.67–106.54	5.66–5.96
XIV		6	661–992	83.3	70.94–107.52	8.27–9.78
XV		13	713–1209	92.3	76.83–128.91	6.02–9.09

Note: “-” means no corresponding information found.

**Table 2:** Statistics analysis of *cis*-acting elements detected in promoter of *SsLRR-RLK* genes.

Types	Functional Classification	Element Species (ID of PlantCARE)	No. of Elements
1	Light responsive elements	GATT-motif, MRE, 3-AF1 binding site, Sp1, CAG-motif, GA-motif, 3-AF3 binding site, chs-CMA1a/2a/2b, Gap-box, LS7, I-box, 4cl-CMA1b/2b, AAAC-motif, ACA-motif, ACE, GT1-motif, AE-box, TCCC-motif, AT1-motif, Pc-CMA2a/2c, ATC-motif, ATCT-motif, Box4, Box II, chs-Unit, LAMP-element, GATA-motif, G-Box, GTGGC-motif, L-box, sbp-SMA1c, TCT-motif	36
2	Hormone responsive elements	AuxRR-core, P-box, TGA-box, ERE, ABRE, ABRE2, JERE, TCA-element, ABRE3a, TATC-box, ABRE4, AT-ABRE, AuxRE, CARE, CGTCA-motif, SARE, TGACG-motif, TGA-element, GARE-motif	19
3	Environmental stress-related elements	MBS, ACTCATCCT-sequence, LTR, AP-1, STRE, MYB recognition site, ARE, as-1, box-S, DRE, MYC, DRE core, DRE1, W box, GC-motif, MYB-like sequence, MYB, WRE3, WUN-motif, TC-rich repeats	20
4	Development-related elements	GCN4_motif, AACA_motif, CCGTCC-motif, AC-I, CCGTCC-box, circadian, dOCT, E2Fb, HD-Zip 1, MSA-like, motif I, Myb-binding site, NON, NON-box, AC-II, O_2_-site, OCT, RY-element, re2f-1, CAT-box, telo-box	21
5	Promoter-related elements	A-box, AT-TATA-box, Box II-like sequence, CAAT-box, HMG-TATA-region, TATA, TATA-box	7
6	Binding site elements	AT-rich element, HD-Zip 3, AT-rich sequence, MBSI, BOX III, CCAAT-box	6
7	Other no functional description elements	AAGAA-motif, CTAG-motif, F-box, GC-repeat, GRA, H-box, plant_AP-2-like, TCA, Y-box, Unnamed_1/2/3/5/6/8/10/12/14/16	19

## Supplementary Materials

Supplementary Materials can be found at https://www.mdpi.com/article/10.3390/cimb43030116/s1. Additional data S1: Multiple sequences of LRR-RLK from A. thaliana and S. spontaneum were aligned by MUSCLE software with default parameters; Additional data S2: Phylogenetic tree constructed; Table S1: The primers used in the qRT-PCR analyses; Table S2: Detailed information of all identified SsLRR-RLK genes; Table S3: Signal peptide analysis; Table S4: Subcellular localization prediction of all LRR-RLKs proteins in sugarcane; Table S5: Statistics of A. thaliana and S. spontaneum LRR-RLKs distribution among different groups and subgroups; Table S6: Sequence logos of the conserved motifs of all SsLRR-RLKs among each group and subgroup predicted by MEME; Table S7: Cis-acting regulatory element analysis of the SsLRR-RLK genes; Table S8: FPKM values of 437 SsLRR-RLK genes based on five different stress RNA-seq data; Figure S1: Exon–intron structure of all identified LRR-RLK members in sugarcane; Figure S2: Detailed schematic illustrations of the conserved motifs identified in SsLRR-RLK proteins. Fifteen conserved motifs were distinguished by different colors; Figure S3: Detailed schematic illustrations of the TF binging sites predicted. Only shows the top 12 transcription factor families; Supplementary Material S3: The upstream 2000 bp sequence of 437 SsLRR-RLK genes; Supplementary Material S4: a diagram showing all of the steps developed; Supplementary Material S5: morphology of disease of sugarcane leaf blight (SLB).
