# Supplementary material for: Genome-Wide Identification of LRR-RLK Family in Saccharum and Expression Analysis in Response to Biotic and Abiotic Stress
Source: Curr Issues Mol Biol. 2021 Oct 18;43(3):1632–51. doi: 10.3390/cimb43030116 (PMC8929030; doi:10.3390/cimb43030116)
Supplement: Supplementary file 1 [file cimb-43-00116-s001.zip › 09082021 supplementary materials.pdf]

Supplementary Materials: **Additional data 1:** Multiple sequences of LRR-RLK from *A. thaliana* and *S. spontaneum* were aligned by MUSCLE software with default parameters, **Additional data 2:** Phylogenetic tree constructed. **Table S1:** The primers used in the qRT-PCR analyses, **Table S2:** Detailed information of all identified *SsLRR-RLK* genes. **Table S3:** Signal peptide analysis, **Table S4:** Subcellular localization prediction of all LRR-RLKs proteins in sugarcane, **Table S5:** Statistics of *A. thaliana* and *S. spontaneum LRR-RLKs* distribution among different groups and subgroups, **Table S6:** Sequence logos of the conserved motifs of all SsLRR-RLKs among each group and subgroup predicted by MEME, **Table S7:** *Cis*-acting regulatory element analysis of the *SsLRR-RLK* genes, **Table S8:** FPKM values of 437 *SsLRR-RLK* genes based on five different stress RNA-seq data, **Figure S1:** Exon-intron structure of all identified *LRR-RLK* members in sugarcane, **Figure S2:** Detailed schematic illustrations of the conserved motifs identified in SsLRR-RLK proteins. 15 conserved motifs were distinguished by different colors, **Figure S3:** Detailed schematic illustrations of the TF binging sites predicted. Only shows the top 12 transcription factor families, **Supplementary Material 3:** The upstream 2,000 bp sequence of 437 *SsLRR-RLK* genes.
