# Supplementary material for: Genome-Wide Identification of LRR-RLK Family in Saccharum and Expression Analysis in Response to Biotic and Abiotic Stress
Source: Curr Issues Mol Biol. 2021 Oct 18;43(3):1632–51. doi: 10.3390/cimb43030116 (PMC8929030; doi:10.3390/cimb43030116)
Supplement: Supplementary file 1 [file cimb-43-00116-s001.zip › Supplementary Material 3.pdf]

Additional file 3: The 2000 bp nucleotide sequences upstream of the translation strat site of 437 *SsLRR-RLKs* genes

>SsLRR-RLK327
GACGTCTCTCATCTGGGTACAATCGTAGTATTTGTTTATTTACAAACTCACACCAACCGTACACACGCACACCAAACCTACAA
CTACACTCAGATCATATCGTGTTCTTCGTGAAATCACCGAGTCCATCCATGGCATTGGCGACGGGCGCGTCTCATTCCCTTTG
TGACTCCACGCATGAGAGGCAAACGAAATTTATTGGGGACTAACCCCGGTGAGAGGCGCGAGTAGGATCCAGAGTAGGATC
CAGGAATCGAACTCGCGTCGACTGCCTGCACACCCGCAGAGCTAGCCACCCGAGCTCTGCTCGGTTCTTAGACTTCCCCATT
CACGGGTGAATGAGTATAGCCAATTCCTTACAACTCCAAAGCGCTTAAATCCACTCCTTACAACCCCACTATAGGATTGTTGT
GGCGGAGCTAGCAGAGCCCCATGTAGCATAGTGTTGTACCGTTCTATGGCGGAGCTGTCAGAGCTGGAGCCAATAGAATTGA
TGGCAAATGGCCTAAAATTATATTTATTCTGGTATTGTAAACAATAGTGCAATATGTTTTTTTGGCAAAATAGTTATTTTCATCC
CTAAACTATTGCCCGAGGCTCAGTTTGGTCCCAAAACTTTTAAATTGTCCACTTTAGTTCTTAAACAATGGATTTGCAACTCA
ATCTCGTTCACCCCCTTCATGTCTCGCCATGTGGCTAGCACTGCACACGAAATGTTGCTCCTACCCCTCGCTTTTCCCTTTAGT
TTAGGGACCAATCTGAGCCGCAGACAATAGTTTAGGGACGAAAATAGCTATTTTACCTATATTTTTATATGTTAAAGTTTAAAA
TATTTAAATTATAGGATAAACGTTGAACGTTAGACATACATACAAGGGAGAAAGAAGAAGAGAAGGATTCTATTCTGTACATT
TACATTCACCGCAACGTTGCACTGGTCCCCGCATTAATCTACTACTCCTCCTGAACAGGGTACTTAGACCACCTGCACCGTTG
AACTAAATCCCATCCTCTACGTCTCCACATCAGTGGTTACCGTATCAGATATGAAAAAATAAGGCTGTGTTTAGGTGGGGTTG
TAAAATTTTTAGAAGCACAGTAACTTTTTCGTTTTTATTTGGTAATTAGTGTCTAATCATGGGCTAATTAGGTTCGAAAGATTC
GTCTCGTAATTTACAACCAAACTGTGTAATTAGTTATTTTTTTATCTATATTTAATACTCCATGCATGTGTCTAAAAAATCGATAT
AACAGGAAATCTTATAAAATCTTTGAAACTTTGAAACATCTAAACACAGCCTGAGCTGCAGCGGTGTGCGCTATGCACGCGC
TATCAAGCATCCACCGCTATTTCTGGCGCATGGAGGATGTACTTTGAGCAGCACTTACGTGAGAGAGAGGAGAGAGGTGAC
AGCGGCGGGCTCCACTTGTTTTCCGCAACAAAAAAATGTTATAACATTGTATCATTAAATTCGTTTCAATAAATACTTTAAAAT
AAGATGTCACATGTATATATTTAGATAAAGTAACAAGTGAGTCCCACAGTGAATAGAGGATGATATAGATGATGGATGGATGC
GAGAATTCTATATATTTTATGATGAATAGGATGTAATATTCCTTTTAAAGAATGAATATAGAGATGCATGAGTGCGGATAATCTT
AAGCAGATACAGTTTCTGATCCGTCATTTTCACCAGCAGCTAGTACACATCGCCAGCCTATTTTGTACGTATCCTCGTATGATA
CCACAGACAGGCACATGACACATGCACTCGTGTACCATAGTAGCCTCATCATGCATCCACGCTGCCACTAACAATCCCCATAA
ACCCCCTCCCTTTTTCACCTTTTACTATCCATCCAAACGCCTTTCCAAGCCTCCTACAGCTTTTGACTCCCCCACTTTTTACCA
CAAACACCCCCTATAAACCTCCGAATTCCCACACCCCACACATTGCCCAAGAACTCACTCGGTCACTCTCACTCTGCCTCCG
CCA
>SsLRR-RLK336
AGTAGTTTCTACGAAAATTAAGTTTCTAGATATGCTCTAATGTTTATTTTTGACATTGAATTTTGGTAATGATATATATCTTGTCC
TCCTCCTCTTGTCTCTTTGTGGAGGAAAATCCTCCCATCGGTATAGTTTGCACTTTTTTAGATTAAGGAATAAAGAAGTATTCC
AGACTTTTCCATTCTTTTTTTTTTGACGCCTCTCGTCTGGGTACCGTCGTAGTATTTGTTTATTTACAAACTCACACCAACCGT
ACACACGGACACCACACCACGCGTACACAAACAAACCTACAACTACACTCATATTATATCATGTCCTTCGTGAAATCACCGG
GTCCATCCATGGCATCGGCGATGGGCGCGTCTCATTCCCTTTGTGACTCCACGCGTGAGAGGCAAACGAAATTTATTGGGGA
CTAACCCCCGGTGAGAGACGCGAGTAGGATCCAGGAATCGAACTCGCGTCGGCTGCCTGCACACCCGCAGAGCTAGCCAC
ATGAGCTCTGCTCGGTTCTCAGACTTCCCCATTCACGGGTGAACGAGTATAGCCAATTCCTTACAACTCCAAAGCGCTTAAAT
CCACTCCTTACAACCCCACTATAGGATTGTTGTGGCTGAGCTGGCAGAGCCTCATGTAGCATAGTGTTGTACCGTTCTATGGT
GGAGCTGTCAGAGCTGGAGCCAATAGAATCGATGGCAAATGGCCTAAAATTATATTTATTCTGGTATTTGTAAACAATAGTGC
AATATATTTTTTTTGGCAAAATAGTTATTTTCATCCCTAAACTATTGCCCGAGGCTCAGTTTGGTCCCGAAACTTTTAAATTGTC
CACTTTAGTCCTTAAACAATGGATTTGCAACTCAATCTCGTCCACCCCCTTCATGTCTCGCCATGTGGCTAGCACTGCACACG
AAATGTTGCTCCTACCCCTCGCCTTTTCCTTTAGTTTAGGGACCAATCTAAACCTCAGTCAATAGTTTAGGGACGAAAATAGC
TATTTTATCTATATTTTTATATGTTAAAGTTTAAAATATTTAAATTATAGGATAAACGTTGAACGTTAGACATACATACAAGGGAG
CAAGAAGAAGAGAAGGATTCTATTCTTTACATTTACATTCACCGCAACATTCCACTGGTCTACATGGACCGCAATAATCTACT
ACTCCTCCTGAACAGGGTATTTAGATTATCTGCACTCATAGACCTCTATATTCATTCTCTAAAAGAAATATTCCATCTTAGTCAT
CATAAAATATACAGTTTCTCCGCATCCATCCATCCTCTATATCATCCTCTATCCATTGTAGGGTCCACTTGTCACTTTATCTAAAT
ATATTCATATGAGATCTAGTTTTGAAGAATTTATTGAAACAAATTTAATGGTGCAATCGGAATTTAATTTAATTTTATAGTTTGT
GATTTATAACATTTTTTAGATAAATGAAACTGTGGGGTCCGCCACTGAGATTCCCTCGTGGCACGCACGCTCTCTGCTTTGTC
GCTCCCGACCGGTACCGTAAAGCTCAGTACTCCTTCAAATTTGCCGGAGAAAGAGGCATACAGGCCGGTTTACCGTCCCCG
AGTGCAGCTCATTTCAAACGCTATAAGGGACGTGGAGCTATACCGGTGGCCTTTACAGGCACCGGTGCAGAAGGTCTTAAGC
AGATACAGTTTCTGATCCGTCATTTTCACCAGCAGCTAGTACACATCGCCAGCCTATTTTGTACGTATCCTCGTATGATACCAC
AGACAGGCACATGACACATGCACTCGTGTACCATAGTAGCCTCAACATGCATCCACGCTGCCACTAACAATCCCCATAAACC
CCCTCCCCTTTTCACCTTTTACTATCCATCCAAACGCCTTTCCAAGCCTCCTACAGCTTTTGACTCCCCCACTTTTTACCACAA
ACACCCCCTATAAACCTCTGAATTCCCACACCCCACACATTGCCCAAGAACTCACTCGGTCACTCTCACTCTGCCTCCGCC
>SsLRR-RLK405
GTTATCTTCTTTCTATATTTCTATTCCTCTCTACTATGCAATCTCATTGCTATTCGATGATTTTTTTTTGTTTTCTATTCCTCTATTC
CAAATAGGCTCTAAGATATTTTTTCCGTCTGTTTCTATTTCTTGTGTATCTCGCACCATGACACACAAGCACACTTAGCGACCT
GAGGATGTCTATAAGGGTGAGATGCAACTATAAATAGAAATCTCGCTAGGCGTCATGCAGTATACTTTTTTTTGATAGGATCAT
GTAGTATATAGCATTAAGGTATATTTGGATCTCCTAGAAATTACCAAGATGAAATGCAAGGATGAGAATAAAGTCCAAACAAG
TGGATTCCATTTCTACATGGGATTTTTGTAAATCAACCATCAAATTCTCACAATCCAACCATCCGTATAACATTGCTATGAAGA
GATAACACATCTAACCAACTCGGATCTCTAATAATACTATAGAACACAAACTAGCTTCTTTTTTTTTCTAGATCTAGATTCTACG
ATCACCGTCTAAAATCCCAATTTTTAGAATCATAGGGAGCGTAAATCCATATTTAGAAAGCATTTAGTTCACAGCCAGAGGTC
AATGCATCATGCCGCAAGTTTCGTGCCTAAAGTTAGTTGCGTGTTTGGTTTGCTGCTCGAGTTGCGGCACCGCCAACCCTTG
TCATCGCGCGCCTAGTTCATTTTTCACACACAGTTTATCAAATATCTAGGGGACAAATTGTCCACCGCACTTTTGGCGCGGCC
CTCGCAGAGAATGCATAGGTGGACACCTAATAGGGCCTTAACTTTTATATTGAGCCACAATACATAGAATTCTATATTTTTGGA
CATTCTTACTTGGGTAGAATCATAGAAATATTTAGGAGATCGTGAGAAGAGATGAGAGGGACGAGAGAGGAAGACGGCACC
AACACATAGGTCCAATGTGTCTGGCTCTAGTCATCGTATGCTTCTAGATAACTGAGATTAAAAATTTTGGGACTTGAAAATGT
ATTTTGAAAATTTGAGACTCGATACAGTTAGCAGTTCACATAAATTCAGGAAATTATATTACGATGCAATCCTTGAATTTCAAA
CTATGCCTGAAATTTTAGGCTTAGGCTATCCGCACCGCGGAGCGCCAAATCCATCGGAACGCTAGGGATACCGTTTCAATTCG
TGCACCGCGGAACGGCAAAGCAACCGCTATCGCTCAGTTCGTCACAGTGAAACGCCAAGTTCGGCGTTCCTCTCTCCTCTC
GCTATCCTCTCCAGGCGGGCTCCGCTCCGGCGGAGGTCGAACTCCGGGCGGCGGACGAGCTCCGGACGGGCGCGCGGACG
GCGGGCGAGGTCCGCGCCGGCGAGCTCCGCGCGGACGCGAGTGCGGTGGGCGACCTCCGCGCTGGCCACCTCCATGGCCG
GACGGGCGTGCGGACGGCGGGCGAGGTCCGCGCCGGCGAGCTCCGCGCGGGCGCGAGGGCGGTGGGCGACCTCCGCGCT
GGCCACCTCCATGGCCGGACGGGCGTGCGGACGGCGGGCGAGGTCCGCGCCGGCGAGCTCCGCGCGGACGCGAGGGCGG
TGGGCGACCTCCGCGCTGGCCACCTCCATGGCCGGCGGGCGAGCTGCGCGCGGGAAGGAGTGGAGGGAGGGAGGGGAGC
CGCGCCGCTGGGAGGCCGCCGGGTGCTGCCGCGCGAGCTCCGCCGGCCCACCTCCATGGCCGTCCGGAGAGAGCGAGGAG
AGAGCGAGGAGAGGATGGCGGTCCGGATAGCGTTGTGCACGCGGCAGCTCCCTCGCCTGTACGCCGCCTGCCAAGCGGGC
CCCACCTCTTTTAGCGTGGGCGATGGAGATGGGGTGGTGCGCATAGCCTTAAACCCCGTCACAACTCGCATCCTCGCCGTCA
CAACACAAAAAGGGCGCACGAAACCTGCGGAATCAGGTCTTCCCCCTCACTCTACATCACTCTCTCCGGTCCTCCCTCCTCA

CTCCGCCGCTTCAAGCCCCCTACTCCCA
>SsLRR-RLK415
TTGCTATCAAGAGATAAAACACTTCTAACCAACTCGGATCCCTAATAATAGTTTAGAACACAAACTAGCTTCTTTTTTTTCAA
GATCCAGATTCTACTATCACCGTCTAAAATTTTGATTTTCGGAATGTTAGGGAGCATTTGGTTCACAACCAGAGCTCATTGCA
GCATGTCGCAAAAATCGTGCCGTGTTTGGTTTGCTCCTCAAGTTGCGGCGCGGCCAACCCTTGTCATTGCATGCCTAGTTCAT
TTTTCAAGTCGCAATTTGTCAAATGTTTGATGGACAAACCGTTCGCCGCACTTTTGGTGCGGCCCTCCGGCGGAGCGTGCAT
AAGTGGGCACCAAACATACACTTAACTCTTTATATTGAGCCACAATACATAGAGTTCTAGATTTAGACTACTCGCACTCGTC
GTACTCTAAATTCATCCTCTAAAAGGAATATTCTGTCCTGGTCATCGAGATTTCATCCTCTATATCAATTTCTCCCGCACTCATT
CATACTCTATATCATCCTCTATCCACTGTGGGGCCCACTTGTCAGATTTTTTCTTTTTTTTCCTTTTTCTCTTTTCTTTTCTTTTCT
TTTCTTTTTCCCCTCTCCTCCTTTATCCATTCCACCGCACTGCCCTCCCTCCCCATTCTCTCTCTCTCTCGGGCGCGACGGAGG
CGGAGGCCCGCGCGGCGGTGCTCCCCTTCTCCCTCTCCCCTCCCGGCTCCCTCCGCCGGCTCCCTCCACCACCGGCTCCCTC
CACCGGCCCCCTTCTTCTCCCTCTCCCCTCCCGGCTCCCTCCGCCGGCTCCCTCCACCACCGGCTCCCTCCACCGGCCCCCTT
CTTCTCCCTCTCCCCTCCCGGCTCCCTCCACCGGCGCGGCGTCCATCTCCGAGGGGCTCCGAGGGCGGCTGATCTGCCTCCG
GCGGGCGGCGCGCCGACGGGCCTCCAGCACCAGCGCGCCCGCGTCCGACGGAGATGGCGAGGCGGGCCTCCGGCGGCAG
GCGAGCCGCGCGCGGGCGCGGGGACGACCTCCTCCTCCGGCTCCGGGCATGAGGCGAGGGCGAGGCAGAGCTTCCTCAGC
GGGCGCGGCGGAGCTTCCTCGGCGGCGCGTCGAGGCCGTGGTGGGGCGTGGTGGGGCCAGGGCAGGCGTAGCGGACGCA
CGCTACTCCGCCAAAGATAGCGGACGGAGGCCCCGATGCCGGATGAGCTAGCGACATAGCGGACGCCGCTGCAGCAGTTTC
GTCCGCTATCCGCGCGTAGCGGACGCGGATAGCGGACGCGGTGCGGATAGTCTTAGGACATTCTCACTTGGGTAGAACCGTA
GAAATCTTTAGAAGATGATGTGAGGGATAAAGAAGAGAGAGGAAGACGGCACACATAGGTCCCATATGTCTGGTTCTAGTC
ATCATATACTCCTAGATAACTGAGATTAAAAATTTTGGGACTTGAATATGTATTTTGAAAATTTGAGACCCGATACAGTTAGCA
ATTTCACATAAATTCAGAAAATTATATTACGGCTGTGTTTAGTTCCTCCAAAAATGCACTATGCAAAAAAAAGATTCCCCGTC
ACATCAAACTTGCGATACATGCATAGAGTACTAAATGTAAATGAAATCAAAAACTAATTGTACAGCTTGGTTATACTCTACGA
GACAAACGTTTTGAACCTAATTAGTCAACGGTTAAACACTATTTACCAAATAAAGCTAAAATATGCTACGGTATATTTTTCACC
ATTTTCGAGGAAACTAAACACAGCCTACAATGCAATCCTTGAATTCCAAACTATGCCTGAAATTTTAGGCTTATAAACCCCGT
CACAACTCACAACCAAAACTCAAAACCTACAAGTTAAAAAAAAAACCCTCAAAACCTACAACACAGAGGGCACACGAACC
TGCGGAATCAGGCTCCCCTCACAGCCCGCTCACTCTACTTCACTCTCTCCGGTCCTCCCTCGTCCCCCCACCCCTCTCCGCCG
CTTCAAGCCCCCCTGCTCCCA
>SsLRR-RLK436
TGATTAGCTCGCGAACCGACAAGTGGGTGCATCGCCACAACGAGGAGTAGCTTGCCGGGAAGCAAGTGAACCTCGGGATA
AATTCTTGTGTCATTCTCTTCCCGAGGATTTCTCTTGAGTGATCGATTGTTTGGTACTTCTCGTCTCTACACGGCGGTATAATC
ACTCGAAGCTCTTCTCTCGTATTACTTCTCGTTTCTAGCTTGGTGTTGTTGTTGTGTTTAGTGGATAGCCTTGTACTAGAATTG
TGTAGGTGGCTTGCTTTTTTAGTTAGAGCTAGTGCTTTGTGCTTCGCCACCTTTCGTACTAACTTGTGGTTAAGTAATTTGTGT
AGAAGTTTTTATTATGCTATTCACCCCCCCTCTAGCCTATTTGGACCTTACAACCGTAGGTGGTGTGCAGGAGCAACGCGCGG
AGGTCCTCGCTGGCGGTGCAGGACACGTCGACATCATAGCCCGGGAACCTCACCATGGCCACCACGAACGTGGACTTGCAG
GTGTCGAGCATGGCCACCGCGAACGTGGACTTGCAGGCCCCGACGCCCAGCGAGGTGCACCTGTGGCTGCATGGGTGCCC
GTGTTAGCTAAGAGTCGCAGATCGCAACGGCGCTGCTCCATCTCCTCGCCCAAGGACTCTACATGCTGTTGGTAGGGTCGCA
GTACGGGGGAGCGGTCGCGGTGGCGCATGCGCAACACCAGGTGCTCACGTCGTCCTCAGCTGTCGCTGCTCGCGCTGGTCC
ACGCGAGACACACGCCAAGGAATGAGTCTTGCAAGTGCACGGTGTGGCCTGGGCCAAACTGGAGGGAGACCCGCCATTGG
TCCTGGACGCGGATGACACCACACACGCGGCTTCCGCGTCCTCCAGCGATGGCAGCGCGGCCACGTACGGGGCGGCCTTGC
GGAACGTGAGGTCCGAGCCCCGACATACGAAGGGCGAGTACGGAACTACCATGGCACCGTGGAGTAGTGCCAGCACGACG
GCGTCAGGCAAGCGAAATCACGGACGATGGACGCAGCAGCAGGGCGCGGCAGTGGCGGGCGGTACGAGGACTGGTCGAG
CAGCGTGCCAGGACAGGTCTGCGGTGGTCAGGGCATGGATGCCGGAGCAGCCGGAGGCCCGGAGCAGTGGTCAGCTAGGG
CTGGATGTTGAAGAAGAAGCTGACATGTGGGGCCCATATGTCAGTGAGGGTGAATGAGAGAAGCAGCAGGGGTAAATAAGT
CATTTCGCATGTCCTATAGAGGTTATAAGGGCGCTGTCGTGCTGGCATGAACACGTGGCATGCCACATCATCTAAAGTGCAAA
TACGTAGGAGCTTTACTATTTTGTGTGGCAAATATGTAGGTGCCCGTGTAAAACTGGCAATTGGAGAGGTGGCATCGGTAAA
GATGGCAGTTAAATATTCCTCGATACAGTTAGCAGTTCACATAAATTCAGGAAATTATATTACCATGCAATCCTTGAATTCCAA
ACTATGCCTGAAATTTTAGGCTTAGACTGACCGCACCGCGCAACGCGAAAACACGCGCTACGCCACATTTGGTGTTTGCACG
CCCGCACTGCGCGACGCTAAGCCAGCCGCTATGCTTGCGTTCACTACAGTGACCCGGTACAGACAGAGTTCGGGCGTGAGC
GCCTGTAAAAGGAAAGGAGGAGAGAGAAAGAGGGAGGGAGGGGAATAAAATATGGAATAGTGGGTATAGAGTATGGGATA
GAGATAACATAGGTGCAGGAGAAATAGATATAGAGTAGAGAATCTCGATGACTAGGATAGAATATTTCTTTTTAGAGATGAAA
ATAGAGGTCGATGGATGCGGATAGCCTTAAACCCCGTCACAACTCGCAAACAAAACAACACTCTCCACTCTCCAGTCCTCCG
CCGTCACAACACAAAAAGGGCACACGAAACCTGCGGAATCAGGTCCTCCCCCTCACTCTACATCACTCTCTCCGGTCCTCC
CTCCTCCCTCCTCCCTCCGCCGCTTCAAGCCCCCTGCTCCCA
>SsLRR-RLK176
AGCGATATGATGCCCCACTCGTCTCGTCTCATCTCATCGCCTACGCATGCCGGCTTTTCCTCTCTGGGCCGCCGTGATTATTCC
ATTTCCATCCGGTCGTCCTTTTGCTCCTCGGCCGATGATGACGACGACGCTGATGACACGAGATGGAAGAGAATGAGGAATT
GAGGATGCAGCAGCATGAGATGAATGAAACGCAATCCTATGAATGGTTATTATCATGCGGCTGACTTCACAGCACGGCCTGT
CTGCTAGCTGCGCCGTGCCGACTGGTGGTACTTACGATACGAGCACGGGCACGTAGTACACGTTTGGGCACCACACCAGAC
AGATATACTGCTACGTACGGAGGAGTGTATACGAGGCAGGCCGCACGCAGGCATCATGCGTGCAACGAATTGAATGAGTACG
AATGCCGCAGCAGGACTAGCAGCATGGCAGGGTCGGGTCGCGTCACGTGAGGCCGAGGCGGAAAGGAATGAGCGCACCTT
GACCTTGCCCCCCGGAGGACGAGGATGCTGACGCTGAGGCGCGCCGAGCCCGCAGCCGCGCNNNNNNNNNNNNNNNNN
NNNNNNNNNNNNNNNNNNNNNNNNNNNNNNNNNNNNNNNNNNNNNNNNNNNNNNNNNNNNNNNNNNNNNNNNNNNN
NNNNNNTACTATATTTACATTGTTTCTTCGGTGTATTTATTGGATAGATGGATGTAATTTGGGATAACACAAAAATATGCTAATT
TTTTAAAAAAAAAATTATAAATTATTTTATAGATTGCAGATACCAAAGAACATATGTCAAATTATTCATACATTTCAGAAAAAAAT
ACAAATTGTTCACAAATTCTAGTGAGACCCACCCTCCCGAACCGTATCCCTTCCCTTCTGTCTTCCGTGGGGTACTTCTTGTC
ACGTACTACGACGTATACTACTCGCTCGTGCCAAACTCTTGGCCCAGCAGAGCAGCACACACCCACTGACATACAAGGCCC
ACAGCTCTTTCGCTCACGGAGTCACGGTCACGGCTGACCCGACCACGCTATTATCACTACCACTCCACTCAGCTCCGTTCAG
GTCAGCTGCCTGCGCCCATTCAGCCATTCCATTCCTCCAAGCCCTCACACCTGCACGCGCCCACGCCCGGACGCCCTCGTCT
TGTCTCCCTCTCCTCTCCTCTCCCTCCTCGCCCACCCCCACCCGCACCAAGCACCACGCGCCGGCCTGTCCCTGTCCGTCCC
AGCTCACACGCGCAACAGGCCGGGCGCGCGCCGCGGGGGGCGATGGCCTCCGCCCGCCTCGTCGCGCTCGCCGCCTTCCTC
CTCCTCCTTCTCCTCGCCCTGGGCCCGGGCCAGGAGGCGCGCGCGGCGGACGACGGCGAGGTGCGCGCGCTGCTGGCTCT
GGGGCCGCGCTGGACCCGACGGGCCGCCTGCTCCCGTCGTGGGCGCCCGGCCGCGACCCCTGCGCGCCGCCGCCGTCGGG
GGGTTCGAGGGCGTCGCGTGCGACGCCCGCGGCGCCGTCGCCAACGTCTCGCTGCAGGGGAAGGGGCTCGCCGGCACGCT
CCCGCCCGCCGTCGCGGGGCTCCGCTCCCTCACCGGCCTCTACCTCCACTACAACGCGCTCCGCGGCGGCATCCCGCGGGA
GCTGGCGCGCTCGCCGCGCTCACCGACCTCTACCTCGACGTCAACAACTTCTCGGGGCCCATCCCGCCGGAGATCGGCGCC
ATGGCGTCACTGCAAGGTACTGCCTCTACCTCTATCTAGTCAGATGCGCGTCGAGATTCCTCGGAACCTTCATCGCCAACCTG
CTCGTGCCGACGCTCGCTGACTCGCATCGGCATGCATGTGGCCGTGCCTTGCTCCTTGCATCGATGATTGGTTTGCCCCCTGT
GTTTTTAATTCGTTTTCGGTCGACTTGCTTGGCGTTTCTCCACTCCAAGTCAATCAAATCAAAAACAAAATGCGGCTTTACCT
AACTACTCGTACCATGTATCCGTTGAATTTTTGGAAGATGTC
>SsLRR-RLK162

TACCTTAGGACATGTCTAGGTGACATTCAGTTGTTTTGGCCCCTTCTAATAACTGAATTTTGTCCATTTATTTGCACTTTCTTCT
AGCTATTTTTACATTATCTTATTACTATATTTACATTGTTTCTTCGATGTATTTATTGGATAGATTGATGAAATTTGGGATAACACA
AAATATATGCTAATTTTTTTTAAAAAAATTTATAAATTATTTTTATAGATTGCAGATACCAAAGAACATATATCAAATTATTCACAC
ATTTCAGAAAAAATTACAAATTGTTCACAAATTCCAATGGGACCCACCCTCCCGAACCGTATCCCTTCTCTTCCGTCTTCCGT
GAGCGAACGAACGAACGAGGGAGGCAACGTACGAGACTCAGGTTGCCGCGCTCGCCCTCGTCGTCGTCGGCCGCCGATGA
GACGCCTCGAGGTCGCCGCGCTTGCCCTCCTCGTAGCCCGTCGCCGGCCCTGCGCGCCACGTGCCGCGCCTGCCATGGCCG
CATGCCCTCCATGCCCGGGGCCGTGAGCTCCGAGGCCAGTCGCCTCGCCTGCTCCCCGGCCGCGCTCCCCTGCAGGCCCCG
CGCGCCCTCCATGCCCGTCCGCGCCAGATCAGACGGCCCAAGATAGAACCCTGTTTTACACTTTTGCATTGGTCACATCACA
ATTCACATGCAAGGCTGTGGGGTACTTCTTGTCACGTACTTCCTCCGTCCGAGAAAAGATGCAATTATGGAATACATGCCAGT
CAAACTGACTCAAGTTTGACCCAGTTTATAGCAAATAGTATTACTCCCTCCTTTCAAATATCTAGAGCGTATTAGCAAAAAAA
AAAATTTTCAAAAGTCTCCGTCGTTTAAGTCCGGGACCACGCCCTCGCATCGTTCCCAGGCGCCCGTGCGCTTCCTCGTACG
CGTGCGCATTCACTGCTGCGCCGTTGCCCGTCACTTCGACTGGCCCCTCCGTTCCCTCCCGTTAGAATGCAAAGCCTTTCCGT
TGCTTCAAATACTCCCCGTCGCAACCAAGTACTACTCCATCTGTCGATGTGTTAGCCTCCACAAAGAACTCGACTGCAATGG
ACCAGGAAGGAGACGCCAGCCATCAACGTCTCGTGCGTGGCGTGAACTGGGCGCCGGATTCAGATGGCTGATCATTCAAAT
CAACGTCAAATGGAGGCATCTAATCCAGCGTGAATCAGAGGTGGAGAAAGAAGAGGGAGTCAGGTGGAGAGAACAGAAG
AGTTGGCTGGACGTTTCCATCGTCGGCAACTTATATAGGCCACGGAAAATTGAAATTCGAACTGGGCAGTGGCGCCAATGAA
GGAGCGCGATTAGGAAGGAGCGATTAGGTTTCAATTTTGGCTGCCAAAATTTTCGGAATCTGATGCTGTTCGAGGGCGCGAT
TAGGAAGGAGCGATTAGGTTTCAATTTTGGCAGTCCGTGAGCGCGGGAGAGCGGGGTTGCGTACGTGAACGCGGGCGCGC
GATCTTGAGCGCGCTAACGGCGTTTTCAGCGGTGTTTACCTGGGCAAACGAAGGGACGCCGGGCATTAAGACGAACGCAGC
GATTAAATGAAAAGTGGCGCTCGTGGCTGCATGTTTCCATGCTCCTTGGTCATCGCGTCCAAGCCTAATACGCTCTAGATATT
TGAAAGGAGGGAGTAATATTTATGTCTTTAAATAAATTTATTATAAAAATATATTCTATAATTAATCTAATAATACTTATTTTGTAT
CATAAATATTAATATTTTTTAATATAAATTTAATTAAAGTTGAAACTGTTTGACTTTTCGAAAAACGAGAATCGCAGGTTTTTAT
GGACGGAGGGAGTACTACGACGTATACTACTCGCTCGTGCCAAACTCTTGGCCCAGCAGAGCAGCACACACCCACTGACAT
ACAAGGCCCACAGCTCTTTCGCTCACGGAGTCACGGTCACGGCTGACCCGACCACGCTATACCACTCCACTCAGCTCCGTT
CAGGTCAGCTGCCTGCGCCC
>SsLRR-RLK149
TTCATTCTCAAAACTTTACGTCCTATTCTGTTAGATATTTAGACACACACACATATATATATATATATATATATATGGAGTATTAAA
TGTAGACTAAAAAATAACTAATTACACAGATTATGTGTATTTTGTGAGATGAATTTTTTGAGTCTAATTAATCCATGATTAGACA
ATGTATGCTACAGTAAACATATGCTAATAATAGATTAATTAGTCTTAATAAATTCGTCTCACAATTTATATACGAGTTCTGTAATT
AATTTTTTATTAGTATCCAAACACCTCTTCTAATATTCCGTAAAATATCTGATGTGACACCTAAAAACTTTACACCCTAAAACTA
GAGAAGCCCTCAATACTGGCAGCTCAGGGCAATGCCAAACCCGAGTCCCCAAACGTCGCAGGCAATGCAACCAAGGTTTG
GCATTCAAGTCGGATTCACGGACACAAGCTGCATGCAGCGACACGACATATTGGATTCGTCGCTAAACGCTGCGACGTCATA
GCATAGTTTAGACGGTGCAGCTTGCTGCCATGGATCGTTGTTAACCTCTTGGCCCACCCTCGCAAACATATTCATCCCTGGCT
TTATTTGATTTTGTTCGATAAAGTTAGGGACCAACGAAATGTGAGCCAGCGAGTTTGGTAATGCACCGATACCTTCTTTGGGC
GGTAATTGAATTGGACTCCACGTAGTTTTTTTTTTTGACAAAAAAATGGACTCCACGTAGTTGACCGGAGACCAAACATTCAAA
CTAGGTACCTTAGGACGTGTCCAGGTGACATTCAGTTGTTTTGGCCCCTTCTAATAACTGAATTTTGTCCATTTATTTACAGTT
TCTTCTAGCTACTTTTACATTATCTTATTACTATATTTACATTGTTTCTTCGATGTATTTATTGGATAGATTGATGAAATTTGGGAT
AACACAAAATATATGCTAATTTTTTTAAAAAAATTAAAAATTATTTGTATAGATTGCAGATACCAAAGAACATATGTCAAATTAT
TCATACATTTCAGAGAAAATTACAAATTGTTCGCAAATTCCCGTGAGACCCACCCTCCCGAACCGTATCCCTTCCCTTCCGTC
TTCTGTGAGCGAACGAACGAACGAGGGAGGCAACGTACGAGACTCAGGTTGCCGCGCTCGCCCTCGTCGTCGTCGGCCGC
CGATGAGACGCCTCGAGGTCCCCGCGCTTGCCCTCCTCGTCGCCCGCTGCCGGCCCCGCGCGCCACGTGCCGCGCCTGCCA
TGGCCGCATGCCCTCCTTGCCCGGGGCCGTGAGCTCCGAGGCCGGGCGCCTCGCCGGCCCTCGCTGGCCGTGCCCCCCTGC
AGTGCAGGCCCGCGCGCCCTCCATGGCCGTCCGCGCCAGATCAGACGGCCCAAGATAGAACCCTGTTTTACACTTTTGCATT
GGTCACGTCACAATTCACATGCATGGCTGTGGGGGGTACTTCTTGTCACGTACTACGACGTATACTACTCGCTCGTGCCAAAC
TCTTGGCCCAGCAGAGCAGCACACACGGGGGGCGAAGCTACAAGCAAATGAACCATGATGCACTGTTTAAGTAATTTACGC
AACTTTATTAAAACATACGGTAAAAACAAAGCTGTTGAATGGCAAAAAAAATTTTACCCTGGTGCCTGTGCACCAGGTAACT
TCAACGTGGCTTCGCCCCTCACACACCCACTGACATACAAGGCCCACAGCTCATAAGCTCACGGAGTCACGGTCACGGCTG
ACCCGACCACGCTATTATCACTACCCACTCAGCTCCGTTCAGGTCAGCTGCCTGCCTCTGCCTGCGCCCATTCAGCCATTCCA
TTCCATTCCTCCAAGCCCTCACACCTGCACGCGCCCACGCCCGGACGCCCTCGTCTTGTCTCCCTCTCCTCTCCTCTCCCTCC
TCGCCCACCCGCACCAAGCACCACGCGCCGGCCTGTCCCTGTCCGTCCCAGCTCACACGCGCAACAGGCCGGGCGCGCGC
CGCGGGGGGCG
>SsLRR-RLK177
CAATTATAGATTAAAAAATAACAATATTTATGATATCAAATAAATATCATTAGATTAATTATGAAATATATTTTCATAGTAATAAAT
TATTTTGGAGCTGTAAATATGAATAGTATTCACTAGAAATTTGGTCAAACACGAACCACTTTTACTGGCACACAACTCGTAGT
TGCATTCTTTTCTGGACAGAGGGAGTATTAGTATTTATGTCTTCAAATAAATTTATTATGAAATTATATTCTAGAACCAATTTAAT
AGTATTTATTTTGTATTATTAATGTTAATAACTTTTTATAAAAAATTACTTAAAATTCAATGCGTTTCACTTATCGAAAAGCGAG
ACCGAGCGAGAATCCATGTCCCACGGATGATGATTAGATTCTCGTTTGCGGCCCTTCACAGTGTCGGCCGGCCCTACGTTGT
GACTAGTTCCCGATGGATTTTTTTTTTGATGTGCACTGAAACCGTTGCCGCAGCGTGACGCCCGGCTCCTGGTCGATTCATGG
TCTTCTTTAGTTCATTCTCAAAACTTTACGTCCTATTCTATTAGATATTTAGACACACATATATATATATATAGAGTATTAAATATAGA
CTAAAAAATAACTAATTACACAGATTACGTGTATTTTATGAGATAAATTTTTTGAGTCTAATTAGTCCGTGATTAGATAATGTAT
GCTACAGTAAACATGTGCTAATAATAGATTAATTAGTCTTAATAAATTCGTCTCACAATTTATATACGAGTTCTGTAATTAGTTT
TTTTATTAGTATCCAAACACCTCTTCTAATATTCCGTAAAACATCCAATGTAACACCTAAAAACTTTAGGTCATAAACTCATAAT
ACTAGAGAAGCCCTCAATACTGGCAGCTCAGGGCAATGCCAAACCCGAGTCCCCAAACGTCGCAGGTAATGCAACCAAGGT
TTGGCATTCCAAGTCGGATTCACGGACACAAGCTGCATGCAGCGACACGACATATTGGATTCGTTGCTAAACGCTGCGACGT
CATAGCATAGTTTAGACGGTGCAGCTTGCTGCCAACCTCTTGGCCCACCCTCGCAAACATATTCATCCCTGGCTTTATTTGATT
TTGTTCGCTAATCTCTCCAGTCACGATAAAAGTTAGGGACCAACGAAATGTGAGCCAGCGAGTTTGGTAATGCACCGATACC
TTCTTTGGGCGGTAATTGAATTGGACTCCACGTAGTTTTTTTTTTTTGACAAAAATGGACTCCACGTATTGACGGGAGACCAA
ACATTCAAACTAGGTACCTTAGGACGTGTCTAGGTGACATTCAGTTGTTTTTGGCCCCTTCTAATAACTGAATTTTGTCTATTT
ATTTGCACTTTCTTCTAGCTATTTTTACATTATCTTATTACTATATTTACATTGTTTCTTCGGTGTATTTATTGGATAGATGGATGT
AATTTGGGATAACACAAAATATATGCTAATTTTTTAAAAAAAAAATTATAAATTATTTTATAGATTGCAGATACCAAAGAACATAT
GTCAAATTATTCATACATTTCAGAAAAAAAATACAAATTGTTCACAAATTCTAGTGAGACCCACCCTCCCGAACCGTATCCCTT
CCCTTCTGTCTTCCGTGGGGGTACTTCTTGTCACGTACTACGACGTATACTACTCGCTCGTGCCAAACTCTTGGCCCAGCAGA
GCAGCACACACCCACTGACATACAAGGCCCACAGCTCTTTCGCTCACGGAGTCACGGTCACGGCTGACCCGACCACGCTAT
TATCACTACCACTCCACTCAGCTCCGTTCAGGTCAGCTGCCTGCGCCCATTCAGCCATTCCATTCCTCCAAGCCCTCACACCT
GCACGCGCCCACGCCCGGACGCCCTCGTCTTGTCTCCCTCTCCTCTCCTCTCCCTCCTCGCCCACCCCCCACCCGCACCAAG
CACCACGCGCCGGCCTGTCCCTGTCCGTCCCAGCTCACACGCGCAACAGGCCGGGCGCGCGCCGCGGGGGGG
>SsLRR-RLK346
GCGCTACAAGGCTCGCTGGGTCCTCCGCGGCTTCACTCAGCGGCCCGGCATTGACTATGCTGAGACGTTCAGTCCCGTGGTG
AAGCCAGCTACAGTCCGCACCGTCCTCTCCCTGGCTCTCGAGCGCTCCTGGCCTGTGCATCAGCTGGATGTCAAGAATGCCT
TCCTTCATGGCACTCTGACTGAGACGGTCTACTGCAGTCAGCCGCAGGTTTTGTGGATGCTTCTCGTCCTGACTTCGTCTGCC

GGCTCAACAAATCTCTCTACGGGCTCAAGCAGGCACCCGGGCTTGGTACTCTCGCTTCGCTACCTTCCTGCAGACTCTTGGG
TTCACGGAGGCCAAGTCTGACACCTCCCTGTTCATATACCACTATGGGGGTGACACTGCTTACCTGCTCCTCTATGTGGATGA
CATTGTTCTCACCGCCTCCAGCGAGTCTCTGCTCCACCGCATCATTGCCTCTCTCCAGAAGGAATTTGCTATGAAGGATCTTG
GGGTGCTCCACCACTTCCTCGGCGTCACTGTTGAGCCCCGACCTTTAGGCCTGTTCCTCCACCAGCGGCAGTTCACTTGTGA
CATTCTGGAGCGAGCCGGGATGACCGCCTGCCAGTCCTGCTCTACACCGGTGGACACCCAGGGCAAGCTGTCAGAGGCTGG
GGGTCCGATGCTTGGGCCAGCAGACTCCACCGCCTACCGGAGTCTTGCAGGCGCGCTTCAGTACCTCACTTTCACACGGCC
GGACATCACTTATGCCGTCCAGCAGCTCTGCCTCTACATGCATGCTCCGCGGGAGCCTCACCTGGCTGCTATGAAGCGGCTT
CTCTGGTACCTCCGTGGCACCCTTGGCTACGGGCTGCTCCTCGGCCGGTCTTCCACTGCCGAGCTGGTTGTCTACACCGATG
CTGACTGGGCGGGATGCCCGGACACCCGCAGGTCCACCTCCGGCTACGCGGTCTTCCTCGGCGGCAACCTGGTCTCTTGGT
CGTCCAAGCGCCAGCCCGTGGTTTCCCGCTCCAGTGCCGAGGCAGAGTACCGTGCCGTGGCCAACGGCGTGGCTGAGGCC
GCCTGGCTCCGCCAGCTCCTGGCTGAGCTTCACCGCCCACTCGCCAGGAGCACGCTCGTCTACTGCGACAACGTCAGTGCG
GTCTACCTCTCCACCAACCCCGTGCAGCACCAGAGGACCAAGCATGTGGAGATCGATCTACACTTCGTCCGCGACCGGGTG
GCCATCGGCGACGTCCGGGTTCTGCACGTCCCGACCACCTCCCAGTTTGCGGATATCTTCACCAAAGGCCTCCCTTCCTCGA
CCTTCTCGAGTTTCGCTCCAGCCTCAACGTCTCCAGTGGCTAGTTGTGGCTGCGGGGGGTCCTGTGTGTTGTGCTCTTACTT
GTTTTCTTTCTTGTTCAGCCTTGAACTCCGCTGCGCCGGTAGTTCAGACTGCGGGGGGGTGTTGGAGTGTGTTTGTATTAAGC
CCATGAGGCTAGGCCCATGTGGCCCATGTATGGTACCTATATAGCTACCCTTCTAGGGTTAGCCCAATTAATGAATCACACATT
CTAGCACTATGGACTAGGTTATCTCTACTGACCACATAAGTGGATCTTAGACATTTGGCTCCCTGTCTGTCACCAGTCAAATG
TTTGAATTAGAAACTCTAGAAAAAAATCTGCGCAATAGATAATAATTCATACCAATGGAATGGTCAGACCACCTGCTTGACCC
CACTAATTAGTGCCCTTGTAATATTCTCACAAAGAGGGGCATAAAATCACTTCTATTGAGCACTGCTCATTTTCCTTGGTTGAA
ATATATATATCTACAATTGGCGAAATATTGTCTGCCATGACCTGTGTATTTAAGTGCTACAGATTCCAGATTGCATGTGAGGCAC
TGAATAGATTCAATAAGAAGTATTTCACTTTTTGAGAACTTGAGTCTACCTTGGGACCCCAGAAATAAGACTGTTTTGTCTGC
AGTCGAAGCACTGGTTGAAGTGAAG
>SsLRR-RLK316
ACTCCCGAACCAGATCACCGTGGCCAACGGGATAGGGCGTTCGGGGTCAGCCATCCAGGAAAAAGGCGGGAAAGCGACGT
TGGCATGCAGCGACCCCCTATCTGGCGTGCCAACTGTCGGTGTTCGGACCCGAGGGGGTCCTAGACCAACGAGTAAATGCT
GCGTGTCCCTTTCCTGGATGGTGATGCAAGGAGAACACACGGGGGTTTATCCTGGTTCGGGCAAGAGAAGGCCCTACGTCC
AGCGGGGGAGGGGAGATTTGTATTTCCTTGCACCGAGGTGCTTGCGTAGGGGTTACAAGCTTGCGAGAGAGGGCGGTGAG
CCCAGGTCTCAAAGAGGAGAAACCGAAATACAAGACGATTGCTTGAGATGTTGATCGCTATCTTGTGTATGGCTTGTCGTCC
GTCGTCTTCTCCAGAGCCCTGGCCTACCCTTTTATAGGACGAGAGGAGGCCAGGGCAACGGATGCCTTGAGATGATGTTTTG
TCGATCACTGTCGAGGTGACTGTAGCGACGTGCCCTAGAAATGGGAGGCCATCCCTGGACACCATGGCGTTATAGAGGACG
GGACACCGAAATGACCCGGAGCGCCATGCCATGCCGTCCTTCCTTCCCTGTTGAGTTGTGCCGGACGTCGAGGCCGGGTCG
AGGCCGGGTCGAGGCCGATTGGATAAGTCCGAATCCCTTGGATCGCGGTGTACGGGCCTTGTCGTATCCTCCCTCGAGGGTA
ACTAGTGACGTACAGTGTGCGGTAAGGTGGGTACAGTGATGTTTTTCACCTCCCATCACGGGATGGACGGTAAAACAGTGA
GGACTGACACTGGTATTGTTCCCACGTCGACCCTGGTTGCGGCCATGCCGGTAGGTGGTAGTAGGTAATTGCATTAAATGCTA
TGACTTTTGTCCCGGGGTAGGTGGAGAAAGAGGGGATCGAGCGCCTCGACCCGGGCTCACACGAGACGGAGTTCGGTCGA
GGCGTTTTGTCGTGCGGGACGGGGCGCGGTCGAGACCTCCCGTCGAGGGGGTCTCATACGAGTCGGAGACGGGATGCGGT
CGAGGCATTCCGTCGAGCCGGCCTCATACGAGGCGGACCTGACGCACGTTTTGAAGTGGGCTGTGTTTTGTCATGGCCTAGT
CGTTTTAGTTTTTATTATCGATGAATGTGGGTACCCCTATACTACTGGTACCGACAATTTATGTCTTCCATTCTTCTCTGTTTGCT
CTCCTCTTACACGCTTTGTTCCATTGATGCAGCAGCCTAGCAGCAATTTCACTTCACAAGGGCTTCTTTTTTATTGAAGTAAA
CGGAATCAGAGTATTCAGCGGCGCATCTGTTCCTGAAACAAAAAAAAATCATGATGATAGATTCAGAAATAATGAATCTATTAG
CTTTTGTGTTACTTCTGTGGGGATGTCAACAGCTTTCTCTTTCCCTCGCTATCGAATCCCAAGGTATGGCTATGGCATTTAACA
TTTTCGCCAAATAAGATGTTCTTGATTAGCTGGCATTAGATAAGCAATTTATATATTTAAACTGAAACTTTGATTTTGAGTGAC
AAGCTTTCCATCAGAGGCATACAAATTATTTGCTATGGACTAGATTATCTCTACTGACCACAGAAGTGGATCTTAGACATTTGG
CTCCCTGTCCATGTCACCGGTCAAATGTTTGAATTAGAAACTATAGAAAAAATCTGCGCAATAGATAATAATGCATACCAATG
GAATGGTCAGACCACCTGCTTGACCCCACTAATTAGTGCCCTTGTAATATTCTCACAAAGAGGGGCATAAAAGCACTTCTATT
GAGTACTGTTCATTTTCTTTGGTTGAAATATATATATCTCCAATTGTCGAAATATTGTCTGCCATGCCCTGTGTATTTAAGTGCT
ACAGGTTCCATATTGCATGTGAGGCACTGAATAGATTCAATAAGAAGTATTTCACTTTTTGAGAACTTGAATCTACCTTGGGC
CCCAGAAATAAGACTGTTTTGTCTG
>SsLRR-RLK317
TCACTCAGCTCAGTGCTGAAGCTTCTCTCCGCTATCCTCTTTCCATGTCTCCGAACAACTGAGTGTTCGTTAAAACTGCTGAG
GTTCAGAGAAAGTTCTAAGAAGCTGGGTGCACCGCAGGTCTGATGCTTCTTCCCCCTCCTCTTGGTAAGTTCTTGCCGGTCT
TCCTTGCTGCATGCATGCCCGTCGTCGTCTTCTTTGAGTATCTGTTTGCTTGACTTGATTGCTAGTTCTTGCCGGTCTTCCTTG
CTGCATGCTCAGTTGCTCAGATACAAGTATTGCCTGTTTGACTTTGTCGTCGTCGTCTTCTTCGAGCATCTGTTTGCTTGACTG
GATTGCTTGCTCTCCATTAGAGCGGTTTCCCCTGCTTCAGGGCTTGTAGGCGCCTCTGCTTCTTCCTTTGCCTTTTGTGGCAA
AACAAATGGAAGGATAGTAAAGCTTCGTTTCAGCCCAAACACTACCAAACAGGAAAACACTGCCTCTAGCGACCGGAAGCT
TCCACCTGCATAGCAAAATGGACAGGAAATGGGTGAGAAAGTTCACCTGCATGCATATCATGATCCCTAGTTCTTGAAACAG
AGTAGGAGCTAGGAAGTGACCTTTGAGAAGTTGCTATGTAAGCGCCAAAAAAGGAGAGACAAAATCATTTTGTCAAAAAAC
AAGGAGACAAAAACATGCTTACTGCTCAACGCATCTTGATGCTGGCAGAGCTGGAAGCGATTCTGCTTTGCTGTACATTCCA
CTACGTGATTAATAAATAAAAGGGGGGCTTTTATCATGTATGAGAATATAGGGGCTGGCTGTAGTAACTGACAAGGTATGTAG
GGTCATTAGGTCTCATTAGATACAGATAATCTCACTGGCTCTGGATTCTGGACTTACATGTAAATGTCTATGCCGGATGAACTT
TTCTTTCTGAGAAATGCAACAGAGGAAACTTCTGGGGACTGCTGCATACTAGTGAGCTACTGAAGTTTAGTTACCACACAGA
GACTCATTTCACACATACAAATCTTTTTCCACTCTCAAATATGATACACCTTTTTTTAACACAAGAAGTCAACTAAACTTTGAC
TGCTGTAGATATAATCACAACAAACTGATGTAGATTCTTGGCATGTTGTTATACCATTCCATCTTGCTTCCAGAATTCCAGTAC
TGAACCATGCCAGATTTTCTTCTCCGAGTATTTCTGTCTTCCATTCTTCTCTGTTTGCTCTCCTCTTACACGCTTTGTTCCATTG
ATGCAGCAGCCTAGCAGCAATTTCACTTCACAAGGGCTTCTTTTTTATTGAAGTAAACGGAATCAGAGTATTCAGCGGCGCA
TCTGTTCCTGAAACAAAAAAAAATCATGATGATAGATTCAGAAATAATGAATCTATTAGCTTTTGTGTTACTTCTGTGGGGATG
TCAACAGCTTTCTCTTTCCCTCGCTATCGAATCCCAAGGTATGGCTATGGCATTTAACATTTTCGCCAAATAAGATGTTCTTGA
TTAGCTGGCATTAGATAAGCAATTTATATATTTAAACTGAAACTTTGATTTTGAGTGACAAGCTTTCCATCAGAGGCATACAAA
TTATTTGCTATGGACTAGATTATCTCTACTGACCACAGAAGTGGATCTTAGACATTTGGCTCCCTGTCCATGTCACCGGTCAAA
TGTTTGAATTAGAAACTCTAGAAAAAATCTGCGCAATAGATAATAATGCATACCAATGGAATGGTCAGACCACCTGCTTGACC
CCACTAATTAGTGCCCTTGTAATATTCTCACAAAGAGGGGCATAAAAGCACTTCTATTGAGTACTGTTCATTTTCTTTGGTTGA
AATATATATATCTCCAATTGTCGAAATATTGTCTGCCATGCCCTGTGTATTTAAGTGCTACAGGTTCCATATTGCATGTGAGGCA
CTGAATAGATTCAATAAGAAGTATTTCACTTTTTGAGAACTTGAATCTACCTTGGGCCCCAGAAATAAGACTGTTTTGTCTGC
AGT
>SsLRR-RLK329
GGGAGAGAAGCATACACCTTGTTCGTTTGAGCATATCAGCAGAATCAGCAGCCATTCAGCAGTGTTTTTCTCTCACAACAAA
CCAGCGAACAGTACTTTCAGCCACAACAAACCACCGAAACGAACAGGCTGATAGTCGTCTTAAACAATTTTTAGGCTCAGA
CCCCAATCCTATTTATTCAGCACCCTATGCTCACCTTCCCTCTTCTTTTCTACTCCTATTCAACTTGCTCCACTCCTACCAAAAA
ACTTATGCCCAAAAGACTGTAGGACCATGTTATGAGATTAGAAGATCTACTAGTGGGATAATTTAGTAGGATAAAGATCTCCG
TGCAGATATGGGTTTGTGAGCCGGTATTCACAGCCCTAGGATTACAAGTGGAGAATCTGTACGGTAGCCAATCCGAGCCGTT

CACGCGCGATCCAATGAGATGGTTAGAATTACCTCCTCCTTGCTTCGGAGCGCTCCGATAAATTTGACCATCCAATTGTCTCC
TCACTCAGTCTCAGCTCAGGGCTGAAGCTTCTCTCCACTATCCTCTTTCCATGGGGGTGTTTACTTCCTTCAAAATTCCAAAA
ATTTTACAAGATTCCCCGTCACATCGAATCTTTGGATGCATGTATAGAGTATTAAATGTAGGTAAAAAAATAACTAATTGCACA
GTTTGTCTGTAAATGTCGAGACGAATCTTTTAAGTCTAGTTAGTCCATGATTGGATAAATTTTATCAAATAACAACGAAAAGT
GCTACAGGAACTTTACACCAACTTTTACACCATCTAAACACTACCCATGTCTCCGAACAACTGAGTGTTCGTTATAACTCCTG
AGGTTCACAGAAAGTTCTAAGAAGCTGAGTGCACCACAGGACTGATGCTTCTCCCCACTTCTCTTGGTAAGTTCTTGCCGGT
CTTCCTTGCTGCATGCAAGCTCGTCGTCGTCTTCTTTGAGTAGCATCTGTTTGCTTGACTTGATTGCTAGTTCTTGCCGGTCTT
CCTTGCTGCATGCTCACTTGCTCAGCTAAGTACTGCCTGTTTGACTTCGTCGTGGTCGTCTTCTTCTTCGAGTATCTGTTTGCT
TGACTGGATTGCTTCCTCTCCATTAGAGCGGTTTCCCCTGCTTCAGGGCTTCCTCTGCTTCAGGGCTTGTAGGCGCCTCTGCT
TCTTCCTTTGCCTTTTGTGGCAAAACAAATGGAAGGATAGTAAAGCTTCGTTTCAGCCCAAACACTACCAAACAGGAAAAC
ACTGCCTCTAGCGACCGGAAGCTTCCACCTGCATAGCAAAATGGACAGGAAATGGGTGAGAAAGTTCACCTGCATGCATATC
ATGATCCCTAGTTCTTGAAACAGAGTAGGAGCTAGGAAGTGACCTTTGAGAAGTTGCTATGTAAGCGCCAAAAAAGGAGAG
ACAAAAACATTTTGTCAAAAAAACAAGGAGACAAAAACATGCTTACTGCTCAACGCATCTTGATGCTGGCACAGCTGGAAG
CGATTCTGCTTTGCTGTACATTCCACTACCTGATTAATAAATAAAAAGGGGGGCTTTTATCATGTATGAGAATATAGGGGCTGG
CTGTAGTAACTGACAAGGTATGTAGGGTCATTAGGTCTCATTAGATACAGATAATCTCACTGGCTCTGGATTCTGGACTTACAT
GTAAATGTCTATGCCGGATGAACTTTTCTTTCTGAGAAATGCAACAGAGGAAACTTCTGGGGACTGCTGCATACTAGTGAGC
TACTGAAGTTTAGTTACCACACAGAGACTCATTTCACACATACAAATCTTTTTCCACTCTCCAATATGATACACTTTTTTTTAA
TACAAGAAGTCAACTAAACTTTGACTGCTGTAGATATAATCACAACAAACTGATGTAGATTCTTGGCATGTTGTTATACCATTC
CATCTTGCTTCCAGAATTCCAGTACTGAACCATGCCAGATTTTCTTCTCCGAGTATTTCTGTCTTCCATTCTTCTCTGTTTGCTT
TCCTCTTACA
>SsLRR-RLK330
AATAACATATAGATTAAATAAAGTGATCAGAGTGATCGTTGATATAAACCCACTAGAATAAATATTATATCACTAATAATAGTCC
AAAGTACACATTATATCAACAAAATTCAAATGTTCGGTTAATTCGGTCAGTTCGGTTACCTGAGACCTATGACCGAACTAACC
GTACAAAGTTCGGTTAGTTGGAAGCAGGGACCGAACTTGTGACCGAAAAGTTCGGTCTCGGTCTCGGTTAGTTTAGTTCGG
TCTTCGATTAATTATGCCGGCAGACAGTGCCGAATGAATTCACTCTAGAAGAATTAGCTACACTTTAATTTGCTAATAACTGTA
CTGATAACAACACACGATTAATCTAAGAAGGGATGACAACACGCAATTATCAACCCTAATCCAGTGGAGCTAACAGACATAT
CCATACACTCAACACTCTTTAGTGTCATGGGCCCATGTCATCATGAAAGGTACATTTTGGCCGAGTAAACAGAAACACGCGG
AGAGCGGAAGACTGACGACCTGTAATTTGTCATCCACGTCTCGTCGTTTCAGTATTAGACTACCCGCACTCATGAACCTCTAT
ATTCATCCTCTATACGTCCTAGTCAGCAAGATTCTCTCCTCTATACTTAATTCTTCCGCACCCATCCATCCTCTATATCATCCTCT
ATCCACTCTATCCACTGTGGGACCCACTTGTCACTTTATCTAAATATATCCATGTGTGATCTAGTTTTGAAGGATTTATTGAGAC
GAATTTAATGGTGCAATCGAAATTTAATTTTGATTTTTGGTTTGTGAGTTATAGCATTTTTCCCTTTGTTTAGCTAGTGGGGCCC
GCCACTATAGAGTATAGAGTAAGCATTGTACTACTCTAAATATAGAGTGAGAGAAAGGATAGAGTATCGTTTTAGAGTACCCA
GTGCATCAGTTTTTACTCTATATTCATACTCTAAAAGTTTAGAGTATCGTTTTAGAATACCCACTGCGGATAGCCTTAGGGAGT
GGGTACTCTAAAACGATACTCTACAGCTTTAGAGTATGAATATAGAGTAAAAACTGCTGCACTGCGTACTCTAAAACGATACT
CTATCTTTTCTCACTCTATGGCCCTATTCGGCAGGCTATTTTTTGGCTTATAAGCCGGCTATGGCTGATTTGCTGTGAGAGAAA
AATATTGTTCCATGGCTGATAAGCCGTGCTGATAAGCTCAGCCGAACATGGTGTATATTTAGAGTAGTACAATGCTTACTCTAT
ACTCTATAGTGGTGGACCCCACTAGCTAAACAAAGAAAAAAAAATGCTATAACTCACAAACCAAAAATCCAAATTAAATTTC
GATTACACCACTAAATTCGTCTCAATAAATGCTTCAAAACTAAATCACACATGGATATATTTAGATAAAGTGACAAGTGAGTC
CCACAGTGGATAGAGGATGATATAGAGGATGGATAGGTGCAGAAGAATTGAGTATAGAAGAGAGAATTTTGCTAATTAGGAC
GTATAGAGGATGAATATAGAGGTCCATGAGTGCGGATAGTCTAAATAGCTCAGCAAGGAGCAGCATCAGTAGGTACAGTGGT
CACAGTTAGACGACTGACGGTCTCCTTAGTTTTGTTCAAGAAACATAACTACTACGCAGCTCAGTTCACTTGCTCCACTAATT
TTGATTTTTTTAAAACTAAAACTTCTGCCATGGTTGAAATCCACGTCGATATTCTTCAGAGTGCAGTCTCATAATAATTGTGTG
GGACCTGGATGCTGCAGAAAAAAAATATATGGAAAAGTCAGGGTGGTTTGGGTGAATTTCCTTGTTCATTAGCAAGTCAATGG
GGAGGGGAAGCATGGTCGTCTTTAAACAATTTTTAGGCTCAAACCCCCATTCCTATTTATTCAGCACCCTATGCGATGCTCAC
CTTCCCTCTTCTTTTCTACTCCTATTCAACTTGCTCCACTCCTAACAAAAATCTTATGCCCAAAAAACTGTAGGACCATGTTAT
G
>SsLRR-RLK246
CACGGACACCCCCTTTTTTCCCCCTCCTCTAAGCCCTTGTCTAGAGCATGTTTAGTTACACCCCAAAATGCCAAATTTTTCAA
GATTCCCCGTCACATCGAATCTTTGGACGCATGCATGAAGCATTAAATATAGATAAAAAAATAAAACTAATTACACAGTTTAG
ACGAAATGGACGAGACGAATCTTTTAAACATAATTAGACCATGAGTGGACAATATTTGTCAAATAACAACGAAAATGCTACA
GTAGCATTTTGCCAAAAAATTTGGCATCCAAACGGTGCCCTAGTTTCCAAAATTTTTTCCAAAAAGTGCTACAGTAGCCATCA
CATCGAATCTTTCGACGCATGTATAGAACATTAAATATAGGTAAAAAATAAGTAATTATATAGTTTGTCTCTAAATGTCGAGATT
AATTTTTTGAGCCTAGTTAGTCTATAGTTGGATAAATTTTTTACCAAATGATACAAGAATTTTTCCAAAAAAATTTTGCAACTG
AACACGGCCTAAAAAAATCATCCCATGGGCTGGCGCGCTGGGAACGACAGCCGGACAGGTGGGTCCAGGCCGGCGCTGGC
TTTTCTGCGGCGGCGCGGGGGTGTAGACCCGGCCCACGGACGGGACGGGGTTTATCTCCCTGTCACGGGACGGGAGTGGAC
TTTTGACTGTTCTCCAGCTGGCCTTGTGCGGAGGCGGGCCCACCGCGCAGTGATCCATTCTCTCCCTTTTCACAGCTCTCGG
GATCCGGACCTTAACTAACCTTTGCCGCGAAACCTGCTGCACTGTAGCAGCACAGCACTGCTGCTGCTGGTGTACGGTACAG
CTCGGATCTCCGGTTTCGTCCTTTGGCATAAGCTAATTTCTCACTTAACATGTCGTGGTGTAACGAAGCATTAGGTTAGCCCG
ATGATTCTCAATTATAATATAGAAAAAACGGGGACCTTTGCATTTTTTTTCTCTCCCGTAGAAATCCAATCACGAGCCGGTGTG
TCCGGTCCACGGACGGATCAAGCAAACGTGATGATTTTCCGAATCGATGCGCGCGCGTGCAGTGGCAGTTGGCCAGCTGCA
GTGGGAGCTGTACGAAGCGACACCATCACTCGGAGAATGACGAGACCGGGCGTACCGAGGACCGTGTCCACGAGATCCCC
GGCGCCCGCAGGCCGCCATGTCAGCAGATGCCCGGGGCCCGCACCTCCCCTGCTCCCTTCCCTCCTCGCTCCTCCCACTGGC
AGACTAGCTCCATCAAGTCACCCACCCCACCACTCCCCGGAAAGCACGCCGCCCGCGCCTTCCCGCCGCGCAGCCCTCTCG
CTTCCACGCGGGCCCCGAGGGTGTCCTAGTCCCGCACGTCGGTGACGTGCTGTGGAGGTCGCCGCGGTGGGTCGTCCCCTT
GCTCCGAGAGGGTAGGCGGTGGTCCCACCGCGGAGTAGAACAACAATTTAGGTCGGCACACAGCCGCACTTCGTTTCCAGG
GCGTTGTCTAGTTCCCCTCAAAATTCCAAGTTTTTTCACTCTCTCTATCACATTAATTTTTAGTCGCTTGTATGGAATATTAAAT
GTAGGGCCTCGTTTAGATGCTGAAAAAATTTCAACCCGATAAATAGTACCACTTTCGTCTTATTTGACAAATATTGTTCAATCG
TGGACCAGCTAGGCTCAAAAGATTCATCTCGTGATTTCCAACTAAACTGTGTAATTAGTTATTTTTTTACCTACATTTACTACT
TCATGCATGGTCCAAAGATTGATGTGATGGAGAGAGTGAAAAAATTTCAACTTTTTGAGTGCATCTAAACGAGGCCTAGGTA
AAAAAATAATTAATTACACAGTTTAGTTGGAAATCACGAGATGAATCTTTTGAGCCTAGCTGGTCCACGATTGAACAATATTT
GTCAAATAAGACGAAAGTGGTACTATTTATCGGGTTGAAATTCCAGCTACAGAGAGAATTCGTCCCCGAAAAGAAAAATAAA
TCTCCCTTCCATTCGAGAGAGA
>SsLRR-RLK326
TAGTTTTAAGAGAACGTGTATGTCAAGCTACATTTTACAAAAGAAATTGGAGGGAGTAGATGTTATTGATTAACTTACATTGA
AATGTGCACACTGCTAACTATTTTCTTGGAAATGGTGTTCAGGTTTTAGTAACCACTCTACCAGTGGCGGACCCAGGAATTG
GGAGCAGGGTATGCCCATGGTAAAATTTTTTTGGATGAACAACACAAAATGCTCAGATCTGCTTAGATAAAAGATACATGTAG
TTCAAACTAAATTTGTTCCATAACAAGTTGATATTACAAAATATTGGAAGAAAATTACAATTAAATTTTAACTCTTCGATCCCT
CATTGCTTGATAGTGACTGATGATGTCATGATCTTTGACTTGCAAAAAGAATTCACGCTCAATAAATGTGACCAAACAATCAT
TCAAGTACTGGTCTCCCATCCGGTTTCTCAACTTGTTCTTCACATAATTTATTATTGAAAACACTCGTTTATCAAAATGTCGCG
AGCGGCGGCGCGCGGTCGAGACTCGCGGAAGCGCTGCTGCCTGCGCGTTAGTGTGCGGCTGCCCTACCCCTACGACCAGGC

CTGATGGTTTTGGGATTGGAACAGCTGAATCTTTGTTAGATGGGCCATGGGCCGTCTGGATTCGCGTGGCTTATGGGCTGCTT
TCGAGTTAGAGGTGAACTGCTGAATAAAATGGCCCAAAAACGCAGAGAAACATAGTTTAACACCTTATGTATATTATACATG
TATATACCTCAAATACCTATTAAATTTTTTTCCAAAAATATAGGGTATGCCCGGGAATACCCGGGCACAACTGTAGGTCCGTCC
ATGCACTCTACTAGCCACCGTGATGACGACGGCCAGCTTGCTCTTTGGCAGGATAACTGCATACTGTTTATCGCTGCTGTATT
ATTAAGTAACAAGAGTATAATGCCAAAACCACCTTGTTGAGAAGCGAGATTTGGCCATGCTTTAGCACTTGTTCCATATAGTT
TTATCCTTGGCAGGTGCCACTAGGGTAGATCTAGCGGTGAGGTGGGCGGAGCTCAAGCTCCTCGTATCAATTCTACAACAAT
AGAGTCCCTCTAACTCGCATCAAATTCTTATAGACATGAAAAGAAAACATGCTTTCAATGTTGAAGATGACATGAGCCGCTTT
TCTACAATTTTTTAGACCCACTACCGTCCGGTATTAGTTTGAAGTCATTGTTCTCACCTACTCCCTCCATCCCAAATTATAAGG
CATTCCAAGATTCAACAGAAGCTCTCAAGCAGAGAATAGCTGGCTTAGGGGATGAATAAAAGAACAACCACACATGATGAT
GAATGGAAGCGAAGTAAGTGATTAGCAAGGGAAAAAATGGAGCACTAGCACGCCTAAACAAGAAGAGATCTTGTGGATGA
TCAGTACATCCGGTTTTCTCGGAGAGTCAAAGTATCTTAAATTTGACCAAAATTATAGAGAAAATTACAAAAATTTATAACAT
CAAATAGGTATACTGTGAAAATATAATTAATGACGAATCTAATGATACTTAATTGGTGTCATAAATATTATTAACTTATCATATAA
ATTTGGTCAAACTTGAAATTCTTTGACTCTCTAAGATTCTTGGAATGTCTTATAATTTGGGATGGAGGGAGTATATAACAGGCC
AGCAGAAAAATTTTGCTAAGCAGTGAAACTGTGAAAGCTAACAAACAGTCTAGAGCGGAAAACCCACAGAGAACCAGTGA
GTCGGTGGTGATCATATAAAACTTCCTGCTGTCCCTGCCCGGTGTTCGGCCATCCGCACGGGATCCCGCGGCTCGCCGCCCC
CGCCGTCACGAGCCCGTTTCGTTGACTCCGTTCCGGCACCGCAGCGCAGTCCCAATCCCAGTCGCACCGCAATCGCAACCC
ACACCCCCCTCTGCTGCGTGCCTGCGTCTTTGGCGCCGCTAATCCTAGCTCTCGAGTCTTGACTTCTAAACCCACGCCACGC
CTGCTTAAACAAAAGC
>SsLRR-RLK248
GAGGATGGCGGCAGGCGGGCGGCTCGACGAGGTGGAGAGAGGAAGGATGGCGCCAAGTAGGCGCCTGGATGCGCCGCAG
CGGTCGTCGCGGACCACGCCGGAATCTGCTGCGACACAGGATAGACAGCGAGGGCCCAACGGGGGAGGCAGCAGCGGCG
GTCAGGTGTGTGCGGCCTCTTGCGGCGAGGTGCCCCCCCGCTGCTGTAGAAGGTCGCGGCAGGCTGGTGTCGTGCCCGCGG
CGAGGCGCCCCCGTGCTAGAGAACGTCGCGGTGATCGGCTGGCGACGCGTGGAATCTACCGCAGCGGTCGAGGGCATGGT
GCGGCGGTGAAGAATGCCGCGACGAGGATTGGAGAATTGGTGCACAACGGTTGTTTATTAATAGAAGCGCAGGTCTGTTGG
GCCGGTCCGTTGCACAAGCCTGAGAAGCCATTTGCCCTGTTTAATTGTCTTTTCAACTGTACTTTTTCAGCGAATGAACGGTG
TTTTTCTCTTGAGAGAATTAAGAATTTGGCCTCCAAAAAACTTGCTCTTAGGAATCAGGCTCTTTTTTTTGAATTTGGGAATC
AGGCCTCAAAACAAAAATCGCTTAGGAATCAGGCCTTCCGTCAATTTTGACACTTAACGGTGTTAAATACGGAGTAGAAAG
ACCATTTTACCCCTGAGTAAAAAATCAAAAAAGAGTTTTTGTTTATATATATATAGAGTATATAATCACATTCAACCACACATAT
ATTCTCGTATATGTATGCTTGTAGTATTATTTTAAGGCACGACCATCCAAAATGTTGTCTTTATCAAAATTCCAAGATTTTTAAA
AATTGTTGTAATTTTTTGCAATAAATTAAATTTCCAAAATTAATTAGCGCTAATGCATGCATAATTATTAGAGGTCAAAAATATT
TCATGAATCAATCAAACTAATAAGATATATGTTGTAAATGATTGTTGCATTCATGCTACAAATAACAGACTCTGTCGTGTGTAC
GGGAAGTGAGGAGCAAAGTGGTCTTTTCGCTCGAGCTACAGTACCCAAACTAACAGACTACACTCACAGACCTGACGGAA
GGCCTAATAACTAAGAATTTTTCTGGGCCATATACCCAAATTTAAAAAAGGGCCTGATACCTGAAACAACTTTTTCCAGG
GCCAAATTCCTAATTCTCCCTTTTCTCTTACAACAAATCAGCCAACAATACTTTTCAACTTGTCTTTTCAGCAAAACGAACGG
GGCCGTCCAAACGGTCAGGCGTCCTAAAGAGAGCATTTCCGCTATATCAACGGAGGCGGAGGTTTTGGTTAATATGAGAGCG
GTAGGGACTAGGGAGTGTTAGTACACACATATTTTCAACATTTGTGCCTAAATAACAAAACCATTTGAGGACGGAGTAATGA
GAAAAGCAGAGAGAGATAAACACAACAACCAGCCGTTTGATTTGAGGAAAGAAAAACAGGAAGGAGCAAGCAGACGAAC
AAAACACAACAACCGGTCAAGGAGAGCAGTTGGCGTAATTGGCAGACCGGAAGCGCCAATTCCCTGGCCAAACCCTTCCA
AGTTTCCGTCCATCCCGAGGAATAGAATAGGATCTCTCCGTTTTTACCCCTCCTCCGTCCTCCCCAGTCTTCTGTTCTAGGACC
ACACACCCAGGAACAAAACCGCGGCGACAAAAAAGAAGGGGAATCGAGGCCGGACGCCAAACACTGGGCACAATTCCCC
ACCCGTCCTCGGCAGCCACCCGCCTGCCCTGCCTCTTAATTAACGTAATCTATTTTTTCAAAAAACTAATTAACGTAATCTACT
CCACTCTCTCGCCTTAATTAAACCCCACTCTAAATCTACTCGCAAATCTACGCAGCGGTGTCCGTCACCGCGCTCGACTCGAT
ACGCCCCCAGCCGGGTAATATACCATGGCCACCACCAACACCACCCACGCCCTCGCCGCCGCCTCCGCTCGCGCTACGCTTG
GCTTCCCCAGTCTCCTCCCCTCCCCACGCCCGC
>SsLRR-RLK309
GATTTGGTAATGCGCACTGTACTAATTCGTTTGTATTTGGTAAAAATTGTCCAAACTTTGACTAATTAGACTCAAAATGTTCGT
CTTGCAAAGTACAATAAAACTGTGCAATTAGTTTTTAATTTCATCTACATTTAGTACTTCATGCATGTATCGACATTTTGATGTG
ATAAGAATCTTCTTTTTGCATAATGAAATTTGGGGTTTAAATTTGTCATCTAAACAAGGCCGAGCAGCAGTGCAGGACGCGG
CTGCCAGAGGCAGTCAGGTTCAGCAGGGCGGTCTGTCTTTTGACCCGTTCCTCCTCCACGCCACGCGACCGAGGGAACCAC
CTATCGCTTGCATGCGGGCCCAGGATATCTTTGCAGGCCCACATGACAGTGGCCGATACCTCCTCTTGCCGCCTGCTCGGGCC
TCGGGGGGACCCACCGACTCGGTTCTCTGACGCCCGGCGCGAAGCAATCAACACCATGCTGATTCCGAATTCCGTTGTCCCC
TCTCCCCTCCGATGAGGAGGGCAAGTTCCAGCAGCAGACTTGCAGTTGGCTTCCCTCTCTACCAGAACTACCACAACAAGG
AAGAAATCAGCGCATCATTCCTTGGGAGCAGGGAGCAAAATGGCGTAGTAGTTGTCGTTGTCTTGTGGGAACCACGGCAGT
TTCTTGCGGGTTTTGATTGGACGGGGCCTCAAACTTCTTCACTGACAGCTTTTGAATAACTACTCGCTGATGACAAGATAGTT
CTTCGGTTCCACTGATGTCTTCTACGGCCACCTAGCGTGGTTGCTCCCAGCTAAGATCCAAATCCTTCAAATCAGTAGTCGAC
TCTTGGCGTTTTTCTCACTGGACTCCAAGGATCGCATTACTTAGGGTTTGTTTAGTTGGACTTGGAGATCAGACTGTATTTGA
CACTTTTGACAGAGAAGGATATTCTCTGCACGGCTCCCGGAGACCAGCTTGGCAGCTACAGTCGATATTATATATATAAACCG
ACAATCGGATCTAAGCTTTTCTGTTATTTGGATTAATTAGCAGTACGGGGCACCAGCTTGACTGAACCTGAACGGATATGCCA
CTTTGACAGCGATCAGCATGGGCTAAAACTGCCATCATCAGCACCACCAGGCTCTTCAGACTTGCGCTACTACCAAGTCCCA
GCTCCATGCAGCACCAATAAAAGAAAGCCCGGCATTGCTCAAAATCGCGCACCCACTGCACGCCGGGTCCCGCGGACTCAT
CAACGGCCCCCCGCCCCTGCCCCAGCCCCGGCCTCGGGCCCCACCCGTCAGGTTCCATGGCATCCATGATCATTTCTCTCTC
ACTGTCACTGCCACCCAAAGATGCCACGGCAATGGCTGAGTGGGCATGCAGACAGCAGACCAGTAGACCACCGCACCGGG
GCATCGGCATCCATCGCATCGCCCCCATTTCCTTCCTGCCGTCTTGCTTTGCATGACACGAGAAAAAGCAAAGCCAAAGGCA
CTCCCGCCCAGCGGCCAGCTCTCACTTTTCTCTCTCCCTCCTCCCCACCGGGCGGCGCCTCTCGCACTCATCACTCCGACGT
TCCCCTTCCCCGAGCCAGCTCTTCCGATGGGTTCCAGCGGCCTCCTCTCCCTCCCCGCCGCCGCCGTCCTCGTCGTCCTCCTC
TCCTCCGCGCCGCTCGTCGCCCTCGCCTCCGAGCCCCTCAACCCCGAAGGTAACGCCGTTCGGCAGCTGCCCATGGTGCAG
CAACATTCATTTGATTGCCCTTCTGGTTTTTGATTCGAGCGCCGCGTCGCGTCGCGAGCTCTGCGTGCAGTGCAGGCGCTGAT
CGCCATCAGGCAGGGGCTGGTCGACCCGCACGGCGTGCTCAGGAGCTGGGACCAGGACTCCGTCGACCCCTGCAGCTGGG
CCATGATCACCTGCTCCGCGCAGAACCTCGTCATCGGCCTGTAAGCAAGCTCTCATGCTCACCTCTCCTCCTCGGCTCGTCTC
GTGACGAGCCGTACGAATAAACTGGAA
>SsLRR-RLK197
GCACGGTTGCAAAAGGATTATCCAAAGCTCGTCCTTACAAAACCAACCAGGCAAATGATGCAGGCTTCCCTTGGACAGGAA
GTTGAGATTTGGACAAGTGAAGCAACATTATGACTCACGCCCCCATGCTTAGAGCTTTATCAAGCATGCTCACTTCTGCTAGA
ACATCAGTAAAACCTCCAGGCTATCATCTTTGTCTACCCTGACGACCTATCTTTTTCAGTTTTTCATGCCCCCGTGTACGCTTC
AATCTCCTTATCTCTTTGCGCTACCAACCAATGCCTGTACGGCATGGTATGGTATGGCACATTGCAGTATTGCAGATTGGCAAT
TGGCTTTTGCTCTTTCACACACACACACACACACATACAATGACATAAAGGAGAGGTGCGGCAGAGGAAAAGAGAAAAGCGA
GGGGGGGGGGGGGGGGGGACAGGCAGAGGCAGAGGCAGCAAAGCATCCCTCATTTCACTGAAAAGAAAGGTCACGTCT
TTCGTGCAAGCTGCCTCGCAATGATTTCAATCCCAAGGACAGGAAGAGGATGCACATCTGCCCATCCCTTCTCCTTTGCAGA
TCAAACAAGGAATAGAGGTTGATGGCATCCTGGATCATCACCAGGCTGATAGCAATCTCTCTGCCCTAGACATCACATAAAC
CTTCAGTCTCCCTTCCTTAAACACCACTCTACATGCGCCCGTCTACTTCTAGACAACAAGTCCCATCACATGACACAGGAAA

GCACGGATGACCCTCTAATAATTGCTTATATATGAAAGAGAAGCTATTAGAATCTCTATCTTTGTTTTCCTTCAAACTTTTTTAG
ATGCCTTTTTTTAGAAAATGGAAAATATCCGACCTCTGCAAGACTACAACCACACATAGCCAATCCTTACAAGATTTTCAGCT
CAAACACTACTAAGAACTTTTACAAGTATACATGTGTGCAAGCATTTGCATGATAGTAATCTTTCTTTTGTACTAACTGCCAAG
TGAAGTGATACAAACCAGACATGGTAGTAACCCAGCGCCCAGGCTACTGCTATTCTGCTACAGTAGACAGAAGGAACATGAT
GGTAGTACTCCTATTGTTCCTCTTGTTCTTGTGACTGGGATTGCGTTGCTTTTAGCTGGTTCTTGATTCTCCATCCACACTGCA
AGAAGACCAAAATTCCCTTGTCCACTTCCCCCTCCACTTCAGGTCAGGTGAGAGCGGAGAGGCACTTCCTCCCTGTCATTAT
CCTCCCTCCTCCCTCCCCACTCCACAGTCCCTCCTACTAAAGACACTTTGCCCCGGTCGTTTATCGCTCACACCAGAATTCTC
ACTTCCCGAGCTAATTGTCCAACTGCCCATGCTTTGGAGAATTCTTTTGTTGCAAGAATCCCCAATCAACTGAAAGGTGCTGT
TGTGAGTCGTGACCTGTCATAGTCTCACTCCACTGCAACACACCCACACTCCAATAAAGCTGATGTCTTTCTTGAGTGGGAT
CAATAATAATCTGTAAACTTTGGACTAGATTAGCCTAGGCACCTTAAGTTAACAAACACACCGTCACTGTTTTTCATAGCCTG
GTTTCGTTCTTCACAGGCCCCAAGAACATAAAAGGGAGGAAGAGAGAGAGAGGGGAGAGGGAGGAGGAATTTTCTTGCCT
CCTTTGCTGTAATTTTCTATTCCAAGCAGGCTCAGGTGGCAGGCATGTTTGTCCCCCCCTCGGCCCTCGCGGCCTCGCCCCCT
CCTTTTGTGCTCCTGCTTGCTTGCGGCCAGAGCACAGGCATCTTCTTTTGTGTCATTTGCATGCTATACTGACAGCTTCTCAG
AGCAGAGGCATCCCGCATCCGGCACCCCTTCCAGGCTTCCACTGCCACTGGCAATTTGGTTCTGTGGGAAGAGAAAGGCTG
CATGATTCTTCCTCGCCTTGGCCTCACCCTTGCTTCCATGTTTGAACACGGAGCAAGCTCCTGAACTCGTTGCCGAGGAGGC
CACGCTCTTGCGCTGCCTTGGCA
>SsLRR-RLK243
ATTCTGGGGCGGTTTAGATGGTGAAAAAGTTTCTGTAAAGTTCCTGTAGCACTTTTCGTTGTTATTTGGTAAAATTTATCTAAT
CATGAACTAACTAGGCTCAAAAGATTCGTCTCGACATTTACAGACAAACTGTGCAATTATTTATTTTTTTTACCTACATTTGAT
GCTCCATGCATGTGTCTAAAGATTCGATGTGACGGAGAATCTTGTAAATTTTTTGGAATTTTGAAAGAACTAAACACTGCCTT
TGTCCTTGGGTCAGTATTCTTGTTGACCTCGAAGATTGCTGTAGTAGTAATGCTGAATCTATGCAGCAGCTGTTGCTGTAGTC
GCAGCAGCCTAAACTAGAACAGCTGATCAGGCCACATCTTCTTCGCAACTACTAAGTGTCATCATTCAGATGATGACCATGC
ATAAGTTGCACGGTTGCAAAAGGATTATCCAAAGCTCGTCCTTACAAAACCAACCAGGCAAATGATGCAGGCTTCCCTTGGA
CAGGAAGTTGAGATTTGGACAAGTGAAGCAACATTATGACTCACGCCCCCATGCTTAGAGCTTTATCAAGCAATGCTCACTT
CTGCTAGAACATCAGTAAAACCTCCAGGTTATCATCTTTGTCTACCCTGACGACCTATCTTTTTCAGTTTTTCATGCCCCCGTG
TACGCTTCAATCTCCTTATCTCTTTGCACTACCAACCAATGCCTGTACGGCATGGTATGGTATGGCACATTGCAGTGTTGCAGA
TTGGCAATTGGCTTTTGCTCTTTAGGCTGCGTTTAGTTGGGAAAGTTTGGGTGAAAAGTTACTGTAGCACTTTTCGTTGTTAT
TTGGTAAAAATTGTCTAACTATGGATTAATTAGACTCAAAAGATTCGTCTCGAACTTTACAGACAAACTATGCAATTAGTTATT
TTTTTTACCTACATTTAATGTTCCATGCATGTGTTTAAAGATTCGATGTGATGGGTGGAAGAGGAAATATTTTGGGTTTGCATG
GCAACTAAACAGGGCCTTACACACACATACAATGACATAAAGGAGAGGTGCGGCAGAGGAAAAGAGAAAAGCGAGGGGG
GGGGGGGGGACAGGCAAAGGCAGAGGCAGCAAAGCATCCCTCATTTCACTGAAAAGAAAGGTCACGTCTTTCGTGCAAG
CTGCCTCGCAATGATTTCAATCCCAAGGACAGGAAGAGGATGCACATCTGCCCATCCCTTCTCCTTTGCAGATCAAACAAGG
AATAGAGGTTGATGGCATCCTGGATCATCACCAGACTGATAGCAATCTCTCTGCCCTAGACATCACATAAACCTTCAGTCTCC
CTTCCTTAAACACCACTCTACATGCGCCCGTCTACTTCTAGACAACAAGTCCCATCACATGACACAGGAAAGCACGGATGAC
CCTCTAATAATTGCTTATATATGAAAGAGAAGCTATTAGAATCTCTATCTTTGTTTTCCTTCAAACTTTTTTAGATGCCTTTTTTT
AGAAAATGGAAAATATCCGGCCTCTGCAAGACTACAACCACACATAGCCAATCCTTACAAGATTTTCAGCTCAAACACTACT
AAGAACTTTTACAAGTATACATGTGTGCAAGCATTTGCATGATAGTAATCTTTCTTTTGTACTAACTGCCAAGTGAAGTGATAC
AAACCAGACATGGTAGTAACCCAGCGCCCAGGCTACTGCTATTCTGCTACAGTAGACAGAAGGAACATGATGGTAGTACTCC
TATTGTTCCTCTTGTTCTTGTGACTGGGATTGCGTTGCTTTTAGCTGGTTCTTGATTCTCCATCCACACTGCAAGAAGACCAA
AATTCCCTTGTCCACTTCCCCCTCCACTTCAGGTCAGGTGAGAGCGGAGAGGCACTTCCTCCCTGTCATTATCCTCCCTCCCT
CCCCACTCCACAGTCCCTCCTACTAAAGACACTTTGCCCCGGTTGTTTATCGCTCACACCAGAATTCTCACTTCCCGAGCTAA
TTGTCCAACTGC
>SsLRR-RLK293
CCGACTCGTACGAGGCACCCCCGACAGGAACGCCTCGTCCGCGCCCCGTCCCGTACAACAAGGCACCGCGCTCCGACTCGC
ACGAGGCTCGTTCGGCCACCGGTAAAACTCCGACTCGCATGACCCCGGGTCAGAGCGCTCGACCCACTCTATTCACCTACC
CCGAGACAAAAGTCAGAGCATTTAATGCAATTACCTACTACCACCTACCGACATGGCCACAACCACGATCGACGTGGGAGC
AACGCCGGTGTCAGTCCTCACTGTTTTACCACCCATCCCGTGATGGGGGGTGAAACCTGTCACTGTACACACCTTACCATGC
ACTGTTTATCGCCAGTCACCCTCGAGGGAGGATACGACAAGGCCCGTATTCCGCGATCTCGGGGACTCGGCCTTATCCGTCT
GGCCACGGCCCGGCCTCGACCCAGCCTCGACGCCCAGCGCAGTTCGACAGGGAAGAAAGGCCGACGTGGCACAGGGTTCC
GGGTCATTTCGTCGTCTCATCCCCGATGTCATCATGGTGTCCAGGGATGGCCAGCCATTCCTAGACCACGCCGCTACAGTCAC
CTCTACAGGGATCGACAAAACATCACTTCAAGACTTCCCGGTGTCCCGGTCTCCTCTTACCCTATAAAAGGGCAGGCCAGGG
CTCTACGGAGGGACGAACGGGCAAGACACAAGTCCAAAGCCAAGTGCCAGCAAGCAAAGGCTCCCAACTCAGAGATCAA
CATCTCAAGCAATCACCCCTATACTCTTTTTTCTCTCCTTGAGACTTGGGCTCGGCGCCCTCTCTCACAAACTTGTAACCCCT
ACGCAAGCACCTCGGTGTAAGGAAATACAAAACTCTCCTCCCCCCGCTGGACGTAGGGCCTTCTTTTGCTCGAACCAGGATA
AACCCCCGTGTGTTCTTCTTGCATCACCATCCAGGTTAGGGACACGCAACAATCACTTGTCGGTTTAGGACCCCCGCGGGAC
CGAACACCGACAGGCGTCGTCACCGGAGACCTCACCGGCGGTGAGCTCCACCACGTACCCGGCGGCGCGGTCACAGTCAA
GGTTGGGCAGGGTAGGTTTGACCGCGGGTCCGCGCGTCAGTGAGAGTGGGTCACGGGGGAGTGAGTGAGAAAAGTTTTCA
ACTGGTTTTTGATTTTTCAGAAAATATAACATGTGCTGAACTTTTGAAATTCATAACTGATTGTTGGAGTGTGATAAAAATGTG
AAACTTTATGTGTAGTGCTCTATCCATGAAAAATATTAGTTGGTCATAATAAGTAATTTTGGTATGCTTAAAAATTCTATCTTTG
CGTGTGTACGGACACGCACGCGTGTGTACGTGTGATGTGAGTATAGAGTGTGTGAGTTGTGTGTGTCCAAGTTGTACCTGAT
TAGAAAAAAATCAATCTCCTCACTCAGCACACGAAAGCTTCCTGCCAAATCAAACTTGTGCACTCTCAACGATGCTCGATTC
AATCCAAATTGAGTCCACCAATGCCTAAATCGAACACAGGTATGGTAATGGTAAAGTTAGCTTGGCCAATGGATAAGCATATA
TGTGCTCGATGAAGATACGAAATGGCTACAAAGATGTTGTTTAGTATGGATCACACTAGATTGCGTGAACATACAAAGCATGG
TGTTCCTTCAAAAAAAAAAACATACAAAGCATGGTAAACAAGTGACATGGCATAAGCACTTACTGTTAGCCATGCTTTGTGG
TAAACAAGTGACATGTTAAACTTGGTAACAAAACATTTCCACCACCAACTCCCCCGCTCGCTTTGTAGCGCTTTGGCCTCTT
CCTCCTGTCTCTGCTTCTGGGCCCGGCAGCCAACCGCTGCCCTTCATCGTCACCGATGCCTGCGGCTAAGTTTTTTTACTGG
TACTTGACCGGCAGGAGTAAACAATGGAGGACGGATGCCCGTGCCCGTGATTTATATAACTCGCTCCGAGCCGAGGAGGCC
ACCGCTGGTGTCGCCCACCGGTCACTGTCCAGGGC
>SsLRR-RLK294
TGTGAAAATGAATTATCACATTTCTGAAACCAAATACCAAATTATTGTGTTACAACTTGTGTGTTTCCCATGTGTCAATCACAC
TGGCTCTTCGGGCTGTTTGTTAGGATGGAGCCCCTTTGTTTTTTAAGAATGTAGTACTCCCAAGGGCTGTAAAATGATTAGTA
TGTTTTTGTGATATACTTACATATGGGTCATGTTCATGCAGACTGGCCTTTGGGGCTGTTCTTAGGTCGGTGCCCCTTTGTAAC
AAGAGGTCCGTATTCCTAACGGTTCAACTGGGGCAGAAAGAGCTACACTTGTAATAATGGTGGACTGATTCCTCTTCTTGAA
GAGGATAAAGCTAAAACTTTCTTCCATTGTCTAAAAAAGGATTGGCGTGCTTTTGTGATATACTTACACTGTATATTCAGGGTA
GATTTGTTTAATGACATGACCAACTGGAAATACATTCTAGTCCAGAAGTTCTGACATACTGTTTGACACCAAAGATGCAATCT
GATTCCTTAAACAGAACAGATTTTGATAGATCTTTTTTAGGCACTATGTTTAGAGAAATAAGTGCAAAAATGATGTATGATTTT
GTTCGGAGCTCTGTTTCTCATTGTCTTAGGGCTTGTAATTCANNNNNNNNNNNNNNNNNNNNNNNNNNNNNNNNNNNNNNN
NNNNNNNNNNNNNNNNNNNNNNNNNNNNNNNNNNNNNNNNNNNNNNNNNNNNNNNNNNNNNNNNNNGCGGCCACGCTGGTG
TCGCCCACCGGTCACTGTCCAGGGCCCATTAATGTCCTCCTGCAGGACAGCAGGAGCGGGCTGCGCTGCTTGTTGCCTTTGC
CTGTGTTGGGCGGGGGAGTGTGGCTGCGTAGGTGAGAGCTCATCCAGTGTGCACTGGGGGCTCTCGCTCCTCCCCTCCTCC

CTCTGCTGTGTCCATTCTTGCCTGCCCCGGCTGCTTGCTAGGTGTTGAGGAAGAATGGGGGAGAGAACGTTGGGGATTCGCG
GCGTTCAGGAGCTGAGAAGCTTCTTGCGATTTAACTGAGCTCTGCAGTTATTCTTGCTCCTGCGTTCCCTGCATTTCAGGTCA
GAAGCGCAAAATTTCTTGCTGCATTTCCAATTAGAGCTATGAATGTCGTTTCTCCTCTGACGCTAATTTGGTTCCACATGAGA
CATGTGCCTTCTCTGCTCTGTACTACGGTGTGATTCTGTTTGCAGACACACTCTCTTTTTTTTTTCTTTCTAATTTCCCCTCTTT
TTTCGCCTTCTTTTTAAGTTCGTATGCTCTGAGAGTCTGAGTTGTTCCTCGCTGCAAATCTCAGTTGCTTCCGGTTCGGGCATC
CTAGTTTGCTAAACTCTCATTTTCCCCTTCAGCTGAATTTCTTCTTTCACCTTAGCGGGATTCGTTTCCAAGCCTGCTGCATTT
CATGGTAGGTTTCGCTGCAATTCTTGCTTGCAAGCTCAGATTTGTTTCTTATTTGTTGCCATTTTTTTTACCAAATCCCTCTCAT
GGGTTCTTGCAAACCACAGTTTCCAACTTGAAATTTGTATTTGTGCCACCAGGGTCTTAAGGAATGGGAATTCCGAATTCAC
ATTCGCAGTACAATCTGCCAGCCACACCTGTCTGTCATTTTCTTTGTCGGGTTCCAAATGATTTATATTTAAACAAAAGAAAC
CAAATATGTTGCTTTGTCTCATCCTACGTTCGATCTGACGCACTGGTTCTTCCGTCCAGCATCCAATGTGGTTTTTCTAATGAT
AAGACAAGGACATGAGTCAGTTTACTGACATGTTTGCCTTGTACTTCTGTTGTTACTATACGTTTTGCTACATCCAAATTGGG
CTGAAAATTGTACTGTAACCTTCTGCAGGAAACTCATATTTGGGAGCAAGCAAGGCAAGGTTTCTCTCCGAGGAAGCATTGT
TCAACTGCAGTGCTCCTGATTTTGGTGGCTTGGAAGTGATAAAGTTTAGCAAGACACTAAGCCAATTGAGAAAGGATAGTGC
TAGCTGAGCTCCAA
>SsLRR-RLK273
CTTTGTGGTAAACAAGTGACATGTTAAACTTGGTATAAAAACATTTCCACCATCAACTCCCCCGCTCGCTTTGTGGCGCTTTG
GCCTCTTCCTCCTGGCCAGTGGTCAGTGTCTCTGCTTCTGGGCCCGGCAGCCAACCACTGCCCTTCACCGTCACCGATGCCT
GCGGCTAAGACTACCCGCAGTGGGTACTCTAAAACGATACTCTAAAGTTTTAGAGTATGAATATAGAGTAAAAACTGCTGCA
CTGGGTACTCTAAAACGATACTCTATCCTTTCTCTCACTCTATATTTAGAGTAGTACAGTGCTTACTCTATACTCTATAATGGCG
GGCCCCACTAGCTAATCAAAGGGAAAAAATGCTATAACTCACAAACCAAAAATCCAAATTAAATTTCGATTGCACCATTAAA
TTCGTCTCAATAAATCCTTCAAAACTAGATCACACATGGATATATTTAGATAAAGTGATAAGTGGGTCCCACGGTGGATAGAG
TGGATAGAGGATGATATAGAGGATGGATGGATGCGGGAGAATTGAGTATAGAGGAGAGAATCTTGCTGACTAGGATGTATAG
AGGATGAATATAGAGGTCCATGAGTGCGGGTAGTCTAAGGGTTTTTACTGGTACTTGACCGGCAGGAGTAAACAATGGAGGA
CGGATGCCCGTGCCCGTGATTTATATAACTCGCTCCGAGCCGAGGCGGCCACCGCTGGTGTCGCCCACCGGTCACTGTCCAG
GGCCCATTAATGTCCTCCTGCAGGACAGCAGGAGCAGGACCGCTGCTGCTTGTTGCCTTTGCCTGTGGCGGGGGGAGTGTG
GCTGCGTAGGTGAGAGCTCAATCCCGTGTGCACTGGGGCGCTCTCGCTCCTCCCCTCCTCCCTCTGCTGTGTCCATTCTTGCC
TGCCCCGGCTGCTTGCTAGGTGTTGAGGAAGAATGGGGGAGAGAACGTTGGGGGATTCGCGGCGTTCAGGAGCTGAGAAG
CTTCTTGCGATTTAACTGAGCTCTGCAGTTATTCTTGCTCCTGCGTTCCCTGCATTTCAGGTCAGAAGCGCAAAATTTCTTGC
TGCATTTCCAATTAAGAGCTATGAATGTCGTTTCTCCTCTGACGCTAATTTGGTTCCACATGAGACATGAGTGCGTGCCTTCTC
TGCTCTGTACTACGGTGTGATTTTGTTTGCAGACACACACTCTTTTTTTTTCTTTCTTTCTAATTTCCCCTCTTTTTCTCCGACT
TCTTTTTAAGTTCGTATGCTCTGAGAGTCTGAGTTGTTTCCTCGCCGCAAATCTGAGTTGCTTCCGGTTCGGGCACTCCTAGT
TCGCTAAACTCTCATTTTCGCCTTCAGCTGCATTTCTTCTTTCACCTTAGCGGGACTCGTTTTGAAGCCTGCTGCGTTTCATGG
TAGGTTTTCGCAGCAATTCTTGCTTGCAAGCTCAGATTTGTTTCTTATTTGTTGCCATTTTTTTTACCAAGTCCCTCTCATGGGT
TCTTGCAAACCACAGGTTTCCAACTTGAAGTTTGTGTTTGTGCCACCAGGGTCTTAAGGAATGGGAATTCCGAATTCACATT
CGCAGTACAATCTGCGCAGCCACACCTGTCTGTCATTTTCTTTGTCGGGTCCCAAAATGACTTATATTTAAACAAAAGAAACC
AAATACTGTTGCTTTGTCTCATCCTACGTTCGATCTGACGCACTGGTTCTTCCGTCCAGCATCCAATGTGGTTTTTCTAATGAT
AAGACAAGGACATGAGTCAGTTTACTGACATGTTTGCCTTGTACTTCTGTTGTTACTATACGTTTTGCTACATCCAAATTGGG
CTGAAAATTGTACTGTAACCTTCTGCAGGAAACTCATATTTGGGAGCAAGCAAGGCAAGGTTTCTCTCCGAGGAAGCATTGT
TCAACTGCAGTGCTCCTGATTTTGGTGGCTTGGAAGTGATAAAGTTTAGCAAGACACTAAGCCAATTGAGAAAGGATAGTGC
TAGCTGAGCTCCA
>SsLRR-RLK285
CGTGTATGTCCAAGTTGTACCTGATTAAAAAAATCAATCTCCTCACTCGGCACACGAAACCTTCCTGCCAAATCAAACTTGT
GCACTCTCAACGATGCTCGATTCAATCCAAATTGAGTCCACCAATGCCTAAATCGAACACAGGTATGGTAATGGTAAAGTTA
GCTTGGCCAATGGATAAGCATATATGTGCTCGATGAAGATACGAAATGGCTACAAAGATGTTGTTTAGTATGGATCACACTTG
ATGGGGTGAACATACAAAGCATGGTGTTCCTTCAAAAAAAAAACATACAAAGCATGGTAAAACAAGTGACATGGCATAAGC
ACTTACTGTTAGCCATGCTTTGTGGTAAACAAGTGACATGTTAAACTTGCTATAAAAACATTTCCACCACCAACTCCCCCGCT
CGCTTTGTGGCGTTTTGGCCTCTTCCTCCTGGCCAGTGGTCAGTGTCTCTGCTTCTGGGCCCGGCAGCCAACCACTGCCCTT
CACCGTCACCGATGCCTGCGGCTAAGGTTTTTACTGGTACTTGACCGGCAGGAGTAAACAATGGAGGACGGATGCCCGTG
CCCGTGATTTATATAACTCGCTCCGAGCCGAGGCGGCCACCGCTGGTGTCGCCCACCGGTCACTGTCCAGGGCCCATTAATG
TCCTGCAGGACCGCTGCTGCTTGTTGCCTTTGCCTGTGTTGGGCGGGGGGAGTGTGGCTGCGTAGGTGAGAGATCAATCCC
GTGTGCACTGGGGCGCTCTCGCTCCTCCCCTCCTCCCGCTGCTGTGTCCATCCTTGCCTGTCCCTGCTGCTTGCTAGGTGTCG
AGGAAGAATGGGGGAGAGAACGTTGGGGGATTCGCGGCGTTCAGGAGCTGAGAAGCTTCTTGCGATTTAACTGAGCTCTGC
AGTTATTCTTGCTCCTGCGTTCCCTGCATTTCAGGTCAGAAGCGCAAAATTTCTTGCTGCATTTCCAATTAGAGCTATGAATGT
CGTTTCTCCTCTGACGCTAATTTGGTTCCACATGAGACATGACTGCGTGCCTTCTCTGCTCTGTACTACGGTGTGATTCTGTTT
GCAGATGCACTCTTTTTTTTTCTTTCTAATTTCCCCTCTTTTTTCGCCTTCTTTTTAAGTTCGTATGCTCTGAGAGTCTGAGTTG
TTCCTCGCTGCAAATCTCAGTTGCTTCCGGTTCGGGCGCTCCTAGTTTGATAAACTCTCATTTTCCCCTTCAGCTGCATTTCAT
CTTTCACCTTAGGCCTTGTTTGATGCATGCACGTGTGTTCAGTGGAATAGAATGTAATTTAGTTTAATTTTACTTCAATCCACT
TCAACATACGTGGATTGAGACGAATACATGCACATCAAACAAGCCCTTAGTGGGATTCGTTTCCAAGCCTGCTGCATTTCATG
GTAGGTTTCACTGCAATTCTTGCTTGCAAGCTCAGATTTGTTTCTTATTTGTTGCCATTTTTTTTTTACCAAATCCCTCTCATGG
GTTCTTGCAAACCACAGGTTTCCAACTTGAAATTTGTATTTGTGCCACCAGGGTCTTAAGGAATGGGAATTCCGAATTCACAT
TCGCAGTACAATCTGCCCAGCCACACCTGTCTGTCATTTTCTTTGTCGGGTTCCAAAATTATTTATATTTAAACAAAAGAAAC
CAAATATTGTTGCTTTGTCTCATCCTACGTTCGATCTGACGCACTGGTTCTTCCGTCCAGCATCCAATGTGGTTTTTCTAATGA
TAAGACAAGGACATGAGTCAGTTTACTGACATGTTTGCCTTGTACTTCTGTTGTTACTATACGTTTTGCTACATCCAAATTGGC
TGAAAATTGTACTGTAACCTTCTGCAGGAAACTCATATTTGGGAGCAAGCAAGGCAAGGTTTCTTTCCGAGGAAGCATTGTT
CAACTGCAGTGCTCCTGATTTTGGTGGCTTGGAAGTGATAAAGTTTAGCAAGACACTAAGCCAATTGAGAAAGGATAGTGCT
AGCTGAGCTCCA
>SsLRR-RLK424
GCTATTTGCCTACGTGGCAGGAAAGAAAATGGGAAAACTACAGTCACCAGCGACAAACTCATCGCCTAGCTCGCACATTCC
CCGTACACTCACTATTTCTTACAGCACACCCACTCCCATAAAGCTGTTTTTTTTCTTATTTCTTTTATTTCCAATCGGCAGGCG
ATTTGCAAATGCTCGTTGAGGACGCCCTAAAAGATCAATGCCATGTCCCACACTTAAATGGCACAACCTGAGTCTTACCTCA
AGTAGTGAGTATACGGTTGAGCTTAAGATCTTCCACAGCCCGATAGTATTTTCTGTTAGTACCTGAGCGTTTAGCTTTATGAAA
AGAAAGCATAAATATCCATCTAATCTAGGTTTTTATAGGAGGAAGTCCATTTTTGGTCCTTGAATTAGTCATATTTTAGCTCCCA
AATTCAAATATCCGACTACTTTGGCATTTCAAGTATCCAAACCAAAGAATTTTATCCCTCGAATTGTTTCTAAACCTGATTTTT
AATTTGCTGATGTGGTATCGAAGGATGTGCTTTTTGACATGCATTTGAATGCAGTTTAACAGCACAGTACAATATCGTTTAAA
AAAAACAGCACAGTACAATACTTTGCAGGTAAAGTCCCGCGTCATCAGGTGTTAAGCAACCTAATCTATTTGGACTCTGATA
CGATCTTCCGGCTTTCGCAATCCCCGAAGCATAACGAACACATGCGCCCCTCACCAAATCCACTCAAACCCCTGTCGTCGCC
CGTAACTAGCAGCCGATTGCAAGTCGTCGCCGACGCCGAGCCGGCCGGTTACTGCCAAATCGTGCCAGTTCTCGCACTGATC
CGCCGCGGAAGGAATCAACGCTAGCCGGCCGGTCTGACGCGTTTGACCCCGGCACTCGGCGGCCCCCGTCGTGGCGCTGCG
GCTGCTGCGGCACGCAGCTTGGGTTTTCAGCAAAGGCGACGAGGAAAAGCGAAGTTCCAAGCATGAGCAGGAGAGCAGTT
CAGTGCCCTTTCGTAGTACTCGATCGATTGACTCGTCGCGTGGAAGTTCGGTGCGCTCCACTCCAGTCCATGGTCCATGCCTT

GCCGAGGAGTGGACTGTGCGATGCGACGAGCCATGTGTGCTGTGTGCATTGCATTGCATTGCTAGTGGTGGCTGCGCTGGCA
TTCCATCGCGTGTGACCGCGATTGCGCGGGCCAAGTCGTGAGTCGTCGCACCTCGCTCTGACTTTTGGAGAGCATCGTTCGC
CGCGTTTCTCTTCTGCGAATCGTGGGTGATGATGCCCAGCGGACTCTCGCGGCCTGCGGGGCTGCGGTTGCCAAAGAAACT
GGCCTTGCCAGGACAAGCCGCCCGGCCGGCCGCCACGATACGCGGCGGCATGCTTCGGGCTCCGCCTTGTGTTCCGCACCG
TCCTTGGAGCCACGCATCGTTTTCACTACTGCACAGACCACACAGCAGAGGCCAGTTTTAATTCCTCCCGACACTTTAAATA
CAACAGCCACCTGGCACTGACATGTGGACTACCACCAGGGATCCACACACCAGTGAAACCGCCCCACCAGGCCAGATGGG
ATCTGCCGTCAGGCCAGATGGGATCTGCCCGACGTACGGTCCCACGCGTTCACGCAAGCCATCCATTCCGCGACGCCACGTC
TTCCGCGGGCCCGGCCCACCGCCTCACTGCCACCGGTATCCCACCCGAGCAGGCGGCCCCACGCGTCAGAGAGTCCTGTAG
CCAGACGCGAGGGAACAGACGACAAACCACACAGTGCAAGGCTGCTGCTAGCGTTAGTGCGACCGTCCCCACTGCAAGTT
CGGCTGGAGAGCACTACTCGCGCTGCTCCGTCATCCATGGCGACCTCCACTCCACCACCTGCCTCCCATGCCTTCCTCCTCC
CCCCACTGAGTCACGCTGACCAGTAACCCCTTCCTCTCCCTCTGTCTCTAGCGTCGACATTCCCCGGCCACCCCTGTCCACA
GCCGTGACAACCGTCTGGGACACACGACACGA
>SsLRR-RLK398
TATATTTAGATAAAGTGACAAGTGAATCCCACAATAGATAGAGTGGATAAAGGATGATATAAAGGATGGATGGGTGCGGGAG
AATTGAGTATAGAGGAGAGAATCTTGCTGACTAGAACATATAGAGGATGAATATAGAGGTCCACGACTGCGGATAGCCTAAA
AGATCGATGCCATGTCCCGCACTTAATTGGCGCAACCTGAGTCTTACCTCGAGTAGTGAGTATACGGTTGAGCTTAAGATCTT
CCACAGCCCGATAGTATTTTCTGTTAGTACCTGAGCGTTTAGCTTTATGAAAAGCAAGCATAAATATCCATCTAATCTAGGTTT
TTATAGCAGGAAGTCCATTTTTGGTTCTTGAACTAGCCATATTTTAGCTCACAAATTCAAATATCCGACTACTTTGGCATTTCA
AGTATCCAAACCAAAGAATTTTATCCCTCGAATTGTTTCTAAACCTGATTTTTAATTTGCTGATGTGGTGTCGAAGGATGGGCT
TTTTGACATGCATTTGAATCCAGTTTAACAGCACAGTACAATATCGTTTCAAAAAAAAAACAGCGCAGTACAATACTTTGCA
GGTAAAGTCCCGCGTCATCAGGTGTTAACCAACCTAATCTCTTTGGACTCTGATACGATCTTCCGGTTTTCGCAATCCCCGAA
GCATAACGAACACATGCGCCCCTCACCAAATCCACTCAAACCCCTGTCGTCGCCCGTAACTAACAGCCGATTGCAAGTCGTC
GCCGACGCCGAGCCGGCCGGTTACTGCCAAATCGTGCCAGTTCTCGCACTGATCCGCCGCGGAAGGAATCAACGCTAGCCG
GCCGGTCTGACGCGTTTGACCCCGGCACTCGGCGGGCCCCGTCGTGGCGCTGCGGCAGCTGCGGCACGCAGCTTGGGTTTT
CAGCAAAGGCGACGAGGAAAAGCGAGGCAATATAGCAGCAATCCATCCAAGTCCAAGTTCCAAGCATGAGCAGGAGAGCA
GTTCAGTGCCCTTTTGTAGTACTCGATCGATTGGCCATGTTTAGTTTTCAAAAAATTTCAAAATTTCTCGTCACATCGAATGTT
GGACATATGGAGCATTAAATGTAAAAAAAACTAATTGCATAGTTCGGTAGAAAACCACGAGACAAATCTTTTAAACCTAATTA
GTTCATAATTAAACACTAATTAACAAATACAAACAAAAATACTACAATAGCCAAAGTCAAATATTTTGGCGAACTAAACACAC
CGTGACTCGTCACGTGGAAGTTCGGTGCGCTCCACTCCAGTCCATGGTCCACGCCTTGCCGAGGAGTGGACTGTGCGATGC
GACGAGCCATGTGTGCTGTGTGCATTGCATTGCTAGTGGTGGCTGCGCTGGCATTCCATCGCGTGTGGCCGCGATTGCGCGG
GCCAAGTCGTGAGTCGTCGCTCCTCGCTCTGACTTTTGGAGAACATCGTTCGCCGCGTTTCTCTTCTGCGAATCGTGGGTGA
CGATGCCCAGCGGACTCTCGCGGCCTGCGGGGCTGCGGTTGCCAAAGAAACTGGCCTTGCCAAGGACAAGCCGCCCGGCC
GGCCGCCACGATACGCGGCGGCATGCTTCGGGCTCCGCCCTTGTGTTCCGCACCGCACCGTCCTTGGAGCCACGCATCGTTT
TCACTACTGCACAGACCACACAGCAGAGGCCAGTTTTAATCCCTCTGAGGCCCGGACACTTTAAATACAACAGCCACCTGG
CACTGACATGTGGGCTCGGACTACCATCAGGGATCCACACACCAGTGAAACGCCCCACCACTTCCTTCGTTTCTGCCGTCAG
GCCAGATGGGATCTGCCCGACGTACGGTACCACGCGTTCACGCAAGCCATCCATTCCGCGACGCCACGTCTTCCGTGGGCCC
GGCCCACCGCCTCACTGCCACCGGTATCCCACCCGAGCAGGCGGCCCCACGCGTCAGAGAGTCCTGTAGCCAGACGCGAG
GGAACAGACGACAAACCACACAGTGCAAGG
>SsLRR-RLK412
CGTCGCCGACGCCGAGCCGGCCGGTTACTGCCAAATCGTGCCAGTTCTCGCACTGGTCCGCCGCGGAAGGAATCAACGGTA
GGCGGCCGGTCTGACGCGTTTGACCCCGGCACTCGGCGGCCCCCGTCGTGGCGCTGCGGCTGCTGCGGCACGCAGCTTGG
GTTTTCAGCAAAGGCGACGAGGAAAAGCGAAGCAATATAGGGCCTGTTTAGTTTGCAAATTTTTTTGCAAAAAGTCACTGTA
GCAGACTGTAGCATTTTCGTTGTTATTTGGTAATTAGTGTCCAATCATGAACTAACTAGGCTTAAAAGATTCGTCTCGTGATTT
ATAGCTAAACTGTGTAATTAGTTTTATTTTTTATCTACATTTAATGCTTCATACATGTGTCTAAAAATTGATGTGACAGGGAATC
TGAAAATTTTTGGAAAAATTTTTGGGACCCATAGCAGCAATCCATCCAAGTCCAAGTTCCAAGCATGAGCAGGAGAGCAGTT
CAGTGCCCTTTCGTAGTACTCGATCGATTGGCCATGCTTAGTTTCCAAAAAAAAATTTAAGGTTTTCCGTCACATCAAATGTT
TGGACATATACATGCACCATTAAATGTAAATAAAAAAAGTAATTACATAGTTTGGTAGAAAACTACGAGACGAATCTTTTAAG
TCTAATTAGTTTATAATTAGACACTAATTGACAAATACAAATGAAAGTGTTACGGTAGCAAAGCCAAATATTTTCGCGAACTA
AACACACCGTGACTCGTCACGTGGAAGTTCGGTGCGCTCCACTCCAGTCCATGTCCATGCCTTGCCGAGGAGTGGACTGTG
CGATGCGACGAGCCATGTGTGCTGTGTGCATTGCATTGCTAGTGGTGGCTGCGCTGGCATTCCATCGCGTGTGGTGTGGCCG
CGATTGCGCGGGCCAAGTCGTGAGTCGTCGCACCTCGCTCTGACTTTTGGAGAACATCGTGGGTGATGATGCCCAAGCGGA
TTCACGCGGCCTGCGGGGCTGCGGTTGCCAAAGAAACTGGCCTTCGCCTTGTTTAGTTACCAGAGTCGGGGGTGGCCGATT
CGGCACAGTGCCACCCCCGACACTGTAGCACTTTTTGTTTTGTTTTTTTTTTTTTGCTAATAATTGTCCAAATCTTAATTAATTA
GGCTTAAAATGTTCATCTCGTAAAGTACAACAAAACTGTGCAATTAGTTTTTTATTTCATTTATATTTAATATTAAATATATGTGC
CGACATTTTGATGTGATGGGGAATCTTCTTTTTGCATAGTAAAATTTGGGGTGAATTCTGGAATCTGAACAAGGCCCTTGCCA
AGGACAAGCCGCCCGGCCGGCCGCCACGATACGCGGCGGCATGCTTCGGGCTCCGCCCTTGTGCTCCGCACCGTCCTTGGA
GCCACGCATCGTTTTCACTACTGCACAGACCACACAGCTGAGGCCAGTTTTAATTCCTCTGAGGCCCGGACACTTTAAATAA
AACAGCCACCTGGCACTGACATGTGGGCTCGGACTACCACCAGGGATCCACACACCAGTGAAACGCCCCCACCACTTCCTT
CGTTTCTGCCGTCAGGCCAGATGGGATCTGCCCGACGTACGGTCCCACGCGTTCACGCAAGCCATCCATTCCGCGACGCCAC
GTCTTCCGCGGGCCCGGCCCACCGCCTCACTGCCACCGGTATCCCACCCGAGCAGACGGCCCCACGCGTCAGAGAGTCCTG
TAGCCAGACTCGAGGGAACAGACGACAAACCACACAGTGCAAGGCTGCTGCTAGCGTTAGTGCGACCGTCCCCACTGCAA
GTTCGGCTGGAGAGCACTACTCGCGCTGCTCCGTCATCCATGGCGACCTCCACTCCACCACCTGCCTCCCATGCCTTCCTCCT
CCCCCCACTGACCAGTAACCCCTTCCTCTCCCTCTGTCTCTAGCTTCGACATTCCCCGGCCACCCCTGTCCACAGCCGTGAC
AACCGTCTGGGACACACGACACGATG
>SsLRR-RLK433
TAAATACCGAAAAGTATCCTTCTTGGCCACCACTTGCCCATCGAGACTAACGTCTCCCTCCTCATGCCTAGTCGCGCTAAAAT
CGCACCTTATGTACTCGGTCTTAGTCCTACTAAGTCTAAACACTTTCGACTCTAACGTGTTCCTCCATAGTTCTAACTTCCTAT
TAACCCCTTCCCTACTGTCGTCAACTAGCACCACATCATCAGCAAAGAGCATACACCAAGAGATACCACCTTGTATGTCTCTT
GTGACCTCATCCATCACCAAAACAAATAAATAAGGGCTTAAAGCTGACCCCTGATGCAGTCCTAGCACAGTACAATATCGTTT
AAAAAAAACAGCACAGTACAATACTTTGCAGGTAAGGTGTTAACCAACCTACACCCTGTTCGTTTCGCTGGTTTATTGTGAC
TAAAAGTACTGTTCGCTGGTTTGTTGTGAGAGAAAAATACTGCTGGATGGCTGCTGATTCTGCTGATCAGCGAAACGAACAT
GGCGCTAATCTAATTGGACTCTGAACTCTGATACGATCTTCCGGCTTTCGCAATCCCCGAAGCATAACGAACACATGCGCCCC
TCACCAAATCCACTCAAACCTCTGTCGTCGCCCGTAACTAACAGCCGATTGCAAATCGTCGCCGACGCCGAGCCGGCCGGTT
ACTGCCAAATCGTGCCAGTTCTCGCACTGATCCGCCGCGGAAGGAATCAACGGTAGCCGGCCGGTCTGACGCGTTTGACCC
CGGCACTCGGCGGCCCCCGTCGTGGCGCTGCGGCTGCTGCAGCACGCAGCTTGGGTTTTCAGCAAAGGCGACGAGGAAAA
GCGAAGCAATATAGCAGCAATCCATCCAAGTCCAAGTTGCAAGCATGAGCAGGAGAGCAGTTCAGTGCCCTTTCGTAGTACT
CGATCGATTGGCCATGTCAGTTTCCAAAAATTTTCAGATTTCACGTCACATCGAATGTTTAGACACATACATAGAGTATTAAAT
ATAGACGAAAAAAAACTAATTACACAGTTTGATAGAAAACCACGAGACGAATCTTTTAAACCTAATTAATTTATAATTAGACA
CTAATTAACAAATACAAACGAAACTACGGTAGCCAAAACCTAAAAATTTTCGGGAACTAAACACACCTGACTCGTCACGTG
GAAGTTCGGTGCGCTCCACTCCAGTCCATGGTCCATGCCTCGCCGAGGAGTGGACTGTGCGATGCGACGAGCCATGTGTGC

TGTGTTCATTGCATTGCATTGCTAGTGGTGGCTGCGCTGGCATTCCATCGCGTGTGGCCGCGATTGCGCGGGCCAAGTCGTG
AGTCGTCGCACCTCGCTCTGACTTTTGGAGAACATCGTTCGCCGCGTTTCTCTTCTGCGAATCGTGGGTGACAATTGTCCTTG
GCAAGGCCAGTTTCTTTGGCAACCGCAGCCCCGCAGGCCGCGAGAATCCGCTGAGCACCTGGCCAAGGACAAGCCGCCCG
GCCGGCCGCCACGATACGCGGCGGCATGCTTCGGGCTCCGCCCTTGTGTTCCGCACCGCACCGCACCGTCCTTGGAGCCAC
ACAGCACAGACCACACAGCAGAGGCCAGTTTTAATTCCTCTGAGGCCCGGACACTTTAATAAATACTACAACAGCCACCTG
GCACTGACATGTGGGCTCGGACTACCACCAGGGATCCACACACCAGTGAAACCGCCCCACCACTTCCTTCGTTTCTGCCGTC
AGGCCAGATGGGATCTGCCCGACGTACGGTCCCACGCGTTCACGCAAGCCATCCATTCCGCGACGCCACGTCTTCCGCGGG
CCCGGCCCACCGCCTCACTGCCACCGGTATCCCACCCGAGCAGGCGGCCCCACGCGTCAGAGAGTCCTATAGCCAGACGCG
AGGGAACAGACGACAAGCCACACAGTGCAAGGCTGCTGCTAGCGTTAGTGCGACCGTCCCCACTGCAAGTTCGGCTGGAG
AGCACTACTCGCGCTGCTCCGTCATCCATGGCGAT
>SsLRR-RLK16
GCTTGAATCTTGGTGTATATACTAATGATCTTCCTAAAAGGGGAAATTGCTAATGCGCTATATGTTTACCTACCAATTTTACCGG
AGCACATGCAAACAGAACACGGATCGCCAAATCACCGCAGCTAAAATGCAGCCCACAGCTCAGTGAATGCTTAGATCTAGC
CAGTTCAATGCTCCAGAAAATGGACAGCCATTCACGTGCTACTAATTCGGCAGGCAGTGAAAAGTCCAACACAGGCTGTGT
TTAGTTCCGGAGCGCCAAAACTCACTGTAGCGACACTGTAATATTTCGTTTGTATTTGGTAATAATTGTCCAATCGTTGACTAA
TTAGGCTTAAAACGTTCGTCTCGCAAAGTACAACTAAACTGTACAATTAGTTTTTTATTTTATCCACATTTAGTACTCTATGCAT
GTATTACAAGTTTGATGTGACGGGAAATCTTTTTTTTGCATAGTGCACTTTTTAATGGAACTAAACACAACCACAATATACAA
ACACAGAAGATACTGAGAAAGGATCACGCACATTCTACAGCCTGTAACATTAGCAGTGTTCCATGTTTACGGTGCATCGAAT
GTCGCTGTACCCAGCCGGTTGACTCGAACACAAGCCAAAGCAGAAGCAGCAGCCTCGTTGACCCTCCTCCACAATCCTCCC
CGTCCGCTGTGCCCATCCATCCCATCAAAAACCCCATCAAACCCCCGGAATCGGCCACTAAACGCCGGCACCCACCCGCCC
AACCCACCGCGCACCAGTTCATCTCATCAGTCATCACACCTGCTCCTGCACACCGCTAGCCACGGTCACAAAATTCCCAGAA
CGGCCTGGTTTAGATTGCAGAATTCACTCTCAAATTCCACTATGCAAAAAGAAGATTCTCCATCACATCAAAATGTCGGCAC
ATATATGGAGTACTAAATGTAGATGAAATCAAAAACTAATTGCACAATTTTATTATACTTTGCGAGACGAACATTTTGAGCCTA
ATTAATTAAGATTTGGACAATTATTACCACAAACAAACAAAAATGCTACAGTGTTCGGGGGGTGGCACTGTGTAGATTTGGC
CACCCCCGAATTCGTGTTCTAAACAAGGCGAACGATTGAGTCGTGGAACGAGAAACGTAGACCTTGAAAAAGATTTTCCAC
TGTTAGGATGAAACTGATCCACGTTTTTAAAACGAGAATGTACGTTCCCGAAACGTGAAACGCGCCACTCTACCCCATTCCC
GTGACTATGCCGCTGGCACGGCGCTCATCATCCGCGCTCGGCTCCTCGCGATCGCGACTCGATCCGGCCCCAGAAACACAGA
GCCGGCGCCGTCAGATCCCAGAAACCCCAAACACCCAGCGGGTTCGCCCCTCCTGCTGCTGAGTAATAAAAAACAACCGAA
CGTGGCAGCAAATCGCGGACAGCGCAGAGGAAGGAAGCAAAAACAAACCCTTTCCATTCAAAGCCCCCGAGAATTTCAAA
AAAACATTTAACAGCTTCCTCCTTCCTTGCGCAAAACAAAACCCCGCAAAACAAGCATCGGCTTCGTCCCTCCTCCACTTCG
TCTCTCTCTCTTCCCCAGCTCCGGCCGCGCCCACCGTCGCAGCCAGCGCGTGTGCTGCTTGCTCTGCGCTCCCCGCCGTCGC
CGTGGCCGCGGGCGAGCTCGAGAGGTGCGGAATTTGGGTGCTGCTCATGCTGGGTTCGAATCGGAGGAGGGAGAAGTGGA
GGAATAATAATTGGGAGGCCGTTGGGGGAGTGTAACTTTGGGCATTAATGGCGGGGTGGTGAGAGACCCCCAGCCCAAGGG
AGGCCTCTCTGTCTCTGGTGGTGGCGCCCTGTAGTTACCTGCGTAGCTCGGCCTCTGCCCCCCTTCGCCTTTATTGCGTTTAA
TTTCGCTCCGAATTCCAGCGCTTCCGCCTCCTCCCCAATCCCATCGACACCTGTAACGCGGAGTGGACATGGGGGACGGCGG
CGGCGGCGAAGGCAGGAGGAGGCGCTCGAGG
>SsLRR-RLK32
AAGAGGGCGCAGGCACCAGCCTGCAGCGGAACATTCCCCCGTGCGCCCGGCGCGCGGACGTGCAGATGCTTCCACTCCTC
CGCGCTTTCCATGCTCCAGTCTCCAGCATCAGCCGCCGCGGGGACGGCAGGGCGGCAGGCGGCAGCAGCCACCGGTTCGTT
GAGCCTGAGCCGGGCCATGGGCACGTCAGGTGGTTTTCTCGGAGACCTCGCGCGGCGGGCGCCGCTGCCGCGTAGGCGGC
CGGCCCACGCGCTGGGTGCGCCGTGCGCGGCGTGGATCCCGTGTCGCCCTGTCGCCCGTGACCGCGCGTACAGTACGTTCT
GAGCTCCACTCCACGCATCGGTGAGAGGGCGCATGGTGGCTCCACGTAAGCAGCCGCCAGGTGGATTCTCAGCTGTGCCAG
ATCGGCTGGTTAGACCAGGGTCTTTTGCGACGTAGTACTAGTACGCGGTACGCCTGCTTCGATATGGGCTGGCATGCCGCCCT
GTGGCTGAAAGTCTGGATGAGTGGATGTTACTCCAACGTTTTAGCCTGTAAAAATGATGAGCAATTTCTGCAGGGTTCCTTC
GGAATACAGCTAGTAGGATTCTGTAAGCTGTTCCAGCTCATCAAAATGCTTGAATCTTGGTGTATATACTAATGTTCCACCTGG
CAATTTTACCGGAGCACGCGCAAACAGAACACAGATCGCCAAATCCCCGCTGCTAAAATGCAGCCCACAGCTCAGTGAGTG
CTTAGATCTAGCCAGTTCAATGCTCCAGAAAATGGACAGCCATTCACGTGGTACTAATTCGGCAGGCAGTGAAAAGTCCAAC
ACAATATACAAACACAGAAGATACTGAGAAAGAATCACGCACATTCTACAGCCTGTAACATTAGCAGTGTTCCATGTTTACG
GTGCATCGAATGTCGCTGTACCCAGCAGGTAGATCGGCTGGGCAGGGGCCGCAGGGCGCAGGAAAAAAAAAACGAACCAG
AAATGAGAAATCCACCGGCCGGCGGCCGCCCACGAATCCACAACCCCGCAGGCCGCCCGTGCTGCCACGCCGGCCGCTCG
CAGCCGCAGAGCCGGTTGACTCCCAAAGCAGAAGCAGCAGCCTCGTTGACCCTCCTCCACAATCCTCCCCGTCCGCTGTGC
CCATCCATCCCATAAAAAACCCCATCAAACCCCCGGAATCGGCCACTAAACGCCCGCACCCACCCGACCAACCCACCGCGC
ACCAGTTCATCTCATCAGTCATCACACCTGCGCCTGCACACCGCTGGCACGGCGCTCATCATCCGCGCTCGCGACTCGATCC
GGCCCCAGAAACACAGAGCCGGCGCCGTCAGATCCCAGAAACCCCAAACACCCAGCGGGTTCGGCCTCCTGCTGCTGAGT
AATAAAAAACAACCGAACGTGGCAGCAAATCGCGGACAGCGCAGAGGAAGGAAGCAAAAACAAACCCTTTCCATTCAAA
GCCCCCGAGAATTTCAAAAAACATTTAACAGCTTCCTCCTTCCTTGCGCAAAACAAAACCCCGCAAAACAAGCATCGGCTT
CGTCCCTCCTCCACTTCGTCTCTCTCTCTTCCCCAGCTCCGGCCGCGCCCACCGCCGCAGCCAGCGCGTGTGCTGCTTGCTC
TGCGCTCCCCGCCGTCGCCGTGGCCGCGGGCGAGCTCGAGAGGTGCGGAATTTGGGTGCTGCTCATGCTGGGTTCGAATCG
GAGGAGGGAGAAGTGGAGGAATAATAATTGGGAGGCCGTTGGGGGAGTGTAACTTTGGGCATTAATGGCGGGGTGGTGAG
AGACCCCCAGCCCAAGGGAGGCCTCTCTGTCTCTGGTGGTGGCGCCCTGTAGTTACCTTCGTAGCTCGGCCTCTGCCCCCCT
TCGCCTTTATTGCGTTTAATTTCGCTCCGAATTCCAGCGCTTCCGCCTCCTCCCCAATCCCATCGACACCAGTAACGCGGAGT
GGACATGGGGGACGGCGGCGGCGGCGAAGGCAGGAGGAGGCGCTCGAGGTG
>SsLRR-RLK64
TTCTGTATTTTTTCATGTAAAATTTTTGTGCGATTCCTATGAAATTCTTGCATTTCAAACGGATTCAAACTACTTGATAATCAAT
CTTCAGTCTAACCCCCCCAATCCCACTAAACTACTCTCTCTCCTTTTAAAATTATTGGTTGTTTTGGCTTTACTGAATATAACAT
TTTGTTATGTACGTAGATGCATAGTAACTTGTAACGTACCTAGAAAAATCAAAAGAACTTGCTACCTTTTGAACTTTTGGAGA
GCATTTGATTAGTTCGAGTATATTTGTTGAATGGGACAGGCATGCATTCAGCGAAGTGACATGTAGTCTAGAGCCTACATGAT
TATCAGTTTTGTCTAGGACAGAGGATTGAATTCTAATTCGGACCAACTTTAAACTTGCGGCATCTCCTAATAATGTGAATTTGA
GACCACTTAGAGTTAATTTGGACTCTGAACTTGGCTAGTTTAAGATAAGCTCATCGATCTCGTCGGCAGCGTGTACTGTGACT
GAACTTTCCTAGTGACACCTAGCTAGCTTGTCACAATCTGGAATAACCAGTCGTCATCAGCCATCGTTCTACAAGACCCAAA
CGACACACCGGAGACTTGGACCCGGCGGCCGGCCAGTCGGCAGCGTGGATCATTCGCCGACACGACGCATCAAATCATTCA
CGCATCAACTGGCATCTGAAAGGATGCATAGACTACAAAACAGAGACCAAGAAATTAGCAAGAAACCTGACGGCACTGGA
GCATCGTCTGTCAGCTTCTTCTTCCACTTATTCCACAAGCATTTGTCATTTATTGATACTTTCTCACGAACAAGCAGGTTTGAC
TGGTTGACTAGTTCTATTTGAGTACATTTTCGTCTCTAATACGTAAAAGGGATGTCTAGAGACATATTACAGGTTCCAGTCTTC
TCAGTGTTCTCGTTGGCCAGGAGAGATCGAGCAGCAGTGGGGGAAAAAAAGATTAGTTAAGCAAACCCAGAAACCCAACA
CACTTGCCTGGAGACTCGAGTCACGACTCATTACCGTATTTGTGCAGTCTAGTAAAGAAAGCTGTGCCCTTCCACTTAGACT
TTGTTTGAACGTTGTCGTATTCACCTCAATTTATATGGCCCTGTCGTTTCGTTGCAATTTAGCTTATGCTGAACTGCTATGTGAA
AAGTATCGCTGAAGTAGTGCAGCAGAATAGGACCATGTGTTGGTGTGGTTTGGGAAAATTTAGTTCAAGTTCCACTCTAATC
GACCCTAACCCATATGGATTGATGCAAATCCGACTATGTCCGAAGAATACCTTAACAACAATTTTTAAGTAAAAAAAAATGGC
GTAATCCTAACACCGCACGTGCACATATGAGGCACGTTGTACTGTACCCACACGGCGCTGTTCATTGTTGCCTCGTTGCACA

GGAACGATGGCGACGCAACCTTTTGTTTCGTTTGCCTTGCGAGATGCGAAAACGACAGCCACCGAACATGGTGGGGGCGTG
TGAGAACATTCCATACACGCACGGGCGAAAAGGGCCATCCATCCAGTCGCGTCCTCCGACCCACATGTCAGCGGCACGATG
CCATGGCACAGTTACACTAGTACAGCTTGCACACAAAGCACTCGCGTAATCGCGTTCGTCGGGTCAGAATCCATTCGCGCCG
CCCCTGCCCGCCACCCGCGGGAGCGGAAGTGAGCGACGTCTCCACGCATCTGCACCCGCCGCTGGCGCTGCGGGCCTTCTC
CCCGCCACCCCCCACTCCCACTCATCCCGCACCCGCGTTCCCCGTGGTCAACTCGCTGCCTCTCCAGCGACGACCGTGAGG
GGGCCGAAATAGGCAGGCAGGCAGGCCACCCAAAAAAGCGAGCACCCTACACGCACCACCTCGGCTGCCATTGCCATTGC
CATTGCCATACCATGGCCACGCCATGACGCCCCGCCCCGCTGCACGCGCTCACACCCCCACCGCTCCACTCCGCTCCGCCTC
ACCCTCACCCACCACCACCTCCCC
>SsLRR-RLK18
CGCACAGAGCTCTCTGGTGAGCAGGCCTCCGGAACTTCTCCCGTCAAGCGTACCTGCTCGGGTAGGCGGGAAATCAAGTTT
TTGGGGAGCGCTTCCTAGGCGCGACTGCTCTGGTGCTCTGGTGCCGGTGACAAGACTGACTGCTCTGGTGCCGGCGACAAG
GCTGTCTTCTGCTGTTGGACTCTGCAAGATGGTGCTGATGAGAACTGGTGCCGGCAACAACATCGCCACTGGCGATGGTGG
CAACGACGACAATGGTGGTGGTTCACTGTCCGCGGGTACCAGCAGTACCACGCTAGGGTATAAACCGAAACCTCCTCTGTTT
GTACTTCATATTATTATTGCTCCTGCAGTTCTGTTGTGCACAATAAGCTGTGCTGGAAGTAGTAGCAAATATAATGCTTTTCTGT
TATGCATGATTAGCAGTAGCAAATATGAAGTTGTCGTTTGCATGATACTTGTGTTAGTATTTTGCTTATCATATTTATGGATTAAT
GCATGGTGTAAAATGACTAAATATCCAACACTATGATTATCAGTTTTGTCAAGGACATATGATTGAATTCTAATTTGGACCAAC
TTTAAACTTGCGGCATCTCCTAATAATGTGAGTTTGAGACCACTTAGAGTTAATTTGGACTCTGAACTTGGGTAGTTTAAGAT
AAGCTCATCGATCTCGTCGGCAGCGTGTACTGTGACTGAACTTTCCTAGTGACACCTACTAGCTAGCTTGTCACAATCTGGA
ATAACCAGTCGTCAACAGCCATCGTTCTACAAGACCCAAACGACACACCGGAGACTTGGACCCGGCGGCCGGCCAGTCGG
CAGCATGGATCATTCGCCGACACGACGCATCAAATCATTCACGCATCAACTAGCAGGCGAAAGGATGCATAGACTACAGAAC
ACCCAAGAAATTAGCAAGAAACCTGACTCACGGCACTGGAGCATCGTCTGGCAGCATCTTCTTCCACTTATTCCACAAGCAT
TTGTCATTTATCGATACTTTCTCACTAACAAGCAGCTTTGACTGGTTGACTAGTTCTATTTGAGTACATTTTCGTCTCTAATACG
TAAAAGGGGTGTCTAGAGACATATTACAGGTTCCAGTCTTCTCAGTGTTCTCGTTGGCCAGGAGAGATCGAGCAGCAGTCGG
GAAAAAAAAGATTAGTTAAGCAAACCCAGAAACCCAACACACTTGCCTGGAGACTCGAGTTACGACTCATTACCATATTTGT
GTAGTCTAGTAAAGAAAGCTGTGCCCTTCCACTATTTTGTTTGAATGTTGTCGTATTTATCTTGTCTTATTCGTTTCGTTGAAAT
TTAACTTATACCGAACTGCTATATGAAAAGTATTACTGAAGTAGTGCAGCAGAACATAGCCATGTGTTGGTGTGGTTTGGAGA
AATTTAGTTCGAATTTCACTCTAATCGACCCTAATGCACATGGATGACAAATCCGACTACGTCCCAAGAATACCTTAGCAACG
ATTTTTAAGTAAAAAAAAATGGCGTAATCCTAACACCGCACGCCCACACATGAGGCACGTTGTACTGTACCCACACGGCGCT
GTTGCACAGGAACGATGGCGACGCAACCTTTTGTTGCGAGATGCGAAAACGACAGCCACCGAACATGGTGGGGGCGTGTG
AGAACATTCCATACACGCACGGGCGAAAAGGGCCATCCATCCAGTCGCGCCCTCCGACCCACATGTCAGCGGCACGAAGCC
ATGGCACAGTTACACTAGTACAGCTTGCACAGAAAGCACTCGCGTAATCGCGTTCGTCGGGTCAGAATCCATTCGCGCCGCC
CCTGCCCGCCACCCGCGGGAGCGACGTCTCCACGCATCTGCTGCACCCGCCGCTGCCGCTGCCGCTGCCGCTGCCGCTGCG
GGCCTTCTCCCCGCCACCCCCCACCACTCCCACTCGTCCCGCACCCGCGTTCCCCGTGGTCAACTCGCTGCCTCTCCAGCGA
CGACCGCGAGGGGGCCGAAATAG
>SsLRR-RLK19
ACTGCTCTAGTGCCGGTGACAAGGCTGTCTGCTCTGGTGCCGGCGACAAGGCTGTCTTCTGCTGTTGGACTCTGCAAGATG
GTGCTGACGAGAACTGGTGCCGGCGACAACACCGCCACTGGCAATGGTGGCAACGACGACAATGGTGGTGGTTCACTGTC
CGCGGGTACCGGCAGTACCATGCTAGGGTATAAACCGAAACCTCCTCTGTTTGTACTTCATATTATTATTGCTGCTGCAGTTCT
GTTGTGCACAATAAGCTGTGCTGGAAGTAGTAGCAAATATAATGCTTTTCTGTTATGCATGATTAGCAGTAGCAAATATGAAG
TTGTCGCTTGCATGATACTTGTGTTAGTATTTTGCTTATCGTATTTATGGATTAATGCATGGTGTAAAATGACTAAATATCCAAC
ACTATGATTATCAGTTTTGTCAAGGACATATGATTGAATTCTAATTTGGACCAACTTTAAACTTGCGGCATCTCCTAATAATGT
GAGTTTGAGACCACTTAGAGTTAATTTGGACTCTGAACTTGGGTAGTTTAAGATAAGCTCATCGATCTCGTCGGCAGCGTGTA
CTGTGACTGAACTTTCCTAGTGACACCTACTAGCTAGCTTGTCACAATCTGGAATAACCAGTCGTCAACAGCCATCGTTCTAC
AAGACCCAAACGACACACCGGAGACTTGGACCCGGCGGCCGGCCAGTCGGCAGCATGGATCATTCGCCGACACGACGCAT
CAAATCATTCACGCATCAACTAGCAGGCGAAAGGATGCATAGACTACAGAACACCCAAGAAATTAGCAAGAAACCTGACTC
ACGGCACTGGAGCATCGTCTGGCAGCATCTTCTTCCACTTATTCCACAAGCATTTGTCATTTATCGATACTTTCTCACTAACAA
GCAGCTTTGACTGGTTGACTAGTTCTATTTGAGTACATTTTCGTCTCTAATACGTAAAAGGGTGTCTAGAGACATATTACAGGT
TCCAGTCTTCTCAGTGTTCTCGTTGGCCAGGAGAGATCGAGCAGCAGTCGGGAAAAAAAAAGATTAGTTAAGCAAACCCAGA
AACCCAACACACTTGCCTGGAGACTCGAGTTACGACTCATTACCATATTTGTGTAGTCTAGTAAAGAAAGCTGTGCCCTTCC
ACTATTTTGTTTGAATGTTGTCGTATTTATCTTGTCTTATTCGTTTCGTTGAAATTTAACTTATACCGAACTGCTATATGAAAAGT
ATTACTGAAGTAGTGCAGCAGAACATAGCCATGTGTTGGTGTGGTTTGGAGAAATTTAGTTCAATTTCACTCTAATCGACCCT
AATGCACATGATGACAAATCCGACTACGTCCCAAGAATACCTTAGCAACAATTTTTAAGTAAAAAAAAATGGCGTAATCCTA
ACACCGCACGCCCACACATGAGGCACGTTGTACTGTACCCACACGGCGCTGTTGCACAGGAACGATGGCGACGCAACCTTT
TGTTGCGAGATGCGAAAACGACAGCCACCGAACATGGTGGGGGCGTGTGAGAACATTCCATACACGCACGGGCGAATAGG
GCCATCCATCCAGTCGCGCCCTCCGACCCACATGTCAGCGGCACGAAGCCATGGCACAGTTACACTAGTACAGCTTGCACAC
AAAGCGCTCGCGTAATCGCGTTCGTCGGGTCAGAATCCATTCGCGCCGCCCCTGCCCGCCACCCGCGGGAGCGACGTCTCC
ACGCAGCTGCCGCTGCCGCTGCCGCTGCGGGCCTTCTCCCCGCCACCCGCGTTCCCCGTGGTCAACTCGCTGCCTCTCCAGC
GACGACCGCGAGGGGGCCGAAATAGGCAGGCAGGCCACCCAAAAAAGCGAGCACCCTACACGCACGCACCACCTCGGCT
GCCATTGCCATTGCCATACCATGGCCACTCCATGACGCCCCGCCCCGCTGCACGCGCTCACACCCCCACCGCTCCACTCCGC
TCCGCCCTCACCCACCACCACCTCCCC
>SsLRR-RLK24
GTATTTGAATTAAGGTGATATTAGTTTCAAACTACTTTGCTTCCGCTACTTTTGAACTTGAGTTGTAATAACTCTACTGAACTC
CGATATATGTTGAATTACTTATGAAATGGATGTAATATATGACCTGTTATGTTGGACCACTACGATCTTGGCTTGTATGTTGGTT
GGTTTGAAATCCCTCGGGATTTTATTGGACTACCGGGGTTATAGGAGCTTAAGTACGGGAATTTAATTGCTCTGGCGATTGTTT
TTGTACTTTTGCTCTTATAATTTGGGCGGTTCCGTGACACCGGCAGTACCATGCTAGGGTATAAACCGAAACCTCCTCTGTTT
GTACTTCATATTATTATTGCTGCTGCAGTTCTGTTGTGCACAATAAGCTGTGCTGGAAGTAGTAGCAAATATAATGCTTTTATGT
TATGCATGATTAGCAGTAGCAAATATGAAGTTGTCGCTTGCATGATACTTGTGTTAGTATTTTGCTTATCGTATTTATGGATTAAT
GCATGGTGTAAAATGACTAAATATCCAACACTATGATTATCAGTTTTGTCAAGGACATATGATTGAATTCTAATTTGGACCAAC
TTTAAACTTGCGGCATCTCCTAATAATGTGAGTTTGAGACCACTTAGAGTTAATTTGGACTCTGAACTTGGGTAGTTTAAGAT
AAGCTCATCGATCTCGTCGGCAGCGTGTACTGTGACTGAACTTTCCTAGTGACACCTACTAGCTAGCTTGTCACAATCTGGA
ATAACCAGTCGTCAACAGCCATCGTTCTACAAGACCCAAACGACACACCGGAGACTTGGACCCGGCGGCCGGCCAGTCGG
CAGCATGGATCATTCGCCGACACGACGCATCAAATCATTCACGCATCAACTAGCAGGCGAAAGGATGCATAGACTACAGAAC
ACCCAAGAAATTAGCAAGAAACCTGACTCACGGCACTGGAGCATCGTCTGGCAGCATCTTCTTCCACTTATTCCACAAGCAT
TTGTCATTTATCGATACTTTCTCACTAACAAGCAGCTTTGACTGGTCGACTAGTTCTATTTGAGTACATTTTCGTCTCTAATACG
TAAAAGGGATGTCTAGAGACATATTACAGGTTCCAGTCTTCTCAGTGTTCTCGTTGGCCAGGAGAGATCGAGCAGCAGTCGG
GAAAAAAAAAAGATTAGTTAAGCAAACCCAGAAACCCAACACACTTGCCTGGAGACTCGAGTTACGACTCATTACCATATT
TGTGTAGTCTAGTAAAGAAAGCTGTGCCCTTCCACTATTTTGTTTGAATGTTGTCGTATTTATCTTGCCTTATTCGTTTCGTTGA
AATTTAACTTATACCGAACTGCTATATGAAAAGTATTACTGAAGTAGTGCAGCAGAACATAGCCATGTGTTGGTGTGGTTTGG
AGAAATTTAGTTCAAATTTCACTCTAATCAACCCTAATGCACGTAGATTGATACAAATCCGACTACGTCCCAAGAATACCTTA
GCAACAATTTTTAAGTAAAAAAATGGCGTAATCCTAACACCGCACGCCCACACATGAGGCACGTTGTACTGTACCCACACGG

CGCTGTTGCACAGGAACGATGGCGACGCAACCTTTTGTTGCGAGATGCGAAAACGACAGCCACCGAACATGGTGGGGGCG
TGTGAGAACATTCCATACACGCACGGGCGAAAAGGGCCATCCATCCAGTCGCGCCCTCCGACCCACATGTCAGCGGCACGA
AGCCATGGCACAGTTACACTAGTACAGCTTGCACAGAAAGCACTCGCGTAATCGCGTTCGTCGGGTCAGAATCCATTCGCGC
CGCCCCTGCCCGCCACCCGCGGGAGCGACGTCTCCACGCATCTGCTGCACCCGCCGCTGCCGCTGCCGCTGCCGCTGCGGG
CCTTCTCCCCACCACTCCCACTCGTCCCGCACCCGCGTTCCCCGTGGTCAACTCGCTGCCTCTCCAGCGACGACCGCGAGG
GGGCCGAAATAGGC
>SsLRR-RLK409
AACACGCACTGCAAAGGCGCGGCTCAAGTAGATATATAATTCTTAACTCAACGGTCAGTCAAAGGCGCGGCTCAAGTAGAC
GCGGTCACCGGTCAAGTGCACACACACTGCCGAGGTGGTTTCGCTCTCCCGTAGGCTCGGCAGTCGCCATCGGCCATCGTC
GTCAATCGACGTTGTACTGCGCGTCTAGATCACCCGGTTTTGGAAGGATCCTGGCCATTTCGGTCCCCCGAGAGACTCCCCG
GCTACTGTACTACTGAGAGCTACTAGAGCACGCAGCTACTACCAGCCAGTAGTAAACGCAGAGGTCACGTTCCAACTTGTG
GCCGGGGTAGTTGGTCAAACTGCGTGCCTCGTCGTCACTCGTCAGAAACCAGATGAAACGTGTTACGAGCAGCCGGCCGGC
CAGCCGCTAGCGGGCCAGGACAGGAGGAAACGAGACGAATAGGAAACAAAACCCGGCAAGAACAGCTATGCCCACGAGG
GCGAGCGGGAGGGAGGCGCAGCGCAGGGCCGCAGGCCGCGACCGCAACATCCGTCCGTCGTCCACGCGCAGGCGGGCGC
ACATGCCTCCCCCCCGCGGTCCACGCCGGCCGCACACTGTTGCCGCCGAGGACCAGCCAGCAAGCCGCGAGCCGGCACCC
CCACGAGTCCCCCCCCGCCTCGTCACCCGGGCCCCGCCCAGGCGCTAGCGTGAGCCGACGCGCCCAATCATCCCATCCCCA
CCCCACGCAGCGCAGTAAACAAAACGAAACAAAGCCACCCCCGTGCCGTGTCTCGGCCAACGCCCGACGCCGAGGCAAGC
GGGCCCACCTTTTTTACCCACGTTGTTGCGTTGCGTTGCGTTGCGTGCTCCCCTCCTCCCCTGCTGGTGCCTGCTAGGCAGCC
AGCCGCGCGCTACTTGCCTTGCCAGTTGCGTCGCTGCTCGTCCCAGTCCCATACCACACCACCCCAACCATCTCCTCTGTCTC
TGCCCCCGTCTCCGCCTCGTCGCTTCCGTCCACTCCTCCTCTGCCTGCCTCACGCCCCCATTGCTCCGAGCGCGGCGGATCTA
GGTGACCGCCGCCGCCGTCCGAGCGGTTGTGATCCGACCCGTGGGGCGCGGCGCCGCGAATGGTGTGATCGACCCCGTTGG
GAATTGGGAGCGAGGCCATGCAGCGGGCTCCGGAGCGCCGCCGCGGCGCCGGGCCGCTCCTGCTGCTGGCGCTGGTCGCA
TTGGCGGCCGCGCCGAGGCTCGTGCGCGCGGTCACCGACGCTGCTGACGGTACGCTCGTGGCTTGTGCTTCCCCCCTCCCC
TCCCCTCCCCTCCCCGCCTTGCCCCCGTCTGCTTGCTCTGCTGCTCGCAGCGAGTTGGTTCCGTGGACTACCGCTTCGCCGCC
GCTCGATCGTGCGCTGGTTTGGGGTTTTGCGTGCGCGGGTGGGTTGTTCCGGTCTTGTCGGTGGTACTCTACTGCGAAGCGA
AGCATCCAGCCACCCAAGCAGGGGGATCAGATTGATTCTGTGCGGTGCTGACGTGCTGGTGCAGCTGTGTGTGCTGCCGGG
GAGCGGCGATTCGCGTTGCTCTCGCACTTTGCTTTTCTCGCCTGCCCGCCCACCTGTAGTTAAGGATATATGCGGTGCGGACG
TGCGGTGAAATTATGTCGGTGCCGGCGCTGCTGTGGTGCGGTGACAAAGTGCGGAATGAGGAGGGCCTCTGCTTTTGCGGA
TTATTACAACTTCGTCTCGACTCCGTGAGCGAACCCGACATCCGAGCTCGCTCGCTGGCTCGCCCGACTGTTAAGTTTTTGTC
ATCGAACCTGCAACCTCGTGTCTGCTTGCTAGTGCAGGGCGGCTGTAATTTTGATCGCTTGGTGTTTTCATTCTGTATTTCTGT
TGGTTTGTACCACGGGGGCCGTCCCTTTCGGCTGGGATAACGAAAGCAACTCTTGAGGCGTTATCGAAACAAGCTGCCTGC
CCTCTGCTCCTGTCTTATCTCTTATGACAGCAATGACAGACGTGGA
>SsLRR-RLK85
TCGATCGGGCTGGAAAGACGGGACAAAAGGCTCACGAGCTAGTAGGAGGGAGTACGCGATCATTCCGTGACCCCATCCATT
TTGTCCATTGGCCTGCGCCAATGCCATCATCGTTGCCAGAAATCGAAAGCCGAACGGCTCCTACTCACTCACTCGGGACCTG
TTGAATCCATCGTATTTTCACACGCCTCCTTGTGCCTAGGCCCCACAGTGACCCTCGACACAGATTTTGGCAGCAGGATTTTG
ATTTCGTTCTTTCTTTTTCTAAAACATCCACATGCACGCCATAAGGATGCACATATGCACGTGGTAGCTTATACAAACGTCGGT
GTGTCTGGACCACTGATAGATCTCACTGTTTTCTACGCCATATTAGTAAGACACAAAACGTTAAAACTAGGGTTTAATCCTCT
CCTATGGCTATTGATATGTAGAACTACCTTCACTAATAAGCATCTAAGTTATTTCTTGTTTCTCGGATTATTTGCTCTCTTCTTAG
GCCTTCTGCATTTGTATGAATAAATTGGTTCACTATTTGGTGAGCTAGATGGCATGCGAATTTGACAAGGTCATTTATATTTAC
GAGCAAATATACATGTGCATTGTAATGAAACATACAACTTGTAAATGCGCTGTGTTCGAGATAAGACCTCATGTTGGATCTAA
ATTTGGAACATGTGGACGACAAGAGTGAGATGTGACGAGAATTGGAAACTTAAAGGGTTGTTTGACACAGTTGTAAAGACC
TTGAGCGTACACATTGTATAATAAAAACATATTTATGTAACACATACATGATTAATCTAAAATAATGTCTTTTTTGACCATAAAT
AACATCAATGTGGCCATTCCCGTTGCTCAAAAATAGTAGGCGGTTAGTAGTAAGAAAATTCTTGCCCCTAAGACTTTTTTTGC
AACCTAAAATCCCATGAGCTGCTCCGTACAGCTGCTGTCGCCAGCACTGCTCATTCCCAGCGGCGAGCCGATGGACACTGGC
GCTGGCAGCGGCAGCAGCGGTGAATTCAAAAGCGGCTCGTTTCGGTGGGGGCTTCTGCGGCCCTGCCCCGAGTGCCCCAG
CTGTTCCGCTCGCCTCGCCTGCGCGCACTCAACACGGACGCTCCCCACCCTCGCCGCAGTCCCGCCGCATTACACGTCCCGC
TTTGTCTCCTTTTCCGGCGCCCGCGACGGACTGCGACGAGCGGATAGATAGATCTGTGCTGTGCGTGACGACCCAGCTCTCT
ATCTATCCCCGTCCCTGTCTCGCCATCCTCCTTTCTCCTCCTCCTCCTCCTCTACTCCCCACCGAGCAGAGCGAATCCTTAGCG
GGCAAGTCTTTCCGAGCTCGTGGAGTGGGGTTTAAAACGCGGCCCGAGAAAAGGAGGACCGCACTCGCTGCTGCTGGTCG
CTTGCTTTCGGTGGCGAGCGTCTCGGTAAAGCGGCGTCGGCGTCGGAGGCGGCGGCAGAGACTGGACTTGCTTGCTGCGCA
GTGCAGTGCTGCCTTGGAGACAGGATCCTGCTGCAGGTATCAGGGCTTCTTCTTCGCCTTTCTTCTTTCTGGATTTTGGAGGT
TGTTTGGAGTCCCTGGTTTTTCCGGCTCTACTTCTCTCGTCCCTGCTTCCGCTTTGCATTCCCACCTATGTGCTCTCTTCCATG
CGAAGAAGATGTTAAGGCGTTAAGCGATTTTTTTGGAAAGATATGTTAAGCGAACTTCAAGTGATGCTTTCATACGCACAG
GCGAGAAGAGACTGCCTGAAAACAATTGCAGTATAGGAAGCGTCGTGATTTCTTTAACACGCGTAACCGCAAGAAGCGAGA
ATTAGTTTTTTTCCCCTCCAGAAGATGTTGTGAGCTTGTCAGTTTGATACTGAATTTTGGTCCTTTTTCCTTTTTGCGTTCCG
AGAATCTTCCCGTATCAGCATTGTTTACTTTCTCGTGCGCCTCTCGTCCCTTTGTCCCCTGCGTTCTACTGTTCCCTCCTTTTTT
TTTTGACCCCAGGCT
>SsLRR-RLK214
TACAGATAGCGAAGGTTCGTCAATGAGCTAAACGATCTTGGTAGGTCACCATTGAAAGCATTGAATGACAGATCCCTGAAAA
ACAGGGTAGGGGACAAACTTCACATGAGTCGATGAAGATCAAATAAATTGGGACATTTTTGTTTGCAAGGATGGTCAGGATA
TGAGAATGCCATTGCCATGAAGGGTGGAAACGAAACTGCTCTAGCATCAAAATGTGCACTTTTTCAACAAACAAAAATGAA
CTGGATCCTTGGAAACGGACAGGTGGAAACCTGTTTGAAGGAAGCACAGAACAGATCTTAACCAAAAGAAAAAGAAAAA
GAAAATAAAGTGCAGACATTTAGGACAGCTTGCTGAGCACACACAACATATTCTGCATGATAGGAACAAACCACACCAAAC
CATCCATTGTGAATGACAAAACATCATACAGACATCGGTCCGATGCAAGAAAACATGTATGGGCACAACTGAGTAACCACAA
GGACCATATCCACAAATGTATTAGATGAATAAAAGCTAGCAAACGAATGTGTAGACACTGTATTAGACGAATAAAAGCTAGC
AAACGAATGTGTAGACACTAATGTACTGCTGAAGTTTCTACTGTAGATGCATACATNNNNNNNNNNNNNNNNNNNNNNNNN
NNNNNNNNNNNNNNNNNNNNNNNNNNNNNNNNNNNNNNNNNNNNNNNNNNNNNNNNNNNNNNNNNNNNNNNNNNNNG
GATTCAACACGGTCTGGAGGGTCGGATGGAGGTGGCGGATCAGGAGGGACATGCTCCATTGCCGAGGGGACCGGAGCATT
GGGGTCGTCGTCGCTCATGGCTAGGAGCGGCAGGAGGAATCGAAGAACAGGAGCATGAAAGGAAAAAGTGGTATTTGAAT
GCAGTTCATAAAGCATGATAGCTGGGGAGATTCACAGAGGGAGGGCCACAAGCTCGAGGGGAGTAAGACGGGCGGCGTGT
GGCACGGAACGGCAGAGGGGCGGGCAGGTGTCGACGCCGCCGTGCAGGATGAGGGGCAGGTGTCAATGCCACCGTACCTG
GAGCAGGAGCATCGGAGACTCCGGTGAGTGGTGGATCCCACCGATGGAATGAGCAGAAGGAGTCAAAAAACCATGGATCG
GAATCAACACAGCACGAAGATTAGGGATCTGCAATTATTGACGCAGATTGAGGAGATGAAGATCACGGATTGTGGAGGATTA
GGGAGGAAGGCAAGAAGGACACGGCAAGTCGCCCAAAACTACTCAGAGACTTCCGAGCCAAGCATCGACAAACCGGCGT
TACCCCCCTCTGAATAACAATTTTAGTTCCTTAGTCTATATTTAATACTCCATACATAAATTAAACGTCAAATATAAAGGGGACC
CAACTTTAGGGCGCAACTAAACAAGCCTGAGTTTTGTTTCACCACAGCGCTAGTTTCATTGTGTGTTTCTGCTGTGCGTTAA
ACGGGGCGACTGATGCTCCTGTAACTTTAAGACACTCTGACGATCTGCTGAGACCACTAACATATGTGACATGGCACATGTT
CGATGACATGGAAGTTGAGGCTGTACGCCGCCTCGGTGCGTTGAGTCTGGCCTCACGCGCCGACATGTGACAAGTGACAAC
CGCCCATCCTCCGCACTCTTTCGTCCCCTCCTTTCCCTCCCAAAACCGAAGCCCTCCATCCGAATCAAACCAAAACGGCAAA

ACCCCAAAACCCTCACCATCTCGGAAGCACCAGCATCATTCCGCAGGCCACGCCGGGCGATTCGCCCCGCTACGGCTGCCC
TGTTCCTCCAATCCGCGATCTGGTACGTGATCCGAGCGCGATCTCGCGGGTCGGTGCGGAATTGGGGGAGGAATTCAAACGG
TGCCGGAAGCGCGCGGTTCCCCGGAAGCTTCCAGAACGTTCGGGGCCTCCCTGCTCCGGTCCGCGCTCCGGATCCGACCCA
GATTCCGGGGGCGGATTCCGCGGGGGGATTCGCTGGGTGCGCGCGGGAGGGGTTGGA
>SsLRR-RLK239
CATTAAATATGATAGGACTAAACTTTAGGAGCCACAGCGCTAGTTTCATTGTGTGTTTCTGCTGTGCGTTAAACGGGGCGATT
GATGCTCCTGTAACTTTAAGTTTGACATTCTGACGATCTGCTGAGACCTCGGTGCGTTGAGAGTTTATGGAAGTTGAGGCTGT
ACGCCACCTCGGTGCGTTGAGTCTGGCTTCACGCGCCGTACGTGTGACAACCGCCCTTCCTCCGCACCCTTTGGTCCCCTCT
TCCTTTCTCTTTCTGACTGTTCGCAGTGGGATACTCTAAACGATACTATAAAGCTTTAGAGTATGAATATAGAGTAAAATCTGT
TGCACTGGGTACTCTAAAACAATACTCTATCTTTTTCTCTCACTCTATATTTAGAGTAGTACAGTGCTTACTCTATAGTGGGTAG
TGACGGACCCCACTAACTAAACAAAAGAAAAATGCTATAACTCACAAACTAAAAATCCAAATTAAATTTCGATTGCACCATT
AAATTCGTCTCAATAAATCCTTCAAAACTAGATCACACATGGATATATTTAGATAAAGTGATAAGTGGGTCCCACGGTGGATA
GAATGGATAGAGGATGATATAGAGGATGGATGGGTGCAGAAGAATTGAGTATAGAGGAGAGAATCTTGCTGACTAGGACGTA
TAGAGAACGAATATAGAGGTCCATGAGTGCGGATAGGCTTAGTCTATTTTTAATACTGCATATGTCAAGCATACTAACTTTAAA
GGGCGTAACTAAACAAGCCTGAGTTTTGTTTCACCACAGCGCTAGTTTCATTGTGTATTTCTGCTGTGCGTTAAACGGGGCG
ATTGATGCTCCTGTAACTTTAAGACACTCTGACGATCTGCTGAGACCAGTAAGAGCATCTCCAAGAGTTTGCTATCTTAACTT
GGCATCCATATAATTTTAGCAAAACAAGAAAAAATAGTCACCAACAGTTTGGCAAAACAACTTGACATCAATAGCAAGTTGG
CAAAAACGCCTCCTCAACGCGCAAATATACGCGCTCATCGCTCGCGCGCATCCTCGTGCGCATCCGGTGATTCTGTGACAGC
TTGCTTGTGCCTCTCCCGAGCCTGTACATTTTGTCGAGTTGTCTGCTACTCCTAGCTAGTGCATCTCGGTTTGTCTTGGCTGCT
AGCTGGTGCATGTCGCCAGCATTAGCTGTTGCGCTTTGTCTGATTGATGCCGTTGTTTCTTGTTTACTGCTGCTACGGATCCAT
GTCCCCTCGTTGCTGCTCTTTGACGACAAGCAAGCACATACAGTAGAAGTATACGTACCTGACACCAAATGAACATGTTTCC
AGCTGGGACGGTGACCGGGAAAACAGATGGATCGCACGGGAAAAATTAGCCCATGGGCCTTCTTTTTTTTTTTGTTTGCCAA
GTCAATAATAGCAAACTATAGGAGGGGGACAGTTTTTTTACTTGGCATATAGTTTTGGGAGCCAAGTAGAAATTGACAAACTC
TTGAAGATGCTCTAACATGTGTGACATGGGCACATGTTCCATGACATGGAAGTTGAGGCTGTACGCCACCTCGGTGCATTGA
GTCTGGCTTCACGCGCTGTGACAACTGACAACCGCTCTTCCTCCGCACTCTTTCGTCCCCTCTTCCTTTCCCTCCCAAAACC
GAAGCCCTCCATCCGAATCAAACCAACGGCAAAACCCCCAAAACCCTCACCATCTCGGAAGCACCAGCATCATTCCGCAGG
CCACGCCGGGCGATTCGCCCCGCTACGGCTGCCCTGTTCCTCCAATCCGCGATCTGGTACGTGATCCGAGCGCGATCTCGCG
GGTCGGCGCGGAATTGGGGGGAGGAATTCAAACGGTGCCGGAAGCGCGCGGTTCCCCGGAAGCTTCCAGAACGTTCGGGG
CCTCCCTGCTCCGGTCCGCGCTCCGGATTGCCCGAGCCGATCCAGGATTTCGCGGGGGACCGGCGCGGCGGATTCGCTGGG
TGCGGGCGGGAGGGGTTGGA
>SsLRR-RLK107
TGGTTGTTGGATTCATTTCCGAGTATTGTCAGCTTAGGCGAGATTGGTTCTAGATTTGGTAATGGGAGCCCGCGGTGCAATTT
ATTCCCCGATTCGGTGATGCAAGTTTCTTTCTCATTTTCCCCTCCCTTGTGAGGGGACGCATGTTGTTCAGTTGACTAGTACTT
TGTTCTAAGGTTTCACTAAGCTTTATTTTGATGCTCCGAATGTTTAGGATATCCGAAAGTTAATTTCATCCACGCCATTAGCCT
GTTTTTGTGAAGCAGTCTTCTCTTTAGGAGACAAATCTGAACTTTGCGTGACAATCTTCTCTTCCGTTGGACTGTGCAAAGA
ATGCCGACGCCTTTGCACACCTGAATGGAAATTCTTAGTTAAGCAGTAAGCTAAATACGTGGCTGTTACAATTTGGATGCGCA
TGTTAGTTTCCCTTCTGTGCCAAATTCACTTATTTTCCCCAAACCGAGTAATTATATGTGAAGAATGCAAAAGGTTATTTGTAG
GTGTCGTATAGGTCTTTTTTTACTGTCAGTATGTATATTATCCAGATTGCTTGGTTGATAAGCAATCAGGATCCCTGTTGCTATG
AATATCAGATGCTATTCATCAGTGAAAGACTGTCATTCGTTCTGTTTAGACTGTGGTGCTAGTTATTTACACCATTGAACAGGA
AAACTGTGGTCTTAGACTCTTATTTTAGCGATTAGCGAAGTGTAGTGCATATCCCCCATCCCCACATCTGACTTGATATATTGC
CCTGGTGCATGACAGTGATAATGGGATAATAATCTGTTCTCTTCATTTCTGCTTGCTTATTTCAGATGGAGGATGGCGAATCTA
CTTATACTGTGGATGAGGCACTTGTGTCTATGGGGTTTGGGAAATTCCACGCATTCGTTCTTGCATACTCTGGAATGGCTAAA
ATCTCTGAAGCAATGGAGATGATGCTTTTGTCATTTGTTGGGCAATCAGTTCAAGCTGAGTGGGGGCTTTCTGCACAGGAAG
AAAGTCTCATAACAAGTGTTGTCTTCGTGGGGACAAGTGTTGTCTTCGTGGGGATGCTTGTANNNNNNNNNN
NNNNNNNNNNNNNNNNNNNNNNNNNNNNNNNNNNNNNNNNNNNNNNNNNNNNNNNNNNNNNNNNNNNNNN
NNNNNGCACCAGATTGTTACATTACTGTACTATTGCTTTTCAATAGAGATGTACAATTGCTACATGTTTTTGTTCTCTCCAAAT
GGTATTAGAGCAGCTCCAGTGTAGTAAAAAAAGGAGAGAAAGAGCAGGGTTCGCTTTTATACACTGCAGGGATCCAACTCT
GGGGAACACTTCTTTGGATCAGTGCAAGCAATTGTTTATTTCAACTTAACACCCATCTAAATCGAACTTTACACAACAGCAA
GCGAACCATGAGAAACATCGGCTTTTACTGGGCCCACAGCTTATAAAACCGAACTCTACACTGGAGCTGCCCGTCTGAATTC
TTAACGTCTTGAATCTTAAAACAGTTTGAAAAGTAACATGTTTTCTTGTCCGGATCATGTGTGGGAAAAAAAAATACAAACA
TGAAGCTGGAAAAAATTAAGATGTGGAAAAAATACAAAAATGAAGCTGGAAAATATAAAAAGATTTCCGTTGCCGGGACTC
GAACCCGGGTCTCTCGGGTGAGAGCCGAGTATCCTAACCAGCTAGACTACAACGGATTTTGTTGATGGTAGACATGATATGC
TTTAATTAATTAGTGACATTAGAGCCATTCAAAGCAATGGTTTGGCGACACTCTCGCTGCTCCAATGGCGACGGTCTCGTTCC
ATCTCTGTTCCCCGTCTCTTCCTCCGTCTCCTAATTTTGGGAAGGCCGCACTTTCCCACCGCGAGACCTGTATCCCCATGAAT
CCATGAGAGAAAGGCGGAGGAGATGGGCGACGTACGCGCGGGGGTCTCCTCCTGCACGCTCTCAGCTGAACCGCGCCGTG
TTCCCCTTGGCGCGCAATGGC
>SsLRR-RLK29
TGAACGTCTATGTAGCTAAACGGACATGAGTTCTTACATTGTTCAAGACCAAATTATCTGAGATTCTATACAATCTAAAAAATG
CAAAAGACAAAATTAAAATTTTAAATAGTTTAGAAATCACAGAGGTAGTAGCGATGTTTCAATTTATGCTCCAAATATTATAGA
TGGTTTGTCATGCAAATGACACCATATTAGGAGCCCAAGTCTCTCTCTCTCGGAGGTTGGGCTATGATTTTTAAAGTTGAAAC
TTTGATAATTTTGGAGCTACCTGCTTCTACAGTCTTGCAGTGGCGGCTGGCCGGCTGCAGTGCAGCTGTCAGATTTCTTCATT
ACGGCCTTGCTTAGATTCCAAATTTCATCCCAAATTTTACTATGCAAAAAGAAGATTTCCTATCACATCAAAATGTCGGCACAT
ATATGGAGTACTAAATGTAGATGAAATCAAAAACTAATTGCACAGTTTTGTTGTACTTTGCGAGACGAATATTTTAAGCCTAAT
TAATCAATATTTAGACAATTATTATCAAATAAAAACAAAATTGCTACAGTGTGACAGGACATTTACTATACAAAGTTTGGATCC
CAAATTTGGATCTAAACAAGGCCTACATGAACAATAGAGGTTGTATGCTTGTTGTAGCAGTAGCGACAATATTGCTGTGACAA
GTACATCCAGCTCATACAATATTTTTTTCCTCCTCTAAACAAAACAAGTACTTTATATATCTGTTTTTTTAAAAAAGATTCACAA
AATATTAGTCCGCAAACAGAAAATTCCGTTGTTCATGTAGAAAATCATATGGCCCTCTTTCATAAGATAAGTGTATATCTCATAT
TTCAAGGAGTAAACTAATTCTAAATTTAACTAGATTTATAGAAAAGTAGTGTTAACATTTGTATATCTAAATTGTTTTACTATAA
AAACATATGGAATGGCTAAACTAATAATGTTTATGTGATATTACAAATATTAGTGGGTATATATAGTCAAAATTAAAATTAATTTA
CTCACCGAAATTCGAGATACGGAACGAGGGGAGGAAGTTTTTGTGCGCTCCAGGATCCGAGCTGCAGAATCCTAGAAGCTA
ATCGATTCCAACCGTTCGATTTAAAGCTGTTGATCGGTCAGTACTCACTACTCTAATGGGTCCCTGGTTTGCGGGGCCCCACC
AAACGTAGCCTCGGGTGCTGGCCTCGGCTGGCGCGCCGGGCGCAGCAGCTCGCAGAGTCGCAGTGCTCCGCTGCTCCCAAT
TAAAATATACTCCCAAAGAATTCGGCAGCGCCGAGTGAAGAATTAAAAAACAGGGAAAACCCGAGAAAATCGAGTGTACTG
ATTGCTGAAGCCAAAAGGCGGGATCTTTTTAGCCGTGAAAGCAGCTTTCTTGCGACCATAGTTGTGAATTCCATTCCGGTAC
CGGCCGGAATGGCCGGAATGCCTCGTACCGGCCTCTAAACCGGAATAGAAGAGCTCCAATCCCGTACCGGGTCAAATACCG
GTCAATACCGGTCAACGCCGGTCAATACCGGTCAAATACCGGTCAATACCGGCCAATACCGGCCAATACCGGTCAAATACCG
GTCAATACCGGCCGTGGCTGCATGTATTATATTTTTCCATGTATGAACTTTGGAGTTTAAACTTTGAAATAAGGTCTTATGAAC
TTGTTGAATGATATCTTAGTTTGGTAAAAATGTTAATTACGCCATAAGTTAAGTCAAAATATATTTATATATACATTTATGTATAA
GTTTATTCACATATTATTATTATTATTATAAAGTCCGGAATGGTACACCGGTATGGGCCGGTACCGGAATGTTCCATTCCAATAG
CCAAAACGAAATGGGCTCCGGAATGGAATTTACAAGTTAGCTTGCAACTCCCATGGCCCTATAGAGAGAGAGAGGAGAGCG

AGCGAGCGGGAGAGATTGTGCAGTGCTGTGGCCCTGTGAGAGAGAGGACCTGAGGAAGAGGGAGGAGGCTTGGTGGCGG
AGCA
>SsLRR-RLK58
TAATAGCCGTATTTTATTTCTTATCAACCTTCTATGTAAGCACCTAGTCTATAATCAAGCAGGGTTAAGTAAAAGAAATCATTTC
TTCATCTTTCAACTGCCAGTTCTTACTACGATGCCAGAGCGTAGTCAAGCCGTACCGTCACGCAGAAACGGCGATTCGCGAA
TCAATATGCCCAGCTGGGTGCCCCGAAAACACACGCCTTACTCGTACCCCGGGCACAAGCAAGGTCAACCCGTTCCACTCC
TGTCAAGGGGTCCAGGTCCCCGTCCAAGCTCGGACTCTAAGCCCCCAACACTGTAGGCCCGGCCTCAGTATGGTGCTTAGA
CCTCCTCAACCCAATATATAGGACCACCCTAAAAAGCCAGTCCGGAAAGAGCCAGAACCCATGACAAGAGGGAAACAAGTC
TTCCCGCCCCCATAAGTAAGTATATGCTCGGGATAATAAATCTGTGACTTGACCAGGGTCCATGCAACGAACGGTCCTTAATT
GACATAGGCGGAACAAATGCAACTAGAGCCCTGCTCTATTGCCCAACCAGGTCCAAGTCCATCCAACCCGTTCCGCTCGGTC
TCCAATTATCCACATATATTTATTTTCCATGTGATAGTAACATGATAATAGTAACAATATTATAATCCCTATCTCTCGCGAGTGAC
AGGTAATCACTCGACTTCTACCGGAATCCTATAGCATAGCACACTACACGATCCTATCATACTAGTAAGACTTATAGGATATTAT
ATATATATATATATATATATATATAAGTGGGTTTCATTTAACTCTTTTAAACTTAATGCACAAGCATAATTTAAAGTGCAGAAGAA
TAGGGGTTATGCACCGGGGCTTGCCTGGGTAGAAAATAATCAAATTGGCATTCCATCATGGTGAACAATCAAGGCGTCATATN
NNNNNNNNNNNNNNNNNNNNNNNNNNNNNNNNNNNNNNNNNNNNNNNNNNNNNNNNNNNNNNNNNNNNNNNNN
NNNNNNNNNNNNNNNNNNNNNNNCGAAATTCGAGATACGGAACGAGGGGAGGAAGTTTTTGTGCGCTCCAGGATCCGAG
CTGCAGAATCCTAGAAGCTAATCGATTCCAACCGTTCGATTTAAAGCTGTTGATCGGTCAGTACTCACTACTCTAATGGGTCC
CTGGTTTGCGGGGCCCCACCAAACGTAGCCTCGGGTGCTGGCCTCGGCTGGCGCGCCGGGCGCAGCAGCTCGCAGAGTCG
CAGTGCTCCGCTGCTCCCAATTAAAATATACTCCCAAAGAATTCGGCAGCGCCGAGTGAAGAATTAAAAAACAGGGAAAAC
CCGAGAAAATCGAGTGTACTGATTGCTGAAGCCAAAAGGCGGGATCTTTTTAGCCGTGAAAGCAGCTTTCTTGCGACCATAG
TTGTGAATTCCATTCCGGTACCGGCCGGAATGGCCGGAATGCCTCGTACCGGCCTCTAAACCGGAATAGAAGAGCTCCAATC
CCGTACCGGGTCAAATACCGGTCAATACCGGTCAACGCCGGTCAATACCGGTCAAATACCGGTCAATACCGGCCAATACCGG
CCAATACCGGTCAAATACCGGTCAATACCGGCCGTGGCTGCATGTATTATATTTTTCCATGTATGAACTTTGGAGTTTAAACTT
TGAAATAAGGTCTTATGAACTTGTTGAATGATATCTTAGTTTGGTAAAAATGTTAATTACGCCATAAGTTAAGTCAAAATATATT
TATATATACATTTATGTATAAGTTTATTCACATATTATTATTATTATTATAAAGTCCGGAATGGTACACCGGTATGGGCCGGTACCG
GAATGTTCCATTCCAATAGCCAAAACGAAATGGGCTCCGGAATGGAATTTACAAGTTAGCTTGCAACTCCCATGGCCCTATAG
AGAGAGAGAGGAGAGCGAGCGAGCGGGAGAGATTGTGCAGTGCTGTGGCCCTGTGAGAGAGAGGACCTGAGGAAGAGG
GAGGAGGCTTGGTGGCGGAGCAA
>SsLRR-RLK59
TGTGAAATATTTTCAAGAATTTTGCCTGAGAATTGACAATAGCATTACTGAATCTGTACATATGCATTACTGAACCTGTATGTA
CATATGCATCACTGAAAATTGACAGTAATAGTTTCACTGTCTACCTGTTGACCTGTATGTATGATAGGGAGGCTAATATCCATG
TATGTCAAGCAGCAACCATTTGAGTTGATAACTAGGAGAAGCAAGTATGACAATATCATTACTGAACCTGTGAACACCATTGC
TGACAATATCATTACTGAACCTGAGAACACTATTGCTGACAATATCATTACTGAACCTGAGAACACCATTGCTTGACCATAGA
ATTTCTGAGAACAACAATTTATGACAATATCATCATTGAAACTGAGAATAGCAGGACACTAAGAAAATGATTTCAGCTTGTTC
AAGTTTACAAGCATTACAGGCATCTAGAAAATAGATCATTACAGGCCATCTAGTTCCCAGGTCCTCACATCCACTCAAACTCC
ACATCCAGAAACAATACAAAAGAAGCCTAAGGCCCTACATCTTAGTCCATAGTTCACAGGCAATGTACATTCAGTTCACATTT
CGACGCCATCACAAGCATCCGCAAAATAGGCCATCACAGGCATCGCTCACAATTCACTGCCATCACAGGCATCTCTCACCAT
CCAGTTGACTGCCATTATATTACAAAAGAAAGGTCACACAAAATCTTGAAATGCACCATCAGGTTCACCATCCTCTCCAACC
ATCATCAGCTGTGCAATCTGTCCTCTTCCTGTACATGCAATTTTTTCCAGTAAATAACAGTAAATAATTGACTTAAGCATAATT
GAACTAAAATGCATATAACAGTAAATAATTCAAAGGAAAGGTAACCTTTTCTTTGTTGTAGCAGTTTCACCATCCTCTCCAAC
CATCTCAAGCTTGCATGTCTTTTGAAAATGTACAGTAGCGGTTATAGCAGCACTATTGGGTTGATTAGACATGCTAACAACTG
GCTCTAACTGAACATCTGCATCAGTATGAGGAACCACACAGAGAGGCTCCAGTGCATCAAACTCAGGTTCCACACAACCTG
TATGTAAGATTTTCTACCAGACAAGTCTTTGGAAGCGAAACCTTCAACTTTAACCTCTAGCATAAACATTCCATCCCTACCGT
GCAGTAAATCGAACTCCAAATCAAAGTGTATACATGCACAATACATCGAAATTTCCAAGCAAATCAACACTAATCCCCAACT
GTGATGACCACACAGAGAAAAATTAGGAGGAAAAGGGTACCTGCTAGCGCCGTCCTCCATTGCTGCCGCACCAGTGCGACG
AGGAGGCACGAACAAAGCCGATGTCACCATCAAGGCGCCTCCATCACCTCCAGTAGACGCGCACCAGTGGGTAGGGTCTCC
AATCGCCGTTGCCAGCCTCCAAACGCCACTGCCCGACTCCAATCGCTGCACCCTCACCTGCTAGGGTTCGTCTAGGGAGAA
AGGGGAAGAACATGGGGAGGGAGAGAGAATAAACGGGGCAGGCCAGCTTCACTTACCTTATCTGATTGTAACAAGGGGCA
ATATGGGCCAAGAAACAAATACAGGACCTGCAAGTGGGCCCAGCGACGTAGCATACGTAGAACAGGGCAAGAGTGAGTGA
GGAGCAGAATAGTAATGGCAATTTTCCAATAACGTGAATTATAATGGCGTTTTTCAAAAGTTGAAAAATTATAATGGCGAGAA
TCAAAAAATCCCATTAAAAATATACTCCCAAAGAATTCGGCAGCGCCGAGTGAAGAATTAAAAAACAGGGAAAACCCGAGA
AAATCGAGTGTACTGATTACTGAAGCCAAAAGGCGGGATCTTTTTAGCCGTGAAAGCAGCTTTCTTGCAACTCCCATGGCCC
TATAAGGCAGAGAGAGAGAGAGAGAGCAAGCGGGAGAGATTGTGCTGTGCTGTGGCCCTGTGAGAGAGAGGACCTGAGG
AAGAGGGAGGAGGCTTGGCGGGGGAGCAA
>SsLRR-RLK12
ACATCAATTTTTAACCGCTTGCATGGAGTATTAAATGTAGGTAAAAAAAATAACTAATTACACAGTTTAGTTGGAAATCACGA
GATGAATCTTTTGAGCCTAGTTGGTCCACGATTGGACAATATTTGTCAAATAAGACGAAAGTGGTACTATTCATCAGGTTGCA
AAAATTTGCAATCTGAACGAGGCCCTAGTAGAAGCTAATCGATTCCAACCGTTCGATTTAAAGCTGTTGATCGGTCAGTACTC
TAATGGGTCCCTGGTTTGCGGGCCCCCCACCAAACGTAGCCTCGGGTGCCGGCCTCGGCTGGCGCGCCGGGCGCAGCAGCT
CGCAGAGTCGCAGTGCTCTGCTGCTCCCAATTAAAATATACTCCCAAAGAATTCGGCGGTGCCGAGTGAAGAATTAAAAAAC
AGGGAAAACCCGAGAAAATCGAGTGTACTGATTACTGAAGCCAAAAGGCGGGATCTTTTTAGCCGTGAAAGCAGCTTTCTT
GCAACTCCCATGGCCCTATAAGGCAGAGAGAGAGAGAGCGAGCGAGCGGGAGAGATTGTGCTGTGCTGTGGCCCTGTGAG
AGAGAGGACCTGAGGAAGAGGGAGGAGGCTTGGTGGGGGAGCAATGGCGACGGCGGCCTGGGCCGCTTGGCTGCTCGTC
CTCATGTGCTTCGTCGCTTGGACCCCGCGGCAAATCTTGGTCGCCGCGGCCACCGACGCGAATGATGGTGGGTGATACACTG
AATCTTGCCTTGTTTCTGATAGCATTTCTGCCATCCCTTATGTCCTTCTCATGTTTCTTGTTTGCTGCGCGGCTTCCTTCTATGG
CCGCCCTGGATTCGATTGGGCCAGGGGCTCGGGATTTTGCTTTGCCGCGGCATTGGGTGTATGGGTTCAGTAGGTTTGGTTG
CTGCTCTGGTTGTCCCTTCTTTGAATGCTTGTGTCGTGCCACCATCTGCATCGCCTCTGCTTGGTTTGGGATTATGTGGAGACT
CGTTCTGTTCTGCAGGTTCTGAATCTCGTGTTGTTGGGTGGGGTGGTCAAAATCAAAACTAGTTACTTCCTTGTAATGGCGTC
TTCGACAGATTGGGGACCTGGGAGGCATGAGGGGAACGCCGAGCTGTTTCCCCAACTCTTCAGCTGATGACTACCAACTCC
AGTCACTTCTGTTGATCTATTTCATTCTTTGGCTGCTAATTTTCGAGGAGCAAGGACCTGCATGCTCTGTGGGAAATATTGCAT
CTCTTGGAGCATCTCCAGTAATACTACATAAACTATTCTATTCAAATTCGTTTAAGCAGTCAATGACGCGTGGGGTTCTTTTGG
CATCTCTTTCATCTAGATTTTGCACGGTGGAGAGTAGGGGCCAGCAGAAGTAGAGGATGGAGGGGAAAAAATAGGAGCGAA
CAAATGCGGGTCTAGTATTTACCAACTCCTATTGGAGTTGGGTTTTTTTTTTTTTTTTTTTTTTTTTTTTGCCTCCGCTACTCAAA
TCTGAAGCACGAGGTCCTGTTGCAGATGCTCTTACCTTCTCTGTAGTCGCTGAGCCAAATCTTGTAGTTTGCTTGTTGCTTAC
TTCTGCTGTTCTAAATACAGATTAGGTGGAGAAAATGAGAGGATTATGGCATATGAACATGTGTTCTGGATTACTACTGACTG
GATTGGAAATTGTAGTTTAAGGAATGAAATGTCAGCTCTGTTAGCATGGACCCCTTACCCAGCTTCTGAGCTTATTTCTGGCC
TCAAAGTGTTGATCCAAATTTCTCATTGTCACCATTACAATTAAATTTTCACAACATGGATCTGTGATATCACTGCACAAGCAA
ATTAGATTGGTTTTACTACGTGTTTTCGTGATTCATGTCGTTTGTCCACATTATACCATGGTCCAACTTCCCATTCCATGTAATC
ATAGTAACTGGACTTTATTCAGTGGTCCGCCAAACTCAATGTAACGATTCTTCTCTTTCTGACATGCAGTTACTGCCCTCAAC
ACGCTGTTTACCAGTA

>SsLRR-RLK36
TTATATAATAAAATAATAACATTTATGATACCAACTAATCAGATTCTTCATTAATTATTTTTTAGTATATCTATTTGATGTCATAAAT
CTTTGTAATTTTCTCTGTAATTTTAGTCAAACTCAAGATTCTCAGGAATGATTTATAATTTGGAATAGAGGGAGTAATAGACAC
CGTTATTTGATCTAGCTGTAAATTTTCTAGAGCCGCTAACTCTGATGTTAGAACATGTTTATTCTTTTACTCTATATATAGATGGT
GCAGTCTACACTCATCGTTGTTTATTGTCTACTACTTGTTATTGATTTTTTTTACAAAATTAAATTCATGGCATGCATACAATTCT
CTACATAGCCGATATTATTCAACTCCTTAAACCCATATCCAATCCGATTCCGCTCCCAGTACCAGTTCTAACGTCCGAATAAAA
TCTATATTCACACCTGCACTCGTGTATTACCCACTCCGACCGAATTCGATAAAAGAAAAGTGGATTAGAATACGAGAGATGCA
TTATCCGTTCTAATCCGATTCGTTTTCATCCATATCTAACAGTGTAAACTAGCTTTTGTTGGAATTTGTGGTAATCAAATTCAGG
TGGGGATAGTTCGTCCACCATTGATCTCCTAAACAAAAGTTGCATAACCGATCACATTTTTTTTATAAGAGATCATTGTTACAT
AGCTTAGCCCATTATTTAAAACTTTGAGATACCCGTGCGGTAAACAAAGCCACGTGTTCACTTTTCCGAGTCCCAGCAGTAG
CAGCCCGTGTTATAACCGGGTCACTCACCCATATTTTTAACTATCCCCACCTGAATTTGATTAGGGCTTCTTCAACGCAGACC
AACAATCTTGTTGGCTTCGACCATAAAGATCAGTCCACGGGGGCACAAATTTCTAGAGCTACTAAAATCCACAACGCAAAGT
TATACTCTCCGCTCTCTCTGTTGCTTTATTCCACGGGCAAAAACAGACATCTTTTTGAAAAAGCTGCAGTCCTTCAGTACCTA
GCATGCGCCTCAGCTTGCTACAACGCGGTCTCTCGTTGATGCCTTGCTCTTTTGTCAGGTGAAGACATGACTGCTTCTGCAA
GCGTCACTGACCGACTGCGAGCTATCAGTTGGTGTGATATGGAACATCACTGCCGAGTTGGACAATGGGGCAGCACGCTATG
GTGTAGCGTTGGGGAAGCCCTTAGGTATCCTGGAGAGCTGTACTACACAACAAAGAACAGAACAAAGCACCAGCCATCGTG
CTGACAGTATCAGAATAATCTGCGGCGGTTCGTGGACAGTATCACTAATTCTTTTTGCCTGTCACAGCTGGCTAACACAACAC
AGGGGCCAAAATTCCTCTGCCACTGACCACAGCTCAGGCTGCCAACTGCCAAGGCATACTTGCCACGTTATTCCCTCGCAAG
GACTCCAAAATAGCCAGGGACATAATCCCATGTTCAGTGGTTAATGAACGACCCCTGTCCTGTCCATCCCCAATAATTGCCTG
TTGATTGTCCTTGATATGACCTACAGCACGTAGGAGTACTAGCATAGCATTCTCAATGATAAAGGAAGAGATTGATTGGGATT
TTTTTTCCAAACAAAAAATTCCGGAATTCTCATAAAATGAATTGATTGGGATTTTTTCCCGGAATTTTGAACGTTACTCCTGCC
TCCCGGGCGAAGCGAATCACGAGGATGCCCATGCCCTCACACTGACAGGGATGCCTAGCAAAGCAATTGACCTCACTCTCA
CCCGGGTTCTTGTCCCGGCGCTCTGCTGCCAGCTGCTGCCGTGCTAGCTTCCAATGCGCGGACACGCGATTCATTCATCATCC
GTCTTGAAAAGCGGCAGCGTGATCTGTAAAAAACAGCGGTAAACCAGTCGGATTTCGAGGAGCTGAATAAACAAGAGAGG
AACTCCTCCCGGTCCCGGCCCCGGCCCAAAAAGCACTTTCCTCCTCGCGTCCCGTCAGGGAGGGAGGGGGAGCATTGCTGT
GCGGG
>SsLRR-RLK25
ATACGCCTTGTTTAGTTTGCCTCAAAGTTCCAAGTTTTTTCACTCTCTCTCCATCACATCAATTTTTAACCGCTTGTATGGAGT
ATTAAATGTAGGTACAAAAAATAACTAATTATACAGTTTAGTTGGAAATCACGAGATGAATCTTTTGAGCCTAGTTGGTCCAT
GATTGGACAATATTTGCCAAATAAGACGAAAGTGGTACTATTTATCGGGTTGAAATTTTCTTCAATCTAAACAAGGGTTAAAA
ACGTCCGTGAAACAAGTCCTTTCCAAGGGCACTTCTGTCGTCCTGATTACCTCGCTGACGCTAACAAGATTGTGGACGTACT
ATTATATATAAACGCGGGAGCGCGCAGAGGGAACGCGAGGAGAAAACAAAACACACACCGTCACACTGACAAACAAACAG
GGGAGAGCGGACAGAGGAAGGGAGAAAAGGGCAGTGAACAGAGGCAGATAACTTTTATTTCCTTTTCCCCCATTCACCTTC
TTCCTTTCCTTGGATACTCATACTCCAGGGGAGAATGTCTCCATGCTTAAAAGCGAGAGCCAGTGAGTGCGGAGCAGCTCCA
GGCATGGCGTTGGGCGTGGCCGGAGTGGCGGCGCTACTCTTGCTCTCCGTCTTCACCGGGGCCACCGCCGACACCAACTCC
GACGACGGTTAGCGATTCATGTTCCCCTAGTTCTCCCATCTCTCCTCAACTGAAGCGTATGTTCAGTTTAGCATGCCAACAAC
CTTTGCCCCCTTGTTCGTGATGCTCCTTCCTCTTCAGTCTGCCGTCAGACTGCTAGAGTACCAATGGAAAAAAATCTGCTTCT
GACCATTTCGTCTCAGGCCTTGTTCAGTTCCCCAATTCTCTCCCTAAATTTCACTATGCAAAAAGAAGATTCCCTGTCACATC
AAAATGTCGGCAAATATATAGAATACTAAATGTTGATGAAATCAAAAACTAATTGTACAGTTTTGTTGTACTTTGCGAGACGA
ACATTTTAAGCCTAGTTAGTCAAAATTTGGATAATTTTTACCAAATACAAACGAAATGCTACAGGAATTCTGATACAGTGTTTT
TCTCCCCAATTCTCTCATCTAAACAAGGCCTCATTACAAGTGGTTTGGAGGCAGTAGCAGTATTGTGCACAGACATCATTACT
CGTTCTATCTCATATGCGAGTATGCCACTGACGTTTCTGCTACTGCTTGTTCTGATAACCTCCCCCTTCCTTTGCTACGGACTG
GGGAAATCACGGTCATTTCTAGTTTTTACTATAGTTTCTGAAATCACTGTGACGACTTCTTTATCCAGCCTTGGGCCCAGGGG
AATGCCAAATTGCTGACCTGTTCAGTTCTTGTCTTCCTGATGTTCTTTCCGATTTATTTTCTTTGACTTTTCCGCTTCAGAAGG
CCGAAATTCTATAGGCTTAAGTTTCATTAAGCTTCGGATGATTGGGAATTTGGTCTTGGACTCATACATACAGTGTCACAGGTT
TGGCCGAGTGATTCTCCATTTTGTCCTCGGCGGTTTAGTAGCTTTGTTTGTCCTATCCGGCTAATCCGGAAGAAAAGAAACAT
GCCCAATTGTTTAATCTGTTCTATGGTTCTTCCTTTTGTCATACCTAATAATAATAAAATTTATGCACACCGGGATGCGAATGTT
GTGGTTATTCAATTCAAGGCACCTTTTTTTATTGGATTGCTTAAAAAGTTAGGTTCATGGTACTGCAAGTAGGACTGCTACCTT
TGACTTCAGAGTAGTATATGCTTGGAGATTTTGGAGTCTCAGATGATGGGAAAATACAGCTAGGATAATACTAGTATTTGTTCC
CTTTACTTTTTTTTTGAAAGGAAAACCTAATATTAATTTTCATATTCATTGTCAAGAGGCTGTACTGTTGATTGCTGAAAGCTG
ACAACTTGTTGACGAATAAACACTCATCATGGTATTTCCACCCTAATTATTGTTGTCACGCTATCTGAGTTGATGTTTCTGCCT
ACTCAG
>SsLRR-RLK48
GTCACGCACAACACAAGCAACACAAACGCAAATCTGACTGATGCATCTGCTCTTGTTTTTTCGTCATCACAAACATCGTGTC
CGCCTCAGGTCGCCGAAAGGCATTCGTTCCTACAGGCTACATGTAGTAGTAGTACACTCCATTGGCTATACCAATCTTGCTATT
GGGCAGCTACCGGGTCCGTGTCCGTCCGGACATGATCCAGCCGACGGCAGAGAATCATCCATCTCCACTCCACCACCAGCC
GGCCTCCATCTCCATGTGTAGCAGTGACAATGCGCGCACAGTGTGACAGCATAGGGCATAGGGGAAAGATGTGCGAGCCGC
ATTCCAAAATTACTACGCGCTCCCCTGTTTTCGTTTTAGAGCTTGTTTAGTTGCCTGTTTATGTATGGATTATAGAAGCTTTTCT
GTCCTCTTCACCTTAAATGAGCGAGCTCGATGGCGATGGAAAAAAAAATTTCCAAATATATTTCTAAATTGAACCAGACTGTA
CTTTTGGAAAGCAGTAAATACATACTAGTTAAAAACGTCCGTGAAACAAGTCCTTTCCAAGGGCACTTCTGTCGTCCTGATT
ACCTCGCTGACGCTGACAAGATTGTGGACGTATATATAAACGCGGGAGCGCGCAGAGGGAAAGCGAGGAGAAAACAAAAC
ACACACTGTCACACTGACAAACAAACAGGGGAGAGCGGACAGAGGAAGGGAGCAAAGGGCAGTGAACAGAGGCAGATA
ACTTTTATTTCCTTTTCCCCCCTTCACCTTCTTCCTTTCCTTGGATACTCATACTCCAGGGGAGAATGTCTCCATGCTTAAAAG
CGAGAGCCAGTGAGTGCGGAGCAGCTCCAGGCATGGCGTTGGGCGTGGCCGGAGTGGCGGCGCTACTCTTGCTCTCCGTCT
TCACCGGGGCCACCGCCGACACCAACTCCGACGACGGTTAGCGATTCATGTTCCCCTAGTTCTCCCATCTCTCCTTAACTGA
AGCGTATGTTCGGTTTAGCATGCCAAGAACACTACCTTTGCCCCCTTGTTCGTGATGCTCCTTCCTCTTCAGTCTGCCGTCAG
ACTGCTAGAGTACCAATGGAAAAAATCTGCTTCTGACCATTTCGTCTCATTACAAGTGGTTTGGAGGCAGTAGCAGTACTGT
GCACAGACATCATTACTCGTTCTATCTCATATGCGAGTATGCCACAAACGTTTCTGCTACTGCTTGTTCTGATAACCTCCCCCT
TCCTTTGCTACGGACTGGGGAAATCACGGTCATTTCTAGTTTTTACTATAGTTTCTGAAATCACTGTGACGACTTCTTTATCCA
GCCTTGGGCCCAGGGGAATGCCAAATTGCTGACCTGTTCAGTTCTTGTCTTCCTGATGTTCTTTCCGATTTATTTTCTTTGACT
TTTCCGCTTCAGAAGGCCGAAATTCTATAGGCTTAAGTTTCATTAAGCTTCGGATGATTGAGAATTTGGTCTTGGACTGATAC
ATACAGTGTCACAGGTTTGGCCGAGTGATTCTCCATTTTGTCCTCGGCGGTTTAGTAGCTTTGTTTGTCCTATCCGGCTAATCC
GGAAGAAAAGAAACATGCCCAATTGTTTAATCTGTTCTATGGTTCTTCCTTTTGTCATGCCTAATAAAAAAAAAATTTATGCAC
ACCGGGATGCGAATGTTGTGGTTATTCAATTCAAGGCACCTTTTTTTATTGGATTGCTTAAAAAGTTAGGTTCATGGTACTGCA
AGTAGGACTGCTACCTTTGACTTCAGAGTAGTATATGCTTGGAGATTTTGGAGTCTCAGATGATGGAAAAAATACAGCTAGGAT
AATACTAGTATTAGTTCCCTTTACTTTTTTTTGAAAGGAAAACCTAATATTAATTTTCATATTCATTGTCAAGAGGCTGTACTGT
TGATTGCTGAAAGCTGACAACTTGTTGACGAATAAACACTCATCATGGTATTTCCACCCTAATTATTGTTGTCACGCTGTCTG
AGTTGATGTTTCTGC
>SsLRR-RLK422
TTGTCCAGCTTTCATATGTTGACAGGTTATATGTCGTTGGCATTCCTTCTTGGTGCTGCATAATGGGTCTGGTCGATCGCTAGT

CTTTTCCAACGTTAAATAACTGCACATTCATGTGCTGCATTTGGTTGACCATAATTTGCGCTCCCATTCCAGTTGCGCGTTGCT
ATCTGGTGAGCTGAACGGTGAAAACCCCAGGCGCCCATTCCGGTGATTCCGTCCCGTCGATCCTTCCGTTTTCGTGCATTTTC
TTTCCTTTCGTTGTCCAGTAGTTGCGACGTGGCTTTTCAAGCAGCAACGGTAGTGTGCGTCTCAGCGTGATCATCATCGCCCG
TCAAGTAGGTGAGCGGTGAGCCTCGTCGGCTCGTCACTCCGCTGCCCCAGTCGTCCAGCCCCTCAGGCCCTACCGCAAAGA
ACCCAATGATTCGCGCGTCGTCGTCCCACATCACTGTAGAGTGTGTTTCTCAAAAAAAAAAAAAAAAAAAAAAAAACAGGAG
CGTGGAGTGTGGACTACTGGACTGTGTGGTCACTGGTCAGGCAGGCATCATGGTCATGGGCATGGCGTCCCATCCGGAATCC
TGGATCATGGCTTGAGCCTTGTCGCTTGATGATGCGTTGGCCGCCGAGCGATTCCTCCCCTTTCCGTACGTGCCCAAGGCCGC
GCGACACCCGGCCGGCCATGATGTGAGACTTTGGAGACCGCCGAGCGCCGACAAAACAGACGCGCGGACGGAATGAATGA
GTTCAGCTGTAAAACTGGCTGCTAGCGCACCCAATCGAGAGTAAACGACGCCGGAACGCGAGGGAACGCCACATGCCGTG
GCTGGCCGCGCACCCGTCTGGATCAGCGTGGCCGATCGCGGGGGCGAACTGGGGAGTGGCCGAATGGCCGCGGGCAGCTG
GCTCATCCGGGCGGCCGCAACGGTAATAGCCAGTACTATCTACATAGTTAGCTAACGTGGAGGTGAAAGAAAAGAAATATAA
TTATTTAAAATTATTGGACCCATATGCTCCTCTTAAATGATCTTGGAGTACGTGAAATAGATTACTCTTTTTTCTACTTTTTGCTT
ACCATATCATATCTAGCTAACCTATAGTTAGTTGTTGGAGACGCTCTTACACGGGCACAGTGACAGTGCAGGACAAACCGGA
AGCTCGCTTTGTTTGGCGCTGCCTTCTTTCTCGAGTGTCTTTGGCAGTGGCTCCTTGTCCTTGGGTAGCTCGTCCCTGGGAGG
TGGGCCCAATCTGTCACGGGCAGCGGGGCCACCGCGCAGGGGAGGTGGCCACCCAAGTCACGGGCGCTCGATTACCCTGC
TTACGCTTATCATTAAGCGGCGAGATGATTGGTGCACCGGGCTTAAGAGTCGGCGTCAGATTTCCCTCGGCACATGCCATGTC
TAGCAGGACGTTCACGAGAGTTGCCATCACTTGCTGCTATGATGCCAACTTTCGACGCGCTAGTATTGTAGCAGAGCCTGGT
ATAAAAGCAGACTGATCGAATCGAGTTTGTATTATGCAGGAGTACTCCGTAGTAGGGAGGTGCTCAAGTGCAGTACCAACTA
CCAACATGTTCCAGACAGAAGGTGAGGTGAAGTCAAACTCATCTGGATCTTTTAGACAGCAACTAGACGTTTGAACGTACA
CTATCTGTTACAGTACTGCTAGCTTGTATGTATTAATTGCGTTCGCCTGTTCGTTCGATCATATCAGCTATCCACTACGACTACT
TTTTTAACCAACGAGAGTGTTTTTTTACTCACAGCAAATCAGCAAACAGTACCTTAAACGGTGACTTTTCGGCCAAACGAAC
TGGGCGCGAAGAAGAGGTGTCGGTTTGCCTTTGCCCCCCATGATGAACAAGAAACCACCTTTTGGGGGTGCTGGAGCCTGG
AGCGGGCCCTGCATGCTTGACTAATGGTTTTTCTGATGTTCCACCCAACGAATACTAATCAATATATCCGGCACCCGTCATTCC
GATCGCCCACTTTTTCGAGTTGTTTAG
>SsLRR-RLK205
TAATTCTCTCTCTCAGCACTAGAACTACTACATTAATGTCTGTGAGAGAGCACGCCTTATATCATGGGATATGAATAAAAAGTG
GTTACGCCTTATATCCTAGGACGGAGTACTATTTACCAAGTTGTAACGGGCAGCTCAGATCAGATACTAGCTGTAGTATTTTAC
CTAGTGGTGTTTAGTCAACGCCCAACTTGCCGTTCGGCTTGACCCATGCATCATATGCCAAACCAAACAGAGAAACAGGTTT
GGAATTCAGAAGGAAGACAAATGTCCTGAGCATGACCGTTAGCCTCACCTCTCAACCTTTTATTTCCTCCTTTCACCAAACT
GTCAACCATTTCAATCTGGTGATTTGGCGGGGGGTAGCACAGCTGCAGGTTTGCAGTACTAGGCCATTATTCCAACTTTCCAA
GGTAGCTGATGTGACATGTAACGAACTGCTATACGGAGTATGCTATAGTACCTGTACCTGGATCTGGATCTTAAATATAAGCTT
ATTAATCAGCATGCATGCCCACATTTCCACAAGACAAAAGGGGACGCGTCCCTGGTTCTAGAATTGTTTCGTGCAAAAGGGA
ACGAACGCGTCTGAACGTAACTTTATCAGATCTACTATGTTCGTGCCCCCTGTTCGGCTGAGCTTATCAGCACGGCTTATCAG
CCGTGAAACAGTGTTTTTCTCTCACAGCAAAACAGTCACAGCCGGCTTATAAATCAAAAAAAAACAGTCTGCCGAACAGGAC
GATGCCTTCATGGCATCCAGCAATTTAAAAATACTTAAGGAATGTAGCACTAGCTAGATTAGAGGCATGAAACTAGTGGAAA
GTTAGAATCTAGTAAAGTGTGTTTCAGGTGAGGAGGAAGATGGAACATGCAATGCAATGGTGAAAACTTGTTGTCTTGCTCA
GTCAAACAACAGCTCATGGTTGGTTGACTGTACTGGTACAAACCTTGGTTCACAGTCCACCATTAGCAAAAGCATCAATCAG
CATACTCCCTTCCCATTGCTGGTGGCCTCTGCAGGCTCTGCTCGGACTTCATCACCTTTCACCTACTCCCATTGCTCCCCCATT
AATTTACCGGATCAAAAGCACCTTCGTTCCTGTCCATCGAGGAGAGACAAAGTGTGTGTGTGACAGTGGCTGTGTTTAGGTA
GCCTTTAGTTGCCAAAAATTTGGCAAAATGGCACTGTAGTTACTTTCGTTGTTATTTAGCAATTAGTGTCCAATTATGATCTAA
TTAGGCTTAAAAGATTCCTCTCGTCCATTTCGTCTAAACTGTGTAATTAGTTTTATTTTTTATCTATATTTGATGCTTTATACATG
TGTCTAAAGATTCGATGTGACGAGGAATCTGAAAAATTTTGTAAAAATTCTTGAAAACTAAACAGCCCCTTACTTCCTTAAA
AAAATACTGTGCAGAAAAAAGATTCCCCGTCACATTAAACTTGTGATACATGCATGGAGTACTAAATGTAAATGAAATCAAA
AACTAATTGTACAGCTTGGTTGTATTTTGCGAGACGAACGTTTTGAACCTAATTAGTCAACAATTAGACACTATTTACCAAAT
AAAACCAAAACGTGCTACAGTATTTTTTTTACACCTTTTCGCGTAACTAAACACAGCCAGCGAAGAGAGCGAGTCAAGAAC
AGCAAGCAGCCCAGAACCCAACAGACATTTAAGAGGGGGAGCTAAAAAAAATTGCAGTCTTTACCGGCTCCATCCCTACCC
CGCCGCATCCAAGCAGCCGTGGCATCCGCCGTTTCTTGCGTGCTTCCTTTCCTTGGTTCTTACTAGCTGTCACCCTTGGTAGT
GAACCAGCGGAGAAGAGAAAGAGAGGCGAACCGCCGTGCGGCGGTGCGGCCCATGGTGATCCCCGTGCTGGACACCATGG
CCGTGTCCTCCTGCTAGGGGATATAAGCAACCGAGGAAAGCAGCAATCTTTCGCTGCCGCGGAGAGAAGGGCCAAGAGGG
AGTGTGTGTGGGGACCAGGCC
>SsLRR-RLK229
CATATTTCATCATCAGCCCAGCCAGCACAGCGTCCGATCCTACCTTCAATGTAACAAATCCCAACCACAAGTTGTCGATGAAA
TCAATCACTCACCACATGCATACCTGCAGCCAGCAGGCAAGACCTGACTAGTTTAGGGCTAGCTCCTATGCCAGTACACATG
TTCGTTAAGTGCCACGGGACACGCCTGATCATGGATTAGGAACATAATAATAATATTATAAAATATTGCTCGGTTCTAGTACTAT
TATTTACCAAGTTGTAACGGGCAGCTCAGATCAGATAGTAGCTGTAGTATTTACCTAGTGGTGTTTAGTCAACGCCCAACTTG
CCGCTCGGCGTGACCCATGCATTATCTGCCAAACCAAACAGAAACAGGTTTGGAATTCAGAAGGAAGACAATTGTCCTGAG
CATGACCGTTAGCCTCACCTCTCAACCTTTTCCCCCTGTTCGGCTGAGCTTATCAGCCGTGGAACAGTGTTTTTCTCTCACAG
CAAATCAGCCACAGCCGGTTTATAAGCCAAAAAACAGCCTGCCCGAACAGGGCCTTTATTTTCCTCCTTTCACCAAACTGTC
AACCATTTCAATCTGGTGATTTGCCGGGGTAGCACAGGTGCAGGTTTGTAGTACTAGGCCCTTGTTTAGATTGCAAAATTTTT
CAACTCTAGGTACTGTAGCACTTTTCGTTTGTATTTGGTAATTATTGTCCAATTGTGGACTAACTAGGTTTAAAAGATTCGTCT
CGTGATTTACTACTAAACTGTACAATTAATTATTTTTTTACCAATATTTAATGTTCCATACATACGTTCAAAAATTGATGTGACG
GAGAAAGAGTGAAAAAATTTGAAATTTGGAGACAATCTAAACATGGCCTAGGCCATTATTCCAACTTTCCAAGGTAGCTGAT
GTGACATGTAACGAACTGCTATATGCTATAGTACCTGGATCTGGATCTTAAATATAAGCTTATTAATTAATCAGCATGCATGCCC
ACATTTCCACGAGACAAAAGGGGACGCGTCCCTGGTTCTAGAATTGTTTCGTGCAAAAGGGAACGAACGCGTCTGAACGTA
ACTTTATCAGATCTACTATGTTCATGCCTTCATGGCATCCAGCAATTTAAAAATACTTAAGGAATGTAGCACTAGCTAGATTAG
AGGCATGAAACTAGTGGAAAGTTAGAATAAAGTAAAGTGTGTTTCAGGTGAGGAAGAAGATGGAACATGCAATGCAATGGT
GAGAACTTGTTGTCTTGCTCAGTCAAACAACAGCTCATGGTTGGTTGACTGTACTGGTACAAACCTTGGTTCACAGTCCACC
ATTAGCAAAAGCATCAATCAGGGTGTGTTCACTTCCCGTGCAAACCCAAAATATTTTTTCTTTCACCCATCACATCAAATCTT
TGAACGCATGCATGGAGCATTAAATGTAGGTAAAAAAGATAACTAATTGCATAGTTTATCTGTAAATGTCGAGACAAATTTTT
TGAGCCTAGTTAGTTCATGATCAAACAAATTTTGCCAAATAGAAGTAACAACAAAAAAATGCTACAGTAACTTTTCACTCCTTT
TCACCGACTGAACACACCCTCGGCATATCTTTCTTGCTGGTGGCCTCTGCAGGCTCCGCTCGTACTTCCGCCTCGTTTAGTTC
CCCGAATCGGGGGTGGCAAACTTTACGCAGTGCCACCCCTGAACACTGTAGCACTTTTGTTTTTTTAGTAATAATTGTCCAAA
TTTTGATTAATTAGGCTCAAAATGTTCGTCTCGCAGAGTATAACAAAACTGTGCAATTAGTTTTTGATTTCATCTACATTTAGT
ACTCCATGCATATGCCGGCATTTTGATGTGATGGAGAATCTTCTTTTTGCATAGTAGAATTTGGGAGTGAAATCTGGAATCTAA
ACAAGGCCTTCATCACCTTTCACCTACTCCCATTGCTCCCCCATTAATTTACCGGATCAAAAGCACCTTCCTTCCTGTCCATCG
AGGAGAG
>SsLRR-RLK70
ATATGGTTTGGATTGTTGACCAGAAGTTGCTGAGGCTGCATGCTCTGAATCTCTGCCTACAGAAGGGCGAGCTGCTGCCTGA
TCTCCGCCGCCTGGGCGGATGTTTGCATTGCTTGCTGACTTGCAGCGTAGGCGGCAGCAACTTTGTCTCTCTCTTGCTGCATA
GCTTGTAGTTGGGCTTGGAGCTACTGTAGCTCTTGCTCAGGGGTAAGGCTAGGCGCTTGCTGAACCTTCGACGTTGGCTGCT

GGTCCTGAGTCTCGGTGTCACCATCATCTTGTATTGGTGCGGCGTAAATGCTGTCGACATTGCGTCGGGGCCTTATGTTTTCG
ACGGAGGCTGCTGGCTGCCTAGGTGGTGCCTTCCTTTTTCCTGCCATTGTGGGTTTGTGTCGGCCAAACTACGGTGGGCGCC
AATGTTGGGAAACTGTTCCTAGCAAGGGGGTTAGCGTGGTTGGATGAGACCTCCGCCAAGGTGTGGGTGGAGGTTAAGGAG
GAGAGGTCTTATGGAAAACACAAGTGGGACCCCAGAAAGCTAGGGTTTCATTACTATATTCACCTACCTCTTGGAGCATTTA
CAACCCCCTATTTATAGAGCACCAACTGATACAAATCACAATTACATTCATACCCCGTGACGGTGGCAGTATATTCTTCGTAGA
TACCTTTCCTAGGCTAGTCTCCTAAGGGTAGCCGGTCATTCTCGGATGAAGGCGGCTTCATGGTGGCCGACGCTTGCGGCC
CCTGGCCCCTGGGGCCTTAGTGCTGACGCCGGAGGAGCAAGGTTCGGACCTCCTCCCTTGGATGGCGGAGGTTCCACCTCT
GCCTCAGAATTTGAACACCCACCGCCTGTTGGCCTCGCCCGACACATTCCCGTGAGCGGCTTTCCAGGCCACCGAGCAGCT
CCCGGACCTTCGCCCGCACGCACAGGTCTAACCTCTGCCCGCCAGAGGCGGAGGTAAGGTATCTGCTTGGGCTCCGCTCCC
ACGCATCCTGCACAGCAGCACAAACAAACAAAAATATCCAAGTCGGGATTAGGGCGGAACTCCTGTATCTTCGCCTGAAAC
CTCCGCCCGGGAGTTGTCGAAGGCTCCATCGCGTGGCGCCATAGCTCGACCGCGTCCTGAGCGCGCACCCTGCTGTCGCGT
TACCTCCGTCGCCAGGGGGAGATGATCCCCCAACAGTTATTATTGTTTGACCTTAATCAAGTGGTTGCTATAGAACAGGTGCT
AATCTAGATGACAGGTTAATGATGATTAGCTTGATGCTAAGCATAAAATGAGTACTTATGTTACTTGAGCTTTTAATGCAAAAT
GTCATCAAGCAAGCTCCACTTCATAGTAGCCTTGCATACCCTTGGTGTCTTTTATTTATGGTTTTGACAGGTAAGTCTATCTGA
TACCTTCTCGTACTCAGGGTTTATTTCTCATCTGTTGCAGATGACATTTTCTATCCGGTTGCTACAAGTACTGCCTTCTCCCAC
CGGGGATGAAGACTAGACCGTTGGGGCGATCTCTGCTAGAAGTTCTCATCATGTGCTTTTGTGGGACGTGACTATGAACTGG
CAATGTATTTGAACTGCTAAGTTATGTCTTAAAACTATGTTGCTTCCGCTACTCAAACTTAGTTTGTAATATTTTATTTCAACTC
CGATGGATGTAAACTGTAAGTTACTTGTGAACTGTTGTAACAGATGGCATGTTGTGTTGAATCATGTACGATCTTGGCTTGTT
GTAAGTTGTTTGAAATCATTTGTGATTTCACGGACTACCGGGGTTATGGAGNNNNNNNNNNNNNNNNNNNNNNNNNNNNNN
NNNNNNNNNNNNNNNNNNNNNNNNNNNNNNNNNNNNNNNNNNNNNNNNNNNNNNNNNNNNNNNNNNNNNNNNNNNNTTTTA
CCTATATTTAATACTCCATACAAACGGCTAAAAATTGATGTGATAGAGAGAGTGAAAAAACTTGGAATTTAGATGACATCTAA
ACATGGCCAGTAACAGCAGCAGAAGCAGC
>SsLRR-RLK369
AATAGTTGTTTGTTAGGTACTCAACTAACAGTTAGCTAAATCTTTTGATCCAAGGAGATAGTAATTATTAGCAGGATTCAAACA
GGGCCTTAGTATCTTCATGGATGACAGGGCCTTAGTCTTTTTTTTGTCACTTGAGTGTTACCCCTCCATTTCAAATTATAAGAG
CTTTGACGTTTTAAAATACATAACTTTTGCTAGGTATCTACGTGCATAACAAAAGCTATGTATCTAAAAATAGCAAAATAATTT
GGAATGGAGGGAGTACTTGAGTGGTGGAATCAAGTACTTATTCATAATTACGTCTTACTCCCTCTGTCCAGAAATTATAGGCG
TATTAGGCTAGAGGAAAAGTCAAATTTTACAAAGTTTGACCAACACTTAGTCAAATTATGTGTAAGTTTAGAGCATAAAACTT
ACATCAATAGATTTGTGTTCAAAGTGGCTCTAAGATGATATTGATTCTTTAGCAATTGACTACATACTATAAGAGAAATTGTGG
ATCAAAATTAATTTTGCATATTTTTTCCTATGGTCAAATACACCTATCAAGAAATTATTTAGATGAGACGAAGCCATGGCCCATT
TGGCCCTATATATAGAAGGTCTGCCTCTGCAGTTATTACACGTGTTTCCGTTACTAGAACATAAACCTACAACTATGGCCTTGC
TTCGTTTCACGTCAGCGACGTGTTTGAGCGTAGAATGCGGAAAAAACCCGTCGAATGGTTTGCGTTCGACGCGCGTTGGCT
GACCGCGCGCGATGTGAAACACAGTAAAATGAACTGTGCGATGGGAAGTCGCAAACGCAGCGTTAGAGCCACGTTCGCTTT
TCTCGGTTGCGCGATGGGTTGTCGTAGAAACATATAGGCCCTATGTTCATGTACCATAACATATTTTCCTTTTTTGTAAAAGTT
CCAATGACTAGTCTTTTTATAGACGCTTTTGTCATGCCCAAAAACTATCGTATCGGATAGCATACACATACATAATATGATATAT
ATTGTTGTTCGTAAATTTATAAATTTTTTAAAAAAGGAATAATGGTCTTCTGTATATTACCCATCCCCGAGCAATTTCAACCTGC
TCCAAGACTCAATTTTGAAACCGTCTGATGAGTACAGTGATTTGTAATTTAGGAACCTAGATGACATCCCAAACGAGCGAGC
TAAAATATTTCACTTTGTATTGCAATAAAGTAAATAAGGAAGATATAAAGGTGTTAATTTAATATGTAGTAGGTCCTGATATCCC
ATTTGAGCGTTGCACTTCTATATGATTTGCAACTAGAAAAAAAATTGTAGTATCTGCAGATTGATGTTTTGTTTGAAAGCAAA
ATATATTTGGTAAAGGCATGTGCACGTTAGCAACCCAGACAAAAGGATTAGCTGAAACAGAAGTGGGCGGTTGGTGACGAA
AGCGGGGCGTTTTGTGTTGTGTGTCACAAGGAGTGTTGAGTTGGCACCAAAACTCGGTTGGATTTTGATTTGTGTTGCACCGAC
AGACAGGAAATTCGTGACCCCACCCACCCCACCCAAAGTCCCAAAATCCCAAACCAATCTTTTCTTTTGTCCTCCGGTTCCG
GTTTGTACTGCGCAGACAGTCAGTCCCACACTCCCACACATTCTCTCTCTTTAACGACCAATTAATGTCTGCCTGCCTCCTAC
CTCGATTTGTTTAATTATAAATTACTCCTGCTTTGCAATCAGTCACTTTATCACTAGTTAAAGACGAGCGCCGCATGGAGACGG
AGTGGTAGAAAAGATGGATTTCCGTCTCATCTCCTTGTTGTTAAGTTGTTGATTGAGATTAGGGAGTAGAGTAGAGTGAGCA
GGTGGAATTGTCCTCCAACCATATCCACCACACTAATCTGCCTGCTCGTAACAGCTGCAGCAGAAGCAGCGCAGCGGCTTTG
GTTGAAAAGGAGCCAGCTGCTGCCTTGCTCTTGCTCCGTCCCCTCCCTGCGCCTGCTGGTGGCGCTGGCTGCGAGAGAGGA
GGACGA
>SsLRR-RLK89
CCAGCAGGCCGCCGCCGCCGCCGCTACCGCCTCGATCAACGTTCCCTTTTTTTCTGTACGTATCGGTTTTGGAATTTTTTTG
CGAGGCGCCAGTGGCGAGGGATTCGATCCCTAGCCGGCTGCCGCGGAGGGAGTGCGGGGGGAAGGCAGGGGCTTCAACGC
GTTGAAGCCTGGGACCAGGGGGTGAGAAGCCTGGCTTCAAACTTCTTCTCTCAGGCATCAGATCTGATCTAGGTGTTTATTA
GCCCTTGATTTTAATAAAGATGAAATCTTATGTGTATCTCTACTATAATAGACCAATCAAAATTATACTACGTCCCTCCAGAAAA
ACGTCCTCCGCAGCGGAGGACGTTTTTCTGGAGGGACGTAGTATAATTTTGATTGGTCCATTATTGTAGAGATACACATAAGA
TTTCATCGACCAAGCATTGCGGTTTGATTGCACACCCTCCGTAACTCTAACAAGTTGTTCAGTTGTTGTATGTGATCAGTGTC
ATTTTCTCTTTGATATTTAATTGAAGGAAATTTTTGCTGTAGAACATCCATATTCACTGGAATTGGCTGGTTCACACCGCCAAC
GTCGGAATATGCTGAGTACCATTATGAAATTGCGGTTATTTGTCAAGACATTGCATCAATTATTTTATTCATTTCTCAGTTATGC
AGAGAGAGAAGGTTGGAGAAAATATCCTTTTTGCCCTTATCTTCAACCTCTGGCCGATGCCTGTTCTCGTGCTCCCACTTCGC
AGCCGCCGCCGGACCTAGTCATCCCATGCCATTCGCATCCACGTGCCTTTCTGCTGCCGGTGCCTCAATCACCCTCGCGTTCA
CGAGCGCGGGCTGCCCAAGCCACGATGGACTCAGCGTTGGCGGTGGTGATGAAGCCCTCGTCGCTGGACTTGCTGGCGGC
GCTGCTAATGGGCTGGGGCAAGCAGGTCTTCCTTCGTCTCGCTCCCTCCTCAAGCCTTTTTCGGTTCTCCCCCCACCACGTTC
CAATCCCAATCGCGAGCCAGCTAGAGGTGACACGAGGCGAGAAATTCCCCAATCCCCTCGCCACCGCCTCCCGTCCCCATTT
CCTCCAATCCAATCACCACCGAGCACCAAGTCCCACCCACCCATTGGGAGGATCCACCCACCAATTCCTAGTCATAGCTAGC
CATGGAGTGCGAGCCGGAGGAGCTGCAGTCCTAGGCGTCCTGTGCATGTATCGCTTCTCCACGGAGATCCTCTGTGGCCTGC
CCTTCACGTGCATCGTGGGGCCGCCTTCGCCCCCCCTTCCTCTTCCTCCTCCACATCTCCATCTTCCATGCACTCTTCACCCAC
ATCAACTCCCATGACACGATGCTCGACACACCTCCTCCCCGGGCACTCGTAGCGGTTGCCAGAGAGGTGAGAGCACGAGAA
TAGGCATCAGCCAGAGGTTGAAGATAAGGACAAAAAGGACATTTTCACCACCTTCTCTCTCCGCAAAACTAAAAAAATAAA
TAAAATAATCGGTGTGGTGTCTTACGACAAATTAAATAACCACAATTTCATAATGGTACTCATCATATATTTCATCGTTGGTAGT
GTGAACTGGTCCATCGAAGTTGGATGTCCTGCACAAAAATTTGCCTTAATTAAACTACTCCATTCATTTTAAATTATAAGATGT
TTTTGCTTTTTCTACATACGTTGTTCTTATTATGTATCTAGACATAGTATATATTTAAGTGCATAGCAAAAACTACGAATATAGAA
AAGTTAAAATGTCTTATACTCCCTCCATTCCAAATCGTAAGTCGCTCTGGCTTTTAGATACATAACTTTATTTTGCTACGTATT
ATGATATACATTACATCTAGATACATAGTAAAAGTTATGTATCTTAAAAAGTTAGAATTGCTTACAATAATTTAGAACGGAAG
GTGTACGTCATTGGATATGATCATTAGCTAAATCTGGTCAATTTTTAATTAACTCTGAACTACTCTCCTTCCTATTGTTCTTTTAT
AAGG
>SsLRR-RLK79
CGAGCCAGAGCAAGGGTCTCGCCATTTTTGTGTTATGTACTGACCTCCTTGAGTCCTTGGCGTCTGCTCTGTGTGCATGGGG
AAGGACAAGGAGGGTAAGAGTATATATACATGCGTGCAAATGAGTGAAGAATTATTGGATGGGAAATAAAAGAACCAAATG
CCAATATGCCGACATTGAAACCTTTTAATTTGCAAATGGGTGTATTTTGAAATTTGCTTTTTTTTGGTAAAATCCAAAAAAAA
GTTTTGATAGGATAAGCACAAGGTTTTATATACAGATACACACGATTATTCATGAATTAAGTATCTGCTAGCTATGTTATTGACG
AAGTTTATCTATCTTATCACGCGGATTATTTGATCATGCATCTCGTCCAGAATGAATCAAGTTGACTAGACTTGCTTCATCCGT

CTATATGTTTTGCTAAGTTANNNNNNNNNNNNNNNNNNNNNNNNNNNNNNNNNNNNNNNNNNNNNNNNNNNNNNNNNNNNNNNNNNNNN
NNNNNNNNNNNNNNNNNNNNNNNNNNNNNNNNNNNNNNNNNNNNNNATGCTCTTCTCAGGGCGTCCGATGCTTTCGACGCGCCC
TTCCTGGGCTGTGGAGCCAATACGAAGCCTGCTCCTAGCTTCTCCATAAAAAAATAAATGTCGATTCTCAATCCTCCTCTCCC
ATCACAGCCTTCTTTCACCGAGTGCATCCGTCTTTCTGCGGCCGACTCCACGAGCCACCCGCCGGCGCTCACCTTCCTAGCA
AGAGACGCTCGCGGGCAGTGCTGTCGCCTCCACTGCAGGGACTGCACCCGCCCACCCTGTCCCTAGGAGCTGCGCAGCAG
CTCGTCGGTGGCCGCATAGCCCTCGCGGAGGCCGCGCAGCCGCCCGCCACTGGCATCCCTCCCACGGCATCGAGCTCCACA
TCGACGAGCCTCGATTCGTCCAGCAGCAAGTAGATGCTACGCTTAAAGCACATGTTGCAAGAGTATGTTTCAAGTATTTCAG
ATGTTTCAAAGGTATGTTGCAAGTGATGTATATCAATGTTGCAAAAATAGATCGGGATGTTGCACATGTTGCAATGGCTATACA
CGTATGTTTCAAGTGTATGTTCGAAATGTTTCATTTGTTCCAGACGTATGTTACAAGTGTTTTATCTGGATGTTGCAGAAGTAG
ATCTGGATGTTGCATATGCATGCATGTTGAAAGCATATGTTTCAAATGTTTCAGGTATTTCATACGTATGTTGCAAATTCGTCTA
GATGTTGTATATGTTTACAATGGTTTTCAGATGTTTTTGCAAGTGTTTTTTTTAACAAGCGCCACCTTGGGCGTGGCCTTCCTG
GAGGCCAACGTAGGGAGCCGGGGTTTGAACCCCGGACTAGCAGCTCCACCCTCGGAGACTTTGCCAGCTGCTCTAGGCACT
CTTCCCGTTTTTTCAAATGTTTTTTATGTGTTTTTGCAAGTGTCGTTTCAGACGCTTGTTTTAAGTGTTTCATATGTCTTCTTTT
GTATGTTGCAACTGTTACATCTAAATGTTTCAAAAGTAAATCGGGTGTTGCACATGGGATGCGTGTGGGAAGCGGACGACGT
CCGGGATGGCACAGGACCACTACTGGGGTGCTCGCTCACAAGCCCGACGCGGTGGGGCGTTCGCTCGCTCGCTGTGTGGGC
ACCGTCTGATGCTAGCGCCCTGAACCGGACGTCCGGGGCGCTGGCAAGTCAGATTTACTAAAGCATTTTTTTCATAACCATG
GTATCAGTAATTTTTGTAATGACAATGTATTTACTAAAGCATTTTTTCATAACCATGATATCAGCAATTTTTCGTAACCATGATAT
CAGCAATTTTTTCATAATACATGGTATCATAGATTTCAGAATAATCTTGTCTTCCCATGGGTGTTGCCTCCGTTGATACTTCCTA
CCAAAGTAATTTGTCTATTTCGCAGTACATTTCCTGTCAAATATGCATTGTTTTTTGTTAAATGAATGAATTCATCATAAGTAAC
GTGAGCATTTACAGG
>SsLRR-RLK78
GACGACGAGTTTCGGCGGCAGCGATAGGTCCTAGACAACGAGTTCCTGCATCCACCGCGATGAGTTTCGACAGCGGCGCCC
TCCACCAGTGGCATCTACTCGTCGTCGAGGAGCACCAAGGTGATGAGCAGTTGTCCAGGTGAGGGTCCGGAGCAATAAAAT
ATTTTTTATGAACTTGGCACCGCAGCAAGTAGTTGTGTCACTCTGTTTTTCTAGCATTGTTGGGAGTTCACGCTGCAATGTGG
AGAGCGACGCCAAAGTCTTCTCTCTTCTTTTGGGCAACACTCAAGCGTACATTGTGGAGGGCCTTAACCATCCAAGGCCATT
GCACTAGCACAGTTGATGTTGATGGATGCGAAGCCGAAGAAGTAGCGCCAGTTGCTAAAGCTCGGCGCCAAGATCCACGGC
GCCGAGCTCGGCGCCAACCTTGGTGCTACATCAGCTCTCGGAAGTTGCTAGCGCATCCAACCTCGGCGCCATGACTCACGG
CGCCGAGATGTGTTACCTCGGCGCCGTGATCCACGGGGCCAAGCACAGGGTCCAAAAACGAAAATAAAACCATCAGGGGT
CTAAATGTGAATATTTTTCAAGAAAAGGGCTAAAAAATAAAAAAAATCGGTATCATCACCTCAACACTCCATCATCCTCAACT
CGCAGTTGCTAGAGGTGGGATCATCAGCCATCGTGGGCACTCTGGTCCCTCTATGGCGGCTACCGTAGCTCAGCAAGAAGCC
TGTGACCTGCAAACACAACCACAAACAAACGGTTAGAACAAGCTAAACGAAGTTAGGGTTAGGGTTATGAGTGTGGCTTAC
CTGCGCAACCGGAGCACTGGAGATGCCATTGAAGATTGAGGTCCACATATCTACAAGAAGAGGATGGAGAGACTAGGTTTT
GGGGGACGAATCTGATGAAGAGAAGGAGGGTGGAGGGAGTGAGCAGGGGAAGAGATTGACGATTAGAGGGGAGAGAAGA
GACTATAGATGACCAAAATGGGACTGTGGATCCGGCGAGCGGTGACCGGAGATGAAGACGAAGACGGAGGAAGGAGGAA
GGATGCGTGGAGGAGACAAGTGAGAGGAGTCGTCGATGCGCCTTTGATGGTTGCAATCGTAGATCCGGCAAGGAGGCGACC
TCAGACAAGCCGAGAGAAGCTCGCCGGCGCCCCACGATGGTTGGAGGCGAGTGGCGGCAGCGCTCCCTTTCTCCGCTCTCT
AGCTAGTGTTAGGCGAGCGAGGCGAAGGCTGATGTAAATGAGGGGGGATGGAGAGAGGCGGGAGAAGGGGCGCGCCATTT
ATACGATGGGCGAATGCGGGGATTGTGGGCGCTTGGGCTTGCAGGGCCTACGGGCCAGGTCAGGGCCCCTCGTGTTGACCG
TTGCGTCGGGCCGAGCTGGGCCGGCATGCTGTGCCGATCGAGGGTGAGGCCCAGGCACGGCCCTAGGATTAGATCAGGCCG
GCCCGAGCCTGACGGAGACCATGTCGTGCCGAAAGACTGGGCCATGGGCCGTGCCATCGGGCTTTATGGCCAAGTATAGAA
ACCAGATCTCTTAGGCATAATTAAACTTTTCTAAAAAAAGGCGTAATTAAACGCAACCTCCGAGCAGGTAATCAATAGTCTCC
GGCTCCTGTGCGCAGAGGTGCACGTATTATCATCTCATAGTCAACCTTGTTGCAAATAGAGTAGTATATATAGCTGCAAAAAG
ATTGGGTATAGCTGCCCGAACATGAGTAAAGCTGATCATGTTACTCGACAGAGAACCCATGTGATGTGAGAGCAAATATATAT
AGCTAGAGAATGGCCGCAGCTAATTGTATCATACAGTGTGCTATGGTATTTGACTTTGATATACATGTGAATTCAGGCGTTTTA
ATATCCTTTCGCGTCTTATATATAGTTATACATACATGGTACCATTACCATGTCAACTTCATAGATTACTTCGGTTTTGATGTGTA
CCATTTTTTTGCCTAGAGAAAACGTTTCTTCTGTTGCTCCTA
>SsLRR-RLK124
TGCCTGGGCTGTGCGTCCATCACTCATCGTGCCCCCGTGAAACACTTGTAACATGAAACAGTTGGATGCAACATACGTCGAA
CACAAATGACATATTTGAAACGTACTATTGCAACATATGTGTGAAACATATGCAATATCCAGATAAAACACTTGCAATATGTGT
GTGAAAACATATGCAACATCCAGATAAAACACTTGTGGCAACATACGTCTGAAACAAATAAAACTCTTTTCAAAACGCTTGC
AACATACCACTACAAACACTTGCAAAAATATGCAACATGTGCAACATCCCGATCTACTTTTGCAACATCCATATGTAACAATT
GCAACATACCTCTAAAACATTTGAAACACTTGAAACACACATTTGCAACATAGGGGAGGGGAAGGCCAAGGCCAGTCGATT
ATGGTCGTCGGGGTGGGATCTGGCAGTGAACAACGACATGCGAGCACCACCAGCACCGGTCATGCTCATGTGTGCCCTTGG
CTCGGCAAGAGAAGACTTGAGGCGCCACGGCACGTGCGCCCCAATGGCCATCGGGGTCAGCGACGCTCCCGATGGCGATG
GGCGATGGACGGATGGTGAGAAAGCGAGTGGCGTAGGTGACTGGGACGCAGGTGAGCGACGCAGGGAGCGAGGGCACGC
GGCGTGGCCACGATCAGTGGCGGAGCTAGGATGTTACACCAGGGTATTCCATGTGAATAAAATTTTGCAATGTAAAAGTCTT
TAGCAAGGAGTATTTGGTGGGGATGTGAAAGCTATTTTCTTTGTCTTTGTAATTGTCTTTCACAATACAAGATAAATATGCTAA
CATATATATTTGTTTGCTTTACCAAAATCACGGGTATTGCTTGGGACTAAAAGGCTTTGTTGTTGTTGTTGTTATTGTACCAAA
ATCATGGGTATTCCAAGGAATACCCTCCCGCCGCCCATGGCCGCGATGGGGAAGGGCACGGCGCGGCAGCCCTGACAAGCA
CGCGTTGGGGGAAGGGGTGGATGGATAAGCGGCCACACTGCTTCGGGCAAAGGGCGATAAGTATTCGAGGGACTAGGGATT
TGTTTTTTGTTTCTAAGAGAGATAGGGCCCGTTCCTATGGAGCCGTGTCTATGACCCATGGAGCCACGTCTGGACAGGAGTC
ACGGACGGATGTCCGTGTTGGACTATTATCGCGAAAGTAATCGCTATGCGTTGCATGATTGATATCATGAAGCAAAATGTATTA
AATTCGTCGAAACTCATCATCAATCGATTAGAATTTGGTTAAAAAGGGTATCCAGAGTGATTCGTATTCGCTCGACGATCCTTT
TGTTAATTGCGCCGATAATGCCGGCCTCGGCAGTTGATTGCACGCTGAGCTCGTGCACGTACGCATCATGCATGTTCTCACTT
AGGCCTGGTCTAGTTGTCAAAAATTTTTGCCTCCACCCATCACATCGAATGTTTAGACACATGCATGGAGTACTAAACGTAGA
CGAAAAAAAACTAATTACACAGTTCTCAGGAAATCACAAGACGAATCTTTTAAGCCTAATTAGTCTATGAAAAGTCTTAAGT
GCTACAGTAACCAACATGTGCTAATGACGTATTAATTAGTATCATTAGATTCGTCTCGTAGTTTCTTGACGGGTTCTGTAATTTG
TTTTTTATTAGTATCCGAAAACCCCTCTCGACATCCTTCCAACACATCCGATGTAACACCCAAAAATTTTCTTTTTTCGAACTA
AATGCAGCCTTATTCCCTACCCAGAGTTGACTTTTGATACCAGACTTCTCCATCCAATCACTCCCGGTCCCAATCACGACGCT
GTTGACCCAGCGCCTGTGAGCCTACCCACAACACGAGACGACACGCTGCTTGATCTATTTATTTAGTTATTATTATATATAATT
AGTAAATTCCGATGCATTATATTGGCCACTCCACCTATCCACCAACCAATGTCCAATGCAAAGTCCTCAGGTGAGACAACAA
GCAGCTCATGCAAGTTGCCA
>SsLRR-RLK143
TAAACAACCTATAATTCCGAATGGTTACAAGTAATTATTTATTCTTCTAACAAAATCTTAATATTTTTCTACTATAAGCAAATCTC
CAACAAATAAAATTTCAAAACATGGAAACCAAATTGAAAACGTAGAAAAAGACAAAAACTCAAATCGGAAAGAAAGAACT
GATCGGAACAAAAATTGGTAACATCGAAAAAGGTGGGTTCGAAGTGAATTAGTAGAGGTTGGTCGAAAATTTTATTTTAATG
GTAGAAATTTTGCCTCTTTAGTATTAGGTATACATATACATATACATATAGATTCATTTCGGGCGGTGGGATTTCTCTTGAGCGG
CGGACGCCCCCTGGCCGCACCCCCTCCCGGCCACACCATGCCCTCCCCTGACAGGGCACAGGAGACGTCACAGGCCAGCC
ATCGCTCTGTGCTGTGCGTCGCTTGGCCCACTGTGCCCGCCATCTAGGGTCCATATTGGGCACATAACACTAAGTTTAGCTAG
ACATATAAAAAAAACCACAATATTTATGGTACATAATAAGTATCATTAGACGTATCATAAAATATATTTTCATAATAAATCTATTT

AAAGATAAAAATGTTGATTCTACCAACCTAGTTAACCTTAAGAATGCTACACTTAGTCCAAACCTAGAATGACACTTTTTCGG
ATGGAGATAACATAATATAGGGAAAATGTATCACACAATTCCTCCCATTACAAAGATATTCGGTGGATCATATCACGTCTTTGA
CTTAAAACGTAAGGAAATTTATGCACACAAGTTAAGTACGTGAAGGGGCTCTGCAAAATGGAAATTGATTCATAGATGGCTA
TTGACAAAAATAAGGCTTGTCTCTTTTTTAAAATAAAATACGTGCATGAGCGCGATAACGTGTGGATCACGAGAAATAGGAA
ACATATGAATAGGAAAAGGGCAAGGCTATACATGAAAGCACACAATAGTCAAAAGTTTCATTCTGATATTAAAAGAACATGT
AAGATTTGCGAACGGATGGTTGGGTTCTTTGTGGTGGAACCTGCCCATCTGGATTCAAGTCCTCGACTTGGTACGTGTGCTC
GTATTTTTCTAGATTTATTTAAGGATTTAACGACACTATGCTTTTAGTGGTGGGCAGCCTCTCTCTTGTTAGTATGACTTCGTCG
TGAATCTCAAGATCTGCCGGCTCAGTCCTTCGAAGGTGCTCATAGAGGTAGGGTTTATATGCTTTTAGTGGTAGGCGACCTCT
CTTTCGATAGTATGACTTCGTGAATCTCAAGATCTGCTGGCTCAGTCATTCGGAGGTGCTCATGGAGGTAGGGTTTATTCTTA
CAAAAAAAGAACACGTAAGATTTAAATGGTACTCTATATGGGTTAGGCAGTGACAGACATGAGTGAAAAGGAGAAACGCTG
CTTCCAACAACTCTAAGACTCCAAAACTCCGAAGAAATTTCAGGGTTAGGCAGAGACAGACATGAGTGAAAAGGAGAAAC
GTTGCTTCCAACAACTCCGAAACTCCGAAGAAATTTCAGGATCAGCTTTGTTTAAAAAAAAATCAGGATGACCTTAGACTAG
TATCACATTTGCCCTATAAGGACACCTGCAACAGTAAGAGCTAGTTACTGCCTCTAAGCCAACATCTCCAATAGTGTCAACTT
TTAGCAATGACTCTCAGTATGATTACGTCTGCATAACTTTCTCACATGATCTTGGAATATGTGTAAGAGCTTAGCTAGCTTTTC
TCTTTCTTGTATTAAAATATGAAAGAAATATTTAGAGCTAGGTCAAAAGTCAATTGTTGGAGCGCCCTAAGTAGTGTGTGAGA
TGAGAAACTTTTTCACAAGATTTCTTTAAGTCAACTTCCTCCAAGCCGGGTGTTAGGAAGCCTTTCCAGTGTCCATTCGTTTC
GATTGGACTTCTTGAGCATTCTTCCGTTTTTAGTACTTTCCAAAAGGGAGCCCTAAGTTATGGTTTGATTCTGATTGTTAAATC
CGAGTCACCAAA
>SsLRR-RLK80
TATCAGTTCCATCAGGTGCGTCCAAAACAATTTATAAGCCTGTGATACGTTCAGGATTAGCACTATCTTCAAACGGACTGAAT
CGAGCTTCCACTTGAGGTTCCACTTGGACTGAATCGAGCGTCCAAAACGATTCCTGAGGCTGTTATGTTTGGCACAAACGGT
GCACGTATCTTGCACAAACACTTACACTGTCTCCAAACGGACTGAACTAGATTCCACATGACCCACGTCACCTAGGCGCTAC
ATCTGGTGCATTCAAAATGATTTTCGAGCCTATGGTATGTTCGGCGCAAACCGTACGTCTATCTTGCGTCAAGATGAGCACTA
TCTTGAAATGGACTGAAATGAGCTTCCACTTGAGCCTCGCAACCTAGGAGTCCCCTCGGTGATTCCAAAACAATTTATGAGC
TCATGGTACGTTTGATGCAAACCATGCACCTATCTCGGACCGACACCAACATTGTCTCCAAATGGACCAAAATTAGATTTCAC
ATGACCTACGTCACCTAGGAGTTCAATCTGGTGCGTCCAAAATGATTTCCAAGCCTATGGTACTTTCGGCCAAACTGTGCACT
ATCTTGGATCAAGATTTGCACTATCTCCAAATGGACTAAAATCAGCTTACACACTTGAGCCTCAAAGCTAGGAGTGCCATTAG
GTGCTCCATAATGATTCTATGCCAATGGTAGTTTGGCGCAAACCGTGCACCTATCTTGCACCGACACTAAATTGTCTCCAAAC
GGACCAAAATCAGATTCCACATGATCCACGTCATCTAGGAGTTCAGTCGGGGTGTCCAAAACAATTTCTGAGCATGTGGTAC
ATTCGGCGCAAACCGTGCACCTGTCTTGCACCGACACTTACACAATCTCTAAACGATGAACATGATTCCACATGACGCACGT
CACCTAGGAGTTGCATCCGGTGCAATTAAAATGATTTTCGAGCCAATGGTTCGTTCGACGCAAACCGTGCACCTGTCTTGCG
TCAAGATCAGCACTATCTCGAAACCGGACTAAAATGAGCTTCCCCTTGAGCCTCATAACCTAGGAGTGCCATTGGGTGCGTC
CAAAATGATTTCTTGAGCTTATGGTAGTTGGCGCAAAGTATTACCTATCTTGCACTGACACTAACATTGCCTCCAAAAGGACC
GAAACGAGATTCCACAAGACCACGTACCTAGGTGTTCCATCTGGTGTGTCCAAAATAGTTCCAGGGCCATGGTATGTTTGGC
CCAAACCGTGCACCTTTCTTGCGTCAAGATTAGCATGTCTCCAAATGGACTNNNNNNNNNNNNNNNNNNNNNNNNNNN
NNNNNNNNNNNNNNNNNNNNNNNNNNNNNNNNNNNNNNNNNNNNNNNNNNNNNNNNNNNNNNNNNNNNNNNNNAAAGG
GCAAGGCTATATATGAAACAGCACACAGAGTCAAAAGTTTCATTCCGATATATATAAAAGAACACGTAAGATTTAAAAGGTAT
ATGGGTTAGGCAGAGACAGACAGGAGTGAAAAGGAGAAACGTTGCTTCCGACAACTCCGAAGAAATTTCAGGGTCAGCTT
AGACTAGCATCACCTTTGCCTTATCCCTTATAAATACTGTGTGATAAGAGACTTTCCTACAAAATTTCTTAAGTCAACTTCCTC
CAAGCAGGCTGTTAGGATGCCTTTCCATTCATTTCGATTGGACTTCTTCAGCATTCTTCCGTTTCCAGTACTTTCCAAAACGG
CCCTAAAATTATGGTTTGATTCTGATTCTTAAATCCGAGACACCAAAACTTTCTGAAAAGGAAAACCCACCAAGATCTTCTAA
AAGCGCCTATGGCTCTGACTGAAACCCCCATTCCTCTTCCAACGTCAAACAAGCTACAATTCTGCATTCAGATCTCCAGTGTT
CTACAGTTGCTGTGCCCTACAGACCTAGTACTTTCTCTTATAGTCTTCTGCATAAATTTCAGCACCCTTTCGTCAATTCTCTTC
ATTATGGTCTTCTGCAAACAACAGGTA
>SsLRR-RLK123
TCCAGATAAGCACATTTGCAACATATGTCTGAAAAAACAGATAAAATATAGGGAACAAAAGCTTGCAACATACATGTACAAC
TATTACAACATATGCAAACATCTCCATATGCTTTTGCAACATCTGCATGAAACAATTGCAAACTACCTCTGAAACATCTAAAAC
ATTTGAAATGTACACTTACAACATATGCTTTCGGCGCAACATCTCTTTGCTACTTGGAAGAAATGGAGGCTCGTCAGCATTTA
AAGTTCATCGTTGTAGAGCTCACCGGTGGCGCGAAGCGATCGCCCAAGGGATCATGATGCCGTCGAGCACATCACAACCAC
GCTCTATGCCCGTCATAGGCCCCTTGAGGTGAAATGAGAACTGGATCTGGATCCACGCGGGGTAGGATGCCCCCCGCCACTG
CCACCATCGGTGGGAGAGGGGAGGCGGCTTGTTGCCACAACCACCATTGAGGAGGAAGGGAGAGAATCGGGGCAAAATCC
ATAGGCGAGGAGGAAGCACCATCGGAGAGGAAGGAGAGGGAGTGTTGCCGTCAGGAGAGGAATGGCCACCACGGCCACC
GTCGGAATGGGACAGGGTGCCGCTGCCACCGTTGGGATGGGAAATGGCATATCGGCACACCGGGCCAGGCTGCGGCCCCTC
GGTCTCGGTCGCGTGAGGTGGAGAGAACCTTATCCTTTCGTCTGGATGATGGAGGTAGCAAGATAAGATTAGAGACGTTGTT
GGACCGTTGGGCTGGTGCCGCAGCCCACGAACACAACCGTCTGGATGATCGGATGCCCTGACCAGATAATTTCCGATTTTCA
ATCGGTGGAGCACCTTTGTGTTCACTTGTTAGTTGTTATACCATCCATCTAATTTTATTCTACTTATTACTCCATTGGTCCTTTAA
AAAATGTCATTTTTGTTTTTTGAAGAGTCAATAAACTAAGTTTAACCAGATATATACAAAAAATAATACTAATATTCATGCTAC
ATAACAAGGATCTAATCATTAGATGTATCATTAAATATATTTTCATAATAAGTCTTTTTGGAGATACAAAGGTTGATTCTAGAAA
GTTATACTTGGTCCAAATCTAGCATGACATCTTTTTTTGGATGGAGATAACAGAACATAAGGAAAATGTATCATAAAATTGTTC
CCACTACAAATGTATTTTGTCAATCAAATCACGTCTTAGACTTAAAACATAAGGAAAATTATGCACACAAGTTAAGTACATGA
AGGGGCTCTGCAAAATGGAAATTGATTCATCGCTGGCTATTGACAAAAATAAGGCTTGTAGGGATCTTCTTTTAAATCAAATA
CGTGCATGAGCATGATCACGTATGGATCACGGCTCACGGAAATAGGAAACATATGAATAGGAATAGGAAAAGGGCAAGACTA
TATATGAAAGCACACAACAGTCAAAAGTTTCATTCCGATATATATAAAAGAACACGTAAGATTTAAAAGGTATATGGGTTAGG
CAGAGACAGACAGGAGTGAAAAGGAGAAACGTTGCTTCCGACAACTCCGAAGAAATTTCAGGGTCAGCTTAGACTAGCAT
CACCTTTGCCTTATCCCTTATAAATACTGTGTGATAAGAGACTTTCCTACAAAATTTCTTAAGTCAACTTCCTCCAAGCAGGCT
GTTAGGATGCCTTTCCATTCATTTCCGATTGGACTTCTTCAGCATTCTTCCGTTTCCAGTACTTTCCAAAACGGCCCTAAAATT
ATGGTTTGATTCTGATTCTTAAATCCGAGACACCAAAACTTTCTGAAAAGGAAAACCCACCAAGATCTTCTAAAAGCGCCTA
TGGCTCTGACTGAAACCCCCATTCCTCTTCCAATGTCAAACAAGCTACAATTCTGCATTCAGATCTCCAGTGTTCTACAGTTG
CTGTGCCCTACAGCCCTAGTACTTTCTCTTATGGTCTTCTGCATAAATTTCAGCACCCTTTCATCAATTCTCTTCATTATGGTCT
TCTGCAAACAACAGGT
>SsLRR-RLK363
TTTATCCATTTTTTAAAATTAAATTCTCCCGTTGGTTCGAATTTAATAATGAAATTAATAACATCAAATACGCAGCGGAAACATT
TTTACCATTCTCATAATAATATTTTCCAGAAGCAACTTTACTGTTAACTGAACAAAAAATATCGTTGGATAAATTTTGTACAGA
AAATATCATCAAATTTAGGTCAAAGTTGCATCAAAAGGTCATTTAAATTAGATTCTGGAAAAATTAAAATGGAATTTTGTTTTG
TACTATACAAATTTCACATGTGGCCCGAAAGCATGATCTCTGTGTGCTCGCGGCCCAGCATAATGCGGCCCAGTTGGCCCGTT
TGCGCGCGCTCGCTGGCGCTCCCGCGCCCAGGCCGCAACCTGGGCCTGGGCCAGGATTCTGGCCCTTCCGCGCTGCCCGCC
TGGGCCGGAAGCTGGTCCAGCTGATTCTGGCCGTCCATCTGGATCTGACGGTTCACCGTCCAGATCGGCGGATCAAAACCCC
CGGCTGCTGCCCTGTGAACCCTAATCCCGTTTCATTTGGTTCCTCTTTTCTCTCCTTCGTTCATCTGAGCGGCGCACAAGAGC
ACCAGCAGCAGCTGCCGCCCATGGCGGCCTGGAGAGGAAAGTTGGCGCCGCCCCGCGCCCTCTCGCCGGCGTGCGTGCTC
GCCCAAGGCTGAGCACGCCGTCGTTGAGGGGCCGCATGGTGGTGCCCTGTTACCGTCGGATGTTCTTTTCCGCGCGGCTCTG

TGTTCACCGAGGTGAAGCGTGGCTCTGTATTCTGTCCCGCGCGCCGGCCATGAGCGGCACCGCGGTCTTCTTTTCGCGCGGT
GGCGGTGCCGCCGGCGGTGAGGAAAGCCCAGGTAGGCCCCCTATCTTTAGGGTTAGGGTTTGTATGGAAGTTCTTTTCCGAT
TTCAAATCGGTTCTTTTCTTTCTATTTCTGTTCCCCGATCTAGATCTACTACCTAGGCCCGAATGTACACTTGTTGTCTATGCAC
AAGTTGGAAAAATACTGAAACATAGTCAAGCGGAATGCAATCTATCTTTTGACACCCTAGGCTCCATTGTATTGAATATTTTCT
CTATTTTAATACAAAGACGCGCAAATTTTTTGCGTAATCCACGAAAGAATACAACCTATCTGCATAATCTAAAGTCTTGTCCGT
AGTACGCTGCAGCCTTCTCATTCAGCCTGTTCGCTTGAGATTATCAGCCAAATCAGCCAGCCAGCCAACATTGTTTTTCTCTC
GAAAAAAAATCAGCCAACGATACTTTTAGCCAGCCAGAGCAAACACGGTTGTCAACGGATTCTTTCCGGCGCGAGCTGGCA
CAACCACCTCTGTACCAAGCACGAACTCCCCTACAAAATTTAGACCGCAGTTTCCGGCGCCGCGAAAAAGTCAGAAGTCCA
CGGGGGCCACGGGCCTCGGCCAACGAAGTCGTGCTCGCAGCAAGGCAGTTGACCAGCCCCTTGCGAAGTCAACCCTCCGC
CCATGTGAATACGTATATGTGATCCACTGCCACTACAACAGACAGCCACTGACATGCCGGCCCACAAATGACGTGGCAAGCG
GCTGTGAGCATGCATGGCCCACTTGCCAATGACTCCCCCGGGTATGTTTTCTCGTACACGCCAGCCGATCGACGTCGTCGAC
GACTTGCAAGTTGCCATGCAACGGCTACGGCGTTGGGCCGGATAGCTCGTAGCTGCGCTGACACGGACACACGGTTTAATC
ATAGTGGTGTTGATGTAGTACAAGTGTCCTTCGCTAGACCGTGTGTATATGACGCTCTGGTCGCCGCTTGCATGTGCAATGCA
GCAATCCACGCCGCGCACAATGGCACCGGCAGTAGTGTCGTCGTCGGGGCTGCTGCTTCTCTGCCTCGCCGCCGTCGCGTCC
GTCGCCGGCGTCCTCCAAGCTCGCGCGCAGCCTGACAGCATTGGTTTCATCAGCATAGACTGTGGCCTGTCCGGGACGGCG
AGCTACGTGGACGGCACCACCAA
>SsLRR-RLK383
AATCTTTATACACATGCATGAAGTATTAAATATAGATAAAAAATAAAACTAATTACACAGTTTAGACGAAATAGACGAGACGA
ATCTTTTAAGCCCAATTAGATCATGATTGGACACTAATTACCAAATAACAACAAATTTTACTATAGTATCATTTTGCAAAAATTT
TTGCAACTAAACGGGGTGCGGAGCCAAAAGCGCACCCGTTGTTTTGCTGCAATCCGACGGCGCTTCGCTTTCGTGGCGCCG
TGGTCCGCCTCAAAGGATAGCTTCCGATTTGTCCACCGTGCCCGTGGTCCTCGAGCAAAGCGGTGCCGATGAACCGTGCGG
GCAACTGGGGGTCTGTTTAGTTGGGAAAATATTTTGGATTCGGCTACTATAGCATTTTTTTTATTTGGTAATTTGTGTCCAATTA
TGGATTAATTAGGCTCAAAAGATTTATCTCGTGATTTATAACTGAACTGTGTAATTAGTTTTTTCTTTCGTCTACATTTAGTACT
CCATACACGTGCCACAAGATTTGATGTGACAGATACTGTAGCACTTTTTGGGTTCTTTTTGAACTAAACAGGCTCTAAAGATT
TGTATACAGATCCAATGAACAGAAGGTACGGGACAAAAAAATCTCGTGGCTACACGCACTGGACAGTCAGTTGCCACTTGC
GAGATGACTCATAGTCGTGTTTTCTTTTGTTTCAGAGAAGAAAAGGAAGAGGCGGGGGGNNNNNNNNNNNNNNNNNNNN
NNNNNNNNNNNNNNNNNNNNNNNNNNNNNNNNNNNNNNNNNNNNNNNNNNNNNNNNNNNNNNNNNNNNNNNNNNNNNN
NNNCAGAATGCATTGCATTTCGCCACCATGCCCGGCCTTCTATTGTTGATACCTTCTGTAATGAACTCATTTCGTTACCCCGCC
TACCCGTGGTCCATCGATTGCTAAAAAATAACTCATCCAACTTGGAGACATGGCGGCACATATATTGCGTTTAGTCTACTGTG
GTTGGAATGGAATAGTGTTGGTATCAGACCAGCAGAGTGCGTTCAGTTATCATGCAACCGTGTACTCTGTCAATATGCTTAAT
GCATGACTCCAATACTTCAATTTGCCACAATATATACACTAGTGTCTTTATTAAACCTTAAGTACGCAATGACATTCCGTAATGC
GTTCCATGAACCCAAGTTTGACAGGGTTATTATGGTGCTCTTTTCTTATTCAAAATGCCAGAATGTATACTTGCTGTCTATGCA
CAAGTTGGAAAAATACTGAAACATAGTCAAGCGGAATGTAATCTATCTTTTGACACCCTAGGGTCCCGTTGTATTAAACATTT
TCTCTATCTTAATGCAAAGACGCAAAAATCTTTTGCGTAATCCACGAAAGAATGCAATCTATCTGCATAATCTAAAGTCTTGTC
CGTAGTACGCTGCAGCCTTCTCATTGTCAATGGATTCTTTCCGGCGCGAGCTGGCACAACCACTCTCTGTACCAAGCACGAA
CCCCCTACAAAATTTAGATCGCAGTTTCCGGCGCCGCGAAAAAGTCAGAAGTCCACGGGGGCCACGGGCCTCGGCCAACG
AAGTCGTGCTCGCAGCAAGGCCGTTGACCAGCCCCTTGCGAAGTCAACCCTCCGCCCATGTGAATACGTATATGTGATCCAC
TGCCACTACAACAGACAGCCACTGACTGATTATTCAAAAAAAAAACAGGCACTGACATGCCGGCCCACAAATGACGTGGCA
AGCGGCTGTGAGCATGCATGGCCCACTTGCCAATGACCCCCCGGGTATGTTTTCTCGTACACGCCAGCCGATCGACGTCGTC
GACGACTTGCAAGTTGCCATGCAACGGCTACGGCGTTGGGCCGGATAGCTCGTAGCTGTGCTGACACGGACACACGGTTTA
ATCATAGTGGTGTTGATGTAGTACAAGTGTCCTTCGCTAGACCGTGTGTATATGACGCTCTGGTCGCCGCTTGCATGTGCAAT
GCAGCAATCCACGCCGCGCACAA
>SsLRR-RLK351
ACACACTTGTGCAAAGATTGCCCTTTCTCAAAACAAGTATGGTCAGTATTGAAAACCTGGTTTGGACTATCAGCTATCGACA
CAGTGGGAGACAATGGGTCACTCCATGGATACTGGCGTAGATGCAGGCTGCGGATAGACAAACCCCCACAGGAGGAACTTT
GATGGCCTCATGATCTATTTTTGGTGGAACATATGGAAGGAGCAAAATAGAAGAACTTTCCAAAACAAAGGTTTGCAACCAA
GGGAGGTGGCACGTCTATGCAAGGAGGAGATAGATCAATACCAGTTGGCCACTAGACCAAATGCAGAAACTCAGCAATAGC
CCCCCATTTTTTCTCTCTATGTCTTGGTGTTCTACTTTTTTCAGGTTTGTTTTGGCTAAAATAGAGTTGAGTCTTTAGTGGAGG
TTGTAAAAAATCTGTAACATTTTTTTTCTTTTTTCTATCGTCTAATAAAAATCTAGTAAAAGTTTTTTGTCGTCTTTTGAAAAAT
ATACTTGCTGTCTATGCACAAGTTGGAAAAATACTGAAACATAGTCAAGCGGAATGCAATCTATCTTTTGATACCCTAGGGTC
CCGTTGTATTGAATATTTTCTCTACCTTAATACAAAGACGCTCAAACCTTTTGCGTAATCCACCAAAGACCACGTTTAGATTGC
AAAAAAAATCCAACCTGATGAATAGTACCACTTTCGTTTTATTTGGCAAATATGGTCCAATCGTGGACCAACTAGGCTCAAA
AGATTCATCTTGTGATTTCCAACTAAACTGTGTAATTAGTTATTTTTTTACCTACATTTACTACTTCATGCATGGATCTAAAAAT
TGATATGATGGAGAGAGAGTGAAAAAATTCTAACTTTTAGAATGCATCTAAACAAGGCCAAACAATGTAATCTATCTGCATAA
TCTAAAGTCTTGTCCGTAGTACGCTGCAGCCTTCTCATTGTCAATGGATTCTTTCCGGCGCGAGCTGGCACAACCACCTCTCT
GTACCAAGCACAAACTCCCCTACAAAATTTACTCCCTCCGTTCAATAAAACATGCAATTCTACCATAGTATCACGTTTTAGTAT
CTTAACTTTGACCAATTATAAATAAAAAAGTATTAATATCTATGATATCAAATAAATATCATTAGATTAATTATGAAATATATTTTC
ATAATAAATTGATTTGAAGCCATAAATATGAATACTATTTACTGTAAATTTGGTAAAAAATGAACTACTTTTTTTAGATATATAA
CCCATAATTACATGTTTTTACGGACAGAGGAAGTAGATCGCAGTTTCTGGCGCCGCGAAAAAGTCAGAAGTCCACGGGGGG
CACGGGCCTCGGCCAACGAAGTCGTGCTCGCAGCAAGGCCGTTGACCAGCCCCTTGCGAAGTCAACCCTCCGCCCATGTGA
ATACGTATCTCTAGGTGATCCACTGCCACTACAACAGACAGCCACTGACATGCCGGCCCACAAATGACGTGGCAAGCGGCTG
TGAGCATGCATGGCCCACTTGCCAATGACTCCCCCGGGTATGTTTTCTCGTACACGCCAGCCGATCGACGTCGCCGACGACT
TGCAAGTTGCCATGCAACGGCTACGGCGTTGGGCCGATAGCTCGTAGCTGTGCTGACACGGACACACGGTTTAATCATAGTG
GTGTTGATGTAGTACAAGGTGTGTTCACTTCACATGCAAACCTAAAATATTTCCTCTTCTACTCATCACAAGGAATTTTTAGAC
ACATGCACGGAGCATTAAATGTAGGTAAAAAAGATAACTAATTGCATAGTTTGTTTGTAAATGTCGAGACAAATCTTTTGAAC
CTAGTTAGTCCATGGTTAGATAAATTTTACCAAATAATAACAAAAAATGTTACAGTAACTTTTCACTCATTTTTCACCAACTAA
ACACAACCCTAGTGTCCTTCGCTAGACCGTGTGTATATGACGCTCTGGTCGCCGCTTGCATGTGCAATGCAGCAATCCACGCC
GCGCACA
>SsLRR-RLK173
TACTTGACACTAGTGTGCAACACGGTCACACACCTCAAATTACGCAGGGCATGGACTAAGCATTTGCGGTGACATTCGAAAT
TAGGATTTTCGGGTTCGGATCATACCACGGAGAAGCACATCCAATCTAGATAAGTAAACTTGTTTTATTTGATGCATAACAAA
GTTAACCTAACATGGATGCTTTGCAATGCATTATGATGACATGGCAGATTAATTAGCACTATAACACCCGGGGTGTTACAAGA
ACTTTATATATTCTTGTGTTCTAACAAGTTAATTCAAGGCCATGTTCAGTTGGCTGGAAAAACGGCTGATGCTGATTTGTTTTG
TGAGAAAAATATTGTTATTTCGCTGAAATGGTACAACTAATATAAATTATTATGAAAAAGTAATTCAATTAACCACAAAGTACG
CTATATATGTTTCCAAATTACTCACCATTGTTAGATTAAAAGAAAGCATAATGTTATATAACGCTATATAAGTAGTAACATGCAT
ACATTCAGATAAGTATTTATTTTATATGTGAACAAATTTCTCTTGATACTGACAAACCATTATAAATTGCATTAATACTCTATGAC
TACAAATTTTATAAATTGTTATTTAAAATAGAACTTTATATATTCTTGTGTTCTAACCAGTTAGATTCAAGGTCTTGTTCGGCTG
GCTGGAAAAATAACCGATGTTGTGTTGATGCTGATTTGTTCTGCAAGAAAAATACTGTTATTTCACTGAAACGGTACAACTAA
TAAGTTCAAGCGAACGGGAGGCACGACTAATGGACTAGTTGAAGGATAATAGACATTTGCTTCGTGTATGATACCGTATATCC
TCTTGTTGAGTATAGAATAGAATAGAATAGAATAGAATACTAATTTATGCACCATGATTATCCTATATATAATGTCTCTAGTGGCT

AGTTTTCCTTAAAATGAGCCTATTCGCTTCGCTGAAAAGCCATGGCTAAAAGTTCTGTTTGCTGATTTGTTACGAGAGAAAA
ACGCCGTTTAAAAATACAAGCTGAAAAGTTAGGTCTTCGATGGCAATACTGTTATCCTATTTTTCCTCTAAGAAATGGCGACC
CGTGGATCCATTGGATCACGCACAGAGCGACTCGGACGCAATGACAGAGAAAATCTAGAATATTGAACACTAGAGTAAGTA
CTCCATTCCATCAAGATACCTATCAGAGTACTATACATAGATCATTATAGAAACACATCGAAAACGACAGTACGCATGATCATG
TACTGATGTACCACCTACTACGAAGTACGAACTGTTACTTTTTGTGACCTGATTCCTCAAGAAACTGTACAACAACTGATAGC
AGGGAGCATCGCAAGTCCTCTCCGCGTACCACTGAGTCACTTGGGCAGCAGCAGCTACATCAGTTGAGATGAACACTGTTG
CATTGGCCACGCACCCGTACTATCTATTCCAAACTGCCAAGTGGCAATCCCCGTGTCGATGGAGATGGAGGTCGAACCGTGG
ATCCCAGGGACACAGGACAGCAGAAAATATCCCAAATCGCCACCAGAAAACCAGCACCAACCAGCAGGCAACCACCCCAT
CCATTCATCCATCCATCTCGTCGCAGGTCGCAGGAGAAGAGAAGCAACCAAAGCCACCCTCCGCATTTTTCTGCGGGATCAT
TAACGCCGACTCGGACGCTTTCGGGCCGGATCCTCCGAACGCGAACAAAGGAAGGAGCTATCCGCCGGGGCGTCACTGCA
CGCGTGGCGAGATAAAATACGCGACCAGTTTGTACAGTTGTAGGGTATTTCTACCGTACCGGGACGCGGTCGCCCGCGGTTG
ACTGACCTGAAACCACGGCCAGCGCACGCGTTTCTCCCTTTCTAGCCCCTGTAGTCGTTCGTTCCCATCCCATCCCACCTGTA
GAGCGGAGCCGGACAGAGGCAGGTAGGCCCGGCTCCGGGGACGGAACGATTTGTGAGCGCACGGCGCGGCGACGTGCGC
GATATAAAAAGCAA
>SsLRR-RLK271
GTTAGTGTTAACCAACTGGTGCAGATACCTTCATTACTATCCATGCCTTTTCACATTACAAATCACCATAGATACTTAGTATCCC
TGTTGGTTGATATTACAAGCCAGCACTGATAGTTTCCTGTTTCAGAATCTNNNNNNNNNNNNNNNNNNNNNNNNNNNNNNNNNN
NNNNNNNNNNNNNNNNNNNNNNNNNNNNNNNNNNNNNNNNNNNNNNNNNNNNNNNNNNNNNNNNNNNNNNNAGCCCCAC
CCGGACATCTGCGCTCGCTCGGTGGAGCCCAGCAGCTGCGCAAGTGGGTCCCCAGCAGCAACACCCTAGATAAAATATTTG
CAACATGCTTCTACATGAAAACACTTGAAGCGTCTGAAACACATGCAAAAAAAACATATAAAAACACTTGAAAAAAATCAT
TGCAAAACATATAAAACATTCAGATAAAACTTGCAACATATACGTGAAACATATGCAACATATAAATAAGCACACTTGCAACA
TATGCCTACAAAGCTGATGAAACATTTAGAATGTACGCTTGCAACATATGCGTACAATATTGAAACATGTCCAATATCACAATC
TATTTTTGCAACACCCATATAAAACACTTCCTAACATACCTCTGAAACATCTGAAACAATTAAAACATACTCTTGCAACATGC
GCTTTTAACACAACATCCGCTTGCTACTCGGTAAAATGGAGGCTTGTCGGTGTGTCATGTTCATCGGAGGTAGCGGCCCGGC
GACGCTTCTAGGTGGCGAGCCAGCGGCAGTGGCTGCATGTTGCGGAAGGGAGGCATTGGCTGTAGGAGTGGTAGGGAGGA
CGTGGCCGCGCGACTTGGAGAGGGCAGCCGCGCGCCGTGACTCGGAGGCAACCACGCGCTGCGCCTGAGAGGTGGCCAC
GCATAGCGCCGAGCAGGGCCTAGGGCTGTGCGCTCAGAGGCGCAGACGAGCGGATAAGAGCGATATGAAGAGGAACGAGT
GGATAGAAATGAGTAGATGAGCTTTTTTATTTTTTTTGAAACGAAGGATAGTAGGTTGTTGGGTTTGCATTACGGCCTAGCAA
CGGATTGTCCGGATGAACGGATGCCACCATTACCGCAGGACTATCTGTATCATCCCCAAATGTTCAGTCTTTATGCACACACA
GAGGAACTAATGGCTAATGGTTTTTGGTATAGTTTCACGAGCAAAATTAAGCGGCTAGTGACAAATGAAATGAAATCGTCAA
CAACCTTGAGTTGAAGATTTGCAACAATTAGTATTTTTTAATTTTATTTCTTTGATTTGATCCTTTTTTTTTACATGTGTTTGATT
TTGATGCAACGTGTAAAAAGCTGTGGCCGCCTAATATATAGTGACGGGCCCACCTGACAGTGGTAGCACACTTTTTTTTTTCC
CGCCCTTTGCCAAGGGAGGGGACAGGTCGTCGTCAGGGAGGGAAGGCGGTGGTGGCGGCGGGTGGGAATGACGTCCGAC
GAGCTCCAGTCCAGGATGGGGATGAGGACGAGGAGCTTCACCTGCCGTTCGCCGCTCGAGTTTGACCTCTTCTTCCTGGTTT
TTTTCGATATTTCCTCCCCGCGCGTGGATTGGCTCGGTCTATTCAATCGCAGCAAAATTCGACGAGCTGATGTCAGAAAATGG
GGAGAGGAGACAGACGACGGGTGGAGAGCGAGATGCCGAGAGGGGAGGGGAAGGCGAAGGCGAGCAGAGCAAACCAAG
TCTGTTTCGGCACCAACCCCGTCCAATCCAATCGCTGATGCTGGATGATTGGTCTGTGTCTGAGGCTCAGCCTTGCGTTCATT
CCTCTTCATTTAATGCTTCTGAGAGCGGTCAATGCGGGATGGTTGGGTGGTGGATTGGGGTTTGGAGATTCCATCTACTCTCT
CCACCTTCCCGGAGAGGCAGCAGCAGAGGAAGGAAGGAGGATGAAAGAAATGGTCGCAAAGAGGTGGAGGGGAGGAAA
GGGTCAATTGCAGCTCGGTGTCACTCAACCTTAACACGAGC
>SsLRR-RLK283
CGTCGTCGCCGGTGTCTGCTCCGCCGGCATCGCCACGCTCTCGCGCCGACCAGCCGGTGTACCACCCGCCTCTTCTTCACCG
GGACCCTCGTCACACTCACCCGATGGTGACGAGGCAGGCGTCTCGCCCGCAGGCCCTCACCGCCGCCCCTGCGAGCCGGG
ATCTCTCCGGTACCCTCTTCCGTCCGCGCGGCCTTGGCGGATCCTCACTGGCGCCGCGCGATGGACGAGGAGTACGCGGCCC
TCCTCGCCAACCAGACGTGGGACCTCGTGCCTCGTCCGCCGGGCTCCAACATTGTCACCGGCAAGTGGATCTGGACGCACA
AGAGGCGGGCTGATGGTTCTCTGGAGCGCTACAAGGCTCGCTGGGTCCTCCGCGGCTTCACTCAGCGGCCGGCATTGACTA
TGCTGAGACCTTCAGTCCTGTGGTGAAGCCAGCTACAGTCCGCACCGTCCTCTCCCTGGCTCTCGAGCGCTCCTGGCCTGTG
CATCAGCTGGATGTCAAGAATGCCTTCCTCCATGGCACTCTGACTGAGACGGTCTACTGCAGTCAGCCGGCAGGTTTCGTGG
ATTCTTCTCGTCCTGACTTTGTCTGCCGGCTCAACAAATCTCTCTACGGGCTCAAGCAGGCACCCCGGGCTTGGTACTCTCG
ATTCGCTACCTTCCTGCAGACTCTTGGGTTCACGGAGGCCAAGTCTGACACCTCCCTGTTCATATACCACTATGGGGGTGAG
ACTGCTTACCTGCTCCTCTATGTGGATGACATTGTTCTCACCGCCTCCAGCGAGTCTCTGCTCCACCGCATCATTCCTCCTCCA
GAAGGAATTTGCTATGAAGGATCTTGGGGTGCTCCACCACTTCCTCGGCGTCACTGTTGAGCCCCGACCTTCAGGCCTGTTC
CTCCACCAGCGGCAGTTCACTTGTGACATTCTGGAGCGAGCAGGGATGACCGCCTGCCAGTCCTGCTCTACACCGGTGGAC
ACCCAGGGCAAGCTGTCAGAGGCTGGGGGTCCGATGCTTGGGCCAGCAGACTCCACCGCCTACCGGAGTCTTGCTGGCGC
GCTTCAGTACCTCACTTTCACACGGCCGGACATCACTTATGCCGTCCAGCAGCTCTGCCTCTACATGCATGCTCCGCGGGAG
CCCCACCTGGCTGCTATGAAGCGGCTTCTCCGGTACCTCCGTGGCACACTTGGCTACGGGCTGCTCCTCGGCCGTCCTCCAC
TGCCGAGCTGGTTGTCTACACCGACGCTGACTGGGCGGGATGCCCGGACACCCGCAGGTCCACCTCCGGCTACGCAGTCTT
CCTCGGCGGCAACCTGGTCTCTTGGTCGTCCAAGCGCAGCCGGTGGTTTCCCGCTCCAGTGCCGAGGCGGAGTACCGCGCC
GTGGCCAACGGCGTGGCCGAGGCCGCCTGGCTCCGCCAGCTCCTGGCTGAGCTTCACCGCCCCTCGCCAGGAGCACGCTC
GTCTACTGCGACAACGTCAGCGCGGTCTACCTCTCCACCAACCCCGTGCAGCACCAGCGGACCAAGCATGTGGAGATCGAT
CTACACTTCGTCCGCGACCGGGTGGCCATCGGCGACGTCCGGGTTCTTCACGTCCCGACCACCTCCCAGTTTGCGGACATCT
TCACCAAAGGCCTCCCTTCCTCGACCTTCTCCGAGTTTCGCTCCAGCCTCAACGTCTCCGTGGCTAGTTGTGGCTGCGGGGG
GTCCTGTGTGTGTTGTGCTCCTACTTGTTTTCTTTCTTGTTCAGCCTTGAACTCCGCTGCGCCGGTAGTTCAGACTGCGGGGG
GGTGTTGGAGTGTGATTGTATTAGGCCCATGAGGCTAGGCCCATGTGGCCCATGTATGGTACCTATATAGCTACCCTTCTAGGG
TTAGCCCAAAATAATTGAGAATCACTTTCTAGCAAATATCTTGTTGGATGGCAAGCAGGCTAAGTGCGAGGCATAAGGTTGT
GGGTTCGACTCCCCCGAATACCAAGTGTGTGAGAAAATG
>SsLRR-RLK284
ACAGGAAACTATCAGTGCTGGCTTGTAATATCAACCAACAGGGATACTAAGTATCTATGGTGATTTGTAATGTGAAAAGGCAT
GGATAGTAATGAAGGTATCTGCACCAGTTGGTAACACTAACTGGCATAGATAGTCACCAAATCCATGCCAATTGATAACACTT
GGGGCCTTGGGGGGCTCATTGTTTCCAGCTCGTCTTATGATCCAGCAATGATGCTTATCTGAGTCATTTCCCATTTTTTTACGC
AGGGATGAGTCTTTTTTTTAGTAGTGAGATTTGTTTAAGTAGTTTTGATTCATTATTGATCTTGAATAGTAGTAAAGCCCCCGA
AAATAGAGTGAAGTATATTGCAACAGCAGGCTAGCTTGAGCTACTGATCACATGATTATAGTGTTATTGTTAGTGAGAAAATA
GTGCATTAGAGATCAGAATAAAACCAGATTTAAACCAAGACTACCACTTGATCTACAGACAGTTCGATGAAGTACTTATAGA
GAAAATTAAAGAAATCATCACAAGTTAAGTAAGCATCTATTTCACAAAAGAAATTTAGACCCACACTCCAGGTTAATATATTG
TGTGGTTCCCAAAAATGTAGGTCAGCTCTAGTTAAAATTCAATGAAGCTATGAGATAACAAATGATGGCCTTTATCAGCGGCT
AGGCACGAAGGAAGGGGAGAAGGACATTTATAGGATGGCTAGAATCCGCGAGAGGAAGACAAGGGACATCAACCAAATAA
AATGCATCAAGGATGGGACAGATCGACTTCTGGTGAAGGATGAGGAGATCAAGGACAGATGGAGAGAGAGTACTTCGACAAG
CTGTTTAATGGGGAGAATGAGGGTCCTGCCCTTGAGTTGGACGACTCATTTGACGATACCAACAGACGCTTTGTGAGGAGA
ATTCAGGAGGCAGAGATCGGAGAGGCTTTGAAGAGGATGAAGGGAGGTAAAGCGATGGGCTGATGGTATCCCCATCGAGGT
ATGGAGATACCTAGGGCGCCAGAGCGATAGTATGGTTGACTAAGCTTTTTAACCTTATTTTTCGATCTAACAAGATGCTGAAG

AATGGAGGAGAAGTATATTTACCTATCTTCAAGAACAAGGGAGATGTTCAAAGTNNNNNNNNNNNNNNNNNNNNNNNNN
NNNNNNNNNNNNNNNNNNNNNNNNNNNNNNNNNNNNNNNNNNNNNNNNNNNNNNNNNNNNNNNNNNNNNNNNNNNNNNTC
ACGTCCCGACCACCTCCCAGTTTGCGGACATCTTCACCAAAGGCCTCCTTCTCGACCTTCTCCGAGTTTCGCTCCAGCCTCA
ACGTCTCCGGTGGCTAGTTGTGGCTGCGGGGGGTCCTGTGTGTGTTGTGCTCCTACTTGTTCTTCTTGTTCAGCTTGAACTCC
GCTGCGCCGGTAGTTCAGACTGCGGGGGTGTTGGAGTGTGATTGTATTAGGCCCATGAGGCTAGGCCCATGTGGCCCATGTT
ATGGTACCTATATAGCTACCTTCTAGGGTTAGCCCAAAATAATTGAGAATCACTTTCTAGCAAATATCTTGTTGGATGGCAAGC
AGGCTAAGTGCGAGGCATAAGGTTGTGGGTTCGACTCCCCCGAATACCAAGTGTGTGAGAAAATGTGTAACCCTGCTGGGA
AAAGGTGTGACCTGGGGTGGGGGGGTTGTTGAGGTGGCTTTTTTCTTACAATTGTTTTGGGGCCAAAGGACATCTTTTTTTA
TTTGATTATCCCTGTTGGTTGTCAGGGACACTCACATCCCCCGCCGGTGGGCAACTAGCTGGGATACTTTTAGTATCCCTATCG
TTTTCACGGTCATGGGTTACAAACCTGGATGGATAGCGTGTTCTGTAGAATTGTAGTAGTGTCGATTCAGTGTACAAAAAATC
ATTGCACATTGAGCTTCTTGTTTTCTTGAAATATTTTAATGCATTTGCAATTTCAGTTTGGAATTGCATTGCATCCATTGTCCTA
ACTACATCTTTTTTAAATGATTTGCAGA
>SsLRR-RLK272
GGTCTCTTGGTCGTCCAAGCGCCAGCCGGTGGTTTCACGCTCCAGTGCCGAGGCGGAGTACCGTGCCGTGGCCAACGGCGT
GGCCGAGGCCGCCTGGCTCCGCCAACTCCTGGCTGAGCTTCACCGCCCACTCGCCAGGAGCACGCTCGTCTACTGCGACAA
CGTCAGCGCGGTTTACCTCTCCACCAACCCCGTGCAACACCAGAGGACCAAGCATGTGGAGATCGATCTACACTTCGTCCG
CGACCGGGTGGCCATCGGCGACGTCCGGGTTCTTCACGTCCCGACCACCTCCCAGTTTGCGGACATCTTCACCAAAGGCCT
CCCTTCCTCGACCTTCTCCGAGTTTCGCTCCAGCCTCAACGTCTCCAGTGGCTAGTTGTGGCTGCGGGGGGGTCCTGTGTGT
TCTTACTTGTTTTCTTTCTTGTTCAGCCTTGAACTCCGCTGCGCCGGTAGTTCAGACTGTGGGGGGGTGTTGGAGTGTGACT
GTTTTAGGCCCATGTGGCTAGGCCCATGTGGCCCTTATATGGTTCCTATATAGCTACCCTTCTAGGGTTAGCCCAAATCAATAG
AGTCTAAACACTTTAGCAGATGGCAAGCAGGCTAAGTGCGAGGCATATGGTTGTGGGTTCGACTCCCCCGAATACCAAGTGT
GTGAGAAAATGTGTAACCCTGCTGGGAAAAGGTGTGACCTGGGTGGGGGGGGGTTGTTGAGGTGGCTTTTTTCTTACAATT
GTTTTGGGGCCAAAGGACATCTTTTTTTATTTGATTATCCCTGTTGGTTGTCAGGGACACTCACATCCCCCGCCGGTGGGCAA
CTAGCTGGGATACTTTTGAGTATCCCTATCGTTTTCACGGTCATGGGTTACAAACCTGGATGGATAGCGTGTTCTGTAGAATTG
TAGTAGTGTCGATTCAGTGTACAAAAAATCATTGCACATTGAGCTTCTTGTTTTCTTGAAATATTTTAATATGCATTTGCAATTT
CAGTTTGGAATTGCATTGCATCCATTGTCCTAACTACATCTTTTTTAAATGATTTGCAGACAATTGGACAATAATAATTTCTCTG
GAAGTACAATTCCTTCATCTTATGGCAACATCTCAACACTTCTGAAATTGTAAGTTTGCTTTATCTCCTTTTGTATTCTGTATTC
TTATATGAGTTCTCACGTAAGTTTTTGTATCAGTAATAGGAATCCACATTTGTTTGACAACCCTCGTCATTATTTGCCAATTACA
ATGATTAGATTCAATTCTTGACACGAAGAAGTATCTGGACTTGTAGATTTATACTTCTGTATTTTTGTCTTAGTTATGCACTAGT
TACTATTGCATGGATATTGAGTTAATAACATGTTTGGTCCCTCTGAAGGACCTACCAGTTGTCTACATAGCAAAATGCATACTA
TACATTTTCCAATTATTGATTATATGCTTAAAATGATGATTAACGAAACTTAGACATTTGATTAATTTCTGATCAAGCTACTTATT
TCTTGTAATGATCCAGTTGAGTTGCTCTTTATTCGTAACATGGTACCAAAGCTCTAGGTTTAGATCCATTCACCGCTCTTCCCT
CTCCTGCGCTGCCCCATCTCTGCTGCTCTGGGGGGTGGCCTCGTTGATCTTTTTTTTTATTCATACCATTATAACTTTCGAAGTT
TGGAGAAACATCATTACTGTTCACCTATTTTTAAGCGTACCATTACAATTATTCGCTGCCCACAAGAATGCCACAGAATACGTA
TAGGGACGACTTGGGCCCAGCTGCAGACGTATACGACAACGTGTATTCTCGTATGGACTTAAATGCCCTTGGCTCCCTATCCA
CAGCAGCCCCTCTCATGTACACAGTAGACATCCAGTTTTTTACCCCATATGACTAGCATCTCAGGACACTGGCATTTGAGTGC
CTTGCAAATTAAGGTGTAAAGCTTTAATGCATCTCAAGAATTCAAGTTTGCTTCATATTTATTCCTCCTCTCTTTTTTCCTGTA
>SsLRR-RLK174
ATTGTGTTGGGAAATGGTACTTCTCAAAATATTAGTTCTGGAATTGTTACCGGTATGTCTTACCTCGATCACATTCTCCAGTTA
GTTAGCTACCTGATCTATCTCTATCGAGGCACTATTTAATTATTTATATATAAAGTATTTTTTGGAACTTTATAAAATATCATGGCT
CTGCAAAAACCATGAGGTGTTTGGATGTGAATTTTGATTAGTTTTGTAAACCATATCATTCATAAAACTAGTCTTTTGGAGTTT
TATACCCGCCCCCCCCCCCCCCCACCCCCAAAAAAAAATGTAGTTTTGCATGTCTCTGGGTAAAACTACTCTCTTTTTCATCC
ACTGACCCTCTGATTCAGAATCAAGGAAAAAGTGTTGCCAAGGTCCCAGTGGAACTAGAAATTGTCGTGCACAATCAGCCT
TCTTTGTTTGACAAGTTCTGTCCATCAAATTGAACTCGGTCTAGGAATGAGATGGACTCGGCCATACTTCCCGCCGCACAGA
ATTCACGTGTTACCAGGAATCCAGGATAGACTGAGTAGAGCAGTCAAATTAAACTTGTTCCAAAATCATTGAAGTTGTTGAG
ATGAGAAGTAAACGGCATCTGGCTTCCCATTTGACCGTTCTTCCGCCCAAGAACCGCATTTCATCGTCCACAAAAGTAATAA
GGGGAGGTACTGCCAAACTCCGTTCAGCGTTCACCAACGGGCGCAGTGGCGATCAAGAATAATACAAAAATTGCAATCACG
CACTGAAGGAACAGGAGAGAGAAGACGGACAATTTCTCTCCAGAAATCTTTCATCGGCCAGTGCAAGCATCAGCCTGCCGG
CCACAATTTGGCTCCGTGCAGAAAGAACCTTCTGTGTGCAAGCCGTCCTTTATCTGCATTTTCTGGAACATCATGCACCAAA
GGCTTATCTGCCTGGCAAGCTTTCGGCAATCCCGACAAAGGAGCCAGTTGAGCAAGTGCATCCAAAACCTGCCCTTCCGGA
CCCTTCCAAATGTGTTTAAATAAGCACGTTGTGTTAGAGCCAGGTTTAAACCGAAGTGTTTGCAAACTTCCAAGGATTCCAG
GAGAGCGGTGCAATCGTCTGCACATTCTTTCTGACATTCCTTTGAACCTTTTAGTGTCTGTGCAAGTGAGAGAAACCCTGCC
TCATCATCATTTATTTTTCAAAAGAAGGGCAAAAGCATTGTCGCGCATTATTAAAAGAAATAGAGTTGATCCCGTTTCTATGG
CAAAACCGGGTCTAAAAACCAAAAACTGCTCGGCTAAAAAGGTCGGCATGAATAATCAGGTATCCCAAGGACAAACCAAC
AATGGGTACATTCGAGGGGACTCAAAGCATACTTACGTAATATCTTTGTCATGATGCCAAATCCACATCATCAGAGCTAGCAG
CCAGGCCCAGGTCCAATGCACATCTCCTTTGGCTCTTACCAGCACAACGGAAAGAATACAGCAGTTGCACAGCATAACACG
TTTCTGAAGTGTGGGCCTATGTCACAAGAGTCCAACCTGTGGCCGTATTAATCTAGTTAATTAATAACCATGACCGGGTCAAA
GCTTAGAAAGGAGCAATGTTTAAGGTTTTACCCCGATGAGGACAAGAAGCAACTGCCCCACTACCAACTAGGAATACAAAA
ACATTTTTCTTCAACCATTTCATGTTTTGATCAGTTCAACTGAGCTCTTTGTAGCCTTGTTCAAGGCCTCCCATACCCTTGCAT
ACGTGCGTTCTTATGAGCATATCCATTGCTTGGACCCCGTAACCGGCCCATACAATAATTGGCGAGAGATTCTTCTGAATCTG
GTGATGCATAATTGTTTTGCTTGAAACGCTATTTGCTTTGCCTGGTTCCAACCTCAGGAAGAAGAGGACGCCATTTGGTAGCA
GCTGACTTGTCCATTAGCTTGCATAGACTTCTCCAAAGATAGCCATCAGAGTTAGCTGTGGTCTGCTGCAATTTGTGTGGAGG
CCTAGCCCTGAAGGGCACGGCA
>SsLRR-RLK386
NNNNNNNNNNNNNNNNACCGTAAAAGAGTTATGCAACATAGGTTCCAATCAACTAACCTAGCAAGCTAAACTAGGAAATAT
AAGCGCACAACCTAGGAAAGCAATAAAGTGGGAAACATAAATGAGATAGAGAATGCAAACTCTTATTAACGGCCGATTTTTC
TAGAGGTATCGAGAAGCTCACACTCCCCCTAGTCTTAGTTGGAGCCCCTCGCTAGGGCCAAACTCCAAGTGGGGTAACTCCG
TAGATAGCCCTCGGGCCTTCCCCATGTGCAAGTGGGGCTTTGAGATGGCTTCTTCCGGACCGCTCCCTGCCATTTTCACTATT
GAACTTTTGGCTCAAACATCGCGGGTTGCGTTCCCTTCGGTACATGCTAGTCGGCCATACTATAAACACAGTTGGTGTGTTCG
TGTAAGATTACAAGACCCTAGTGTATAATAATAATGCGCACAAGAACCGAGTTGGCAAGAGGTATGCAAAAATTACTAAACA
CTAAGCCTAAGCCTAGAGCAAAGCGCCAAAGTGGCGGTCTAACTAGCCTAAAAACTTCTCAAAGCACTACACTAATCACCA
AATATTTTTCTTAAGAATTTTTGGTTGCTAGAGCACTTGGATATGAGTTGCAACTCTTCTACAACTTACACTCTAACACACTTG
AAATGGCTGTCCAAGAGGGTATTTATAGCATCCACTCCTCAATATAGTCATTGGCTTTGAACTGCCCACAAACAGCACTCACC
GGAAGGTCTGGTGAGGTCACTGGAGAAATCCGATGCCTGCCATGTGAACTAGCTATTGCCGACCATTGGAGCTCCTTTCCCT
GTTGTATCTTCTCGGGTGATCACTGAAGAATTCCAGTGGCTCAAAAACCCATCTTCATAGCCTCTAGACATCAACCTTTTCTC
TAGTGCTTGTTTTCATGCATCACCCAAGAAATCCGGTGGGTGATGAAACCTTCTGCAGAAACTATTGCCCGTTAGTTGAACC
GAGCCTAAGCTCCGGTGCCTTCAATTCGACACCACCGAAGAATTCCAGCTGGTGCTCTCTGTACAACATTGTTTTGTCTCGTT
TAAAAATTAATAAATTGTTTTATGTTTTGTGACACTGTTGGCTGATTTTTTGTGAGAGAAAAATACTGTTCCGGCTGGAATTAT
AAGCCGGCTGAAAAACACTGTTGGCTGATTTTTTGTGAGAGAAAAATACTGTTCCGGCTGAAAAAATAAGCCGAATAAGAC
GGATTATAAGCCAGATGAACAGGCCTAAAACTCTGCCATAGATTGGTGCTCTCTGTACAACATTGTTTTGTCTCGTTTAAAAA

TTAATAAATTGTTTTATGTTTTGTGACACAAAAACGCTGCCATAGATTGATGGCATTTGTAAAAATAACTGCTAAATCTCAAAT
GAGGGGTGAACCAACTGTGATAAATGCTTTCCAACCAGCTGTGAAAATATTTACGTAGTAGTGCATTACCATAAATAACTGCT
AAATCTCAAATATGTTTTGTCTAAAAAAAAATCCATCAGAAAATAACTCGGGTCCAACAACGGCAGGGCTGCTTCATGCCCCC
GGCGCTTAGCGGCGACGCCGACTTGACCAGTTCACGACATCTCTGCAGCCTCAGGGCCTCACGCGCGGCGCGCTAACCCTC
GCCTGCGGTGCAGCCCTAGCCAGCCACCGGCACCCAAGCCAAAGGCAGGAGCTGGCTGTGCGCTACAGGACAAGCAGGGA
CCCGCCCGTCCGCCCGCGTTCGCGGCATGCCAAGCATCATAGCGGTGGCGTCACCGCGTCTCCAATGCCTTTCCCCCACCGA
GAGAGCGAGGAGAGACTGAGAGAGTCCGTGCCGGTCGGCGTTCCCCCGTTTTGTGCTCATTGCTGCTGCCTCTCGCTCTATC
CTCTCAGTCTCTCACTCGCCTCGCCTCACTGACCGCGGCTGCTCCAGCACCAGCAGTTAGCGTTCCCAGTATCCCACTCATC
GCGCCATCGCCACCTAGAGCGCCAT
>SsLRR-RLK323
CCAATCTTTAACCTCCTTTGCTTTCTCTCGCTTCTTTTCCCCTTTTCTTTCTCTCTCCTCTTTCCCTCACGCCTCGTTTAGTTCC
CCGAATCGGGGGTGGCAAACTTTGCACAGTGCCACCCCCGAACACTGTAGCACTTTTGTTTTTTTGCGGTAATAATTGTCCA
AATCTTGATTAATTAGGTTTAAAATGTTCGTCTCGCAGAGTACAACAAAACTATGCCATTAGTTTTTGATTTCATCTACATTTAG
TACTCCATGCATATGCCGACATTTTGATGTGATGGGAAATCTTCTTTTTGCATAGTGAAATTTGGGAGTGAAATCTGGAATCTA
AACAAGGCCTCAATCTCTTTTTTCCCCTCGTCATCTTTTGTTGGCTTTGTCAGGAGGACCCAGAAAGCGACCGTCGCGTACT
ACTCCATCGTGTGCTGAAACAGAGGAGGCGTTCTGGTCTCTGATCAACGAAATGCGTTCCCATGGCGCCGGCGGCCGAGGA
TGCTAATAATTACCATGTCTGCCGTCTGCGAGCGGCACGATGCACTGCGCTGCGCGGAGCATTCCGTCGTTCGTTTTTACCGA
GCAGCAGTGCAGGACGCGGCTGCCAGAGGCAGTCAGGTTCACCAGGGCGGTCTGTCTTTTGACCCGCTCCTCCTTCACGCC
ACGCGACCGAGGGAACCACCTATCGCTTGCATGCGGGCCCAGGATATCTTTGCAGGCCCACATGACAGTGGCCGATGCCTCC
TCTTGCCGCCTGCTCGGGCCTCGGGGGGACCCACCGACTTGGTTCTCTGACGCCCGGCGCGAAGCAATCAACACCATGCTG
ACTCCGAATTCCGTTGTCCCCTCTCCCCTCCGATAAGGAGGGCAAGTTCCAGCAGCAGACTTGCAGTTGGCCTCCCTCTCTA
CCAGAACTACCACAACAAGGAAGAAATCAGCGCATCATTCCTTGGGAGCAGGGAGCAAAATGGCGTAGTAGTTGTCGTCTT
GTGGGAATCACGGCAGTTTCTTGCGGGTTTTGATTGGACGGTGCCTGAAACTTCTTCACTGACAGCTTCTGAATAACAACTC
GCTGATGACATGATAGTTCTTGGGTTCCACTGATGTCTTCTACGGCCACCTAGCGTGGTTGCTCCCAGCTAAGATCCAAATCC
TTCAAATCAGTAGTCGACTCTTGGCGTTTTTCTCACTGGACTCCAAGGATCGCATTACTAATAGGAATTACGTAGCAGCAGCT
TGGTTTGTTTAGTTGGACTTGGAGATCAGACTGTACTTGACACTTTGACAGAGAAGGATATTCTCTGCACGGCTCCCGGAGA
CCAGCTTGGCAGCTACAGTCGATATTATATATATAAACCGACAATCGGATCTAAGTTTTTCTGTTATTTGGAGATTAATTAGCAG
TACGGGGCACCAGCTTGACTGAACCTGAACGGATATGCCACTTTGACAGCGATCAGCATGGGCTAAAACTGCCATCATCAGC
ACCACCAGGCCCTTCAGAGTTGCGCTACTTCCCAGTCCCAGCTCCATGCAGCACCAATAAAAGAAAGCCCGGCATTGCTCA
AAATCGCGCACCCACTGCACGCCGGGTCCCGCGGACTCATCAACGGCCCCCCGCCCCTGCCCCAGCCCCGGCCTCGGGCCC
CACCCGTCAGGTTCCATGCCATCCATGATCATTTCTCTCTCACTGTCACTGTCACCCAAAGATGCCATGGCAATGGCTGAGTG
GGCATGCAGACAGCAGACCAGTAGACCACCGCACCGGGGCATCGGCTCGGCATCCATCGCATCGCCCCCATTTCCTTCCTGC
CGTCTTGCTTTGCATGACACGTGAAAAAGCAAAGCCAAAGGCACTCGGTTCCGCTCCACTCCACTCTACTTTGCGCTGCCGC
CCCTGCCCGCCCAGCGGCCAGCTCTCACTTTTCTTTCTCCGCACCGGGCGGCGCCTCTCGCACCCATCATTCCGACGTTCCC
CTTCCCCGAGCCAGCTCTTCCGA
>SsLRR-RLK299
CGGATTGACACGCCCAAGTACGTGCCAAACGACACCACTGCTAAAGTGGAAACCACAACCGCACAAGTGTAGCCTCTCAA
AACTCATCCACGGATTGTTTCAAAAAAAAAAAATCATCCACGGACCACGGTGTCAGTGTCAGGATGCCGTCCACAGAAGCTG
AGAAGCATAAAACCAGTCTCAATGGGGTTTCACGTCCCAGTTTTCAACACATCTTATTTTGAAAACAGTGCAAAAGAGTTTC
ATCCATATGAAACTCTCTCTGCTCCCATAAAACTCTTATCATCTCTCTCTTTATTGATACGGTGCCACATCAGCATATTTAATGC
CCATAAAATTCTGATAAAATTTCATTGGGACTGGCCTAAGTGAAGAAGGCCGAGAATCGAGCAACCGCAAATTCGAAGGATT
GCTGGGTAGGCATGGCATCCAAAGTGACAGGATGGGTCCTGAATTCTGATGCTCGGTCAAGTAGCAAGTAACAAATGGCGA
AATGGGAGGTCCACAAGGGCAAAGAACAAAGGGAGAGGCCACAGGCCAGTCAAACCCACACATTTTTGACGAATCAGAC
GGGAGCTAACATAAACTAAGCAAAAAAAAAAAAGCCATAAAAAGATCAGTGCAAAAGACAGACAGGAGTCCCAAGTGGCG
AGTCTACTCTATCCTACCGGGAGATTAAATTAGTACTGATACTATTTCTTTTTATAATAGTTAAAGCCCTAGCAGGCTCCTTGAG
GGACTTCAGCTTTAACTAATTAACCTCATATATGTGAAGTCCGACCAAACTCTAATTCAAAATACAGTTTCATGAGACAAACT
AAAGAAAAGGAGAGAAATTGTTTTCTTGGTACAAAAGGTAAAACTATCTAAATGTCTGGCTAAAACAGCTATAAAGAATGTC
CTATTTGGCAAGGATTTTCGTGAGGCTCTGCCAAACGTCACCTAAAAAATGGCTCCACGAGAAAAGTTCTAAATGGGAGGA
CAGCTGCTTTTCCTACTACAGAGGTGAAGCCATCTAAGGGCATTTCGCAAAGCTTCGGGTCGGTTTCTTTTGTGGAGCAATTT
CTCTAGTAGAGCTGAAACCGTTTAGAAAACATTTGACAAAAATGTTTTTAGTGGTAGCTTTGTTGTAATTTATTCTAAGTTTGT
GAAGAGGATTCGAGAGAAGCCAAGTTTTCGCAGCTCTACTAGCCCAACATGAAAGTTGCTCTGGCTCGGCTGGGCTTCTTC
GTGGAAGCCATATCTTGATGGTCATTTGCAGGACTTCGACTTGAGCCGGAGCCACTCAAAAAGCACTGCCAAACGAGTCCT
ATATACTGCTTCTCTAGCCTCTTCCACTTTGTTTCATCTATGATGTGTCCAGTTAACCTCCAAACCAAGAAGATTGGAGGAGAT
CGATGAGGATTAATGGGAATTTTGACTTCTAAGGATTTAAGCCACCTCAACCTCCTTCAATTCTCTTGATGTGAGGATTAACC
AAACAAGCCCTAAGGGCAGTCCCAATACTAGTAATTTCTATACATATTAATTTCTATAGATATTACATATACCATGGTTCCAATG
GATAGTTTATACAGAATAATTTTTTATTCCTTCACATACATTCTCTCTATTCTCTACCAATCACATTTCTTTATGTCCTGATTCTTG
TGTAGACATGGTTTCTAACTTAGACCCGGTTTCTATGATTTTTGCTCTTTCTTCAATAACTACCTCGCCACATCAGCAAAACGT
TTAGGTGATAGTACAATTAATGTCCATAGAAATTATGGTGATTTCTGTATTGAGCGTGGCCTAAGTGAAATACGTATTTCCAAA
GAAACCATGTCAAACTAGGACAAAACCTTGGACCTAGGCTACTCCAGGACTGCCACGCGATAATTAATGAGTCTTAAGAAA
AGAGAGATGCTGATGCCTCCCAAATCATGCTCACTCCTGCTTTGATTATGTTAGTGCGCGCAATAACTACTCCGCCACCTTCC
TCCGTCCTCCTCCGATCCCC
>SsLRR-RLK265
ACAAGCACTTCGGTGCAAGATAATACGAGACTCCCCTCCCCCATTGGACGTAGGGCATTCTTTGGCCCGAACCAGTATAAAT
CGTTTGTGTTCTTCTTGCACACCATCCGGATCTCCGACGCGCAGACACATATTACTCGTTGGTGCTAGAACCGCCGTTTCTTA
CACCGACACATCTAATTTAGAACGGAGCAAGTATTTTTTTCCTGTGAAACAAGCCACAAGAAAAGAAACAAGGAGGGTGAA
ATGTGAAGAAAATTAAATCATTTTGGAATGAAGACTAAGGTCTTGTTTGGATCCAGCAGCTGCTAATAGCTCATACCTCGTAG
CTAATATTAGCTAGCTAACTATTAGTTGTTATTAGCTAATTTACTCCAACTAGTGAGATACTTGATGCTCATAATTAGTAGCTGTT
AGTTAGTACTTCAGCTTAATTAGCTAGACTATTAGTGGGACCTGTTTAGATCGTATTGATTTGTAATGCATTTCTTTCTGTATCC
ATGCTCTTTTTGGGAATTGCAAGCGGTTACAGCTGGTACTTGATGCTCAATGTGGTGAAACAACTTTTTGAGAGGCGAATGT
AGAAAGGAGAGATAAAGTAAAGAATGCCATTCTCAATAGAGTACCAGGGAACCTTGTGTCTGAAGGCTATATCACACCGCAT
CCCATTTGGCAGCCACAGGAATCATGATTAGTCTTGTGTTGATTTGCTTGGAAGCAGCACCATCTCCCTTTTCGGGCCACCTT
TAACTTAAACAATCTCCACTGGCCTGCATGTGCATATTCGTGTGGGAAAAAAATTCTCTCTCCTCTAGTAGGCTCCAAGAAAT
TCACAGATGCTAAATGAAACTGAACACCGGAAAGTGAGGAAACGCCTCACATTTCTCCAAAAAAAAAAACACAGCAGGCAT
TTTCTTTTTCTTTTAGAAGTTCCGCACCTTGTTTGATGTTGTCGGATTCACCTCAATCCACATATGTTGGGGTGAATTGAGACG
GAATTTAGTTCAAGTTCCACTCCAATCCACCCCAACACATATGGATTAATGCAAATCCGACTATATCCAAATAAGGTCTAAGA
AAAGAACTAGGAAGCATGTGTTCTGAATCAAAGCTTAAGAACATGATTGTGACTTTTGAGTTACCTCATTTCTTCTGATCAAC
TAGTGTACCATTTATTGCTTTTCCTTGCAGGCTAACATTTGGCTAGGTGACGGCATGATGATGGCGCCCTGTCAAACTAAGTA
GTACGTGACAACGGTGACGTGTACCCCCATCTCTGATCAGTGATTACTCTAAATTTCCTTTTTTAAAACGAAGGTGATTACTC
TAGAATTGTCGTAAGTTTCTGCAATATAGTGGACTAGTGGGTGCAACAACTCTTCTCAGGTTATTGCGTCATCCTTCCCTAATA
AAAATGTCAAAGATATCAGGTCGCAAGCAAGTGCGAGGAAACGACCACGGCGCAAAGAAACACCCGGTTCGGCCGTGACA

CGGAACGAAAGTGTATTCTATACCCCTGATCCCAGGAAGATTTGAAGCGTCACCTAACCGGCCAGTCGCTCTCAGTGTGCCT
GCGTGAAGGAGCGTGTTGGAACCGGACCTCCAAGCTGAGAAGACCTGAATTGGTCCGGAAGATTTCAAACTCCGATGGAG
GGATGACCGGATGAACACCCGTGCTCTGATAAGGGGATAAGCTCAACTGTCAGCTTCAGGTAAAGATGATTTGCTACCTGAT
CTGGTACTCTGGAGCTTGCTGTGTTTGAAGCGAGAGTAGCATGTCACGTGCGGCCGTGCGGGCCATGGGGCGAGTGTGTGC
CGTGTCCGGAAGAGTAGGTAGGCGGCACGTGCCCCAGGTGTACAAACTCGGCTCCGGTACGTGTACATACAGCGGCTGTAG
AGTGAGATCCGAAAAGGGGCGAAGAGTAAAGTGGAGCTGATGCACGGCAGGATTAATACCTGGGGGCGGGTTTGGTTGGG
GCTTTGTCCTCGTACGATTAGACT
>SsLRR-RLK365
CCTCATGAGCCGCCATGGTGAGCAGCATCTAAATGGACTTCATTCGAGCAACAAGTGTGGAGACCTCATCGTAGTCGATCCC
CTACCGTTGGACATAGCCCTTCGCCACAAGCTGAGCTTTGTGCTTAACAATGGGTCCCTTATTCGTCATGCTTCAGCTTGAAG
ACCCCCTTCAGGCCAATTGCATGATGGCCTGGAGGTAGGTCGACAAGCATCCACATGTTGTGTTCTGTTCGATCGACGTCATC
TCCTCCATCATCGCCTTGCACATGGCAGGTTCATCACCCCACGACAACACCATTGGTCTCGAGACATTCTCTAGAGTGTTCAA
AACGAGAGCAAAGCCTTGCCATCATGCTCGTCGTCGAGGTCCAGATCACCCAATGGGGCGACGCGAACTCCGTAGCTGGTG
CAGGCATAGGGGTTGCCGGTGGCATGTTAAGATTGTTGATGGTCATTAACTCATCATTTTAGGCCATCAACTCGTGCTTAATTT
GAGGATATGAATGATATCCACCATAGGCATGGGGCATATTTCTAATCAACTCCATAAGTTTTGGTAACTATGTGAGTGTAGGAA
CAAATAAGTGAAAGATGAGCCAGTTGGGAGGTTGGGTGACCTAGCATTAGGTTGGTTGACCCAACCCTAGCCTATCCAACCC
CACTATGTCACCACATCGAAGGAAACATACCTAGATGTCATCACACATGTTGGTTCAACATCGGTTTACATCAAAGGGATGAA
ATGAAGCCAAGTGGAGACAGTGGCGGAACCAGAACTTTTGATCAAGGGGGTCAGACAATACGATGACATCTTGTAAACATA
ATAAATATATACAAAAAAATATACGTCTAATAGCTTTTTAGCGTTTTTACATATATATATTTAGTACTTGTAACAAAAAAACACA
ATATTTATCATAAAAAAGAGTGAAACAATAACTTTTTACTATGCAATTAAATCTTTTATATGTATTTAAAAAAATCATTGCGCTT
GGGGGGGGGGCACGGCCCTTGCTGCCCCCTGGTTGCGCCAGTGAATGGAGAGCACAAGGATCCATAGACCCATCCAATAGA
ACAAACACACTTCAACTCACCACATTTCATATGGGTTATAGATCAACTCGCCAACCAAAGATAAATGACAAAGAGGTTGGAT
CACTTCGGGCGATCCCATATAGGATCGGCTGACCCGTCTTAATTTTACCTAAATTTATTTTAAAAAACAGCATCAACATTTATG
ATTTCAAATGGGTATGCTATTAAAATATCTTTCATGACAAATATAATGAAACATGTTCACTATGATAAGTATTACCTGCTTTTTAC
AAATTTGGTCAAAACTTAAGGGAGTTTGACTCAACACTATTCTATAATTATTTTCTTTCTGGGATTAAGGTTATAGTTCACTTT
GGCTCTGTCTTAACTCTAACTTTAAGCAAGTTTTTTAAAAATGCACCGATGTCTAAAATATCAAGTTAGTTTCATTAAATTCTC
CATTGAATATGTTATCATATTGTATTTATTTGATTTTATAGATGTTAATATATTTTTCTAAGAACCTAATCAAAATTACATAAGCTT
TATTTAGGACAAAGTTATTAAATTAAACTATAATTTAAAATGAAGAGAGTAATTTATAATTCGTGTACCTAAACGGATGGGTTT
GCCTAGGAATATGTAGATAGTAGTGGTTTGGGCATGATAACAACTTTGAAAAAGAATCCAACTTTTCAGAAAATACCCATGCA
CACAAAAAAAAAATGAGAGGAATCACACCCGAGGAATACGTCGCTTCCCGCGCGCAACATCCCCCAGTCGCTATTCAACAAC
CATCGACCAATATCTCGAGCAAGTACAATTTGGAAGAACGAACTGATCAATCCAACTATAAACAAGTGGGAATACGATATATA
TTCTGGACTTGGCCACTCCAATTGTATAATGCATATACAGTAGCTTTGATTCTTTTGAGCGAAAGGAATTGATCAACTGAAGG
CT
>SsLRR-RLK153
TTCCATGAGGTTTTCCCTCTGTATCTTTATACTGGAACCTCAGATCAAGCAGGTGATGAGTACTCATTGGATTCACTTGTCAAA
TTTGGTATCAATGAGAAGAAGACCTCTGGTGGGAAATTGGGCTTCTTGTACAGCTCCCTGCGGAATGCACAATCAGCGCAAG
GCACTATGAGGGTGAAGAAGAATTTGGTGGTCAGTGGATTGGGGAAGACCCATCAGGTGTATAAGTATGTGGGAACTGATG
GATGCTACTTCAGGGATGTGAGCAGCAAGAACTACACTGTACTTTTTGACCGCTCTGATGATTCTTGTTCAAAAGGAGCATAT
AGAGGTGCCAGCGCGAAGTTGAATGATCAGCCAGCAAGAAGAAGGTTGCTGGCGAACAAATTGTAAAGCACGGAAAAGAT
CTAAGTCGTCCACGTTTCAGTTTTTTTACTTTGCTTCTTCAATAAAGATTGCTTTGGTAGTTCATGCTTCAGAAAGTAGGTAAG
AAACTTGGATGTGCCTTTATAAACACGCCAATTCAGATCCAGTGGCATGCTATTGTCATCAGTAACTATCAATTGCATCATCAT
GTCTACCATTTAGCTGGCATGACAGGACGAAGGATGATTTAAACCATTATGCTGGCAGTCAATACTAGCACTAGTACTAATCA
AGATTCTGACACAATCACACTATTAATTTATGCCAGCATTGCTTACTCCACATTAACTGGATTTGTTCCGAATTTCCGATCTTAA
TCAAGGCATCAGCAGAGCTGCTCTTGTCTGTCCTCTTTGCAGATAGGACTAGGAGTATGTATTTCTGAAAAGCCTTTTTTTT
GTCTTTCGAAAAGCTCTTTGCAGACTTATGCTGTTTTTTTATAACATGTATGCTGGTACTTAATTTTGAAGTCAAGACATACTT
GGACCTTAGATTGCCATGTTTCGCCAACTGTGATATTTTTGGCACTGGGAAATCACTGATATCCTTCCAATAAGTGTAGCAAAT
AACGACATCACGCCATGAGTAAAGTTTGGGACCTCGACATGGCAAAGAGATTACAAGAGGTAAACCTGGTATTGTCTGAGA
TGGTGTGCTACAATTTTGCCCATGTTTGTCTCATCTTTACATTTTTAAGCTTCTTTTTTTAATCATTCACCTGATCATCACAGAA
CCAACTCTGTAATTCATGGAAATTATCATAAATTAAAATAGCTAAGTATCACCATCACATGCAGCGTTTTCCCCAAATGCCTGA
ATCCTTTTCTTCAGATGAACTGAATCATTTCATTTCAATGACCTACCTCGAAATTCATAGCGGAAGGAATGGTGGGGGTGGGT
GAAGGGATGGATCATGGATGAACGTCGAACAGAGCAATGGACGGTTGCGATGGGTATCGTGCGAGCATAGATGATGGGGGT
GGTCATGCCATGCCTTTCCCCACGAGGGCCCAACGAGGCAGTGGCTTGGCGCGCGCGGCGGCGACGGGGACGCTCCCTCG
CTTCCCTGTCCCTGGCCACCTCTGAACCGAATCTAACCGCCGCCCGCCACCTTCGCGCGCGAAAACTCCCCCTCCCCTTCGC
GCCGCCGCCGCCGACCACCACCCATCCCGGCGACAAGGGCTCTATCCGGCTGCTCCGCCAGGAACCCAATTTTCTCGGCGC
GAGAAACGCCCGGGGGAAAAGGCCAGGACCCCATTCCGGTGCGGCGGGACCAATGGGCTCCGGCGAATAGCTCGAGCACC
TCGAGCTCGCCGGAACAACCGAGGCGGCACAGCGTCTGTTCTCCCCGGCGTGATGGCGATGCAGGGGATAGATAATCGACC
CCCAAACTGAGCCAACATTTCCGTGCCCGGCCGTTGAACGCTGCGGTGGTCCCGTCTTGTTCGTTCGTTCGTTCTCCAGAAT
ACTGGGAACCTGAGAGGCAGAGGCTCGGCCAGCGACGGTGGGTAGCGAACTGGCGCCTGGTTCCCGAGTTGCTAATCTCC
GGGAGGAGGCGCTCCCGTTGCCA
>SsLRR-RLK194
TTTCCCTCTGTATCTTTATACTGGAACCTCAGATCAAGCAGGTGATGAGTACTCATTGGATTCACTTGTCAAATTTGGTATCAA
TGAGAAGAAGACCTCTGGTGGGAAATTGGGCTTCTTGTACAGCTCCCTGCGGAATGCACAATCAGCGCAAGGCACTATGAG
GGTGAAGAAGAATTTGGTGGTCAGTGGATTGGGGAAGACCCATCAGGTGTATAAGTATGTGGGAACTGATGGATGCTACTTC
AGGGATGTGAGCAGCAAGAACTACACTGTACTTTTTGACCGCTCTGATGATTCTTGTTCAAAAGGAGCATATAGAGGTGCCA
GCGCGAAGTTGAATGATCAGCCAGCAAGAAGAAGTTGCTGGCGAACAAATTGTAAAGCACGGAAAAGATCTAAATTCGTCC
ACGTTTCAGTTTTTTTACTTTGCTTCTTCGATAAAGATTGCTTTGGTAGCTCATGCTTCAGAAAGTAGGTAAGAAACTTGGAT
GTGCCTTTATAAACACGCCAATTCAGATCCAGTGGCATGCTATTGTCATCAGTAACTATCAATTGCATCATCATGTCTACCATTT
AGCTGGCATGACAGGACGAAGGATGATTTAAACCATTATACTGGCAGTCAATACTAGCACTAGTACTAATCAAGATTCTGACA
CAATCACACTATTAATTTATGCCAGCATTGCTTACTCCACATTAACTGGATTTGTTCCGAATTTCCGATCTTAATCAAGGCATCA
GCAGAGCTGCTCTTGTCTGTCCTCTTTGCAGATAGGAGTATGTATTTCTGAAAAGCCTTTTTTTTTTGTCTTTCGAAAAGCTC
TTTGCAGACTTACGCTGTTTTTTTTTATAACATGTATGCTGGTACTTAATTTTGAAGTCAAGACATACTTGGACCTTAGATTGC
CATGTTTCGCCAACTGTGATCTTTTTGGCACTGGGAAATCACTGATATCCCTCCAATAAGTGTAGCAAATAACGACATCACGC
CATGAGTAAAGTTTGGGACCTCGACATGGCAAAGAGATTACAAGAGGTAGTAAACCTGGTATTGTCTGAGATGGTGTGCTAG
AATTTTGCCCATGTTTGTCTTATCTTTACATTTTTAAGCTTCTTTTTTTAATCATTCACCTGATCATCACAGAACCAACTCTGTA
ATTCATGGAAATTATCATAAATTAAAATAGCTAAGTATCACCATCACATGCAGCGTTTTCCCCAAATGCCTGAATCCTTTTCTTC
AGATGAACTGAATCATTTCATTTCAATGACCTACCTCGAAATTCATAGCGGAAGGAATGGTGGGGGTGGGTGAAGAGAAATA
AGGGATGGATCATGGATGAACGTCGAACAGAGCAATGGACGGTTGTGATGGGTATCGTGCGAGCATAGATGATGGGGGTGG
GGTGGTCATGCCATGCCTTTCCCCACGAGGGCCCAACGAGGCAGTGGCTTGGCGCGCGCGGCGGCGACGGGGACGCTCCC
TCGCTTCCCTGTCCCTGGCCACCTCTGAACCGAATCTAACCGCCGCCCGCCACCTTCGCGCGCGAAAACTCCCCCTCCCCTT
CGCGCCGCCGCCGCCGACCACCACCCATCCCGGCGACAAGGGCTCCATTCGGCTGCTCCGCCAGGAACCCAATTTTCTCGG

CGCGAGAAACGCCGGGGGGAAAAGGCCAGGACCCCATTCCGGTGCGGCGGGACCAATGGGCTCCGGCGAATAGCTCGAGC
ACCTCGAGCTCGCCGGAACAACCGAGGCGGCACAGCGTCTGTTCTCCCCGGCGTGATGGCGATGCAGGGGATAGATAATCG
ACCCCCAAACTGAGCCAACATTTCCGTGCCCGGCCGTTGAACGCTGCGGTGGTCCCGTCTTGTTCGTTCGTTCTCCAGAATA
CTGGGAACCTGAGAGGCAGAGGCTCGGCCAGCGACGGTGGGTAGCGAACTGGCGCCTGGTTCCCGAGTTGCTAATCTCCG
GGAGGAGGCGCTCCCGTTGCCA

>SsLRR-RLK181

CGAGGAATTCCATGAGGTTTTCCCTCTGTATCTTTATACTGGAACCTCAGATCAAGCAGGTGATGAGTACTCATTGGATTCAC
TTGTCAAATTTGGTATCAATGAGAAGAAGACCTCTGGTGGGAAATTGGGCTTCTTGTACAGCTCCCTGCGGAATGCACAATC
AGCGCAAGGCACTATGAGGGTGAAGAAGAATTTGGTGGTCAGTGGATTGGGGAAGACCCATCAGGTGTATAAGTATGTGGG
AACTGATGGATGCTACTTCAGGGATGTGAGCAGCAAGAACTACACTGTACTTTTTGACCGCTCTGATGATTCTTGTTCAAAA
GGAGCATATAGAGGTGCCAGCGCGAAGTTGAATGATCAGCCAGCAAGAAGAAGGTTGCTGGCGAACAAATTGTAAAGCAC
GGAAAAGATCTAAATTCGTCCACGTTTCAGTTTTTTTACTTTGCTTCTTCAATAAAGATTGCTTTGGTAGCTCATGCTTCAGAA
AGTAGGTAAGAAACTCTGATGTGCCTTTATAAACACGCCAATTCAGATCCAGTGGCATGCTATTGTCATCAGTAACTATCAAT
TGCATCATCATGTCTACCATTTAGCTGGCATGACAGGACGAAGGATGATTTAAACCATTATGCTGGCAGTCAATACTAGCACT
AGTACTAATCAAGATTCTGACACAATCACACTATTAATTTATGCCAGCATTGCTTACTCCACATTAACTGGATTTGTTCCGAAT
TTCCGATCTTAATCAAGGCATCAGCAGAGCTGCTCTTGTCTGTCCTCTTTGCAGATAGGAGTATGTATTTCTGAAAATCCTTT
TTTTTTGTCTTTCAAAAAGCTCTTTGCAGACTTCAGCTGTTTTTTTTATAACATGTATGCTGGTACTTAATTTTGAACTCAAGA
CATACTTGGACCTTAGATTGCCATGTTTCGCCAACTATGATCTTTTTGGCACTGGGAAATCACTGATATCCCTCCAATAAGTGT
AGCAAATAACGACATCACGCCATGAGTAAAGTTTGGGACCTCGACATGGCAAAGAGATTACAAGAGGTAAACCCGGTATTG
TCTGAGATGGTGTGCTACAATTTTGCCCATGTTTGTCTCATCTTTACATTTTTAAGCTTCTTTTTTTAATCATTCACCTGATCATC
ACAGAACCAACTCTGTAATTCATGGAAATTATCATAAATTAAAATAGCTAAGTATCACCATCACATGCAGCGTTTTCCCCAAAT
GCCTGAATCCTTTTCTTCAGATGAACTGAATCATTTCATTTCGCGAAATTCATAGCGGAAGGAATGGTGGGGGTGGGTGAAG
AGAGATAAGGGATGGATCATGGATGAACGTCGAACAGAGCAATGGACGGTTGCGATGGGTATCGTGCGAGCATAGATGATG
GGGGTGGTCATGCCATGCCTTTCCCCACGAGGGCCCAACGAGGCAGTGGCTTGGCGCGCGCGGCGGCGACGGGGACGCTC
CCTCGCTTCCCTGTCCCTGGCCACCTCTGAACCGAATCTAACCGCCGCCCGCCACCTTCGCGCGCGAAAACTCCCCCTCCCC
TTCGCGCCGCCGCCGCCGACCACCACCCATCCCGGCGACAAGGGCTCTATCCGGCTGCTCCGCCAGGAACCCAATTTCTCG
GCGCGAGAAACGCCGGGGGGAAAAGGCCAGGACCCCATTCCGGTGCGGCGGGACCAATGGGCTCCGGCGAATAGCTCGAG
CACCTCGAGCTCGCCGGAAAAAACCGAGGCAGCGTCTGTTCTCCCCTGCGTAATGGCGACGCAGCAGGGGGTAGATAATCGA
CTCCCAAGCTGAGCCAACATTTCCGTGCCCGGCCGTTGAACGCTGCGGTGGTCCCGTCTTGTTCTTTCGTTCTCCGTATCGG
GAACCCGTATTGGGGAACCTGAGAGGTTCGGCCAGCGGCGGTGGATAGCGAACTGGCGCCTGGTTGCCGCGTTGCTATTCT
CCGGGAGGAGGCGCTCCCGTTGCC

>SsLRR-RLK166

AAACTTGGATGTGCCTTTATAAACACGCCAATTCAGATCCAGTGGCATGCTATTGTCATCAGTAACTATCAATTGCATCATCAT
GTCTACCATTTAGCTGGCATGACAGGACGAAGGATGATTTAAACCATTATACTGGCAGTCAATACTAGCACTAGTACTAATCA
AGATTCTGACACAATCACACTATTAATTTATGCCAGCATTGCTTACTCCACATTAACTGGATTTGTTCCGAATTTCCGATCTTAA
TCAAGGCATCAGCAGAGCTGCTCTTGTCTGTCCTCTTTGCAGATAGGAGTATGTATTTCTGAAAAGCCTTTTTTTTTGTCTT
TCGAAAAGCTCTTTGCAGACTTATGCTGTTTTTTTTATAACATGTATGCTGGTACTTAATTTTGAAGTCAAGACATACTTGGAC
CTTAGATTGCCATGTTTCGCCAACTGTGATCTTTTTGGCACTGGGAAATCACTGATATCCCTCCAATAAGTGTAGCAAATAAC
GACATCACGCCATGAGTAAAGTTTGGGACCTCGACATGGCAAAGAGATTACAAGAGGTAAACCTGGTATTGTCTGAGATGGT
GTGCTACAATTTTGCCCATGTTTGTCTCATCTTTACATTTTTAAGCTTCTTTTTTTAATCATTCACCTGATCATCACAGAACCAA
CTCTGTAATTCATGGAAATTATCATAAATTAAAATAGCTAAGGGTGTGTTTAGTTCCCCATTTCACCCAAAAAATTTTGGGTTT
TGGTCCATCATTTTGCAAAATGGAACTAAGCACCATGCAAATTTTTTGGCAAAAAGGTATCTATTCTCCATTTTGGATTTTGGC
ATCAAGAGGCAAAAAAAAAAAAAACAAAAAAGCCAAAAAAACTCTCACACACCTCATGAAAAATTTGGTATTTTGGATGA
AACTAAACACACCCATAAAAATTTTGGCATTTTGCATTTCATCGTTCCAAAGTAGTTGGTATTTTGTATTTTGTATTCCATGAG
AAACTAAACACAGCCTAAGTATCACCATCACATGCAGCGTTTTCCCCAAATGCCTGAATCCTTTTCTTCAGATGAACTGAATC
ATTTCATTTCAATGACCTACCTCAAAATTCATAGCGGAAGGAATGGTGGGGGTGGGTGAAGAGAAATAAGTTGGTATTTTGTA
TTTTGCATTCCATGAGGAACTAAACACAGCCTAAGTATCACCATCACATGCAGCGTTTTCCCCAAATGCCTGAATCCTTTTCT
TCAGATGAACTGAATCATTTCATTTCAATGACCTACCTCGAAATTCATAGCGGAAGGAATGGTGGGGGTGGGTGAAGAGAAA
TAAGGGATGGATCATGGATGAACGTCGAACAGAGCAATGGACGGTTGTGATGGGTATCGTGCGAGCATAGATGATGGGGGTG
GTCATGCCATGCCTTTCCCCACGAGGGCCCAACGAGGCAGTGGCTTGGCGCGCGCGGCGGCGACGGGGACGCTCCCTCGCT
TCCCTGTCCCTGGCCACCTCTGAACCGAATCTAACCGCCGCCCGCCACCTTCGCGCGCGAAAACTCCCCCTCCCCTTCGCGC
CGCCGCCGCCGACCACCACCCATCCCGGCGACAAGGGCTCTATCCGGCTGCTCCGCCAGGAACCCAATTTCTCGGCGCGA
GAAACGCCCGGGGGAAAAGGCCAGGACCCCATTCCGGTGCGGCGGGACCAATGGGCTCCGGCGAATAGCTCGAGCACCTC
GAGCTCGCCGGAAAAAACCGAGGCAGCGTCTGTTCTCCCCTGCGTAATGGCGACGCAGCAGGGGGTAGATAATCGACTCCCA
AGCTGAGCCAACATTTCCGTGCCCGGCCGTTGAACGCTGCGGTGGTCCCGTCTTGTTCTTTCGTTCTCCGTATCGGGAACCC
GTATTGGGGAACCTGAGAGGTTCGGCCAGCGGCGGTGGGTAGCGAACTGGCGCCTGGTTGCCGCGTTGCTATTCTCCGGGA
GGAGGCGCTCCCGTTGC

>SsLRR-RLK148

GGTGACGATGTCGACATGGAATAGCATGGCCGGCGTTGTTGGCGCCCTCGCGGCGTTCCCAACCCGTGCTTTCGCCGGCGCC
AGGAGGATCCTAGCCGGGCTGACTTTCTCACGCAAGCGCACATGGCCGCGCCGCCGCCGCTGACCACCAGCAGCAGCAAG
GTCACGGCGGCGCGGGCGCAGAGGTGCGGGGGTGGCCAAGGTGCTGCTGTTCCTGGCGGCCGTCGCGCTCCCGGGCCTCG
TGCTGTACCACCGCGCCGTCGTGGCACCAGGGGACCTGCGCTCGATGCCACCGCCTTGCCGTGGCGGCGCGGCGCGCCGAG
CGACGACGACACTGTGGCGGACCTGGTGAGTACGTGACCTCGCTCCGCGATGCAAGAGGCATATGGCTAAATGATTGTAGTA
ATCCTCGTTGGATGCCAGCGGCTCGACAGCTTAGCTTCCGGCCATCCGCGACGGCGAGTAGCGCGGCAGCCCCAGCGATGC
ACTGTGCTTGAGATGGTGGTGCCCATCTCCTCCACAGCGGCGAGTAGCATGCGAGGACGGACGGGGTCTCGTCCGCACGGT
GGCCGTCATCCACGCTGCCCGCTCGTCCGCGCGTGGGCTGCCGTCCTCCCGCCCGCTCTCGCGTGGTCACTGGCGACGTCCC
GGCCTTGCTCTCGCCGGCGCCCTTGCCTCCCACTTCTCCCGACTTCCGAGCTCGCGGTCGCCGCTCCCCGGTGCTTGCGCTC
GCTGTTGGCGCTGTCCTCCGCGTGCGTTGTCGAGCGCCAGCTCGGAGCGTCAGACTGTAGACGGGTTTGCGAGCGTGATGG
CTGAACCAACGCATGTCAGCTTCGCCGCTATCGACGCAGAGCTCCTGCGCGTTGCTTGCTCCGTCCAGGAGGCTGGCCTGG
AACTGATGTCGATTTAACTGCAAGTAGCATGCGTTCTGCCAGAGTGCCACAATACAAACATAAGCACACATTGCAAATGTCA
CAAGCATGATGGATTAAACCAATTACTCGATTTGTATAATAGATTCAAGGTTCGATGCATCAAGTGCACAAGCAACGCGAGTG
TCTGTGTCTCCAAGTTCGTCAGCGCTCCAGGGCCTCCCGGCGTGGGTCTTCTTCTCCAAGGGCGGCTCGGCCAAGCAGCAC
AGGAGGCGGCGCCGGGCAAGCGCGGCGGCGTGCGAGCAGGCGCGGGCAAACCCCGCGGGAGGGCAGCACGGCGGCGCG
GGAGGTGGCTCGCTGGAAAGGCCCCTTCGGCATCAGAAGCTTGGAGCGTTCCCGCGACCGGCGGCTCCAGCCCGCTGCAG
CTTTGCCTGCCTCGCTAAAAAAAATTGAGATTGTGCTTCGATTGGTCGCACGCCACGGGAGCCCTTCGATCTCGCTTGGAGG
GTGGATATACGTGGGAAAGTTACGTCGTGACTTCCTTCAGTAAAGTTACTGAATCCGCGTCCACTACTACCCGTTCCACCAAC
AGCGGACGACCGCTGTTTCCCTCTCCCTCCCGTCCTCTCTCTTTCTCTCTCTTCCCCCTCGTCTCCGTTCCCCAACCCGCCGC
CGTGTAAGCACCCCTCCTCGAATCCTCTTTTCAGTTTCTTGATTCGAATCGCGTGCGGTGTAAGCAAAAATCGTCAGGCAAA
ACAAAAAGGCGCTAAAGCTGTTTGTTTGCTTTATGGTGATTGCAAGTTTCCTCTGCTCCTCGTCGCCCTTTTTCTGGCACCTC
TGAGCTGTTCCTGTCTGTTTTCTCTTTGCATTCAGGCACGAAGCGGACTTGAGGCTGCCGTGCCTGGGTGGAGCTCGCATCG

TCGCTGCAGCGGGCGCAATGCCTGCCCCCCGCGCCTGACCATGCATTCCAGGTTGCTCAAGAGGCTCGCGAAGAGGATCAT
GTGACTACGCCCTGCCGTCGTCGTCTCGATCGTCGCGCGCCTGCGGAACGGGAAAGGATTCGCAGGATGGATGGCTTCATTT
CGTCGTCGTTGGCGCTCGTCGTGCTGGCGCTTCATCTTGCCGTGG
>SsLRR-RLK163
ACATTTATGAGGTACTGCAGGACTGCAGGTCAAGGTGGTTTTTTATTATTCACATCTAAATGGTTTGTTGTTCTCATCTATCCA
AATGTTACTTGTTTTTTGTCAAGAGAATCACAGGTAAACAAAAATATTATCAATAGCACATAACACATTTATAAAATGGATCAC
AAGATTTTTTTTCAGCCTTTGTAACTTATATGGGAGTGCGATATACCAAAATAAAATCACAATACGGTGAAACAATCTCATGAT
CAATTCAAAGATAACAATGTATCACACAAAGACAAGTAGATAACACTAACACACCAGTATTGCCGTCCAAATTATGGAAAAC
ATGTTAAACACTGTCTACGTGACAATATGACATGGAGAAAACAGTAAAGCAACAGCAGGATACTATTCTAAGTGGAGATTTC
TTTCATCCGAGGACTAAGATTTGAGGAGAAATGTTCACGGACAAATAACAAGTAGTCAATTTAAATGTACATTTTCTCTTCTA
ATTTTTATAGTGTTTCTATGAAAGCTATGCTGATACATCTGAAGTGCTCATGTATTTTAATCACGGCTTCATCCTTTTCAAGGAA
ATTAAGGAAGGAGACAACACTATTGCAGATTCTACTGAAACATAAGCAAAAGACCCACAAACTAGTTCAACATAAAACATG
AAGTTTTCTTTAATGTTGAACCACCTTCCTAGTCTATCTCACTTGTTCCGTTCTCAATACCAGTGGTTGGTTTTCTTTTCTTTCT
CAATACCAGTCTGATTAGGTCGATGTTGTAGTATGAACTTTTGGGTTCTAGAAATTCAGAGCAAAATCCATTACAATGCGGCT
CTGAAACTGAGATAGCGATGATAACCAAACAGACCATGCATTCCATGGGTTCCACGGGTGGTCGTAAACCCGTGAATGACAA
GTGGCTTCTCCAATCTTAGTCCATGCTTTATTTTGCTTCGAACTATACAAAAAAATGTCTATTAACTGAAACAATCCGGTTTGC
CTTTCGTGCTTTGCGACATATCTCGATTTTTAGATTGTACCATTAATCTCACACAAGATGCGAACTAAATAATCAAAAGATTAC
ATTTGGTGCAGGACTGCAGGTCAAGGTGGTTTTTATTATTCACTCTAAATGGTTTGTTGTTCTCATCTATCCGAATGTTGCTTT
TTGTCAAGAGAATCACAGGTGAACAAAATATTGTCAATAGCACATAGCACATTTATAAAATGGATCACAAGATTTTTTTTCAG
CCTTTGTAACTTATGGGAGTGGGATGTACCAAAATGAAATCACAATACTGTGAAACAATCTCATCATCAATTCAAAGATAGCA
ATGTATCACTCAAAGACAAGTAGATAACACTAACACACCAGTATTGTCATCCAAATTACGGAAAACATATTAAACCCCTGTTT
AAATGACAATATGACATGGAGAAAACAGTAAAGCAACAGTAGAATACTACTCTTTTAAGGCCCTGTTCGTTTATGCTGAAAA
ACAAGCTGAAACGCTGTTCCGGCTGATTTTTTTGAGAGAAAAATACTGTTTTCGACTGAAAAAACAAGCTGAAAGTACGGA
TTATAAGCCGGCTGAATAGGGCCTAAGTGAGGACTTCTTTCATCCGAAGACTAAGGTTTGAGGAGGAATGTTGAAGCACAA
ATAACAAGTTGTCAATTTAAATGTACATTTTCTCTTCTACTAATTTTTATAGCTTTTCTATGAAATCTATGCTGATATATCTAAAG
TACTCGTGTCTTTTAAGCAGGCCGAGACTAAGCGTTCTCTAAACCAACTAGCCATGATTACTCTTTTACAAAAATAACCCTGG
TCCATGGTCAAAAAAGACGAATTGAATACACATAAAAAGAGGAAAAATAAATGGAGAAATTTTAAAATCTACGGCACGGCA
CGGCACGCTTGCCTTCCTCGTCTTCTCTCCATCCACTTCCACTCCACTCCCTCCGCCGCCGCCAGCCTGCCCGGCCGCCGCC
ATGGCGAC
>SsLRR-RLK178
ATTCACATCTAAATGGTTTGTTGTTCTCATATATCCAAATGTTACTTGTTTTTTGTCAAGAGAATCACAGGTAAACAAAAATAT
TATCAATAGCACATTAACACATTTATAAAATGGATCACAAGATTTTTTTTCAGCCTTTGTAACTTATATGGGAGTGCAATATACC
AAAATGAAATCATAATACGGTGAAACAATCTCATGATCAATTCAAAGATAACAATGTATCACTCAAAGACAAGTAGATAACAC
TAACACACCAGTATTGTCGTCCAAATTACGGAAAACATGTTAAACACTGTCTACGTGACAGTATGACATGGAGAAAATAGTA
AAGCAACACCAGGATACTATTCTAAGTGGGGATTTCTTTCATCTGAGGACTGAGTGTCGGTGTAAGGAACGGCGGTCCTAGC
ACCAACGAGTAATGAATGTCTGCGTGTCGGGAGTCCGGATGGTGTGCAAGAAGGACACAAGAGATTTATACTGGTTCGGGC
CGAGAAATGCCCTACGTCCAGTGGGGGAGGAGAGTTCTGTATTATCTTGTACCGAGGTGCTTGTAGTAGGGGTTACAAGCGG
ATGTGAGAGAGAGAAGAGCTCCCAACTCTCAAGGCGTGAGTGGAGAAAGTGGGTGCGTGCGACTCCGCTGGTGACCTCGC
AAGGTCTAGGAGAGTAACCTGCCCGTATCTCGCGTTGTATTGCTATGGTGGTTCAGTTGAGTTGGAATGTGTTCGACCTTTTG
GAATGATCCCTGCATGCTCCTTTTATAGTTTCAAGGAGGTGCAGGGTTATATGATGTCAGCGTATGGTGTGGCATTGCCGGTG
GAGAAGCCTGGAGGCCTCGGAAGGGCCTTATCTAGTGGCAGTGTGGTCTGCACTGTGGAGGCATGGCGTCGGTACTTCCGG
TACTGCTGCTACATATTCTGGTATGTGTGCCACTGCTGAAGGAGTGGTCTCTGTCTTTTGGCAGTGTAGCGTCGTGCCTTGTC
ATACCTGGTCTTCGTAGCCGGCGTGGCGTGTTTGTCCTTATAGTTTGACTTGACGTAGAGGGCCGAGGCCTCGGAGGCGGAA
AGTGCGGTGCGATCTTATCCTGATCGTGGGAATAGTGGCCCAGGGCAGTCGTTCCGTACACTGCGCACGCACGAATCGTGCG
TATAGAGCCAGTGTGGGTGTCATAGTGGTCAGCAGCTCCTCCCCATGCCTGTTACAGACGATGGCGCGGTGATTTGACCCCG
GTCATTCCCGGCCTGTCCGGCTGGGCAGACGCGAGAACGTGGGTGACGTCACAGGGGGTCTCGAGCGCGCCAATGGGGGA
GCGGCGTTAGAGGCCGGTCTCGGGCGAGTCGGAGACGGATAATGCTGCCTCTGGGGCTCGGGCGAGGCGGTGATGTTGCCG
GGGGTCGGCCTGTGTCCGAGACCCCAGGGCGGAGACCTCGGGCGAATCGGGTGTTCCGAGACCCCTATGAGTGGGGCCTC
GGATGAAGTGGGGAGATGGGCTGGTTCGCGTACACATCTTGCTGGAGGCCCAGCAAAGGGGAGTTTAACCCGTCTCTCTTT
TGTTGATCCTTTCTCTTAAATGGGGAGGCCGGGTGATTTTCCATCACAATTTGGGTACCCCTAATTATGGTACCCGACACTGA
GATTTGAGGAGAAATGTTCACGGACAAATAACGAGTAGTCAATTTAAATGTACATTTTCTCTTCTACTAATTTTGATAGCTTTT
CTATGAAATCTATGCTGATATATCTGAAGTACTCATGTCTTTTAAGCAGGCCGAGACTAAACGTTCTCTAAACCAACTAACCAT
GATTACTCTTTTACAAAAATAACCCTGGTCCATGGTCAAAAAAGACGAATTGAACACACATAAAAAGGGGGAAAATAATTGG
AGAAATTTTAAAATCTACGGCACGGCACGGCACGCTTGCCTTCCTCGTCTTCTCTCCATCCACTTCCACTCCACTACCTCCGC
CGCCGCCAGCCTGCCCGGCCGCCGCCA
>SsLRR-RLK102
TCAAAATATTTCGCAAAAAAAACAGTACATTTTCTGTCACATCGAATCTTTAGACATATATATATAGTATTAAATATAGATAAAA
AATAAAACTAATTACACAGTTTAGATGAAATGGACGAGACGAATCTTTTAAGTTTAATTAAGCCATGATTGGACACTAATTAC
TAAATAACAACGAAAAATGCTACGGTTATATTTCGCAAAATATTTCAGGAACTAAACGAGGCCCGAGTGTTTTACAGAGTGG
GGTTTTGCGGCCGCCCACGTTGGTTTGCTCGTATTCTCATAAACGACCCTCACCCATTGACCATGGTTCTCTTCCCTACAGCT
AAGTTCAGCTAACAAATGCAGGCGTATTAGAGTGAGTGGTAACATATTGTCACATGTATAACGTGCTAGTATCTTGGTAGCTG
CTTGCATGTGCTTGCTCGCATTTCCAAATCAAGTAATGTGCTGCTACCTTGTCTTCTCTACTAATTTTTTCTAGCAAAAAAACC
ATTCTAGCAAAGAAAAAACATAACAATAGCTGCATATGTCATTGAGGTTAATTTCACGAAAGAATTAGGAGAGTTTAGAGGC
TATTAGCTCCTTGATACACTTGGTGGTTGAGCAAAGTAGGGTCCAATTTTACACACATCTCTAGTGATTGTGCGACGAAACTA
AGGGTCTGTTTGGCATGATTTTGGCTCTGTCAGCGCCTTCTGCAGAGAAGACAGATGACTCCTATCAAACACTATAGGAGGA
GAAGTCGTTTTCTAGTGGATACCAGGCCATCAATTGGTCTGGAGGAACATTTGAGGATGAGCATTGCCAAGCGGGTCCTAAG
ATATACGTATTTTTCTTCGCGAGAGAACTAAGTACCAATACGTCATAATATAATGCATATCAACGAATAACCTCTTCTACTGCCC
CAAATTTTCCATTGTATTGATTATATTCTCTTTGATGTTATGGTTGCATAGTCATATACCATCTCTTCCTTATCTTCCAACTGACAT
TTGCATTTTTCTTATGATCACATCCCTTGTTTATATGCTGTCTTTCCTTTTGACACCTGAGATAGAATTGACCGTTCCATTTCTTT
ACACTATATTTAAGTATAATATGATCTTCTTTACTATTTGGTCCTTGCCAAATTTATCAAGCCACTTATCAATTAACACAGACGT
GATATACTTGACAACTATCTTCAATGATGCTAATTTTCTCATTTTAGAGTTTATCTATGCACACATCTTTTTTTTCTAGGCAATAC
GGGAGGTACGACGGACACCAACAAACTGGGTATTAGTTGGTTAACACTAGAGCAACTATCAACCACAGACTTTTTATGTTCC
AACTTTAAGATAACCTAATTTAGTAATTTTAGTTATTCGCCTTTATCTCAGAATCTTACGGTGCTTAGTGCCTATGATTGACATA
ACATTCACTTTTTCACCTACATTGCCTCGTGTAGAAATTCTAGGAAGAATTTTCCATATCTAACCAGCAGCCCAAAACACATC
CCAATGCTATTACGGTGCGTGCAATTTGAGGGTAACCGGAGGCGTTCGCCGTAAACCACACTGTGCGGGGTGCAACCCAGC
CGCTGGGTCACATGCTCCAGCCTCCAAGCTTCCAAACCAAGCAACCCCCACCCCCACCACCTCCATCCGAGGGCGCAAAGC
GCAAAACCCCACCCACCCCGAGGTCCCCTCCGCAAAAGATCCACGCGTGCCCCGTCCCTCTCGCCGCCGGCCGCCGCCGCC
ACATTCTTACTACACTCCACCGCGGGCCAAGCACTCCGCCACTCCCCCACTGTCCCGCCTCAATGCGACGGCGGCCCCGCCG
CCGCCGCCGCCACCGACTCCGCCCATGGCCGTTCGAACCCTGCTGCTGCTCGTGCTGCTCTTGCTGCTGCTCGGTGACGCCA
CCTCGGCTTCCGTCATCGGCGAGCGCGAGGCGTTGTTGAAGTTCAAGGCGGCCGTGACGGCTGACCCGGGCGGGCTGCTGC

GGGA
>SsLRR-RLK82
TCGCTTTGACCGCGGCACGTTGACGGCTGCGGGTCCCGGGCAGCTGACCGACGGGGCGAGCGTCCGTCCTCGCGGGCGCC
CTCGCGCGACGCTGACAGCGGTCCCCGTGGTCCCGACCTCCGCGCGGACACGGGTCGCTTTCCGAACCCCCGCCGCGGAA
AGCAACGGGAGGGAGCGCACCAGCGCGCGGGGGGTGGGGGCGACCGACCGAACGGCACCGGGCCGAGCGCGGGGAGGC
CGCGGGATCGGGCGCGGACGGCGGGGATGCTGTGGGTGGGGGATGAGCCCGGTGGGTTTAGTTAGGCCTTTGGGGGCGCG
GTGTGAAAGGCGGCGCTGGAAAGCAGAGGCAGCGGATGGATGGATGGAGCGGGGCTGGGCTGGCCGGGGAAAAAGCAAA
GCGTGGGGGGCCTTTTCGCCTTCCACACCGCCTTTTCGCGAAATGGCACGCGGGCGCGCCGCGCTGGTGCTCTGCTCCCGA
GTGTTTTCCAGAGTGGGGTTTTGCGGCCGCCCACGTTGGTTTGCTCGTATTCTCAGAAACGACCCTCACCCATGGACCATGG
TTCCCTTCCCTACAGCTAAGTTCAGCTAACAAATGCAGGCGTATTAGAGTGAGTGGTAATATATTGTCACATGTATAACGTGCT
AGTACCTTGGTAGCTGCTTGCACGTGCTTGCTCGCATTTCCAAATCAAGTAATGTGCTGCTACCTTTTCTCTACTAATTTTTTC
TAGCAAAGAAAAAACATAACAATAGCTGCATATGTCATTGAGGTTAATTTCACGAAAGAATTAGGAGAGTTTAGAGGCTATT
AGCTCCTTGATACACTTGGTGGTTGAGCAAAGTAGGGTCCAATTTTATGCACATCTCTAGTGATTGTGCAACGAAACTAAGG
GTCTGTTTGGCATGATTTTGATTCTGTCAGCACCTTCTGCAGAGAAGACAGATGACTCCTATCAAACACTATAGGAGGAGAA
GTCGTTTTCTAGCGGATACCAGGCCATCCATCGGTCTGAGGAACATTTGAGGATGAGCATTGCCAAGCGGGTCCTAACATATA
CCAATACGTCATAATATAATGCATATCAACGAATAACCTCTTCTACTGCCCCAAATTTTCCATTATATTCTCTTTGATGTTATGGT
TGCATAGTCATATACCATCTCTTCCTTATCTTCCAACTGACATTTGCATTTTTCTTATGACCACATCCCTTGTTTATATGCTGTCT
TTCCTTTTGACACCTGGGATAGAATTGATCGTTCCATTTCTTTACACTATATTTAAGTATAATATGATCTTCTATACTATTTGGTC
CTTGCCAAATTTATCAAGCCACTTATCAATTAACACAGACGTGATATCCTTGACAACTATCTTCAATGATGCTAATTTTCTCATT
TTAGAGTTTATCTATGCACACATCTTTTTTTTTCTAGGTAACACGGGAGGTACGACGGACACCAACAAACTGGGTATTAGTTG
GTTAACACTAGAGCAACTATCAACCACAGACTTTTTATGTTCCAACTTTAAGATAACCTAATTTAGTAATTTTAGTTATTCGCC
TTTATCTCAGAATCTTACGGTGCTTAGTGCCTATGATTGACATAACATTCACTTTTTCACCTACATTGCCTCGTGTAGAAATTCT
AGGAAGAATTTTCCATATCTAACCAGCAGCCCAAAACACATCCCAATGCTATTACGGTGCGTGCAATTTGAGGGTAACCGGA
GGCGTTCGCCGTAAACCACACTGTGCGGGGTGCAACCCAGCCGCTGGGTCACATGCTCCAGCCTCCAAGCTTCCAAACCAA
GCAACCCCCACCCCCACCACCTCCATCCGAGGGCGCAAAGCGCAAAACCCCACCCACCCCGAGGTCCCCTCCGCAAAAGA
TCCACGCGTGCCCCGTCCCTCTCGCCGCCGGCCGCCGCCGCCACACTCTTACTACACTCCACCGCGGGCCAAGCACTCCGC
CACTCCCCCACTGTCCCGCCTCAA
>SsLRR-RLK65
ATATCTCACAAACAAAGTCAAGTTTGGACAAAAAATTTTACAAGGTTGTGTATCATCATATCATCTATATATGTAATTTTTTGGG
TGAATTTAGATGACTTTTTTTACCATGGTTTATATGGGTTTTCACGAAGGGTGTGGTTTCCACCTGATACGTTGCCTTTCCATA
GTGGGGTTTTGCGGCCGCCCACGTTGGCTTGCTTGTATTCTCAGAAACAACCCTCACCCATTGACCATGGTTCCCTTCCCTAC
AGCTAAGTTCACCTAAGAAATGCAGGCGTGTTAGAGTGAGTGGTAACATATTGTCACATGTATAACGTGCTAGTACCTTGGTA
GCTGCTTGCATGTGCTTGCTCGCATTTCCAAATCAAGTAATGTGCTGCTACCTTGTCTGTCTCTACTAATTTTTATCTAGCAAA
AAAATAATAATTCTAGCAAAGAAGAAACATACACAGCAGTTGCATATGTCGTTGAGGTTAATTTCACGAAAGAATTATGAGA
GTTTAGAGGCTATTAGCTCCTTGATACACTTGGTAGTTGAGCAAAGTAGGGTCCAATTTTACGCACATCTCTAGTGATTGTGC
GATGAAACTAAGGGTCTGTTTGGCATGATTTTGACTCTGTCAGCACCTTCTGTCAAGAAGACAGAGGACTCCTATCAAACAC
TTTAGGAGGAGTCGTTTTCTAGTGGATATTGGATAGTATATAAGCCGGACCAGGCCATCAATTGGTCTGGAGGAGCATTTGAG
GATGAGCATTGCAAAGCGGGTCCTAAGATTTACGTGTTTTTCTTCGCAAGAGAACTAAGTACCAATACGTCATAATATAATGC
ATATCAATGAATAACCTCTTCTACCGCCCCAAATTTTCCATTATATTGATTATATTCTCTTTGATGTTATGGTTGCATAGTCATATA
CCATCTCTTCCTTATCTTCCAACTGACATTTGCATTTTTTTTATGACCACATCCCTTACTTATATGCTGCCTTTCCTTTTGACACC
TAGGATAGAATTGACCGTTCCATTTCTTTACACTATATTTAAATATAATATGATCTTCTTTACTATTTGGTCCTTGCCAAATTTATC
AAGCTGCTTATCAATTAACAGACGTGTTATACTCGACAACTATCTTCAATGATGCTAATTTTCTCATTTTAGAGTTTATCTATGC
ACACATCTTTTTTTAGGCAATACGGAAGGTGTGACAAACACCAACAAACTGGGTATTAGTTGGTTAACACTAGAGCAACTAT
CAACCGCAGACTTTTTATATATGTTCTAACTTTAAGATAACCTAATTTTAGTTATTCGCCTTTATATCAGAATCTTATGGTGCTTA
GTGCCTATAATTGACATAACATTCACTTTTTCACCTACATTGCCTCGTGTAGAAATTCTAGGGCCTTGTTTAGATTGCAATTTTT
TTGAACTTTTGGCTACTGTAGCACTTTCGTATGTATTTGATAATTAGTGTCCAATCATGAACTAATTAGGCTCAAAAGATTCGT
CTCGTGATTTGCAACAAACTGTGCAATTAGTTATTTTTTTATCTATATTTAATGCTCCATACATGTGTCCAAAATTTGATGTGAT
GAAGAGAGAGTGTAAACATTTGGAATTTGGAGTGTACCTAAACAAGGTTTAGGAATAATTTTCCATATATAACCAGCAGCCC
AAAACACATCCCAATGCTATTACGGTGCGTGCAATTTGAGGGTAACCGGAGGTGTTCACCGTAAACCACACTGTGCGGGGT
GCAACCCATCCGCTGGGTCACATGCTCCAGCCTCCAAGCTTCCAAACCAAGCAACCCCCCCACCCCCACCACCTCCATCCG
AGGGCGCAAAGCGCAAAACCCCACCCACCCCGAGGTCCCCTCCGCAAAAGATCCACGCGTGCCCCGTCCCTCTCGCCGCC
GGCCGCCGCCGCCACACTCTTACTACACTCCACCGCGGGCCAAGCACTCCGCCACTCCCCCACTGTCCCGCCTCAAT
>SsLRR-RLK103
GGGGTGCTGGATGGATGGAGCGAGGCTGGTCTGGCGGGGAAAAAGCAAAGCGTGGGGGGCCTTTTCGTCTTCCACACCGC
CTTTTCGCGAAATTGCACGCGGGCGCGCCGCGCTGGTGCTCTGCTCCGAGGCCTCTTGTTTAGTTTCAAAATATTTCGCAAA
AAAAACAGTACATTTTCTGTCACATCGAATCTTTAGACATATATATATAGTATTAAATATAGATAAAAAATAAAACTAATTACAC
AGTTTAGATGAAATGGACGAGACGAATCTTTTAAGTTTAATTAAGCCATGATTGGACACTAATTACTAAATAACAACGAAAAA
TGCTACGGTTATATTTCGCAAAATATTTCAGGAACTAAACGAGGCCCGAGTGTTTTACAGAGTGGGGTTTTGCGGCCGCCCA
CGTTGGTTTGCTCGTATTCTCATAAACGACCCTCACCCATTGACCATGGTTCTCTTCCCTACAGCTAAGTTCAGCTAACAAAT
GCAGGCGTATTAGAGTGAGTGGTAACATATTGTCACATGTATAACGTGCTAGTATCTTGGTAGCTGCTTGCATGTGCTTGCTCG
CATTTCCAAATCAAGTAATGTGCTGCTACCTTGTCTTCTCTACTAATTTTTTCTAGCAAAAAAACCATTCTAGCAAAGAAAAA
ACATAACAATAGCTGCATATGTCATTGAGGTTAATTTCACGAAAGAATTAGGAGAGTTTAGAGGCTATTAGCTCCTTGATACA
CTTGGTGGTTGAGCAAAGTAGGGTCCAATTTTACACACATCTCTAGTGATTGTGCGACGAAACTAAGGGTCTGTTTGGCATG
ATTTTGGCTCTGTCAGCGCCTTCTGCAGAGAAGACAGATGACTCCTATCAAACACTATAGGAGGAGAAGTCGTTTTCTAGTG
GATACCAGGCCATCATTGGTCTGGAGGAACATTTGAGGATGAGCATTGCCAAGCGGGTCCTAAGATATACGTATTTTCTTCAG
CGGAAACTAATACCAATACGTCATAATATAATGCATATCAACGAATAACCTCTTCTACTGCCCCAAATTTTCCATTGTATTGATT
ATATTCTCTTTGATGTTATGGTTGCATAGTCATATACCATCTCTTCCTTATCTTCCAACTGACATTTGCATTTTTCTTATGATCAC
ATCCCTTGTTTATATGCTGTCTTTCCTTTTGACACCTGAGATAGAATTGACCGTTCCATTTCTTTACACTATATTTAAGTATAATA
TGATCTTCTTTACTATTTGGTCCTTGCCAAATTTATCAAGCCACTTATCAATTAACACAGACGTGATATACTTGACAACTATCTT
CAATGATGCTAATTTTCTCATTTTAGAGTTTATCTATGCACACATCTTTTTTTTCTAGGCAATACGGGAGGTACGACGGACACC
AACAAACTGGGTATTAGTTGGTTAACACTAGAGCAACTATCAACCACAGACTTTTTATGTTCCAACTTTAAGATAACCTAATT
TAGTAATTTTAGTTATTCGCCTTTATCTCAGAATCTTACGGTGCTTAGTGCCTATGATTGACATAACATTCACTTTTTCACCTAC
ATTGCCTCGTGTAGAAATTCTAGGAAGAATTTTCCATATCTAACCAGCAGCCCAAAACACATCCCAATGCTATTACGGTGCGT
GCAATTTGAGGGTAACCGGAGGCGTTCGCCGTAAACCACACTGTGCGGGGTGCAACCCAGCCGCTGGGTCACATGCTCCAG
CCTCCAAGCTTCCAAACCAAGCAACCCCCACCCCCACCACCTCCATCCGAGGGCGCAAAGCGCAAAACCCCACCCACCCC
GAGGTCCCCTCCGCAAAAGATCCACGCGTGCCCCGTCCCTCTCGCCGCCGGCCGCCGCCGCCACATTCTTACTACACTCCAC
CGCGGGCCAAGCACTCCGCCACTCCCCCACTGTCCCGCCTCAATGCGACGGCGGCCTCCGCCGCCGCCGCCGCCACCGACT
CCGCCCA
>SsLRR-RLK83
CTGACCGACGGGGCGAGCGTCCGTCCTCGCGGGCGCCCTCGCGCGACGCTGACAGCGGTCCCCGTGGTCCCGACCTCCGC

GCGGACACGGGTCGCTTTCCGAACCCCCGCCGCGGAAAGCAACGGGAGGGAGCGCACCAGCGCGCGGGGGGTGGGGGCG
ACCGACCGAACGGCACCGGGCCGAGCGCGGGGAGGCCGCGGGATCGGGCGCGGACGGCGGGGATGCTGTGGGTGGGGGA
TGAGCCGGTGGGTTTAGTTAGGCCTTTGGGGGCGCGGTGTGAAAGGCGGCGCTGGAAAGCAGAGGCAGCGGATGGATGGA
TGGAGCGGGCTGGGCTGGCCGGGGAAAAAGCAAAGCGTGGGGGGCCTTTTCGCCTTCCACACCGCCTTTTCGCGAAATGG
CACGCGGGCGCGCCGCGCTGGTGCTCTGCTCCCGAGTGTTTTCCAGAGTGGGGTTTTGCGGCCGCCCACGTTGGTTTGCTC
GTATTCTCAGAAACGACCCTCACCCATGGACCATGGTTCCCTTCCCTACAGCTAAGTTCAGCTAACAAATGCAGGCGTATTAG
AGTGAGTGGTAATATATTGTCACATGTATAACGTGCTAGTACCTTGGTAGCTGCTTGCACGTGCTTGCTCGCATTTCCAAATCA
AGTAATGTGCTGCTACCTTTTCTCTACTAATTTTTTCTAGCAAAGAAAAAACATAACAATAGCTGCATATGTCATTGAGGTTAA
TTTCACGAAAGAATTAGGAGAGTTTAGAGGCTATTAGCTCCTTGATACACTTGGTGGTTGAGCAAAGTAGGGTCCAATTTTAT
GCACATCTCTAGTGATTGTGCAACGAAACTAAGGGTCTGTTTGGCATGATTTTGATTCTGTCAGCACCTTCTGCAGAGAAGA
CAGATGACTCCTATCAAACACTATAGGAGGAGAAGTCGTTTTCTAGCGGATACCAGGCCATCCATCGGTCTGAGGAACATTT
GAGGATGAGCATTGCCAAGCGGGTCCTAACATATACCAATACGTCATAATATAATGCATATCAACGAATAACCTCTTCTACTGC
CCCAAATTTTCCATTATATTCTCTTTGATGTTATGGTTGCATAGTCATATACCATCTCTTCCTTATCTTCCAACTGACATTTGCAT
TTTTCTTATGACCACATCCCTTGTTTATATGCTGTCTTTCCTTTTGACACCTGGGATAGAATTGATCGTTCCATTTCTTTACACT
ATATTTAAGTATAATATGATCTTCTATACTATTTGGTCCTTGCCAAATTTATCAAGCCACTTATCAATTAACACAGACGTGATATC
CTTGACAACTATCTTCAATGATGCTAATTTTCTCATTTTAGAGTTTATCTATGCACACATCTTTTTTTTTCTAGGTAACACGGGA
GGTACGACGGACACCAACAAACTGGGTATTAGTTGGTTAACACTAGAGCAACTATCAACCACAGACTTTTTATGTTCCAACT
TTAAGATAACCTAATTTAGTAATTTTAGTTATTCGCCTTTATCTCAGAATCTTACGGTGCTTAGTGCCTATGATTGACATAACATT
CACTTTTTCACCTACATTGCCTCGTGTAGAAATTCTAGGAAGAATTTTCCATATCTAACCAGCAGCCCAAAACACATCCCAAT
GCTATTACGGTGCGTGCAATTTGAGGGTAACCGGAGGCGTTCGCCGTAAACCACACTGTGCGGGGTGCAACCCAGCCGCTG
GGTCACATGCTCCAGCCTCCAAGCTTCCAAACCAAGCAACCCCCCACCCCCACCACCTCCATCCGAGGGCGCAAAGCGCAA
AACCCCACCCACCCCGAGGTCCCCTCCGCAAAAGATCCACGCGTGCCCCGTCCCTCTCGCCGCCGGCCGCCGCCGCCACAC
TCTTACTACACTCCACCGCGGGCCAAGCACTCCGCCACTCCCCCACTGTCCCGCCTCAATGCGACGGCGGCCCCCCGCCGCC
GCCGCCGCCACCGACTCCGCC
>SsLRR-RLK17
ATACATAATGCTATTGTGTTCGGTAATTTTACATGGAAATCCTTAATGTTGTTGTTAAAGTGAACAAACTGTACAACCATATTTA
CACAATAAATGAATTTCTTTTTTTAATATAATTCTGATGTATAAGAATTTATTTTTTCCCCATTGCAATGCACGGGCAAGTTTGC
TAGTTATCTGTTTATGTACTAAATTTGCATAGTTGAAAATGATACGGATGGATCTATATCAAATAGTTTCATTGGGAGGTATATC
GTTGTTGTGTTTTACAATTATCTAAAATAAGAAATTAGTGGTCAAAATTATATCTTGTAGATTGTTTCAAGCAAAAGGCCACTT
ATCGACTGGAGAGACTATCTTGTACTCCCTTCTTTTCAAATTGTTACTCATCTTGGTTTCTCCAGATATATAGCTTTTGATATAT
GTATCTACAAAAGCTAAAACGACTTACAATTTAAATGGAACGAGTATTGTTTAACCAAAAAGTAAAGAAAATAATAAAAACA
TGGGAAATAATAAAATTATAAAACGGCTTATGCCTATTACTTCCTTTTAACCCAAGTATCTGTGTCAAGTTAAAAATGGCCAAT
GATGCATACTAATCATTTCTCATTTCTACATTCAAGAGAAAGTAAATCATCATATTAAAAAACAATTAGCAGAGCCAATGTAAC
GCCCCAAGGTAGCGTTCAGTTGCCAAAAATTTGGCAAAATGGCACTGTAGCCACTTTCGTTGTTATTTGGTAATTAGTGTCCA
ATTATGGTCTAATTAGGCTTAAAAGATTCGTCTCGTCTATTTCGTCTAAACTGTATAATTAGTTTTATTTTTTATCTATATTTAATG
CTTCATACATGTGTCCAAAGATTCGATGTGACAGGGAATCTGAAAATTTTTGCAAAATTTCTTGGGAACTAAACAGCCCCCA
ACTCAGAAAGACGGCTAAGGTCCACTATACACGTTGAATGAACTACACCTGCTAATTAACTCTCTTGCGCTTTCATCCTCGGT
TCACGTCGAAGTATCGATCCGGAGTTAAAAGCCTCCCAGATTCAGGCTTTTAACTTGCCTCAGCTCTCGTCTAACTTCCCGCA
TGTACTGAACTTTATAGCTATGCTGCTACAGTATCCTAAGGTACAGTGTTGCGAATTCGGTCCAGCCCAACTGGTCCAATGGA
CTGGGTCTGACAGCCATCAGGTAGCGAAGTACTTCTGGTACCTCGACGAGATACTTCCGTACTGTACTAACAGAGTACTCCC
TCCGTCCTAGAAAATATGTAATTCTAGCACAGTGTCATTGTTTAGTATCTCAAGTTCAATTAATTATAGATAAAAAATATCAATA
TTTATGATATCAAATAAATATCATTAGATTCATTACGAAATGTATTTTCTTAATAAATTAATTTAAAATCATAAATGTGAATAGTAT
TCACTAGAAATTTGGTCAAACGTAAACTACTTTTAACGGCACACATGCATGTTTTCTTGGACGGAGGGAGTATCAAGCAATT
CTGTTACGCCGTCTCCTCAAATTCGTTTGCACCAATGGAAACATGGGCACGAGGGGTGAACGAGTCAACAGCCCCAAAGCA
TGGGCAGGCAGCCCGCAGCCACCTCCATCATCAAACAAAACCAATCTTTCCAAGGGCAAAATCGTCACGTCAAGTACCTTT
AAAAATCAAGAGAATAAAAAAGAGTTGTCGCTGGACCTCGACGAGCAGGGGACGGTGGCGGAGAAGAAGAAAGAGCTGG
AAGGAAGCAACAGAACGAAGACCAGCACCAACGAGACCAGAGGAGAGGAGAGAAGAGAGATCAGAGAGTCACAGAGAG
AAATTTGCCGGCAAAAACTTGCCTTCTCCCCTCCGGAATCTAGCTAATTACTTTCCGGTCCTTCTCCCCCATGTAAGTCTCCC
CTCGTCGTCGCCTGCACCTACCGGAAGCGAAGCCTCCTACAGTTCAGGTCCACATCCGGGTCGCCGCCGTCGGCTAGCTAGC
CGGAT
>SsLRR-RLK33
TGCTCGTCATCAGCGACGAGCGTGCCAGGACAGAGGAGGCCGGCAAGGACGACGCGTGGTGACTTGTTGAGCACAACAGT
GGCAAGTTTCGGTTCCGCCTGCCGTGCGGCGCCAAGCTGGACCAGGTGCACATGTTAAGAAGCTGTAGGTAAGGATCGTCC
AGATCTGGTTGATAGATCCGTTCATTGCTTGTCCGCATGCATGGACGCGATGACAATGATACAGAGGGTTAAGCACTCAAGTT
GTAATACAAAGTCTTCCTGCCTGTAGGATTAGAAGAATAAAACTGTCTATCTATATCTATTCATCGTTTGTGTGTCTAATAAGTG
CTGTGTTTTGATGTCAAGTGCTTCAGTTGGGCATGGCTGTGGCTGCGGGCAATACAACTCGCAAAAATGCATGTTGCAACCA
TGGCAGATGGTAATGGGAGGGAACAACCCCGTTTTACTTCATATATAATCGAAGGAGAACAATACATAATGCTATTGTGTTCG
GTAATTTTACATGGAAATCCTTAATGTTGTTGTTAAAGTGAACAAACTGTACAACCATATTTACACAATAAATGAATTTCTTTT
TTTTAATATAATTCTGATGTATAAGAATTTATTTTTTCCCCATTGCAATGCACGGGCAAGTTTGCTAGTTATCTGTTTATGTACTA
AATTTGCATAGTTGAAAATGATACGGATGGATCTATATCAAATAGTTTCATTGGGAGGTATATCGTTGTTGTGTTTTACAATTAT
CTAAAATAAGAAATTAGTGGTCAAAATTATATCTTGTAGATTGTATCAAGCAAAAGGCCACTTATCGACTGGAGAGACTATCT
TGTACTCCCTTCTTTTCAAATTGTTACTCATCTTGGTTTCTCTAGATATATAGCTTTTGATATATGTATCTACAAAAGCTAAAACG
ACTTGCAATTTGAATGGAATGAGTATTGTTTAACCAAAAAGTAAAGAAAATAATAAAAACATGGGAAATAATAAAATTCTAA
AACGGCTTATGCCTATTACTTCCTTTTAACCCAAGTATCTGTGTCAAGTTAAAAATGGCCAATGATGCATACTAATCATTTCTC
ATTTCTACAGCCAGTCTCAGTTTCAAGAGAAAGTAAATCATCATATTAAAAAACAATTAGCAGAGCCATTGTAACGCCCCAA
CTCAGAAAGACGGCTAAAGTCCACTATACACGTTGAATGAACTACACCTGCTAATTAACTCTCTTGCGCTTTCGTCCTCGGTT
CATGTCGAAGTATCGATCCGGAGTTAAAAGTTTCCCAGATTCAGGCTTTTAACTTGCCTCAGCTCTCGTCTAACTTCCCATAT
GTACTAAACTTTGTAGCTATGCTGCTACAGTATCCTGAGGTACAGTGTTGTGAATTCGGTCCAGTCCAACTGGTCCAATGGAC
TGGGTCTGACAGCCATCAGGTAGCGAAGTACTTCTGGTACCTCGACGAGATACTTCCGTACTGTACTAACGGAGTATCAAGC
AATTCTGTTACGCCGTCTCCTCAAATTCGTTTGCACCAATGGAAACATGAGCACGAGGGGTGAACGAGTCAACAGCCCCAA
AGCATGGGCAGGCAGCCCGCAACCACCTCCATCATCAAACAAAACCAATCTTTCCAAGGGCAAAATCGTCACGTCAAGTAC
CTTTAAAAATCAAGAGAATAAAAAAGAGTTGTCGCTGGACCTCGACGAGCAGGGGACGGTGGCGGAGAAGAAGAAAGAG
CTGGAAGGAAGGAACAGAACGAAGACCAGCACCAACGAGACCAGAGGAGAGGAGAGAAGCCAGCGAGAGATCAGAGAG
TCACAGAGAGAAATTTGCCGGCAAAAACTTTCCTTCTCCCCTCCGGAATCTAGCTAATTACTTTCCGGTCCTTCTCCCCCATG
TAAGTCTCCCCTCGTCGTCGCCTGCACCTACCGGAAGCGAAGCCTCCTACAGTTCAGGTCCACATCCGGGTCGCCGCCGTCG
GCTAGCTAGCCGGATATGG
>SsLRR-RLK172
TAGTACGTCTACTACTTCGGTTAGCAATGCAACAGATGGATGGAGCCGAGCCTTGCATCGCCCGGCCGTGCTGTTGGAGCCA
AGACCTGCACTGTTCTGCCCGGACGTGCTGTACATTTACTCGGGCAAATTTTTTATCTTAAGTATTTCTAGACTATTTAAGTGA
TCAAACAGTAAGTCACTGTAAAAACTCTAGATTTCTCAATATCCAAGTCATAGAAAAAAACCAAATTTGTCAATTTCCATAGC

TCGGCGCCGTAGTAATCGGTGCCGAGCTTGGCGCCAAGATCTACGGCGTCGAGCTCAAGACACGTCCGCCCCACGCAGGTA
GCCACGTCAGCGACACCTAGAAAATAACCCTGGACTACGGCGCCAAGGATGGGTCTATTTTTGACAGGACCAGGGGCGAA
GCCAGGATATATCATCGTCGGGGTCAAGTGGAAGTACAAAATTATGGTTTCGTTTGGCAGAACTTTAGCTCCGGTTTCTTTTG
TGGCTCGTAGCAAGGAGCCCTGCCAAATGGACCTTAAAAAAATGGCTCCTTGCAAGGAGTCTGGAGAAGCTGCAGCAAAG
AAAAAAATGGTTTCTCGTGACTTCTCCTCACAAACATAACACAAATTACTACAAAACTACTCACTAGAGTTGTTTTTGCCAA
ACATGTTTTAAAACGGCTCTAGCTTCACATGGGGAGCCAGAGCCGAAGCCTTTTTAAGAGAAGCCGGAGCCCTAGCAAACA
GGTCTATAACTTGCGATAATTTATACAATAACATGTATAACATAACCCTACAATGGATAAGATAGAAGCAACAAAATGACATGT
TTGCTTTACGATTATATATTAGACCTTGCTATGGTATTCCTGTCCCACTTAAGAGGTAATATTCAATTAAATTTGAACTCTTGATT
TTTGTGTTTTTAAAATGCATGCAATAAATAAATACTCCCTCCGTATCGGATTTAGAAGTTGTTTAGGACAGCAACACGGTCTCC
AAAACACAACTTTGACCTCTTGTTTTTATAAAAAAATATTTATGGAAAAGTGATATATGTATACTTTTATGAAAGTATTTTTCAA
GACAAATCTATTCATATAATTTTTACATTTGCAAACTCAACAACTTAAAAGTTATTGATGATTTATATTCTCAATGTTTGACCCA
AACCTTGTCCCAAACGATTTCTAAATCTGATACGGAGGGAGTAGTTAAGTTATTTTTCTTTTAGAAAAGAATATCCCAGAAAT
CTTGACTCTTAAGTCCCTATGCCTCCGTCCTTCGTGTTCTGTTTCCTATTCAGCATTTCCGTCTCCCGTCGAGCCACCAGGGCC
ATTCACGGTCAGGCGCACAGACATGAGAAAGCCGACAAGGCAAGAACGAACATCACAAGGACGGGGAAAGGCCGAAAGC
GACTTGGAGCGTTGGACAACCGGAGTGATCAGAGTAGAAGGGGATTAAATTGGACTGCTACTGGGCTGCTGCATGCGTTTTT
TTACTTCCATTAACTTATCATTAGATGGGCCATCGGGGCTGTCGGGGTCTCGAATATTATTTACCGGGGTCACAAATGGTGAAA
AATCGCAGGGCACAGGAGGTTAGCGGAAGGTCGTTGGGGTCGGACGACCCCGACGCCATAACGTAGCTTCGCCACTGGAC
AGGACCACCAGAGGCACTTTTGTGAAAATCTTTTTTAAAAAAAGGTCAAAAAGCTAAAAATTTGAACGCAGCGATGAACAA
TTGAACACCACAGAGCCAAAAAGCCAAGCATCCCGTCGCACTATGCCCCCTCGCATTTCCTGAAATCACTCCACAGTCCTCC
ATTCCTGACCTATGAAAGTTCAGCCCCCGGGCGTGGGCCCCACCTCGTGTACCAGTACTTGTCTCCGAGTCCATCGACACTT
CGACAGCAGTCGGCAGACTCGGAGAGTCACTCGGTCGCCACTGACTGACCCAGACAAAGCTCCCCACCACCGCTCCCGTT
CCGCTTCGGCGACTGGCCGGAG
>SsLRR-RLK97
CGCTTAGTGAGGTAGTGTGTCCCACAACCATAAGCAACGTGAAGTTATGCATGCGAGCTATGTGTAACTCATGCAAGTGTTA
CTTTTCATGGCAAAATTGATAATTAAGTATCTGTTTTTGTCGTATGACCGAACTACGCAACACAAAAGAGATACATCTCTAGAT
CGCTTGTCGAGAGAAATATGTCCTCGAATCAAAGAAAAGGCACTGACGTTGTTTGAGTGCAAGCCATCAATATGCCCCAGA
ATCTAGAGTACAACACCGCCTAATAAGGTAGTGGGTCCCACGACTCCATGCACCGCTAACATGGAGTCGCATGTAAGTTATGT
GTAACTCATGCCAAGTGTTGCTCCACACATAAAGATATAATTAAGTATTTATTTTATTGTTGCATGGCCTGCATACTCAACATAA
AAGGTAGTGCTCGTAATGAGGCTAGGCAAATAAATTGCATTTGAGACCCTCTTTGGGATTAAAAGAATGAAGGGGTTGACAA
GAAAATTAGAAAAAATAATAGGGTTGAAGGGCCAAAGGTTCACTTTTATTGTCCCTAATAAGCTTTATTCTAGATTTACCCTT
GGTTTTGCTCTTATTAGTTCCTTCTCATACTACCCATGTTGATGTTGCCTAAGCAAGAACTTCGTAGAATATACTAAAAATAAAT
TATAGCATTTTATTTATGCCCGTTTACCATCGACGTTTTTGGACTGAAAGAATTTCACATGAAAGAATTATAAGTTTTCCTTACT
GTGACGTAGTTGTGCCACACATGCTTTGAATCATATTAAAAAAAATCCTTAACTACCATGGACACTTGTATGGATGGAAGGTG
ATTGAGCTTAATAAAAGGAAATGGATGTCAAAACTACAATACATGCTTCTGGAGCTTCCTTGATAGGCTAACTCCTTGTAACA
AAAGTCTTCCAAGATTGGAGAATGTAAGCAATAATATGATGACCTACTGTTTCATTTGCTAAATGTCAATCAAGAAAATATAG
AATGACAATGCATCTCAAGATTTATGGGCAGTTAATTCATCCTAACCAATGCCAAGATTCCACACTATATCCACCCTAGCTTCT
TTTCTCGTGATTCATTATGTCGCTTATAACGAAAATGGACAATTTCCTCGTGGGCTCACATGCCCATCTTAAATCTCACACATC
CAGTGGGCCGTTAATGAGCCGTGATCTCCTAGACTAGGTTGGCTTGATCCATAGCCTAGTGATAAGAGGTTGCTTTGTCGTAT
GTGATGGAGAGTACATGTTATCCGAGAGTCGTGGTGCAAGGGACTTCTTGTAGTGGTACGGTTTGGGCTTCGAGTTTATGGG
CCGATGCTATTGGACCGCGGCCTCTAGAAAAATATGGGCTACTTTTCGTAACATTTGAATATATGAAATTTCGCGCCAAGACA
GTTATTCCACTTTTAACTCATACAAACTAGCCACTCTTTGCAGCAATGTGCAAAATTAGTTTATAGCATCTACCAAACGAATTG
ATAATCATATTTTCCAAAAATAAATGATTAGGCCACAACCAAAATGATTGCAATTCCTTCCACCTCGGTAAATTCTCAAACTAC
GATGGAATTCCCAAAATGGGTGACCATGACCAAGCAACGAAAAGTAAAACACCAACAAGTCACAGCAGGTGAATGCTGCG
TTGAACAGAAGAGGACGACGGCAGCAGCCGCCAGGAAGCCGGCGACGCAGCAATGAAACAATTGAACACCACAAAACCG
ACCATCCCCGTCGCACTCCGCCCCCCTCCGATTTCCTGAAATCACTCCACGGTCCTCCGTTACCAGGCCTATGAAAGTTCAGT
TCAGCCCCGGGCGTGGGTCCCACCGCGTGTACCAGTACTAGTCTCCCAGTCGGCAGACTCGGGAGTCACTCGGTCGCCACT
GACAGACAAAGCTCCCCACCACCGCTCCCGTCCCGCTTGGCGCTTCGAGGAGGTCCAGGTCCAGGTCCGCGTCCTCGTCGG
CGACTGGCCGGAG
>SsLRR-RLK119
ACCACCGCTTAGTGAGGTAGTGTGTCCCACAACCATAAGCAACGTGAAGTTATGCATGCGAGCTATGTGTAACTCATGCAAG
TGTTACTTTTCATGGCAAAATTGATAATTAAGTATCTGTTTTTGTCGTATGACCGAACTACGCAACACAAAAGAGATACATCTC
TAGATCGCTTGTCGAGAGAAATATGTCCTCGAATCAAAGAAAAGGCACTGACGTTGTTTGAGTGCAAGCCATCAATATGCCC
CAGAATCTAGAGTACAACACCGCCTAATAAGGTAGTGGGTCCCACGACTCCATGCACCGCTAACATGGAGTCGCATGTAAGT
TATGTGTAACTCATGCCAAGTGTTGCTCCACACATAAAGATATAATTAAGTATTTATTTTATTGTTGCATGGCCTGCATACTCAA
CATAAAAGGTAGTGCTCGTAATGAGGCTAGGCAAATAAATTGCATTTGAGACCCTCTTTGGGATTAAAAGAATGAAGGGGTT
GACAAGAAAATTAGAAAAAATAATAGGGTTGAAGGGCCAAAGGTTCACTTTTATTGTCCCTAATAAGCTTTATTCTAGATTTA
CCCTTGGTTTTGCTCTTATTAGTTCCTTCTCATACTACCCATGTTGATGTTGCCTAAGCAAGAACTTCGTAGAATATACTAAAA
ATAAATTATAGCATTTTATTTATGCCCGTTTACCATCGACGTTTTTGGACTGAAAGAATTTCACATGAAAGAATTATAAGTTTTC
CTTATTGTGACGTAGTTGTGCCACACATGCTTTGAATCATATTAAAAAAATCCTTAAGTACCATGGACACTTGTATGGATGGAA
GGTGATTGAGCTTAATCAAAGAAAATGGATGTCAAAACTACAATACATGCTTCTGGAGCTTCCTTGATAGGCTAACTCCTTGT
AACAAAAGTCTTCCAAGATTGGAGAATGTAAGCAATAATATGATGACCTACTGTTTCATTTGCTAAATGTCAATCAAGAAAAT
ATAGAATGACAATGCATCTCAAGATTTATGGGCAGTTAATTCATCCTAACCAATGCCAAGATTCCACACTATATCCACCCTAGC
TTCTTTTCTCGTGATTCATTATGTCGCTTATAACGAAAATGGACAATTTCCTCGTGGGCTCACATGCCCATCTTAAATCTCACA
CATCCAGTGGGCCGTTAATGAGCCGTGATCTCCTAGACTAGGTTGGCTTGATCCATAGCCTAGTGATAAGAGGTTGCTTTGTC
GTATGTGATGGAGAGTACATGTTATCCGAGAGTCGTGGTGCAAGGGACTTCTTGTAGTGGTACGGTTTGGGCTTCGAGTTTAT
GGGCCGATGCTATTGGACCGCGGCCTCTAGAAAAATATGGGCTACTTTTCGTAAATTTGAATATACTAAATTTCGCGCCAAGA
CAGTTATTCCACTTTTAACTCATACAAACTAGCCACTCTTGCACAATGTGCAAAATTAGTCTATAGCATCTACCAAACGAATTG
ATAATCATATTTTCCAAAAATAAATGATTAGGCCACAACCAAAATGATTGCAATTCCTTCCACCTCGGTAAATTCTCAAACTAC
GATGGAATTCCCAAAATGGGTGACCATGACCAAGCAACGAAAAGTAAAACACCAACAAGTCACAGCAGGTGAATGCTGCG
TTGAACAGAAGAGGACGACGGCAGCAGCCGCCAGGAAGCCGGCGACGCAGCAATGAAACAATTGAACACCACAAAACCG
ACCATCCCCGTCGCACTCCGCCCCCTCCGATTTCCTGAAATCACTCCACGGTCCTCCGTTCCCAGGCCTATGAAAGTTCAGTT
CAGCCCGGGCGTGGGTCCCACCGCGTGTACCAGTACTAGTCTCCCAGTCGGCAGACTCGGGAGTCACTTGGTCGCCACTGA
CAGACAAGGCTCCCCACCACCGCTCCCGTCCCGCTTGGCGCTTCGAGGAGGTCCAGGTCCAGGTCCGCGTCCTCGTCGGCG
ACTGGCCGGAGA
>SsLRR-RLK76
AATTAAGTATTTATTTTATTGTTGCATGGCCTGCATACTCAACATAAAAGGTAGTGCTCGTAATGAGGCTAGGCAAATAAATTG
CATTTGAGACCCTCTTTGGGATTAAAAGAATGAAGGGGTTGACAAGAAAATTAGAAAAAATAATAGGGTTGAAGGGCCAAA
GGTTCACTTTTATTGTCCCTAATAAGCTTTATTCTAGATTTACCCTTGGTTTTGCTCTTATTAGTTCCTTCTCATACTACCCATGT
TGATGTTGCCTAAGCAAGAACTTCGTAGAATATACTAAAAATAAATTATAGCATTTTATTTATGCCCGTTTACCATCGACGTTTT
TGGACTGAAAGAATTTCACATGAAAGAATTATAAGTTTTCCTTACTGTGACGTAGTTGTGCCACACATGCTTTGAATCATATTA

AAAAAATCCTTAACTACCATGGACACTTGTATGGATGGAAGGTGATTGAGCTTAATAAAAGGAAATGGATGTCAAAACTACA
ATACATGCTTCTGGAGCTTCCTCGATAGGCTAACTCCTTGTAACAAAAGTCTTCCAAGATTGGAGAATGTAAGCAATAATATG
ATGACCTACTGTTTCATTTGCTAAATGTCAATCAAGAAAATATAGAATGACAATGCATCTCAAGATTTATGGGCAGTTAATTCA
TCCTAACCAATGCCAAGATTCCACACTATATCCACCCTAGCTTCTTTTCTCGTGATTCATTATGTCGCTTATAACGAAAATGGA
CAATTTCCTCGTGGGCTCACATGCCCATCTTAAATCTCACACATCCAGTGGGCCGTTAATGAGCCGTGATCTCCTAGACTAGG
TTGGCTTGATCCATAGCCTAGTGATAAGAGGTTGCTTTGTCGTATGTGATGGAGAGTACATGTTATCCGAGAGTCGTGGTGCA
AGGGACTTCTTGTAGTGGTACGGTTTGGGCTTCGAGTTTATGGGCCGATGCTATTGGACCGCGGCCTCTAGAAAAATATGGG
CTACTTTTCGTAACATTTGAATATATGAAATTTCGCGCCAAGACAGTTATTCCACTTTTAACTCATACAAACTAGCCACTCTTG
CACAATGTGCAAAATTAGTTTATAGCATCTACCAAACGAATTGATAATCATATTTTCCAAAAATAAATGATTAGGCCACAACCA
AAATGATTGCAATTCCTTCCACCTCGGTAAATTCTCAAACTACGATGGAATTCCCAAAATGGGTGACCATGACCAAGCAACG
AAAAGTAAAACACCAACAAGTCACAGCAGGTGAAGTTGAACAGAAGAGGACGACGGTAGCAGCCGCCAGGAAGCCGGCG
ACGCAGCAATGAAACAATTGAACACCACAAAACCGACCATCCCCGTCGCACTCCGCGCCCTCCGATTTCCTGAAATCACTC
CACGGTCCTCCGTTCCCAGGCCTATGAAAGTTCAGTTCAGCCCCGGGCGTGGGTCCCACCGCGTGTACCAGTACTAGTCTCC
CAGTCGGCAGACTCGGGAGTCACTCGGTCGCCACTGACAGACAAAGCTCCCCACCACCGCTCCCGTCCCGCTTGGCGCTTT
GAGGAGGTCCAGGTCCAGGTCCAGGTCCAGGTCCAGGTCCGCGTCCACGTCGGCGACTGGCCGGAGATGGCCTTCCTCCA
CCGCTCCGCCACCACCGCCGTCCTCTTCGCTCTCCACGTCCTCCTCCTCACCGCCGCCTCCGTCGCGTCCTCGCCGTCGTCC
CTGCCGGAGCAGGAGGACGACATGCTGGCGCTGCTCCACCTCAAGCGCGCCCTCACCTCCGGCGGCGGCGCCGGCGACGC
TCTCCGCCAGTGGTCGCCGGAGTCCGGCGCCCACCACTGCTCCTGGCCGGGGGTCACCTGCGACGCGCGGTCCGGCCGCGT
CGTGGCCCTCGCGCTCGGCGGGCGCCTCGGCGGGCAGCTGTCCCCGGCGGTCGCTCGCCTCACCGAGCTTAAAGCGCTCTC
CTTTCCCTCCGCGGGGCTTGGC
>SsLRR-RLK104
TAACTCGATTTATAAAAAAATGTGCAACATTTATATCTCCAAATAAATTTATTTAAAAACTAGATCCAAAATTCTTTCTAATGAT
ACTAATTATGTACTATAAATATTAATATTTTTTATTACATGTTTAGTCAAAGTTGTTTATCAGGAAACGTAAAATATAGATATTCA
GGACAGAGGGAGTATACGTGACGGCTGAGTAGGACTTCCAAAACGCAGGAACCCATGGCGAGTGTGCGAGCAGAGCCCAC
CGATGCAATGCAAAAGAGGCGCACCCACGCAAGCAGTCATGATACGATTCCCCCCGTCTGTGGACTGTGTGGGCATCTTCAA
CTTCAAGGTGGGCCTCTCTTCAGCTACCTCTCTTTTCTTTTCCCCCTTGGAGACCTTGTGGTACAATTTCCATGAAAAGCACT
CCTACTGCTTGCTGTAATACACAACACAAGCTGCCTGCGTTCAGATGCGACCTTGCGCTCAGACCTGGTTTGGTAGAGGCCA
CGCACGCGCTCAGACACGCGGATCCACCATCCACCCACCGCTTTTTCGCTTTACGACGGTGGTCTTCGACGGGGACGGGGG
ACATTCCAGGCCTGACGCCACTCGCGTCTGCCGGCGCGCGCCCCACTGGCCTTTTCTATCCTCCGTGTTGGCGGCATCACCG
CGCCTGAAACTTCATGGAGTGCCCTTGGATCGAGTGCGGGCGGCGGGGCGGGTGCACGCCCACCACACGTCCACACGCATG
CCCGGCGGAGGCGTGCGCGACGCGCCTGCCCTTCCCGAATGCCCCGATACCCTACGGGCTACTACGGCCCCCGCTCCTCTTC
GCTTCGAAGTAAATTTTGGACGCGAGACCGCCGCAGCAAAGGCGTCACGCCTCACGCGTCAGTAGTAGTCGCGTTGGAGTA
TGGCGTCGTCAACATGGGCGGCTGCCGTCGCTGACCTGACGGGCAAATATCCATTACATGTACATGTACTGTACGCACTCCG
ATTAAAAATATTTCCACGATCTTGCAAACTTTTCTCCCTACATTTGATATATATATATATATATATATATATATATATATATATAT
ATATATATATATATATATATATATATATATATATATATATATATATATATATAAGTTATAATATATATAACACAGAAAATTATGGTCAA
AGTTGCTGCCTAAAGGCACACAAAATGTAAAAGAACACGAGTTCTACCGGCAGCCCTTAATTAATTACACTCAAGCCTCGCG
GTAACCCTGTAGGAGTACCTTTCTATGTTCAGTGGACAAGTTACGCTACAACTTCTCTTTAACCCAACGTCACTGTCGAGGC
GTTCGTGCCCGTGCGACAGCAGTGTGAACCAGTGGTGACTGGTGAACCAACCGCTGTTTTTCCAGTTGAAGATCCCCCCTA
ACCCCAACCCGGGGAGGATTTAGCAAGTGGTGAAATTATACAATATGGTGACAAGTCCAGGCCTAATTCGGAAAGCAAGGA
GACAAATTCAAAGACCACTACCCAAGCAAAGCTACTAGTTGCCCCTTTCTCTTTCTCATCTGCTCCCCGTGCAGTCAGCAATT
CAGCGTCATACAAGTACTTAGCAATTAAAAAAAGTTGAACTCAAGCCACAGCCGATAAACCCAAGCTCCGTTGTCTGACGA
ATTGAGCAGTGTGGTGTCCAGTAAACGCAGGGATTTAGGCCTTGCTTAGTTGGCCGAATCCGGCGCCGCCGAAATCACTGTA
GCGCATGTAGTATTTCGATTATTTGGTAATAATTGTTCAACCATTACTAATTAGCTCAAAACATTTGTCTCGCAAAGTACAACC
AAACTGTGCAATTAGTTTTTGATTTTATCTATATTTAGTACTTCATGCATATACCGCAAGTTTGATGTGACGGAGAATCTTCTTT
TTGTATAGTGCCAAATTCAGGAGCTTAGGGACTAAACATGGCCTTAATCCATTCAAATGCCAGGCCCGGGCGCAACAACGAC
AGCCGGTAG
>SsLRR-RLK66
GTCAGTCGGTCATATGCTGCGGTTTGACTAGAATCGCACTATCTATTTATTCCTCCGTCCCTTTATATATGTCGCGCGTTGGTGT
GTATACCACAAGTTTAACTCGATTTATAAAAAATGTGCAACATTTATATCTCCAAATAAATTTATTAAAAACTAGATCCAAAATT
CTTTCTAATGATATTAATTATGTACTATAAATATTAATATTTTTTATTACATATTTAGCCAAAGTTGTTTCTCAGGCGTAAAATATA
GATATTCAGGACAGAGGGAGTATACGTGACGGCTGAGTAGGACTTCCAAAACGCAGGAACCCATGGCGAGTGTGCGAGCA
GAGCCCACCGATGCAATGCAAAAGAGGCGCACCCACGCAAGCACTCATGATACGATTCCCCCCGTCTGTGGACTGTGTGTG
CATCTTCAACTTCAAGGTGGGCCTCTCTTCAGTTACCTCTCTTTTCTTTTCCCCCTTGGAGACCCTGTGGTACAATTTCCATGA
AAAGCACTCCTCCTGCTTGCTGTAATACACAACACAAGCTGCCTGCGTTCAGATGCGACCTTGCGCTCAGACTTGGTTTGGT
AGAGGCCAGACACGCGGATCCACCATCCACCCACCGCTTTTTCGCTTTACGACGGCGGTCTTCGACGGGGACGGGGGACAT
TCCAGGCCTGACGCCACTCGCGTCTGCCGGCGGCGCCCCACTGGCCTTTTCTATCCTCCGTCTCGTGTTGGCGGCATCACCT
TCATGGAGTGCCCTTGGATCGAGTGCGGGCGGCGGGGCGGGTGCACGCCCACCACACGTCCACACGCATGCCCGGCGGAG
GCGTGCGCGACGCGCCTGCCCTTCCCGAATGCCCCGATACCCTACGGGCTACTACGGCCCCCGCTCCTCTTCGCTTCGAAGT
AAATTTTGGACGCGAGACCGCCGCAGCAAAGGCGTCACGCCTCACGCGTCAGTAGTAGTCGCGTTGGAGTATGGCGTCGTC
AACATGGGCGGCTGCCGTCGCTGACCTGACGGGCAAATATCCATTACATGTACATGTACTGTACGCACTCCGATTAAAAATAT
TTCCACGATCTTGCAAACTTTTCTCCCTACATTTTGATATATATATATAACACAGAAAATTATGGTCAAAGTTGCTGCCTAAAG
GCACACAAAATGTAAAAGAACACGAGTTCTACCGGCAGCCCTTGATTAATTACACTCAAGCCTCGCGGTAACCCTGTAGGA
GTACCTTTCTATGTTCAGTGGACAAGTTACGCTACAACTTCTCTTTAACCCAACGTCACTGTCGAGGCGTTCGTGCCCGTGCG
ACAGCAGTGTGAACCAGTGGTGACTGGTGAACCAACCGCTGTTTTTCCAGTTGAAGATCCCCCCTAACCCCAACCCGGGGA
GGATTTAGCAAGTGGTGAAATTATACAATATGGTGACAAGTCCAGGCCTAATTCGGAAAGCAAGGAGACAAATTCAAAGAC
CACTACCCAAGCAAAGCTACTAGTTGCCCCTTTCTCTTTCTCATCTGCTCCCCGTGCAGTCAGCAATTCAGCGTCATACAAGT
ACTTAGCAATTAAAAAAAGTTGAACTCAAGCCACAACCGATAAACCCAAGCTCCGTTGTCTGACGAATTGAGCAGTGTGGT
GTCCAGTAAACGCAGGGATTTAGGCCTTGCTTAGTTGGCCGAATCTGGCGTCGCCGAAATTACTGTAGCGCATTGTAGTATTT
CGATTATATTTGGTAATAATTGTCCAACCATTAACTAATTAGGCTCAAAACGTTCGTCTCGCAAAGTACAACCAAACTGTGCA
ATTAGTTTTTGATTTTATCTATATTTAGTACTCATGCATTACCGCAAGTTTGATGTGACGGAGAATCTTCTTTTTGTATAGTGCC
AAATTCAGGAGCTCGGGGGACTAAATTCAGGAGCTCGGGGGACTAAACATGGCCTTAATCCATTCAAATGCCAGGCCCGGG
CGCAACAACGACAGCCGGTAG
>SsLRR-RLK84
TTCTCAGAAAACGTAAAATACAGATATTCAGGACAGAGGGAGTATACGTGACGGCTGAGTAGGACTTCCAAAACGCAGGAA
CCCATGGCGAGTGTGCGAGCAGAGCCCACCGATGCAATGCAAAAGAGGCGCACCCACGCAAGCACTCATGATACGATTCCC
CCCGTCTGTGGACTGTGTGGGCATCTTCAACTTCAAGGTGGGCCTCTTCAGTTACCTCTCTTTTCTTTTCCCCCTTGGAGACC
TTGTGGTACAATTTCCATGAAAAGCACTCCTCCTGCTTGCTGTAATACACAACACAAGCTGCCTGCGTTCAGATGCGACCTT
GCGCTCAGACTTGGTTTGGTAGAGGCCACGCACGCGCTCAGACACGCGGATCCACCATCCACCCACCGCTTTTTCGCTTTAC
GACGGTGGTCTTCGACGGGGACGGGGGACATTCCAGGCCTGACGCCACTCGCGTCTGCCGGCGCGCGCCCCACTGGCCTTT
TCTATCCTCCGTGTTGGCGGCATCACCGCGTCTGAAACTTCATGGAGTGCCCTTCGATCGAGTGCGGGCGGCGGGGCGGGTG

CACGCCCACCACACGTCCACACGCATGCCCGGCGGAGGCGTGCGCGACGCGCCTGCCCTTCCCGAATGCCCCGATACCCTA
CGGGCTACTAGGGCCCCCGCTCCTCTTCGCTTCGAAGTAAATTTTGGACGCGAGACCGCCGCAGCAAAGGCGTCACGCCTC
ACGCGTTGGAGTATGGCGTCGTCAACATGGGCGGCTGCCGTCGCTGACCTGACGGGCAAATATCCATTACATGTACATGTAC
TGTACGCACTCCGATTAAAAATATTTCCACGATCTTGCAAACTTTTCTCCCTACATTTTGATATATATATATATAAGTTATAATATA
TATAACACAGAAAATTATGGTCAAAGTTGCTGCCTAAAGGCACACAAAATGTAAAAGAACACGAGTTCTACCGGCAGCCCT
TAATTAATTACACTCAAGCCTCGCGGTAACCCTGTAGGAGTACCTTTCTATGTTCAGTGGACAAGTTACGCTACAACTTCTCT
TTAACCCAACGTCACTGTCGAGGCGTTCGTGCCCGTGCGACAGCCGTGTGAACCAGTGGTGACTGGTGAACCAACCGCTGT
TTTTCCAGTTGAAGATCCCCCCTAACCCCAACCCGGGGAGGATTTAGCAAGTGGTGAAATTATTCAATATGGTGACAAGTCC
AGGTCTAATTCGGAAAGCAAGGAGACAAATTCAAAGACCACTACCCAAGCAAAGCTACTAGTTGCCCCTTTCTCTTTCTCAT
CTGCTCCCCGTGCAGTCAGCAATTCAGCGTCATACAAGTACTTAGCAATTAAAAAAAGTTGAACTCAAGCCACAACCGATAA
ACCCAAGCTCCGTTGTCTGACGAATTGAGCAGTGTGGTGTCCAGTAAACGCAGGGATTTAGGCCTAGCTTAGTTGGCCGAAT
CCGGTGCCGCCGAAATCACTGTAGCGCATCGTAGTATTTCGATTATATTTGATAATAATTGTTCAACCATTAACTAATTAGGCTT
AAAACGTTCGTCTCGCAAAGTACAACCAAACTATGCAATTAGTTTTTGATTTTATCTATATTTAGTACTTACCGCAAGCTTGAT
GTGACGGAGAATCTTCTTTTTTATAGTGCCAAATTCAGGAGCTTGGGGGACTAAACATGGCCTTAATCCATTCAAATGCCAGG
CCCGGGCGCAACAACGACAGCCGGTAGCACTTAGCAGCGGTTGGCGTGCTCGTCGCCCACATGCGGGCCTCGGGCCATTAG
ATAACTTGCGATAAGTGTCAAAGATTTTGTCTCTTATTCTCTGCTTACTTCACCTCTCCCCGTGGAGAACCAGAGCAAGAGAG
CGCACGCGCGTGTGGATGTGGAGGCTGCTCGCCGCTCGGCACGTCGTCCCCGTCGCCGGTGCTTCCGCTTCGCCTCGGTGT
CTTCCATGATTCGGGAAGCAAGGCCAT
>SsLRR-RLK279
GTTTATGCGTGTTTCTACTCAAATGCTTTTTAGCAGCCTGTCTGGTGTAGAAAACTGGAGAACTGTCTGTCCTGGGTGTCTG
CTGTACTGTACATGTTTCTCAGAGAGAAGTGCATTTTATCATGGAATCAGCGGAGCTCACGGGTTGCTAAAATCCAAGGAC
TGCTCGCATTGACCATTGAGCCATGAAAGTCAGGTGACATGAGAACCGGAAGACAAGGACAGCACCTGTGTGGGCTGTGG
ACTGTGGCAGCCCAGGCATCTATTACTGTCGCCCTGCGTATCTGCTTGCTGATTTCTTGTGCAGTATTTCTTTATTTGTTTTGTT
TCGATTTGTGGATGGAGCTGGCGCTGAGGATTAAAATATCAACCAGGGCGGTTGTTCTACTGATTGTTTCTGGGTGCATTTGT
CTGGCTAACTCTTCAGTATGATTGTGCGGTAAATTTTCATTGGAAAGCTATCTCATCCGATGTTCCCAGCAAAGCATGTCTTTT
TTTTTTTACACCTTTACATCATAGGTTGTTGCGGAGTAGCGTTCTTAGTATGGCAGGGAACAATGGTAACGAGTAACAATGAT
CACCCACTCTGTAACGAGTTACAACGATCAAAGTATGGCAGGGAACAATGGTAACGAGTAACAATGATCACTCACTCTGTAA
CGAGTTACAACGATCAATCACTCACTCTGTAACGAGTAACAATGATCACTCACTCTGTAACGACTAACAATGATCAATCACTC
TGTAACTACTCTGCTATCCAGCCAAAGGCCGTGGGAGTTGTGACTCCGTTGACATATCCCTCACCCACCTTCCGTTGGCAGA
GCTATCCGCTTAGCTTTTCAGTCATATTTCTTTAATAAAAAATAATATTTTTCTCTTACAATAAATTAGTAAACGATATATTTAGT
CTGCCATCTCAGGAAAGCGAACCTCGTTCCTCGTCCGGGGGATGAACGTCACGTCGCCTCTTTTTTTTTCGCGATCGTGCAT
GAGCACGTTTTTCATTAAAGAGAAGAGAGTTTGTTACAAACTAACCGCCTTGGTAACCCGCAGTTACCTCGGCAAAACCAA
CAGCACCTAGAACACGTAGAACAACAAGTCGGTATTACAGGACCGAAACTCGCGACAACGGGTGGGACAAACAGGCAGAA
GACAACAAAGTCCGCAGCTGACTACACCCCGCAAGGCTCCAGTTCTCGATCTCGTCCTGGATCAGCGATTCCAACTGCGCC
GGCGTAGTCGCGACACCATTGAAGGTTCTAGCGTTCCGCTCCTTCCAGATCAACCAACCAATGAGGAAGAAGAGGGAATCA
AAACCTCTACGCATCGTCTTCGGGATCCTCTTCCTGAACTGAATCCACCAAGATATCGCCGGCCCCGGCTGAACGGTGATCAT
ATCACCTAGATGCAGTTTCATCAGGCAGCGGCTCCACACCTCACGAGCGAAGGGGCATTCAAGTAGGATATGATCCAAAGTT
TCGGCGCTTTGGTCACAGATGATGCAGGTATTAGAGTCTTGCAGCCCATGGCGATACCGTCTGTCCCCGGTCCAGCATCTCCC
GTGCACAGCCAGCCACCAAAAGAACTTAACCCGAGGAGGTGCGGAAGTCTTCCAAACCTGCCGTGCACCGAACGGTTGCG
AAGACCCAAGGAACATGGCCCTATATGCTGACGCCGCCGAATAGTTCTGATCGGCCGTCAACCTCCAGGCAAAGGTATCCGG
CTGCTGTGACAGCTGCACCTCCTCCAAGATGTCACACAGGCAATCGTATTCCATCACGCAGGACAAAGGCGCCACTCCAAC
AAGTCCAAAAGTGACAGTGCAAAAGTCCAAACGAAATAATGCAAAAGGCGAGAGCTCGCCGGCAAAATGTACTGCCATCC
TGCAGGCGATGAAATGTTTACTCCACCACTAGTACTACTCCCAACCACCCTGAGTCTACTCGTGCAATGACCAGACGTGCTG
ATCTTGTTGACGAGGATTAGACGAGCCGA
>SsLRR-RLK201
GCCACTCGTAATCTTGATGTTTCGATAGGATAAAAAAAATCCTCCGGCGGCAACTTCCATCTTAACTCCGAATCTGTCAATAG
AGGCATCCCCTCGCAATACTGAGAAATAGAGCAAGCAATAGAGGTTCCTTTTTGTGAGTTCTAATTGTGAGTTAGGGTGCCG
GAGATGATAATCATGGTCGCTCGCCACCATCTGGGAGCTTCATCAGAATTAGATCTTCATCCACTACTTTATATCTAAGAATAA
TAAGAAAGAGAACCGACAAGGGAGATTCAGTTTTGTAGCACATGAAAAAAACGACTCCAGCTCCTTAAAAATGAACTGAG
AAACGAAGGACCCGGAGAAGCTAGGGTTTAGGTGCTCCAGCCGGCTCCGCCACATTGCTGCTACAGTGTAACTACGGTGCA
GGAGCTAGAGCCGGTCGGAGCCCCTCCAACCGGGCCCTAACTCTCACAACCAACGTGCGAACGCTCCAATGTTAACCGCTA
ATGCTAGGAAGCGGGCGCCCGTCCGCCCAGACGCTCCATTGGTGGACCATGACGCCGAGCCTAACAACTCTGCTCTTTCATT
TCTCAAAAAAAAAAGAAAAAAATTTTGCTCATCCAATCATTCCCATCCACTCGTTCCTATTGCTCCTATCTGCTCGCCTGCCACC
TCCAAGCCATGCCCGTGTCGCGGCATGAGGCCGAGCCCGCCAAGGCTCCTGCGATGTCGTCAAAGTCTAAGGCCAGCATATT
CTCCCGCATCAGCTTCCTCGGAGACGGCGCCAACCTCTCCGACGCCAAGCGTAGTCGTAGGGCCTCCGACAAGCCCCCCGC
ACACTCCTCTTCCTCCTCCATGAAAAGCGTTGTGGCAGAGAGTGGCGAGAACCGCGGCAACCGCCACCACCAACAAAACC
ACCGTGAGGCCACACCGATAGAGAAGCGTAGGCCTACTACCATCGCTACTGGCGACTACTATGGCTGCGCGGACGAAGAGG
AGAGCGAGGAAGAGGAGCAGCACTCGTCCTCCCGGCACGAGCGTGAGCAGGGCGGCGCGACCGCTGCTCCTGCTAGGCTC
GGGCTCATGGCCGCCTTCGAGGCGGCTGCTGGCCTTACTAGCATGTTGCAAGCGCCTGTTTCAAGTGTTTCAAATATTTCATT
TGTATGTTGCAAGTGTTTTCATACGGATGTTGCAAAAGTAGATCAGGATGTTGCACATGTTGCAATAGATGTACACGTATGTT
GCAAGCACCTATTCTCAATGTTTCATTTGTTTTTTCCGGCGTATGTTGTAAGTGTGTTTATCTGGATGTTGCATATGTTTTCACA
CATATGATGCAAGTGTTTTTATCTGGATGTTGCATATGTTTTACAATAACTTCAAGTGTTTTTCAGGTGTTTTTACAAGTGTTTC
AGATGTATGTTTCAAATGTTTCATTTGTTTTCAGACGTATGTTGAAATTGCTGCATCTGGATGTTTGAAAACTAGATCGGATGA
TGCATCTCCCTCCCCTTCTTTCTGCTGTCTCACCTCGGTGTCAGGCGCGGGAAGCGAGCGCAGGCGGAGGCGGTCCCCACT
GGCGCGGGCAGACCCTACGTGCTTGCAGGCGGGTGCAGCAGGCGAGACATGGGTGGGCAGACACGTGACACGGGATGACG
TGCAGCAGCGCGGACGTCCGGATGTCCAGGGTGGGTTTTTTCATCCCATGGTGCTCTTAATTGGGCCGTCCATGCGCGTGCT
GCGTGCAGCACTTAACTACGGCCCATCACAGAGGACAAACTACTACAAAGCATCTTCCGCGCCTTCCGAATTCTGACAAGG
AACAATTTGTTCCCTGTGCTCGTGGCCCGTTGGCCGACGTTGCGGAGTGCGGATTGTCCCCTTTCCTCTTCGACTTCGACTAT
TCCACCCCCAATCAGATCAGGACGCTGCCGCGGTCCGCAGGTCGTCTCCTACAAAACCACAAGAGAAAACACGGCCGGGC
ATCACGCGCAAAAGCGAGACTACCTGGAGCTAT
>SsLRR-RLK247
CGAGATAAGGCTGCGGAAGTGTAGCGGAACGGAACCGCTCAAAGATACCATGAAAGTGGAGAATAAATCAATCGTATTCCC
ACTCAATGCATGAGTACATATGGCCGAGGGTGGCTCACAAGCCAAACCGCACAGGCGTGTACAAGCCACTAATTAAGGGCA
GCCTACTATAATTACACTTACTAAAAGGCTAACACCAGAGATGACAATCATAGTCGCTCGCCACCATCTGGGAGCTTCATCAG
AATTAGATCTTCGTCCACTACTTTATATCTAAGAATAATAAGAGAGAGAACCGACAAGGGAGATTCAGTTTTGTAGCACATGA
AAAAAACGACTCCAGCTCCTTAAAAATGAACTAAGAAACGAAGGACCCGGAGAAGCTAGGGTTTAGGTGCTCCAGCCGGC
TCCGCCACATTGCTGCTACAGTGTAGCTACGGTGCAGGAGCTAGAGCCGGTCGGAGCCCCTCCAATCGGGCCCTAACTCTCA
CAACCAACGTGCGAACGCTCCAATGTTAACCGCTAATGCTGGGAAGCGGGCGCCCGTCCGCCCAGACGCTCCATTGGTGGA
CCACGACGCCGAGCCTAACAACTCTGCTCTTTCATTTCTCAAAAAAAAAGAAAAAAATTTGCTCATCCAATCATTCCCATCC
ACTCGTTCCTATTGCTCCTATCTGCTCGCCTGCCACCTCCGAGCCATGCCCGTGTCGCGGCATGAGGCCGAGCCCGCCAAGG

CTCCTGCGATGTCGTCAAAGTCTAAGGCTAGCGTATTCTCCCGCATCAGCTTCCTCGGAGACGGCGCCAACCTCTCCGATGC
CAAGCGTAGTCGTAGGGCCTCCGACAAGCCCCCCGCACACTCATCTTCCTCCTCCATGAAAAGCGTTGTGGCAAAGGGTGG
CGACGGCCGCGGCAACCGCCACCACCTGCAAAACCACCGTGAGGCCACGCCGATAGAGGAGAAGCGTAGGCCTACTACCG
TCGCTACGGGCGACTACTATGGCGGCGCGGACGAAGAGGAGAGCGAGGAAGAGGAGCAGCACTCGTCCTCGCGGCACGAG
CGTGAGCAGGGCGACGCGACCGCTGCTCCTGCTAGGCTCAGGCTCGCGGCCGCCTTCGAGGCGGCTGCTGGCCTTGCTAGC
ATGTTGCAAGCGCCTGTTTCAAGTGTTTCAAATATTTCAGATGTATGTTGCAAGTGTTTTCATACGGATGTTGCAAAAGTAGA
TCAGGATGTTGCACATGTTGCAATAGATGTACACGTATGTTGCAAGCACATATTCTCAATGTTTCATTTGTTTTTTCCGGCGTA
TGTTGCAAGTGTGTTTATCTGGATGTTGCATATGTTTTCACACATATGATGCAAGTGTTTTTATCTGGATGTTGCATATGTTTTA
CAATAACTTCAAGTGTTTTCAGGTGTTTTTACAAGTGTTTTCAGATGCATATTTCAAATGTTTCATTTGTTTTCAAACGTATGTT
GCAATTGCTGCATCTGAATGTTTGAAAACTAGATTATGTGTTGCATCTCCCTCCCCTTGAAGCGAGCGCAGGCGGAGGCGGT
CTCCACTGGCGTGGGCAGACCCTACGTGCTTGCAGGCGGGTGCAGCAGGCGAGACATGGGTGGGTAGACACGAGACACGG
GACGACGTGCAGCAGCGCGGACGTCCGGATGTCCGGGGTGGGTTTTTTATCCCATGGTGCTCTTAATTGGGCCGTCCATGCG
CGTGCTGCGTGCAGCACTTAACTACGGCCCATCACAGAGGACAAACTACTACAAAGCATCTTCCGCGCCTTCCGAATTCTGA
CAAGGAACAATTTGTTCCCTGTGCTCGTGGCCCGTTGGCCGACGTTGCGGAGTGCGGATTGTCCCCTTTCCTCTTCGACTTC
GACTATTCCACCCCAATCAGATCAGGACGCTGCCGCGGTCCGCAGGTCGTCTCCTACAAAACCACAAGAGAAAACACGGCC
GGGCATCACGCGCAAAAGCGAGACTACCTGGAGCT
>SsLRR-RLK402
CATAGTCAAATCCAACCGTTACTTGCCAACTCAGCAAAAAACGAGGTGACCGGACGCGCCGGTCAAAGTGACCGGACGCG
CCGGTCGCTCGTAACGGTCGAAAACCTGAAATAACGGCTGAGTGACCGGACGCGCCGATCAGGGGTGATCGGACGCGTCC
GATCGTGCAGGGAGCTTACTATACTCTAACCTGACACGCGACCGCGTCCGATCAGTGATGACCGGACGCGTCCGGTCGTCAG
AGTACCTTACTGGAACATGACCGGACGCGCCGATCACACCCAAGTCGCGTCTGATTAGGTGATTTCCCTCAAGTGACACGTG
TCAGCCTGAACACCGCGTCCGGTCAACAGCGCTGAGACACAGTAGCGGTTTAGAACTGACCGGACGCGTCCGGTCAAGCC
AGACACCGCGTCCGGTCAAGGGAAAACGTCTCTAAACACTCCGAAACTCGACGCAACTTGAACCAAGTTGCTTCCAACTG
AACTTTGAGCTATTCTAGAGCTACCTAGTGCTAAGTTTGACAAGTGTGCACCACACCTAAACACAAGACTCACCTAGGTCAA
GCTACTCGCTCATGCCCCCCTTTATAGTACGGCCAAAGGAAAAGCGAAGATCCTAACGACTATTCCGAATGTAACTCAACAC
CTTGACATCCGGAACTAACCGTCCTTAATCTTGACGTCAATCCTTTGAAAACCGAAACGATTTCCATCAAGGGGTATGAATAC
CATAGTGCCCAAAGCAGTTATCAACCCCATGACCTAACTTACCAAAGTTTCTGCAAAACACACGTTAGTCATGGTAATAAAG
CGTTGTCATCAATTGCCGAAACCAACTCTCGGGGCCTAGATGCTTTCATCGATAATACACAAATTTAGTGTTTCGTTTGAGGA
TGAATAGGATGATCTTTAGCTTTCTAACTCATGAATACTGAGTTTGGTTTGAGACTAGAATGTGTTGATCAAACGGTACATCAT
ATGCAAACCTCATATGCACATGCTTATATTAATACATAAACACGAAGGATGTAGTCATCCCAACAAAAATAATAAAATCAACTT
ATCATATACTACTTCATTTACGATGAACTGATTTCTCAAACAAGCACTATAAACCTGAGGAATTGTTTATATGGCATCTTAGTCC
TCTCCTAGTGCCGGTGTCCACCACCTCTACACTGACCCCATGCCTGCTTCTCCGAGCAGACATGAGGACGTCAACACCCCTT
AGGACTAATTTGGAAGCAAGGGGGAGGCACACCTAAGGATGAAAAACCCTCCATGAAGAACTAAATAATTATTCAAATTATT
TTTCCATGAATTTCCCACCCTAGAAAAAGTCAGGATTCCAAGGAGATTTGAGTTTCCATAGTAGCCCTTAGGGTCACATCTAC
ATCTCCAGCTAATTAAGTGATTGAGTGATAATTTAGTTGATTAGGTGTTCACGGGTTAGGTTAATCTTGATCTTAGTTAAGCAT
AAGTTTAATGGTGTTATTGTTATATGAGATTTTTCCTTTCGCATTGCACATATTGGTTGAACTGATTATTTTACAATGCATTCTGC
ATGGTATGCAACACAAGCCAACATATGGAAGTTGAGTGTATTTGGTCCAATAACAGGGCCCTTGTAGATTGCAGGTTCATTGG
GGAATTCAGTTTTGAAATTAGGGGAGGTTTTGGTTACATCTAGTGATGTATTTTTTTTCCTTGAGCATGATACACCTATCAATC
TATCCTCGATATTCCATTATAGCCGTATTTTAGATGTTTGATTAGTGAAGCTAGTAAACCGAAAAGAGGGAACCCTGGACAAG
TAGGAACACCAGAACCATCTATATACGCTGACTGACCCCTAGAATGGGAAACCAGCAACTCCTCAGTTTACTTTCCCTAGTG
TCACCAGCTTCGCTTGTATTAAACCATCTCATCCCATCCTTCCTTCCTCTAGTGTAGCTGCACTATTCCAATCTGTTCCGTCCGT
ACTCCCCCAGCTTCCCC
>SsLRR-RLK425
AACTATGGCTTATTTGGGCATACATTCGTTTTCAATAATAGTGGTGCAAAGGATATGTGTGAGGATATTAAGGAATAACTAATT
CTAAATTTTCTTTTTTTTAGATAATGGAAAATATCCGGCTTCAGCCTTCTTCTGAGAAGAAGAAGCATCAGTCCAAAGTTATTA
CAGTAGCACAACTAAAGTTTAAGACGGTAAATAAAGGACCATCCATGCGATGCGAAGAACTCCATGGCTCTCGATTCTAGTA
GCTTGCAAGCGTTAAGAATCTGTTCCTTGTCGTCATCCGAGCGCTGCAACAGAGCCCAAAATCGTAGCCAGTGTGTTCCCCT
GAAAAGAACCTGATAATGGTGTGAAGACATTAAGATTGGAAAGTAGCAGTTTTACCTATTTTGAGACTAGAGCATAGATGTG
GTGCACACTTATAAAAACTAGACGGCTTGATTGAGGTCCTAATAAGATGCAAAGAAATGAAGCTAGATACAAAAGATGTTGA
ATTGGAATATCCCTGAAGTTTTGTTATCACCACATCCTTAGGTGATAACACTAGATGCTTTCACATGATCACTTGCCACGCCAC
TTGAAGCTTTTAATGTTCCTAGAATTCTAGCTCCAAGTATTTCTTGAGCTAGAGACAGATAGCACCAAAAATTCCTAAAGGTT
AAGCTATGTCAAAAGGACCCTTACAAAGGAGGTAAGAACAAATGGAGAGAAGACTAGGAAAATCAAGCCAGTCAATGAAT
AGGTGGATGGCTCAGGGGAGAGCTTGCAAATACACAAGAACTCCCTGAAGTTTCTATTGCAAAACCAAATGGTCTTTTAATT
GTTGGCACGTTGTTAGAGGCCGGACTTGAAAATACACAAATTTAGTGTTTCGTTTGAGGATGAATAGGATAATCTTTAGCTTT
CTAACTCACGAATACTGAGTTTGGTTTGAGACTGGAATGTGTTGATCAAACGGTACATCATATGCAAACCTCATATGCACATG
CTTATATTAATTAATACATAAACACGAAGGATGTAGTCATCCCAACAAAAATAATAAAATCAACTTATCATATACTACTTCATTT
ATGATGAACTGATTTCTCAAACAAGCACTATAAACCCGAGGAATAGTTTATATGGCATCTTAGTCCTCTCCTAGTGCCGGTGT
CCACCACCACCTCTACACTGACCCCGTGCCTGCTTCTCCGAGCAGACATGAGGACGTCAGCATCCCTTAGGGCTAATTTGGA
AGCAAGAGGAGACACACCTAGGGATGGAAAACCCTTCATGAAGAACTAAATAATTATTCAAATTATTTTTCCATGAATTTCCC
ACCCTGGAAAAAGTCAGGATTCCAAGGAGATTTGAGTTTCCATAGTAGCCCTTAGGGTCACATCTACATCTCCAGCTAATTAA
GTGATTGAGTGATACTTTAGTTGATTAGGTGTTCACGGGTTAGGTTAATCTTGATCTTAGTTAAGCATAAGTTTAATGGTGTTAT
TGCTATATGAGATTTTTCTTTCACATTGCACATATTGGTTGAACTGATTATTTTACAATACATTCTGCATGGTATGTAACACAA
GCCAACATATGGAAGTTGAGTGTATTTGGTCCAATAACAGGGCCCTTGTAGATTGCAGGTTCATTGGGGAATTCAGTTTTGAA
ATTAGGGGAGGTTTTGGTTACATCTAGTGATGTATTTTTTTTCTTGAGCATGATACACCTATCAATCTATCCAAGATATTCTATT
ATAGCCGTATTTTAGATGTTTGATTAGTGAAGCTAGTAAACCGAAAAGACGGAACCCTGGACAAGTAGGAACACCAGAACC
ATCTATATACGCTGACTGACTACTCCCCTAGAATGGGAAACCAGCAACTCCTCAGTTTACTTTCCCTAGTGTCACCAGCTTCG
CTTGTATTAAACCATCTCATCCCATCCTTCCTTCCTCTAGTGTAGCTGCACTATTCCAATCTGTTCCGTCCGTACTCCCCCAGCT
TCCCC
>SsLRR-RLK413
GTTATCACCACATTAGGTGATAACACTAGATGCTTTCACATGATCACTTGCCACGCCACTTGAAGCTTTTAATGTTCCTAGAAT
TCTAGCTCCAAGTATTTCTAGAGCTAGAGACAGATAGCACCAAAAATTCCTAAAGGTTAAGCTATGTCAAAAGGACCCTTA
CAAAGGAGGTAAGAACAAATGGAGAGAAGACTAGGAAAATCAAGCCAGTCAATGAATAGGTGGATGGCTCAGGGGAGAGC
TCACAAATCCACAAGAACTCCCTGAAGTTTCTATTGCAAAACTTAATTGTTGGCACGTTGGTAGAGGCCGAACTCGATAATA
CACAAATTTAGTGTTTCGTTTGAGGATGAATAGGATAATCTTTAGCTTTCTAACTCATGAATACTGAGTTTGGTTTGAGACTGG
AATGTGTTGATCAAACGGTACATCATATGCAAACCTCATATGCACATGCTTATATTTATACATAAACACGAAGGATGTAGTCAT
CCCAACAAAAATAATAAAATCAACTTATCATATAGTACTTCATTTACGATGAACTGATTTCTCAAACAAGCACTATAAACCTGA
GGAATTGTTTATATGGCATCTTAGTCCTCTCCTAGTGCCGGTGTCCACCACCACCTCTACACTGACCCCATGCCTGCTTCTCCG
AGCAGACATGAGGACGTCAGCACCCCTTAGGGCTAATTTGGAAGCAAGGGGGAGGCACACCTAGGGATGGAAAACCCTCC
ATGAAGAACTAAATAATTATTCAAATTATTTTTCCATGGATTTCCCACCCTGGAAAAAGTTAGGATCCGAAGGAGATTTGAGT
TTCCATAATAGCCCTTAGGGTCACATCTACATCTCCAGCTAATTAAGTGATTGAGTGATAATTTAGTTGATTAGGTGTTCACGG

GTTAGGTTAATCTTGATCTTAGTTAAGCATAAGTTTAATGGTGTTATTGTTATATGAGATTTTTCCTTTCGCATTGCACATATTGG
TTGAACTGATTATTTTACAATACATTTTGCATGGTATGCAACACAAGCCAACATATGGAAGTTGAGTGTATTTGGTCCAATAAC
AGGGCCCTTGTAGATTGCAGGTTCATTGGGGAATTCAGTTTTGAAATTAGGGGAGGTTTTGGTTACATCTAGTGATGTATTTTT
TTTTCTTTGAGCATGATACACCTATCAATCTATCCAAGATATTCTATTATAGCCGTATTTTAGATGTTTGATTAGTGAGGCTATCT
GCACTCATGGACCTCTATATTCATCCTCTATACGTCCTAGTCAGCAAGATTCTCTCCTCTATACTCAATTCTTCCGCATCCATCC
ATCCTCTATATCATCCTCTATCCACTGTGGGACCCACTTGTCATTTTATCTAAATATATCCATGTGCGATCTAGTTTTGAAGGATT
TATTGAGACGAATTTAATGGTGCAATCGAAATTTAATTTGGATTTTTGGTTTGTGAGTTATAACATTTTTTATACGAAAAAAAA
TTGACACGCTTTTCTATGCACCGCTGTTTGTTCTACTGCCTTCACGCGCGCGCGTCCGTCGGTGTCGTCCCGTACTTTAGCGG
ATGAACAGTAAAACGCTATATATAGAGGACCGCGTTGCTTCCTGGGAGATAGAGTAGTATTTACAGGGTACCGGTGCGGTGG
TTTTTTACTCTATCCGATCCTGCAGGAGACGTAGCGTACGTGAGTGCGGTCGGTCTGAAGCTAGTAAACCGAAAAGAGGGA
ACCCTGGACAAGTAGGAACACCAGAACCATCTATACGCTGACTGACCCCTAGAATGGGAAACCAGCAACTTTCCCTAGTGT
CACCAGCTTCGCTTGTATTAAACCATCTCATCCCATCCTTCCTTCCTCTAGTGTAGCTGCACTATTCCAATCTGTTCCGTCCGTA
CTCCCCCAGCTTCCCCTTCAATGCCACCCCAGCTCGAATCTCCTCCCTGACGTGCAGCTCTCCACACGTCGCGCTTCTCCGA
>SsLRR-RLK266
ATCTAGAGAAACCAGAAAAATATGTTTCGCTTGATAATCGGTATCATTTTTTCTCAACAAACGTTTGTCTAAGGAACCAAATA
CATGCCATAATGTCCGAAAATAGAGAAATTTTTCTATATGACACTAGGAAAACTCGTGTTTTTCTATTTGAAATCCAAACTCGA
AATTCGTCCTGTTTGTCATCCTTTTTTTTTCTATTTGACACTTTTGTCAGTTTATTAGAGCTCTAACGATGTTAAGTCATAGACA
AAAAGACTGTTTTGCCCCTATTATTTATTTCCAGTCACGGTATTTACTTTTGCTGGCTATTGATGTTTTTTGTTGCATTTTTATGT
TTTCTCAAATAAAAAAGCTCAAAACTAAAAAGTTGTTGAACTCATCGAGACATATAACTTTAATATAAAAAGCTATCTTTATC
CGACACCATACAAGAAATATACGGTTCCTAAGAACACAAGCCCGGGTATGCGATAGATATGTTGCTAAAAATTTGTATTATTTT
TTGAGCATCTTAATGGCCTCAAATAAAAAACTTAAAATTAAAAAATCGAGAGCTGACATTTTTACTTAAAAAGTATCTTAACA
CGACGCTATACAAAAAAGATATAATTTTTATAAGGCAACAAGCCCTGGTACGTGATTAATATGCTACTCAAACTTTATATTATTC
GTTTGAGCATCTTAACGATCTCAAATAAAAAAACAAAATAAGAAATTTGTAGATCTCATCGAGAGCTAAAAGTTTTGTATAAA
ATCATCTTCGTCCGACATCATATGAAAAAGATAATTTTTCCGAAGCAGAGCATTTTAGTCTATATGCACAAGGGCATTTTTGTC
ATTGGCCTACAGGGAGATTTAACACTAACATTGTTGACAAACGGAGTTGACGGAAGTGTCAAATAGGACAAAAATTAAAAG
AAGTGATGTATAGGAAAGCTTTGAAGTTTAGGTGTTAAATAGGAAAGTAGAAAATTTTCTAGTATTAAATAGAAATTTCTCCC
TAAAAAATGAGTTGGATTCCCTTTTTTTTAAGGAAAAGTTGGATTCCCATTTAGGCTGTGTTTAGTTTCCATGAAATGTAAAAT
GCCAACCACCTTGGAATGATAAAATGCAAAATGCCAAAATTTTTAAGGGTGTGTTTAGTTACATTTAAAATGCCAAATTTTTC
ATGAGGTGGGTGATGACTTTTTGACTTTTTAGCTTTTTTTACCTCTTGAGACCAAAGTCTAAAGTGGAGAATAAATACCTTTT
TATCAAAAATTTTATATGATATTTAGTTTCATTCTATAAAATAACAAGCTAAAACCCAAAACTTTGTTGGTGAAACGAGGAACT
AAAACCCTTGTTCACCTCACTCGGGCGACCTAGCCACCCGTGGCCTGGCAGCCTGCACGCCCCCAGACGATGGTCCGGCCC
TGTTTACTTCCCATGCAAACCTAAAATATTTCTCTTTCACTCATCACATTAAATCTTTAAACGCATGCATGAAGCATTAAATGT
AGGTAAAAAAATAACTAATTGCATAGTTTGTCTGTAAAGTTTGAGACGAATCTTTTAAGTCTAATTAATCCATGGTTGGACAA
ATTTTACTAAATAACAACGAAAATGCTATAGTAACTTTTCACCCAATATTTTCCTCAACTAAACACACCCCGGTCTCACCCAG
CCCAATCAGGTAACCATTCCGTTTCCATCGCGCCGTCCCCGTGCCCCGCTGGCCGCTGCTCCGCCATCTCGGCGACCCCACT
GCGCAGTCCACGCACGACCCGTCCCACCCCAGACCAACCCCACTAGTAGAGCCCAGCGCGCAGCGCACACGCCGCACCAG
TATACTACTACTGCTCCGTCCCTCGCTGCCCCTGCCTGCTCCCCTGCCCTCCCCATCCACTCCACCACCACCACCCCCGGCCG
CAACAAGCACGCAGTCTCCCACCTGGTCAAACCGCTGCCCTTCCCCACCACCACCGCGCCCCAATCCGCGTGCGTCCGTCC
GCCA
>SsLRR-RLK292
GAGAGTCCCTAGAAACGAACTTCAATGCTAGCAGAGCACAAGCCGCCTCATCAAAAAAAAAGAAAAAAGAAAAAAAAAG
AGCACAAGCCGCACTCGCGCCGTAGTGCCACCGTGAGAAGTGTGGGAGACGGGGGACCTTGGGTCTGCTCTGGCCTCTGG
CTCTGCAGACACCGCCAGATATGAACAGAGCGCCTTTTGTGCAAGGCAGGCCGTGTGCACGTATTGGAGACTACTCCCTGTG
CCGAATACTGATATGTAATAAACACCTGTTCGGCTTAGGGCCTGTTAAGTTCCCGAAAATTTTTGGGAAATGCTACTGTAGCA
ATTTTGTTGTTATTTGACAAATATTATCCAATCATGAACTAACTAGGCTTAAAAGATTCATCTCGTGGATTTCGTCTAAACTGTG
TAATTAGTTTTATTTTTTTATCTATATTTAATGCTTCATGTGTCTAAAGATTCGATGTGATGGGGAATCTGAAAAATTTTGCAAA
ATTTTTTGCAAACTAAACCAGCCCTTATCAGCACAGCTTATCAGCCGTGGTACAATATTTTTCTTTCATAACAAAACAGCCAC
AGCCGGCTTATAAGCCGCAGAAACAGTCTACCGAACAGGGTGAAATATTTTTGTGTGATTTTGCTAGAACAACTTCAGGGTG
GATTTAGTTAGGTGAAAAGCTTTTGAAAAAAAAATACCACATCGGATGTTCCATGCGATGTCGGAAAGAGTTTTCGGATACTA
ATAAAAAAACTAATTACATAGCTGGCTTGAAAACCACGAGACAAATTTATTAAGCCTAATTAATCCGTTATTAGCACATTTGTA
CTGTAGCACTTTAGTGTCAAATCATGGATTAATTAGGCTCAAAACGTTCGTCTCGCGATTTCTAACCAAACTGTGCAATTAGT
TTTTTCGTTTACATTTAATGCTCCATGCATGTGCCGCAAGATTTGTTACTGTAGTTTTTTTTGTGAAAAGTTTTTGGATCTATAC
ACACCCTCAATTAGAGAAATGAGCATATGTTGATGTAAGCACCGCATATCGATTCGTTAGAGCATCTCCAAGAGTTTGCCAAA
TCAATTTGGCATCTATCTATTTTTGGTAAAACAGGAAAAATTGTGCTCCAACAATTTGACATCCAACTTGGTAAATTTTAGCA
ACTTGGTAAATCTCCCCCCTCCACGCGCATATTTGCGCGCTGAGCGCGAGCGCGCATCCGGCTTTTTGTGTGGGGCTCTTCGT
TTCTTAACGGGACTCTTCGTTCGCTTAGCGGGGCTCTTCGTTTCTTAGCGGGGCTCTTCGTTCGCTTAGCGGGCCTCTTCTTAT
TTTACCAAGTCCAAATGGCAAACTCTTGGAGATAGTCTATTTTTTCACTTACCATATATTTTAGCAAGTTGTCAAATCATGATAT
TTGCCAAGTGAATTTTAGTAAACTCTCGGAGATGCTCTTACTTGATTCACCTAGCATTCTTAGTTGGAGAATGGCATTTTCACT
AAATGTTTCCATAATCAATCGTTCCATAACTTCATAAGGGCTACGACATTCCACATGACACAACTTTGTATTCCCTCAATAGCA
ATTGTAAGACGTTTTGTCTTTTCTAGATACACATAGTATTTGTTATACACTTAGATATATATTATGTCTAAATTCATAATAAAAAC
AATGTATCTAAAAAAGTAAAAATATCTTACAATTTGGAACGGGAAGACTACTAAGTAATTTTATTAGTAAACTGTTTCAATATT
TCAAGAAGTTCCTAATGCCACCAGGGACCAATGTTTTCCTTAGAAATAAAATTTAATTAAAGAAATAAATCTCGAAGGTGAA
AGGATCAAAGGTGTCGGCTGATTAGTCGCTATTGGACCCCCAATTTCTGATCCTAGCTAGGAAACGAGGAAGTGACTGATAT
ACCAAATTAGCAAATGAAGTAGACATACGAGTATCCTTGGTTTTTATATTATATGGAAAAAAGTTTTATTGATGGGTATACTTG
GCC
>SsLRR-RLK385
TTTGGCTTTGATTTTTGAGAGTTTTTTTAAATAGCAGGAGGCTCTAACTGTAGTAAGGATTGGTTATAGGCAAAACTCTTGC
TGAGATCAGAATAGACTTCCTTTTCCAAAAAAAAAAAGATTTGTTGTGTGTTCTTGAGGAACACAGTAAGGACTACTTTGGA
TGTTATAGTTTTGGAACCTCGAGACGAGCAAAATGATGGTTTCCGGAAAAAATAGTTGAAGGTGGAGTTTCTAAAACTCAAAA
ATAATACCCCTTGAGTTTTTTTACAAAGTCATGGTTTTCAAAAAATTCTAAAAATATTTTGCATCCAAACATAATCTAAGTGTAT
GCTTATATGCACTAGGCTCTGTTTAGTTCCGCGTCGGCGGCACGTTTTAGCCGAAAACACGAAAAAATCCCGCCGAAGGGTC
TACGGTGGACCCGCGTGGGACGACCACGCACGGCATGAAACGCTGCGCGCTCGAGCCGTGTTCGCTTATTGTGGTCGCATG
ACCGGCTGTCGCGGAACCAAACAGGCCCGTAGTGGAGTCATTATGAGCGTCTAGATATGGATACTTGGTTTTCCTATTTGAGA
TAAGATCACCATTTATTGATATTATTAGATAAACAATTTATAGGAGGTGAATATTTACTCTACGACGTCTTATCCAATCCACACT
GCCATCCTGGTCCGTCCGATCATCCAGAGGAGAGAGAAAAGAAAAGCGAAGGGTGGTGCAGTGCCTGAGCAGGCGGCCGG
GTAGACTGTTAGAGTTGACCGGAAAGTCCTGGGTTTCCGCGTGCCGGCCTCACCTCACTCAGCACACCACCGGCTCTCGCG
GGCCCCACTGGGCCCCCACACGCTTTGAAAACTCCCGTCTCGCTTTCTAATTCCACACTGGACCTGCCTGCCTGCCCTGCTC
GCTCGCTCGCTCAAGTTCTCCCTCCGACTCCGAGACTCCGAGTCCTCTCCCCTCCCTGAACCTGCAGCTTCGTCGTCTCTCA
CCCCGCCGGCGCTCCCTCCCCTGGTCCGCCCGGCTACAGGTTCCCTCCAGGGCGCGGGAAGCAGCTCCCGGTGAGCACCCA
TGGAACCCGTCCCTCCCACGCTCCCGTCCTCTCATCTTTTTTGTTTGCACTACCTATTCTTGTGGTTTAATTATTATATCGTTCT

TGCAAAATCTGCCATGGTCCGGTACGATCTTGCATGCTCTGGTCTGGGCGAGGGTTCTTAAATTCGAGAGGACCTGTCAGCC
GTGGCAAGGAAAGCGATCGATTTTGTGTTTTCGCAAAGTACTGTGCTGGAATTCGGGAGAGAAACCCTGTGCATGGGTGTT
CGTTCTAGCGGGTTTGTGTCCTCAGGAGGAAGGGTTTGCGACAATCGAAAGGTTCTTTTTTTTTTAAAGAACGACCCGGCGT
GTGAAATCAGAGAGAGACGGAGGAGCATGGGCTCTTATGGTGCCCGCTTTCGGGAGATCTTTTTCCTTAGATTTTTTTTTCTT
CTGAAAAGTTGTGCGCCTTTTTTTTGCGGTTGGAGCCGTTTATGTTGGGCATGAGAGATCAATCAAGAGCTATAATAGATATA
ATCGAGGATTTGTTTATGATCTGCCTCTGGTTTTTTTTTTGTTGCTTTCCATGCATATGGCTGCACCTCTTCTTTGTTGCAAAAA
CCTTAACAAAAGTTGCCGACTCGTGTTATTTGCTTGCCTCTAGTGGGGATCGGTTCTTTGACTGACGTTGCCTGTGTGTGGTT
CACAGTATTTGGCGTTGTCGATATCACATTTGAATCGAATTTTGCATTTTAGGTAGTGGAAGAGAGAAGTTGCATAGCGACTT
TCAGGTCCCCATATGGTTGCTGTCACCATTTACCAATTAGTTTATATCTTGTTTATTTTTTTTTTTTGAAAGTCCAGCGTCTCTA
ATTCTGTGCGTTTGAATTTGATTTTGTGTAGGTGAAGCTTGAATCGAGAAGTTGTGTTAACATCATCAGAGAGAAGGATTAGT
TCACTAAAGTGCTCGA
>SsLRR-RLK296
CAGAAGGAGATCGTCGAGTCCCACTCCATAACGTGCCCGATATAGAAATAAGAGTGTGGGAGACCTTAACGTTTCCACTCCA
CCATCTTTGCCATTTCTAACTTCGTCTTCCACTCATAATCCACAGTAAACCCCAACTCCTCCATTAGTTCCTGCACTCTTGCTC
GTTTGCTGCCTTAGTCCCCAAGGCGAGCACATCCTCCATTCTTGCATCCAACAAAGTAGATGGGGGAAGGGGAAGTGCGCC
ACACTCAAGGCAGAGGGGCTCACCGTCGACGATGATGGAGGTTCTCGATGTGACAGGTTTCTAGGAACCGTTGAATGTGAA
GGAGGTCGATCTCTGCGAAGGGTTGGGCTAGGACCACGAGAGCTCATGGGATGGAGGCCTGACACGAGGGAGAGCTTCCC
TACCTCCATACATCGGCGAGGTAGTTGTCTTCGTCTCCTTCTTTGAAGGGATTCTTGGTGCTGAATAGTGTCCTACGAGGCCT
AGGTCCATCATCTAAACTGAATAGTGTCCTACATATTTCAGTCTTCATTTCTCTCTATGAGAATTACCAAGGTTTTGAGCCGCT
TTTCACGTTTTTGGAAGGAGTTAATCGGGGGATGACTCCAAAGCTACCATAGGACTCCTTTACTAGTGGGGCTTGAACTTCT
CCGGCGCAAGAGTACCTACTTCAACACCCCCTTTATATCTTCCAATACGGGATGGCACAAGCATTGGTTCTACATCTTGGGAG
AGATTGACGAGCTACAGTCCTATGACATGACAGGTGAAAGCCTGATTGGAGTCTTAATTCGTCGCCGAGTGCAGCTGATCAA
GGATCATGTGCATCGATGTATCATTAAAAGGGCTAGCAGGATCTGACTAGAGAGTTGAATTAGGAATTTTTTGTGTGACTTAG
TCGAATTAGGAGTTGGGAGCACATGAAGCGAACGCGTGGAAGCTCCCAACTCCTAGTCAAATTAGGAGTTGGGAGCACATG
AAGTGAACGCGTGGAAGCTCAACAAGGATGAAGAGTCTAATATCTTATTCATCAAATACTATTACTTTTAGTAAAGCCCGCTA
GAACTCGAATGCTTTTAAAGAGTACAATACACCAAAACTTTAAGATTTTTTTTTCATTTCTAAATATTATTATTGGTATAAATAT
AGATACATTCCTAGTACATATAGTGGGTGTTTGTAGGATGGAGTAGTCTGTCATTGTCTGATTCTTTAGTTTTTTGTCTGGTTTG
TAAAATAGAATAAATTAAAATTGATCCATTATGACCTTGTTCCTTATAAGCTAATAATTAGTATTACTACACCCTCCATCCCAAA
ATTATTTGACGTTTTAGGTTTGCACACGAAGATTTAAAAAGATTGAAAAGGTGTGGCGCACAGAAAATAAAATGGAGAGATA
TAGAGAAATATAGAGATACAAATGTATTGAAAAAGGTATATTAGGAAAAGAGTGACAACTATTTTAAAACAAAATTTAAATTT
TGTAATGTCAATTATTTTAGGACAAACGGACTAGTATAAAATACCCTATCCGTCTCAAAATAAGTGTCACTATAGGATGCGTGC
TAGTTAAATTTTTCTAAGTTTGGCTAGGTTTATGGACAATATTAGTGACATTTATATCTCCAAATAAGTTTATTACAAAATTATAT
TCAACGATTCGTCTAATCATACTTATATATATATATATTTGGACAAAGTTAAAAATGTTTGACTTCTTGGTAAGTTAAACTAACA
CTTATTTTGGCGGAGAGTGTGCGACTATTTTACTAAATTTCAGAAATAAATTTATGACGCATCAGCCGATTAGAATGAGTTCTA
ATCTAAACACTCCAATGCAGTGCTGCGTGGGACCGTTTGTGTCACACATCCTCATCAGCCGATAAACGACCCGTTTGTGTGA
GATGGACAGTGTTGTCAAATCAATCTCGCAATCAGCACAAACCGTCTGACAAAAGTACCAAACTGCCCCTCTCCCGCTCAC
AGAGTCACA
>SsLRR-RLK304
TAGTTTCTATGAACATTAATTATACTGCGACCTAAGTGTTTTGCTGACATGGCAATGTAGTTATTGAAGAGAGCAAAAATCATA
GAAACCGGGTCTAAGTTAGAAACCATGTCTACACGAGAACCAAGATATGAAAGGATGTAATTGGTAGAGAATGAAGAGAGA
ATACATGTGATTGGATAAAAAATTGTTCTGTAAAAACTATCCATTGGGACCATGACTTTTATATGTAGTGTCTATGAAAATGAA
TAGGTATTCTAGCATTGGAACTGCCTTTACAAGTTCAAGGGCCTCTCATGCATTTAACTCAAAAAAAACAATCCAGAGTACA
AACATTTTTTCCCCATCATGTGGATATGACGCGAGACAATACGGTTGAAGTGAAGTAATAATTTTACACCTGAATAATTATGAC
TTGGCCTATGTCTCGAAGGCAGCTTGCACAAAATTGCAGGTCAGTTAGTATAAAAATAAGAAGCATAACCAAACAGTAAAAT
TGTACCCATTCATATGCAAGTTGTACCTCTTCTTCAACTGAAGATAGAACTGGGCAAGATCAGTTGCTTGTTTCCTGCTTATAT
TCATTCTTTCCCATTGGATGAGCTCAGGACAATGGTCTCTGTAGTCCAAGTCCAAGATGCTGATGAAAATGGATGGGCATCAT
ATTTTCCATCAGTGGAAAAAAAAAATCTAGAACGATTTTTCCGTTAGTGTCAAATCTAAAACAAAAGTAAACAAAAATGCCAT
GTCGCTAGCCTAGGAGGGACACCAATGGTCAGCAGAATTTTTTGAATGCTCGATTGTAACTGGGAGGAGAGAAGGTAGAAC
CAGAAGAAAGGAAGAAGAACAGGAAAAAGGCGCCTGACTTTTTTTTTCAGAGGATGTTTTGACATACTAACACAGAGCGC
TGCGGACAGTCGCATTGGGCTCAGTTGGCACGGATCGGCCACAAGAACACTGAGATGGACGCGCACATCCAAGGCACCATC
TTCTTCACAATCTGTAGAGTTGAAAGGAATGCATGATAGATCGGACACTTCAAGCAAACTTGGCATGACCTGATGAACTCAG
CCGGTTGGAGGCGTCGACGTCCACGGCCACATCCCCACCGGAGGCTGGAGCTGCAGCTGCCTGCTGCCTTCATGGCTCCAA
TATTGAGCGACTGAACCGCCTGCCACCTTCGGCTGCACCGGGGACGGGAATGCCACAGGGCGCGATTGGGCGAGGATGGG
AAAGGGGCGACGCATGCGGGAAAGCGGGAGGAGGAGTTGCGCACCGGATCTCTGACGATGGTAGCGGATAGGGGCTCGAC
GTCCTCCAGAAGCACGCCTCGCTCCCGGACCTGTCTGACCCTCCAGGCTCCAGCACCTACCAGGGGGAGGGGGCGCAGATC
CAGGCCGCCCCGACCTGCTGGTCGCCATGGTTGGCTGCCTGGCCGTCGCCGCCGCCGCCGCCGGCATGGCTGGCCGCCGCA
GGGACGCGCGCGTGCGCGCGTAGGCACGCCCGGCTGCCGGCTTGCGCGCCCCCAACCATGGAGCCGGCCAGGGGAGCCAC
GGGGCTGGCCACGGCGGAGGACCGGGCGGTGTAACACACGGTACCGCGGCAGAACGAAGGTGGCGTACTCAGGGAAAGG
CGTCTTCGAAATCATTAGTCCGGTGCTTGTGTGTTTGGTCCCTCTAGCTGTTATCGGTGACAAACTTAGTCCGGTAGCTGAAT
CATTAGTCTGGCGTACTCAAGGATTTATGCTTGACATTTCTGATGCATTCAGACATTCAGTCTCCTAGCCTAGCTTATCTTAAG
GATGCTAATTTTTGGGATAATTGGCGATCTTGTGATTTGTAATTTCACTGCTAAACCTGATATATTCTTTGGCATTTGCAGGTTA
TCTTGTATTCTTGTCAAGACAACTCTCTTGTTCCTCAGGGCACTGAGCAATCCTTTTGATTCATGACCAACTCTGAGTGGTGA
AAGGATTCAGAAAGTAGAGAATAGAACACCAGG
>SsLRR-RLK159
TACTTCATGATGTCACAAAAAAAAAGATGGAACAAATGCTTATATAATTAGATTTCAACTCAATACATGTCACCCTAGAAAAAA
AGAGACGTCCTATAAAATCTAAATAGAGGTTTTCACACTAGTTTTCATGAGCCGTGCTAAAAAAGATAAATTCTAAAGATTGT
CTAAAGAAAATGAAGATACATGACTATCGGTGCTCAAGTAGCATATAAAAGAGTGCAATATATTTTTTTCATAAAATACAAA
ATTAGAGTTAATAAAGTTACAATAAACATAGGATTTTTAACACAAATTAAGGATAAAAAAATCAATATTAATCAGTGTTGTCCT
GAACCTAAACGGCAAGTACTCGATCACATACAATTTACCATTATTTAGGCTAAACAATCACTTTAATGGGCAAAACGGTTATAT
ATAAACAGGATAAACGAAAACAATGTACCATCATGTCACGTTTAAACGATTTGTAAAGGGACTAAAGAACCATTTAGATCTG
TCTGATAAGATAAAAAATTAAAATAAAATTCTAAAACTGTTCAGTCTTGTAAGCTTGTTCGGCTGGCTCGTCAGCCAACCAGC
CAACAGTGTTTTTTTCTGACATCAAATTAGCATTAGCCATCAGCCACCTGCCAGCCAGCAGTATTTTTCTCTCACAGCAAATC
AGCACCGACCATCAACCACCAGCCAGCCGAACAAAACGGAGTAAATTGAAATCGTCATGCATATTGGTGTCAGCCTAACTG
GTTGCTGTGTGTTTTTTTTTGAAACTTTGCTGGTTGCTGGCAGTGCCGCTGATCAAAACAAACAAGCACTCAGCTGACGGCG
TACAACGGGACCTTATCCACTCTCACGGCCTCGCCATCATAACACACGCTCGGCGGCTCACACCGGAAAGGTGGCACCGGC
ACGGCACGGGCGCACGGCGCAGCGTCGACCGCACGCACGCACGCACGCCGCCCCCGGATCGGATCTACGTCTACCAGCTC
ACCCGCGCTACGGGCGTTAAAGACAAAAACCCTCGTCGAAGCAACGCGGCCCACGCGTCCGCCTCCCACCACGCCCGCCA
GGGATCCCGCACGCTCGGACCGCGACCCCGAATTCCCCAAACTGCCCATGCCCATGCCCGTGCCCGTGCCCGTGGGTGGTG
GAGTGGATTGACGAGCCCCCCCGGAGGCCGCGCCCGGCGGCACGTGACCCCGCCTCGGGCAGAGCACCGCCTGCCCCGAT
CGGGGCGGCCCCCGCCGTCGGATCCGATCGGGATTAACCTCGCCGCGGGCAGCGGATAAGCGCCGTGGTTAGAATACTAATC

TCCCTCGGCACTCGGGTTAATTAGGGCAGCGATTCGCGATGAGGCTAAGGCTATCCGCACTCGTCCACCTCTATTTCTACCTC
TAAAAAGGAATATTCTATCACAGTCATCGAGATTCTCTACTCTATATCTATTTCTCCCGCACCCGTGTTATCTCTATTTCATACTC
TATACCTACTATTTCATATTTTATTCCCCTCTCTCCCCTCTTCTCTCTCCCCAACTCTCTCTTTCTCTCTTGCTACAGTATTTAGC
GTAGCTGCACAGTACAACTCCAGGCGTAGCGCGCGCACCGTCCGACCGCTACGCGCCCGTGCGTGGTACAGGCGCGCGGTG
CGGACGCGCGCACGCTACGCCTGGCGTAGCGGCCGCTTTGGCGGTCTGCGGTGCCGACAGCCTAAGCAGGGGGCACATGC
CATGTGACGTGACCCCACCACCACTCCCTTCCCTCACCTACAGCTGTTAATTTGCTCCCCGTCTCCTCCTCCCCTCTCTCTTTG
CACTGTTGCTTCTTTCTTCCTGCCGCTTCTCTCTCTAGTCTAGGTATAGCAGCCCATTCCTCCACACTCCCCACTTCCCTCCCT
CCTCCACTCACCACCATCTCCTCCCACCATCTCGTCGGTCGGCTCCTCCCCGTCGCCGACCCGCTTCTTTAATGGTTTTAAGG
TAGCGCGCTAGGAGCTAGTTC
>SsLRR-RLK189
ATAATTCATTTTAAATTATATCGATATTTCAGGACAATAGTTTTTGAAAATTATTGGTTTCAGTAAGTTTATAATTTTGACTAAAG
GATTATGTCATATGAATACTGGTAATTTAATTTTAAGCTATATGCTCGTTAAAACAATGATAACATAAGCAATTCCTTGACACAA
CAAATTCAACATTTCAAGTGCCACAGGTGTCCAAGCTGAAATGATAGTTTAAAGCCATCGTAGTAATCGAGAATGTAGCTTA
GGGGCCTGTAAACATTTTTTAGTAAAATAAGAAGACACCAAATTTTAACGAAAAATGCAGTAAGTAGTAGAGTTTCAACTTA
GCATGTGAATTGTGCACCCCCCAAAAAAACCTTAGCATGTGAATATAAATTCGGCAACAGCTAATGAGATCAAAAGTTGAAA
GCTAATATATACTTCATGATGTCACAAAAAAAAAGATGGAACAAATGCTTATATAATTAGATTTCAACTTAATACATGTCACCTTA
GAAAAAAAGAGACGTCCTATAAAATCTAAATAGAGGTTTTCACACTAGTTTTCATGACCGTGCTAAAAAGATAAATTCTAAA
GATTGTCTAAAGAAAATGAAGATACATGACTATCGGTGCTCAAGTAGCATATAAAAGAGTGCAATATATTTTTTTCATAAAATA
CAAAATTAGAGTTAATAAAGTTACAATAAACATAGGATTTTTAACACAAATTAAGGATAAAAAATCAATATTAATCAGTGTTGT
CCTGAACCTAAACGGCAAGTACTCGATCACATACAATTTACCATTATTTAGGCTAAACAATCACTTTAATGGGCAAAACGGTT
ATATATAAACGGGATAAACGAAAACAATGTACCATCATGTCACGTTTAAACGATTTGTAAGGGGACTAAAGAACCATTTAGAT
CTGTCTGATAAGATAAAAATTAAAATAAAAATCTAAAACTGTTCAGTCTTGTAAGCTTGTTTGGCTGGCTGGTCAGCCAACCA
GCTAGCAGTGCTTTTCTCTGACATCAAATCAGCATCAGCCACTTCTCCCGCAACAAATTAGCACCGACTATCGCCACCAGCC
GGCCGAACAAAACGGAGTAAATTCAAATCGTCATGCACATTGGTGTCAGCCTTACTGGTTGCTGTGTTTTTTTTTGAAACTTT
GCTGGTTGCTGGCAGTGCCGCTGATCAAAACAAACAAGCACTCAGCTGCCGGCGTACAACGGGACCTTATCCACTCTCACG
GCCTCGCCATCATAACACACGCTCGGCGGCTCACACCGGAAAGGTGGCACCGGCACGGCACGGCGCAGCGTCGACCGCAC
GCACGCCGCCCCCGGATCGGATCTACCAGCTCACCCGCGCTACGGGCGTTAAAGACAAAAACCCTCGTCGAAGCAACGCGG
CCCACGCGTCCGCCTCCCACCACGCCCGCCAGGGATCCCGCACGCTCGGACCGCGACCCCGAATTCCCCAAACTGCCCATG
CCCATGCCCGTGCCCGTGGGTGGTGGAGTGGATTGACGAGCCCCCCCGGCGGCCGCGCCCGGCGGCACGTGACCCCGCCTC
GAGCAGAGCACCGCCTGCCCCGATCGGGGCGGCCCCCGCCGTCGGATCCGATCGGGATTAACCTCGCCGCGGGCAGCGGAT
AAGCGCCGTGGTTAGAATACTAATCTCCCTCGGCACTCGGGTTAATTAGGGCAGCGATTCGCGATGAGGCTAAGCAGGGGGC
ACATGCCATGTGACGTGACCCCACCACCACTCCCTTCACCTCACCTACAGCTGTTAATTTGCTCCCCGTCTCCTCCTCCCCTC
TCTCTTTGCACTGTTGCTTCTTTCTTCCTGCCGCTTCTCTCTCTAGTCTATAGCAGCCCATTCCTCCACACTCCCCACCTCCCT
CCCTCCTCCACTCCACTCACCACCATCTCGTCGGTCGGCTCCTCCCCGTCGCCGACCCGCTTCTTTAATGGTTTTAAGGTAGC
GCGCTAGGAGCTAGTTC
>SsLRR-RLK188
TAAGTTTATAATTTTGACTAAAGGATTATGTCATATGAATACTGGTAATTCAATTTTAAGCTATATGCTTGTTAAAACAACGATA
ACATAAGCAATTCCTTGACACAACAAATTCAACATTTCAAGTGCCACAGGTGTCTAAGCTGAAATGATAGTTTATGCCATCGT
AGTAATCGGGAATGTAGCTTAGGGGCCTATAAACATTTTTTAGTAAAATAAGAAGACACCAAATTTTAACAAAAAATGCAGTA
AGTAGTAGAGTTTTAACTTAGCATGTGAATTGTCTCCCCCCACCCCCACCCCCCAAAAAAATAGCATGTGAATATAAATTCGG
CAACAGCTAATAAGATCAAAAAGTTGAAAGCTAATATATACTTCATGATGTCACAAAAAAAAAGATGGAACAAATGCTTATATA
ATTAGATTTCAACTCAATACATGTCACCCTAGAAAAAAAAAAGAGACGTCCTATAAAATCTAAATAGAGGTTTTCACACTAGTT
TTCATGAGCCGTGCTAAAAAAGATAAATTCTAAAGATTGTCTAAAGAAAATGAAGATACATGACTATCGGTGCTCAAGTAGC
ATATAAAAGAGTGCAATATATTTTTTTTCATAAAATACAAAATTAGAGTTAATAAAGTTACAATAAACATAGGATTTTTAACAC
AAATTAAGGATAAAAAAATCAATATTAATCAGTGTTGTCCTGAACCTAAACGGCAAGTACTCGATCACATACAATTTACCATT
ATTTAGGCTAAACAATCACTTTAATGGGCAAAACGGTTATATATAAACGGGATAAACGAAAACAATGTACCATCATGTCACGT
TTAAACGATTTGTAAGGGGACTAAAGAACCATTTAGATCTGTCTGATAAGATAAAAATTAAAATAAAAATCTAAAACTGTTCA
GTCTTGTAAGCTTGTTTGGCTGGCTGGTCAGCCAACCAGCTAGCAGTGCTTTTCTCTGACATCAAATCAGCATCAGCCACCA
ATCACCTGTCAGCCAACAGTACTTTTCTCCCGCAACAAATTAGCACCGACTATCCGCCACCAGCCGGCCGAACAAAACGGA
GTAAATTCAAATCGTCATGCACATTGGTGTCAGCCTTACTGGTTGCTGTGTTTTTTTTTGAAACTTTGCTGGTTGCTGGCAGT
GCCGCTGATCAAAACAAACAAGCACTCAGCTGCCGGCGTACAACGGGACCTTATCCACTCTCACGGCCTCGCCATCATAAC
ACACGCTCGGCGGCTCACACCGGAAAGGTGGCACCGGCACGGCACGGCGCAGCGTCGACCGCACGCACGCCGCCCCCGG
ATCGGATCTACCAGCTCACCCGCGCTACGGGCGTTAAAGACAAAAACCCTCGTCGAAGCAACGCGGCCCACGCGTCCGCCT
CCCACCACGCCCGCCAGGGATCCCGCACGCTCGGACCGCGACCCCGAATTCCCCAAACTGCCCATGCCCATGCCCGTGCCC
GTGGGTGGTGGAGTGGATTGACGAGCCCCCCCGGCGGCCGCGCCCGGCGGCACGTGACCCCGCCTCGAGCAGAGCACCGC
CTGCCCCGATCGGGGCGGCCCCCGCCGTCGGATCCGATCGGGATTAACCTCGCCGCGGGCAGCGGATAAGCGCCGTGGTTA
GAATACTAATCTCCCTCGGCACTCGGGTTAATTAGGGCAGCGATTCGCGATGAGGCTAAGCAGGGGGCACATGCCATGTGAC
GTGACCCCACCACCACTCCCTTCCCTCACCTTCACCTACAGCTGTTAATTTGCTCCCCGTCTCCTCCTCCCCTCTCTCTCTTTG
CACTGTTGCTTCTTTCTTCCTGCCGCTTCTCTCTCTAGTCTAGGTGCACCTTCCTCCACACTCCCCACCTCCCTCCCTCCTCCA
CTCACCACCATCTCCTCCCAAGTCCCACCATCTCGTCGGTCGGCTCCTCCCCCGTCGCCGACCCGCTTCTTTAATGGTTTTAA
GGTAGCGCGCTAGGAGCTAGTTC
>SsLRR-RLK147
GATAATTCATTTTAAATTATATCGATATTTCAGGACAATAGTTTTTGAAAATTGTTGGCTTCAGTAAGTTTATAATTTTGACTAA
AGGATTATGTCATATGAATACTGGTAATTTAATTTTATGCTATATGCTCGTTAAAACAATGATAACATAAGCAATTCCTTGACAC
AACAAATTCAACATTTCAAGTGCCACGGGTGTCCAAGCTTAAATGACAGTTTAAAGCCATCGTAGTAATCGAGAATGTAGCT
TAGGGGCCTGTAAACATTTTTTGGTAAAATAAGAAGACACCAAATTTTAACGAAAAATGCAGTAAGTAGTAGAGTTTCAACT
TAGCATGTGAATTGTGCACCCCCCAAAAAAAAAGAAAAAACCTTAGCATGTGAATATAAATTCGGCAACAGCTAATGAGATCA
AAAGTTGAAAGCTAATATATACTTCATGATGTCACAAAAAAAAAGATGGAACAAATGCTTATATAATTAGATTTCAACTTAATAC
ATGTCACCTTAGAAAAAAAGAGACATCCTATAAAATCTAAATAGAGGTTTTCACACTAGTTTTCATGAACCGTGCTAAAAAG
ATAAATTCTAAAGATTGTGTAAAGAAAATGAAGATACATGACTATCGGTGCTCAAGTAGCATATAAAAGAGTGCAATATATATT
TTTTCATAAAATACAAAATTAGAGTTAATAAAGTTACAATAAACATAGTATTTTTAACACAAATTAAGGATAAAAAATCAATAT
TAATCAGTGTTGTCCTGAACCTAAACGGCAAGGCTAAATGGTCACTTTAATGGGCAAAACGGTTATATAAACGGGATAAACG
AAAACAATGTACCATCACGTCACGTTTAAACGATTTGTAAAGGGACTAAAGAACCATTTAGATCTGTCTGATAAGATAAAAA
AAATAAAAATAAAAATCTAAAATTGTTCAGTCTTGTAAGCTTGTTTGGCTGGCTGGTCAGCCAACCAGTCAGCAGTGCTTTT
CTCTGACATCAAATTAGCATCAGCCACTAGTCACGTGCCAGCCAACAGTATTTTTCTCTCACAACAAATCAGCACCGACCAT
CGGCCACCAGCCAGCCGAACAAAACGGAGTAAATTCAAATCGTCATACACATTGGTGTCAGCCTTGCTGTGTTTTTTTTTTTT
GAAACTTTGCTGGTTGCTGGCAGTGCCGCTGATCAAAACAAACAAGCACTCAGCTGCCGGCGTACAGCGGGACCTTATCCA
CTCTCACGGCCTCGCCATCATAACACACGCTCGGCGGCTCACACCGGAAAGGTGGCACGGGCACGGCACGGCGCAGCGTC
GACCGCACGCACGCACGCCCTCCCGCCGCCCCCCCCGGATCGGATCTACCAGCTCACCCGCGCTACGGGCGTTAAAGACA
AAAACCCTCGTCGAAGCAACGCGGCCCACGCGTCCGCCTCCCACCACGCCCGCCAGGGATCCCGCACGCTCGGACCGCGA

CCCCGAATTCCCCAAACTGCCCATGCCCATGCCCGTGCCCGTGATTGACGAGCCCCCCGGAGGCCGCGCCCGGCGGCACGT
GACCCCGCCTCGAGCAGAGCACCGCCTGCCCCGATCGGGGCGGCCCCCGCCGTCGGATCCGATCGGGATTAACCTCGCCGC
GGGCAGCGGATAAGCGCCGTGGTTAGAATACTAATCTCCCTCGGCACTCGGGTTAATTAGGGCAGCGATTCGCGATGAGGCT
AAGCAGGGGGCACATGCCATGTGACGTGACCCCACCACCACTCCCTTACCTCACCTACAGCTGTTAATTTGCTCCCCGTCTC
CTCCTCCCCTCTCTCTTTGCACTGTCGCTTCTTTCTTCCTGTCGCTTCTCTCTCTAGGTATAGCAGCCCATTCCTCCACACTCC
CCACCTCCCTCCCTCCTCCACTCACCACCATCTCGTCGGTCGGCTCCTCCCCCGTCGCCGACCCGCTTCTTTAATGGTTTTAA
GGTAGCGCGCTAGGAGCTAGTTCA
>SsLRR-RLK68
CACTAGAATATATAATATACAAAATTTCATTTCACACTTCATTTGTCACTTTGTGACAAAAACAAAGGTGTGATAGGCTAAGAC
CATTAAAGCAAAGGTACACATAGAAACATTGGTTCAAATACTTATTCATCATATTACAAAATTGAAGAGAAAAGCACAATTTC
CACTCCTTCAATACCTACTTATAATTTATAGTAGACTCGAGTACCCTTCTGCAACCAATCCCTTCATATCCAACTGATTCCATTA
TGAAGTTGTAGAATCTGCTGCTACAGTAGTAAAGGCTATGGTATCTAAATAGTGTCCCTTGGAAAAACCATTCATGTAGCAAT
GGTTCTCCTTACAAAACTAATGTGAAAGTGCATTTCGACATACCCATTGTTGATTTGCGATGAGGATGTCAATTGGACGAATG
ATCTTATTGTTCTCTATAAACAAAAGCTAGGGCCTTAATATCTACTAGTGTCTTCGCCCATATCATCTATCTATATAAAAGATGA
CAATGTAGCCAATGGCTACGGTTTTGTAAGGCAATGGTACATGAAGATAGCATGAGTCGTGTACAAACCAATTTTGGCGTATA
GATAGAACCGTTCAATGATCTTGTATGCAATGCATTTTGCCGCTTCACTAGAATATAATAATTTACAAAATTTCATTTCACATTT
TATCTGTCGCTTTGTGACAAAAACAAAGAAGGTGTGACGGGCTAAGACCATCAAAGCAAAGGTGCACATAGAAACAATAAG
ATACCCAAAGAATTCCTACTACTAGAAAAAGAGAGAGAGATCAAGACAATGAAATATCTTTTTTCACATCAAAAAAGAAAA
ATCACAAACTACTCTATAATTATCCACACACAATTCTCCAAGACAAATTCTTAGTGGAAAAAAATGTAAATAAACCTAAAAGG
CTAAAACCATAGAGAAATTTGTTTGGGTAAGAGAGAGAGCTGGGATGGATGGGAGATGCCAAAAAGCCGGGCTCGGGGGA
ATCAAATCCCAAGTTTGACCGGGCTCGGATGCTGAGACACACAACTGAGAAAGGAACGCCTTTTCCTACCAGCCCCTCGCA
ACAAGCCAATATTATTCTTCCATGGCCTCCCCTAGACACATGGATTAATTTTTTGATTAAACAATCCGGCCCTCTTTCCGGGGC
TAATTGCCGTCGCAAGCAAACAAAGCTCGCCGAGGTTTTGTTAATCCGAAGTGCCAATGGCGGGGTTCAGTAGCTGAGTAG
CGCCCTTTCTCTCTCTTTCCCTCGTCCTACTTAAATACTCGCAAGTGCCGCTGTGTGTGCTCTTCTACTTTCTCTCCTCCTCTG
CCCCTTCTTCTCCCTCCCTCTGCCTCTCGTCGTCTCCAATTGCAGAAGTAGTAGTAGGTTGGTTGGTCTGTTGGTGGTGTTCT
TCCATTCTTGATGAGGGGTTGGGTTCTTCGCTTGACCAAGGTGAGCAGCAGCCTCTTCTTGGATTTGCTTTCGTGCCAGTAGT
GTAGTGGTTTCCGGTTTGTTATTGGGTTGAAGAAATGTTTGGTTGTTCATTTGGGTACCTGATTAATCTTGATTTTTTTTGGTG
GCTTTCTGGTCCTCTGGTTTATATTCGTACAACCTGATTAGGTTCGTTTCTTTCGTCCCAAATTGGAGTTGCTTTTCTTCCCGG
TATAGTTCATCATCATGGCATCTTTGAATTTTGGAGGAAAAAACTACCTCGGGGGTTTGGTGCGCAGTTTGTGCCGATAATTT
CGCATTTGCAACTGTATGACTGTTCTTGGCACGGGCGCTTTCCCTTCCAAATAAGATTCCTTGTGCTCTTCTATCGTTTTGCTT
CTTTGTTAAATGATTCGAGAATTTCCTGTGCGAATATGCAGTGAGGTCATCCGCTCCACAGTCATGTGAAGTTGTCCAAGAAT
TCGGCAGGACGCAAGAACCGGCGGCCCTTCTTCTCCATTGTTTGCTCATCTTGTTCCCTGCGACAAATCGACGCTCGCAAAT
TGGAGCCA
>SsLRR-RLK69
AATATATAATATACAAAATTTCATTTCACACTTCATTTGTCACTTTGTGACAAAAACAAAGGTGTGATAGGCTAAGACCATTAA
AGCAAAGGTACACATAGAAACATTGGTTCAAATATTATTCATCATATTACAAAATTGAAGAGAAAAGCACAATTTCCACTCCT
TCAATACCTACTTATAATTTATAGTAGACTCGAGTACCCTTCTGCAACCAATCCCTTCATATCCAACTGATTCCATTATGAAGTT
GTAGAATCTGCTGCTACAGTAGTAAAGACTATGGTATCTAAATAGTGTCCCTTGGAAAAACCATTCATGTAGCAATGGTTCTC
CTTACAAAACTAATGTGAAAGTGCATTTCGACATACCCATTGTTGATTTGCGATGAGGATGTCAATTGGACGAATGATCTTATT
GTTCTCTATAAACAAAAGCTAGGGCCTTAATATCTACTAGTGTCTTCGCCCATATCATCTATCTATATAAAAGATGACAATGTAG
CCAATGGCTACGGTTTTGTAAGGCAATGGTACATGAAGATAGCATGAGTCGTGTACAAACCAATTTTGGCGTATAGATAGAAC
CGTTCGATGATCTTGTATGCACTACATTTTGCCGCTTCACTAGAATATAATAATTTGCAAAATTTCATTTCACATTTTATCTATCG
CTTTGTGACAAAAACAAAGAAGGTGTGACGGGCTAAGACCGTCAAAGCAAAGGTGCACATAGAAACAATAAGATACCCGA
AGAATTCCTACTACTAGAAAAAGAAGAGAGCTCAAGACAATGAAATATCTTTTTTCACACCAACAAAGAAAAATCACAAAC
TACTCTATAATTATCCACACACAATTCTCCAAGACAAATTCTTAGTGGAAAAATGTAAATAAACCTAAAAGGCTAAAACTATA
GAGAAATTTGTTTGGGCAAGAGAGAGAGCTGGGATGGGATCGGAGATGCCAAAAAGCCGGGCTCGGGGGAATCAAATCCC
AAGTTTGACCGGGCTCGGATGCTGAGACACACAACTGAGAAAGGAACGCCTTTTCCTACCAGCCCCTCGCAACAAGCCAAT
ATTATTCTTCCATGGCGTCCCCTAGACACATGGATTAATTTTTTGATTAAACAATCCGGCCCTCTTTCCGGGGCTAATTGCCGT
CGCAAGCAAACAAAGCTCGCCGAGGTTTTGTTAATCCGAAGTGCCAATGGCGGGGTTCAGTAGCTGAGTAGCGCCCTTTCT
CTCTCTTTCCCTCGTCCTACTTAAATACTCGCAAGTGCCGCTGTGTGTGCTCTTCTACTTTTGCTTCTCTCTCTCCTCCTCTGC
CCCTTCTTCTCCCTCCCTCTGCCTCTCGTCGTCTCCAATTGCAGAAGTAGTAGTAGGTTGGTTGGTCTGTTGGTGGTGTTCTT
CCATTCTTGATGAGGGGTTGGGTTCTTCGCTTGACCAAGGTGAGCAGCAGCCTCTTCTTGGATTTGCTTTCGTGCCAGTAGT
GTAGTGGTTTCGGGTTTGTTATTGGGTTGAAGAAATGTTTGGTTGTTCATTTGGGTACCTGATTAATCTTGATTTTTTTTGGTG
GCTTTCTGGTCCTCTGGTTTATATTCGTACAACCTGATTAGGTTCGTTTCTTTCGTCCCAAATTGGAGTTGCTTTTCTTCCCGG
TATAGTTCATCATCATGGCATCTTTGAATTTTGGAGGAAAAAACTACCTCGGGGGTTTGGTGCGCAGTTTGTGCCGATAATTT
CGCATTTGCAACTGTATGACTGTTCTTGGCACGGGCGCTTTCCCTTCCAAATAAGATTCCTTGTGCTCTTCTATCGTTTTGCTT
CTTTGTTAAATGATTCGAGAATTTCCTGTGCGAATATGCAGTGAGGTCATCCGCTCCACAGTCATGTGAAGTTGTCCAAGAAT
TCGGCAGGACGCAAGAACCGGCGGCCCTTCTTCTCCATTGTTTGCTCATCTTGTTCCCTGCGACAAATCGACGCTCGCAAAT
TGGAGCCA
>SsLRR-RLK111
ATATAATATACAAAATTTCATTTCACACTTTATTTGTCACTTTGTGACAAAAACAAAGAAGGTGTGATAGGCTAAGACCATTAA
AGCAAAGGTACACATAGAAACATTGGTTCAAATACTTATTCATCATATTACAAAATTGAAGAGAAAAGCACAATTTCCACTCC
TTCAATACCTACTTATAATTTATAGTAGACTCGAGTACCCTTCTGCAACCAATCCCTTCATATCTGACTGATTCCATTATGAAGT
TGTAGAATTTGCTGCTATAGTAGTAAAGACTATGGCATCTAAATAGTGTCCCTTGGAGAAACCATTCATGTAGCAATGGTTCTC
CTTACAAAACTAATATGAAAGTGCATTTCGACATACCCATTGTTGATTTGCAATGAGGATGTCAGTTGGACGAATGATCTTATT
GTTCTCTATAAACAAAAGCGAGGGCCTTAATATCTACTAGCGTCTTTGCCCATATCATCTATCTATATAAAAGATGACAATGTAG
CCAATGGCTACGGTTTTGTAAGGCAATGGTACATGACGATAGCATGAGTCGTGTACAAACCAATTTTGGCGTATAGATAGAAC
CGTTCGATGATCTTGTATGCACTGCATTTTGCCGCTTCACTAGAATATAATAATTTACAAAATTTCATTTCACATTTTATCTGTC
GCTTTGTGACAAAAACAAAGAAGGTGTGATGGGCTAAGACCATCAAAGCAAAGGTGCACATAGAAACAATAAGATACCCG
AAGAATTCCTACTACTAGAAAAAGAGAGAGCTCAAGACAATGAAATATCTTTTTTCACATCAAAAAAGAAAAATCACAAAC
TGCTCTATAATTATCCACACACAATTCTCCAAGACAAATTCTTAGTGGAAAAAAATGTAAATAAACCTAAAAGGCTAAAACTA
TAGAGAAATTTGTTTGGGTAAGAGAGAGAGCTGGGATGGATGGGAGATGCAAAAAGCCGGACTCGGGGGAATCAAATCCC
AAGTTTGACCGGGCTCGGATGCTGAGACACACAACTGAGAAAGGAACGACTTTTCCTACCAGCCCCTCGCAACAAGCCAAT
ATTATTCTTCCATGGCCTCCCCTAGTTTGATCAATTTTTTGATTAAACAATCCGGCCCTCTTTCCGGGGCTAATTGCCGTCGCA
AGCAAACAAAGCTCGCCGAGGTTTTGTTAATCCGAAGTGCCAATGGCGGGGTTCAGTAGCTGAGTATCGCCCTTTCTCTCTC
TTTCCCTCGTCCTACTTAAATACTCGTAAGTGCCGCTGTGTGTGCTCTTCTACTTTTGCTTCTCTCTCTCCTCCTCTGCCCCTTC
TTCTCCCTCCCTCTGCCTCTCGTCGTCTCCAATTGCAGAAGTAGTAGTAGGTTGGTTGGTTGGTCTGTTGGTGGTGTTCTTCC
ATTCTTGATGAGGGGTTGGGTTCTTCGCTTGACCAAGGTGAGCAGCAGCCTCTTCTTGGATTTGCTTTCGTGCCAGTAGTGC
AGTGGTTTCGGGTTTGTTATTGGGTTGAAGAAATGTTTGGTTGTTCATTTGGGTACCTGATTAATCTTGATTTTTTGGTGGCTT
TCTGGTCCTCTGGTTTATATTCGTACAACCTGATTAGGTTCGTTTCTTTCGTCCCAAATTGGAGTTGCTTTTCTTCCCGGTATAG

TTCATCATCATGGCATCTTTGAATTTTGGAGGAAAAAACTACCTCGGGGGTTTGGTGCGCAGTTTGTGCCGATAATTTCGCAT
TTGCAACTGTATGACTGTTCTTGGCACGGGCGCTTTCCCTTCCAAATAAGATTCCTTGTGCTCTTCTATCGTTTTGCTTCTTTG
TTAAATGATTCGAGAATTTCCTGTGCGAATATGCAGTGAGGTCATCCGCTCCACAGTCATGTGAAGTTGTCCAAGAATTCGGC
AGGACGCAAGAACCGGCGGCCCTTCTTCTCCATTGTTTGCTCATCTTGTTCCCTGCGACAAATCGACGCTCGCAAATTGGAG
CCATG

>SsLRR-RLK313

CGCATGGTCCTTCGCTTCGAAGCCTCGACTTGCCCTTCACCTTCACAACCGGTCCATCGAGCCAAGCCTCATCTTGGTCTTC
ACCATCTTGGTCACATGAGTCAATGTCATGTCTCATATTCAACGACCACCTTTATCTAAATCATCCACTTAGATACAAAGGTTA
GTCCTAGGTTTCATCAATTAGCCAAAACCAAACTAGGGACTTTCAATCTCCCCCTTTTGGTAATTGATGACAACCCTCACAAA
GATGATTTTGATAATAAAAGTTTTGAACTTGTCATACTTGTCCAATCATTTTGCTCATAAGTAAAGTTTTGGACAATTATCACA
AGCTCGGTTTTGGTAGCATTAGCTCCCCCTACATATGTGCATAGAGTTTGGATTGAAAGCTTTGCACATATGCATGGATAGAA
GTGTTGGGAGAGTAATTCTACCAAAAGGATGCTAAGGTGTAAGAAATGGACCTTTGAAGCGTGATACCAATCGGAGTTGCCT
TTTACACCATCCTTAGCATCATGAAGTAACTAGACAACCATCAAAACTCAAGAACCCCGTGAGATCAACNNNNNNNNNNNN
NNNNNNNNNNNNNNNNNNNNNNNNNNNNNNNNNNNNNNNNNNNNNNNNNNNNNNNNNNNNNNNNNNNNNNNNNNNNNNNN
NNNNNNNNNNNNNNACGTATTATATGTTTCGATCCACAACTTAAGTTTCTCAATATTGCTTCTTCTAATTGTAACCATTAGATTTAT
TCGAATGGATGCACTAGTTTTTTTAAGCACCGTAAAATTTCTCTTAGTGTAATAGCAAGAAATTGTGAGTAATGCTACAATTTT
TATTTAAACAAATACACCCACATGTATATGATGCTGTATATAAAACAAAAGAACCATAGTAAGGTTAATAAAGTTTTTCCTATT
GCAAGACACGAGTTCTCACCTAGTAAAATGCAAAAGTGAAAAATATGACGTTCCTATGGACACATGGGTAAAAGTTAAATAA
CCTATGGTATATTTAGCTCAAGAACAAGTGATTGATAAGATTAGCAGACTCCTATGTACGTGTCATAAATCTAAATTTTTGCAC
GCCTTGTATTAGTTCATATAAAAACGAAATAAGGTTGGCTTTTGCGGTGCAATTGTACAGAAGCGGCCGCACACAGCAAAAG
GCTAAAGTGGTTGGGTCCAAGTTTGACCGCACCAGGAGTCCCGCACGCGCAAGCAAACAAAAACAAAGTCCAAATGTGCC
CCGGCAATTTCGTCGTAATCTTGCCAAATCATCCCGACTACTCGTGCACTTGTGCTCCATTTTCCGAGCTATATCGCCGCCAGT
AAAGGGGGTCAAGGTTTTGTTTTTGTCCGGCCAGCAGCAGCCTCCTGCTGTGTTCGCGACCAGTGTTCTGCTTGCTCCCACC
CCCACTTTGTGGCTCCGTCTCCACAGCTCTCCAACCGCAACGGCGAGGAATTCTCCTCCCCTTCTCTGCCTACGAAATGCTAT
GTTCTTGATGCGGCTTACGGCCACGAAGAAACTATTGAACCGAACCAAGGTTGGATTTCTGTATATGTCTCACCTTTCTTTTC
TTTTATAAGTACATAAATAACATCCATTTGGTTTTGTCTTATTTGCATTTCTATCGAACAAATTGTGGGTAGACTTACTCATAGT
TTAGCAGTTAATACGTAGTTACGTGTGCTTCCTATCTGAAAATTTCACACTGTTCAAATGTTTCTGGGTGCCTAACGCTTTCCT
CCCAAGTATTGTTGCCCCACTTTTCTCCAAAGAACGATCCATTTATAGATTACAAACTGCAAATTTGCACCGGGTTGTTCTGG
GCGGGGGATCCCCATCGGATGTGTTTAATTTTTAACCAAACTTGGTTCTTTCCCTTCCAGGCGCACTCGAATTCGCTCAGGAT
TAATCCCCCTTCGATGTGAAGCCATCCAGAGCGCCAATTTGTGTTCTTCTTCAATCATTTCGACAGCAAGAAGAAGCATCCAT
GGGCGCCGCTCGTA

>SsLRR-RLK343

TGGCGTGCGCCTACTCAGGCTGGCATTGGCCGGTGGTGGCACGGTGTCGGGGTGTCATCGGGCGGCCGTGGCGGCGGGTGA
AGAAGAGGAGATAGCACACAAAGGAGATGACGAGGAGCTCCTGTGAGTATGAATGTACTGTTATACCCTTGGGATTAAATTG
AGGACTAACACACGGTCCACGTATTATATGTTTCGGTCCACAACTTAAGTTTTTCAATATTGGTTCTTCTTAATTTGTAACCATT
AGATTTATTCGAATGGATGGCACTAGTTTTTTTAAGCACCGTAAAATTTCTCTTAGTGTAATAGCAAGAAATTGTGAGTAATG
CTACAATTTTTATTTAAACAAATACACCCACATGTATATGATGCTGTATATAAAACAAAAGAACCATAGTAAGGTTAATAAAG
TTTTTCCTATTGCAAGACACGAGTTCTCACCTAGTAAAATGCAAAAGTGAAAAATATGACGTTCCTATGGACACATGGGTAA
AAGTTAAATAACCTATGGTATATTTAGCTCAAGAACAAGTGATTGATAAGATTAGCAGACTCCTATGTACCTGTCATAAATCTA
AATTTTTGCACGCCTTGTATTAGTTCATATAAAAACGAAAGAGTAAAGTGCACCAGCGGTCCTTAAACTTGCGCTGGTGTGTC
ATTCCGGTCCCTAAATAGTTTTAAATTTCAAAATCTGTAAACATACAACAATATTTTGGGACTCTAAACAGTTTTAATTCAAAA
ACTTTTGAACTACAAAGTTGTAGATCGCGTCGAGGGCTACAATTTTGATATAAAGTTTGTCCTCATTTGAGTTCATATGAAAA
AGTTATGAATTATTGTTTGATATCAAGTTTTTAAATCGCCTAGTTGGACCCAGATGAGATCCAAAACCAAGTTTAGGGACCGG
GATGACACAACTGAACAAGTTTGAGGACCTAAACGGTCGATTTGCAAGTTTAGGGACCGGGATGACACAACCGAATAAGTT
TAGGGACCGAGATGACACAGATGAACAAGTTTGAGGACCTAAATAGTTGATTTACGAGTTTAGGAACCGGGATAACACAGC
AGCGCAAGTTTAGAGACCGCTACTGCACTTTACTCAAAACGAAATAAGGTTGGCTTTTGCGGTGCAATTGTACAGAAGCGG
CCGCACACAGCAAAAGGCTAAAGTGGTTGGGTCCAAGTTTGACCGCACCAGGAGTCCCGCACGCGCAAGCAAACAAAAAC
AAAGTCCAAATGTGCCCCGGCAATTTCGTCGTAATCTTGCCAAATCATCCCGACTACTCGTGCACTTGTGCTCCATTTTCCGA
GCTATATCGCCGTCAGTAAAGGGGGTCAAGGTTTTGTTTTTGTCCGGCCAGCAGCAGCCTCCTGCTGTGTTCGCGACCAGTG
TTCTGCTTGCTCCCACCCCCACTTTGTGGCTCCGTCTCCACAGCTCTCCAACTGCAACGGCGAGGAATTCTCCTCCCCCTTCT
CTGCCTACAAAATGCTATGTTCTTGATGCGGCTTACGGCCACGGAGAAACTATTGAACCGAACCGAGGTTGGATTTCTGTATA
TGTCTCACCTTTCTTTTCTTTTATAAGAACATAAATAACATCCATTTGGTTTTGTCTTATTTGCATTTCTATCGAACAAATTGTG
GGTAGACTTACTCATAGTTTAGCAGTTAATACGTAGTTACGTGTGCTTCCTATCTGAAAATTTCACACTGTTCAAATGTTTCTG
GGTGCCTAACGCTTTCCTCCCAAGTATTGTTGCCCCACTTTTCTCCAAAGAACGATCCATTTATAGATTACAAACTGCAAATTT
GCACCGGGTTGTTCTGGGCGGGGGATCCCCATCGGATGTGTTTAATTTTTAACCAAACTTGGTTCTTTCCCTTCCAGGCGCAC
TCGAATTCGCTCAGGATTAATCCCCCTTCGATGTGAAGCCATCCAGAGCGCCAATTTGTGTTCTTCAATCATTTCGACAGCAA
GAAGAAGCATCC

>SsLRR-RLK335

TCCCACCACCGCGAGCAGCAGGGGCTGGCTGCCCAGGGTCACTGCCGTCAATCCAATCGACCTACACGGCTTCCTTCATCC
ATTCATGTTGGTTAATTCCTGTGTTAAAATGCTTGGCTACCCGTATAGCAAAGATGCAAATTACATTCTCTCTGTATTATTGTTA
ATTACATGGTTGTTCAAATATTATTTTTGCTTTGCTACTTGGATTCCCTCTTGAGCAAGGTGCTGTCAGTTCTGAAATCGGAAG
AGTTACTCTGCTTTCAGGGGACTATGTTTTCTAGATGATTTCTTTTGGCTGCACGGCTTTACTCTGATAGTACAAAGTGTTAGC
ATTGTTTGAACAGGGTAGCAAATTTACGAGGTGAGGGAGAAGGGAAAGCAAGATACAGAGGCCTGCCGCGCCATCGCCGA
ACGCCAGCCCGCAGCCGCTGCTCCGGCTCCTACACTGGAGGTCCACGATTGGACGGCTTGAGTTGGGGGCAGCTCGGCCAT
GGCTGGCGTGCGCCTACTCAGGCTGGCATTGGCCGGTGGTGGCACGGTGTCGGGGTGTCATCGGGCGGCGTGGCGGCGGGT
GAAGAAGAGGAGATAGCACACAAAGGAGATGACGAGGAGCTCCTGTGAGTATGAATGTACTGTTATACCCTTGGGATTAAAT
TGAGGACTAGTACACGGTCCACGTATTATATGTTTCGATCCACAACTTAAGTTTCTCAATATTGGTTCTTCTTAATTTGTAACC
ATTAGATTTATTCGAATGGATGGCACTAGTTTTTTTAAGCACCGTAAAATTTCTCTTAGTGTAATAGCAAGAAATTGTGAGTA
ATGCTACAATTTTTATTTAAACAAATACACCCACATGTATATGATGCTGTATATAAAAACAAAAGAACCATAGTAAGGTTAATA
AAGTTTTTCCTATTGCAAGACACGAGTTCTCACCTAGTAAAATGCAAAAGTGAAAAATATGACGTTCCTATGGACACATGGG
TAAAAGTTAAATAACCTATGGTATATTTAGCTCAAGAACAAGTGATTGATAAGATTAGCAGACTCCTATGTACGTGTCATAAAT
CTAAATTTTTGCACGCCTTGTATTAGTTCATATAAAAACGAAATAAGGTTGGCTTTTGCGGTGCAATTGTACAGAAGCGGCCG
CACACAGCAAAAGGCTAAAGTGGTTGGGTCCAAGTTTGACCGCACCAGGAGTCCCGCACGCGCAAGCAAACAAAAACAA
AGTCCAAATGTGCCCCGGCAATTTCGTCGTAATCTTGCCAAATCATCCCGACTACTCGTGCACTTGTGCTCCATTTTCCGAGC
TATATCGCCGCCAGTAAAGGGGGTCAAGGTTTTGTTTTTGTCCGGCCAGCAGCAGCCTCCTGCTGTGTTCGCGACCAGTGTT
CTGCTTGCTCCCACCCCCACTTTGTGGCTCCGTCTCCACAGCTCTCCAACCGCAACGGCGAGGAATTCTCCTCCCCCTTCTC
TGCCTACGAAATGCTATGTTCTTGATGCGGCTTACGGCCACGAGAAACTATTGAACCGAACCAAGGTTGGATTTCTGTATATG
TCTCACCTTTCTTTTCTTTTATAAGAACATAAATAACATCCATTTGGTTTTGTCTTATTTGCATTTCTATTGAACAAATTGTGGG
TAGACTTACTCATAGTTTAGCAGTTAATACGTAGTTACGTGTGCTTCCTATCTGAAAATTTCACACTGTTCAAATGTTTCTGGG
TGCCTAACGCTTTCCTCCCAAGTATTGTTGCCCCACTTTTCTCCAAAGAACGATCCATTTATAGATTACAAACTGCAAATTTGC

ACCGGGTTGTTCTGGGCGGGGGATCCCCATCGGATGTGTTTAATTTTTAACCAAACTTGGTTCTTTCCCTTCCAGGCGCACTC
GAATTCGCTCAGGATTAATCCCCCTTCGATGTGAAGCCATCCAGAGCGCCAATTTGTGTTCTTCTTCAATCATTTCGACAGCA
AGAAGAAGCATCC
>SsLRR-RLK26
AAGGGATGCCAGTTAGGTCGTTCATGAACTTTAAAAATACAATTTTGGGTCCCTAAACTTGTTAAGTGGTGCAGCGCACATC
TCGAAACGTGTAAAATGGTGCACCACAGTTCCATTTGTAGATCATATCGACCACTTTGATGCACCATTACAAGTTTAGGATAT
AGAAATGTATTTTCAAAGTTTATTGACCTAAATAGCACATATTGATAAGTTCAGAGACTATTCATGCATTTGACTCTAAACAAT
ATGATGGAAAAGAAACCAACAATGGTCCAAATATATTGTCATGATTTTTACGTTCCAAAACAGCATGTCTTCAATTGTTGGAT
GGGAGGAGCGCTCCGTCTCCAAATTTAAGACACGATTTGACCTATAATTTATCTCGTACTAAATCCTTCAGTGTTACAAAATC
ATAAAAAATACGAGAGCACTAGCACCCGGACATCCGGCACCCGTAGCGCGTCCGGGACGCATCTCCCAGTCCCCGGCCAAC
CGAACATGGGCTGACGTCGTGGCCCATAAACGAGACCATCTTAGCATCCTGTGTAAAATAGAAAACTAAAGGAGACTGAAG
CGCTACCTGCCCGCGTAACGAAAGGGGAGAGCGAGCGCTCGCGCCCATGCCACAATCCCCCGGCGCATCCCATCCGCCGGC
CCCCAGCGCACCCTCTTTGCTGACCCTCACTGCGCCACCTCTGCAGTATAAGAAGATAGATATGCAAGGCAGCAGAAGGTGA
TAAGGAGACACCAAAGCGAGGCAGCAGAAAGCGAGGAGGGAGATGCAACACCCGATCTACTTTTAAAACATACAGATACA
ACACTTGCAACATATGTTTGAAGATAGATGAAACATTTAAAACATGCGTTTGAAACACTTACAAAAACACATGAAAAAACAC
TTGAAAAACAATTGCAACACATACGTAACATCCGGATAAAACGCTTACAACATATGTATAAAATATATGCAACATCCAGATAA
ACACACTTGCAATACAACAGATGAAACATTGGGAACATACGTTTGCAACATATATGTATAACCATAGCAACATACGCAACATC
CCGATCTATTTTTGCAACATCCATACGAAACCCTTGCAACATACCTCTAAGACATATAAAACACTTGAAACATGAGCTTGCAA
CATGCGCTTTTAGTGCAAGCGCAATGTCACCTTGCTGCTTGGACGAATGGAGCTCGTCGCTGTGGAGCTTAACACCAGAGCG
GAGCTCGATGCCACGGAGTGCGTGGAGGTTGCCGGTGTGGAGCTCGTTGGTGGCATGGATCTCGGCAGTGGCCACGACGG
GAGGTGCGATTACGGGGGGTGGCTTGGGCGGGGGTGGCGCCGGCATGCGGCGCGGGTAGAATCCGTCGTGCGACCGCGAG
CGATGGCGCACAACACAGAGCGTCGCGAGCGACGCGGGCGAATAGATGAGGATGAGCCGTAAGGTCTGGACGGGAAACAA
CGCGAGCGATGCAGGCGAGTGGATGAGGATGAGGCACGTCTGGACGGGAAGTCACGGGACGGAAGCCCGAGCCATATCAT
TATCGAAAAAAAATAAGATACTTCTTTCAATTATGAACCCAATGGTATCACTTTTGTGCGACAAAAAATTACATTTTGCAAGG
CTAATTGTGAGTTGACAATTACGTGTTTTGAAATCTTAAAATGATTCCGCACGTTATAAACAATGAAGGGATTATACGTCATAA
TATGTCCATCCAGCTGCCTCTCTAGGTTGTTGAGCTAACAGTCCCCAAAGGCACACCAACACAAGCAAGAGAGCAGCACAG
ATTGTGTCACGCACTGATCCTTGCTTTCATATATATACTAATCCGAAACCAAAGACATGCATCATACTGGTTCCCAACACAAAC
AATTTTACTTGCATCATCTCTCTCGTGTAGCTTTCTCGCTCCTTGTACCATGCTTCTTTGAACTCCGTCGCCTCTCATCTCCCAT
CCACTGTCGCCCAGCTTTGCTGCCATCACC
>SsLRR-RLK42
AACTTGTTAAGGGATGCCAGTTAGGTCGTTCATGAACTTTAAAAATACAATTTTGGGTCCCTAAACTTGTTAAGTGGTGCAGC
GCACATCTCGAAACGTGTAAAATGGTGCACCACAGTTCCATTTGTAGATCATATCGACCACTTTGATGCACCATTACAAGTTT
AGGATATAGAAATGTATTTTCAAAGTTTATTGACCTAAATAGCACATATTGATAAGTTCAGAGACTATTCATGCATTTGACTCTA
AACAATATGATGGAAAAGAAACCAACAATGGTCCAAATATATTGTCATGATTTTTACGTTCCAAAACAGCATGTCTTCAATTG
TTGGATGGGAGGAGCGCTCCGTCTCCAAATTTAAGACACGATTTGACCTATAATTTATCTCGTACTAAATCCTTCAGTGTTACA
AAATCATAAAAAATACGAGAGCACTAGCACCCGGACGTCCGGCACCCGCAGCGCGTCCGGGACGCATCTCCCAGTCCCCAG
CCAACCGAACATGGGCTGACGTCGTGGCCCACAAACGAGACCATCTTAGCATCTGCGTAAAATAGAAAACTAAAGGAGACT
GAAGCGCTACCTGCCCGCGTAACGAAAGGGGAGAGCGAGCGCTCGCGCCCATGCCACAATCCCCCGGCGCAACCCATCCG
CCGGCCCCCAGCGCACCCTCTTTGCTGACCCTCACTGCGCCACCTCTGCAGTATAAGAAGATAGATATGCAAGGCAGCAGAA
GGTGATAGGGAGACACCAAAGCGAGGCAGCAGAAAGCGGGGAGGAAGATGCAACACCCGATCTACTTTTGAAACATACAG
ATACAACACTTGCAACATATGTTTGAAGATAGATGAAACATTTAAAACATGCGTTTGAAACACTTACAAAAACACATGAAAA
AACAATTGCAACACATACGCAACATCCGGATAAAACGCTTACAACATATGTATAAAACATATGCAACATCCAGAAAAACACA
CTTACAATTCAACAGATGAAACATTGGGAACATACGTTTGCAACATATGTGTATAACCATAGCAACATACGCAACATCCCGAT
CTATTTTTGCAACATCCATACGAAACCCTTGCAACATACCTCTAAGACATATGAAACACTTGAAATATGAGCTTGCAATATGCG
CTTTTAGTGCAAACGCAATGTCACCTTGCTGCTTGGATGAATGGAGCTCGTCGCTGTGGAGCTTAACACTAGAGCGGAGCTC
GATGCCACGGAGTGCGTGGAGGTTGCCGGTGCGGAGCTCGTTGGCGGCATGGATCTCATCAGTGGCCACGACGGGAGGTGC
GATTACGGGGGGCGGCTTGGGCAAGGGTGGCGCCGGCATGCGGCGCGGGCAGAATCCGTCGTGCGACCGCGAGCGATGGC
GCACAGCACAGAGCGTCGCGAGCGACGCGGCGAATCGATGAGGATGAGCCATAAGGTCTGGACGGGAAACCACGCGAGCG
ATGTAGGCGAGTGAATGAGGATGAGGCACGTCTGGACGGGAAGTCACGGGATGGACGCCCGAGCCATATCATTATCGAAAA
AAATAAGATACTTCTTTCAATTATGAACCCAATGGTATCACTTTTGTGCGACAAAAAAATTACATTTTGCAAGGCTAATTGTGA
GTTGACAATTACGTGTTTTGAAATCTTAAAATGATTCCGCACGTTATAAACAATGAAGGGATTATACGTCATAATATGTCCATC
CAGCTGCCTCTCTATGTTGTTGAGCTAACAGTCCCCAAAGGCACACCAACACAACTACACAAGCAAGAGAGCAGCACAGAT
TGTGTCACGCACTGATCCTTGCTTTCATATATACTAATCCCAAACCAAAGACATGCATCATACTGGTTCCCAACACAAACAAT
TACTTGCACCATCTCTCTCGTGTAGCTTTCTCGCTCCTTGTACCATGCTTCTTTGAACTCCGTCGCCTCTCATCTCCCATCCAC
TGTCGCCCAGCTTTGCTGCCATCACCA
>SsLRR-RLK10
ACGAAAGGGGAGAGCGAGCGCTCGCGCCCGTGCTACAATCCCCCGGCGCATCCCATCCGCCAGCCCCCAGCGCACCCTCTT
TGTTGACCCTCACTGTGCCACCTCTGCAGTATAAGAAGATAGATATGCAAGGCAGCAGAAGGTGATAAGGAGACACCAAAG
CGAGGCAGCAGAAAGCGAGGAGGAAGATGCAACACCCGATCTACTTTTGAAACATACAGATACAACACTTGCAACATATGT
TTGAAGACAGATGAAACATTTAAAACATGCGTTTCAAACACTTACAAAAACATATGAAAACACACTTGAAAACCAATTGCA
ACACATACGCAACATCCAGATAAAACGCTTACAACATATGTATAAAACATATACAACATCCAGATAAACACACTTGCAATACA
ACAGATGAAACATTGGGAACATACGTTTGCAACATATATGTATAACCATAGCAACATACGCAATATCCCGATCTATTTTTGTAA
CATCCATACGAAACCCTTGCAACATACCTTTAAGACATATGAAACACTTGAAACATGAGCTTGTAACATGCGCTTTTAGTGCA
AACGCAATGTCACCTTGCTGCTCGGATGAATGAAGCTCGTCATTGTGGAGCTTAACACCAGAGCGGAGATCGATGCCACGG
AGTGCGTGGAGGTTGCCGGTGCGGAGTTCGTTGGCGGCATGGATCTCGGCAGTGACCACGACGGGAGGTGCGATTACGGG
GGGCGGCTTGGGCGGGGGTGGCGCCGGCATGCGGCGCGGGCAGAATCCGTCGTGCGACCGCGAGCGATGGCGCACAGCTC
AGAGCGTCGCGAGCGACGCGGGCGAATCGATGAGGATGAGCCATAAGGTCTGGACGGGAAACCACACGAGCGATGCAGGC
GAGTGAATGAGGATGAGGCACGTCTGGACGGGAAGTCACGGGACAGACGCCTGAGCCATATCATTATCGAAAAAATAAGAT
ACTTCTTTCAATTATGAACCCAATGGTATCACTTTTGTGCGACAAAAGATTACATTTTGCAAGGCTAATTGTGAATTGACAATT
ACGTGTTTTGAAATCTTAAAATGATTCCGCGCGTTATAAACGATGAAGGGATTATACGTCGTAATATGTCCATCCAGCTGCCTC
TCTATGTTGTTGAGCTAACAGTCCCCAAAGGCACACCAACTCAAGAGAAAAATTCACCAGCGGTCCCTCAACTTGGCAGCT
GGTGTCACTTAGATCCCCGAACTTTGAACAGTGTATTTCTAGGTCCCTGAACTTGGCTTCAGGTGTTATCTAGGTCCATGAAC
TTGGCTTTAGGTGTCACTTAGATCCCCGAACTTTGAACAGTGTATTTCTGGGTCCCTGAACTTGGCTTCGGGTGTCATCTGGG
TCCCAATGCTGATGTGGCGCGGTCCTACATGTGGGGCCCACATGTCAGTCGGTTATATTTCTATTTCGGGGATACTGTAGCTAC
AATTTCTAAAATATTCAAAAAATTGTATCTTTTTCATATGATCTCGGGTGAAGATGATTTTTATATAAAACTGTAGCTCTCGACG
AAATCTACAACTTTCATATAGAAAGGCACACCAACACAATTACACAAGCAAGAGAGCAGCACAGATTGTGTCACGCACTGA
TCCTTGCTTTCATATATACTAATCCCAAACCAAAGACATGCATCATACTGGTTCCCAACACAAACAATTACTTGCACCATCTCT
CTCGTGTAGCTTTCTCGCTCCTTGTACCATGCTTCTTTGAACTCCGTCGCCTCTCATCTCCCATCCACTGTCGCCCAGCTTTGC
TGCCATCACCATGGACATGCTCAACCTCTTGCTCCTCATATCCTCCATCTACGTGTTGTCAGCGTTTACACCGGTGGCGACAA
CTGACGCCGATGCCCTTCTCCGGTTCAAGGCCTCCATCCAGAAAGACCCTGGGGGTGTCCTCTCGTCTTGGCAGCCGAGCG

GGAGTGACGGCCCATGCAACTGGCACG
>SsLRR-RLK56
ATGAACTTGTCAAGGGATGCCAGTTAGGTTGTCCATGAACTTTAAAAATGCAATTTTGGGTCCCTAAACTTATTAAGTGGTGC
AGCGCATATCCCGAAACATGTAAAATGGTGCACCACAGTTCCATTTGTAGATCATATCGACCACTTTGATACACCATTACAAG
TTTAGGGATCCAGAAATGTGTTATCAAAGTTTATGGACCTAAGTAGCACCTATTGATAAGTTCAGAGACTATTCATGCATTTGA
CTCTAAACAATATGATGGAAAAGAAACCAACAATGGTCCAAATATATTTTCATGATTTTTACGTTCCACAACAGCATGTCTTC
AATTGTTGGATGGGAGGAGCGCTACGTCTCCAAATTTAAGACACGATTTGACCTATAATTTATCTTGTACTAAATCCTTCAGGT
TACAAAATCATACAAAAAAAAAAAATACAAGAGCACTATCGCCCGGATGTCCGGTGCCCACAGCGCGTCCGGGACGCATCTC
CCAGTCCCTAGCTAACCAAACATGGGCTGGCGTCGTGGCCCACAAACGGGACCATCTTAGCATCCTGCGTAAAATAAAAAA
CTAAAGGGGACTGAAATGCTACCTGCCCGCGTAACGAAAGGGGAGAGCGTGCGCTCGCGTCTGTGCCACAATCCCCCGGCG
CATCCCATCTGCCAGCCCCGGTGCACCCTCTTTGCCGCCCCTCGCTACGCCCCCTCTGCAGTATAAGAAGGATAGATATGCAA
GGCAGCAGAAGGTGATGAGGAGACACCAAGGCGAGGCAGCAGAAAGCGAGGAGGGAGATGCAACACCCGATCTACTTTT
GAAACATCCAAATACAACACTTGCAACATACATTTGGAGACAGATGAAACATTTAAAATATGTGTCTAAAACACTTACAAAA
ACACCTGAAAAAACACTTGAAAAACAATTGCAACACATACGCAACATCCAGATAAAACGCTTGCAACATATGTATAAAACAT
ATGCAACATCCAGATAAACACACTTGCAATATAACAGATGAAACATTGGGAACAGACGTTTGCAACATGCGTGTATAACCAT
AGCAACATATGTAACATCCTGATCTATTTTTGCAACATCCATACGAAACCCTTGCAACATACCTCTAAGACATATGAAACACTT
GAAACATGAGTTTGCAACATGCGCTTTTAGTGCAAGCGCAATGTCACCTTGCTGCTTGAACAAATGGAGCTCATCGTTGTGG
AGCTCAACACCGGAGCGGAGCTCGATGCCACGGAGTGCATGGAGGTTGCCGGTGCAGAGCTCGTTGGCGGCATGGATCTCG
GCAGTGGCCACAACGGGGAGGGGGGCGGCTTGGGCGGGGCCGGCGCCGGCATGCGGCGCGGGCAGAATCCGTCTTGCGA
CCGCCAGCGGGGGCACACAACACAGAGCGTCGCAAGCGAATGAATGAGGATGAGCCATAAGGTTTGGACGGGAAATCACG
TGAGCGACGCGGGCGAGTGGATGAGGATGAGGCACGTCTGGACGGGAAGTCACGGGACGGACGCCCGAGCCATATCATTAT
CGAAAAAATAAGATACTTCTTTTATATGAACCCAATGGTATCACTTTTATGCGACAAAAATTACATTTTGCAAGGCTAATTATC
AGTTGACAATTACGTGTTTTGAAATCTTAAAATGATTCCGCGCGTTATAAACGATGAAGGGATTATACGTCATAATATGTCCAT
CCAGCTGCCTCTCTATGTTGTTGAGCTAACAGTCCCCAAAGGCACACCAACACAATTACACAAGCAAGAGAGCAGCACAGA
TTGTGTCACGCACTGATCCTTGCTTTCATATATACTAATCCCAAACCAAAGACATGCATCATACTGGTTCCCAACACAAACAA
TTACTTGCACCATCTCTCTCGTGTAGCTTTCTCGCTCCTTGTACCATGCTTCTTTGAACTCCGTCGCCTCTCATCTCCCATCCA
CTGTCGCCCAGCTTTGCTGCCATCACCAT
>SsLRR-RLK57
AGCTATAAATAATAGAGTTAAATACATCAGCGATCCATGAACTTGTCAAGGGATGCCAGTTAGGTCGTCATGAACTTTAAAAA
TAAATTTTGGGTCCCTAAACTTGTTAAGTGGTGCAGCGCACATCTCGAAACGTGTAAAATGGTGCACCATAGTTCCATTTGTA
GATCATATCGACCACTTTGATGCACCATTACAAGTTTAGGATATAGAAATGTATTTTCAAAGTTTATTGACCTAAATAGCACATA
TTGATAAGTTCAGAGACTATTCATGCATTTGACTCTAAACAATATGATGGAAAAGAAACCAACAATGGTCCAAATATATTTTC
ATGATTTTTACGTTCCAAAACAGCATGTCTTCAATTGTTGGATGGGAGGAGCGCTCGTCTCCAAATTTAAGACACGATTTGAC
CTATAATTTATCTTGTACTAAATCCTTCAGTGTTACAAAATCATAAAAAAATACGAGAGCACTAGCACCGGATCCGGCCCCAGC
GCGTCCGGGACGCATCTCCCAGTCCCTAGCCAACCGAACATGGGCTGGCGTCGTGGCCCACAAACGGACCATCTTAGCATC
CTGCGTAAAATAGAAAACTAAAGGGACTGAAGCGCTACCTGCCCGCGTAACGAAAGGGGAGAGCGAGCGCTCGCGCTGCC
ACAATCCCCCGGCGCATCCCATCCGCCAGCCCCGCGCACCCTCTTTGCCGACCCTCACTGCGCCACCTCTGCAGTATAAGAA
GATAGATATGCAAGGCAGCAGAAGGTGATAGGAGACACCAAGCGAGGCAGCAGAAAGCGAGGAGGGAGATGCAACACCC
GATCTACTTTTGAAACATACAGATACAACACTTGCAACATACATGTTGAAGACAGATGAAACATTTAAAACATGCGTTAAAC
ACTTACAAAAACACCTGAAAAAACACTTGAAAAACAATTGCAACACATACGCAACATCCAGATAAAACGCTTACAACATAT
GTATAAAACATATGCAACATCCAGATAAACACACTTGCAATATAACAGATGAAACATTGGGAACATACGTTTGCAACATATGT
ATAACCATAGCAACATACGCAACATCCCGATCTATTTTTGCAACATCCATACGAAACCCTTGCAACATACCTCTAAGACATATG
AAACACTTGAAACATGAGCTTGCAACATGCGCTTTTAGTGCAAGCGCAATGTCACCTTGCTGCTTGGAAATGGAGCTCATCG
TTGTGGAGCTTAACACCGAGCGGAGCTCGATGCCACGGAGTGCATGGAGGTTGCCGGTGCAGAGCTCGTTGGCGGCATGGA
TCTCGGCAGTGGCCACAACGGGAGGTGCGGGGGGGGCTTGGGCGGGGGGGCGCCGGCATGCGGCGCGGGCAGAATCCGT
CTGCGACCGCGAGCGGGGCACAACACAGAGCGTCGCGACGCGAATGATGAGGATGAGCCATAAGGTTGGACGGGAAATCA
CGTGAGCGACGCGGCGAGTGGATGAGGATGAGGCACGTCTGGACGGGAAGTCACGGGACGGACGCCCGAGCCATATCATT
ATCGAAAAAATAAGATACTTCTTTCATATGAACCCAATGGTATCACTTTTATGCGACAAAAATTACATTTTGCAAGGCTAATTA
TCAGTTGACAATTACGTGTTTTGAAATCTTAAAATGATTCCGCGCGTTATAAACGATGAAGGGATTATACGTCATAATATGTCC
ATCCAGCTGCCTCTCTATGTTGTTGAGCTAACAGTCCCCAAAGGCACACCAACACAATTACACAAGCAAGAGAGCAGCACA
GATTGTGTCACGCACTGATCCTTGCTTTCATATATACTAATCCCAAACCAAAGACATGCATCATACTGGTTCCCAACACAAAC
AATTACTTGCACCATCTCTCTCGTGTAGCTTTCTCGCTCCTTGTACCATGCTTCTTTGAACTCCGTCGCCTCTCATCTCCCATCC
ACTGTCGCCCAGCTTTGCTGCCATCACCA
>SsLRR-RLK98
AATTTTTATACTACTCCCTCCATTTATAATTTATAAGACGTTTGGTTTTTATGTTTTTTTATGTACGTGGATATACACTATATCTA
GATACACAGGAGAAAAAACAATATGTCTAGGAAAATAAATCAAAATGTTTTATGATTCGGAACGAAGGAATACTTTTTTTATA
AAGTTTGACTGGATTGTACTCCGTATTATTTTGTACGCAAAGGTAGTATTTGATTGTCTCTAAATATAAATAAATACATTTGTTG
CTATATACTGTATCTAGATAATAAATCTTAGTACTTGTCTACAAATCTATAGTAGTACTGGTACATCAGAGATACAATTATTTATT
TTTGAGATCGGGGTATCTGCGCTTGGGAAAGAGGAGGCGTGAATTGATTATACTCATATATTAATTAATTAATGTAGCAGAGA
GATTGTATGGCTCTGACAGCCAACAACCAGTAACCTCCGCCGCAAACTTGTGGCTGCTGCCGGCGAGAGCTATTCCGTTTCT
TCGTTTTCGTTTTCCAAAAACAACTTTGATTAAATATATATTAAAAATATTAATATTTATGGTACATAATTAGTATTATTAGAAAG
ATTTTTAAATCTAGTTTTTTTTAAATATTAAATTTATTTGAAGATAGAAAATGTTGCATATATTTTTTATAAATCAACTTAAACTT
ATAGTATGTACATGACAGATATTTAGGGACAGACGGAGCACACTGGTTATGGCCTACAGGCTACAGCTACGCTGGGTGCGCG
GCATGGAGGAACGGAACGGGACGGAAACGGGAATCACACTCCAACCGCCCGCAGCCCACTGTGGCTAGGCGACTGCTGAC
TGCATTCAACTCAGACCGACTGCAGCCGTTCCGGTAAAATCGTCCTGCAGGACGCGGATGGCTGCACCGGCGTACGCTACG
CCACACGGTATCGTAGCGGATGGGCGGACGTCCGCTACTCCCGGCGGATGAGCAGAGCGTCCGCTACGCGCCTGCGTGGTG
CGGGCCCGCGCGGTCTCGTCCTTCACTCGTCGTGCGGCGTCGGGAGGGAGTGGGTAGGGGGGAGTTAAAAAAAGAAAAAA
AATCTGATGCATGGGTCCCATCTAAGTATAGAGGATGAATATAGAGGATAGATGGATGCGGAAGAAAATGGTATAGAGGAGA
GAATCTCGATGACCAGGATAAAATATTCCTTTTAGAGGATGAGTATAGAGGATAAAGAGTGCGGATAGCCTCAACTCTTCCAT
TCTCCAACAAAAGACTGCGTCTGACCTCACTGACGCACGCAACCTCTCTGTCGCTCTCCTCCCGACGCAGGCAAGCGGCAC
GCACAGCTGCACACGCAACGCAATACGAGGAGGAGGTGGACGGGGATCGGGGACGGAGGGCTGCGTGGACTTTGGAGTCT
GGACAGCAGAGCAGACTGCCATCCCCGCCACCTCGCACGCGCACACACCTTTCTCCCTCCCTCCCTCCCTCCGGTTCCTCCT
TCCCTTTCCCTGCTCTGCTTCGCTTTCAACCAACCTACTCACGCCCACTCCACTCCCCTCCTCGCACTCTCCATCATCACCAC
CGCAAGCCCCTGCGCTGCGCTCTCCCTCCTTCCTCCACGGCACGCGAGAGCAACGTGCCAAGGAGCAGAATTCCACAACGC
CGCTGCCGCTTGCATCGGGATGCCGCCGCCGCCGAGCCCGCATTGCTCCCCTCTCTCCTCCTCCGCCGCCGCCGCCGCGTGA
CCGGCTCCCTTCCATTCCCGCAACCACACAGTCGCGCCTCCGCGGGTGCCAGCCCAATCCCCTGAATCCTGAATCCGAATCC
ATGGCACCCAAGCGCGCGCCGCCACTGCTCCTGCTCCCGCTCCTCCTCGCCTTCCTCCTGCCGCTGCTCATCCCGCGGGCGC
GCGCCGCTGCGGACCCTAACCCGGCGCCGTGCCACCCGGACGACCTGCGCGCGCTGCTTGCCTTCGCCGGGAACCTCACGT
CCGCGGCGGACGCTCTCCT
>SsLRR-RLK77

GTTCCTTGGTCCTTGGCTCCGTGCTGTGCCTCAGGACAAATCGATTTCAAGCTGGATGGATGCATACGGTGGCAACCTTGGT
CCAACAGTCAAATTACCATGGTCCTGTTCGGCAGGCTGTTTTTTGGCTTATAAGCCGGCTGTAGCTGATTTGCTGTGAGAGA
AAAACACTGTTCCACGGCTGATAAGCCGTGCTGATAAGCTCAGCCGAACAGGGGGCATTTCTAGTTCAAGTGGCGGTTACA
CCCTCCCTCCGTCCGTGTCTGCAACATACTGTAAATTTAATTCATAATAACAATAATAGCATATCAAGCCAAACCCTTGTAACT
TTGCCTCGAATTTATACACACAACGAAATATGAATATTTATGCCATACTTGCATCGTTTTAAATTGTAAGTCATTTTTAGATACA
CAGCTAGTGTTTGGGAGCACTGAAACATTTGTTCCCGTTGCGGGTGGCTTGGATACACTTGAGCTTATTCAGTGCTACTTTTT
AGTCATGGAACAATATTTTCTTCTCACAACATTTTAGCATAAACATAAGCGTAAGCCAAATTTCAGTATAAGCGAACAGGGGT
TGCAGCAACAGTTGCGTTTAGGTGCAACGAACATGTCTACAGATATTTACTTCGCATCTAGACATAACCCATATCTAGGTGCA
CTACAAAACCTTTGAACCTAAAAAAAAAATGCCAAAACGACTTATGATCTGGAACGTAGTGAATAATTGTTATGAAAATATATT
TGCATTATATATATAATATAATAATAATTTTTTATACTACTCCCTCCATTTATAATTTATAAGACGTTTGGTTTTTATGTTTTTTTTAT
GTACGTGGATATACACTATGTCTAGATACATATGAGAAAAAACAATATATCTAGAAAAATAAACCAAAATGTTTTATGATTCGG
AATAAAGGAGTACTTTCTTTATAAAGTTTGACTGGATTGTACTCCGTATTATTTTGTACACAAAGGTAGTATTTGATTGTCTCTA
AATATAAATAAATACATTTGTTGCTATATACTGTATCTAGATAATAGTAAATCTTAGTACCTGTCTACAAAGCTATAGTACTAGTA
CATCGGAGATACAATTATTTATTTTTGAGATCGGGGTATCTGCGCTTGGGAAAGAGGAGGCGTGAATTGATTATACTCATATAT
TAATTAATTAATATAGCAGAGAGATTGTATGGCTCTGACAGCCAACAACCAGTAACCTCCGCCGCAAACTTGTGGCTGCTGCC
GGTGAGAGCTAGTATGGCGTGCTGGGTGCGCGGCATCGAGGAAGGGAACGGGACGGAAACGGGAATCACACTCCAACCGC
CCGCAGCCCACTGTGGCTAGGCGACTGCTGACTGCATTCAACTCTTCCATTCTCCACCCATGACCCAACCACACCACAAAAG
ACTACTCTGACTGCATCTGACCTCACTGACGCACGCAACCTCTCTCTCGCTCTCGCTCTCCTCCCGACGCACAGCTGCACAC
GCAACGCAATACGAGGAGGAGGAGGAGGTGGCCGCGTGGACGCCGGACGGGGATCGGGGACGGAGGGCAAGCGAGAGTG
GACTTTGGAGTCTGGACAGGACTGACGCTCCCTCCGTAGCAGAGCACCTCGCACGCGCACACACCTTTCCCCCTCCCTCCG
CCTCCTCCTTCCCTTTCCCTGCTCTGCTTCGCTTTCGACCAACCTACTCACGCCCACTCCACTCCCCTCGTCGCACTCTCCATC
ATCACCACCACCACAAGCCCCTGCGCTGCGCGCTCCCTCCTTCCTCCACGGCACGCGAGAGCAACGTGCCAAGGAGCAGA
ATTCCACAACGCCGCCGCCGCTTGCATCGGGATGCCGCCGCCGCCGAGCCCGCATTGCTCCCCTCTCTCCTCCTCCGCCGCC
GCCGCGTGACCGGCTCCCTTCCATTCCCGCAACCACACAGTCGCGCCTCCGCGGGTGCCAGCCCAATCCCCTGAATCCTGA
ATCCGAATCCA
>SsLRR-RLK140
CAACACATTGTAAATTTAATTCATAATAACAATAATAGCATATCAAGCCAAACCCTTGTAACTTTGCCTCGAATTTATACACAC
AACGAAATATGAATATTTATGCCATACTTCCATCGTTTTAAATTGTAAGTCATTTTTAGATACACAGCAAGTGTTTGGGAGCAC
TGAAACATTTGTTCCCGTTGCGGGTGGCTTGGATACACTTGAGCTTATTTAGTGCTACTTTTTCATTCATGGAACAATATTTTT
CTTCTCACAACATTTTAGCATAAACATAAGCATAAGCCAAATTTCAGTATAAGCGAACAGGGGTTGTAGCAACAGTTGCGTTT
AGGTGCAACGAACATGTCTACAGATATTTACTTCGCATCTAGACATAACCCATATCTAGGTGCACTACAAAACCTTCGAACCT
AAAAAAAAAAGCCAAAACGACTTATGATCTGGAACGTAGTGAATAATTGTTATGAAAATATATTTGCATTATATATATAATATA
ATAATAATTTTTATACTACTCCCTCCATTTATAATTTATAAGACGTTTGGTTTTTAGATTTTTTTATGTACGTGGATATACACTATG
TCTAGAAACATAGGAGAAAAAACAATATGTCTAGAAAAATAAACCAAAATGTTTTATGATTTGGAATGAAGGAGTACTTTCT
TTATAAAGTTTGACTGGATTGTACTCTGTATTATTTTGTACACAAAGGTAGTATTTGATTGTCTCTAAATATAAATAAATAAATTT
GTTGCTATATACTGTATCTAGATAATAGTAAATCTTAGTACCTGTCTACAAATCTATAGTACTAGTACATCGGAGATACAATTATT
TATTTTTGAGATCGGGGTATCTGCGCTTGGGAAAGAGGAGGCGTGAATTGATTATACTCATATAGTATTAATTAATTAATGTAG
CAGAGAGATTGTATGGCTCTGACAGCCAACAACCAGTAACCTCCGCCGCAAACTTGTGGCTGCTGCCGGTGAGAGCTAGTA
TGGCGCTATGACTTATGTGTTTAGTTCGCGACAAATTTTGGCTTTGGTTACTGTAACATTTTTATTTGTATTTATCAATTAGTGT
CTAATCATAGATTAATTAGGCTTAAAAGATTCGTCTCGTGATTTTTTACCAAACTGTGTAATTAGTTTTTTTTTACATTTAATGCT
CTATACATATGTCCAACATTCGACAATGTTGAAAAATCTGGGTAACTAAACACCCCATGCAGGCTACAGCTACGCTGGGGTGC
GCGGCATCGAGGAAGGGAACGGGACGGAAACGGGAATCACACTCCAACCGCCCGCAGCCCACTGTGGCTAGGCGACTGCT
GACTGCATTCAACTCTTCCATTGTCCACCCATGACCCAACCACACCACAAAAGACTACTCTGACTGCATCTGACCTCACTGA
CGCACGCAACCTCTCTCTCGCTCTCGCTCTCCTCCCGACGCACAGCTGCACACGCAACGCAATACGAGGAGGAGGAGGAGG
AGGTGGCCGCGTGGACGCCGGACGGGGATCGGGGACGGAGGGCAAGCGAGAGTGGACTTTGGAGTCTGGACAGGACTGA
CGCTCCCTCCACAGCAGAGCAGACCGCCATCCCCGCCACCTCGCACGCGCACACCTTTCCCCCTCCCTCCGGCTCCTCCT
TCCTTTCCCTGCTCTGCTTCGCTTTCGACCAACCTACTCACGCCCACTCCACTCCCCTCCTCGCACTCTCCATCATCACCACC
ACCACAAGCCCCTGCGCTGCGCGCTGCCTCCTTCCTCCACGGCACGCGAGAGCAACGTGCCAAGGAGCAGAATTCCACAA
CGCCGCCGCCGCCGCTTGCATCGGGATGCCGCCGCCGCCGAGCCCGCATTGCTCCCCTCTCTCCTCCTCCTCCGCCTCCGCC
GCGTGACCGGCTCCCTTCCATTCCCGCAACCACACAGTCGCGCCTCCGCGGGTGCCAGCCCAATCCCCCTGAATCCTGAATC
CGAATCC
>SsLRR-RLK226
TGTGACATGGTGACCTCCTGTTAATGAAGAGGCTTACGAAGCACCAAAGAAGCAATTCCGAAAGCTGATTCAGAACATGCA
TTCTTCTCTCTTCTCTCTCTAGTTTCTCTATCTAGTTTACTTGAATCTCGGGGACGAGATTCTTCTAAGTGGGGTAGATCTGTA
ACACCCGTATTTTGCATCATGTTAAAAACCACTAAAAATTTGAACTTTTAAAAATTTTATAACAAATTAAAACCTTGACTATTT
ATTTCCTGCAAAATCCTTTTCTAAATAAAATGATTTCCAAAACCCTTGCATCAAAGTTCACCATATGCATTCTCCTTGCCTTGC
GATTGTTTGACTATTTGTTTGACTGAATGCTTGCTTGCTTGAATGCTTGTATGCTTGCCCTTCTAGAATTTATTGCGGCACCAA
AATAGTATTAACTGGACCCTTAGTGTAAGGACCTTTCTCAATAATACAATAGTAGATAATACACCCTGTTAGTTTGCTATTCAC
AATATTAGTTATATTTAGTTTCTCTCTCTATATATATGCATGTTATATTCCTTAGCAGTATATCCCTTGCCATAGTATAAAAAAAAT
GCCACCTCTTCCTTTTAGTTATACATACAGGTCATGCCATAGTCATACACACATATGCCTCCCTTCAATTCCGTAGTAGCATATG
TATACATACTTTTAATCATACATATATCGTATGCATACATATACTTGCCGTGAATCATATAGATGTATATATATTGCTCTAGTTTTAC
ATACATCATATACCGACATGCACTAGTCGTATATACATGCCTAGTTAATACATATGTATATCCATATGTATGTACATATATGCACTT
CTGAGATTTTGAAAGATGCAAAAGCAACTACTGTGTACGCCTTCCTCCCAAACTGGCTTGAAGTGCTAGCCTCGGCACAGG
CAGAATAGCGCAGGACACAGCCCAGCTCAGCTCGGCCAAAGCCGAGCAACGCACACGACGCCGTGTCTACTACGCGTCGA
AACCTCGTAAACGCCGCGGAAGCTGTCTTCCACCTTGCGCACGACGGGATATTTTCCGGCAGTCAGCTGACTCACTGTGCG
AGGTCGCCACGGCCGCCATGTCACGTCCCGTCGAACAGCTGGAGCTTGCGACAACCCCTGGCCACCGAGATATTTCTTCGC
GACCGCCTTACTGTGACGCGAACTCGGCTATAAATAGCCGGCCATCGCCACTGCCGCGAAGCACTCGACGGAGCTCGAGGA
GGAGGTGCCCGACGAAGTCTGGGACACATGAAGCTGGAGAACGAGCTCTGCTTCTCTTCGGCTGCTTGCTCCAGCAACGTC
GACGAGCCAGCCCTTGCCCTCGCCGTAACGCGGACCCAGCCCTCGCCTGCGAAGCTCNNNNNNNNNNNNNNNNNNNNN
NNNNNNNNNNNNNNNNNNNNNNNNNNNNNNNNNNNNNNNNNNNNNNNNNNNNNNNNNNNNNNNNNNNNNNNNNNNN
NNAAAGCTCTCACCCACCCTATGAAAAATTTAGCATTTTGGATGAAACTAAACACACCTTTAAAATTTTGGCATTTTGCATTT
CATCATTCCAAAGTAGTTGGTATTTTGCATTTTGCATTCCATGAGGAACTAAACACGGCCTAAGAAACTTTAGAGATGGGTCT
CAAATCATGACAGGTACTACCTACTTTATAAATCACTTACCTGAAGAATTAGGACAGGTCAGGTCAGGGATGCTTAGCATTAA
ACTTGAGCTGCAGAGTCAAACCCAGAGTACCTTCTCTCTCTTTTCCTTCTCACTCCCTTCCTCTTTCTCTCTCTCACTCACTTC
TCTTCTTCCTGCAAGGGAACAAGAAGAGCAAGGCAGCAAGCAAGACTGAAGAAGGACGATCTGACCATGATCCCAAGATG
TGAGAGAACACATGGCGCTTGCTTATCGTCACCATTAGCTAGCTAGCTACTAGCTCCTTGTCGACGTTGTCGTGTGGCAAGG
CAGGCCGGAGATCGACA
>SsLRR-RLK261
AATAATAATGGCGCTAGTAGCACATTTTGATTTAGAATTACATCAGATGGATGTTAAGACGGCATTCCTAAATGGGGATCTTGA
GGAAGATGTCTACATGGCACAACCGAAAGGTTTTGTCATGGAAGGAAATGAGAACATGGGGTGCCGCCTAAGGAAGTCTAT

TTATGGACTAAAACAAGCTTCAAGACAGTGGTATTTGAAGTTTGATAATACAATAAGAAAGTTTGGGTTTAAGGCAAATGTA
GAGGACAATTGTGTTTATGTAAAGTTTAAGAATTCTAAGTTCATCTTCTTGTCTATATGTGGATGATATTTTGCTAGCAAGCAAT
GATGTCACCTACTAATGGAGACTAAGAAGTTCTTGTCCTCGCATTTTGATATGAAAGACCTCGGTGAAGCTTCTTTTGTTCTG
GGGATTGAATTCACCGTGATAGAAATAGAGGGGTATTAGGGCTGTCACAAAAGGCATACATAGAAAAGATTCTTAAGAAATA
TAGTATGCACAAGTGCAGTCCATCACCTGCACCCATAGTCAAGGGCGATAAGTATGGGGAACACCAGTGTCCCAAGAATCAG
ATCGAGCTCGAGCAAATGAGATCAGTACCTTATGCTTCAGCCGTCGGAAGCTTACAATATGCTCAAATTTGTACTCGCCCTGA
CATAGCGTTTGTTACCGGGTTACTTGGCAGATATCAGAAAAATCCAGGTGCAGAACACTGGAAGTTAGTTAAGAAAGTCTTG
CGTTACTTGCAGGGCACTAAAGGCTTCATGCTAAGTTATAATAAGACGGACTCCCTTGAGATAGTGGGGTTCTCAGATTCAG
ATTATGCGGGGGATGAAAGGAAATCCACGTCAGGCTACATATTCACCCTCGCAGGTGGAGCCATATCGTGGAAAAGCTCCAA
ACAGACTCTCGTCACGTCATCGACGATGTACGCTGAGTTTGTAGCATGTTATGAGGCCTCGGGGCAGGTGAGTTGGCTTAAG
AAGTTTTTGCCCGGATTAAGAGTGGTAGATAGCATTGAGAGACCACTTAAGTTATACTGCGATAATAATCCCGCAGTTGAATA
TGCTCACAATAATAGGTCAAGTGGTGCTGCCAAACACATTGACATCAAGTACTATGTTGTGAAAGAGAAAGTTCAGGATCAT
GTTATAAGCCTTGAGTATATTAAGACGGATAATATGTTAGCGGATCCGCTCACAAAAGGCCTACCACCCAACGTGTTTAGAAA
ACACCTAGCCGGCATGGGGTTAAGGGAAAGCCTATGATTCCTGAACACTTAGGGCCCTTTAGTTAGAACTTTATCTCAAATTA
GAGAGATGTACTGTAGCCGTCAAGTCCAACAGAAATTGTTTCTGTGACGACGAGACGCGCTCTACGCATTTCTTTATAATGA
GACGGAGTTAAGTATTTAAATTAAGAATAAGACAAGGTTAAGATAAGTTATTAGAAGAGATCAAGGGGAGAATGTTAGGGTT
TTGATCTCTCCTCCTATTGGACCCAACGGCCCATTAGGGACCTTGATCCGCTCCTTGATCGGGGGAGCCCAACCCACCTATGG
TTGGAGGGCCCCTGTCACGTGCGGTAAATAAATAGTGGTGGGAGGCCGGGGCACGGTACACAAGGTTCACCGCCGCCACTG
TTCCCACCTACAAACCCTAGACCCTACTCCGTTCACAGGGACCGCACAAGTGACGGGAAGCCCTTCTCCGCCGCCACTGCG
CCTCCAAGCCCGACATGGCGGACTCCAGCACCTCGGCGCCTAAAACTGACGGTCGACACTTCTCCCTATCTCCTTCTTCTTG
CATAAAGGACAAGCTCTTTCTCTCGTTTTAGTTAAGCACCGATTAATCTCACTGTTAGTAGATCTANNNNNNNNNNNNNNNN
NNNNNNNNNNNNNNNNNNNNNNNNNNNNNNNNNNNNNNNNNNNNNNNNNNNNNNNNNNNNNNNNNNNNNNNNNNNNNNNN
NNNNNNNNGGCACAGAGGATGCACAAAGAC
>SsLRR-RLK418
AAGTAGCAATATAGTGATATTTCAACCAAAAAAACATAATCGAAAAGGTTTCTACCTGATCTGTTTGCAGAAAGGACTCGAA
ATAACTATAATAGAAAAAAAAATCAATAATAGAGGTTCATAATGAAACCTAGAAAAAAGGTCAAAGGAGATAAACTAGATCC
AACATAGTGATTGATATTCATATCATTGAGAAGAGAAACATCCACTTAATAAAAAAACAAAATATAAATTATTCTCTTTCTCCC
AACCACGATTTGAGCGGTTGACGTTGCTTCCCACTTTACAACAGCGGATGGGCCAGGGATACCTTAATACCTAGCGTCGGAT
AAGACGATGGTAAGTTCATGCTAAGAATGTTACAAAAGCATGGTAGTGTTAAAAGTGGGAAGTAAGTTCATGCTACATGCTC
AATGGCAGAGACCGGAGAGATGAGTGCAACTAAAAACTATAGACCTAGTAGCCCTTATTATGATCACCGAAATCAATGAAGA
TAACAGTCAACATGGGATTATGAGTGTTGCATCACAAACATGGCTCCATAGCTTCAACCAAGCAATAGAGCAAGATCAATAT
GCAGAACTTATGCTAAAACTGCAACCTTTTGACAACGACAAGGAATGCGAAAATTAAAATTCCCTTAGGGCCCATTTGGTTA
TCACGGATTTGACCCCTGGAACAATTCCTAGCCGGATTCCTTCTCTAATTTATATAAGATTTCATCATCTGGAATAATCCCTGG
GGGAATCCAGACTAACCGCACAGGGCTTTAATAGTTTAGATTGAGGAGATATGAATGTACGAAATGGTACAGTAAAATGAGT
ACACTAGGCTTAAAAATGTAGCTCGAGGTCATAGCGGTCATGAAATGTAGGTGCAAATGTTGATGTTATTATAACACCTATTCT
AGCAACAAAGAACACTAATAAAGTCATGAAGAGCTAAACATGGAGCAAAGTAAGTTAGAATGGTACTAACCATATTGCGCTA
CATATAAACCACGTGCTGCAAACTCTTCCAAAAAAACACATGACCACCACACTAGTTAGAACTTCGTACCTACGCCTTTTTTT
TCTAAATTTTAGGTTATTTTGGTTTTTCTAGGCATATAGTTTTTGTTATGTACCTCAACATATTGTATATCTAGGCATATAACAAA
AGGCTATTTAATATAATAGTTAAAGAAGCCAAAACGACTTATAATTTGAAACAGATAAAATATCTTACAAAAGAAACAAATTG
CTCGCCAGATAGAATGAAAATGCATACTATCTATTCCTTGTAGACTTAGAAGTCTTAATAATGTAACATTCTGTTATAAAAAAA
CTCAAAATCATGACAGAGAAATATCAAATACTTACGTCAGGCTTGCACCTTCTAGCATTTTCATTCGCCAAGGACACCGAAC
GGCAACAGAAAAGAGCAATATCCCTAGAAAAACCATCCTGCAGTTGCCATCGCAACACGCAAGGCTAGTGCGACATCGATG
AAGTCAAAGCACCACTAATCTACTACTCGTACACGCACTTGATCCTAATATTCTCTCTGATCAACTAGCCATAACTTACCACTT
GTCAGTTGTAGACAATTGCAACTTGGTCCTTAATTAGAGGCTTAAACTGCCGCCCTTCACACGCGGAATCTCAAAAGTCACA
GATAGTTCAGCCACAAGGGGCAATCTCAAAAGTCAACCCTGCCCCTAGCCCTCCCTGCAAGTGCAAGGCTACAACGGAAAC
ATGACTTGCAGCGCATTTGCAGTTACTACTAGCATCATGTCAGCTGTCTGCGGTTGACCTGCATTTTGATAGGGCATGAGCCA
GTAATTGACAGTTGGATACTCCTATTCTTTTCATCAAGACTCAAAGTCAGAGAGCTGGGCATGAATCTTTGCATTAAGACAAG
ACAATTGGAAGCTTCTCTTTCGGATCCTCGTCTAGGGTTGAGAATTCTGTGATCCCATTGACTACAACCATGCGTAGCGCCCG
GAACTCAGCAGGTTC
>SsLRR-RLK395
TTAGTCCCTCCATGCAAAGGTGGTTCGGCCATATGGTCATTGTATCGTAGAGATTGGTATGGCTGGGCAATCACGGCCCTGTA
GTGTGTTGAACTTTTCAGTTGGACTACCGACTGCTGGCCTTGTAGCCTTCAGCGAGTGTTATGTAACATTCAGAGATGGACC
CTGGGGGAGAACTTTCTTGGCCTTGTGGCTTTGTTGCCTTCGGCCACCCCCGGTTGTGACTTTCTAGTTGGTTTGCCTGGGT
GCAACCGTTGTATGCTGGCAAATGGTAGTTGCAATATTTAATCAATGTCAGCAAGAGATTGCATCATATTATGTTGGATTTGGA
ATGTTCGAGGATTAAATGATGGAGCCAAGAAAGCGGCTGTGCGAAACCAAATAATTTCCTCTGAAGCCACAGTGGTATGTCT
ACAGGAAACTAAAATTGCAACATGGACGTATTCACTGTTAATAGAGATGGTCGGGGCAGACATGGCACAAAACTTTGTGTCC
CTGCCTTCCGCAGGTGCTTTCGGAGGAATTTTAATTGCAGTGTCAGATCGTTTCTTCGAACTTCAAAACCCCCGCGTCATGGC
AAATACTATCTCGGCTACTATCACAATGTTGGCCGAAAATATAGACTGGTCAATTACAGGAGTTTATGGTCTGCAGGGGAACA
ACGAGAAAATCTTATTCATGCAAGAGATCGCAGATCCAAAACAACAAATGATGCCTGCTTGGCTGCTACTAGGTGATTTCAA
CCTAATATACCGTGCTGAGGACAAAAAAACGGACGCCTTAATCTCCCTATGCTCAGCAGATTCAAGACTACCATTGATAATCT
CTTATTGGCGCCTTTTGATCTTAGGGGAAAGAAATATACATGGTGCAATGACCAACAATCGCCGACGATGACAAAAATAGATC
ACCTGTTCGTCACAGGTGAATGGCTAGATATTTTTCCAAGAACGGACCTCCATGCTCTTGCTTCCATAGGTTCCGACCATTGC
CCACTACTCCATGGAGATGTAGTTTTTGACTTCTATAGAGGATTTCGATTCGAAGCTTCCTGGACCAGATGTCCGGGCTTCTC
TGATGCAGTGCAAGAAGCTTGGAAACCGGTTCTCAAACAAGACGCGATACTGTGGGTTCAAATTACTGCGGACTGCAAAGG
CTTTGAAAAACTGGAGGCGGAAACAGTTCAGTGGATGGAAATTAAGTTGGGCAACGAAATAGAAGAATATTCTAACAAAAA
TCTCTACAACCAATCCAGGTAGCGTTACTGTGCAATGATGAAATTCAACAGTATCAAATGGCAACAAGACCATTCGTTGGAG
ATGAGTAGCAAGAATCGCTGTTTAGGAAGTGTGTTTTTTCTTCCTTGTTTTTGCTAGACCAGCTGAGTAGTGTGCTATGTCAC
TGCTGGTGTGCCAAAGTAGCTGGGAGCCTGGTCTCCATGTAAATAAGTTGTAATTTTGTTTTGCTTTGTGCTGCTTTTTTTTTT
CATCGTCTAATAAAAATCCGGCAAAGCTTTTACCGCCTTTCTAAAAAAACTACTACATGAGTACATGACACTGCTGACATACG
TACCCCGTTATGGCCTCATCACTGCAAGAACTCGCTACTGAACTTCGCCCCAAGTTTGTGTGGGAGTCACTTTCTTGTGTTTC
CAAAAATATAAGAAAATGCATCTCTTTGACATTTATCCTATATTGAGAAGACTTGGACTGGAACACCGAAGGTGTAATCTCCA
ATAGATAACAGGATTGGAAATGTTAGTGGCTCTGAGCTCTGACCGTACTTACCCAGCGACTTTTTTAGTGCTCTAATATTCTAC
TGTAACTTTGTGTGGTTGGCTTTCCTGATTTTTTTTATTTGCAGCGTTATGTAATTTGCGTCATCAACTAGCACAAGTTTTCTTC
TTCTTTCCATTGAGATCTTTAGATATATACAACAACCGAACTGCTCCAGTTTCATCAACAAGCATCTCTGCAGCAGGTGAGTT
TCATGAGAGAATC
>SsLRR-RLK407
GGTTTACGCCGGCGGCGAGCTGCTGTGTGGCGAGGTGGATGAACAAGAAGAGAAATGAACTGGACACCGTGAATTTGTGTT
GCAAAGCGCAACGTTTTCTGATGCATATATTTGATATAAATAGGGGAAACAAACTGATCATGCCGCTCATGGCGCGCACGACG
CGAGAGAGGTGCGCGTCCATCCCCACGCACGCACGACGTCCGGCCATGGCTGCATGTCTGACTATGTCTAACTACCTCTAAT
CCTGGCGCACAATCACCACAAGTCGGTGTCTGTGCGCATGCATAAAGGAGTGAACGAAAACATGCTTACTATCTATAGCACA

TAGGAGTATTCAACAAAGATGAACTTGTTGGCGGAGGTGCCGACAAGGAAGGCCGTCGGACAGCCGAAGAGGCGCAGCCG
CGACACGGGCCCGTACGTGGCCACACACCGCCGGAGCCAGTCATCGGCGGTGTTGGCCCGGAGGGCGCGGACCAGGCTCA
GCGTCTGCCGGATGAAAGGCAGACCGAATGAGCCCGGAGGCAGAGGTGCTTTCAGATTCGTCTTCTTGCCGCCGGCGCTGG
CAGAGAGGAGACGGAACAGTACGGGGACCGATGCTACAACCGCGAGGATAACGGCAACTGCAGCCGCCAATGTCGCCGGA
GCAGCATCCATTGCTTCCGATCAAGAGCTTGCACTTTGCAGTAGTACTTCCTTGTTAGGTTCCTATTCCACATTTCTTTCGAGG
TGATGCCTTGTGGTTGGAAGGTGGATGCCATTGCAGTAGGGACAAAATGATTAGTCATTAGTCAGCATATGATGACAACATAA
AGATAAACAAGAAACAAGATGATGAAAAAGACAGATAGGATAGGGAATGGTATATTGGTAATCTCTATATAGGCATAGCCCGC
TAGAAAAACAACTTAATGTTCCTTTCACTAGGGTGTTGCCTGTTGGGACTTGAACGCCAAGATAAATATTTCTCAGTATTATA
CATTGGTGCCATTTTTTTTTTAAAAAAAAGAAGTGAAGTTTTCAACGGAACAAAAATTTTTCTGTATTCCTGCATTTGCAAGA
GGCTCCAGAAGAATAGACAGACTAACAGGGGGTTGGGGGAAACTGCCCACATGACTTTGAATTTCACAAACAACTTGTGAG
ATGGTGTGTCCCTGTCTATGTTATGATGAATAATTGATAAGATGGTATGAGCCTTTTGGTTGTGTTCAATTGAGATTGACAATG
GTGGACTAATCAGAGATGTTGAGTTGATGAGTGTGTTGTGTGGAGGAAATGTATCTCTTGGTTTGTGATGTGTAGATATTGGA
TGCGATATGGTGGCTAACGCTGAGTGATTAAGGTCAAGCAAGTTTTAGCGTCGATAATCCAAAGGCAGTGAAGAACGAACA
AGGAGGTTTAGGACAGATCAACCAAGGAGTTAGTGTGTAGGCCATAGGCGATCCGCATCATCCTCAACTAAATTTATGGAGA
CAACATCGACTAAGTCAAGGTGGGCATCAAGGTCCACGAAAACATTTAGAGTAGTCATCATCAACTACTACATGAGTACATG
ACACTGCTAACATACGTACCCCGATATGGCCTCATCACTGCAAGAACTCGCTACTGAACTTCCGCCCCAAGTTTGTGTGGGA
GTCACTTTCTTGTGTTTCCAAAAATATAAGAAAATGCATCTCTTTGACATTTATCCTATATTGAGAAGATTTGGACTGGAACAC
CGAAGGTGTAATCTCCAATAGATAACAGGATTGGAAATGTTAGTGGCTCTGAGCTCTGACCGTACTTACCCAGCGACTTTTTT
AGTGCTCTAATATTCTACTGTAACTTTGTGTGGTTGGCTTTCCTGATTTTTTTTTATTTGCAGCGTTATGTAATTTGCGTCATCA
ACTAGCACAAGTTTTCTTCTTCTTCTTTCCATTGAAATCTTTAGATATATACAACAACCGAACTGCTCCAGTTTCATCAATAAG
CATCTCTGCAGCAGGTGAGTTTCA
>SsLRR-RLK419
ATTTCTGCATTTGACATCTACATTTAAGGTCATTTGTCTCCTAAATTTATTTTCAAAGTTTCAAAATTAAAAGTTTTTATTCTTTT
AAAGAAGAAACTATGCATTTGTATGTATAAAAAATCACATAAATATTTGGAATGATTAAAAAATTATATAAATATTTTTGGAATT
TTAAATTATATAAACCTATTTTTATCTAAATAGATTTGATTTTTTTAAAAAAACAACTGTAAGAGCGGCTGATAAACTCAACCG
CCCTTACTGAGCCACGGGCCAAATATAAGGAGGGGGTGCCCGAGCGGCTAAGTCATTGGGCGAAATCAACACATTTGGCGC
CGTCAGGCTACCAAAATCTGGCGCCGCCTCTCCTTCCCTCCGACGCGGCCTCCTCCACCTCCGGAGCCGCCTCCTCCCCTCC
CTGGCCCCTCCTGCGCCGCCTCCACCCCTCACTGACCCTTCCGCTGCCACCTCCTCCCTCTCCCGACCCTGCCGCCACCGCC
CCGTCTCCCGCACGTGCCGCGCGCCGTGGTTAGGGTTTGAGCCGACGGAGGACGCCGGCGGACCGTGAAGGACACCACGC
AGGGCTGCCGCGGTGAGGGTCACCGAACTCGACGCCCGCAAGTGCTGTGGCGACCTCCCGGCGGCGCCCCCAACCCGCTT
GTGCGCTCGGGCTGTCATCCCCGCCACCGACATTCTCTCCTTCATCGACGGTTGTGCTGCAGCCAGATCCCTGCGCACCACC
ACCGCGCGCCCGCCCCTCCCTCCTCCCCGCACCACACTAGGGTTTGAGCTTCCCCTCCCCTTCTCCATCCCGTCTTGTGCTTG
CAGTGTCTTGTGATGATGTTCATGAAGTTATTGGGATGAATTTTTGTTCATGATGAGCTTCACCCGCCACTGCCTATGATGCCT
TAACCCAAATCTAAGTACATGCTAATGCCACTACCTATGATGCATAAACCCAAATCTGAGCACATGTCATGCTGCCTTTATTGG
AATCTTTGATTTAAGTCTGAGTACACTAAATTGAGATGAATTGGGATGATAATTGTCTCATGTGTTTTGTCTAACCTATCAGTTT
GATCTATGCGTTCTCCAAGCGTGTTCTTTTGGGGCTGTTCTTGGTTCCTGTTTTTTAGTGCTCTGATCCTCCCAGGTTCCTGAA
TCTGTGTTGTCAGATTCTTGCTCGTTGATTGTGACCATAGCTGGATATGTATCGCCTGATTCTTGGCTGCGATTTAGTTTTGAA
GGTGGTCATATACTCGGTAGAAGCTTCAATATGAAGGCATTTTCAGTATCTCTGCTGCCATGACATTGTGATGATAAATCATTG
TTAGTACAAGCTTTGGTCTCGTACGCTCTGCAGTACACGGAATAAACCTTTTTTCACAATGTTTAATCCTTGTTTATGAGCGTA
AGCAATAATTTGGAGTTTGGTCACCTAAGTGTTTGAGTAGTAGCGATCATGATGTATTTTCTGTTACTTGCATGCATCATATGC
TGCCAGAGACTACGACATACACGACGAATATCAGCAAACTGACATCAAGTAGGGCCCACCCGTAATGCCCAATGCGACAAC
AAGGCCACTTGCATTTGTACGGAAGTACTTTAGCAATGCAATTTCTTATCTTTTGCTTATGTTCAAAATTGCCATTCAGTATATA
GGGAAGTTATGTTGTTTTTTCCCATTACACAATCTCGCACAGCTAAATGGTATACATGACAGCCAATGGCATAGGTAATTGTAA
CTGACAAATGCTACTTTTCTTTTTTCAGCTTTGAAATCCTAAAAAAATGAGAATGTAAATCTTCTANNNNNNNNNNNNNNNNN
NNNNNNNNNNNNNNNNNNNNNNNNNNNNNNNNNNNNNNNNNNNNNNNNNNNNNNNNNNNNNNNNNNNNNNNNNNNNNNNN
NNNNNNNNACAATCTTGTCGCTCTTAATGCAGCAACAACAGCTTTACAGGACCATTGCATCTTCTATCTGCATCAACGCCCCAT
CACTTGCCATG
>SsLRR-RLK236
TGAAACTAATTATAACCTGATCAGGTTCATAAGTTGTGGTCCAAAAAATCTAAGCATAGAGAATGAAAGGTGTTTGACAATCC
TGACTATTTAACAATCCTAAGTGTAAAAAGACTTTTATGCCCTTCTTCTTCCTCAGATCCCCACCTGTTTCCTTCGGCCGCCGC
CGTCGCAGCTGCCTCGCGATCACCACGCCGCCGTCATGGTTGACTCGTGCCCCACCACGCCGTCGTCCGCGGCTGCCTCGC
GGGGGATGGAGCTCCCTGACCACCCCGCTGTCTGTGACTGCCTCGCAGGGAGAAGGAGGCGCGCTGTGCTGCACCGGGG
GAGAAGGAGGTGCGTCGTTGGAGACTTGGAACGGCAAACAGAGAGGAAGAGGGGAGGGAGGGGAGGTCCGATCGCCGGT
GCGGGAGGCGGAGGAGCGGTCCGACGTGCGAGGGCTAACGGATGGAAATCGGCCCGCGGTGGCCGAAACGTGAGGGGTC
CCTCGCGGTACAGAGATCGCGAGACGTAGTTCAAAGCGCGATTTTTCCCACTGCTGCGATCAGAACAATGGAGAAATAATCG
GCGCGTTTAGGTGGGATTCTCGATTGTTTTGGTCCAGAATCTGATTATAAATCGAAAACAAACCCGCCCGAGCCGTTGCAGC
GGCTGCGGCCGGATGCTCAGCAGTGAAACCGAGGCGTCGCCGGCTCCGGCCAGGAGTTGTGGGCTTGTGGCCTCATGGGA
GCCCGAGAGAGAAGAGGGACCGTTTGCACCTGACGGCCCAGAACGTAGCTCATCTTTTTTTTTGGAAAGATAGCCCAGGT
TGACCTCAACAGGATTGCCGTTGTCAGCTTCTTTCTTTTTTTCTCCCCGGTTTCATATAAATCAATTCCTTTGATTTTTTTTTGA
AAATTCATTGCATAGAAAACTCCACAATAATTTGAGAGACCATTCCCTTTGTTCAGAGGGATAAAATTCTTCTCATATGACTTG
ATGCCTTTCAAAATGTTCCTAGTAGGGGATTATCATTATTTAGGGTCATATTTTAATTTTGCAGAAGTCACTTTCTGTTTGGTTC
AGCAACGCTAGTTGCCAAGTAAAGAAATGATGCACAGATAAACAACAGAATTTTGCAGAAGAATATCAGCCAGTGGCACCG
AACTACGACAGCCAAGTCAGAAAAATATAGATAGAAAAGTACAACATCAACTGCACCACCAGCTCCCAAACAAAACCGTCT
ACAATATACAAAAACATAGCCGCCAAAAAACAAATCCATGCAATATTTTCTGTTCAGAGCTGTTGTCCCCGTCTGAAACACC
GGCTGGGTTCATTGACTCACTTCTACACCTAGCAACAGGTCGTCGTCCAAAGCCTACCGCAAACCACTAGTTGCAGCAGAAT
GCAGAATTGAATTTGCAAAGGCATACATCAGCCACAGCTACTCTTGGATGATGACAACAGGTAGAGAAAAGTAAACGCCTT
GGGTCAAGAAGAAGCAAGAGCACTCAGCGATAATTCCTCCGTAAATCTGCAATCTTCGTCTGCTCAACTTGCCCTGCTGCAG
CCACGTCGATCATGACATGGATGGACGCCTGCTTTGAATCAGTCACCCCAAGAACACGGTAGGATCTCATTAAGAGAACAGA
AGGATAAGCAGCCCAAAAGGCATGAGCACCCTGCTTTGAATGCATGCTTTACATGATGCTCATGGATTCATACCTTGCAGTTG
CTGATTGAGGGAATTTCGTTGTTGCCCCGAAATCATGTCTGTTCCTGAACTAATCATGGATGACTGTTCCACTTACTTTCTGTT
TCACGCATGGAAGCCAGGCAACTCAGACACGACACCATGGTGAGAACTCACAAAACGCTGTAGCAACTAGTCCTTGACTAG
TATTTTCTTTTGGTGACTTGACAACGACCTGGCTGAGAGAATCAAACAAAACGTGAACAGCACCTGCTCTGTTTGTTCCTCA
ACCCTCATGCCCATTCTCCATCAGACGCAAAAAT
>SsLRR-RLK212
AATCGGCCCGCGGTGGCCAAAACGTGAGGGATCCCTCGCGGTACAGAGATCGCGAGACGTACTCCCTCCATTCCAAATTATA
AGACATTCCAACAATCTTGGAGAGTCAAAACATCTTAAGTTTGACCAAAATTATAGAGAAAATTATAAAAATTTATAACATCA
AATAGGTATACTATGAAAATATAATTAATGAAAAATCTAATAATACTTAATTATTGTCATAAATATTATTATCTTATCATGTAAATT
TGGTCAAACTTGATATCCTTTGACTCTCTAAAATTCTTGGAATGCCTTATAATTTGGGATGGAGGAAGTAGTTCAAAGGGCGA
TTTTTCCCACTGCTGCGATCAGAACAATGGAGAAATAATCGGCGCGTTTAGGTGGGATTCTCGATTGTTTTGGTCCAGAATCT
GATTATAAATCGAAAACAAACCCGCCCTTGCCGTTGCAGCGGCTGCGGCCGGATGCTCAGCAGTGAAACCGAGGCGTCGCC

GGCTCCGGCCAGGAGTTGTGGGCTTGTGGCCTCATGGGAGCCCGAGAGAGAAGAGGGACCGTTTGCACCTGACGGCCCAG
AACGTAGCTCATCTTTTTTTTTTTGAAAGATAGCCCAGGTTGACCTCAACAGGATTGCCGTTGTCAGCTTCTTTCTTTTTTTCT
CCCCGGTTTCATATAAATCAATTCCTTTGATTTTTTTTTTTGAAAATTCATTGCATAGAAAACTCCACAATAATTTGAGAGACC
ATTCCCTTTGTTCAGAGGGATAAAATTCTTTTCATATGACTTGATGCCTTTCAAAATGTTCCTAGTAGGGGATTACCGCTATTTA
GGGTCATAGTTTAATTTTGCAGAAGTCACTTTCTGATAGGTTCAGCAACACTTGTCGCCAAGTAAAGAAATGATGCACAGAT
AAACAACAGAATTTTGCAGAAGAATATCAGCCAGTGGCACCGAACTACGACAGCCAAGTCAGAAAAATATAGATAGAAAAG
TACAACATCAATTGCACCACCAGCTCCCAAACAAAACCGTCTACAATATACAAAAACATAGCCGCCAAAAAACAAATCCATG
CAATATTTTCTGTTCAGAGCTGTTGTCCCCGTCTGAAACACCGGCTGGGTTCATTGACTCACTTCTACACCTAGCAACAGGTC
GTCGTCCAAAGCCTACCGCAAACCACTAGTTGCAGCAGAATGCAGAATTGAATTTGCAAAGGCATACATCAGCCACAGCTA
CTCTTGGATGATGACAAAAGGTAGAGAAAAGTAAACGCCTTGGGTCAAGAAGAAGCAAGAGCACTCAGCGACAATTCCTC
CGTAAATCTGCAATCTTCGTCTGCTCAACTTGCCCTACTGCAGCCACGTCGATCATGACATGGATGGACGCCTGCTTTGAATC
AGTCACCCCAAGAACACGGTAGGATCTCATTAAGAGAACAGAAGGATAAGCAGCCCAAAAAGCATGAGCACCCTGCTTTGA
ATGCATGCTTTACATGATGCTCATGGATTCATACCTTGCAGTTGCTGATTGAGGGAATTTCGTTGTTGCCCCGAAATCATGTAT
GTTCCTGAACTAATCATGGATGACTGTTCCACTTACTTTCTGTTTCACGCATGGAAGCCAGGCAACTCAGACACGACACCAT
GGTGTGAACTCACAAAACGCTGTAGCAAGTAGTCCTTGACTAGCATCTTCTTTTGGTGACTTGACAACGACCTGGCTGAGA
GGATCAAACAAAAAGTGAATAGCAGCTGCTCTGTTTGTTCCTCAACAGTCATGCTTTTTCTCCATCAGACACAAAAATGGAC
CTATGGCTTCTCAGCGCACAGCTTATGCCTCTTTCTCCTATGTTCATATCTTGGGAGCGAGAAAGCATGCTGCCGCTCCAATTC
TCACCGAACAAATACAGCAGCAGATTTCCCACAATTTCCCTTGCCTTCGCTATTGTTCTGCTCCTCTTCTTGGCCTCTCCTGC
AAGCTCTTGCACCGAGCAG
>SsLRR-RLK259
CCCCCCTCTAGCCATCTAGGACCTTACACACTGCCATCTTCTCTAACTCCGGTGAGACTCCCCCGCCCTAACCTGACTCGGCC
ATCGCTACCACCCAGGGCTTAAACCGTTCCGTCACCATCCCCACCGTCTGTCCTAGTAGCCCCTTCTATAGCCGCCGGAGTTG
GAGAAGAAAGAGAGAGAAAGGCTCGTTTGAACCCTAGCCCATCGCCATCATCTACTCCTCTTCATCTTTGGTGGCCACAGAT
CCAGGAGGAGGAGCCTGCGTACCTAGTTGGCGCTGGCACCATCGGAACCCTAGCTGCCACGCCGGAGCTGGTGAAGAAGA
TGACGGACGCAGTCACGGACACACGCCGGCGCAAGTCGAGCACGGGCAAAAATCATAGTCGTAGAAGGGAGCAAAACACT
CGGTTGTTGTTTAACAATTCTGATTAGGAATCGCGCGAACGACGAGAGCAGACGGAGGGGATAAGTGGACCAGAACGAGA
AGGGGCACTTGTTTCCTTACACTCACCGGCTGTACGACCATATGATTGTTGGGTAACAATTCCTTTTTTTTAAAAAAAAATTC
ATTTGCATAGAAAACTGCACGATAATTTGATAGACCATTCCCTTGGTTAAGAGGGATAAAATTCTTTTCATCTGACTTGACGTC
TTTCAAAATGTTCCTAGTAGGGGTTTATCAATATTCAGGGTAATAGTTTAATTTTGCAGAAGTCACTTTCTGATTGGTTCAACA
AAACTAGTTGCGAGTGCCAAATACAGAAATGACGCATAGATGAATAACAGAATTTTGCAGAAAAATACCAGCCACGGCACC
GAACTACGACAGCCAAGTCACAAAAATATAGATAGAAAAGTACAACATCAATTGCACCACCAGCTTCCCAACAAAACCGTC
TACAACATACAAAAACATAGCCGCCAAAAGACAAATCCATGCAATATTTTCTGTTCAGAGCCGTTGTCCCAGTCAGAAACAC
CGGTTGGGTTCATTGACTCACTTCTACACCTAGCAACAGGTCGTCGTCCGAAGCCCACCGCAAGCCACTAGTTGCAGCAAA
ATGCAGAATTGAATTTGCAAAGGCATACATCAGCCACAGCTACTCATGGACGATGCCAACAGCTCCTGTAGAAAGTAAACGC
CTCGGTCCAAGATGAAGCAAGAGCACTCAGCGACAATTCTTCCATAAATCTGCAATCTTCGTGTGACGTGTGTCTACTTGTC
CTGCTGCAGCCACGTCGATCATGACATGGATACCTGCTTTGAACCAGTCACCCCAAGAACACGGTAGGATCTCATTAAGGGC
CGGTTTAGATCTAAATTTTTTTGCAAAATGGCACTGTAGCAAGTTTCGTTGTTATTTGGCAATTAGTGTCCAATCATGGTCTAA
TTAGGCTTAAAAGATTCGTCTCGTCCATTTCGTCTAAACTGTGTAATTAGTTTTATTTTTTATCTATATTTAATGCTTCATGCATG
TGTCCAAAGATTCGATGTGACGGAAAATCTGAAAAATTTTGCAAAATTTTTAAGGAACTAAACAGGGCCTAAGAGAACAGA
AGGATAAGCAGCCCAAAAAGCATGAGCACCCTGCTTTGAATGCATGCTTTACATGATGCTCATGGATTCATACCTTGCAGTTG
CAGATTGAGGGAATCTCGTTGTTGCCCTTCCTGAACTAATCATGGATAACTGTTCCAATTGATGACTGTTCCAATTACTGTATT
CCTGTTTCACGCATGGAAGCCAGGCAACTCAGACACGCCATCATGGTGTGAACTCAGAAAACGCTGTACCCACTAGTCCTT
GACCAGTATTTTCTTTTGGTGACTTGACAACGACCTGGCTGAGAGAATCAAACAAAACGTGAACAGCACCTGCTCTGTTTGT
TCCTCAACCCTCATGCCCATTCTCCATCAGACGCAAAAATGGACCTATGGCTTCTCAGCGCACAGCTTATGCCTCTTTCTCCT
ATGTTCATATCTTGGGGGCGAGAAAG
>SsLRR-RLK223
TTATATTTGACTAAATTTTGTCTTAAACTATAGTATGTTTTTATAATAACCAAGAGGGAGGAGAAGAGCTATTCAGGCTAGTAA
ACATCACAAACCATTGATGAATCTATTCTAGCAGAATATAGAACCGAACGTCCACCAGTATTTGCGTGTAAACATACAAATGA
ATTGGCCATGGCAACGAGCTTTACAATACAAAGAAAAAACGCAAAAAAGGGTGATTTATGTGTCAGAGACACAAGCTGGCC
CTGCAATCACCTGCTCCATTCCAAATTATAAGGCATTTTAAGAATCTTGGAGAGTCAAAGCATCTTAAGTTTGACCAAAATTA
TAGAAAAAATTACAAAAATTTATAACATCAAAATAGGTATACTATGAAAATATAATTAATGAAGAATCTGATAATATTTAATTGG
TGTCATAAATATTATTATTTTATCATATAAATTTAGTCAAACTTAAGATACTTTGACTCTCTAAGATTTTTAAAATATCTTATAATT
TGAGACGGAGGGAATAGTAAACTAGTAAATCCGACATTTCTAGTCCGAGATAACTTAGTAAATCCGTTATAGTACATTTCTAG
TCCGAGATAACTTCATCGTTCTCGTGCACCAAGTCGCTTTCCATAGTCCGCAGCAAACCGCAGGTGTCGTATACAATGTAAA
ATTTCCAAACGTTTACGTCAAACCATAGCTAACCTTGGTGACGACTGACGACAACGGCTACAAAGTAAACGCCTCAGGTCA
AGAAGCAAGAGAGCTCAGCGACATTTCCTCCATCGATCTGCAACCTTCTTGCAGCTCCACCTGCCCAGTGTCGACCACGAC
ATCAATGACTGCTTTGAATCATAATGCATGTCCGTTCTTATATAAATCTTGATGGATGATTTCATCATCCTCACCATCATGCTCCT
TCTTGGCAAAATCATGCATGTTCCTGGTCGGAAGGGGGTGCGCCCTTGTCTCAAATTCTGTATGCAACAACAAAAACCAAAC
AGGTTTTGTATGGTGAAACAGGTAAGACCATAAGAGAAATGATGTCAAATGAAACAGAATAATACGTGATGTTAATTGTTAA
CAGTTTCAAGAGGAATAAGTAATGATGATTAGTACACGAATTATCATACTGTTCTGGGAAGGAGTTCCTCAGACGTAACGTG
GCAATAGTCACCCAAGAGCGATGAACCCACATGTCAGCAACAATGACAGGTGCAGTTATGTTTGATGATCTATTCCTGCAAG
AAAGTCAAGCATATCAATCTCAGATGAAGCGTCATGTTGTGAACTGAGAAAACACTGTCAATGTAGCAGATAACCCTCGACC
CAGATTTTCTTTTGGTGACTGCCTTGTCCTGAAAGACAACAGCTTGTCCTCATGCTCTATCTTGATCAGACTCCTTTGGCCCT
ATAGCCTCTCAGGACACAATTTATGCCTCTGCCTGCTTCCATGCTGATTTCCGGAGAAAACACATGCAGGAACTCCACATCTC
ACCATGAAGTGCTGCAACACTTTCCCCGCATCTTTCCTTGGCTTTGCTCTTGTTCTGCTCCTCTTGTTGGCCTCTCACACAAG
TTCTTGCACTGAGCAAGAGAGCAACTCACTCCTACAGTTCCTTGGTGGGCTTTCACTGGACAACAACCTCACTCTGTCATGG
AAGAACGGCAGACTGCTGCACATGGGAAGGGATCACCTGCAGTCCAGAGAGGAAGGTCACTGATGTCTTCTTGGCTTCTAG
GAGCCTTCAGGGATCCATCTCACCTTTCCTTGGTAATCTCAGTAGCTTGTTGCGCCTCAACTTATCATACAATTGGCTGTCTGG
TAGCCTACCACTGGAATTGGTGTTCTCCAGGAGCATCATCATCCTTGATGTCAGCTTCAATCAACTCAGCGGAAACCTCCAG
GAACTGCCATCTTCAACCCCTGTCCGGCCTATCAAGGTACTGAACATCTCAAGCAACTTATTTACAGGGCTGTTTCCATCCAC
CACTTGGGAGTCG
>SsLRR-RLK224
CATCATCCAATGAGTATGAAATTTTTACTACAGTTGAAGCATATAATAATTAGCCCACTACAAAAATTTTACGATAATTGGACC
ATAGAAACTGTAGCTATAAATTAATTATAGAAAAGCTTAGATCTAAAGCTACAGCAAAAAAAATGTACTAAAGCATACCATAT
TATATGTTCACTGTATAGATCTACTCATGTGGAGTCTGACAAAATTAGATTTTCTATTTTATGATTTTTCTGTGATTTACTATGAT
TTTTCAAAGATTCAGCCGAAATAAAAAAAGGACAAAACCGCCTTTGAAAACCGCTTATAATTGGTCAAGGAGGTAAAACGA
ACGATTTTAAAAATTCAGGGAGGTGGTTTTACCCATTTTTAGAGTTCAGGGAGGAAAAGCAGACTTTTGCAAATGTTGAGGG
AGGTAATGTGGACTTTCGAGCTGTTCGGTTGATGGTTTCTGCAGTTGATAAGACGGCTGAAGCTGATTTGTTGTGAGAGAAA
AATACTGGGCCGATAAGTTTAAGCCAACAGGGCGTTTTTCCTTTTTTAAATTAAGGAAGTCCAACCTCCTCGGTGAGGAAAG
GACAATATTTTGAATTTTTTAGCAATCTCCATCCTTTATTTAGGGGAAAAATATATCTCAAAATAAAACTGATATCAGCATGAA

ATTTAGTATACTTGTTTAGCCACCTCTAATTATGGATTTTGTACAGTATAAATATACACTCTTTTACTCTGAAGTCTAAATTGGTC
ATGTCTGTTTTACCAGTAAAGAATTGCATTCACATGTAATTACAATTTTATCTATGATAGTTCACAGAACAAACAAAAAGTGTG
ATTATTGTCAACAATATTTAGGTTCAAATGGAACCACGAAGGGAGCCAAATCCTTTTCGACCTGGCACTGCACGCTTTAGTAG
AAAGAGCTGTTGACATCACAAACCATTTGTGCAGACTGCCCAAACCATTCTAGCAAAAGCAGCCCCAGTGAAGATTTGGAG
TGAAAATGAAGTTTATTAGGCCAACATAATTGCACTAAACTCACAAATCATTGCCCACAAGAAAGGTGCTTTATTACAAAAA
AGATAGGGAAAAAATTGTATGCGGTATTTTTTTCTGAGATATTTACTGAGTCTGAAACACAAGCGGGCCGGCCTGAAATCAC
CAGCTGAGCCTGACACACATTTGCACCAGGTCGTTGTCCACTGTCCACGAAACCCACCGGTGCCGGCAAGATAGGAAAAAA
AATGTCCCGAGATATTTTACTGAGTCCGAAACACAAGCCGGCCTGAAATCACCAGCTAAGCTTGACACATTTGCACCAGGTC
GTTGTCCACTGTCCACCAGAAAACCACCGGTGCCGTCACAATGGAAAATTGCAATTGTCAAACGCGTAGGACAGCCACAGC
TAGCTAGGCTTGATTATGACGACAGCCAGAAAGTAAACGATTCCTTGGGTGAGCAATTAATCTGAAAAAATCTTCTTGCCCTT
CTGCAGCCATGTTCATACCCCCATCAATGGATGCTTTGAATTATAGTGTGCAATTTGTCCTTCCAAGGGGTGTGTCCTTTCATT
CAAGATTTTGTCTGAGGTTTGTTATAATGAAACTGATAGGAGAAGTGATCCCAAACAAAGTAAATCAGGAGATGTTGTAGCA
TTTTTAAAAGTAATCAATCATATATGGTTGTGCCTATAGCAGCCGGTGCTTGACCAGATTTTTTCTTGTTGAATACGACCAGGA
GTTAAGGTCAAATACACGGATAAATCAATAATGATGACATCAGCTGCTCCTGTTTGTACTTCGTGCTCTTTCTTCATCCAATCT
CATTTAAATGTATAAACTCTCTGGACACAGCATACGCCTATGCTTATGCTTCCGTGCTGATTTCTTGAGAAAATGTGGACTCTC
CATTTCTCATTCATGAAATACAGCAACAGATTTCCCACGAGTTTCCTTGGCCTTGTTCTCATGCTGCTCCTCTTCTTTGCATCT
CGACAAGT
>SsLRR-RLK225
AGGATCCAAAGTTGAGTTTACTTCACCCAAGGGTCTAAACGTGATTTTTTTCAAAAATAAGGCCAAATAGTAAAAAAATCAC
GAAAAGGCCATTTCCGCATACGTATGGGCCAGCAGCGGGCCCATGAAAGGCCCATATGCGTTAGGTGCTGGGTTTTTTAAGT
TAAAAGTCCACATTACCGCCCAACTTTTGCGAAAGTCTGCTTTTTCTCCCTAAACTCTAAAACCGGGTAAAACCACCTTCGT
GAACTTTTAAAACCATTCGCTTTACCTCCTTGACCGATTAAATAAGCGGTTTTTCAAAGGCGGTTTTGTCTTTTTCTTTTTATT
TATTTCGACTGAATCTTTGAAAAATCATAGTAAATCATAGAAAAATCATAAAATGGATAATCCAATTTTTTTTACTCTACATGAG
TAGATCTACACAGTGAACATATAATATGGTATGCTTTAATACAATTTTTTTGCTATAGTTTTAGATCTATGCTTTTCTATAATTAAT
TCATACATCATCCAATGAGTATGAAATTTTTACTACAGTTGAAGCATATAATAATTAGCCCACTACAAAAATTTTACGATAATTG
GACCATAGAAACTGTAGCTATAAATTAATTATAGAAAAACATAGATCTAAAGCTACAGCAAAAAAATGTACTAAAGCATACCA
TATTATATGTTCACTGTATAGATCTACTCATGTGGAGTCTGACAAAATTAGATTTTCTATTTTATGATTTTTCTGTGATTTACTAT
GATTTTTCAAAGATTCAGCCGAAATAAAAAAAGGACAAAACCGCCTTTGAAAACCGCTTATAATTGGTCAGGAGGTAAAAC
GAACGATTTTAAAAATTCAGGGAGGTGGTTTTACCCATTTTTAGAGTTCAGGGAGGAAAAGCAGACTTTTGCAAAGTTGAG
GGAGGTAATGTGGACTTTCGAGCTGTTCGGTTGATGGTTTCTGCAGTTGATAAGACGGCTGAAGCTGATTTGTTGTGAGAGA
AAAATACTGGGCCGATAAGTTCAAGCCAACAGGGCGTTTTTCCTTTTTTAAATTAAGGAAGTCCGACCTCCTCGGTGAGGAA
AGGACAATATTTTGAATTTTTTAGCAATCTCCATCCTTTATTTTAGGGGAAAAATATATCTCAAAATAAAACTGATATCAGCATG
AAATTTAGTATACTTGTTTAGCCACCTCTAATTTGGATTTTGTACAGTATAAATATACACTCTTTTACTCTGAAGTCTAAATTGG
TCATGTCTGTTTTACCAGTAAAGAATTGCATTCACATGTAATTACAATTTTATCTATGATAGTTCACAGAACAAACAAAAAGTG
TGATTATTGTCAACAATATTTAGGTTCAAATGGAACCACGAAGGGAGCCAAATCCTTTTCGACCTGGTACTGCACGCTTTAGT
AGAAAGAGCTGTTGACATCACAAACCATTTGTGCAGACTGCCCAAACCATTCTAGCAAAAGCAGCCCCATGAAGATTTGGA
GTGAAAATGAAGTTTATTAGGCCAACATAATTGCACTAAACTCACAAATCATTGCCCACAAGAAAGGTGCTTTATTACAAAA
AAGATAGGGAAAAAATTGTATGCGGTATTTTTTTCTGAGATATTTACTGAGTCTGAAACACAAGCGGGCCGGCCTGAAATCA
CCAGCTGAGCCTGACACACATTTGCACCAGGTCGTTGTCCACTGTCCACGAAACCCACCGGTGCCGGCAAGATAGGAAAAA
AAATGTCCCGAGATATTTTACTGAGTCCGAAACACAAGCCGGCCTGAAATCACCAGCTAAGCTTGACACATTTGCACCAGGT
CGTTGTCCACTGTCCACCAGAAAACCACCGGTGCCGTCACAATGGAAAATTGCAATTGTCAAACGCGTAGGACAGCCACAG
CTAGCTAGGCTTGATTATGACGACAGCCAGAAAGTAAACGATTCCTTGGGTGAGCAATTAATCTGAAAAAATCTTCTTGCCC
TTCTGCAGCC
>SsLRR-RLK260
TGAACATATAATATGGTATACTTTAATATATTTTTTTGCTGTAACTTTAGATCTATGTTTTTCTATAATTAATTCATACATTATCCAA
TGAGTATGAAATTTAAACTACAGTTGAAGCATACAATAATTAGCCCGCCACAAAAATTTCACAACAATTGGACCATAGAAAC
TGCAGCTATAAATTAATTATAGAAAAACATAGATCTAAAGCTACAGCAAAAAATTTGTACTAAAGTATACCATATTATATGTTCA
CTATGTAGATCTACTCATGTGGAGTCCAACAAAATTGGATTTTCTATTTTATGATTTTTCTGTGATTTACTATGATTTTTCAAAG
ATTTAGCCGAAATAAAAAAAGACAAAACCGCCTTTGAAAACCGCTTATAATCGGTCAGGGAGGTAAAACGAACGGTTTTAA
AAATTCAGGGAGGTGATTTTACCCATTTTTAGAGTTCAGGGAGGAAAAACATACTTTCGTAAAAGTTGAGGGAGGTAATGTG
GATTTTCGAGCTGTTGGGTTGATGGTTTCTGCAGTTGATAAGGCGGCTGAAGCTGATTCGTTGTGAGAGAAAAATACTGGGC
TAATAAGTTCAAGCCAACAGGATGTTTTTCCTTTTTTAAATTAAGGAAGTCCGACCTCCTCGGTGAGGAAAGGACAAGATTT
TGAATTTTTTAGCAATCTCCATCCTTTATTTTAGGGGAAAAATATATCTCAAAATAAAACTGATATCAGCATCAGTTTTGTACTG
AAATTTAGTATACTTGTTTAGCCACCTCTAATTTTGGATTTTGTACAGTATAAATATACACTCTTTTACTCTGAAGTCTAAATTG
GTCATGTCTGTTTTACCAGTAAAGAATTGCATTCACATGTAATTACAATTTTATCTATGATAGTTCACAGAACAAACAAAAAGT
GTGATTACTGTCAACAATATTTAGGTTCAAATGGAGCCACGAAGGGAGCCAAATCCTTTTCGACCTGGTACTGCACGCTTTA
GTAGAAAGAGCTGTTGACATCACAAACCATTTGTGCAGACTGCCCAAACCATTCTAGCAAAAGCAGCCCCAATGAAGATTT
GGAGTGAAAATGAAGTTTATTAGGCCAACATAATTGCACTAAACTCACAAATCATTGCCCACAAGACAAGGTGTTTTATTAC
AAAAAAGATAGGGAAAAAATTGTATGCGGTATTTTTTTCTGAGATATTTACTGAGTCTGAAACACAAACGGGCCGGCCTGAA
ATCACCAGCTGAGCCTGACACACATTTGCACCAGGTCGTTGTCCACTGTCCACGAAACCCACCGGTGCCGGCAAGATAGGA
AAAAAATGTATGCCGTATTTTTTTTCCCGAGATATTTTACTGAGTCCGAAACACAAGCCGGCCTGAAATCACCAGCTAAGCTT
GACACATTTGCACCAGGTCGTTGTCCACTGTCCACCAGAAAACCACCGGTGCCGGCACAATGGAAAATTGCAATTGTCAAA
CGCGTAGGAAAGCCACAGCTAGCTAGGCTTGATTACGACGACAGCCAGAAAGTAAACGATTCCTTGGGTGAGCAATTAATC
TGAAAAATCTTCTTGCCCTTCTGCAGCCATGTTCATACCCCCATCAATGGATGCTTTGAATTATAGTGTGCAATTTGTCCTTCC
AAGGGGCGTGTCCTTTCATTCAAGATTTTGTCTGAGGTTTGTTATAATGAAACTGATAAGAGAAGTGATCCCAAACAAAGTA
AATCAGGAGATGTTGTAGCATTTTTAAAAGTAATCAATCATATATGGATGTGCCTATAGCAGCCGGTGCTTGACCAGATTTTTT
CTTGTTGAATACGACCAGGAGTTAAGGTCAAATACACGGATAAATCAATAATGATGACATCAGCTGCTCCTGTTTGTACTTCG
TGCTCTTTCTTCATCCAATCTCATTTAAATGTATAAACTCTCTGGACACAGCATACGCCTATGCTTATGCTTCCGTGCTGATTTC
TTGAGAAA
>SsLRR-RLK237
TGTGAAATAATTTTACTCTTCCCCTATATTTTAGGTCATTTTTTGTTGTTGTTGTTCAGTCAAAATGTAATCTTGTCATTGTTTGA
TTATGGAAAAATATTTTTATGAATTATTTCTTGTGAGTTGTCCTTTTAATTGTAGTAGTTATTGGTTAGAATTCATGAAATTTGTC
TTTTTTCATCATAATAAAACTTACATCTCGACATAGGGAGTATTTAGGTTCAAAAAATAGAGAGGCATGCCCTACTCCAGCCA
GTTATATTTGACTAAATTTTGTCTTAAACTATAGTATGTTTTATAATAACCAAGAGGGAGGAGAAGAGCTATTCAGGCTAGTAA
ACATCACAAACCATTGATGAATCTATTCTAGCAGAATATAGAACCGAACGTCACCAGTATTTGCGTGTAAACATACAAATGAA
TTGGCCATGGCAACGAGTTTTACAATACAAAGAAAAAACGCAAAAAAAAAAGAGAGGGTGATTTATGAGTCAGAGACATA
AGCTGGCCCTGCAATCACCTAGTAAACTAGTAAATCCGTTATAGTACATTTCTAGTCCGAGATAACTTCATCGTGCTCGTGCA
CCAAGTCGCTTTCCATAGTCCACAGCAAACTGCAGGTGTCGTATACAATGTAAAATTTCCAAACGTTTACGTCATTTTCTTCT
CGAATATGCAGGAGGACTACATATCATTGCATTAAGAGAAGGAGAGTTAGGCCAAAATAGCCATACACAGCGTACAAATACA
CACCCACAAACGCCTACACCATTCGCCCGTGAACACCGGGCTAAACTTTTACATTTTTACATTTGCGCCAAAGATATGTAAGT

TACGACCAGCTAGACCAACTCCCAACTATAGGATGACGGGGGGCTAGAAGCGACCAAGGCGCCAAGATCTTTTGCACCGGC
CAGTATCCAGAATTCAGATTCCTCCCGCGCGATAAGGAATGCTCTGTCCACTCGAGGGGAGATCCCATTGAATACCGTATCAT
TCCTTGCTTTTCCAAAGGGTCCATGCTCCTAAACGACCAAGGTGTTGAAACCATCACGCACACCCTTGCTTAATTGCTCATTT
GCTTGTTGCCACCATAAGAAGAAATTATTACTGTCCATTTGGGGAGTGACATCCGGAATCCCAAAGAAACTCCATAGCTGAT
GCCAAAACTGCCGACAACGTTTACGTCAAACCACAGCTATCCTTGGGTGACGACTGACGACAACGGCTACAAAGTAAACGC
CTCGGGTCAAGAAGCAAGAGAGCTCAGCGACATTTCCTCCATCGATCTGCAACCTTCTTGCAGCTCCACTTGCCCAGTGTCG
ACCGCGACATCAATGACTGCTTTGAATCATAATGCATGTCCGTTCTTATATAAATCTTGATGGATGATTTCATCATCCTCACCAT
CATGCTCCTTCTTGGCAAAATCATGCATGTTCCTGGTCGGAAGGGGGTGCGCCCTTGTCTCAAATTCTGTATGCAACAAAAA
AAACCCAACAGGTTTTGTATGGTGAAACAGGTAAGACCATAAGAGAAATGATGTCAAATGAAACAGAATAATACGTGATGTT
AATTGTTAACAGTTTCAAGAGGAATAAGTAATGATGATTAGTACACGAATTATCATACTGTTCTGGGAAGGAGTTCCTCAGAC
GTAACGTGGCAATAGTCACCCAAGAGCGATGAACCCACATGTCAGCAATAATGACAGGTGCAGTTATGTTTGATGATCTATTC
CTGCAAGAAAGTCAAGCTTATCAATCTCAGATGAAGCATCATGTTGTGAACTGAGAAAACACTGTCAATGTAGCAGATAACC
CTCGACCCAGATTTTCTTTTGGTGACTGCCTTGTCCTGAAAGACAACAGCTTGTCCTCATGCTCTATCTTGATCAGACTCCTT
TGGCCCTATAGCCTCTCAGGACACAATTTATGCCTCTGCCTGCTTCCATGCTGATTTCCGGAGAAAACACATGCAGGAACTCC
ACATCTCACCAT
>SsLRR-RLK213
GGAGGCTTGTGGTAGACAGTTGTTCTTTCTAAAGGCTCTCTTGAACACTTTTGCTACTTCCACAGGGCTGAGAGTTAACTTC
TCCAAATCAATGATGGTCCCAGTTAATGTGTCTGTAAGAAAGCTACAACATCTGGCTGCCACCTTTGGTTGTTCAACTGGTAA
CCTTCCTTTTACCTACTTGGGATTGCCTCTGGGACTCACCAAGCCTAAGGTGGATGACTTCTTGCCTCTAGTTTCCAGATGTG
AGAGAAGACTGGTTAGTACTTCACTCTTCCTATCTCAAGCAGGGAGATTACAGATCACCAATTCAGTCTTCTCATCTCTTCCA
ACTTTCTATATGTGCACCTTTCTTCTCCATGTCACTGTTAGAGAACAAATTGATAAGTTTAGAAAACATTATTTATGGAGAGGG
TCTGATGAAAATAATAGAGTAAATGCCAAGGCTGCTTGGACACTGGTTACAAAAAGCAAAGAGGAGGGTGGATTAGGAGTG
ATAGATCTCAAAACTCAAAATGAGGCTCTTCTACTAAAAAAATTTGCACAAGTTCTTCAACAGAGCAGACATCCCTTGGGTGC
ATCTTGTTTGGGAGAAGCATTACAAAAATGGTAAGTTACCTAGTCTTGCTAAGAAAGGATCCTTTTGGTGGAAAGACTCCCT
GAAGCTTCTGGATAATTTCAAAGGCATGGCCTCAGTTTCAGTTATGGATGGTGCTTCCTGTATGCTTTGGGAAGATGGCTGGC
AAGGCCAGCCCTTTAAACTGGTTCTTCCAGAGTTATACTCTTTCACCAAGAAGCCCAAGATCTCCATCAAAGATGCTATTGCA
GCCAGTTCTTTTACCAGCCTGTTCAATCTTCCCCTTTCTGTTGAGGCTTATGGCCAATTTCAGCTAATTCAAAATCTTCTTCAG
AGCTTACAGTTATCTGGTAATCCTGACACCTGGAAATACATTTGGGGAAACTCCACTTTTACCTCCAAGAGAGCCTATTGTCA
TCTCAGGGGTTCCAGCCAGTCCCATCCAATTTTCAAATGGCTCTGGAAGTCTTCTTGCCAACATAAGCACAAGGTATTCTTCT
GGCTGCTCACCCAAGATAGGTTGAGTACAAGGAATATTCTGAAAAGGAAAAACATGCAATTACCTTCCTATAATTGTGTGTTA
TGTAATCAAAACATTGAGGAGACCGTGGAGCATCTGTTCTTGGATTGCGAATTGGCTAAAGCTTGTTGGGGTAAAATTGGCC
TAACAGTGGATAGTTCCCTTAACCCCCTTCAGATTTTTCAGAGCTTCAGACTGCAGCTGGGCATTGCTTTTTTCATGGAAGCA
ATCATTGTTATGAGCTGGAGCATTTGGACTCTTAGAAATGATGTCATCTTCAGAGGGATTCCTGCAAGTACTCTGCGTGGCGC
TGAAATTTTCAAGGACATCTTTGGTCTGCTCCTATGGAGGGCCAAGAAGAAATATTTCCCTGCAATCAGTTCATGGCTAGAGC
AAGTTGTTTAATAGCTTTGATTTTCTTTGTTTCTTTCTTTGTTTCTTGAACCTCTCCCTTTCCTGGTTTCCTTTCATGTTTTGAA
CTCCTGTGCAACTGTCTTGGTTGCTGTTTTTTAATAATATTTTCAGTAGAGGCTCCTGAGCCTCTCCTGTTCCCTAAAAAAACT
GCCTTGTCCTGAAAGACAACAGCTTGTCCTCATGCTCTATCTTGATCAGACTCCTTTGGCCCTATAGCCTCTCAGGACACAAT
TTATGCCTCTGCCTGCTTCCATGCTGATTTCCGGAGAAAACACATGCAGGAACTCCACATCTCACCATGAAGTGCTGCAACA
CTTTCCCCGCATCTTTCCTTGGCTTTGCTCTTGTTCTGCTCCTCTTGTTGGCCTCTCACACAAGTTCTTGCACTGAGCAAGAG
AGCAACTCACTCCTACAGTTCCTTGGTGGGCTTTCACTGGACAACAACCTCACTCTGTCATGGAAGAACGGCACAGACTGC
TGCACATGGGAA
>SsLRR-RLK232
GGTGGGAGGCATCTCGCGAGACCGCGTCGACGCGATGGGCACGCGATCGCTCGATGGGTGCACGCGAGCGGACGTGAGCG
ATCGGGGCGATCGGATCACGTCACGATCGGATCGGGCCGGGCGACGCGCTCAGGAGGGAAGGCCGCGAGCGGACGTGAGC
GATCGGGGCGATCGGATCACGTCGGATCGGGCTGGGCGACGCAGTCACGAGGGAAGGCCGGCGTCCTGGGAGACAGCGAC
GGCCGGAGGAAAAAAGAAAAAGGTGGTGGCCAGAGAAGGAGAGGAGGCCGGCGTCGAGAGGACAGCGGCGGCCGGAGG
AGGAGCCGGCGACGGATCAGCGGCGGCTAGGTTTTGCGGAAGCGCGGCCGCAGGACGCGAACCGCGTGAGATACCATGAA
AGCGAGTAGTGTAATACGCGATACATTCTCAGAGACAAGCGTTGTGTACATATAGACTCAATGGGGAGCCAGGTACGGCAGG
CCCATGGGCCGTGACTCGTTTACACTCTAACAAGAGTCAATATAAAATGTGCAATTGCATTCATACTGTAACCAAGTAAGCAT
AATCATCAACATAATCTCAACCAATAAAAAATTACTTCCCCTCCGTTTGGGTTTATTTGACATCACCTTTTTACCTAGCGATTTT
GACTGTTCTACAGTAGGTCAGTAAATTATTGGTGACAGGTAAAACCAAACAGAGGGAAGTACAAAAACACGGTGACCACAG
ACGGTGAGACAGGTAAACCCAAACGGAGGGAAAGTAAATTATTGGTGACAAGTGAACCAAACAGAGGCAAGTGCAAATAC
ACGAGATTATTGGTATCACCACCTGAATAAATTTGATAGGCCATGTTCACACTTCACAGCAGCACCTCCATAGGCCACGGCTG
TTACTTCCAAGGCTTTTGAATTCCTGTCAGCAACACGAGCCGTGGCCCGTGGACCAAAGCTCCATCATGCAAGCGCCTCTGG
AACCAGGGCCATGGAAAACAGCCATGCACATCACTGAATCAGCAGTAAGGATAACAGAAAATAAGCAGCCAGTCAGAAGA
AGAATAGGGCACCAGGTCAGTCGCCCTCGCCCTGTCACCCTCACCCACCACCTCAGTAGCTCAATTGCTTTCTTTGCCTCCA
CTTGTGCCTAAGTCCAAACCATGTACACACCTTGACCGTCAACCTGATGATTGCTCTAAATTGTAGGCTCTTTCGGAATCATG
CGCTTTGCATACTCTCTCACGCCAATCGGGTCTTGCAGATCATCAGCAATGTGGCTTTCAGAGATACGAGCCTTTTCCTGCTC
AACAAAAAGGGCACATCGATTAGTGATTCAAATTTATTTATTTTTTGTCTTAGATTTGTTAGCACTAACGAATGAGGTTACCAT
AACTGGTTGTTTAAGTGAAGCTAGTAGGACAAGTGATGTCAGATCCACAGGCAGAGCCAAAAATGCGTTTTTTTATGGACC
AACAATACAAATTTTATCAAAATTTGGTCAGATTCCAAAAATCTCAGTGGAGCCTATACTCGCCCCTGGTCAGATCCTTAGAT
CAGGCCAATTGAGGGGTTATACAAATGTGGCAACATAAATTATGTCAAATCAGGTAAAATGGTAAATCACAGCAGACTGTAG
AAACTCAACAAGGATTGATTCAATGATGACTGGACAGAGGCAAATATTTTGTATGGTCAGGAAGCTTCGTCAGTTGGATTCC
TAGTGAACCACAGCAGATAGCCACCAATGAAACATCTAGGTGTGAACAAGCGAGCTGCTGTGTCTGTTGCATATAATAGAAT
CATAATGAAGACTTTGTTGTACCATCTAATGCTTGCCTCTATTTTTCTTTGCGGTGGACACTACTAGAAGGGATCAGCAAGAT
GGAACCAGCAGCCTCTTCCTTCTTCTACCTTATGTTCTTCCCCGGTTAGACCATTATAACTTAGTCTAGATTGGGCACAAGTTA
AGTGTCTGATTTCCATGCTAGTTTCAATAGGAAGCC
>SsLRR-RLK231
TTTCTATGGCGAACAGTAGTTCCGCGTGCTACAGTGTTTTCTGCGCGTGCTACAGTGATTCCTGGTTGCTACAGTGTTTCTTC
GCGCTACAGTACTTTTGCGTGTTCTCTGGTTCGCTCGCTGGCGTTCTGCTCAGTACGACGTGTGCTCCGGTTTACTTTTTGGT
GCTCAGTACGACGTGTTGGTAAGTTTTACGAGCTGCAGTCGGTTGTCGGGCAGGATGAAAATTTTGCAAGGGACTAATAAA
GACTCGTGTACTCTGAGAGTAGACTTGGCAGAGACGAAATAAATTGGACGTGCTGCACACTCTTCTCTTTCTCCTTCATGCTT
GGTTGAGCAGCTTTGATTACTTGACGTGAAGACCAGCTGAGGAGGCAACATGCAGCAGCTTTGATTGCTCGACGTGGAGAC
CGGGTGAGGAGGTAGCAAGCAGCAGCAAGCAAGCAGTACGTGCATGCCAGGCGTGCAAGTGGCCGGTGACTTGTGTCGGA
GCGTGTCATTCACGAATAATTGATCTTTCCCGCTTTGGTTTGATAAAATTTGTGAATATTCGCACTTGATAATTTAGTTAGCGA
ATTCTTGCTGATCTAGGTTTTATATTATTCGTTTTCTGTTTTTCATTTCCGAGACTCGAATTGCATTTCGATTTTTATTTCCAGCT
CATGCACTTTAACTAAATGCAATGCTCATAAATATTTTTAACCTGTGATTTACAACGTAAACCAAGGGTGTTACAGATACTACT
GACACATGAGGGACAATTGCAAGGACTACTTGACTGCTGTGCTTGATGAGTCAACAACCTTAGAATGCTAATAAGAAATTGT
GGCATTTAGGTGTGTCCGATGATACAATTAGAACATAATTGCACCTCTTAGCCATGCACAAAATTAAGTTTGAAATTTTGATGT
ACCAAACATCACGTTCGAAAATTGTTTTTTTAACACATTCATACTATTGTTTTTCAACAATACACCATACGCTCGAACTTAAAT

TAATTACCATATCATTATTATGTTCCCGTGCAACGCACGGGTATTTATCTAGTCTAGTTAAATTTAAGAAAGTTTAACTATCATC
AATAGAAGACACCTTTTTTTTGGTATAGGTGGGTGGGGGACGGAGGGGGTAAGGTACGCCCCCAAATTCGTGCTTCGTTTC
TTCGCTCCGATGACATGCAGCTCATGTCACTCCTAATGGCTGTTTAAATCGGCCAAAACAAATTGTTAACCAGAGCTAAAAAT
TAGATATCTAACTGTTAGCCCGTACATGTTTGGATCGGTGCTTACATCTCTAGCAATGAACTATTAGCTCTTGTTAGGATAAGTA
CATTGGTTTTGTCTCATAGGTGGTCTCATGTAGTGCTATGTAATTTTAAGACGATTTAATAGACAACATATACAATGGTTGTTTT
TTAACTTGTCTTGTAGTAATTCACAATTCCTTAATTTATGCATAGAATAAAAGTAAAGATGTGAGTTGCATGGCAATAACAACT
CATACAATGGTAGAATAGTTGTTTCATGATCCTAAATTTTAGCTCACGAGGTGGTTGTTTCGTAAAATAACGACCTCGTTCCCT
CTCTCCTCTCTCTTTCCTCTACATCAGTAAAAATCCTACGTAGCACTGCACGAGACGGCCAACGAGACCGCTGATGTAGCAA
TGTACCTGTCCTGCTAGTTGTGCTTGGCTAATAGACTTTTTTAGTGGCGAGTTATGTATGAGTATGTCATCGATCGGTGACGTC
AAACTGAGAATACGTGGGCTGTTATATACAAGTATATGTAAGTAAAACACTGCCATAAGGAGGATTTTCCTATACCATTTAACC
ATCGAATTCATCATTCTTTGCGGTAACTACGACCAGGAACGTACTGGTAGCCAGGATGAAAGCACGCTCTCTTTTTCCTTCTA
CCTCGTGCTCATCCTTATTAGACCATTCCATTTGGCTCTTCATCTAAACAGAGCACAGGCTAAGCTCGTTTAAACAGGAAGCC
>SsLRR-RLK255
TTACTTTTGATCCTGATCAGCTGTATCCTAGTTGCTAACTTCAAATATCTAATAACTTCTAGGAATAATGCATGTTACTGTGGCT
GAGCAATTAAATAGCTAAAAACTGATGCACTGACATTCAACATCTATCATTTGTTGAATTAGTGTGCTTGTAAAGAATTAGCA
CACACTTGTGGCTTAATATTCTAAATTTTTTTGTAAAGAGGGAACACTGAAAGAAAAGCTGTTTGATAATATCCGGGTTCCTC
AGTTGATTTTTTTCAATCTTTCTGCAGCTGCCATTGAGAAAGAGATAGCTAGCCACTAGAACCCCTGCTGCAGTTGTTAGGAC
AGTCCCTGGGCAAGAAGGTTATGCTGAAGCTGCAGTGCCGAAGTTGCAAGCACTACCTGTAGCACCTGATCTAGGTACTGC
TTATTTTTCTTGGGCTGAACATGTTAAACCAATCATTCCTTTATCTCTCTAATCTCTCTATCTGTGATTGTCATTTTTTCTGCTTC
GTCTGCCACAGTCTAGGGATAGAGTTAGCGCTTGGAATGTCCATTTCCATGCCAATTCGGCTTCAATGCTGCACGCCTGATGA
GGGTACTAGGATGCTTATAGAATTTGTCTATTGCCATGACGCCCGAAATTTACTGTGAACTCATCACTGACAAACTGCCATTTC
GTTTTTAACATATGGCATGAAATGCCATGTGTCCTAGTTGCACTGGTGCTCACGTATACAGCTTGACATCATACTGTCACCTTG
CTTAACAGTAACCCAAACTAAGGAGAATGAATTCTTCCACATGTACATGCAGGTACACACTATTGGGAATTACAAATTTCTAT
GCAAACTCAGGAACTGGCCCGCAAGTCGACCCTTGTTATGTTTTTATATTGTCGATTACTAAATTAGCTAGAAGAGATTAGTA
CACAGTGTCAAAACTTAATTGTGTTTCTTTGTCAGCTCTGGGTGTAATTTTGGCAATGCTATTGACAGCATCAGCAGTATTTAT
TGTTTTTGGCCGTTCATAAAAAAATAAAAAAAATCCCCAAACAGGAGCGTGCCTCGCACCTCCTCACTCCCCTTTCCCCTTG
GCGGATACTTACAATTGTTTACAGGATAGACCTAGTTTTAGCCTGGCGGACTAATTCTCGACATCGAGGATTTCGCCCCTGAC
CTTCAACCACAACCTGCAGCCATGGAAAAAATCTTACGCTCGCTTCCTCCTTGGTTTCGCCATGGCACTGATGACTCCGTGC
TGCCACCAGAATCATGAATCGGGCGAACCATCGATTCCAGAAAGGCCGCCTTTACCCCTCTTGATGTTGAGCTGTGCGTTGA
GACGACGGATCAGTTCTATTTATGCTGAGTTTTCCTATGCTAGGATTACAAAAAGATCTGGTTGGCAGCTCTTTAATGCAGTTT
CTGCTATCAATTATCCATAGATTTGTTGAGTGTTTTGGTTCTGTTGGATTTGAGATGCTGGTGTGATTTGGCCATCTGGGTACC
GGTAATACAAGGAGTATGAAATTTTGGTTCACCAACTTGAGCCTTTTTTTGTGTTGTGCAATCCAAACAACTAATAGAATGGT
TGTCCCAGTTGTTTTTATGGGTTCAAAGTTGCATCTCTTATCAAATGAAGCAGGATTTAAATATCTGCTTCTTTTTTTTTTTTGA
AGAGTTGGAGTTAACTCTAATACTCTTATTAAGTTGAAGTAAGATTGAACNNNNNNNNNNNNNNNNNNNNNNNNNNNN
NNNNNNNNNNNNNNNNNNNNNNNNNNNNNNNNNNNNNNNNNNNNNNNNNNNNNNNNNNNNNNNNNNNNNNNNTTCAGTG
GAGAGCTTGCCAAGGTCAATTTCTTCAACTTAGAAAAATTAAAAATATTAGACCTTAGGAAGAACAACTTCAGTGGCAAAAT
TCCAGAAAGCATCTATTCATGTATCAAACTAACTGCGCTGCGTCTATCTTTTAATAATTTCCAAGGCCAGTTATCAAAGGGACT
AGGCAGTCTG
>SsLRR-RLK254
CTCTGGCAGCTCCCTAATACTATCCCGCATTCTCATAAAACTGTCATATTGGAAGAGATGGGACAATTTCTTTTGCTCCCGTAG
CTCCCCAAAAGCTCTCCCTTTCTCATCTCCCCTCAAATAAAGAGGTAGTTGCATCTCCCCCAATAGTGTTGGGACCACAATAA
CCGTCCTCTTTTTCCTCCCATCCTCCATTCTGGCATGTGGATTCCATGTATTGGAGCATGTTATTGGTGATCTGCTGGAGCATCC
TCGTCAATATTCAAAAAAAAAGTAGTATTGATGATCACCAATACTATTTTATTAGGGATGAGCTATTGGTGAGCTAATGGAGAT
ACTGTAAGATGCCATCGATTCATAGATTGTAACTTTCTTTGAATTGAGATTGCAAGCAAAACTCAATTGATGGGCATCATTGC
GAAGCCTACAACTTACGTGTTCCAGCCTCTTATATAGGGAAACACTTTTACCAATTTCGCCATTTCGATGAATAGATGGCTTAC
TCCAATAAGCTGTTATTAGCAATAAACTATAGTAGTAGTTATACCTTTTATTTTTATCGATATGATTAATTATGTTTGGGGATACT
ATGATACTAAAACTTGCGCACAGGTACTTATTGTCTTTTACCGTAGCGTACTATTTCAAAGAAACATATTGTTTTTTTTTATCGT
AGCGTTAACACGGCAATATACTATGAACCTTAATATCTCATTCTATCCTTAAATACCAAGATATGTATTGCCTTTGGCTAGCTTG
ATTTGTATTCTGAGACGTCCCGGCACAGAGACACGTTGGGTCGGTATCGGTACGCCGTACGCACGCGCCTACGTACATGGCA
GTACAGTACAGCCCGTTTTCACGTCACGTCGGACGTCGCCACCATCTCCCCCAGTATTTCAGCCCGTTCTCAGAGTTGGGAC
GACTTTGAATCGGAGAAGGTAAGGACTTTCAGACTGATGATGATGAACGGAGCACGTACTACGTGCGTACGCGATGGGTGG
CGGAACACAAGAACGCGGCGCGGACGGCTGAGTGGCTGACAGACGTGCGAGTGCGACGACGTGTCTACTGGCATGTGTCG
CGTTGCCGCCGCCCGCCGGCCCGCTGGTTTGGCAGTCGTGCGTTGAGGGCGGGGCGCTGACCAGACCGCGTCCATGCATGC
CGCTTGTTGGAGCCTGGACGCGTACGGCACGCTGCGCGCTCCGTCCGATGGCGACCCCGACCCGCTCACTGCCTTGACTTG
ACTGCTGCCACTGCGTGGTCCGTGGAGTCCAGACTCCAGAGTGCACAAACAAATGGCCATAGGGCCGCAACGCCTGCTCAG
CAGATTCTTCGGCTCAGCCAGCCACACGCTGTTCTGTTCTGCAATAATCATCACAAGAAAACAGTAAGCCAATCGTGATAAC
AGATAATAATCATACAACAGCCACTAAGAGCCAGCATGAAATGTACAAATGGGCTCGCTAGTACTGTAACCAATAAACTACTT
CCCTCCGTTTGTGTTTACTCCACAATCACCTTATTACCATAGCAATTTTGACTGAGTTTTTTTACTACATAATCTTTAGGTACTT
CAGCTTTTGATATTACACAATTTGTCACGAAATACACTTCGTGAGTATTATATATTTTGAACTATGCGAAATTCTTTTGTAGAAA
AAATTTGGATGGTAAGAGCTGCTACAGTAAATTATTGGTGACAGGTAAAACCCAAATATAGGCAAGTACAAAAACACGATGA
CCACATGCAGGTAAACCCAAACAGACGGAAAAGTAAATTATTGGTGACAGGTAAACCCAAAAAAACGGAAAGTAAATTATT
GGTGACAGGTAAACCCAAACAGAGCACAAGATTATTGGCATCACCACCTGAATGAATAAATTCGATAGTCCACGTTCACACT
TCACAGAAGCACATCCACCATGGCTGTTACTTCCCGCGCTTTTGAATTCCGGTCATCAACATGAGCCGTGGTCCGAGGACCA
AAGCTCCATCA
>SsLRR-RLK253
TTGCGCAAGTCTAGCTAAGAGACCTTACAGGGAACCAAACAGGCCCAGTTTGCATTATGTGGCTAGGGGCTAAGCCAGGAA
ACCAAACACACCTAAATGATGAAACATAATCTAGAAGGAAAAAAAAAAGTCTAAAAATAGGCAAGTATGATGGCTCGATCAA
CTGGCTAGCTGAATACACAGCATGAGCAAAAATAGGATAAAGTGGTCACGGTTCGATAAATTTATGAGGAAGTTGTCTAGAT
CGGTACAATAACGTTTACATCAAGCGGTTGATACTGAATACTCTCTCCGTCTCTCAAAAATACAATTCTACATCTCAGCCAAC
TCAGAATTCCTTAAATTTGTTTTAAAAAAGTGATATAAACATTTATTTATTTAATTATAGATATAATTAAACTTAGAGCTTTTTTA
CGGTTCTTGGGGAACTACCGGTTCCTTCCTTAAAAACTATTTGGAGCCGAAAAAGTTGTTTTTCACCATTTTGTTTCTTGTTC
CATGATTTTATGTAGAGAACAAAGATCCCACGTGCCATGTTCCATTGAAGAAAAGCAATAATATTCGTCCACGCGCATGCATG
CCGTTGCCGTGGCTTCTGCTGCATCCTGCCGTGCTTCTACTCCATCTCCATGCCTGGCTGCAGCCTGCAGGAAGCAGAGAGT
CCGAGCCGACACGCCAGCCACTCTGCTATGCTCCCAGCACCAGCAGGTTCCTCTGCGATTGGCTCTGAATCCTGATAGCGGG
ATTATGTAACATCGATAGATACGTCTGCGAAAAATAAACAAATAGTGTGTGAATGGAAGGAGCAGACAAGACCCAAAGTGTT
CTTCTAGGAAAAAAGACACAGAAAGGGAGCCTTTCAAAAAGAGAGGAAAAAAAAAAAGACACGGAAAGGGACGCAGCGC
TAGGCCGCTCTAGCTCTGCTCAGCATTCAGCATTCAGCAGGCTCTTAAAAGCCTCGACAGTGTTTAGTGCCATCCACTTGCTG
TTCTGCAGTCGACACCACGAGAAAATAGTAGCCACCCGTGATGTCAGATAATCATACAACGACCACTAGAAATGTGCAACTG
CACCCACTAACCAATCAATCAGTCATCATCAACATGAACATGATCAAGATGCATTGAAAAAAACACGAGATCTTGAGTATCA
CTACCTGAAAATTTTCGATAGGCCATGTTTCACAGCAGCTTGCTTCAGGCACTCCACTCTCCGGGGCTGCTTCAAATCTGTCT

GCCAGTCCTCGTGCACACGCTGCATGCCCAGTTTACTTCCAGCCAGAGCCGAGGTCCATTACGCACCTGTGGAACAGGGGA
GGGGGAGCATGGGAAACAGCCACGAGCGTGCACATCACCGCATCAGCTGCAAGGATAACATAAAGTAAACGCCTCGGGCC
GAGGCGGCTAATCAGATGAAGAATAGTACACCAGGTCACCACCACCATGTTGATACCTTGACAACGTCGCCGATGATTGATC
TAAATTATGTGCCTTTTCCTGTTCACCAAATAGGGTGTAAGGACATGCGATGCCAGATCCTGAGATAAGGTAAATTCATGCAG
GCTCTGTGTTGACTGTAGAGTGAATCTGTCAAAATAGGTTATGTCAGATCAGGTAAATCATGTCACACTGTAATAATTCAACA
CGGATTGATTCAAGGATGACTGGGAAAAGACAAATAGTTTGTGCGACCAGTAAGCGTGGTCAGTTGGACTTCTTAATTGATC
ATAGAAGGTAGCCACCAATGAAACGTCAAAGTGCCAACTAACAAGCGACCTGCTGTGTCTACTGCATAGAATACAATAGGTA
TGAGGAAATTGTCATAGCTTCTAATCCTTCACTCCATTTTTCTCTCTGCTAACCACACCAAGAAGGGATCAGAAAGATGGAA
GAGAGGGCTTCCTCCTCCTACCTTATGCTCTTCCCTGATTAGACCATTTGGTGCTGCCTAGACAGGACACAGGTTCAGCATCT
GCTTTCCATGCTTGTATCGAAAAGAAGCC
>SsLRR-RLK209
TCACGAGGCGCGTCCTCGGCATTCACGATGACTATCACGCATTGAAAAATCATGCATCACGCAAAAATTCGATGCCAGGAGG
GATCAAACCTTGCCCTCTTGCCTCAAGCATCAACGCGTTAACCAGACCAGCTACGAACATTTGTTGAAGGGGGGAAACCGG
CTATCCTATTTAATTAGGGCAAACAGGGAAAACACACCTTAACTAATCACTCCTTGGTATGCTAAATCCTATTAAAACGACTTT
ATTTTTAGTATGGAGGGAGTACAATACAAGCAACTTCAGCCTGGACCCAGGTTAGGGAACCGTGGGCCTTGATTTGGCTGC
CCTCTGCCTTTCATTTCAAAAGGCCAAATGGCATGCACCTATTGGTTGCATGGTTTTTCAAAAATTTGTCGTTGTTACCCAGAT
TTTAATGCTAATAACATCTCCCAAAAGTTTGTCGTTGTTACCCATTGCAGCTCTAACTAGAAATTATAAACTAGCTATGAGGAA
GGAAGCAACACCCCACAAGGGATGGCAGAAACCTCGGCCGGAAGGCATGAACATGGTGAATGTTGATGCATCTTTTTGATG
ATGATGTGGGGTGCAGAAGTACAGGGTAGTAATAGAAATTGTTCAGGGGGAGTGTTGGCTGCTTCACACAGTTTGTTGCTCA
TGTGGTTGATGTGCCAATGACGGAAGCATATGCATTGAAGGAAGGTTTGCTGCTGGCACAACACATTGGAGTTAATTGGCTC
ATCATCCAAGCTCCAAGCAGACTGCATGGAAGCTGTAGAGACAATGAAGGAAGGCGGCTTTTTGGCGAGCTCGGCGGCACC
CATCTACGACAAACGCAGCACTATGCGGAGCGGATTTCAGGACATCTCATCTCGATTGAGCATTGTAGCATGTATGTAAATCG
AGTGGCACAGAGTTGGCAAGGCAAGCTATGCAAATCAAGATAAATTGTATCTGGGACGATGAGCCCACTAGTTTTATTCTTG
AGTTTGTATTAAATGATGTAACCATCCATAATTAATAAAGCTCGCTGGATGCATTTGTCAAAAAAAAACATCTCCTTAACCATA
TATCCAATTTGCAAGTTGTTTTTCACCATTTTATTTCTCGTACCGTGATCCCAACGACAGAACAAGATCCCACGTTTTATATTTT
TTTCACGAACGTGCTTGATTTCCATGCACGTGTGCCATGTTTTACAGCGAAGAAAAAGATAATAGCATGTGAATCCTACAGGT
CATTTTCACACAGGTCTAGCAAAATTTGGCAGGACAACTGTCCAAACACTCTTCGCAGCTCTACATTTTGGTATTGGCCAAA
ATTTTGTTCCGTCGCAAACCAAGCATTAATTTATAGCCTTAATTTTATCACTAGTCAAATTTGGCCATAACCAAGATTTGACTC
AACCAGGCACCTCCACGTTTGTTCTGGGTGGGTCCCGTCTCCATCCAGGAGTGGGAAGACCGCGTCCTCACGCGCAATCGC
GCATGCCGTTGCCGTGGCTTCTGCATCCTCCTGCCGTACGTGCTTCGGCTTCCACTCGATTATGTAACATAGATAGATACGTCT
GCGAAAAATAAACAACTAGAGTGTGAATGCCAGGAGCAGACAATCACAAGATCCAAAGTGTTCTTCTAAGAAAAAGACAC
AGAAAGGGACGCATTGCTAGCTCTGCTGCGCTCAGCTGGCGGCTCTTAAAGCCTCGACAGTGTTTAGTGCCATCCACTCGCT
GTTCTGCAGTCGACACCACAAGAAAATAGCAGCCACCCGTGACCGTGATGATAGACAATCATACAACGACGGGACGACCAC
TTTGAAATGTGCAACTGCACCCACTAACCAAGCAATCTTCATCAACATGAACATGATCAAAATGCCCAGAAGAGAGGGCTTC
CTCCTCCTCCTACCTTATGCTCTTCCCTAATTAGACCATTTGGTGCTGCCTAGACAGGACACAGGTTCAGCATCTGCTTTCCAT
GCTTGTATCGAAAAGAAGCC
>SsLRR-RLK208
GAGGCGCGTCCTCGGCATTCACGATGACTATCACGCATTGAAAAATCATGCATCACGCAAAAATTCGATGCCAGGAGGGATC
AAACCTTGCCCTCTTGCCTCAAGCATCAACGCGTTAACCAGACTAGCTACGAACATTTGTTGAAGGGGGGAAACCGGCTATC
CTATTTAATTAGGGCAAACAGGGAAAACACACCTTAACTAATCACTCCTTGGTATGCTAAATCCTATTAAAACGACTTTATTTT
TTAGTATGGAGGGAGTACAATACAAGCAACTTCAGCCTGGACCCAGGTTAGGGAACCGTGGGCCTTGATTTGGCTGCCCTCT
GCCTTTTCATTTCAAAAGGCCAAATGGCATGCACCTATTGGTTGCATGGTTTTTCAAAAATTTGTCGTTGTTACCCAGATTTTA
ATGCTAATAACATCTCCCAAAAGTTTGTCGTTGTTACCCATTGCAGCTCTAACTAGAAATTATAAACTAGCTATGAGGAAGGA
AGCAACACCCCACAAGGGATGGCAGAAACCTCGGCCGGAAGGCATGAACATGGTGAATGTTGATGCATCTTTTTGATGATG
ATGTGGGGTGCAGAAGTACAGGGTAGTAATAGAAATTGTTCAGGGGGAGTGTTGGCTGCTTCACACAGTTTGTTGCTCATGT
GGTTGATGTGCCAATGACGGAAGCATATGCATTGAAGGAAGGTTTGCTGCTGGCACAACACATTGGAGTTAATTGGCTCATC
ATCCAAGCTCCAAGCAGACTGCATGGAAGCTGTAGAGACAATGAAGGAAGGCGGCTTTTTGGCGAGCTCGGCGGCACCCAT
CTACGACAAACGCAGCACTATGCGGAGCGGATTTCAGGACATCTCATCTCGATTGAGCATTGTAGCATGTATGTAAATCGAGT
GGCACAGAGTTGGCAAGGCAAGCTATGCAAATCAAGATAAATTGTATCTGGGACGATGAGCCCACTAGTTTTATTCTTGAGT
TTGTATTAAATGATGTAACCATCCATAATTAATAAAGCTCGCTGGATGCATTTGTCAAATAAAAAACATCTCCTTAACCATATAT
CCAATTTGCAAGTTGTTTTTCACCATTTTATTTCTCGTACCGTGATCCCAACGACAGAACAAGATCCCACGTTTTATATTTTTT
TCACGAACGTGCTTGATTTCCATGCACGTGTGCCATGTTTTACAGCGAAGAAAAAGATAATAGCATGTGAATCCTACAGGTC
ATTTTCACACAGGTCTAGCAAAATTTGGCAGGACAACTGTCCAAACACTCTTCGCAGCTCTACATTTTGGTATTGGCCAAAAT
TTTGTTCCGTCGCAAACCAAGCATTAATTTATAGCCTTAATTTTATCACTAGTCAAATTTGGCCATAACCAAGATTTGACTCAA
CCAGGCACCTCCACGTTTGTTCTGGGTGGGTCCCGTCTCCATCCAGGAGTGGGAAGACCGCGTCCTCCACGCGCAATCGCG
CATGCCGTTGCCGTGGCTTCTGCATCCTCCTGCCGTACGTGCTTCGGCTTCCACTCGATTATGTAACATAGATAGATACGTCTG
CGAAAAATAAACAACTAGAGTGTGAATGCCAGGAGCAGACAATCACAAGATCCAAAGTGTTCTTCTAAGAAAAAGACACA
GAAAGGGACGCATTGCTAGCTCTGCTGCGCTCAGCTGGCGGCTCTTAAAGCCTCGACAGTGTTTAGTGCCATCCACTCGCTG
TTCTGCAGTCGACACCACAAGAAAATAGCAGCCACCCGTGACCGTGATGATAGACAATCATACAACGACGGGACGACCACT
TTGAAATGTGCAACTGCACCCACTAACCAAGCAATCTTCATCAACATGAACATGATCAAAATGCCCAGAAGAGAGGGCTTCC
TCCTCCTCCTACCTTATGCTCTTCCCTAATTAGACCATTTGGTGCTGCCTAGACAGGACACAGGTTCAGCATCTGCTTTCCATG
CTTGTATCGAAAAGAAGCCA
>SsLRR-RLK233
ATTCGATGTGACGGAAATCAACTGTTCTTTTGCAAAAATTTTTGCAACCAAACGGGGCCTAGGAAAAAGACACAGAAAGGG
AGCCTTTCAAAAAGAGAGAAAAAAAAAAAGACACGGAAAGGGACGCAGCGCTAGGCCGCTCTAGCTCTGCTCAGCATTCAG
CATTCAGCAGGCTCTTAAAGCCTCGACAGTGTTTAGTGCCATCCACTTGCTGTTCTGCAGTCGACACCACGAGAAAATAGTA
GCCACCCGTGATGTCAGATAATCATACAACGACCACTATGAAATGTACAACTGCACCCACTAACCAAGCAATCAGTCATCGT
CAACATGAACATGATCAAGATGCATTGAAAAAAACACGAGATCTTGAGCATCACTACCTGAAAAATTTCGATAGGCCATGTT
TCACAGCAGCTTGCTTCAGGCACTCCACTCTCCGGGGCTGCTTCAAATCTGTCTGCCAGTCCTCGTGCACACGCTGCATGCC
CAGTTTACTTCCAGCCAGAGCCGAGGTCCATCACGCACCTGTGGAACAGGGGAGGGGGAGCATGGGAAACAGCCGCGAGC
GTGCACATCACTGCATCAGCTGCAAGGATAACAGAAAGTAAACGCCTCGGGCCGAGGCGGCTAATCAGATGAAGAATAGTA
CACCAGGTCACCACCACCATGTTGATACCTTGACAGCGGCGCCGATGATTGATCTAAATTATGTGCCTTTTCCTGTTCACCAA
ATAGGGTGTAAGGACATGCGATACCAGATCCTGAGATAAGAGCTTGTTTAGTTCCAAAAAGAAACCTAAAAAGTGCTACAGT
ATCTGTCACATCAAATCTTACGGCACATGCATGGAGTACTAAATGTAAATGAAAGAAAAAACTAATTACACAGTTTAGTTGTA
AATCATGAGACAAATCTTTTGAGCCTAATTAATCCATGATTGGACACAAATTGTCAAATAAAACAAAAAGTGCTACAGTAGC
CGAACCCAAAAATTTCCCCAACTAAACAGGCCCTAAGGTAAATTCATGCAGGCTCTGTGTTGACTGTAGAGTGAATCTGTC
AAAATAGGTTATGTCAGATCAGGTAAATCATGTCACACTGTAATAATTCAACACGGATTGATTCAAGGATGACTGGGAAAAG
ACAAATAGTTCGTGCGACCAGTAAGCGTGGTCAGTTGGACTTCTTAATTGATCATAGAAGGTAGCCACCAATGAAACGTCAA
AGTGCCAACAAACAAGCGACCTGCTGTGTCTACTGCATAGAATACAATAGGTATGAGGAAATTGTCATAGCATCTAATCCTTC
ACTCCATTTTTCTCTCTGCTAACCACACCAAGAAGGGATCAGAAAGATGGAAGAGAGGGCTTCCTCCTCCTACCTTATGCTC

TTCCCTGATTAGACCATTTGGTGCTGCCTAGACAGGACACAGGTTCAGCATCTGCTTTCCATGCTTGTATCGAAAAGAAGCC
ATGCAGCCGCTCCATTTTTCATACAAGAAATACAGAAGTAGATTACCCATACCAGTTCTTGCCATTGCTCTTGTGCTGCTGATC
TCTCTGGCCACCCCCACCAGTTCCTGCACAGAGCAGGAGAAGAAATCTCTCCTCCAGTTCCTTGCTGGGCTCTCGAAGGATT
CTGGTCTCGCCAAGTCATGGAAGGAAGAAGGCACTGATTGCTGCAAATGGCAAGGGATCACCTGCGACGGTAACAAGGCG
GTCACTGAGGTCTCTCTGCCATCTAGAGGCCTTGAAGGGTCAATCAGACCTTCTCTTGGTAACCTCACCAGTCTACAGCACC
TTAACTTGTCCTACAACTCTCTGTCCGGTGTCCTGCCGCTAGAATTGGTGTCTTCTAGCAGCATCATCGTCCTCGACGTCAGC
TTCAACCACCTCAGCTGCATGAGCTGCCATCTTCAACCCCTGGCCAGCCTCTAAAGGTACTTAATATCTCAAGCAACTTGTTC
ACAGGGCAGTTTACATCCACAACATGGAAGGGG
>SsLRR-RLK256
AGGCGTGTGCCCCCACCTCCACGTTTGTTTCTGGGTGCCGTCCAACGTCCTTCCAGTCGTGCAATGCAATGAGCGCGCTGAC
CGACCGCGTGGGAAGGCCGCATCGTCCACGCGCATGCATGCCGTTGCCGTGGCTTCTGCTGCATCCTGCCGTGGCTTCTGCT
GCATCTCCATGCCTGGCTGCAGCCTGCAGGAAGCAGAGAGTCCGAGCCGACACGCCAGCCACTCTGCTATGCTCCCAGCAC
CAGCAGGTTCCTCTGCGATTGGCTCTGAATCCTGATAGCCGGATTATGTAAGGGGGTGTTTAGTTCCAAAAATTTTCACCCCA
AAACATCACATCGAATCTTGCGGCACATGCATGGAGCATTAAAATGTAGACTAAACAAAAAACTAATTGCACAGTTTACCGT
GAAATCGCGAGACGAATCTTTTAAGCCTAATTAGTCAATGATTGAACAGTAATTGCCAAATAAAACAAAAGGCTACAGTATC
CGAATCCAAAAATTTTTGCCAACTAAACACCACCTAACATCGACAGATACGTCTGCGAAAAATAAACAAATAGTGTGTGAAT
GGAAGGAGCAGACAAGACCCAAAGTGTTCTTCTAGGAAAAAGACACAGAAAGGGAGCCTTTCACACGGAAAGGGACGCA
GCGCTAGGCCGCTCTAGCTCTGCTCAGCATTCAGCATTCAGCAGGCTCTTAAAGCCTCGACAGTGTTTAGTGCCATCCACTT
GCTGTTCTGCAGTCGACACCACGAGAAAATAGTAGCCACCCGTGATGTCAGATAATCATACAACGACCACTATGAAATGTAC
AACTGCACCCACTAACCAAGCAATCAGTCATCATCAACATGAACATGATCAAGATGCATTGAAAAAAACACGAGATCTTGAG
CATCACTACCTGAAAAATTTCGATAGGCCATGTTTCACAGCAGCTTGCTTCAGGCACTCCACTCTCCGGGGCTGCTTCAAATC
TGTCTGCCAGTCCTCGTGCACACGCTGCATGCCCAGTTTACTTCCAGCCAGAGCCGAGGTCCATCACGCACCTGTGGAACA
GGGGAGGGGGAGCATGGGAAACAGCCGCGAGCGTGCACATCACTGCATCAGCTGCAAGGATAACAGAAAGTAAACGCCTC
GGGCCGAGGCGGCTAATCAGATGAAGAATAGTACACCAGGTCACCACCACCATGTTGATACCTTGACAGCGGCGCCGATGAT
TGATCTAAATTATGTGCCTTTTCCTGTTCACCAAATAGGGTGTAAGGACATGCGATGCCAGATCCTGAGATAAGAGCTTGTTT
AGTTCCCAAAAAGAAACCTAAAAAGTGCTACAGCATCTGTCACATCAAATCTTACGGCATATGCATGGAGTACTAAATGTAG
ATGAAAGAAAAAACTAATTACACAGTTTAGTTGTAAATCACGAGACAAATCTTTTGAGCCTAATTAATCCATGATTGGACACA
AATTGTCAAATAAAACAAAAAGTGCTACAATAGCCGAAACCAAAAATTTTCCCCAACTAAACAGGCCCTAAGGTAAATTCAT
GCAGGCTCTGTGTTGACTGTAGAGTGAATCTGTCAAAATAGGTTATGTCAGATCAGGTAAATCATGTCACACTGTAATAATTC
AACACGGATTGATTCAAGGATGACTGGGAAAAGACAAATAGTTTGTGCGACCAGTAAGCGTGGTCAGTTGGACTTCTTAATT
GATCATAGAAGGTAGCCACCAATGAAACGTCAAAGTGCCAACAAACAAGCGACCTGCTGTGTCTACTGCATAGAATACAATA
GGTATGAGGAAATTGTCATAGCATCTAATCCTTCACTCCATTTTTCTCTCTGCTAACCACACCAAGAAGGGATCAGAAAGATG
GAAGAGAGGGCTTCCTCCTCCTACCTTATGCTCTTCCCTGATTAGACCATTTGGTGCTGCCTAGACAGGACACAGGTTCAGC
ATCTGCTTTCCATGCTTGTATCGAAAAGAAGCC
>SsLRR-RLK207
GTGTGTACACTAATTTTTTGGTGTGGAATTAAATGTCACTTTATGGTGGTATTTTAATTTTTACAGTATTGTTATTTTATTGTGCG
ATCCGTATGTTTTTAATACAAAACGTTAGTTATCCCGTAGCAACGCACGGGCACGCTACCTAGTAATGGTAAAAAGTTAAATC
TCCCGAGCCTGCGACGTCGCAGATTAAGTACTATTTTACAAAAAGCCCCATTGTTTTATTGTTTTTGGATCTTGATATTTCGTA
ACGTAGAAGCCCCCCACATTTTACAAGCTACCCCTATTAAGAATTGTGATTAAATAATACTCATTCCATTTTAAATTGTAAATC
GCTTATTGTAAAATATATGTATCAGAACGACTTACAATATAGAACAAATGAAGTACTCCTTGTGTTTCAAATTATAAGACATTTA
AACTTTTTTAGATAATTGCTTTTACTATATATCTACGAGTAGACTAGATCTAGTGTATATCTAAGTGCGTAACACAACATACATC
TAGAAAAATCAAAACACTTATAATTTAAAACAGAGGAAGTAACATTTTACAAAAGTCTAATTTTATCGTTCAATTCTCACCGG
AGTCTAAATTTGACCACGCGACCGAATATCACACCGGAGTCTAAAAAAGTTGAGGGCGGGGCGCTGACCAGACCGCGTCCA
CGCCCGCGCGTGCCACTCGTTGGACTCTGGACGCGTACGGTATGCTGCGCGCTCCGCTCACTGCCTTGCCAGCCGTGGTCCG
TGGAGTACTGCGGCCGCCCCTGTCTGCCTGCACTCCAGCAAGTCAGTAAGACTACCCATAGTTGGTACTCTAAAGGGATACT
TTAAAGCTTTAGAGTATGAATATAGAGTAAAAACTGCTGCACTGGGAACTTAAAACGATACTCTATCCTTTCTCTCACTCTATA
TTTAGAGTGGTACAGTGCTTACTCTACAGTAAGCAGTGGTGGGCCCCACTAGCTAAACAAAGGAAAAAAATGCTATAACTCA
CAAATCAAAAATTCAAATTAAATTTTGATTGCACCATTAAATCCGTCTCAATAAATCCTTCAAAACTAGATCACACATGGATAT
ATTTAGATAAAGTGACAAGTGGGTCCCACAGTAGATAGAGTGGATAGAGGATAATATAGAGGATGGATGGGTGCGAGAGAAT
TGAGTATAGAAGAGAGAATCTTGCTGACTAGGACATATAAAGGATGAATATAGAGGTCCATGAGTGCGGATAGCCTTGGTAG
AGCGCAAGCCGGCACGCGTACGCCAGCCCCTCGCTCCATCTTCAGCTGCAGGCCTGGCTCTGCAGCTATACGCTCCATCTCC
CAATCTCCCAGGTCCCTCCAATTGCCTTTCAACCTGAACACATGATAGATGTGTTAAGTTACGATGTGTTTGAGGAAATAAAC
AAACTCAGCGTAAATGCAAGGAACACAAAAAACAAGTATATATGCACAAACAAATAGGGACGCAACGCAACGCCTGCTCA
GCAGATTCTTCGGCTCAGCCAGCCACATGCTGTTCGGTTCTGCAATCAACACCACGAGATGACAGATAATCATACAATGGCT
ACTAAGAGCCAATACAAAATGTGCAATTGCGCTCATACTGTAACCAAGCATAATCATCAACATAATCTCAACCAATAAAAAAT
TACTTCCCCTCCGTTATTAGCTGTTCTACACTAGGTCAGTAAATTATTGGTGACAGGTAAACCCAAACAGAGGGAAGTACAAT
ATCACGATGACCACAGACAGGTAAACCCAAACGGAGGGAAAGTAAATTATTGATGACAGGTAAACCCAAACAGAGGCAAG
TGCAAACACACGAGATTATTGTCATCACCACCTGAATGAATAAATTTGATAGGCCAATTTCACACTTCACGGCAGCACCTCCA
TGGCTGTTACTTCCAGCACTTTTGAATTCCGGTCAGCAACACAAGCCGTGGCCCGTGGACCAAAGCTCCATCATGCGCCTCT
GGAACTTGGGCCA
>SsLRR-RLK220
TTGTAGCATGTACCTGTAAATCGAGTGGCACAGAGTTGGCAAGGCAAGCTATGCAAATCAAGATAAATTGTATCGGAGACGA
TGAGCCCACTAGTTTTATTCTTGAGTTTGTATTAAATAAATGATGTAACCATCCATAATTAATAAAGCTTTGCTGGATGCATTTG
TCAAAAAAAAAACATCTCCTTAACCATATATCCAATTTGCAAGTTGTTTTTCACCATTTTATTTCTCGTACCGTGATCCCAACG
ACAGAACAAGATCCCACGTTTTATATTTTTTTCATGAACGTGCTTGATTTCCATGCACGTGTGCCATGTTTTACAGCGAAGAA
AAAGATAATAGCATGTGAATCCTACAGGTCATTTTCACACAGGTCTAGCAAAATTTGGCAGGACAACTGTCCAAACACTCTT
CGCAGCTCTACATTTTGGTATTGGCCAAAATTTTGTTCCGTCGCAAACCAAGCATTAATTTATAGACCTAATTTTATCACTAGT
CAAATTTGGCCATAACCAAGATTTGACTCAACCAGGCACCTCCACGTTTGTTCTGGGTGGGTCCCGTCTCCATCCAGGAGTG
GGAAGACCCGCGTGGCTCGTCCTGCACGCGCTTGCATATGCCGTTGCCGTGGCTTCTGCATCCTCCTGCCGTACGTGCTTCG
GCTTCCACTCGATTATGTAACATAGATAGATACGTCTGCGAAAAATAAACAACTAGAGTGTGAATGCCAGGAGCAGACAACA
AGATCCAAAGTGTTCTTCTAAGAAAAAGACACAGAAAGGGACGCATTGCTAGCTCTGCTGCGCTCAGCAGGCGGCTCTTAA
AGCCTCGACAGTGTTTAGTGCCATCCACTCGCTGTTCTGCAGTCGACACCACAAGAAAATAGCAGCCACCCGTGACCGTGA
TGACAGACAATCATCAACATGAACATGATCAAGATGGAAGAGAGGGCTTCCTCCTCCTCCTACCTTATGCTCTTCCCTGATTA
GACTATTCGGTGCTGCCTAGACAGGACACAGGTTCAGCATCTGCTTTCCATGCTTGTATCGAAAAGAAGCCATGCAGCCGCT
CCATTTTTCATACAACAAATACAGGAGTAGATTACCCATACCAGTTCTTTCCATTGCTCTTGTGCTGCTGATCTCTCTGGCCAC
CCCCACCAGTTCCTGCACAGAGCAGGAGAAGAAATCTCTCCTCCAGTTCCTTGCTGGGCTCTCGAAGGATTCTGGTCTCGCC
AAGTCATGGAAGGAAGAGGGCACTGATTGCTGCAAATGGCAAGGGATCACCTGCGATGGTAACAAGGCGGTCACTGAGGT
CTCTCTGCCATCTAGAGGCCTTGAAGGGTCAATCAGACCTTCTCTTGGTAACCTCACCAGTCTACAGCACCTTAACTTGTCCT
ACAACTCTCTGTCCGGTGTCCTGCCGCTAGAATTGGTGTCTTCTAGCAGCATCATCGTCCTCGACGTCAGCTTCAACCACCTC
AGTGGAGACCTGCATGAGCTGCCATCTTCAACCCCTGGCCAGCCTCTAAAGGTACTTAATATCTCAAGCAACTTGTTCACAG

GGCAGTTTACATCCACAACATGGAAGGGGATGGAGAATCTAGTTGCGCTCAATGCCAGCAACAATAGTATTACTGGTCAGAT
TCCAAGCCATTTCTGTGACATATCTCCATCCTTTGCGTGCTTGAACTCTGCTTCAACCAATTCAGTGGCAGAGTTCCACCAGG
GCTTGGTAATTGCTCAACACTGAGAGTGCTCAAAGCTTGCCACAACAACCTCAGTGGGACACTCCCAGATGAACTGTTCAA
CGCTACTGCGTTGGAGTACCTGTCTTTCTGTAACAATGATTTGCACGGGGTACTTGATGGTGCACGCATAATCAAACTCAGAA
ATCTGGAAATTCTTGATCTTGGGGGAAACAACTTCACTGGCAGGCTTCCGTATGCCATAGATCAGCTGAAGAGACTGAAGGA
GCTCCATTTGGACCACAACAAC
>SsLRR-RLK234
GCATCCTCCTGCCGTACGTGCTTCGGCTTCCACTCCATGTCTGGCTGCAGGAAGCAGAGTCCGAGCCGACACGCCAGCCAC
TCGCTCCTCCTCAGCTGCTATGCTCCCAGCAGGTTCCTCTGCGATTGGCTCTGACTCCTCATAGCCGGATTATGTAATACGTCT
GCAAACATAAACAACTGTGAGTGTGAATGCAAGGAGCAGACAATCACAAGATCCAAAGTGTTCTTCTAAAGAAAAAGACA
CAGAAAGGGACGCATTGCTAGCTCTGCTCAGCTCAGCAGGCTCTTAAAGCCTCGACAGTGTTTAGCGCCATCCACTCGCTGT
TCACCCGTGACCGTGATGATAGACAATCATACAACGACGGAACGACCACTATGAAAAGTGCAACTGCACCCACTAACCAAG
CAAGCAATCATCATCAACACGAACATGATCAAGATGCCCAGAAAACACACACACACACACACACGAGATCTTTAGAATCAC
TACCTGAAAATTTCGATAGGCCACGTTCATTCATGCACTCCACTCTCGGGGGGCTGCTTCAAATCTCAGCTTGCCTGCCTGTC
ATCGTGCACACGACGCGGCCCAGTTTACTTCGAGCGCGTTTGGATTCCGGTCAGCAGCCAACAAGAGCCGCCGGGGTCAGG
GGCCAGTGTACCTGTGGGTCAGGGGCCATGGGAAACAGCCACGAGCGTGCACATCACTGCATCAGCAGCAAGGATAACAG
AAAGTAAACGCCTCGGACGAAGAGTAGGGCTCCAGGTCGCCCTCAGCCACCACCTTCTGCCTTTCTTATTTCTCCGGCCTCC
ACTTGCGACAAATGCTATACTCCAAAGTCCAAACCATGTTGATACCCTGACCACATCGCACGATGATTGATCTAAATTATTGTA
CGCGTGCCCGGGAGTGATGCTCTCCGAGTACTGTAGTGTCTTAAAATTGAATCTTGAAGATCTTCGGTTTCTTTGGGAAACGT
GTAGCTTTTCCTGTTAACAAAAAAAAGGTGCATTATGTATAAAAATAGAAACATTCATCAAGCAGTGGTTCCATTTTTCTTTTA
CCTTTTCAGTACTAATTACTGAGGTTAAGACAACAGGTTGGCTAGCTGAAGCTGGTAGCACAAGTGATGCCATATCCTTTTTT
TTTATAAGTGTGATGCCATATCCTGATGTCAGATAGAAAGGAGGCTGTATATCCAGCCCTTGTTTAGTTCCCAAATTCAACCCT
CAAATTTCACTATGCAAAAAGAAGATTCCCATCACATCAAAATACCGGCACATGCATGGAATACTAAATGTAGATGAAATTTA
AAACTAATTGCACAGTTTTATTGTACTTTACGAGACGAACATTTTAAGCCTAATTTGTCAAAATTTGAACAATTTTTACCAAAT
ACAAACGAATCGCTACAGTGCGCATTTAATGCGCACTGTAGCGAACTGTTCCAAATCGGGGGTGCCCGATTTGGGAACTGAA
CAAGGCCCCAGTCAAATTTGTCACAAGAAGTTATGTCTGATCAGGTAAATCTCGGCAGCCTGTAATAATAATTCAGCCAGGAT
TGTTTAATGATGACTCGGAGAAGGCAAATATTTCCCCTGTTCGGCTGAGCTTATCAGCCGTGGAACAGTGTTTTTCTCTCACA
GCAAATCAGCCACAGCCGGCTTATAAGCCAAAAAACAGCCTGCCGAACAGGGCCATTTTATATGGCCACGAAGCTTGTTCA
GATGGATCAGGTATGAAACGTCAAAGTGTGAACAAGCAGGCTGCTTCGTCTGGTGTATAAAATTGAACCATAATGAGCATTT
TGTTCTAGCATCTAATCCTTGACTCCATTTTTCTTTGGGTGGCCACGGCTATCGAGGGAAAAAGCAAGAGCCAAGAGCCGCC
TCCTTCTACTTCTATCTCATGCCCTTCCCTGTTTAGACCATTGGTAGTTCGTCAAAACAGGGCATAACTTAAGCATCTGGTTCC
CATGCTAGTTTCAATAGGAAGCC
>SsLRR-RLK210
GTGAACGTGCTTTGATTTCCATGCACGTGTGCCATGTTTTTATAGTGAAGAAAAAGATTTGGCAGGACAACCATCCAAACAC
TCTTTGCAGCCCTGCATTTTGGTATTGGCCAAAATTTTGTTCCGTCACAAACCAAGCATTAATTTATAGACCTAATGTTATCAC
TAGTCAAATTTGGCCATAACCCGGATTTGGCTCAACCATGCACCTACCGTACGTGCTTCGGCTTCCACTCCATGTCTGGCTGC
AGGAAGCAGAGAGTCCGAGCCGACACGCCAGCCACTCGCTCCTCCTCAGCTGCTATGCTCCCAGCAGGTTCCTCTGCGATT
GGCTCTGACTCCTCATAGCCGGATTATGTAACATAGATAGATACGTCTGCGAAAAATAAACAACTAGAGTGTGAATGCAAGG
AGCAGACGATCACAAGATCCAAAGTGTTCTTCTAAGAAAAAGACACAGAAAGGGACGCATTGCTAGCTCTGCTCAGCTCAG
CTCAGCAGGCTCTTAAAGCCTCGACAGTGTTTAGCGCCATCCACTCGCTGTTCACCCGTGACCGTGATGATAGACAATCATA
CAACGACGGAACGACCACTATGAAAAGTGCAACTGCACCCACTAACCAAGCAAGCAATCATCATCAACACGAACATGATCA
AGATGCCCAGAAAACACACACACGAGATCTTTAGAATCACTACCTGAATAATTTCGATAGGCCGTGTTCATTCATGCACTCCG
CTCTCGGGGGGCTGCTTCAAATCTCAGCCTGCCTGCCTGTCATCGTGCACACGACGTTGCCCAGTTTACTTCGAGGAGCGCG
TCTGGATTCCAGTCAGCAGCCAACAAGAGCCGCAGGGGTCAGGGGCCAGTGTACCTGTGGATCAGGGGGGCCATGGGAAA
CAGCCGCGAGTGTGCACATCACTGCATCAGCAGCAAGGATAACAGAAAGTAAACGCCTCGGGGCCGAGGCAGCCAATCAG
ACGAAGAATAGGGCTCCAGGTCGCCGTCATCCCACCACCTTCGTCATCCTCTCTGCCTTTCTTATTTCTCCAGCCTCCACTTG
CGACAAATGCTATCCTCCAAAGTCCAAACCATGTTGATACCCTGACACATCGCCCGATGATTGATCTAAATTATTGTAGGTGC
ACCCCGGAGTGAATCTTGAAGATCTTCAGTTTCTTTGGGAAACGTGTAACTTTTCCTGTTAACAAAAACATGTATAAAAATAG
AAACATTCATCAAGTAGTCATTCCATTTTTCTTTACTTTTTCATTCAGTACTAACAGCTGAGGTTAAGACAATAAGTTGGTCAG
CTGAAGCTGGTAGTGGTAGCACAAGTGATGCCATATCCTGATCTCAGATAGAAAGGCAGGATGTATATTCGGTCAAATTTGTC
ACAAGAAGTTATGTCTGATCAGGTAAATCTCGGCAGCATGTAATAATAATTCAGGTAAATCTCGGCAGCATGTAATAATAATTC
AGCCAGGATTGTTTAATGATGACTCGGAGAAGGCAAATATTTTGTATGGCCAAGAAGCTTGTTCAGATGGATCAGGTAGCCA
CCAATGAAACGTCAGTGTGAACAAGCAGGCTGCTTCGTCTGGTGTATAAAATAGAACCTTCTAGCATCTAATCCTTGACTCC
ATTTTTCTTTGGGTGGCCACGGCTATCAAGGGAAAAGCAAGAGCCAAGAGCCTCCTCCTTGTACTTCTACCTTATGCTCTTCC
CTGTTTAGACCATTGGTAGTTTGTCTTAAACAGGGCATAGCTTAAGCATCTGGTTCCCATGCTAGTTTCAATAGGAAGCCATG
CAGTCACTCCACTTTTCATACAAGAGGAACCAAAACAGATTGCACATACCTTCCATTGGCCTGACACTTGTGCTGCTCATCTC
CTTGGCGCCTTGCACCAGTTCCTGCATGGAGCTGGAGAGGACATCCCTCCTCCAGTTCCTCTCAGAGCTCTCGTGGGATGCC
GGTCTCACCAAGATTTGGCAAGGT
>SsLRR-RLK257
CCGCCACGAAAAATTCCCGATCGTCGTCGTCGGGGGCGACATCCAGCGGGGCTGGTTCGATTTTGGCACACGCCCGCCCCA
AGATGGCCTGCACTACGCTCGACGAACGCGCGTGCAGCGGGACGCGTTGCAATGCCACCAGAACCCTGAACCGGAAGGAC
TCTGCCGTGGCTAAGGACGTCCGTCTCCATGGATGCCAAATGAGGAGGAAAGGTGGGTCATGGATCCTTGTATGCAACACCC
TTAACATGTCGTCGCAGCTCGTAAATCTTACAATGAAATCTTCAGGTTCATGCCGACGCACAGACGCATCCCTGATGCCGAA
GCGCTCCTCAAGGTAGGCACGGACCATGGCGGGGGACACATGGGGCCTGGTGCCGGCAATGACAGCCACCAGAGCCCGGG
CAAGGTCCCGCTCCGCCGCCTCCAGCTCCGCAGAGCGAGGCACCACTACCAGGTCGACCGGCAGCTCCCGCACTAGGCCA
GGCGCTTCACGTGGCGGTACATCGGCCGGCTGCGACGAAGGGGCGCAGCACTGTGGCAACGAAGTAGATCGCCCCGTAGA
GACGTGGCGGCCGGATATCGTGTCGCCGGAGCCCCGTGAGCGCCCACTCCGCGAGGACGGCTGTCTGGGCGCCTGCTGACG
GTGCGCGGCCGCCGCATACCGGGAACGGCGCTGCTTCCACGAGTGGCGCCGCTTCGTCGAGTGGCCCGCCACCAAGGGGG
GGAATGGGCAGCCCCGCAGGCGATGGCATTCGCTCCCACAATTGGTGCAACACGGCGGTGAGGAGCATTGGGCCTTGACAT
GCTGTTCGCTCCCGCAGTTGAAGCATTTGCCCACTAAGTGCGGCGGCACTGGCCTGAAAGGCCTGGAAGAGGCACGCCTGC
GCCACCGCTTCCTGCTCTGGACGCAGCGGAAGCCGTCGGCATCAGGGACGCCGGAAGCACGCGCCGGGTGGGCGGCCGGG
GGGTGCCGACGGCGCCGGTCATCACTCCCACGTTGCCGGGAGTCCTGAGAGAGGCCTTCAACCGGCGGGTGGGAACGGCG
TGCCGCAGCCATGAATCCGCCCACAATCCCAGCACGACGGCGACGGCGTCGGTGGCGTCGGCGCCGGGCGCCCCCCTGAA
CCACCGTCTTCCCCTTGTCCTCCGGACGTGGAGAGGCAGCCTCAGAACCTTCCTCCAACCCGAAGGAGGCTTCCGAGTCAA
AGAAGGAGAGGTGGCCGCCGAAGCCCTCAACAGGAGAGCCGCCGTAGAATGGCGCCGCGGCCAGGCCGGGGCCGACCGG
ACCGGAGTCGGCCGGCGAGGAGGCACCATAGAAGGGGGCCGCATTGGGGTTAAGACCCGCGGACGCGGCCCGCACTTCCC
CGGCTACTTCATCCACCCAGGAGTATCGCGAGGCGGATCCACCCTCCATGGCACCAAACCGACGAGGAGGGGGGGGGGTGC
AGATCCGGGGCGGCCAACGCCCGATCCGCGCCCGAGAGGGCGGGATGTAACGCCGGAGCTCCCCGACCAGAGAGCAGGGG
CCGGGGCAAAAAGGGGGGGAAAAAGGGGGGTCGACGGGAGAGCGAGCGCAGAAACGGTGGGCGGCGCAGCCGGAGTGG
CAAGCGGCGCGGCGGCGGGGCCCGGGGGGGGGGGGTGGCACCGGCGCTGGGGGGTGGGCGGCGGGGCCGGGGTGGCC

GCCGGCGCTGGGAGCGGGCAGGGTTAGTTAACTACTTTCATCCATGGACAATTATCAGAAGGACTAGGCAATCTGAAGTCCC
TCTCGTTCCTGTCGCTTACAAACAACAGTCTTTCAAATATAGCAAATGCACTTCAGATACTAAGGAGCTCCAAGAACCTGAC
CACACTTCTTTTTGGGATCAATTTCTTCAACGAAACCATACCAGATGATGCCGAAATTTATGGTTTTGAGAATCTTCAGGTTTT
GGACATCGGAAGTTGCCTCTTATCGGGGGAAATACCTCTTTGGATATCAAAGTTAGTAAATTTGGAA
>SsLRR-RLK221
CGCGAACGTGCTTTGATTTCCATGCACGTGTGCCATGTTTTATAGTGAAGAAAAAGATTTGGCAGGACAACCATCCAAACAC
TCTTTGCAGCTCTACATTTCGGTATTAGCCAAAATTTTGTTCCGTCGCAAACCAAGCATTAATTTATAGACTTAATTTTATCACT
AGTCAAATTTGGCCATAACCAGGATTTGGCTCAACCATGCACCTCCACGTTTGTTCTGGGTGGGTCCCGTCTCCATCCAGGC
GTGCAGCGCAATGAGCGCGCTGACCGACCGACCGCGTGGAGTTGGAGTGGGAAGACCGCGTCCTCCACGCGCAATCGCGC
ATGCCGTTGCCGTGGCTTCTGCTGCATCCTGCCGTGCTTCTACTTCCATGTCTGGCTGCAGGAAGCAGAGAGTCCGAGCCGA
CACGCCAGCCACTCGCTCCTCCTCAGCTGATAATGCTCCCAGCAGGTTCCTCTGCGATTGGCTCTGACTCCTCATAGCCGGAT
TATGTAACATAGATAGATACGTCTGCAAAACATAAACAACTGTGAGTGTGAATGCAAGGAGCAGACAATCACAAGACCCAA
AGTGTTCTTCTAAAGAAAAAGACACAGAAAGGGACGCATTGCTAGCTCTGCTCAGCTCAGCTCAGCTCAGCTCAGCAGGCT
CTTAAAGCCTCGACAGTGTTTAGCGCCATCCACTCGCTGTTCACCCGTGACCGTGATGATAGACAATCATACAACGACGGAA
CGACCACTATGAAAAGTGCAACTGCACCCACTAACCAAGCAAGCAATCATCATCAACACGAACATGATCAAGATGCCCAGA
AACACACACACACACACACACACACACACACACACACACACACACACACACACACGAGATCTTTAGAATCACTACCTGAA
AATTTCGATAGGCCACGTTCATTCATGCACTCCACTCTCGGGGGGCTGCTTCAAATCTCAGCTTGCCTGCCTGTCATCGTGCA
CACGACGCGGCCCAGTTTACTTCGAGCGCGTTTGGATTCCGGTCAGCAGCCAACAAGAGCCGCCGGGGTTACGGGCCAGTG
TACCTGTGGATCAGGGGGCCATCGGAAACAGCCGCAAGCGTGCACATCACTGCATCAGCAAGGATAACAGAAAGTAAACGC
CTCGGGGCCCAGGCAGCCAATCAGACGAAGAATAGGGCTCCAGGTCGCCGTCATCCCACCACCTTCGCCATCCTCTCTGCCT
TTCTTATTTCTCCAGCCTCCACTTGCGACAAATGCTATCCTCCAAAGTCCAAACCATGTTGATACCCTGACCACATCGCCCGA
TGATTGATCTAAATTATTGTACGCGTGCCCCGGAGTGATGCTCTCCGTGTACTGTAGTGTCTTAAAAATTGAATCTTGAAGATC
TTCAGTTTCTTTGGGAAACGTGTAACTTTTCCTGTTAACAAAAACATGTATAAAAATAGAAACATTCATCAAGTAGTGATTCC
ATTTTTCTTTACTTTTTCATTCAGTACTAACAGCTGAGGTTAAGACAATAAGTTGGTCAGCTGAAGCTGGTAGTGGTAGCACA
AGTGATGACATATCCTGATCTCAGATAGAAAGGCAGGATGTATATTCGGTCAAATTTGTCACAAGAAGTTATGTCTGATCAGG
TAAATCTCGGCAGCATGTAATAATAATTCAGCTAGGATTGTTTAATGATGACTCGGAGAAGGCAAATATTTTGTATGGCCAAG
AAGCTTGTTCAGATGGATCAGGTAGCCACCAATGAAACGTCAAAGTGTGAACAAGCAGGCTGCTTCGTCTGGTGTATAAAAT
AGAACCTTCTAGCATCTAATCCTTGACTCCATTTTTCTTTGGGTGGCCACGGCTATCAAGGGAAAAGCAAGAGCCAAGAGCC
TCCTCCTTGTACTTCTACCTTATGCTCTTCCCTGTTTAGACCATTGGTAGTTTGTCTTAAACAGGGCATAGCTTAAGCATCTGG
TTCCCATGCTAGTTTCAATAGGAAGCC
>SsLRR-RLK258
ACGTGTGCACCGACCAATGCATCCATGCAACAAGCACGGCATCGATCCCACCGCAGCAACAACCAGCAGCATACGGCGAAG
CACGCTTTATTCGTTCTGCAATGTAAGTTCATAGCCTCTCAGTTCACACTGCTGCTCCGGTTTACGTCGCCTGGTGGTTCGTC
AGTCGCCATCGTCATGCTACGCAGGATCCCACAGCAGGCCGACCGCTGAGTACATGTCGGAAAGCAATGGCTGTAGTGCTG
AGGAGGTGAGCTCTGGTTCAGCACCAGCAAGCAAGCAGAGAGATTAGAAGAAAGATTTAGGGGAGGAAGATGACGTGGCA
AAACCTGGCCATTGTATAACCGTGTCCTGTGGCGGAGCCAGAAGCTTTTAGAAATCCAAGTAATATTTATTATAAAAAATATG
TACTTAGCAGATTACAATGTTCTTCTTTAATATAAAGAATATTTTCTTTTTAAATTTCATCGACAAGCTCGGCTGAGCTGCGCCT
TCGCTATTGGCTATGCCACGTCATCAAAACCACCTAGACCTAATGCCACACACCTAAACCGGTTTTGGTTTAGAGCAAAACG
TGAAAAACAATCTATTGGAAAATGACACAGTTAAGTAGACGATTTCATAAGGTTTTTCTTGATAAGAGACGATTTCATAGTTG
AGTGTTACTTGAACCGAGGGCGAACTTGAAGTTGAAGATGGTAATTTGGACCTATTCCAATAGCATACCACCACCTAATAAAT
ATATCCGGTAACAACTGAATGGTGTGGCTGCCAAAGAAACAAACACAAGAGATTAGGCCAGTCTCAATGGGTAATTTTATTA
TGTTGTTTCCAAGATTGCCATGTCATTTAAAATGAAATGAAACTAGGATGAAACACTCACTCTCAATCGAGAGTTTTATTCCA
TGGTTTCATAGTCATTTAATTCCAAGACTCATAGAGAGTTTCATCTAGATGAAACTCATTTCTTCTCTCTCTTCTTAAATATATT
TACTTAGCAAATTACAATGTTCTTCTTTAATATAAAGGAAATTTTCTTTTAAATTTCATCGACAAGCTCGGCCGAGAAGTGCCT
TCACTATTGACTGTGACACGTCATCAAAACCACCTAGACTTGATGCCACAGCTAAACCGGTTTTGGTTTAGAACAAAACGTG
AAAAGCAATCTTTTGAAAAATGAGAGAGTTAAGTAGACGATTTCACAGTTTTTTTTAATGTAAGAGATTTTTTCAATGTAAGA
GACGATTTCATAGTTGAGTGTTACTTGAACCGAGGCTGAACTTTAAGTTGAAGAGGGCAGTTTGGACCTATTCCAATAGCAT
ACCACCACCCAATAAATATATCCGGTAACAACTGAATGGTGTGGCTGCCAAAGAAACCATTTTCTTTGCACAAAAACACAAG
AGATTATATCAGCCTCTCAAGTGGACAGGGTTGCTTCGATTTTCGCCATATTGATTATATATAAAATATAATTGATAGATATATAG
AAACGGTACACTGCAAAATGTCAGCCGACACTTGAGGTCAACTAAAGCAGAAGAAACAAGGGCAAAATAATTGAACCGGT
TCATGCACACGTGAGCACCACATTTTGGTCAATGAAACAGCTGATGAAGATGAAATGAGAACTAGAAAAGTCTATCATGCTG
CTTTGGGGACTCAAACTGGCTACAACAGCACTGAAGAGTAGCTGCATTAAGTAAAGACTGAGATAATGAATGAAAATAATAA
CAGTAGTGTATATATTAACAATTATTAATATGAGATCGAGTCACCATCTAAAGTTATGCATATCCTGCAACTTTGACTCAATAAT
CACATAATCAATGGGCGGGTGGGTACCAAAGCAGACTTTTGTTTGCGCAGAAGGTTTGGACTTTTTCATAGATTATCATATAG
TATATACCAGGCTGGCTCATCCAAAACGTGAAGGTCTGACCGTGTGAATAAGCCGGGAAGCTTGCCGGAAACGGGTGTGGA
CGTGTGCCCACATCAGC
>SsLRR-RLK222
CCGCAGCAACAACCAGCAGCATACGGCGAAGCGCGCTTTATTCGTTCTGCAATGTAAGTTCATAGCCTCTCAAGTTCACACT
GCTGCTCCGGTTTACATTGTTTCAATGTGCTCATAGAGATAGTGTATGCACACGTGTTTATAAAAGATAAGTGTTAAAGTGTAT
ATACATATATGTAAGCGTCTATATATGTATTGTAATTCATAAAAATATATACAATATATAGTAATTCACATATGCAATGATGCATTG
GTTCTTTGTTTAAGCACATATGTGGCAACGATGAAAAACTTTCGAGGAGCAATGACTTCACCGATAAGTTCGTAGCCTCTCGT
CGCCTGGTGGTTCGTCAGTCGCCATCGTCATGCTACGCAGGATCCCACAGGCCGACTGCTGAGTACATGTCGGAAAGCAATG
GTTGTAGTGCTGAGGAGGTGAGCTCTGGTTCAGCACCAGCAAGCAAGCGTTGAGATTAAGAAAGATTTAGGGGAGGAAGAT
GACGTGGCAAAGCCTGGCCATTGTATTACCGTGTCCTGTGGCGGAGCCAGAAGCTTTTAGAAATCCAAGTAATATTTATTATA
AAAAAATTTACTTAGCAAATTACAATGTTCTTCTTTAATATAAAGAATATTTTCTTTTAAATTTCATCGACAAGCTCGGCCGAG
CTGTGCCTTCGCTATTGGCTATGCCACGTCATCAAAACCACCTAGACTTGATGCCACACAGCTAAACCGGTTTTGGTTTAGAG
CAAAACGTGAAAAACAATCTTTTGGAAAATGACACAGTTAAGTAGACGATTTCATAAGGTTTTTCTTGATAAGAGACGATTT
CATAGTTGAGTGTTACTTGAACCGAGGCTGAACTTTAAGTTGAAGAGGGTAGTTTGGACCTATTCCAATAGCATACCACCAC
CCAATAAATATATCCGGTAACAACTGAATGGTGTGGCTGCCAAAGAAACCATTTTCTTTGCACAAAAACACAAGAGATTAGG
CCAGCCTCTCAATGGGACAGGGTTGCTTCGATTTTCGCCATCTTGATTATATATAAAATATAATTGATAGATATATGGAAACGGT
ACACTGCAAAATGTCAGCCGACACTTGAGGTCAACTAAAGCAGAAGAAACAAGGGCAAAATAATTGAACCGGTTCATGCA
CACGTGAGCACCACATTTTGGTCAATGAAACAGCTGATGAAGATGAAATGAGAACTAGAAAAGTCTATCATGCTGCTTTGGG
GACTCAAATTGGCCACAACAGCACTGAAGAGTAGCTGCATTAAGTAAAGACTGAGATAATGAATGAAAATAATAACAGTAGT
GTATATATTAAAAATTATTAATATGAGATCGAGTCACCACCTAAAGTTATGCATATCCTGCAAGGCCTCGTTTAGTTCAGTGAAT
TGGGGAGAAAAACACTGTATCAGAATTCCTGTAGCATTTCGTTTGTATTTGGTAAAAATTATCCAAATTTTGACTAACTAGGC
TTAAAATGTTCGTCTCGCAAAGTACAACAAAACTATACAATTAGTTTTTGATTTCATCAACATTTAGTACTCCATGCATTTGCC
GACATTTTGATGTGACGGAGAATTTTCTTTTTGCATAGTAGAAATTGGGAGAGAATTGGGGGAACTAAACAAGGGGCAACTT
TGACTCAATAATCACATAATCAATGGGCGGGTGGGTACCAAAGCAGACTTTTGTTTGCGCAGAAGGTTTGGACTTTTTCATA
GATTATCATATATATACCAGGCTGGCTCATCCAAAACGTGAAGGTCTGACCGTGTGAATAAGCCGGGAAGCTGCTACCAGCTA
TGGCAATTGTGCCGGAAACGGGTGTGGATGTGTGCCCACATCAGCCATGCAGTACTTGACCCAATTTTCATTTCCTGCGGCT

GCAAGCCTGCAACAGATAAACAGGACAGAATCAAACTGGGGGCTGCTTGTTGTCTCCTACATCTTCTTCCTCCTCGCGCTCT
TTCCTCATTGGGC
>SsLRR-RLK211
GTATTACCGTGTCCTGTGGCGGAGCCAGAAGCTTTTAGAAATCCAAGTAATATTTATTATAAAAAAATTTACTTAGCAAATTAC
AATGTTCTTCTTTAATATAAAGAATATTTTCTTTTAAATTTCATCGACAAGCTCGGCCGAGCTGTGCCTTCGCTATTGGCTATGC
CACGTCATCAAAACCACCTAGACCTAATGCCACACACCTAAACCGGTTTTGGTTTAGAGCAAAACGTGAAAAACAATCTATT
GGAAAATGACACAGTTAAGTAGACGATTTCATAAGGTTTTTCTTGATAAGAGACGATTTCATAGTTGAGTGTTACTTGAACCG
AGGGCGAACTTGAAGTTGAAGATGGTAATTTGGACCTATTCCAATAGCATACCACCACCCAATAAATATATCCGGTAACAACT
GAATGGTGTGGCTGCCAAAGAAACAAACACAAGAGATTAGGCCAGTCTCAATGGGTAATTTCATTATGTTGTTTCCAAGATT
GCCATGTCATTTAAAATAAAATGAAACTAGGATGAAACACCCACTCTCAATCGAGAGTTTTATTCCATGGTTTATAGTCATTTA
ATTCCAAGACTCATAGAGAGTTTCATCTAGATGAAACTCATTTCTTCTCTCTTCTTAAATATATTTACTTAGCAAATTACAATGT
TCTTCTTTAATATAAAGAAAATTTTCTTTTAAATTTCATCGACAAGCTTGGCCGAGCAGTGCCTTCGCTATTGACTGTGACACG
TCATCAAAACCACCTAGACTTGATGCCACAGCTAAACCGGTTTTTTGGTTTAGAACAAAACGTGAAAAGCAATCTTTTGAAA
AATGAGAGAGTTAAGTAGACGATTTCACAGTTTTTTTAATGTAAGAGATTTTTTTTAATGTAAGAGACGATTTCATAGTTGAG
TGTTACTTGAACCGAGGCTGAACTTTAAGTTGAAGAGGGTAGTTTGGACCTATTCCAATAGCATACCACCACCCAATAAATAT
ATCCGGTAACAACTGAATGGTGTGGCTGCCAAAGAAACCATTTTCTTTGCACAAAAACACAAGAGATTATATCAGCCTCTCA
ATTGGACAGGGTTGCTTCGATTTTCGCCATCTTGATTATATATAAAATATAATTGATAGATATATGGAAACGGTACACTGCAAAA
TGTCAGCCGACACTTGAGGTCAACTAAAGCAGAAGAAACAAGGGCAAAATAATTGAACCGGTTCATGCACACGTGAGCAC
CACATTTTGGTCAATGAAACAGCTGATGAAGATGAAATGAGAACTAGAAAAGTCTATCATGCTGCTTTGGGGACTCAAATTG
GCCACAACAGCACTGAAGAGTAGCTGCATTAAGTAAAGACTGAGATAATGAATGAAAATAATAACAGTAGTGTATATATTAA
AAATTATTAATATGAGATCGAGTCACCACCTAAAGTTATGCATATCCTGCAACTTTGACTCAATAATCACATAATCAATGGGCG
GGTGGGTACCAAAGCAGACTTTTGTTTGCGCAGAAGGTTTGGACTTTTTCATAGATTATCATATATATACCAGGCTGGCTCATC
CAAAACGTGAAGGTCTGACCGTGTGAATAAGCCGGGAAGCTGCTACCAGCTATGGCAACTGTGCCGGAAACGGGTGTGGAT
GTGTGCCCACATCAGCCATGCAGTACTTGACCCAATTTTCATTTCCTGCGGCTGCAAGCCTGCAACAGATAAACAGGACAGA
ATCAAACTGGGGGCTGCTTGTTGTCTCCTACATCTTCTTCCTCCTCGCGCTCTTTCCTCATTGGGCATGGTCATCCAGTCAGC
ATCAGATTCAAGAACCTGCTTTGATGCTAGTTTCATTCATCAGGAAGCCATGCAGCAGCAACCAGTGCATCATCCCTCATGCG
AGAAGTACAGCAATGGATTTCTCAAACCTTTTCTAGGCCTCGCGCTTGCGCTTTTGCTACTCGCCTTTCTCCCACCTACTACA
AGCTCGTGT
>SsLRR-RLK235
AACCTTGAAATGCATTTTTCACGAACCATATCGCACGCTAGTAGTAGTAGCCCATACGTGTGTGCACCAACCAATGCATCCAT
GCAACAAGCACGGCATCGATCCCACCGCAGCAACAACCAGCAGCATACGGCGAAGCACGCTTTATTCGTTCTGCAATGTAA
GTTCATAGCCTCTCAGTTCACACTGCTGCTCCGGTTTACGTCGCCTGGTGGTTCGTCAGTCGCCATCGTCATGCTACGCAGGA
TCCCACAGCAGGCCGACCGCTGAGTACATGTCGGAAAGCAATGGCTGTAGTGCTGAGGAGGTGAGCTCTGGTTCAGCGCCA
GCAAGCAAGCAGAGAGATTAGAAGAAAGATTTAGGGGAGGAAGATGACGTGGCAAAACCTGGCCATTGTATAACCGTGTCC
TGTGGCGGAGCCAGAAGCTTTTAGAAATCCAAGTAATATTTATTATAAGAAAATATGTGTACTTAGCAAATTACAATGTTCTTC
TTTAATATAAAGAATATTTTCTTTTAAATTTCATCGACAAGCTCGGCCGAGCTGCGCCTTCGCTATTGGCTATGCCACGTCATC
AAAACCACCTAGACCTAATGCCACACACCTAAACCGGTTTTGGTTTAGAGCAAAACGTGAAAAACAATCTATTGGAAAATG
ACACAGTTAAGTAGACGATTTCATAAGGTTTTTCTTGATAAGAGACGATTTCATAGTTGAGTGTTACTTGAACCGAGGGCGA
ACTTGAAGTTGAAGATGGTAATTTCGACCTATTCCAATAGCATACCACCACCCAATAAATATATCCAGTAACAACTGAATGGT
GTGGCTGCCAAAGAAACAAACACAAGAGATTAGGCCAGTCTCAATGGGTAATTTCATTATGTTGTTTCCAATATTGCCATGTC
ATTTAAAATGAAATGAAACTAGGATGAAACACCCACTCTCAATGGAGAGTTTCATTCCATGGTTTCATAGTCATTTAATTCCA
AGACTCATAGAGAGTCGGTAATCGTGTCAAGAGAGTTTCATCTAGATGAAACTCATTTCTTCTCTCTCTTCTTAAATACACTG
CCATGTCATCAAAAATGCCTACGTAGTAACCTATTTAATGCAAATGAAACTCACGTGAAACTCCCACTGAGACTGGCCTTATA
TCAGCCTCTCAAGTGGACAGGGTTGCTTCAATTTTCGCCATCTTGATTATATATAAAATATAATTGATAGATATATAGAAACGGT
ACACTGCAAAATGTCAGCCGACACTTGAGGTCAACTAAAGCAGAAGAAACAAGGGCAAAATAATTGAACCGGTTCGTGCA
CACGTGAGCACCACATTTTGGTCAATGAAACAGTTGATGAAGATGAAATGAGAACTAGAAAAGTCTATCATGCTGCTTTGGG
GACTCAAATTGGCCACAACAGCACTGAAGAGTAGCTGCATTAAGTAAAGACTGAGATAATGAATGAAAATAATAACACTAGT
GTATATATTAAAAATTATTAATATGAGATCGAGTCACCATCTAAGGGCCTGTTTGGTTTCCAGGAGTTAAAGTTTAACTCCCAT
CACATCAATGTTCCCTCAAATGCATGGAGTACTAAATGTAGACTAATTACATGCATGTCGGCATAGATTAGGAGTAATTTACGA
GACGAATCTATTAAGCCTAATTAGTCCCAGATTGAAAACTTTTTGTCAAATAAGACAAAAGTGCTACAGTGTTTGTTAAACTT
TAACTCCTCCAACCAAACACCCCCTAAAGTTATGCATATCCTGCAACTTTGACTCAATAATCACATAATCAATGGGTGGGTGG
GTACCAAAGCAGCCTTTTGTTTGCGCAGAAGGTTTGGACTTTTTCATAGATTATCATATAGTATATACCAGGCTGGCTCATCCA
AAACGTGAAGGTCTGACCGTGTGAATAAGCCGGGAAGCTGCTACCAGCTATGGCAATTGTGCCGGAAACGGGTGTGGACGT
GTGCCCACATCAGCCAT
>SsLRR-RLK263
ACAGTGGGTGACTCATAAACTAGCTCTAAATATTTTTTTATTTTAATAGAAAGGAGAAAAGCTAGTTTTTATCAAGAGTTAGG
CTCATACACACAGTCCAAGAAATATAAGAGTGTCATGTGGGCCTAATCATATTATGAGCCATTGCTAAAAACTAGAGATTGGC
TTAGAGCTAGTAACTAACTCTTACTGTTACAGGCGCCCTAAGTGGTGTTTAAGGATCTGTTTGGTTCCCTGCAACTAATAGTT
AGCTGCTAACTATTATCTGAAGATCTTTTTAGATCCTCAATTAATAAGCTCAACTAGTATTTAATCTATCAGCCAGCTAGCAATT
GGCTATTTGGCTATTAGGTGCAGATCTATTTACATCCTCAACTAATAAACTCAACTAATGTCTTACCTATCATCCAACAAGCAAT
TAGCTGTTACCTGAGGATCTGTTGAAATCCTTAGCTAAAAAACTCAACTCAACTTATGATTTACCTATTATCTAGCTAGCAACT
GATTTTTTTCTAGCTAACACAACTAATAGTTAGCTGGTGGACAAAGAGGGCCTTAAATGACTATTGTGTATCATAAATTCATAA
TCGAGAGTTTTTAGGTACTCTCTCTCCATCTGGAAAAAAAACCAATGGAACTACTACCGAATGAAATTATTTTTATTTTTTTAT
CAAAATTATATAAAACCCAATATTTATATAGTACTATGGGTGCGTTTGGTTGGCGACCAAAGGATGTCAAGCCGTGCCACACC
TTCCGTGCCACAGTTGCGGCAAGCGTTTGCTTCGTGGCCCTAGTTCCGGCATGCCGCAGAAGTGCCGTGGTCATATAGTTTAT
TTTGAAAGCCAAATCGTGCCGTAGTTGCGCACTCCGTTTTCTGCGCCGCACCTTCGGCATGCCAACAACTCGGCGTGGCATC
TGTTGGACATCGTCCAAACACGCCCTATATATATAGGTATCATTAGATTCATCAAGAACATATTTTTATAGTATATCTGTTTTGTT
TATTATAAATTTTTAATCTATTGTATAAATTTGTTTAATTTTTAAGTATTTCTACTAGATAGGATCATAGGATTATATCTTTTTCATT
TACTAACACAATCTTGAAGAACGCTCTGGATATAATCGCTGATGAAAAACTGCGGTGTAGTACTCGCACGGCTGTCGCCGTC
GCCCCGCTTGACTCCACCTCTGTGGCTCTGTGGCGTCACTCTCCCAAGTCCAAACTCCAAACAACCGCCCGCCCACCACTG
GGGCAACCAGGAGCATTTATTATTTTCAGCACACTCCGTTTGATCCGTCTGCCCACCCATTTCGTCCACTGGTTTTATTAAACG
CCTGCCGCTTATTAATTGCAGCCGAATCAAACGCACAACTAGCACTGGTGCGCCTACCATGTCCGTCGATGTCTGTCTCGATG
AGAGAGAGAGCACGAGAAAGTATGAGAGAGAGAGGTGTGTGTGTGTGGAGAGGAACAGAAATTGGATCTCTCTGAATAAA
GAAAACGCAAGCGAACAGAGGGAAAGATAAACAAGTCCGATCCGCACTGCTGAGCGCATCGGAGTCAAAACCAACCGTCC
CTGTCTGTCCTCTGACTTTCCCCACCACTGCTTCCCTGTGATGTCGTCGTCTTCTTTCGGCGCCTCTCAATCCAGCGCGGACC
AAGCAGAGCAGGGATGACGAGGTCGACGAGATCCTCCTCCATGCAACGCTGCTTGCGTTTCTTTCTCCTCCTCTGAGCCTGC
CGTGCCGCCTGTCCGTCTGGATTTCGCTCGCTTTTAAAGGCGGAGCCTTTCCTCTTCTTGGGAGCTTCTGCGACGACTGCTG
CTCTGCTCTCGTCGTCTCCTGTTCTTGGTGCTCGGTCGATGCATGCAGCGTAGCAAGGTGGTGTGAGTGAGCTCCGGCGAGA
ATTGCCTGCTTCCCTTGCGGCGCTCGCGGTCGTTCATGCCCTGCAGCAAAAGAGCCTCCTTCCTGGATCCCGCCGCCGCCTG
TCGCCG

>SsLRR-RLK157
TATCACTACCTCTTTATTAGTTTGAGCGGACGCTATGAAATATAATGGCTCTCAACCAAGGCTAACCATCGAATTGACATGGTG
TTGACTTTTTTTTCTCCAGGATATGTATTGATATACGTTGCCGCTTTGATTGTTGGTCTTCTTCTTCTGCCACTACTTGAATGGA
GAAGGCTATCAAATTAAAGGTATTGACAATAGTTTCAGTTTCAGTTGTCGTCCTGTCTTTCGAATCTGTATATATAGTTAGGGC
ACTGCAAGCATACAGTTCTCGGAACTACAAAAGTTTGGACACTTTGTATATGTTGTGGGTTCCTTTTAAGGCACTCCCCCTTA
TCTACTTGAGAAACAAAATTTAGCCCTAAATTTATATAGGAGCTAATGTAATTAAAATGATTAATAATATGTTAACTGTCTAATT
AACCTGCTATTGCTGGTGCCACGGGATTTTATTTTAAGGTCATCATCAACAAAAGGAAACTCTTCCAGCATTGATAGATTTTG
TCCTTCAATTCCTTTGGCTTCCTTCCTTACCCCAAATCACGAGATAGCAATGCAATATAATCTCCCCTTCCTTGCTCGTAATGAT
GTTGCAATGTTGCATGCATAAGCAGCAGGTCATCAGGCCTAGAGTACTTTTTCATACGGATACTCTGAAACTAGACGGATACT
TCCAGCAGCGCACTTTGATTAATTTGCCTTCTTGAAGCAGTTGATAAGTTTCTTTGCTATCTCTCAGGATACACATTATTATTAT
TATTATCTCCTCTCTGAGTTTTACGTAAATAAAAACAGCAAGCAGTTAGTTCACGTTCCGGAGCATTTCAGTCATTACAATGG
CAGCAAACATGTGCTGGTACAAAACGATCCACAAGTACATAAGCTTTGTTTGGCAAGGTTACAGCTCTAAGCTCCACAAAGT
AGTTTTAAGCCTGTTTTAGTCACACAAGATGGTTCATCCAAACACCATCATGTGCCTCAGCTCTATGCTCTGCCAAACATGCT
GTACTCAGCTTTATGATCTGCCAAACATGCTGTACTAAACTACTAAAGTACAGGAATCAAAACCAGTAACTGATGTCTGAATA
ACTTGTGGTCCATTTTTTTATGACCTACCTCTGATCACATTTTTTTATATCAGCTGTCACAATGTCACTCCTCTGTGAAACTGTA
TTCTTTAATTATTTTCTGAAATGTCATCAATTAGCACTTTTTAGCTCTATGTCATTGCCATCCTGTGTGTTAGATCAGTAAACAA
TATGTGTTCTAAAATAATTTGTCATGTCCAGATGTTGTATGGTGCATGTCACATTCACACCAATTGAATTAGTAGTATCTCATCT
CTTGCTATAAATGTTTGTAATGTTGGATACGAATGAAAAGATTAGAAAGAACGTTGCATAAACGCTAGCACACAAATGCTTTA
TATCAGGTACATATGCACAATATTAGTTTATGCGCGCACCTGCTTGGTGCATGGTATAATTAGGTAACAGATCATGGTTATATTA
GGCAACAAATAAATATTTTATTTCATGCATGCTATCTTTCCAAAAGCATGATTAGTTTGGAGCCTACTAGTTTTGGAGCACCTT
AAAATTTTCCATGTGTACTTCAGGGTTTAGGCATTAAACAAAAGTATGATTGATGATCTCCTAGTCATTTTAATTTTGTCTTTGT
GCTCTTTCTTTTTTTAAATAACCAACCAAAAAAAAATCTCAATGTGCTGTTGGAGGCAGAGCCAAGTTTTTAAAGATCTGTTCC
AGGACATAAACTATCTATTTGGTTTCAGGATATGGCTTGGTCCAAAACATTTGAAACCAGATTGCCACTTTAATTGACTTCTTT
TGTAAAGCCGAGGAAAAACTATGGAACATCTTTTCTCCTGCAGCTCATGTTGATCCAGTGTGGTCCTTTTGTAGCTGCTAACT
TGACTTTTTTCTTTGTGCAGATTACAATTTGAACTGCTAGCAATGGGACCCAATTGTTTCTTCATCTTGA
>SsLRR-RLK170
ATGTTGGCCGTTGTTCGTTGGGCATTTGTTCTCAGGCCATTTGCCGAGTGTTTGTCTGTGCGGTCATCTATACTGCAAAGCATT
TAAAAGGTTAGGTTTTGAGTTGTGCTATTCACAGTGTGGGTACTATTTTGTAATCCAGAGTGCTTGTAATGTGTTGATGTACCC
AGCTATCATATAATATAATTCTTCTTTCGTCGCAAAAAAAAAAAATTTATATAGGAGCTAATGTAATTAAAATGATTAATAATATGT
TAACTGTCTAATTAACCTGCTATTGCTGGTGCCACGGGATTTTATTTTAAGGTCATCATCAACAAAAGGAAACTCTTCCAGCT
TTGATAGATTTTGTCCTTCAATTCCTCTGGCTTCCTTCCTTACCCCAAATCACGAGATAGCAATGCAATATAATCTCCCCTTCCT
TGCTCGTAATGATGTTGCAATGTTGCATGCATAAGCAGCAGGTCATCAGGCCTAGAGTACTTTTTCATACGGATACTCTGAAA
CTAGACGGATACTTCCAGCAGCGCACTTTGATTAATTTGCCTTCTTGAAGCAGTTGATAAGTTTCTTTGCTATCTCTCAGGATA
CACATTATTATTATTATTATTATTATTATTATTATTATTATTATTATTATTATTATTATTATTATTATTATTATTATTATTATTATTATTA
TTATTATTATTATTATTATTATTATTATTATTATTATTATTATTATTATTATTATTATTATTATTATTATTATTATTATTATTATTATTAT
TATTATTATTATCTCCTCTCTGAGTTTTACGTAAATAAAAACAGCAAGCAGTTAGTTCACGTTCCGGAGCATTTCAGTCATTAC
AATGGCAGCAAACATGTGCTGGTACAAAACGATCCACAAGTACATAAGCTTTGTTTGGCAAGGTTACAGCTCTAAGCTCCAC
AAAGTAGTTTTAAGCCTGTTTTAGTCACACAAGATGGTTCATCCAAACACCATCATGTGCCTCAGCTCTATGCTCTGCCAAAC
ATGCTGTACTCAGCTTTATGATCTGCCAAACATGCTGTACTAAACTACTAAAGTACAGGAATCAAAACCAGTAACTGATGTCT
GAATAACTTGTGGTCCATTTTTTTATGACCTACCTCTGATCACATTTTTTTATATCAGCTGTCACAATGTCACTCCTCTGTGAA
ACTGTATTCTTTAATTATTTTCTGAAATGTCATCCAATTAGCACTTTTTAGCTCTATGTCATTGCCATCCTGTGTGTTAGATCAGT
AAACAATATGTGTTCTAAAATAATTTGTCATGTCCAGATGTTGTATGGTGCATGTCACATTCACACCAATTGAATTAGTAGTAT
CTCATCTCTTGCTATAAATGTTTGTAATGTTGGATACGAATGAAAAGATTAGAAAGAACGTTGCATAAACGCTAGCACACAAA
TGCTTTATATCAGGTACATATGCACAATATTAGTTTATGCGCGCACCTGCTTGGTGCATGGTATAATTAGGTAACAGATCATGGT
TATATTAGGCAACAAATAAATATTTTATTTCATGCATGCTATCTTTCCAAAAGCATGATTAGTTTGGAGCCTACTAGTTTTGGAG
CACCTTAAAATTTTCCATGTGTACTTCAGGGTTTAGGCATTAAACAAAAGTATGATTGATGATCTCCTAGTCATTTTAATTTTGT
CTTTGTGCTCTTTCTTTTTTTAAATAACCAACCAAAAAAAATCTCAATGTGCTGTTGGAGGCAGAGCCAAGTTTTTAAAGATCT
GTTCCAGGACATAAACTATCTATTTGGTTTCAGGATATGGCTTGGTCCAAAACATTTGAAACCAGATTGCCACTTTAATTGAC
TTCTTTTGTAAAGCCGAGGAAAAACTATGGAACATCTTTTCTCCTGCAGCTCATGTTGATCCAGTGTGGTCCTTTTGTAGCTG
CTAACTTGACTTTTTTCTTTGTGCAGATTACAATTTGAACTGCTAGCAA
>SsLRR-RLK184
CAATATTTGCTATCACTACCTCTTTATTAGTTTGAGCGGACGCTATGAAATATAATGGCTCTCAACCAAGGCTAACCATCGAAT
TGACATGGTGTTGACTTTTTTTTTCTCCAGGATATGTATTGATATACGTTGCCGCTTTGATTGTTGGTCTTCTTCTTCTGCCACT
ACTTGAATGGAGAAGGCTATCAAATTAAAGGTATTGACAATAGTTTCAGTTTCAGTTGTCGTCCTGTCTTTCGAATCTGTATAT
ATAGTTAGGGCACTGCAAGCATACAGTTCTCGGAACTACAAAAGTTTGGACACTTTGTATATGTTGTGGGTTCCTTTTAAGGC
ACTCCCCCTTATCTACTTGAGAAACAAAATTTAGCCCTAAATTTATATAGGAGCTAATGTAATTAAAATGATTAATAATATGTTA
ACTGTCTAATTAACCTGCTAATGCTGGTGCCACGGGATTTTATTTTCCACGGAATTTTATTTTAAGGTCATCATCAACAAAAGG
AAACTCTTCCAGCTTTGATAGATTTTGTCCTTCAATTCCTCTGGCTTCCTTCCTTACCCCAAATCACGAGATAGCAATGCAATA
TAATCTCCCCTTCCTTGCTCGTAATGATGTTACAATGTTGCATGCATAAGCAGCAGGTCATCAGGCCTAGAGTACTTTTTCATA
CGGATACTCTGAAACTAGATGGATACTTCCAGCAGCGCACTTTGATTAATTTGCCTTCTTGAAGCAGTTGATAAGTTTCTTTG
CTATCTCTCAGGATACACATTATTATTATTATTATCTCCTCTCTGAGTTTTACGTAAATAAAAACAGCAAGCAGTTAGTTCACGT
TCCGGAGCATTTCAGTCATTACAATGGCAGCAAACATGTGCTGGTACAAAACGATCCACAAGTACATAAGCTTTGTTTGGCA
AGGTTACAGCTCTAAGCTCCACAAAGTAGTTTTAAGCCTGTTTTAGTCACACAAGATGGTTCATCCAAACACCATCATGTGC
CTCAGCTCTATGCTCTGCCAAACATGCTGTACTCAGCTTTATGATCTGCCAAACATGCTGTACTAAACTACTAAAGTACAGGA
ATCAAAACCAGTAACTGATGTCTGAATAACTTGTGGTCCATTTTTTTATGACCTACCTCTGATCACATTTTTTTATATCAGCTGT
CACAATGTCACTCCTCTGTGAAACTGTATTCTTTAATTATTTTCTGAAATGTCATCCAATTAGCACTTTTTAGCTCTATGTCATT
GCCATCCTGTGTGTTAGATCAGTAAACAATATGTGTTCTAAAATAATTTGTCATGTCCAGATGTTGTATGGTGCATGTCACATT
CACACCAATTGAATTAGTAGTATCTCATCTCTTGCTATAAATGTTTGTAATGTTGGATACGAATGAAAAGATTAGAAAGAACGT
TGCATAAACGCTAGCACACAAATGCTTTATATCAGGTACATATGCACAATATTAGTTTATGCGCGCACCTGCTTGGTGCATGGT
ATAATTAGGTAACAGATCATGGTTATATTAGGCAACAAATAAATATTTTATTTCATGCATGCTATCTTTCCAAAAGCATGATTAG
TTTGGAGCCTACTAGTTTTGGAGCACCTTAAAATTTTCCATGTGTACTTCAGGGTTTAGGCATTAAACAAAAGTATGATTGAT
GATCTCCTAGTCATTTTAATTTTGTCTTTGTGCTCTTTCTTTTTTTAAATAACCAACCAAAAAAAATCTCAATGTGCTGTTGGAG
GCAGAGCCAAGTTTTTAAAGATCTGTTCCAGGACATAAACTATCTATTTGGTTTCAGGATATGGCTTGGTCCAAAACATTTGA
AACCAGATTGCCACTTTAATTGACTTCTTTTGTAAAGCCGAGGAAAAACTATGGAACATCTTTTCTCCTGCAGCTCATGTTGA
TCCAGTGTGGTCCTTTTGTAGCTGCTAACTTGACTTTTTTCTTTGTGCAGATTACAATTTGAACTGCTAGCA
>SsLRR-RLK250
TCCTCGGTCAAGCAAACTTTTCCTTTCCTTTTTTATTTCCTTGGTGAAGTTGCAGAGTAGAATGGTTGTCCGTTATTCTACTGA
TTTAGCATGTGTATACCAGCCCATCAAATCCTTTCTCAAACCACGAATCATATAGACCCTGCTTCGCACGAGGCGTGGCAAAT
CCTGCTATATGGGTTGAGGCACTTACAAATAAGAGCTTTTCTGAGAAAAAAACAGTCAGCAATTCACAAACTAGAAATATAT
AATGACATGTTATTGCTTCTAAGGTAACTAGAAAGTACTCGTTCCAAGTTCTTTTATGTCATCAGCATCCATGTGTTCTTCATC

TGTTGGAGTCATTGATATTTTTCAGAACTGTGTTACCTGCACAATTATTTCGAGACCACCTGAACATCCACAACTTTTCTTTTA
ACATTTTCTTTGCTTTTGGCAGGATACATTCAGCTTGTTCGTCCTATTTGCCTGCTTCGTCACTGCTTTTGCTGGATCTGATATA
AAGAATCTATATGCTCTGAGGGATGAACTCATCGAATCAAAGCAATTCCTTTGGGACTGGTTCGATACAGAAACTCCTCCATG
CATGTGGTCACATATCACTTGTGTGGACCATGCTGTTACAGCCATTGATTTGTCATATATGCCACTCCATGTTCCTTTCCCATTA
TGCATCACGGCATTCCGGTCACTTGTTCGTCTCAACTTAAGTAGGTGTGATTTATTCGGTGAGATTCCAGAAGCCTTGGGAAA
TTTGACGCATCTCCAGTACCTGGACTTGAGCAGTAACCAGCTTACTGGGATCGTGCCTTACGCATTATATGATTTGAAGATGC
TGAAAGAAATACTGTTAGACAGAAACAGCTTGTCTGGACAATTGATCCCTGCCATTGCTAAGCTTCAGCGACTCGCTAAGTT
AACCATATCCAAGAACAACATCTCTGGAGAACTTCCTCCAGAGATGGGCAGTCTGAAGGACCTAGAGGTCCTGGACTTTCA
CCAGAACTGCTTCAATGGATCAATACCAGAAGCATTAGGCAATCTGTCTCAGCTCTTCTACCTTGATGCAAGCAATAATCAGC
TCACTGGATCAATATTTCCAGGAATGTGCACATTGTTGAACCTGCTGACGCTTGATTTCTCTTCAAATGATTTGGCGGGGCCA
ATACCTAAGGAGATTACTCGCCTGGAAAATCTAGAGCGCTTGGTTCTGCGGTCTAACAATTTCACTGGAAGCATTCCAAAGG
AGATTGGTAACCTGAAGAAGCTAAGAAAGCTCATTCTCTCTAAATGCAACTTATCAGGCACCATCCCTTGGTCAATTGGTGG
TCTTAAAAGCCTACAGGAACTTGATATATCAAATAACAACTTCAATTCGGAACTCCCAGCATCTATTGGTGAGCTGGGAAATC
TGACTGTCCTGATTGCAATGAGAGCAAAGCTCATAGGCAGCATACCAAAAGAACTTGGTAACTGCAAGAAGCTTACTGTCCT
GCATCTGTCCTTCAATGCCTTCACTGGTTGTATCCCCAAAGAACTTGCAGATTTAGAAGCTATTGTCCAGTTTGAGGTGGATG
GTAACAAACTGTCAGGTCATATTCCTGATTGGATTCAAAACTGGGGAAATGTTGTATCTATAAGACTGGGAGGCAACAAGTT
CAATGGTTCTATACCTCCTGGCATATGTCAAGCCAACTCTCTTCAGTCACTTGACTTGCATTCTAACAATCTGACAGGGAGTA
TCAAGGAGACATTTATAAGGTGCAGGAACCTTACTCAACTGAATTTGCAAGGCAACCATTTCCATGGAGAGATACCTGAATA
CTTGGCGAGTTACCGCTTATAATTCTGCAATTGCGCTACAACAATTTACAGGAGTCTTGCCTGCCAAGTTGTTTAATTCATCA
ACAATTTTGGAGATAGATCTCAGCTATAATAAGCTCACTGGCTGTATTCCTGAAAGCATTGGTGAGCTCTGCGGCTTGCAGAG
GTTGCGGAT
>SsLRR-RLK204
ATTTGCATATGATAAGATGCTTACGACGAATGTTAGGTAAAGTAGTAAGTTATTTAAGCACCAATTGCATTGGAAGGATTAACG
TTTTTTACAAGCGTTTAATGGTGTAGTAGCATTGTCTCCCTAATTAAGCACAATATATCCAAGTATCTTGTATTGTACGTGTCCT
ATAGAATACTTTCAATAATATGCATGTAGTATATCTTTGTAGATATGGCTAGCTATTTGCATAATACACTAGAGATTATCGCTTAT
GTTTAGGATTCAGGGAAGTGCTTGTAGTGGTACGTCATTAAGAGAACATCTCCCATGTTATTGTGACTCTTACTAGGGGCGGA
GCTAGAGAAAAAAAAAAGTGGATGCAACACTATATAACTATAGCTGCTAGTTGAAATATATATTGGAAAAACGAGAGCGATAG
TTTTTTTTGAGGGAAAACCGTGGAGCATTGCTCCCCAGTGAAAATATTAAAACAAACAAGGAGTACAACACAGGAAAACAA
AGGCATTAGCTGGCTGCTAACAAAAAAAAGAAACCTAAGGCTCACCTATTACAAAGAAACTGAGACTAAGCACAGGAGCC
ACCAGGGCAAAGTGAAAAAAAAGGATTACAAACTATTGATCCAATTCATCACTAAACCACGCTCGGTAATAGGCAATCTGAA
GAGATGAAGTTTAACTTCATTCTTAAAGAGCTGCTTCCAGTTTTCCAGAGACGGTGGCTTGTAATTGAAGATGAAATCATTCC
TCTCCTTCCAAATACACCATGCACCAATCATGAAGAGATCCATGAAGTAAGGGCCACCAAAGTTGGCATGCTGATGCTGTAG
CATTTCAAAAATCGACACCTGCTGACTTTGCTGATCCCACTGTAAGCCAGTGGCGAACCACCTTGCAACACTTGAAGAGCA
CTCAAAGAAAAGATGCAAGGAGGTTTCATCTATCTGTTCATGACATAGAACACAATGGTATCCTTCCTCAAGATGTTTATGTC
TTCTTCTGAGAATATCTCTGGTGTTGAGCCTGTCAACCAGCAACAGCCAAGCAAAGAACTTTATTTTTGGCATGCATTTTGCT
TTCCAAATCCAGCACAGAGGAGTAGGTGGCACAATTTGCATGTAGTGGTGCTTGTAGTATCGACTAGAACTATAGAGACCTC
CATTCCAATTACATACCCAAACATCTGTTTGGTCATTATCAATTCTGAGGGACTCTAACTCTTCTCTGAATACCAGATACTCATT
ATTGATTTACCAACCACAACAATGCTTCCAAAACACACAACAATGACAACAAAAGTATGGATGTATATACCTTCATGTAAAAA
TTTGGGCTTCCAAAAATACACAACAAACACAACAAAAGTATGGATAGGTAACCTTCTTGTAAATTTTGAGTTGCAAAACCAT
TCTACTCTGCAACGGTTACTGTATGGGTTGAGGCACTTACAAATATAGACCCTGCTGTCCAAAACCGCTCCAGTGTTACTGTA
GCAAAACCGCTGTCCAAAAACCGCTCCAGGGTGTAGTTTGAACGGTTTTGAAAAGTTCAGGGTGTAAAACAGACGGTTTTA
TAGTTTGGGGTGTAGGTTAGACACTAGTTGATAGTTCAGGGAGTTAAGTAGACTTTTTCCTCTACTAATTTAGCATTTGTATAC
CAGCCCATCAAAGATCCTTTCTCAAACCACGAATCATATAGACCCTGCTTCGCACGAGGCGTCGCAAATCCTGCTATATGGGT
TGAGGCACTTACAAATAAGAGCTTTTCTGAGAAAATACAGTCAGCAATTCACAAACTAGAAATATATAATGACATGTTATTGC
TTCTAAGGTAACTAGAAAGTACTCGTTCCAAGTTCTTTTATGTCATCAGCATCCATGTGTTCTTCATCTGTTGGAGTCATTGAT
ATTTTTCAGAACTGTGTTACCTGCACAATTATTTCGAGACCACCTGAACATCCACAACTTTTCTTTTAACATTTTCTTTGCTTT
TGGCAGGATAC
>SsLRR-RLK238
TCCTACAAATAAACACAAAGAGGTTCAGTTTGGACAGTATAGCAGGTTGCTGGGTTCATACAAGAAAGAAAAGGCTTACTG
TTATGCACCAGTTTTTTAAGAAACAACAAAACAGTAACATTATATATTCCAGCTCTAGTATCGTAGTGTAAAGAAAAGAAAGA
AAGGTTTCAATGCACCATAGTATTAAATTAGACGGAAAACAAGCAATTATTCGGCATAGTATAGAAAATTAGATGACAGGAGC
GAATGGAATCCCCACTGAATCCTAATCAAAGATGACCAGAACACAAACGCTCTAGCAGCATGAATGTATCTTCTCCAATGAC
ACTCTCAATGTCAATGGCTGTGTCAAGGGTGCACATAACTGAGAAGAGACGGCTGGGCATAAGGCTTGTTACCCATTGCCTG
TATTGTCAGTATGTCCCGCTCAGGCATTCTACAGCATCATCCTCAGCCACTCCCTGAAAACAATACATTCAACTTAGATTTTAG
CTTGCAGGAAGGAAAAAATGGAGAACCAGACCTGTCACATTAAAACTAAGAAGATAGTTCAGAATCTAACAATTTAAATTTA
AATCAATATAAACAATTCAGATTGGTGGCTGCACTATAGCCGAGCAAAGACAAACTCAACACGGCCCCATTGTGTAGCTGAT
CAAATTCATCTTATGGGAATTTTTAGAAGATCCCTCAAAAGTTCCTCAGAAATGCCATTGTGAAAATCTTTTGTATACACTCAA
GGCCATGCATAGCTATAAATATTTTAGAATGATAAATGACAGCAATGACACCAGCGCATGCAGGTCTGAATAAACAGGGCACC
ACGTAACAACACCAGCAAGGCTCACAGGTTATATCCAACCAAAAGTCTACAGCTATGACACCAGCTAAGCAATCTACATGCA
GCTCTGACTACACAAATTTAGAAATTGTCTTTCAAACAGTTTTGACACAAAGGGGTTGCAACTGGACAGAATAAACATTTTG
ACACATTGCCCATAAGCCATATATTGTAGCAGTATGTCTTTGGACAACATAACAGATAGCAAAAATATACATACCTGTGAAGCA
GCACTTAGATCAAAACTCTTCTCCACACTCCATGAGGCTGTCCAACCTTTCATATGAATGTTTTCATCCCGTAAAAGTTTAATT
ATGCATTTAAAAGGAGAAAGCAGTAAATATAGATAGGTATGGAGCAACAAAGTCAAATGCACTCTTCAGTATACTCTCACATG
ACGACACCAAGCATAACATCTAATCTCAGTATAGCATCACACATGTCCAATGGAAACGCATTTGAGGGTTGACCACAATCAA
ACCATCACAGCTGAAGGCATTTATTTGGTGACCAATGTGGTAGAGAAAACGTTCCAGGAAATTAAAAATTCACTGATTCATAT
CCTCACAGCAGATGAAGCATTTATTTAGCAACAGATCCTGGGACAGTTGGTACGGTCAAAAAGCAAGCTCCTAATTGAGATT
TCTGATCTCGCCACTTTGCCAGATATAGACAAAAAAGAAATTACAGTTGCAACTAAACAATCGTGGATGTAGATAGAACAGC
TAAATATCAACAGAGATTTTTTCCCTTTTGCCTCTGTTATGATCTTGACAAACCTCTATTTGGTATGTTTTAACTCTGAATTAGT
ACAGATATGGGCACAAACAAAAGAAAACAGCCATGACTATGTTACATTACTGACAATTTGTTAGACTTAATTTGTTCAATTCT
TCAAGGCCTCCTGTACATGCTATACGATCCAAGAGTTAATTCCTCTGCCCAACATCTACAGAGCACAGCACAAATCGAAACA
GAAGTGAGGCAAGGAGAAAGAGCGGGGCACTGACCGTGAGCATGAGGACGCATGGCTTGGTGAGCCTGCCGAGCTTATTG
AAGATGCCGCGGCCCTTGGCGATACGGATAGAGAAGGCCACTTCGTCACTACGCGCGCCGGACGCCGTGTTCGAGCCTGTC
CGGGCCGCCAATTTGAGGA
>SsLRR-RLK264
CGGACGTCGCAGACGCGGATGGTTGGCATGTGGCAATTCATTGCTGTATTGATAATTGATATACGGGGTAGTATGTGACAAGC
CTGGACGATTCAGGGTGCGCTGTGCACGGCTTCTGAGTTAAAGCATCACAACAGGTACGATTTCAGTCAATTTTCTGTGTTT
ACTCCCGTTCTTGCCTTCGCACGATTCAAGCTTTGACATCTCTTCTCTATCTATAACAGGATACAAGAGGAAACCAGCATCAA
AAGAGTGTTAGACCGTCTCCAACGAGGTTGGCAAACAGCGATGCAAACGCAAAATGCGTTCTGCACAGGATTTTAGCCTTC
AGAATCGTGCTCCAACAGAAGACGTATACAGGCCACCCATTTTGCATCGCGCGTGAACCAGAACGCAAAAACGCGTCCCCT
CTCTCCTGCGACGCAAATCTGGGTGAAGCCGGCGTCGGGGCCCTCTTCCCTCTCCCTCTCCCTTCTCTTCTCCCACCCCACTC

CCACTCCCACTCCCACTAGCCATCCGCTCGGGCGAGCGGCGCCGGTTCCCACCTCTGCCTGGAGGTCGGAGCAGAGGGAGG
AGAGGAGGAGCGACTCGAGGAGGCCGGCCGGAGCGGAGGGAGGAGCTCGGAGCGGCCCGCGGACGACGACGGCCGGCG
CTTCTAGGCGGCTCGACAGGGAGGTGGGGTTGGAGGACGAGCTCACTTGCACGGTGGCGGCGGGTCCAGATCTCGCCGGG
CCCAGACCCCGCTCCTCTTCTTCCTCAACGCCGGCAGCCACTCCCTTCTTCCTCAAGCTCCTCCTCGGACGACGGCAAGGCG
AGCCCGCGGCAGGGCGCGGTCGGCGTGGCCAGGCGGGACCGCGGCGGGGAGTCGCCGGCGCGGCGAGGAGCGGGCGGCG
CTTGGCTGGCCGGACTTTTGCGTTGGCCGTTGGAAATGTGAAATTTTGGGTGCGCGGCCTTTTCCATGTGCGCGACCTATACC
GACGAATGCGTCTCGTATATGGGTTCGTGCTGTTGGAGTCAGTCTTAGTGCTTTTTTGAATTTAAAACTGCTGTGCTGTTAAAT
CGCCCGCCATTTCACCCTTTGTTGAGGTGAAAGTGAAACCAAAAGGTTCAGTAAAGGCCGGTGAATCCAGTCTCCGGCCAG
CGGTTAGAGCCGTCCATCAAACCATCACCAACATTGACCAGATTCTGGTTCAAAAGAAGTCCCGGACAATGATTTATAGGCA
CTGTTCCAACATCATCCTGTCATGCACTAGATGTAGGTGACGACGGATTACAACAATGGACGGCAAGGCAACATTGGGCAGG
CAGGCATGGAGCAGTGCCTGTGTCCAGGGAGCACAGCCTACGTGGCACGTCGAGCCCAGTCAACATCTGCTGCACCTCCTA
AACGCCAGCTAAACTAAACTAATCCCTCCCTTTAGCCATCCGTGTAGCCTCTCCAGCTCCAGGATGATGCGGTGCAGACACA
GCGGCCCCAGCTCACGGCGTCGCACACAACGCGCGTCCAGCTTTTCGCCGGGCCCCGCTCGTCGGTGTCGCGGCGCCGGGC
ACGCCCCCGTCAGCGTTCGTCCCCAGTAAAAGGTCTTTACGGTGACAGCCTGTCCCGCGCACTAAAAGCGAGTTTAACGTT
AACCTAGCACTAGCACAGTCTCTCTCTCCTCTCTCAAATGACGTTAAAGCCTGAGGAGAGAGAGGGACGGAGACGGACCGG
GGCAGAGTAAAAAACAAACAAGGCTGCACAGAAAACGCACACCGGAGTGAAAAACGGCCTCGAACGCCACCCAAACCCG
TCCAAAACCTCTCAACCAAAAACCCCTCCTTTTTCTGGCGCGCGCTGTTTCTCTCTCTCCCTTCTCCGGTTCTCCCTTCACTC
GCGCTCTCCCTCCCCTCCCATCTCGTCCCTCCATGAGAGCCTAGGCGCTACAGCCGCCAAAGTTGACAAGACAGTCAGAAA
AAGAGGCGGCTCTGCTCTCTGCCCCCTCCCCTCCTCCCGGGCGCCC
>SsLRR-RLK277
GGCGAGGTGGACGAGGACGAGCAGGGTCGGAGGAGGACGACGCGCGGGCCCCGTGTGGCATATACCGGGACTCCGTACGT
CCGCCATAGCTAGCGGTCGAGAGCCCTTCTGGAATTTTATCGGGGCCTTTACCGTCCCCCGCTGCAGGAGTTTCACACGGTAT
ATGTGTTTGCCGGATGTATTTATAGGTCCCGCTGCGGATAGGGTATGTTCAGATAAAAATAAAAAAGATCCAGTGGGAGGAAC
AGCCGAACAAATTGGTCCGGCCTTGTCCGTTCTGGTGTGCATTTGGGTCTAGTGGTTGCATGAACTCATGAACTCTCCAAAT
CTGATGGAACCAGCATTTTCAGCTCCTGGACTGGACCTGAAGTTGGCAATGCACGGAGGACTGCAGGAGTGTGGTGCTGCC
AGAGGCGGACGTCGCAGACGCAGATGGTTGGCATGTGGCAATTCATTGCTGTATTGATAATTGATATACGGGGTAGTATGTGA
CAAGCCTGGACGATTCAGGGTGCGCTGTGCACGGCTTCTGAGTTAAAGCGTCACAACAGGTACGATTTCAGTCAATTTTCTG
TGTTTACTCCCGTTCTTGCCTTCGCACGATTCAAACTTTGACGGCCCTGTTCGGCAGGCTGTTTTTTGGCTTATAAGCCGGCT
GTGGCTAATTTGCTGTGAGAGAAAAACACTATTCCACGGCTGATAAGCCGTGCTGATAAGCTCAGCCGAACAGGGGGGACA
TCTCTTCTCTATCTATAACAGGATACAAGAGGAAACCAGCATCAAAAGAGTGTTAGTGCTTTTTTGAATTTAAAACTGTTGTG
CTGTTAAATCGCCCGCCATTTCACCCTTTGTTGAGGTGAAAGTGAAACCGAAAGGTTCAGTAAAGGCCGGTGAATCCAGTCT
CCGGCCAGCGGTTAGAGCCGTCCATCAACCATCACCAACATTGACCAGATTCTGGTTCAAAAGAAGTCCCGGACAATGATTA
ACAGGCACTGTTCCAACATCATCCTGTCATGTACTAGATGTAGGGTCTTGTTTAGATGCCTCCCAAATTCCAAATTTTTTCACT
CTCTCTTCGTCACATCAATTTTTGGACGTATACATGGAGTATTAAATGTTGGTAAAAAAATTAACTAATTGCACAGTTTGATAG
TAAATCACGAGATGAATCTTTTAAGCTTAATTAGTTTATGATTGGACACTAATTACTAAATACAAACGAAAGTACTACAGTAGC
TATAGTTGAAAAAATTTAATGTAAACGAGGCCTAGGTGACGACGGACTACAACAATGGACGGCAAGGCAACATTGGGCAGG
CAGGCATGGAGCAGTGCCTGTGTCCAGGGAGCACAGCCTACGTGGCACGGCGAGCCCAGTCAACATCTGCTGCACCTCCTA
AACGCCAGCTAAGCTAAACTAAACTAATCCCTCCCTTTAGCCATCCGTGTAGCCTCTCCAGCTCCAGGATGATGCGGTGCAG
ACACAGCCGCCCCACCTCACGGCGTCGCAGACACCGCGCGTCCAGCTTTTCGCCGGGCCCCGCTCGTCGGTGTCGCGGCGC
CAGGCACGCCTCCGTCAGCGTTCGTCCCCAGTAAAAGGTCTTTACGGTGACAGCCTGTCCTGCGCACTAAAAGCGAGTTTA
ACGTTAACCTAGCACTAGCACAGTCTCTCTCTCCTCTCTCAAATGACGTTAAAGCCTGAGGAGAGAGAGGTACCGAGACAG
GGGAGAAAAAACAAACAAAGCCTGCACAGAAAACGCACACCGGAGTGAAAAACGGCCTCGAAGGCCACCCAAACCCGTC
CAAAACCTCTCAACCAAACCCCTCCTTCCTTTTTCTGGCTCGCTCTCTCCCTTCTCCGCGCTGCAAACGCAAACACGATCCT
CACTCGCGCTCTCCCCCATCTCCATGAGAGCCCTACAGCCGCCAAAGTTGACAAGACAGTGAGAAAAAGGAGCGGCTCTGC
TCTCTGCCCCCTCCCCTCCTCCCGGGCGCCCGCG
>SsLRR-RLK39
TCCGCCTCTAGAGTTGTGGCAGCGCCACAAAGGATGGCGGCAGAATCCATTGCCACAGAGTGTGGCAAGAAATGAGAGTG
ACGGTGGTGTGGCTGTCTAAAAGTACGTGTGAACGAAACACTACATCAGTAAAATGATGGTGTGCTTGGTGGCGTGGCCGA
GTGTGGCTCCAATCCAAACAGCTCCGGCTATCTATGGTTGGAGAACTAGACCTCGTCGTGCTCTGCCTGAACCCCCCTGCTC
TGAAGTTTTGGTGCCACTGCCATGTCCTGCCCTGCACGGTGGTGGCCATTTCTTCATAAGGCAAATGTGGACTGAATTGCAG
CCCGAAATCTGAGCTCGATTACGGAGTTCCGTCACCCGGTCCCGGTAACATCAGTCGATGTGACTTGTGGCCTTCCCCTTCTT
TCCCGCTCCACCACATTGCCGGGAGTTGAGGCAGAGCCGCCGAGTCGCCGCTTTCGATTGCCCTTTTCGTCCCTCTTTTGGG
CCTGTTTAGTTCCCAAAATTTTTCAAGATTTTCCGTCTTTAGACGTATGCATGAAATATTAAATATAGATAAAAAAATAAAACT
AATTACACAGTTTAGACGAAATTTACGAGACGAATCTTTTAAGCCTAATTAGACCATGATTGAACACTAATTGTCAAATAACA
ACGAAAATGCTACAGTACCATTTTCTAAAAAATTTCCAAAAATCTTTTAACACTAATTAGGCCTTTGGACCCATCCGTTCGCT
GGTGCATGGGTATCAGCACCTACCTGGCTATTGGCCGTTGGCGATGCAGCCCATATGGCAGGCAGGTCAAAAACGCACAGTG
AATGGATCAATGAAGCATGGGCTGGTGGGTGCACGCGCCACGCGCGACGCGCGCCCTGTGCACATTCCTCCAGTACATCATG
GCTCGGCGACCATCAGTGGCTGGCAAACAAATCCATCGGTCTTCCCGTTGCGAAACGACGTTTTCTCCCTGCGCCCGGCCGC
TGACGCGCCGGGACCCGACGGCCGTGGGGATCCACGCCCAGTGGCCGCGTGCGGGTGCGGCCGGCAGGTCGAGCCGAGCC
CGAGCGGGGCAGTCTCATTTTGCCGGATCGCTGCCTGTCCTGTCCCTCTCCCGCCGTCCCGCAAATCTCTCGCTCCACGGCT
CCAGCTCTTGTTAATTACTACTCCGTACTAGTACTGCTGCCATTAACTCCCACGCTGCTTTCCATGTACAGGCGCACGTGCTCG
CCTTTCGATCCCCGCGCGGCGGTCGCTGTCCGTCGGGCTCCACCGCCGGGGAGGTAGCCGTTGGGGGCCGGTGGCGACACC
ACGGCACAGGACAGGGTGCAGAACTCCTGCTGCGCTGAATGGGAAAGATCGGATTCTTTCCTGCCGCGCGGGACCCGCGAT
GTCAGCGCCCGCGTCCGTAACGTATAGGACGAGATGGGTTTTAAAAACTCCAAATCGTCTCCCTCTGCAGAAGGACTGAGCT
AGCCGTTAGTTAGCGCATCTGAGTTGAATCGGGTTAGCAGCTAAATTATTCCCTCCTTTTTCCGTTCTTCTGCCATGTTCCTGC
AACGCTTATGGCCTTGTTTAGATTCCAGAATTCATCCCTAAATTCCACTATGCAAAAAGAAGATTCCCCATCATATCAAAATGT
CGGCATATATATAGAGTACTAAATGTAGATGAAATCAAAAACTAATTGCACAGTTTTGTTGTACTTTGCGAGACGAACATTTTG
AGTCTAATTAATCAAAATTTGGACAATTATTACCACTAAAAAACAAAAATGCTACAGTACTCGGAGTGGCACTGTGCAGATTT
GGCCACCCCGAGTCGGGGATCTAAACGAGGCCTATATAGAGCGCGGTACCATCGCTTTCCCCGCTAGCAGAACCAGAAACC
AAGAAAGAGCTCGCTCAAAAGTTCGAGCTCCTCCTAGATATAGGAGCGGTTGCGTTGGGCTAGGTACACTAGATTCTAGCTA
GAGCTACCAGCTAGTTGCACTAGCCA
>SsLRR-RLK23
CTTTTGGGCTAATGTCTCCAAACAGGTGATCTAAAGTTAGCTCTAAATTTTAGCGATAATGCTTCTTATCAGACGTCTGACAC
GACATATGCACCTCCTCCACTCATCCGCGCTCTATTAAAAAAATAAACCGCACTCATCCGCTCGCTCCCTCGCTTCTCTCCTCC
ACGCACTCATCCGCTGGCTTGCTCGCTCACTGCCGCCCCGCCCGTGCCGCGCTCCAGGCCTGCTAGCGCCCCCAGGACGAA
CGCCGCGCCCGCGCCTAGCTCGTTGCCGCCCCGCTCGCCCGCGCTCCCTCCCTCCCTCCCCCTCCGCGACCTCCAATGCTAC
CCCACTCCCCGGGCAACCCAACTCCCGCTCCCCCCACCGGAGATGAGGACGACGGCGGCGGCTCCGACGAGATAGGTCGG
TCCTCCTCTGAATCTGAGCCCTCCTCTTCTGGATTTGAGGGACGCGGCGGTGGCTAAGGTTCCAGCGAGCTCTGGGTCCTAT
TCCCTCCTCCCTCTAGTGATCTTGAAGGTGATGGGCCAGGGACTGGAGCAGCCTACACCTCGAACCCATCATGTTGTTGTTT
GTACGCCCTTGCTATTTGTCCATGTTGCAAGGGGAATTTTGGGATGTTGCAATAAATGATTTCGAATGTTGTAATAGGTGATT

CATGTGTTACAGAAAGAAAGTTCTATTGTTGGGATTATATTTTTTCCTTAATGTTGCAACTTGTGAGTTTTAATTTTTTCTACGT
GTCGTGACAAGCGATTTTAATCGTTGAAAATTACCTTTTCTCAAGGGAGTTCATACAACGTCGACCCCAAGGCCCGAAGCGT
ACGTCGACTCGGTCGGCCGCCGCATTTTTCTTCTTCTTTTGTTTCTTCGCTGGCCCGCCTGTCAGTCGGTCTCCTCCCCCCCT
GGCTGGTTGGCAGGCTCTTTTTACACTGCTCGATCGGCCACCCATTTTTGTCTCTCAGTAGCTTGCCTGATCAGCTATAGGAG
TACGTATCTCCGTTTGCAAAAAAAAAAAAAAAGGAGCACGTATCTCCGTGTGACATGGGCAGACACATGGGCAGTGCATGTC
TATTCGGGCGAGGATCTCGGTGGCAGCCGGCTGCGCCACTNNNNNNNNNNNNNNNNNNNNNNNNNNNNNNNNNNNNNNNN
NNNNNNNNNNNNNNNNNNNNNNNNNNNNNNNNNNNNNNNNNNNNNNNNNNNNNNNNNNNNNNNNCCGGCCGCTGACGCGC
CGGGACCCGACGGCCGTGGGGAGCCACGCCCAGTGGCGCGTGCGGGTGCGGCCGGCAGGTCGAGCCGAGCCCGAGCGGG
GCAGTCTCATTTTGCCGGATCGCTGCCTGTCCTGTCCCCCTCCCGCCGTCCCCCAAATCTCTCGCTCCACGGCTCCAGCTCTG
TTAATTACTACTAGTACTGCTGCCATTAACTCCCGCGCTGCTTTCCTTGTACGGGCGCACGTGCTCGCCTTTCGATCCCGCGC
GGCGGTCGCTGTCCGTCGGGCTCCACCGCCGGGGAGGTAGCCGTTGGGGGCCGGTGGCGACACCACGGCACAGGACAGGG
TGCAGAACTCCTGCGCTGAATGGGAAAGATCGGATTCTTTCCTGCCGCGCGGGACCCGCGATGTCAGCGCCCGCGTCCGTA
ACGTATAGGACGAGCTGGGTTTTAAAAACTCCAAATCGTCTCCCTCTGCAGAAGGACTGAGCTAGCCGTTAGTTAGCGCATC
TGAGTTGAATCGGGTTAGCAGCTAAATTATTCCCTCCTTTTTCCGTTCTTCTGCCATGTTCCTGCAACGCTTATATAGAGCGCG
GTACCATCGCTTTCCCCGCTAGCAGAACCAGAAACCAAGAAAGAGCTCGCTCAAAAGTTCGAGCTCCTCCTAGATATAGGA
GCGGTTGCGTTGGGCTAGGTACACTAGATTCTAGCTAGAGCTACTAGCTAGTTGCACTAGCCATGGGTGTGCGGAGCTGCAG
AAGGAAGCAACAGCAATGTAGGGGTGGGGGCTTGCTC
>SsLRR-RLK9
AAAAAGTACGGCTCATAACACAAGCGAACAAGACTAAGATATAATACAAACAATGGTCCATCGCATGAGCAAAAAACTGAA
CAAATATTGAATAGTGAATAACAGTGAGTACTGAATAATTTCAACTATCAAGTTCCAGCCGTGAAGGCGGTCGCACACTACTC
AACGATAGCTCAATCACTACAGATGTACATTTAACTAAACTAATGAGCCTACCTTATGTTGGAATAGGAGCAAGGGTGCTGCT
ATTCACCGGCGGTGGCGGCTCCAGGAAGAAACGGCGGTGTGAAAATAAACTAGTTTTAAAGTTGATAAAGTTGGAAAAAAA
ATCCAGTTCTATAATGAGGGAGAAAAATCAAACTACTTTAAAATAAGATAAAGTGGGTTAAAAGGACTTTGTGTAATCTAGA
CCTCTGAAAATTGTGGCCACGTTTGTATCACCAATATCTCCCTCTTGATGAATCATTTTTGCTGGGGGTTCTACGCCATCATTT
ACCACGCCCAACTGTAGCGAGTTGTGGCAGCGCCACAAACGATGGCGGCAGAATCCATCGCCACAGAGTGTGGCAAGAAA
TGAGAGTGACGGTGGTGTGGCTGTCTAAAAGTACGTGTGAATCAAACACTACATCAGTAAATAATGGTGTGCCACAGATGGT
GGCGTGGCGGAGCGTGGCTCTAATCCAAACAGCCCCGGCTATCTATGGTTGGAGAACTAGGCCTCGTCGTGCTCTGCCTGAA
CCCCCCTGCTCTGAAGTTTTGGTACCGCTGCCATGTCCTGCCCTGCACGTTGGTGGCCATTTCTTCAGAAGGCAAATGTGGA
CTGACTGACTGAATTGCAGCCCGAAATCTGAGCTCGACTACGGAGTTACGTCACCCGGTCCCGGTGGCATCGGTCGATGTGA
CTTTTGGCCTTCCCCTTCTTTCCCGCTCCACCACTGCCGGGAGCTGAGGCAGAGCCGCTGAGTCGCCGCTTTCGATTACCCT
TTTCGTCCCTCTTTTGAACCCATCCGTTCGTTGGTGCGTGGGTATCAGCGCCTACCTGGCTATTGGCCGTTGGCGATGCAGCC
CAGATGGCAGGCAGGTAAAAAAACGCACAGTGAATGGATCAATGGAAGCATGGGCTGGTGGGCGCACGCGCCATCGCGCG
ATGCGCGCCCTGTGCACATTCCTCCAGTACACATCAGTGGCTGGAAAACAAAATCCTGAGCAAAATCCATCGGTTTTCCCGT
TGCGAAACGACGTTTTCTCCCTGCGCCCGGCCGCTGACGCGCCGGGACCCGACGGCCGTGGGGAGCCACGCCCAGTGGCC
GCGTGCGGGTGCGGCCGGCAGGTCGAGCCGAGCCCGAGCGGGGCAGTCTCATTTTGCCGGATCGCTGCCTGTCCTGTCCCC
CTCCCGCCGTCCCCCAAATCTCTCGCTCCACGGCTCCAGCTCTGGTTAATTACTACTAGTACTGCTGCCATTAACTCCCGCGC
TGCTTTCCATGTACGGGCGCACGTGCTCGCCTTTCGATCCCCGCGCGGCGGTCGCTGTCCGTCGGGCTCCACCGCCGGGGAG
GTAGCCGTTGGGGGCCGGTGGCGACACCACGGCACAGGACAGGGTACAGACTACAGAACTCCTGCTGCGCTGAATGGGAA
AGATCGGATTCTTTCCTGCCGCGCGGGACCCGCGATGTCAGCGCCCGCGTCCGTAACGTATAGGACGAGCTGGGTTTTAAAA
ACTCCAAATCGTCTCCCTCTGCAGAAGGACTGAGCTAGCCGTTAGTTAGCGCATCTGAGTTGAATCGGGTTAGCAGCTAAAT
TATTCCCTCCTTTTTCCGTTCTTCTGCCATGTTCCTGCAACGCTTATATAGAGCGCGGTACCATCGCTTTCCCCGCTAGCAGAA
CCAGAAACCAAGAAAGAGCTCGCTCAAAAGTTCGAGCTCCTCCTAGATATAGGAGCGGTTGCGTTGGGCTAGGTACACTAG
ATTCTAGCTAGAGCTACTAGCTAGTTGCACTAGCCA
>SsLRR-RLK358
AGTGTGGATGATAAAGTGCTTACCATGGTGATGAAGGAGGAACAAGGTGCAACCCTATAGAGCTCAAGGCAAAGGTATCAA
TAGGGTTTTGCATTTTGGTGATCAAGACACCTAGAGGGCGTGATCACATTTAGGATCGATAACCGTACTATAAAGAGGGGAAT
TTCTGGGGCTATTAATCACTTAAGTGCCACTAGATGAAGATTCATTTGCATATGCATTTAGATCTAGTGGAGAACTAACTTAAC
GCCTCGAAAAATGTTTGTGAAAATGCTAACACACTTGCACATGACATTGATCACTTGGTGGTGTTAGCACATTTGCGAAGGT
GGTGGAGTTTGAGCGGTAGAGAGGGGTTTAGGTTTCTCTCTCCTTCCCGCCGAGCTTGCGAGGCGGGATTCAGCGCTTTTCG
GGAAATGAAACACCTATTTTCCACTACGCCAGACGCGAAGAAACAGAGGCGAACGGAGGTCACAGAGGTGACCGGACGCT
GAACCGGACGCGGTCTCAGCGTAACCGGACGCGTCCGGTCAGTGTCAGCGGTCAACCTGGCGTGGAGCAAAATCAGCGAA
AACACATGATATTAAACACTGCTACAATGGTCAAAGGCCTTTTGCCATGCCGCGCCATCCTCGCAAACCGACATGTTGGGGA
GTCTGGTGGCTGTCCAGTTTGTCAAAGTGGAGCTGAGGATATTAAACACATGATATTTACATGCGAACGAGCGAAACAAGTG
TGGCGTTCGCTGGGAGTTGAAGAGAGGATGCAACGCTTGATCATGACTGATAGATCGGGCTCTGTAATTCTTGAGGAAATAT
TCTTAATGGAGGATCAAGTTCAGAACCTTGAGGTGGGGCTTGCAGAGCTAGTAATCACAACCGCATGGTATATATGGTGGGA
ACGTCGGCAATTGGTCCATGGAGAGACTGTCCAGCGACCGTCAAGGTCTGCCTTGGCTATTGCGGCGCTTACTAAGAATTAC
AAGCTGGCGACCAAGAAAGCTTCAATTCTAAGGCAGTGATGGAAGAAACCCCCAAAGGGTAAAGTCATGGTGAATGTTGAT
GCTGGTTTCGACGAAAATGGTGGTTGCGGCAGTGTGGGCTCTATTATAAGAGATTGCTCGGGTGGAGTGTTAGCAGCAGCCC
ACGGTTTTGTACCACATCTTATTGATACACCGATGGCGGAGGCTTATGCGCTGAAAGAGGGATTGATGCTAGCGCAGCACATT
GGGTGCAATCGATTAATCATTCAGTCGGATTGCATGGAAGTGGTACAAATTATGAAGGATGGAGGCGTCTCAGCAAATTCGG
CGGCTGCAATATATGATGATTGCAATATCATATGGAGTGGTTTTCATGATATTTCAATTGAACATTGTAGCAGGGAGGCGAATC
AGGTGGCTCACAATCTAGCTAGAAGAGCTCGGCAATATAAACAAAATTGTACTTGGGTCTATGAGCCCCCTAGCTTTATATTA
GACTCTTTGATGAACAATGTAACTATCTTGAATGAATAAAGATTGTCGTATTGCTTAAAAAAAAACTGCTGCGGCTCCCGAGAA
AGGAGCAGGGAGGCGGCCAGGTGGGCCTGCCCATCGAGATATTCTCCCCGACCCAGCCCAACCTGAGACTGGCCGATTTGG
CCAGTCCAAGACCGCTGTTGGCCTGCTTCGGAACTAGCCGTTGAAGAACAAGAAAATAATAATTAAACCGCCCTCCAAATCC
TCGTGAGTGGCTGACTGACTGCTTCCCTCGCTCCCTAGTCCCTACTAGCCGTTGGCGCCCATTCCTCTCCTCCCCTCATCTTT
CTTCTTCACAGAGTTTCGCACACCCTCTCTTCTTCTCCATTCACCGCAGGCAGCAAACTCGACTGCTCTCCTTCCTCCTCTCC
TCTCCGTCGCCATCCTAGCTCATTAGCTATTAGCTTTGCTTCCTAATCCCCCAGCAAAACACTCCCCATTTCCCCTTCAATTTC
CATCCCAAACCATCACCCACCACCACC
>SsLRR-RLK298
GTTTAAAAAATGACAACTTCAAATAGCATTTGGAAACATACGTACCGACAAGTCGGAGTCTCATACGAATCGTGACGCGGTC
GAGGCATCCCGCCGAGGGTGCCTCGTACGAGTCGGAGTCGGAGCACGGTCGAGGTGTCCCGTCGAGCCGGCCTCGTGCGA
GTCGGAACGCAGTCGAGGTATATCGTCTGCTATTTTTGGTCCTTGCTGTGTTTGCGCATTAGTGTTTTCGTTCGTCTTAACCGT
TGATTGTGGGTACCCTATACTATTGGTACCGACAAGTGTCCTCTTAAGTTTGTATCTGCAACCTTGAGGTATTTTTTTCTACCTT
TATAAGGCACGGACATATTTTTCTTGTGAATGTATCATAAATTTACCGTGTATTTGGTTTGGGGATACGGTGGGATGAGATGGT
CCCGTCCGTGAATTGTGGGATAAGACCATTGCATCTCATATTTTGTTGGTCGAGATGTTCTAGTTCTTTGTTTCATTGGAGGGA
CAAGCAGGAAAAAATGGACACTGTTCTGTGCACCGTTAACCTATAGCAGTCATTGGACCTATTTGCCATTGTTTTGACAAAC
GTGACGAAGTTAAATGGTACATGGTTTTTGTTTCAAAAAATGGTACATGGTTTTGACATCCTCCTTTTTGTATATGATCAGGAT
GTTTTGCCATCGTCATTTTATCATATATATACTCACACGCATACAGACCTTACCTTACCTTACCTTACCCATGAGCACTGACAGA
AAAGGTCCTGCTAGCGTCTGGACGTTCGGATGGACACGCATGTTTGGACGCCCGAACATGCTGATGCACCTACCCTCGTGCT

GTTGCGCCCTGCGCCTCCTCTTGCTGCTGTTGCGCCCCATGCCGCCTCCTGCTACTCTTGCGCCTGCTCGGAGAGCCTCCGCC
AAATTGAGAGGGAGCATAGCGTGCGTGTCGCAGTGTGAGAGAGAGACCAATAATTGAAACATACGTTTGTAACATATGTGTA
TAACCACCGCAAACATATACAACATCTAGATAAAATATTTGCAACATACGTCTGAAACAGGTGAAACATTTAGAACATATACT
TGCAACATACGTGTATAATCACTGCAACATATGCAACATGCAAATGAAACACATGCAACATCTAGATGAAACAGCCGAAATAT
TTGAAAACATACTTGCAACATACGTGTATAGTCACTACAACATATGTTTGAAACAGCTGAAACATGAAAACATACACTCGCA
ACATACGTTTATAATCATTGCAACATATGCAACACTAGATCTATTTTTGCAACGTCCAGATGAAATGTATGCAACATACATCTA
AAACGCATGAAACACACAGTCGCAAGATGTGCTCATCTACTTGCTGCCGCCCAACGAAGGCTCGTTGACGCAGCGGGAGGC
ACGAACGGCACGCGGCACGAGGTGGAATGCCGCAGCGGACGAATAGGAAAGAGAAGCATCCATGTTTTTTTCAATCACCCT
GTTGTCTCGTGCTTATCAGCGCTTATCAGTTAGCCATTCAGCAGTATTTTTCTCTCACAATAAATCCGCATCAGCCAGAGTCAG
CCAGCCAGAAGCCATCCTACGGGTAGAAAGCATTACCGTTGTAGGAAAAATAAAACAAAAACTAGAGAAGGTCGATTGGCT
CTGCTCGGTGTCGTGTCGTGTCGTGTGCGCCAAAGCCAAAACGCGTAAAGGAAGGGAGCATCAGGTTGTTCTATTTGTTGG
ACGACGTGGGCCCAATACCTTGCTCTAACAACCGTTGCGGTTCCCGAGAAAGGAGCAGGGAAGCGGCCAGGTGGGCCTGC
AGACTGAGACATTCCCCGACCCAGCCCAAACCCTGAGAGTGGCTGATTGGGCCAGTCCAAGAGCGCTGTTGTTGGCCTGGT
TCGGAACTAGCCGTTGAAGAAGAAGAGAAATAATTAAACCGCCCTCCAAATCCTCGTGACCGCTGACTGCTTCCCTTCCTCC
CTTTTCTCCCTACTAGCCGT
>SsLRR-RLK393
ACTCTGGTGCAATTGTTCACAAGAACAGTGTGGTGTTTCCTCCGATGCTCACTAGGGTCTGACGCCCTTGCTCAGTTCGACA
CCTGCTGATACCATGGCTAGGCTTAGCTCTTAGCCTATGGTCTGGTGCCCCTTCTCAGTCTGACGCCCTACTAAATCCTCTTCC
AATTTGTTTCTTCATGGTCCTCGCGTTCGGCTGGTTCCATCTTATGTCTTGGACCTTTGCATGTCTTGTGAGTCTTCTTTTTTGC
TTCTAATGTCTCGCTTGAGGTGTTGATCATCCAGATCACATCATTGCCTAAGTTTAAGTTCACTTTTGCACCATTTGAACTATAT
CCAAAAACACTAGCAAATAATGTTAATCCAAATGGTTATGTTGATCATCAAATACCAAAATCCAAACTAAATGGCCCATGGGG
TCCATTTTCCTTACAATCTCTCCCTTTTTAGTGCTTGATGACAACACGACCAAAGCAAGCAAATAATAGAAAAAAATCAAAAT
GCAATCTACTTGCTAGGATGCAATGCAAAGGCAAGATCATATGATGTTAAAAGATACTATTTGAAGTAAATAAGCGGTACTCA
TCTTGCCCTTGCCATTTGTCCCCTTGTGTGGTATTATGGACTTAAGCCTCCCATAACTCCATAAATCACTTCCTCTCTTTCTAGG
ACCATCTCCCAAAGTAGGATTCTCCCCCATTTTTATAATCTCCCATAGCCAAAACCACTTGTATGCTTGAAAATTTTTGAATTT
GTGATTTTGGTATTTATTATGGGTTGGGCTTGCTTTTGGTTGTACAAATTCTCTCCCTTTGGAATCAAACACCGAAAAGAAAA
ACATTAGTAACACAAAGATTGAATCTGGCTTGCTTTATTCCCTTAGCGGATTCATATAAATAGCCTAGATTACGACCAACCGAT
CTACGAATCCTCAACTAATCAAACAAATAAACCAATAATGAAAATCTAAACAAATTCTAATCTGAAACAAACCAAAACTTCT
GCCGCCTTATCCTAACCGAACAGCTTACTCCCTACGCCGCCTCCTCTGATATGTGTTGCCAACCATAACACTGAACCATAACA
CTGGAGGGAAGCTGTTTCAAGAATGGGCAACAAATCTAATAAAAAATATGCCCAGGATTAAAAAAACTTCAATGAAACAAA
GGAGCATGACTGTTTAATGTATTTGGCCTTCTAAAAGGAAACAAAGCATTATGTAGTTCTATGTCTTAAGCTAGTATTTGGCCT
TCTAAAAGGAAACAAAGCGTATGCAGGGGCAGGCTTTATCAGTATGCAGGTCAGACGGGGCAAGCGCAGCACATGCCGGG
CGGACGCAGCAGGCGGGCAGACGCGGGCGTCTAGACAGGAACTAGCCACGCCGGAAAAAGAAACTAGAGAGGGTCGATT
GGCTCCGCTCGGTGTCGTGTGCGCCAAAGCCAAAATTGGTATAACGGAACGGAGCATCAGGTTCTATTTGTTGGACGCTGGG
GATTGGCGTGGAGCCGTGGGCCCAATACATCTAGTCTACCAACCGTTGCGGCTCCCGAGAAAGGAGCAGGGAAGCGACCAG
GTGGGCCTGGCCATCGAGATATTCTCCCCGACCCAGCCCAACCTGAGAGTGGCTGATTGGGCCAGTCCAAGACCGCTGTTG
GCCTGCTTCGGAACTAGCCGTTGAAGAACAAGAAAATAATAATCAAACCGCCCTCCAAATCCTCGTGAGTGGCTGACTGCTT
CCCGCCGTGCAGCCGTGCAGGCCACCCACGCTGCCTTTTCTGCTCTTCACTCCCTAGTCCCTACTAGCCGTTGGCGCCCATTC
CTCTCCTCCCCTCATCTTTCTTCTTCACAGAGCTTCGCACACCGCAGGCAGCAATCTCGACTGCTCTCCTTCCTCCTCTCCTC
TCCGTCGCCATCCTATTACCTTTGATTCCTAATCCCCCAGCAAAACACTCCCCATTTCCCCTTCAATTTCCATCCCAAACCATC
ACCCACCACCACCA
>SsLRR-RLK381
GATGTATTTGGCCTTCTAAAAGGAAACAAAGCATTATGTAGTTCTATGTGTTAAGCTAGTAAAGTGAATACATCAACTGCTTG
CAGCAGGAATCTTCCATAGAAGTTTATTGACAGTACTACTATTTCATAAGCAAAGATAGATGCGATAGTCATACAGGTCAATA
ATTTGATAGAAATATTAAGATACATGAATGATAATATCTTAGTCTTGTAGGAGAGACATTTAATGAAATAATACAACAGCAACA
ACCTTTAAGGTTCCAAGTGTGCTAATGGAGCTACCCATTCACATGCAAGATGGTTCAACTTTATTTTTAGGTTTGAGTGTCGT
TGAAAAGAAAGCACCATACAGTATACTATACTTACACTGATAGCTAGGCAACAAGAATATGTTTAGCTTTCTCGCTATTTAAA
ATCTGTTTTAAAGCACACCATATAAAGAATTGTGTGCCCGTGAGTTTAGCAACCAGAAAAAGAAAAAAAATGATGCATGCTT
AAATCAACTCATGATTTTGTATCGGTTTCATTCTCTCTCATTGGTACGTCACATGAGATGATGAGTCGGTTTCAAAAAGGTGCT
AGAGAAGCCCTTATACCTTACCTATGAGCGGTTAGAGAAAAAAGGTAATGCTTGGTTGAGGGCGTCTGTTTGACCAGACGG
ACTGACTCATGCTCGTGACGACCAAGAAAAAAAAGAGGGCCCTGCATCGGTCCTTTTTGCAGGGTAGCGCGTGGGAGTGCA
TGTGGCCACTCTCGTTCAGTGTGGGGGTGATGTGGCGCAGGGCAGTGCATGGGAGCACGTGCGGCCATTCACTTGGGCGT
AGGGTGCATGCGGCGCGTGGGGGCCGTGCATGCCCTGTGACCACAGAGAGAAGGACCGGTGTTGGCAGTTTGCGAGGAGA
GCGCATGGCCCGCATCTGCATGTGTCATTTGTTGCTCTCGTGACGGTTGAGCGTCAGCGGCTAAGTGCTGGCCCAAATTTTTC
TGCCACTCCATGGCATTGAGCCCCATATGTGTTGTGCGTTTCAGATGTACGTTTCATATGTTTCATCTAGATGTTGCATGCGTTT
CATCTGGATGTTGCATAGCAACGGTTATATACGTAAGTGTATATCTCATCTGTTTCAGATATATGTTGTAAGTGGACGTTGCATA
TATTTAAGTGGCTATACACATATGTTGCAAACGTTTATTTAAAGTGTTTCATCGGTTTCAAACATATATTGCAAGTGTTTTATCT
GGATGTTGCATATGTTGCAATTGATATATATATGTTGCAAATATTTCACCTGTTTCAGACATATATTGCAGAAATACTTCAGGTT
GCAACATCAATAGGCACAAGAAGCGGGCACATGCGAATACGGTCCCTACGAACACAGCGGTCCCACGTGCGTGCGTGACA
AGCAAAGCGGGCATGGAGGCACGGGCGCACCAGCAGAATCGATTGGCTCCGCTCGGTGTCGTGCGCGCCAAAGCCAAAAT
TTGCATAACGGAACGGAGCATCAGGTTCTATTTGTTGGACGCTGGGGATTGGCGTGGAGCCGTGGGCCCAATACATCTAGTT
TACCAACCGTTGCGGCTCCCGAGAAAGGAGCAAGGAGGCGGCCTGGTGGGCCTGCCCATCGAGATATTCTCCCCGACCCAG
CCCAACCTGAGAGTGGCCAGTCCAAGACCGCTGTTGGCCTGCTTCGGAACGAACTAGCCGTTGAAGGACAGAAAATAATAA
TTAAACCGCCCTCCAAATCCTCGTGAGTGGCTCACTGACTGCTTCCCTCACTCCCTAGTCCCTACTAGCCGTTGGCGCCCATT
CCTCTCCTCCCCTCTTCTTTCTTCTTCACAGAGCTTCGCACACCGCAGGCAGCAATCTCGACTGCTCTCCTTCCTCCTCTCCT
CTCCTCTCCGTCGCCATCCTATTAGCTTTGATTCATAATCCCCCAGCAAAACACTCCCCATTTCCCCTTCAATTTCCATCCCAA
ACCATCACCCACCACCAC
>SsLRR-RLK290
CATCCATATATTAAAAATAATTTTAGTAGCAATATCAGTGTCCTATTAAGTTTGTATCTGCAACCTTGAGGTATTTTTTTCTACCT
TTATAAGGCACGGACATATTTTTCTTGTGAATGTATCATAAATTTACCGTGTATTTGGTTTGGGGATACGGTGGGATGAGATGG
TCCCGTCCGTGAATTGTGGGATAAGACCATTGCATCTCATATTTTGTTGGTCGAGATGTTCTAGTTCTTTGTTTCATTGGAGGG
ACAAGCAGGAAAAAAATGGACACTGTTCTGTGCACCGTTAACCTATAGCAGTCATTGGACCTATTTGCCATTGTTTTGACAAA
CGTGACGATGTTAAATGGTACATGGTTTTCGTTTCAAAAAATGGCACATGGTTTTGACATCCTCCTTTTTGGTATATGATCAGG
ATGTTTCGCCATCGTCATTTTACGTATACACTCACACGCATACAGACCTTACCTTACCTTACCTTACCTTATCTTACCTTACCCA
TGAGCACTTACAGAAAGGGTCCTGCTAGCGCCTGGACGTTCGGATGGACACGCATGTTCGGACGCCCGAACATGCTGATGC
GCCTACCCTCGTGCTGTTGCGCCCTGCGCCTCCTCTTGCTGCTGTTGCGCCCCATGCCGCCTCCTGCTACTCTTGCGCCTGTT
CGGAGAGCCTCCGCCAAATTGAGAGGGAGCATAGCGCGCGTGTCCCAATGCAAGACAGAGAGAGCATAGCATGCGTGCCG
CAGTGTGAAAGAGAGACCAATATGAGAGAGAGAGACCAGTAATTGAAACATACATTTGTAATATATGTGTATAACCACCGCAAA
CATATGCAACATCTAGATAAAATATTTGCAACATACGTCTGAAACAGGTGAAAGATTTAGAACATATACTTGCAACATACGTG
TATAATCACTGCAACATATGCAACATGCAAATGAAACACATGCAACATCCAGATGAAACTGTCGAAATATTTGAAAACATACT

TGCAACATGCGTGTATAGTCACTACAACATATGTTTGAAACAGCTGAAACATGAAAACATACACTCACAACATACGTTTATAA
TCATTGCAACATATGCAACACTAGATCTATTTTTGCAACGTCGAGATGAAACGTATGCAACATACATCTAAAACGCATGAAAC
ACACAGTCGCAAGATGTGCTCATCTACTTGCTGCCGCCCAGTGAAGGCTCGTTGACGCAGCGGGAGGCACGAACGGCACGC
GGCACGAGGTGGAATGCCGCAGCGGACGAATAGTAAAGAGAAGCATCCATGTTTTTTTCAATCACCCTGTTCGCTCGTGCTT
ATCAGCCAGCCATTCAGCTATATTTTTCTCTCATAATAAATTTGCATCAGCCAGAGTCAGCCAGAAGCCATCCAGCGAACCGG
GTAAATAAAAATGGCGGGTAGAAAGCATTACCGTTGTAGAAAAATAAAACAAAAACCAGAGAAGGTCGATTGGCTCTGCTC
GGTGTCGTGTCGTGTGCGCCAAAGCCAAAACGCGTAACGGAACGGAGCATCAGGTTGTTCTATTTGGTGGACGCCGTGGGC
CCAATACCTTGCTCTAACAACCGTTGCGGTTCCCGAGAAAGGAGCAGGGAAGCGGCCAGGTGGGCCTGCGGACCGAGACA
TTCCCCGACCCAGCCCAAACCCTGAGAGTGGCTGATTGGGCCAGTCCAAGAGCGCTGTTGTTGGCCTGGTTCGGAACTAGC
CGTTGAAGAAGAGAAATAATTAAACCCACCTCCAAATCCTCGTGACCGCTCAGTGCTGGCTGACTGCTTCCCTACTAGCCGT
TGGCGCCCATTCCTCCCCTCCCCTCCCCTCATCTTCTTCACAGAGCTACTAGGCACACTTGCAGGCAGCAATCTCTACTGCTC
TCCTTCCTCCTCCTCTCCGTCGCCGACCTCACTAGCTCGCTTTGCTTCATAATCCCCCCAGCATTCCCCTTCCATTTCATCCCA
AATCATCACCCACCACCAC
>SsLRR-RLK275
GCGCATTAGTGTTTTCGTTCGTCTTAACCGTTGATTGTGGGTACCCTATACTATTAGTACCGACAAGTGTCCTATTAAGTTTGT
ATCTGCAACCTTGAGGTATTTTTTTCTACCTTTATAAGGCACGGACATATTTTTCTTGTGAATGTATCATAAATTTACCGTGTAT
TTGGTTTGGGGATACGGTGGGATGAGATGGTCCCGTCCGTGAATTGTGGGATAAGACCATTGCATCTCATATTTTGTTGGTCG
AGATGTTCTAGTTCTTTGTTTCATTGGAGGGACAAGCAGGAAAAAATGGACACTGTTCTGTGCACCGTTAACCTATAGCGGT
CATTGGACCTATTTGCCATTGTTTTGACAAACGTGACGAAGTTAAATGGTACATGGTTTTTGTTTCAAAAAATGGTACATGGT
TTTGACATCCTCCTTTTTGGTATATGATCAGGATGTTTTGCCATCGTCATTTTATCATATATATACTCACACGCATACAGACCTTA
CCTTACCTTACCTTACCCATGAGCACTGACAGAAAAGGTCCTGCTAGCGCCTGGACGTTCGGATGGACACGCATATTTGGAC
GCCCGAACATGCTGATGCACCTACCCTCGTGCTGTTGCGCCCTGTGCCTCCTCTTGCTGCTGTTGCGCCCCATGCCGCCTCCT
GCTACTCTTGCGCCTGCTCGGAGAGCCTCCGCCAAATTGAGAGGGAGCATAGCGTGCGTGTCGCAGTGCGAGAGAGAGACC
AATAATTGAAACATACGTTTATAACATATGTGTATAACCACCGCAAACATATACAACATCTAGATAAAATATTTGCAACATACGT
CTGAAACAGGTGAAACATTTAGAACATATACTTGCAACATACGTGTATAATCACTGCAACATATGCAACATGCAAATGAAACA
CATGCAACATCTAGATGAAACAGCCGAAATATTTGAAAACATACTTGCAACATACGTGTATAGTCACTACAACATATATTTGA
AACAGCTGAAACATGAAAACATACACTCGCAACATACGTTTATAATCATTGCAACATATGCAACACTAGATCTATTTTTGCAA
CGTCCAGATGAAACGTATGCAACATACATCTAAAACGCATGAAACACACAGTCGCAAGATGTGCTCATCTACTTGCTGCCGC
CCAACGAAGGCTCGTTGACGCAGCGGGAGGCACGAACGGCACGCGGCACGAGGTGGAATGCCGCAGCGGACGAATAGGA
AAGAGAAGCATCCATGTTTTTTTCAATCACCCTGTTGCCTCGTGCTTATCAGCGCTTATCAGCGCTTATCAGTCAGCCATTTAG
CAGTATTTTTCTCTCACAATAAATCCGCATCAGCCAGAGTCAGGCAGCCAGAAGCCATCCTACGGGTAGAAAGCATTACCGT
TGTAGGAAAAATAAAACAAAAACTAGAGAAGGTCGATTGGCTCTGCTCGGTGTCGTGTCGTGTGCGCCAAAGCCAAAACGC
GTAACGGAACGGAGCATCAGGTTGTTCTATTTGGTGGACCAATACCTTGCTCTAACAACCGTTGCGGTTCCCGAGAAAGGAG
CAGGGAAGCGGCCAGGTGGGCCTGCGGACCGAGACATTCCCCGACCCAGCCCAAGCCCTGAGAGTGGCTGATTGGGCCAG
TCCAAGAGCGCTGTTGTTGGCCTGGTTCGGAACTAGCCGTTGAAGAAGAGAAATAATTAAACCGACCTCCAAATCCTCGTG
ACCGCTGACTGCTTCCCTTCCTCCCCGCCGTGCAGGCCACCCACGCTGCCTCCTCCTCCCTTTTCTCCCTACTAGCCGTTGGC
GCCCATTCCTCCCCTCCCCTCATCTTCTTCACAGAGCTACTAGGCACACTTGCAGGCAGCAATCTCTACTGCTCTCCTTCCTC
CTCCCCTCTCCGTCGCCGAGCTCGCTAGCTCGCTTAGCTTCATAAACCCCCCTGCAGCATTCCCCTTCCATTTCATCCCAAATC
ATCACCCACCACCACC
>SsLRR-RLK431
ATTCCCGAACATACGAAGGGCCCAGAAGAAACTTCATTCCAAACAGAGAAATATGTTGTGGCTGTTAGTCTAACGGCACTAA
TTGCTGTGACGATGAAACAGTTCAATGCACTAATCTGCAATGGAATAGGATAGAAGCAAGAAAAATATGAATCGAAGGTTTT
GAGTATGAAATTAAAGAACGAAGAAAATGATGAGAGATCAAGGGGGAGAATGTTAGGTTTTGATCTCCCACCATTAGGCCC
AACGGCCTTTTGGGCCTTGCTTCTCGCGCCCTGATCGGGGGCGCCCAACCCTATATGGTTGGTGGGCCCCCGTCACACTGCG
CCATAAATAGAGGTGGGGGGCCGGCCGGCTCGAGATACGAGGTTCACCTTAGCCGCCACTCCCCACCGACATCCCGATCTGT
GAAGGGCGCAGCCAGTGATGGGAAGTCGCCACCGTCTCCACCGACCGTCGCTGCTAGCGCATAGATGGCACCAGCGAACG
AGACGGCCGCCCCTGCTCCGACCACTGCTGTCACCGATGGTTTGCGATCCCATACTCTACGTATCCCTTTTCACCCCTCTATG
TCTTTGTAGTTCATGTATTAGGACTTGTGTATATACTGTAAATCACTGTTTGGTTATTTCCTATGACGAATGCACCCGATCTAAT
GAATCTGTGAACTACGAAAACTATCACTTTTGAGTCTAGTTAGTCCATGGTTAGACAAATTTTACCAAATAATAACGAAAGTG
CTACGAGAATTTTTTTCTCAAAAATTTCGGATGTAAACGAGGCCTGTGTATGAAAGCGGCAGGCAGCTGCCCAGACGTAGCA
TTAGCATGCAGCGTTGAAGGATGTAAACGAGGCCTGTGTATGAAAGCGGCAGGCAGCTGCCCAGACGTAGCATTAGCATGC
AGCGTTGAAGTGTATGAATGAATGAAGTGTCCGTTTCCTGTCCAAAAGATTAGTTTTTTTAAAGGAGACGGCAGACGAGAG
ACGACCAGCACCCCGTCCGGTACAGTACCAAACTCGAGACGCCGATCTAACCGAAAAAGAAGCCGTCGACGTGTACACGT
ACACCCGTCACTTTCGTTTTTTCTGCATCAACGACCCTCTTCGCTTGGCTACTTTTCAGTTACGGAACATCGTCTTCTTCTCTC
GTACCATTTCAGTATAAATATCTGTATAAACTAAATTTCAGCGTAAGGGATGACCAGACAACGACCTAATATCTACAACAAAA
ATCCGTTATCCGTCCGTCCCCAACCGCGACCCAACTCAAAAGCATCGCGCGGCCGGCCGGCGGGCCGGCGATGGAGACCCG
AGCCGAGCCGACCGACCGGAGGCGGAGGGCCTGGGCCTGAGCACGCACGGCCGGTCGAGGCTCAGCGTCACGCTTTACGA
TGCCATGATCCACGCCAGCACGCGACGGCCTGGACCTCGAGGCTCGAGCACAGCTGCTGCTCCTACTCGACTTGCCTGCTA
CTGCATCCATCCATGGAGACGGACCGAGCTGTCTGTCCCCTCGGTAGTCGGTAGGGAAGCGGAGCGGAGGTCAGAGGCGAC
AACGACCTGTGTACTGCATGCCGGCTGCCGTCCGGGCTCCCAAACTCGAAAATTCATAGCGGCCGGGGCGTGGAATCTGAG
CCTCGGCCGGGCAGCGCAGCTCAAAACCGGGACGGCCTCGGCTTCCCAGTTCCCACACCCCTTGCAGCCCACGCGGCCAC
CCACCCGGCGCATCGCTTCTTTATTCCGCTCCACTCACCCAGCCTCACATGCCATTGCCAACCTCTCCTCACTGCTCGCACCG
CCTCTCCACCTGCTGCTGCTCCTGAGCCCTGACCGCGAGGGGAGGGCGCCGAGCTACCAAAAGGAACCCGGCCGGGGCGG
GAGCGGTCAACGCCTGCTCTGCAGCCGAGGAAGCTAGGAACCAAGGAGGAGGAGGACCGACGACCGAGGACGGACATGC
TGGCGCCGGCGCTGGGCGTCTAGGAAGCTCGCCGCAGCGGCAA
>SsLRR-RLK421
CAACACCTAACTGAAGGGACACGGGAACAGGAGCTAGCAAAGCAAAACCGTAGCAATGCAAAGCTGCGGCGTGCGTGCAT
GGGCACATGTGCGTGGGTGGCCACCATCACACACTGGTTTGGTTGGCATATGTATGCATGCATGCATGCATCAAGATTCGATC
TCCTGGCCTTTCGTCCTCCAACTCCTTTTTCACACTAGAGCATTGTTGTACAGTAACGTGACAACCGAGTTTTCCTCTTCCGG
GGGACCACAGTATCAAGGTTTATGGAGAAATCGTCTCGCATTCATTGCAAGGCGATCATCCCATCATCCATGCATTTATTCCAG
AGGTCCCCAATGCGAGCCTAACCAGGTCCTCTCTCTGTAACTGTTCGGCTGATGATTTTTCCGGCTAATGTTTTGTTATTAGA
GAAAAACACAGTACCATGACTGATAAGTTCCAGTAAACAGGGTTGTCTTGTGTTGATCTATCGTCTGCTGGCCAGGTTATATA
GCATGTTATATTACTGTTGTACTTTTATATCTAGTTTTATTAAAAAAAAAATGCCCGGTAGGAGGAGGGTAGCGCGAAGTGTCAC
TGTGTATGAAAGCGGCAGGCAGCTGCCCAGACGTAGCATTAGCATGCAGCGTTGAAGTAGAGTATGAATGAAGTGTCCGTTT
CCTGTCCAAAAGATTAGTTTTTTTAAAGGAGACGGCGGACGAGAGACGACCAGCACCCCGTCCGGTACAGTACCAAACTCG
AGACGCCGATCTGACTGAAAAAGAAGCCGTCGACGTGTACACGTACACCCGTCACTTTCGTTTTTTCCGCAGCGACCTGTT
GGCTTGAGCTACTTTTCAGTTACGAAATAGTGTTTTCTTCTCGCAATATTTCAACATAAGCATCAGCATAAACTAAGGCTTGTT
TAGTTCTTACCCAAAAACTTTACAGGCCATCCCATCGAATGTTTAAACACATGCATAAAGCATTAAATATAGATAAAAAATAAC
TAATTACACAATTTACATATATTTTACGATGCGAATCTTTTAAGCCTAATTAGTCCATGATTAAACACTAATTGCTATAGTTATAT
ATGTGCTACAGTACCTCCTAAACCTTTATAGCCCCTGAAACACGCCCTGAATTTCAGCGTAAACCAACAGGGTGAGCACCCG

CGCACCACACACATGGTAAGACAACGACCGCGTAGTAATATCCACGAAACTAATCCGTTATCCGTCCGTCCCCAACCGCGAC
CCAAGTCAAAAGCATCGCGCGGCCGGCCGGCGGGCCGGCGATGGAGACCCGAGCCGAGCCGACCGACCGGAGGCGGAGG
GCCTGGGCCTGGGCACGCACGGCAGGTCGAGGCTCAGCGTCACGCTTTACGATGCCATGATCCACGCCACCACGCGACGGC
CTGGACCTCGAGCACAGCTGCTGCTCCTACTCGACTTGCCTGCTACTGCATCCATCCATGGAGACGGACCGAGCTGTCTGTC
CCCTCGGTAGTCGGTAGGGAAGCGGAGCGGAGGTCAGAGGCGACAACGACGTGTGTACTGCATGCCGTCTGCCGTCCGGG
CCCCCAACTCGAAAATTCATAGCGGGCGGGGCGTGGAATCTGAGCCTCGGCCGGGCAGCGCAGCTCAAAACCGGGGAGGT
TTCAGGTTTCACGGCCTCGGCTTCCCAGTTCCCACACCCCTTGCAGCCCACGCGGCCACCCACCCGGCGCATCGCTTCTTTA
TTCCGCTCCACTCACCCCGCCCCGCCAGCCTCACATGCCATTGCCAACCTCTCCTCACTGCTCGCACCGCCTCTCCACCTGCT
GCTGCTGCTCCTGAGCCCTGACCGCGAGGGGAGGGCGCCGAGCTACCAAAAGGAACCCGGCCGGGGCGGGAGCGGTCAA
CGCCTGCTCTGCAGCCGAGGAAGCTAGGAACCAAGGAGGAGGAGGACCGACGACCGAGGACGGACATGCTGGCGCCGGC
GCTGGGCGTCTAGGAAGCTCGCCGCAGCGGCAA
>SsLRR-RLK430
AAGATCAAGATGGAATAGTAGTAAGGAACAAAGCAAGACTAGTGGCTAAGGTTACACACAAATAGAGGGTCTTGACTTTGG
TGAAACTTATGCCCCGGTTGCAAGATTGGAAGCAATTAGGATCTTACTTGCCTATGCTTGTGCTCATAATATCAAGCTATATCA
AATGGATGTGAAGAGTGCATTTCTCAATGGGTACATTCAAGAGGAGGTGTATGTGGAGCAACCTCCCGGTTTTAAGATGACA
AGAAACCCAATCATGTCTACAAATTGAGAAAGCTTTGTATGGATTGAAGCAAGCACCTAGAGCATGGTATGAAAGATTGAGG
GACTTCTTGCTAACCAAAGGATTTGTCATGGGCAAGTGGATACTACTCTCTTCACCAAGAAGATTGGGAAAGACTTGTTTGT
GTTGCAAATATATGTTGATGATATCATCTTCGGTTCAACCAATCAAGATTATTGTGATGAGTTTGGGAAAATGATGGCAAAGG
AATTTGAAATGTCAATGATTGGTGAACTTAGTTTCTTTCTTGGACTTCAAATCAAACAAATGAAGGAAGGAACATTTGTGAG
CCAAACCAAGTACATCAAAGATATGCTCAAAAGTTTGGCATGGAGATGCAAAGGGCATTACAACTCCAATGGGCACTAGTG
GAAGTTTGGATAGGACAAGAGTGGGCAACATGGTGGATCAAAAGCNNNNNNNNNNNNNNNNNNNNNNNNNNNNNNNNNNNN
NNNNNNNNNNNNNNNNNNNNNNNNNNNNNNNNNNNNNNNNNNNNNNNNNNNNNNNNNNNNTGAAAGCGG
CAGGCAGCTGCCCAGACGTAGCATTAGCATGCAGCGTTGAAGGATGTAAAACAGGCCTGTGTATGAAAGCGGCAGGCAGCT
GCCCAGACGTAGCATTAGCATGCAGCGATGAAGTGTCCGTTTCCTGTCCAAAAGATTAGTTTTTTAAAGGAGACGGCAGAC
GAGAGACGACCAGCACCCCGTCCGGTACAGTACCAAACTCGAGACGCCGAGACGCCGATCTAACCGAAAAAGAAGCCGTC
GACGTGTACACGTACACCCGTCACTTTCGTTTTTCTGCATCAACGACCCTCTTCGCTTGGCTACTTTTCAGTTACGGAACAT
CGTCTTCTTCTCTCGTACCATTTCAGTATAAATATCTGTATAAACTAAATTTCAGCGTAAGGGATGACCAGACAACGACCTAAT
ATCTACAACAAAAATCCGTTATCCGTCCGTCCCCAACCGCGACCCAACTCAAAAGCATCGCGCGGCCGGCCGGCGGGCCGG
CGATGGAGACCCGAGCCGAGCCGACCGACCGGAGGCGGAGGGCCTGGGCCTGAGCACGCACGGCCGGTCGAGGCTCAGC
GTCACGCTTTACGATGCCATGATCCACGCCAGCACGCGACGGCCTGGACCTCGAGGCTCGAGCACAGCTGCTGCTCCTACTC
GACTTGCCTGCTACTGCATCCATCCATGGAGACGGACCGAGCTGTCTGTCCCTCGGTAGTCGGTAGGGAAGCGGAGCGGAG
GTCAGAGGCGACAACGACCTGTGTACTGCATGCCGGCTGCCGTCCGGGCTCCCAAACTCGAAAATTCATAGCGGCCGGGGC
GTGGAATCTGAGCCTCGGCCGGGCAGCGCAGCTCAAAACCGGGACGGCCTCGGCTTCCCAGTTCCCACACCCCTTGCAGCC
CACGCGGCCACCCACCCGGCGCATCGCTTCTTTATTCCGCTCCACTCACCCAGCCTCACATGCCATTGCCAACCTCTCCTCAC
TGCTCGCACCGCCTCTCCACCTGCTGCTGCTCCTGAGCCCTGACCGCGAGGGGAGGGCGCCGAGCTACCAAAAGGAACCC
GGCCGGGGCGGGAGCGGTCAACGCCTGCTCTGCAGCCGAGGAAGCTAGGAACCAAGGAGGAAGAGGACCGACGACCGAG
GACGGACATGCTGGCGCCGGCGCTGGGCGTCTAGGAAGCTCGCCGCAGCGGC
>SsLRR-RLK410
CTACCACAAGGGACCCACTAACCATAACCTCGAAGATTGTCACATGCTCCGCAGGTACTTTGAGGGCGTAGGCATCAAGAA
GGACGACAAGAAGGAAGACCCCAAGGACAACGACAAGGATGGGGGATTCCCCGAGATCCATGACTGCTTCATGATCTACG
GAGGACCCTCGACCCGCCTCTCGACGCGCCAGCGCAAACAAGGGCGGCGTGAGGTTTTCTCCGTACAAACGGCGGTCCCC
CGCTTTCTTGACTGGTCTGATATGGCCATCACTTTCGACCGTGATGATCATCCTGACTACGTCCCTAATCCAGGAGTCTACCCG
CTTGTTGTTGATCCCGTCATCGACAACACACGCCTCACCAAGGTACTCATGGACGGCGGAAGTAGCCTCAACATCATCTACG
TCCAGACCCTCGACCTCTTGGGGGTCGAGAGAACGTGCCTCCAACCAGCGTCGGGGGTTTCCACGGTGTTGTGCCAGGGA
AACGGGCAGAACCCGTCGGCCGGGTCGATGCTGCCTTGCTTTGGCACACCGTCCAACTTCAGAAAGGAGACTTTCACTTTC
GAAGTGGTCAGATTCCGTGGAACATACCACGCTATCCTTGGGCGCCCGTGCTATGCCAGGTTCATGGCGGTCCCGAACTACA
CCTACCTCAAGCTCAAGATGCCGGGCCCAAACGGTGTCATCACTGTGGGGCCCTCATACGAGCACGCCTATGAGTGCGACG
TCGAGTGCGTCGAGCACGGCGAGGCTGTTTTGGAATCGGCGACCTTGGCTGCGGACCTCGACGGTCTCGCCAATGAGATTC
CCGACCCAAAGCGCCATGCCGGCAGCTTCGAGCCTGCAGAGGACGTCAAGCTCGTGCCCCTCGACCCTGCTGATCCCAAGG
GAAAGGCGCTGAGCATCAGCGCCACCCTCGATCCCAAATAGGAAGCAGTGCTCGTCGACTTTCTCCGCGCGAACATCGACA
TCTTTGCGTGGAGTCCCTCGGATATGCCGGGCATACCCAGGGAAGTCGCCGAGCACGCCTTGGAGATCCGGGCCGGATCCA
AACCGGTGAAGCAACACCTGCGCCGCTTCGACGAGGAGAAGCGCAGGGTCATCGGTGAGGAGATACACAAGCTGCTGGAG
GCCGGATTCATCAAGGAGGTTCATCATCCTGAGTGGCTAGCTAATCCTGTATTAGTTAAAAAAAGAGTGGGAAGTGGAGGAT
GTGTGTAGACTATACTAGTTTAAATAAAGCATGTCCAAAAGTTCCCTTTCCTTTACCACGCATTGATCAAATCGTTGATTCCAC
TGCGGGATGTGAAACTCTCTCATTCTTAGATGCATACTCCGGCTACCACCAAATTAAAATGAAAGAGTCCGACCAGCTCGCG
ACTCATTCATTACCCCATTCGGCATGTATTGCTACGTTACGATGCCTTTCGGTCTCCGGAACGCAGGGGCTACGTACCAGCGA
TGCATGCAACACGTTTTTGGGGAACAATCGGACCAACCGTCGAGGCTTACGTCGATGACATCGTTGTCAAGACCAAACAAG
CCGACGTTGGTAACGACCTTGACGTTGCGTTCAATGCCTCCGGGCAAAAAACATCAAGCTCAATCCTGAGAAATGCGTCTTT
GGCGTTCCTCGAGGTATGCTTTTGGGGTTCATCATGTCTAAGCGTGGCATCGAAGCCAACCCAGAAAAAATCACTGCGATAA
CCAAGATNNNNNNNNNNNNNNNNNNNNNNNNNNNNNNNNNNNNNNNNNNNNNNNNNNNNNNNNNNNNNNNNNNNNNNNNNN
NNNNNNNNNNNNNNNNNNNNNNNNNNNNNNCTGCTGCTCCTGACCTGAGCCCTGACCGCGAGGGGAGGGCGCCGAGCTA
CCGAAAGGAACCCGGCCGGGGGGGAGCGGTCAACGCCTGCTCTGCAGCCGAGGAAGCTAGGAACCAAGGAGGAGGAGGA
CCGACGACCGAGGACGGACATGCTGGCGCCGGCGCTGGGCGTCTAGGAAGCGGC
>SsLRR-RLK133
TCTCTGATTGGGACACACGTCTAATGTATTTGGCTATAAACATCAGGCTTTAAGTATCATTTTTTGTGTAAGTGTACACGTGAC
GTTTGTATCTTCGGGGGTGAGTGTGCGTGCGTGTTATGGACATCTACAGTTGTACTGTGCCGTGTAATTCTAAAAAAACAACA
TTAGCAATATGAGGACGGAAGTGATTCCATCATGCTAGCTTAAGTTCTATCAAATCTCATCACAATGAAAAAATTTTAAGGTG
AGGTATTGGAAAAATAGTTTTTATTGTTTCAGATGCACTTTAGATTGATTTCGAAACATTGGATCTTAGATATATGTTCAATGT
TTTGAAATTTAATAATAATATTTGACATATGGGTTACACTCTAGCTTCGGTTTTAAAAAGTTTTTGTTGATCTAGAACAACGAA
TGCATCGAGAAAAAACATTATATCTTATTGATTTTGTAAATTTAATATTAATTTAGGGATATATTCGAGTTTTAATATAATTTCTCC
TCCCCTATTAAAGAAAGTTAAGTAAGTTTGATGGACATACTGAAATCCTTTACTGACTTAGGGCAACCACAATGTACAGTAAG
TAGTCCATGTATATAGGACCTATGTGATCAGTTCATAGCACTATTTTCTTACAATGTTGAGTCAACATGTATTATAAGAAGAATC
CCACTAGAGATTAAAAAAACTTCCTCTTCCACCCTTTTAGCCAACAAAAGACAGAGAAGTAAGAAATGATAATATTTTTATTG
TTTGTACGTAGTTCATAATCTTATGAGTAGCTTTTGCTACACTTTAGAGCCTATTTTGATTTCTTCCTCTAAACTTTAGAGCTCT
AAAAACTTTATAGCACTTTAGATCACTTTTGAACTATAAAGCTCTAATGTGAGGTGGATTAAAATTTAGAGCTAACTTTAGAC
CATTTGTTTGGAGCTTTGGCTCTAAAATTTAGAGAAGGAAATCTAAACATGACACATAAATCGAACAATAGCCCGGTTTGTTT
TTCCCACTCCTTTCAATGACATGTAAATTGAACAATTAGCCCTGTTTGTTTTTCCCACTCCTTTCAATCTCCTGGAAAGGTAGT
CCATAGGATTAAAATATTTGCTATCAGGTAGCTGGAACATTGTGAGACCAGTCTATAAGGTAGTTCATAGCAAAAGGTACCCA
TAAGCTTATGGACTACCCATAAGGAATAAAAAATATTACTTTTTCTCACTCCTCTCTCTTTCTTTCTTGGTTTTTGCACAGGATT
GTAAAGAGAAATAATTTTTTATCTCATATGGGACCCACCTTATGGACTACTTGGTGGCTGAAACATTGTAGAGACAACGATAG

CTATAGTCTACTGACACAAAAGCTGTTCATATGAACTACTTAGCCCATATACATTGTGGTTGCTCTAAAGGCACATAGGAGAAT
GCGGGAGGAGCAAAGCTGGAGCAGGAGTCACCTTTCTACCAATGCGTCCAAATCATGAAAGAGGGGTCGAAAAAGCTGAC
AAACAAAACAAAAGTACTCAAGCGTGATGCATCAACATCTTCTCTTTCTTCTTGGCCATTGCACACGCACCATGGGACTGGG
GAGTAGGGTACCGTATCCATGTAAGATCCCTCTCTACTCCGTCTCCGCCCATGACTCGTTCGGACGATATATCCCTTTCACCCC
ATTCCCTTTAGCCCCTTTTGATACGATACCTCAAAGACAGGGAGGGAGGGGAGAGGTCTCCGGTGATCCAGTCTCCATTGCT
ATCAGTATCGTATTTTCCTCACTCTCCTCTGCTCCATTGGTAGTCTCCACTCCACTCGTTCTCATCCCCCTCCAAAGTCCTCTC
CTTCCTCCCTTTGCCATTGTTTTGGTGAACCACCTTCTCAACAAATCCAAGCGAGCCAAAGCAATACCGGCACCACCATTGC
CATTGCTGACCACACCTCTTCTTAACCTCCATGCATGCTGCATGCCACGGCACCACCGCTTCACCTCACCTCCACCGCC
>SsLRR-RLK91
GTGTATTCTGGGTCCCTGAACTTGGCTTTGGGTGTCATCTGGGTCCCTGAACTTGGCTTCGGGTGTCATCTAGGTCCCAATGC
TGACGTGGCGCGGTCCTACATGTGGGGCCCACATGTCAGTCGGTTATATTTCTATTTCGGGGTACTGTAGCTATAATTTCTAAA
ATATTCCAAAAATTGTATCTTTTTCATATGATCTCGGATGAAGATGATTTTTATATGAAAAATGTAGCTCTCGACGAGATCTACA
ACTTTCTAGTTTTGAGTTTTTTTATTTGAGATCATTAAAATGCTTAAAAAAAATTAAACAAATTTTCAGCAGTATATTAATCGCGT
ACACGCGCTTGTCGCATTGGAAAAATCATATCTTTTTTATATGCTGTAAAATGGAGATGATTTCTATATGAAAGTTGTAGCTCT
CAATGAGATCTGCGACTTTCTAGTTTTGAGTTTTTTAATTTGAGGTGGCTAAAATGCTCAAAGAAATTAAACCAATTTTCAGG
AATTAATATACTGTTGAAAATTAGTTTAATTTTTTCAACAGATCTCAAATTGGTTTAATATGATCTCGGATGAAGATGATTTTTA
TATGAAAAATGTAGCTCTCGACGAGGTCTACAACTTTCTAGTTTTGAGTTTTTTAATTTGAGGTCGCTAAAATGCTCAAAAAA
ATTAAACAAATTTTCAACAGTATATTAATTCCTGAAAATTGGTTTAATTTTTTTGAGTATTTTAGCGACCTCAAATTAAAAAAC
TCAAAACTACAACTTTCATATAGAAATCATCTCCATTTGACAGCATATAAAAAAGATATGATTTTTCCAATGCGACAAGCGTTG
GTACACGATTAATATACTGCTGAAAATTTGTTTAATTTTTTTAAAAATTTTAATGATCTCAAATTAAAAAAACTCAAAACTAGA
AAGTTGTAGATCTCGTCGAGAGCTACATTTTTCATATAAAAATCATCTTCATCCGAGATCATATGAAAAAGATACAATTTTTGG
AATATTTTAGAAATTATAGCTACAGTACCCCCGAAATAGAAATATAACCGACTGACATGTGGGCCCCACATGTGGGACCGCGC
CACGTCAGCATTGGGACCTAGATGACACCCGAAGCCAAGTTTAGGGACCCAGATGACACCCAAAGCCAAGTTCAGGGACC
CAGAATACACTGTTCAAAGTTCGGGGACCTAAGTGACACCTAAAGCCAAGTTCAGGGACCTAGATGACACCTGAAGCCAAG
TTCAGGGACCTAAAAATACACTGTTCAAAGTTCGGGGACCTAAGTGACACCTGCTGCCAACTTCAGGGACCGCTGGTGAAT
TTCTCTCTTTTCTCTTTGGCCGTGTTTAGTTCCGGATTCGGTGCATAATAAATGCGCATTTTGTTTGTATTTGGTAAAAATTGTC
TAAACTTTGACTAATTAGGCTCAAAATGTTTGTCTCGCAAAGTATAATAAAACTGTGTAATTAGTTTTTAATTTCATCTATATTT
AGTACTTCATGCATGTGCCGGTATTTTGATGTGATAGAAATCTTCTTTTTACATAGTGAAATTTAAGGTAGAAATTGGGACCTA
AACAAGGCATTTCTTCTTGGCCATTGCACACGCACCATGGGATTGGGGAGTAGGGTACCCTATCCATGTAAGATCCCTCTCTA
CTCCGTCTCCGGCCCATGACTCGTTCGGACGATATATCCCTTTCACCCCATTCCCTTTAGCCCCTTTTGATACGATACCTCAAA
GACAGGGAGGGAGGGGAGAGGTCTCCGGTGATCCGGTCTCCATTGCTATCAGTATCGTATTTTCCTCACTCTCCCCTACTCCA
TTGGTAGTCTCCACTCCACTCGTTCTCATCCCCCTCCAAAGTCCTCTCCTTCCTCCCTTTGCCATTGTTTTGGTGAACCACCTT
CTCAACAAATCCAAGCGAGCCAAAGCAATACCGGCACCACCATTGCCATTGCTGACCACACCTCTTCTTAATCTCCATG
>SsLRR-RLK71
AAGAGGTCTGAGAGGTCCACCTCTGAAATGGATTTTGGGAGACGGGCCTATGCTAATGGATTTTGGGAGACGGATTACTTTT
GACAGCCTACGTAAATCTATTTTGTGAGACGGTCAATATACGATCACCTCCGTAAATAAAGTGACCACAGCCGTTAATGATTG
GGTGGTGCTTGCGAAATCATTTATGTAGTAGGGAATATTTTATTAAATTTCAAAGCAGTTGCATCAAGTTTGATATAACCCTCT
TGAAAGCACTATCGATCTGAAATCACATGCACTCTAAGAGTAATCAATCCAAAAACTACCCATGTGCCTAACAAAAACACGC
TTTGGCCAGTGCTCCAATTGCTAACATGCCACTGCAACCAAATCATTGGAAACGCCCGAGGTGTTCCCACGACCATGATAGT
ATTCGCTCACATGTAAGTGCTGGAAAAAAGTTTTTTTATTGTTTTAGATGCGTTTTAGACTGATTTCGAAACATTGGATTTTA
GATATATGTTCATTGTTTTGAAATTTAATAATAAGATTTGACATATGGGCTGTGCTCTAGCTTCGGTGCTTAAAAAGTTTTTGTT
GATCTAGAACAACGACTACATGGAGAAAGAACATTAGAGATGAAGATGATAGAAAAAAACTAGATAGAATATTATATCTTATT
GATTTTATAAATTTAAATATTAATTTAGGGATATTCGAGTTTCAATATAATTTCTCCTCCCCTATTAAAGAAAGTTAAGTTTGATG
GACATACTGAAATCCTTCAAGACTTACCCCCTGTTCGGCTGAACTTATCAGCACGGCTTATCAGCCGTGGAACAGTGTTTTTC
TCTCACAGCAAATCAGCCACAGCCGGCTTATAAGCCAAAAAACAGCCTGCCGAACAGGGCCTTAGTTCAACCACAATGTAC
AGTAAGTAGTCCATGTATATAGGACCTACGTGACCACTTCATAGCACTATTATTTTCTTACAATGTTCAGTTAACATGTAGTTAT
AAGAAGAGTCCCATTAGAGATAAAAAAAACTTCCTCTTCCACCCTTTCAACCAACAAAAGAGAGGTAAGAAATGATAATATT
TTTTTATTGTTTATATGTAGTTCATAATCTTATGAGTAGCTTTGCTATACTTTTGTCCTTCCTCTAAACTTTAGAGCTCTAGAAGC
TTTATAGCACTTTAGATCACTTTTGAGCTCTAAAGCTCTAATGTGAGGTGGGTTAAAGTTTAGAGTTAGCTTTAGACCATTTAT
TTGGAGCTTTAGCTCTAAAATTTAGAGAAGAAAATCTAAACAGGCCCTTGTCCTATATGGTAAATTGAACAATAGCCCGGTTT
GTTTTTCCCACTCCTTTCAAAGACATGTAAATTGAACAATTAGCCCGGTTTGTTTTTCCCACTACTTTCAATCTCCTGGAAAG
GTAGTAAAGGCACATAGGAGAATGCGGGAGGAGCAAAGCTGGAGCAGGAGGAGTCGAAAAAAAGCTGACAAACAAAACA
AAAGTACTCAAGCGTGATGCATCAACATTTTCTCTTTCTTCTTGGCCATTGCACACGCACCAGGGATTGGGGAGTAGGGTAC
CGTATCCATGTAAACTGTCATATTCTCTTAGAGCATCTCCAGCTCCGTCTCCGCCCCATGACTCGTTCGGACGATATATCCCTTT
CACCCCCATTCCCTTTAGGCCCTTTTGATACGATACCTCAAAGACAGGGAGGGAGGGGAGAGGTCTCCGGTGATCCAGTCTC
CATTGCTATCAGTATCGTATTTTCCTCACTCTCCTCTGCTCCATTGGTAGTCTCCACTCCACTCGTTCTCATCCCCCTCCAAAGT
CCTCTCCTTCCTCCCTTTGCCATTGTTTTGGTGAACCACCTTCTCAACAAATCCAAGCGAGCCAAAGCAATACCCGCACCAC
CATTGCCATTGCTGACCACACCTCTTCTTAACCTCCATGCATGCTGCATGCCACGGCACCACCGCTTCACCTCACCTCCACCG
CCAT
>SsLRR-RLK112
AACATGTCTACTCGGGTTTAAGTCATCGACTTGGCACGGGTGCTCGCATTTTTCTGGATTTATTCAAGGATTTAACGACGCTAT
GCTTTCAGTGGTAGGCGACGTCCCCGTCGACAGCGTGACGCCTGTGGTGACTTCGGGGGTGAGTGTGCGTGCGTGTTATGG
ACATCTACAGTTGTACTGTGCCGTGTAATTCTAAAAAAACAACATTAGCAATATGAGGACGGAAGTGATTCCATCATGCTAGC
TTAAGTTCTATCAAATCTCATCACAATGAAAAAATTTAAGGTGAGGTATTGGAAAAATAGTTTTTTATTGTTTCAGATGCACT
TTAGATTGATTTCGAAACATTGGATCTTAGATATATGTTCAATGTTTTGAAATTTAATAATAATATTTGACATATGGGTTACACTC
TAGCTTCGGTGTTTAAAAAGTTTTTGTTGATCTAGAACAACGAATGCATCGAGAAAAAAACATTATATCTTATTGATTTTGTAAA
TTTAATATTAATTTAGGGATATATTCGAGTTTTAATATAATTTCTCCTCCCCTATTAAAGAAAGTTAAGTAAGTTTGATGGACATA
CTGAAATCCTTTACTGACTTAGGGCAACCACAATGTACAGTAAGTAGTCCATGTATATAGGACCTATGTGATCAGTTCATAGCA
CTATTTTCTTACAATGTTGAGTCAACATGTATTATAAGAAGAATCCCACTAGAGATTAAAAAAACTTCCTCTTCCACCCTTTTA
GCCAACAAAAGACAGAGAAGTAAGAAATGATAATATTTTTTATTGTTTGTACGTAGTTCATAATCTTATGAGTAGCTTTTGCTA
CACTTTAGAGCCTATTTTGATTTCTTCCTCTAAACTTTAGAGCTCTAAAAACTTTATAGCACTTTAGATCACTTTTGAACTATAA
AGCTCTAATGTGAGGTGGATTAAAATTTAGAGCTAACTTTAGACCATTTGTTTGGAGCTTTGGCTCTAAAATTTAGAGAAGGA
AATCTAAACATGACACATAAATCGAACAATAGCCCGGTTTGTTTTTCCCACTCCTTTCAATGACATGTAAATTGAACAATTAG
CCCTGTTTGTTTTTCCCACTCCTTTCAATCTCCTGGAAAGTAGTCCATAGGATTAAAATATTTGCTATCAGGTAGCTGGAACAT
TGTGAGACCAGTCTATAAGGTAGTTCATAGCAAAAGGTACCCATAAGCTTATGGACTACCCATAAGGAATAAAAAATATTACT
TTTTCTCACTCCTCTCTCTTTCTTTCTTGGTTTTTGCACAGGATTGTAAAGAGAAATAATTTTTTATCTCATATGGGACCCACCT
TATGGACTACTTGGTGGCTGAAACATTGTAGAGACAACGATAGCTATAGTCTACTGACACAAAAGCTGTTCATATGAACTACT
TAGCCCATATACATTGTGGTTGCTCTAAAGGCACATAGGAGAATGCGGGAGGAGCAAAGCTGGAGCAGGAGTCACCTTTCTA
CCAATGCGTCCAAATCATGAAAGAGGGGTCGAAAAAGCTGACAAACAAAACAAAAGTACTCAAGCGTGATGCATCAACAT
CTTCTCTTTCTTCTTGGCCATTGCACACGCACCATGGGACTGGGGAGTAGGGTACCGTATCCATGTAAGATCCCTCTCTACTC

CGTCTCCGACCCATGACTCGTTCGGACGATATATCCCTTTCACCCCATTCCCTTTAGCCCCTTTTGATACGATACCTCAAAGAC
AGGGAGGGAGGGGGAGAGGTCTCCGGTGATCCAGTCTCCATTGCTATCAGTATCGTATTTTCCTCACTCTCCTCTGCTCCATTG
GTAGTCTCCACTCCACTCGTTCTCATCCCCCTCCAAAGTCCTCTCCTTCCTCCCTTTGCCATTGTTTTGGTGAACCACCTTCTC
AACAAATCCAAGCGAGCCAAAGCAATACCGGCACCACCATTGCCATTGCTGACCACACCTCTTCTTAACCTCCATGCA
>SsLRR-RLK150
TGCTGTACGATACCCTACCGTTGGGAGTCGGGAATAATTTGTTCCACCCACTGATTAACTATGCCGCAACCGTACGGAAAATA
CAACGGACAATCATGATCACTCCTAGTGCTACACTGCTACACGTGCCAAACACAAACACAATTATGCATACCCTACCGTTGG
GAGTCTTAACGCTAGACTAGCGCCCGCGCGCAAGCTGTACGGCTGGACTTGCCTGCTTGCTTTTGATATTTCGTGCATGCCTG
GACAATGGCCAGGCGCCAACCTCGACCGGCATTTCCCACCAAAGTTGTACAGGCCAAAATAGTTTCCCATGAAACTTGCG
CATACAGGTTTTTGTTAGAAACAGATTCTACGCTATGCGTCTCGAGCTGCAGCTTCCAACAACTGATTGTGGCATTTGGCAAA
GGTGTTCTTGGTTTGTGAATCCCCTTTGTTGCTCGAACATGTTTTTTTTTCAAATGACTATGTGTCTCGACTCGGCACGTTTT
CCATCGAGAGGGAGGGAATTGTTTGGACAAGTTAACATGAATACAGATAAGATAATTACTAAGGACATGCTTTTATTTCATAA
GTTTTAGAAAAATACTTACAACTTAATCCCATGCCCTGCACTCTTGGAGTCCATTTGAAACACGGGAATTTATAGAAATTGTC
CAAAGTGTTTACATGAACTAGTTCACTTTCACAAAAAAATGTTGAATTTAGTAAGAACATTCATGTTTTGGAGTTTTTGTCAC
TTTTAGACTACTAGCTTTTGGACTTTTCAAAAGTCAAACAACTCTCACAAACCAAAAGTCCAAAAATTCATTCTAATGAGCT
TGTGGTTTTTGCCTTCCAGAAGCTTTTTGGTTATTATTAAAAAATCCAAAAGGAGGTCCAAAAGTTCCTAGAAAGCCCAAGC
AAATAGATCCTAAAACTCTTGTAAGTTATGCTATCACTATAGCTGTAGCAAGTGCAGCTGAACAGAAAACTTATACTAGTGCT
AACCACTCTGCCTTGTTCGTTTCGCTAAAATTTGGCTTTATGCTACTTATGCTGATTTGTTGTGAGAAGAAAATATTGTTCCTT
TACTAACAAGTACCGCTGAAGTAGTGCTAAAAAACATGGCTCAAAATATTTAGGACCTAAAACTACTACTTTCCTATAAAACT
ATATACTAGTACTTTCTTATTCCTATAATACGAGAACATGGCAATCATCCAAGAGGAGTCCTCAAACTAGTTACCCATTTGTTTT
TTTACTGTCCTATACATTGATTGTTAATTTTAACTTTGTATTGAAAGTCTCGTTTGGATGGGCTTCTTCGGGCTCTCACCCGGCC
TGCATTGGAGGAGTCAGAGAGAGAAAAACGGTTCTTCTGCATAGTGCTAGTTCAGCGTGGGGTTGGAGAGGAGGCAAGAC
ACACGAAACTCTAAGGAACACCCCGCGGAGTGTTGCGGCTGCCATCAGAGAAACTGCGGTGATGTTCATTCAGCTTATAATC
CGTATTTTTCAATTTATTTTTTAGTTAAAACAGTGTTTTTCTCTCACACCAAATCAGTAAACAGTGTTTTTTAGTCGACTTATTA
GCACTGAACGGGGCTGCGCCTGCAGTGCCGGGCGCCGCACGGCCCTTGAGGCCAGGGCCCTGTTCGGCAGGCTGTTTTTTT
TGGCTTATAAGCCGGCTATGGCTGATTTGCTGTGAGAAAAAAAAACACTGTTCCACGGCTGATAAGCCGTGCTGATAAGCTCA
GTCGAACAGGGGCAGGACGCCAGGTCCACGCCGGACTACACGGCGCGGCGGACTAGAATAAACTGAACCTAACTTTGTTT
GGAGGATTCTGGTAGCAAACCACGCTAAAAAAACATCAGAAACGAGTGTGAAATCCGCCCCGTTCTCGCGATTAGCGACCG
CCGTTTGCGCTTGGGTTTGGCAGTGGCCAGTGAGTGGGGTGCAGTCATCAGTCAAACCATCCCCACCACCACACCTCGACA
CCAGCGGCCAGCCCCGTCC
>SsLRR-RLK332
CTCGAAATATACTCTTGTCTCGTCGATCGGCGCGGTGCCCCCCACGGCACGACGATCGACGAGGACTTGAACCTTCTTGTTT
GTTTGTTTGGGTTTTGTTGCTGCATCGGCATTTGACGCACGATGCGGTGCGTGCGTCATACGGCCAAAAGGCGGCCGCCATG
GCCGAGCGCCCGAGCTGTCGTGTAGTGTTGACAAACTTGCAGCGCAGTAAGGCCAAAGCACACAGGGCCCTCTTTCTGTTT
GTCCATGCGGTGCCAAGGTTAAAATTAAAAGCGAGCAGCCTGCTCCCGCCGTAGCCGTCCCAGTATAGAAAGAGTGCAATT
GCCTGGTTTTCAAATAAGTCAATATCGATTTTAAATTTGACTAATAAGTTTATAAAATCAAATATTTATGTTCCTAAATAGATTTA
ATACGGAAATATATATGTAATGTGATTAACCTAGGGCCTGATTGGTTGTGTGCCAAAACTTGCCCAACCAAAATGAAGGTATG
CCTACAAAATTTGGGTATTGATTGGTTTATTACTTAAAAAATTGGCACACCAATTTGTATGGGCCAAAGGCCCCGTTCGGCTG
TGCTGATAAGTTGGCTGAAAAACATCGTTGAGTGATTTGTTATAAGAGAAAAACACTGTTTCGGCTAAAAAAACAAGCTGA
AAAGTACGGATTATAAGCCGGATGAACATGACCAAAATCATGGCAAAGCAATTAGAAACTTTCTCAATCTCATAAGAGGCAA
AAGTGCTCACTACTATGTCATGACACTCGACCAAATGTAATCCTATATTTTTTACGGTAAGCGCATGTTTTGGCTTGTCATGAT
TTTGATCGGACAAGTTTTGACACCCAATCAATGCCCTAATTGCTCATTTTAGTTGAGGAGTAGTAGCACTGTGGGCAGCTGGC
TAGCAAACCAGCTGTCCACTCCCGGCGGTCCCCGCCCTCCGCCGTACGCACTGGCACTGTGCTAATGCACGCCGAGGCCCC
GAGAGTGTGGACCAACTCCAACCGGAACCGGACCAGGCCATCTCTTTTTTACCCCAAACTCTTACCCTGATTCTGATCGTTG
GTGCGGATGCGGACTCAGACGGACGGGACGGCCCAAAAACGTGAGTGTCTCCGGGCGTGGACAGTGGCTTGTGCTTTCCA
CTCTCGGCTCTTGCGGCGCGACAAAGGACTCGCGGTCGCTACGAGTTTCAAACGCCAAACGGCGTCACGACATTGGGCCTC
TCCTCCTGTGTTGGTATTTTGCTGATAAAATATACTCCCAAATTCGAGTCAAAGCTGCTGAGGCACCCCGAGCGGCATGTGCC
ACTGCCAGATTTCGAACGGGGACGCCGAACCAGATGCACCGCCGGCCCCACCGCGCAGCGTCACCCGCACAGCCCTCACT
CCACTCCACCTGCGGGAACCGGAGGAAACCTCCAAGGCCAAGGCAGCAGCAGCACCCATTTCACCATTATTAGTACTTCCT
ACTACTACTGTAGAAGCTTGGGGATTAGATTAGAGTAATCATGGGGATCAACGATAACCCTTGAGATTTCCCACATGCAAACA
CGCTCAATTAACTTTACTTTCCCAAAAGTCGCCCTCCCGACCAACATATCAAATGCTTCGAAATCGAATCCTCGCCCACCTCA
CCACTAGGCCTCCCTCCAGCGCGGCTCCCAACTGCAGGGCATCTGAGCATTCACTTCAAATCTCTCTCCCCAAACTACCCCT
ACCTTCCGCCATAAACCGTGATCCTCCCGGCGGGTCTTCCCGTCATTTCGCCGCTAAATCTCAGTCACCTAAAACCCACACCC
CACGCCGGCACGCCGCCCCTGTCGCTCCCCTCCCTGGCCGCTTCCTCCCTTCTCTTCACCCCTCTTCCCTTCTCTGCAACTGC
GACCCTGCAGCCTCGCCTCACGGAGTCCCAGGGAATGCCACCCCTGTGATTCCGTGATTCCTCACTCACTCACCAGCACCAC
CACCATCCGTTCGCGCTCGCTCCGGGCA
>SsLRR-RLK379
TCCAGGTCCCTAAACTTTTAAGGTGATCACCGCAGGTCCTGGCCGGCGGTGTCATCTCAGTCCCTGAACTTTCAAGGTGATA
TCACTGCAGGTCCCTAAACTTGGCAAGCGGTATCGTTCCTGTCCAAATGTGGTCTAACCTACTCTTTAAGTTGGCGTGGCAC
GCTGAGTGTATGTTAGACGTGGATCCAACATGTGTCAATTAACCAATCATTATTTATATAATATTTATCATAAAAATATAAATTAT
ATAAATAATTTACATCAGGAAAAATAAATTAAGTTATTAAAATAATTTACAACAACAAATATTAGGTTTAAACTAAAATAGAAA
AAATACAAAATTAATTTAATATTTATGTATCATTAATAGTATTAAAATTCCTATAGTATTAAAATTGTAATAAAATACTTCTAATTT
TAGTATTATTTTAAAAGTTTTAATAGTAGTATTATAGAAGTAACTTTCTCTATTATTAATACTACGTATTATAGTATTAGAACTTTT
AATAATAGTATTATAGTATTAAAAGTTTTAATAATAATGTTATAGTATTAATAACAGAGAAAATTTACTTCTATAATACTACTATTA
AAACTTTTTAATAGAGAAAACTTTTAATAATATGGGTAACTTTTAATATTATTATATTGGGACAGGGATTACATAAGTTGTCAAG
TTTAGGGACCTACGGTGATCATCTTGAAAGTTCAGGGACCGGGATGACACCGCCAGCCAAGTTTAAGGACCTGCGGCGATC
ACCGTAAAAGTTGAGGGACCTGGATGACACCGTCTGCCAAGTTTGAGGACCCACATATATCACTTTAGAAGTTCAGGGACCT
GGATGACACCGCTGGCCAAGTTCTGGGACCAGGGGTGAATTTTGCTCAAAAAGAATAATAATATTCCCTCCATTCTGAATAA
CATTTTATTTTGGTTTTCCCCTAAGGCAAACTTCTCTAGAAAAATATATTCATATATCTATAAGTTCAAATAATTTCTCGATCAAA
ATATATTGCTTGAAGAATCTAAAGAAATAGTTTTTGATGTTCTAATTGTTGGTGGATTTTTATACAAACTTGGTCGAATATAGTA
CAAAGTCACTATTGGAGACTTAAATGTTCTTGTCGGTTTAACACCCACCGACAGGGAAATTACTAATATTTCTGTCGGGAACC
TAGAGCCGATAGAGTAATTCGAAAACCGATAGGGTGAATACATTTCTCTGTCGAGTTTTTGGTCAACCGCTAGAAAAATGTAT
ATTTTTCTATGTGTTTTGGTGCCACCCACAGAAAAAAATCAATTGGAAGAGCACCGGGAAACGAAACACGCAAAACCGGGCT
TGGGCCGCGTTGACCTTTCAGCCGGCCTAGAAGCGGGCGGGTTGTGCCACCGATAGGCAAATTTTATTTTTCTGTTATTTTAT
TTCTATCAATAATGATATATCGACAGAAAAATTATTTTTTTAATTTTCCTATCGGTTTTGTGTATTTTTCTGTCGGTTTTTCACCG
ATAGCAAAATTTTAGTAGTGAGTCAAAGCAAATGGAACAGAAGGTGTGCTAGATAAAAATGGAGTGCCAGTGCCAGCGCCG
GTGAAAACAACTGATAACCGTTGCAGAATGAATACGTTTTCCCATTCTGTCGAGAATTAAAAGCACTTTCATTACCCACCTGG
CCTTCCTCCATCTTCTGGATTGTAAACTCTAGAATCCTACTCTGTACTGGCAGTTTTCCCACATGACAAGATGCCAACATAGC
CTTTGCCTGGTGCCTGCCCTCCGGCAGATCTATAACCTTCCCGCTAGCCTCTTCTTGACCCTCCCTCTCGCGTTGCATTGGCG
GCCTCTGCCTCTGCCTCTCTTCCGCTTCCACACACCACTGACTGACCCAACATACGTACACTAGCTAGCTAGGCATCGGCAG

CTTCACAAACACTACCGAAGAAGCAGCAGCCAGCAAGTAGTGTGTCAGCCAGCCGCTAGCAGCTAGCCTAGCTAAT
>SsLRR-RLK389
TGAAATCATGGATAGAGTAGCTATAATTCATATCAATGTGGGGTGGGGGGAGGTGAGGTTGGATCCTCAACCTGTTCCGCCTC
CAGTAGCCACTGGCATGAAGCTCTGATGAGACTCTACACAACCACCCCACGCGCACGCCTTACCAATCGACTTCCTCTACAT
CACATTCGTCGTCCTTGCATCCACCTATGCATCCCACTTTCGCCAACATTGTCTACTCTGAGCTCAAGGAAGCTTGGGAGGCT
CGCTAGACCCCCAAAACAACAACTCCCACACCCTGCTCCTCGCCAGAGACACTTAGGGTATATCTGGATTCTTTACCAAACC
TTATCCAACTTTGCTAAGCCAGAATATGTGTTTGGTAGTGGATGAAAAATCATCAAAGTTTGAAATCTCCGGCTATAGCTGCA
GCTATAGCCGGAGATAGCTGCTGGGCAGGATTAAAGCTAGGATAATCAACTTTTTGCGCTAATAAATGGTAGACGCTAATAGC
CGGAGATATCTGGCGATAGCCGCTAAATGGGCCATACTTTCGCGGCAGCTAAAATTTTACCGGGCCTAGGAAGTCCGCCAGC
CGGCGGACCGAAGACCTTTGCGCAGCCAGTGGAGCCCCCAAGCGCGAGCGGCCAGTGGAGCCCCCAAGTGCGAGCGGCC
GGTGCAGCACAGCGGCGGCAGAGGCTAGCAGCGACGCGGTCCCGGAGCCCCCAAGCGATGGCGACGGCGGGGGTTCTGCT
GCAGTCCTGTAGCACGAGCGGCCACGGATCTGCTGCAGCACCGTGGCAGCGGCAGCTGCGACGTTCTGCTGGAGCACGGC
GGTGGCTGCAGGTCTGCTCGAGTTCTCTTCTTCTCCAACTCCAAGCTACAGCACCGAATGTTCTATTTGACGTTAAACTAGA
GTGTGTTGTACTGCTGTTCGTATGTCCTCTGTCAATGAATGGTTAAACTGTTAACCTGTGTGCCAATATTCTGTGTTTTTTTCCT
GATCTTTAAAAAAACAGCTAAATGTTTTAGCTGCAGCTATCTTCGGCTATAGCTTTACTTAGCTGTTGAGGAGGGCAGCAGCT
AAGTTGCTTAGCCGGAGATCTAAAACCTTGAAAATCATGCTTTGCTAGGCTAGCTTGCCTCTCCAAGCAAGGAAATCCTTGC
CCAGCTAGGAGAGCCAGATTGGCTCTCTCACGCCGGCAAGGCAAGCGGTAAGCGCATCATGTCCTGTGCGGTAGCAGACCT
GCTCCTGTGACACCCATGTATGGCTGAGTGACCATCGGGCCAGATGAGCACTTGGTGTTATAAAAAATCAAAGTGCTTGCAT
GCGGTGCTAGACAAGCTTTCACACTGAGCAAAACAACCAAACATAGTTTTATTTGGACTGGCCTTGCTTGGCCTCACCTTGC
CTGCTTGCCTGGCTAGGTAAAATTGGGCAAAACATCCATGCATCAACAAGATAAAGTTGTGGTGTTACGAGATCATCAAAAA
GAATAATACTATTCCCTCCATTCTAAATAACATTTTATTTTGGTTTTCCCCTAAGGCAAACTTCTCTAGAAAAATATATTCATATC
TATAAGTTCAAATAAATTCTCTATCAAAATATATTGCATGAAGAATGTAATGAAATAATTTTTGATGTTCTAGTTCTTGGTTGAT
TTTTACACAAACTTGGTCAAATTTAGGACAAAGTCAAAGCAAATGGAACAGAAGGTGTACTAGATAAAAAAAATGGAGTGC
CAGTGCCCGGCTGCCCGCGTCAGTGAGAGCAAAGGATTTCCCATTGCAGAATTGAATACCGAATTGAATACAGTGTTCTCCC
ATTTTGTCGGGAATTAGAAGCAGTTTCATTATCCACGTGGCCTCCCTCCATGGCTCCATCTTCTGGATTCCAAACTCTAGAATC
CAACTCTGTCCTGGCAGTTTTCCCACATGACAAGATGCCAACATAGCCTTGCCTGGTGCCTGCCCTCCGGCAGATCTATAACC
TTCCCAGCCTCTTCATCCTCTTC
>SsLRR-RLK276
CGATGTCTTTCGTTACGCAAAAGCAAACACACAACCAGAGAGGGAGAAGAAGCCGCTTAGGGTTCTAGCGAGGAGCTCGT
CGGAGACGGCGAGTACGAGGTCCCGGAGAGGGAGAAGAAGTCAACTCTGACGGGCTTAAATGGAATGGGCCCTAGGCAAC
CAGGCGGTTTGGGGCTCTGGCGGCGACGGAGGTGGCCAGACCAGGGAAGGGCGGGGGCGCCATGGCTGGAGGAAGGTCG
CCGCGGCGGGAGCTCGCCACTGCAGGAGGGGGGAGGGGAAGAAGGCAGCTGCCACGGGTGTGCTAGGGTTCATCGGAGC
TCTGACAAACCTTAACGCGCCGCGGCTGGGTGGAGGTTGGTGGTGGAGGCGAAGACGAGGAGGAAGGTGGGGTCGAAGA
CGAGCGCGGGTACTCGATGGCGAGGGACGAGGCGGCGGAGGCGTGGGGAGAGAGAAAGGGGCGGGAGAAGACGGACGC
GAGCTTGGAGACAGGTCACGCGTTCCCAGAACGCGTCCTCCCTTTCGTTACCACTTGTGTATCGTGTCATCAAGCGAAGCAA
TCTAGGACTCCGGTAGTTAGTCTGCATCAGTGCATGTACGTTTGCTGATGTGCATTTCGTTTCGTTTCAGAGCTGTTGGCGCT
GGCCAGAGCCGTCCAAAGTTCCGTAGCGGTTACACGGAAAACGGCCAGCGTCCCTTCTCTTCTCCGTGATCTGCGTTTGAAT
TGAACTGCCATGCCAAGCAGCTAGCTGCTGCGTGCGTGCTTTTCTTGTTGCTGTCCCCGCTAAGACTATCCGCACTCGTGGTA
CTCTAAATTCATCCTCTAAAAAGAATATTCTATCCTGGTCATCAAGATTCTTTCCTCTATACACGTTTCTTCCGCACTCATCAAT
CCTCTATATATCATACTCTATACCATGGGGCCCACTTGTCATTCTCTCACCTCTCTGTCCTCTTTTCCTCCCTCTTCTCTCTCCTC
AGTCTTATCCACTCCCCACCGCTGGCGCTCGTGCCTCCTCCCCGATGCTGCTCGTGCCATGGCGAACGGCGGCCCCGATGCC
CGCGGCCGGCTCATCCCTGGCGCGCCCGGCCATGGCGCAAGCCACCGAGCTCGCCCCCTCCCCGGCCATGGCGAGGCGGAG
GTCGGCCGCGCGCCCGGCCATGGCGCGCCCCGGCCATGGCGCAAGCCGTCGAGCTCGCGCCCCCGAGGGTTGAGCTCGGC
GCTCCGGCGGCGGCCTGGGCGGCGCGGCCTGCCTTGGCGCGGAGCAGGCGGACGCGGAGCTAGGCGCCGGAGCTCGCGCG
GCGGCGGCCCTCTTCTCCCCCTCCGGCGCCGTTTCCAAGATCCGTGCTAGATCCGCCTCTCCCCCAAGTCCGGCGAACATGG
AGGCAGTGGCGCCACCGCGACGCCGTCGGCGGCATCGGGCTCGAGGGGCCAGTTCCTCTGCTCGGGGGGCCGAGCTCCTC
TCCTCGGGGGGCCACGCCGGAGGCCGAGCTCCTCTGCTCGGGGAGGGCCGCCGTCGGTGCTCGGGGAGGGCCGCCGCCGC
GCCGCCGTCGGTCCTCGGGAGGGGAAGATGAGGACGAGCAGGCTCGGAGAGGACGACGCCGGGCCCCGCCTGACACATAC
CGGAACCTCCGTACTTCCGCCACAGACACCGGACGCTCGCCCGTCCGCTTCGATTGCGGACGGGGTAGCGTACCCCGCTGC
GGTGTTTTTTACTGGAAACGCGTCCTCCAGTACGCGATCCAGTACGCCGCTGCGGATAGTCTAACGAGCCTCTCCGTACGCT
AGGCCCAAAAAGCACGCTAAACTCCACGTACCACCCACGTGCCGCGGCGACCACCCTCTGACCCCCGTCCCCTTCCGACCC
GATCTCCTCTCGCGTCTCGCATCTCGGCGTGCCACCCCCACCGCAGCCCACTCCACTCCACCGCTTCACTGAAACCAACCAC
CACGGCCACGGCACCACCCCTCCCTAAATCCTGAGCCCCAAAACCCCCGTACG
>SsLRR-RLK300
GTTCCTATGCGGTATCCGCAGTAACACTTGTTGGCACCGTGGAACGTGGATCCGCAGCCGTGCACCGACCGTTCCTGTGTGC
ATGGGCGCCAGCGCCATGAATTTGCCGCAGGCCGGCACCTCCTCTCACCGGAGCCGTGCACTGCCAGTCTGGCTGCAGCAG
CAACACAGCTCAACGGCTGTTGCTTTGCTTCACCGAGTTCACCAGGATACAAGGGACATCTTGCGTTTGCATGGAAACTACG
GTCTACGGAATACAACCATACAGTGACGATGTCGCCGTCGGTGCCGTACATGTATAAGTATAATGTATGTAAGCCCTGCGTTT
GGCTGCACTGCAGTGACATTGCCTGGCCGTGGCGTATGCATTTTGGCGTGGCGCGTGCGCAACAACTAGGAGCTGCGAGAG
ACCTGTCTGCGCCCGTGGATCCGCGGCCCACCGGTCGGCGGGGTGAACGTGACTTGGTTGCGCCATTTTTGGGCCCTGTGG
GCGAACGTGCAGCCCAGATCGCGCCCATTTCTTCTTCATTTACCGGGCGTCATGCGAAGGTTAGGACAACTCACCTGGGCCC
CGCAGCCTGATTGAATCCGTGGGTCCTGTCGCTACTGCAGACCGGGGTCCCTGAGTGTCAGGCACAGTAAAACTCGCATTG
ATTTTGGGCGTGTACCACGTCGTCCTCGGTTATTATTAAAGAAAAGGTGGCAAGTAGCGTCCTGCACCGCGCGAGGGCGAG
ACCCGCAGGTATCAAGGGCCAACGGGTCCTTTTCAAACATTCCCCCGCCGTGGCCCGTGCATTCGTCGCTTCCGGCATAAGG
CTACTGTTCGGCTATATTTCCAGCCGTGTTTTTGCACGAATCCTACATGGCTAAATGTTTGCCCAACTGTTCCTATAAAGAAAT
CAGGGACATATTTTGTGGAGGTCCTCATAATACGCACGATGTCTTTCGTTACGCAAAAGCAAACACACGACCGTTGCGCTCC
GCATGCTCGCATTCGATCCCGCACCAGAGAGGGAGAAGAAGCCGCCTAGGGTTCTGGCGAGGAGCTCGTCGGAGACGGCG
AGTAGGAGGTCGCGGAGAGGGAGAAGAAGCCAACTCCGACGAGCTTAAATGGAAGGGGCCCTAGGGAGATAGGGCAACC
AGGCGGTTTGGGGCTCCGGTGGCAACGGAGGTGGCCGGACCAGGGAAGAGCGGGGGCGCCATGGCCGGAGGGAGGTCGC
CGCGGCGGGAGCTCGCCGCCGCAGGAGGGGGGAGGGGAAGAAGGCGGCCGCCACGGGGATGCAAGGGTTCATCGGAGCT
CTAACAAACCCTAACGCGCCACGGCTGGGTGGAGGTTGGTGGTGGAGGCGAAGACGAGGAGGAAGGTGGGTTCGAAGAC
GAGCGCGGGGTTCGAAGACGGCGAGGGACGGGGCGGTGGAGGTGGGGCGTGGGGAGCGAGAGAGAAGAAGGATGCGAG
CTTGGAGACAGGTCACGCGTTCCCCTCCCTTTCGTTACCACTTGTGTATCTCGTCATCAAGCGAAGCAATCTAGGACTCCGGT
AGTTAGTCTGCATCAGTGCATGTACGTTTGCTGATCTGTATTTCGTTTCGTTTCAGAGCTGTTGGCGTTGGCCAGAGCCTTCC
AAAGTTCCGTAGCGGTTACACGGAAAACGGCCAGCGTCCCTTCTCTTGTCCGTGATCTGCGTTTGAATTGAACTGCCATGCC
AAGCAGCTAGCTGCTGCGTGCGTGCTTTTGTTGTTGCTGTCCCCGCTAATGAGCTTCTCCGTACGCTAGGCCCAAAAAGCAC
GCTAAACTCCACGTACCACCCACGTGCCGCGGCGACCACCCTCTGACCCCCGTCCCCTTCCGACCCGATCTCCTCTCGCGTC
TCGCATCTCGGCGTGCCACCCCCACCGCAGCCCACTCCACTCCACCGCTTCACTGAAACCAACCACCACGGCCACGGCACC
ACCCCTCCCTAAATCCCGAGCCCCAAAACCCCCGTACGCGCCCGCCGCGCC
>SsLRR-RLK216

TTTCTTACTATTATTGTTCAGTAAGACAGTAACAGTAGGGAAATTATTGATCTGCTATGTGCATTTGGTTAAATCAACTGATCA
AAACGGTTATGCTGTCCATTACCATGCATCGACCGATCTCAGAGTCGCCCACAGTGTGAGTCTTTGATGGTCCCAAAATAACA
GGATTAGTCCTCACAAAGAATGAAGGACGGAAGGAAAGAAAAAGGTTAAGTTAAAGGCAGTTCGTCAAAGTGAAGGCCAT
GCTCTCTCTCTGTCTGTCTCACTTGCCAGGGCATTGTGATGGCTGGATCTGAGACAATAAACCCTGTGGCCACTATGGCATTG
CATGTACTAGCCCTCGTTTAGATCGAAGATTTTTTCAACCTGATGAATAGTACCACTTTCGTCTTATTTGACAAATATTGTCCA
ATCGTGGACCAACTAGGCTCAAAAGATTCATCTCGTGATTTTGAACTAAACTGTGTAATTAGATATTTTTTTACCTACATTTA
GTACTCTATGCAAACAGCTAAAAATTGATGTGATGGAGAGAGTAAAAAAACTTGGAATTTTAAGGTGATCTAAACAAGGGGC
TAGTTGCTATTTCAGCCAGCACAAGCTCATTTCCAGCACAAGCTCAGGGACAAGAGCATACAAACATAGCTGCCCCGAGGC
CAGGCGTACAGGTAGGCAGATGATGGGTGTAGCTTGCTTGGGATCGTTGATAGCATCATCGGACATACTCCCTCTCTCCATAA
AAACATATAATTCTCGTTTTTTAGAAGTCAAACAATTTCAACTTTAACTAAATTTATATAAAAAATACTAATGTTTATGATACAA
AATAAGTATCATTAGGTTAGTTTAGAATATATTTTATAATAAATTTATTTATAGACATAAATGTTAATACTATTTGCTATAAATTT
AGTCAAACTTGAGTTAGTTTAACTGACACGCATCCTATAATTACATCTTTTCTCGGAAGTATCTAATGTTGGTCCACGCGGCCC
TGCTGCCGAGTTGCTGTGGCACTGCTTTCCCTGCCAGCATGCATTGAGCAGGAGAGGCCTTCAATCAGAGTCAGACGATGC
ATGCCATGGGCCATGGCAATATGATGGCCTCACACAGATACAGCTGTGGGCCTTACTTAATGTAGGCCTCGTTTAGATGCTAA
AAAAATTTCAATCCGATAAATAGTACCATTTTCGTCTTATTTGACAAATATTATCTAATCGTGGACCAATTAGGTTCAAAAGATT
CATCTCGTGATTTCCAACTAAACTGTGTAATTAGTTATTATTTTTACCTATATTTACCACTTCATGCATGGATCTAAAGATTGATG
TGATGGAGAGAGCGAAAAAATTCTAAAGATGGATGTGATGGAGAGAGCGAAAAAATTCTAACTTTTCATGTGAACCCACGT
GCAAGTAGTACTGGTATGGGACGAGGCGGGCACAGAAAGAAATTAATGGCAGCACGCAGGCTTTTTATAGGCTCGAGGAGG
GTAAGGTGATTACTACCGTCTTACCCATTGACTGTCATCAGTGTCTGGACGCGGCCCTAATTCCGAATCTCTCTCCCCTCTCCT
AGCTTTTTGTGTGGGTGCTTGCTAATCTCAGCCACACACAGACCATAGCTGTTGGTGGGCGGATGAGGCTGAGCTGCTTCTG
CAGTGTGCAGAGTGCGACTGCGGACAGAGACGACGCTGCTGCATTGGCCTGGCTGTTGCTCCAATCAAAAAAAATGGTCGG
TCATGGCTATCCGTCGTCAGCCCGTGACCGTGAGCAGCATTTAAAGTCTTTCCTTTCCTTCATGCCTCTCCATCGACTCGCGG
TCGCGGGGCGTTGCCGTCGCCGGCCTCGTTCGCTTTCCTTTCCAGCTCCCTCTACAACACCGGCAATCGCCAATCTTAAGGC
AATGTCGCCGCCCGCCCATTGGCTTAAGATCGCTAAAACCTAAACCTAAACCCCCACCCACCCCACTCTGACCCACCAGCCA
CCCTCACCA
>SsLRR-RLK241
CATTAGGAGATCCACATGGTTGATGGAAGCCTCCATCATGGGCCATAGTTGATCCTCCATCTCCACAGTCACTGCTCAAGATT
ATGTATAAATGGAACTTCTCATAAAGAAGGAAAAAAAATAATAAATAACAGGGGGTATCTCTGACTGACTGATTAACAAACAA
TCAAACAAACAAAGAGTCAGAGGCCACAGTGCATGAACATGGGTGCCCTCTGGTTCATGTGACATGTGAGTGGTGAGTCAA
ATGGACTACTCCCTAGTCACTACACATGTAAGTTTCTCGTGCATCTTCTTTTTCCTTTTGTAAAATGAAATGTTCTCTTTGTCTT
ATTATTCTTAGCCAGTAAATAGAATATCAGATGGCCTGCATTCAATGGCCATCTTTCTTACTGTTATTGTTCAGTAAGACAGTA
ACAGTAGGGAAATTATTGATCTGCTATGTGCATTTGGTTAAATCAACTGATTAAAACGGTTATGCTGTCCATTACCATGCATCG
ACTGATCTCAGAGTCGCCCACAGTGTGAGTCTTTGATGGTCCCAAAATAACAGGATTAGTCCTCACAAAGAATGAAGGACG
GAAGGAAAGAAAAAGGTTAAGTTAAAGGCAGTTCGTCAAAGTGAAGGCCATGCTCTCTCTCTATCTGTCTCACTTGCCAGG
GCATTGTGAATTGTGATGGCTGGATCTGAGACAATAAACCCTGTGGCCACTATGGCATTGCATGTACTAGTTGCTATTTCAGC
CAGCACAAGCTCAGGGACAAGAGCATACAAACATAGCTGCCCCGAGGCCAGGCGTACAGGTAGGCAGATGATGGGTGTAG
CTTGCTTGTGATCGTTGATAGCATCATCGGACATATCTACATGGTGGTCCAGGCGGCCCTGCTGCCGAGTTGCTGTGGTACTG
CTTTCCCTGCCTGCATGCATTGAGCAGGAGAGGTCTTCAATCAGAGTCAGACGATGCATGCCATGGGCCATGGCAATATGAT
GGCCTCACACAGATACAGCTGTGGGCCTTACTTAATGCTAGTACTATCCGGCTGAGCTTATCACCCGTAGAACAGCGTTTTTT
TCTCACAGTAAATCAGTCACAGCCGACTTATAAGCCAAAAAACAGCCTGCCGAACAGGGCCTATATGTCATGAAGCAACCC
ACATGCAAGCAGTACTGGTATGGGACGAGGCGGGCACATAAAGAAATTAATGGCAGCACGCAGGCTTTTGATAGGCTCGAG
GAGGGTAAGTAAGGTGATTACTACCGTCTTACCCATTGACTGTCATCAGTGTCTGCACGCGGCCCTAATTCCGAATCTCTCTC
CCCGGCCTCGTTTAGATGCTGAAAAAATTTCAACCCGATAAATAGTACCACTTTCGTCTTATTTGACAAATATTATCCAATCGT
GGACCAACTAGGCTCAAAAGATTCATCTCGTGATTTCCAACTAAACTGTGTAATTAGTTATTTTTTTTACCTACATTTAATACT
CCATGCAAGCGACTAAAAATTGATGTGATGGAGAGAGAGTGAAAAAACTTGGAATTTTGATGTCATCTAAACGAGGCCGCT
TTTTGTGTGGGTGCTTGCTAATCTCAGCCACACCACACCGGCTAATCCTCATCTGATTGACAGACCATAGCTGTTGGTGGGCG
GATGAGGCTGAGCTGCTTCTGCAGTCTGCAGAGTGCGACTGCGGACAGAGACGACGCTGCTGCATTGGCCTGGCTGTTGCT
CCAATCAAAAAAAATGGTCGGTCATGGCTATCCGTCGTCAGCCCGTGACCGTGAGCAGCATTTAAAGTCTTTCCTTTCCTTC
ATGCCTCTCCATCGGCTCGCGGTCGCGGGGCGTTGCCGTCGCCGGCCTCGTTCGCTTTCCTTTCCAGCTCCCTCTACAACACC
GGCAATCGCCAATCTTAAGGCAATGTCGCCGCCCGCCCATTGGCTTAAGATCGCTAAAACCTAAACCCCCACCCACCCCACT
CTGACCCACCAGCCACCCTCACC
>SsLRR-RLK242
TGGACGATCCGACTAAGCCGCGCGCCTCGTGTTATTCGCGCCCGCTCGACGCCCTCCCCCATTCCCATCCCGTCTCTTTTTCG
CGGCTCCTCCACTCTCTGCTCTGCTCGTGCGCTGCCGCCCCTTCCTCCATCTTCGACCTTGACACCGTGACGGCGGCGGCTT
CTGGCGAGCGTGTCCTCCCTCCCTTTCTCCCTCCCTCCCTCCATCCCTTCGTGCCCCCATGACCTCCATGCCACCGAGAGAAG
AGGGCAACGTCGGAGTCTTTCCAGCGAGCCACCCCTCCCTCCTTCCCTGCCTCCCTCCCTCGCTCTGCGACATCCATGCCAC
TAGAGAGGTGTGTGACGGAGGTGGCTTCCGACGAGGTAGGTAGGTCTTTTTCTGAATCTGAGCCCTCCTCCCTTCTCTCTTT
GGATCTAAGGGATTGCGGCGGTGGCTAGGATTCTGGCGAGCTCTCGGGGGTTAGATCTCGCCGTGCTGCAACGGTTTTATTT
GCATGTTACAGTGTCTTTCAGTTGATGTTGCAGTAGTACTTTTTTTAGATGTGTGCAGTATGTTTTAGTTGTTTCAATCTCATGT
TGCAGTAGTTGTTCCATGTTGTTGCAGCTGTTTGTTCGATGTTGTTGCAATAATATGTTCATAGTGTTTCAGCTGCTCCAGCCT
AATGTTTCAGCTGTTGTTCTTTGTTGCTGCAATAATATGTTCACGGTGTTTCAACTTCTTCATACCTAATGCTACAGTGTTGTCC
CTTTTTGTTGTTGCAATATATATGTTCATGGTGTTTCAACTGCTTCAACCAATTTGTTGTAGCAGTTTGATCAGTGTTGTTGCA
ATAATACGTTCATGGTGTTTTAGCTGCTTCAGCCTAATGTTTTAGCTGTTGTTCTTTTGTTGTTGCAACAATATGTTCATGGTGT
TTCAACTGTTTCAACCTAATGTTACATCCGTTTGTTTCTTGTTGTTGCAATATATATGTTCATGGTGTTTCAGCGGGGGGCGATT
TGGGGGCGTGCGGTCTTGTGCGGCATAGAGGCTAGCGGGGCTCTTCGTAGGCAGCGGGGCTCTTCGTTCGACGAAGACGCA
GCAGCGGCGGTTCGAGCGGGCGCACAATCTTAGGACGGCGACATCGGTGCGCCCACAATCGGTGAAGAGAAGCGAGTGGC
TGGGAGCGGGGGGTTAGGCGGGGAAGAAGAAGTGAAGCGCGGTTGGTGTGGGCGGATTTTGCGGACGGGTTTGGCTCGGT
GCGACGCGCCCTGCGGCACCGTCCGATGGATCAGTTTGCGGGTATATTGGGCTGGGCCGGTTGCGGGAGCAGCGGTGCTGG
GCCAGATGGGGAAATATACAAGGCCTTTGTCTCTCCGTCTGGGGGAAGAAAAAGGAAGGAGCGAGCAATCCACATGCAAGT
AGTACTGGTATGGGACGAGGCGGGCACAGAAAGAAATTAATGGCAGCACGCAGGCTTTTGATAGGCTCGAGGAGGTAAGTA
AGGTGATTACTACCGTCTTACCCATTGACTGTCATCAGTGTCTGCACGCGGCCCTAATTCCGAATCTCTCTCCCCTCTCCTAGC
TTTCTGTGTGCGTGCTTGCTAATCTCAGCCACACCACACCGGCTAATCCTCATCTGATTGACAGACCATAGCTGTTGGTGGGC
GGATGAGGCTAAGCTGCTTCTGCAGTCTGCAGAGTGCGACTGCGGACAGAGACGACGCTGCTGCATTGGCCTGGCTGTTGC
TCCAATCAAAAAAAATGGTCGGTCATGGCTATCCGTCGTCAGCCCGTGACCGTGAGCAGCATTTAAAGTCTTTCCTTTCCTTC
ATGCCTCTCCATCGGCTCGCGGTCGCGGGGCGTTGCCGTCGCCGGCCTCGTTCGCTTTCCTTTCCAGCTCCCTCTACAACACC
GGCAATCGCCAATCTTAATAAGGCAATGTCGCCGCCCGCCCATTGGCTTAAAACCTAAACCCCCACCCACCCCACTCTGACC
CACCAGCCACCCTCACCA
>SsLRR-RLK333
TCAGTCAAGATACAAGACTGCTTTGATTTACGAGTGATTCTCTCTTTTCTTTTTGAGAAACAGAGAACTTCTATTGATCAATC
ACAAGGTGTAGCCCTGTTCGCTTGAACTACTTCAACGATACTTTTCAGCGAACGAATAGTGTTTTCCTTTCACAACAAATCA

GTATAAACTAAATTTCAGCGACTACCAAATGCTGAATCAACACAAAAGGGGGACATTTTAATGAGGTGCATGGCCAGGCAAT
TTGCAAATCTGTACAAATGGTCTGTCATCACATTATGGGAGCCATGCTGTCTCTGAATATTACTGCTTAATAATGCATTCACAC
CATTATATTCAGCCTTCTCATGAACCGAATGAACAAAACAAATCAAACCAAACCAGACCATTCCTCATGTTGTTTGTTGCATT
CCTAATCCAATTGCACCTCAGTATTTTGAATGGCTGCTGAGGCAGCTAGCCAGGTGCATCAAAATCTAGGAAAATAAGCCAA
GAGGGGGCTTATCTTTCATAATCTTTCTCCCTAACCTCTGCTTCCAAGCTTGAATAGGTGCAAATTTGTAGAAGAGGCTTAGA
ATGGGCTCCATGGTCCGAACAACGGTAGCCGGGCTCAAGCCCACCCCACCCCGGTTGTTGGATCCGCCCATGTGCAGCAAC
CTGCGCACTCAAGAGTTGGCCTTGGGACCTTGTTGCTACTTTTGCCATGTGTAGTAGTAGGCCCAGTGAGGAGCCAAAACA
ACGGTAACAGTACCGGCACAAAGCATATAAGTCAACTGAAACAAGCTACAGATCAAGGGGGGAATCGAAATGAATAACCCT
GCGATATTCAGTTTGGAAATTTTCCAAGGTAAAACAAATTTTCTAGGATTAAACTAGATTTTAGGATTTTTAATTTCATTATTTT
CTTTGTGAATATTTATATAAGTTTTGAAAATTTTATTTTTACAATTTTTTGGATATTTTTTGAAAACATTTCTAGTCTAATTTAGCT
CCATAAACTTTCAACTTAGTTCGATTTACTCCTCTAAGGTGGCGCCACATTAGCAGAATTATCCAAGGTGCAAAAACAAACTG
TTTTGTTTTATTATTCAGTGAGAAAAATCAGATGACCGCGATAAGGGTGCAAAAACAAACTGTTTTGTTTTATTATTCAGTGA
GAAAAATCAGATGACCGCGATAATCGAGAGAGATAATTTGGATTGTTTTCAAAAAAATGAAGCCTGTTTTTTAGCAGAAAAA
AAATGAATCGTGATAACCGAAAGGGAAATCGATCTAGCCTAGATCGGGCCCGTTTGTACCGTGGGCCTTTTTGGGCCCATTC
CGCCCTACTGCCTCGTTTCCTCCGGTGCGTTCGCGTCTGCCACATTCTGCTACTCGTCGGCGTCGGCGTGGTCGGGCGGCGA
CCGCGGCTCCGTGGCGGCCGTCGGCGTGGAGGACGTGGTGAAGGAAATGGTGAACCATGCTCGCATCAACAAGCACTAAC
GTAGCCGGGCAAAACTGAGCAAGGAGGCGGGCGGCAAGACAAGAGAGCATCCGACTACCGTGCATGCCCGATCGACCACC
GTAGCTCAGAGAAGAGCCGTTGAAACGACGACAACCTCGTCAGTCGTCAGAGAGCCGCGCAATGCGGGCATGGCTATTCTT
GGCCAAAACCAGCAGCCGCACCCCCCAAATCCCACCATGGCCGGCCCATGCCATGCGGACGCCTCTCATCTCTCGCCACCG
TCCTCGCCGCACGCGCGTGGGTGCGTGCGTGCGCGCGGGGAGCTCCTGAATCCTCTCTCCTCCCCCTTCTCCGCCTCCCTTT
CCCTCTCTTCGCGTCGACCTCCCGCTCTCCTCCCTCCCTGGAAAAAAACAGCGTGCCGGCCAGCCTTTTCCCCACTCGCGCG
CGCCCGGGAACAGGGGCCGCCGGCCAATTCACCCGCCGTCGTCAAGCGCTGGCGCCGGCGGGCTGGCTCCCTGGTACCGG
CGCGGCGGCCGCCTTCCCGGACATGGCC
>SsLRR-RLK308
GTCGAATAGTAATGCTACTACAATTTTCGCTCGCAACTGTACCTATATTGCCTATTGGTGCAAGTATAAACTCAGTCAAGATAC
AAGACTGCTTTGATTTACGAGTGATTCTCTCTTTTCTTTTTGAGAAACAGAGAACTTCTATTGATCAATCACAAGGTGTAGCC
CTGTTCGCTTGAACTACTTCAACGATACTTTTCAGCGAACGAATAGTGTTTTCCTTTCACAACAAATCAGTATAAACTAAATT
TCAGCGACTACCAAATGCTGAATCAACACAAAAGGGGGACATTTTAATGAGGTGCATGGCCAGGCAATTTGCAAATCTGTAC
AAATGGTCTGTCATCACATTATGGGAGCCATGCTGTCTCTGAATATTACTGCTTAATAATGCATTCACACCATTATATTCAGCCT
TCTCATGAACCGAATGAACAAAACAAATCAAACCAAACCAGACCATTCCTCATGTTGTTTGTTGCATTCCTAATCCAATTGCA
CCTCAGTATTTTGAATGGCTGCTGAGGCAGCTAGCCAGGTGCATCAAAATCTAGGAAAATAAGCCAAGAGGGGGCTTATCTT
TCATAATCTTTCTCCCTAACCTCTGCTTCCAAGCTTGAATAGGTGCAAATTTGTAGAAGAGGCTTAGAATGGGCTCCATGGTC
CGAACAACGGTAGCCGGGCTCAAGCCCACCCCACCCCGGTTGTTGGATCCGCCCATGTGCAGCAACCTGCGCACTCAAGAG
TTGGCCTTGGGACCTTGTTGCTACTTTTGCCATGTGTAGTAGTAGGCCCAGTGAGGAGCCAAAACAACGGTAACAGTACCGG
CACAAAGCATATAAGTCAACTGAAACAAGCTACAGATCAAGGGGGGAATCGAAATGAATAACCCTGCGATATTCAGTTTGGA
AATTTTCCAAGGTAAAACAAATTTTCTAGGATTAAACTAGATTTTAGGATTTTTAATTTCATTATTTTCTTTGTGAATATTTATAT
AAGTTTTGAAAATTTTATTTTTACAATTTTTTGGATATTTTTTGAAAACATTTCTAGTCTAATTTAGCTCCATAAACTTTCAACT
TAGTTCGATTTACTCCTCTAAGGTGGCGCCACATTAGCAGAATTATCCAAGGTGCAAAAACAAACTGTTTTGTTTTATTATTCA
GTGAGAAAAATCAGATGACCGCGATAATCGAGAGAGATAATTTGGATTGTTTTCAAAAAAATGAAGCCTGTTTTTTAGCAGA
AAAAAATGAATCGTGATAACCGAAAGGGAAATCGATCTAGCCTAGATCGGGCCCGTTTGTACCGTGGGCCTTTTTGGGCCCA
TTCCGCCCTACTGCCTCGTTTCCTCCGGTGCGTTCGCGTCTGCCACATTCTGCTACTCGTCGGCGTCGGCGTGGTCGGGCGG
CGACCGCGGCTCCGTGGCGGCCGTCGGCGTGGAGGACGTGGTGAAGGAAATGGTGAACCATGCTCGCATCAACAAGCACT
AACGTAGCCGGCAAAACTGAGCAAGGAGGCGGGCGGCAAGACAAGAGAGCATCCGACTACCGTGCATGCCCGATCGACCA
CCGTAGCTCAGAGAAGAGCCGTTGAAACGACGACAACCTCGTCAGTCGTCAGAGAGCCGCGCAATGCGGGCATGGCTATTC
TTGGCCAAAACCAGCAGCCGCACCCCCCAAATCCCACCATGGCCGGCCCATGCCATGCGGACGCCTCTCATCTCTCGCCACC
GTCCTCGCCGCACGCGCGTGGGTGCGTGCGTGCGCGCGGGGAGCTCCTGAATCCTCTCTCCTCCCCCTTCTCCGCCTCCCTT
TCCCTCTCTTCGCGTCGACCTCCCGCTCTCCTCCCTCCCTGGAAAAAAACAGCGTGCCGGCCAGCCTTTTCCCCACTCGCGC
GCGCCCGGGAACAGGGCCGCCGGCCAATTCACCCGCCGTCGTCAAGCGCTGGCGCCGGCGGGCTGGCTCCCTGGTACCGG
CGCGGCGGCCGCCTTCCCGGAC
>SsLRR-RLK321
ACTGACATGGTGAGCAGCATCTTCATGAGCTTCTTTTGCCGGCTTCTTATTTAGGCATTCTCTCGCCCAATGCCCGAAGACTC
CACACTTCTTACATCTCCCATTGCGTCGGGGTGTTCCTGACGACGGATGACAGCCGGGATCACGCTTGTCGCGGTCGTTCTT
GCTGCCACTTGTGCGTGCTCCAGCCTTGTTCTCTTTGCATAGTGGCTCCCACCTTTGCCGCTCGAGGAGGACGACTCTGAGC
CCACCATGCGACTCTTGTTCTTCGCCATCCACTCCTCCGTAAGAATAAGCGAGGGCTTGGTCTTATCGGTGCCTTGATCTCCT
GAGGGTTCAAACCTGTCGTCGGCTGCCCTGAGACGACCGACAAGCTCTTCGACTGACAATGTTTTCAGATCACAAAACATC
TCTATCGTCACCGCCACCTGATTGTAGCGTGGAGGCACAACTCTGAGAAACTTCTTCACAACCCTTGTGTCAGTCACACTCT
CCTCACCAAGGCCCTTTAGATCGGTAGCCAGCTTTGCAATCTGGACCGCGAAGTCGTCGATCGTCTCTCTAGGTTTGAACGA
GATCGACTCAAACTCCGCGAGCAGCTTCTGTGCATTGACCTCCTTGACTCGTTCTGCCCCCAGACACATCGATTTGATTGCCT
CCCATGCCACCTTTGCCGACTTCTTGTTCAGCAACATGGAGTGCATCTCAGGTGGCACGCCACGGATCATGGCACCGAGCGC
AAGCCGATCACGTCGCCGTTCGACCTTGTCGCTCTCGATTGCATCCCACAGATACATCGCCTCAAGATTGCATTGCACATGCG
CAGACCACTCCTGATAATTTGTGCGAGATAACATTGGCCAGACCAGATTGCCTGACGTTTGATTCCATGCACGCTCCATGGTC
TCCTTCTCGCCGTTGGAACCCATGTCGACGGTCGTCGATGTTCACGTCTCCAAGATCTTGATCTCGATGGATTCCTCCACCCA
GCACCTTCCTCAATGTCTCTCCGCCGCCAAACGTCGTCTAACTGGCTCCGATACCAGTTGTTAGAACTAGATCGACTCCTCGA
CGTTGTGCACGCCGCCGGAACTGATGGTGATCGCGGCGACTTAAACCCTAGGAAGCATACAACTTAATATCACAAGAAAATA
TGACGCTGCAATCAACCACAGCTTAGATGGCCTTGTGTTCCTTGTATTTATAGATATGCTCGAGACTTACAGACTCCAAGCCC
CCCACGCCATGTCCCATAAAGATCGCAACACCGTTTCACCGCGGCGTGCGCTACACGCGCTCTACAAGACATGGCGTAAGGC
TGACGGACACAACCGAGTACATCGTGATCCCGCACGCCTACGATCACGGTTACCCCTACCTGACTATTACAGACTCTTCATGA
CTAGGATTACTTAATGACCGTGCAACAATTTAGCTAACATAACGTAGCCGAGCAAAACTGAGCCAGGAGGCGGGCGGGCAG
ACCCTGGCACCCTGCTGCCGGCAAGACAAGAGAGCATCCGACTACCGTGCATGCCCGATCGACCACCGTAGCTCAGAGAAG
AGCCGTTGAAACGACGACAACCTCGTCAGTCGTCAGAGAGCCGCGCAATGCGGGCATGGCTATTCTTGGCCAAAACCAGCA
GCCGCACCCCCCAAATCCCACCATGGCCGGCCATACCATGCGGACGCCTCTCATCTCTCGCCACCGTCCTCGCCGCACGCGC
GTGGGTGCGTGCGTGCGCGCGGGGGAGCTCCTGAATCCTCTCTCCTCCCCCTTCTCCGCCTCCCTTTCCCTCTCTTCGCGTCG
ACCTCCCGCTCTCCTCCCTCCCTGGAAAAAAAACTGCGTGCCGGCCAGCCTTTCCCCCACTCGCGCGCGCCCGGGAACAGG
GGCCGCCCGCAGCATTGCCGCCGGCCAATTCACCCGCCGTCGTCAAGCGCTGGCGCCGGCGGGCTGGCTCCCTGGCACCGG
CGCGGCGGCCGCCTTCCCGGACATGGCC
>SsLRR-RLK206
TAGCTAGGGAAACTAGGTAAGTGTTGATGAACCATGAAAACTAGCCACAAAAGTTTTTCAAAATCGTCAAGAAATTATCAGA
TGTAGGGACTAGGGAGGCATGTCCTATATCTGTGTAAAATGAAACTTGAAGTCCAACTCATTGGCAGGAGGAGAAAACTTAC
CACCTTTTTTTTAAAAAAATAATAGAAAAAGATTTTTAACTTTTAGCTTCACTAGATTTGCTCTCTCTAATGTATTGTGATATCT
TACACGTCATTTAATCCCTTGTTACTGTCTTGTGGTTGTGGTCTATAAAAGCATGAAGCAATTTTGTGCAGTCAGCTTTTGGTT

CGGTGCCCTGGTAAGGTGAAGCAATTAAAAATGATTTATGTTTTGTAAAATTGCATTGGACAACTTTTTTGTAGCTTATAGTCC
TCAAGATATTCATATCAAAGCTTAGACTGTCTAACTTTTGAAGGACCTGCTTAAAGCCTCTTGTCCTGTATTTTTGGGAAACTT
TTGGCATACATATAGTTTCATGTATAGTATCAATTAGCCAATGTTTGTAGCAAATGAGCATCAGTCGCCAAGTAACACAATATT
GCATTGTTTGATAATACATGCACAATCATGTTAGACAGATGGTTTGCTTTAAGAACTCATTTATGTTCATCGAACATACATTTGT
TCCTGGTGTCGAAAAGGATTATATTTTATGCTGCTCTGAGCCAGCACAATACGAGTTCCCTATAGTTCAAAGTTCCAACTCTG
ATGTATTCTTGTTCCTTTTGTCTTGTCATATTTTTTGCTGCTGCCTTACTTTCTTCACAATATATTTGTGTAGAAGGGCAAGAGA
CCGGGACAAGTATTGAACCCAACAAAAGCCAAAATATTGCATGGGAGCTGGCAAACAAGTTACACGAGAACGCGCAGCATA
TCCACACCATACTGAACGAAGAGGCCGACCAAGATGGTGGCCAGAACCATGCTCTTGGTGGTTTGATGCTGCCTGATGCAAT
GGAGGCCACCCGCCAAAAAGCCGATGAGCTGATCAAGTCGCTAGGCGGGCTTGTCAGCTACCTGAACCAATTCACCGAGCT
GGTCAAGGAAACTGGGTTTGAGAACATCGCCGGTATGACCTGATGCACGAGACGCAGCCACGCAGGCCTCCTTTGGAGGAT
TAGATGATCATGCTGCTTAATGTTTTAGCGCTGTAGTTTCTCTGTGTCGTCCTCCATCTTTGTAAATATGCGGCACTGTATGACG
ACCGCCCAGCCCGGGTGGTGTAGGGTTTGTAGCAGACAGTGTTAGGTGCCTTGACAGCACCGCTGTACTGGGCTTCTGGCC
TTGATTAGTGAGATCAGATGCTCACATCTGTTGTCTGGCGTTCCTTTCAGTTCTGGCCCTGAAAATCCCTGATGATGAGAGAT
CAGATGCCTCAATTTGGTAGCAGTAGCAGATGTTCTCAGATGCATCACATATTCACATGTGAGATATAAGATGGGTGGGTGAT
GAGGCTGTAGTAAATTGGGATTAAACCAGAATGTTGCGAGGTTGGTGTCCTGGGGACATGGCATCTGTTGTTGCTGACAAAA
ATGAACCATGAACTGGCCAAGGCTAAAACCTGCTCTCGCAGCCAACTCCAACCACCAATGACACAGAAACCGGCCGCAGG
CAGCAACAGGCACAACAGACGCTGCTGTATCTGCCGTCTCTTCTCCCGCCTATTCTTAGGCTCTCTACCTCCCTCCCCACCCG
GCGTCGCGACCCATCACCGCATTTCCTCTCCCCGCCACCTCCTCTCTCCCCTCCATTCTCTTGACCCATGCACACGCGGGACG
CGTAGCTTCGCCCTCGCCTCCTCCCACGACGACGCGCACGGGCGTGCGTGCATGCGGCGAGCGGTCACAGCCCCTCCGGCA
AGGCGACCACCGCCGCGCGCGTGAAGACACCGGCTCGCCTCGGCTCGGTTCATCTCGTCTCGTCGGACGACTCGCTCGGCG
CCGCCCGCGTCCCCA
>SsLRR-RLK230
ATAGCTAGGGAAACTAGGTAAGTGTTGATGAACCATGAAAACTAGCCACAAAAGTTTTTCAAAATCGTCAAGAAATTATCAG
ATGTAGGGACTAGGGAGGCATGTCCTATATCTGTGTAAAATGAAACTTGAAGTCCAACTCATTGGCAGGAGGAGAAAACTTA
CCACCTTTTTTTAAAAAAATAATAGAAAAAGATTTTTAACTTTTAGCTTCACTAGATTTGCTCTCTCTAATGTATTGTGATATCT
TACACGTCATTTAATCCCTTGTTACTGTCTTGTGGTTGTGGTCTATAAAAGCATGAAGCAATTTTGTGCAGTCAGCTTTTGGTT
CGGTGCCCTGGTAAGGTGAAGCAATTAAAAATGATTTATGTTTTGTAAAGTTGCATTGGACAACTTTTTTGTAGCTTATAGTC
CTCAAGATATTCATATCAAAGCTTAGACTGTCTAACTTTTGAAGGACCTGCTTAAAGCCTCTTGTCCTGTATTTTTGGGAAACT
TTTGGCATACATATAGTTTCATGTATAGTATCAATTAGCCAATGTTTGTAGCAAATGAGCATCAGTCGCCAAGTAACACAATATT
GCATTGTTTGATAATACATGCACAATCATGTTAGACAGATGGTTTGCTTTAAGAACTCATTTATGTTCATCGAACATACATTTGT
TCCTGGTGTCGAAAAGGATTATATTTTATGCTGCTCTGAGCCAGCACAATACGAGTTCCCTATAGTTCAAAGTTCCAACTCTG
ATGTATTCTTGTTCCTTTTGTCTTGTCATATTTTTTGCTGCTGCCTTACTTTCTTCACAATATATTTGTGTAGAAGGGCAAGAGA
CCGGGACAAGTATTGAACCCGACAAAAGCCAAAATATTGCATGGGAGCTGGCAAACAAGTTACACGAGAACGCGCAGCATA
TCCACACCATACTGAACGAAGAGGCCGACCAAGATGGTGGCCAGAACCATGCTCTTGGTGGTTTGATGCTGCCTGATGCAAT
GGAGGCCACCCGCCAAAAAGCCGATGAGCTGATCAAGTCGCTAGGCGGGCTTGTCAGCTACCTGAACCAATTCACTGAGCT
GGTCAAGGAAACTGGGTTTGAGAACATCGCCGGTATGACCTGATGCACGAGACGCAGCCACGCAGGCCTCCTTTGGAGGAT
TAGATGATCATGCTGCTTAACGTTTTAGCGCTGTAGTTTCTCTGTGTCGTCCTCCATCTTTGTAAATATGCGGCACTGTATGAC
GACCGCCCAGCCCGGGTGGTGTAGGGTTTGTAGCAGACAGTGTTAGGTGCCTTGACAGCACCGCTGTTCTGGGCTTCTGGC
CTTGATTAGTGAGATCAGATGCTCACATCTGTTGTCTGGCGTTCCTTTCAGTTCTGGCCCTGAAAATCCCTGATGGTGAGAGA
TCAGATGCCTCAATTTGGTAGCAGTAGCAGATGTTCTCAGATGCATCACATATTCACATGTGAGATATGAGATGGGTGGGTGA
TGAAGCTGTAGTTATCTGGGATTAAACCAAAATGTTGCGAGGTTGGTGTCCTGGGGACATGGCATCTGTTGTTGCTGACAAA
AATGAACCATGAACTGGCCAAGGCTAAAACCTGCTCTCGCAGCCAACTCCAACCACGAATGACACAGAAACCGGCCGCAG
GCAGCAACAGACACAACAGACGCTGCTGTGTCTGCCGTCTCTTCTCCCGCCTATTCTTAGGCTCTCTACCTCCCTCCCCACC
CGGCGTCGCGACCCATCACCGCATTTCCTCTCCCCGCCACCTCCTCTCTCCCCTCCATTCTCTTGACCCATGCACACGCGGGA
CGCGTAGCTTCGCCCTCGCCTCCTCCCACGACGACGCGCACGGGCGTGCGTGCATGCGGCGAGCGGTCACAGCCCCTCCGG
CAAGGCGACCACCGCCGCGCGCGTGAAGACACCGGCTCGCCTCGGCTCGGTTCATCTCGTCTCGTCGGACGACTCGCTCGG
CGCCGCCCGCGTCCCCA
>SsLRR-RLK420
CCTGTTTGTTTTCGCTTATAATCATTGTACCATGAGAATTTTTTTCGATTCTCGGTTGTGCTAGTTCTAAGTTTCAAAACGGCA
GCTAGAAAAATAAAAAAAATCAAAGTGTTTAGTGGGATTCTCGCTTTTTTCATCGATAATCTAATTGTAAATGAAAACAAATA
GTTTAGTTTTGGGCCCATAATTTAAGTTCCTTTTGACTGGTTCCTCTTATTTTTCAATGAAGACTGTTTTTTTAGGCACCATAAA
ATTTCTCATAGAGACATGTTTTTTTTTTTGAAACTTCATTGAATCAGAATTGAGCTAAACCTAGATATTTCCAACAAAATGCGT
ACATATATATGAATATTATTTGCTAGCAGAACGTCGTCACGCCGTGATCGAAACCAAGGTACACGCCGCATATCCTTATATAGA
GCATAAATATGTCGCCGGAACACGCATAGCTAGTCGTGATAGTAGCTAGGGAACAAAGCATTTTACTCGCGGCATATCCCGGC
AAAAACATCTGCTTCGGCTCCCATACATCGCATAACATAAGGACACACCCCTCGTTCTCATCGTGCATTCTGTTAACCCGGAT
GCAGAATGCAGAATTGCAGACAACGACTTCGCGGATGGCATGCTCTCGCCCGCGTCGCCGAGATCGTGGCCGCGACTCGGG
CGCACGAAGCTGGAAGCCTGGCTTATCTGAAAGCCTGAAACGTGAGGCCACCGTGCCGTCGTGCGTCTCGTTTATTCCATCA
TCAGGACACACTAAAAAATAGCTATGGATTGGGTGCAGGCTGCAGGTGCAGTGCAGTACGGTTCCCGTTTACCTTAGCGTCG
GTTGCTCGTTTGGTAGGTAGTAGACGGTGAGTGTGCATCGGTTGAAAAAACGAGTGCGAATGCACGGACACCAAGGTACCG
TTTAGATGCCAAAAATTTTGCCAAATTTTTCAGATTCTCCGTTACATCGAATCTTTGACGCATGTATGAAGTATTAAATATAGAT
AAAAAATAAAACTAATTACACAGTTTAGACGAAATAGACGAGACAAATCTTTTAAACCTAATTAAACTATGATTGGATACTAA
TTACCAAATAACAACGAAACTGCTACAGTTACATTTCGCCAAAAATTTTGCCAACTAAACAGGCTCCAAGTGAAACGTCTCC
ACAGGTCAGACGACTCTTTTATAGCTCTGCAAGGACCGACTGCACAACGTGTCACACAGAAGTTGTCTGTGCAGCTCGCGT
GGCAATCTCACACCGCTGTCGGTCGCTGTTTGGCTAGAGAAAAACTATAAATTATCTCTTGTAAGTTGTTTTTTAATTGTACTA
TTTGTTTGTGTAAAATTACTGTCCCAAAAATCTTAAAAATTCGATCGGACACCAAGAATATAGCGACTTCTTTAACTTTATGCT
TACATTTCTCTTTCGGAGCACGTCGGTTTCACTTCACCGCTCGATTCGCAGTGCTACGGGCACAACAGCTGCTTGAATGAAA
AGTACTGATTTTTGCGTGAGCTAGTTCCAAACCGCCTGTAAAAAAGGTACCCCATTTCCCCGCTGTAAATGACAGTGAAAAC
TTAGGGTCCAATAGAGAAATGAGAAGAAAAGTTGAGCTGGGTTTCAAGTTCGTCCTCTTCCGCTGGCACCAGACTTGCGTC
GCTGATGAAATCTTCAGAAGCTTCGAATACTAGTATACTACTTCATTTGTTACTGATGCGGTCTACTCCTAATCAACAGCTGAC
AGCCTGCGCGAGCGAACGACGGCAACAAGAAAGACGTTGCGGTCGCAGCGGCGACTCCCAAACGCGCCCGCCTAGGTAAC
GCAGCCCGCGCCCCTATTCTTAGCTCGCCACGAGGCCGGCGCGGGAGCCGTGTCCGCTGTCCGCCCGCCGCCTCCTTGCGC
CATGCGCACCCTCTCCACCCCTTCAGCACGTCGCCGCCAGCGCTAGTGCGCCGCACGCTTCCACGCACGCCCACGCATCCGT
CTCCGCCTCCCGCGTCC
>SsLRR-RLK408
TAAGAAGGGGGGAATGTTAGGGACTACCCTAATTGGAATTTCTTTTGGATAGTTCAATTCAGATTATTTCAAAATCTTATTATT
TGTTTGCTTACAGACAACCCAACAGGTTATCCCCTACCTAGAATTTCCCTCTCTCTCTACTGTTCTTCCTTCCTCTCCAACCCT
AAGCTTCCACTGCTGATCTTCTAATTCTCCACCTCTCTCTGCCAATCCAGCTACCAGGTTGCTAACATCATCCGTGCCTTTCCT
GTTTGTTTTCGCTTATAATCATTGTACCATGAGAATTTTTTTCGATTCTCGGTTGTGCTAGTTCTAAGTTTCAAAACGGCAGCT
AGAAAAATAAAAAAAATCAAAGTGTTTAGTGGGATTCTCGCTTTTTTCATCGATAATCTAATTGTAAATGAAAACAAATAGTT
TAGTTTTGGGCCCATAATTTAAGTTCCTTTTGACTGGTTCCTCTTATTTTTCAATGAAGACTGTTTTTTTAGGCACCATAAAAT

TTCTCATAGAGACATGTTTTTTTTTTGAAACTTCATTGAATCAGAATTGAGCTAAACCTAGATATTTCCAACAAAATGCGTACA
TATATATGAATATTATTTGCTAGCAGAACGTCGTCACGCCGTGATCGAAACCAAGGTACACGCCGCATATCCTTGTATAGAGCG
TAAATATGTCGCCGGAACACGCATAGCTAGTCGTGATAGCAAAGCATTTTACTAGCGGCATATCCCGGCAAAAACATCTGCTT
CGGCTCCCATACATCGCATAACATAAGGACACACCCCTCGTTCTCATCGTGCATTCTTGTTAACCGGATGCAGAGTGCAGAAT
TGCAGACAACGACTTCGCGGATGGCATGCTCTCGCCCGCGTCGCCGAGATCGTGGCCGCGACTCGGGCGCACGAAGCTGGA
AGCCTGGCTTATCTCAAAGCCTGAAACGTGAGGCCACCGTGCCGTGTCGTGCGTCTCGTTTATTCCATCATCAGGACACTAA
AAAATAGCTATGGATGGGTGCAGGCTGCAGGCGCAGTGCAGTACGGTTCTCGTTTACCTTAGCGTCGGTTACTACTGGTTTG
GTAGGTAGCAGACGGTGAGTGTGCATCGGTTGAAAAACGAGTGCGAATGCACGGACACCAAGTGAAACGTCTCCACAGGT
CAGACAAATCTTTATAGCTCTGCAAGGACCGACTGAACAACGTGTCACACAGAAGTTGTCTGTACAGCTCGTGTGGCAATTT
CACACAACTGTCGGTCGCTGTTAAATTATCTCTTGTAAGTTGCTTTTTAATTGTACTATTTGATTGTGTAAAATTATTATCCAAA
AAATCTTTAAAATTATTATCCAAAGGCTGTGTTTAGATCCAAATTTTGGAGTGCCAAAATACACTGTAGCATTTCGTTTGTATT
TGGTAATAATTGTCCAACCATTGACTAATTAGGCTCAAAACGTTTGTCTCGCAAAGTACAACCAAACTGTGCAATTAGTTTTT
GATTTTATCCACATTTAGTACTCCATACATGTATCGCAAGTTTGATATGACGGGAAATCTTTTTTTTTGCATAGTGCACTTTTTA
ATGGATCTAAACACAGCCTTTATGCTTACATTTCTCTTTCGGAGCACGTCGGTTTCACCGCTCGATTCGCAGTGCTACGGGCA
CAACAGCTGCTTGAATGAAAAGTACTGATTTTTGTGTGAGCTAGTTCCAAACCGCTTGTAGAAAATGTACCCCATTTCCACG
CTGTATATGACAGTGAAAACTTAGGGTCCAATAGAGAAATGAGAAGAAAAGTTGAGCTGAGTTTCAAGTTCGTCCTCTTCCG
CTGGCACCAGACTTGCGTCGCTGATGAAATCTTCAGAAGCTTCGAATACTAGTATACTACTTCATTTGTTACTGATGCGGTCT
ACTCCTAATCAACAGCTGACAGCCTGCGCGAGCCAACGACGGCAACAAGAAACACGTTGCGGTCGCAGCGGCGACTCCCA
AACGCGCC
>SsLRR-RLK396
CGTCCTGGTGCGCCGCGGCCTGCAGTACCCACAGGTCAGGTGACGTCGCCCACCCGGAGCCAGAGGTGGCCTTGACCACG
TCCTCGTCGAAGAGGAAGGTGGCAACGAGGAAGACGTCGACAAAGAGGTCCACGAGCAAGAGGTGCTCCAAGAGCCTGC
ACGCGGACTCCTGCTTGGTGGCCAGCGGAACCAACAGAGCTGCGAAAACGAACAGGGATCTAGCAGGCGAGATCCACCGA
GGAGAGCTGCCAAGAACAGGGAGAGGGGAGGGAGGAGGTGACCGCGACGGTGGATTGAACAGAGATTGGGCGGATTGAG
GTTGGGGATTTAGAGATTTGGGGGAGGGGAAAGGGGATACGGCATATCGCCAAGCTTCACCACGAAGGGTCTCGGTCTGCA
CCAACTGGACGCCTCCTACATATGTCTAGAGATATGTTTGATTGAATCAGAATTGAGCTAAGCGTAGATATTTTTTTATATCAA
AGGCTTGAGCTAAGGCCTTGTTTGATTCACGAATGTTAAAGTTTAACTCTCATTACATCAATGTTGCGGTAAATGCATGAAGT
ACTAAATGTGTAGACTAATTGTATGCATGTCGGCACAGATTAGAAGTAATTTACGAGACGAATCTATTAAGCCTAATTAGTTTA
AAATTAAAAACTTTTTATCAAATAAAACGAAAGTGTTATAATGTTTGTTAAACTTTAACACCTCTAACTAAACAGGCCCTAAG
CCTAGATATTTCCAACAAAATGCGTATACATATGAATAATTATTGGCTAGCAGAACGTCGGCACACCGTGATCGAAACCAAGG
TACACGCCCCGCATATCCTTTGTAGAATAAACATGTCGCCGGAACACGCATAGCTAGTCGTGATAGCAGCGAGGAACAAAGC
ATTTTACTCGCGGCATATCCCGGCAAAAACATCTGCTTCGGCTCCCATACATCGCATAACATAAGGACACACCCCTCGTTCTC
ATCGTGCATTCTTGTTAACCCGGATGCAGAGTGCAGAATTGCAGACAACGACTTCGCGGATCGCATGCTCTCGCCCGCGTCG
CCGAGATCGTGGCCGCGACTCGGGCGCACGAAGCTGGAAGCCTGCCCTGGCTTATCTCAAAGCCTGAAACGTGAGGCCACC
GTGCCGTGTCGTGCGTCTCGTTTATTCCATCATCAGGACACACTAAAAAAATAGCTATGGATTGGGTGCAGGCTGCAGGTGCA
GTGCAGTGCAGTACGGTTCCCGTTTACCTTAGCGTCGGTTGCTCGTTTGGTAGGTAGTAGACGGTGAGTGTGCATCGGTTGA
AAAAAACGAGTGCGAATGCACGGACACCAAGTGAAACGTCTCCACAGGTCAGACAAATCTTTATAGCTCTGCAAGGACCG
ACTGAACAACGTGTCACACAGAAGTTGTCTGTACAGCTCGTGTGGCAATTTCACACAACTGTCGGTCGCTGTTAAATTATCT
CTTGTAAGTTGCTTTTTAATTGTACTATTTGATTGTGTAAAATTATTATCCAAAAAATCTTTAAAATTTCAGACACCTAGAATAT
AGCGACTTCTTTAACTTTATGCTTACATTTCTCTTTCGGAGTACGTCGGTTTCACCGCTCGATTCGCAGTGCTACGGGCACAA
CAGCTGCTTGAATGAAAAAGTACTAATATTTGAGTGAGCTAGTTCCAAACCGCTTGTAAAAAAAATGTACCCCATTTCCACTC
TGTAAATGACAGTGAAAATTTTATGGGTCCAATAGAAATATGAGAAGAAAAGTTGAGCTGGGTTTCAAGTTCATCTTCTTCC
GCTGGCACCAGACTTGCGTCGCTGATGAAATCTTCAGAAGCTTCGAATACTAGTATACTACTCCATTTGTTACTGATGCGGTC
TACTCCTAATCAACAGCTGACAGCCTGCGCGAGCCAACGACGGCAACAAGAAAGACGTTGCGGTCGCAGCGGCCACTCCC
AAACGCGCCCGCCTCGGTAACGCAGCCCACGCAC
>SsLRR-RLK382
CCACAACCTAGTGTGGCTATCAATGGCGGGGAACCGAACAACCCTTATATTTCTTTACGATTTGATTGAATTTCAAAATCTAC
AATCCAAATTCTCAGACCAACTGGTACCCAAAGATGTGCACTTGAGCATCTCGAAGGGTGCAACACAACCTCGTCACGAGT
ACACACCACAATACCCTAAATCCCAACGACAAGGAATAGTTGGTTAAGAGTAGGATATTCAAATCAAACAGTGGGTGAGATG
ATGAATCGTATTCGGTGAACACAGAATCTTCAAATCAAGCCGGCCGCAGCAGGCAACGTGCCACGGCGCAGCCACACCAAA
GATGTGCGGCACACAAACCGTGCACCAAAGACGTAGTACTAGGCCAATCGCGCCATAAAGAACTGGAGACCGCAATTGGTT
GGGTCTGTTTGGAACGCAGGAATTTCACAGAAATCAATTCATTTTCACATAAAAACACAGGAACATGGAAAAATTCCCACGT
TTCCATTGCTAGGACGTACACGACGCTGGGCATATGGCTTGCTGGGCCTATCCGTGCCTTTTTCAGGCTGATGAAAAGCTCTT
AATGTGCACCCATATCGTGTATGCCGGCTTATATAAGAAAGAGAAAAGTGAAGAAGGAACGTTTTAATACCTCAGATAGCTA
GTATAATCATTTCGTTTCTTTAGAGAAAGGAAGAAACAGATAGTATACCGAAATTTCTTTAGTTATCAGGTACGTAGTATACAC
ACATCATCATCGTAGATGAAGCAGTGACGCTGTGACAGCGATGGACGCAATCCGAATCTTAATTTCTGCGTGCATGATTGCGA
GACGGAGCCGCCGCCATCATGACGAGAGCCGGCTGGATGATGAAATCAAAGTGAAAAGAGCAATAAGGGCGCGTGCTGCT
TAGCCTAAGACCGTCGGTTGAGAGTGTCAGTTAAGCGAGCCTGCCTAAGGACTGACTCGTCTGGTATGCTCTGGTCGATCGA
TCCATTGCTTCCGAACCACCCACCCAGGGGGGCCAGGGCTGGCATCGATCAGAGGATCCATCCGGCATCCGGTCCCGTCTCC
CGTTGCAGCCTCTCATGAGATGAACCTCGAAGAAGCTCTGGCCGTCAGTGGAGGCGTGACAGCTGTCTGGTGTCGCCCTGC
CACAACATACTGCACAGGAGTCTGTGTACTACCATAGTACGTAGGTGACACTAGTCAGCACGGTGGATGCCTCTGGGTGTCT
GCACGCAACGCATACACGGCCAAGCGAGCCCACAAGACAGCGTGACAGGCCGTAGCAGTAGCGTGCGCCGCGAGCCCGAT
GCTCGGCCGCCTCGGCTGGAACTGGATTCCCATGATTGCCAGGTGGAGGCCGCCATGATGACACACAGGACAGGAGCCCTG
CTCGGCGTCGCTGTTGTCGCACGCAGCTCACAAGGCCGGGCATCCATCCATTGCTTCCGCCCCACGGCTGGGCTGGCACGC
AGAGTACTACTGTGCCCAATTGGGGTAGGGCGTTCCTGCTGTCGTACGATCGATCGATCGATCGATCGGCTGTTCCCTTTCCC
TTCCTCAGATTGCATCGAAGCAGCGCTGCTTTTATGACTGCATCTGGCGCCCAGCCGCCGTTGCCGGTCAGCCTGAAAGCGC
CGCTGCAATTCCAATTCTGCATGCATGCTAGCCGTAGCGGACACAACCCAACCGCGTCGCGTCAGGCCCCGGCCTGCCCGCC
TGGGGATCGGAGTTCGGAATCGATCACCAGCTTGGGCGGCTTCCAATCCAATCCGCCCCGCGCCCACAGCCACTTCGCCAC
GCATACATAAAGCCACCCAACCCTGCCCACCTCTGCTCTGCTCCGATGACAACACCAACGGCCGCCGCCTCAAGCCATGCCT
CGGTCTCCGGCGCGCCCGCGTCCACCCAGCCCCAGCACCCCGTAGGCCGCCCGTAGCCTACGTGCCGCCACTCCCACTCTC
CTCTCCGGCTCTCCCTCTCCACACCCATCCATCCAAAT
>SsLRR-RLK361
ATAGAAAAGGTAGCTGTTGTTTGGTTCCTGCGTGAGCCGTTGAGAAAGCCACAAAACTTTTCGGTTTGTTCCTTTAATACTTT
ATGATATAAAGTGCGGCTGTCTTCTTCTCCGCCACAAACTTTGGCAGCGCCACAACCTAGTGTGGCTATCAATGGCGGGGAA
CCGAACAACCCTTATATTTCTTTACGATTTGATTGAATTTCAAAATCTACAATCCAAATTCTCAGACCAACTGGTACCCAAAG
ATGTGCACTTGAGCATCTCGAAGGGTGCAACACAACCTCGTCACGAGTACACACCACAATACCCTAAATCCCAACGACAAG
GAATAGTTGGTTAAGAGTAGGATATTCAAATCAAACAGTGGGTGAGATGATGAATCGTATTCGGTGAACACAGAATCTTCAA
ATCAAGCCGGCCGCAGCAGGCAACGTGCCACGGCGCAGCCACACCAAAGATGTGCGGCACACAAACCGTGCACCAAAGA
CGTAGTACTAGGCCAATCGCGCCATAAAGAACTGGAGACCGCAATTGGTTGGGTCTGTTTGGAACGCAGGAATTTCACAGA
AATCAGTTTATTTTCACAGAAAAACACAGGAACATGGAAAAATTCCCACGTTTCCATTGCTAGGACGTACACGACGCTGGGC

ATATGGCTTGCTGGGCCTATCCGTGCCTTTTTCAGGCTGATGAAAAGCTCTTAATGTGCACCCATATCGTGTATGCCGGCTTAT
ATAAGAAAGAGAAAAGTGAAGAAGGAACGTTTTAATACCTCAGATAGCTAGTATAATCATTTCGTTTCTTTAGAGAAAGGAA
GAAACAGATAGTATACCGAAATTTCTTTAGTTATCAGGTACGTAGTATACACACATCATCATCGTAGATGAAGCAGTGACGCT
GTGACAGCGATGGACGCAATCCGAATCTTAATTTCTGCGTGCATGATTGCGAGACGGAGCCGCCGCCATCATGACGAGAGCC
GGCTGGATGATGAAATCAAAGTGAAAAGAGCAATAAGGGCGCGTGCTGCTTAGCCTAAGACCGTCGGTTGAGAGTGTCAGT
TAAGCGAGCCTGCCTAAGGACTGACTCGTCTGGTATGCTCTGGTCGATCGATCCATTGCTTCCGAACCACCCACCCAGGGGG
GCCAGGGCTAGCATCGATCAGAGGATCCATCCGGCATCCGGTCCCGTCTCCCGTTACAGCCTCACATGAGATGAACCTCGAA
GAAGCTCTAGCCGTCAGTGGAGCCGTGGCAGCTGTCTGGTGTCGCCCTGCCACAACATACTGCACAGGAGTACTGTGTACT
ACCATAGTACGTAGGTGACACTAGTCAGCACGGTGGATGCCTCTGGGTGTCTGCACGCAACGCATACACGGCCAAGCGAGC
CCACAAGACAGCGTGACAGGCCGTAGCAGTAGCGTGCGCCGCGAGCCCGATGCTCGGCCGCCTCGGCTGGAACTGGATTCC
CATGATTGCCAGGTGGAGGCCGCCATGATGACACACAGGAGCCCTGCTCGGCGTCGCTGTTGTCGCACGCAGCTCACATTCT
CACAGGGCCCGGGCATCCATCCATTGCTTCCGCCCCACGGCTGGGCTGGCACGCAGAGTACTACTGTGGCCAATTGGGTAG
GGCGTTCCTGCTGTAGTAGTACGATCGATCGATCGGCTGTTCCCTTTCCCTTCTTCAGATTGCATCGAAGCAGCGCTGCTTTT
ATGACTGCATCTGGCGCCCTACCGTTCCAGTTCCACCCAGCCGCCGTTGCCGGTCAGCCTGAAAGCGCCGGCATGCATGCTA
GCCGTAGCGGACACAACCCAACCGCGTCGCGTCAGGCCCCGGCCTGCCCGCCTGGGGATCGGAGTTCGGAATCGATCACCA
GCTTGGGGCTTGGGCGGCGTCCAATCCAATCCGCCCGGCGCCCACAGCCACTTCGCCACGCATACGTGCCACTCCCACTCTC
CTCTCCGGCTCTCCCTCTCCCCACCCATCCATCCAAA
>SsLRR-RLK375
CTCTCGTTAGGCAAATTCGAACTAGGTGATGGCTCTCAAGTGCGTTTTTGGGAGGATGCATGGATAACGCCACGTCCTCTAA
AGTCGATCTTTCCTGCTTTATATAATATTGTTAGGAGGAAGGGGGTATCGGTGAGAACGGTGTTATCTACAATACCGTTGAATG
TGGCGTTTCGAAGATCGTTGGTAGATGTTAATTTACAATCATGGCACGAGGTGGTTGCTATGGTGGCAGATGTACAATTAACA
AACCAAAGGGATCGTTTTGTTTGGGGGCTACATCAGAATGGATATTTTTCAGTAAAATCCATGTACCGAGCCTTGTTAGAATA
TGAAGCGATACCGTATAACAATGTAATATGAAAATTGAAACTACCTCTGAAGATCAAAGTATTCTTATGGTATTTGTATAAAGG
AGTAATTCTGACAAAAGATAATCTAGCAAGAAGACATTGGCAAGGAGACACCAAGTGTTGTTTTTGTTCATCTAAAGAGACT
ATACAACATCTTCTCTTTGATTGTCATTTTGCTAGATTTGTGTGGCGGGTTATTCATATTTCATTTAACTTAACTCCTCCAACTA
GTGTACACAATTTATTTACAGATTGGTTACTTGGGGTGAATAGGAATTTGAAATATAAGATTCTAGTGGGCGCGGCTACTTTTT
GCTGGATCATATGGTTAACCCGAAATGACTTGGTCTTTGACAAGATCCTAGCACCATCTTATTTACATGTTATCTTCAGGGGGA
CTTACTGGATAAGATTTTGGACCCTTCTTCAGAAGGAGGAGTACCGTCAAATAATGATGATGGGATGCAGGAAAATTGAGAC
AGCCGCAATGGAGGTGTTTGCAAGAAACGGTTGGAGTTTCGTTAATAGAATTATAGTTTAGATGTTTCTTCGACGGACTTTTC
CTTCGTCCTTTTTAGTTCAAAACTTTGTGTTTGAGATTGCCTGGACCTTTTTGCTTCGGTGTGTAATAAGAAACGGCTATATGC
ATTAGTCGATGCAGAAGCTGGAATATTAATATATTCTCTTTTCTAAAAAAAAAAAAAGAGTGTCAGTTAAGCGAGCCTGCCTAA
GGACTGACTCGTCTGGTATGCTCTGGTCGATCGATCCATTGCTTCCGAAGCACCCACCCAGGGGGGCCAGGGCTAGCATCGA
TCAGAGGATCCATCCGGCATCCGGTCCCGTCTCCCGTTACAGCCTCTCATGAGATGAACCTCGAAGAAGCTCTGGCCGTCAG
TGGAGGCGTGGCAGCTGTCTGGTGTCGCCCTGCCACAACATACTGCACAGGAGTACTGTGTACCATAGTACGTACAGTACGT
AGGTGACACAAGTCAGCACGGTGGATGCCTCTGGCTCAGTGGCTCTGGGTGTCCGCACGCAACTCATACACGGCCAAGCGA
GCCCACAAGACAGCGTGACAGGCCGTAGCAGTAGCGTGCGCCGCGAGCCCGATGCTCGGCCGCCTCGGCTGGAACTGGAT
TCCCATGATTGCCAGGTGGAGGCCGCCATGATGACACACAGGAGCCCTGCTCGGCGTCGCTGTTGTCGCACGCAGCTCACA
TTCTCACAGGGCCGGCCGGGCCCGGGCATCCATCCATTGCTTCCGCCCCACGGCTGGGCTGGCACGCAGAGTACTACTGTG
GCCAATTGGGTAGAGCGTTCCTGCTGTAGTACGATCGATCGATCGATCGGCTGCTCCCTTTCCCTTGTTCAGATTGCATCGAA
GCAGCGCTGCTTTTATGACTGCATCTGGCGCCCTACCATTCCAGTTCCACCCAGCCGCCGTTGCCGGTCAGCCTGAAAGCGC
CGCTGCAATTCCAATTCTGCATGCATGCTAGCCGTAGCGGACACAACCCAACCGCGTCGCGTCAGGCCCCGGCCTGCCCGCC
TGGGGATCGGAGTTCGGATTCGATCACCAGCTTGGGCGCCCGGCGCCCACAGCCCCACACACGAGCCAGAACCAGAACCA
CCTCTTCCTCCCGCGCCAGCC
>SsLRR-RLK161
CTCCCCGCCCGGCCGGTGCGCGGTGACGGACGGATGGGAGTGGAGTTGGGTACGGTTTTTAGATGCGAGCAAGCGACTGCG
ACGCGAGCCGATCTGGTTCTAGGTCTCGCGGGTCGGTCTGTGTGGCACGTACGCCGCCGGCCGACCAGCACTCATCCAGAT
CCAGGGGCAGGCAGGCAGGCCTGACAGCGGGCTTCATGCATGGCGCCATCAGGCCGCCCGACGTACGGCGCGGACCCGGC
CCTCGAAGGCAAGACGGCAACAGTACTGAACGTGATGATGGCACACTTGAAAACAACGGTGTTGCATCGCATTGCATCTGC
ATCGTGTCCCACACACTCCCACCCACGACGCTCGCAACGCGCGCTTGCGGTCGACGTGGCCCGTACTCGCTTCCTTTCCAGG
CCTTTTAAAATAATTAGCGCGGGTACGGTGTGCATGGTCGGACGGACGGCTCCAGAGCTCTGATTTGGTTTGGTTGGTCATCC
AGGTGGTGCTCGCGGTTCGGAACCATCGAGGTCATCACTCATCACCACCACGTTGCTTCTGGCTCGCGCTAGCTTTGTGTGT
GTGGCCGCCCGTGGTGTGGGTGCGTTGGTGGCTAATGTGCTCCCGGTCGCTCGCCTAATCCAGACCCCGACGGACGGACGG
AGACCGGCGAGCTTCCGCCTCGACCTCGAATATTTATTTGACTGGCCACATTAGGCGTAAACCGCCGGTTAACCGTCGGCGA
CGTACGTGCTGTGCTGGCACGAGGAGCGTATCTCTGTTGGCACGCGGTTATCCGTTTCGTCCCGAGATTTCTTTCTCGGGGCC
AAGCAACCGCAGATTCGATTTATGGTGGTAGAATTTGTGCATCAGTTTGCTGGCAAATAACATTCTCCTGTTCCTTAGCACTG
CCGCGAAAGCGTGCGTGCGTGCGTGCGTGTGTGCTTAAAAAGGAACCCGTAGGCTAGGCTCCGAAGCCCAATCCAATCCGG
ACTCGTGAGAACGCGTGACGGCAAGGGCGATGTGTAAAAGCAGCGATCAATGTGGCCAGGAAAAGAATGCACGCCTAAAA
GTAGAGCTCTACAAGATACCATACCGGTCGGTACAACTGAGTTTTCGCTTTTATTATTATTATATATTTCTATATTCCTATAGGTG
TAGAGTAGTAAGATCATTTTGTACCAAAAAAAAAGTATGGAGTACGGCATGTTTGCATGCCAACGGTGTCACTTCCAGCTCG
ATGCCTCGATGACAAGCACATCCAGCGACCGCGGTGGGGAGCACAGAGCAGCATCCAGTTCACGGTTCACACGCGTGCAA
GCCGGCGCCGGCCAGGACCAGCGCAACCACCAAGCGCGCAGGCGGCGGACGGGACGGAAACACGCGCCGGTCCGAGGCA
GCTATCGATGCCGCCGGCCGGCTGGCTGCAGCGCGCACTGAGGGGCTGTTTAGTTCTCAAGAAATTTTGCAAAATTTTTCAG
ATTCTCCGTCACATCGAATCTTTGACACATGCATGAAGCATTAAATATAGATAAAAAATAAAACTAATTACACAGTTTAGACG
AAATGGACGAGACGAATCTTTTAAACCTAATTAAATCATAATTAAACATTAATTATCAAATAACAACAAAAGTGGCTACAGTA
CCATTTTGCTAAATTTTTTGCAACTAAACGCTACCTCACTAGTCCTAGCAGTAGCAGGCCCGAGCGGCACAAGACGGCGTCA
CGGGTGCCGCCACTGTCCGTGCCGTCTCGATCCCACACCAGCACCACACTCCACACTCCCCGCCATTGTACCCGCGCGCATA
AAAACAGCTCACTACTACCACGCGCACATTCCCACCTCTTCCTCCCGAAATTTCCCACCCCCGAGAAGCAAAACCACGGCC
CGCAAAAAGCCACCGTCCGCGCCAGCCAGGGAACAGAAAAGCTCCAGCTCCAGCAGCTCAGCTCAAGCTCCCCCAATGCC
TCGCCCGTAGCCGCGTCCGGAGCTCCACCGCACGTCGCAA
>SsLRR-RLK191
CTTCGGGGTCGGCTGCAACTGCCGGAGGTGAGGTGAGGAGGTGGATCGAGTTGCCTGACCCCGGACGCGACGATGACCAT
GCCATGCCTCCTCTGACCTCTCCGTCCGCCCAGGCCCAGCCGAGCTCAGCCTCATCAGGCCCTCACTCAGCTCCCCGCCCGG
CCGGTGCGCGGTGACGGACGGATGGGAGTGGAGTTGGTACGGTTTTTAGATGCGAGCAAGCGACTGCGACGCGAGCCGAT
CTGGTTCTAGGTCTCGCGGGTCGGTCTGTGTGGCACGTACGCCGCCGGCCGACCAGCACTCATCCAGATCCAGGGGCAGGC
AGGCAGGCCTGACAGCGGGCTTCATGCATGGCGCCATCAGGCCGCCCGACGTACGGCGCGGACCCGGCCCTCGAAGGCAA
GACGGCAACAGTACTGAACCTGATGATGGCACACTTGAAAACAACGGTGTTGCATCGCATTGCATCGCATCGTGTCCCACAC
ACTCCCACCCACGACGCTCGCAACGCGCGCTTGCGGTCGACGTGCCCCGTACTCGCTTCCTCTCCAGGCCTTTTAATAATTA
GCGCGGGTACGGTGTGCATGGTCGGACGGACGGCTCCAGAGCTGATTTGGTTTGGTTGGTCATCCAGGTGGTGCTCGCGGT
TCGGAACCATCGAGGTCATCACTCATCACCACCACGTTGCTTCTGGCTCGCGCTAGCTTTGTGTGTGTGGCCGCCCGTGGTG
TGGGTGCGTTGGTGGCTAATGTGCTACCGGTTGCTCGCCTAATCCAGACCCCGGCGGACGGACGGAGACCGGCGAGCTTCC

GCCTCGGCCTCGAATATTTATTTGACTGGCCACATTTGGCGTAAACCGCCGGTTAACCGTCGGCGACGTACGTGCTGTGCTG
GCACGAGGAGCGTGTCTCTGTTGGCACGCGGTTATCCGTTTCGTCCCGAGATTTCTTTCTCGGGGCCAAGCAACCGCAGATT
CGATTTATGGTGGTAGAATTTGTGCATCAATTTGCTGGCAAATAACATTCACTTGTTCCTTAGCACTGCCGAAAGCGTGCGGT
GGCCAGGAAAAGAATGCACGCCTAAAAGTAGAGCTCTACAAGATACCATACCAGTCGGTACAACTGAGTTTTCGCTTTATTA
TTATTATTATTATTATTATTATTATTATTATTATTATTATTATTATTATTATTATTATTATTATTATTATTATAATATATTTCTATATTCC
TATAGGTGCAGAGTAGTAAGATCATTTTGTACCCAGGCCTCGTTTAGATCGTGAAAAGTTAGATTTTTTCTAACTGTAACATTT
TCGTTTGTATTTGATAATTAGTGTTTAATCATAGACTAATTACGCACAAAAGATCCATCTTGTGATTTATAACTAAACTGTGTAA
TTAGTTATTTTTTATCTATATTTAATGCTTTATATATGCGTGCAAAGATTGATGTGACGAAGAGAGAGTGAAAAAAATTAGAATT
TGGAGTGTATCTGAACAAGGCAAAAAAAAGTATGGAGTACGGCATGTTTGCATGCCAACGTGTCACTTCCAGCTCGATGCCT
CGAAGACAAGCACATCCAGCGACCGCGGTGGGGAGCACAGAGCAGCATCCAGTTCACGGTTCACACGCGTGCAAGCCGGA
GCCGGCCAGGACCAGCGCAACCACCAAGGGCGCAGGCGGCGGACGGGACGGAAACACGCGCCGGTCCGAGGCAGCTATC
GATGCCGCCGGCCGGCTGGCTGCAGCGCGCACTGACTACTTAGCAGGCCAGAGCGGCACAAGACGGCGTCACGGGTGCCG
CCACTGTCCGTGCCGTCTCGATCCCACACCAGCACCATACTCCACACTCCCCGCCATTGTACCCGCGCGCATAAAAACAGCT
CACTACTACCACGCGCACATTCCCACCTCTTCCTCCCGAAATTTCCCACTCCCGAGAAGCAACCCCAGCCCGCAAAAAGCC
ACCGTCCGCAGCAAAAAAAAAAAAGCGGC
>SsLRR-RLK175
ATCAGGCCGCCCGACGTTCGGCGCGGACCCGGCCCTCGGAGGCCAGACGGCAACAGTACTGAACCTGATGATGGCACACTT
GAAAACAACGGTGTTGCATCGCATTGCATCTGCATCGTGTCCCACACACTCCCACCCACGACGCTCGCAACGCGCGCTTGC
GGTCGACGTGCCCCGTACTCGCTTCCTCTCCAGGCCTTTTAATAATTAGCGCGGGTACGGTGTGCATGGTCGGACGGACGGA
CGGCTCCAGAGCTGATTTGGTTTGGTTGGTCATCCAGGTGGTGCTCGCGGTTCGGAACCATCGAGGTCATCACTCATCACCA
CCACGTTGCTTCTGGCTCGCGCTAGCTTTGTGTGTGTGGCCGCCCGTGGTGTGGGTGCGTTGGTGGCTAATGTGCTCCCGGT
CGCTCGCCTAATCCAGACCCCGACGGACGGACGGAGACCGGCGAGCTTCCGCCTCGACCTCGAATATTTATTTGACTGGCCA
CATTTGGTGTAAACCGCCGGTTAACCGTCGGCGACGTACGTGCTGTGCTGGCACGAGGAGCGTGTCTCTGTTGGCACGCGG
TTATCCGTTTCGTCCCGAGATTTCTTTCTCGGGGCCAAGCAACCGCAGATTCGATTTATGGTGGTAGAATTTGTGCATCAATTT
GCTGGCAAATAACATTCACTTGTTCCTTAGACTACCCGCAGTGGATACTCTAAAATAATACTCTAAAATTTTAGAGTATGAATA
TAGAGTAAAAACTGCTGCACTGGGTACTCTAAAATGATACTCTATCTTTTCTCTTACTCTATATTTAGAGTAGTACAGTGCTTA
CTCTATACTCTATAGTGGCAGGCCCCACTAGCTAAACAAAGGGAAAAAATGCTATAACTCACAAACCAAAAATCCAAATTAA
ATTTCGATTGCACCATTAAATTCGTCTCAATAAATCCTTCAAAACTAGATCACATATGGATATATTTAGACAAAGTGACAAGTG
GGTCTCATAGTGGATAGAGTGGATAGAGGATGATATAGAGGATGAATGAGTGCGGGAGAATTGAGTATAGAGGAGAGAATCT
TGCTGACTATGACGTATAAAAGATGAATATAGAGATCTATGAGTGCGGGTAGTCTTAGCACTGGCGAAAGCGTACGTGCGTGT
GCTTAAAAAGGAACCCGTAGGCTAGGCTCCGAAGTCCGATCCAATGAGGACTCGTGAGAACGCGTGACGGCAAGGGCGAT
GTGTAAAAGCAGTGATCAATATGGCCAGGAAAAGAATGAACGCCTAAAAGTAGAGCTCTACAAGATACCATACCGGTCGGT
ACAACTGAGTTTTCGCTTTATTATTATTATTATATATTTCTATGTTCCTATAGGTGTAGAGTAGTAAGATCATTTTGTACCAAAAA
AAAGTATGGAGTACGGCATGTTTGCATGCCAACGTGTCACTTCCAGCTCGATGCCTCGATGACAAGACCGCGGTGGGGAGC
ACAGAGCAGCATCCAGTTCACGGTTCACACGCGTGCAAGCCGGAGCCGGCCAGGACCAGCGCAACCACCAAGCGCGCAG
GCGGCGGACGGGACGGAAACACGCGCCGGTCCGAGGCAGCTATCGATGCCGCCGGCCGGCTGGCTGCAGCGCGCACTGAC
TACTTGCAGAGTAATCCTAGCAGTAGCAGGCCAGAGCGGCACAAGACGGCGTCACGGGTGCCGCCACTGTCCGTGCCGTCT
CGATCCCACACCAGCACCACACTCCACACTCCCCGCCATTGTACCCGCGCGCATAAAAACAGCTCACTGCTACCACGCGCA
CATTCCCACCTCTTCCTCCCGAAATTTCCCACCCCCGAGACGCAACCCCCGCCCGCAAAAAGCCACCGTCCGCGCCAGCCA
GTGAACAGAAAAGCTCCAGCTCCAGCAGCTCAGCTCAAGCTCCCCCTCGTCCCTTCCCTTCCTCCTCCCCCAATGCCTCGCC
CGTAGCCGCGTCCGCAGCTCCACCGCACGTCGCA
>SsLRR-RLK154
AAGGGAAAGAACAAACGGGGAAAGGGAAGGCTGTGCCTGTGCGGTGGCAGCCGCCGGCTCAGCTCAAACTCAACCACAC
GAGTAGGAGGAGTACAGTACTTGGTGAGTCCGTGGTGTTCCGACACGGACACGACGACTTGGCATGCTAGCTGTGCTGCTA
TCGAGGCGGCATGCAGGCGCTCAGCCTATTCGAATCCGGGCACCGGGCGGTTCGTGCACGATGCATGACACGGTAGCAAAA
TTAAAGTCGACCATTGCTAGTTCACTCCTTTTCTAAACCAACAAAACAGCACAAGAAAAAAAGAAGAAGAATCGACCATGA
TTACTCAATCCCCACGTCCCAACCGGTGGCGACGTACAGTATCAGTACATGTACACGCATGTGCCTACGTTCTCGTCCATCTG
TACGTACGTGTGGTCCACGTACGTACGCCCATGCTGCTACGATTTATTATATTTCTGGTATCTGAATTTTTAGATGGTCGATTTC
AGACGATAATCTTACGTACAATCAAAACCAGAGACGGACGGACCACACAGGACGGGAGCACCGTACCGACCACGCAGCAC
AACAGCACGGTACCGTAGCACTAGCTTAGTTTCCGTGCCGTTGTTCGTATGTGCGACAAGAGTTTATAACTACTACTAGTACA
ACAAGCTGCAAACATATCATTTTTTTATTTAAATAGAAAAAAATACATTTTAATTAAAAGTGAGTCTCGTACGTATATTTTTAGC
TTTATTTATCGGCTCCTTTAGCAAACAGAGGTTTAATTTAAACTGGGCCAAGCAAGTTCTTCTTGTGCCTACTGCTTCCGTGC
GCATACAGTACAGCGCGGTGCAGGGAAAGACAATGCATGATTGCCCCGTGATGGTATGCATGCCAAGTTGCCAACCCAATGG
CAGTGGCAGAGTCCCACAAGCACAAGCAAAGCATGGCCAAACAAACCACGGGCTGCAGGCAGGCATGGTCATCGGCCAAT
GTCATCTGCCTTGGCTTGGGGTGTTCTTGTTCATTCAAATCAAAGCTTCGATTTTCAAACTAAAATGAACTTGACCAAAAGG
GGCAAAGCGAAAGCCAAGTCAAGCTGCCTGCTTCTGACGTGCTGCGTGCACTCATCATCATCGCCCACACACACCGACCGA
CCTTTTTTCTTTCATGCGCTGCTGGGCTGTGCCTGTGCAGTCTGCAATTCGATTCGATCCATTCACAGCCAATCGCTTTCTTTA
CTTCTCCTCGTTCCCGCCGACCTAATCCGAAGTTGGCCAGCTTCTACTGAGACTTCCGTGTTCACTGACGAGACAGTGGTTA
GAGTACTAATCCTTGTCATGCATGATTGATCGACGGACGTGTAAAATGTTGGAAGTCCTGTACAGTACTTGGGACTGGACAC
GTGTCAAGGGGTCAGGGCCTTGTGTTCAGTTGAATGGCTCGCTGAAGCTTCAAGCCTATGTGCTAGTCACATGATCAGATGG
ATGGTTAAGGTAACCACTAATCAGATGGGATCACCAACGTAGGAGTATTACTGTAGTATTCTTGCGGCGCTGACTGTCACAAA
CTCACAACGGAATCTCAAGCAGATGGGTCGCTCAACCACTGTAACAAGAAATTCTCGCAAAACGAATTCAGAATTCTATTCA
CGTTCCTGTTGGATCGCCACACGGTGACGGTGAGACGGCCACCCACCAGCAGCGCAGGGAGCAGGCTCACATGGGAATTG
AAGGCGCAGATCGCTGACCGTGCCGCCGCCGTCACTTGCCGAGCACGCACCGCCACTCCATTCCTCTCTCCCCTCAGTCAC
GTGCGAATGCGAGAGCCGCGGCAGGCAGCTCAGCTCTGCTCCCCAACCGTCCCCAAGAATCTCGCTCGCTCTCGGCGGCGT
CTCCGCTCTCCCTCACTCGCAAACACGCGGACGCGGCCTCCCTCGTCCCTTCCTCTCTCTCCATTCCGCAGCGTTCGTTTCGT
TGCTCGCTCAGATCCATCCAAGCTGGGGTGAGCC
>SsLRR-RLK182
CGAATAAATTTGTAATGTTTCTTTACTAGTTTAGGCTTCACAAATATTGTACTCCCTCCGTTCCAAATTATAATTCGTTTGACTT
TTTTTTTTGACCCCAAGTTTGACTACTCGTCTTATTCAAAAAATTTTGTGTAAATATTGTCAAATTTAAGTCATTCTTGAAGAA
CTTCGGTTAATAAAGCAAGCCACAACAAAAGAAGTGATATTTAGCCTATTTTTTTGAATAAGACGAGTAGTCAAACTTGGAA
TCTTAAAAGTCAAATGAACTAAAATTTGGAACGGATATTAATTAATTGTACTAATGTACTATGTTCTAGTACTGTTTGCTTTGA
ATTGATAGATATTTAGCTTTACATATGTCACGATGGTTTGAATCTGTATATGCATATATGTATGCATATATGTAGTTTGTACACCTA
ATTTTCAATTCCTGCGTCCGCCGCTGGCTGTGCCTGTGCAGTCTGCAATTCGATTCGATCCATTCACAGCCAATCGCTTTGAG
CCAGTTTAGTTCCTAAAAAGAAAACTCAAAAAGTGTTACAGTACCCGTCACATCAAATCTTGCGTCACATGCATGGAGTACT
AAATGTAGACGAAAGAAAAAACTAATTACACAGTTTAGTTGTAAATCACGAGACAAATCTTTTGAGCTTAATTAGTCTATAAT
TAACACAAATTGCTAAATAAAACAAAAAGTGCTACAGTAACCGAACCTAAAAATTTTCACCAACTAAACATGCCCTTTCTTT
ACTTCTCGTTCCCGCCGACCTAATCCGAAGTTGGCCAGCTTCTACCGAGACTTCCGTGTTCACTGACGAGACAGTGGTTAGA
GTACTAATCCTTGTCATGCATGATTGATCGACGGACGTGTAAAATGTTGGAAGTCCTGTACAGTACTTGGGACTGGACACGT
GTCAAGGGGTCAGGGCCTTGTGTTCAGTTGAATGCAAGGTAATAAATAGCGTATAGCGGGTTGATAGCGGATTGGCGTTGAG

GTAGCCGATTGCGGATAGCGGGCGATATTGCGGACGATAAGTTCCAATAATAGCACCCGATAATCCCATATAATTTAAGTACA
TATATCATGAATAATGAGTATAAATAATTTAAGTGCATATATCATGAATAATTTTAAGTTCCACAACCGATGTGTGAGTATTTGA
GGGCGTGAGTCATAGTGGGCTAGACTAATTAGGGTTATTTGCTTTCATGCTATTATGGCCTGCTACGCGCAATTGCAGCTGCA
ATATCAGCCCGCTATTCAGAATTGCGACCACAAAGTTTCAGAATGCAAAGTTCAAAGCGCTATCTCGTAGCGGTAGCGGCAA
TGGACCGCTACTGCTATTGCGGATACAATTACGGCCGCTACCACCGCTATTTATTAGCTTGGTTGAATGGCTCGCTGAAGCTTC
AACCCTATATGCTAGTCACATCATCAGATGGATGGTTAAGGTAACCACTAATCAGATGGGATCACCAACGTAGGAGTATTACT
GTAGTATTCTTGCGGCGCTGACTGTCACAAACTCACAACTCACGAGTCACAACGGAATCTCAAGCAGATCGGTCGCTCAAC
CACTGTAACAAGAAATTCTCGCAAAACGAATTCAGGCCTGTTTAGCTGGTGAAAATTTTTACGTTTGCCTACTGTACTACTTT
TCATTTTATTTAGTAATTTGTGTCCAATCATGAACTAATTAGGCTCAAAAAATTTGTCTCGTGATTTACAACTAAACTGTGTAAT
TAGTTTTTTCTTTTATCTATATTTAATACTCCATGTATGTGCCGTAAGATTTGATGTGACGAGTAGTGTAGCACTTATTAGGTTTT
TTTTTTGAACTAAACATGCTCTCAGAATTCTATTCACGTTCCTGTTGGATCGCCACACGGTGACGGTGAGACGGCCACCCAC
CAGCAGCGCAGGGAGCAGGCTCACATGGGAATTGAAGGCGCGGATCGCTGACCGTGCCGCCGCCGTCACTTGCCGAGCAC
GCA
>SsLRR-RLK167
CTAATATTAATAATATTTTTCAAAATCTAGTTAGCCTTAAGAAAATTTGAGTGCAATTTTTTTGGGGACGGAGGGACTATAGGA
CAACTTGAACCTGATTATTAAGCCTGTTCAGATAAAACCAAAAAAGTAGTGCCCCTCAAGTTAATTATTGACACGGGCTTTTG
GCAGCAAAAATAGGCCCTAAACAAAGTGGGCCTATCCCGAGCCTGGCCCAAAGTAGTGTGGGCTGACACAGTTGAGCATGT
CTGGTGGGCATGCATGAGCAGAAATTTTGGGTCCAAAACGACCTGTTTTCTTTTTTCCTGGGCCCGGTTAAGAAATTCTTATT
TTAGAGCAGCAGTTTTTGGTTCATGTTTTTCAAAAAAAGAGAGAGTTTTTGGTTCATAATACTTCCTCTGTTGTACGCCGTCA
TACAGTTTGCTACTGCTAGAGCATCAGCAGCTGGTATCATGGCTCACCTCCAACATTGCATGCTAGCTGGTATCACAGACTTA
CAACTAAATTTGCTTTGCTAAACGGCCAATAGGGTGGTCTCAAGGTGTAGTGTTATAATAATGTTATTTCCTTGCAGTGTGTAG
TGTATGCAGCTGATATAGAGTTCTGCAAAGTCATTCAGTCTTGTACCAGTTTTCAGTACTATAGTTATTTTGTTCTGAATAGTGT
AGTTGAATAGTTGGTTAGTGAAGGCATGAAGATCGGGAGATGTACTGCTCCATAGAATACATAGTCGAGGCTTTGTGCAACA
TGTTTCCATTTTCTGTTTGTTATTGTTCGTTGATTTGTTGCGAGAGAAAAATATTGTTTCTTTGCTGAAACAGTACGGCTCATA
AGACAAGCGAACAGCGAAGCACAAGGATTTCTTCTTTTGTGCTTGACTATATGCTAGGAATAGGAGTGAATTTTTTACTAATA
CTACTGGCCTGTTCCTAAGCTAATAATGTGGCAACTTTGTTCTAGACTGTTTTCTCCAGCAAACAACACAGTTCAGCGTAGCT
AAGCCAGTGATGCAAAGAGTAGCAGTAGTCGGTGATGGAGTAGCAGTATAGTCGGTGATGTAAACCTGGCTTGACACTTCTC
TTTAACTGTGTTATATATCTCATCGAATTTCAGTATGGGGTGTTGGATGAATCATATAATTCATATATACTAGGGTCTGTGGCTCT
CCAAGGTGAAGAATTTGGTCTGATGTATCCTTTTATGTTGTAGTTCTTTTTATTGTTCCTATAGCTCAGCTAACTCTAAAGATGA
TGGTGACATCATGGCCGAGGATTGATAATTTAAGTACACGCATGACAGACACTGTTTGGACTGAGGCCTGAACCTAGGCATC
TAAAATCGGACACCGAGAAACCTGCCGGTCCAAGCTGCTTTTTATAGTTGCTAATAACAGGCCTAGAGTTCTATTTTTTAAGC
TGCTTTTTAAAGTTGCTAACCAAACAGGCCTGGAGTTCCATTTTCCAGTAACTACAACAAGTTAGGGGCTAGATGTTGTGCG
GTAGCACAACCAATAGCATTATAGAAATAATGTCATGTTAACTTCAATTTATCTTTTAGGTCTTGTTTGATTGGAATCCAATCCT
TCGAAATATTACTTGTAGATGAAAAATTAGTTGCAAACTATTTCACCTCATTCCATCCTAATATGTTAGGATTGGGTGTAGTGA
AGTACAACTAATCAAGCCTTCAAAAATTTTGTAATGTATTGTAGTGTACTTTCGATTGGCATATTGTTACTTGGGGATCAAATT
TAACATGCAAACTTGTANNNNNNNNNNNNNNNNNNNNNNNNNNNNNNNNNNNNNNNNNNNNNNNNNNNNNNNNNNNNNNNNNN
NNNNNNNNNNNNNNNNNNNNNNNNNNNNNNNNNNNNNNNNGTATTTAGGTGACACTATAGAAGCGGCCGCGACAACTTGT
CCACGTGGAATTCTAAGCTTCAAGCCTGCGTGCTAGTCACATCATCACATGGATGGTTAAGGTAATCACTAATCAGGTGGGAT
CACCAACGT
>SsLRR-RLK195
TAAGAGTTAAAAGGCCATGTTTTCTTGTTCCTCCCGTTCACCATCTAGCTGGCCCGATCTGCTAGCCCAAGAAGGGAAGTCA
CGGAGGGAACCAGAGCCTGAGACGCACCTCTCCCCGTCCGTGGTCGCGTCCAGCGTTCGTCGCCCTCTGGTTCGACTCGAA
CACTAATCTGCTGATTTGCATGTCATCATTCAATCCCAAAGATTGCTGATTTGCATGTCATCATTCAGTCCCAAAGATTCATCT
GCTTCTTTTGACAAGGACAACTCAGTAAAACTCGTAAAGTTTTATCCCAAAGACTTCCCAATCACTGACTTGAGGTGTCTAA
CCTATCAGCTTGGTAACTTTATTGCTGATATACGTGGTGATGAAAGATTTAACAAAGTGAAAAATATTGCCGAGCTTTCAGCT
TTACTTGTCGAAACAAATAAGCATGTCTTACACTTGTATGTCTACAAGCTTCTGAAACTAGTCTTATTGCTACCGGTTGCAACT
GCAAGTGTTGAAAAAGCATTTTCAGTGATGAACTCCGTAAAGAACAAGTTAAGAAATAGCATGGGAGATCATTACTTGAATG
ATTGTCGGGTTACGTTCATTGAGAAGCAATTTTTTCTATGTGTCGCTGATGAAGACATCATCTCTCGATTCCAAAAGCCGCCC
TGTCGAATAAATTTGTAATGTTTCTTTACTAGTTTAGGCTTCACAAATATTGTACTCCCTCCGTTCCAAATTATAATTCGTTTGA
TTTTTTGACCTCAAGTTTAACTACTCGTCTTTTTAAAAAAAATTGTGCAAATATTGTCAAATTTAAGTCATTCTTGAAGAACTT
CTGTTAATAAAGCAAGCCATAACAAAATAAGTAATATTTAGCCCAAATTTTTTGAATAAGATGAGTAGTCAAACTTGGAATCT
TAAAAGTCAAACGAACTAAAATTTGGAACGGATATTAATTAATTGTACTAATGTACTCTGCACTAGTACTAGTACTGTTTGCTT
TGAACTGATGGATATTTAGGTTTACATATGTCACGATAGTTTGGTTCTGTATATACATATACGTAGTCACCCAATTTTCAATTCCT
GCGTCTGTGCAGTCTGCAATTCGATTCGATCCATTCACAGCCAATCGCTTTCTTTACTTCTCCTCGTTCCCGCCGACCTAATCC
GAAGTTGGCCAGCTTCCGTGTTCACTGACGAGACAGTGGTTAGAGTACTAATCCTTGTCATGCATGATTGATCGACGGACGT
GTAAAATGTTGGAAGTCCTGTACAGTACTTGGGACTGGACACGTGTCAAGGGGTCAGGGCCTTGTGTTCAGTTGAATGGTTC
GCTGAAGCTTCAAGCCTATGTGCTAGTCACATGATCAGATGGATGGTTAAGGTAACCACTAATCAGATGGGATCACCAACGT
AGGAGTATTACTGTAGTATTCTTGCGGCGCTGACTGTCACAAACTCACAACTCACAAGTCACAACGGAATCTCAAGCAGATC
AGTCGCTCAACCACTGTAACAAGAAATTCTCGCAAAACGAACTCAGAATTCTGGTCACGTTCCTGTTGGATCGCCACACGG
TGAGACGGCCACCCGCCAGCAGCGCAGGGAGCAAGGCTCACATGGGAATTGAAGGCGCAGATCGCTGACCGTGCCGCCGC
CGTCACTTGCCGAGCACGCACCGCGGTAGCCGACACCGCCGCGCTCCATTCCTCGCTCCCCTCAGTCACATGCGAATGCGA
GAGCTGCGGCAGGCAGCTCAGCTCTGCTCCCCAACCCCAAGAAACGCACCAAAAACGCCCTCCCCGACCCCAAACTCCTC
TCGCTCGCTCTCGGCGGCGACTCCGCTCTCCCGCACTCGCACACACGCGGACGCGGCCACGCTAAGCTAGCAGAGCTACCT
TTGGCGCACCTCTCTCTCGGCCTCCCTCCCTCTCTCCATTCCGCAGCGTTCGTTTCGTTGCTCGCTCAGATCCAAGCGAGGAG
AACGAGCTGGGGTGAGCCA
>SsLRR-RLK145
AGCAAGCAACTAATTAACCCTAACGGGCTAAGGCCCAATAGGCCCTTGACTCTTCTAACACTACACCCCACCTGGACATGCA
GCTCGTCCTCGAGCTGCAACTTAACGATGACTCTGCTAGACATAAACTTAACACCTAAAAATAAGCCTTTTACATCTCGGCTT
ATTTTATTGACCTGAAATAGATTGCGACTCTTTATTTTTTATTTTTGGAGATACGATGGACACCCTCCTCGTAATGGACATGCAC
GTGTATAGCCACCTGGATCCCATGGAGCCCACCGGAACGAGAGGGTGCACGGGTATAGCCACCAGGAATTAGTCGCGATAG
CGAATAGCGGAGACGTCCTCGCGGCGGCCGGTGCCGGAGCACGGCAGCGCTAATTAAGGTCCGCCTATGGGTGGCCACACA
CCCGCGTCCCCAAACGCCGGCGCTGTAGACACCGAGTTCGCCACCCGAGGTGGCGATGCCACGCCCGATCTCCATGACGCA
GCGGACGAGATCGGGGCCCTCCATGTGGCGAAGCGTAGCCTGGTGGCGAAGCAAAGAAGCACGGGGCACAGGCACCAGCT
GCAACCCACGCATCTCCCGCGCCTGCCGTCGCGCCAGGAGTCCGCGTGCTGCAGCTGTAACCGCACCGCCGCCAGGACCTG
GTGGGTATGCATCTTTGTCTCCTGCTCCATCGTCTTCTTGAACTGTAGCAGTTGCAGCAACTCATCCGCCTTGGCTTGCATCT
GGGCTATTGTGCTGCTCATGTAGGCGTACAGATCCTTGGGTGATGGGACAATCGCCGCAGGAGACACTCTCGCCTTTGGGAG
TCGCAGGGCCATGTCTTCCCGTAGCACAGCTGCTGCTGCAGAGAGCCGAGCTGCAGGGGTGACATGGCGCTGGCGCTTGAA
GAGGGGGCGTTGCCCACGCCGGAGGAAGGATCGACAGCAGCAAAAGCAGTGCTGTCGATATGAAGCAGGTGATCGATGTT
GGAACAAATCGGCAGCGGAACCTGGCATTAGGGTGGTGGTGTAGGTCGGCAGCGCTGTCGTGCCGCACCCAGGCATCCAGT
AGGGGAAACTCTGGGGCACGGCGCCCGCCGAGGACGACGCCTGATGGATCTCCATAGTGGCCAGCCGTGAGTTGAGGTGC

GCCTGCTGCGCGTTGATGTCGGCCAGCGCCTTGGCGATCTGATCCCACAGCGGCTCATCGAAACCTGGCATCTCTGATACCA
GATTGTTATGGACGTCGTTGGGATGGGGAGAATTGAGGAGGAAGAGACCGACGGTGATACAGTACCCGCGCGCTACAGTAC
CTCGCGGGTACTGTTCACGAGCTAGAGGCAGAGACCCGGCGGCTAGGCTGTGAGGGGCGCTGGGAACGCCGGCCGCGGGG
CTTTGCCCACGGCCGGCAAGAGAGAAGGTTTTTCCTTCTAATCTCTTGCTTGATTAGATTGATACATCTCCTCTCCTTATATAG
AGAAGAGGTCTTACTTAATCCCTAAGCAAGTGACTAATTAACCCTAACGGGCTAAGGCCCAATAGGCCCTTGACTCTTCTAA
CACTACACCCCACCTGGACATGCAGTTCGTCCTCGAGCTGGGCTAAGGCCCAATAGGCCCTTGACTCTTCTAACAGTCCCAC
TCCCCACCTATCTATTTTTGAAATATCTTTGTATTTTTGGGATCCAAGTATTTGATGTACTATTTTCTGCAATATTGCCAACATGT
GCAAGATGTGCTCCTCACACTTTTTCTCAAATTTCTCAGGTTCTCTTAATCATCTTTATTTTTCTTGTTAGGAAGTTAGTGATG
AAAGTATATCAGAGGGCACGAGCTTATGTATCATGCTTACTATCCTTATTTCCTTCTTTTGTATGAACTATGATTTTGTTGTTATG
CTCTATTTTCACTAACTTTTGTTTCTTTTCTATACTGCTAGAGGATTGAAGTTATGATGGAAAAGTTTGTATAGTAGCGCTGAAT
CCAGTTCTCTTCAAGCAAGTCTCAACC
>SsLRR-RLK353
GTCTTTAAAAAAGGCAAAGAGAATGCACTGGAATTGCGCTTTTGTTAGTAGTAGTAGTAGTTGAGCATTTCGAATCTCTACA
GACTGCACGGCCACTGTTACCTTCCGTTTATTGGTGTGCTAGTGATTAGTGAAATACTGCATTCATTGTTTTTACGGGAAAATA
ACAGTGTGGTACTGAACTAGTATGATTTTGCACTTAACGTTCGTGTAAAAAACATGCAACTGTTAGATAAACAAACATCAAC
TTGAAAATCATGCAATGGAGCAGGTCAGGCATGTAGTATACTGAACTGCACCTTGCAGCAACATTTATGGGCAATTCTCAAG
CTTGATGCATCCATGGCCCATGTGGTAGGAGAACATGGAAGTGTGAACTCTCAGTCATCTTTTTTTAAAGAGGAGCTCTTACT
GATCCTACCGGTACATCTACTGTTGCAATTGTCTTTTGAGGTGTCGAGATAGTGATGGATCTTGCCTTCGTTGTGTCCTATGGT
GTGGAGCCTGAGATCCCTCCTCACGTCCTCAATGTGCCGTGCTGCCCATGATCGCCCCCATAAACGAGCAGAGCCCCACACC
CCTGCCTGCAAGCCCTCTTCCCTTTGGTTTCTTGCATCTGAGACCACCTGCTTTACGTGACTGCCTTTTCCCAAAGCATTTTG
GTCCATGAGAAAACAATGGTATTAGGTGTAGTTATTGGGTCTAGGTTAGGTGCCCTGCAATGCTGCTTTTGTCTAAGTTACAC
ATGTGCAAGAGAGTCCACCAACGTGCTGTGCCTCACCCTCACCTACCAGGGGTAATGTTTTGTCGCCATCCTTGTGTCTGTT
CTGGTTATGACCAGGAGCCATTGTTTAGGCGTGGTGGTTGTTTTGTTAAACACAGTGGAGTTATCCTTTTCAGAAAAGGCTA
AAGAGTGATGGCTACTAGCTAAAAGATGTGTTGGTTTCCTTTTATATGGCACATGATCCTAGCTGTCCTTTTCATAAAGAGATT
TGAGATAAACCAGTGGATTCGTTTGGGTTCGGGCCTTTCGGTTGAGGCTAAGGAGAAGACAAGATGTGTCATTCTGGTTTCG
TTTCGTTTTACTATTTTCAGAGAGGAGATTGCTGTGTAGCAGTTGAGGAATGTGATGGTCCCCTTGAAACATCAATGTGGGCC
GCACTTTGTCATTGTCTCATGAATTTACGATTCATTCCAAGAGTGACCTTCCACCAGCCTTCTCTCATTGACGTGAATTAGTTA
TGATGCAAAGTATAGGATCCTTGCTCATTATGATGAAAGATGGGCAAGGACAGTGGCAATTAAAATATGCTGGAATCTCCTAA
GAAAGTCGTATTTATATTTTACTGTCTGTGTAAGCTGCTTGTGTTTCTGTTGGAGCTGATGCACCAACTTCGTAAACTATGGGT
CAAACTCTATCGGCCTCACAATAGTGGCCATTCTTGTAAGTATATTTGCTATTATTCTTTAGCAGGTGGTACGAGAAAACTTGT
TTTTTTAGCATGATCGTGAATGTGATATTTAACGAAGGAAATGCCAGGTTTCACATTTAAACTTAAGAAGTGGTAATGCTTCTT
ATAAGCTGTTAATTGATGAAATTGCATCCGATTGTACATTAGAATTGCTCAGCTTTTTTGGTCCTATCTAGATGATCGAAATTAT
AAAATGAGTTACTCCTCCTTTACCTTCTGTGGTTTCCTGTAGCTGTTTACTTGTTTGTTGCAGAGTATATAAATGTCCACCGGA
AAACTTAAATCTTTTATGCTTGTTATCCACATTCACATTCCATGTTCTATGCTTCCACATTCACATTCCTTTCCTTTCTACCATGT
TTCTATGCACCGAACTGAAATTTTGACTGTTCTTATTTATTCTTAAATTTGGACCATTATGTATTGCTGACTTTTGTTAGCCTTTT
ATGTGTCAGGAGTGATGCACATGCACTGATGAGGACTCTTGGATTATAACTGGTTTCTTCTTCTTCCAGCCAATCCCAACAA
>SsLRR-RLK376
ATACGGGCCGCATGGGAAGTAGAACTAGAAAAAGGAAGACGAACATGTCGGCCCAATTGGCACGCATGACACAGTTGCTCA
TCATGAGCTCGAGTACATGGAAGACCGAGCTACGACTGAGCTGTATCAGGGCATCACGTCCAGGATGGCCGAGGCGCCGGT
GCCAGGTAGTAGAGGAAGTAGAAGCGACGAAGGCAGCTGACAAAACGGGCGGCGAAGAAGACGAAGGAGCCGCCGGGA
AGCGAAGGGTGTAAAGGGTCCCGGTGCTGTCACATCGGAGCAGAGGACGCCAAGAAGCCAAATCCTTTACAGTAAGACCA
GAAGAGTCAAATTCCACAGAACAAGAATTGTCAGCTGTAAAACGTCGAATAGAAAGAAGATTATGAACCATAGAAGGAGCA
ACAAGAACATCAGGAAGACGAAAGGAGCCATGAGCATCGGCGGTACCCACAAACGAGACTGGAATACAAGAACCATTTGT
GACCATAATGGACGAAGGAAGAGAGGAAGAGGAGAGTGAATAGACGAAAGTATACCGGGGTGAGGAGTAGTGTGGTAGGT
GGCCCCCGAATCAGCGATCCACTCGGGTCCGACCGGAGGTGTCAGGCCCATGGTGCTGAACGAGCGAGCCAGGGCCGCCT
GGTCCCAGCCGGTGACCCCCGGTGGAGTCCCGGAGGGTGGAACCCACGGCGTAGCAGGGAAGGCGCCGGGTGGAGGACC
AGCAGGAGGAGCTCCGGTGAGCATGGCCACCGGTGGACGAGTGTCGGCCCGGGGGCGTGGTAGGGCCACATGGAGATGCG
ACCGGACCAGGGTTCTGGAACGAAGGCCATGGTGCCCCCCGTGTGGCCCAGCCGGTGCCGGAGCCTGGGGGTGACCCCCC
GGCCACCCCCGCGGCCCCTCCACGGCGACGACGAGAGCCACGACCCCCACCTCCGCTCGGCCCGGAGGGAGGAGGGCCCA
GGAGAGACGACGGTGGCGGAGCGGCAGCCGCGGTGGAGCAGGAGGACGCGGTGGAGTAGCTGCCAGAGCCGTCGAGGAC
CCCGGAGAAGCGGCAGACCCGGCTGGATCCCTGCGTGAGCTCCTCCATGACGAGATCGTCACGGACCTCCAGGAAGGTGG
GGAAGGGCCGCTGACGGTGATCCACGTCCGGAGGTGGGCATACCGGTCGCTGAGCCCCGAAGACGTTGAGGACCAAGATG
CGATCCTCCACGGGCCACCGAGGTCCCAAGGGAGTCCGCCATGCCCTTCATCCGACGGCAGAACTCGCCGACGAAGAGTCA
CCCTGGACGAAGGTGCGGAAGCTCGCATCGAGACGCAGAGCCCGGGCCTCGCGTTGCCCAGGAACTGGCCCTCAAGGGCA
AGCCACGCCCGGCGCGCGTCAGGGGAGGTGCGGACGAGGTCGTGGAGGTCCAGAGAGATCGTCCCCAAGATCCAGGAGA
GGACGATGCTGTCGAGACGGAGCCACGACGGCGTCTGGACGGCGACCGAGGCGTCGACGAGGACGTGGTCGTCGAGGGC
GTAGCGGCGAAGAGTGAGANNNNNNNNNNNNNNNNNNNNNNNNNNNNNNNNNNNNNNNNNNNNNNNNNNNN
NNNNNNNNNNNNNNNNNNNNNNNNNNNNNNNNNNNNNNNTGTACATTAGAATTGCTCAGCTTTTTTGGTCCTATCTA
GATGATCGAAATTATAAAATGAGTTACTCCTCCTTTACCTTCTGTGGTTTCCTGTAGCTGTTTACTTGTTTGTTGCAGAGTATAT
AAATGTCCACCGGAAAACTTAAATCTTTTATGCTTGTTATCCACATTCACATTCCTTTCCTTTCTACCATGTTTCTATGCACCGA
ACTGAAATTTTGACTGTTCTTATTTATTCTTAAATTTGGACCATTATGTATTGCTGACTTTTGTTAGCCTTTTATGTGTCAGGAG
TGATGCACATGCACTGATGAGGACTCTTGGATTATAACTGGTTTCTTCTTCTTCCAGCCAATCCCAACAA
>SsLRR-RLK354
CTTTTGTCTTTAAAAAAGGCAAAGAGAATGCACTGGAATTGCGCTTTTGTTAGTAGTAGTATTAGTTGAGCATTTCGAATCTC
TACAGACTACACGGCCACTGTTACCTTTCCGTTTATTGGTGTGCTAGTGATTAGTGAAATACTGCATTCATTGTTTTTACGGGA
AAATAACAGTGTGGTACTGAACTAGTATGATTTTGCACTTAACGTTCGTGTAAAAAACATGCAACTGTTAGATAAACAAACAT
CAACTTGAAAATCATGCAATGGAGCAGGTCAGGCATGTAGTATACTGAACTGCACCTTGCAGCAACATTTATGGGCAATTCT
CAAGCTTGATGCATCCATGGCCCATGGGGTAGGAGAACATGGAAGTGTGAACTCTCAGTCATCTTTTTTTAAAGAGGAGCTC
TTACTGATCCTACCGGTACATCTACTGTTGCAATTGTCTTTTGAGGTGTCGAGATTGTGATGGATCTTGCCTTCCTTGTGTCCT
ATGGTGTGGAGCCTGAGATCCCTCCTCACGTCCTCAGTGTGCCGTGCTGCCCATGATCGCCCCCATAAACGAGCAGAGCCCC
ACACCCCTGCCTGCAAGCCCTCTTCCCTTTGGTTTCTTGCATCTGAGACCACCTGCTTTACGTTCTGGACTTTTGGTTTCGTG
CTGACTGCCTTTTCCCAAAGCATTTTGGTCCATGAGAAAACAATGGTGTTAGGTGTAGTTATTGGGTCTAGGTTAGGTGCCCT
GCAATGCTGCTTTTGTCTAAGTTACACATGTGCAAGAGAGTCCACCAACGTGCTGTGCCTCACCCTCACCTACAGGGGTAAT
GTTTTGTCGCCATCCTTGTGTCTGTTCTGGTTATGACCAGGAGCCATTGTTTAGGCGTGGTGGTTGTTTTGTTAAACACAGTG
GAGTTATCCTTTTCAGAAAAGGCTAAAGAGTGATGGCTACTAGCTAAAAGATGTGTTGGTTTCCTTTTATATGGCACATGATC
CTAGCTGTCCTTTTCATAAAGAGATTTGAGATAAACCAGTGGATTCGTTTGGGTTCGGGCCTTTCGGTTGAGGCTAAGGAGA
AGACAAGATGTGTCATTCTGGTTTCGTTTCGTTTTACTATTTTCAGAGAGGAGATTGCTGTGTAGCAGTTGAGGAATGTGATG
GTCCCCTTGAAACATCAATGTGGGCAGCACTTTGTCATTGTCTCATGAATTTACGATTCATTCCAAGAGTGACCTTCCACCAG
CCTTCTCTCATTGACGTGAATTAGTTATGATGCAAAGTATAGGATCCTTGCTCATTATGATGAAAGATGGGCAAGGACAGTGG
CAATTAAAATATGCTGGAATCTCCTAAGAAAGTCGTATTTATATTTTACTGTCTGTGTAAGCTGCTTGTGTTTCTGTTGGAGCT

GATGCACCAACTTCGTAAACTATGGGTCAAACTCTATCGGCCTCACAATAGTGGCCATTCTTGTAAGTATATTTGCTATTATTC
TTTAGCAGGTGGTACGAGAAAACTTGTTTTTTTAGCATGATCGTGAATGTGATATTTAACGAAGGAAATGCCAGGTTTCACAT
TTAAACTTAAGAAGTGGTAATGCTTCTTATAAGCTGTTAATTGATGAAATTGCATCCGATTGTACATTAGAATTGCTCAGCTTT
TTTGGTCCTATCTAGATGATCGAAATTATAAAATGAGTTACTCCTCCTTTACCTTCTGTGGTTTCCTGTAGCTGTTTACTTGTTT
GTTGCAGAGTATATAAATGTCCACCGGAAAACTTAAATCTTTTATGCTTGTTATCCACATTCACATTCCTTTCCTTTCTACCATG
TTTCTATGCACCGAACTGAAATTTTGACTGTTCTTATTTATTCTTAAATTTGGACCATTATGTATTGCTGACTTTTGTTAGCCTTT
TATGTGTCAGGAGTGATGCACATGCACTGATGAGGACTCTTGGATTATAACTGGTTTCTTCTTCTTCCAGCCAATCCCAACAA
A
>SsLRR-RLK125
CTGAATCTAGCGTGCTGAGCGAGATGAGATTTCTCTTCATTCCTGGTATGTATCTCACATTTTTCAGCGTGCGTGTCACACCAT
CATGCGCCTTTATCTGAACGGAGCCGATGCCCACAACATGACACGGGTTATCATCACCCATACGCACCACATCTCCTTTCTGC
ACAGATTGATAGGAACTAAACCAATCTCTGTTAGTGCAGATATGAAATGAACATGCTGAATCAAGTATCCATTCATCATTACC
AGATACACAACCAGCAAAAACAAGCAGACAATCTCCAGAATCAGAGTTATCAGCAGCCGAAACAACAGAAGCCTTACCATC
AAAATTATTTTTCCTCTTCTCCTTATTCTGCAATTTCTTACAATCCTCAATGAAATGAGTATTTTTCTTGCAATACTTACAGAAC
GGCTTAGACGATTTGGACTTTGAACGGCCTCTCCCATTGTAACTTTTGTCACGATTGCTGCCACCATTATTAGAGTATCTATGC
TCAGATCTGCCTCTCACATGTAATGCTTCGCCCTTTGAGGACGACGACCCGTCAAACTGCACCATACCTTTCATCTTCTCCCT
GGACTGGAGCGCCTCATAAACTTCCACAAGGGTTAGTTCATCACGGCTTAAAAGTATGGTGTCACGGAAATTAGCATATGAA
TTTGGCAATGAACATAATAGTAGAAGACCTAAGTCCTCATCATCGTACTTCACCTCCATCGACACTAGGTCGGCAACGATCTC
CTTGAATAAGGATACGTGACTCATTACCTCATCACCCTCCTTCATCTTGAGAGTGAACAACTTCATCTTCATATGCATCTTACT
GGTTAGATCCTTGGACATACAGATCGATTCTAGTTTCAACCAGAGTTCTGCAGCGGTCTTTTCCTGCAGCACCTCTTGCAAAA
TATCATTATGTAAATGAAGCTGAATTAGGGACAAAGCCTTACTATCCCTGCGAACTTCCTCAGGAGTCCAATTCGCCTTCTGC
TTCTTCCCGAAACTATCTAGCGCCTCATCGTAATCTTCTTTCTGCGCCAGAATCGCCCGCATCTTGACTTGCCATAGCGAGAA
CCTCGTCTTGTACGTCAGCGGCTCTAGATCAAACTTCATCGACGTCGTCATGGAACCCTAATCTGCAAACCACAGCTCTGGT
ACCACTTGTTGTAAATGACGGCAAATAAACGATGAAGAATAAGAGATCAATCACAGCGAGACACAAGATTTAACGTGGAAA
ACCCCTCCAATGTGAAGGGGAAAAACCACGGGCGCCAGCCAGCAAACTCTCCTCACTATTTTCAAGAGTCGGGTTACAACG
CCATGCGGCGGCTTACAAGAGAATGAATGAATAATATTCTGTTGTCTCGATGGAACTCTAACATAAGGTATATGAACCATACG
TAGGGGAGGCTAAGACCATACATAACCATTCATAAGTATGTCCACCAAAAATAGCTCCCGATGACTAGGCCTTCACGTCTTCA
GAGCCATAACTCTTTTGGCTTCGTACTCGGTCAGATCTCCAAGTGCCATGCACACCACGCATGTATTTGGAACATATTCAACA
TGTAGCAGCAACGGGCGAACTATTGCGGCGGCCGGCAAGCGTTCGTGGCGGCACGAACGAGCGCAGTGGAGGGGTGCGGG
TGAGCGGGCCGACGCGGGCGAGCGTGAAGAAACCAGCCGTTTCTTCCCAACGGCGTTTTCATCGTTTCTGGCTGCATCGGT
TCTAGGGAAGACGTGGTTTCTCCTCCAGGAAACGGTTTCTTTATATTTTGTTCTCTTTCCTCATTAACTATGTTGTCATATCAGT
TATTTTACTTATGTAACATCTCATTTAATAAAGATAAAAACCTCCATTAATACCCATTGGGACTGCCCAAGCCAAGAGTGGAGT
GATGGGGCTGAGGAGCTGCAGCCAGTGCAGTGTACAGTAGTTGGCTAGTTGCTCTCTTAAATGCACCTCCTTTCTCTCTCCTT
TCCGTCCCGTCCC
>SsLRR-RLK126
GTGAGTTACCACCGGCGCGTCCGGTGGTCCCGCAGAAAACGTGAGTGGAGTTGCAACGGCTCTATTTGGATGGGATGCTTAT
AAATACTTGTTGGCCGGCCCTTGGGGAGGTCTCTTGGCATTTTCTAACATACTTGTGCATCCTTTGAGCTAGAACAACTCCTC
CCACTCATCTCCCTTGCTTGATTGCAAATCAAAAGTGAGATTGAGTGATCCCAAAGTGCATTGCATAGCTTGAGGGCATCTAG
TGGCACTTGTGGGTGGTTTAGCCGTGGATTTCTTGTTACTCTTGGAGAGTACCGCTCCTAGCCGGCTTGGAGATTGTTGGAG
GCGTTGGCAAGAGAAGGTGATTGTTCTTGGCCACCTCCGGTTGATTGTAAGAGGTTCTTGTGCTTTCCTCGGCGGAGTGCCA
AAAGCAACTCTATTACATTGCGCGTGTCATTGAGTTACCTCACTTGAGAGTGGTGTCTTGCGGTGCCTTGCGGGGCGTGCCT
TGAGGGGCAAGTGACTTGAGGGTCACACCGGAGAACCATCTAGGTGTTGGTCGACACAACCGGTGACGTAGCTTGTCGGT
GCACAAGTGAACACCGGGTGAACAAATATTGTGTCTCTTGTCTCCCGGTGATTGGTCTTCATATTGTGTTGTTGTCAATATTG
ATTGATTGTATATCATCTTGGTGATTGGCAACATCTTCACTTCACCGTCGGTATAAGAACCACAATCCTCTCTCTTGCATTTAC
ATTCACTAGTTTATCTTGTGTAGCTCTAGTAGTAGCTTTGCTAGTAGTGTTAGCTTGCTCACTAGTGTAAGTTAGTGACATAGC
CCTTGTGTGAACTAGAGATCTTAATCTACTAGACTTGTGGATAGGAGGCTTGCATCACAACCTTTGGCTAAGCAAAACTCGCT
TTACGCCGTTTGTTTTTCTAACAAGTTTGCTTAGTGGTTCTTGTAGAATTTTTAATAGGCTATTCACCCCCCCTCTAGCCATCTA
GGACCTTACACTAGGCCTTCACGTCTTCAGAGCCATAACTCTTTTGGCTTCGTACTCGGTCAGATCTCCAAGTGCCATGCACA
CCACGCATGTATTTGGAACATATTCAACACGTAGCAGCAACGGGCGAACTATTGCGGCGGCCGGCAAGCGTTCGTGGCGGC
ACGAACGAGCGCAGTGGAGGGGCGCGGGTGAGCGGGCCGACGCGGGCGAGCGTGAAGAAACCAGCCGTTTCTTCCCAAC
GGCGTTTTCATCGTTTCTGGCTGCATCGGTTCTAGGGAAGACGTGGTTTCTCCTCCAGAAAACGGTTTCTTTATATTTTGTTCT
CTTTCCTCATTAACTATGTTGTCATATCAGTTATTTTACTTATTTAACATCTCATTTAATAAAGATAAAAACCTCCATTAATACCC
ATTGGGACTGCCCAAGCCAAGAGTGGAGTGATGGGGCTGAGGAGCTGCAGCCAGTGCAGTGTACAGTAGTTGGCTAGTTGC
TCTCTTAAATGCACCTCCTTTCTCTCTCCTTTCCGTCCCGTCCCGTCCCTCTCCTCCTGTCCTGGGCTGGTGGGAGCCACCCC
TCTCCCTTCTGCCGTCTCCACAGGCTCCCAACAAAGCCCCTGCTCCCAGCGGCACTCAGCAGCACTGCAGCAGCGCGCAAA
TCCTTGCCCTGCCGACAGGGGCGCGCCACGGCCGAGCCCTGAGCCCGCACGCGCATTTGCTGCGGCTCTCGGTGCGCTCCG
CTCCGCTGTACCGGCGGCTATCGCCGGCGGAGGTAAACCCCGCCTCGCCCTCGCTGCTACTGCTTCTGCTCCGGCTCTTCGT
TGAAACCCTGCTGCTCCGAGCTCTGCTTCCTTTGCGATTTCGGTTGCTCTTCCTGCGCGCGCTCATCCTCCTAGGCCGCTTCG
CGGGATGTGCATACCTTAGCTAAGCGTTTCGTCCCTCCCTGAAAAAAAAAAATCTCGTCTTTTACTTTAGGCAATTTTTCTTGA
AATGGACGGGGAGCATGTTG
>SsLRR-RLK53
ATAGTTGCTAGGTTCAGTGAAAAGTTTTCCATGGTGCATTTTTTCCCCGTATTTTTTTGGGTGCATATGAGTTGCCAATAATCA
GTGTCAGGCATGCAAGGTTCCACAAGTGTTAGTACCGTTTCAGTTTACCTGTCACCAGCAAAACATCGTAGCAGTGACAAG
AAAATTGAAACCGAGGTTAGTAATTCGCATGACTGATACCTGTTTTGATCAGTAATAATCATTTGTTAGGGAGTGGAAAGCTG
CGAAGTCGTGAACCATGAGCATGAGCCCCTCGCAAATCATGTCTTTAGCATTGGGTTCTGGAGGACTGACTGCCCAGTAGTT
GATTGTTCTCTGGAATTAAAAAAATGTGATGCTTTTAGGTTGAGATTAAACAACTGGACACTAACCAAGATCTTGTAGTCAGT
AAACAATCCTGCAAAGTTGAAAACCAGTACGTCCAGTAGTTGCATGTACAGTTTCACCCCGGCTAATCTCCTGCAAAAATTT
ACCTTTGTCACTGCCCTACCATCCCTAGTGCACATGTTTTACTTAGGCTGGAGGTTCCGGCTCCTTTTGTCCAAAAGAGCAAT
TCAGAATCGCCTTTGTTTGCCCCACTTTTCTTTCACCTTTTTTAGTGGTATGTATTCTCTTAACAAGTAGCTTTTCTGTAGTGGC
CACTGCCTTACTCGTGCTGCACTAGTGTATGGGAGGCTCCTCTATGGGCATGGGCATATGCTGCCAGTTTTTTTTTCTTTATCC
AACACGCTGATGGAGTTGATTATTACTTGGCCGTGGCCGGTAATGATTAGTCCAAAACTGCAATCATTTGATCCTTGGCCTGC
GAGCTTAATCTTGTTGTTTTCTGCGGCTAACTTAGCAGCATGCTCTGGTCTAGAGTTTAGACTATGTTTTTGCAGGGCTTACTT
TATTGTTTCATTCCAGAAATTCTTGAACCATGTGATTTACTGGTTTTTAGGCGATCACTTGTTTTTTTTTGTTTAAACATGCTTG
TGTTAATCATAATTTGGTGTTTCATTCAAGAAAGCTATTCATGATGTGATATGCTCTCTTTGAGGCAAGAAGGGTTGTGCTTTA
GCCCCTGTCTCGCATTGTCTCCTCTTTTTTTGCTTTCATGGATGCCTTTCGACTTTAGATGCTGGCCCTACCATTTCAACTATTT
TGGGCCTTCAAATTTCATTGCTTTCTTACCGTAACTTCTAGCCATTCTTTGGATGTAGAACCCTTGTCCCTTGTGTTGACTAGC
TATGGTTTTTTTATTCCCATGATTTAACATTGCTTCGAGGTAAGAAAGAAGAATCTCTTTAGTAGTTTAGTGAATGGGAAGTGT
CGGTAAAGTGATTCTCTCCTAGACTGTCAGGGCCCTCTAAAAGCATGATCGAGCAGACTTCACAGTTTATAGTTCACACTGA
GTAATTTCGTGCTATCACCACTTTCCCACCATTATTTTATTCCTTTATTGAAAAGCATTTTGATCTAGTTTGGAACAGATAGCTT
CTTATAATCTTAGATGACATTGTCCTTTAGAAAGCACCTTGGTATTTTGATGGTCTGAGCCTCTTGCGGGGTAAGTGCTTCCTC

GCTGTGGCTTCCATAGCTCAGTCACAGGCACATGCTTCACTGCTTGTGCATACTCAAATCGCACTGTGCTTTCTTCTGGGCTC
AGCACACACATATTTCTTCAGAATCATGATGAGTTAAATACTGATACTAAGTTGTACTTTCAGTTTCGAAATTTAGCACTAAC
CTTTTCGTTTTCATTGCAGACTGCTTGATTATTTCTCAGTCTTAAACTGATGATGCTGGTGCTCGTTTAATCAACTTGATTCAG
AGATATATCAACTATGGGTTATCTGAATCATGGAAGGTCTCTCCCTCTACTTCTCTGTCTACATCTTTTGTTGCCACTACTTCCG
TTTGCTACGGCAGATATAGCCTCCGAGAAGCAAGCACTCCTTGCTTTTGCCTCTGCAGTCTACCGTGGCAACAAGCT
>SsLRR-RLK54
AATCTAATGATACTTAATTGGTGTCATAAATATTATTATCTTATCATATAAATTTGGTCAAACTTGAGATCCTTTGACTCTCCAAG
ATTCTTGGCCTTATAATTTGGGATGGAGGTAGTAATAATCATTTGTTAGGGAGTGGAAAGCTGCGAAGTCGTGAACCATGAGC
ATGAGCCCCTCGCAAATCATGTCTTTAGCATTGGGTTCTGGAGGACTGACTGCCCAGTACTTGATTGTTCTCTGGAATTAAAA
AAAATGTGATGCTTTTAGGTTGAGATTAAACAACTGGACACTAACCAAGATCTTGTAGTCAGTAAACAATCCTGCAAAGTTG
AAAACCAGTACGTCCAGTTGTTGCATGTACAGTTTCACCCCGGTTAATCTCCTGCAAAAATTTACCTTTGTCACTGCCCTACC
ATCCCTAGTGCACATGTTTTACTTAGGCTGGAAGGTTCCGGCTCCTTTTGTCCAAAAGAGCAATTCAGAATCGCCTTTGTTTG
CCCCACTTTTCTTTCACCTTTTTTAGTGGTATTCTCTTAACAAGTAGTTTTTCTGTAGTGGCCACTGCCTTACTCGTGCTGCAC
TAGTGTATGGGAGGCTCCTCTATGGGCATGGGCATATGCTGCCAGTTTTTTTCTTTATCCAACACGCTGATGGAGTTGATTATT
ACTTGGCCGTGGCCGGTAATGATTAGTCCAAAACTGCAATCATTTGATCCTTGGCCTGCGAGCTTAATCTTGTTGTTTTCTGC
GGCTAACTTAGCAGCATGCTCTGGTCTAGAGTTTAGACTATGTTTTTGCAGGGCTTACTTTATTGTTTCACTCCAGAAATTCTT
GAACCATGTGATTTACTGGTTTTTAGGCGATCACTTGTTTTTTTTTTTGTTTAAACATGCTTGTGTTAATCATAATTTGGTGTTT
CATTCAAGAAAGCTATTCATGATGTGATATGCTCTCTTTGAGGCAAGAAGGGTTGTGCTTTAGCCCCTGTCTCGCATTGTCTC
CTCTTTTTATGCTTTCATGGATGCCTTTCGACTTTAGATGCTGGCCCTACCATTTCAACTATTTTGGGCCTTCAAATTTCATTGC
TTTCTTACCGCAACTTCTACCCATTCTTTGGATGTAGAACCCTTGTCCCTTGTGTTGACTAGCTATGGTTTTTTTATTCCCATGA
TTTAACATTGCTTCGAGGTAAGAAAGAAGAATCTCTTTAGTAGTTTAGTGAATGGGAAGTGTCGGTAAAGTGATTCTCTCCTA
GACTGTCAGGGCCCTCTAAAAGCATGATCGAGCAGACTTCACAGTTTATAGTTCACACTGAGTAATTTCGTGCTATCACCACT
TTCCCACCATTATTTAATTCCTTTATTGAAAAGCATTTTGATCTAGTTTGGAACAGATAGCTTCTTATAATTCTTAGATGACATT
GTCCTTTAGAAAGCACCTTGGTATTTTGATGGTCTGAGCCTCTTGTGGGGTAAGTGCTTCCTCGCTGTGGCTTCCATAGCTCA
GTCACAGGCACATGCTTCACTGCTTGTGCATACTCAAATCGCACTGTGCTTTCTGCTGGGCTCAGCACACACATATTTCTTC
AGAATCATGATGAGTTAAATACTGATACTAAGTTGTACTTTCAGTTTCGAAATTTAGCACTAACCTTTTCGTTTTCATTGCAGA
CAGCTTGATTATTTCTCGGTCTTAAACTGATGATGCTGGTGCTCCTTTAATCAACTTGATTCAGAGATATATCAACTATGGGTTA
TCTGAATCATGGAAGGTCTCTCCCTCTACTTCTCTGTCTACATCTTTTGTTGCCACCACTTCCGTTTGCTACGGCAGATATAGC
CCCAGAGAAGCAAGCACTCCTTGCTTTTGCCTCTGCAGTCTACCGTGGCAACAAGCTGAATTGGAATCAAAATATCCCACTG
TGCTCTTGGCATGGTGTGACATGCTCACCGGATCGATCGCGTATATCGGCCCTTAGGGTGCCAGGTGCTGGTTTG
>SsLRR-RLK21
CATGCAAGGTTCCACAAGTGTTAGTACCGTTTCAGTTTACCTGTCACCAGCAAAACATCGTACTCTCTCTAGCAGTGACAAG
AAAATTGAAACCGAGGTTAGTAATTCGCATGACTGATACCTGTTTTGATCAGTAATAATCATTTGTTAGGGAGTGGAAAGCTG
CGAAGTCGTGAACCATGAGCATGAGCCCCTCGCAAATCATGTCTTTAGCATTGGGTTCTGGAGGACTGACTGCCCAGTAGTT
GATTGTTCTCTGGAATTAAAAAAATGTGATGCTTTTAGGTTGAGATTAAACAACTGGACACTAACCAAGATCTTGTAGTCAGT
AAACAATCCTGCAAAGTTGAAAACCAGTACGTCCAGTAGTTGCATGTACAGTTTCACCCCGGCTAATCTCCTGCAAAAATTT
ACCTTTGTCACTGCCCTACCATCCCTAGTGCACATGTTTTACTTAGGCTGGAGGTTCCGGCTCCTTTTGTCCAAAAGAGCAAT
TCAGAATCGCCTTTGTTTGCCCCACTTTTCTTTCACCTTTTTTAGTGGTATGTATTCTCTTAACAAGTAGCTTTTCTGTAGTGGC
CACTGCCTTACTCGTGCTGCACTAGTGTATGGGAGGCTCCTCTATGGGCATGGGCATATGCTGCCAGTTTTTTTTCTTTTATC
CAACACGCTGATGGAGTTGATTATTACTTGGCCGTGGCCGGTAATGATTAGTCCAAAACTGCAATCATTTGATCCTTGTCCTG
CGAGCTTAATCTTGTTGTTTTCTGCGGCTAACTTTGCAGCATGCTCTGGTCTAGAGTTTAGACTATGTTTTTGCAGGGCTTACT
TTATTGTTTCATTCCAGAAATTCTTGAACCATGTGATTTACTGGTTTTTAGGCGATCACTTGTTTTTTTTTGTTTAAACATGCTT
GTGTTAATCATAATTTGGTGTTTCATTCAAGAAAGCTATTCATGATGTGATATGCTCTCTTTGAGGCAAGAAGGGTTGTGCTTT
AGCCCCTGTCTCGCATTGTCTCCTCTTTTTTTGCTGTCATGGATGCCTTTCGACTTTAGATGCTGGCCCTACCATTTCAACTATT
TTGGGCCTTCAAATTTCATTGCTTTCTTACCGCAACTTCTAGCCATTCTTTGGATGTAGAACCCTTGTCCCTTGTGTTGACTAG
CTATGGTTTTTTTATTCCCATGATTTAACATTGCTTCGAGGTAAGAAAGAAGAATCTCTTTAGTAGTTTAGTGAATGGGAAGTG
TCGGTAAAGTGATTCTCTCCTAGACTGTCAGGGCCCTCTAAAAGCATGATCGAGCAGACTTCACAGTTTATAGTTCACACTG
AGTAATTTCGTGCTATCACCACTTTCCCACCATTATTTTATTCCTTTATTGAAAAGCATTTTGATCTAGTTTGGAACAGATAGCT
TCTTATAATCTTAGATGACATTGTCCTTTAGAAAGCACCTTGGTATTTTGATGGTCTGAGCCTCTTGCGGGGTAAGTGCTTCCT
CGCTGTGGCTTCCATAGCTCAGTCACAGGCACATGCTTCACTGCTTGTGCATACTCAAATCGCACTGTGCTTTCTTCTGGGCT
CAGCACACACATATTTCTTCAGAATCATGATGAGTTAAATACTGATACTAAGTTGTACTTTCAGTTTCGAAATTTAGCACTAA
CCTTTTCGTTTTCATTGCAGACTGCTTGATTATTTCTCAGTCTTAAACTGATGATGCTGGTGCTCGTTTAATCAACTTGATTCA
GAGATATATCAACTATGGGTTATCTGAATCATGGAAGGTCTCTCCCTCTACTTCTCTGTCTACATCTTTTGTTGCCACTACTTCC
GTTTGCTACGGCAGATATAGCCTCCGAGAAGCAAGCACTCCTTGCTTTTGCCTCTGCAGTCTACCGTGGCAACAAGCTGAAT
TGGAGTCAAAATATCCCACTGTGCTCTTGGCATGGTGTGACATGCTCACCGGATCGATCGCGTATATCGGCCCTTAGG
>SsLRR-RLK49
AGATGAGCTCCGCAACGGCCAGCCCGTCCATGGCGGCCGTCCCCGCACGTGCGCGCGGCTCGCCCGCGGCCGGGAGAGAG
CAGGGGATGGCGTCGGAGAGCAGGGGACAAGGAGGAGGCCACGCCCGTCGGAGGGAGCAGATGAGCTCCGCGGCAGCCG
GCCCGTCCATGGCGGTCGTCCCCACGCCCGCACCCGCCGGAGGGAGGGAGTGAGGTAAAAAAAGGAAAAAAAAATCTGAG
GTGTGGGTCTCACCTTGGTATAGAGGATGAGATAGATTATGGATGGTACGGGAGAATTGGGTATAGAGGAGAAAGTCTTGAT
GACTAGGATGTATAGAGGATGAATATAGAGGACCATGAGTGCAGACAGCCTAAGTAGCTGGGCACCTGCGGAAGACTGCGT
CATTTTGTCGTACACTGCAAGATATGCAAAGCAGCAAAGGGCTTGTTTAGTTGGTGAAAATGTTTGGGTTTGGTTACTGTAGC
ATTTTGTTTTATTTGACAATTTGTGTCTAATCATGGACTAATTAGACTCAAAAGATTTGTCTCGTGATTTACAACTAAACTGTG
TAATTAGTTTTTTCTTTCGTCTACGTATAGTACTCCATGCATGTACCGTAAGATTTGATTTGATAGGTACTGTAGCATTTTTGGG
GTTACTTTTTAGGAACTAAACAGGCTCAAACAAATTGAAGTAGAACTGTAGTCAGCACAGTTTGTCACTTTGGGTGGGCGA
ATCATTTTGCCAGAATGGGTCCAGGAACGAGACGTGTCAACATCCCATGGTCCATTCCTACCAATTTCATCAACAAAGAACA
GCGGCAAACGTGTGACGAATACGAGGGTGGTGGCTGATGATCACAGCCATGACCCATGATCCATCATTAAGCCCTAGCCGTG
TTACGAAATCGTTCAGAATCCACTGGTAAGTGAGCGGCTCTCGCCTACCGGGCACCGGCCGCACCATCGACCTCTTCTTGGT
TGCTTATCGGGAACGAAAGCTTGGTACGTGGATGCGACACGCTAGTGATCCAATGCGGTTTTGGCCCAGGCAACGGCGCAG
GGCCATGTTCAACACGGGAACGCAATTTCAGCGAAGGAAGCATGGAAATTCCTTTTTTTTTCGTCGAATCAATGAAGCCTGG
AACCAGTAATGTCACCACTAAATTTCGAGGACTGGATAAACGGAAAGCAATATTTATTTCTGGTCATAAACGCGTAGTTCCTC
CGAATGGAGTTTATTTGTGGCCATTATGTAAATCTAGCTGTTCGAATCAACCCGCAGTTTTTTTTTTAAAAAAAATGAAGAGC
GCTACCATAGCAGTAGGGAAACCAGCCAGACCTGCCAGAACAATCCGTCTGCTCAGCCCGCCCGGTGGTGGGCCCAGCTGT
CAGGCCAGCACGCGAAACAACACGGCATTTGTTGGTTTGTTTGTTCCAGAACAGGTCTCGGCCGCAGCCGGAGTCCAACGC
ACCAGCACGGCACCGTTCCCCAGGCAAGTCTCTGAAGTCTGAACCCAACCACCCACCCGGCACGCCGGCAGGTGCGCCAG
CGCGCACGCATCCCCGCAGGCCGCAGCGCTACGCACACTCCCGTCCCCGTCCATCGGATCCCCTCCCCTCTACCCTCTGAGG
AGAGGGAAACCCCATTCCGATTCCCATCCAGGTCCTCGTAGTCGTGCCACCTGTTTGTTGCCTCCTATTCCCGTCGCTCTCGC
CCGGCCCCGTCGGCCCCACCAACCCCCAGCGGCGCAAACCTCCCAAGTCCCAACCCCCGGATAAATTCCGCCCGCGAATTA
CCCAGCTCCCCGAGGCCCCATTCCGCGAAACGGCACGGGAGTGTTTAGGGTTTTTCTCCCCCGCACGCCACAGACGCCCAC
ACAACAACACAGCAGACGCGGCCGCGTCCCCCCACAATCCCCCAGAAACGGACGCCACCCCCTCCCCGCCTTGCCTCGCTC

CGCTGCGGCCCGGCTCGTCGCCACCCCAGCCGACGCCGCG
>SsLRR-RLK15
ACTCGCCAAAGGGCTGGTTCGGCCGGTGGAAAAGCCGGCTGATTTCGGTTGGTTGAATAGTGTTCTGTGAGAGGGAATAAG
CTGAAATAAGCTGAAACCAGCCGAAACCTGACCAGCAGAACAGGGCCAAATTACGCGTCTTTAAGTAGCTGGACACCTGCA
GAAGACTGCGTCCTTTTGCCGTACACTGCAAGATATGCAAAGCAGCAAAGAAATTGAAGTAGAACTGTAGTCAGCACAGTA
TGTCACTTTGGGCTGCGTTTAGATGTAATTTTTTTTGCAAAAACGGCACTGTAGCTCTTTTCGTTATTATTTGACAAATATTGT
CCAATCATGATCTAATTAGGTTTAAAAGATTCAGCTCGTCCATTTCGTCTAAACTGTATAATTAGTTTTATTTTTTATCTATATTT
AATGCTTCATGCATGTGTCAAAGATTCGATGTGACGGAGAATCTGAAAAATTTTGTAAAATTTTCTGGGAACTAAACAGTGC
CTTGGTGGGCGAACCATTTTGCCGGAATGGGTCCAGGAACGAGACGTGTCAACATCCCATGGTCCATTCCTACCAATTTCAT
CAGCAAAGAACAGCGGCAAACGTGTGACGAATACGAGGGTGGTGGCTGATGATCACAGCCATGACCCATGATCCATCATTA
AGCCCCAGCCGTGTTACGAAATCGTTCAGAATCCACTGGTAAGTGAGCGGCTCTCGCCTACCGGGCACCGGCCGCACCATC
GACCTCTTCTTGGTTGCTTATCGGGAACGAAAGCTTGGTACGTGGATGCGACACGCTAGTGATCCAATGCGGTTTTGGCCCA
GGCAAGGGCGCAGGGCCATGTTCAACACGGGAAGGCAATTTCAGCGAAGGAAGCATGGAAATTCCTTTTTTTTTTCGTCGA
ATCAATGAAGCCTGGAACCAGTAATGTCACCACTAAATTTCGAGGACTGGGGCCACGTTTAGTTGGCTCCAAAATTTAAAAA
GTTTTTTAATTACCCGTCACATCGCATATGCCGGCATATGTATGAAGTATTAAATATAGGTAAAAAAATTAATTGCACAGTTTGT
CTGTAAATGTCGAGACGAATCTTTTGAGCCTAGTTAATATATGATTGGATAAAGTTTGTCAAATATAAACGAAAATACTACAGT
GTTTTTTAAAAAAAATTTGCCATCTAAACATGGCCTGGATAAACGGAAAGCAATATTTATTTCTGGTTATAAACGCGTAGTTCC
TCATAATGGAGTTTATTTATGGCCATTATGTAAATCTAGCTGTTCGAATCAACCCGCAGTTTTTTTAAAAAAAATGAAGAGCG
CTACCATAGCAGTAGGGAAACCAGCCAGACCTGCCAGAACAATCCGTCTGCTCAGCCCGCCCGGTGGTGGGCCCAGCTGTC
AGGCCAGCACGCGAAACAACACGGCATTTGTTGGTTTGTTTGTTCCAGAACAGGTCTCGGCCGCAGCCGGAGTCCAACGCA
CCAGCACGGCACCGTTCCCCAGGCAAGTCTCTGAAGTCTGAACCCAACCACCCACCCGGCACGCCGGCAGGTGCGCCAGC
GCGCACGCACCGTCGCGCTCGCATCCCCGCTGCACTCCCGTCCCCGTCCAGGCGTCCATCGGATCCCCTCCCCTCTACCTCT
GAGGAGAGGGAAACCCCATTCCGATTCCCATCCAGGTCCTCGTGCCACCTGTTTGTTGCTTCCTATTCCCGTCGCTCTCGCCC
GGCCCCACCTACCCCCAGCGGCGCAAACCTCCCAACCCCCGGATAAATTCCGCCCGCGAATTACCCAACTCCCCGAGGCCC
CATTCCGCGAAACGGCACGGGAGTGTTTAGGGTTTTTCTCCCCCGCACGCCACAGACGCCCCCTCCGCCCACACAACAACA
CAGCAGACGCGGCCGCGTCCCCCCACAACCCCCCAGAAACGGACGCCACCCCCTCTCCGCCTTGCCTCGCTCCGCTGCGGC
CCGGCTCCTCGCCACCCCAGCCGACGCCG
>SsLRR-RLK63
GACAAATATCATTTGAAGATATTAGTGCCTGGAGTTTTAGTTTCTTAATTAATAATATGGAGTCTGAGGATTTCGTGGACTGCC
ACAAATGCTCTATAAACATTAAGAAATATTCTAAAGCACTTTTTTACATAAAGCAAACATGCCAACATGCGGTACCTCATTGG
GTGATTGTTTGATGGTGGTGACTACAGCCTACTGCATCCATATAGACATTTCATCGAACACAAGAGAAGGCACCTCGCCAAC
ATTCTACCTGCAGCGCACATATTTGGTAGCTGTCAAATGCATTCAGGGCAGATAGGCTCCGGATGGGGCTGGAGGACAGGAG
GAGGTGCTTCGAACCTTCGCATACGTCGGGAGGAAGAGTAGGCCCGTCCCCGGCGTCAACATCCGCTGCCTCACCTGCCGG
AAAGTAGAATGGGAAGATTTTTTCGGTGTGTGCCAAAAAGAGCTAAACGCACGAGCGGCTCAATAGAGGAAGATGGGAGG
AGAATGCAGTAGCGCCTACCTGCGGTGCCAACGTCGCAGACGGGAGGGTGGGTGGCGGCAGTAGTCGCGGGGCGTCGTGA
GCGTCGTGAGACTGAGCCGGTGGCGAGCGCGTCCATCACACGACAGTAGCCCACAGGGGCTGCGCGTCCAGGTCGCAGGG
TGCGAGAGGACGCCACCAGGCCGGGAACGAACGGATCCGGCCATGGAGGCCCTGGATCCGGTCGTGGAGGCCACGGATCG
ACCCCAGGAAATGTCGGCGAGTGAAGGAGAGGAGGAGCAAGGGAGAGAGGCTGCCTTCACCTGGGGCTACTCGTGCCGG
AGTCCTCGCCAGCCGGCTGTACCTAAGCCGCCGCATGGGGCTTGCTCGCGCGCGGGGCGCCGGCGGCCTCTGCACCTGGAC
CCGCCCGCCGCGCCAGGCCGCTCTAACCGGGGCTCGGGCCTCCCTCGCCCGGGGCTGCAGCGCCCACGCGCGGACGCGTG
CGCGGCACCGGCCCTTGCCGCCGCCGAGAGAGGAGGGAGAGGGCAGGAGAGGCGGCTGTGGGCGCGAGAGAGAGTTAGG
GTTGAGGGTTGGAGCCGGGCCGGTATATATTTGGCAAGGACCACATGTACGGCGACGCCACTAGCCATCCGATGGAGCATCT
GACGGCGCACAGTATTTCCGATGACGTGTCCACGCTGAGGCCGGGTCGGCTCCTGGTGTTCTCTTGTCTTTAGATGCGGTTT
TGGCCCAGGCAACGGCGCAGGGCCATGTTCAACACGGGAACGCAATTTCAGCGAAGGAAGCATGGAAATTCCTTTTTTTTT
CGTCGAATCAATGAAGCCTGGAACCAGTAATGTCACCACTAAATTTCGAGGACTGGATAAACGGAAAGCAATATTTATTTCT
GGTCATAAACGCGTAGTTCCTCCGAATGGAGTTTATTTGTGGCCATTATGTAAATCTAGCTGTTCGAATCAACCCGCAGTTTTT
TTTTAAAAAAAAATGAAGAGCGCTACCATAGCAGTAGGGAAACCAGCCAGACCTGCCAGAACAATCCGTCTGCTCAGCCCG
CCCGGTGGTGGGCCCAGCTGTCAGGCCAGCACGCGAAACAACACGGCATTTGTTGGTTTGTTTGTTCCAGAACAGGTCTCG
GCCGCAGCCGGAGTCCAACGCACCAGCACGGCACCGTTCCCCAGGCAAGTCTCTGAAGTCTGAACCCAACCACCCACCCG
GCACGCCGGCAGGTGCGCCAGCGCGCACGCACGCACCGTCGCGCTCGCATCCCCGCAGGCCGCAGCGCTAGCGCTACGCA
CACTCCCGTCCCCGTCCATCGGATCCCCTCCCCTCTACCCTCTGAGGAGAGGGAAACCCCATTCCGATTCCCATCCAGGTCCT
CGTGCCACCTGTTTGTTGCCTCCTATTCCCGTCGCTCTCGCCCGGCCCCACCTACCCCCAGCGGCGCAAACCTCCCAAGTCC
CAACCCCCGGATAAATTACCCAACTCCCCGAGGCCCCATTCCGCGAAACGG
>SsLRR-RLK341
TTGACACACATGTATGCACCGATCCTGGATGCATACGGTATGCATATAATTTTTTGCCGTTAGCGCCTGGGGAGGGAGGGCAA
CGGTCCTGTAGTGCGCTGGATCCATTTCCAGGGTGACCTATTATGGACCGAAGCATCCATTCATACACGAAATAACGTAATCT
CTTGACGCTGTGACTTGTTGACGGGGCGAAAACGCCGAACCAAACAGCACGTTAGTACAACTCACTATATTTAGAACATAG
GGAATGGGTGGTGTGACTTCAACGGTAAATGTTTCTTTTTCAGTGGTTGAATTCCATATAGGTCGGGTGTGCTTTTGCATGGT
TAGGGCTCCTTTAGATCATAGGAATTCCAAAGGAAAAGTGGAGGATTCTTATTCCTATGGATTTAAATACTGTAGCACCGTTT
GGAACATAGGATTGAGGCATAGGAGAAGTGAAGGAAATTTTCCTATGCTGCAGGTTTCATAGGGAAAACAAAGGATTCCAA
ACATCTGCTTTGAGCTCTTTTTTTCATTCCTTCGCGTAGGACCGAGGCAAAGTGTGATTTGACAAGGATAGATTGATGATATG
GTACGGCCATCTGTGCATAATGTTGATGATTACCATTCATGATCATCGTTTAATACCAAATCTCTTCTAATGTTTCCTGTGTTTTT
CCTATGGCTCATCCAAACGACTTTTCCTATAGAATTTCTACGTTTTTCAATCCTCTGTTTTACGTATGCATTCCTATCTCGTTCCT
GTGTTTTTCCCATTCCTACATTTTTTCAATCCTACATTCCAAAGGAGCCCTTATATGGGTGAGAGCGCACTATTGTCAATACGA
GGACCTAGCGTGTTTTCTCTTTGATTTTTTAAGAAAAAACATGGAGAGCAGAGCATGTAGGCATGTACTACCTCCGTCCACG
AAAGAATGTAATTATGGAAAGTGTGCTGGTCAAATTAGCTTAAGTTTGACTAAGTCTATAATAAATAGTATTAGCATTTATGTC
TCCAAATAAACTTATTATAAAAAAACATTCTATAACTCATCTAATGATACTTATTGTATCTCATGAGTGTTATTAATTTTTCATAT
AATTTTAGTCAAAGTTTAAACCGTTTGATTCCTCAAAATGTGAGAATTGTATTCTTTTATGGACTGAGGGAGAGCAGCTGGAA
ATGCTGGAATACAAACGAAGGAATGCAGGACGTCTACGGTCCTCCTGGAGGAGAACGCGAGGCGTTTCCGGGGCGACGCG
TTCGTCGCGGCGCGCCGGCCCGCTCCCATCCAAACGCCAAGCCTCAGTTCTTTCACCTCATGTCGTCAGTTCACCATCTGTC
CCTCGACCAATCCCTCGCTGAGTCTGATTGACGCCGACCCCGATGCCGATTTAACTAATCTCCAATTAACCTTTAACCACCTT
GTTGCTTTGGCTAATTAGCTGGAGTGTCACCACTACATCCAGAGACCAGCAGAGTTGCCAATTCTGGCAGCACCACAGCATA
CTAATATAATCTGCCGTCCATGGCGATCTAGCTTAATTAGCAGCAGCTGTAGCTTTGTCTATGGCTAAAGTGTTAAACTAGTCG
AGTTAATTACGAGTGTTAAGCAGAACTGTGCTGCATATTTGCCTCGGCGCAGCTGGACGGACGAGCTCACCACCAGCTTCAC
TTCACACACGTATTTAGAGATAAAGCCAACGGAGAAGAACGTACGATGCGAACCAAAGGATTACGTATAAAACGATCCTGTC
TTTCCCGCCATGTTTACACACCTCCGACCCAACCGCGTCCGCGCCACCGATTCCTCCGCCGCCGCTGATGAACTCTAGGCTG
CGGCCTCCAAACCTCCATCCCCTCTATTGACACACGCGGCGACGTATTCTCGCCTGCCGTCGCCGTCGCCGTCCCCTTGTTGC
GCGCGCCCAGCCGGCTGCTTTATTAGTTACTACTTCCAACTGCCTTTCTTGATTACCTGGAAGCTGCCGACCTCGCAGCGGCG
CCGGGCGA
>SsLRR-RLK310

TCAAGTATCGTAAGACACGAAGTAAATGGCCAAAATGAACGTGAGGGAACGGTAATAATGGGATAAGCTGCCGGCGCTAAC
TGTTACAGGGGACAAAATACGCTACCTGTTTGGTTGAGTGCTTTCCACCGCGAGTTCGATGCCATACGGGTGAGGTGGCGCG
AGAGGGGTGGGCCTGGGCAGCGCGCATGGCCGCATGCATTGAGAGCTGCGGCGCTCTCTATTTTTATTTTCGCGAGTCCCCT
CCGCAACCACGAAGGAGATTGGAATCGGTTAGCGCCGGAAGAAAATTGTATGCATACCGTATGCACCTAGGAACAGTACATA
CAGACGTGAGAGCCAATCATAGGACGAGAACATGGAGTGGAGCACGTGAGAGCCGAGCCAGTCACAGGATGAGGACGGCT
TCCAACTGCCACCCGATGAGCGCATACCGTAGATGCTGTTGCTTTGTCCACGTGGCAGCAGATTGACACATGTATGCACCGAT
CATGGATGCATAAGGTATGCATATAATTTTTTGCCGTTAGCGCCTGGGGAGGGAGGGAGGGCAACGGTCCTGTAGTGCGCTG
GATCCATTTCCAGGGTGACCTAATACGGACCGAAGCATCCATCCATACACGAAATAACGTAATCTCTTGACGCTGTGACTTGT
TGACGGGGCGAAAACGCCGAACCAAACAGCACGTTAGTACAACTCACTATATTTTAGAACATAGAGAATGGGTGGTGTGAC
TTCAACGGTAAATGTTTCTTTTTCAGTGGTTGAATTCCATATAGGTCGGGTGTGCTTTTGCATGTTTTATATGGGTGAGAGCGC
ACTGTTGTCAATACGAGGACCTAGTGTGTTTTCTCTTTGATTTTTTAAGAAAAAACATGGAGAGCAGAGCATGTAGGCATGTA
CTACCTCCGTCCACGAAAGAATGTAATTATGGAAAGTGTGCTGGTCAAATTAGCTTAAGTTTAACTAAGTCTATAATAAATAG
TATTAGCATTTATGTCTCCAAATAAACTTATTATAAAAAAACATTCTATAACTCATCTAATGATACTTATTGTGTCTCATGAGTGT
TATTATTTTTATATAATTTAGTCAAAGTTTAAACCATTTGATTCCTCAAAATGTGAGAATTGTATTCTTTTGTGGACTGAGGA
GAGTAGCTGGAAATGCTGGAATACAAACGAAGGAATGCAGGACGTCTACGGTCCTCCTGGAGGAGAACGCGAGGCGTTTC
CGGGGCGACGCGTTCGTCGCGGCGCGCCGGCCCGCTCCCATCCAAACGCCAAGCCTCAGTTCTTTCACCTCATGTCGTCGG
TTCACCATCTGTCCCTCGACCAATCCCTCGCTGAGTCCGATTGACGCCGAGCCCGATGCCGATTTAACTAATCTCCAATTAAC
CTTTAACCACCTTGTTGCTTTGGCTAATTAGCTGGAGTGTCACCACTACATCCAGAGACCAGCAGAGTTGCCAATTCTGGCA
GCACCACAGCATACTAATATAATCTGCCGTCCATGGCGATCTAGCTTAATTAGCAGCAGCTGCAGCTTTGTCTATGGCTAAAGT
GTTTAACTAGTCGAGTTAATTACGAGTGTTAAGCAGAACTGTGCTGCATATTTGCCTCGGCGCAGCTGGACGGACGAGCTCA
CCACCAGCTTCACTTCACACACGTATTTAGAGATAAAGCCAACGGAGAAGAACGTACGATGCGAACCAAAGGATTACGTATA
AAAAGATCCTGTCTTTCCCGCCATGTTTACACACCTCCGACCCAACCGCGTCCGCGCCACCGATTCCTCCGCCGCCGCTGAT
GAACTCTAGGCTGCGGCCTCCAAACCTCCATCCCTTCTATTGACACACGCGGCGACGTATTCTCGCCCGCCGTCGCCGTCGC
CGTCCCCTTGTTGCGCGCGCCCAGCCGGCTGCTTTATTAGTTACTACTTCCAACTGCCTTTCTTGATTACCTGGAAGCTGCCG
ACCTCGCAGCGGCGCCGGGCGA
>SsLRR-RLK324
CGGCCGTGCCGCGTGCCGTGTCGGCACGGCACGGTGGCCATATATAGCAAGACACGAACGGTAATAATGGGATAAGCTGCC
GGCGCTAACTGTTACAGGGGACAAAATACGCTACTTGTTTGGTTGACTTGAGTGCTTTCCACCGCGAGTTCGATGCCATACG
GGTGAGGTGGCGCGAGAGGGGTGGGCCTGGGCAGCGCGCATGGCCGCATGCATTGAGAGCTGCGGCGCTCTCTATTTTTAT
TTTCGCGAGTCCCCTCCGCGGCCACGAAGGAGATTGGAATCGGTTAGCGCCGGAAGAAAATTGTATGCATACCGTATGCACC
TAGGAACAGTACATACAGACGTGAGAGCCAATCATAGGACGAGAACATGGAGTGGAGCACGTGAGAGCCGAGCCAATCAC
AGGATGAGGACGGCTTCCAACTGCCACCCGATGAGCGCATACCGTAGATGCTGTTGCTTTGTCCACGTGGCAGCAGATTGAC
ACACATGTATGCACCGATCCTGGATGCATACGGTATGCATATAATTTTTTGCCGTTAGCGCCTGGGGAGGGAGGGCAACGGTC
CTGTAGTGCGCTGGATCCATTTCCAGGGTGACCTATTATGGACCGAAGCATCCATCCATACACGAAATAACGTAATCTCTTGA
CGCTGTGACTTGTTGACGGGCGAAAACGCCAAACCAAACAGCACGTTAGTACAGCTCACTATATTTTAGAACATAGGAATGG
GTGGTGTGACTTCAACGGTAAATGTTTCTTTTTTCGGTGGTTGCATTCCCGGGTGTGCTTTTGCATGTTTATATGGGTGAGAG
CGCACTGTTGTCAATACGAGGACCTAGTGTGTTTTCTCTTTATTTTTTAAGAAAAAACATGGAGAGCAGAGCATGTAGGCAT
GATCTACCTCCGTCCGCAAAAGAATGTAATTATAGAAAGCGTGCTAGTCAAATTAGCTTAAATTTGACTAAGTCTATAATAAAT
AGTATTAGCATTTATGTCTCCAAATAAACTTATTATAAAAAAACATTCTATAACTCATCTAATGATACTTATTGTATCTCATGAGT
GTTATTATTTTTTCATATAATTTTAGTCAAAGTTTAAACCGTTTGATTTCTCAAAATGTGAGAATTGTATTCTTTTGTGGACTGA
GGGAGTAGCTGGAAATGCTGGAATACAAACAAAGGAATGCAGGACGTCTACGGTCCTCCTGGAGGAGAACGCGAGGCGTT
TCCGGGGCGACGCGTTCGTCGCGGCGCGCCGGCCCGCTCCCATCCAAACGCCAAGCCTCAGTTCTTTCACCTCATGTCGTCA
GTTCACCATCTGTCCCTCGACCAATCCCTCTCTGAGTCCGATTGACGCCGAGCCCGATGCCGATTTAACTAATCTCCAATTAA
CCTTTAACCACCTTGTTGCTTTGGCAAATTAGCTGGAGTGTCACCACTACATCCAGAGACCAGCAGAGTTGCCAATTCTGGC
AGCACCACAGCATACTAATATAATCTGCCGTCCATGGCGATCTAGCTTAATTAGCAGCAGCTGTAGCTTTGTCTATGGCTAAAG
TGTTAAACTAGTCGAGTTAATTACGAGTGTTAAGCAGAACTGTGCTGCATATTTGCCTCGGCGCAGCTGGACGGACGAGCTC
ACCACAAGCTTCACTTCACACACGTATTTAGAGATAAAGCCAACGGAGAAGAACGTACGATGCGAACCAAAGGATTACGTA
TAAAAAGATCCTGTCTTTCCCGCCATGTTTACACACCTCCGACCCAACCGCGTCCGCGCCACCGATTCCTCCGCCGCCGCTG
ATGAACTCTAGGCTGCGGCCTCCAAACCTCCATCCCCTCTATTGACACACGCGGCGACGTATTCTCGCCCGCCGTCGCCGTC
GCCGTCCCCTTGTTGCGCGCGCCCAGCCGGCTGCTTTATTAGTTACTACTTCCAACTGCCTTTCTTGATTACCTGGAAGCTGC
CGACCTCGCAGCGGCGCCGGGCG
>SsLRR-RLK88
CATGAATTAGTCTTTGTTTATGTAATTAGTTTTATAATTAATCTATATTTAATACTCCTAATTAGTATCAAACATCCGATGTAACAG
GGACTAAAGCTTAGTCGCTAGCAATCAAACACTAGAACATCCTCACTGTTTATTTTGATAGAATAGGAGTAGATAAAAACAG
CTATTTGATACAGTGCATTGAGAAGCCAGAGCTGAAGTCGTCCTAAGCTTTACTAAATGGGCCATAAACGCACACATCACATA
GTTCACTGTTCTGGTTACTTACAACTCAAAATCTCAAATGCAATTCTAAAGTGCAGACACGGTCACTAGGGACACTCACGTA
GACACAGTTTAATAATCCAGAATCTCGTATTCATTCTCATGAAACAAAATCAGAAAGGTAGCAAGGCGGTAAAAAAACAGG
AAATGTGAAAAATGGCTTAAAACACTATTTCTTGTCCCACTTTACGTTTTTTCACCATGTTTCAATTTTCTGTAAAAAAGTACG
AAAATTAGAAAATGAGACGAGACGGGAAAGCCATTTCTCTATCTACGTAGAACATCCGAGGGCTTAAAACTGGGATGTACAT
AGATGGTCGAGAAATGCCCAAGCGGGCCGTACATTCTGTTGTTTGGCACAACAGAATGTTTTTGATTGGATATTCAGCTTCAC
AGTTTGGGTCGGCCCAATCTGTGTCGACTCGATCGGTGTACGTTCCGGCGCGAGGAGCCTTTGGGCTTGCGACGTGTTCCTC
ATTCGGATCCGAGTCATCAATAACTATCACAGACCGTTGACGACGATTACGGGGTATAGAGAGCAGTAGACGCAGCCAAGGC
AGCTACGGGCTACGGTCTTTGGGTTCGTACGAGTACGACGCATCACTCGGGAGTCTGTCCTGTACGCGTTCTCTCGCTGTCG
CCGACTGGAGAGAAGGAAGCGCCCAAGAAAGAAACGGCCTGGCCAGGCCGCCGCGCTGCCGAGGGAGACCGGTTCAACG
GAGCGACACACGTACGCCTCCCCTCCCTCGCCGTCCATATCACCGCTCTTGATCGACTCGCCGCCGGCCGTTTCACTACCAG
TCTACCACCACGCAATCAGTCGCAGGCCCCTCCGATCCGACGCGTCTCTCTAGGTCGCGCCGCCCGCGGTGCATCCAACCAG
AACCAGTGTGTATGCCGTGTGCGGCCTGCCGCTACAGTTACGCCGCCGCTGCCGCCTGTAGCTGTAGAGCATCCCGTGTCGC
CCCTACCCAAGCACGCCGGCCCTGTCTTGCTGATCCCGGCCCATGTCCATGTGGGCATGTGGCTGACGAAAACGTGATTTGC
CCCTGCCGCTGAGTCTCGCCGGCCCACGTCCGCACACGTCCGGTCCATCGTATATACTAGATAGATAGAGTACGGAGTAGAAT
ACAATAATATACTATATGGAAGCATAGCCGCAGTACCAAGCACGCAATTGGTAGTAAACGAACGAGAAAGACGGGTCAGGG
ATGGAGGGTTTATTAGTCACAGCAGACGCTGTATCCTTTTTGTTTTTCTCCATCTCTTCGTGCAGCAGACGCCGTCTCCGGCT
CCTCAAAGACGTCCAAATGGAAAGCGCCCCGGCCGTGGCCGTGGCCGTGTTCCCGCCACCAGCTAGCTTGCGCTGCGACTG
CGAGCTGCAGCCACCATCTATCTATCCATCTCCTTCCATCGAGCTGTAACACATCAGCGTCTGAAAAGAAAATGTAATCAACG
CGCGACGCCATCGCGTATCGTCTCGTCTCCTTCCTGCAAAGCCCCGGCCGCTAGCTTCCGCCAGCGCCACGTTCCTGTCACC
ACCGTCGCGGCCAATCCGGGCGGAGCTATATCAGGTCCCTGGACCACTGCGACTCCTTCTTCGCTTAACGAATTCTGCCTGT
CGCCGCACTGCTCCTGCGCAGACGAGACGACGTACGTACGCAGCCGTTGTATCGTAGCGCGCTGCCCCTTGCACCGATCGAT
CACTATCCACTCGCCGTGCCGGCAAA
>SsLRR-RLK131
CGTAGACACAGTTTAATAATCCAGAATCTCGTATTCATTCTCATGAAACAAAATCAGAAAGGTAGCAAGGCGGTAAAAAAAC
AGGAAATGTGAAAAATGGCTTAAAACACTATTTCTTGTCCCACTTTACGTTTTTTCACCATGTTTCAATTTTCTGTAAAAAAG

TACGAAAATTAGAAAATGAGACGAGACGGGAAAGCCATTTCTCTATCTACGTAGAACATCCGAGGGCTTAAAACTGGGATGT
ACATAGATGGTCGAGAAATGCCCAAGCGGGCCGTACATTCTGTTGTTTGGCACAACAGAATGTTTTTGATTGGATATTCAGCT
TCACAGTTTGGGTCGGCCCAATCTGTGTCGACTCGATCGGTGTACGTTCCGGCGCGAGGAGCCGCCTTTGGGCTTGCGACGT
GTTCGGTGTTCCTCATTCGGATCCAGACCGTTGACGACGATTACGGGGTATAGAGAGCAGTAGACGCAGCCAAGGCAGCTA
CGGGCTACGGTCTTTGGGTTCGTACAACGCATCACTCGGGAGTCTGTCCTGTACGCGTTCTCTCGCTGTCGCCGACTGAAGA
GAAGGAAGCGCCCAAGAAAGAAACGGCCTGGCCCGGCCGCCGCGCTGCCGAGGGAGACCGGTTCAACGGCAGGCTGCGG
AAACGGAACGAACGAGTGGCCGTCTCTGGCTCAGAGCCAGTCCGTCGGCGTAGCTGTGCACCCACGCAAAGATCGCACAG
CGACACACGTACGCCTCCCCTCCCTCGCCGTCCATATCACCGCTCTTGATCGACTCGCCGCCGGCCGTTTCACTACCAGTCTA
CCACCACGCGATCAGTCGCAGGCCCCTCCGATCCGACGCGTCTCTCTAGGTCGCGCCGCCAGCGGTGCATCCAACCAGAAC
CAGTGTGTATGCCGTGTGCGGCCTGCCGCTACAGTTACGCCGCCGCTGCCGCCTGTAGCTGTAGAGCATCCCGTGTCGCCCC
CACAGCCCCAGCGGCTATCAGTTGCGACGACCGTCGACCGGCGTATCGTCTGAAATGTACTGATTTCCCGCCGCGCCACTAC
CCAAGCACGCCGGCCCTGTCTTGCTGATCCCGGCCCATGTCCATGTGGGCATGTGGCTGACGAAAACGTGATTTGCACCTGC
CGCTGAATCTCGCCGGCCCACGTCCTCACACGTCCGGTCCATCGTACATACTACTAGATAGATAGACTACGGAGTAGAATACA
ATAATATACTATATGGAAGCATAGCCGCAGTATCAAGCACGCAATTGGTAGTAAACGAACGAGAAAGACGGGTCAGGGATGG
AGGGTTTATTAGTCACAGCAGACGCTGTATCCTTTTTGTTTTTCTCCATCTCTTCGTGCAGCAGACGCCGTCTCTCCGGCTCCTC
AAAGACGTCCAAATGGAAAGCGCCCCGGCCGTGGCCGTGTTCCCGCCCACCAGCTAGCTTGCGCTGCGTCTGCGAGCTGCAG
CCACCATCTATCTATCCATCTCCTTCCATCGAGCTGTAACACATCAGCGTCTGAAAGAAAATGTAATCAACGCGCGACGCCAT
CGCGTATCGTCTCGTCTCCTTCCTGCAAAGCCCCGGCCGCTAGCTTCCACCAGCGCCACGTTCCTGTCACCACCGTCGCGGC
CAATCCTCGCGGAGCTATATCAGGTCCCTGGACCACTGCGACTTCTTCTTCGCTTAACGAATTCTGCCCGTCGCCGCACTGCT
CCTGCGCAGACGAGACGACGTACGTACGCAGCCGTTGTATCGTAGCGCGCTGCCCCTTGCACCGATCGATCACTATCCACTC
GCCGGTGCCGGCAAATGCGGTGGGCGCCGGCGACAGCGCCGGCCGGGCGGTCTACGTCCTCCTCGTCGCAGCGCTGTGGT
GCGCCTCGCTCACCCCGCGCGCGGCGCCGGCGGACCTCGCGGCGGACCGGGCGGCGCTGCTCGCGTTCCGGGCCGCGGTG
GGGCCGCGGCTCCCGTGGGACGCGGCGTCGCCGTGC
>SsLRR-RLK160
CTGATTTAGGTCTGGTTTAGTTCTCAAAAAATTTTGGTCCATCATATCAAATGTTTGGACATATGCATTGAATACTAAATGTAG
ACGAAAAAAACTAATTGCACAGTTTGGTTGGAAATCGTGAGACGAATCTTTTAAGCCTAATTAGGCCAGTATTAGTCTTAAG
TGCTACAGTAACTTACATGTGCTAATGACGGATTAATTAGGCTTAATAAATTTGTCTCGTAGTTTCTAGGCGAGCTATATAATTA
GTTTTTTATTAGTATCCGAAAATCTCTTTCGATATCCCATAAACATTCAATATGACACCTAAAATTTTTCTTTTCACAAACTAAA
CACAGCCTTAGTAGAAGACCTGACAGCAAAACACTTTCCAAATGAAATGTGGAGCCCGAGACATACGGAGAAAAAGGCCG
CATGAGAACAGAAACTCGATTTGTTTTCATACTGGAGGCAGAGACAATGCAGCCGTGCTTGCAGGAGCAGGATAGCTACGG
CCGTGTCGTGTCGTGTCGTTTTGTTTTTCCGGCCAGACTGAGACTCGCCGCGGTGGGTACTTTTAGAATGATACTCTAAAGCT
TTAAAATATAAATATAGAGTAAAAACTGTTATATTGGGTACTCTAAAACGATATTCTATCCTTTCTCTTACTCTATTTAGAGTGG
TACTGTACAAACTATATACTCTATAGTGACGGCCCCACTAGCTAAACAAAGGAAAAAATGATATAACTCACAAACCAGAAATC
TAAATTAAATTTCGATTTTACCATTAAATTCGTCTCAATAAATCCTTCAAAACTAGATCACACATGAATATATTTAGATAAAGTG
ACAAGTGGATCCCACAGTGGATAAAGTGGATAGAGGATGAATAGAGGATGGACGGGTGCGGAAGAATTGAGTATAGAGGAG
AGAATCTTGCTGACTAGCACATATAGAGGATGAATATAGAGGTCCATGAGTGCGAGTAGTCTGATGACGCTCAGATACATAAG
AAATCATAAGACTACCCGCACTCATGGACCTCTATATTCATCCTCTATACGTTCTAGTCAGCAAGATTCTCTTCTCTATACTCAA
TTCTCCCGCACCCATCCATACTCTATATCATCCTCTATCCACTCTATCCACTGTGGGACCCACTTGTCACTTTATCTAAATATATC
CATGTGTGATCTAGTTTTGAAAGATTTATTAAGACGAAATTAATGGTGCAATCGAAATTTAATTTAGATTTTTTGTTTGTGAGT
TATAGCATTTTTTTTCATTTGTTTAGCTAGTGGGGCCCGCCACTATAGAGTATAGAGTAAACACCGTACCACTCTAAATATAGA
GTGAGAGAAAAGATAGAGTATCATTTTAGAGTACTTAGTGCAGCAGTTTTTACTCTATATTCATACTCTAAAGCTTTAGAGTTT
CGTTTTAGAGTACCCACTGCGGGCAGTCTAATACGGCATACTGCATACACCAGTGATTCAGTCCAGTGATCGACTCAGCGAC
AAGTGATGAGCATTCAACGGCAGATCAGACACTAGTTTTTCCGGTTGAATCCTGAACGCAAAGCACCATTGCTGGTTTTCAT
GTGAGCTTCAGCAGAACGAATACGATGCGCCAGTGTGAGTGTTCTGACTTTCTGAGTGAGCAAGAAGCCTCATATAAACGA
CGGCTGGGAGGGAGAGTTCATGGGATGCAGACTGCGCTGCCAATGTCGCGTCGGCCTTTACTCCGCGTGGCTCCAACGGTG
CACAAATGGGCTCACGCCGGTCGCACTCGTTCCGGCGGAGGCGCCACGCCCCGCGCGGCCGCGCCCTCCTCCCGCACACGC
CCATTAATCCCTCACGCACCGGGCCGTTGCACCATCAACTCCCTCCCCTCCGCGCCATTACTGCTACATACACGGATACACCT
ACGTGCCCTGCGCCGAGGCCGAGCCGCTCCCCGCGCCCACGCTACCTGCACTCGCCGCTCGCTTGCCTCGGGCTCGAGCAT
TGCAATGGC
>SsLRR-RLK190
TGTTCCCCTAAATATTGGTGAATTGTAGAACTTTCTCCTCATGTAAACACTAACATGCAACTTGGTAGATCTGGTTTGCATTAG
TGGAACCTGAAACATATTTTAGAACTAACACATGTATCTTTTGCCAGCTGAAATCTGCTCTGTTACTTCACGTTGATGGATCC
ACCGCAGTTTCTGAGAATAATGGTCGCCAGGTATTCTACTATTCTAGCCTAAACTTCTCAGAATTGCAGCAGCTTCTCCCATC
CTGATTAATAAGATTTTTCCTCATACTATCTCATGCAGATGTTAACTTATGGGCGAGTCATGCCGTTCCTAGAGCTTTTTGCGC
GCATTGATGCTGTTGACTGTGCTACAGTAATGGAAACTGCCAAGGAGTACATAATTGATAAGGTAAATCTCCAAAAGTGGTG
GCTATTCTACTTATGCAGAGTACTACAATATCTTTGGTGAACGGGTGTGTAGTATAGTGGTCACAGGAGCTTTAGTAGCATATT
AGTCCTGGGTTCGACTGTCCGTGAGAGCGAATAACAGACCTAGGTTAAAAAAACACCCCTTGTCGGCAGTCTCCCTGAGTC
TAGGGCAGCTGGCCTTGGGGGTGTTTCTTGGCCTGTGTACGAGAAACTTAATGCAATGTTTCCCCGCAGGAGTTTTAATACA
GTATCTTTGAATACTTAGTTACTGGAGGCCTCATACACTGACATGATTGGTTTGGCAATTTACAGGATGTTGCTCTGGCTGGA
GTGGGACAGCTCACGAATTTGCCAGAGCTTAGTTGGTTTCGCTCGGAAACATGTTCTGATGATGATTTTACTCGAAGGATATT
CTTTGGGAATGCAAAATAGTTGAAGCTGTGGCTCTGTTATAGTGTTGTTGTAACTCTAGGCCATTCATTCCAAGAAATTGGTA
CCATTTATATATATCCGACTAAATGATATTTGTGGGAGAAATTACATTTGCATCGAACACTATTTATTCTTTAGGAATACATGGAT
ATATTGAAACAATAGCAGATCTATAATTTTGATAATTCATGAATGAAATTGTGCCTAAAATATAAATTTTTCTTTTTGAATAGAA
CAAATTGAGTTGAAAATAAAAGGTAAAATGCATTTTGATATTTTATGGATGAAATTGAGCCTATATTGCAAGTTTGAGGATTGA
GACGATCATCTTCACAGTTGTTGGATGAAATTGAGTCTAAAATTAAAGTTTGAGGAGTAAAACAATCATTTTCATAGTTATAG
GATGAAATTGAGTATAAAAGTAGAGTTTGAGGACTAAAACGGTCAATTTAAAAGTTCAGGGACGAAGCTGAGCTTCGAGTA
ATAGTTGAGAGATTAAAATGGCTATCCTGCCTGGAGGCAGAGACAATGCAGCCGTGCAGGAGCAATGATAGCTACGGCCGT
GTCGTGTCGTTTTGTTTTTCCGGCCAGATTGATGACGCTCAGATACACAAGAAACCATAATACGGCATACTGCATACACCAGT
GATTCAGTCCAGTGATCGACTCAGCGACAAGTGATGAGCATTCAACGGCAGATCAGACACTAGTTTTTCCGGTTGAATCCTG
AACGCAAAGCACCATTGCTGGTTTTCATGTGAGCTTCAGCAGAACGAATACGATGCGCCAGTGTGAGTGTTCTGACTTTCTG
AGTGAGCAAGAAGCCTCGTATAAACGACGGCTGGGAGGGAGAGTTCATGGGATGTAGACTGCGCTGCCAATGTCGCGTCGG
CCTTTACTCCGCGTGGCTCCAACGGTGCACAAATGGGCTCACGCCGGTCGCACTCGTTCCGGCGGAGGCGCCACGCCCCGC
GCGGCCGCGCCCTCCTCCCGCACACGCCCATTAATCCCTCACGCACCGGGCCGTTGCACCATCAACTCCCTCCCCTCCGCGC
CATTACTGCTACATACTACGTGCCCTGCGCCGAGGCCGAGCCGCTCCCCGCGCCCACGCTACCTGCACTCGCCGCTCGCTTG
CCTCGGGCTCGAGCA
>SsLRR-RLK52
GCGCCCATTAGCCTGTGCCTTTGCCGTAATCATTTGTGCGCTCAGGGTCATGGCAGCAGCGGCTGTCCCGGCCCGACTGCGT
CCACTAGCTCACGGCGCCATGATTTGCATTTGTTCCAGTCCACCACTGATCCCAGCACTACCAGCGGCTTGTTCGTTTGCACG
AGGTTACTATCTCGTAGTAGTGCTAGCACAGAGCAAGAACTACGGCCACAGTTTTTCTGAAATCCTAATGTTGTCGTCATGGA
AAAAGGGCCGGCAGCCGGCTACGGCCAACATCCTGAGCCCTCACGATCACCGATGCCCCTCCGGGCTCCGGAGCTCACCAC

GGCCTCGGATCCGTCCGCGGCGACACAGTGGCAGGCAAGAGGCACTGAGGCAGGCAGGCAGCCCCGCGCGTCGGCGTCG
GTACAGCGCAAGGGGACAAGGCCGTAGATCAGCGCCATCGCGCTGGCCATAAAGCAAACAAAAGCGGCAGGAAACACAG
ACGTCCGGACGAGCGCTACGGCGCACGGAGAAGCGAACAAGTTGGCTTTCCACGGCGTCGTCTTGTAGCCTCCGGAAGCG
GGAGAAGGGCCCTCCGTGGCTCCGTCCCGACCCGCTCTGCTCGTTGCTCCTGTCGCCGAGCGATTTGCGAACCAAAACAAA
ACGACCATGTGTGTTTTTTGTTCATAATGCAGGAAGGAGAGGCCAGCCCCGGTGTTTGTTCTCGCCTTTCTTTTTCAAGCAG
AAGCTTTTGCTCTGTGCCATGCGTGTGACCACTGACCACCATGTGTTTCGCTAGAGAGCTAAAGAAATGAAGGGAGATCTAA
GCACAGCACCAGAAATAATAATTCTGCTACTCCACTTGTCCACTTGTACAGAAGCGACCAAAAATCAAATAAAACCCCGCGT
CCGTCTGGACGTCTGGAGCCACCGCAGACCAGCATTTTTTTCCCTCCTTCTCGCAAGCAGTCACGGCTGATGCATACAGTAC
ATGAATTATCAGCAGTCGGATCGGAGGACACTGGCGATTACTGTACGAGCCGGTCCGGGGCCACGTCACCAAGGACAGCGG
CACAGCGCAGGACTGAGGATACGACGGCTGTATGCTGTACCATGCTTCCCCTGTGCGCCGGCAACCGTCGTGCCATCGTCGC
CTGCCTCCCAGAGTCCCAGTCTCAGAGGCCGCCACGCTCCTCCCCTCCGACCCGCGTCTCAGTCTCAGCCCCGCGCATGTTC
GGTGGCAGTGTCACCGCAGCTGACGTCTGGGCCTCCAAATGCAACGGGGGAGGAAAAGGGGATTAAAAGCACTCGATGGC
CGCTTCCCTCGTCGTCTCGCCTCAAATCATGGCGATATGAGTACTACGCTTCACACGCTCAACAGTTCATCAGCAACATAATC
GTAGGACCCAGACCAGCACGACGAAAGTTGAAAGTAGCACTGGTCACTGGACTACTCCTACAGTCCTGCTAACGGGAAAG
AGGAGAATGAATATTTCTTTGTAGTGGTAGTGCCCAGGGCCGAAGTCAGGATCACACCGGAGTCAGGGCTGCTTCCTCTTCT
TCTTTCTCCCGTCCCTCCTTCCCTTCTTCTTTCTCCTTACAGGGCTTAAGAGGGGTTGTATGTTCACATGGCCAGTAAGGAGG
CTTCCCTTCTTCTTCCTCCTTGCAGGGCTTAAGAGGGGCTGTGTGTTCACATGACCGGTAAGGGGTGGAGCCGGGTTGCCCC
CCGCTGGCTTCGTCCATGGTAGTGCCTACACAAACTACTGTTGTCTCGGATGAGAGTAGTACGATCGTGTACAAAAGGTACC
TTTGCGAAGTAAAGCCACGCACGCATTGGACTGCTTCGTTTTTAATTTCGGTTTTCTGATGATCTCCCAAAAAGAATTCGCAA
ACCTTTTCTGCACGGTCACGCTTTTTGGTTGACAGTGCCAGACAAGGGGCCTCGAATCATGTGTAAAAGAAATGCGGTCACT
GGATCCATCCTTTCCAGCTGCTCCTTCTCCATCCCTCGGTAC
>SsLRR-RLK3
ACTGACCACCATGTGTTTCGCTAGAGAGCTAAAGAAATGAAGGGAGATCTAAGCACAGCACCAGAAATAATAATTCTGCTAC
TCAACTTGTCCGATTCGTCGAGCTGTAGATTTAATTAATCAGTGCACCAGAGTTGGTTTGGTACAGTACAGTACAGAAGCGA
CCAAAAACCAAATAAAACCCCGCGTCCGTCTGGACGTCTGGAGCCACCGCAGACCAGCATTTTTTTCCCTCCTTCTCGCAAG
CAGTCACGGCTGATGCATACAGTACATGAATTATCACTGTACGAGCCGGGCCGGGGCCACGTCACCAAGGACAGCGGCACA
GCGCAGGACTGAGGATACGACGGCTGTATGCTGTACCATGCTTCCCCTGTGCGCCGGCAAGAGGCCGCCACGCTCCTACCCT
CCGACCCGCGTCTCAGTCTCAGCCCCGCGCATGTTCGGTGGCAGTGTCACCGCAGCTGACGTCTGGGCCTCCAAATGCAAC
GGGGGAGGAAAAGGGGATTAAAAGCACTCGATGGCCGCTTCCCTCGTCGTCTCGCCTCAAATCATGGCGATATGAGTACTAG
TACGCTTCACACGCTCAACAGTTCATCAGCAACATAATCGTAGGACCCAGACCAGCACGACGAAAGTTGAAAGTAGCACTG
GTCACTGGACTACTCCTACAGTCCTGCTAACGGGAAAGAGGAGAATGAATATTTCTTTTTAGTGGTACACAAACTACTGTTG
CCTCGGATTAGAGTAGTACGATCGTGTGCAAAAGGTACCTTTGCGAAGTAAAGCCACGCACGCATTGGACTGCTTCGTTTTT
AATTTCGGTTTTCTGATGATCTCCCAAAAAGAATTCGGGCCACGTTTAGATTCCCAATTCTCCCCCCAAATTCCACTATGCAA
AAAGAAGATTCCCCGTCACATCAAAATGTCGGCAAATGCATGGAGTACTAAATGTTGATGAAATCAAAAACTAATTGTACAG
TTTTGTTGTACTTTGCGAGACGAACATTTTAAGCCTAGTTAGTCAAAATTTGGATAATTTTTACCAAATACAAACGAAATGCTA
CAGGAATTCTGATACAGTGTTTTTCACCCCTATTCCGCAATCTAAACGAGGCCTCGCAAACCTTTTCTGCACGGTCACGCTTT
TTGGTTGACAGTGCCAGACAAGGGGCCTCGAATCATGTGTAAAAGAAATGCGGTCACTGGATCCATCCTTTCCAGCTGCTCC
TTCTCCATCCCTCGGTACCGACACCTCACTCCCTTCGCCTACCTCCCTTCTTCCTGCACTCCACTCGGTCAGTACCAAGAGCA
GGCTCGCGCTGGTGCGGTGCTAGTCCCACACTCCCACTACTCAGCCACTCCCTCACCCACTCTCTCCAGTGTCCACACACGC
CATTGCCGCCGCTCCTCATCGTACCCACTCCCCAAATGGAGCAGCCTGCAATTCCCCTCTTGCTCCGTGGAGCGCATTCGCTC
CAGCGCTGACACGGGGAAGCTCTAACGCGTCGCCGCTGCCGAATCCTTCCACCCGACGGCCACAGCTCCTCTCTCAGTCTA
GTCGGGGTGGAGATCCGCCAAGGCCATCTCGTCGTGTTGTTGGTCAGTAATCCTAGCTTAGCTTGCTTGGACGAAGCAACCA
ACACAATGCCTTTTCTTTTCTTGGATGACAAAATTCGACCTTGGCCTGCTCACATCTTCAGCGGTGTCCGTCTCTTCATGTCT
CGGTTCTTGGCTCCGTCCTTGCTGATTTCGCTGCTTTCACACTGAAAATCCTCAAACCCTGCATCCTTAATCCCCCTTCTGGG
TTCCAAGGGGCTTAAACCCTATCCTACTTCCTTTTTCATCTCGAGCTCCGTATTCCGAAAAATTCATCTCATTTGCATGAGCAT
TGCTTCAATAACCTGGCGGCAAAGAAGCATCTTGCAATGCGGCTCATCGTATTGTGGCTCTCGATCTGGGCCGTGTGTTCCGC
TGCCTCGGCGGGGGCTGGTTCAGAC
>SsLRR-RLK40
TTGCTCCTGTCGCCGAGCGATTTGCGAACCAAACAAAACGACCATGTGTGTTTTTTGTTCATAATGCAGGAAGGAGAGGCCA
GCCCCGGTGTTTGTTCTCGCCTTTCTTTTTCAAGCAGAAGCTTTTGCTCTGTGCCATGCGTGTGACCACTGACCACCATGTGT
TTCGCTAGAGAGCTAAAGAAATGAAGGGAGATCTAAGCACAGCACCAGAAATAATAATTCTGCTACTCCACTTGTCCACTTG
TACAGAAGCGACCAAAAATCAAATAAAACCCCGCGTCCGTCTGGACGTCTGGAGCCACCGCAGACCAGCATTTTTTTCCCT
CCTTCTCGCAAGCAGTCACGGCTGATGCATACAGTACATGAATTATCAGCAGTCGGATCGGAGGACACTGGCGATTACTGTA
CGAGCCGGTCCGGGGCCACGTCACCAAGGACAGCGGCACAGCGCAGGACTGAGGATACGACGGCTGTATGCTGTACCATG
CTTCCCCTGTGCGCCGGCAACCGTCGTGCCATCGTCGCCTGCCTCCCAGAGTCCCAGTCTCAGAGGCCGCCACGCTCCTCCC
CTCCGACCCGCGTCTCAGTCTCAGCCCCGCGCATGTTCGGTGGCAGTGTCACCGCAGCTGACGTCTGGGCCTCCAAATGCA
ACGGGGGAGGAAAAGGGGATTAAAAGCACTCGATGGCCGCTTCCCTCGTCGTCTCGCCTCAAATCATGGCGATATGAGTACT
ACGCTTCACACGCTCAACAGTTCATCAGCAACATAATCGTAGGACCCAGACCAGCACGACGAAAGTTGAAAGTAGCACTGG
TCACTGGACTACTCCTACAGTCCTGCTAACGGGAAAGAGGAGAATGAATATTTCTTTGTAGTGGTAGTGCCCACTGCCCAGG
GACGAAGTCAGGATCAGGGCTGCTTCCTCTTCTTCTTCCTCCCATCCCTTCTTCCCTTCTTCTTCCTCCTTGCAGAGCTTAAG
AGGGCCTGTGTGTTCACATGGCCGGTAAGGGGGGCTGGAGCCGGGTTGCCCCCGGCTGGCTTCGTCCATGGTAGTGCCTAC
ACAAACTACTGTTGTCTCGGATGAGAGTAGTACGATCGTGTGCAAAAGGTACCTTTGCGAAGTAAAGCCACGCACGCATTG
GACTGCTTCGTTTTTAATTTCGGTTTTCTGATGATCTCCCAAAAAGAATTCGCAAACCTTTTCTGCACGGTCACGCTTTTTGG
TTGACAGTGCCAGACAAGGGGCCTCGAATCATGTGTAAAAGAAATGCGGTCACTGGATCCATCCTTTCCAGCTGCTCCTTCT
CCATCCCTCGGTACCGACACCTCACTCCCTTCGCCTACCTCCCTTCTTCCTGCACTCCACTCGATCAGTACCAAGAGTAGGCT
CGCGCTGGTGCGGTGCTAGTCCCCACTCCCACTACTCAGCCACTCCCTCACCCACTCTCTCCAGTGTCCACACACGCCATTG
CCGCCGCTCCTCATCGTACCCACTCCCCAAATGGAGCAGCCTGCAATTCCCCTCTTGCTCCGTGGAGCGCATTCGCTCCAGC
ACTGACACGGGGAAGCTCTAACGCGTCGCCGCTGCCGAATCCTTCCACCCGACGGCCACAGCTCCTCTCTCAGTCTAGTCG
GGGTGGAGATCCGCCAAGGCCATCTCGTCGTGTTGTTGGTCAGTAATCCTAGCTTAGCTTGCTTGGACGAAGCAACCAACAC
AATGCCTTTTCTTTTCTTGGATGACAAAATTCGAGCTTGGCCTGCTCACATCTTCAGCGGTGTCCGTCTCTTCATGTCTCGGTT
CTTGGCTCCGTCCTTGCTGATTTCGCTGCTTTCACACTGAAAATCCTCAAACCCTGCATCCTTAATCCCCCTTCTGGGTTCCA
AGGGGCTTAAACCCTTCCCTACTTCCTTTTTCATCTCGAGCTCCGTATTCCGAAAAATTCTTCTCATTTGCATGAGCATTGCTT
CAATAACCTGGCGGCAAAGAAGCATCTTGCA
>SsLRR-RLK22
TTTATCCTGGTTCGGGCAAGAGAAGGCCCTACGTCCAGCGGGGGAGGGAGAGATTTGTATTTCCTTGCACCGAGGTGCTTGT
GTAGGGGTTACAAGCTTGCGAGAGAGGGTAGTGAGCCCAGGTCTCAAAAAAAAGAAAAAGAAAGTACAAGATGATTGCTT
GAGATGTTGATCTCGATCTTGTACGCGGCTCGTTGTCCGTCGTCCTCTGTTGTGCTGTGTTAGGCGTCGAGGCTGGGTCGAG
GCCGGGTCGAGGCCGATTGGATAAGTCTGAATCCCTTGGATCGCGGTGTACGGGCCTTGTCGCATCCTCCCTCGAGGGTAAC
TAGTGATGTATAGTGTGCGGTAAGGTGGGTACAGTGACGTTTTTCACCTCCCATCACGGGATGGGCGGTAAAACAGTGAGGA
CTGACACTGGTATTGTTCCCACGTCGACCCTGGTTGCGGCCATGCCGGTAGGTGGTAGTAGGTGATTGCATTAAATGCTATGA

CTTTCGTCCCGGGGTAGGTGGAGAGAGAGGATCGAGCGCCTCGACCCGGGCTCACACGAGACGGAGTTCGGTCGAGGCGT
TTTGTCGTGCGGGACGGGGTGCGGTCGAGGCTTCCCGTCGAGGGGGTCTCGTACGAGTCGGAGACGGGATGCGGTCGAGG
CATTCCGTTGAGCCGGCCTCATACGAGGCGGACCTGACACGCGTTTTGAAGTGGGCTATGTTCTACCGTGATTTAGTCGCTTT
TGTTTTTATTATCGTTGAGTGTGGGTACCCCTATACTACTGGTACCGACAATTAAATGTAGGTAAAAAATAACTAATTGCACAA
TTTGTCTGTAAATGTCGAAACGAATCTTTTAAGCCTAGTTAGTCCATAATCGAATAAATTTTGTCAAATAAAACGAAAAGTTG
TACAGTAGCCGAACCCAAACTTCTTCACCAAGGCCGCGTTCACTTCCTACAGGAACCCAAAATATTTCCTCTTCCACCCATC
ACATCAAATCTTTAAACGCATGTATGAAACATTAAATGTAGGTAAAAAAAAACTAATTACATAGTTTGTCTGTAAAGTTCGAG
ACGAATCTTTTAAGTCTAATTAATCTATGGTTGAACAAATTTTACCAAATAACAACGAAAAGTGCTACAATAACTTTTCACCC
CCAACTTTTTCATCTGAACACACCCCCAACTAAACAACCCCTCCGCAAACCTTTTCTGCACGGTCACGCTTTTTGGTTGACA
GTGCCAGAGAAGGGGCCTCGAATCATGTGTAAAAGAAATGCGGTCACTGGATCCATCCTTTCCAGCTGCTCCTTCTCCATCC
CTCGGTACCGACACCTCACTCCCTTCGCCTACCTCCCTTCTTCCTGCACTCCACTCGATAAGTACCAAGAGTAGGCTCGCGCT
GGTGAGGTGCTAGTCCCACACTCCCACTACTCAGCCACTCCCTCACCCACTCTCTCCAGTGTCCACACACGCCATTGCCGCC
GCTCCTCATCGTACCCACTCCCCAAATGGAGCAGCCTGCAATTCCCCTCTTGCTCCGTGGAGCGCATTCGCTCCAGCACTGA
CACGGGGAAGCTCTAACGCGTCGCCGCTGCCGAATCCTTCCACCCGACGGCCACAGCTCCTCTCTCAGTCTAGTCGGGGTG
GAGATCCGCCAAGGCCATCTCGTCGTGTTGTTGGTCAGTAATCCTAGCTTAGCTTGCTTGGACGAAGCAACCAACACAATGC
CTTTTCTTTTCTTGGATGACAAAATTCGAGCTTGGCCTGCTCACATCTTCAGCGGTGTTCGTCTCTTCATGTCTCGGTTCTTGG
CTCCGTCCTTGCTGATTTCGCTGCTTTCACACTGAAAATCCTCAAACCCTGCATCCTTAATCCCCCTTCTGGGTTCCAAGGGG
CTTAAACCCTTCCCTACTTCCTTTTTCATCTCGAGCTCCGTATTCCGAAAAATTCATCTCATTTGCATGAGCATTGCTTCAATAA
CCTGGCGGCAAAGAAGCATCTTGCA
>SsLRR-RLK101
CGTTTTCGTTGTTATTTGACAAATATTATCCAATTATAGTCTAATTATGCTTAAAAGATTCGTCTCGTCTATTTCGTCTAAACTGT
GTAATTAGTTTTATTTTTTATCTATATTTAATGCTTCATGCATACGTCCAAAGATTCGATGCGACGGAGAATCTTGAAAAATTTG
GCATTTTAAGTGCAACTAAGCGGGCCCTAACAGCAAGCATCACGGATTGGGAATTTGGGATATCATCCATCCAGTACCACCTT
TAAGTAGCACTCCTACATTATTACACGACCTGACATGCACTCTTCTCGCCATTTCTTCTGCTCACCGTCCTTAAATACTACCTA
GGAAAAATGTACTCAGCCCTGGTTTAGTTTTCAGAATTTGCCCCAAAATTCTACTATGCAAAAAGAAGATTTCCATCATATCA
AAATACCGGCACATGCATGCAGTATTAAATGTAGATGAAATCAAAAACTAATTGCACAATTTTATTGTACTTTGCGAGACGAA
CGTTTTGAGCCTAATTAGTCAATGTTTGGATAATTTTTACCAAATGCAAACGAAATGTTACAGTACACTACAGTGCTGTACAG
TGATTTTGGCAGCGCCAACTTTGGGCAACTAAACATGGCCTTAGAAAAGGGCAAAATTACGTGGAGCAAATTGGGTGGTGT
ACGTACGTATTTGATGAGAAACCATTGTGTTTTGACCTGTGAATTTCAAGTTTCGCGGAGGGATAAATATGTCTCCCGTGATC
TGCATCTACCTGGTACAGAAATCTTCGGTGGCTGGACTCGGACATGTCTCCTGAATCTAAGGTCCCAATGTCTCTCTGAAACG
AGGCTCTATCCGTGAGTTGCTGAAATTAATCCCCACACTAAGCAAACTTCCATTGTTTTATCACGTCAACACGTCCTCATCAC
CAAATATGTGAATTTGATACGCGTTGATTTTTTCCCACACTAGTTCGGTTCTGAACTTCTTGACGTGGGCAGCTGGCCTGTTG
AGTGGTCTGAAGAAAGAAAGCATATGGAGAGAGAGAGAGGTGAGGAAATGGAAAGCAAGGGGCCGGGCAGGGGCAGGG
GCAGGGGCAGCTCACCTGAGCTGGAGAGGAAAGGGGTCAAAGACTGGAACAGCCTAGGGGCATGTGGGGGGCTCTTGCTT
TCCTACCGGTACCCAGTGCCTACACACATCAGTTTTGCAGATTCTTTTGTGTTTTCTTTTGCCCTCCCTTTTTTTTTTGATTAAG
ATGGATGACAGGATGACTGGATGAGGGAGCAATCTTTTTGCCTTCTTGGTTGCTCAAATCTTTGTTCATTACTGTACTTGATAT
GTTCATCAGGAACAGGCTAAATGCTGGATGAACAGCTAACAGTTATCAAAATTTTATTATGTTGGTACAAAACCCCTCATTTC
TAGACAGAGAAACAAACAGGTAAACAGACAGACAATAAAGCAAGAATTCACTAGTACATACAAAGAACGAAAACAAGACA
TAAATGAGTAGGAAAATGCCTCACAAGGATCACATTTTGCTACAGATGTGTCGTCTTTTTTTTTTGATAAGGCTACAGATGTG
TCGTCAGCAGCCCAAAACCATGTACCCACTCCACAGACCACAGTAATACCAGTCCAAAGCAAAGCAAAGCAAAGCACCCA
GGTCACAGCAGTCTCACTCACAGCTAAGCACTCCAAAACTGCTAAGCACACCACTCTCGCTACCCCTACTAGTCACCACTCA
CCAGTCTCTCTCGCACAGCACAACCACCTGTGATGTGACTTTCCAGTCTGTACTCTCTCTCTCTCCTGTTATTACTCACCCATC
CCCTCCCGTTTCCAAAAATTTGGCCTTTCTCTCGGCTCTCGCACATCTCACTACCACCACCTCACCTCTCTCTCCCATCTCCA
ATGGCGGCCGCCACCCGCACCTCCCTTCCCTCCTCCTCCGCTTCCCGCAAGCCCTAACCCTAGGTAGGTCACTCCAGTTCCC
CACACCCCCCATTTCT
>SsLRR-RLK100
AGCGTTTTCGTTGTTATTTGGCAAATATTATCTAATTATAGTCTAATTATGCTTAAAAGATTCGTCTCGTCTATTTCGTCTAAACT
GTGTAATTAGTTTTATTTTTTATCTATATTTAATGCTTCATGCATACGTCCAAAGATTCGATGCGACGGAGAATCTTGAAAAATT
TGGCATTTTGAGTGCAACTAAGCGGGCCCTAACAGCAAGCATCACGGATTGGGAATTTGGGATATCATCCATCCAGTACCAC
CTTTAAGTAGCACTCCTACATTATTACACGACCTGACATGCACTCTTCTCGCCATTTCTTCTGCTCACCGTCCTTAAATACACC
CTAGGAAAAATGTACTCAGCCCTTGTTTAGTTTTCAGAATTTGCCCCAAAATTCTACTATGCAAAAAGAAGATTTCCATCATAT
CAAAATACCGGCACATGCATGCAGTATTAAATGTAGATGAAATCAAAAACTAATTGCACAATTTTATTGTACTTTGTGAGACG
AACGTTTTGAGCCTAATTAGTCAATGTTTGGATAATTTTTACCAAATGCAAACGAAATGTTACAGTACACTACAGTGCTGTAC
AGTGATTTTGGCAGCGCCAACTTTGGGCAACTAAACATGGCCTTAGAAAAGGGCAAAATTACGTGGAGCAAATTGGGTGGT
GTACGTACGTATTTGATGAGAAACCATTGTGTTTTGACCTGTGAATTTCAAGTTTCGCGGAGGGATAAATATGTCTCCCGTGA
TCTGCATCTACCTGGTACAGAAATCTTCGGTGGCTGGACTCGGACATGTCTCCTGAATCTAAGGTCCCAATGTCTCTCTGAAA
CGAGGCTCTATCCGTGAGTTGCTGAAATTAATCCCCACACTAAGCAAACTTCCATTGTTTTATCACGTCAACACGTCCTCATC
ACCAAATATGTGAATTTGATACGCGTTGATTTTTTCCCACACTAGTTCGGTTCTGAACTTCTTGACGTGGGCAGCTGGCCTGT
TGAGTGGTCTGAAGAAAGAAAGCATATGGAGAGAGAGAGAGGTGAGGAAATATGCCGGGCAGGGGCAGGGGCAGGGGCA
GGGGCAGCTCACCTGAGCTGGAGAGGAAAGGGGTCAAAGACTGGAACAGCCTAGGGGCATGTGGGGGGCTCTTGCTTTCC
TACCGGTACCCAGTGCCTACACACATCAGTTTTGCAGATTCTTTTGTGTTTTCTTTTGCCCTCCCTTTTTTTTTGATTAAGATG
GATGACAGGATGACTGGATGAGGGAGCAATCTTTTTGCCTTCTTGGTTGCTCAAATCTTTGTTCATTACTGTACTTGATATGTT
CATCAGGAACAGGCTAAATGCTGGATGAACAGCTAACAGTTATCAAAATTTTATTATGTTGGTACAAAACCCCTCATTTCTAG
ACAGAGAAACAAACAGGTAAACAGACAGACAATAAAGCAAGAATTCACTAGTACATACAAAGAACGAAAACAAGACATAA
ATGAGTAGGAAAATGCCTCACAAGGATCACATTTTGCTACAGATGTGTCGTCTTTTTTTTTTGATAAGGCTACAGATGTGTCG
TCAGCAGCCCAAAACCATGTACCCACTCCACAGACCACAGTAATACCAGTCCAAAGCAAAGCAAAGCAAAGCACCCAGGT
CACAGCAGTCTCACTCACAGCTAAGCACTCCAAAACTGCTAAGCACACCACTCTCGCTACCCCTACTAGTCACCACTCACCA
GTCTCTCTCGCACAGCACAACCACCTGTGATGTGACTTTCCAGTCTGTACTCTCTCTCTCTCTCCTGTTATTACTCACCCATCC
CCTCCCGTTTCCAAAAATTTGGCCTTTCTCTCGGCTCTCGCACATCTCACTACCACCACCTCACCTCTCTCTCCCATCTCCAAT
GGCGGCCGCCACCCGCACCTCCCTTCCCTCCTCCTCCGCTTCCCGCAAGCCCTAACCCTAGGTAGGTCACTCCAGTTCCCCA
CACCCCCCATTTCTA
>SsLRR-RLK81
CTCCTACATTATTACACGACCTGACATGCACTCTTCTCGCCATTTCTTCTGCTCACCATCCTTAAATACACCCTAAGAAAAATG
TACTCAGCCCTTGTTTAGTTTCCAGAATTTGCCCTAAAAATTCCACTATGCAAAAAAAAGATTTCCATCATATCAAAATACCG
GCACATGTATGCAGTATTAAATGTAGATGAAATCAAAAACTAATTGCACAGTTTTGTTGTACTTTGCGAGACGAACGTTTTGA
GCCTAACTAGTCAATGTTTGGATAATTTTTACCAAATGCAAACGAAATGTTACAGTACACTACAGTGCTGTAGAGTGATTTTG
GCATCGCCAACTTTGGGCAACTAAACATGGCCTTAGAAAAGGGCAAAATTACGTGGAGCAAATTGGGTGGTGTACGTACGT
ATTTGATGAGAAACCATTGTGTTTTGACCTGTGAATTTCAAGTTTCGCGGAGGGATAAATATGTCTCCCGTGATCTGCATCTAC
CTGGTACAGAAATCTTCGGTGGCTGGACTCGGACATGTCTCCTGAATCTAAGGTCCCAATGTCTCTCTGAAACGAGGCTCTA
TCCGTGAGTTGCTGAAATTAATCCCCACACTAAGCAAACTTCCATTGTTTTATCACGTCAACACGTCCTCATCACCAAATATG

TGAATTTGATACGCGTTGATTTTTTCCCACACTAGTTCGGTTCTGAACTTCTTGACGTGGGCAGCTGGCCTGTTGAGTGGTCT
GAAGAAAGAAAGCATATGGAGAGAGAGAGAGAGAGGTGAGGAAATGGAAAGCAAGGGGCCGGGCAGGGGCAGGGGCAG
GGGCAGCTCACCTGAGCTGGAGAGGAAAGGGGTCAAAGACTGGAACAGCCTAGGGGCATGTGGGGGGCTCTTGCTTTCCT
ACCGGTACCCAGTGCCTACACACATCAGTTTTACAGATTCTTTTGTGTTTTCTTTTGCCCTCCCTTTTTTTTTTGATTAAGATG
GATGACAGGATGACTGGGGCCATGTTTAGGTTGCAATAAAACTTCAAACCGATGAATAGTACTACTTTCGTCTTATTTGATAA
ATATTGTCCAATCGTGGATCAACTAGGCTCAAAAGATTCATCTCGTGATTTCCAACTAAACTGTGTAATTAGTTATGTTTTTTA
CCTACATTTAATACTCCATGCAAGTGGCTAAAAATTGATGTGATGAAGAGAGAGTGAAAAAACTTGGAATTTTGAGGGGAAC
TAAACAAGGCCTGGATGAGGGAGCAATCTTTTTGCCTTCTTGGTTGCTCAAATCTTTGTTCATTACTGTACTTGATATGTTCAT
CAGGAACAGGCTAAATGCTGGATGAACAGCTAACAGTTATCAAAATTTTATTATGTTGGTACAAAACCCCTCATTTCTGGACA
GAGAAACAAACAGGTAAACAGACAAAGACAATAAAGCAAGAATTCACTAGTACACATACAAAGAACGAAAACAAGAAATA
AATGAGTAGGAAAATGCCTCACAAGGATCAGATTTTGCTACCGATGTGTCGTCTTTTTTTTTTTGATAAGGCTACAGATGTGT
CGTCAGCAGCCCAAAACCATGTACCCACTCCACAGACCACAGTAATACCAGTCCAAAGCAAAGCAAAGCAAAGCAAAGCA
CCCAGGTCACAGCAGTCTCACTCACAGCTAAGCACTCCAAAACTGCTAAGCACACCACTCTCGCTACCCCTACTAGTCACC
ACTCACCAGTCTCTCTCGCACAGCACAACCACCTGTGATGTGACTTTCCAGTCTGTACTCTCTCTCTCTCCTGTTATTACTCA
CCCATCCCCTCCCGTTTCCAAAAATTTGGCCTTTCTCTCGGCTCTCGCACATCTCACTACCACCACCTCACCTCTCTTTCTCCC
ATCTCCAATGGCGGCCGCCACCCGCACCTCCCTTCCCTCCTCCTCCGCTTCCCGCAAGCCCTAACCCTAGGTAGGTCACTCC
AGTTCCCCACACCCCCCATTTCT
>SsLRR-RLK117
CGGATTAAACAACCCCAGCTACCGTGGAGACCGGAGACCGCCGGGCAGCAGACGGCTCGGGGGTACCTTCTTCGGGCGCA
CCCGATGCGACGAGGTGAGCGTGCGCGCCTGTTCCGCCTGTGGTTGTGGGGGCGGCCGGATTGGACGGGGAGTTGAACCG
CATTTGAGCCTCGCTTTTGGGGTCCGCGAGGGAAGCCCGACGACGGTGGGGCTTTTGGCTTTTGGCGGGTGCCGCTGCCTG
CCACTGCCACGGCCACCCCAGGTAAAACTCGCGGGAAATCACCCCTCTCCGTTTCGCACTTTCGCGCCTCCACCCTTGCCAA
GTCCTGCGCTGCAATTCGCGTCCCCGGTCCGCGGCCGTACCGTGCCCGTATCCCAGTGTTCCTCCGGAGACCCGACACGTAC
AAATACGTACGGGCACCACCACGCTCCACCTCTGTCCTGTTTCCATGAACTGTGCTCCCTCCCTTCCTTAAAAAAAAAAGTC
ATCAGTCAAACTTTCTCAACTTTGACTAAACTTATAAAATATACGTGCAATATTTATATCTTAAACTAAAGTTAGTAAGAGAGG
TAGTACATGCGTGCTTGCGTAGGTACAAGCGTGCTGCGCCATGCTTGTGCACTGCTATTACGCTTCAGTGTCAAATCTCAAGC
CCAAGCACGCTTCATCTTCGGCCTTTGACCACAGTCCACAAATCCAAAACTACGGTATAATACTATACCGAACTACAGTCTAC
ACACCACACTCTGTAGTTCTGTACACACATCCTCAGTCGCAAAAATTACAGCTTCTCCAGAGGACCACATGGCAAAAAAGG
ATAATAAAAAAAAACGGCGCCGTGACCGTGAGGGCGGAAGCCGGGAACGGGAACCAAAAGGCAACGGAGGAGGACCACG
GACAGAGAAAAGACACTCCCATCCCCCACCCCGCTGCTGCCCGCCTCCACTCTCCACTCCATTGCCCCCCAAGCTGACTCCC
TTCCCCTCTTCTCCTCCGCCGCCCCACGGCCACAGCAGCTGCCGGGAACCAATCCATCCCTAGCTAGCTAGGGTTAGGGTTC
CGCGGCTGCCGTCTTCCCCAGGGCTCCTCCCGCCGGGGCCCCATTGCATTCCCCTGCTCCCCTGCAACCCCCATTATTCTTTC
TCTCCTCCACCCATTCCTTCCTTCCTTCCTTCCCTCCCTCTCCACCCTGCTGCTCGGTCGCCCCAGTGCGGTGCTGCTGGTGG
GCGGTGGCTGCTGCTCTTCGTGCACTGCTAGCACCCGCCCGGTCTCGACGCAAGGTAAGGACACCCGCGGTCCCGCGCCGC
GCCCTGCTCTTCTTGCTTGCATTCGCATCCGCATTGCATCTGGTGTCTGGTGACCGGATGTGTTCTTCCGTGTTTGATGTTTGG
ACGCATTCACCGTGCGAGTGCTACTAAAAGGGTGCGTCCTTTTGACCTGCGTTTATGGGCGGTTCCGCCCGCGGTTGGTGCC
TGTTTTTGCGGCCAAAGATGCCACTTTTACCGCCGTGCTTGTTGGGGGATTAATAAAGCCGTGGGGTTTTTTTGTGTGCTCAA
AGATGATTTTTGTGTTGTCAGAAAAAAAGAAGCATTTATTCTGGCTATTTTTGCTACATTTTCCTTTTTTTTTTTGAGGAATATG
CTAGATATTTACTGCTCTAGTAACTCTTTTCGGAGCTCAAAGATTCGGTCATTCAGGGGGTTTGTTCAAGCTGGAATCTGTGC
CTGAGTTGACATTTTTGTGCTCGTTTCAGGTTAAAAGGAGTTGAAACGGAGAAATCTGGGGGGGGTTTATGTCCATACTGTC
TGCTGCTGCAAAGATCAGCTGAAAGGGACAAGTGTTGTGGCTGGGCTATGAGTACCAAACCAGCTCTCTGATAGCTACGCA
CGCTCGCTCCAACTCCAGTCTCCTCCCAGAAATGCGTCAGTTGCCGCGCTCTTGAATCCTACTCGCAAACTCCCAGCCGCCC
AACCAATCTGCGGAGGAAGAAAGTGAGTGA
>SsLRR-RLK95
TTGGCGCGGCTCTCCTTTATATACGGCAAAACATATTTTTAACCTAGGAGCAAACGGGTACGTAAGGGCAACACAAAGAATC
TAAGAAGTAGATATGGACCGAGTACTCTAGCAGATAGGGTCGGATATATATGAGTAGGGAATACATGGGCAATGGTTACTTCA
TTCACCCATGTCTGTGAGTGGGGGAACTAAGACACCTTGTTGCAAGTAGCTGTAAGGCTTGGAGGCAATGGGCGACAGAGC
AACCATAGGGATGGCACGCCTTGCCATTGGCGGAGCCTCTTTGAGGCGAAGTTGAGCTGCTGTAGGCTATAGTCATCTTATTC
AGCACCGGACCTAAGGCATCAGCTCATCCATAATCCAAGCACGTCTTTTAGGTCAGGTTTTGCCGCTACACCCTGCACCAGG
TAGTTCTCGCACGTCACCGATGGGAGTGTTGGGGTGGTGCCGATGATGATGTGCACGGTGACCACATAGGCGAGCAATGAG
ACCCTGTAGCGTGGTCTATGCGTGTGGGCTGTCTTCTGCTTTGTTCTGGTGCCAGAGCTGCCAGTGGGGTTGTAGGTGTATTT
GCTATAGTAGACTATGCCAAAGAGCGCAAAGTCATCGTCTTGTGTGCTGTATCTATCTAATGTCCATTGTTCGAGCAGAGCGC
ATAGCGGGCGAAGACGATGCTGGAGGGCATGCTCCCTCTAACTTTGTTTTCCAACATACAAGGCTAAGTGGCTAACTTTGTT
GCTTTTCATTACACTGTTTAATGACCTAGGTAATCTATCATGTAATTGAAGAGTAAATGAGCCATACTGAGATTTCCCCCTTCC
ATCATAAGTTCGTACAGTTGGCACTAATCAAATTCCGAGAGAATTTACGCTGCTTCCTCCCAAACAAAAGAAAAACCACGCA
TGTTTTGCTTCGTGTTTAAAGTGATGACTATTGTCAGTGGCGGGTGTAAAAATTCATAGAAGAGGGGCTCAAAGTCATCTTCA
ACGTGGAGCCCCTCCTTTTCTTTCATGCCTCTAAAAATTGAGATAGGAGGGGCTAAAAGAGGGGATCATCCATTATCGTGGA
AGCAATATGAGGGGGCTTGAGTCCCGCCCGCTCGCTCCATCGCTGGATCCGTCGGGGCCAGAGTCCCGCCTGCCCGCTCCAT
CGCTAGATTCGCCGCGACGGAGTGGCGACGGGAACGCGAGCTACCAGTGTTTTGGCCGTGGTTTTCTCGGGGCGGACCCTC
GCGGCGCACGAGGCCAGGACGGCCCCCACCAATTAAACAACCCCAGCTACCGCGGAGACCGCCGGGCAGCAGACGGCTTG
GGTCTCCTTTCCTGCGGCGCGGGCCCTGATGCGACAAGGAGGGCGAGAGCGTGCGCGCCCGTGGGAGCTTGGGTGGGGGC
GGCCGGATTGGACCGGGAGTTGCGCCGCATTTGAGCCGCGCTTTTGGGGTCCGCGAGGGAAATCACCCACAGAAAATCGCG
GGAAATCACCCCCTCCTCTCCGTTTCGCACTTTCGCGCCTCCGCCCATGCATGCCAATTGCCAAGTCAAGCATTCGCGTCCCC
GGTACGCGCCGGCGCCGGCGTACCGTGCCCGTACCGTATTCCAGTGTTCCTCGGGAGATTGGACACGTACGTACGGGCACG
ACCACGGTACCCACCTCCGTCCAGTTTCCATCTACTGTACGTGCGTACGCCCGTGCTGCACCATGCTTGTGCACTGCTATTAC
TACGCTTCAGTGCCAGGACTCTCAAGCCCAAGGACGCTTCACCTTCGGCCTTCGACCACAGTCGACAAGTCCAAAACAACC
GTATACTACACTGTAACTACACCCCACACCCTGTAGTTCTGTTCCTGTACACACATTCTCAGTAGAGAAGTTACAGCTTCTCC
AGAGGACCACACCGCAAAAAAGGACAATAGAAAAAAAAAACGGCACCGTGACCGTGAGGGGGGAAGGGAAACCAAAAGG
CAACGGAGGACCAAGGACAGAGAAAAGACACCCATC
>SsLRR-RLK72
TAAACAACCCCAGCTACCGCGGAGACCGAAGACGGCTCGGGGTACCTTCTTCGGGCGCACCCGATGCGACGAGGTGAGCG
TGCGCGCCTGTTCCGCCTGTGGTTGTGGGGGCGGCCGGATTGGACGGAGAGTTGAGCCGCATTTGAGCCTCGCTTTTGGGG
TCCGCGAGGGAAGCCCGACGACGGTGGGGCTTTTGGCTTTTGGCGGGTGCCGCTGCCTGCCACTGCCACCCCAGGTAAAAC
TCGCGGGAAATCACCCCTCTCCGTTTCGCACTTTCGCGCCTCCACCCTTGCCAAGTCCTGCGCTGCAATTCGCGTCCCCGGT
CCGCGGGCGTACCGTGCCCGTATCCCAGTGTTCCTCCGGAGACCCGACACGTACAAATACGTACGGGCACGACTACGGTCC
ACCTCTGTCCTGTTTACATGTACTGTGCTCCCTCCCTCCCTTAAAAAGAAAAAAAGTCATTTTAAAAAACGTATGATCAAACT
TTCTCAACTTTGACCAGACTTATAGAAAATATGTGTAACATTTATATCTTAACTAAGTTTAGCAAGAGAGGTAGTACGTGCGTG
CTTACGTAGGTACAAGCGTGCTGCGCCATGCTTGTGCACTGCTATTACGCTTCAGTGTCAAATCTCAAGCCCAAGCACGCTT
CATCTTCGGCCTTTGACCACAGTCCACAAATCCAAAACTACCGTATAATACTACACTGAACTACAGTCTACACACCACACTCT
GTAGTTCTGTACACACATCCTCAGTCGCAAAAATTACAGCTTCTCCAGAGGACCACATGGCAAAAAAGGATAATAAAAAAA

ACGGCGCCGTGACCGTGAGGGCGGAAGCCGGGAACGGGAACCAAAAGGCAACGGAGGAGGACCAAGGACAGAGAAAAG
ACACTCCCATCCCTCACCCCACTGCTGCCCGCCTCCACTCTCCACTGTCCACTCCATTGCCCCCAAGCTGACT
CCCTTCCCCTCTTCCCCTCTTCTCCTCCGCCGCCCGCCCGCCCCACAGCAGCTGCCGGGAACCAATCCATCCCTAGCTAGCTA
GGGTTAGGGTTCCGCGGCTGTGCCGTCTTCCCCAGGGCTCCTCCCGCCGGGCCCCCATTGCATTCCCCTGCTCCCCTGCAAC
CCCCATTATTCTTTCTCTCCTCCACCCATTCCTTCCTTCCCTCCCTCTCCACCCTGCTGCTCGGTCGCCCCAGTGCGGTGCTGC
TGGTGGGCGGTGGCTGCTGCTCTTCGTGCACTGCTAGCACCCGCCCGGTCTCGACGCAAGGTAAGGACACCCGCGGTCCCG
CGCCGCGCCCTGCTCTTCTTGCTTGCATTCGCATCCGCATTGCATCTGGTGTCTGGTGACCGGATGTGTTCTTCCGTGTTTGAT
GTTTGGACGCATTCACCGTGCGAGTGCTACTAAAAGGGTGCGTCCTTTTGAGCTGCGTTTATGGGCGGTTCCGCCCGCGGTT
GGTGCCTGTTTTTGCGGCCAAAGATGCCACTTTTACCGCCGTGCTTGTTGGGGGATTAATAAAGCCGTGGGGTTTTTTTTTG
TGTGCTCAAAGATGATTTTTGTGTTGTCAGAAAAAAAGAAGCATTTTTCTGGCTATTTATGCTACATTTTCTCTTTTTTTTTGA
GGAATATGCTAGATATTTACTGCTCTAGTAACTCTTTTCGGAGCTCAAAGATTCGGTCTTTCAGGGGGTTTGTTCAAGCTGGA
ATCTGTGCCTGACTTGACATTTTTGTGCTCGTTTCAGGTTTAAAGGAGTTGAAACGGAGAAATCTGGGGGTTTATGTCCATAC
TGGCTCCTACAGAGATCAGCTGAAAGGGACAAGTGTTGTGGCCGAGCTATCAGTACCACACCAGCTCTCTGATAGCTACGC
ACGCTCGCTCCAACTCCAATCTCCTCCCAGATGCGTCAGTTGCCGCGCTCTTGAATCCTACTCGCAAACTCCCAGCCGCCCA
ACCAATCTGCGGAGGAAGAAAGTGAGTGA
>SsLRR-RLK138
GGAGTATCCTGCTGGCTCGTCTGGATCTGGCAATTCCCCATCTGGGTCAGGAGGTGGAAAATCACTCAGTACAGGTGCTAAA
GTAGCAATTGTAGCAGCTAGCATTGTTGTTCTTGTCATCCTTATACTTATTGCTATTGTATGCCACTACAAGCGAATCTCACGG
CAGTTTCCTAGCTCTGAAAAGGTTTCAGACAAGAGCCTCCACAGGGCTACTAAAGACAGTGCTGTTATGAAAGGAAAAGAC
AACAAAGGTGGTTTGGTCTCAGCTGATGAGCTTGTTACTCCTCGAAAAGGCTCTACATCTGAAGCACTTAGCCAAGAAGAG
AAGTCAGCCACAGGGGGCTTTTCACCGTCTAAGGGCAGCCGCTTCTCTTGGTCACCAGATTCCGGAGAGGCATATGGTCAG
GAAGGATTGGCGCGGTTGGATGTGAGATCACCTGACAGGCTAGCAGGGGAGTTGCATTTCCTGGACGAAACAATCACACTA
ACACCAGAGGAACTTTCAAGGGCACCAGCTGAAGTTCTAGGCAGGAGCAGCCATGGGACTTCATACAGGGCGACACTGGA
GAATGGTGTGTTCCTGACTGTGAAATGGCTGAGGGAAGGTGTTGCAAGGCCCAAGAAAGAGTTTGCAAAGGAGGCCAAGA
AATTTGCTAACATCCGGCATCCAAATGTGGTTGGTTTGCGTGGCTACTACTGGGGCCCAACCCCACATGAGAAGCTGATCTT
GTCAGATTATGTTGCGCCAGGAAGTCTTGCAAGCTTCTTATATGGTAATGCCATCATCTACTACTGATTACTTGTTTCTGCAGT
GCCTTGGATCTTACTAACGGTTCTTGCATACTTCATGTGCATGTCACACTAGTAGTTTCCTCTTACTCATAGACAGCAAAATTC
AGTCACCTACTCTGCTGAGATGGTTCTCTGATTGCACAGATCGACCAGGAAGAAGAGGTCCTCCACTGACCTGGGCACAGC
GGCTGAAGATCGCGGTCGACGTCGCACGGGGCCTGAACTACCTCCATTTTGACCGCGCAATGCCCCATGGAAACCTCAAGG
CCACCAACATCCTGCTGGACGGCCTTGACCTCAACGCCCGTGTAGCTGACTACTGTCTGCACCGGCTGATGACACAGGCTG
GCGTCGTGGAGCAAATCCTGGACCTGGGCGTGCTGGGCTACCGTGCCCCAGAGCTGGCGGCCTCCAAGAAACCGTCTCCCT
CGTTCAAGTCCGACGTGTACGCCTTTGGCGTTGTGCTTCTGGAGCTCCTGACGGGCAGGTGCGCCGGGGATGTCGTCTCGG
GGTCTGAAGGTGGTGTTGACCTCACTGACTGGGTCCGGCTGCGGGTGGCAGAAGGACGGGGATCCGACTGCTTCGATGCG
GCCATGGCCTCAGATTCTGAGAACCCACAGGCTGTGAATGGCATGAAGGAGGCCCTGGGCATTGCACTGAGGTGCATCCGG
CCGGTCTCCGAGAGGCCGGGAATCAAATCTGTGTATGAGGATCTTTCGTCAATCTAGTTAGCTAGCCTCATTGCGTAGTTTAG
ATTAACACTGCGTGTTAAAATGTGTGCGTGTTCTGTCTTGGACTCATAGAACTGCGTANNNNNNNNNNNNNNNNNNNNNNNN
NNNNNNNNNNNNNNNNNNNNNNNNNNNNNNNNNNNNNNNNNNNNNNNNNNNNNNNNNNNNNNNNNNNNNNNNNNNNNNNN
NTGATACAATAAGCAACAAGACTAGCAGCTCCAGCTGTATAAACTATGATTGAATTGAACCAGTGCATTTCATGCTATGGTAA
TGATACCTGAGGTCACACGAATCATTGCCTTCCTTTGAGTAAAAGTAATGGTTAGCCCCCAGAATCAGTACATTAGCAAATAC
TGTTAAGTGTGCTAATGCATTCAGAGTGTTTGCATATTTCCTTTCCTATGATGGCATTCTCTATGATGATAGTATGAGAACCACT
CATTTCTTATCTTTTGTACTAATTGTATTTACTTTATTATGCA
>SsLRR-RLK139
TTCATGACAAATCCGAAGTGGCAAGTTGCTTCAAGAAGTTTGCAAAGAGAGCTCAAAATGAATTTGAAGTCAAGATAAAGA
AAATTAGAAGTGACAATGGCAAAGAGTTTGACAACACCAACATTGAAGCTTATTGTGATGAAATTGGAATCAAGCATGAGG
TTTCTTCAACCTACACTCCTCAACAAAATGGTGTAGTTGAGAGAAAGAACCGGACTTTGATCACCCTTGCAAGAACAATGCT
AGATGAGTACAACACTCCGGAAAAGTTTTGGGCCGAGGCAATCAACACCGCATGCTATGCTTCAAATCGCCTCTTCCTCCAA
AAGTTTCTTGGCAAGACCCCTATGAGTTGCTTAATGGGAAGAAGCCGGACGTCTCATTCTTCCGGGTGTTTGGTTGCAAATG
CTACATCTACAAGAAGCGTCAACACCTAGGGAAGTTTCAAAGACGTTGTGATATTGGTTTTCTTGTTGGTTACTCATCAAAGT
CCAAAGCATATAGAGTATTTAACCATACCACCGGTTTGGTTGAAGAAACATATGATGTGGAATTTGATGAATCTAACGGCTCC
CAAGGAGCAATTGAGAATTGTGATGATGTAGGTGATGAACCCTNNNNNNNNNNNNNNNNNNNNNNNNNNNNNNNNNNNNN
NNNNNNNNNNNNNNNNNNNNNNNNNNNNNNNNNNNNNNNNNNNNNNNNNNNNNNNNNNNNNNNNNNTTTGCGTTTCTGAT
CCTTTATCATGTCTTATTTATCCAATCTAAATGACTGTTTGACCTGCTAGTTACAGAAACAAAAGAACTGAATGATATATGTTG
AGTTTGCAAGAATAGAGTTTAAAGTAGTGTTAGTCCATACAGCTTGTGAAAGAACTAGCCATCTTGCCTATTGGTTGGCTTTA
AAGTAGTGTTAGTCCATACAACTTGTGAAAAAAAAATATCAAGCATAAATTCTCATCTAGCACCGTGTATTGTTGTATACAAATG
ATGATTTGAATTGCTGATTACCCAATAATATGAACTGAATGCTATGTGCTTGAGTCTGCACCAGTTAGCTATTCTGTTTTGCCC
CTTAGGTAAGTTCTATATAGAGCTTTCATTCAGTATAGATTTTTTTTTGGTGCAAATGTGTACTCATAATGTTCCCTACTGTGCC
GTTGCCTAAAATTTATTAATTTTGTCCAGTGCAACTAATATGTTGGTCCTCGTTCAAAAATTCGCTGATAACAAACTTGATATG
GTAGTTATACATGCAGAGAGGCTGACTGGATAATGCAGTAGCTTGAGTTAGCATTTATAAAGGAAAGGAGATAAAACAACAG
CCAACACAAATAAAAAAATTCTATGAAGTGGAAACGAGTACTGGATTTGTGGGCATGTATCCTGATGGCAAGATTTCTCAAT
GATATAAGCAGATAATGCAGCGGGCACAATATACTAATGTCACGTCCAGTTAAACTGCACCATGAATTTCTGTTTTGTTACGG
CATAAGTATTATGTAAATGTGAGATGTAATTGTTTTATTTACCCTCTTCTGTACTAATTGATTAGAGTTGCAATGCAATGCATCT
CCACCATTCTTGTTATAGTACCTTACAGTACTCTAAGCTTCTCAAAATTAGTACCCTGCCTGGCCTACAGAGTAAAGAACTAG
GACATCTTGCCATGTGGTTGGCTTTAAATGTTGTGGACTCCTTTCTAATACAATAAGCAACAAGACTAGCAGCTCCAGCTGTA
TAAACTATGATTGAATTGAACCAGTGCATTTCATGCTATGGTGATGATACCTGAGGTCACACGAATCATTGGCTTCCTTTGAGT
AAAAGTAATGGTTAGCCCCCAGAATCAGTACACTAGCAAATACTGTGAAGTGTGCTAATGCATGCACAGTGTTTGCATATTTC
CTTTCCTATGATGGCATTCTCTATGATGATAGTATGAGAACCACTCATTTCTTATCTTTTGTACTAATTGTATTTACTTTATTATGC
AGGGCATATCAAT
>SsLRR-RLK1
CCCAAGTCAGGCCATCCTCTCTTTTATTTTGTTTTGATTTGAAAAGGTCATCCTCTCTTTTAGCAGTGGCAGGCGGGCAGCTC
ACATGGCCACCTCAGCCTTCCATTAGTCTCTAGTCTCTACTATTATATTCACGATATTCTACAATAAGCATGTCAAAGTTGTCTA
AATACTCCATATTTTTGTTGTGATAATTTGTGTGTGTTGTCACATGCATCCTATTTTGTTTCTCTATGCATGTGAGACTATAAAA
AAAATAAAACAAAAAATTTAAAAAGAATATGTATCCATATAAGTAAATAATGTTTCACTTCTATCACAGTGAGTTATATTATGA
CCACATCAAATTTATTCCACTAGCATCAAGAGAAAACTATTGTATCATCAGGAGTTTCGAACACTGAAATACACAAACACAA
AAAAGATATATTTAGAACAATTGGACCAACTAAAGGTCTTTATTTTTTTTGGGAAAAAGGTAAAACCAAACAAAGACAGCAA
TTCGAAAGCTGAAAATATTGAATAACGAACAGCCAAGACAAGCGATATTTGATTAACCAAGGTAAAAAAACAAATAAATTTT
GGAAATAGAACATAAAAAAGAAAGGGAAAAAAATCTTGGCCAAGATAATTTTCTTAGATCATTATACAGTAGATCATGTGGC
AATAGCGGTTTGTATCCACGCGCTTCTTTTCATTTTGGGCACTTTTTTTTTGGCACTTATAGTTAAGGACCTTCGCGAGGAAAC
TGTCAAGGCGTCAAACTTTGCCAGAGAGACCTCTGCCATTTGATCTGTTTTTTTATCTTTGCTTGCTGAACCTCGGTCATAATT
CACAAAGGTATGACAATGGCGCGCACCCGCGAGCACGTCTATGATCATGAACTGTTAACGAATCATTGCCGACCAAAAGGC
ACAGCAACTCCAAGATGCATTGCTAAAAAGGAGCACAACAGGCGTGCTACAGTGCTCCAAATCGCCTATTTGACTTGGCTA

AAAAGAACAATTTGGTGGCCCGTCGTAGCGCACCCCGTCCCGCTCCCGCCTTGAACTCAGCCCCGTTGCCCGCACAGGCTG
CAGCACAAACACAACGGTTCGTTTCTCAGCTCAGCAACCGCCGTCCCGTCGCTCACCCACCGCAACCGGCGTGCACCAGTA
CAGGTACGGTGACCAGGACGACACGGACGCTGCACGTACGTGCGCGTGAGTGACGTAGTCGTATTCATGGGCGTGGGCGAC
ACGCGCCTCCGTAGCCTGTACGAGGACGTAGTTGTACATGCGCGGCCTTGAATGGAGTCCATTCCGTGGCCCGTAGACGCAC
GTAACGGGCAACTCGCCGTGCCGGATCCCGGCTGCCAATGCCATGCATTCCCAACCGCAGAAACAGCGTCCCGCGGCCTTG
GGAGGCAAATTGGCAAAATTCCTCGTCCCGCCCACGCGGGCACGCCCATACAGCCTCCCTCCGCGGCTCCGCCTTGATCTGA
ACTCCGATCACGCATTCACGCCGAGCCGGGCAGATCCCCAACCCGGAAAGCCACGCACTACCCTTTTCCGCACCATCATTCC
ATCTACTGCCCCCGCTGACCTGACCTCCCAAAAGCCAAAGCGTTTCGCCTAGAATTCACGTGTAGATGCCGGCAGGCGCTAC
GGCCACCCGACCTGACCTGCCCCGGGCCGCCGGGCGACGACCTACGCACATCCGGTTGGTTCGCGACAACGTCAAGGCAA
AACGTCCCACGCGGGCACGCGGCCCTCGCGCCGCGCGACACGAAAACTAGGCCAGTGCGTAGCGGGCGGCCGCTCGCTTG
GACGCTTCGCACCATAACGCACGCCACCCACCGGCCACCGCCACCTACTCGCTACTGCTCGGCGCCGTGGCTGCCAGTTAA
CGGCTGCTTAAACTACTGCTCCTCCGCCTACTAGTAGCGGCAGCGGCCGAGCTGGTCGTGCTAATGCCGCTGCGGCTGCGTG
CGCTGGCACAGCGCGGCCTGCCCCTGTCGC
>SsLRR-RLK34
ACCAGAGCCGCCACGTCCTGATCCACCGCGGCCGCCGCTAGGGTAGTCACCCCCACGGTGTCCGCCTTTGGCCACCATGTT
GACGGAGGAGGAGGAGCCGCCCTTCTTCATCTCCACGCGCTGCTCGTACGCGACGAGCTGGGCGTAGAGTTCAGCGATGGT
GATAGGCTTGACGCGCGCGGAGATGGAGGTGACGATGGAGTCGTAGTCGAGGTCGAGGCCGGTGAGGATGTACGAGACGA
GCTCCTCGTCATCTAGCTTGCGGCCCGCCGAAGCCATCTCGTCGGCGAGACTTTTCATTTTGGTGAAGTACTCGGCCACCGT
AGAGGACCCCTTTGACGTCGTGGCCAACGTCATCCTTGTTGCGATGACGCGAGCACGAGACTGCGAGGCATGCTGTCCCGC
GATGGCAGCCCAAAGTTCGGCTGCAGTCTTGGCAGTGGACACCTGCGAAAAAATTTCCTTGGAAAGTGAGGAAAACAAGT
AGCTTAGGATCTGTTGATCCTTGGCCACCCACTTGGCGTAATCCGGGTTGGGGACCGGATCGCCCGCCTTGCCAGCAGCATC
CGCTGCCGGTTCCGCCGTGATGAAGGGCGATGGTGCTTTTTCAGTGCCGGTGACGTAGCCCCAGACTTTAGCGCCTTTCAAG
GTGGAAGAGACTTGGGCATACCACATCCAGTGGTTTCCCCGGTGAAGCTTCTCGGTTACGACAGGGAAGAAGCCGAAGGA
AAACGGATTGGAAGGTTGGGCCATGGATCGGTTTTTCGAGTGGAATTGGTCTCCTGATACCATGTAAGAAAAATAAGAATAG
GAGGTAACGGGGTGACCATGCTGGTTCCCGGTTCCGTGTTTATATAATGAAGGGAAGAGCCCCTTCGTGGCTGTACACAGAG
ATCGTTTACAATGAAGACTTAATCTACAAGCTCTCCTAGCCTATGCATGTATGCATGAATCTACAAGCTCTCCTAGCCTATGCA
TGTATAACAGGGTTGATGGCGCCTATTCAACACTCCTGAATATCGCGCCATGTGTCATGCTTCTAGTACTTCTATCAAACACAA
CGGTTCGTTTCTCAGCTCAGCAACCGCAGTCCCGTGGCTCACCCACCGCAACCGGCGTGCATCAGAACAGGTACGGTGACC
AGGACGACACGGACGCTGCACGTACGTGGCGTGAGTGACGTAGCCGTATTCATGGGCGTGGGCGACACGCGCCTCCGTAGC
CTGTGCGAGGACGTAGTTGTACATGCGCGGCCTTGAATGGAGTCCATTCCGTGGCCCGTAGACGCACGTAACGGGCAACTC
GCCGTGCCGGATCCCGGCTGCCAATGCCATGCATTCCCAACCGCAGAAACAGCGTCCCGCGGCCTTGGGAGGCAAATTGGC
AAAATTCCTCGTCCCGCCCACGCGGGCACGCCCATACAGCCTCCCTCCGCCTTGATCTGAACGGAAATGCTCCGATCACGCC
GAGCCGGGCAGATCCCCAACCCGGAAAGCCACGCACTACCCTTTTCCGCACCATCATTCCATCTACTGCCCCCGCTGACCTG
ACCTCCCAAAAGCCAAAGCGTTTCGCCTAGAATTCACGTGTAGATGCCGCCCGGCGCTACGGCCACCCGACCCGACCCGAC
CTGACCTGCCCCGGGCCGCCGGGCGACGACCAACGCACATCCGGTTGGTTCGCGACAACGTCAAGGCAAAACGTCCCACG
CGGGCACGCGCGACACGAAAACTAGGCCAGTGCGTAGCGGGCGGCCGCTCGCTTGGACGCTTGGCGTGGTACCGGACGCC
CGCGGCCGCACCATAACGCACGCCACCCACCGGCCGTCGCCACCTACTCGCTAATGCTTTACTGCTCGGCGCCGTGGCTGCC
AGTTAACGGCTGCTTAAACTACTGCTCCTCCGCCTACTAGTAGCGGCAGCGGCCACCGCGGCCGAGCTGGTCGTGCTAATGC
CGCTGCGGCTGCGTGCGCTGGCACAGCGCGGCCTGCCCCTGTCGCG
>SsLRR-RLK156
TTACTGGATCTCAGAAAAACTATTTCTGTTGGAGAATAAATTGGATGTGAAGCTTTCTTGTATCCTATTGACAGTCCTGTTTTT
ATGTATTGCGTTGTCCTTAACATGTCTTTTCAGCAAGCGAGCAATACCATGAGTTGTTGGACAATGTGCTGAATGGGCCATTT
AGCATCACGCTGAGAAATCGCATCCAGTGTCATGTAATACGTGATGCAGCAAAGGATGAAATCATAACTGAGGAGCCATTCT
TAGTGTTAAACGAAGTTACAATTGACCGAGGAATATCATCTTACCTTACCAACCTGGAAGTCTACTGTGACAGTTCTTTTGTT
ACTTGTGTACAAGGAGACGGACTAATCATATCAACCACATCTGGAAGTACAGCATATTCATTAGCAGCCGGAGGATCCATGGT
TCATCCTCAGGTACGCTCCCAATTGATTTATATGGCGTTTATTTGACCAATTTCAGTCGTAACGCTTGGATTGTTACACAAATG
TTCTTCCAGTGGGTCAGCAATCAGCATTAGTTGCCCATCCTGCCATTTCGCCGTGGTTTCTAATGTCTAGCCGGCGTTTGCAG
GTCCCAGGGATCCTTTTCACCCCAATCTGTCCGCACTCCTTGTCATTCAGACCTTTGATTCTGCCTGAATACGTGACTCTCCG
CGTTCAAGTGCCGTACAACAGCAGGGGGAATGCCTGGGCATCCTTCGATGGCAAAGATCGACAGCAGCTATCGCCCGGTGA
CGCCCTGATCTGCAGCATTTCTCCTTGGCCTGTGCCCACAGCCTGCCTGGTGGACTCGACCACCGATTTCCTCCGCAGCATC
CATGAGGGTCTCCACTGGAACTTGAGGAAATCCCAATCATTTGACGGCCCGCGTGATTGATGCAACAGATTTTTCAGTTATAT
ATCAGTCCAGTGCCAGAATTCAAACTGGGAATCCTCAAAGTGCAGCTAACATCAAGAGGAATAACTGTACCTACTCGAGGG
TGCTCACCTTTACCGTTCCATTACTTATATATATATATATACACACACAAAGTTGCGTGCGTTTAGATATGAGGTATAGTGCAGA
GTCCAAATATATAGCCATGGCCATGCATGTGTGTGTACAGGTTTCTGTGCTTCAGTGCTCGATTATAGCACACGTGGCAGAAG
ATATGTACACTGATTCCGGATAAAGTATGGGGTGAGATAGATAGGTATGGATGGCAACGACTGAACATGTTGAATAATACACC
ACCAACGTATGCATGTATACATGTGATCTGCTCCCCAGATCTGAATGAAATAATCCGCTAGCCATACAAGATGAGATGCTTCCG
TTACAGCTTGCAGTCATGCGATCTGGGCACTTGTGACGGCTGATGGGGAAGCCCTCCGTCCCTTTTTCAGCTTCCATGCTGAT
AACTGAACTGATGAATGGAGTCATGGAGTGGGCATGCTCTGCTGCTCCTCCTGCTCCTGTGACGGCCAGATGGGTGCCTGCT
GCCCACTTGGAGTTGAGGGGCGAGGGCGATCGGTCACGAGTTTTGAATCCGTGATCAGGATTAACAAAAGTCGAGTAGCTT
TCGACGTGATTCTGCGGCTGCTTTTGCGTGAATTAAAGGCCGAGTATATAAAAATTAAAAACGGCCGGCCCATGATTCTTTAA
TCATTCCCTTTCCACAGCTGCGGAGTGTATGTACTCCAGTACGTAGTAGTCCAGTAGCGAGCTTGTGCGCGCGCGCGTCGTC
GCCTCAATCGCCGTGCGGTGTCGTGTTCCGGCGAGCTGGATTCCTCCGGCGACCCCACCGCCCAGCGTCACAGAGCCGGGG
GCGGGGGTGGCATGGCCATGGCCAACCCGAGGACCGGGCCGGCGGGACCGGAGAACGTGAAGTCCTTTGTTGCTTTCCTTC
TATCCAAACCTACCCCCCGCTCGCGAGTCACGGGCACTCCAGAGACGCGCGCGGCCTGCATTTTCTGTTCCTCTCCTCCCCG
CGGCTCCGATGGATGCCT
>SsLRR-RLK168
ATTGGATGTGAAGCTTTCTTGTATCCTATTGACAGTCCTGTTTTTACGTATTGCGTTGTCCTTAACATGTCTTTTCAGCAAGCG
AGCAATACCATGAGTTGTTGGACAATGTGCTGAACGGGCCATTTAGCATCACGCTGAGAAATCGCATCCAGTGTCATGTAATA
CGTGATGCAGCAAAGGATGAAATCATAACTGAGGAGCCATTCTTAGTGTTAAACGAAGTTACAATTGACCGAGGAATATCAT
CTTACCTTACCAACCTGGAAGTCTACTGTGACAGTTCTTTTGTTACTTGTGTACAAGGAGACGGACTAATCATATCAACCACA
TCTGGAAGTACAGCATATTCATTAGCAGCTGGAGGATCCATGGTTCATCCTCAGGTACGCTCCCAATTGATTTATATGGCGTTT
ATTTGACCAATTTCAGTCGTAACGCTTGGATTGTTACACAAATGTTCTTCCAGTGGGTCAGCAATCAGCATTAGTTGTCCATC
CTGCCATTTCGCCGTGGTTTCTAATGTCTAGCCGGCGTTTGCAGGTCCCAGGGATCCTTTTCACCCCAATCTGTCCGCACTCC
TTGTCATTCAGACCTTTGATTCTGCCTGAATACGTGACTCTCCGCGTTCAAGTGCCGTACAACAGCAGGGGGAATGCCTGGG
CATCCTTCGATGGCAAAGATCGACAGCAGCTATCGCCCGGTGACGCCCTGATCTGCAGCATTTCTCCTTGGCCTGTGCCCAC
AGCCTGCCTGGTGGACTCGACCACCGATTTCCTCCGCAGCATCCATGAGGGCCTCCACTGGAACTTGAGGAAATCCCAGTC
ATTTGACGGCCCGCGTGATTGATGCAACAGATTTTTCAGTTATATATCAGTCCAGTGCCAGAATTCAAGCTGGGAATCCTAAG
TGCAGCTAACATCAAGAGGAATAACTGTACCTACTCGAGGGTGCTCACCTTTACCGTTCCATTACTTATTATATATATATACAA
AGTTGCGTGCGTTTAGATATGAGGTATAGTGCAGAGTCCAAATATATAGCCATGGTCATGCATGTGTGTGTACAGGTTTCTGTG
CTTCAGTGCTCGATTATAGCACACGTGGCAGAAGATATGTACACTGATTCCGGATAAAGTATGGGGTGAGATAAATAGGTATG

GATGGCAACGACTGAACATGTTGAATAATACACCACCAACGTATGCATGTATACATGTGATCTGCTCCCCAGATCTGAATGAA
ATAATCCGCTAGCCATACAAGATGAGATGCTTCCGTTACAGCTTGCAGTCATGCGATCTGGGCACTTTGTGACGACTGATGGG
GAAGCCCTCCGTCCCTTTTTCAGCTTCCATGCTGATAACTGAACTGATGAATGGAGTCATGGAGTGGGCATGCTCTGCTGCTG
CTGCTCCTGCTCCTGTGACGGCCAGACTTGGAGTTGAGGGGCGAGGGCGATCGGTCACGCGTTTTGAATTCGTGATCAGGA
TTGACAAAAGTCGAGTAGCTTTCGACGGCTGCTTTTGCGTGAATTAAAGGCCGAGTATAGTATAGAAATTAAAAACGGCCGG
CGTATGATTCTTTAATCATTCGCTTTCCACAGCTGCAGCGCGCGTCGTCGCCTCAATCGCCGTGCGGTGTCGTGTTCCGGCGA
GCTGGATTCCTCCGGCGACCCCACCGCCCAGCGTCACAGTGGCATGGCCATGGCCAACCCGAGGACCGGAGAACGTGAAG
TCCTTTGTTTCTTTCCTTCTGTCCAAACCTACCCAGCGACTCGCGAGTCACGGGCAAGGGCAACCCAATCCAGCGACGCGCC
TGTTCCTCTCCTCCCCGCGGCTCCGATGGATGCCTCCACCATGCACACCCCCAAAACACAGCTCGGCGCCGGTACCACTACG
GCACTACCCCCTCCTCCGCGCCACTAGATCTCATCCACCGTCATCGCCACTGCCACCATATCTATCCCATCCCGGAAAATACAT
TCTGCACCCGCAGAAA
>SsLRR-RLK41
TGGAGGGAGTATTTTACTACATCGTACTAATCTATGTTATACTCCCTAAAAATATTTCCATGTTTTGATACCTCTGACTACTTATT
ACATTACATTGGTGAATCCACCAGTACGGAGTACGCCCTAACGAAATCTCTCTCTCCCTCCCTCTCTCTCCACAAACTAACAA
GAGTTTTATGCAGATCTTGTAGGACGGACGATAGCTGGCATATCAATACCCAGTGTTACGCTTGAAGTACAACGAAGGCCTT
GTTCGGCAGGCTATTTTTTGGCTTATAAGCTGGCTGTGGCTGATTTGCTGTGAAAGAAAAATACTGTTCCATGGCTGATAAGC
CGTGCTGATAAGCTCAGCCGAACAGGGGGGAAACATCAGCATATAATCCTCAGTTGCAATTTGATTGGACCTCACACTCACA
CACTTCGGTTTCAGGTTTGCCTTGCAGAATTTCAGATTCTATCTATATCTATTTGAAAGCCCCTTTTCTCTCTAATGCCCTCAAG
CATTTTTCACAGTCGTCCATCAATTTAGCGCCAAATACAAACAAACATGCGTGCGTTCCATCGTGACTTACTCCAATATCCTC
ACAAATCACAAAGAGCAGAGTACTACTACTAGTTAACTGGAATGTCTTTTGCCAAGGTAGTATCTCTTGCCGTCTAGTTTCCC
GTGGATTCTAAGATTTTAGAATTTAAACAGAGAGATTTTGATTATCTACTCTATTATCCCTCCCTTTGGGAGGCTAAAGAGAT
TTTTTTTCCACGGTTCTAAAATCGAATCAGGCCTTGTTTAGATGCCTTCAAAATTCTAGTTTTTTACTTTTCGTCTCGTCACATC
AAATCTTTAAACATATGCATGGAGCATTAAATATAGATAAAAAATAATTAGTTTGGTTGTAAATCACGATACGAATCTTTTAAA
CTAAATTAGTCCATAATTGGACACTAATTGCTAAATACAAACGAAAATGCTACAATAATTAAATTCGAAAAGTTTTTGCATTAA
ACACCCTCTCAAACAGGGCGAATGAATGAACAACTTGTTGGCGTGGCGTATCCAAACGATAGAATCTGATCGCGCGGCCATG
GATTCCTTTGCAGCACGCTGCAGCGCCCAGAACGCAGAATTCACACCACCTCCAAAGACGGGAAGAAGACTAGCAAAGCT
CCGGTGCCCGTAGGCCCTATACCAAACGCAAACCCGACGCAGCGTAGAGGAGGCAGGCGGGCACGCCTCGCCTCAAAAGC
ACCGGCGACGAGTCCAGCTGCCGGTCCTACTCGCGGACTCGCCCGGACCGGACCGGTCCAGTGCGCGCTGCGGCCTCGAA
CCCCGCGGCCCCCGCGCGAGTTTCGAGGAGGAACCCAGCGCACGCACGGCACGGGCGTCCGCCCCGTTGCCCCCCTCGGT
CCCGGTCCGTGCGCCCGGCCCATCCCGTCTGCGCCGCGCCTACCGCCCCGCCCTTTTAGCCGGAGCCACCGCCATCCTCGCG
GCCCATCCCGTCCCTCGGTCGTGCGGTGAGCCTGCTCGTCACCGTCCGATCAGCTCTGGCCCTACCCTGGAGCTGGAGCACC
TTTCTTTCCCCCTCACTCCTTGCTCTTTTTCTCTCTCGCTACTCCAGTCTCAGCCCACTCTCCGCTGCTCCCTCTCTCTCTCTCC
CTGCCTGCATTCTTCAATGGAGTTTTCCGGCGAGTCGTCTGCGGCGGCGGCAACGACGTAGACGGGTGCATCCTGGCTCGGC
AGGTACTCAACCAACTCTGCTACTACAAGGCTGTTCCTGCAGCCCATATCTGGAGCTCAATCCACTCACATTTGTGTCTTCAG
TACTCCGCAGGAAGGGAGAAGGTATACCATTAGAAGCTTTGAGGTCGTCACGCGCAGATTTAATCAAGCCACACCCCCGGA
GGCTCCGTTCCTCCCGCCGATTCGTTTTCCTGCATCGACCCTGAGCATTTATTTGCGCCGCCGCCGCCTCCTTGTGCTTCGCCT
CGTCTATCTGAGCAA
>SsLRR-RLK366
GGAGGGAGTATTTTACTACATCGTACTAATCTATGTTATACTCCCTAAAAATATTTCCATGTTTTGATACCTCTGACTACTTATTA
CATTACATTGGTGAATCCACCAGTACGGAGTACGCCCTAACGAAATCTCTCTCTCCCTCCCTCTCTCTCCACAAACTAACAAG
AGTTTTATGCAGATCTTGTAGGACGGACGATAGCTGGCATATCAATACCCAGTGTTACGCTTGAAGTACAACGAAGGCCTTGT
TCGGCAGGCTATTTTTTGGCTTATAAGCTGGCTGTGGCTGATTTGCTGTGAAAGAAAAATACTGTTCCATGGCTGATAAGCCG
TGCTGATAAGCTCAGCCGAACAGGGGGGAAACATCAGCATATAATCCTCAGTTGCAATTTGATTGGACCTCACACTCACACA
CTTCGGTTTCAGGTTTGCCTTGCAGAATTTCAGATTCTATCTATATCTATTTGAAAGCCCCTTTTCTCTCTAATGCCCTCAAGCA
TTTTTCACAGTCGTCCATCAATTTAGCGCCAAATACAAACAAACATGCGTGCGTTCCATCGTGACTTACTCCAATATCCTCAC
AAATCACAAAGAGCAGAGTACTACTACTAGTTAACTGGAATGTCTTTTGCCAAGGTAGTATCTCTTGCCGTCTAGTTTCCCGT
GGATTCTAAGATTTTAGAATTTAAACAGAGAGATTTTGATTATCTACTCTATTATCCCTCCCTTTGGGAGGCTAAAGAGATTT
TTTTTCCACGGTTCTAAAATCGAATCAGGCCTTGTTTAGATGCCTTCAAAATTCTAGTTTTTTACTTTTCGTCTCGTCACATCA
AATCTTTAAACATATGCATGGAGCATTAAATATAGATAAAAAATAATTAGTTTGGTTGTAAATCACGATACGAATCTTTTAAACT
AAATTAGTCCATAATTGGACACTAATTGCTAAATACAAACGAAAATGCTACAATAATTAAATTCGAAAAGTTTTTGCATTAAA
CACCCTCTCAAACAGGGCGAATGAATGAACAACTTGTTGGCGTGGCGTATCCAAACGATAGAATCTGATCGCGCGGCCATGG
ATTCCTTTGCAGCACGCTGCAGCGCCCAGAACGCAGAATTCACACCACCTCCAAAGACGGGAAGAAGACTAGCAAAGCTC
CGGTGCCCGTAGGCCCTATACCAAACGCAAACCCGACGCAGCGTAGAGGAGGCAGGCGGGCACGCCTCGCCTCAAAAGCA
CCGGCGACGAGTCCAGCTGCCGGTCCTACTCGCGGACTCGCCCGGACCGGACCGGTCCAGTGCGCGCTGCGGCCTCGAAC
CCCGCGGCCCCCACGCGAGTTTCGAGGAGTAACCCAGCACACGCACGGCACGGGCGTCCGCCCCGTTGCCCCCTCGGTCCC
GGTCCATGCACCCGGCCCATCCTGTCTGCGCCGCGCCTACCGCCCCGCCCTTTTAGCCGGAGCCAGCACCATCCTCGCGGCC
CATCCCGTCCCTCGGTCGTGCGGTGAGCCTGCTCGTCACCGTCCGATCAGCTCTGGCCCTACCCTGGAGCTGGAGCACCTTT
CTTTCCCCCTCACTCCTTGCTCTTTTTCTCTCTCGCTACTCCAGTCTCAGCCCACTCTCCGCTGCTCCCTCTCTCTCTCTCCCT
GCCTGCATTCTTCAATGGAGTTTTCCGGCGAGTCGTCTGCGGCGGCGGCAACGACGTAGACGGGTGCATCCTGGCTCGGCA
GGTACTCAACCAACTCTGCTACTACAAGGCTGTTCCTGCAGCCCATATCTGGAGCTCAATCCACTCACATTTGTGTCTTCAGT
ACTCCGCAGGAAGGGAGAAGGTATACCATTAGAAGCTTTGAGGTCGTCACGCGCAGATTTAATCAAGCCACACCCCCGGAG
GCTCCGTTCCTCCCGCCGATTCGTTTTCCTGCATCGACCCTGAGCATTTATTTGCGCCGCCGCCGCCTCCTTGTGCTTCGCCTC
GTCTATCTGAGCAATG
>SsLRR-RLK4
TTTTTTAAAAAATCTACTCAAAAATAATTAGAGTAACTAGACTTAGAACAAAACTAAAATGAACTATGATTTAAAACAGAGA
AAGTACTTGAATGGCTAGGACAACTTAAAAACTAGAAAAAGGGTTTGTTTTATTGCGAACACATCGGTAACAATTTGGACCA
TTCACTTTCCACCTCTCACGTTCAGTACAACATATGAAACTAGGAGAATACAGATATATAAAATACTGTATATCGGAAGATGAT
CTCTTTCTCATTTATTTTCGGAATAATTGAACTGTACAATGTTTTTTTTTCCTCTCCTATATCCTAGGATAACGTCATGTTATGGT
ATGAAGCCATGAGTATTCAGGTATATGATTATTAATAACATGTTTTACTACATCGTACTAATCTATGTTATATACTCCCTAACAAT
ATTTCCATGTTTTGATACCTCTGGCTACTTATTACATTACATTGGTGAATCCACCAGTACGGAGTACGCCCTAACGAAATCTCT
CTCTCTCTCTCTCCACAAACTAACAAGAGTTTTATGCAGATTTTGTAGGACGGACGATAGCTGGCATATCAATACCCAGTGTT
ACACTTGAAGTACAACGAAACATCAGCATATAATCCTCAGTTGCAATTTGATTGGACCTCACACTCACTTCGGTTTCAGTTTT
GCCTTGCAGAATTTCAGATTTTATCTATATCTATTTGAAAGCCCCTTTTCTCTCTAATGCCCTCAACCATTTTTCACAGTTGTCC
ATCAATTTAGCGCCAAATACAAACATGCATGCGTTCCATCGTGACTTACTCCAATATCCTCACAAATCACAAAGAGTAGTTAA
CTGGAATGTCTTTTGCCAAGGTAGTATCTCTTGCCATCTACTTTCCCGTGGATTCTAAGATTTTAGAATTTAAACAGAGAGAT
TTTGATTATCTACTCTATTATCCCTCCCTCTGGGAGGCTAAAGAGAATTTTTTCCACGGTTATGGATTCTAAAATCGATTCAAA
CAGGACCCGAATGAATGAACAACTTGTTGGCGTATCCAAACGATGGAATCTGATCGCGCGGCCATGGATTCCTTTGCAGCAC
GGCTGCAACGCCGGAACCAGAAGCCCAGAACGCAGAATTCACACCACCTCCAAAGACGGGAAGAAGACTAGCAAAGCTC
CGGTGCCTGTAGGCCCTATACCAAACGCAAACCCGACGCAGCGTAGAGGAGGCAGGCGGGCACGCCTCGCCTCAAAAGCA
CCGGCGACGAGTCCAGCTGCCGGTCCTACTCGCGGACTCGCCCGGACCGGACCAGTCCAGTGCGCGCTGCGGCCTCGAAC

CCCGCGGCCCCCACGCGAGTTTCGAGGAGGAACCCAGCGCACGCACAGCACGGGCGTCCGCCCCGTTGCCCCACTGGGTC
CCGGTCCATGCACCCGGCCCATCCCGTCTGCGCCGCGCCTACCGCCCCGCCCTTTTAGCCGGAGCCACCACCATCCTCGCGG
CCCATCCCGTCCCTCAGTCGTGCGGTGAGCCTGCTCGTCACCGTCCGATCAGCTCTGGCCCTACCCTGGAGCTGGAGCACCT
TTCTTTCCCCCCCTCACTCCTTGCTCTTTCTCTCTCTCGCTACTCCAGTCTCAGCCCACTCTCCGCTGCTCCCTCTCTCTCTCC
CTGCCTGCATTCTTCAATGGAGTTTTCCGGCGAGTCGGCGGCGGCGGCGGCAACGACGTAGACGGGTGCATCCTGGCTCGG
CAGGTACTCAACCAACTCTGCTACTACAAGGTTGTTCCTGCAGCCCATATCTGGAGCTCAATCCACTCACATTTGTGTCTTCA
GTACTCCGCAGGAAGGGAGAAGGTATACCATTAGAAGCTTTGAGGTCGTCACGTGCAGATTTAATCAAGCCACACCCCCGG
AGGCTCCGTTCCTCCCGCCGATTCGTCTTCCTGCATCGACCCTGAGCATTTATTTGCGCCGCCGCCGCCTCCTTGTGCTTCGC
CTCGTCTAGCTGAGCAA
>SsLRR-RLK45
TAGAGTAATCCCCAGAACTCAATAGTTCTCTTCATAACTATGTACCTCTGATTCGCAATAAATAATAAATAAATAGACTATTTGT
GACACTCTGGTGCCTAGTCATGATCTTGATACTGAACTTGCAATGGGGGATGCTCTAATCTCCAGTTGAGCATTGGCATGCGG
ATGCTCTAATCTCCTTTAGGAAGCAAGTGCAATGCTGTTATGCTCAATAGATATATAAAAGGCATTTGTGGTTCAGCTACAAAG
ATGTCATAATGTTTTTTAGTGGAAAAAAACGTTTTAGAAATATGAGTCTAGAAGGAAAAATGGANNNNNNNNNNNNNN
NNNNNNNNNNNNNNNNNNNNNNNNNNNNNNNNNNNNNNNNNNNNNNNNNNNNNNNNNNNNNNNNNNNN
NNNNNNNACAATATTTCCATGTTTTGATACCTCTGGCTACTTATTACATTACATTGGTGAATCCACCAGTACGGAGTACGCCCT
AACGAAATCTCTCTCTCTCTCTCTCCACAAACTAACAAGAGTTTTATGCAGATTTTGTAGGACGGACGATAGCTGGCATATCA
ATACCCAGTGTTACACTTGAAGTACAACGAAACATCAGCATATAATCCTCAGTTGCAATTTGATTGGACCTCACACTCACTTC
GGTTTCAGTTTTGCCTTGCAGAATTTCAGATTTTATCTATATCTATTTGAAAGCCCCTTTTCTCTCTAATGCCCTCAACCATTTT
TCACAGTTGTCCATCAATTTAGCGCCAAATACAAACATGCATGCGTTCCATCGTGACTTACTCCAATATCCTCACAAATCACA
AAGAGTAGTTAACTGGAATGTCTTTTGCCAAGGTAGTATCTCTTGCCATCTACTTTCCCGTGGATTCTAAGATTTTTAGAATTT
AAACAGAGAGATTTTGATTATCTACTCTATTATCCCTCCCTCTGGGGGCTAAAGAGAATTTTTTCCACGGTTATGGATTCTAAA
ATCGATTCAAACAGGACCCGAATGAATGAACAACTTGTTGGCGTATCCAAACGATGGAATCTGATCGCGCGGCCATGGATTC
CTTTGCAGCACGGCTGCAACGCCGGAACCAGAAGCCCAGAACGCAGAATTCACACCACCTCCAAAGACGGGAAGAAGACT
AGCAAAGCTCCGGTGCCTGTAGGCCCTATACCAAACGCAAACCCGACGCAGCGTAGAGGAGGCAGGCGGGCACGCCTCGC
CTCAAAAGCACCGGCGACGAGTCCAGCTGCCGGTCCTACTCGCGGACTCGCCCGGACCGGACCAGTCCAGTGCGCGCTGC
GGCCTCGAACCCCGCGGCCCCCACGCGAGTTTCGAGGAGTAACCCAGCACACGCACGGCACGGGCGTCCGCCCCGTTGCC
CCCTCGGTCCCGGTCCATGCACCCGGCCCATCCTGTCTGCGCCGCGCCTACCGCCCCGCCCTTTTAGCCGGAGCCAGCACCA
TCCTCGCGGCCCATCCCGTCCCTCGGTCGTGCGGTGAGCCTGCTCGTCACCGTCCGATCAGCTCTGGCCCTACCCTGGAGCT
GGAGCACCTTTCTTTCCCCCCTCACTCCTTTTTCTCTTCTTTTTCTCTCTCGCTACTCCAGTCTCAGCCCACTCTCCGCTGCTC
CCTCCCTCTCTCCCTGCCTGCATTCTTCAATGGAGTTTTCCGGCGAGTCGGCGGCGGCGGCGGCAACGACGTAGACGGGTGC
ATCCTGGCTCGGCAGGTACTCAACCAACTCTGCTACTACAAGGCTGTTCCTGCAGCCCATATCTGGAGCTCAATCCACTCAC
ATTTGTGTCTTCAGTACTCCGCAGGAAGGGAGAAGGTATACCATTAGAAGCTTTGAGGTCGTCACGCGCAGATTTAATCCAG
CCACACCCCCGGAGGCTCCGTTCCTCCCGCCGATTCGTCTTCCTGCATCGACCCTGAGCATTTATTTGCGCCGCCGCCGCCTC
CTTGTGCTTCGCCTCGTCTAGCTGAG
>SsLRR-RLK55
TCTCTAATGCCCTCGACCATTTTTCACAGTTGTCCATCAATTTAGCGCCAAATACAAACATGCATGCGTTCCATCGTGACTTAC
TCCAATATCCTCACAAATCACAAAGAGTAGTTAACTGGAATGTCTTTTGCCAAGGTAGTATCTCTTGCCATCTACTTTCCCGTG
GATTCTAAGATTTTTAGAATTTAAACAGAGAGATTTTGATTATCTACTATAGATCCCTCCCTTTGGGGAGGCTAAAGAGATTTT
TTTTCCACGGTTATGGATTCTAAAATCGATTCAAACAGGGCCCGAATGAATGAACAACTTGTTGGCGTATCCAAACGATAGA
ATCTGATCGCGCGGCCATGGATTCCTTTGCAGCACGGCTGCAACGCCAGAACCAGAAGCCCAGAACGCAGAATTCACACCA
CCGCCTTGTTTAGGGCCTGTTTAGTTGCACCCTAAAATACCAATTTTTTTAAGATTTCTCGTCACATCAAATCTTTGGACGCAT
GCATGAAGTATTAAATATAGATAAAAAATAAAACTAATTACACAGTTTAGACGAAATGGATGAGACGAATCTTTTAAGTCTAA
TTAGACCATGATTTGACAATATTTACCAAATAACAACGAAAACGCTACAGTAGCGTTTTGCCAAAAATTTCGGCATCCAAACG
GTGCCTTAGATACCAAGCCCTTGTTTAGTTCCCAACTTCTTCCCTAAATTTCACTATGCAAAAAGAAGATTCCCCGTCACATC
GAAATATCGGCATATGCATAAAGTACTCAACGTAGATAAAATAAAAAACTAATTGCACAGTTATGGAGTACTTTGCGAGACGA
ACATTTTGAGACTAATTAATCAATGTTTGGACAATTATTACCAAATAAAAACAAAATGCTACAGTACCAAACAGGGTTGCTAC
AGTAAGTCAGATGCCCCCGAATTCAGTATCTAAACCAGGCCCAAATTCACTCCAAATTCCACTATGCAAAAAGAAGATTCCC
CATCACATCAAAATGTCGGCACATATATATAGAGTACTAAATGTAGATGAAATCAAAAACTAATTGCACAGTTTTGTTCTACTT
TGCGAGACGAATATTTTAAACCTAATTAATCAATGTTTGGACAATTATTATCAAATAAAAATAAAATTACTATAATATGACAGGA
CACTTGGTATGCAAACTTTGTCTCCAAAGTTTAGAATCTAAACAAGGCCCACCTCTAAAGACGGGAAGAAGACTAGCAAAG
CCACGCCCGCCTCAAAAACACCGACGACGAGTCCAGCTGCCGGTCCTACTCGCGGACTCGCCCGAACCGGACCGGTCCAG
TGCGCGCTGCGGCCTCGAACCCCGCGGCCCCCACGCGAGTTTCGAGGAGGAACCCAGCGCACGCCCCGTTGCCCCCCTCG
GTCCCGGTCCATGCACCCGGCCCATCCCGTCTGCGCCGCGCCTACCGCCCCGCCCTTTTAGCCGGAGCCACCACCATCCTCG
CGGCCCATCCCGTCCCTCAGTCGTGCGGTGAGCCTGCTCGTCACCGTCCAATCAGCTCTGGCCCTACCCTGGAGCTGGAGCA
CCTTTCTTTCCCCCCTCACTCCTTGCTCTTTTTCTCTCTCGCTACTCCAGTCTCAGCCCACTCTCCGCTGCTCCCTCTATCTCTC
CCTGCCTGCATTCTTCAATGGAGTTTTCCGGCGAGTCGGCGGCGGCGGCGGCAACGACGTAGACGGGTGCATCCTGGCTCG
GCAGGTACTCAACCAACTCTGCTACTACAAGGCTGTTCCTGCAGCCCATATCTGGAGCTCAATCCACTCACATTTGTGTCTTC
AGTACTCCGCAGGAAGGGAGAAGGTATACCATTAGAAGCTTTGAGGTCGTCAAGCGCAGATTTAATCAAGCCACACCCCCG
GAGGCTCCGTTCCTCCCGCCGATTCGTCTTCCTGCATCGACCCTGAGCATTTATTTGCGCCGCCGCCGCCTCCTTGTGCTTCG
CCTCGTCTAGCTGAGCA
>SsLRR-RLK355
AGCTGGAGGCTAACTTCACATTTTTTCTAGAGCACCTCAAGAGAGAGAGGTATTCCAGTTTTTTGAGTTTTATACTCCCACAT
GGAGTTGTGCGGTAATTACCCACCTTGCCACTCATTAATCACCAATTACTTTTCCTTGTTCGCTAACGGACCATTTGCAATTTC
AATTAGGGGCATAATGGACCATTTGCATGTAAACAACTTATTCCTGGAACTAGAGCTAGACTATGTGCCAAACACGTACCACT
TGCTCCACAAACTCTATGGTTGAACTACTTTATGGTGGAATTTGCGGAGCGGAGTTGCAAAACCAGGAGTAGAGCAGCAGT
CCCGCACCACACGAACTCAATTAACAATGGTACCATGATCTCACCCTCCACATCCTCTTCTACCGTCACGGTATTATGGGACC
CCTGCCTTCTAGTCACGTTAGTCTTGGTTGTACATAGTATGTTACTAGACAAAAATGATTTTAAATGCTAAAATCTTCTGCAT
ATTAAAAAAATGAATGACGTATGTGTGCTTGCTTAAAATGTGAAATCACGAGTATACTACTAGCACACGAACTCAATTAATAA
ACTACCACACCATGATCACCCTCCACCTCCTCCTCTATCGTCGTGCCAATGTGGATCCCTCCTGCCTTCTCCTAGTTATGTTAG
CCTTCTTTTCTAAAAAAAAGTCATGTTAGCCTTGGATGTAGTAGATAATAGTATGTTGCCAGATAAAATTGATTTTAATAAATG
CTAAAAGCTTCTACATATTTAAAAAAAAATAAAATGCTATATCTATGCTACTTAGAATATGAAATCTCGAGTATATTACTACCAGC
ACATGAACTCAACCAATGAACAACCTTACTGTTGTTCTCACCCTCATCGTGGCAATGGCCATGGTGGAACACCCCCACTACC
TTTCCCTAGTCACACTAGCCTTGGTTGTAGATAATATACGGGTAGACAAAATTGAATTTTAAGTGCTATCGGCGTGTTTGTTTC
ACGCCGAGCCATGCCTCGCCTAAGCCGCGGCTGCGCCAAAATGCAGCAAATAAAACGGCCACCACACCTGCGGCGTGAGTT
GGCGATGGAAATGAACTACGCGCGTGTGGTGTGAGTGCGGCGTGCCAAAACCGCTGCGGCAACCAAACAACGATGTGAGA
CGCCGCATTTTTGGCGTGACGGTACGTGCGCAAGTAGGTGTGAACCAATCACGCTCTAAAATCTTCTGCATATTAAAAAAGT
ATAATATATCTATGCTCACTTAAAATATGAAATCTCGAGTGTGACGATGGACCAATGCCAAAGGAAGAAAACATTAATTGCAC
CGGTTTTTCATCTCTCACCAAACCAACGAGTCTAATAACAAAGACAAGACACATCATATTAGACCACTATGGTTAATGGTCAG
CACCTCTCAAGATTTCGCTGCTCTACTCTTGCTCCTCTTGCGTTGTGGTAACACTGCCAACCCCTCCCAATCTCTAAAAAATT

TCGACTCCCGCACGAAAGAAATGAGGTACAGGTACTCCCTAGGTTGATGTTGGCAAGCAAATTTGCTCCATAGTCTTTGAAA
TTAAACGCATGCACACAAAAAAAAACCTCATGAATGAATCACCGTCACCGCCCGGTGGACGAAGACGTGGCATGGTCGCCCA
CTCCGCCAGTCACGACGAGTCGACGACAGCCTTCGACCATCCGTCACCCACGGGCCACGAGCCACGACCCCACAGAAGAT
TTGATTTTGAAATGCTAAAATTGTGGAACAACCTCGAGCCAGACGATGGCCCCACCGCGCAGTGGCCAGCGGAGCATTTAAT
TGCAAGCGGTTTCCCACTCCCCTCTCCTGACGAAACCAGCAAGCCGGCCGCAAGGACAGCAGTCAGCGACAACAGTGCAT
CTGCGCCGCGAACCGCCGCTCCAGTCCAATCAATGAACGAACACTTCCTATGATCTCGTCGCCCACCACCTCCTCCTCCTCTT
CCGCCGCCGCCGCGGCAATG
>SsLRR-RLK362
ATTGCCAAACACCCTCTGCTACAAGAACCCCGCTCCACGAAAAAGGTGGAGTTGGAGGCTAACTTCACATTTTTTTCTGGA
GCACCTCAAGAGAGAGGTATTCTAGTTTTTTGAGTTTTATACTCCCACATGGAGTTGTGTGGTAATTACCCACCTTGCCACTC
ATTAATCACCGATTACTTTTCCCTGTTCGCTAATGGACCATTTGCAATTTCAATTAGGGGCATAATGGACTATTTGCATGTAAAC
AACTTATTCCTGGAGTTGGAGCTAGGCTATGTGCCAAACACGTACCACTTGCTCCACAAACTCTATGGTTGAACTACTTTATG
GTGGAATTTGCGGAGCGGAGTTGCAAAACCAGGAGTAGAGCAGCAGTCCCGCACCGCACGAACTCAATTAATAATGGTACC
ATGATCTCACCCTCCACATCCTCTTCTACCGTCACGGTATTATGGGACCCCTGCCTTCTAGTCACGTTAGTCTTGGTTGTACAT
AGTATGTTACTAGACAAAAATGATTTTTAAATGCTAAAATCTTCTGCATATTAAAAAAATGAATGACGTATGTGTGCTTGCTTA
AAATGTGAAATCACGAGTATACTACTAGCACACGAACTCAATTAATAAACTACCACACCATGATCACCCTCCACCTCCTCCTC
TATCGTCGTGCCAATGTGGATCCCTCCTGCCTTCTCCTAGTTATGTTAGCCTTCTTTTCTAAAAAAAAAGTCATGTTAGCCTTGG
ATGTAGTAGATAATAGTATGTTGCCAGATAAAATTGATTTCTAATAAATGCTAAAAGCTTCTACATATTTAAAAAAAAATAAAAT
GCTATATCTATGCTTACTTAGAATATGAAACCTCGAGTATATTACTACCAGCACATGAACTCAACCAATGAACAACCTTACTGT
TGTTCTCACCCTCATCGTGGCAATGGCCATGGTGGAACACCCCCCCTCTACCTTTCCCTAGTCACACTGGCCTTGGTTGTAGA
TACTATATGGGTAGACAAAATTGAATTTTAAGTGCTATCGGCGTGTTTATTTCACGCTGAGCCACGCCTCGCCTAAGCCGCGG
CTGCGCCAAAATGCAGCAAATAAAACGGCCACCACACCTACGGCGTGAGTTGGCGATGAAAATGAACTACGCGTGTGTGGT
GCGAGTGCGGCGTGCCAAAACCGCGGCGGCAACCAAACAACGATGCGAGACGCCGCAGTTTTGGCGTGGCGGTATGTGCG
TAAGTAGGCGTGAACCAATCACGCTCTAAAATCTTCTGCATATTAAAAAAGTATAATATATCTACACTCACTTAAAATATGAAA
TCTCGAGTGTGACGATGGACCAATGCCAAAGGAAGAAAACATTAATTGCACCGATTTTTCATCTCTCACCAAACCAACAAGC
CTAATAACAAAGACAAGACACATCATATTAGACCACTATGGTTAATGGTCAAGCACCTCTCAAGATTTCGCTGCTCTACTCTT
GCTCCTCTTGCGTTGTGGTAACACTGCCAACCCCTCCCAATCTCTAAAAAATTTCGACTCCCGCACGAAAGAAATGAGGTAC
AGGTACTCCCTAGGTTGATGTTGGCAAGCAAATTTGCTCCATAGTCTTTGAAATTAAACGCATGCACACAAAAAAAAACCTCA
TGAATGAATCACCGTCACCGCCCGGTGGACGAAGACGTGGCATGGTCGCCCACTCCGCCAGTCACGACGAGTCGACGACA
GCCTTCGACCATCCGTCACCCACGGGCCACGAGCCACGACCCCACAGAAGATTTGATTTTGAAATGCTAAAATTGTGGAAC
AACCTCGAGCCAGACGATGGCCCCACCGCGCAGTGGCCAGCGGAGCATTTAATTGCAAGCGGTTTCCCACTCCCCTCTCCT
GACGAAACCAGCAAGCCGGCCGCAAGGACAGCAGTCAGCGACAACAGTGCATCTGCGCCGCGAACCGCCGCTCCAGTCCA
ATCAATGAACGAACACTTCCTA
>SsLRR-RLK377
TCAATCAGGGGCATAATGGACCATTTGCATGTAAACAACCTATTCCTGGAGCTAGGCTACCTGCCAAACACGTACCATCTGCT
CCACAAACTCCATGATAGAGGCTACTTTATGGTGGAATTCGCGGAGCGGAGCTGTAAAAACATGAGTAGAGCAGCAGTCCC
GCACCACACAAACTCAATTAACAATGGTACCATGATCTCACCCTCCACGTCCTCTTCTACCGTCACGGTATTATGGGACCCCT
GCCTTCTAGAGTTCTAGTCACGTTAGTCTTGGTCGTACATAGTATGTTACTAGACAAAAATGATTTTTAAATGCTAAAATCTTT
TGCATATTAAAAAAATGAATGACATATGTGTGCTTGCTTAAAATGTGAAATCACGAGTATACTACTAGCACACGAACTCAATT
AATAAACTACCACACCATGATCACCCTCCACCTCCTCTATCGTCGTGCCAATGTGGATCCCTCCTGCCTTCTCCTAGTTATGTT
AGCCTTCTTTTCTAAAAAAAAAGTCATGTTAGCCTTGGATGTAGTAGATAGTATGTTGCCAGATAAAATTGATTTTTAATAAATG
CTAAAAGCTTCTACATATTTAAAAAAAAATAAAATGCTATATCTATGCCTACTTAGAATATGAAATCTCGAGTGTATTACTACCAG
CACATGAACTCAACCAATGAACAACCTTACTGTTCTCACCCTCATCGTGGCAATGGGCATGGTGGAACCACCCCCCTCACCC
CTCTACCTTTCCCTAGTCACACTAGCCTTGGTTGTAGATAATATACGGGTAGACAAAATTGAATTTTAAGTGCTATCGGCGTGT
TTGTTTCACGCCGAGCCACGCCTCGCCTAAGCCGCGGCTGTGCCAAAATGCAGCGAATAAAACGGCCACCACACCTGCGGC
GTGAGTAGACGATGAAAATGAACTACGCGTGTGTGGTGTGAGTGCGGCGTGTCAAAACCACGGTGGCGACCAAACAACGA
TGCGAGACGCCGCAGTTTTGGCGTGGCGGTATGTGCGTAAGTAGGCGTGAACCAATCACGCTCTAAAATCTTCTGCATATTA
AAAAAGTACTATAATATATCTACGCTCGCTTAAAATATAAAATCTCGAGTGTGACGATGGACCAATGCCAAAGGAAGAAAAC
ATTAATTGCACCGGTTTTTCATCTCTCACCAAACCAACGAGCCTAAAGCCAGTCTCAATGAGGTTTCATTAGAGTTTCATAGG
CATTAATTATGCTGATGTGGCACCATATTAATAAAGAGAGAGATGATAAGAGTTTCATGGGAGCAGAGAGAGTTTCATGGGGA
TGAAACTCTTCTGCACTGTTTCTAAAATCTGGATGTGTTGGAAACAGTGACATGAAATTGCCACTGAAACTGACGTAATAAC
AAAGACAAGACACATCATATTAGACCACTATGGTTAATGGTCAAGCACCTCTCGAGATTTCGCTGCTCTACTCTTGTTCCTCT
TATGGTAACAATGCCGACCCCTCCCGATCTCTAAAAAATTTCGACTCCCGCACGAAAGAAATGAGGTACAGGTACTCCCTAG
GTTAGGTTGATGTTGGCAAGCAAATTTGCTCCATAGTCTTTGAAATTAAACGCATGCACACAAAAAAAAACCTCATGAATGAA
TCACCGTCACCGCCCGGTGGACGAAGACGTGGCATGGTCGCCCACTCCGCCAGTCACGACGAGTCGACGACAGCCTTCGA
CCATCCGTCACCCACGGGCCACGAGCCACGACCCCACGACCCCACAGAAGATTTGATTTTGAAATGCTAAAATTGTGGAAC
AACCTCGAGCCAGACGATGGCCCCACCGCGCAGTGAACCAGCAAGCCGGCAGCAAGGACAGCAGTCAGCAGACAACAGT
GCATCTGCGCCGCGAACCGCCGCTCCAGTCCAATCAATGAACGAACACTTCCTATATGATCTCGTCGCCCACCACCTCCTCCT
CCTCTTCCGCCGCCGCCGCGGCAA
>SsLRR-RLK144
GCTTCTGCGCGTTGGACACAAACGCTGGTTTCCTCCCGTCGTCGTAACCTGACCTCGCACGTCTGCACATGAGCTCGCATCG
CCGCCTGCGGCTCCTCCGCCCGCACGCAAACTCGCTTCGGCGTTGGTCGCGCTTCCATGTGGGTGTACGCGCGAGCATGCCT
CGCCGCCGGCCGGGCCTCCGCATGGGCGTACGCACGCGAGGACCACGCCTCACCGGCGGCTGCGCCTCCAAGCAGAAGCA
CGCGAGCGCACAGCGCTCGCCTCGTTTCTGGCCACGCTTCCGTCGGCATCCGCACGAGCACGCCTCGCCGCCGGCAGTGCC
ACTGCGTGCACGTGCGTGCGAGGAGACTTCGCTGCCAGCCTCACCTCCGTGCATGCAAACACGCCCACATCCAGTTGATTC
CTCGCTGCACTGTACCATCTATGGTCACTGACTGGATAAGGATGGCTGGAGATAGACAACGAGATATGGATTAGAAGGAGAT
TTTCGCTGCACTGTACTGTACCATCCTCGCTGCACTGTACCATCTAGATTAGGGTGCTAGATCAGTTGGTCTAGACACTCCGG
CGGCACCCACCTAGTCCTGAGTTCAACTTCTCGTGAGAACGAATTTCAGGCTGAAAGTAAAAAAAAAAAATACCCTCGTCTG
TCCCAATAACCAAAGCACTGGGGGCCTGGCCCAATTCTCACTTGGGCGACGGTAAAGCCACCGTGTAAGGGTGGGGGCGAG
GGTTCGGAGATTTTTTCGGCCGGGTGAGAAGACCATCTCCTTCTTATTTGGAAATGTTGCGGCGGTAGTCTTAACTGCCCCGA
TCGAGTTTTTTTTACTGTACTTTCTCCGTTCTAAATTATAAGTTGTTTTGTTTTTTTCTAATTGCATATCTTTTATTGTATAGTCTA
ACCTGACCTATATTTAGGTGCATAGTGTACCAATATAACTAAAACAATTAGATCAACTTAGAATTTAGAACAGAGGGACTAATA
AGCTAGACTATTTTGATCTCTAGATTATATAGGTGGATTATTATTAATTGGAGTACAAATGATCTATAATCTTGAAAAAGCATATT
AATTGGAGTACAAATGATCTATAATCTTGAAAAATCACATTTGAGATATTGTAGGACTACAATAATCTAATGGTTGAATTGTTAT
AATCCAACCTATGATTTGAGGTGTTTGGATTACCTTTAGATTGTTTTTTTCTGGTCACTATAATCTAGTACTAGTTGGTATCAAC
ACTCTAGGACATGTTTGATTTAGTTTGTACTTCATCTGTTATAAATTATAAGACATTTTGACTTTTTAGGTGCATTGTTTTTTATT
ATGTATCTAGATATAATGTATATCATGTACATAGCGAAAGCTATAAATCTAAAAAAAAATCAAAATGTCTTTTAATTTGAAAATTG
AAACGGAGGGAATGCTTTGTAAGAACGTGCGATCAGTAATCCATAGTCTAGGCGGGAACTGAAATAGTTAAACCGTCGAAAT
ACACGGCGCATGTACAGCACAAGACACTAGCTCCTGATGCTCCTTTTTTTTTCTTTTGCTTTTCCCCCAAGGGAATTTCCAAT
TGAAGGAAATAATAAAACAATGTATCTTCAGGATTTAAGTAGGTATTTATAACTGCAGAAACGCGAAGATATTAAATGTTCATC

AGAAAAATCATATCCATTCAGGCAAAAGCTCATGGAAAATGATAAAACAGATCATTTTGTCCATTCTTACTTGTTCTACTCGT
AGCGCATTAATAGTGCAAGGGGCCGCCTCCTCTCCTACTCGCTTCTCCCCTCTCCCCACTCGTGTCTGGCTGACTGCTCCACG
CCGCGCGCGCGACCACGACCGCGCGGCCACGCCCCACGCCCACCACCACCGCGCTGCACTGCGCCACTACCCCTCTTCCCC
GCACTGCCCCAGAACTGCTCCGCGCAAATGGACAGAGCACCGCTGTGATCCCTCCACCGTCCTCATCCTCCTACTGCCCGCC
GTGTCGCG
>SsLRR-RLK185
AAAGTTTTCTTTGTGTCACTCTCTCATCCAATTTATGTTTCTGTGCTCTATTCAAATCTTATAGTTTTCCTTGTTTTATGTTTTCT
CAGTCCATAGAATTTAAGATGTTAGGGTTATTTCTACGTTTTTATAATCCTGGGTTCTAAAGAGTCCCTTAGGCGTAGTACAGT
TAATATAAAAATAGGTAGATATAGGTCCACTTTGGTAAGAGAAATTGAAGGCAAGTAAATTTTTGGTTAAGAAAATTTTTGTT
GAACCGTAACAAGCACATGGCTTGTACAATTTGTGTTGTTACAATCCAGGCCTCTTTCTGGATTATGGATAAAATTACAATAAT
CTAGATTAGGGTGCTAGATCAGTTGGTCTAGACACTCCGACGGCACCCTCCTAGTCCTGAGTTCAACTTCTCGTGAGAACGA
ATTTCAGGCTGAAAGTAAAAGATACCCTCATCCGTCCCCATAACCAAAGCACTGGGGGGCCCTGACCCAATTCTCACTTGGG
CGACGGTAAAGCCACCGTGTAAGGGTGGGGGGCGAGGGTTCGGAGATTTTCTCGGCCGGGTGAGAAGGCCATCTCCTTCTT
ATTTGGAAATGTTGCGGCGGTAGTCTTAACCGCCCCGATCGAGTTTTTTTAATTGTACTTTTTCCGTTCTAAATTCTAAATTG
TTTTGTTTTTTTCTAATTGCATATCTTTTATTGTATAGTCTAACCTGACCTATATTTAGGTGCATAGTGTACCAATATAACTAAAA
CAATTAGATCAACTTAGAATTTAGAACAGAGGGAGTAATAAGCTAGACTATTTCGATCTCTAGATTATATAGGTGGATTATTATT
AATTGGAGTACAAATGATCTATAATCTTGAAAACGCATATTAATTAGAGTACAAACGATCTATAATCTTGAAAAAGCATATTTG
AGATATTGTAGGACTACAATAATCTAATGGTTGAATTGTTATAATCCAACCTATGATTTGAGGTGTTTGGATTACCTTTAGATTG
TTTTTTTCTGGTCACTATAATCTAGTACTAGTTGGTATCAACACTCTAGGACATGTTTGATTTAGTTTGTACTTCATCTGTTATA
AATTATAAGGCATTTTAACTTTATAGGTGCATTGTTTTTTTATTATGTATCTAAATATAATGTATACCATGTACATAGCGAAAGCT
ATAAATCTAAAAAAATTAAAACGTCTTTTAATTTGAAAATTGAAACGGAGGGAATGCTTTGTAAGAACGTGCGATCAGTAAT
CCATAGTCTAAGCGGGAACTGAAATAGCTAAACCGTCGAAATACACGGCGCATGTACAGCACAAGACACTAGCTCCTGATGT
TCCTTTTTTTTCTTTTGCTTTTCCCCCAAGGGAATTTCCAATTGAAGGAAATAATAAAACAATGTATCTTCAGGATTTAAGTAG
GTATTTATAACTGCAGAAACGCGAAGATATTAAATGTTCATCAGAAAAATCATATCCATTCAGGCAAATGAGAAAACAGATCA
TTTTGTCCATTCCAAGGCCTTGTTTACTTTGCAAAAAATTGAACCTGATGAATAGTACCGCTTTCGTCTTATTTAGCAAATATT
GTCCAATCATAGACCAACTAGGCTCAAAAGATTCATCTCGTGATTTCCAACTAAACTGTGTAATTAGTTATTTATTTTATCTAC
ATTTAATACTCCATGCAAGCAGCTAAAAATTGATGTGATGGAGAGAGAGTGAAAAAACTTTTGGATGGCATCTAAACAAGCC
CTTGTTCTACTCGTAGCACATTAATAGCGCAAGGGGCCGCCTCCTCTCCTACTCGCTTCTCCCCTCACCCCACTGGTGTCTGG
CTGACTGCTCCACGCCGCGCGCGCGACCACGACCACCGCGCTGCACTGCGCCACTACCCCTCTTCCCCGCACTGCCCCAGA
ACTGCTCCGCGCAAATGGACAGAGCACCGCTGTGATCCCTCCACCGTCCTCATCCTCATACTGCCCGCCGTGTCGCGA
>SsLRR-RLK244
GTGTAGCCTTTATCACATAGGAGTAAGTAGCTTAGTGACTTAGTCTTGTTGGTGTGTATTAGAGATCATAGCCTTGCTTCACTA
GAATTATATAGATAGCTTGTATAGTGTAGTTGAGCTAGTGCTAGAATTAGCTTAGCCATCTTTTATACTAACGTAGTTGTTTAGT
GAAGCTTATAGATTTTTAAATAGGCTATTCACCCCCCTCTAGCCATTTGGACCTTTCAAGCTCACACCAACAACCTAAGTTACT
AGGCTAATTACTCCTATGAGAAAAACTATTCTACTCAAGCTAAACAAGGCAAAGAACAATAACTAAAGAAGAGAGCTTGAAT
AGATATACCTACGTTAATGAGAAGAGATATGTCCAATATACCAATCAATCAATGTGATCACATACACAAATGAAATCCTCGGCA
ATAAGATGACATAAGATTTTTTATCGAGGTTCACTTGCTTCCCGGCAAGTTACTCCTCGTTGTGGCGATACACCCACTTGATG
GATCACGAGCTAATCAACTATCAAGAGTTAAATGCTCAACGGGTTCCGCACATCCACTCACAAGATGACCCCACGCGACATC
TGCAAACCCACCTAACCCCACACGATGTCTGTGCCCCCGTCCGACCCCACGCGGCGTTTGCACCCCACACGACCCGTCGTG
CCCTCTAGCAGCTGCAGCATGTTGCAACATGCACGTGCAAAATATTCGATCTACTTTTGAAACAGCCAGATGCAACATTTAAA
ACATATCTCTTAAGGCAGATGAAATACTTGAAGCATGCGTATGAAGCACTCACAAAAACACCTAGAAGACACTTGAAAATCA
TTGCAAAACATACACAACATTCAGATAAAACACATGCAACATATGTGTGAAACATATGCAACATACAGATTACATACTTACAA
CATGCATAAAAAAACAGATGAAACATTGGAAACAGACGTTTGCAACATGGGTGTACACCCATTGCAACATATGTAATATCCC
GATCTACTTTGCAACATCCATACGAAAACACTTACAACGTACCTCTGAATCATCTGAAATACTTGAAACATACGTTTGCAACA
TGCGCTTGCAACATCTGAGGAAGGGAGCGCGAGGTGCAGCCAGAGAAGCTCGATTCAGGTCTGGCCACTCCGGATCTCGTG
ATGTCGCGAGCTAAGGTCAAGCGTCGCGGCGGGGTCGACGTCGCAAGGGCCATGACCTCTTGGTGGGGAACGGCGGCGGC
AGCAGCACCTCGACCCGGCGAGGGATGCGACACCGTGCACAGGGGCAGGGACCTCGCACAACACGACAAGCCGGAGCGG
GCACGGCGGGGATGGAGCACGGCGCGGCTCGACACAGGATGGGGGAGCCGCCGCGAGGCGGCTTGGGACAGACGGATGG
CACGGTGAGCTGCGACGAGCCAAGTAAGGAGTTGAGGATTTCTATCGGCTGTCGTAGTGCAGATAAAGTAGACAGAGAAGA
GAACGTAGATGGGTCTATTGGGCCCAGTCGTTGTGCTGGCTCGAGAACCGAAATAGGTCGTCAGAACGGATGGACGCACCA
GAGTATTAGTGAAACAATCACCGCAAAAGCACATGAATTTCTTGCTGAATACTGGCCTGGTCTATAAACCGAGTCCGATAATC
GGAGGTCACCTTTCCGTTCTTCCGTCTGGAACATGTACACAGCCCAAAGGCAAACGGAGAATTACCGAACAGAGCACTCCG
GCCTCAAACCGAGTCCGATGATCCGAACGTCCGAGACCACCGAGTCCGATGATCCGAACGTCCGAGACCACCGAGTCCGAG
TCCGAGTCCGAGTCCGAGTCCGATTCGTAAAGAAGCTACTCCAGCCAGGCTAATAAAAGGAGGCGGCGCCCTTGGCTTCTC
TCCACCAAACCGAAGGCACCGTTCGCCAAGCCTTCAGCAAACGAACGCAAGGTCGAAGCCACCAAGCCCACGACACGAA
CACTGTCTGCGGCTGCGCCCTCGAAAACCGCAGAAA
>SsLRR-RLK108
TATCTCTGATGGCAACAGGGATATCGATGATCTTCCCCTGATATTGGCTGACCATTAAGTCAGATGACTGCAGCATATCTATGA
CTGAGCAGAACTTTGCCTGCATTCTCTGGTAGCCTTGTTCTTCCCAGTTCTCTTTTGCAAAATTCCCAAACTCTGAACATTCT
AACCCTCTGTTAAGATATTTTTCAGATAGTCTGACTATCTACCCTCTAAAAACTATCTAGGCTGTATTTTTTTTGCTTTCCAACT
TGTTGGTTGCATGCAAATATCACATGGGTGTTATTCCTTTCAGAAATGACATGATAGACTACCCTGTGAAAAATAGGATAGCT
AACAGCCTAACACCAAGGACCAAATCTGGCATTCCTCTAGTTTTGGTCCCTATCCCTAGAGGGGAATGCTGACTGATGGCCT
GTGAACTTTTGAGGATGCAGTAATGATGTTTGGTTCAGATAACTAGGCTAAACTTTAGGCAACGTCGCTAGCCACTAGTTAAC
AGTCAAGTTTGGATGTTGAGAAATCTTCATTCTTTTTAATATAATGATATGCAGCTCTCCTGCGTGATCGAGAAAAAAATCTTC
AAGAGGAACATCAGGAGGGAAATCTCGCTTATTCCTTCTGAAGTTCTCTGTGTTGCTATGTTTCTGAACCTATGGGCTATGGT
CACTTTTAAGGTATGATTTGGATGTTTGGTTCAGCTGACTCTGACAAGACATGTACCCAGTATCAATATAGTCTGGAATTGATT
ATGGGTGGTTAAGGGGGTACGGCAGAATAGATAGTCCGTGCTTGCCAGTAGTTTCAGAAGGTCAGCTTATTAGTTTGGGTTA
CCCTAAGAAAATATGTAGCCCTCACCTCTCAGCCTTAAAAATACCATGCAAATAGGCATGGAAATTTTCTCAACACTTTGATG
TATAGAATGTGATATCATGTAGCATCCTACTAAATTTTCAACTCAAACTCTGAATATACAAGAGAAATCGAAAATACAAATTGG
AATGCTCGTTATTATCCTTTCCTGATTCCCTGAAAAGTTATCTTTTTTTTTTGTTTCCTCCTGTAAAAAGTCACTGTTTGAATTG
GAATTGGAATCTGTTTGGATGGATGCTTGATGCTAGCATACTGCACATCCATGATTTTTCTTCGAGCTTTTCATGACTTACTTAT
TTGGTGTTTCAAAGATTGGGATAGTTTTTCTTCCAGCTTTTCATGACTTAGGCCCTATTCATCCGGCTTATAATCCGTCTTTTTC
AGCTTGTTTTTTCAGCCAAAACAGTGTTTTTCTCTCACAACAAATCAGTCAACAGTGTTTTTCAGACGGCTTATTAGCACAA
CCGAACGGGGCCTTATTTATTATCCTAGTAGCGTCTAAGAAGAAGCTGAAATGCTTGGAGGGAAGCATATTCTGTTGCCTAGG
CCACTTGAGGCGGTGCATGCACTGACCTACTGGCGCTCGGGCTGAGTGCGGCAGCAAACCTGAAACTAAGTGCAGGGCCCT
GTTGTCATCCTTCCCGCAATCTCCTGCGCTGTGCTCGCCGGAGCCCATCGCGGCTGCCGCACCCGTCACTTTCGCCACCGTC
GCTGTCCCCGGGCGCGGCCGCGCACACATGCTCCCCGCCCTCCCCGCGTCTGGCTCAGTGGCTCGTGCGCGCCCCAACCCA
CTCATCATGCACAAACCGGGTTGAAAAGCCGTGCGTCGTGCGCCGCGCAATCATGGCATCCGGGACGACGTCTGCGGCCTC
CATTAGGCCGTTCCAACCCACGGAAGGCAGACCTGCAGCAGCTTCAATGCTTGACTCTCTCCACTCTCCAGCAGGTGCAGT
GCAGCTCACTTCGGTCGTTTCATAGCAAGGTCGATTGATCTCGATCGAGCTAGCGGCCGATGACAGATGCCACTGCCATGTA

GTAGTAGCTTAGGTGCAATCTAGCTAGCTAATAGGTCTGATCACCGCCATCATCCCCCGGTGTTTTACTAGTGCCATCCGACG
AGGAGAGGA
>SsLRR-RLK129
ACATGTCCGGTAGGCATTGTCCAGTATGTGCAAGACAAAACTAGTGAATTTTACACTTGTGATTCTTCATTTGGCTCAAGTAT
TTCTGGTATTCCTGCATCTCAACAAATATATTGCTCTTCTAAAGAGCTATTAAAAGCACCATATGGCAAACAGAATAATTATTG
AATGAATCTAATTAGCCACTTACAATATTTCACAAATATGATTATTTGCGAATTATATTGAGCACACTATTGTAACACCCAAAAA
TTTCAACAATTAGAATTAGTGGGTTTAAAATAATTTAGATAATAATTAATGGGGAATTAGGNNNNNNNNNNNNNNNNNNNN
NNNNNNNNNNNNNNNNNNNNNNNNNNNNNNNNNNNNNNNNNNNNNNNNNNNNNNNNNNNNNNNNNNNNNNNNNNN
NNNATTCCTCTAGTTTTGGTCCCTATCCCTAGAGGGGAATGCTGACTGATGGCCTGTGAACTTTTGAGGATGCAGTAATGATG
TTTGGTTCAGATAACTAGGCTAAACTTTAGGCAACGTCTTGGCTAGCCACTAGTTAACAGTCAAGTTTGGATGTTGAGAAAT
CTTCATTCTTCTTAATATAATGATACACAGCTCTCCTGCGTGATCGAAAAAAAATCTTCAAGAGGAACATCAGGAGGGAAATC
TCGCTCATTCCTTCTGAAGTTCTCTGTGTTGCTATGTTTCTGAACCTATGGGCTATGGTCACTTTTAAGGTATGATTTGGTGTTT
GTTCAGCTGACTCTGGCAAGACATGTACCCAGTATCAATACAGTCTGGAATTGATTATGGGTGGTTAAGGGGGTACGGCAGA
ATAGATAGTCCGTGCTTGCCAGTAGTTTCAGAAGGTCAGCTTATTAGTTTGGGTTACCCTAAGAAAATATGTAGCCCTCACCT
CTCAGCCTTAAAAAAACCATGCAAATAGGCATGAAAATTTTCTCAACACATTGGTGCATAGAATGTGATATCATGTAGCATCC
TACTAAATTTTCAACTCAAACTCTGAATATACAAGAGAAATCGAAAATACAAATTGGAATGCTCGTTATTATCCTTTCCTGATT
CCCTGAAAAAAGTTATCCTTTTTTTTTCCTCCTGTAAAAAGTCACTGTTGAATTGGAATTGGAATCTGTTTCGATGCTTGATGC
TAGCATACTGCACATCCATGATTTTTCTTCCAGCTTTTCATGACTTATTTATTTGGTGTTTCAAAGATTGGGATAGTTTTTCTTC
CAGCTTTTCATGACCTATTTATTATCCTAATAGCGTCTAAGAAGAAGCTGAAATGCTTGGAGGGAAACATATTCTGTTGCCTAG
GCCACTTGAGCGGTGCATGCACTGACCTACTGGCGCTCGGGCTGAGTGCGGCAGCAAACCTGAAACTAAGTGCAGGGCCCT
GTTGTCATCCTTCCCACAATCTCCTGCAATGTGCTAGCCGGAGCTCACCGCGGCTGCCGCACCCGTCACTTTCGCCACCGTC
GCTGTCCCCTGGCGCGGCCGCGCACACATGCTCCCCGCCCTCCCCGCGTCTGGCTCAGTGGCTCGTGCGCGCCCCAACCCA
CTCATCATGCACAAACCGGGTTGAAAAGCCGTGCGTTGTGCGCCGCGCAATCATGGCATCCGGGAAGACGTCTGCGGCCTC
CATTAGGCCGTTCCAACCCACGGAAGGCAGACCTGCAGCAGCTTCAATGCTTGACTCTCTCCACTCTCCAGCAGGTGCAGT
GTAGCTCACTTCGGTCGTTTCATAGCAAGGTCGATTGATCTCGATCGAGCTAGCGGCCGATGACAGATGCCACTGCCATGTAA
TAGTAGCTTAGGTGCAATCTAGCTAGCTAATAGGTCTGATCACCGCCATCATCCCCCGGTGTTTTACTAGTGCCATCCGACGA
GGAGAGGATGAGGGTCCGCACGGCCATTCTCCTCCTGCTAATCCTCGTCGTCCTTTCCTCGCCGCGTCGACGTCGGCGGCCA
GGAAGGCCGTCGGCGACA
>SsLRR-RLK130
GTTCTCTTTTGCAAAATTCCCAAACTCTGAACATTCTAACCCTCTGTTATATTTTTCAGATAGTCTGACTATCTACCCTCTAAA
AACTATCTAGGCTGTATTTTTTTGCTTTCCAACTTGTTGGTTGCATGCAAATATCACATGAGTGTTATTCCTTTCAGAAATGAC
ATGATAGACTACCCTGTGAAAAATAGGATAGCTAACAGCCTAACACTAAGGCCTTGTTTAGATACCCAAATTCACCCCCAAAT
TCCACTATGCAAAAAGAAGATTTCCCATCACATCAAAATGTCGGCACATATATGGAGTACTAAATGTAGATGAAATCAAAAAC
TAATTGTACAGTTTTGTTATACTTTGCGAGACGAATATTTTAAGCCTAATTAATCAATGTTTAGATAATTATTACCAAATAAAAA
CAAAATTGCTACAGTGTTCCACAGGGATTCGGGCACCCCCGAATCGGGCATCTAAACGAGGCCTAAGGACCAAATCTGGCA
TTCCTCTATTTTTGGTCCCTATCCCTAGAGGGGAATGCTGACTGATGGCCTGTGAACTTTTGAGGATGCAGTTATGATGTTTGG
TTCAGATAACTAGGCTAAACTTTAGGCAACGTCACCTTGGCTAGCCACTAGTTAACAGTCAAGTTTGGATGTTGAGAAATCT
TCATTCTTCTTAATATAATGATACGCAGCTCTCCTGCGTGATCGAGAAAAAAAATCTTCAAGAGGAACATCAGGAGGGAAAT
CTCGCTCATTCCTTCTGAAGTTCTCTGTGTTGCTATGTTTCTGAACCTATGGGCTATGGTCACTTTTAAGGTATGATTTGGATGT
TTGGTTCAGCTGACTCTGGCAAGACATGTACCCAGTATCAATACAGTCTGGAATTGATTATGGGTGGTTAAGGGGGTACGGC
AGAATAGATAGTCCGTGCTTGCCAGTAGTTTCAGAAGGTCAGCTTATTAGTTTGGGTTACCCTAAGAAAATATGTAGCCCTCA
CCTCTCAGCCTTAAAAAAACCATGCAAATAGGCATGAAAATTTTCTCAACACATTGGTGCATAGAATGTGATATCATGTAGCA
TCCTACTAAATTTTCAACTCAAACTCTGAATATACAAGAGAAATCGAAAATACAAATTGGAATGCTCGTTATTATCCTTTCCTG
ATTCCCTGAAAAGTTATCCTTTTTTTTTCCTCCTGTAAAAAGTCACTGTTTGAATTGGAATTGGAATCTGTTTCGATGCTTGAT
GCTAGCATACTGCACATCCATGATTTTTCTTCCAGCTTTTCATGACTTATTTATTTGGTGTTTCAAAGATTGGGATAGTTTTTCT
TCCAGCTTTTCATGACCTATTTATTATCCTAATAGCGTCTAAGAAGAAGCTGAAATGCTTGGAGGGAAACATATTCTGTTGCCT
AGGCCACTTGAGGCGGTGCATGCACTGACCTACTGGCGCTCGGGCTGAGTGCGGCAGCAAACCTGAAACTAAGTGCAGGG
CCCTGTTGTCATCCTTCCCACAATCTCCTGCAATGTGCTAGCCGGAGCTCACCGCGGCTGCCGCACCCGTCACTTTCGCCAC
CGTCGCTGTCCCCTGGCGCGGCCGCGCACACATGCTCCCCGCCCTCCCCGCGTCTGGCTCAGTGGCTCGTGCGCGCCCCAA
CCCACTCATCATGCACAAACCGGGTTGAAAAGCCGTGCGTTGTGCGCCGCGCAATCATGGCATCCGGGAAGACGTCTGCGG
CCTCCATTAGGCCGTTCCAACCCACGGAAGGCAGACCTGCAGCAGCTTCAATGCTTGACTCTCTCCACTCTCCAGCAGGTGC
AGTGTAGCTCACTTCGGTCGTTTCATAGCAAGGTCGATTGATCTCGATCGAGCTAGCGGCCGATGACAGATGCCACTGCCAT
GTAATAGTAGCTTAGGTGCAATCTAGCTAGCTAATAGGTCTGATCACCGCCATCATCCCCCGGTGTTTTACTAGTGCCATCCGA
CGAGGAGAGG
>SsLRR-RLK404
TCAACATGTTTTGCTCATAAAGTTGCTTTAGTGACCATCTTGAGGAATGGCATTTTAAATTTTCTTGATGCTTGGTGCTTGGTA
CACCAATAGCACAGAAAGTACTTGATAGGTAGGCAACTAGGGTGCTTGGTGCATCCACCTGAAATTAACCTTGTCACACTTG
GGCTATAAGGCTATAACAGAGAGCTTCATATGGAATTTCCATGTGGCTCTAATAGTTCGTGTTTGGCAGTGGTGACAAATAGA
ATGGGCCTATGGAAATAATTTCTCAGTGCTCATTAAGTCGTGGCAATACTAATACATGAGTCACCCATGCTCAAGCAGCCCTG
AGCATGCCAACTGTGGCATTAAGATTGTTTGGCTAAGAACTTTAGTTTTACTGGGATATACTTTTACTGATGTTCTGGAGCTCG
CTTTGTCAAAAGAAGCATCCAGTACTGGCTTTGGTATAAAATATTCTCAAGTTGCAGTAATGGATCAGTTTTCGACTTTCATC
GTGTTTTGTGCCGTCATTTTGAAGTGGAAAGCCAGATCCCATCAGTTCTTGTTCCATTTATCTTCCCATTGATTTGAGCAACAA
TTTTGCTTTCAACCTGTTTTTCCTGTCACATTTCAGTGCATTAAGACATCATTGCTTTGTAATCTTTTGGTTGGTTTTAGTTTTA
AAATGCCCACTTTTGTGCTTCATCGAGTTGCACGCTCACCTGTCTTCTTGCGGTTATGATTTCTTAGGCCACTTGCTTAAGAA
AGAAATCCAAACCCTTTGTGCCGACCTCCCTGCGGTTTTCGGTTTACATTACCATTCCACGTTGCTCACAGCCTCCTTGCTAT
GAATTTTCTTGCAGAAGGACATCCAGAAACTCCTTGCGGCATAAGTACTTCCAAGTCTTTCAGTTGAGTATCTGTATGACTGT
ATATAGGCCTCGTCTAGAAGCTAGAGTAATATGTAATTATATATATTTTCTGTATTGTATTTCTTTCTTGTAAAATAACACATTCAT
TATCTATACGTGGTTATAGATGTAACATGAGACTATTAGATTTTTATCAACTCCGAAGTTCATCTGTCCATGAAACTCTGCCCA
ATGGATTAGCTTATGGGGTGCACTGGCTGGGAGCATCCTGAATCCTTGACTTGGATTCACCTGTAGTTTGGTGTAGTTGGTTT
GTGTACCGAGTCCAGATGGATCCGTCTGGTGCTGTCTCCACGCATGCGCGCGGCATTGCTAGTTTATGCCGCTATTCTGTATG
CTGTGTTTAGGCAGTTAGCAACACCCAATGTGATCTCTATCAATTAATTAATGTCTGACCATTTCTGGCCTAATCCACAACTTA
ATCTCCACCAAAATCCAACTAGTCCTCTCCATCATGATATTGCCCAATGCCCCACACCGACCTGACGCTATTGCCGAGTTACT
TGATGGCGTTCGCATGAAGGATTGGATTATGCCCTTTGTTGATCATAATATTCTGGAGTGGCAAAGTAACCACAAAGGCCTGT
TTAGGTTTGATTGGATTTCATCCGAAAGTTATAAGAGTTTTGTATGTAAACCAAGGAATATCCTATATGGTTCCTAAGGAATATC
AGCTTATATCCTAACACTTTTTTTAGAAAGGGCGGCAAAAGCTTTGCCGCGATTTATATTAGACGAAGAAAATAAAAATACAA
GTGTCGTAGCTAAGGTCTTGTTTGGAATGGGAGGAATTGTTCCAAGTTCCGTAGGATTAAGGACCAACACGTGTAGTGTGCC
TGCCTTTAGTTAGTCGTCAAAAGATAGAAAACAAATCTGGATAAGGTCAATGTGATCTCACCAGTAGTAGCCCATGATATCAT
GCTTTTGCATTCACCAGTAGTGAACGCCTGCCAGGGCCCACTCCCCCAACCTTTACTCCCCTATTTAGATGACCATCTACCCA
TAGCGTAGCCTCTCATCACCCCTACAATCCATAGCAATTCTGGAACAACCCAAACCTCATTGTAATTGTAGTCTCCACAA
>SsLRR-RLK428

CGCCGGATCATCACCGCTCTCCAGCAGGAGTTCGCGATGAAGGATCTGGGTGAGCTCCACCACTTCCTCGGTGTCACTGTTG
AGCACCGTCCTACCGGGCTCCTTCACCAGCGACAGTACACTCGGGACATCCTGGAGCGGGCTGGGATGACTGACTGCAACC
CCTGCTCCACTCCGGTCGACACTCAGGGCAAGCTGTCTGAGGCTGAGGGACCACCGGTGGCCGATCCCACTGCGTACAGGA
GTCTCGCCGGGGCCCTTCAGTACCTCACCTTCACCCGGCCGGACATCACCTATGCAGTTCAGCAGGTCTGCCTCCATATGCAT
GACCCCCGGGAACCTCACCTCACGGCTCTCAAGCGCCTCCTCCGCTACCTCCGGGGCACGCTCGACTACGGCCTCCTCCTCC
ACCGGGCCTCCTCCACCGACCTCACCGTTTACACCGACGCTGACTGGGCCGGGTGCCCGGATACTCGCCGCTCCACCTCCG
GTTACGCCGTCTTCCTGGGCGGCAACCTGGTGTCTTGGTCGTCCAAGCGCCAGCCGGTGGTCTCCCGGTCCAGCGCCGAGG
CGGAGTACCGCGCCGTTGCTAACGGCGTGGCGGAGGCCGCCTGGCTCAGGCAGCTGCTTGCGGAGTTGCACACTCCGCCCT
CCCGGAGCACACTGATCTACTGCGACAACGTCAGTGCGGTCTACCTCTCCACCAACCCCATCCAGCACCAGCGGACGAAGC
ATGTGGAGATCGACCTCCACTTTGTCCGTGATCGGGTGGCCATGGGCGAGGTGCGGGTCCTTCATGTTCCGACGACCTCCCA
GTTCGCGGACATCTTCACCAAAGGCCTCCCGTCCTCGACCTTCGCCGAGTTTCGCTCCAGCCTCAACATCACCGGTGGCTAG
TTGCGTCTGTGGGGGGTTGTGTGTGTTCTTTCTTGTTCAGCCTTGAACTCCGCTGCGCCGGTAGTTCAGACTGCGGGGGGTG
TTGGAGTATGTGTGTAGGCCCATGAGGCTAAGGCCCATGTGGCCCATGTATGGAACCTATATAGCTACCCTTCTAGGGTTAGC
CCTAATTAATAGAATATTACATTCCAACAGGGTGCACTGGCTGGGAGCATCCTGAATCCTTGGATTCACCCTGTAGTTTGGTG
TAGTTGGTTTGTGTACCGAGTCCAGATGGATCCGTCTGGTGCTGTCTCCACGCATGCGCGCGGCATTGCTAGTTTATGCCGCC
ATTCTGTATGCTGTGTTTAGGCAGTTAGCAACACCCAATGTGATCTCTATCAATTAATTAATGTCTGACCATTTCCGGCCTAAT
GCACGACTTAATCTCCACCAAAATCCAACTAGGCCTCTCCATCATGATATTGCCCAGTGCCCCACACCGACCTGAAGCTATTG
CCGAGTTACTTGATGGTGTTCGCATGAAGGATTGGATTATGCCCTTTGTTGATCATAATATTCTGGAGTGACAAAGTAACCAC
AAAGGCCTGTTTAGGTTTGATTGGATTTCATCCGAAAGTTATAAGAGTTTGTATCTAAACCAAGGAATATCCTATATGGTTCCT
AAGGAATATCAGCTTATATCCTAACACTTTTTTTTAGAAAGGGCGGCAAAAGCTTTGCCGCGATTTATATTAGACGAAGAAAA
TAAAAATACAAGTGTCGTAGCTAAGGTCTTGTTTGGAATGGGAGGAATTGTTCCAAGTTCCGTAGGATTAAGGACCAACACG
TGTAGTGTGCCTGCCTTTAGTTAGTCGTCAAAAGATAGAAAACAAATCTGGATAAGGTCAATGTGATCTCACCAGTAGTAGC
CCATGATATCATGCTTTTGCATTCACCAGTAGTGAACGCCTGCCAGGGCCCACTCCCCCAACCTTTACTCCCCTATTTAGATGA
CCATCTGCCCATAGCGTAGCCTCTCATCACCCCTACAATCCATAGCAATTCTGGAACAATCCAAACCTCATTGTAATTGTAGTC
TCCACAATGCCTCGATTATTCCT
>SsLRR-RLK426
TAGTCTTTTCTATGCACAAGTTGTGATTTCTCAGTAAATGATCTGGACTTGGAAAAAAAAATGAAATACTGCTGTGCTGCATCT
GTGTGATTTGTATTATTTGCACTCTAGAGGACTTCATAGTTCAGATACAAGCATCTTTCGATGGACCACGGTAAATGATTCATT
GCTGTGTCCAAGACTGCAAAGCTATTGCGGCAGCCTCCACAAGAACACGCTTAATTTGTTCCTGAGGTTGCGGCACACAAA
TTGGCATATGTATTAGCTGGTGATAAACCTGAAATGGAGATGTACTATCCTGATCCTGTTTTGAAGCTGTTTAATGATTATTATC
TGATGAGCTGAAACTAACAATCATCAATAGCATTCATTCATCTGTGATCACAACATGGTACCCAGCAACCAGCATTGATCATT
GGTATGGAAGGCAGATATCATAAATTCATCATGCTAGTATTATGATTGGTCTGAAACTCTGGACATGAAGTAGTGGCGCATGAT
CGGTAGTTTGATACAAATATCTTGGTAGGAATCTTTGTCATTCGTGATCTTATCTCCTAATTTTGCACTTGCATGTCTTGTCATT
GAGGCGCTTTCTTCAGATGATTCCACTTACGGTTCCGCCCAAATTGATATTTCTGAATTTACCTCCAATGGCAAGAAAAAGTT
AACCTGCTAATATCAAATATTCAGAACTAGTATCTTTTCTTTTCTGCAGGATTTTCTGGTTTTTTCCCTTCTGACGAATAATTAC
TCTTTTACATTTTTGGTTATGGGCATAATGTTTCCAGATCCACAGAGCACACTAGTAGTTGTCAGTTGTCACCATCAGCGGTAA
AATCTGCACTCCTGCTAAGTTTTTTTTTATTTTTTTTCATATGAACACCTCTCAGCTGTCAATCTGTCACCAGACCTTTCTAGA
AAGAACCAGCCATAGCTGATACATGCGCATACTTCGGTCCCGTTTGGATCCTCAGGATTTTGATAATCCGGATTAAAAAAATC
CCACAATTCTATTCTCATCAAACGGTCTAGATTTTATAGGGATTTTATCTCACAGATTTTTAGAATCTCGAAATCCAATAAAACT
GTTAGGATTCTTTTTATCCGGATTCTCACAATCCCTTATGATCCAAATGCCACCTTCATCATGCTGTTTATGCTTGTACATACAA
TTTGTTCTGGTTTAGTGTTAACACTTATTGAAGCATTGCCCTCCACCGAGGAGAAACCAAGGCACATTTTTATTCTTTTATCTT
TAAGAGCATCTTCACTACGTCTTCTATCTAATCTTTCTATACACAAAAAACTACTCCCTCCATTTCAAATTATAAGAAGTTTTG
GCTTTTCTAGATACATTGGTTTTATTATGTATCTAGACATAATATATATATATCTAAGTGCATAGAAAAAAGTACCTAGAAAAGC
CAAAATGTCTTATGATTTGAATAAAGGGAATAACATTTAAGGAGAAAAATGTGCTCCTATCTCATCTTATATACCTACAATTTTT
AAGATCTCCTAAAAACAGCCCTCTCCCCCCTAAAGAAAGGAGAAAAAAACACATCTCCTGTCCCTAAAGAAATACAGGAGA
CAAATTTTCAGTAATTTGCTACAATAGTGCTCTCATATATATTTAGTTCAGGGTACTCTTTACTCTCCTAAAACAACTTATTAGA
AGCAATCTCCTAAAACAACTTGTAGGAGACNNNNNNNNNNNNNNNNNNNNNNNNNNNNNNNNNNNNNNNNNNNNNNNNNNN
NNNNNNNNNNNNNNNNNNNNNNNNNNNNNNNNNNNNNNNNNNNNNNNNNNNCCACAGGGAGTCGAACTCAGACCTT
GAGGGGTGCGCCAGAATTCCCTAACCAAAGGTTTATTTACTTTATTGGTTATCAAAATGATCTTATTTGATAGCTGGCTCGTAT
CTGTATTGAGATGTATTTTGAACTATACCATAACACATAGAACCAGAATTATAGTGTTGTTTCGTTATATTTGAGTCATTTC
>SsLRR-RLK403
CCCCCGCCAACGTTGCCTGCTCGCTCTGTTCACGGGACGGACCCCGTCCTCGCCTGCGCCGCTCGCGTCCCCGCCGCCCGTC
CACGCCGTGCACGGCGAGCCCGACGTGCTAGGCACTCGCTAGCTCGCTCACGAACTCGCACCTCCCGTCGCAGCCGTGCTC
CATCCGCCTGCCGCTACCGAGGTCCATGCCGTCGATGAGTCCACACTGGCAACCTCCACGTCACTCCGCGACTTTGAGCTCC
ATGCCAGCGTTAAGCTCCGCATCAATGAGCCTCCATTCGTCCAAGCAGCAAGCAAATGCTACGCTGAAAGCACATGTTGCAA
TAGTATGTTTCAGGTGTTTCCGAGGTATGTGGCAAGTGTTGTATATCGATGTTGTAAAAGCAGATCGGGATATTGCACATGTTG
TAATGGCTACTTGGCTATACACGTATGTTTAAAGTGTATGTTCCAAATGTTTCATCTATTTTAAACGAATGTTGCAAGTGTTTTG
TCTGGATGATGCAAAAGTAGATCTAGATGTTGCATATACATGCATGTTGCAAGCATATGTTTCAAGTGTTTTTAGGCGTTTCAT
ACGTATGTTTGCAAGTGTTTCATCTAGATGTTGCATATATTTGCAATGGTTGTCAAATGTTTTTAAGCCGTTTTCGCAAGTGTTT
CAGATGCTTGTTTCAAGTGTTTTATTTGTCTTCTTCTGTATGTTGCAACTGTTGTATCTGGATGTTTCAAAAGTAGATCGGGTG
TTCAACATGGGATGCGCTGGGAAGCGGCTGGCGGTGCGAACGATATCCGGGGCGGCATAGGACCACTACTAGTACGCTCCC
TCGTGAGCCCGACGCGGTAGGCACTCGTTCGCTCCTAGTGCGGGCAGCGTCCGAACGCTAGCGCCCGAATCGGACGCTAGA
GCGCTGGCAAAATAGGAACACCCTTCCTTGTGTATAGTATCCATTGACGCGTGGAGAAACAGAACTCACGGCCATGGGTAGT
TTGACACGTGGCTTCAATAAGCAAAACTAATCTATGTTTGACATTCACGGGAACAACTTTGAAATCTGCAGGTGGGCTATCTC
AAATGATTAATAACCAAAACCCATCCCTTGATCTCAAGTCAAACATTTGGCGCCAAAGCTAGCTAATCCGCTGCCTTTCAAGT
TTCAACTGCAGTTAGGCAGCCACCTTCCCGCCTTTTCTTTCTCTTCCTCGTATCTTCTCCAGGCTCCAGCTACTACACGTCTAC
ACCTCCGCGCGCGGCCCCTGTTCACCATAAAACCCACGGCATTGTAGCAGACGTTGCTATCAATGGTCACCACATCCGCCTC
GTTGCCACTGCCCTTTCGGCCACCACCATGGCCAAAATGCTCATCCCAATCATCATCTTTCATGTCGGCCTCATCGTGCCGAT
CGCTGTCGCGGCCGCCGCCACCGCACGACCGCCTGCAGTGGCCGACGCCGACGCCGACCGGTCCACGCTCCTCGCGTTCCT
CTCCAACGTCTCGGCGGACCCCGGCGGCGCCCTCTCAGACTGGGGCCGCTCACCGGGGTCGTTCTGCAACTGGACGGGCGT
CACGTGTGGCGGCGGCGGTCCGGGCCGGCAGCGCGTCACGCAGCTGGTGCTGAGCGGGAAGGAGCTCCGCGGGGCGATCT
CACCGGCGCTGGGGCGGCTGTCTTTCCTCACGGTGCTCGATCTTTCGAACAATGCTTTCGCCGGCACGATTCCGTCGGAGCT
TGCCGCGCTCTCGGCCCTGACGCAGCTCAGCCTGACGAACAACCTCCTCGAGGGCGCGGTCCCCGCCGGCCTCGGCCTCCT
CCAGAGGCTCTACTTCCTCGACCTCAGCGGGAACCGGCTCTCCGGCAGCATCCCGGAGACGCTCTTCTGCAACTGCTCCGC
GCTGCAGTACCTAGACCTCGCCAACAACTCCCTCGCCGGCGACATTCCCTACGCCGCCGGCTGCCGCCTCCCCAGCCTCCGG
TTCCTCCTCCTCTGGTCGAACGAC
>SsLRR-RLK427
CCCATGACCGTGAAACACTATCTTGCTCAAAATTAATATAATTGGTAGCTGAAATCAATGACATGTATGTCTGTCTTTTTAAAC
TATGTCTTAATAGAAAATTAAACAAGTTAACAACCGAACTAATTACACTAGGGGCAAGTTAGGTTTTTTAGTAGACTCTTTAA
TTACTTGGTTCTAAAATTGCTTGAGTACCTTGTATTTAGGACGAAGGGAGTACTATCTCGTATAAACAAGTTTGTTTGAGGAA

GGTTTATCGTGGGGCAATTGTAGCAGCAATGTGATGGAAATTGTTGATCCTAGAAACACATACATATGTGTATGTATGAATCAG
CTGGCTTATCTTACCATTTGTTCGTCGATATGTTGTGTGAACAATAACCTTTCTTTGGATGTTTACTTGTGCATTTCTCCTATATT
TTAGGTGTCTATTTTTTTATGTTTAGGTAACGTGTATTTTGTCATTGTTGGATTATAGAGAAGAATTGTGAAATGTCTCTCATGA
CCTGTCCTTTTTAACTGTACTAGTTTATTTTGCTTGAAAACTCAGGCATTAAAATATCGGAAACGCTCTTGTAGGGAGTCCGT
CCGTCCGTCCGGACGGATCCTGCACTCCCCACGTTGCCCCCGCCGCGTTGCTGCTTGCTCTGATCACGGGACGGACCCCGTC
CTCGCCGCGCCGCTCCCGCTCGCGTCCCCCCCCCGTCCACGCCGTGCACGGCGAGCCCGATGCTAGGCATCGCTAGCTCGCT
CACGGACTCGCGCCTCCCGTTGCAGCCATGCTCCATCCGCTTGCGCTACCGAGGTCCGTGCCACCGACGAGTCCACACCGG
CGACCTCCACGCCACTCCGCGACTTCGAGCTCCGTGCCGATGGCAAGCTCCACATCGATGAGCCTCCATTCGTCCAAGTAGC
AAGCAATGCTACACTGAAAGCGTATGTTGCAATAGTATGTTTCAAATGTTTTAGATGTTTCGAGGTATGTGGCAAGTATTGTAT
ATCGATGTTGTAAAAGTAGATCGGGATGTTGCATATGTTGTAATGGCTACTGGCTATACACGTATGTTTCAAGTGTATGTTCCA
AATGTTTCATCTTTTTAGACGAATGTTGCAAGTGTTTTGTCTGGATGTTGCAAAAGTAGATCTAGATGTTGCATATACATGCAT
GTTGCAAGCATATGTTTCAAGTGTTTTTAGGTGTTTCATACGTATGTTTGCAAGTGTTTCATCTGGATGTTTCATATGTTTGCAA
TGGTTGTCAAATGTTTTTAGCCGTTTTCGCTAGTGTTTCAATGCTTGTTTAAGTGTTTCATTTGTCTTCTTCTGTATGTTGCAAC
TGTTGCATCTGGATGTTTCAAAAGTAGATCGGGTGTTCCACATGGGATGCGCTGGGAAGCGGCCAGAGGCGCGGACGACAT
CCGGGGCGGCATAGGACCACTACTAGTGCGCTCCCTCGCGAGCCCGACGCAGTAGGCGCTCGTTCGCTCCCAGTGCGGGCA
GCGTCCGGACACTAGCGCCCGAATCGGACGCCAGAGCGCTGGCAAAATAGGAACACCCTTCCTTGTGTATAGTATCCATTGA
CGCGTGGAGAAACAGAACTCACGGCCATGGGTAGTTTGACACGTGGCTTCAATGATAAGCAAAACTAATCTATGTTTGACAT
TCACGGGAACAACTGTGAAATCTGCAGGGAACAACTGTGAAATATGCAGGTGGGCTATCTCAAATGATTAATAACCAAAACC
CATCCCTTGATCTCAAGTCAAACATTTGGCGCCAAAGCTAGCTAATCCGCTGCCTTTCAAGTTTCAACTGCAGTTAGGCAGC
CACCTTCCCGCCTTTTCTTTCTCTTCCTCGTATCTTCTCCAGGCTCCAGCTACTACACGTCTACACCTCCGCGCGCGGCCCCTG
TTCACCATAAAACCCACGGCATTGTAGCAGACGTTGCTATCAATGGTCACCACATCCGCCTCGTTGCCACTGCCCTTTCGGCC
ACCACC
>SsLRR-RLK171
ATATCCTTGTGTGAGTATGTTTAATTTTATTTTTTTTATTGTTGAACAAACTGTTATTGCATTTTCGATCTTTGGTCTTAAGTATG
CGACAAACAAGACATCAACAACTTATTTTGACATGAAAAATATCCATGGGTCAATTTGCTCCAACATGGTCCAGAATTTACAA
TATGGGCCAAAATCACATATTGGGGATGAGATATTGAGGAACTGCAAAAGCATCTCCCCCAATAACCCAAAAAGTAAATATTG
GGCTACCCAAATACTAAGCTATTGGGGGAGTTTTATTGGGTGACTGGTGGAGATGCTCTTAGGACCTTTCAGCTGTCCAACTA
GGCAATGCACTATCACGTAGAATTAAAATCCACTAAGCAAAACCCCACTAGCTATTTATCTCAACATGCAAGCTATCCAACTA
GGCAATGCACTATCACGTGCAATTAAAATCCACTAGCACATGCAACTGCAATGTCCACGTCAGCAAAATAAATAAGTATTTTT
TGTCCACTCATTATACCACGCAATCCACTAATCCGTAATTTCTGCGGCAATGCGCGAGGTATGCTTCTAGTATACCCTATTTTCC
TTGGCGTAGCATTGATACTTCACAAATCACAATCGCCTCCCTTGTTTTGTTGTTTTTGCCGATTACCACCATGTTCGTTTGAGC
TATTCAGCCAAATCAGCAGTCATTCAGCAGTGTTTTTCTCTCATAATAAACCAGCGAACAGTACTTTCGGCCACAATAAACCA
GCGAAACAAACAGGCTGTACATCGAAGATGCACACGCACACCGCAACACTACTGCTATTGTTTCATCGAGTCCCGCAGGTC
TGCATTGGCCCGCCAGAAGTAGGCAGAAGCGAAGCAAGTCATCAGTTTTTTCACCGTTCCACGGCGTCGTTGCCGGTTTGC
GGATAGACTTTCCCGTGCAAATGACAATTAGTCAGTGAGTTCCCATGCTGTACGCTTTCTCTGATCTCTGTACTCCCATCCCTC
TCAGTCTCAAGTACTTAACATGTATGATGGAGACGCTGTTTTTTAGTTACTTGCGTTCAGGCTCAACCTTTAATTTCCAATGTT
CGAACCCGTCAAATGGTTCTAATTCTATCTTGTGTGTATCGGTCCATGTTCAGGTCAAGTACACGATGACTTGAGTCATGCGT
GCGTGTGGTTTGGAGTGGCCCCTCGGGCTTGACTGACGCTCCCGGTTTCAGTCCCATCGCTTTCACCCCGGCGAGTACGCTT
GGAGCTGTCTCCAGTCCAGTAGCTTTCGGCTTTCCTTTGGGCTTCAGCCAAGTTCGATTGATTCCTGGCTCCAACAGTCAAG
TCTTCGTGAGTCCTGTCACTTCATTAGTTCATTTCTCTTGGGCCTCGTTTAGATCCCCGACTCGGGGTGGCCAAATCTGCACA
GTGCCACCCCGAGTACTGTAGCATTTTTGTTTTTTATGGTAATAATTGTCCAAATTTTGATTAATTAGGCTCAAAATATTCGTCT
CGCAAAGTACAACAAAACTGTGCAATTAGTTTTTGATTTCATCTACATTTAGTACTCCATATATATGCCGACATTTTGATGTGAT
GAGGAATCTTCTTTTTGCATAGTGGAATTTGGGGTTGAATTCTGGAATCTAAACCAGGCCTTGCTTTCTCCTCTTCGGACGAC
GACTAACCTTTGATTTCAGCAAATTCGTTCATACTTCTGTTAGGCCATGTTTAGTTTTCAAAATATTTCAAGATTCTCCATTACA
TCAAATGTTTAGACACATGTATAGAGTATTAAATATAGATAAAAGAAAAAAATAATTACACAGTTTGGTAGAAAACCACGAGA
CAAATCTTTTAAGCCTAATTAGTTCATGATTAAACACTAATTAACAAATACAAACAAAAGTGCTACAGTAGCCAAACCCAAA
ATATTTCACAAACTAAACACACCCTTAGTTTTCTTTTGCAAGATCAGATACTCCCAGCGCTCCACGGCTACGACCTTCACGAG
C
>SsLRR-RLK360
CATACAAGGTGATATCCCTTGGTGTATGCTCTTTGCTGATGATGTGGTGCTAGTTGACGACAGTAGGGCAGGGGTTAATAGGA
AGTTAGAACTATGGAAGCACATGTTAGAGTCGAAAGAGTTCAGACTTAGTAGGACTAAGACCGAGTACATGAGGTGCGATT
TCAGCGCGACTAGGCATGAGGAGGGAGACGTTAGCCTCGATGGGCAAGTGGTGGCCAAGAAGGATACTTTTCGGTATTTAG
GGTCGATGCTACAAAAGGATGGAGACATTGATGAAGATGTTAGGCATAGAATTTCAGCCGGCTGGTTGAAATGGTGTCAAGC
TTCTGGCGTCCTCTGTGACAAAAGGGTGCCGCAGAAGCTAAAAGGTAAGTTCTATATGGCGGCGATTCGTCCGACGATGTTA
TATGGTGCTGAGTGTTGGCCTACAAAAAGGCGACATGTCCAGCAATTGAGTGTAGCTGAGATGCGTATGTTGCGTTGGTGTT
ATGGGCACACAAGGAGGGATAGAGTCCGGAACGACGATATTCGGGATAGGGTTGGGGTGGCACCAATTGAGGAGAAATTGA
TTCAACATCTGTTGAGATGGTTTGGATATGTCCAACGGAGGCCTCCTGAGGCGCCGGTGCGTAGTGGAGTTCTAAAGCGGGC
CGATAATGTAAAGAGTGGTAGAGGTCGACCTAAACTAACTTGGGACGAGTCCGTTAAGAGAGATCTTAAGGAGTGGAATATC
TCCAAGGATTTAGCTATGGATAGGAGCGCTTGGAGATTGGCAATCAACGTGCTTGAACCGTGACCTATTTTTGTCTTGTGCCC
TTTATATCTCTTTTATGTGTCTCCCTGTTTCTCGTCTCTTTTGGGTTTTATCTCAAGCCTACCCAAACTTGCTTGGGACAAAAG
ACTATGTTGTTGTTGTTGCTACTGCTATCACGTTTGACGAACTGTCTTTGGTAGAGATATGTCACTACTCACTACCCCATGAGC
ATTTCTTCAGAATCACGACTGTATGTTGAATTATCATATGGGCTTTGTTGTGGTACACCCAAATGACTGTGAGCCATTCATTTA
ATTAGATCTGATGGTTGAAATCATCTATTACTTTCTATGAGGTAATAGGTGAAATAAAGAAAGTTTTAGATGCAAAGATCCTTG
TTTAATCTAATTGATGCACACATGATCTATTAGATATGGTGAATCTTTTTATTTTGAAACTTTGAATACAACATAACTTAATTAG
AATAAACTAACAATTTTTTGATGCACTTGTAACTCAGGCACGCAACCAGATTTAAATGTTTGTGTGTATGTGCACACTCGATT
ATGTACCGTGTTGGTTTGTAACATGTACTAAATTGCTAAAGTCCACAGCTCGATTAACGTGCTAGATGTCGATATTTTTCATGT
ATGTAACATATGTTTTTTAGTTGAGGATGAAACGTGACCAGTGTACCTTTTTAACATAACTTAATATTATAAAAAAATTAACCA
CTTCTAAGACAAAAAAGGTATTATGCAGAAGGAAGAATACAACGACAGTCATGAAAGAAAGAATACGGCTATGAACAAGGA
AGCGGTTGCGTGCCCACCCACATCTCCCGTGATTAGAGGGATTTGAGGATATTTTTTATTTTTCTAGATTTATTAATTCATTTAA
AATCTAAAAAAATCAATAACTCAGATTTATTTAAGATATTATCTTGTAGCAAATAATGGGTGTACCGTATCATTGCCCTTATCAT
ATAGCATCGTGTGGGTCACGCGGGCGCAATACAAGCTGCCAGATGGCCACTTGTTATTGACAAATACAGCAGGCGGGTCGTA
GCTTGTTCCTTGTCCACACTCCACACTCGTACACCTTTGTTCCTACTATGAAATTCCGTGGGGAAGATTAGCAGGAGCAGCTC
CATCCATGGCTACCACCACATGCATTTCAATCGTCCTACTACTAGCTCTATCAGCCCCTGTAGCTTCATCATCTCCTATTCCGAT
CAACAGCA
>SsLRR-RLK390
AGTGACTTTCCAATTAAAATAGGACTGCATCAGGGGTCAGCTTTGAGCCCTTATTTATTTGCTTTGGTGATGGATGAGGTCAC
AAGGGACATACAAGGTGATATCCCTTGGTGTATGCTCTTTGCTGATGATGTGGTGCTAGTTGACGACAGTAGGGCAGGGGTT
AATAGGAAGTTAGAACTATGGAAGCACATGTTAGAGTCGAAAGAGTTCAGACTTAGTAGGACTAAGACCGAGTACATGAGG
TGCGATTTCAGCGCGACTAGGCATGAGGAGAGAGACGTTAGCCTCGATGGGCAAGTGGTGGCCAAGAAGGATACTTTTCGG
TATTTAGGGTCGATGCTACAAAAGGATGGAGACATTGATGAAGATGTTAGGCATAGAATTTCAGCCGGCTGGTTGAAATGGC

GTCAAGCTTCTGGCGTCCTCTGTGACAAAAGGGTGCCGCAGAAGCTAAAAGGTAAGTTCTATATGACGGCGATTCGTCCGA
CGATGTTATACGGTGCTGAGTGTTGGCCTATAAAAAGACGACATGTCCAGCAATTGAGTGTAGCTGAGATGCGTATGTTGCGT
TGGTGTTATGGGCACACAAGGAGGGATAGAGTCCGTAACGACGATATTCGGGATAGGGTTGGGGTGGCACCAATTGAGGAG
AAATTGATTCAACATCTGTTGAGATGGTTTGGACATGTCCAACGGAGGCCTCCTGAGGCGTCGGTGCGTAGTGGAGTTCTAA
AGCGGGCCGATAATGTAAAGAGTGGTAGAGGTCGACCTAAACTAACTTGGGACGAGTCCGTTAAGAGAGATCTTAAGGAGT
GGAATATCTCCAAGGAGTTAGCTATGGATAGGAGCGCTTGGAGATTGGCAATCAACGTGCTTGAACCATGACCTATTTTTGTC
TTGTGCCCTTTATATCCCTTTTATGTGTCTCCCTGTTTCTCGTCTCTTTTGGATTTCATCTCTAGCCTACCCAAACTTGCTTGGG
ACAAAAGACTATGTTGTTGTTGTTGCTACTGCTATCACGTTTGACGAACTGTCTTTGGTAGAGATATGTCACTACTCACTACC
CCATGAGCATTTCTTCAGAATCACGACTGTATGTTGAATTATCATATGGGCTTTGTTGTGGTACACCCAAATGACTGTGAGCC
GTTCATTTAATTAGATCTGATGGTTGAAATCATCTATTACTTTCTATGAGGTAATAGGTGAAATAAAGAAAGTTTTAGATGCAA
AGATCCTTGTTTAATCTAATTGATGCACACATGATCTATTAGATATGGTGAATCTTTTTATTTTGAAACTTTGAATACAACATAA
CTTAATTAGAATAAACTAACAATTTTTTGATGCACTTGTAACTCAGGCACGCAACCAGATTTAAATGTTTGTGTGTATGTGCAC
ACTCGATTATGTACCGTGTTGGTTTGTAACGTGTACTAAATTGCTAAAGTCCACAGCTCGATTAACGTGCTAGATGTCGATATT
TTTCATGTATGTAACATATGTTTTTTAGTTGAGGATGAAACGTGACCAGTGTACCTTTTTAACATAACTTAATATTATAAAAAAA
TTGACCACTTCTAAGACAAAAAAATATTATGCAGAAGGAAGAATACAACGACAGTCATGAAAGGAAGAATACGGCCATGAA
CAAGGAAGCGGTTGTGTGCCCACCCACATCTCCCGTGATTAGAGGGATTTGAGGATATTTTTTTATTTTTCTAGATTTATTAAT
TCATTTAAAATCTAAAAAAATTAATAGCTCAGATTTATTTAAGATATTATCTTGTAGCAAATAATGGGTGTACCGTAACATTGCC
CTTATCATATAGCATCGTGTGGGTCACGCGGGCGCAATACAAGCTGCCAGATGGCCACTTGTTATTGACAAATACAGCAGGCG
GGTCGTAGCTTGTTCCTTGTCCACACTCCACACTCGTACACCTTTGTTCCTACTATGAAATTCCGTTGAAGATTAGCAGGAGC
AGCTCCATCCA
>SsLRR-RLK371
TAGACGCTTTATGGCTTTGCACTAGTTTTTAGTGACCAATTGTTGTTATAAGTTTTAGAGTTTGAGTCAATTTCAGGATCATAA
TAAACTTTAAAATTTATTCATCACCAGAGTATGCAGGTTCATACCAGTCTATAAATGTACGTAAAGCCTGCGGCCCGGCAGCC
AAAGCCCGCTTTTTTGGCCTAGCACGAGCACGGCACGACCCGATGGTGTGCAGGCCTAGAACAGCCCTATCCGCTAGCTAG
TGCTATGCTTGGACTGCTTCCTAGGCACGACGAGCAGTATGGCATGAACAGTTAAATAGCCTCGGTCCGGTACTAAGCCCGC
TAATAGCCTAATAGGCCAACATCCCTACAGCCTAACACCTTAACTCCCAACGCTAACCGGTCCCCATTCACCCACTTGGCCAT
TTTCCGATTCCCACCCGCACCACCTCCCACTCGTGAGTCGAGTTTCCACCCGCTGAGATGTAGATGGATGAGTGGGGTGACG
TGCATCGCCCAACTCCCCTCGCCACCTCACGCAGGTAGCCTCTACCTCCTCCGCCCTACTACCGCCGCCTCTCCCTTACCCCT
GTCTCACGGCCACAAAGGTTAGCCTCAAGCACCCGAGCATGGGGGCGAGCGCGACACTACACTGGCCACGAAGGCACCGA
CCTTGAGCACACGCCGACCGCCTCTTCCCCATCCTGCTGGCTGCCTCTCCCGGCACCTGCCTCGAGCACCTCCCTTGGTTTC
ACAGTTCGGCGACGGGGCCAATGAAGCGCGAAGACGTGGGTCTCGGGTTGGCCTGACCTACTCTACAAAACCATGCTTATT
GGGCCAGCCTGGCTCGAAAATCAGCTCAGTGTGCCATGCCTGGGCCGATGGCCAGTTGGCGCGAAGCCCTTTTTGGCATGG
CCCGATGGCCTGACATGCCATAGCCGGCCTAGCCTTATCGGGCCGTGCTTTTATTGGACCCGTACCGGGCCGTGTCCGTTTGG
CCATCTATATACCACTCCACATGTAGACGATACATAATTATAACTAGCTCCATGAGGCATATCCCACTTGCGGAATTTAGCCGAT
TAGATCAGGAGTGCAAAGCATCGATGTGTCGTCTCATCTTCTTGTTACTAGATTATACTCTATCCGTTCTAAATTAAAGACATT
TTGGCTTTCTAGATATGTAGTTTTTTCTATATACTTAGATATACACTATGTCTACATATGTAACGTCTTATATTTTGGAATTAAGAG
AGTATATAGAAATTTTTGGAAAATGGTACAGCTGAACAAGATGTGCGTATGTAGGACGCCTCAAAGTCGGTTGACTGAAAAT
CCCTTTTCGGTAAGTACTAATGAAACTTTCAAATTTCAATCATATCCACCACTTCCCAAATGCCAACTTTAATTTCCACATACT
AGCATCTAGTGCACGATGGGTACAAACGTAACTGTTGATCGAATATGGTTTCAGACTTTCAGTGAGGTGTGAGGAGTTCATT
TCTTTCTGAAATGACGTGAGAACTTTATTCTCCAATATCTTGGTATAGTGCCATTCCTGTTAAGAACGACGTATAGAAATATGA
AGTAAACGAAATCAATCACAGCGGAGACACACGATTTACGTGGAAAACCCTTTCAATGCGAAGGGGAAAAACCACGGGCA
CTAGCTGGCAACAATCTCACTATCTCAAGAGTTTGGGGTTACACGCCTGCGGCGGCTTACAAGAGAATCGAATCTCCACGAA
ACCGGCCCAAGCCTTCGGCGACGGGCCTCCGCTTCGCTTCGTCAGAAGCCCGGCCTATATCTTGCAGTGAATTTGGAACAAC
TCCAACAATTCCACTATCAAACATGGAACTTGCCACTTGTTAGTCCAAACTCCGTGTTTGCTGCGCTTGCGACAACTTAGGTT
GACCAGTGGTCAAGGCCTTGGATGTGTTATGTTACTTAAAAACTCCGATTGCTTGCAAACTCTTGCTGCCAGTGCACTACAC
TACACTGCACCCCAAACA
>SsLRR-RLK392
GTACTGCTCCTCCCTCTGCAGCTTGCCAGTGAGGTCATGTTTAGATAGCAAAGTTTGGGAACAAAGTCTGCATAGTAAATATC
ACGTCATATTGTAGCAATTTTGTTTTTATTTGATAATAATTGTCCAAATATTGATTAATTAGGCTTAAAATGTTCGTCTCGCAAA
GTACAACAAAACTGTATAATTAGATTTTTATTTCATCTATATTTAGTACTCCATATATATACCGGCATTTTAATGTGATGGAGAAT
AGAAATTCTGCATAGTGAATTTGGTGGTCAAAATTGGGTATCTAAACAAGGCCTGAATGTCTCCATCCCCATGTGAAACAGC
AGGAGCCACACATGGGAGAGGAACTCGGCACCTCTTTATTACACACACTTGCTGTGTTTTTTTTCTTTTGGTTGCGCGCAAA
GTATTCTCAGCCTGTTTAGTTTGAAAAAGCAAACTCAAAAAGTGCTACAGTACCTGTCACATCAAATCTTACAGCATATGCAT
AGAGTACTAAATATAGACGAAAGAAAAAACTAATTATACAGTTTGGTTGTAAATCACGAGACAAATCTTTTGAGGCTAATTAA
TCCATGATTGAACACAAATTGCCAAATAAAATAAAAAGTGCTACAGTAGCCGAACCTAAACTTTTTCTCCAACGAAATAAGC
CCTCTATGAGGCTATATTGAGACCACCTCTGGCCACTCTCTTCCCTAGCCGCCTCCGTGTATCGTACACTACGAAATAGAGGG
CGTTCTCAATGGCAACTCGCAAATCGTTAGCTAGATGAGACTTGGAAACAATAAAAAAGAGCACAGTGGTGGAGGGTTATG
CAGGCTGTGAGAGAGTTGTTTCTAAAAAATCATACGAGATTAGCTATTATAGAGTCACTAGCTCTAATCTCTCTCCTCAATAAC
CAATAGAATTTTTCCTAGACTAGCTATTTGGTAACCGTTGGGAATAAATAAAGTTGCAACGCAGCAGTACAACAGAATATGAC
AGTTGCCCAGACCACCTCCAGCTCCAGCAACTACATTGCTACAGTTGCCGCCTCCGTTATCTCTTACAGTATGACGGTTGAA
GAGCGTGGCGAGGACGTAGATGGCCACAGCATCGCCGCCAAGGTACGCCAGCCACGTTATGGATCTGACCCACCCAGGAAC
TGCAACCCCACACAACAAGGTACAACTCGATCAGTCATCCCCATCCTTGTCTCAATCCACACCGGTAGATGTGGTGTCCAAT
TTTTTAGCAGCTGAGCAGAAAACACTTGTTTTTCAAATGAAACCGGGTCTCATCTGAATGCTGAGTACATCCCGTATCGCAA
GAAAAAATTGACTACTATACAATACAAGGATAGAAAAAAAAAGATCTTATTGTCGCATATCATCTGAACTGAATGTAGTATCA
TTTTTATCTTATCCTAGTAAAATAAAATTGAACTACGGATAACCATGGCTCATGCTTCCAGCCTGGTCATTTCTGATTTTTGCCT
ACAACTGTGGTTGTTTACTTGTTTCCTATCCTAGCTTACAGTAGAAAGCTGTTGACTGTGTGCAGCCACAGCCAAAAGTAGC
TAGTAAATTTCATAAGCAGATAAGCTAGTAAACTTCAGCACGTTTTAATACAAGATAGCTGTTGACTTGTGTTAACTGTTGATT
GTTGAAGGAGCACAGAGCACTTTGTCTCAGGAGAACACCCCAAGTTGTACAGTTTTTTTTTCTCAACACAAAAATAAGTGT
TGCATAAAACCTTTTGCTTGTCTTTTGAAAAAGAAAATACAATGCACTACTCGTCCGCTTGTACACGGCGCCTACGTGCTTAT
CAGCATACTTCGGCAACCGTAGCAGTCATTAGGCCTGTCATGAATAAATGGAGTGCTAGTACTGCTTGACCCTGTATGCCATA
TTGCCATTGCGAAGGACAACGAAAGCAATATTTTATATGTAGTACGTATGCCTTGATCTTGTATATATCACTTCATTTCATAACT
CCGGCCAGCA
>SsLRR-RLK328
TCTAGGATTTATTTTTTTTATAGCGTGTCTTGTGAGAATTAACGTGGTGCCTCCGTTGGGGCCTGTAATTAGTAATAGATAGTA
AGACGTTAAGAGACATCTATTTAGTGTTATAAATGTTAATAATATTCTTTATGAAACTGACCAAACTTGAAATAGTTTCATTTCC
GCCAAAGACGGAGACGAGGGAGGAAGAGTGCTCGGATGGGGTCACGGGCGCAGGTGCGGGTGCGGTCACTATCGCAGGC
CGACTGGAGCGGGAGGGGCAGTTCGGACGGAGCATTGGGTCCAAACGATCGGACCCGATGATTTTCGAAAAGACATTTACT
TGTGACCAGTATCCATTGCATGCACACATCAGTGCAGTGTGGCACGTTTGCCGACACAATTAAACAGGGCAGGCTGGCTCCT
AGTGTCCAACACAATTAACCAAGTCGGCCAATATACAAGGCCGCAGTAAAAAATATTGAAATGATGGTACGTCATGTGGAGG
ACACATGCACATAGTGGCCAAATTCGCAGTTCTGTTGGAAGCTCCCTAAAGGATCCAGTTTCCTGATAAGTAAGCTTATAACA

CATGCAGAGTCGTATCCGATGCATGATGCATGATGGTTGACTTTCATTAGTTTTTGCACCATGAATTACAGATCAATTAATTCT
GGTTCAATCATTTCCATGAACGACAGGAACAGATTGTTCTTATTTGGATCCTAGCCGCCACTGCACGTGTGGTGCACGTGGG
CGGCGGACTGCTGTTATTACGACTCATGTACTCCCTCCGTATTGGATTTACAAGTCGTTTTAGACAAAGTTTGGGTTAAACAT
TGGGAATATAAATCATCAATAACTTTTAAGTTGTTGAGTTTGCAAATGTAAAAATTATATGAATAGATTTGTCTTGAAAAATAC
TTTCATAAAAGTATACATATATCACTTTTTCACAAATATTTTTTATAAAAATAAGAGGTCAAAGTTATGTTTTGGAGACCGTGT
CGCTGTCCTAAACGACTTCTAAATCCGATATATATATATATATATGTATATATATATATATATATATATATATATATATATATATAT
ATATATATATATATATATGGGAGAGTATACTCTATAGCTAGCTACAAAATAAGTTATTTTGTAGCCACCTCTATTTACGATAATTTT
ATATACTAATTTACGACAATGTCAGTATATATTTACGATAGTTGAATGACTATAACACATGGGGATATTTACTATAATGTTATAGT
AAATCACATAGTAAGGAGTTACTATAATTTTGTAAATTAACATAACAATTATCGTAACTCAAAGTGGCTACAGAATAAGTTATT
TTATAGCCAGCTATAAGTTAATAGTTCTATATATATATATATATATATATATATATATATATATATATATATATATATATATATATA
TATATATTACTCTACAGCTGACTACAAAATAACTTATTCTGTAGTCACTTTGAGTTACGATAATTGCTATGCTAATTTACGAGAT
TATAGTAACTCCTTACTAAGTGGTTTACTACAACGTTACGGTAAATATCCCTACGTGTTATAGTAATTCAACTATCGTAAATATG
TATTGACATTGTCGTAAATTAGTATATAAAATTATCGTAAATGGTGGTGGCTACAGAATAACTTATTCTGTAGCTAGCTATAGAG
TATACTCTCCCTATATATATATATATATATATATATATATATATATATATATATATATATATATATATATATATATATACACACACA
CACCGCAACAGGGCAGACTCGGATCCGATGGCCATTCTAATAGTATTGGCCATCTACATGCACAGGAACAGGTTGTTCTTATT
TGGATCCTAGCCGCCACTACATGCATACAGCGGTGCAGCATGTGTGGTCACGTGGGCGGCGGGCTGCAGTTATTACTACCCA
TGCACATATATATCACAATTAACCACAACAGGTCGGGCAGACTCGGATCTGA
>SsLRR-RLK345
CTGGGACCCCCTACTGGACGACCCGCGCACCAACACCTCCTCCTCCTCCTCGTCGTCGTCGGCGGTCTCCTGCGGGGGGAC
CTGGTACGCCGAGCGTACTGCTCGATGCGGCCGCTGCCGCCGTCCGCCTGGCATTTTGCCGAGTGCCTGCAATGACAAAGA
ACAAAACTGTTAGCATAAATAAATGACAAGTACTTGTAAAGGAACATAATAATAACAATATAGTATTACATGGCCTCGTAATTA
TCTCCGCGAATGGCAATATCTCGGGCGTGGAGTCGTCATCTCTATCACTATCGTCGTCATCGCTATCAATATTGTCGAGCTCCC
TTAAGTCATCACTGTCATCATCATCCTCATCGTCTCCATAGTCATCATCATGTACCTCCTCCTGGACAACTCCATCGCGCTGCA
TCTGCCATCGCTCAAGCATTCTTAAGTCCCTGGCATCCTGCACCTCCTCACCCTCGTCCTCATCGATTTCGTTGTCTACTTCCA
TGCCCATGAGCGAACCAATGTCGATCTCCAAGGTACCTTCGAGCCCCTCAGGTTGATAGAACTCATCGGTGCTTGGGTCAAA
GTTGTAATCGTCGTCATTTGGGAGAGGCGCTTTGCCGCGCGACGACACCAAATGCACAAGGCTCCATCCCGCAAGCTTCTTG
TCTTTTTGGCACGCCCATGGGAGGTAATAAACTTGCGTGGCGTCTCGGTTGGCCACAATGTAGACATCGTCTCCTTTATAGCT
GGAGTCCTGTCGAATCTCGACTTGACCTATCATAAGGTCTTTCCTCGTGATATCAGGATCAAACCAATGGCATTTGAATACCA
CCGGCGCTAAAGTCCCGGCACCCTTAAAGTTGAGCTCGTATATCTCTTCGACTATGCCGTAGTAGTCCTTCCCATCCGTGCCG
GGCGTACGAACTCCGCAGCACATGGTTTTTAGGTTGGGTCGGCTCTCCTCGTAGCTTCTCGTGCGAAAGCGATAGCCATTCA
CATCATAAACGGTGAATGACTTCACCCTACGGGAGAAGCCACGGCCCACCTGTTTCAATTCATCGCTCATTTCTGCATCCGTT
CGGGCCTGCAAGGGTAGGAGATCGTTACATAACCTGGACATCTCCACATGTGATAACAAGTTAGCCACCCGACGTTAGTCCA
GGTTTTAGCCACCCGACGTCGCCACTTGCTCGAAACTTTCTCAAATTTTTACCATAGCCTCCGCATGCGATGGCAAGACACGT
CGTCAAGTTTCGTCATTTTCGGACTCCGTTTCGATTTTATAAAATTTAAAAACACTTTTCACGCGCGTGCCTCGGCGTGTTCC
GACACCAACAAGGTCGAAATTTCACGCGAGGCCCCGCACACGGCCTCAACATGCTCCAAACATCATGAATATCATTTTCTAA
ATCGCCGGATTCCATTATTTCATGTACCTGCAGTTCAAATCTAGAGCTGCCGGAAAAAATCAAAGAAACAGCAAAAATCAAT
GGAATATAGCAAAATAAATCAAAATGACCCCAAACTTGGAATATATCATGTACTTACTGTAGCAAGGCAACACAAAAAAACT
GGGATCAAAAATCGAAAAAAAAATTAATTCTTTGCCGACTGCCATCAGTGGCAGTTGGCAAAGAGCCTTTGCCGACTGCCA
GGGAGGATGGCAGTCGGCAAAGCCTGCAACTTTGCCGACTGCTGACGGAGTGGCAGTCGGCAAAGGTGACGGTGTGGATG
GCGTCAACCATGGCCTGCCTTTGCCAACTGCAATTCTTTGCCAACTGCCTGGCAGTCGGCAAAGCCAGGCAGTCGGCAAAT
CCTGCTTTGCCGACTGCAGGAAATTCCAGTTGGCAAAGGGACCTCTTTGCCGAGTGTCGAAAAAAAACAGTTGACAAAGA
AAATTGCAGTCGGCAAAGAGCCAGTTTCCTGTAGTGGCTCTGTCGCTGCCAGATGTTGTCCTGCAAGGGGTGCTCAGCCCA
CACCTCGGTAATCTTTCGTTCCTCTCC
>SsLRR-RLK122
TGACTGGAACCATGTATGATGAACCACCATGTCCTTTTGATCAATAGGCACAAATGGATGGTAGTAGTGTAAATCCCTGTAAA
GCCTCTTCATCTCACCAGCATACTCTGTTACAGACTTTGTGCCTTGCTTCAAGTGCAACAACTCATGCATAATACGAACAGCT
TGCATCTTGTTTGATTTGCCTGCAAATTGACTCTCTAGAGCACCCCATACTTCAGCAACTGTGGCCATTGTCTCAACTTGTTCT
CGGACTATTGGTTCCATACTACTCAACATCCACACAAGCACTCTGTCATTAATCTGTTTAGTCTGAGCATCCATCTCCGCCTTA
CTGTCACCTTCATTTTTAACCAACAGATTCTCGTAGCCATGAGAGCTCAGTATTAAACGAGCATGACGAGCCCAGCTCATGTA
GTTTTCTGGTCCCATCAGTTTAATCGGATTAGACTCAAGAGCATACTTATAAGTCTCTAGAATCACTTTATGCTGCTCCATCATT
TTACTAAGAATCTTACACAGTTCTCCAATGCTAGAATCAACCTCCTTTTCTCCACTGCTCCCCATACTTCAAAGGGATACGGC
AATGGCAATGGAGAAATAGAAAGAAAATAGCTACAGCCTCCAGAAAGTTACTCCAGATTCCAGACGGAACAACAGCAGCCC
GGCCACCTGAGTGCCAGTCAAGTAGAAGGGTCACGTACCAACAAAGTAGCAGTAGCAGCCCCTAATCCCACGGCCAAGCCT
TTAATCACTGCTGCCACAAAGCAACACACCGCTTGGCGGTGAGCTCAAGCTGCAGAAGCACAAAGACGACCACAGTACAG
GAATCAAATCCGCGGCAACGGCGCGAATCTCAGTAATCAACCAATCAAACGGATTAGGATCCACAGGATTGACCTGCTCTGA
TACCATATTGTGAATTATTTGGTGGATTTAAGAGGGATTTGAAGAGAGTACGCCATGAACAGAGCAAATGGGGAAAAGTCTG
GATCGATCCTTTCTCGATCCTTCTCTGCCTCTATTTATATTTTTGTACATTTACAAATTAGTCCCCATACTATAATATATTGAGGA
CTAAGTACACCTACAGTTCGCATCTGTTATGTTTGTAGCCCACACGTGCTGATTTTTTGTTAGAGTTGGGTGTACCTGCCTTAG
CAGGTTTGGTGAAATCTTTCAAAGAAGTTGCAAGTTTCAAATTTTCTTTTCAATGAATAGGTGAAAGGAAACAAAGCAGGA
ATGGTGTGCAGTTAGTTTCATTGGTCATGTCGATTATAACCAATCTCAAACCAGGACCTAGTCTTAGCTGACTATTGCATGCCT
TCCTTGCAGTAGGAAAACGTACTACTCTTTTGGTTATTCCATACTTTAAGAGGGGAAAAACTCAGCTGGCCTGCAGGCTGCA
ACTAATTTTCCTGGTTCATGAGAGAGCGCAAATAACAAATTATTGTAGTTTATTATATTTCAATGTCTTAATTAGGTTGCATCTG
CTGATAGCTACTTCTATCAAGGATATGCAATTACTTCGAAAGTGGAAATCTAGGTGAGCAGTTGAGAAGGACCTGAGCAAAA
CATCATTGTCTTTGGCTTGCCATGTTAACTCTCTGAATGACACCAGTTCTACATCTCCTGAAAGCTACACTAGGAATAAGCAA
ACTTGGAACAAGTAACCTAGCTCTAGTCGAATTCCTCAGATTAGTTTTCTGGTCCCATCAGTTTAATCGGATTAGACTCAAGA
GCATACTTATAAGTCTCTAGAATCACTTTATGCTGCTCCATCATTTTACTAAGAATCTTACACAGTTCTCCAATGCTAGAATCAA
CCTCCTTTTCTCCACTGCTCCCCATACTTCAAAGGGATACGGCAATGGCAATGGAGAAATAGAAAGAAAATAGCTACAGCCT
CCAGAAAGTTACTCCAGATTCCAGACGGAACAACAGCAGCCCGGCCACCTCGCACAAAGCCCTCAAACACATAGTTGAATA
GCGAGCAACTA
>SsLRR-RLK251
TTGATTGACATAAGCAAAGGTTAAATACTTGAAGTTTTAGAACAAAAATTATTGTCTCGGTATGTTTGGATGAGCTAGGGCTA
GTTACTAGTTGGTTTAAAAATTAGTTCAAATTAGTTAAAGTTATCTAGCTAGTTGATTAATAGCTAGTTGAAGGCTTGGCTAAA
ATATTTTTCCATCTACTAGTCCTCCTATTTAGACACACTAAGGCTAATTTTAGCTAGCTAGCAGCCTAGCAATTAGCTTTTGGCC
CTGTTTAGTTCCACGAAAAAAACACTATGCGAAAAAAAGATTCCCTGTCACATCAAACTTGCGATACATACATGAAGTACTA
AATGTAGATGAAATCAAAAACTAATTGTACAGCTTGGTTGTACTTGGCGAGACGAACGTTTTGAACCTAATTAGTCAACAGT
TGGACACTATTTACCAAATAAAGCCAAAACGTGCTACAGTATTTTTTTTACACCTTTTTACACATCTAAACACAGCCTTTGTAT
CAAACATACCCATTTCAAAAAATAAAAATATGTTTTTATTGAGATCTTAAAAAAAATATAAAAACCGTTTCAAAAAAAAAACATA
CCCATATTTTAGAAAAAGACAATACTGTAACAATTGTCTCCGGTGCCGCAGTGCCGCTGCCGCACAGCCGCAGCCGGCCGGC
CGGCCACCCACTCTAGCAAGATTGCGAGACGGTGACGACATCGGATGCGGCGGCGTACGGCGGCCTCGGGCACTTCCTGCG
GCGTCGAGAAGCTGCGGCGCTGCGGAGCGGGAGTGCGGGGTTCACCATTCGCGGGCTCGCCGTCCGGGGCTCCGGGCAGC

CGGCGAGAGCCACCAGCGACCGTGAGCGACGCGACCTGACTTCACCTGAGGTGTGTTCTTCCCCGCACGTTCCATCCTCAT
TTGGGATCTGACGTTCCTGCTCGTGGTTGCGAGTCACGTTCCATCGTCTACCCGGACGGCGTCCCGCCATGTTCCCGTTCCGC
GAATCGGAATGAAGTGGGCACGTTCCCGTTCCCCGGCTGCTAAGCGCCCATTGCCTTCCCGACCTCCCGTGCCAGCCAGCA
AGGACGGCAGCGGCTTGCTGCCTGCCTGCCTGGAAGGGCGGCGGCCAGCAAGGGCGGCGGCGGCGGCTTGCTGCCTGCCT
GCCTGCCTGCTTGGTACGGGATATTCTTTCTTTATGCTTATATAAAGCACCATGTCTCTATAGTCAACGTCCTCTTTGGCTGTCT
CGTAGCAACTGCTGCCTCTAACCTCTATATAAGCACCATGTCTATACGTCCGGGTTGCCTATCTAATAAAGGCTTGCTGTCTGC
AAACATTATACAATACAATTACTATGGTGATGCTCCTCATCAAGCCTCTCTTAAGCATGCTCGCCTTCTTCCTCATTTGCCTTGC
CACATTCGCAGCACAGACCTCACTGGCAGCTCATGGAGCAATCGCCCCTCACACCCCCAACTCCGACCAGCTCGCGCTCAT
GTCATTCAAGTCGCTCGTGAGGAGTGACCCATCGGGAGCCCTAGCTTCGTGGGGCAACATGTCCATTCCCATGTGCCGGTGG
CGCGGCGTGGCGTGCGGCTTGAGGGGACATCGCCGGGGCCATGTGGTGGCTCTGGACCTCCCCGTGCTCAACCTGACTGGC
ACCATCACCCTGCGCTGGGAAACCTCACTTACTTGAGGCGACTCAATCTTTCATCGAATGGCTTCCAAGGGATCCTGCCTCC
AGAGCTCGGTAACATCCATGACCTTGAGACTCTACAACTTACCGACAACTCCATCTCTGGTCAGATTCCACCGTCACTTTCA
AACTGTAGCCACCTGAAAGAGATTTCCCTTGACGACAACAATTTACATGGGGGAGTACCCATTGAGTTAGGCTCGTTGCGTA
ATCTGCAGATACTCAGTCTTGGCAAAAATAGACTTACAGGAGGAATCCCTCCCACCATCGCAAGCCTTGTAAATCTGAAGAA
ACTATTCCTAAGATATAACAACA
>SsLRR-RLK217
GCATTGCTCTCTAGTAGTAGCATTGTTCATCTTGTAGTACTCTATCATATCAAGAATCATCTTTGTTATGCAATGTTCATCTTATT
CATGCTAATTTGGTTATTCTAGTACATTGTTCTTATATGTTCTTATGATCATATGTTCATACTAGAAATGTGCCTACCATGATCTT
GTTTATGCTAGTTCTTCATTCTAGCATGAACTTGCTCATGTTCGTTCATCGTTTGTATGCTAGTATGATTATGTTCATCGTTCAA
GAGCTAGTTTGATCCCATTAGTATTTTGGTTACAAAGTCTATATGATTAACTTATGCAAATGCATAGTTAGGGCTTTGATCCACA
CTACAGTGTTGGTTTTGTGAGCCTCTATTTTGCTCTTGTTCTTTCTGGGAGAGTAAGAGTAGTGTCTACTAGTTTAAGTGTGG
TACTTATACTAGTGTACACCGTCAAATGCGTGCATAATCCATAGGTTGTAGGGGTGGGTGGCAGGTGGTGAAATCCATGTCGG
TCCTTTGTAATCCCCCCACATTCGCTTATGGCGTAGGCGCTTGGTAGTAAGAATTTATCCCCTGGGATACATAAAACTAGTACC
GGCGGGTAACCCTGTTACGCGCCTATAAATATGGAGCTAAGTCCTTGTAACACCCCAGTTGTTTGTCACCAATTAAGTATTGG
GTTTGGGCTCTAGCATGACAAGTGCTCTGTGCCGACAGGATTTTTGCCGAGTGCTCTGTGCCAAGTGTTACACTCGGTAAAG
ACTTTTCCGAGTGTATTCAGGCCTTTGCCGAGTGTAACAGGCACTCGGCAAAGCGACGAGCTCCCGCAGTGGTTCACAGGA
AGCTGACTTCCTGGAATTCTATGACGAGATGCATATTCGAGTATCTAGGCTGCTATGGCACAACCGCAGACCAATGACCAAGT
TGAAAGGGACTATGATCTCATTTTGCATGGCCTCAAATCGTGAATCATAAACAGATTAGCGCTGTTTGCTAGCAAGTGGGTGC
AAGAACTACCCGCAGTCTTGTGGAGTCTTTTATAGATCAACTACTTTCATGCCATTCTTCCTAGCCTACAGTGGCGACGCAGT
CCCGCCGACCGATCTCGACTTTGGGTCCCCCCCAAGTATGGGTCTATAACGAAGAGACGGACGCACATAAGGATGGAAAAT
GGAAATGGATATTCGAATTATCCGATATCTGTATCCGCTGAAGCCTGTAATATGGATATCCGTATTCGTATCCGCTTTTAATGTG
GTAACTACATGGATATATCCGAATTCGTTTTTTTAGTGTTTTTCTCTAACCGATTTCAGATCTGTATTCGACAATATCCGGCAGT
ATCCGTATCCACAGAGAAAACAAGGTATCATGAACCTATTTAAAAGTTTTCTCTTCATAACGAGTAACCCGTTATTTAATTTTA
TTGTTACTAATGATGCATTGGAACCATTTAATAGTATATCTATAAATAACTTTTAAATTTCTTTTAATATGATCAGCAAGCACCAG
CAACAACTACCAAAGTACTAAATTCTTTCCAAATTATAAGATGTTTTACCTTTTCTAGATATATTGATTTTATTATGTATCTAGAC
ATAATATATATCTATATGCATAACAAATGCAACTTATCTACAAAAAGCTAAAAACGTCTTGTTTGAAATGGAGGGAGTACCACC
GGAAAAATATCTCTCTCAGCTCCATTTTTTTATTGAGCATAACGTTGGATCTAATCTGAGCCACGCACCTTAGAAAAGTAACA
TAATTGTGTATTTTTATCCAACCGTGCGCAAGGCAATGGTATGAGCACATTCGAGCCATTTTCCGCGTCTTGGACTCAGACTG
CCGAGTGCCGACGCGCCTCAGCCCCACTAGTCACTAGAAGATCCACCTAACTTTCATCCCATTGGATGCTGACCTGTTGACA
TGTCTGGAAAAGAAGACCAGTGACTTGGAAAGCCCCGAATCAAGCATGAGTCCATAAGCTACGACTACGAGGCT
>SsLRR-RLK219
TTTGTTTGGTATTATAGATGTTAATATACTTTTTCTAAAAACTTATTAGTCAAAACTAACAAAGTTTGACTTAAAATAAAACTG
AAGTAAACTATAATTTAAAGCGGAGTGAGTATTTATGAAATCAGACCTGGAGCAGCATATCCAATGGTTCCCCTTCTTGTAAC
CCAACCGCTTGATATGTCTGAGAGGTTAGTCTGGTCTTGATGCACAAATCTTGCAAGTCCAAAATCACCAACATGCGCAATC
ATGTCATTATCAAGGAGAATATTGCTTGGCTTCAGATCACAATGAACTATTGGGGATGGTTTATAATGGTGAAGATATTCAAGA
GCTGAAGCAACATGAATGACAATGTCTATCCGTTGGACAAGATCAAGACACCTATGGGTGCCTTGATCTGAGTGATGCTGGT
GTAGCCACCGGTCTAGATTTCCATTTGGTAGGAAATCAAATACAATAGCTTTGAAATCAAGACCATCAGAATCAATACTTGAG
CACACTGTCAAGATTTTCACAAGGTTCCTATGCCGAGCACATCTCAGAGTCTCACATTCTGCAATAAAACTTTGTGAAGCTC
CTCGCTGCTGAAGGTTGAGCACCTTCACGGCAACAACTTGTTCTTGTTCATGGTTCATCATTGTTCCCTTGTACACAGAGCCG
AAGCTTCCAACGCCTATGAGGTTCTCAGAAGCAAAACTATTTGTTGCATTCATCAAGTTAGTGTACGAAACCCTATCATAGTT
ACCATTGATAAGCGGTAAGGCATGTTCTTCCTTTCTCAAGTTCCTTTGTTGATGACAGAATACAAATAGTGCAAGCAGCAAG
GAGATGCCTAGAATGGCTGCAGCTATGGAGATGATCATGACAAGCTTGCGCGATCGCTTAATGGTTGGAGTGCCGTGGTTTG
AGCAAGGCGGCAATTTCAGCTGAGGGATGCCCCCACAGAGAGCAGTATTCCCTTCAACTGAAAAGGCACTAGCGTTAAGAA
ATATTCCATGTCTGGGAACTTCACCATCGAAGTTGTTGAAGGAGATATCCAGTTGTTGAAGACCATTCATTGTAGCAAGGAAG
TCAGGAATATGACCAGACAAGTTGTTTCTGGAAAGATCAAGAGCCAAGAGACCCTTCAATTGCCCTATCGAGTCAGGAATGT
TCCCTTCAAAGATGTTCCCTTTCATAATGCAATATTGTAAGATTTGGCAATCACCAAGAGAGGCAGGGATTTGCCCGGTCAAT
CTATTTCCAGATACATCAAGGGCCTGGAGATTTCTCAGGTGACCAACTTCTGATGGAAGTGATCCAGTTAGCATGTTTCCTTG
GAAATAAGCATTGGTGGAGAGTGTGGGTATGAGGAGCACTTCTTTGGTATTGGACCAGTAAGGCGATTNNNNNNNNNNNNN
NNNNNNNNNNNNNNNNNNNNNNNNNNNNNNNNNNNNNNNNNNNNNNNNNNNNNNNNNNNNNNNNNNNNNNNNNNNNN
NNNNNNNNNNNTTCTTTTAATATGATCAGCAAGCACCAGCAACAACTACCAAAGTACTAAATTCTTTCCAAATTATAAGATGT
TTTACCTTTTCTAGATATATTGATTTTATTATGTATCTAGACATAATATATATCTATATGCATAACAAATGCAACTTATCTACAAAA
AGCTAAAAACGTCTTGTTTGAAATGGAGGGAGTACCACCGGAAAAATATCTCTCTCATCTCCATTTTTTTATTGAGCATAACG
TTGGATCTAATCTGAGCCACGCACCTTAGAAAAGTAACATAATTGTGTATTTTTATCCAACCGTGCGCAAGGCAATGGTATGA
GCACATTCGAGCCATTTTCCGCGTCTTGGACTCAGACTGCCGAGTGCCGACGCGCCTCAGCCCCACTTGTCACTAGAAGATC
CACCTAACTTTCATCCCATTGGATGCTGACCTGTTGACATGTCTGGAAAAGAAGACCAGTGACTTGGAAAGCCCCGAAGTCC
ATAAGCTACGACTACGAGGCT
>SsLRR-RLK297
GAGCGCAGTACGCGGGCATGGGAAATAAAATGAAGAGAGGAGGAAGAAGGGGCCGCGTGAGGCTTGCAAGCCCAGACCT
TGGCGTTGAGTCGACCCACGCCGACGTACTATGTTCGGCGTTAACTAAAGCGACGCCGAGCTCTGTGAGGCCATGACCATGT
AAGTGGGCCCCACTGCCACGCGGTCTTACTATGACCAGAGTCCATGATGACATGGCAGGACGTCGGTGTGACCGTCCGCGA
TGCTAAGCCACAGCGTTGGATCCTATGACGCCGTTCTTTGGTCTATTTTTGAAAAATGTTTTGTCAGGGGTTTATTTGTGAAA
AGTGTTTTTGAAAAGGACCAAAATGTAAAATTTTCACTCCCAATGACTCACCTGCGCGATGCCGCTGCAGCAGCCATGCCTT
TTTCCTCTGATCCACACCTGCCTGCTCCGGCTGCAACTCCGGCCAGCTCTGCTGCCTGCCCTGCGACAGATTGCTTGGCCCT
ACCGACAACCAACTGGCCGATGGGGGCGGCGGTGCCTCCTCAGTGCCTGCACGCTAGGAGGCCCCACGAGGCAGAGGCGC
CGCACCGGCCAACTGCGTCGCGTGCGTGACGGCCGCAACCGACGGCTGTGCTCGGCAACCACGACTGGCCATGCCCTGCAT
CCTGCCGACACGGCAACTACTTCAGTGAGAAGTGCCGCCGCCGCAGCTGCACATGGGAGGAGCCCCACCGCGTGCGCGCC
GCTCGCGGCTGAGCCCATAGGGCCCACTGCTGCCTTCCTCTGCTGCGGGTGAACTGTGCTGGAAGAAACCGATTCTTGCCAT
GTGTATGGTATATTTGTAATTTGTGGTGTTCTTGACAAGTGTACGGTATATTTGCTTACTCAATTAGTTTGGCTAAAGTGCCTAT
TCTCACTCCGTGCATTACAGCAGACATTCTCACTCAGTGCATTACAGTAGATCACGAAGAAATAGAGCAAGGTTCCCACGCC
CGCAGATTTTTTTTTCTTTAGTTCTTTCTCCTAATTTCCTAAGGTGAGTAGTCTCTTTTTCTTCTCCATCAGCATATATGTGTTA

TTTCTCCTTAATGATGATTACAGTCACAACTTTGTGTTCTGCATGTGTTATTTCTCCTCAGCGCGGTGCTCCGCGACCCGACGC
GGACTCGTGCTGGTGCATGACCCCCCTGCGTGACCTCCCTGTTGCAGTGCTCCCGCTCGGCGGAGCTTCGACACGGGTACTT
CTGGAGCTGCTCTGTGGTACGGCGCCTCTTCTTCATGTGTGTGCAGGGCCCTAGGCGTGCGGTTTGGCCGCGGGCTTGCTGG
CGGCGGGGCTGCACGGCCTTGGGCGTCTTCACGTATGGGCGCGGTGCGTGCGGTTTGGCCACGGGCGTGCCGGCGGCGGG
GCTGCGTGTCCGCGGCTACCCTCCCCTCCCCTCCCCTATGGCTCTGGTCAGGAAGGGCAGTGTGTCGTCTCCACAGCTCACA
AGCGGGGTTTCTAGGCGAAAGCTCTGCTCAGCTTTTGGCTGGGCCAGCGATGAGGATCGCCATCGGGTGCATTTCCTTCTTG
AAGGCATCGCTGAAGAAGCTCCTGCTGCCTCCTCTCCTTGGTCAGTCAGGCCATTGCGGCTCTGCTCGGCGGACGATGATTA
CGTCTACGCGTCATTCACCTCCCTGGAGCATCGTCGAAGAGTTCTTTTGTCGTGTTGTAGTCTTCTCTCTCTTTTGGTGCACTA
GCAGTGGTTGAGCCACCTTTCGCCCTCGTGTTGGGGCATGTTGATGATGTTGTTGTCTTATACAAAATCCCTTGTAAAACTAC
GGGCTGTTTTGGGCCATAATATAAATTCAGGTGGGGAACGCCTCCCCCCCGGTGACCGTCAAAAAAAAATCCCGGATTCAAT
TTGGAACATTTCGTCTCTAACAGCATTCTCTGTCCAAAACAATTCTCTGAGTGGAGCAATACCTCCAGGAGCTATATTCCTGT
TTCAATATCTAATGCTTCCAAGATACAGA
>SsLRR-RLK399
GTGTCAAATTTTAGCATATTGGTCAAGGTAATGTAGTAAGCCTGCAGAAAAAAAATTGGAAGCCAAAAGCAAAAAAAAAAAA
ATATTTTGCCGAGTGTCCTAGAGAAACACTCGGCAAATAGTACACTTTGCCGAGTGTTTTTGGTTGACACTCGGCAAACCAG
CCATTTTGCCGAGTGCTAACGGAATACACTCGGCAAACTAATGACGGCGGCTAACCCCCCTCCACCTTTGTCGTGTGTATACA
TTTGCCGAGTGTTAACACTCGGCAAAATGAAACGTTTGCCGTGTGTTTTTATTTGCCGAGTGTTGACACTCGGCAAAGTGGT
ACGTTTGCCGTGCGTATTTGTTTGCCGAGTGTTAACACTCGGCAAACTAGTTTTTTACCGAGTGTGAGATTTTTGCCGTGTGT
TTTCACTCTGACACTCGGCAAACGCAGTTTTTACCGAGTGTCCACGTTTTGACACTCGGCAAAAGATAAAACACTCGGCAA
ATCTCGGGTTTCCAGTAGTGTATATAACTCTATACTCATATTTTCTATTATTTATGTTGCATATTCTTTTATAAATTGTTCTAATATT
TTGTTTAGATCTAAAATTTTTAGAAAAATCATACTTCATGTTTTTAAATCTCTAAAAAAGATTCTATAAATTTTCAGCTGAAATT
TTGCAAATCTCAAAAAAATTATGGTGGCTCCCCGTCACTAATCCCGCCCATCGCCCATGGTTGTGTCATGGTCATTACTTATTA
GCCAAGACCCACTTATAGAGCTGCTACCCCACCCCCACCTAGATAAAAAAAAATCTAGGAACCCCAACCCTGGATAACTAAT
CCGTTGTCCCAAGACTTTTGTAGATAAATATACTTTGTTCGGTCAAAGTGTTCGGTAGTTGACTAGCTCAATAGCAGTGTGAA
GAGCTTGGTGACAAACCGGTATCTATGTTCACTACTTCACTGTCAACTGAGAGCATCTCCGACAGCCTTTCTTAAACCTACTC
TCCAAATCCTTATTTAGAGAGTTATTCTAATAAAAATAGTTCTCTATATATGTCCACCCTCTAACAAGTTCTCTATATCTTGTTGG
GTACTCAAGAAAATCAGCTCTGCTCTCTTTGCCGAGCTCGAAATCAGAAAGAGGATGAAAATATTTAGAGACCTAATTGAAG
AAGCTATTGGAGGAGATTTTCTCTTTTTTTTTCTCAAAAACGCAGGAGAGCTACGCTTCATTATATTAAGAAGAAAGAGGGG
GTAAAGAACCCTTAGTACAACACACACCCAACAAAATCTTTATTTCTATCAATACGAAAGGATATAAAGGGTCCCTTGGAGTT
CCTCGTGGCAAATTTCTTAGCACCAACCTATGATTTCAGGATAACAAAGACATAAGCAGTGGTTCAAAGTGTTGTTGTTAGTC
AGTATTCTTTTGCTTAAAACAATTTATGTGAAACCCTTTGCAATCAAAATTTTAAAATATTCAGACACGGAAGGGTTTTTTGA
AAACTGTCTCTAGCGAAAGTTGGTTTGCAAATCATATCAAAATACTATCTTGGTTATTCTTTTTATGAACTGATAATTTAAGTTT
TTGTATCCTGGTTGTGCTATACTAACTAAATAAGAGTTTAGCTAACTGAGAGATAACAAGAACAGATAAAATTTATTTGCAAA
CGAGATGATGATATACGATCCAGCTCCAAATATGTCGACCTTCGAAGCAACTCATCAATTGCCAGACACCCTCCTATAACAAG
AGGCATTTTCATATGGCACAGGTAGAGTCAACCATTTAACAAGAGAGTTCAGAGCAACGAATAGTTTGAATCCAGCAAGCTG
AATACCAAAAGCATGCGTTCATCCATCTTCCTAGTTCCCACCATAAGTTGGACTATTTGTAGTTGTAGAGCAGCAAGGCACTA
CCGGTTTACCGCCCCATATATAAGCATCTTCCAGCAAAGGACAATCTGGATAAACAGCAATAATGGCCCGTCCGTGTGTCTTT
TCACC
>SsLRR-RLK267
ACCAGAAGAAAAAATATCCAAATGGACACCTCTATATTTTTTTGTTAACTAATTTATGGAGAAACTCACAACACAATCTGGAT
GGAGACATCTAGAAAGTTAGTAGAAACAAAAGCTAAACGATGGATGCAGAACTCTGTGCTTTCTAAACGTTTGAATCATATA
TTTTAGTTACAAAGGAATACCGAAAGAAAAGGCATGGCATGTCTGAAACACATTAGCAGAAGACAAAGGGAACTGTAATTT
TTTGCTGTTTGCACCCAATCATCACTTATACTGACAGAGTTATCCACTATATAGTTTTCATGATAACGTGATAACACACATCTGA
TTTTAATAGAAAATGCACTTTAGACTTGTAGCTGCTTGAACTATTTGGAGAACTTCAATTAAAATTCAGTATGATAAGCTGTTG
TACAGAAAGGTTGGTCAATGCTAGAAGATTCATCAAGTCATAGTATATACTGGGAAAGCGGTGCATCGAACTTGCAGATGAG
ATCAGTAATGATCTCTATGTATCTCAAACTCTTCACATATGCAACCGACTGAATTTGAGTTCCTTGTTTGTGGCACTTGATCTG
TTCTATTCTGCACATGAATGTGCAGAGTTAGAGGTATCCTCCCTTAATTTTTCCAACAATTCTGTACAAAATATTCTTTATTTGC
AAGTTTTTCCAGCAGCACAAAAGTAACTGCACGGACTACTGTTTTAGCAATTAGGGATGCAAGAGAATGGGTAAACATTGA
AGCACGTATCTGTAGAACACATGATGAAGCTGGCTAGGGAGTTTAACCAAACAGCCGACATATTTGGCATCCTGAGAACAGG
CATAATGAGATAGCCTGCAGCAAGCACAGATGCAGTAAAATGATGACCAACTCCTTTTAGCTGTGCAAGGCATGATTTATAAT
AGAAGAGAAAACAAACAATACGCCAAAGGGAAATCTAACTATCTCCAGCATTCATACGCAGTCAGAAGCACTGTAGCGAAG
TCATTTCATTCAGGAGGGAATTCCATCGATCTAGCAGGGATCGAGAAGGGAAAAATTCAGTGGTAAAGAGCATATACACGTA
AATAGGTACATGTGGAACTAACATAAGCTATAAATTGTGGTCTCCCGAGCAGTATTTTTTCGACAAGGACCCCAAAACAATGC
ACTGGTTAAAGAGAATGTGTTAGATCTGATACAAAAGCTTAAAAAACTGAACTTAGGCAGGAAAGCGCCGAACATAGAGAC
CTGGACACCTAGTGTACGGCAGCGGCAGCAATGGCCTGGCTGAGTGGCGACGCGGAGCTCACCCCATCGTCGCGTCCTCCG
TCACCACGGACGCAGTAGGAATGAAGGGGGACAAAAAGCGAGGGCAAGAGAGAAGGGACCTGGGAGTTTACCTCGTCGT
CCATGCAGCGACCGCTGTTGTCGTCGGCAAGCGTGTCCATGCGGGAGCAGGGCAGCACTGCTGGGGGTGGGGTCGGCGCT
GGGCGGTCCCGACCTCCGGCACGCCATCCAGCACAGGCACCTAGGGTTGCCTGTAGCACATGAGCACACGTAGAGCAGAG
AGAGAGGGCATCAGCGAGGGGCAGTCACACCTGCGGTGACCGGCTCGCCCACTCAGGCCATGCCGTGGCCGGGGACTGGC
ATCGGCGGCGCCCACAGGCAGTGCCGCATCGCAGTCCGGCGCCCCAGCAGCGTTCGCCGCCGCATCGGGAGGGCAGGGGA
GAGAGAGGAGGAAGGGGTCGGGAGAGAGGAGGAGGCGGAGGCGGAGGAGGGGGACGAGGTGGAGGTGAAGGATGCCTC
ATCCAGCGTCTTGCTGCAAAAGGCCCCAAAACTCGCTCCACCGCTGGTCAGCTCGGGAGGAGCGGAGGCGTCGACAACCT
GCGTCGAGACTTCGGGAGGAAGAGAGGGAGCCGATAGAGACGGAGCGAGCGAGAGAGGAATAGGGCGAGTACTGTACGA
ACACTAGGCAGGCAGGGCGACGGACGAGCAGACGGGACCAGGAC
>SsLRR-RLK269
CCGATACCACTTGTAAGGTCCAAATAGGCTAGAGGGGGGGGGTGAATAGCCTAATTAAAAACTTCTACAATAATTACTTAACC
ACAAGTTAGTACAAAAGGTGGCGGAGCAAAATGCACTAGCTCTAACTAATTAAGCAAGCCACCTACACAAGTCTAGTACAA
GGCTGTTCACTAAACACAACAACAACAACAAGCTAGATAAAAGAGTAATACGAGAGAAGAGCTTCGAGTGATTATACCGCC
GTGTAGAGATGAGAAGTACCAATAAATCGATCACACAAGAGAAATCCTCGGGAAGAGAATGACACAAGAATTTATCCCGAG
GTTCACTTGCTTCCCGGCAAGCTACTCCTCGTTGTGGCGATGCACCCACTTGTCGGTTCGCGAGCTAATCAACTATCAAGAG
TTGAGCCCTCGGCGGGCGCCACACAACCGCACACAAGATGGGGATCCTCCAAGCCACGAGCTCCACTAGAGTATCCAATTC
GCGAACTCCCGCGGGAAGGATCAAGAACCCCTTACAACACTTGGTGAAGGCTCCAACAATCTCCAATTGTGAGCTCAATCA
CCTCCGCTGCTCCAAGCCGTCTACCGTGTCGGGAAACACGGAAGAGTAACAAGAAATCCGCAGCAACTCGAAGATCAAGT
GCCTCTAGAATGCAACTCTCAAGCAATGCACTTGAATCTCACTCAATCTCACTCAAGATGTGCAATCAATCAAGGAGATGAG
TGGAGGGGTTGTTCTCTAGCTCAAAGTATGTCTCTAGGTCTCAAAATGTGCAAGAGTTACCCTCAAACCCCGGCCACCATCT
ATTTATAACGAAGGGGTACGCAGCTAGCCGTTAGAAACGACGCAGCAGAAATCGCGCTGACCGGACGCTCCGGTCAGGGTG
ACCGGACGCGTCCGGTCACTACGCGACAGACGTGAATAGTGCGCGTCAGAACTAGCCGTTACCGTTGACCGGACGCGCTCG
ACCGAGCGACCGGACGCGCTGGTCACGACCGGACGCGCCGGTCCCGTGACCCACCGGACGCGCCGGTCAACACCGAGCCG
CGCACTGCGAAACACCGACCGGACGCGCTGAAGAACGGACCGGACGCCACAGTGCCCGCGTCCGGTGGGACTCCAGTAAG
CACCCTGGACGACCGGACGCGTCCGGTCACCTATGACCGGACGCGCACCGCGTCCGGTGGTCCTCGTGAACCGCTGTTCGC

CTGACCGGACGCGCCGGTCGCACAGTAACTGCGTCCGGTGCAGCGTCCGGTGCAGCGTCCGGTGCACGCAGTGACCTCCGT
TCGCCTCCATTTCTTTTCCGACTTTCGGCGCAATAGAAAATAGGCGTTTCACTTCTCTAAAAAGCGCCGACTCCCGCCTCGCA
AGCTCGGCGAGAGGGAGGGAGGAACCCAAACCCCTCTCTACCGCTTAAACTCCACCGCCTTCACAAAAGTACTAACACCAC
CAAGTGTACACCACCATATGCATGTGTGTTAGCATTTCACAAACATTTTCCAAGGCGTTATGTTGGCTCTTTACTAGATCCTAA
ATGCATATGCTCAAGACATTGATCTAGTGGCACTTGATTCCCGAGCTAACCGTGATTTCCCCTCTTGATAGTACGCTATGTATC
CTAAATCCGGTCAATCACTTCTCTACTCATCTTAGACCGGCAAAAGGAAAACCCTATGGTTATACCTTTGCCTTGCGCTCTTA
GATCCTTCTCTTCCACTTGATCATCATGTCCATCATTTAATGTGCATCTCCACCATGATCTTGCTCATCATTTCATGCACCAAAT
CATGTGGTCTTGTGGCCATCTCTTCATGCCATTTCCACCTCCATCACATGTTGCTTATGCCTAGCTTAATCACACTTGATACAAT
AGCATTAGCAACTTGGTGTCATTAATTACCAAAACCAAACTTGGGCTTTCAGAGGGGTCGTCTGTGGTAAGGCCCTTCCCCC
TTCCCGTGTTGTTTCACTGGAGCTGAACTCTTTAC
>SsLRR-RLK281
GACGGAAAACCCTCCTCCCATTTTCACAAATACGAAAACGGACCCTGCAAATACGAAAACGGGACCCATAAAAATGAAAAA
CGGAACAGTCGAAACGGAAATTTTTCAGTCCGTTTTCAACCTCCTTCGCCCCTCCACAGCCGCCCCATCCGCCGCACGGCA
AGCAGCACCGCGCATCACATCGTGCATTCGGCCGGCAACAACGCGAGTCCGCAATGGGCTCGGCGACCATGTAGCCCTTGT
CGCGCATCGAGTCCCGATTTCCCTGCGCGGTGTGTTTTTTTTTTCCTCTGGCTTGTTTGCACCTGAAATGTTCGATGCAATG
CCTCAAGACAGTTCCGCTCTTAGGGGAGGCACGAAACACAACAGACAAAACACAAGTGCTGGGCATCTAGCACAAGCAGG
CGGAGCAATTCACAACTTCACATCGAGGCACTCACAAGCAGGTGCAGCAATGCACGTCTGAGCAACAACCATAAGAGGAG
GGAGGCGGGTGGGAGGGAAAGAGATCCCTGTATGCCGCAGCCCATGAGCTTGCTTGCGATTGCGACCACAGGCTAATGGGG
ATGGGAATCGGCGGAGGAGAGGAAGGATGCTTGGATCAGAGGAATCGGTGAAGAGGAAGGAGGCATGAACCGAAGGCGC
GAATCAGCAGCGAAGAGGAGGGAGGCACGAGCTGTTAGAGACTCAGAGGAGGCACAAATCGATGGACCAGAAGGGGCCG
GAGCAGCTGGATTCCGGCGGTGCGCAGCCAAGGACCAGGTGCGCTCTCTGCGCTATCCCCTTCGCTACTCCCACGCCCCACT
GCATCGTCCCCTTTCGATTCACCGGTGCGGCGTCCGCCTCAGGCTGCTGGGTTCAAAAACGTCACGGCATCATGCTTAGGTG
ACGACTTAAAACCATGGTGATAGGTTTCATTGTGGGATGAACTAGCCCTTCTGAAGCAGGAAGCCAGGAACAGTGCAAGGA
AAACAAACGTAAAGTATTTATGTGCTATTTTGCTTCCGAAATTCTTTGTTGGATGAGCTATGTTGCACATGGTCACATATCACA
CTGGATTCCTAATGGTAAGAAAATGGATGGATAAGGGTGATCGACCTTGATGCTATACCTACCGTATTATGAACTTCCCAGGCT
AGACCAGTTAATCTAGTCCTGAAAATTCTCTTTAAGATGTGTCAGCTTCTTGTGTAATTGAACCAGAGGACTGCTAGTGTCTA
GGTTTGTTCAGTTATGATTGTTGGCCATTAGCAAAATGTTTTATAAACATTGCATGGGTTCTGGTAGTGGAACTGTGAAAACA
TACTGTAGTCTCCTGTATATATGACTCTTTTCATCTTAATTGAAAGTCAGAGCTCCTGTTGCTTACACTAAGAAAAAATACTGT
AGTCTTGTGACCAAGAGCTGTGCAAGTAACTCTTGATTTTCAGTTGAAAATGCCTCATAGGTAATTATTATACTGATGGTCAG
AAGGGCATCTTTGAACATAACAAAGGAATAATCATGCTTCTAAATTGCTATGGAAAGATCTAAAATGTCAGTTCAGAGTTTTA
TTCCATCTATCACTTGAATTATGCCGATTACTCTTGTATGTCTGGTTCATTACTTGTACTGTAGCTCGACAATTACAATCTATTTC
TCCTGGTACAAAATATATGCACTTTGTTGTTAGAATTTGATTTGATTATTTGATAAATATCATAGTTACTCACTGAGACTCTTTG
GTTGAATAGGGCACTGGCAAGCATGGAAAATTCCCCATTGACCACGTTTCTTCTGGTCACCTTTGTTGCTTTCTCATGTCACT
CCTCATCCACACATCGGTCATCCCAACTGATAAGTCTGCGTTTAGTCCAGGGCTACTTTGAGTATGACAGCAGGGGATGAATG
ATTTATAGTGGAATCTACACACCACGGGATGGATCCCAGAAAGTAAAGGAAAACACACACACACACACACACACACACACA
CACACACACTTCTTAGACGTGGAGAGA
>SsLRR-RLK268
CAATGATCACCGCAAGCTATTGTCAGCCTGAGCCTTAGATCAGTAGCTTTCTTTTATTTAACAGGCAACTTTCACCAGAGTAC
TGGCTTGGTTCATTTTTGTTACCGGTGCATTAATTAACGCTGAAACGGCAGAGTATATGGCTCTTTACAATCAGTTAATCAGGA
TGGATTTTATGAAGAAAAAAAACAAATCTGATTTGCAATATGGCAAGAAATAACTTCAGAATTAATGTTTGAACATAGTCCAG
TATAACAAATTAACAAGCACCCTTTTCGTCAAACTTGAGACTATATCTGAACTCGCTGGAGCTTGGAATACAAAGAAAAAGA
TAGGATGCTGTTGGCAGAAAAATTGCTAACTGTTTCCTGTACTTCTAACAACCCTTCGTTGAATAGTTAAATGACAATTTAGT
GACCGTGTACAAGTGTTCGTGTACTGCTTACATGTTTACTTTCAAAAGGGTACAGATCTAAGAGTATCCTTACCTCAGTACCA
ACCGCGACCAAATGGTTACCCTGAAACTGCAAACTGCAAAGAACCAAACGTTCAATTTAAGTGAATGTTCCGAGGACAACA
TGCTGTGATGTTCTCTCTTGGATTCTGTTTCCTAAACTTCAGGCATAATGCACCGATAGCCTAGATTTTCTTCTACAGCAATAG
AGGATTATTGTCACAAAGGCGCAAACGTTGCAATCAACTGTGTTCTGGTGGCTTCAGTTACAGGCTGTGCTGAAGCCTGTTG
CACAAGCAGCAGGTTGCGACTTCAGAAATCCAATTCAGCCGTCAGGGAGTTTAGCAATTGCGCATGATGCCGTTAAGGGAG
TAGATGGTTTTTTTTCCGTCTGTATGATAGATTTACTTAATCAGTGCACAAGATCTAGCAATAAAACAATGAATGCGCCCACTA
TCTAACCTCGAATGCATGAATAGTCCAAAAAAGTGTCTTTGACAATTGATAGTACTTCAAATATGCATTCATGCCACTCTCTAG
TCTCTAGCCTTCCTTTAGAGTCTAGACTGGCAAATTAAGGAGGTGTTTGGGACTACTCCGTTCCACGTTTTTCAGCTCCGCTC
CACGTTGTTTAGCCAAACGGTTTCAGCTCCACGCACTCTATTCGAGGAAAAATGGTGGAGTTGTGAGAGCACCTAAAGAGA
TACTCCACAACTAAAGTTTTTGTGGAACTGCTCTACGATGAGGTTTGTGGAATAGTCCCAAACACCGTGTTCTTGCTCGATCT
CCTAAAGAGGAAACAAGGCTCCCATGGGCATATATCGGCAAATTAAGCATTGCATCATAGATCAGTGCTTGTTTGTTTCAGGT
GACTATTTAATTTTTTAAATTATTAGCATGAAAAAAAAGGAGAACGATGCGAATCTGTAGATCAATAGAAAATTCCTTGACCAA
AGAATTGTCCTCATCCATCAATTGTTTTTTATTGGTAGTACTACTAGCTTATTTTCCTTCAACTCCTTGATGTTTGACCCGGTAG
CCGGTTATATGAAGAGTCAACTAAAGCGTGCTTGGACTTGTTACACTTGTTTAAATAGCACTTGAATCTAGAACGTTTCTTTC
AAGACAACTATAAAATTGAAATAGCATTGAAATCTAGAATATCAATTGGTTATTAGGTGATATTTTATAATCAAATATTTTGGTG
ATCATGGCGAATCAATCATTGTTAATGATTTGAACTGATAGGTTGAGTCAACTTGAAGCAGGATTAATTAAAAAAATCAAGAT
CTAGTGAATTTGTATTGTAAACACAAGATAATATAACAATATATTAATATGGTACAGACACGAGATAAAATGGTAGGACAAATT
GTGTACAGATTTCTGATCTCACATCCCTCTCATTATAGATGTTCACTTGTTCTGTTCTAGACTTTTCTCCTTGTCGGTAGGTGCA
ATCCAGAGGACAATTCATGCACAAGACGCAAACGAGAGAAGTATAAGTTTCTCTACTCTGCACACATATCTCAAAACAAACA
CATGAC
>SsLRR-RLK370
TATGTTCCACGCATTTCCACTAGAGCGGGCCAGGGATTTGCACTAACAAATATGGAGTCTTCCACTATTTATGTCTTCCAATAA
GGTATCTAGCTGCCTCCATTGCAGGCCAATAATGATTATTCAATCTACATAGTGGAAGACTATGGTGTCTTGTGGGTTAGTTAT
CTGTAGTGCACTTACATAATGGCTACTCCTAATGCATTTATTATTATTGTTAGATTGTACGGTTAGATCCAGATCTAAACGGATC
AACTAACCGAAGAAATTGCAGAAGCTTAACCAATGGAGTGTGGTGTGTAGAATGGGGTATCTTTGTTTAAGCTTTGGGGAGG
CCGAATTTCTAGCCATAGCTGAAAATGTATTTCTAGGGATAGCTTGGTTAGTGCGACCCAATGGAGGCTGCGATAGAGATGGC
TAGAAATTCACCCAGATGTGGCCTAAAACTGTTGTGTTGCTAAAAATCAATTTATGTAGTAGTGAACATAGTTATATATAACAG
GAATGAATAAACTGATGAAGTGTACAATCAGGATTTGCTTCACCACAGAGGACATGTCTACATCGCTAGCTAAAAGAGGTCA
TATATGCATCACCGATATAGGCATATAGCTCCAAAAGTTCTCTAAAATGGTTCAAAAGCATATAGCCATAGACCGTAGTGCGAG
GTCCATTGCCGTTACGAACCATGTCCAAGTTTGACCACTTTGTGTGACTGAAATAAGCATTAGCCATTTTTTCTATTAGGTTCA
GTCCCACATTAGTAATTGATAGTGGGGGAGCACAATAAATAAGACGGAGGCAGCCCTTATCTATCAGTTTAATTAGTCTCTTG
GATTGAGTTAGGCCCAAAGCCTGGGTATGTTGGGTTCCGGTGGACCTGGGCCTAGAGGCACCGGCTTGTGGGTATAACGAG
ATAGGCCAGTATCAGCTGCGCACTCAAACACTCCCGTTCATGGAATATCAGTTTACAGGTCCTCCCATCCTAGTTGGGCGTTC
CCCTTAAGACGAGGGTCCCCGAGTACCATAATAGCGACAACATATGGTCTACACCAAAGTTTTGGTTCTCAATGCTACCTATA
AACTTTTAGTTGACAAGTTCTTTATTTAAAGTCGTGAGTCCTAAATATGTGTTTCAAATTTTTAGATTTCGAAATTTAAAATTTA
AAATTTAAAATTTTCAAAGGACCTTGATTGTTCACATGGTCAATACCATATTTGCAGTGGTCGATGTGTTCTATAGCTTTGTAG
TTGACAAAGTTTTTTTATTTGAAGTTGTTTTGAGTCCCAAAAGTTCATTTTAACTTATGTTTTGAGATTTATTTTTTAATTTTCA
GACAATCTCAAATGGGAGACATGTCCTAAACCAAAAGTTATAGTTTTTGACACGATTTAAACTCTGTGGTTCAAACTTTTTGT

ATTTCAAATTATTTCGTGGTCAAGATATTTAATACAAGATTACAAAATGCTAATAAAAAATAACATCATGTTACCCACAAGTGA
GTATATAGTTTAGTAGTGGAGATGGAGATGTGTCATGTGAGACAGATTATGGGTTCAAATCTTAGAAACCACTCTTTTTTTTT
GTCCTCTAGAAGAGGCGGCCGGCTTGCCTGTAAAGAAATGCTTCTCGCAGCAGTGTACATAGTGCAACTGAACATACACCA
CAGAGGACATGGCTGCATCTCTAAACGAGGTTATTCATCATTGATGTAGTTACAAAAGCTCCCTAAAACGGTTCAAAAGTAG
TTTTGGGCCTATAGCCGTGTAGTGGGCAGGTCCATTGCCGTGTTGGGTACGGTGTCTGGCCACTTTGTGTGACTGAAAAATA
AGCACTACTCATTCTCCCATTCCTGGAACTTTAATCGTGCTAACTGCTAAGGTGGGAGCGATCTAGCTCTATAATAAATATAGA
CTCACATTAGCTTCACAAGCACCACGACTCAGAGTTTGCATTCCAAGTAGTAGGAAGCAGCAGCAGCGCAGCGCCGTAGCT
AGA
>SsLRR-RLK331
TCGTGAACAATACACGGAAACTAGCAGGAGAGAATAATAGTCCAGTCACATATCCATTACATATCTACATTTATGAAATCACTA
ACCAACTGATGCAGACGCACATCAGACTGAGACAGCGGCCGATCAGCCAGATGCATGGCTCAAACACGGTCAAATTGAGTA
CATCATCCAGAAGCAGGTGTTGAAAATTCGTATATAATTGCACTGTGTCAACGTTCTCAGTGGAGACTATAGACCAGTGACTC
TTCCCGCGATCATGTTGAATAATAGCTTCTACTGCTGTAAATGCATAGGTTGACAGGCTTGTATTTCGTGGAAGAAGGCAGTA
GCACAAGATAAAACCATCTATTCTAAAGCTGCATTCATCGTGCATTGCAATAATAGGCATGAATAATTATCCAGACATTCATAA
CAATGTCTCGTACCATGCTGCAGCTGCTTGCTTTCATCTCCACTACTGACGCAACATGCTCCTTTCGTCTAATAATGGCATAGA
GAAACCTGGGTCATTAAAGTGGCATTAGGCCAATCTCAATGGAGAGTTAACAGAGTTGTTTCCAAGAATGCCACCTCAAATA
AAATGCAATGAAACTTCAACGAAACAACCACTCTCAATGCATAGTTACATTCTATAGTTTCATAAATATTTAATTCCATGATTT
ATTGCGAGTTGATAACTGTGCAAAGAGAGTTTCATCCACAACTCATTTCTTCTCTCTCTTCTTAAATACGGTGCAACATCATC
AAAAATTCCTTCATGACAGCTTATTAAATGCGAATGAAACTTAGATGAAACTTCCACTTAGAGCAAGGCTAATAATACAGCCG
GTTGCTGGATGTATGAAAACTTATAATTAATCTATCAGCCCACTCATATAATAGTTAGGTAGCTTATAGCCCACTTGGTTGTCTC
ACATAATTTTTTGGTTCTTGTGCATAAGCTGGCTGTAAGCTTACAATCCGCTTCTCTTCTCTCTCCTCCCCTTTCTCCTCCACGT
CAATATTCAGTCCGCTTACAGCCCCTTATTATACTGGCTCTTAGGGGGTGTTTGGATCCACTAACTAAAGTTTAGTAGGTGTCA
CATTAGGGTGTCGCATGGGGTATTCGGATACTAATAAAAAAACAAATTACAGAATCTGTCGGTAATCCGCAAGACAAATTTAT
TAAGCCTAACTAATTCGTCATTAGCATATGTTTACTGTAGCATTTTATTGTTAAATCATAGACTAATTAGGTTTAAAAGATTCGT
CTCGCAAAACAATCTCAATCTATATATTTAGTTTTGTAAATAATCTATATTTAATACTTTATGCACGTGCCCAAATATCCGATGGG
ATAGCGACCAAAGTTTAGAAGAAGGAACCAAACACCTCTTTAGACTGGCCTTAAGCATATTATTGGTAGAAGAAGATCTACA
ACGCATAAAAAATTGCACCACAGAGAGTGCATCCCCATGTCTTCCGCCTATTACTGCGTCCCACTTGTCCGAGGACGACCGC
TTACTGCCGGTCCAGCTGCTGCATCTCGACGGCCATGTAGACATCGATGTCGCGACCAATACACGGAGACTAGCAGGGAGA
GATTAAGAGTCCAGTCCAGTTACATATCCATCACAATATGAAATCATTGACCTACAGGCTACAACTGATCCTGACGCACATCA
GACTGAGACAGTGGCCGTTCGGCCGGATGGCCCCTCAAACACGGTCAAATTCAGTACATCTTCCAGAAACTGTGTCAACGT
TCTCGTCGGATGAGACTATTGACCAGTGACTCTTTGTATGGAACATGTCGGCTTCCATGTTCCATTGCTGACTTGCTGTAAAT
GCAGAGGTTGACAGGCTTGTATTTCGTGGAAGAAAACAGTAGCACAAGACAGAGAAGCTAGATTAACAGGGAAGCTAGAT
TTTCTTTTTAAATTAAAAAAAAGGCATAGAGCTCTATATAACATCTTTATATACGATTATCCCAAGGCAGAAGAATTGCCCGCA
TATCC
>SsLRR-RLK318
TAGAAACTCTCTATTCCGTTTTTTTCTTTAAGAAAAAAAACGAGATGTTCAACTGTTGGGGAAAAGAAGGGGGAGACGAAA
ATTCATTTTTTTATATTAACTGTAGTTGACTTTTAAAAAAATTGCCCCCCCCCCTTCCATTTGCTTCACCCTCCGTCGCTGCTCA
AGAAATCCTCATTTCTAGAGGTGGATGGAAATAGTGCCGCCTCTAAAAATAGGGGCATTATAGAGGCGGCTCAAAACAAGTG
CCTTTACAAATGTATTTCTAGAGGCGGCTTAGATTGAAAGGCCACTGTAGAGACCGCTGAAAATTGAGCCACCTCTATAAAA
AAGCCTCCCTTGCTAAATTTATAGAAAATAGTATTAACATCTACATCTCCAAATAGATTTACTATAAAGTATATTCTATAATATAA
CTAATTTAATAATACTTTTTTATATACAGATGTCACTACTTTTTTATAGATTTGGTTAAAGTTAAAAATATTTGACTTCTAAAAAT
GAGAATTATATATTTTTTCTTGGATAGAGGGAGTAGAGAACTAAACATGACCAAATACTCAATTTTGCCGGATGGCCATGCAG
TTTAAGATGACCCGAATTTTTTGGTTAACTGAAATTTAAGAAGATGAAAACCCAATAGTTCGGTACTTCGGTTTGGTCTAACC
GAATGACCTGGAACTTGGTGCGAGAAATCATTCCAGGATAATAGGACATTTTGTTATAAGAGCATCCGCAATGGTTGGAGCT
AGTTAGTATAGCTCTAAGCCAACTTCTATTGTGCTAACTTTTAGCACATATAGCTTTTAACATGGATAGTTCCACGTGACATTCT
CATATCACCTTAAAATGTGTATAAGAGCTTAGCTCTTGATTAAGAACTAGCTTTTCTCTTTCTTTTCAAATATAAGAAAAATACT
AGAGCTAGCTTAAAAGCCATTTATTGGAGCTGCCGTAATACATGAGTCCTATTCCCGAGCTTGCGTAGTTGACCGAGACTTTT
TGTTTGAAATCTCTCATTTTCTTTGCTTCGAATCCTCCACTATTTAATCTAAAACAGGCCAAACCAGATGCTTATTTCTAAAAA
GAAGTGCATGATAGTCATACTACAAATCATCACTTGCTCTTGTAGTCTTGTTGATAGGTCAAATGCATGATACTACACATCATC
ACTTGCATGCGTTCAAAAACACATCATCACTTGCAAATACAAAGTTTGGGCAAATGATCCTCTCCATCGACTACAAGCTTTG
AGATATCTGGTGTTGGAGGAGCTCTCTCATTCACTTGATTGCGGTGGCATTTTACTCCTCAACAAACATTATTTTGAAAAAAA
AAACATGACTAGTACATGAGAAAGCAGTGATATAGTGCCTATTAGCACTACTTTGTCAAATAATTTGTTTGTTATATTTTTAAG
CTGTCCAAATTGTCTGCAAGCTCCTTTATTGTGTGTACATCAGGAGCATAGAAAATCTGGGCCATTCTACTTTGGTCAAACAA
TTCACATCATATTTTTAGGAAACGTGTTGACTGAGCTGTCCAAATTGTCTGCAAGCTCCTTTATTTTGTACATCAGGACGCATA
GAAAAGCTCCTTTATTGATTCTTTGCGTCAAGATGTTGAACGGTACTATTGAAGAAGTAGAGACTTGTACATCAGGGTGGCA
ATGGCATGGCACTCTATATAACCTCACCATATACGGTTAGCCCAAGGCAGCAGGGTTATCTACACATGCATCCATCCATATATG
CATAGAAACAATGTCTCACTCTCACATAGCCATCCACAGCAATTCCATCCTGCTCCTGCTTGCTTTCATCTCCATCCACTTCCT
TGCCTTGTGTCAATACACATCACCCGCCGCACTGAATGAATCCAGTGCTCTCCTCTGTCTCAAATCTCAGCTCCGTGACCCAT
CAGGTGCATTGGCCTCATGGAGAGACGACTCCCCTGTGTTCTGCAAGTAGCATGGGGTGACATGCGGCAGCAGACAGCTG
>SsLRR-RLK347
GACGAAAATTCATTTTTTTATATTAACTGTAGTTGACTTTTAAAAAAATTGCCCCCCCCCCTTCCATTTGCTTCACCCTCCGTC
GCTGCTCAAGAAATCCTCATTTCTAGAGGCAGATGGAAATAAAGCCGCCTCTAAAAATAGGGACATTATAGAGGCGGCTCAA
AACAGCAAGTGCCTCTCCAAATGCATTTCTAGAGGCGGCTTATATTGAAAAGCCACTGTAGAGGCCGCTGAAAAGTGAGCC
ACCTCTATAAAAAAAAGCCTCCCCTTGCTAAATTTATAGAAAATAGTATTAACATCTACATCTCCAAATAGATTTACTATAAAG
TATATTCTATATAATATAACTAATTTAATAATACTTTTTTATATACAGATGTCACTACTTTTTATATAGATTTGGTTAAAGTTAAAA
ATATTTGACTTCTAAAAATGAGAATTATATATTTTTTTCATGGACAGAGGGAGTAGAGAACTAAACATGACCAAATACTCGATT
TTGCCGGATGGCCATGCAGTTTAAGATGACCCGAATTTTTTGGTTAACTGAAATTTAAGAAGATGAAAACCCAATAGTTCGGT
ACTTCGGTTTGGTCTAACCGAATGACCTGGAACTTGGTGCGAGAAATCATTCCAGGATAATAGGACCTTTTGTGATAAGAGC
ATCCGCAATGGTTGGAGCTAGTTAGTATAGCTCTAAGCCAACTTCTCCTATTGTGCTAACTTTTAGCACATATAGCTTTTAACA
TGGATAGTTCCACGTGACATTCTCATATCACCTCAAAATATGTGTAAGAGCTTAGCTCTTGATTAAGAACTAGCTTTTCTCTTT
TCTTTTCAAATATAAGAAAAATACTCAGAGCTAGCTTAAAAGCCATTTATTGGGGCTGCCGTAATACATGAGTCCTATTCCCGA
GCTTGCGTAGTTGACCGAGACTTTTTCTTTGAAATCTCTCATTTTCTTTGCTTCGAATCCTCCACTATTTAATCTAAAACAGGC
AAACCAGATGCTTATTTCTAAAAAGAAGTGCATGATAGTCATACTACAAATCATCACTTGCTCTTGTAGTCTTGTTGATAGGTC
AAATGCATGATACTACACATCATCACTTGCATGCGTTCAAAAACACATCATCACTTGCAAATACAAAGTTTGGGCAAATGATC
CTCTCCATCGACTACAAGCTTTGACATATCTGGTGTTGGAGGAGCTCTCTCATTCACTTGCTTGCGGTGGCATTTTACTCCTCA
ACAAACATTATTTTGAAAAAAAAAACATGACTAGTACATGAGAAAGCAGTGATATAGTGCCTATTAGCACTACTTTGTCAAAT
AATTTGTTTGTTATATTTTTAAGCTGTCCAAATTGTCTGCAAGCTCCTTTATTGTGTGTACATCAGGAGCATAGAAAATCTGGG
CCATTCTACTTTGGTCAAACAATTCACATCATATTTTTAGGAAACGTGTTGACTGAGTTGTCCAAATTGTCTGCAAGCTCCTTT
ATTTTGTACATCAGGACGCATAGAAAAGCTCCTTTATTGATTCTTTGCGTCAAGATGTTGAACGGTACTATTGAAGAAGTAGA
GACTTGTACATCAGGGTGGCAATGGCATGGCACTCTATATAACCTCACCATATACGGTTAGCCCAAGGCAGCAGGGTTATCTA

CACATGCATCCATCCATATATGCATAGAAACAATGTCTCACTCTCACATAGCCATCCACAGCAATTCCATCCTGCTCCTGCTTG
CTTTCATCTCCATCCACTTCCTTGCCTTGTGTCAATACACATCACCCGCCGCACTGAATGAATCCAATGCTCTCCTCTGTCTCA
AATCTCAGCTCCGTGACCCATCAGGTGCATTGGCCTCATGGAGAGACGACTCCCCTGTGTTCTGCAAGTGGCATGGGGTGAC
ATGCGGCAGCAGACAGCTGGCATCTGGATCTCGAATCCGAGAACATCGCTGGCAACATATTCCTTGAAAGGATCCACAT
>SsLRR-RLK348
GCTTGAGCTAGCAAGAGCTAACCTTGCACGCACAAGACTCGAGAAAAGTTTGTTCCAAATCCAAGTGCCTGGTTCTCGCTC
GTTATCCTGACTAGACAAGACAGCACAGTACCAGCCAAGAACCCAAGCACGCTCTTGCTCTCTCCATGTCAGGCTGGTCAC
ATAATGCCAATTCATAGGCCTATAAAACTATATTTTGTGTTGATTATTAAATCCACGCTACTGCAATTCACTGGTTTTAATTTTAA
AACACATTGCCATAAGTTTTGAAATCAGAAATATGCCATTTGCTTTTGTTTTTGGCGATATATATACCACTACGTAAATTTCTCG
TACGGTTCTACCATACGTGGGATTGTAAATAATCCAGCCTGCTCACCTCAGTTAAATGCATTAAAATTTCAAGAAAATAGTTTC
TATAACACATGAAAAACCCTTTTGTGCTCATGATGAATACTACCATTATATTGCTCTTGCATCTATCTTCTACAAGGAAAAAAA
AAACCTAAAATGGCAAGTCTTGTTTGCAAGGCTGTTCTCTGGGTGTACCTACAGCATGCCCTGCCCTAATCAAGGAGAACAT
TCTTTGCAACAATTATAGCCCACCTACTCTGCTCACCTCCCACCGTGTCATCTGTACTACGTCTTGCAGTTGTCATGCACGCCT
AGCTGGATATGTGCACACACATCAACTCCTAGCCTTTCTTGCTCCCCTCCTCCAAATCAGTAAATGCAGGATTTTTTTATTTA
GGTTTTTCCTTTCCAAACAAAAATTCAGCACAGCTTGATGCCAATTTGTTGCTTGCCAACTCATTCCAATACACTCTTATGTTT
TCAGCCATCATTTTGGTTAATTTGAAGAGTATGATGATTATGTCTCTCTGGACACTCATCACGGATTGAAGAAAAAAAGGGGT
TAATAAGGTTCATGTTTCCTCCTCCTTATAAGCTTGACGGTCTATCTATTAGTGCTGTGGTACTACCATGTCAACATGATGTCAA
TCAAGTAAAGAAAATGTTGGCTAAATACTGTACTTAAGTGCCACCCGCAAACTCCAAAGTATCCAAGGAAGGAATTGGAGG
GGGTCCCACGCTCAGATGCAGCACAGGGACCCACATAAAAGACCCCAGCCCCACCTGTCATCATGATTGGCCCAGCTGCCT
TGTGGGTCTGTTCCAAGCTTAGAATTCCACGTGGACAAGTTGTCGCGGCCGCTTCTATAGTGTCACCTAAATANNNNNNNNN
NNNNNNNNNNNNNNNNNNNNNNNNNNNNNNNNNNNNNNNNNNNNNNNNNNNNNNNNNNNNNNNNNNNNNNNNNNNNNNNN
NNNNNNNNNNNNNNNNGATCAATGTTACAATGACTTTGTCGGATGAAAAAATGACTAAAATAAAAATTGTAGATCTTAATGAG
TTGTACAACTTGTATGTTCATAAACTTTTCAAATAAGATCATTTACTCTTTGAAAATTTTGTTTCAAGTTGGCATTTTTTTGAAA
TTCAAATTTTGAATTGATAAAATAAAGTCACATGGAAAGGTGACCAAAATGAAAGGTGTAGATCTTAAAAAGTTATAGAAGT
TTGTAGTTGACAACTTTTTTATTTGAAATCATTTTGTCATAAAAACTATGTTTAAATTTCTTAAATTTGAAATTTAAATTTTTTG
GAAGAAAAAAAGAGTGTAATCGAGCCCATTACTATGCAGGCCAATAGCCCATCTATAATGGCCTGCTCAGCCCATGTCATCCT
GACCCTCGATTCATGATCTGATGGCCTTCGTTGCGCCCATGAAACATAAAAATTAAAGCAGCTGAAACCCTAACCCCCACAG
CTCACACGGAGGCACCGCGTCTCCACCACCCCGCACCGCGCTCCCCACGCGCCGGCCGTGCCCTCTCCGCAGCTCCGCACC
TCATCTTCCCCACAAGCCTGCACCGGGCCCCTCGCCGTGACCCGCATCCCTGCACAGCGACCTCTCCCTACAGCTCTGCGGT
GCTCCGGCATGGATC
>SsLRR-RLK349
AGTTTGCATCTAAGTCACCAAGATAAATCAAAATAACCTCATACGTTTTTGTATAAAGTACCCACATCTCCCATTATGACAAGT
AATTCAACCCCCCCTAAATTTGCATCTAAATTATCCAGTTATGTCATTATGAAATATAATTCAAAATAACCCCCTAATATTTCATT
TAAATTATCCAACTTTGTCATTATGAAAGATAACTTAAAACAATCCTCTAACTTTATATCTAAATTACCCATGTCTACAATTACA
AAAGATATTTAAATATACCTTTTGATTTTTATATATGACCCACCTTTATCATTATGAAATACTCCTTGCAGAAAAGAATGCAACT
ACGGGTTGCATGCCAGCATGAATGGTTCACGTTTGACCAAATGTCTAGTAAATACTATTCATATTTGTTGCTCCAAAATAATTT
ATTATGAAAATACATTTCATAGTTAATCTAATGATATTTATTTGATATCATAAATATTGATACTTTTTTATCTATAATTGATCGAACT
TAGGATACTAAACCATGACACTGTGCTAGAATTGTATTCTTTCATGGACGGAGGCAGTATAAAGCAAGTTAAACTCCTAAGTT
TGCATTGAACCTACCCGTATATGTTGTTGTGAAGATAATATAAAATATTACTTTTATATTTCTATCTACCTCATCCATTATAAAAA
ACTAACCCAAAATATATCTCAAATCTACATGTAAATACTTATGTATATTATTAAAAAATGAATATCCATAAATTGTGATACATATA
TGTATTTCTAAATCAATGAATTATGCAACACTAAAATTAAATAACCATAATATTATGTTCTACCGTGTATTCCCTCTGTCCCAAA
ATACTTGACCTTATAGGTGTGGGCATGGAATTTTTTTAAGAATGAAAAAGGTGTGGTTCACAGAAAACAACAAGGGAAAAG
ATAGAAATAGAGATGTACTGAGAAAAGAGAAAAATGTATTAAAAAGGAGAAAGGTGCATCGAGTGCTGTAAGCTCAAATAT
GTTGGGACAAAATTTGAAAGCGATAAGTTCAAGTATTTTGAGACGGAGGGAATATATCAAATAACATGTATACATTAGAACAA
ATATTTATTTTAATAACTTACTGTCGCCCCTCATTTTGATTTAGACCCGTCCATCTTCTCCTTGAGGAAGGCGTGTTCTGCCGTC
CCCATCGCGCGCTTGGTCTCCTCCTCAAGCTCCTTGGCAGTAGCGTTGTGCTGCAACTTCGCCTTGGCCGCAGCTCGCACCT
TACGCAGGATCTTGTCGGTGTGAATCTCTTTAGCCTCTTTGCGTCGGGCTTGCACGTTTCGAACCCTCCTCTATTAGTACTTAT
TTCTAAAAAGAAAAGTGGATGCTTATTTCTGAAAAGAAAAAGTGCATGATATTCATACTACATATCATCACTTGCTCTTGTAGT
CTTGTTGATAGGTCAAATGCATGATACTATACATCATCACATGCAAATACAAAGTTTGGGCAAATGATCCTCTCCATCGACTTC
AAGCTTTGAGCTTTCTGGTGTTGGAGGAGCTCTCATTCAATTGTTTGCTGTGGCATTTTACTCCTCAACAAACATTATTTTGG
AAAAAACATGTCTAGTACATGAGAAAGCTGTGATATAATGCCTATTACCACTACTTTGATCAAATAATTTATTTGTCATATTTTA
AAAATGTGCTGAATAAGCTGTCCAAATTGTCTGCAAGCTCCTTTTTTTGTGTACATCAGGCGCATAAAAAAACCTGGGGCATTG
TACTTAGGTCAAATGATTCACATCATATTTTTAGGAAACGTGTTGACTGAGCTGTCCAAATTGACTGCAAGCTCCTTCATTTTG
TACATCAGGGCGCATAGAAAAGCTCCTTTGTTGACTCTTTGCGGCAAGATGTTGAATGGTCCTATTGCTGAAGAAGCAGAGG
GTCACAGGCACAAGATAACACCATCCATTATGAACTGTGAAGTCAGATTCATACAGTGCAATAGGCA
>SsLRR-RLK337
CACCATCCGGCGGGCTCGGCGAAGCCCCTCCTCGGGGGTGAGGGTCTCGTGCGACATCCGGCTCCCCTCCAAAGACGCCTC
CGGGGTCATCTGGTCGAGGCGCAGGCGGCGTCGGCATAGCGGCATCACCCGACGACGGTGGAACGCCGTCGCTACTGCCGC
CGCCGTCAGGCCTCGAGCACGAAGCTGCTGCATGCGCGCGCCAGCAGTGGGTCCAGGCGCGGCTTCTCGACGTCCGGCAC
GCCGTACATCCAGTTGTGCTCCCGGGGGTCAGCACGCGGCCGGTATACCTCGGCAGCTGGTTCTCGTCGTTGCGGAGGTAGA
ACCAGCCCGTATGCCACCCGCTGTTCGAAGAGATGAGGCGCGCCGGGATGTACTGCTCCGACCGACTCGACCTCAACTGGA
GGACGCAGCTCCCAACTCGCACGGCGTAGAAGGCGCCCTTCGCTCCCGCGTTCCGCGCGAAAAGCTCGGCCTTGAAAAGG
TGGAGCCACAGGTTCCACTGCGGCTCCACCCCGAGGTATCCCTCGCAGACCGCCGCGAAGATGGCGGCCTGCGCAACGGCG
TTGGGGGCGAGGTGGTGGAGCTCCACCCGGTAGTAGTGGAGGAGCGCCCGCATGAAGGCTGACGACGGGATGCCGAACCC
CCGCTCGTGGAAGCTTACGAAGCTCACCACGTAGCCTTCCGGGGGGGCCGGCTCCGATGGGTCGACGGCGGCGCAATCCAC
TCCGGCCGCGACCATCGACTCGTCTTCGGCCGCAGCAACCATCCCTCCGCGACCAGCTCCTCCAGCTTCTTCTCCGNNNNNN
NNNNNNNNNNNNNNNNNNNNNNNNNNNNNNNNNNNNNNNNNNNNNNNNNNNNNNNNNNNNNNNNNNNNNNNNNNNNNNNN
NNNNNNNNNNNNNNNNNNNTGAAAGCGATAAGTTCAAGTATTTTGAGACGGAGGGAATATATCAAATAACATGTATACATTAG
AACAAATATTTATTTTAATAACTTACTGTCGCCCCTCATTTTGATTTAGACCCGTCCATCTTCTCCTTGAGGAAGGCGTGTTCT
GCCGTCCCCATCGCGCGCTTGGTCTCCTCCTCAAGCTCCTTGGCAGTAGCGTTGTGCTGCAACTTCGCCTTGGCCGCAGCTC
GCACCTTACGCAGGATCTTGTCGGTGTGAATCTCTTTAGCCTCTTTGCGTCGGAGCTTGCACGTTTCGAACCCTCCTCTATTA
GTACTTATTTCTAAAAGAAAAGTGGATGCTTATTTCTGAAAAGAAAAAGCGCATGATATTCATACTACATATCATCACTTGCTC
TTGTAGTCTTGTTGATAGGTCAAATGCATGATACTATACATCATCACATGCAAATACAAAGTTTGGGCAAATGATCCTCTCCAT
CGACTTCAAGCTTTGAGCTTTCTGGTGTTGGAGGAGCTCTCATTCAATTGTTTGCTGTGGCATTTTACTCCTCAACAAACATT
ATTTTGGAAAAAACATGTCTAGTACATGAGAAAGCTGTGATATAATGCCTATTACCACTACTTTGATCAAATAATTTATTTGTC
ATATTTTAAAAATGTGCTGAATAAGCTGTCCAAATTGTCTGCAAGCTCCTTTTTTTGTGTACATCAGGCGCATAAAAAAACCTG
GGGCATTGTACTTAGGTCAAATGATTCACATCATATTTTTAGGAAACGTGTTGACTGAGCTGTCCAAATTGACTGCAAGCTCC
TTCATTTTGTACATCAGGGCGCATAGAAAAGCTCCTTTGTTGACTCTTTGCGGCAAGATGTTGAATGGTCCTATTGCTGAAGA
AGCAGAGGGTCACAGGCACAAGATAACACCATCCATTATGAACTGTGAAGTCAGATTCATACAGTGCAATAGGCATGGCGAT
CTATATAACCTCATCAAATACGGTTAGCCCAAGGCAGCAGGATGATCTACACATCCATCCATCCAACCATACATGCATAGAAA

CAATGTCTCACATAGCCACCCACAGCATTTCCAAC
>SsLRR-RLK350
TTTGCATCTAAGTCACCAAGATAAATCAAAATAACCTCATACGTTTTTGTATAAAGTACCCACATCTCCCATTATGACAAGTAA
TTCAACCCCCCCTAAATTTGCATCTAAATTATCCAGTTATGTCATTATGAAATATAATTCAAAATAACCCCCTAATATTTCATTTA
AATTATCCAACTTTGTCATTATGAAAGATAACTTAAAACAATCCTCTAACTTTATATCTAAATTACCCATGTCTACAATTACAAA
AGATATTTAAATATACCTTTTGATTTTTATATATGACCCACCTTTATCATTATGAAATACTCCTTGCAGAAAAGAATGCAACTAC
GGGTTGCATGCCAGCATGAATGGTTCACGTTTGACCAAATGTCTAGTAAATACTATTCATATTTGTTGCTCCAAAATAATTTAT
TATGAAAATACATTTCATAGTTAATCTAATGATATTTATTTGATATCATAAATATTGATACTTTTTTATCTATAATTGATCGAACTTA
GGATACTAAACCATGACACTGTGCTAGAATTGTATTCTTTCATGGACGGAGGCAGTATAAAGCAAGTTAAACTCCTAAGTTTG
CATTGAACCTACCCGTATATGTTGTTGTGAAGATAATATAAAATATTACTTTTATATTTCTATCTACCTCATCCATTATAAAAAAC
TAACCCAAAATATATCTCAAATCTACATGTAAATACTTATGTATATTATTAAAAAATGAATATCCATAAATTGTGATACATATATG
TATTTCTAAATCAATGAATTATGCAACACTAAAATTAAATAACCATAATATTATGTTCTACCGTGTATTCCCTCTGTCCCAAAAT
ACTTGACCTTATAGGTGTGGGCATGGAATTTTTTTAAGAATGAAAAAGGTGTGGTTCACAGAAAACAACAAGGGAAAAGAT
AGAAATAGAGATGTACTGAGAAAAGAGAAAAATGTATTAAAAAAGGAGAAAGGTGCATCGAGTGCTGTAAGCTCAAATATG
TTGGGACAAAATTTGAAAGCGATAAGTTCAAGTATTTTGAGACGGAGGGAATATATCAAATAACATGTATACATTAGAACAAA
TATTTATTTTAATAACTTACTGTCGCCCCCTCATTTTGATTTAGACCCGTCCATCTTCTCCTTGAGGAAGGCGTGTTCTGCCGTC
CCCATCGCGCGCTTGGTCTCCTCCTCAAGCTCCTTGGCAGTAGCGTTGTGCTGCAACTTCGCCTTGGCCGCAGCTCGCACCT
TACGCAGGATCTTGTCGGTGTGAATCTCTTTAGCCTCTTTGCGTCGGGCTTGCACGTTTCGAACCCTCCTCTATTAGTACTTAT
TTCTAAAAAGAAAAGTGGATGCTTATTTCTGAAAAGAAAAAGTGCATGATATTCATACTACATATCATCACTTGCTCTTGTAGT
CTTGTTGATAGGTCAAATGCATGATACTATACATCATCACATGCAAATACAAAGTTTGGGCAAATGATCCTCTCCATCGACTTC
AAGCTTTGAGCTTTCTGGTGTTGGAGGAGCTCTCATTCAATTGTTTGCTGTGGCATTTTACTCCTCAACAAACATTATTTTGG
AAAAAACATGTCTAGTACATGAGAAAGCTGTGATATAATGCCTATTACCACTACTTTGATCAAATAATTTATTTGTCATATTTTA
AAAATGTGCTGAATAAGCTGTCCAAATTGTCTGCAAGCTCCTTTTTTTGTGTACATCAGGCGCATAAAAAAACCTGGGGCATTG
TACTTAGGTCAAATGATTCACATCATATTTTTAGGAAACGTGTTGACTGAGCTGTCCAAATTGACTGCAAGCTCCTTCATTTTG
TACATCAGGGCGCATAGAAAAGCTCCTTTGTTGACTCTTTGCGGCAAGATGTTGAATGGTCCTATTGCTGAAGAAGCAGAGG
GTCACAGGCACAAGATAACACCATCCATTATGAACTGTGAAGTCAGATTCATACAGTGCAATAGGCA
>SsLRR-RLK319
GAAGACGACCGGCGCGGCGGCCCACCATTGCCGGCGACGAGAAAACTCGCCGGTGCGAGTGAAAATGGCACTAGAGGCCA
TGGATTGGCACGGGATTCTCACAGGAAGGAAGAGGAGATCAATGGGGTTCTCACCGACGTGAATACGGAGGCAGAGATGG
CTCACGGGCAAGAATGGCAGACGGCGATCCCCTGTAAAGTCCGGTGTCGGCATGTGAGCATCGCAAAGAGCCAGAGAGAG
GGAAAACAGTGTGGGGCGACTCCGTACCACGTGAAGACGACCGGTGACGAGTCGAATTCGGCGGCGACATAGCGAGCCGG
CGGAACCCGAGCTGGCGGCGCGAGTTAGGATTGCGGCGGCTGCGAGCTCGTCTGGTGGCGTTGAGATGGGAAAGGGGGTG
CGCAGGGGGGTGTGGTGAGCTTTATAGCGTTGATTGGCGTGGAAAACGGGCCGAATTGCGGAGAGAACGGGAACGGGCGC
GAGACGCTGTGCGGCGGCGTCGGGATGAGAGCGAGCTCCTCCGTTTTCGTCGCACGGTTGGCGCTGTGGATGAGGCTGCTG
CTGCGGAAGGATAAGGGCGGCGCGAGCAGAGGGTGACAGGTTTGTATCTTGCACTCCATCCAAAAAAAATATGCAATTTTA
GGTTAAGAACAAGTCAAATAAATTTAAGTTTGATTAAATTTATAAAAAAAAATAGTACCAACATTTATATCTTCAAATAGATTTA
CTATAAAATATGTTATATAATTAATCTACTATAATGCTTATTCGATATCACAGAAGTTACTGTATTTATATAATTGGTTAAACTTGA
AATTATTTGACTTCTAAAAAGAGATTTACATTGTTTCTAGGACGGCGGGAGTAGAAAGTTACACATGCACTGAATGCTTGATT
TTCATGTCGGATGGCAACCCAGTTTAAGATGAACCGAATTTTTTTGGATACCTGAGATTTAAGATGAACTAAAGAACCAATTA
GTTCTGCTTAGTCTAACTGAATGGCCTGGAACTTGGTGTCTACTGTGAGTTAAAAAAAATGGGAGAAATCATTCCGGGCTAG
TAGGTCCTTTGGTCATAATGCATGAGTCCTGTTCCAAAGCTTTGTGTAGTTGACCGAGACTTTCTTTTTTGACTGTTTGAATAT
CACATTTTCTTTGTTTTGGATCCTCGTCTGTTAATCTATGCCAGGCAAACCGGATGCTTATTTCTAAAATGAAAAGGTGCATGA
TACTAGACATCATCACTTGCTCTTATAGTCTTCTAGATTGGTCAAATGCATGATACTCACATGCAAATACAAAGTATGGGCAAA
TGTGGTGTTTCATTATTCTCACTCGTTTTGATATTTTTCTCTCTCCTTTCCACTCTGTAGATTTCAAATCCTCTCCATCGACTTCA
AGCTTTGAGAGAACTGGTGTTGGAGGAGCTCTCTGATTCACTTGTTTGCTATGGCATTTTACTCCCCAACAAACATTATTGTG
AAAAGAACATGACTAGTACATGAGATAGCAGTGATATAGTGCCTATTAGCACTACTTTGATCAAATAATGTATTTGTCATGTTC
TTAGGAACGTGTTGAATAAGCTGTCCAAATTGGCTCCAAGCTCCTTTATTTTGTGTACATCAGGCGCATAGAAAATCTGGGCC
ATTCTACATTTGTCAAATAATTCACTTATCATACTTTCAGGAAACGTGTTGACTGAGCTGTCCAAATTGTCTGCAAGCTCCTTT
ATTTTGTACATCACAGCGCAATATAAAAGCTCCTTTATTCTGAAGAAGCAGAGGGTGACAGGCTTGTATCATGTGGAAAATTA
CACATGCACAAGATAAACCATCCATTATAAACTGTGAAGTCAGATTCATACAGTGCAATAGACATGGAGCTCTATATAACCTC
ATCATATACGGTTAGCCCAAGCCCAAGGCAGCAGGATGATCTACACATCCATCCATCCATATATGCATAAAAACAATGTCTCAC
ATAGCCATTCACAGCAT
>SsLRR-RLK344
TGATTTGTTGTGAGAGAAAAACACTGTTCCGGCTGAAAAAACAAGCTGAAAAATACGGATTATAAGCCGGATGAACGGGGC
CAGAGTTACCATGTTTCTTGTTAGTGGAATTCTCCCCACTGTTCCATTCATTGATCAAGAGTTTATAAAGGAGTTACATGACTC
TTCCACTGCTAAGACTCTTCATTCTAGACTCTTCCACTACTAAGACTCTTCATTCTAGACTCCACAAATTCTAGGCTATTCATAT
TCTAATACCCTCCCGCAGTCTCAACTGGGAGATGATCGAACATTGATACTGGATCGAAAGTTGAGGAAGACAGCAGTTAGTA
ATCCCTTAGTAAATATGTCGGCATATTGAAGGGATGTAGGAACATGTAGAACCCGCACAGCGCCAAGGGCCACACGCTCCCG
AACAAAATGGATATCGATTTCCACGTGTTTGGTCCTTTGGTGATGAACTGGGTTTGTTGACAAATACACGGCACTGATGTTGT
CACAATAGACAAGAGTAGCACGTGGTATAGGATGATGAAGCTCGCCCAAGAGTTGACGAATCCAACAAGCCTCAGCCACGG
CATTGGCAACAACCCTGTATTCGGCCTCAGCACTGGACCGTGAGACAGTGCGTTGGCGCTTTGGCAATGCTGAGAGAAGGG
GTAAGGTAATTGGATTGGAAACCATGTTTCATTCAATAGAACACAAAGAGATCTTAATACCAGTTTGAACTCTTTGAAGGTCC
ACATGTAGAGTCAACCTTTTTCTCTTCTTATTAGTTGTGTTATCTTCTTAGTTGGAGACTTCTTGGTTGTAGTTCCCAAGTTTTG
ACTATGTTATCAAAGAATGTAATTAGAAATCAGCACAAGTTAAATGTTTGGAGTAGAGAACTGAACATGGCCTTTGCGCGAT
GAACCATGGCCCAGGGAGGCCGCCCCCTAACCTCTGCTCAACAAACCGCAGCTTGGGGAGGGGGCCTGTGGCCTGTGCAC
CAAGAGCCACCACTGCGGCCTGGCAAGGATGCCGTGCCACCTTCGCCACGCCGCGGGCTGGCCGGAGCACCAGCCCGCGA
GCCTGCACCGCCGCCGCGACCGCGCCGCGGCCTGGCTCTACTGGGTGAGGCCGCCACCGCCAGAGGACACGCGTAGCTGG
TGCTGGGCACCGCCAGCCGTGGGATCCTCGGCTCCATCATCCTCTCCTCCTTCCATCGATCTAGGGTTAGGTTTTTTCCGAG
GAAGGTCACGGGTGGACTCGGCCGCTCTGGGAAGAAGGTCATGGGAGTCGGGGTGAAAAATTTGGATGCATGAGTAACAG
AGGGGTATGTTGGTCATACGGAGAAAAAACTGGCACGGTGAAAACAGTCAACCAATAGATGGATGGGTCAAATGGACAGCA
GTGCCGTATAGAGCAGAAAAAGCTCATGTGATGCTGAGTTGTGCAAAACAGGAACTTAATGATGTAGCCCAGGAAATTGCC
CCAGTAGGCAGCAGCCTTGATAATCCCTGCAGAGGAAAAGTCAGCACAAATATGGAAACATGTGCAGCATACACGCTCAGC
CAGTCAGCCCTCTGGGTTGTTACGTAAAGAGAAAAGTCAGCAAGAACAATAAAGGACGATATCATCATAACCGAATTCCGC
GGTTGGTTTCAGTCAGTGTCGGTATCATCATCATCATCATACTGTGTTTGAATTGGCTGGTATGTGAAATCACCAGGAAGAAA
AATGGTCAAACAATGGACACCAAGAACAGTCTAGCTTTAGTCAGCTACTCATGTGGCAAAAGTCACTCACAGAAGTCTACG
CACGTAATGATCATGATTCATGATCGCTGTCAAGACAAGTGTACGACGCTGCCTAAATTGAAGACTTCTTGCAGGACTGTTCG
GCACAACTCTGAAACCAGAGCAGCAGACACCATGCATGCATGCTCTCTGTTTGTACTAATCAGCACTCAGCAGTGGCAGCTT
CCCGCAGCAACACCCTTATAAGCCTGCACATGCCG
>SsLRR-RLK378
TTTTTTGAAATTGCTGATTATAATTGTCTTTCAGTACTTTAAATATCAGGACAAACCACAATCAGCTACTTCACTGCAACTTTC

GCAACTTTTGCAGCACTCGCAGTTTTTCCTGCACAGGCGCGCGGAAGCGCGCGCCCCGCCACTAGTTAATAATACTTTGATG
CGACTAAGAATTCCAACGAAAGTAAACACTTCACAACCACTACGCCTTGCCGTCTGCGGCAATAAGACTTATTTTGAAAAAA
ACAAAAAAAAAGAGTTTGTTTTTTCATTTGAGTGAAAAGAAAACAAGAACAAAGAATAGATTGAACTGTGCTTGGCTTTGTT
GATTGGTCCGATTTTATACACGGCGAATCGTCGCCTCAAAAGAACGACCGACGTCTCACGTCCCTTAGGGAGATGGGATCAA
CACTCTCCTATTGGTCGATCTTGCTGTAGTTGGAGTAGAAGAACGTAAATCAACAATCAAAAAGAGCACATCAACGTACCAA
AACTTTCTCCCCCCACCCCACCCACCCACCCCCAAACCCCAATATTGATTTCAAATTGAAACCTTACACCTCATACTAGAAAC
CATGGCGACTCTGCATTCCATGGTGACCCTGCTGCTGCTAGTGCTCCTACTCACAGCCCGGCCGGCGTCTTCCTCCTCCTTTA
TCAGCAGGTACGAATACCATGATCATGAAGCACTGCTGGCACTCAAAGAGGCGCTGACAAACAACTCGGGATTACTAAGCT
CATGGAACAGCAGCAGCTCCGACTTCTGCCGTTGGACCGGCGTGACATGCAGCCGCCGGCATCCGGGCAGGGTGGTTTCTC
TGAACCTGCGCCAGAAAAACCTGGGAGGCAGCATCTCTCCTGTTATCGGCAACCTCACCTTTCTTCGGTCGATCGATCTCTT
CGACAACACGCTGTCAGGGGAGATACCGCGCACCATCAGCCGGCTGCGTCGTCTGAGCTTTCTTGAGCTGGCGTACAACTAT
CTTGCTGGTGAGATTCCTGCAGACCTGGCCAACTGTTCCAACCTTGTGTTCCTAAGCGTAGAGGTCAATGAGCTTCATGGGG
CAATCCCTAGTGGTCTGGGCTTGCTATCTCAGCTGCGAGTCCTCTATGTTGGCGAGAATAATCTCATAGGACATATACCGCAAT
CACTCGGCAATCTCTCGGTACTCCAAAGGCTATACCTCTATCAGAACAAACTGGAAGGAACCATCCCAGAGGGTCTTTCCCG
TTTGAGGTATCTCCGACACATCCAGGCGGCAAGGAACAGCCTCTCTGGCACAATTCCACCTCACTTCTTCAACATATCGTCTC
TCCAATATCTTGGTTTCAGTTCAAATAAGCTGCATGGTAGATTGCCACCAGATGCAGGCAGGCACCTGCCTGACCTTGAAGT
GCTCCGTTTGGGTGGCATTGGGAACAACTTTTCTGGAACACTCCCAGCTTCCCTCTCAAATGCCACTAAATTACAAGAGCTG
GGTCTTGCTCATAACAGCTTCGAAGGAAGGGTGCCTCCTGAGATAGGGAAGCTCTGCCTAGAGAGTGTTGAAATGGGGTCT
AACATGCTGCAAGCTGAGAACGATGCGGATTGGGAGTTCCTTAGATATTTCACCAACTGCACCCGTCTAGAGGCTCTAGATG
TTAGTGACAACATCCTTGGAGGAGTGCTTCCAGGTTTTGTAGCTAATTTCTCTGGGCCAGTAAAGTCGCTAATCATGGCAAA
GAACCGGATGTCTGGAGTAATCCCTCTGGGTATTGGAAACCTTGTTCACCTTGAAGACCTAGAATTCGCCGGAAATCATCTG
CATGGTGTTATACCTGAGGATATTGGAAGACTTCAGAACCTGAAATTTTTCACGCTGGAGGAGAATCTCCTATCAGGAGGTAT
ACCGGCCTCCTTTGGCAACCTCACACAACTGCTCACCCTTATATTGTCAAACAATGAGCTCAATGGCTCCATTCCTGAGAAC
CTTGGGAGCTTGCACAGGTTAACATCCA
>SsLRR-RLK315
CGCTGAAAAAAGACTAAAAAACAAAAGACCCTTAACAAGACTAATGAGCTTCTCCCTGGATGCGGAGTAGGAGTGAAGGC
TAAACGGTACTGCTGAATCTCATCTTTACATAGCACAGCAACTTGTCTTGGGGTGGGTGTCTTCTGTTGAAATGTTCTCCTGT
TCCTTTCTTTCCAAATGTTCCACCAAAAGTAGATCAAGGCCCCATCAAAATTGCATTTCTGATTCTTGTAGAATTTACTTCTAC
ACCGCTGCTAAAATTTATACAGTGATCCTGTTGAGTTGATTGTGTTCAATACCGAGATGCCGAGCCATTGCTTAATTATCTCCT
AGACTTACTTGGTGAAGGGACAGTCTTTGCAAAGGTGTGAAAGTGTTTCAGGGCTGCTACCGCAAAGTTTGCAAGTTGGAT
CATTAGGTGATTAGATTGTTGGCTGTTAGGATTTTTTTTTGTGGATCAAAGTCCATGCGAAGAAACGACATCTCGGTTCTACTT
TGGCTTTCCAAATCGGGCAAAGATTGATTTTGCTAAAACTTCCTTCAAATTGGATTTCGTATACACTCCTTGTTGTATATTCTC
CATCAGCTGTCCAGCGCCTAGTGATGATAATATCCTCCCTCGAGGGGTCCTGCTGGACTTCCTTTATTGCCTCTCACAGGTTCACA
TATTCTAACACCTCTTCTTGAGACAACAGGGGGCATATATGGTCAATCCATCGATTGTTTTCTAAAGCTTTGTGCACCGTAATC
TTCTTCCTTCTTGTCTTTTGGAATAGGGAAGGTGCAATTATTTTCGCCCAAGATCCGTTGAGCCAACTCGAAGTCCAGAAAA
GGGCTGTTTTTCCATTACGAACAGAAACTAAAGTTGACGCATTGAAGAGATCATGGTCAGACTTATCACACGGGACGATATC
CGGGTTGTTCCAAGCCCTTCCTTTTTGCACCCACTGAAACCACATCCACCCTTCATTTAGGGAGACATGCAGTCGGCCAGT
TAATCAAGGAGTGACCCCCACTAACATTTTCCGGTGTTTCTCCTCTGCAAAGGAAAGGGCGTCAGGCACCGATCTTGTCGAT
TAGTGGTTGAACTTCAATCTTTCGAAGTTTCCGAGTGTGGAGGGGGAGCCCTAAGTATTTGCCTAGGAAGCTGCTGATTTTA
CCCGGGAAGTTCTGGATGAGGGGCGAGACCATGCTATCTTGTAGTCTTATTGGGAACATTTCTGTATTTGACATATTAATTGAG
CCCCGAACATCCGCCAAAGAAGTGCAAGATTTTTTGTAAGCCATTGAGTTCCACGGGTGATGGATTTGCAAATACGGCTGTA
TTATCCGCATACAGGGAACATCCGAGATTTGCTGCTTTTGGAGAATCGGTTGCAAGACAACCTGTTGCGCTGCCTGTTCAAT
GATGCGCTGAAGCGAATCTGTAGCAATGATGAAGAGCAGCGGCGATAGTGGGTCACCTTGTCGTAACCCATGGACATGCTTA
TAATTTGTTTACTCGGTCTCCCATTGATGAGGATTTTGGAAGAGCTGGAAACTGAGGACTGGGAGTTCTGCGCAGACTGCAC
ATGAGTAAACTACATCCAGTGACGAGGTGGATGTTTTTCTTTCTTTAGTTGCATGGAGCCATGGACCCGTCAAATGGGAAGT
AACTTAATACAAGAAAAGGAAGATACATAGCGTGAACATACGGGCAGCGTCGAACGCTATTACAAAATACAAATATAAAGCC
TACCAAGATGGACTCCTAAGATTGACTACCGAAAAAGAGGTCTTTGTCCTAAAAAAAAAAAAGAGGTCTTTGCACATGACTT
GGAGTCTTGGACTGATAGATGTGTATACCAAGGCAACTCCGTAGATTATCAGCTCACTAGTAAAAAGGTGCCCACTATTAGCA
GGCAAGCAAAAAAGAGATATCCACCATTTGACACTCCACAGCTGAAAGCATGAGCACTCTGCATGCTCTAGTCCTGATGCTC
CTGCTCCATTCTATGTGCAGG
>SsLRR-RLK14
ATTCGTTACTCGATGTTCAACTCTGTGATGAGCATAGCTCCAGATGCTCTGCAGTTGATGAAACCGATAAGCACAGATCCTTT
CTTTTACCAAGAAATAGTACTGAAGAAAAGGTAAATTATTATTTCTTTCGTGCAGTATGTGGTTTCAGTTTAGTCCAAAGTAC
AACAGTTTAAGCTGAGGAATATAACATTTTATTCAAACTCAGAAATACTATTGTATTTTGAAGCTAGGAAATTTTGCTCTGTTC
TTTGTACATATGCTGAAATTGTGCCTTTTCTCCCTTTTTTAATAGCTGTTGGATTTGTTGTCACCATATAAGGATGTCAAAATCA
TACAATTCTCATCTATGGTTGATGCCTTCGGTGGTTTTGCTGATGCAGTAAGCTGCTATACTCTTATACATGTTGCCTAATTTTC
TGGATAAAAGTTTCCACCTTAACGCAAATTACTGAAACCAGGCTGTGGAGAAGAAGTTCCGAAATCGTGTGAAGAGGTCTG
TGGGCCTATGGTGCTGTGTAGAGTTCCGTGAAATTGGTCACATATATTGACATGTACTGGGACGAGAAACCAGGGTGGAA
GCCACACCCTCCAGAGACTAGGGAACAGGACCATCCACCTTGGTCATGACTGCTGACAAGAAGGTGCAAGAAAAGATTGTA
ATGATGAAGATCTGAGATCTTGTAACCAAAGCAAGATAGATTACATGCTACAGGTTTACCTGGGTCACCGGAGCAGCCTGCT
CCTTTTTGTTGGTGGTCCAATCGATTCTTCAGGCCAATTTTGTGCTTCGTAGAAAAATGTCGACCGGCTTTGTAGTTCTGTTCT
TCCATAGAGTGATTCACCTTTGGATTAGTGGCAGCCCCTTGTTCGCAGCTCATCGGTTGAAGCTTGGGGCTCAATGATTTAGT
TGGAGGCAACATTTAGATCATGACTTAGTTCCCTTTTCTTTAGTTTTTGCATCATGTTCATAGTTGATACAGGCATCAGCTCCA
AAGGAGAAAAGGCCATTGTTGTTAAATGTTGTGGAAAACTCGGCTGTCAGCTGCTGCAATAGGAAGCTGTTCAACTTATCTG
GCTGCTGATACATAGGCATCTCTCGTAATTGTTGTGCAAAACTTTGCTTAATTTTTCTTACACAATCAATCAGAACAGATTTTT
GACCCTTAATTTTTCTTACAATACGTTTACATTTGCTAAAAAATCAATATTATCGTAGAGGCAGTTTTGAATACAAATGTTTTTG
ATGTAAATTTCATGTTTTAGTTTTATATATAATTTGAACAATTGTTGATCAAAGTTTGCAAAAAAAAAAATTCTGTAGAAGATATG
ATTACCATTCCGAGATGACAGATATAACATGGCCAAACTGCTTGCAGCTGAAGCGAGCCCCGCGGGTCGCACTGTTGCTCTG
CATTCCAGTTACCGTGACCTGGTCAATGACATTCAGGTAAGGATGAAAACGGTACGGATATTTTTCGAACGTATTCGAGACC
GAATTCGTTTAGAGAAGTTCAGATCTGTCCATATCCGAGTCCAAATATTTAACATCCGATACCGTATTCGTATCTGAATACTAA
ATCTCATATTTATTATATCGACATCGAATCGTATCCTATCCGATATGGCTGACACTATCCGTATTCGAATCTGAATCCAATCAGAA
ATATGAAAACAAATGTAATATTGGTGATATCTTATCGTATCCGATCCGTTTTCATCCTGCGTACAGGCCGCGAACGAGCCGGAA
AGTGACGTCCATCGTATACATGCACTGCTCCAGGTCATGCTTTAGTCGCAAAAGTCCACTCCTGTCTCGATCCTTCGCCGTGC
AGCAGCCGGAGGCCGCCTAGCACGAGTCAAGGTGGTGAAGACTCATTGACGGCGTCGTCGCCACAACGGCAATTGATCGA
AACAGTCGGGCACCGTCTGGCATCTGCGTCGTGCTATAAGTAGTAGCTGTTGTTAGTACATTCCAAGCTCCAAAGCAACGGC
CTGCC
>SsLRR-RLK62
AAATTCGTTACTCAATGTTCAACTCTGTGATGAGCATAGCTCCAGATGCTCTGCAGTTGATGAAACCGATAAGCACAGACCCT
TTCTTTTACCAAGAAATAGTACTGAAGAAAAGGTAAATTGTTATTTCTTTCATGCAGTATGTGGTTTCAGTTTAGTCCAAAGTA
CAACAGTTTAAGCTGAGGAATATAACATTTTATTCAAACTCAGAAATACTATTGTATTTTGAAGCTAGGAAATTTTGCTCTGTT

CTTTGTACATATGCTGAAATTGTGCCTTTTCTCCCTTTTTTAATAGCTGTTGGATTTGTTGTCACCATATAAGGATGTCAAAATC
ATACAATTCTCATCTATGGTTGATGCCTTCGGTGGTTTGCTGATGCAGTAAGCTGCTATACTCTTATGCATGTTGCCTAATTTT
CTGGATAAAAGTTTCCACCTTAACGCAAATTACTGAAACCAGGCTGTGGAGAAGAAGTTCCGAAATCGTGTGAAGAGGTCT
GTGGGCCTATGGTGCTGTGTAGAGTTCCGTGAAATTGGTCACATATATTATGACATGTACTGGGACGAGAAACCAGGGTGGA
AGCCACACCCTCCAGAGACTAGGGAACAGGACCATCCACCTTGGTCATGACTGCTGACAAGAAGGTGCAAGAAAGATTGT
AATGATGAAGATCTGAGATCTTGTAACCAAAGCAAGATAGATTACATGCTACAGGTTTACCTGGGTCACCGGAGCAGCCTGC
TCCTTTTTGTTGGTGGTCCAATCGATTCTTCAGGCCAATTTTGTGCTTCGTAGAAAAATGTCGACCGGCTTTGTAGTTCTGTTC
TTCCATAGAGTGATTCACCTTTGGATTAGTGGCAGCCCCTTGTTCGCAGCTCATCGGTTGAAGCTTGGAGCTCAATGATTTAG
TTGGAGGCAACATTTAGATCATGACTTAGTTCCCTTTTCTTTAGTTTTTGCATCATGTTCATAGTTGATACAGGCATCAGCTCC
AAAGGAGAAAAGGCCATTGTTGTTAAATGTTGTGGAAAACTCGGCTGTCAGCTGCTGCAATAGGAAGCTGTTCAACTTATCT
GGCTGCTGATACATAGGCATCTCTCGTAATTGTTGTGCAAAACATTGCTTAATTTTTCTTACACAATCAATCAGAACAGATTTT
TGACCCTTAATTTTTCTTACAATACGTTTACATTTGCTAAAAAATCAATATTATCGTAGAGGCAGTTTTGAATACAAATGTTTTT
GATGTAAATTTCATGTTTTAGTTTTATATATAATTTGAACAATTGTTGATCAAAGTTTGCAAAAAAAAAAATTCTGTAGAAGATAT
GATTACCATTCCGAGATGACAGATATAACATGGCCAAACTGCTTGCAGCTGAAGCGAGCCCCGCGGGTCGGCACTGTTGCTC
TGGATTCCAGTTACCGTGACCTGGTCAATGACATACAGGTAAGGATGAAAACGGTACGGATATTTTCGAACGTATTCGAGA
CCGAATTCGTTTAGAGAAGTTCAGATCTGTCCATATCCGAGTCCAAATATTTAACATCCGATACCGTATTCGTATCCGAATACT
CAAATCTCATATTTATTATATCGACATCGAATCGTATCCTATCCGATATGGCTGACACTATCCGTATTTGAATCTGAATCCAATCA
GAAATATGAAAACAAATGTAATATCGGTCTTATCGTATCCGATCCCTTTTCATCCTGCGTACAGGCCGCGAACGAGCCGGAAA
GTGACGTCCATCGTATACATGCACTGCTCCAGGTCATGCTTTAGTCGCAAAAGTCCACTCCTGTCTCGATCCTTCGCCGTGCA
GCAGCCGGAGGCCGCCTAGCACGAGTCAAGGTGGTGAAGACTCATTGACGGCGTCGTCGCCACAACGGCAATTGATCGAA
ACAGTCGGGCACCGTCTGGCATCTGCGTCGTGCTATAAGTAGTAGCTGTTGTTAGTACATTCCAAGCTCCAAAGCAACGGCC
TGCCA
>SsLRR-RLK179
GCATCTTTTGCTTATTAACTCCTTTTTTCGCATGCTTCATATTTTCTCTGGCTGTGTCATATTTAATTAGTACTTTCTCGTCATTG
GCAACTTGGACTTGTTACTTGCCCGTTCCAGTTATGTGTAGGACATGTTTGTAGAATTCTATCTACGTCTCTTAATTGTTCCTG
TAATAGTTCATGCCTACAGTTTTCTTGCAGGAATTCATTATTATTGACAAATATTGCAATTTATAGAATTATATCAGTTACAATCA
GACGATGCCTACAGTTTTCTTGCAGGAATTCAATACTATCCCATGTTCATAAATAGTTCACTCTTCATTGACATCTTGCTTCGAT
GCTATCAACAGAGGATGCTGGGAAGAGCACATGGTGCACATCACAATGTCCTATATCTTGTATGTCACTGTCAGTGTCCATAT
CGCAATTCACAAGGGGCCATATCTTGGGCTAAAGGAAATCCTCACTCATCAGCAAATAATCATTGGCATTATTATTATTATTATT
GTATCCCAGAAAGGTTTGCTGTCAGACTTGTGATATCAGAATATGCTTATCTGATTATAGTACAATTTCATGTACTGTTTTACAT
GCTTGTACTCTGAGAGGCATGTGAAATATATCTTTTGTTCATCTGTTATGTGTGCTGTCTAGTTGTTCCCAACGTATGGAGTTAT
GGACACGTAAACTGCGGATAAAATTATGTTTTTATATCTTCCATAGAATCTGGAAAACGCTGAATTAATACTGCTGGTTTCAGA
CGGCAAACAGTTACAAACTGAACTGTTGGTACAATCACTTAACTACTTAAGTACTCCATTTTTGGCATACCTCTGCTTAACTT
GTGCCTCTGTTTTCTTCTGTGAAATTTATAGATCATACGTTGCCTGGCTAGTGGCTACTCAGGATCTCATCTAGTACTGACACA
GTTGTGTTTGGCCACATCCACGCTGCCATTCCTAATCTATTCTCAGGTAAAGCTATTATTGTCTTTTGCTAATTTGCTGCCTCTA
GAGTACTGACTACCGGCCACCGGCCACTTCTCTTTGCTTTTTGTTGACCGTATTTCTTTGCATTGTTTTGATTTTTATAGATAT
CCTTATGTGAGAAATTACTTCCTTATAAAGATGATAGTTCTGTTTGCAGTTTTTTGAATAGTTGTGTTCATGAGTAGGAATAAT
GGTGTTTCTTTGTTTTCCTAGTCGAATTGCACTGATAACTTCTGAAGTAGGAATATTCTAGGACAATAGTGGTTTATGAGAGAT
AATTATACATCCAACAAATACTTTTAAGTTCATACAAAGAATCGCACTGTTAACTACACATCTGTCTGTGTCACACACACACA
CACACCGCTCAGGTACCAAGAAAGGTGCAAATCGACCAGAACAGATAAATAAAACCACCACGGTTTAGACTGCTGGCATCT
GTATGTTCAGTAAGTTCAGAATCCCAACAGAAATCAATTAGTTTGCTAAGGAAACAGAAAAAGAAAACTGAACTTAGCTTT
GCCATGTCAGCCTCCAATTGCTAGAGCAGAATTGGGTAATTCTGTGAATATAACTGAATCGAAGGTGTCTCAGATTAGGAAC
AAAACTTGTTCGCTAATGATCCACAGGTTGGAACATGTTTCTAATCTACTAGTTGAAAAAAATCTCCAGAAACTGGCAAAGC
CGAAAACAAACTTGGAAAGGCCATGTGAAAGTCAAAGATTGAAGGAAGCATCGTGCAGCGACGTCTGACCAGACCACGAA
TCAGATGCGATGCATTTGCATGCTGCATGCATTATTAGCACGCGTCCATCAGGATTAAAAAACACAGCAAGTTGCTCCCCTGC
TCTGACCAGGAAAGGCGTGCCCTTTTTTTCAATAAATATAGGCCAAGAAGGCCTTTTTTTTTTGACAACAGGCCAAGAAGG
CCTTTGTTTGGCTTTGGCAGCCAACAGACTTTTATTGGCTACTCTTACTCGTACTCCACGCTCCAGCCCATCTCCACC
>SsLRR-RLK192
TGGGCATCTTTTGCTTATTAACTCCTTTTTTCGCATGCTTCATATTTTCTCTGGCTGTGTCATATTTAATTAGTACTTTCTCGTCA
TTGGCAACTTGGACTTGTTACTTGCCCGTTCCAGTTATGTGTAGGACATGTTTGTAGAATTCTATCTACGTCTCTTAATTGTTC
CTGTAATAGTTCATGCCTACAGTTTTCTTGCAGGAATTCATTATTATTGACAAATATTGCAATTTATAGAATTATATCAGTTACAA
TCAGACGATGCCTACAGTTTTCTTGCAGGAATTCAATACTATCCCATGTTCATAAATAGTTCACTCTTCATTGACATCTTGCTT
CGATGCTATCAACAGAGGATGCTGGGAAGAGCACATGGTGCACATCACAATGTCCTATATCTTGTATGTCACTGTCAGTGTCC
ATATCGCAATTCACAAGGGGCCATATCTTGGGCTAAAGGAAATCCTCACTCATCAGCAAATAATCATTGGCATTATTATTATTAT
TGTATCCCAGAAAGGTTTGCTGTCAGACTTGTGATATCAGAATATGCTTATCTGATTATAGTACAATTTCATGTACTGTTTTACA
TGCTTGTACTCTGAGAGGCATGTGAAATATATCTTTTGTTCATCTGTTATGTGTGCTGTCTAGTTGTTCCCAACGTATGGAGTT
ATGGACACGTAAACTGCGGATAAAATTATGTTTTTATATCTTCCATAGAATCTGGAAAACGCTGAATTAATACTGCTGGTTTCA
GACGGCAAACAGTTACAAACTGAACTGTTGGTACAATCACTTAACTACTTAAGTACTCCATTTTTGGCATACCTCTGCTTAAC
TTGTGCCTCTGTTTTCTTCTGTGAAATTTATAGATCATACGTTGCCTGGCTAGTGGCTACTCAGGATCTCATCTAGTACTGACA
CAGTTGTGTTTGGCCACATCCACGCTGCCATTCCTAATCTATTCTCAGGTAAAGCTATTATTGTCTTTTGCTAATTTGCTGCCTC
TAGAGTACTGACTACCGGCCACCGGCCACTTCTCTTTGCTTTTTGTTGACCGTATTTCTTTGCATTGTTTTGATTTTTATAGAT
ATCCTTATGTGAGAAATTACTTCCTTATAAAGATGATAGTTCTGTTTGCAGTTTTTTGAATAGTTGTGTTCATGAGTAGGAATA
ATGGTGTTTCTTTGTTTTCCTAGTCGAATTGCACTGATAACTTCTGAAGTAGGAATATTCTAGGACAATAGTGGTTTATGAGAG
ATAATTATACATCCAACAAATACTTTTAAGTTCATACAAAGAATCGCACTGTTAACTACACATCTGTCTGTGTCACACACACAC
ACACACCGCTCAGGTACCAAGAAAGGTGCAAATCGACCAGAACAGATAAATAAAACCACCACGGTTTAGACTGCTGGCATC
TGTATGTTCAGTAAGTTCAGAATCCCAACAGAAATCAATTAGTTTGCTAAGAAAACAGAAAAAGAAAACTGAACTTAGCTTT
GCCATGTCAGCCTCCAATTGCTAGAGCAGAATTGGGTAATTCTGTGAATATAACTGAATCGAAGGTGTCTCAGATTAGGAAC
AAAACTTGTTCGCTAATGATCCACAGGTTGGAACATGTTTCTAATCTACTAGTTGAAAAAAATCTCCAGAAACTGGCAAAGC
CGAAAACAAACTTGGAAAGGCCATGTGAAAGTCAAAGATTGAAGGAAGCATCGTGCAGCGACGTCTGACCAGACCACGAA
TCAGATGCGATGCATTTGCATGCTGCATGCATTATTAGCACGCGTCCATCAGGATTAAAAAACACAGCAAGTTGCTCCCCTGC
TCTGACCAGGAAAGGCGTGCCCTTTTTTTCAATAAATATAGGCCAAGAAGGCCTTTTTTTTTTGACAACAGGCCAAGAAGG
CCTTTGTTTGGCTTTGGCAGCCAACAGACTTTTATTGGCTACTCTTACTCGTACTCCACGCTCCAGCCCATCTCCACC
>SsLRR-RLK314
CAGATAGGGTCCACTGCAACATGTGTAACACGAATTTTTTTTGCAACATCTAGAGGAACACATTTGCAACGTATGATCGAAA
CAGTTAAACACTTGCAACATACGTCTGAAACATGAAGGAAACACATGTGTAGCCATTGCAAAACATATGCAACATCTAGATG
AAACACTTGCAACAAATGTGTGAAAACATAGGCAACATTCGGATGAAACACTTGCAACATAAGTGTGAAACATATGCAACA
ACACGCATGCAACATCCGTCTAAAACAACTTAAACATTTGGAACAGATGTTTGCAACATACGTGTATAGCCATTGCAACATAT
GCAACATCCCGATTTACTTTTGCAACATCCACATAAACATCCACATAAACTAGATTTACTTTTGCAACATCCACATAAGCGTAC
TTCGCCATTTTGGGAGACTAGATTTCTTTTTTTTTCTTTAGCAAAGGTAGACTAGATTTCCTATGGGCTTCGATGAATTGAAAA
AAGGCCCACTCGCTAACCCCGTAATCCACGTTTGGCCTTCTGCGTGGTCCACTGCCCGTTATTTCCCCTCTATTCCTCATCTGT

CCGACCTTGAGCGACGGTGCGCGGTGCGCCGTGCGCCCGATGGTGGGCGGCGGCAGATGCGCCGGAGGCAGAGTTCAAGC
AGCAAGTGCGGACGAGCAACGGAGGCGAAGCGCGAGGTGTATGCGCGGCGGAACGCCTGAGCGACTGAACAGGCTGAGA
AGGCACGGGCACACGCGTGCACGGCAGTGTGGCATGGACGCAGGAGCCGCCGTCGGCCTACGCGCCGCTGCACTGTGCGT
CTGCGCGGGAGCAGGGTGGCCGCCGGCTGTGCTGCATGCGGCATGCCTGCACGGCAGAGGGCAGAGGGCAGAGGCACGG
AGACTGGACTGGACAGGCAAGCATCACCGTCCCGTCCAAGTTCTAATCTGGTGTGTGATAATTAGATATTTGCATGCTTTAGC
AATTGTGACGATTAATTATTGGTTTGGTTAAATAAAGCATGAACTGCTGCCAGTCGGCTACTCATGGAACAACACCTATTCGC
TTGCTATAGTTATGGTGATTGGTGATGCCAGGTCCCAGGCATACTATGGTCTGTTCCATCGTAGTTCTTGCCCTTCACGTGTTC
GACGAAATGCGTCTCCGGCCAGCGTCTCCAGCCATCCGAGTAGTTGCATCCTCTCCTGTGATCTGAGAAATTTCTACACTATA
TATGCATCCTTTTTCTGTTAGTTAGTTACTAATTTGTGTGTCTTCATTGCTTTAGAGTCTTACATTTTTCATTGAAAATATGGAAT
GAAAATAAAATGTGGTACATCTTAGCGTTTGTCACTATAGTAGAAAACCATGTGAAAGCTTAGTGGCATAGATGGTGACTTGG
GTATTGCTGAAAGCACAGTTTCATATTTTCATCATAGTTTGAACTTGACCCCCCTGGCTACGCTGACCTCTTCTGGTCACGGT
CAAGATTACCCACTCACGGAAAAATAAAGGCGCATTAATTAGCCTAACCCACTTCCGCGTAGACCAATATAATAAGAGTATTA
TGGGAGGCAACAGAGATCAAAATTAAGAACACCACTGGTCCTTATGTATTATATACTAGCAGCATTTACCAAGAAAGCTGGA
CAGGTTACAATCAGCAGCATCGGGGCTGTAAGACCATGAATCACTGGAGTAGTAGTCGTGGACTCGATGCACCAGTACAGCT
AAAAACAATGCCATCTACGGAGAAAAGTAGAGCAAGAAGAAATGAATGAAGAATCACACTAATTTTCAATAAAGTGTTCTT
GGTTAGTTCTCTGCACCATACTGCTTTGTAAAGGGCTTCTTTGTACAGATTTACTTTTTCAATAAAGTTTAGTTTCCATTAAAA
AAAGAATCACATATTTCACCTGCTCTCCACCATTAAGTTAAGTTAATTAACTATAAACCCTCAACGACGACTGCCCTCGGCTAT
CGCCACTACACACACTCGAAA
>SsLRR-RLK151
CCGCCCCTCCCGGACCACTACTTCGGCAACGCGGTGTTCAGGCTATGCGTCACCGGCGCGGCGGGGGAAATTGGCACGGTG
GCGTTGGGATCCATCGCTGCCCGCATCAAGGGCACCGTTGAGCGGATGGATGACGAGCTGGTGCGGTCCGCTATTGACTACT
TCGAGATGGCCGAGATGGACAAGAGACCGGCTTTGAGAGGGACCTTGCCACTGACGGACCTGCATATTACCAGCTGGTTCG
GTAGGCCACAGTACGATGCAGATTTTGGGTGGGGAAAGCCGCAATTCATGTCGCGGGCAGAGTCCGTCCGCGGCGGCTATG
TGTACTTGATGAACGATGAGGGGACAACGGACGACGGTGGTGGCAGCGGCGACGTTCGCGTGCTCGTGTGCATGGAGGCT
GTAAATATAAAGGAGCTAGGGCGGCTGCTTTATGCAAAGCTCTAGCAAGCTGCTAATACTACTGAGTACTAATAATAAGGTTT
TGTAATAATATTAACAATAAGGTTTGTAACTAAGCTTAAGGGGAACGCGTGGTACCTCCTGTTTGATTATAGAGCTGTCTCGAT
GCTAGAACCACACGTATCTATTAATATACCTAGACATTATATATAGAAACTATGTTCTCAATGAACATGTTGTATAGAATAATTTT
TTTGTCCAATCACATGTTTTCCTTCTTTCTTCTTTAATTGAAGTGACATATTATCTTTTGGCTTAGTTGATATCATAATTAACGAA
TATAGAAACAATCTAGTAGTATTCTAGTTTCTTGGTTGAGAGTGTCCTTGTTGATTTTAAAGGCAGTCATGCATTATAGGAGTA
GTACTAAAAAAGCATGCATGTACCTATATAATAAGGTTTAAAGGCAGTCACGCATGAATGGTTGATCAATCCAAATAACTTAC
AATTTTGAGAATTGAGAGGCATTTTAAACCTTCAATACCTTATATTTCAGGACCAGACGAGTACTAATCGGGGAAAGTATCCA
CTTCATTCGAGCTTCGAGGCTTTCTGGCTAGGCCTTGCTTCCACGGCCGATACCGTACCACAACATTGGCGTCGTATCGGACC
ATCCCCGTGTGTCCACACATTTTCTCGTATGCCCACATGGTCCACTACACTGCTACAATGCAATCGTTGCCTCCGTCTTGTCTT
CCTGCAGGCATCCTCCCTTCCCCGTTGTGGTCCTCGGCGGGGACATGAACAACGGTAGGACGACGAGACGTCATGCATCTGT
CCTCCCCGATTGGGACATATCTCCTACGGAGTACTAGTACTGAATGGTATTGTTACAAAGACCTTCAGTTTTGCCTTATGATAC
ATGTAAAATTACTAGTGGTTTTTATCCTTCCTAATTCTACAAAAGTACGTACCTCTGTCTAGATGGTGGTTCTGAATGTGGAAC
GCTAGATGTATTTTGCCCATGAACACGCGACCATCATCAGCAAACACCATCAAACATCTCAGTATATATAATAAATATAAATTA
ATAAAAGAAAGTTGTATGGTCACCCAATCAGTATCAGGCCAAGATTTTTCCACACAAGGTGCCATCGAGCACCACTGCGGTC
TACATACGGATAGCAAGTTGCCGCAACAAAACAGATCGATAAAAATCCCACGACTGAAGGTGACATTTTCTCCATGCCTACT
GTTTTTGTTTCTGTTTCTGTTTCAGGTGGACGTCGTAAAAACAGTTGTCGGTGTTGGAGCGACCCTTATCGGTTCCTCATTTG
GCAACTTGTACATGTCATGCATGCCTACTGAACTCTGAAGACTGACTCAAAGTCCACAGAAAATGCCTACTGAACTCTGAAG
ACTGACTTAAAGTCCACAGAAAATGCTAACGTAGCGAACACGTACTTCTGGCAGCCACACATGTACATGTCATGGCGGCACC
TGATAACGTGATCCGCTGTTATAAATTAGAAATACCATCAGCAGGCTGCAAAACACAGCCCATCATTTCCACAATGTTTACCA
AAGCAGCCTGC
>SsLRR-RLK152
TGGGGGGCATAGTGAGGCATCTCTTTTCGAAAGATCCGTTTTACCGGCTTTCATTGATAAGTAGAGAGTAAATTTATGTACAA
GGCAGCCAAACGTAGCGTGAACCACCACGAAGGCGAAGCCCACCCACGCAAACACAACAGAGTAAACGGCGACTCAACT
AAGATGTGAGATCGGATTTCCCGAAGCAGGAGGCCATAGTAGGTCATCGTTGCCCAGCTCAAGCATGTCGCGAGAGCAGCT
CCGACTTCCGACCATGGCCTCACATCCTACCTCGAGACATCGCGACCGAGTCAAATTCCACAACGACACCTTCAGGAAGGTT
ACGACGTATGCGCCGTCGTCGTTCGTCCAAGGGTGGACCGGGTTTTCACCCGTGGAAAATGCCGGGGACGCGACGCCCCCA
ACAGGGAAAGTGGCACCCGCAGGCGTCACCGTCGTGAGGCTTTCGCCCAATGCTCCCAGCACCGACGTCGGCCCAGATGC
CGGATCCTAGCCTGTCGCCACTATCATCGCTGCCAGTCATCCTGTAGAAACCTCGCTAGAGGGGCACCGACGCGCCGGCTAC
GCAAAGCTGCGTCAGCCACCCCCGCCAACAAGACGACATGCCGAACACCGGCCTCACTCCGCCGCGCGCGTCCCCCTGCC
GCACGCCAAAGCCGTCGCCGGCTGAGCACCCACCGAAGTACGCCACCAGCTAGCACCACCATGGCGCTGTTGGAGCAGCA
GGACAGAGCCACGGCCAGCCACGCCACCACGGCGTCGCCGACGAAAGCCACATCACCACTGCCACCATGCGTGCAGAAGA
GCTGGGCGGCCGGAGCACGCCACCAGCTTGTCACCGCACCACCGCGCCGCTGCAGTCGTCATCCACCACCACCAGCCACTA
CCACCGCAGGAGCACCAGATCAGGGCCGAAGAAGTCCAGATCCGGACACCAGGGTGCCGAATCTAGTGCTCGGAGCCGCC
GCCGTCGTCGCCGCGCCCGCAACAGTCGCCGGCAACGTCGAGCAACGAGCAGCGAGCAGCCCCCATCCCGTGCCACCGCG
CACAGCAACCGCCGCCGCCCACCCCGCCGCCGTCACGCGGCCACCGCGCCACGCCGCCATGGCCGCCGAGCGCCGCCTCG
CACGGAGAAGCTCGCGCGCCTGGACGAGAGCCTCGCTGCCACCATCCTAGGGGGGCACGCGGGCCTCCCGGCAGCCTCCT
CCGGCAGCGGCGAGGTGGGAAGGGAGGGGGAGGAAGGGCTGGTGGCGGGCTAGGGTTTCGCCGCCCGCGACGCAGGGGC
TTGCTCCGAGTTTTTCCACTATCAGCTGAACAAGTAAGTCCCTCTGTCCATAGTGAGGCATCTCAATTGTGACAAAATTGTCC
AAGCTCGACAGACAGACTTTTGAGATTCTGAGCGCCTGAGTCATGAGCAACAAGAAAAAAAATGGAAAGCTTTTTTTTTAA
AAAAAAATATTTCAGCGTGTATTATGGGATTGACCTTAACAGCTGGTGTAGTGATTGACCTAGCGACGGAACTACTGTACATC
GTGGTCAGTGGGCCGTCTCAACATTACGCCACGGCTGTGGGCTCATTAGAAAGGGGCAGAGCGCGCGATCTTTTGAGTCTT
GAGGAATGATGAGGGGGAAGAAAGAAACAAGAGAGCAGCGCTGTGCCTTTCAGTCGTGGAGTCAAGTCTTCCCGGTCGTC
CCTGGTCTTCAGTCTTCACAGTTCACACTACAATCAGTCATGGCTATAGCTGTATAGGGAGAGTCTGTGCACCAGGCAGCATT
AACCACTGGCTCATCGCCGCGTCCTTGACCGTTCGTTCACCTCCTGCATGCCAGCCCAACTGATGACGTCTTCCTTCTTTATG
CATGTCAGAGTAACGCCTAACATTGTACTACTACGGCCCGGATTGGCAGTATAAATAACACCACCAACTACAATTCTACTTAT
CAAACACCTAGTAGCTGTTTACACTCACCAGTCACCACGTACCAAAGCAGCCGAC
>SsLRR-RLK164
GAATAATTTTTTTGTCCAATCACATGTTTTCCTTCTTTCTTCTTTAATTGAAGTGACATATTATCTTTTGGCTTAGTTGATATCA
TAATTAACGAATATAGAAACAATCTAGTAGTATTCTAGTTTCTTGGTTGAGAGTGTCCTTGTTGATTTTAAAGGCAGTCATGCA
TTATAGGAGTAGTACTAAAAAAGCATGCATGTACCTATATAATAAGGTTTAAAGGCAGTCACGCATGAATGGTTGATCAATCC
AAATAACTTACAATTTTGAGAATTGAGAGGCATTTTAAACCTTCAATACCTTATATTTCAGGACCAGACGAGTACTAATCGGG
GAAAGTATCCACTTCATTCGAGCTTCGAGGCCTTCTGGCTAGGCCTTGCTTCCACGGCCGATACCGTACCACAACATTGGCG
TCGTATCGGACCATCCCCGTGTGTCCACACATTTTCTCGTATGCCCACATGGTCCACTACACTGCTACAATGCAATCGTTGCCT
CCGTCTTGTCTTCCTGCAGGCATCCTCCCTTCCCCGTTGTGGTCCTCGGCGGGGACATGAACAACGGTAGGACGACGAGAC
GTCATGCATCTGTCCTCCCCGATTGGGACATATCTCCTACGGAGTACTAGTACTGAATGGTATTGTTACAAAGACCTTCAGTTT
TGCCTTATGATACATGTAAAATTACTAGTGGTTTTTATCCTTCCTAATTCTACAAAAGTACGTACCTCTGTCTAGATGGTGGTTC

TGAATGTGGAACGCTAGATGTATTTTGCCCATGAACACGCGACCATCATCAGCAAACACCATCAAACATCTCAGTATATATAA
TAAATATAAATTAATAAAAGAAAGTTGTATGGTCACCCAATCAGTATCAGGCCAAGATTTTCCACACAAGGTGCCATCGAGC
ACCACTGCGGTCTACATACGGATAGCAAGTTGCCGCAACAAAACAGATCGATAAAAATCCCACGACTGAAGGTGACATTTTC
TCCATGCCTACTGTTTTTGTTTCTGTTTCTGTTTCAGGTGGACGTCGTAAAAACAGTTGTCGGTGTTGGAGCGACCCTTATCG
GTTCCTCATTTGGCAACTTGTACATGTCATGCATGCCTACTGAACTCTGAAGACTGACTCAAAGTCCACAGAAAATGCCTACT
GAACTCTGAAGACTGACTTAAAGTCCACAGAAAATGCTAACGTAGCGAACACGTACTTCTGGCAGCCACACATGTACATGT
CATGGCGGCACCTGATAACGTGATCCGCTGTTATAAATTAGAAATACCATCAGCAGGCTGCAAAACACAGCCCATCATTTCCA
CAATGTTTACCAAAGCAGCCTGCATGGCATTGCCCTGCTCGGTGTCCTTGTTGCTGCTATCCTTCTCGACACTGGTGGCTGCA
CACGCCAACGATGAGGCAGCGCTGCTGGCCTTCAAGGCCGCTGCGATCAGCAGCGGGTACAATGGCCCGCTTGCTTCGTGG
AACCGCAGTACCACCGGTGGATACTGCAGCTGGGAGGGGGTGAGGTGTCGGGGCACACACCGCCGAGTGGTGGCGTTGAA
CCTCCCGTCCCATGGGCTCACCGGCGTCCTCTCGCCTGCCGTTGGAAACCTATCATCCGTGAGGATTCTGAACCTAAGCTCA
AACGGGTTCAGCGGGAACATCCCCGCGAGCCTTGGTCGTCTGCGCCACCTCCATACCATCGACTTGAGCCGAAATGCCTTCT
CTGGCCTGCTCCCCGCTAACTTGAGCTCTTGCACCAGTCTAATAGAGATGGTCCTTGATTTAACAAGCTCAGCGGGAACAT
ACCCTCTGAGCTCGGTGATAAGCTGAAGCAGCTCAAGGAGCTCAAACCTGCAGAACAACAGCTTCACTGGAAGGATTCCG
GCGTCGCTGGCCAACTTGACATCATTGACTATTCTTGATCTCGCGTTCAACCTGCTCCAGGGCACTATCCCGACCAGCATTGG
CGTCCTCAAGGACCTCT
>SsLRR-RLK135
GTTTGCCGACATCGGGAGGGACCCGTGCACGCCCGCCGCCTTAGCCGTGGATCCACAACCGCCGGAGGGCACCCACATGCG
CTCATCGCGGATCCACCCACACTAGGAGGGCCCCGGCGGGATCGCGTAGATCCACCACCGCACGCCCCCGCCGTAACAGCC
ACATGTTAGTTTGCCGCCATAGGGAGGGACCTGCTCGTGCGCCCAACACCGCTGGGAGGGTCCCCCACGCACGCACCCACC
ACCATACCCATGGATCTGCCGCCACGGGGGCGCCCACACGCGCTCATCGCAGATCCACCCACACACCAGGAGCCCCCCCC
CGGGCGCTCGCCGTAGATCCACCACCACGCGTGCCCGTTGTTGTAGTAGCCACGTGCTAATTCGCCGCCGCCAGGAGGGAC
CCGAGCGTGCGCCTGCCACCACTGGGAGGGTCCCCTCGGCACTCGCCACAGATCCATCACCCCGCATCCCACCATCGTAGC
AGCCACACGGTAGTTTGCCGCAGTCGGGAGGGACCCGCGCATGTGCCCAATGCTGCGGGGAGGGTCCCCCACGCACATCCG
CCGCCACCCACGCGTGCTCATTGTGGATCCGCCCACACCGGGAGGGCCCCCGCGTGCGCTCGCTGCAGATCCGCCACCGCG
CGCCCCCAACGTCAGAGTAGCCACACGCTGGTTTGTCGCCATCGGGAGGGGCCCGAGCGTGCGCCCGACGCCACCGGGAG
GGTCCCCCGCGCGTGCCCGCCACGTAGTCACGGATCCACCGCTGCCGTAGCGAGATGAGAGAGAGAGAGAACTGAGAGAG
AAGAGAGAGCCCGTATCTGAGAGAAGAGAGAAGAGAGTCGAGGGGCTTCCAAACCTTAACTCACGCTTATATATGTGGACA
TAAGTAGCAGGCCCTGGTCCTGTTGGGCCTCGGGTGTTTTTACGGAAGAGGCCCACCTCGGAAATGACCTCCACTTTTTGAG
GTCCATAAATCTATTTAGGAGATGGATAATTTTTGCACGCCTCCGTAAATTTAGTTTAGGAGACGGGCAATTTTTTCCACCTC
CGCAAATAAAACATCCGCCTCCGAAAATCCTCAGGTATTTTAAGAGACGGTTGAGTTTTGTGACCGTCACCGTTAATGATTCG
GCAGTGTCTCGCGAAATGATTTATGTTGTCGTGAAGGGTCCCATATGTCATGGGTTCTTCCAGAACTAATTTTATAATCATGTG
CCACTCATACGGCCTCCAAAATAAAGTGTCACGCAAATATTGATGCTACTTTAATCTTAACATATTTCTTAGTAAAAATAATCAT
CATACACTCAGCACATGATGACTTTTTTCCCGCCCTACATACCCAAGACTGCCGGTCTATCCTGAGGTCGTCGTGAGTCGGCA
GCCATCAACTTCAGAGTTCAGACCGTGCTAGATACGTTGGGGAGTGGAGCCGCCTTTCCGATTGTTTCAACACATGACTGTT
TCCGCACCAATACCCAACAATACTGTAGACAGTCCATCTTGCGGACACAAGACAATGGTCATCAGTTCTAGAACACCGTTTT
AGACCCGTTAGAGCCGATTTCCCCGGTCTGTGATATGATAGAGCTGTCCTCACCTTTTTTTAGCTGTCCCCGCACTCGAAGAG
CAACATTCAGAATGCGGTGGGTCTTGACGCCTCTTTTCATTTCAAGCGGTTGTGCGCTGCAGCAGGAAATTTCTATAATCAAA
GGGTAAGTATGCGCTCCCTCGGTCACGTTTGCTTGCTTCAAAAGAATGGATGATTGAGCTGCACAACGTGGACAAATCACTA
GCTAATCAATACTTTTTGACTGAATTATTGGCTTGAACCCATCTGTAATGATGCAAGGGCGGTAACCACTCGCTTTTGACTCA
AACTTCAACCCATGGGTACTCCCTTTATTTATATATTATGTATGTATATGTATGAAACTCCAACTACATCAATACATCCGGTATCT
CTCCTCTGGCTCAAAGCCTTCAGCAGCCA
>SsLRR-RLK113
GTGGCCCCAGATTGCTGTTACTGACTGGATCTGATCTCTATCTCTAACCTTAAATAAACTCCCACTTATAATTATCCTAGATCCA
CACCGCAATTAGCTGCTCCATAGAGATGACTTTTACTCCATCTACACCAACAAATGCACCCAGAAATACAACACCCTAGAAAT
GTACGTGTGGGACATAGTCCCCGGTATCCCGCAAGAAAAGGTGAAGACCTGGTTTGACTAGAACTCTTCCTACGTAATCCTA
GTAGTATTGCTAACTTGTAATCTTATTATGACTTATTAATTGTAACAGACTAGAAGACTTGCCTCTTTTACTATACAAAGGAGG
GCAGAGCTCCCTAGATCGACAGACAGCAGATGTACCAACACATCGTACAAAGCAATCCAACGTAAGGCCTCACGCCTAACT
GGACGTAGGGTATTACGCTATCAGCGACCTGGACCAGTATAAATCGTTGTCTCTGCGTTTACCGTCGAGTTCTGCATACACCG
AAGCCCATTAATTATCGTCTCAGGTAACCCCGTGACGAGTTGCCAGTCTTCAAACACCGACAATACTCACTAGATCATCTCTC
CTCCATTCTCTCTCCTTACTCAACCAAATCCAACGGTCATCGTTCAATGGTTCTGCAGTGTCCCCCTCCTGCGTCCCTACCATC
CACCCTCCCTCTACCCAGTGGCGGACCTAGGATTTTACCTCAGGGTATGCTTAAAAAAATTCTTATGCAATTTGGTTAACCATT
TACAACTCAATAAAACCATATTCTAATTCGATAAAGCCACGTACATTAAATAACATGATAAGCCTTAAATTCAAAGATTAAGCT
GGAAACGACGACAAATCTCGATATAAATTGTTCAAATGAAATACAAATAGTTCGAAATACAGCCATAGAAGAGACTTATAATA
TTACTTGTTTATCCGCCGCTTTCGTAATGATATGAATGTTTCGATTATATCATCTTCACATACTACCTTCTTTGATGGATTGATGC
TAAATTGCTAATTAGGTCTGCTGAACGAACTCACGAACACGAACTGAAGCTAAAATAATAAGAAATCACTCATCAATAAATA
AATAAAAATATGACTATACCATACGAGTACACAAGTTTTTTACAAGATCAGCACCTGCAGTCCAGCCAATCCTGCAACAAAG
TGCTGCGCCGGCCCCGGCGACCGACGCAATCCCGGCCCGGCGTGTGGTGTCCCGCGTCCTGGCCTAAGGCCAAACTGCCGA
AGCTCCGCCAAAGTGCCGATCAATGCCCGACGCGCCGTCGTCGCCGAACTGCCGAAGCTCTGTCGTGGCCAGCCAGCGGCC
GCTGGGGTAGAGACGAAGAGTCGATGACGAGTAGACAACGATAGACAACTTGACTTCTCCCATTCTATGCGTGCAGTTACTC
TCAGGCCGGTGCAGTAACTCGGTGTTCGCGGTATGCGCCCGTGCGGGAGCGCAGAAGTCGATTGCTTCATCTCTCTTTTTTT
AACATATAAAATTTCTATTATGTGTGTATGACTGTTGGGCCATAGGGTATGCCGTGGCCCACCTAGCATACCATATGGGTCCGC
CCCTGCCCCCTCTCAATCACTCACCCCATTGAGCTTCAAGGTCATCCATGGCCAGTTGGCCACCACCACCATGTGTCGCCTG
AGGCATCCCATGGATCCAGAATGCGAGCACGAGAAGCCTTCTCCTCGCCATGGCTGTAGGTAGTGTCTCCTCGATGTGTCCT
TCCATGGCCCACATTTCGATAGAGGCAGGATTCTCATGATTAGGCAGCTAAATGATTAGGCAGCTAAATGATTAGGCAGCGCA
CTAGCACTACGGTGGAGGAACTTTCCATTCCATAAAAAGCAATCATGGAACCTCAGCTGAGACAATGCATCTGGCCATCTGC
GCATTTCAGCGATTATCTCTCTCTCTCTCAGCCAGCCAATGCAAAGAAACCAAGCTCTACGCCGCCACGCAATAAACTGAAA
GCCATCAGCGGCC
>SsLRR-RLK114
TCTGTGAATAAGGAATCCTCAGGACGGAGGAATAAAATTCAAAATTTTGTGTGACTACTATACGTTATCTACACGTAGGTTCC
GGGTTGTTTTGACGTGTATACTCAGCCAACCATCATGCATCTATAGGACAGCATTGACCGAAGTGGACAGGAAAAGGGAACT
GACAACTAGTTGTTGCTGTAACTCAATCATCCTTCATTACATATATATTGGATTTGTGATCCGCTAGTACAACCCGTAGTATACG
CAAAGCTGTCTTAAACCATGTCAGCAGTTCAATCAAAGGCGGCAGAGACTCCTTTGCATGCAGTTACCATGAAATTATAGTT
AGCAGCAAGCAGCCAAGCAGGGGAGACCCTGTGAACCGAGTCATGGTCTCCTTTAGTCGTCAGTCTAACCAGGAGGAGGC
TAGTTTCTCTGATCTAGTACTCTCATGCACTAAAAGTCGGTGAATACATACAGCTTTACCGTGAGTGCATCTTCACAACGATG
GAAGACAACAGTTCCTAGAAATACGCGTTTGGTGGAGCCCTGAACTTACACCCTTCCTTTGCATGCTGCTATGAATTATTGTT
AGCAGGCAGCCAAGCAGAGGGAGACCTCGTGAGCAGAGTCAGTCGTCGTCTCCTCAGTCTGGACGACGTGTGGGAGAGG
GAGAGGGAGGCTGCAAGAATTCATGTGCGCCACTGCCACGACTGCCCTCTGGTCTTTCCACCGTCTCCCCAGAACACGGTG
ATGGTTCAATTCAGTTGATGTAGATGTTAGCAACTGTACTAATGCAAACGAATCTGGTGGGCTACCAAACAAGCAGTCCACA
GAGTGGTTCAGTTCAGTTGCTGTCAATTCTTCGGAAGAAACAAATCTTCTACGTTAGTCCGTAGCACGTACTACCTGGTCATG

GTCTCCTTAGTCGTCAGTCTAGGAGGAGGCTAGTTTCTCTGATCTAGTACTAGTAGTACTCTCATGCACTAAAAGTCGGTGAA
TGCATACAGCTTTACCGTGAGTGCATCTTCACAAGGATGGAAGACAAGGGTTCCAAGAAAGACGCGTTTGGTGGAGCCTGA
ATTTACACCCTTCCTCTACATGCTGCTATGAATTACTGTTAGCAAGCAGTCAAGCAGAGGGAGACCTCATGCACCGAGTCAG
TCGTCGTCTCCGAAGTCTGGACGAGGAGCCTGGTGGGAGAGGGAGGCTGCAAGTAATTCCTGTGCGCCACTGCCATGACTG
CCCTCTGGTCCTTCCACCGTCTCCCAAGATCACGGTGATGGTTCAATTCAGTCGATGTAGATGTTAGCAACTGGACCAGTGC
AAACGAGGTTCAATTCAGTTGCTGTCAATTCTACGTTACTTCGTAGCACGTACAAACCTTCAGATGGACGGGATGAAATAAG
TTATTTCCATCGAGCTTACACCGATGGAGATTCGTAATTACCGAAATTGGTTCGGTCATCATACTCTGCAGTTCTTCAAGAGA
AACGAGTCCTTCGAAATTCCAGCTTCATTATTCATCGATGCCAATGCTGCCGTGGCATAGAAACACGTTTCCAGATTCCAGAA
CGTAGAGTAATGAGAAAACCAAGCATAGAAACCCGTTTCCAGAATGCAGAGTGATGAGAAAACCAACCGTAAAACACGTTT
CCAGAACGCAGAGTCATAAAAAAGAAACCGATCATAGAATCAATGGTCCAGTCCTAAGTAACATGGGGAGGACAACTCCAG
CACAGACACAGCCAGCAGTACACGTAAGAATAGTGATTCAGCAGAGTGCACTACACGGAAACACAGACACAGCAAGCTTA
TCGTGTCCACAGACTTTCCACGAAGGAATCATGGCATGGGACCTGATGAGCGAGGGAGGCAATGCATCTGGCCATCTGCGC
GTTTCCATATGTACTACTACTGCTACATGAGCGAATATCATCATTATCAGCCAAAGAAACCAATAAGCTCTGCGCCGCCGCCG
CTGCTGGCAATGAACCCAAAGCTACCACCGGCCA

>SsLRR-RLK73

CAGTGGTGACCGGACGCTGCACCGGACACTGCACCGGACGCGGTCACTGTTTGACCGGACGCGTCCGGTCAGCGTCCAGC
GTCTGCGCGTTTTTCAGTCGGTTGATCGGACGCGAGGCGCGTCCGGTCACAGGCGACCGGACGCGTCCGGTCGTCCAGGGA
GCTTACTGGACTCCGACCGGACGCGGGCACTGTGGCGTCCGGTCAGTTCTCCAGCGCGTCCGGTCGATGGTTCGTTTTGCGC
GACTTAGTGTTGACCGGCGCGTCCGGTCAGTCACGGGACCGGAGCGTCCGGTCGTGACCAGCGCGTCCGGTCGTTGCGACC
GGCGCGTCCGGTCGGCGGTTCGAAAACTAGCCGTTACGCGACCGGAGCGTCCGGTCAGTTGGACCAGCGCGTCCGGTCAG
CGCGACTGCTGCTGATTTCAGTTTCTAACGGCTATATTCGAAGGGGTACGTCTATAAATACTCGTTGGCCGGCTCAAGGCTCT
TACTCTTGGACATTTTGACATACTTGTGAGCACTTTGAGCTAAGCAACTCCCCTCCCACTCATCTCTCTTGCTTGTTTGCATAT
TCGAAGTGAGATTGAGTGATCCAAGTGCATTTGCATTGTGAGATTGCATCTAGTTGGCACTTGGTGATTGATTTGCTGCGGGA
TTCTTGTTTCTCTTGGTGGTTGCCGCCACCTAGACGGCTTGGAGCAGCTTGGAGGCGTTGGCAAGAGTTGGTGATTGTTCTT
GGCCACCTCCGGTAATTGTGAGGGGTTTGTGCCTACCTCGGCGAAGCGCCAAAGGCAACATTAGTAAATTGCTCGTGTCATT
GCAACCTTTTGCCATTTGGTAGGTTCTTGTGGCGCCTTTTGAGAGGCTCGGTGTGAGACCGATTAGCCACCGAACCACGTGG
TGAAGAGGGACACAACGGGGACTAGCGTGCCGGCAAGCACGTGAACCTCGGGATAAATCTTCGTGTCATCACTTTCATATTG
TGATTGTTTGATCCCGGTGTTCGGTTATCTTGGTATTCATTGTTGATCATATTGGTTGGCTCAATACTTCGCCCCTCGGTGAAAC
TCTCCTAACTCAACTCCGCATTACTTTCTTAGTTATCTTTGCTTGCTAGTTAGCTTTGCTAGAGTAGCTCTTTTGTGTAGCTTGC
TAGTTGTAGTTGTAGTTACCTTCTTGGTTTGACTTGTGCGGTTGAGTGCCTTTTTACTAGAATTGTGAATTGTATAGGTGGCTT
GCAAACTCCCCGTAGGCTAAGCAAAACTCGCAGCTCGCTATTTGTTATATTGATAAACTTGTTTAGCTTGTCTTTGTAGAAAA
TTTTAAAAGGCTATTCACCCCCCTCTAGCCATCAAGACCTTTCAAAGCTTTACTCTCTCATTTGTGATCCTGATGTAAAAATGT
GGATCTTCGGGTTCTCTCTTGCGGTGTGCTCTACGGAATCGCGTAGTTTAGTGTTCTTCCTTGGGTACTCAGTGTCTAATGGA
AGACTAGTACTCTTGGGAGGACATTAGATTAGGCGGTACTGCCACATAATCTTTGCAATGATGTTTGGGTGTTCTGGTCTTCT
GGATCGGTCATGAATAAATGACATAGAAGATTGTTTGGTCATCCCCTCTTTTGTAGATTGTTTGCTGTCAAGATTTTTCTGCAT
TAGCATCCAGGTGAAGAACTTGCAACGTGGTTCTGCTCAGTCTTCCAGATGGTCTAAAAGAAGCATAAAACAAATTAGACGT
CAGTCAACGGTCAAATCCTGCTTTCTGAAAACCATGGGCTCATCACGGGGGGGCTCATGATTAGGCAGGCAGCTAACAGAA
TGATTAGGCAGAGCGCACTAGCACTACGGTGGAGGAACTTTCCATTCCATAAAAAGCAATCATGGAACCTGAGCTGAGACA
ATGCATCTGGCCATCTGCCCATTTCAGCGATTATCTCTCTCAGCCAATGCAAAGAGACCAATCTCTACGCCGCCACGCAATAA
ACCGAAAGCCATCACCGGCC

>SsLRR-RLK115

CTCTCCCCCACACTGGTAAGATCCATGGAGCACGAGTAAGATCCACTTGCGTCCGGCAACATCCTTGGAGGTGGCGCAGCG
GTGTTGCAGGGGAGTCTGCGTCTCTCTCTCTCCCCCACCTCGGCGCGGCGCCCTCCTCCCCCTGGTGACACCCTCTCTCACC
CCCGGCGACCGAGCTCCCCCACCCCAGCGGCCATGGCGCTCCCCGCGCCAGGTTCTCTCTCCTCCACCCCTTATGTTATAGTT
GTATGTATTAATTGTTTCAGATGTTTTAGAGGTATGTTGTAGTTGAATTATATGGATGTTGCAAAAGTAGATCGGGGAAAGTTG
TACATGTTGCATATGTTGCAAGCGTTTGTTCGAAATGTTTCATCTGTTCCCAGACATATTGTTACAAGCATTTGATCTAGATGTT
GTATATGTTCCACATATATGTTTACATGTGTTTTATTTGGATATTGCATATGTTTTCTAATTATTTTTTAAAAACTTTTCATGTGTT
TTTGCAAGTGTTTTAGACACATGTTTCGAATGTTTCAGTTGTTTCTTATATATGTTGCAATTGTTTAATCTGAATGTTTCAAAAG
TAGATTACGTGTGTTGTGCGTGTGGTAATGTGAGCCATATAATGTAGCCGCCTCCTGCATCTGCTGGGGCACCGTGCATGGAG
GGGAGCGGCGTAGTAGGCGTGGAGGGGCGCGTGAAGCATGGAGGAGCGACACGGGTGGGCCCGTGTATGCGTGCAGCAGG
CTGGCGTGCGCGTTCGGATGCGGGGGTCTGTCCGGATGTCCGAGCGCTAGTGCTGCCCTACTAGCTGCCCTGTTCTAATTAG
TGTTTCCATGTGGTCAGGGTGCTTGTACGTGCAAATGTATTAGCCGATGTTGCATACGAGACCGAGTGGGGAACCGTGCAGG
CCTGTGTGCCATATGTGCCAGGACTGACATCCCTAGTTTATAACAGATTAGAAATTAGAAATCCTAGAGGTTTATTGATCTCGG
ATTTGTGTTGGTGTGTGATGAGAAATTCTTGCCTAAACGTGTGCTGAAATTCTCGGAGTTCCTCTCTTCAAATCATGGCCGAT
TAAAATATTGTCAGCCTTTTTTGATCTGCCGTGACACGTCAGGTGTGAACAACAGGTTGCTTATTGCCGTGCACTATTGACAA
TCAATTGTATTAATTATATTATCACGGGATATTGAATTGCGCTTGGATATACTTGCTGGCACATATTTAAATATATTGTTTTTCTAT
GGTTGTTTGCTTGATATCAACTTCAGAGACTATTTGTGCTAGCTATTATTCTACTGTGAAAGATCGAGAAAACCGTAGACGTG
TCATCTCATCACCTTTTATATTCTTCTATCTATCATGTCATTCCACGGCTGCTACAGGACATCGTTGAATGATCTAGCATATTTTT
CTTAAATCATTCATACGAAGTAGCAGGTTTGTTGTTTACTAATATACATGATGTGCATCTCTCTATTTTTTTATTCGGTGTATATG
TGTTTTGTTTCTTATCTTTTGTATCCGAGTTCTCTAACTTAAATAGACACTCCTAAATAAACATATATTAATAAACCTTTTATGTA
TCTTTTGTTTCTTATTTTTCTCGTTGTTTCTGATCTTTTAACAAACACATCTTTATTCATTTATGTACGTCACTGTCACTTTGAAA
AAAAATAATGTCTTTAACATGCAAGTTGAACTATGGTACTACCTTTTATTTAGTAAAAAGATATTGCACTTTGAAAAAAATATT
ACATAAAACGATAGAAGCATAGAACAACTCCACGTCATCTAGTCAAACTAATCGCAAAGTGCAAACCAACGGTCCATTCCTG
CTTGCAAAAAAAAAAAAAAAAACTCCCAGTCTCCATCTAGCATAGACCAACAGGCAGCAAGCATAGTATTATACTGCAGAATG
ATTAGCCACTACGGCGTATGATCGGAATCACGTAACGTGAGCCAAATCATCATCATCATATCATAGTAAGCCAA

>SsLRR-RLK218

GTGCCGGCAAGCACGTGAACCTCGGGATAAATCTTTGTGTCATCACTTTCATATTGTGATTGATTATTCCCGGTGTTCGGTTGT
CTTGGTATTCATTGTTGATCATATTGGTTGGCTCAATACTTCGCCCCTCGGTGAAACTTTCCCAACTCAACTCCGCTTTACTTT
CTTAGTTATCTTTGCTAGCTAGTTAGCTTTGCTAGAGTAGCTCTTTTGTGTAGCTTGCTAGTTGTAGTCGTAGTTACCTTCTCGG
TTTGACTTGTGTGGTTGAGTGCTAATATACTAGATTTGTGAATTGGATAGGTGACTTGCAAACACCCCGTAGGCTAAGAAAAA
CTCGCATCTCGCTATTGGTTGCATTGATAAACTTGTTTAGCTTGTCTTTGCAGATAATTTTAAAAGGCTATTCACCCCCATCTAG
CCATCAAGACCTTTCACGTTGTATCACGTTATTGTAGGCCATGTTGTGATGATGATGCTTGTGACCACTTTCTCATAAAGAAA
AAAGATGAATAATGTCCATTGTGGTTCTTGGGAGATTCTCCCACATGCGTAGAGGGGAATCGATGGACCAATTTCACATAATA
AGATATCAAAACTCAGAATACTTTTTATGATTTATTATAATCTTGTCTACTGGAATATTTTCTTCTAGACCACTGCCTTGAAAAC
CTGATGATCAATGTAATGAACCCAGCCAGTATTGTTGTTTCTTCTTCATCAATGTCGATTGTGATGCTAAAACGCTCCGGCCAA
GAACAAGGAAAAATAACGACTCGGTGGCCTTAAGACTACAGCAAGTAGACAGGCGGAGATGGCTAAAGTGGTGGAACTCG
AAATATTCACACCCAGGGCAACTATATGTGTGTATTAAAAAGCCAAATTGGTCTTCATACCAACAGATAGCAATAGAAGCGTT
TGCTGTTTTTCTTCTCCGCAACTTATTTGTAAAGAAGTCCTTGAATAGCTTGAGGAATGACTCTTTATTTCCTTGAATAGCTTG
AGGAATGACTCTTTATTTAATCTAAGTTGTTACATCATCATAAATTCAAAGGTGTAGAAATTTTGCCTTTATTACTAATTATGTT
TTACATGGACTTTTTATTTAGGCATTTTTCTTTCCAAACTTGGGCATAAATCAAACTGCTAATTTTTAAAATTTAAATTCCAAT

TTACTACTTCCTTTGTCCCAGATTATAAGACATTTTGGTATTTCTATATTCGTAGCTTTTACTATATATCTAGACATAATATATATAT
AGATATATAGCAAAAACTATGAATCTAGAAAAATCAAAACGTCTTACAATTTAGAATGGAGGGAGTATTTATTAATTGTATTAA
GCATGGACTTTTTGTGTAATTTAAGCCGTAGGAGCCATTGATCTTAATGACGTTAAAATCCTAGATATAAAATCTTTGGTATGG
TTGAACTATCTTAGCTGGAGAGGGGGACACAAACATGAAGTGTACCAAACTTTATTTTGCTCCTTTATTGTAGATTAGACTGC
TGCTGCAAGCGTGACCGATGAATGAAGTCACCGGGAGACATCAACTAGGGGTGACTAAGAGCCAACAGTTTGGCAAATTTT
GCCCAAATTTCGTTGATTGTCAACTCCCAAAATAATATGGGAAGGAAATTAAGACGTGACTGAAGACGCACAAGTTTCACGA
ATTCATATTTGCATGTCACTACTCGCGAAGAAATTAAGACGAACCCTGTCGCCGTGTCATCAAACGGGTCTCTCCTCCGATCC
GACCGTGTCCATTCATTCGTGTTTTCGTCTGAAGCCTCAAAATTTCCAACGACGTCCAGCGTAAAAAGGAAGCATTCATCGA
CGTTCTCTTCACGCACATCACATCCATCCAGCGTCAAAAAGGAAGCTCAAGTTTTCAACGACATCGGCACGCGCAATGATGA
TCGACACAGACGTCCTGCTACTACTCAACCATATAGCTCCTTCTCCCACCAATTAACCAATGTAATGCAAGAAGGGCA
>SsLRR-RLK274
ACATGGACGGTAGAGACCAACTTTCAGGGACATGGACGAGTAGACCAACTTTTTTAATGGCATACAAGTAAAAGACTCTTG
ATTTTCATGGATGACATTTTGATTTACTTTGCTACTTTAGAGGAACATTTGATTCTGCTACTACAAGTATTTCAAGTCATCAAA
GATCATCAGTTTTTTTCTAAAGAGATCTAAGTGCTTCTTTGCTCAAAAGGAGGTTGAGTATCTTGGGCATCGTATCTCAGGGA
AGGGTGTGGCCACTGAACCATCAAAAATTACTGCAGTTCAGCAGTGGCCTAGACCAAAGAACTTCAAGGAGTTGAGGGGTT
TCTTAGGGTTGACAGGATACTATAGGAAGTTTATTAATCACTATGGAATGATCCGTAAGCCACTTTCTGATCTGCTAAAAAAGT
CATCTCCATTTGTGTGGACATCTCTCTATTACTGAAACAGCATTTATGCAACTCAAAGAAGCTCTAGGGAAGGCCCCAGTGTT
GGCAATTCCTGATTTTCAAAAGCAGTTTGTGTTAGAAACAGATGCTAGTGACATTGGCTTTGGTGCTGTTTTGATGCAAGAA
GGCCATCCAATTGCATACCTTAGCAAACCTATGTGTGCTAAGAATCAAGCCCTCTCAACTTATGAAAAGGAGTGCATGGCCAT
TATTATGGCAGTGGAGAAATGGAGAGCCTATCTGCAAAATCAGGAGTTCATTATCAAAACTGATCATAAGAGTTTAGTCCACC
TCGCTGATCAGAGGGTAACAACTAAGCTCTTTGGTGATGCTTGAGGAGGATATCTCTGCAATACCTGACAATCCAGTGGCTA
GTCTTCAGCCGGTGGCTTTTCTGGACTCTAGAATGGTGAAGAAAGGAGCTTCAGTACTGTCTCAGATTAAGGTCCAATGGGC
GGGCATCTCTGATTCTCTTGTGACCTGGGAAGAAGCTTCAGATTTGCGTCGCCGCTTTCCAACTTGTGCAGCTTGGGGGCAA
GCTGCGTTTCGAGGGGGAGGTAATGTCAGGATCAGCAAGCATCAGCTAAGGAAGACGACTACTTTGGGCGTCCGACAACAG
CTGCCACGGCGACAGGTGATTCTGTTAGCTCTGTTAGCAAGACTGTTAGTGGCTAAGCTTGGGAAGTTAAGGCGGTTGCCTA
ACGTCTGTCATGGCGTGTCTGGGCTGAGGTATATAAAGCCCTGTAACCGGTTGTAAACGATATGTAAACAGAACCTGAACCT
TCCTCTGAATCAATTCGACTTTGTCTGTTCCCCAACCTCTGTTCTTGCTCTTCCCAATCTCAATTCCAATTCCAGCTATCGTCT
CGGTTCCTCGCGGAATCATGACAGCCATATACCATATACCAAGAAGGTTATGAAGATAAGAGTCGAGCCGGAGTAATGATTAT
ACGCCTTGGTACTACTGCCCGCCTGCTATTGTGGCCAATTTCAAAAACGTGTTTACGCCTGGTAAACCGGAATGGAGGGAGT
ATGTGCTAGACACGTTTGCGAAAAAGATGTAGCATTGGACCTACTTTGAGCACCGTGTAAGTCTATACATGAACCTATGCAG
GCAATTAAACACGAGTAAACTGCATTGCACAAGCCTAAATATAGGCTAATGCAGGCAACTGATCAGAGCCTATACAGCGAGT
TTTGTAGAACTGGTACACAATTCAGTTATTCCGTGCTGCCTAGCTTGGTTTAACATGATCAGAAATGCAACGTAAAAATACAC
TGGGATAACTTACGTGGAATTTTTAGACAAAATATTCACTTATGGTGTGTGCTTAAATGGAGATAAAATTATATACTGCCAAAG
TGCTGAAACTGGGTGATTATATTAGGACGTGTTTGCTATTTTGCTGCGGATTTTCCCCAGAAAAAAATTTTGTCCACTATTGTG
TCTGAAGTTTGCCTTTGTTCTTTATCTGCTCGTATAACAGGACAGAAGTTCAACAGCCCCAGCTACCATGCTTCCTACAATCG
CACTACATCTTCTGTTGG
>SsLRR-RLK289
CTATCAATATTTATGCCACCAAATAAGTATCATTAGATTCATCATGAAATATATTTTTGTTGCATACCTATTGGTACGATAAATGC
TGATACTTTTCTCTATAAAGTTTGCTAAACATAAGATAGTTTAATTTATAATAAAGCTAGAGTTATATTTTTTGATAGACGAGTC
GACAATAGTATTCTATTTTTTTAAACTTATGATAGTTAATTACTATCAACTCTTGCTACTGCATGGACTAGAATATTGTAATACAA
CTAACCAAAATTAGCTTGGCTATCTATCAACTAAAGTTAGGGTCCATATAGGTACTATGTAATCCTATCTTGTTAGGATAAACTT
TGGTTATCAGTTACTTAATATGCCTTTCCATCCATATTTATCGTTTTCCCTTTCGCATGACCCCATCGTGTGTTTGCAATATGTAT
TTAGTTGAAACTTCAGCTTTGGTAAACTTATCTCTTGTTGCAGCCCATGTGCTAGAACTTCACGAATCTAGATTTTAGCTTTCT
AGTTTCCTTTGATCTTTTTAGGTATTGCACTTTTTTACTCATTGTGTTAAGTTGAAACTTTCTTTTAGAATTGTTGTAAAACATG
AAGTCTAGATCCTAGACCACACTTTGATTAGTTCAGTGGCGTAACCATGGATTTCAACCTATACCGCAATGCACAAGATATTA
ATTTTCTCCTATCAAAAAATATGCACAGTAGATTAAACTATAATAAAAATGTTATAACGTTTCACTTAAAATGTACAAATTTACT
TAAAGGTACAACTTAAATAATTTATTGTACAACTTAAAATTAGATGTCATAGAGTATTAATATAATTTGATTAAAATATATTGTTA
GTGTCATTGCCTGTGATTGGTTAAGGAAGCACTTTAATAATTTGTGTATACTATGAAAAATATACTTATATACCACGTGCATGTG
CTTCGCATGCATGCTGCCACACAATTTGTAGGCATACTGCAAAATACCTTATGTGGATAATTTAGTTGTAAAACTTAAAGGGCT
CTTTTAGGGGCCGTTTGGATGGCGTCCAGAATTTGCCGCGCCACGCTGCACCTGCCACGCCACACTTTCGCACCGCGTTTGG
TTCGCTGCCGCAAGCTGCCGCAGTTGCGGCACGCGTGGTCGTCTAGTTTATTTGGACTGCCAAAACTTGCCGCAGTTGCGGC
GGCCGATTTCACCGCCGAACTTTCAGCGCGGCCACAGCCTCGGCGCGGCAGCTTCTGGACAGCATCCAAACGGCCCCTTAG
TTTGTTTCTAATAATGGAAGAGGTGGTAATTAAAATACGAACTTTACATAACCTTTCCTAATGATAGAAATGAGCAATTTAGAT
GTAAATTTTGGGAGTCACTACAGATTATGTGTCATAATAGAAAATGTTGTTAAGTTGGATGAAAACTTAGGACGTTAATTTTGT
TTGTTCTAGACAATGCCAAAGTTGGTTAATTTGGAATAACACGATCAAACTATTGTTATTGTTAATGTTGATTAGAGTCTACCA
AATTATTGGCAATACGTTTTTAAAGAATTTATAGGATGCATGTATGACATCACAACACATGGTATGCAAACAGCATAGACATAA
TTTAAGTTAATGTTTCAACTAAAACATATCGAAGACACGTAAAGGTGCAATACACAGGGGAAAACTATAAGCAAGTTATCATA
ACGGGATAGAGGTTATAAAATATATATGGATATATGATGTTAGAAAATGAACAAATAAATATAAGCAATGATAAAGGATTACGA
TTAATTGTGTGTATTAAATTTATCTACGAACATTTTATACTTATTTCTCATATGATTTTAGCTTATGCAAGAGCACGTACAGGTTG
ATAGACTAGTTATAACAGTAACACTTATCTGAGCTTCTTGCACTTCTTATTTGTGCATATAATCAGGATCCATATTCAACCGGCG
TAACAAGACTACCTATGAATATGATGGTTCCGGCAATCCTAGTACAGTTTCTCTTAGTGCT
>SsLRR-RLK305
ATTGGTTTGCATGATGCGGTATCAGGTCATCTTCCGCTACATCCGTATACAGTTAATGCTCACCAAAAAATATCTTGTTAGGTT
TTCTAAACTGTTATAGTAGCCGTACGAAAATAATTCTTCTGCTAGGTTTGGAAAGTATTAAAATTGTTGAACCAGGCTTTTAGG
AATAAAAAATACATATAGCACCTCATTGATTCTCACAGAACAGTGTCGAGTCGGCGTTCAGTATAAATTGAGGAAAGGTAGT
CTCTTATCTCGCAGCCGTCTGCCCCTTGCCATTTGCTTCCTTCTATCGGTTTGCAGCTTTGCACCAAGCCTCCATTGGACTTCT
TCTTCGAGTGCTGCAGAGTGGTAAGAGCATTTTATTTTGCCCTTGTTCGTCCCATGTCTATCTGGCTTTCATCTCTTCTCCATA
ACATGTAACGCACCTGATGGGAAACTGTTTGTTTCCTTAAATAAATTAGTAACCAGAGCTTCTAACAGTGCACAGAAATAAA
GCTTTAAGTTCTAAATTTCCCCAGGTACTTTTGGCAAACATTTATCTGAACCTGCTTTTTGTTCTGACTTGTGCATATAATCAA
GATCGAAATTTGACCAAGAGATCAGACCTAGGCACGATGCTGCTTCCTACAATTGGACAGTTCATGGTGCTCATGGCGTTCT
GTACACAAGTTGTCATCTGCTCCTTCAATGGAAACTCCACGGATAAGGTATCACTATTGGAGTTCAAGAACACTATCAGTGAT
CCACGACAGGCCTTGATATCTTGGAATGATAGCACACACTTGTGCAGTTGGGAAGGTGTCCAGTGTAGTGTAAAGCACCCAC
TTCGGGTCACTTCACTTCGTCTTGACAATCAAGGTTTGGTTGGGCCAATATCTCCTTCACTTGGAAACTTAACATTCCTAAAA
ATCCTAATACTATCGACCAATTCATTCGTCGGCGAGATCCCTCCATCCCTCACTCGCCTGCATCGCCTCCAAATCCTAGATTTA
AGTAATAACACGCTTCAAGGAATGATACCCAGTCTTTTAAATTGTTCAGATCTCAAGGAACTCATTTTGCAGAAGAATCAGCT
AGCTGGACAGATTCCTGAAGATTTGCCTTTTGGCCTTCAAAAGCTGGCACTTACCCTTAACAACCTTACTGGAAACGTTCCA
TCTTCTCTTGCCAATATCACAACGCTGAATTTAATTGCTTGTATGGGTAATAATATTCAGGGCAACATCCCAAATGAGTTCACA
AAATTGACAGCGCTGCAAATCCTGTACGTCGGCTTCAATAAGTTGTCAGGTCAATTTCCGCAGCCTGTCCTGAATCTTTCCAA
TCTACTAAAATTTAGCGTCGCTTTCAATGATTTTAGTGGAGAGGTACCACCCAATATAGGCAATTCTTTACCCAATCTCCAAAT
GCTAGAATTAGGTGCCAACTTCTTTCATGGCCATATACCATCTTCATTGACAAACGCCTCCAAGTTAACTGAAATTGACATATC

ACAAAATTCTTTCAGTGGGGTGGTGCCTAGCTCCATCGGCAAACTTTCTCAACTATCTTTTCTTAATCTCGAGGTAAATGAAC
TCCATGCAAAAAACAAGCAAGATTGGGAGTTTATGAAGAACTTAGCTAATTGCACTGGGCTACAGATGTTATCGGCAGTAGA
CAATCGTCTAGAAGGCAATTTGCAAATGTCACTAGGAAACCTTTCCAGTGAGCTCCAGTATCTACACTTGTCAGGAAACCAA
CTATCAGGGGATTTTCCTTCCAGTATAGCAAACCTCGGTAACCTGTTAATTGTAGCATTGGCCCGGAATCAGTTTACAGGTGT
GGTTCCAGAATGGCTTGGGACAATAAAAAATCTGCAGATTGTGTCATTAGGCAATAATTTCTTCACAGAAGTCATTCCATCAT
CCCTTTCAAATCTGTCTCAACTAGTAGAGCTCCATCTAGAATCGAACCAGTTGAAGGGACACATACCCTAAAGCCTGAGCAA
CCTCCAG
>SsLRR-RLK245
GGACCTGCCGGTGCTGAGAGCTTGTCTGAATAAAAAATTCGCCGCCGCTGTCTTCCCTAGTTCCAGTCGAGCGCAAACTGC
GGCGCCGTCGCCGTCCGGCCGTCCCGTCATGGACAGCCCCGCCCTCTTTGTCTTCGTCTCCTAGCCGAGAGCCGCACGCTCT
TCATCTTCGTCTCCTCCCCGAGGGCCGCACTGTCGCCACCCCCATGCCAAGCGTCGCGCCGTCCCGTCCCTTGCCGAGCTCT
GCGCCGTCACCATCCCTCGCTTCTAAAAAGACCGTGCCAAGCTTGTAAAAAGACCTCCTTAATCAATTCCATCACGAGATGA
CGTAGTAGAGGATGGCAAACAAATTAGGAGAATATTGATGCAATCTGAACACACATGGAGAGGAGCGCAGACCTGCGAACA
TGTTCCTGACGACACAACACAAGCAGCAGCCAATTGCCGACGCCTCACCTTCGTGCTCGCGGGAAAAGCTAAACGCCGAC
GACGATGCCTCACCCTCGCGGCCTTACTCGCGGGAAAAGCAAACCGAAATCGCGCCAAAAAGGAAAAGACGGCACCAAAA
CGGGGGAGAGATGGAGTTATTCTTGGGCCGAGGCCCAGATAACAAAGGAGCGCTGCAGCCAGACCTGCAGGCTCCGAGCG
CATAGGTCGCATTCGTTCGATCCTGAGCTGACGGCATAGATGTGCTGACTGCACGGCGCAGTCGATCGAGGTGAAGTTAGAC
AATCTTATTCCAGCGGTGCTGCTGCGTGATGGCGAGTGCTGTGAGTGGGTTGGTCTGTCCAGACAGACGGATGCTCGGGTCT
GATCATTAATGATAATTACATCGAAAAATATGTAAAATAGAACGGAAATTTTCGAGTAAAAATGGATCCCTAAAAATGGTCGA
CAAAAAGGAAAGGTCGGTCGGGGTTTCCTGTCCTGTTTTTATCTCATGGACTAGTAATGCATAACAAGAAATTAAAATCACTA
TTACTCTTCCATCGATACTCACCACCGTGTGAAGCATGGCGTTGCACATAACTAGACATTTTTTTGTCCACATTGGATACATAC
AAGTTGACTGGACTGGCAAATTTATCCGAATCGTGCCCACACGATGCGTTCTGGAAGACCGATCGTCACAAGGAACCAAAT
CTGATTTGGGAACGAAAATTCAGCGTCGGTAGACCATTCCACGAGCTAATTTTCGTCAGCAGCGAAACCGCACCAGACAGG
TCGCACATCAGCCTGGAATATCTCTGTCGGTTTCTTGCTGAAAAATACTCTGGGATTCGAGGCTGGACACGCCGCAACAATC
TTGTCCAGCTCAAACAACAGCGACGGCGCTGGGCCACGGGAAGGAAGCTGAAAGGTAGCCGTGACTGGTCAGTTTGACGG
ACTCTGGGTCTTCACTGCAATAGCGGTCAAGCTAAGCTAAGGCACGGGTAACGCCAAATATGCGGATGTGACGCCACTGCA
AAATTGTTTTTAAACTCCCTTGGCCTCAGCCAGCTGAGCATCCAGCTCAAACTTCACACACTAGTCCACCACGGAGCAAGC
AAAACAAGCAGAAAAGGCTGTCGTCGTTATTGTTCACGCTCCGGCCGAGCAGAGGTCCGCTACTCTTCTCTCTGCCTTCGTG
TTTCATTTTATTTTGGGCCAAGTTTTACTGTCAAGTTTATCCTCTTTGTTGTTGCCGATGAAGTGTTCGACAAAGGCAAATTCT
CCTACTTTCTGTAATACTACTAGTCTACTAGAGGTCTAAACACTTCTGTCTGCAGTATTATAGGAGAAACACGCTTTAATCAGT
TAGGGCAGGGGCGGACGGTTGGGGGTATAAAGCAGCTGCGCCTTTGAAACGTGAACATAGAAGCCAACGTGTTTATGATTC
TGCGTTTTGCAGCTAACGAAAGCCTTATGTTCTTTAAACAGGCCATGAAACTCTACATGTTTATACAAATGTTGGTACAGCTG
CTGCTGTTTTGCTGTGTTGGTAACGTCTGTTGCTCTA
>SsLRR-RLK106
AAAGTTAAAAGAACTACATAAAATCCATTGGAATATCATCACAGATACATAAAAGATACTACATAAAAGACTACTGAGGAAG
ATGATTAGAGAGAGGACTAAAATGATTAGAGGAGTTTATTTTGCACCCCGTCTATTCTAAATTATAAGACGTTTTGACTTTTCT
AGATTTATTGTTTTTGTTATGTATTTAGATATACACCATGTCTAGATACATAGTAAAAGAATTGTATCTAGAAAAACTAAAATGT
CTTAGAGGAGTAGCAATTAGACACTGTACATTATGAGCTATAGGCTTTTAGATGGTGTCGATCTAATATGGCATATTATTTGCCC
TCATGCCTCATAGGACTCCTCTTCCCCGTGCTAGGGTATTTTAATACTCCAACACTTTGATTTTTTTCGCCTTTATATTAAGTTC
AACTAGTGCATGACACCCTCACCTTAACCTCCCCCTCTTCCATGGTTTCCAATGCTAGAGCCACTATACTACAACAAAACTTT
TGACGCATTAGGCCCTATTTGGTAGGTCTCTGGTTCTGGCTCTTCATGTGAAGCAGCTTCCGTGGTGGAGTAGAACCACTTTG
AAAAACTTTTAGCAAAAAGGACTTCCGTGACAGTTTTGTAATAATTTATATTAAATTTGTAAGTTGGAACCAAGAGAAGTTAT
TTTTTTCGGTTCTGATTGCTCTTTACAAAAACAACTCTAGCTCCTGCGGGCTCCTTTCAAGGTGCTTTTTTTTATATGCTCATTT
GACGGGACTCCTTGCAAAGAGACACAAGAGCCTTGCCAAAGGAGGCCTTAATGTCTTCCAATTTAACACCCTATCTTGCTAT
ACTCGGAGAGGTCCCTTCCCCAAAACTCTAATCTCTAGACCTAACCGTTGCTAGTACTCTTTTGCAGGGTTTAAAGCAACTTC
AATTTCACCTGCAAAAACACTAGCCATATCACAGTTCACATGAACTCCTTTTATTTCTCTCATTCAAATAAACTAGGAGCCAA
TAAAGTTAGTTTTTTTTGGAAATAACTGAAGCCGGTTGAAACCTTGCCAAATAGTTTATAAGCTTGCCAGAGTAGTGGAAAA
CCCAACTCAACGAAAAATGCAGCAAAAGGTTAGCAAGGTTTTCATGTTTTACCCCTTTTTCTTCACAGAAAATACTTGGCGT
ATATAATCTATGCGTGTAGTTATCAAAAAACCTATGCGTATAATTCCGATCAAATCTCAATAATACATGCAGATTTGCGTGGCCT
GAATAAGTATTTCTACTAAAAAAAATGAATGAAAATAGGGAATCTGTCGTTTCAAAAAAAAAATCTCAATAATACATCAGGTC
AGGCGTGTGGCCCCATGCTGCACGTCAAATCATTCCAGCCACTTCATTCGTTTTCGTTCCCACAAGTCACAACCATGGAGGG
GGAGGGCCGAGGACGAAGGCAGGAAGGATTCTGAATCCATCATCCATAAAAAGGACGCCAACCACTCTCTTTCTACGAACT
CCATCTCTCTCCTCTCACCTCTCTTTCTCTATCTCTCTCCTCGGCCTTGCTCTGCTCTACTCACTCCCCCAACTGCCAGCCGAA
TTCTCTCTCTCTCTCTCCTCTGTTTAGCGAGCAGCTCCGCCATTCCCCAAGCACAACAACTCCTTCCAGAGGCGGACGCAGT
CGGTGCCATTCAATGCCCTGACGGCTGCCCGGCCCATGCACTGAGACTGAGACACAGGGCGCCTCTGCTGTCTGCTCTCTCT
CTCTCTCTCTCTCTCTCTGAGACACGACGCAGTCACCGCCACCTCCTTTAACTCCCCTGCCTCGTCGCTTCCCGACGCTGACT
CGCTCGGGAACCCGTGCGCCCATGCACGCACCCGCCGCATACGTGCGCCTCCCTACTTGCCCCTGCCCTGCTCATGTACCCT
CCACAATCTTACACCTAGGAGGAGGCTACGAGCTCAAGCTCTTGGCTAGCTGTGGCGAGCGGCGAGCATTGAGTTGAGGGG
CACCGAGA
>SsLRR-RLK86
AAAATCCATTGGAATATCATCACAGATACATAAAAGATACTACATAAAAGACTATTGAGGAAGATCATTAGAGAGAGGGCAA
AAATGATTAGAGGAGTTTATTTTGCACTCCGTCTGATCTAAATTATAAGACGTTTTGACTTTTCTAGATTTATTGTTTTTGTTAT
GTACTTAGATATACACCATGTCTAGATACATAGTAAAAGAATTGTATCTAGAAAAACTAAAATGTCTTGGAGGAGTAGCAATT
AGACACGGTACATTATGAGCTATAGGCTTTTCAATGGTGTCTATCTAATATGGCATATTATTTGCCCTCATGCCTCATAGGACTC
CTCTTCCCCGTGCTAGGGTATTTTAATACTCACACTTTGATTTTTTTCACCTTTATATTAAGTTCAACTAGTGCATGACACCCTC
ACCTTAACCTCCCCCTCTTCCATGGTTTCCTCTGCTAGAGCCACTATACTACAACAATCTTTTTACGCATTAGGCCCTATTTGG
TAGGTCTCCGGTTCTAGCTCTTCATGTGAAGCAGCTTCCGTGGTGGAGTAGAACCACTTTGAAAAACTTTTAGCAAAAAGGA
CTTCCGTGATAGTTTTGTAATAATTTATATTAAATTTATAAGTTGGAACCAAGAGAAGTTATTTTTTTTTCGGTTCTGATTGCTCT
TTACAAAAACAACTCTAGCTCCTGCGGGCTCCTTTCAAGGAGCTTTTTTTATATGCTCATTTGACGGGACTCCTTGCAAAGAG
ACACAAGAGCCTTGCCAAAGGAGGCCTTAATCTCTTCCAATTTAACACCCTATCTTGCTATACTCGGAGAGGTCCCTTTCCAA
AACTCTAATCTCTAGACCTAACCGTTGCTAGTACTCTTTTGCAGGGTTTAAAGCAACTTCAATTTCACTTGCAAAAACACTAG
CCATATCACGGTTCACATGAACTCCTTTTATTTCTCTCATTCAAATAAACTAGGAGCCAATAAAGTTAGTTTTTTTTTGGCAAT
AACTGAAGCCGGTTGAAACCTTATCAAATAGTTTATAAGCTTGCCAGAGTAGTGGAAAACCCAACTCAACGAAAAATGCAG
CAAAAGGTTAGCAAGGTTTTCATGTTTTCCCCCTTTTTCTTCATAGAAAATACTTGGCATATATAATCTATGCGTATAGTTATCA
AAAAACCTATGCATATAATTCCGATCAAATCTCAATAATACATGCGGATTTGCGTGGCCTGAATAAGTATTTTCATTTAAAAATA
AATAGAAATAGGGAATCTTCCCTGTTCAAAAAAAAAAATCTCAATAATACATCAGGTCAGGCGTGTGGCCCCATGCTGCAGG
TCAAATCATTCCAGCCACTTCATTCGTTTTCGTTCCCACACAAGTCACAACCATGGAGGGGGGAGGGCCGAGGACGAAGGCA
GGAAGGATTCTATCCATCATCCATAAAAAGGACGCCAACCACTCTCTTTCTACGAACTCCATCTCTCTCCTCTCACCTCTCTTT
CTCTATCTCTCTCTCCTCGGCCTTGCTCTGCTCTACTCACTCCCCCAACTGCCAGCCGAATTCTCTCTCTCTCTCTCTCTCTCT
CTCTCTCTCTCTCTCTCCTCTGTTTAGCGAGCTCAGCCGAGCAGCTCCGCCATTCTCCAAGCACAACAACTCCTTCCAGAGG

CGGACGCAGTCGGTGCCATTCAATGCCCTGACGGCTGCCCGGCCCATGCACTGAGACACAGGGCGCCTCTGCTGTCTGCTC
TCTCTCTCTCTCTCTCTCTACGCAGTCACCGCCACCTCCTTTAACTCCCCTGCCTCGTCGCTTCCCGACGCTGACTCGCTC
GGGAACCCGTGCGCCCATGCACGCACCCGCCGCATACGTGCGCCTCCCTACTTGCCCCTGCCCTGCTCATGTACCCTCCACA
ATCTTACACCTAGGAGGAGGCTACGAGCTCAAGCTCTTGGCTAGCTGTGGCGAGCGGCGAGCATTGAGTTGAGGGGCACCG
AGAAG
>SsLRR-RLK105
TCCATTGGAATATCATCACAGATACATAAAAGATACTATATAAAAGACTACTGAGGAAGATGATTAGAGAGAAAGAGAGGAC
AAAAATGATTAGAGGAGTTTATTTTGCACTCCCTCTGTTCTAAATTATAAGACGTTTTGACTTTTCTAGATTTGTTGTTTTTGTT
ATTTACTTAGATATACACTATGTCTAGATACATAGTAAAAGAAATGTATCTAGAAAAACTAAAATGTCTTTGAGTAGCAATTAG
ACACTGTACATTACGAGCTATAGGCTTTTAGATGGTGTCTATCTAATATGGCATATTATTTACCCTCATGCCTCATAGGACTCCT
CTTCCCCGTGCTAGGGTATTTTAATGCTCCAATACTTTGATTTTTTTTCGCCTTTATATTAAGTTCAACCAGTGCACGACACCCT
CACCTTAACCTCCCCCTCTTCCATGGCTTCCACCGCTAGAGCCACTATACTACAACAATCTTTTGACGCATTAGGCCCCATTTG
GTAGGTCTCTGGCTTTGGCTCTTCATGTAAAGCAGCTTCCGTGGTGGAGTAGAACCACTTTGAAAAACTTTTAGCAAAAAGG
ACTTCCGTGACAGTTTTGTAATAATTTATATTAAATTTGTAAGTTGGAACCAAGAGAAGTTATTTTTTTCGGTTCTGATTGCTC
TTTACAAAAACAACTCTAGCTCCTGCGGGCTCCTTTCAAGGAGCTTTTTTATATGCTCATTTGACGGGACTCCTTGCAAAGA
GACACAAGAGCCTTGCCAAAGGAGGCCTTAATCTCTTCCAATTTAACACCCTATCTTGCTATACTCGGAGAGGTCCCTTTCCA
AAACTCTAATCTCTAGACCTAACCGTTGCTAGTACTCTTTTGCAGGGTTTAAAGCAATTTCAATTTCACTTGCAAAAACACTA
GCCATATCACGGTTCACATGAACTCCTTTTATTTCTCTCATTCAAATAAACTAGGAGCCAATAAAGTTAGTTTTTTTGGCAATA
ACTGAAGCCGGTTGAAACCTTGCCAAATAGTTTATAAGCTTGCCAGAGTAGTAGAAAACCCAACTCAACGAAAAATGCAGC
AAAAGGTTAGCAAGGTTTTCATGTTTTCCCCCTTTTTCTTCACAGAAAATACTTGGCGTATATAATCTATGCGTATAGTTATCA
AAAAACCTATGCGTATAATTCCGATCAAATCTCAATAATACATGCGGATTTGCGTGGCCTGGCCTGAATAAGTATTTTCATTCA
AAAATAAATAGAAATAGGGAATCTTCCCGGTTCAAAAGAAATAAATCTCAATAATACATCAGGTCAGGCGTGTGGGCCCATG
CTGCAGGTCAAATCATTCCAGCCGCTTCATTCGTTTTCGTTCCCACACAAATCACAACCATGGAGGGGGAGGGCCGAGGACG
AAGGCAGGAAGGATTCTGAATCCATCATCCATAAAAAGGACGCCAACCACTCTCTTTCTACGAACTCCATCTCTCTCCTCTG
CTCCTGTATCTCCACCTCACCTCTCTCTCTCTCTCTCCTCGGCCTTGCTCTGCTCTGCTCACTCCCCCAACTGCCAGCCGAATT
CTCTCTCTCTCTCTCCTCTGTTTAGCGAGCAGCTCCGCCATTCCCCAAGCACAACAACTCCTTCCAGAGGCGGACGCAGTCG
GTGCCATTCAATGCCCTGACGGCTGCCCGGCCCATGCACTGAGACACAGGGCGCCTCTGCTGTCTGCTCTCTCTCTCTCTCT
CTCTCTCTGAGACACGACGCAGTCACCGCCACCTCCTTTAACTCCCCTGCCTCGTCGCTTCCCGACGCTGACTCGCTCGGGA
GCCCGTGCGCCCATGCACGCACCCGCCGCATACGTGCGCCTCCTTACTTGCCCCTGCCCTGCTCATGTACCCTCCACAATCTT
ACACCTAGGAGGAGGCTACGAGCTCAAGCTCTTGGCTAGCTGTGGCCAGCGGCGAGCATTGAGTTGAGGGGCACCGAGAA
GATGGCAAT
>SsLRR-RLK435
TATAGTCTAAGTAGGCTTAAAAGATTCGTCTCGTCCATTTCGTCTAAACTGTGTAATTAGTTTTATTTTTATCTATATTTAATACT
TCATGCATGCATTTAAAGATTCGATGTGACGGAGAATCTTGAAAAATTTTAGGAACTAAACAGCCTATAAAGTATTAACCCAA
TCCAAACCTGCAGGCCCCACATGTCAGCTGTGCCATGCTCCTCGATCTATTACTCGAGTTCTACTGCTCTAGGCCAACCAGAG
AGGCCAAACCAGTCCAGCTCTAGACCAGCCGAACCAGCCCAAACCAGTCAATGCTTCAAGAGAGAAAATCACTGTAGTCG
TTGATGTGACCTCTCAGTGTCATTCTCCCAATCTCTCTCATCATAAGAAACCAGGGCTTGTTAGATTGCGAAAAAAAAGCG
AACGTGATGAATAGTACCATTTTTGTCTTATTTAACAAATATTATCCAATCGTGGATCAACTAGACTCAAAAGATTCATCTCGT
GATTTCCAACTAAACTGTGTAATTAATTATTTTTTTTACCTACATTTAATACTCCATGCAAGCGGTTAAAAATTGATGTGACGG
AGAGAGTGAAAAAACTTGGAATTTGGATGGCATCTAAACAAGCCCCCACTCTCTCTAGCGCTTAAACAGCCATTCTCTTTCT
CTCTAAACTCCAAACTAGTCCTACTAGTAGGAAGCTGCTCCTCCATAGCCCATAGCCCCTGCCCTGCCACTCTATCGCCCAGC
CATACAGCTACTCGCTGTCACTGAGCCAGCGGCGCAAGTGCGCAAAGCCGAGCGCGAGAACCCACGAAACCAACCAGAGC
TCCGCCCCATTAAGGCAGACAGGCTCGCTGCAGCACGCTTCGCGCTCCATCGTCGCTGTCCTCTCCCTGTAATGTCACTCCC
CCGATGCCTGTCTGCAGCTCAGTGGCCATGACGACGACGGCCGCCCGTGCTCTCGTCGCCCTCCTCCTCGTCGCCGTCGCCG
TCGCCGACGATGGTTGGTGATCTGTTTCTTTTTTCGATATCCATGTCTCTTTATTTCTTTCTTCTCTGGGAGCCCTCTGGTTGCC
TACTGGGGAGCAAAGATTCAGGGGGTTGGGTTTGTTAGGATTAGTATTTTATTAGCGCTGCAAGTACGCGCTTTTGGAGGCAT
GGTGATTCGCTAGTGTTCCATTCCCATTTTTTTATTGCCGTATAAGCCCGTAACCCGAGTCTAACACTTAACTAATCCTTGTGA
GCTGTGCCTCTTCTTCCTTGCCTAGGAGCTTTTAACTAGTCTGCCCATGTTTCAAAATGTGATCTTTTTTTTTCTCTTCAATCAT
GTTCAGTTTTGCTTTTTACATGTGCCTTTTGTGCCTTCTTGTGCTGCTTATTGGAGCACATGCTCAGAGCATTAATATTAAATCG
CTTGAGCTTCGATTCTTGTTTCTTTATTTGGCCCTTCAGTGCTGGAAGCTTGGTCTGTTTTGTTTAGTCTGTTCTATTTTTGTGT
GTTTTAGCTGTGTTTGGTTTGCTATTCTGGAGTATGATCCTGTTTCCTATCTGAATGAATCCAAGTAACTTCATATTTCACCATC
GTGTCATATTTGGGCTTTCCAAATCTGATGTTCCCCAGTTCCTTTGCTTTCCTGCTGAAAACACTAGTCAGAATCCAACTATCT
TGTTATCCGACTCCCTTTTTACGAATATTCCATTTGCCGAATCGTCTGTTCAGATTTCTATCAGCCTGTAATCTGTTCAGTGTCT
CCGTTTTTTTCCTGCTAATGCGGCTGGTTCTGCATCTTCCAGGGGCGACGCTGGTGGAGATCAAGAAGTCCTTCCGCAACGT
CGGCAACGTACTGTACGATTGGGCCGGCGACGACTACTGCTCCTGGCGCGGCGTCCTGTGCGACAACGTCACATTCGCCGT
CGCTGCGCTGTAAGAAGAGCGTCCATTAAACTTTGCTCCTTTCCCAACGATTGCGATGTGTAGCCACTGATGCGGGTTGGCG
CCA
>SsLRR-RLK406
TGCGACAACCGATTGCGAACCTTAGTATGCAGCAACGCAAAAGTACTCCAGTTCCGCTCGCATCCACTGGAGGAGATGCATT
GTGAAACAAGCCGCAAAGCAAGGCGCTGTAAATTTGGAGTTTCTCTACCAAATATCATCCACCAGGCCGCTGCAACTGTATT
TATGATGTATTCCAATCCCTCCCACTTCTGTGTCTCTCGCACCGCACTGACAAAGTATGGATTATCGGCATTTCTTCCGGGGAT
TCCTACATAATGGAAAAACTTTCCCCAAGCTTTACCAATAGCTTCCTTTGCATTTTTCCCCTTCTGCGTCCAAGAGCCTGTATC
AATCCTTTGTTGCTGCATTCCTCTCCTCCCACCCGCTGCCACATGATAGTCCTCCACCTCCACTGGAGTCTCCCTCCGTGATCC
CGCTCTTCTAAACAACCTGTCAATAGGATTCCCACCTTGCGAACCACCACCACCAGCACTACCCCCTCCACGGTGGTAATTA
CCTCCTCTACGTTGCACCCTCTGCTCGTACTCATATTCTGCTCTTGACTGGTTGAGTGCTTCCCGCATCTGAGCCTCATATTCA
TCATCACCGAGGGGATGGTTTCCCTCAGCTGCAGCTTTTTCTTCTGCCAACCTTTGTCGAGCCCGGTCAGCAGTCGTCTTCTT
AGCTCTGTCAAGATCTCGCCGAAAGTACGCACGCACATCTGGTGGCACACGGGAGCAATGCAAGGTGTCCGCCCCCGTACCC
AGCCAAATGCTGTTTAAATCTAGTGCAACCACCGCCTGCTCTCGTCACATGACAATATTTGCATCTCCATCCAGGAGCAAGAT
TGTCACCATGCTCCCATACAACGTCTCTGTTTCTGCCTGACATTGTCTACTCTACCTGCATAAACGGACAACACAAAGTCACA
CCTAAATAAAATGTTTCCTAAATCCTACCACAAATCCTCCATAATATATACCTAAAATATACCTCAAATCTATACCTCACATACA
CAAAGTTACAATATATACACAAAGTCACACAATGTATACCTAAAATCCTAAATCCTACCACAAATCCTACCTCTAATCCTAAAT
CCTAAAAAAACATCCTAAAATAAATCCTACCTAAAACCTAACCAATCCTAAATCCTACCACAAATCCTACCTCTAATCCTAAAT
CCTAAAAAAACATCCTAAAATAAATCCTACCTAAAACCTAACCAACAAACTATCCTAAATCCTAAAACAAATTCTACCTAAAT
CCTAAAACTACTCTAAATAAAAAAAAAACAAAAAAAAACTTACCTGCGCGGTTAGCCGCCGAACCGGTCGGTAAACCGAGC
TAACCGAGCGGCTAACCGAGCTAACCGAGCGGTTTACCGACCGCGCTCAACGGCCTCGGTAAGCCGACCAAACCGGTCGGT
AAACCGACAGATCGGCGCGGTAAGCCGGCGGCGGCGGCCGGTCGCCTAGGGTTCGCCCGGAGCGTCGCGTTGGGGGTGAG
GAAATGAGGTGTCGGCGCGTTTGGACCCCCCCCCCGCTCACTGCCGGCTTGTTTTGTGCCCGTGACGGCCTAATGGGCCTCA
TGGGCCTTAACGGGCCGGCACTGTTTTTTTTTCTTTTTTTTCAAACTTCTATTCGTATAAATAAATACTAAACGAACTCAAATT
TGAAAAATTTTTACACCATTGGCTTCTATAAATCATGGACTATTTTTAGGAATTTTTTCAGCATTTTTTTTTCAAATTTTAAATTT
GAATTTGGGGAAACCCTAACCGGGCCGGACCGGAACCGGACCGGCCCGGTTTGGGCGGTTACCGCGGTAAACCGCGGTAA

ACCGGCCGGTTTTTTTAACCCTGGAGGCACTTGCGCCGGTGCTAGCTGCGACGTGAGCTCGTAGCAGCCAGTAGGAAGGAC
TTCTTGGCCCGCCCGGGTTCGCTGTTGCCGGAAAGGCGGTGGCTTTGGGGAAGAAAGCGACGGTTGCGAAAGGTTCTTGC
GGATTCGCACGCGGGAA
>SsLRR-RLK437
GATACATTCTAGGGTTCATTTGGCAGCTCAGAACGATTTCAATGGTCGGCATGCCACTAGCACATTATTATTGGAACTATATAT
TAGGTACCACACTAATAGATACTATTGCTTATTCATGCCATATCAGCTGACACGTCGTGTCAACATCTTGACCTATACATAGTCA
TACGACTTCTAGTCCAGCGTGCTTGTGCTAACCTGGACACTATAGGCCAACTAGACTAGCTCACCCACTCTATTGTCATCGTC
ACCACGCTTAGGGACGACACATCACTCGATTTGCCGTCACCGTCGTTGTGCTGCCAGCACCGCCATCGCCACACTGGGGCTA
TGTCGCTCGCTCCGCCTCAACCATTGTCGTGCGCCATATGCAACCACGCCACATGCGAGTCCACAAAAGGGAGGCGAACTG
GCGAGAAAGGAAGGAGACGAGATACAAAAGCAGGGAGATAGGGTTCTGGAAGGGGTGTTTATATACTTCACTCTCCACTCA
TCTAACGGTTGTAATCTACTCTAAGCCATCGTTCAATGGGTCGGCTCTGTTAGTGTTGTGCTTTTTAAGGAAGTGCGTAGGGC
CATTGAATGGGCCTAGCCCATTTGATCATCTAAAGAAAGCCACAAACGCTATAGTCGCTCAAATAGGCAAGGACCGAAATAT
TTAACTATGAATCACCGATCATTCTCCTTAATTCCTTATTTAGATTGGGCATGTGAAATGGTCTTGGAGTAGTTTGTATACCTAT
TTTATTTCCTATTGCCTGCTCTCTGTGCTAGCAATTCTATTTGTTCTGTTAATCTTTATTTAGTGAAACAAGTTAAAATCATTTGT
ATTTATGTTGTTGCTGACCTAACAAAAAGTTTAAGATTTTGTTATTCATAATTTCATTTATATGGTTTTTTAAGTAAAATACATTT
TCTTGTTAACCAGAATGTAGTCTACTAGCCAGATGCATGTAGTCATAAAGTGATTTTTTGATTCATCAAAAAAAAAAGATTGGG
ATCCTACCAGAATTTGTATCTTTAACCAAAAACATCGTTTATGTATTTTGTTATGTTAGACGTAGCAAAATACATGTTTCAGTCA
AGTACCCCGTGATGACTTGTACCCGCCTATCTTGCGTCTTCTCTCCTGCGAACCTCAAGCCACATGGATCTCCACGAGCACTC
CGATCCATTAATTCTCTCCTCATCAAGAAAAACAAAAAAGGACCAGTCTCTGTGCAAAACCTGATTAATCCCATGGAGCCAC
GAGCACATGCATGCATATGGTGTGGTCTACGCCTAAGACCGGCCAGGCTCTCCTCCAACCATGATTAATTGCGTCCACGACC
ATCTGCTTGCACCTCCCTTTCTTCAATCATAAATCCTCAAGCATCTCCCTTGACGAGCCATCCGTACGTCGGCCATGGCCCGT
GGGCGACAGCTCGCACGGCTGCACTGCACGTCTGCACGGCTGGTCTTCGTGGTCAGGCCCGGCCGGTGGGTGATGACCGGT
GACCACAACGACGACGAGGACACGCCGGCGCCGGCGCCGGCACGACGCGACGCCACTCTCAGAGTCACAGCCGCGGCGT
GACCCTGTCTGTGGCTACACGGTACAGTACCGATCTGTACGCGCAGCGCGAGATGCCGCGCCGGCCCTGGACCACACTGGA
GGCCACAGGGTTCCGCGCCCGGGGACGATCCCAACCGACTGGCGGGCGGTTCTTTTTTACGCGCGCTCTTTAGCCCCGCTG
ACAGGTGCCGGCCAGGCTTCGCCCACTGACAGGTGGCATCCGCGGTAGGTGGGGCCTACATGTCATTGAAGTCCCAGGCGG
ACGACCGCAACGAGCCAAACGGCGTGGAGGGGTTTTGATTTTTGAAAAATGAAAACAGGAAGGCCACGGCCCACGGGCCC
CTACAGGCTACACGTATGCCTGGGGCCCACTCCCTGACGCGGTGGGCCCGGGGCCGTCAGCCTTAACACCCCCCTCCCTCCG
TCTTCTTTCTCTCTCCTAC
>SsLRR-RLK202
CTAGATAGCCGCCCTGCGTGCTGGCCGCCTGGAGCTGCCCGCAGCGACACGGCCGCCCGGCTACGGCAACGGAAGGCCAA
GGGGAGCTGCCTAGCCAAGCTAATGGGTGCCGCCGCCCGCGTCCGCCTCCTCCTGCTGCTCCCCCTCCTCCTGGGCCTTGGC
TGCAGCCGCAATGGCGGCCGATGCTCCTCGGCGCAGACGCTGCTGCCGGAGCAAGAAGGTGAGCCACAGCACGTCCCCGA
TGCTTTCCTTCTGATCTTGGAGCTCTGCACTTGTTCTCGGATCTACTGTTGCAATTTTTTCCTCATCCGGTGCGCGTAATTCTT
GGAAAAAAACATGGGGAATGCTTCAGTTGAGCTAACCAGGCATCAAAGCATACTATTTCCGATAACGCGTGATTTTTAGCAT
TTTCCAAGAACCATCGATGCATTTTTCTTTTGAAATTATCGATACATTTTTTTATACAAGAAATTGGTGAATGGCTAGCTGTTTC
CTAATGACGGTGTCGGCAGGGATATATATGTGTTATATGTATTCCGGGCGCGATTGAATTTACTACTGCAGGTCTCGCAAAAAA
AAAAAGAGAATTTACTACTAGGGCAGCGGCACATCGTAGAGTTGCTAATTAGGAATTACGATGGTCAAAATATGAATTTGAC
AGCGAGGTTATTTTATCTTTGTATTATCTTTTCACTTCACAAAGTGCGTCGAGTTTATGCTGCATAAACTTTGGCTTGTGCACT
CCACGTCGGACAGAAGACCGCCAATTTGTTCTGGAACCTGCTTTAACGCTTTGGTATTGTTCCGTCGCGTCACGCATCTGTTT
TCGATTCTCCCGTGGGCGGATGAACTCAGCAACTTCGACTTGTTTAGACCGTGGGTGAAAAGTTTTTTTTCACATCGAATATT
TTATAGGATGTTAGAAAATGTGTTCGTATACTAATAAAAAGTACTAATTACATAACTCGTCTGCCGCAAAACGAATTTATTTAC
CCTAATTAATCCATCATTAGCACATGTATGTACTGTAGCAATTATGGCTAATCATGAGCTAATAGGCTTAAAAGATTCGTCTCGT
AAAATCCATATAAACTATGCAATTAATTATTTTTTTTTATCTATATTTAATACTCTATACACGTGTCAAACATTCGAGGAACGTGG
TGAAAAATTTTTAGGCGAGACTAAACCAGGCCGTCCTTGCGCTTGGACGGTTAGTGGTCCCGTACCGCCTCTGTAAATTGTT
GCGCAGGCATGGCCTGAGCAAAATCGTTTCCTAGTACTTCTCTGTCTGATTTCTCTCTGCCAAACTAGTAGTATTTCAATGGT
CTTGGTAAGTCTTGGTAATGAAAAATTGCACTGTTCAGTGATTAGACGAGTTTCTAATACTTGCTGCCACAAACAGGGCACA
GATGCAAATTGTTGTCGTTACCAGGGTTACTAGTGCAAGATAGCAGCTTTAATTCACTGTAAAGTGTGAACTCGTCACGTCG
AAGCCTATCCATCTTCGCGCTTGTTTAGTTAGTGAAAATATTTAGGTTCGGCTACTATAGCACTTTTCGTTTTTTTTGGTAATTT
GTGTCCAATCATGGACTAATTAGGCTCAAAAGATTTGTCTCATGATTTACAACTAAACTGTGTAATTAGTTTTTTCCTTCATCT
ACATTTAGTACTCTATGCATGTGCCGCAAGATTTGATATGACAGGTAGTGTAGCACTTTTTGGGTTCCTTTTTAGAACTAAATA
GGCTCTTCGTCAATTAGGCCAGTTCCCCACTTGCCGAGCGAGCCAAGTAAAAAAACCCTGTCAGCCGACAAATTCTCACCTT
TGAGGCTCTCACGTTTGACAGCGGTGTTCCGTGTTCGCCTTCAACATCCATCCCAACTTGCGCGCCGTGGGACATGCACATG
CCAAAGCTGTTGGGCGTTGGCAGGTGGCTGACCACCTTGCTCTCTCTGTCTCTGGTCCAACGGGTGGGGGCCTGCCAAGTG
TTTGACGAA
>SsLRR-RLK278
GAACAATTTATGGAACAATTACTAATATTTAGGACACCAAATAGATGAACTATATAAAAATAAGTTTCAATCTGAATTTAGTGA
TACATTCGGTAATATAAATATTAGTATTTTGTATATAAACTTTGTTAAAATAAAAAAAGTTTGACTGAGAACAAAATTAAAACA
TTATATTTTTTTAGACATAGGTTCTAATTAATATAAAAAAATCCATCCCGTAGTAAGTACTAGACACCATATGTCTAGAAAACGA
TGTAAAATCATAAGTTTTTCTGTTCTATCCAAAGTAGATGTCTTTAGCTTATTTGCAAAATGTTAGTCAATCATTTTTAACGTTG
ATCTTCATCCAAAAGAATACACTTCTAATATTGTGCTAAGTCAAACATCTTAAGTTTGATCATTTTCTTTACAAAAATATACAA
ATAATTATGATGAATAATCATAATTAGATTCATTATGAAATATATTTTTTGAATATGCATATTTCAAATTATAATTGTTAATATCATT
TTTACAAACTTAATCAAACTTAAAATAGCTTGATTTTAACCAAATTCTAGAAGTATGCTCTTTTTGGTTGGAGGGAGCACGCT
ACAAGGTTCTTATTGTTTAATTAATTAATTATATTTTCTAGTGCCTTTGTGTTGGAATGGAACATACAAAACTTGTTGCACGGTC
ACTTAAAACAATTTGCGGCTCTAAAATTTTATTCATCAATCATGTCATCTGTTCTAATATATCTTGAAATCAAACTTGTACTATG
GTTGTACACACTAACAGGTATGAGCAATGGTTATGCGTCCATGTCACAACCAAAGAAGGTGCGCTAGGAGACCTAGGAAGT
AAAATACGATGGAAACCTGTCCCAGGTTTGGAACGCAAGAATTTCACAGGAATTACGTAAAGAATCAGTTCACTTTCACGG
AAAAAACGAAGGAACCTAGAAAAATTCCCATGTTCCAGACAGAGCCCGAATACTTTGGTCTCGTTTAGTTCCAGGGTGTAA
AGTTTTAGGTATCACGTCGGGTGTCACGTGAGGATGTCGCATGGGGTGTTCGGATAATAAAAAAATAAATTACAGAATCCGC
CAGTAAACCGCGAGACGAATTTATTAAGGCTAATTAATCTGTCATTAGCACATGTGTTACTGTATTGTCAAATCATGGACTAAT
TATGCTTAAAAGATGCGTCTCATAAAGTAGTCGTAACCTATATAATTAGTTATTTTTTAATCTATATTTAATACTTTATGCATGTGT
CTAAACATTCGATAGGATATGATATATTTTTAGGAAGAAATTAAACAAGACCTTTAAGACCTTTGTGCACTTGTCCTTGTGGTA
AATTTGCAGTCAAACTTGAACGCACCCACATGCACGCTGGACACCACGCGTTCCGTTCCGGACCGCGCCTTGTTGGCTTTTG
TGGACACTGAAACCCGTGCAGTCACGTACTGCCACCCGCGGTCTACTGGTAGGCGGTAGCACTAGCAGCACGCAATGGCTG
ATCTCACTCGTAAAGCCATGATGATCGACACACCGCAATCAATCTGCTGAACCAACGCAACGCTCACCCGTCCCAAACGGTA
GAATCAGTTAACGACACATAAATATAGGAAATAGAAAAATGCCCCATGTTCTAAAGAGGACCTAAAATCTGAATCCAGTTCC
TTCGTCGTCCTTCAACAAATTGTACTACTAGTACAGTAACGTATATTATACGGAGTACGAAAAAAATGGGAGAAGAGTAGAG
GACGCAAGCGAAGCAACAAGCAAAGGCATGGAACTCAAAAAATGCCAGGTGGGGCCGGGCGGATTAGGACGAAAAGCAA
AGGCAGGGAAAGCATTAAGCAAAAGAGGAGAGAGAGAGAGGGAGAGGGGGAAAGGAGGCTAGATCGAGGGAGAGAGGG
ACGGCCCGCGATTGCTACGGCCTCCATCGCAGCGAGAGAGAGCTTGAAAGCGTGCTTGCCTGCCATTCCCAGCTTCCAATTC

CTTTCCTTC
>SsLRR-RLK301
CTAATATTGTGCTAAGTCAAATATCTTAAGTTTGATCATTTTCTTTACAAAAAATATACAAATAATTATGATGAATAATCATAATT
AGATTCATTATGAAATATATTTTTTGAATATGCATATTTGAAATTATAATTGTTAATATCATTTTTACAAACTTAATCAAACTTAAA
ATAGTTTGGTTTTAACCAAACTCTAGAAGTATGCTCTTTTTGGTTGGAGGGAGTACGCTACAAGGTTCTTATTGTTTAATTAAT
TATATTTTTCTAGTGCCTTTGTGTTGGAATAGAACATACAAAAGTTGTTGCACGGTCACTTAAAACAATTTGCGGCTCTAAAA
TTTTATTCATCAATCATGTTATCTGTTCTAATGTATCTTGAAATCAAATACTATGGTTGTACACACTAACAGGTATGAGCAATGG
TTATGCGTCCATGTCACAACCAAACAAGAAGGTGCGTAGGAGACCTAGGAAGTAAAATACGATGGAAACCTGTCCCAGGTT
TGAGTTCTTGACCCGTGATAGTATTTTGAAATTTGAGGGCCTATTTGGAACATAAGAATTTCACAGAAATTATGCAGAAATTTT
ACAGGAATCTGTTCATTTTCACAGAAAAAACAAAGGAACGTGAAAAAAATTCCCGTGTTCCAGAGAGCCTGAATACTTTGT
GCACTTGTCCTTGTGGTAAATTTGAGTACTTTGCAGTCAAACTTGAAGGCACCCACTTGCACGCTGGAGTGCTGGACACCAC
GCGTTCCGTTCCGGACCGGCGCCTTGTTGGCTTTTGTGGACACTGAAACCCGTGCAGTCACGTACTGCCACCCGCGGTCTAC
TGGTAGGCGGTAGCACTAGCAGCACGCAATGGCTGATCTCACTCGTAAAGCCATGATGATCGACACACCGCAATCAATCTGC
TGAACCAACGCTCACCCGTCCCAAACGGTAGAATCAGTTAACGACACATAAATATAAAAAGTATCTCATGTTCTAAATAAAG
AAAAAAAATGTAGGAAGTAGAAAAATGCCCCATGTTCTAAGGCCTTGTTTACATTAAGTTTTTTCACCCTCTCTCCATCACAT
CGAATCTTTGAACGTATGCATAGAGCATTAAATGAAGGTAAAATAAATAATTGTACAGTTTATCTGTAATTTCGGAGACGAATC
TTTTTAGTCTAGTTAGGTCATGATTGGACAATAATTGCCAAATACAAACGAAGGTGCTACAATGCGATGAAAATTTTTCACTC
CAATGTAAACAAGGCCTAAAGAGGACCTAAAATCTGAATCCAGTTCCTTCGTCGTCCTTCAAAAAATCGTACTACTAGTATAG
TAACGTACTCCCTCCGTCCATAAAAACATGCAATTCTCGTTTTTCGAGAAATCAAACAGGTTCAAGTTTAACTAAATTTATATA
AAAAAATACTAATATTTATGATACAAAATAAGTATCATTAGATTAGTTATAGGATATATTTTTATAATAAATTTATTTGGAGACAT
AAATGTTAATACTATTTGCTATAAACTTGGTCAAACTTGAGTCAGTTTGACTGGCACGCATCCCATAATTGCATCTTTTCTCGG
ACGGAGGAAGTATATTATACGGAGTAAGAAAAAAATGGGAGAAAATAAAGACCAAGCGACTGGGTGGAGAGAGAAGAGTA
GAGGACGCAAGAGAAGCAACAAGCAAGGGCATGGAACTCAAAAATGCCAGGTGGGGCCGGGCGGATTAGGACGAAAAGC
AAAGGCAGGGAAAGCAAAAGAGGAGAGAGAGGGAGAGGGGGAAAGGAGGCTAGATCGAGGGAGAGAGGGACGGCCCG
CGATTGCTACGGCCTCCATCGCGGCGAGAGCTTGAAAGCGTGCTTGCCTGCCATTCCCAGCTTCCAATTCCTTTCCTTCTTCT
TCTTGCTCGTCCACCCAGTACTCCACCACTGCCCATCCTCCTCCTCCTTCTCCCTCTCCCTCTCCCGCCACCCCCATCCCTGC
GACGACGCGCAC
>SsLRR-RLK291
CGGTTCCCAAGTTTGTTTCAATTTCATCACGAAAGAAACTATCTTTATCTCGAACAATTTATGGAACAATTACTAATATTTAGG
ACACCAAATAGATGAACTATATAAAAATAAGTTTCATGCTGAATTTAGTGATACATTCGGTAATATAAATATTAGTATTTTGTATA
TAAACTTTGTTAAAATAAAAAAAAGTTTGACTGAGAACAAAATTAAAACATTATATTTTTTAGACATAGGTTCTAATTAATATA
AAAAAATCCATCCCGTAGTAAGTACTAGACACCAGATGTCTAGAAAACGATGTAAAATCATAAGTTTTCTGTTCTATCCAAAG
TAGATGTCTTTAGCTTATTTGCAAAATGCTAGTCAATCATTTTTAACGTTGATCTTCATCCAAAAGAATACACTTCTAATATTGT
GCTAAGTCAAATATCTTAAGTTTGATCATTTTCTTTACAAAAAATATACAAATAATTATGATGAATAATCATAATTAGATTCATTA
TGAAATATATTTTTTGAATATGCATATTTGAAATTATAATTGTTAATATCATTTTTACAAACTTAATCAAACTTAAAATAGTTTGG
TTTTAACCAAACTCTAGAAGTATGCTCTTTTTGGTTGGAGGGAGTACGCTACAAGGTTCTTATTGTTTAATTAATTATATTTTTC
TAGTGCCTTTGTGTTGGAATAGAACATACAAAAGTTGTTGCACGGTCACTTAAAACAATTTGCGGCTCTAAAATTTTATTCAT
CAATCATGTTATCTGTTCTAATGTATCTTGAAATCAAATACTATGGTTGTACACACTAACAGGTATGAGCAATGGTTATGCGTC
CATGTCACAACCAAACAAGAAGGTGCGTAGGAGACCTAGGAAGTAAAATACGATGGAAACCTGTCCCAGGTTTGAGTTCTT
GACCCGTGATAGTATTTTGAAATTTGAGGGCCTATTTGGAACATAAGAATTTCACAGGAATTATGCAGAAATTTTACAGGAAT
CTGTTCATTTTCACAGAAAAAACAAAGGAACGTGAAAAAAATTCCCGTGTTCCAGAGAGCCCGAATACTTTGTGCACTTGT
CCTTGTGGTAAATTTGAGTACTTTGCAGTCAAACTTGAAGGCACCCACTTGCACGCTGGAGTGCTGGACACCACGCGTTCC
GTTCCGGACCGGCGCCTTGTTGGCTTTTGTGGACACTGAAACCCGTGCAGTCACGTACTGCCACCCGCGGTCTACTGGTAGG
CGGTAGCACTAGCAGCACGCAATGGCTGATCTCACTCGTAAAGCCATGATGATCGACACACCGCAATCAATCTGCTGAACCA
ACGCTCACCCGTCCCAAACGGTAGAATCAGTTAACGACACATAAATATAAAAAGTATCTCATGTTCTAAATAAAGAAAAAAA
ATGTAGGAAGTAGAAAAATGCCCCATGTTCTAAGGCCTTATTTACATTCAAGTTTTTTCACTCTCTCTCCATCACATCGAATCT
TTGAACGTATGCATAGAGCATTAAATGAAGGTAAAATAAATAATTGCACAGTTTATCTGTAATTTCGGAGACGAATCTTTTTAG
TCTAGTTAGGTCATGATTGGACAATAATTGCCAAATACAAACGAATGTGCTACAGTGCGATGAAAATTTTTCACTCCAATGTA
AACAAGGCCTAAAGAGGACCTAAAATCTGAATCCAGTTCCTTCGTCGTCCTTCAAAAAATCGTACTACTAGTACAGTAACGT
ATATTATACGGAGTACGAAAAAAAATGGGAGAAAATAAAGATCAAGCGACTGGGTGGAGAGAGAAGAGTAGAGGACGCAAG
GGAAGCAACAAGCAAGGGCATGGAACTCAAAAATGCCAGGTGGGGCCGGGCGGATTAGGACGAAAAGCAAAGGCAGGGA
AAGCAAAAGAGGAGAGAGAGAGAGGGGGAGAGGGGGAAAGGAGGCTAGATCGAGGGAGAGAGGGACGGCCCGCGATTG
CTACGGCCTCCA
>SsLRR-RLK94
TCACTGAGGCCGTGTTTGGATGTAGAATCTGACAACATCAAACAACACCTAAAGGTTTATGACCTTCCGTCCAAAACTTGAA
CACACAGAAGTTTCCTCAAACAACCTCCACAGTTCTTCAGTGAAATCGGCACGTTCCCGTACGTTACGTTCCTTTAAGAAAA
AAGGGGTTGTTCTATTTGTGAAAAAACGGTGTTCCAATTTTTTCACTCGCCATAGAGATGAGCTTGTTGAATTGAAGAACAA
AAAACAACCGAAAAGCGCTGTCGCGAGGCCACTTTTTTCCTACCTTGACTGGATCACGGATCGCAAAAGCTGGCTTTCGGC
GACGAGTATAACCCACACGTACGCACGTACGTACTCTCTCTCCCAAAATAAAAGTCATTCTCGCTTTCAAAAAATCAGTTTTT
TTAAACTTTGACTAATTATATATAAAAAAATATTAATATTTATAATACATAATTAATATTATTAGATGGATCGTTGAATATACTTTTA
TAATAAACTTATTTAGAGATATAAATGTTGCACGTATTTTCTATAATCCCAGTTAAAGGTAAGAAAGTTTGATCTGCACGTAGC
CCATAACGTCATCTGTTCTGGGATGGAGGAAGTACTACACGCCCGCCGTATACCTCCACGGCTCTCCCCCTCTACGCGTCGTC
GACCCCGTATGGCCGAGCCCCGCCAACAGTGTCTCTGACAGTGGGCCCGTCCAGGCATCCTCCACCCCCGTCCGCCAGCCC
CTGCTCGACGAAATGCCAGCGCTTCCGGTCAATGGCGCCCGTCGCCCGCGCTGTTGACCCCCGGCGCGGGCGCGCCCCTGT
CCTGTCCTCCACAGTGCTCGCCCCCATAAAGCTCCTTGATCCCTTCTCCCTGCAGCCCCCCACAGTGCCACTCACTCGCGGC
CACCACCCCCACTACCACCACCCCGCCGCCCTGTTGCGCGACCAGTGTCACCCACCTCGCCCCGCCCCCCGCGCATGCCGC
GCTGTCCGGCCTCCTAGTGGCTACTGGTACTGGGTGGTGTGGGGGAGGGGACGGACGAGACGACGGCTTCCTCGGTTGCCG
CTACCTCTGTCCTGTCTTTCCATGGCACTCTGCTCCTCGCCTCTGTCATAGTTCTGCGCCTCTCGCTCCAGCTGCTGGGCCGG
GTTCCGCTTCTTGCCGCGTTTGCCTTAGGATGCGCGCGGCGGCGGCCGCAATGGCGTCCTTGCTCCCGGCGGCGGTCCTCCT
CTTGGCTCTTCTCAATCTCAACTCGGCGGCGGCGGATGCGGAGGGTGCCGCGGCGGCGCTGCTGGAGTTCAAGGGCGCGCT
GGAGGACGTGAACGGCCGCCTCTCGACCTGGGGCGCCGCGGGCGCGGGCGCGGGCGTGGACCCGTGCGGGTGGGCCGGC
ATTGCCTGCTCCGCGGCCGGCGACGTGACCGGCGTCACGCTCCACGGCCTCAACCTGCACGGTGGCCTCTCCACCGCCGTC
TGCGCGCTCCCGCGCCTGGCCGTGCTCAACGTCTCCAAGAACGCGCTCAGCGGGCCCATCCCGCCGGGCCTCGCCGCCTGC
GCCGCGCTCGAGGTCCTCGACCTCAGCACCAACGCGCTCCACGGCGGCGTCCCGCCGGACCTCTGCGCGCTCCCCGCCCTC
CGCCGCCTCTTCCTCAGCGAGAACCTGCTGTTCGGCGACATCCCGCCCGCCATCGGCAACCTCACCGCGCTCGAGGAGCTC
GAAATCTACAGCAACAACCTCACGGGGACGATCCCGACCTCCGTCTCCGCGCTCCAGCGGCTGCGGGTCATCCGCGCCGGG
CTTAACCAGCTCTCCGGCCCCATCCCCGTCGACCTCACCGAGTGCGCCAGCCTCGAGGTGCTCGGCCTCGCGCAGAACAAC
CTCGCCGGGGAGCTTCCTAGGGAGCTCTCCGCCTCAAGAACCTCACCACATTGATCCTCTGGCAGAACCACCTCTCGGGCG
AGGTGCCGCCGGAGCTCGGCGAGTGCACCAACCTCCA
>SsLRR-RLK75

TTAATTAAACAGTAAACGGTTCTATGTAGCATTTAAACGATGGTAAACAGGGTAATAAATGACCGTTTAGATGATCAGTGATTT
TATATTTCACACATGCATGCATGATAAATTATGGGGATCACTCCCTCTTTACCTAATTAAATTGCTAATAATCTAAGACAAGGTT
TAGTTTGGTTTTTTAGAACCAGATCGAAGTTATAATAAGTAATTATATCTCGCCTTTGAAATCTCACCACCTCTCATCCGTACGT
GCAGCCCCACAGTTCATCTCCAACCAACCGCCAGCTGCATAGATCATCTAACCTTCTCATGTGCCATGGCCCTGCCACCAGTT
CCATAACCACTCCAACCACCTCTTCCTCCCCTCACACCCATATCAGGTGAAAGTGCACCAAAATTTAAGAAATGGCGCAAAT
TCACCCAAAAGCATACTTTGAAGTTTTGAAACAATTTTTGTAACTTGCCATCTCTAGTTCATTCATAGATTGATGTGTATTTTG
TCGCAAAAGCTGAGACAAAAACTCATTTTAAAAAGAAACGACATGAAAATTTGGGGCTATATATGAAAAAATGTCAAATTTG
GGATGTACATTTAGAAAATTTAACAAACTTTTGTGGAAAGCACGCCTTTAAGTGAATTTGAATCTATTTTTCCAAAAACAAAA
ACTAACAAATTTTCACTTGATATGCACCCCAACGTCATCGCCTTATTTACACTCTGTTTTAATTCTACCTGACCAAACCATCAG
TCACCGATAAAGAATTTGTTATGCGTACATATATCATCTATGGTTTTGAGAGTAAAATCCAATCATCACTAAAGGATTGTGACA
TTCTGTCCAAAACTTCAACACTGTAGAAGTATTTTTTTTCTGTGTACGAGAGCTACGCATATTTGTATTAGTATAAGGTGTTAA
GTGAATTTGAATCTATTTTTCTAAAAACAAAAACTAGCACATTTTCACCTCATATGCATCCCCACGCCAAAGCCTATTAAACTC
TGTTTTAATTGTACTCGAGCTATCACCAACCATCACAGTCACTGATAAAGAAGTTAAGATGCATACATATCATCTATGGTTTGA
GAGTAGAATCCAATTATCACTGAGGCCGTGTTTGGATGTAGAATCTGACAACATCAAACAACACCTGAAGGTTTATGACCTT
CCGTCCAAAACTTGAACACACAGAAGTTTCCTCAAACAACCTCCACAGTTCTTCAGTGAAATCGGCACGTTCCCGTACGTT
ACGTTCCTTTAAGAAAAAAGGGGTTGTTCTATTTGTGAAAAAACGGTGTTCCAATTTTTTCACTCGCCATAGAGATGAGCTT
GTTGAATTGAAGAACAAAAAACAACCGAAAAGCGCTGTCGCGAGGCCACTTTTTTCCTACCTTGACTGGATCACGGATCGC
AAAAGCTGGCTTTCGGCGACGAGTATAACCCACACGTACGCACGTACGTACTCTCTCTCCCAAAATAAAAGTCATTCTCGCT
TTCAAAAAATCAGTTTTTTTAAACTTTGACTAATTATATATAAAAAATATTAATATTTATAATACATAATTAATATTATTAGATGGA
TCGTTGAATATACTTTTATAATAAACTTATTTAGAGATATAAATGTTGCACGTATTTTCTATAATCCCAGTTAAAGGTAAGAAAG
TTTGATCTGCACGTAGCCCATAACGTCATCTGTTCTGGGATGGAGGAAGTACTACACGCCCGCCGTATACCTCCACGGCTCTC
CCTCCCTCTACGCGTCGTCGACCCCGTATGGCCGAGCCCCGCCAACAGTGTCTCTGACAGTGGGCCCGTCCAGGCATCCTCC
ACCCCCGTCCGCCAGCCCCTGCTCGACGAAATGCCAGCGCTTCCGGTCAATGGCGCCCGTCGCCCGCGCTGTTGACCCCCG
GCGCGGGCGCGCCCCTGTCCTGTCCTCCACAGTGCTCGCCCCCATAAAGCTCCTTGATCCCTTCTCCCTGCAGCCCCCCCAC
AGTG
>SsLRR-RLK137
TTAGTAATTATATCTCGCCTTTGAAATCTCACCACCTCTCATCCGTACGTGCAGCCCCACAGTTCATCTCCAACCAACCGCCA
GCTGCATAGATCATCTAACCTTCTCATGTGCCATGGCCTTGCCACCAGTTCCATACCCACTCCAACCACCTCTTCCTCCCCTCA
CACCCATATCAGGTGAAAGTGCACCAAAATTTAAGAAATGGCGCAAATTCACCCAAAAGCATACTTTGAAGTTTTGAAACAA
TTTTTGTAACTTGCCATCTCTAGTTCATTCATAGATTGATGTGTATTTTGTCACAAAAGCTGAGACAAAAACTCATTTTAAAAA
GAAACGACATGAACTTTTGGGGCTATATATGAAAAAATGTCAAATTTGGGATGTACATTTAGAAAATTTAACAAACTTTTGTG
GAAAGCACACTTTTAAGTGAATTTGAATCTATTTTCCAAAAAAAAACCACAGAAGTATTTTTTCTGTGTACGAGAGCTACACAT
ATTTGTATTAGTATAAGGTGTTAAGTGAATTTGAATCTATTTTTCTAAAAACAAAAACTAACACATTTTCACCTCATATGCATCC
CCACGCCAAAGCCTATTACACTTTGTTTTAATTCTACTCGAGCTATCATCGACCATCACAGTCACTGATAAAGAAGTTAAGAT
GCATACATATCATCTATGGTTTGAGAGTAGAATCCAATCATCTTATTTGGATATAGTTGGATTCGCATCAATTGCAGATGTGTTT
GGGTGGATTGAAGTGAAATTTGAACTAAATTACACTCCTAATCCATTCCAACGCACGTGGATTGAGGTGAATCCGGCAACAT
CCAATCAAGACATGAAGGTTTATGACCTTCCGTCCAAAACTTGAACACACAGAAGTTTTCTCAAACAACCTCCACAGTTCTT
CAGTGAAATCGGCATGTCCCCGTACGTTACGTTCCTTTAAGAAAAAAGGGTTGTTCTATTTGTGAAAAAACGGTGTTCCAAT
TTTTTTTCACTTGCCATAGAGATGAGCTTGTTGAATTGAAGAACAAAAAACAACCGAAAAGCGCTGTCGCGAGGCCGCTTTT
TTCCTACCTCGACTGGATCACGGATCGCAAAAGCTGGCTTTCGGCGACGAGTAGTCGTAACCCACACCTACGCACGTACGTG
CTCCCTCTCTCCCAAAATAAAAGCCATTATCGCTTCCTGAAAAGTCAATTTTTTTAACTTTAACTAATTATATATAAAAGAATAT
TAATATTTATAATACATAACTAATATCATTAGATGGATCGTTGAATATATTTTCATAATAAATTTATTTAGAGATATAAATGTTACA
CGTATTTTCTATAACCCTAGTTAAAGTTGAGAAAATTTAATGTGCACGCATCCCATAACATCGACGGAGGGAGTACTCCTGCA
CGCCCGCCGTATACCTCCACGGCTCTCCCTCTCTACGCGTCGTCGACCCCGTATGGCCGAGCCCCGCCATCAGTGTCTCTGAC
AGTGGGCCCGTCCAGGCATCCTCCACCCCTGTCCGCCAGCCCCTGCTCGACGAAATGCCAGCGCTTCCGGTCAATGGCGCC
CGTCGCCCGCGCTGTTGACCCCCGGCGCGGGCGCGCCCCTGTCCTGTCCTCCACAGTGCTCGCCCCCATAAAGCTCCTTGAT
CCCTTCTCCCTGCCGCCCCCCACAGTGCCACTCACTCGCGGCCACCACCACCACCACCACCCCCCGCCGCCCTGTTGCGCG
ACCAGTGTCACCCACCTCGCCCCGCCCCCGCGCATGCTGCGCTGTCCGGCCTCCTATTGGCTACTGGTACTGGGTGGTGTGG
GAGGGGACGGACGAGACGACGGCTTCCTCGGCTGCCGCTACCTCTGTCCTGTCTTTCCATGGCACTCTGCTCCTCGCCTCTG
TCATAGTTCTGCGCCTCTCGCTCCAGCTGCTGGGCCGGGTTCCGCTTCTTGCCGCGTTTGCCTTAGGATGCGCGCGGCGGCG
GCCGCAATGG
>SsLRR-RLK74
AGATATATTTTTCTTAGCAGAATGTAAATAGATATATAGATGTGTTATAAGTACCTTCGGAAAAAATTAATAGAACTAGGGGTG
GCCCATGAGTGCTTATTTATACAAAGATATATAGCGCTTCCTTCAGTCACATATGAGTGATATTTTGGATAACGACATAATCTCC
AAAAGAAACTTTAACTAATAGGTTTTGCAAAATGTTTATGAAATATAGCAAGAATATACTTTTATTAAATTATATTTCAAGGAA
AAAAGCCATGCCATTAATTAAATTCAACATATACAAAATAACATGCTACCACAAAATGTATATGTCCCTTATCTGCTACTAGTG
TAGAGGGATTATATATAAAAGGCCTAGCTTTTGATATATAATATACCATTGTATCGTCATGGATTTATCATAAGTATCGTTAGATT
TATAATGAGATATATTTTTATAGTATACCTATTTGATCAATGTCATAAACGTTAATTTTTTACTTATATATTTGGTTAAATGTTAGTA
ACTCTAAAAAAGAATGCACCTAGAGTTGCATTCTGTTTTGGGACCGAGGTAGCACATCAAGAGATAATTATATTTGAAATACA
GAACACGATGAGGATCCATGGAACCATCAATTAACACCTTGGTAAATATAGAAAGGATCTTATTTGGTGTGGATCTGACCTGA
ATGCAAAACAGCCACCATGTGCAGCATGGAAAATGGTCTGTACTTCGAAAGAAGGTGGATTAGGAGTGCTTAATCTCAAAAT
TCAAAATGAGGTCCTTATAATGAAAGATCTGCATAAATTCTTCAATATCATGTTTTAATTAATGGAGTCAATCATCACTCCCAAT
TCTAGAACCTTTTCGGCAGCAACTTCAAGTGCCTTTCTTCCTGGAAATAATAGTACTAATATGCTAGATCATTTTGACTACAAG
GAATGGAAAGATCTTTAACAATCAGCAACTAACTTTGGCTTCATATAAAGTGTCAGATTGAATTTCAATAAAGATTTCAGCAT
GGTTATTCATAGAGCAAAGAGTAGATTTTTTTTTGACAAGTTGTAGATTGGCTTGAATCCTTGTAATATATGTAATTAACCTGC
TCTTTACTTTAATACAATTTTCAGTAAGGGAGAAGGCCCTCCTGTCCCATAAAAAAAAATTGAAACTTCAAATGCTTGAATGAA
TGACACTATATATGTGCATTAATATAGTCAAATGATGTTACATTTTTACTGTTGGTGTAAATTTTCTTCCCGTCACGACAAATGG
TGGATAATTTTCTATGTGCTGCTAGCTTGTTGCATCCTTTCTCTTTTTTTCGATCTGTTTTTGTTTAACAGCACGTCAGCACTCC
GTATTTGATCAGGTATATCCTATGTACTTCTCAACATATGCGTGTCGGCATACATCTCTGCATTGGCCACCACTTTTCTCTGTGC
CTGTGTTGTTGGATGTCTCCCGTCACTGTAACTGTATCGATCAGAATGAATAGTCTTGTCGGTATGCTTATCACTGTAACTGTA
TCACTGGTTGTTTTCTGCTGATCGGCAGTGTCCCTGCATTTTCCCTTTCAGTGCACAGTTAGCGCTACTCCAAGACAACTGTA
TAACGGGTGCATCAGTGCNNNNNNNNNNNNNNNNNNNNNNNNNNNNNNNNNNNNNNNNNNNNNNNNNNNNNNNNNNNNNNNNNNNN
NNNNNNNNNNNNNNNNNNNNNNNNNNNNNNNNNNNNNNNNNNNNNNNNTTATAATAAACTTATTTAGAGATATAAATGTTGCACGTAT
TTTCTATAATCCTAGTTAAAGGTGAGAAAATTTGACATGTACGTATCCCATAACGCCATCTGTTCTGGCATGGAGGAAGTACTA
CACGCCCGCCGTATACCTCCACGGCTCTCCCTCTCTACGCGTCGTCGACCCCGTATGGCCGAGCCCCGCCAACAGTGTCTCT
GACAGTGGGCCCGTCCAGGCATCCTCCACCCCCGTCCGCCAGCCCCTGCTCGACGAAATGCCAGCGCTTCCGGTCA
>SsLRR-RLK262
ATTCTTCAATGGTGAAAACTCAGATTAGTCTTTCCAAGTTACTGTTCAAAATCGGTGTATTTAAAGGTATGGATGAATGTATAA
ATTATTGCTGAATCAGGAGGTTATTACTTATGAGGCCTAGGTAGCTGTCTTATTTAGTTTAATTTTGTCATTAAAAAGGCAAGT
AAATAAGGGGACCTAGCTATGCAAGAATAGTGTAGTTAAATAAGGACCAGTGCATAGTGTTTTTCACTCTAACTTGATCTTAC

TTGACAACATTTCTAGTTTAACTTATTAGTTCTTAAATGTTTTATGCACCCTGTTCCTTTCTGCCTAGTTTTTTCTTAAACTTCA
AAAATGCCAACAACACAGCAAGGATTTCAAAATATGCTCGTAGAGTTGACATCCTAGGGTGTATATACATAGCCCCAAGAAA
AAGCCGCTTGCAGCTAAGATTGACATTTTTTTCACATGGTTTTCTGTTGTTCACTGGGTGAACTGTTCATTTCCCTATTGCAAT
AATTTGATGCAGTTTTTATTCTAGATGCAGTATATATTTCTGTCCCTTGCTATTGCTGTTATTGCTTGATCAGATATTCATTCAGA
TCATCAGATACGGAAGAAGTGCATATAGCAGCCTTGCAGGCCAATTTAACAATTTCACTATTCACTTGTGATTCTTTCTCTGAC
TGTGTTTCAGCAGCCTTTCAAACTGTGTCAACTCTCTTGCTAATTAACCCATTTAATTACATTGCTAGTAGTACTTCTGTTCCC
TTTTCATCTGATGCATGTAGGTCAGGTGACATTCTACTGGGGACATGTGCCAAGCAATGCTCAAAGGATCCCCTTGTCAGAC
AAGAGCAGTAACAGCAGCAGCAGCAGCTACTGCATGCATGCCCACTGTCACAGGAATGATTACGCAGGTTTTATTTTAACAA
GGTCAACTTGGGCAGACTCAACACATGCAGTGCACCACTGTACATATGCTTAGGCAGGGTAAACTTTCTTTAGTTGTTTATAT
ATGCATGACATGTGTCCAGATCTCTTAGCTAGTTAGGGTGCTTGCATCAGGACAACACCAAGAACATACCCCTCCACCCTCTT
GTCTCCCTTTGGGTTAAATCTTTTCTTTTAGAGCCTGCAATGGAAGCATCCTGTTCTACTGACTGGTAACTGACATAGGATTAG
TACTGTTTGTTTTGCTCAAATTACTGTAGCTCTTGTTGCCCCGATAGCTTACCTGTACCACAGAGAGTAGGAGCTAGCTTCTG
CATGGTACTAGTAGATCAGGATCAGTTGAAATCTTAGGCTTTCATCTACACAAAATACAGGAGTAGATCTGTTTCAGAACTTG
AGAACAGAAGCTTCCAAAAGAACAGGCCGTACGAACACCTAATTTACAGCAGGTCTGTTGGTCTCTGATTAGTACTCCTTAG
TAATCAATCAGGTTCAGAAAGCTAGAGCAGAGCTAATCTTCTACTGTCTGCAATGACAGGGAATGTAAACTGAAGATGCAAA
TACAATTAATCTACTCTGATCTGATGGGGGCTGCCACAAAGTAAAAGATTAAAGAATGCCAACATTGCCCAAAAGCAGGCTG
CAAAAGCCATGGCTTTAAAACACATTCCCTCCATGTTCCATGTTCTCATGCCCCCTCTCCATAACCCAGCTCACATTCGTTCG
CAGGGTGAGGTGTCCAGGCACTAAACAAACAGCATGGTGGCATGTTAAGCAATGCCAAGAAGATCCATTATCTTATTACATG
CTTTAACCGATCTTGGGACTAGTGAGAATGATGATATAAAGCCTTTTCTGCCACCTTCTTCTTCTTCCACCTAGCTGCAGCCTC
TGCCAGTCCTGCAGATGCAGTAGTGTCAGCAAACAAACTAGTAGGCAAGCCAGACAGTGCCTCTGACATGGCGCAGCACTA
GAAGCAGCAGAGGCACCTGCTGCAGCTAGAAGCTGATCGATTTCAGCTCCCAAAAGTGCTTTCTAGGAAAGTGGCAGAGG
ATTCTAAT
>SsLRR-RLK215
GTGCACGTATTGCTCCTTATGTACCTTCTCATTTGTTTTATGGATCGGAGAGCATGGCAATTGATCTCTGCATTCTTCAATGGTG
AAAACTCAGATTAGTCTTTCCAAGTTACTGTTCAAAATCGGTCTATTTAAAGGTATGGATGAATGTATAAATTATTGCTGAATC
AGGAGGTTATTACTTATGAGGCCTAGGTAGCTGTCTTATTTAGTTTAATTTTGTCATTAAAAAGGCAAGTAAATAAGGGGACC
TAGCTATGCAAGAATAGTGTAGTTAAATAAGGACCAGTGCATAGTGTTTTTCACTCTAACTTGATCTTACTTGACAACATTTCT
AGTTTAACTTATTAGCTCTTAAATGTTTTATGCACCCTGTTCCTTTCTGCCTAGTTTTTTCTTAAACTTCAAAAATGCCAACAA
CACAGCAAGGATTTCAAAATATGCTCATAGAATTGACATCCTAGGGTGTATATACATAGCCCCAAGAAAAAGCCGCTTGCAGC
TAAGATTGACATTTTTTTCACATGGTTTTCTGTTGTTCACTGGGTGTGCAATATTTTGATGCAGTTTTTATTCTAGATGCAGTAT
ATATTTCTGTCCCTTGCTATTGCTGTTATTGCTTGATCAGATATTCATTCAGATCATCAGATACGGAAGAAGTGCATATAGCAGC
CTTGCAGGCCAATTTAACAATTTCACTAATCACTTGTGATTCTTTCTCTGACTGTGTTTCAGCAGCCTTTCAAACTATGTCAAC
TCACTTGCTAATTAACCCATTTAATTACATTGCTAGTACTACTTCTGTTCCCTTTTCATCTGATGCATGTAGGACAGGTGACATT
CTACTGGGGACATGTGCCAAGCAATGCTCAAAGGATCCCCTTGTCAGACAAGAGCAGTAACAGCAGCAGCAGCAGCTACTG
CATGCATGCCCATTGTCACAGGAATGATTAGCGCAGGTTTTATTTTAACAAGGTCAACTTGGGCAGACTCAACACATGCAGT
GCACCACTGTACATATGCTTAGGCAGGGTAAACTTTCTTTAGTTGTTTATATATGCATGACATGTGTCCAGATCTCTTAGCTAGT
TAGGATGCTTGCATCAGGACAACACCAAGAACATACCCCTCCACCCTCTTGTCTCCCTTTGGGTTAAATCTTTTCTTTTAGAG
CCTGCAATGGAAGCATCCTGTTCTACTGACTGGTAACTGACATAGGATTAGTACTGTTTGTTTTGCTCAAATTACTGTGGCAT
GGTACTAGTAGATCAGGATCAATTGAAATCTTAGGCTTTCATCTACACAAAATGCAGGAGTAGATCTGTTTCAGAACTTGATA
ACAGAAGCTTCCAAAAGAACAGGCCGTACGAACACCTAATTTACAGCAGGTCTGTTGGTCTCTGATTAGTACTCCTTAGTAA
TCAATCAGGTTCAGAAAGCTAGAGCAGAGCTAATCTTCTACTGTCTGCAATGACAGGGAATGTAAACTGAAGATGCAAATGC
AATTAATCTACTCTGATCTGATGGGGGCTGCCACAAAGTAAAAGATTAAAGAATGCCAACATTGCCCAAAAGCAGGCTGCAA
AAGCCATGGCTTTAAAACACATTCCCTCCATGTTCCATGTTCTCATGCCCCCTCTCCAAAACCCAGCTCACATTCGTTCGCAG
GGTGAGGTGTCCAGGCACTAAACAAACAGCATGGTGGCATGTTAAGCAATGCCAAGAAGATCCATTATCTTATTACATGCTTT
AACCGATCTTGGGACTAGTGAGAATGATGATATAAAGCCTTTTCTGCCACCTTCTTCTTCTTCCACCTAGCTGCAGCCTCTGC
CAGTCCTGCAGATGCAGTAGTGTCAGCAAACAAACTAGTAGGCAAGCCAGACAGTGCCTCTGACATGGCGCAGCACTAGAA
GCAGCAGAGGCACCTGCTGCAGCTAGAAGCTGATCGATTTCAGCTCCCAAAAGTGCTTTCTAGGAAAGTGGCAGAGGATTC
TAAT
>SsLRR-RLK240
CCTAGTCGAAATTTTTCATGTGCAATATGATAAAATCCAAAGAAAAAGTTTGGAAAACATAGCTATAAATTAACCAAATTACG
ATAATACATTTTCTAGATGGAATTTTCAACTATGCCAACTATCCATGATAAATTTTTCACTTTCTTTACCAAACAATAACAATA
GAAGCAATATACTGCATCATTGTCTACGCTATATCCAATCCAAGGGTACTTATGAAATAACAGCAAATTCATTCTCCAATGTC
CAAACCCTAAGTGGGATGAATGGGATCTGGGAATTGGGAACAGAAACACTGACCAGTTCAATCAATAGGACATTGCCCAAC
GGGGGCAAGGCCACAGGGGTTAGCCGGCCAGGGCCAGCCGTGGGGCACCGCCACAGCGCGCACTGGACGGCTGCTGCCG
GCCTAGCGGGCGGTGGCTTGACGCGTCGGACGCCAGCGGCAGGCATGGGGAGCGACAGGGTGGGCGGGGCGGGCGGCCG
CGCCGACGGCGAGCGGTCAAGTGGGGGCGGCGGGCGTGGGGTGGGCAGGGGCGGGTGGCGGGGTAGGGCGGGGCGGGC
GGCCGCGCCGACGGCGAGTGGCCGACTGGGGGCGGCGGGGGCCAGTCGGAGTCGAGGGGACGAGGGTTGCGTATTGCGG
CGCGGACTCGCGCGCTCGGGGTTGCCTTCCGCCTGGGCTGAGACTGAGACGAGAGTGACTGAAAGGAGGGTGTTTGGGCC
AAATTTTCATGGGCTCTGCAGACTAGTAAAAATACTTTGGGCCAAATTTCAGGGTAGTCCCGGGCCTACCTGGACTACCCGC
TGGGCCCGCCACTGCATGTAGGACAGGTGACATTCTACTGGGGACATGTGCCAAGCAATGCTCAAAGGATCCCCTTGTCAG
ACAAGAGCAGTAACAGCAGCAGCAGCAGCTACTGCATGCATGCCCATTGTCACAGGAATGATTAGCGCAGGTTTTATTTTAA
CAAGGTCAACTTGGGCAGACTCAACACATGCAGTGCACCACTGTACATATGCTTAGGCAGGGTAAACTTTCTTTAGTTGTTT
ATATATGCATGACATGTGTCCAGATCTCTTAGCTAGTTAGGATGCTTGCATCAGGACAACACCAAGAACATACCCCTCCACCC
TCTTGTCTCCCTTTGGGTTAAATCTTTTCTTTTAGAGCCTGCAATGGAAGCATCCTGTTCTACTGACTGGTAACTGACATAGG
ATTAGTACTGTTTGTTTTGCTCAAATTACTGTGGCATGGTACTAGTAGATCAGGATCAATTGAAATCTTAGGCTTTCATCTACA
CAAAATGCAGGAGTAGATCTGTTTCAGAACTTGATAACAGAAGCTTCCAAAAGAACAGGCCGTACGAACACCTAATTTACA
GCAGGTCTGTTGGTCTCTGATTAGTACTCCTTAGTAATCAATCAGGTTCAGAAAGCTAGAGCAGAGCTAATCTTCTACTGTCT
GCAATGACAGGGAATGTAAACTGAAGATGCAAATGCAATTAATCTACTCTGATCTGATGGGGGCTGCCACAAAGTAAAAGAT
TAAAGAATGCCAACATTGCCCAAAAGCAGGCTGCAAAAGCCATGGCTTTAAAACACATTCCCTCCATGTTCCATGTTCTCAT
GCCCCCTCTCCAAAACCCAGCTCACATTCGTTCGCAGGGTGAGGTGTCCAGGCACTAAACAAACAGCATGGTGGCATGTTA
AGCAATGCCAAGAAGATCCATTATCTTATTACATGCTTTAACCGATCTTGGGACTAGTGAGAATGATGATATAAAGCCTTTTCT
GCCACCTTCTTCTTCTTCCACCTAGCTGCAGCCTCTGCCAGTCCTGCAGATGCAGTAGTGTCAGCAAACAAACTAGTAGGCA
AGCCAGACAGTGCCTCTGACATGGCGCAGCACTAGAAGCAGCAGAGGCACCTGCTGCAGCTAGAAGCTGATCGATTTCAGC
TCCCAAAAGTGCTTTCTAGGAAAGTGGCAGAGGATTCTA
>SsLRR-RLK50
TTCAATGTACTCTATCGTTTCTGAACCCGGCCTCGGGCTGGTCCCTTGTAAATGACTTCTATTTCTCCTTAATACAATGATACG
CAACGCTCTTGCGTATTGATAGTTTTTTTTCACATACTATAGAACCAGTATATGCAAAACGTACAAGAAACTATAGCTTATTTG
TAAAAATGTCAAAGTTCAGGACAATGCCTTAAAGGTAAAGATTCCCTAAAATTGACAATCTAGCACACCAATGTGCTTTATAG
GAAACTAAAGCCTGACGTCCTCATCAAGAATCTAATCAATAAGAGATAATCGGACTTCCATGTGTTGGAAATTATTTGGCCTG
GGCAAGTTGTGCATATGCTTCTGTACCATTATCAAGAAAATGTTCAATGACTAACTAACAACAAATTTCCAATATGCCTGTTCC

TCCCAGCTTCCACCAATGTAGTAATCGTTTGCAGATAAGTGCAATTTTACTTTCGAAAATTTAAGAGCTCGGGTACAACGATT
ACGACAAATGCAGACTAGTTTAATGTTTTTTTGTTGGAAGCTGCATAAACATTACTTTCACTGTTGTGGTAATAGTAGATTTAC
TAGGAATTTTTGTCAGAGAACTGCATGTAGTGCATTATGATTCACTTGTATGATTCTACAATGTACCATTACAACCTCACTTGA
CTGTTAGATATGTACAGTGCTATCACTGATTAAGGGCCAATGATTTATAGATTAAGCGTGATTTGTTCTTGCCGGCAGGCAAGC
CTAACCAAAAAAGAAGGATAGAAGATTCCTGCTCACTTTTCACTCTTTGTAAGCTTGTAGCAGAAATTTTAAAAACACGAGC
TACTGATGACACCTGGAACAGAATCTTACAGTGCCAATAGATTTCCAACCTGCAAGGATCACTGACACAGGTTCCATTGTAC
ACTGCAAGATGAGATTAAAGTACTACTTTTAGAATCAGTCATTTGTTAAACACCCACAGCACGCTTGCTGCTGCTCCTATGTA
GCTTTGCTTCCTTTCAGTAGTACATTATACTAACCCAGAGGCCAATAAAGTAATCTTGGCATTAACCATGCCTGTCCAGTTTAG
TGGACTTCCATTGTTCAGACAAGCAGTGTGGGTGAGAATTCTTCCTTAGTTAAGCATGATTTATTTGTTTCTCTGAAGTGATG
ACTGGGTTTTCAGAGGTCTTGATTTCTTCCGAAATGGTGATAAAAATGCAGTTTGTTACCAATATTCAGTGCTTTCCAAACTC
AGGGACACAGTACAACTACTTCATTGCATGCCAGTAATTTACTTGAAATAATTATCTAATTAACAGAAACTAATCATGGTGTGT
GCTTCAGAGATCTTCAGTACAAGAAAGGTAACCTCAAGCTTAATCAACCCTTCATAGCACCAATATTCATCATAACTGAAAAA
TGACTATTCCTGTGCCCTGCCTACATGAACACAATACGGAATAAACTCCAGTGGAAACCCCCATGTTCTAAGTTTATACAATG
ATTGCAGTTCCGTTTGTTTAACCCAACAAGACAAATCTCTCTCTCCAATGACGAGTGACCAATCCGCCTTTCACACATTCCCT
TCACCACATGGGCCATGTCTCCCCAAGCTCTCAATAATTCCAAAGGCATTTCCAGCCTATGTCCATGCCTCCTCCCAACCTTT
ACACTCTCATACCGAGCTGTTCTTGTGCTACCCAAGGCCCCACAGAGGCTACTGAACCATGTACCTAACACTACTACAACGC
AGCAGTATACCAATGCAGTATCATGCCACCAGCAAGTATTTTTTATCATCCAATCCATCTCCAGACACATAAATTATTCGATTAA
TCTGATCCTAATCATAGACATGACCTCCCTATATACTTTCCTGCGAGTTTCCCTCCATTTCTCTATTGCTTCAAACAAAGGCAAT
GAAAACACAACTCTGCCAGTGAAGGAGAGGCAGAAGGCGGCAGAGGAGTGAAGGAGAAGGGAAGAACAGAAGAAACCG
AGCA
>SsLRR-RLK20
AATGTACTCTATCGTTTCTGAACCCGGCCTCGGGCTGGTCCCTTGTAAATGACTTCTATTTCTCCTTAATACAATGATACGCAA
CGCTCTTGCGTATTCGATAGTTTTTTTTCACATACTATAGAACCAGTATATGCAAAACGTACAAGAAACTATAGCTTATTTGTAA
AAAATGTCAAAGTTCAGGACAATGCCTTAAAGGTAAAGATTCCCTAAAATTGACAATCTAGCACACCAATGTGCTTTATAGG
AAACTAAAGCCTGACGTCCTCATCAAGAATCTAATCAATAAGAGATAATCGGACTTCCATGTGTTGGAAATTATTTGGCCTGG
GCAAGTTGTGCATATGCTTCTGTACCATTATCAAGAAAATGTTCAATGACTAACTAACAACAAATTTCCAATATGCCTGTTCCT
CCCAGCTTCCACCAATGTAGTAATCGTTTGCAGATAAGTGCAATTTTACTTTCGAAAATTTAAGAGCTCGGGTACAACGATTA
CGACAAATGCAGACTAGTTTAATGTTTTTTTGTTGGAAGCTGCATAAACATTACTTTCACTGTTGTGGTAATAGTAGATTTACT
AGGAATTTTTGTCAGAGAACTGCATGTAGTGCATTATGATTCACTTGTATGATTCTACAATGTACCATTACAACCTCACTTGAC
TGTTAGATATGTACAGTGCTATCACTGATTAAGGGCCAATGATTTATAGATTAAGCGTGATTTGTTCTTGCCGGCAGGCAAGCC
TAACCAAAAAAGAAGGATAGAAGATTCCTGCTCACTTTTCACTCTTTGTAAGCTTGTAGCAGAAATTTTAAAAACACGAGCT
ACTGATGACACCTGGAACAGAATCTTACAGTGCCAATAGATTTCCAACCTGCAAGGATCACTGACACAGGTTCCATTGTACA
CTGCAAGATGAGATTAAAGTACTACTTTTAGAATCAGTCATTTGTTAAACACCCACAGCACGCTTGCTGCTGCTCCTATGTAG
CTTTGCTTCCTTTCAGTAGTACATTATACTAACCCAGAGGCCAATAAAGTAATCTTGGCATTAACCATGCCTGTCCAGTTTAGT
GGACTTCCATTGTTCAGACAAGCAGTGTGGGTGAGAATTCTTCCTTAGTTACAGCATGATTTATTTGTTTCTCTGAAGTGATG
ACTGGGTTTTCAGAGGTCTTGATTTCTTCCGAAATGGTGATAAAAATGCAGTTTGTTACCAATATTCAGTGCTTTCCAAACTC
AGGGACACAGTACAACTACTTCATTGCATGCCAGTAATTTACTTGAAATAATTATCTAATTAACAGAAACTAATCATGGTGTGT
GCTTCAGAGATCTTCAGCACAAGAAAGGTAACCTCAAGCTTAATCAACCCTTCATAGCACCAATATTCATCATAACTGAAAA
ATGACTATTCCTGTGCCCTGCCTACATGAACACAATACGGAATAAACTCCAGTGGAAACCCCCATGTTCTAAGTTTATACAAT
GATTGCAGTTCCGTTTGTTTAACCCAACAAGACAAATCTCTCTCTCCAATGACGAGTGACCAATCCGCCTTTCACACATTCCC
TTCACCACATGGGCCATGTCTCCCCAAGCTCTCAATAATTCCAAAGGCATTTCCAGCCTATGTCCATGCCTCCTCCCAACCTT
TACACTCTCATACCGAGCTGTTCTTGTGCTACCCAAGGCCCCACAGAGGCTACTGAACCATGTACCTAACACTACTACAACG
CAGCAGTATACCAATGCAGTATCATGCCACCAGCAAGTATTTTTTATCATCCAATCCATCTCCAGACACATAAATTATTCGATTA
ATCTGATCCTAATCATAGACATGACCTCCCTATATACTTTCCTGCGAGTTTCCCTCCATTTCTCTATTGCTTCAAACAAAGGCA
ATGAAAACACAACTCTGCCAGTGAAGGAGAGGCAGAAGGCGGCAGAGGAGTGAAGGAGAAGGGAAGAACAGAAGAAAC
CGAGCA
>SsLRR-RLK2
CAATGTACTCTATCGTTTCTGAACCCAGCCTTGGGCTGGTCCCTTGTAAATGACTTCTATTTCTCCTTAATGCAATGATACACA
ACACTTTTGCATAATCGGTATTTTTTTCACATACTATAGAACCAGTATATGCAAAACGTACAAGAAACTATAGCTTATTTGTAA
AAAGTGTCAAAGTTCAGGACAATGCCTTAAAGGTAAAGATTCCCTAAAATTGACAATCTAGCACACCAATGTGCTTTATAGG
AAACTAAAGCCTGACGTCCTCAGCAAGAATCTAATCAATAAGAGAGAATCGGACTTCCATGTGTTGGAAATTATTTGGCCTA
GGCAAGTTGTGCATATGCTTCTGTACCATTATCAAGAAAATGTTCAATGACTAACTAACAACAAATTTCCAATATGCCTGTTCC
TCCCAGCTTCCACCAACGTAGTAATCGTTTGCAGATAAGTGCAATTTTACTTTCGAAAATTTAAGAGCTCGGGTACAACGATT
ACGACAAATGCAGACTAGTTTAATGTTTTTTTGTTGGAAGCTGCATAAACATTACTTTCACTGTTGTGGTAATAGTAGATTTAC
TAGGAATTTTTGTCAGAGAACTGCATGTAGTGCATTATGATTCACTTGTATGATTCTACAATGTACCATTACAACCTCACTTGA
CTGTTAGATATGTACAGTGCTATCACTTATTAAGGGCCAATGATTTAGAGATTAAGCGTGATTTGTTCTTGCCGGCAGGCAAGC
CTAACCAAAAAAGAAGGATAGAAGATTCCTGCTCACTTTTCACTCTTTGTAAGCTTGTAGCAGAAATTTTAAAAACACGAGC
TACTGATGACACCTGGAACAGAATCTTACAGTGCCAATAGATTTCCAACCTGCAAGGATCACTGACACACAGGTTCCATTGT
ACACTGCAAGATGAGATTAAAGTACTACTTTTAGAATCAGTCATTTGTTAAACACCCACAGCACGCTTGCTGCTGCTCCTATG
TAGCTTTGCTTCCTTTCAGTAGTACATTATACTAACCCAGAGGCCAATAAAGTAATCTTGGCATTAACCATGCCTGTCCAGTTT
AGTGGACTTCCATTGTTCAGACAAGCAGTGTGGGTGAGAATTCTTCCTTAGTTAAAGCATGATTTATTTGTTTCTCTGAAGTG
ATGACTGGGTTTTCAGAGGTCTTGATTTCTTCCGAAATGGTGATAAAAATGCAGTTTGTTACCAATATTCAGTGCTTTCCAAA
CTCAGGGACACAGTACAACTACTTCATTGCATGCCAGTAATTTACTTGAAATAATTATCTAATTAACAGAAACTAATCATGGTG
TGTGCTTCAGAGATCTTCAGTACAAGAAAGGTAACCTCAAGCTTAATCAACCCTTCATAGCACCAATATTCATCATAACTGAA
AAATGACTATTCCTGTGACCTGCCTACATGAACACAATACGGAATAAACTCCAGTGGAAACCCCCATGTTCTAAGTTTATACA
ATGATTGCAGTTCCGTTTGTTTAACCCAACAAGACAAATCTCTCTCTCCAATGACGAGTGACCAATCCGCCTTTCACACATTC
CCTTCACCACATGGGCCATGTCTCCCCAAGCTCTCAATAATTCCAAAGGCATTTCCAGCCTATGTCCATGCCTCCTCCCAACC
TTTACACTCTCATACCGAGCTGTTCTTGTGCTACCCAAGGCCCCACAGAGGCTACTGAACCATGTACCTAACACTACTACAAC
GCAGCAGTATACCAATGCAGTATCATGCCACCAGCAAGTATTTTTTATCATCCAATCCATCTCCAGACACATAAATTATTCGAT
TAATCTGATCCTAATCATAGACATGACCTCCCTATATACTTTCCTGCGAGTTTCCCTCCATTTCTCTATTGCTTCAAACAAAGGC
AATGAAAACACAACTCTGCCAGTGAAGGAGAGGCAGAAGGCGGCAGAGGAGTGAAGGAGAAGGGAAGAACAGAAGAAA
CCGAGC
>SsLRR-RLK35
CAATGTACTCTATCGTTTCTGAACCCAGCCTTGGGCTGGTCCCTTGTAAATGACTTCTATTTCTCCTTAATGCAATGATACACA
ACGCTCTTGCATAATCGGTATTTTTTTCACATACTATAGAACCAGTATATGCAAAACGTACAAGAAACTATAGCTTATTTGTAA
AAAGTGTCAAAGTTCAGGACAATGCCTTAAAGGTAAAGATTCCCTAAAATTGACAATCTAGCACACCAATGTGCTTTATAGG
AAACTAAAGCCTGACGTCCTCAGCAAGAATCTAATCAATAAGAGAGAATCGGACTTCCATGTGTTGGAAATTATTTGGCCTA
GGCAAGTTGTGCATATGCTTCTGTACCATTATCAAGAAAATGTTCAATGACTAACTAACAACAAATTTCCAATATGCCTGTTCC
TCCCAGCTTCCACCAACGTAGTAATCGTTTGCAGATAAGTGCAATTTTACTTTCGAAAATTTAAGAGCTCGGGTACAACGATT
ACGACAAATGCAGACTAGTTTAATGTTTTTTTGTTGGAAGCTGCATAAACATTACTTTCACTGTTGTGGTAATAGTAGATTTAC

TAGGAATTTTTGTCAGAGAACTGCATGTAGTGCATTATGATTCACTTGTATGATTCTACAATGTACCATTACAACCTCACTTGA
CTGTTAGATATGTACAGTGCTATCACTTATTAAGGGCCAATGATTTAGAGATTAAGCGTGATTTGTTCTTGCCGGCAGGCAAGC
CTAACCAAAAAAGAAGGATAGAAGATTCCTGCTCACTTTTCACTCTTTGTAAGCTTGTAGCAGAAATTTTAAAAACACGAGC
TACTGATGACACCTGGAACAGAATCTTACAGTGCCAATAGATTTCCAACCTGCAAGGATCACTGACACACAGGTTCCATTGT
ACACTGCAAGATGAGATTAAAGTACTACTTTTAGAATCAGTCATTTGTTAAACACCCACAGCACGCTTGCTGCTGCTCCTATG
TAGCTTTGCTTCCTTTCAGTAGTACATTATACTAACCCAGAGGCCAATAAAGTAATCTTGGCATTAACCATGCCTGTCCAGTTT
AGTGGACTTCCATTGTTCAGACAAGCAGTGTGGGTGAGAATTCTTCCTTAGTTAAAGCATGATTTATTTGTTTCTCTGAAGTG
ATGACTGGGTTTTCAGAGGTCTTGATTTCTTCCGAAATGGTGATAAAAATGCAGTTTGTTACCAATATTCAGTGCTTTCCAAA
CTCAGGGACACAGTACAACTACTTCATTGCATGCCAGTAATTTACTTGAAATAATTATCTAATTAACAGAAACTAATCATGGTG
TGTGCTTCAGAGATCTTCAGTACAAGAAAGGTAACCTCAAGCTTAATCAACCCTTCATAGCACCAATATTCATCATAACTGAA
AAATGACTATTCCTGTGACCTGCCTACATGAACACAATACGGAATAAACTCCAGTGGAAACCCCCATGTTCTAAGTTTATACA
ATGATTGCAGTTCCGTTTGTTTAACCCAACAAGACAAATCTCTCTCTCCAATGACGAGTGACCAATCCGCCTTTCACACATTC
CCTTCACCACATGGGCCATGTCTCCCCAAGCTCTCAATAATTCCAAAGGCATTTCCAGCCTATGTCCATGCCTCCTCCCAACC
TTTACACTCTCATACCGAGCTGTTCTTGTGCTACCCAAGGCCCCACAGAGGCTACTGAACCATGTACCTAACACTACTACAAC
GCAGCAGTATACCAATGCAGTATCATGCCACCAGCAAGTATTTTTATCATCCAATCCATCTCCAGACACATAAATTATTCGAT
TAATCTGATCCTAATCATAGACATGACCTCCCTATATACTTTCCTGCGAGTTTCCCTCCATTTCTCTATTGCTTCAAACAAAGGC
AATGAAAACACAACTCTGCCAGTGAAGGAGAGGCAGAAGGCGGCAGAGGAGTGAAGGAGGAGGGAAGAACAGAAGAAA
CCGAGC
>SsLRR-RLK198
GGAGAAGCAAGAGGAAGAGGAGCACGACGAAGAAGAGGAGGAGTCAGCAGAGGAGGAGGAGGGGGGAGGACCCTTAG
ATCCAGATTCATGGAGGAAGAGAGAGGAGAGGGGAGGAAGAGAAGAGGTACCTGAGCCTGATCCGGCCGCATCCACGGCC
GCCACCGCCGGATCCGCTGGATCTACAGTCGCCATCGCCGGATCCGGCGGATCCACGGCCGCTGCCACCGCATCCGCCATGG
CGGAGGCCCCGGGCGGGCAAGCATGAGAAACGTTGCGAGAGAGCGAGAGCGAGAGTGAGGGAGCACGAAGGTGCACGG
GGAAGGGCGGAGGATGCGGGCGCGCGGCGCAGGCGGGAGAAAGGGGAGGGAGTGGCGGAGGATGCTGCACGGAGGCGA
AGACAGGATGAGAGGGCGAGACGGGGCCGGGCGGGGGTGAATGCGAGTGAGGAGTGGAGTGCCGTGCGAGTGAGTGGGC
GTCGTCCGACGCGTCCAGACGACCGGGGTATATCTTTACCGAAAGAGGATCGAAGAAGAAGAATATCAGAATCCCCTTACCC
TTTTCTTAGTTACTGTTCTTCTTCCCTTCTAAGTCTATTTTTCTATACTAGTTCCTTCTTCCATCCCTGCTTCGATCTCCCATTCC
TGAGATCTTGTATCCTGTTTTTTGATTGGAGTTATCTGTTAGTGAATTCTGTTGGGTCGCTAACATTGCAATACTACTTACGTTA
CTCTGATGAAAGTAGCAAGATGTAGGGAAGAAAATTAGAAAATGGATCCAGTACGCTGCCCGTACGACCCTGCTTGCTACTC
AGGCAATCGGTCGGCTGCACGTCCTTGTTTCCACGCGCCTCACGCACGGGACGGCGCCATAGCCCCTCTCCTCTTGATCAGT
GCCGTCCCTCGTCAGTGAGGCGTGAGCGTTCGCGGCGCGCGTGCTGTACCTGTACATGGAGGCGTGCAAGGACGCGGTGGA
CCTCCTGAGTCCTGACCGGCCTGCTCCCTGCTCGTGCCCGCGGGCCACGGGCCTCACGGCCAGACTGTGTACGGCGAACCC
ACTCCCTCAGTCCTGCTGCGGCGCTGCCCTCCGCCGTGCTCCCGCCGCAAGCCCACAACACTACTTCCGGTGGGCCGCGGG
AACGTGACCGACGTGTTGGGCCTGCCGTATCCTCGGTAACTGCAAGTCGCTTGCCAGAGTTCGCCTGAACAACCACCTCCT
CAGAGCGAGTATAATAAGAGCATGCTCAGTGATAGTCAAGTTGTTTGGTTACAAGTAAGTTATAAGAGTTGAGTTATACGATA
AGTATCTTCTATTTATTTTAAAAATTCTCTCTTTTCTATTTCTTCTCACAACACTTACTACTTAGGCCCACTAATACATATGCTTC
CGTACCTAGCTTGATGCTTGCATGGGAGCCAAGCTCTCATCTCTCTTATCTCTTCTCTCCTCCACATCAGCATTAGCTCGGCTA
TAAATCAATTATTGTACCTGCTCTAATAGGCTGTACTGGCTAAACACTGAGATGAAGGAGATAGGAGAGGAGAGAGAGAGGG
GAGAAATGAGCTATAAGCTTACAGTCGGCTTATGCATAAAAACCGAGAAATTTTGTAAGAGTGACAAGTGAGCCATGTATTA
ATAGTGAAGACGTAACTACTATATAGATGAGTTAAGAGAAATTTGCAAAAAACCTTGCAGCCAGCAACCTAATTATTAACCTT
GCCCTGCTCCGTTCCGACTGGCCTTCTTGATCTCCCCATGAATGACACGTTGGACCTCAGCAACAACTTGCTCTCAGGGGAG
TTCCCAACGGTGATTCCCAGCCATTGGCGGATCCAGGGGGGGGCTGGGGGGCTGCAGCCCCCCCCCCCGGAGGGTGATTT
TTCCACTGGAATACTGTAGCATAGCATCAAATCACCATTAAATCTTAATTATAAATCAACATCTTCCTTAATTAGCCCCCCTGAG
GTGTCAATCCTGGATCCGCCACTGTTCCCAGCGCTAG
>SsLRR-RLK252
TTATCTCATCATATAAATTTAGTCAAACTTAAGATCCTTTGACTATACAAGATTCTTAGAATAACTTATAATTTGAAATAGAGGG
AATACATTGTTACGGCATGCATCACAGTCACCCAAAGTTTAACCTTTTTTTTTTGCGAAAGACCCAAAGTTTAACATTGACTA
AGAGCAGTGACGTTCCGGTAAAAAGACACTGAATGGAACCACTACGAGTCTATGGATCGATAGCGAAAACGCGTCTCTTTT
GTGAAGGAACCTACTCCAGTAATCCAAAAGTTGTGTTCCCCGGTAGACCGGAAGACAAACAATATGGAAGTGAGTGAGGAG
TTTAATAAGACGACCACTCAGACCACAAGACTTTCAAATGTAAAAGTGGTCAGCTACAACGTTTCATAACATTTTGAGATCT
ACAAACTTCATTTAGAAAGTTTTTCCGTCCGAGATCTTTTGAAAAATTCGAATTTTAAAATTTTGAATTTCAAACGTAGTTTTG
CATAACAAGATGAATTCAAATTAAAAAGTGGTCAACTACAATGTTTCATAACCTTTCAAGATCTACAAACTTCATTTAGGAAG
TTTTTTCATCCGAGATTGTAGTGCTAACATTGTTCACAAATCTTATATATCTCTCTTGTAGTTTCATGAAACTACATGAGAGACA
TATAGATTTTGTGAATAACGTAATTATCACTTTGTCGAATGAAGAAATGACCAATATAAAGTTTGTAGATCTTGAGAAGTTATG
AAACTTTGTAGTTGAAAACTTTTTTATTTGAATTCGTTTAGGGCACCAAAATGCAGTTTGAAAAATTACTTTGCCGAGTGAAA
AAAAAAACACTCGGCAAATGCCTTCTTTGTCGAGTGCCGAGTGTTTTACTTTGGCACTCGGCAAAGAAGTCTTTGCCGAGT
GCAGAGACTTTGCTGAGTGCCGAGTGCCCGAAAAAAAAACACTCGACAAAGCCTCCGGCACTCGGCAAAGAAACGGATTC
CGGTAGTGCCTCCATTCTAAATTATAAGTCATTTCCAAGAATCTTGTAGAGTCAAAGTATCTCAAGTTTGACTAATAGAAAGA
ATTAAAACATAATAATGACTTAAATAGGTATACTATGAAAATATAATTAATAAAGAATCTAATGATACTTAGTTAGTATCATAAAT
ATTATTATCTCATCATATAAATTTGGTCAAATTTAAGATCCTTTGACTCTACAAGATTCTTAGGCCAGTCTCAATGGAGAGTTTC
ATTACGTTGTTTCCAAGACTGCCATATCATTTGAAATGAAATGAAACTTGGATGAAACACCTACTCTCGATGGAGAGTTTCAT
TCCGTGGTTTCATAGGCATTTAATTCCAAGACTCATAAAGAGTTGGTAATCGTGCCAAGAGAGTTTCATCTAGATGAAACCCA
TTTCTTCTCTCTCTTCTTAAATACACTGCCATGTCATCAAAAGTGCCTATGTGGCAACCCATTTAATGCAAATGAAACTCACGT
GAAACTCCCACTGAGACTGGCCTTAGAATAACTTATAATTTGAAATAGAGGGAATACATTGTTACGGCATGCATCACAGTCAC
CCAAAGTTTAACCTTTTTTTTTTTGCGAAAGACCCAAAGTTTAACATTGACTAAGAGCAGTGACGTTCCGGTAAAAAGACAC
TGAATGGAACCACTACGACTCTATGGATCGATAGCGAAAACGCGTCTCTTTTGTGGAAGGAACCTACTCCAGTAATCCAAAA
GTTGTGTTCCCCGGTAGACCGGAAGACAAACAATATGGAAGTGAGTGAGGAGTTTAATAAGACGACCACTCAGACCACAAG
ACTTGTTGGTGCAAACGAATTTATACAAAGCTAAGTTAAGCTATAAAAAGATTGATGCACTCATCGTTGACGACTCATCGTTG
ACGAGGAGTAATTATGTAGAGTACATGATTGATGCACTCGAATCTCAAACTCTCAACCAGCCCTCAGTAGTTCCCTAACCTCA
TGCACGCAAT
>SsLRR-RLK394
AGAATAGGAACGCATATATACAAAATAAATCTGTGACTGTGAGGTTGGCTAGATGGATCGTGGATGCATCAGCACATGTGCCG
CACCACAGCAAGAGACGAGACGTTACTTTGGGGCTGTTTAGTTGGCAAAATTTTTGGCGAAATGCTACTGTAACAGTTTCGT
TGTTATTTGGCAAATATTGTCCAATCATGGTCTAATTAGGCTTAAAAGATTTGTCTCGTCTATTTCGTCTAAACTGTGTAATTAG
TTTTATTTTTTATCTATATTTAATGCTTCATGCATGCGTTTAAAGATTCGATGTGACGGAGAATCTGAAAATTTTGGCAAAATTC
TTGGCAACTAAACGGTACCTTTGACTCGTACCAGTGTTTATGTACGGTACCGTGACAGTGGTAGTGTACTAGTAGTGATACTC
CAGTACGAACGGATCAACGGAGCAGTAACCGCACCAGTACATGTACATGCATGCATGCAGATATGCAACCAGACCTGTGCGT
GGTCACGTTTGAGTTTGAGGCCGGCTGATGAGTCGCTGAGAGAGGCCGAGCAGGATAGAGGACTGTCAGGAAGTGGGACC
AGCAGGGACCAGTCACGTGGCACGAGCCATTTTTTCCCCCTTCTGGTCATCACACGCGACACAAGAGAAAGAAAGCCATGC
AGAGAAAAGAGATGCAGTAGCGACAACAACACAAAGGCCATTGGAAATTTCCTTCTCATGATCTTCAGATCATGGGCCTTGT

TTAGATTCCAAAGTTTGCCTCCTAAATTCACTATGCAGAATTCCTATTCCCCGTCACATTAAAATGCCGGCACATATATAGAGT
ACTAAATATAGATAAAATCAAAAACTAATTACACAGTTTTGTTGTACTTTGCGAGACGAACATTTTAAGTCTAATTAATCAATA
TTTGGACAATTATTACCAAATAAAAACAAAATTGCTACAGTGTGACGGGACACTTACTATGCAAACTTTGCCTCCAAAGTTTG
CCATCTAAACAAGGCCATGATCCTTTCTGGGAAAAAAAGAACGGAAATTTGCTGCCAATTCTGGGAATTCACATCCTCCTCT
TGCACAACCACACATGACCCTGTTCGGCAGGCTGTTTTTGCAGCTTATAAGCCGGCTGTGGCTGTTTTACTGTGAGAGAAAA
ATACTGTTCCACGGCTGATAAGCCGTACTGATAAGCTCAGCCGAACAGGGTGATGGCGGCCTCAACCTGTTCCTGCATCTAG
CTTTAAGCCAGTTTTAGGACGATCTTCGAATATTAATTACTGTAGGAGTTTTATAACAAAATCATTAGATCATTTTTTGTTTCAG
AAAAAATCATATCGTTTTCATGGACGAAACATAGAAAAAATATTTCCACGCTCTAGAATGGTTGTATCCTCTCTTCTTTAGCAG
TGAATACTCTCCGATGAAAAAATAGTATTTTTCTCCAATAATAAATCATCGTGCTCCGTAAGCACATCAGCCAAATTTTAGCAA
AACTAAAGAAGCCGGTAAAGTCACCTCAAAAATGAACAACGGCTGTGCCTGTGCTGTGCTGCGCTGCGCGGCCAAGGGTTT
ATGGTGCATGGAACAATGGATGCTACTAGCCCCTAGTCCGGCTAGTCGCCCGCGTCTCTGGCCCTGGAACCACTGCAACAAG
TCTGCAAGCTTGCAAGAGCAAATTGATGGCGCCCGTCATGTCCCCTCCGACGCCGTCCCGTCCCGCCTCTGCTGCGCCTACG
CCTATCTGACACCGCCCGCCGTCAGCCTGCCGTGCGCCGAGGCCGAACCACCATCCAGCACCACCCCTCCTCCTCCCGCCCT
GCTGCTTGCATTTGACCTGCCTATCATCAGCTCGCCCGCCCCGTCCCCGCCGCCGCGGATTCCATTCCATATGCTCATTCCCTC
CACCACCACCGCCACCACTACCGTCCCCTCCTTTTATTCCATCCACCACCACCACGTCCGCCTCGCCACCACCACAGCCACC
ACACCACAACCACA
>SsLRR-RLK416
TTATCTAACCATGAAGTAACTAGCTCAAAAGATTCGTCTCGACATTTACAAATTATATAATTAGTTATTTTTTTACTTATATTTAA
TATTTTATGTATGTGTTTAAAGATTCGATGTGATAGGTGGAAGAGAAAATATTTTTAGTTTGCATGGCAAGTGAACACACCTGC
AAGTGTTTATGTACGGTACCGTGGCAGTGGTAGTGTAGTAGTGATACTCCAGTACGAACGGATCAACGGAGCAGTAACCGCA
ACAGTACATGTACATGCATGCATGCATATGCAGCCAGACCTGTGCGTGGTCACGTTTGAGTTTGAGGCCGGCTGTTGAGTCG
CCGAGAGAGGCCGAGCAGGACAGAGGACTGTCAGGAAGTGGGACCAGCAGGGACCAGTCACGAGGCACGAGCCATTTTT
TTTCCCTTCTGGTCATCACACGCGACACAAGAGAAAGAAAGCCATTCAGAGAAAAGAGATGCAGTAGCGACAACACAAAG
GCCATTGGAAATTTCCTTCTCATGATCTTCAGATCATGATCCTTTCTGGGGAAAAAAAAAGAACGGAAATTTGCTGCCAGTTC
TGGGAATTCACATCCTCCTCTTGCACAACCACACATGGCGGCCTCAACCCGTTCCTGCATCTAGCTTTAAGCCAGTTTTAGGA
CGATCTTCGAATATTAATTACTGTAGGAGTTTTATAACAAAATCATTAGATCATTTTTTGTTTCAGAAAAAATCAGATCGTTTTC
ATGGACGAAACATAGAAAAAAAGATTTCCACGCTCTAAAATGGTTGTATCCCCTCTTCTTCAGCATTGAATACTTTCCTATGA
AACAATAGTATTTTTCTTTCACAGTAAATCATCGTGCTCAGTAAGCGCCTTAGCTAAATTTTAGCAAAACGAAAGAAGCCGGT
AAAGTCACCTCCAAAAATGAAGCCAACGGCTGTGCCTGTGCTGGGCTGCGCTGCGCGGCCCAGGTGCTGCGTTTAGATGTA
AAAATTTTTGTAAAATGGCACTGTACCTTTTTTTGTTGTTATTTAATAAATATTGTTCAATCATGGTCTAATTAGGCTTAAAAGA
TTCGTCTCGTCCATTTCGTCTAAACTGTGTAATTAGTTTTATTTTTTTATCTATATTTAATGATTCATGTATGTGTCAAAGATTCG
ATGTGACGAGAAATCTGAAAAATTTTACAAAATTTTCTAGGAACTCAACAGGGCCTTATGGTGCATGGAACAATGGATGCTA
CTAGTCCGGCTAGTCGCCCGCGTCTCTGGCCCTGGAACCACTGCAAAAGATTCGTCTCGACATTTACAAATAAACTGTGCAA
TTAGTTATTTTTTTACCTACATTTAACGCTCTATGCATACATTTAAAGATTCGATGTAACACTTACTATGCAAAATTTTTTACCTC
TTTTTGGCATCTAAACACTGCCTAAATCATCGTGCTCCGTAAGCAATAGCATCAGCCCAATTTTAGCAAAACGAAAGAAGCC
GGTAAAGTCACCTCCAAAAATGAAGCCAACGGCTGTGCCTGTGCTGTGCTGTGCTGCGCTGCGCGGCCAAGGGTTTATGGT
GCATGGAACAATGGATGCTACTAGCCCCTAGTCCGGCTAGTCGCTCGCGTCTCTGGCCCTGGAACCACTGCAACAAGTCTGC
AAGCTTGCAAGAGCAAATTGATGGCGCCCGTCATGTCCCCTCCGACCCCGTCCCGTCCCGCCTCTGCTGCGCCTACGCCTAT
CTGACACCGCCCGCCGTCAGCCTGCCGTGCGCCGAGGCCGAACCACCATCCAGCACCACCCCTCCTCCTCCCGCCCTGCTG
CTTGCCTTTGACCTGCCTATCATCAGCTCGCCCGCCCCGTCCCCGCCGCCGCGATTCCATTCCATATGCTCATTCCCTCCACCA
CCACCACCACTACCGTCCCCTCCTTTTATTCCATCCACCACCACCACGTCCGCCTCGCCACCACCACAGCCACCACACCACA
ACCACAATGCCTCC
>SsLRR-RLK429
GTTTTGCTTGACGTTCCTGCAGGTGGCTGCCCTTTGGTTGCGGGCCGGTTACTCGAGGCTGTTGTTACTCTTCGCTCTTTTGT
AGTCTCTCTGTTGTTGTGTTGTTTGGTTTCGGTTTTTTTTCCTTGTTTTTTTACCTTTTCCGTTCTTGCGGCTTGTCGGGGCAA
CCTTTGGTTACCCTTTGTCGACGCTTTCGGTGTTTGTTCGGTTGTTATCTATTTCCTCCTAATGAAACACGTGCTGCAGTGCAC
GTTCGCGAAAAAAAAAAGTATGAAATCAAACGTGAAGAAAAGTTCCCTATGTTTTCTTTTTCTTTTTTTTCTACACACAATTCC
TGAGAACCAAAGGAAGTTACAGAAACTCGAAGCATAGGATGGGGGAGCCTGAATGAACGTTGAAATCAAATCTGTGACTGT
TTTTTTTTTTGGAAAAATAAATCTGTGACTGTGAGGTGGGCTAAATGGATCGTAGATGCATCAGCACATGTGCCGCACCACAA
GAGATGAGACGTTACTTTGGGTGTGTTTAGTTGATCAAAAGTAGGTAAAAAATTACTGTAACATCTTTCGTTTTTATTTGACA
AATATTATCCAACCATGAAATAACTAGGTTTAAAAGATTCGTCTCGACATTTATAAATTATGTAATTAATTATTTTTTTACTTATA
TTTAATACTCTGTGCATGCGTTCAAAGATTCGATGTGATAGGTGGAAGAGGAAATATTTTGAGTTTGCATTGGCAAGTGAACA
CAGCCTTTGACTCGTAAGAGTGTTTATGTACGGTACCGTGGCAGTGGTAGTGTAGTAGTGATACTCCAGTACGAACGGATCA
ACGGAGCAGTAACCGCAACAGTACATGTACATGCATGCATGCATACAGATATGCAGCCAGACCTGTGCGTGGTCACGTTTGA
GTTTGAGGCCGGGTGATGAGTCGCTGAGAGAGGCCGAGCAGGATAGAGGACTGTCAGGAAGTGGGACCAGCAGGGACCA
GTCACGAGGCACGAGCCATTTTTTTCCCCTTCTGATCACCACACGCGACACAAGAGAAAGAAAGCCATGCAGAGAAAAGA
GATGCAGTAGCGACAACACAAAGGCCATTGGAAATTTCTTCTCCTGATCTTCAGATCATGATCCTTTCTGGGAAAAAAAAAC
GGAAATTTGCTGCCAATTCTGGGAATTCACATCCTCCTTTTTGCACAACCACACATGGCGGCCTCAACCTGTTCCTGCATCTG
GCTTTAAGCCAGTTTTGGGACGATCTTCGAATGTAATTACTGTAGGAGTTTTATAAAAAAATCATCAGATCATTTTTTTTCAGA
AAAAATCAGATCGTTTTCACGGACGAAACATAGAAAAAATATTTCCACGCTCTAGAATGGTTGTACCCTCTCTTCTTCAGCAC
TGAATACTTTCCAATAAAAGAATAATATTTTTCTCGCACAATAAATTATCGTGCTCCCTAAGCAATAGCATTAGCTAAATTTTAG
CAAAACGAAAGAAGCCGGTAAAGTCACCTCCAAAAATGAAGCCAACGGCTGTGCCTGTGCTGTGCGCTGCGCGGCCAAGG
GTTTATGGTGCATGGAACAATGGATGCTACTAGCCCCGAGTCCGGCTAGTCGCTCGCGTCTCTGGCCCTGGAACCACTGCAA
CAAGTCTGCAAGCTCGCAAGAGCAAATTGATGGCGCCCGTCATGTCCCCTCCGACGCCGTCCCGTCCCGCCTCTGCTGCGCC
TACGCCTATCTGACACCGCCCGCCGTCAGCCTGCCGTGCGCCGAGGCCGAACCACCATCCAGCACCACCCCTCCTCTCCCTC
TCCTCCCGCCCTGCTGCTTGCCTTTGACCTGCCCCGTCCCCGCCGCCGCGATTCCATTCCATATGCTCATTCCCTCCACCACCA
CCACCACCACTACCGTCCCCTCCTTTTATTCCATCCACCACCACCACGTCCGCCTCGCCACCACTGCGCGCCCGTCCACAGC
CACCACACCACAACCACA
>SsLRR-RLK417
TACCTGGATCTCGGTTAAGGCATCGGACAGCGCACAGAGCACACGCAGCAGAATATGAATCTTCTGGTGTAGTGCTTGCACC
GGCATGCCACCAGGATCTAGAGCAACTATGAAGTCAACAAAAGCAAGCATTTTCAACATGGTTTCTTGGATATATCGTGCAA
ATAGGAGCAGTTCCAGAATGGAACCCTCAGCCTTGGGATCTTCTTCTTCCTCCTCATAAATACTAGGTAGTAGGCCCACGGAA
CCACGATCTGGAAGAGCGACACCGCCACGACCGGATGGTCTGTGTGATCTCATGAAGAGCCCGGATCCAGCACCGTGCCCC
ATCAGAAGTGCTCAGTTCTTCCCATGCAGACGCACTGCTAGCGAGGTGCAGAACCCAGGCGACGCCCAGCTCCGTGAGCCA
CCTCTCCAGGACACACATGTCGCCGTCGCGTTTGATGCTGCAATCCACCAACGGCACATCGAGGAATTCATGGGCAAGCAG
GCTTTTAACGCGCTCTGCATCCGTTCCTTCTTCGTGGATACTATAGTAGTCGAAGTGCATGGTCGCCTCCATGGAGGCAGAGG
AGGACAGACGAGAGGCGTTAGATGTGTAGGCAGCCGAGACTATGGTACCTGATGAAGATGAGCTGCCGCCGCTGCTAATAA
TGGTGCAGTAGTAGTAACTGGATCCCGTCTGCTCGCGGCGGCAGCTCCAGCTGGTCCAAGAAGAAGGCCCGTGGCCGCCGG
CGTCCACGCCCGTGGCCTCCATCTCCATGCGAAGGTAGCTAGCTACCAAATCGGCCCCTAGCGACTATGTCAGTTGTCTCTG
GTGTGGGACGCGTCGGCCGGGATCGAGCTTGAAGTGATTGCGAGAGGCGGGCGCCCAGGTGGGGCGGTGGCCAGTGGCCG

GTGGGGTGCGACTGTGAAAGCGGAAGGCTACGCTCTTTTCGTGCATCTTTACGCGTGAGTGCAAACTTTCTTGGCTGACTTG
ATGTCGNNNNNNNNNNNNNNNNNNNNNNNNNNNNNNNNNNNNNNNNNNNNNNNNNNNNNNNNNNNNNNNNNNNNNNNN
NNNNNNNNNNNNNNNNNNNNNNNNNNNNATTCGTCTCGTCCATTTCGTCTAAACTGTGTAATTAGTTTTATTTTTTTATCTA
TATTTAATGATTCATGTATGTGTCAAAGATTCGATGTGACGAGAAATCTGAAAAATTTTACAAAATTTTCTAGGAACTCAACA
GGGCCTTATGGTGCATGGAACAATGGATGCTACTAGTCCGGCTAGTCGCCCGCGTCTCTGGCCCTGGAACCACTGCAAAAGA
TTCGTCTCGACATTTACAAATAAACTGTGCAATTAGTTATTTTTTTACCTACATTTAACGCTCTATGCATACATTTAAAGATTCG
ATGTAACACTTACTATGCAAAATTTTTTACCTCTTTTTGGCATCTAAACACTGCCTAAATCATCGTGCTCCGTAAGCAATAGCA
TCAGCCCAATTTTAGCAAAACGAAAGAAGCCGGTAAAGTCACCTCCAAAAATGAAGCCAACGGCTGTGCCTGTGCTGTGCT
GTGCTGCGCTGCGCGGCCAAGGGTTTATGGTGCATGGAACAATGGATGCTACTAGCCCCTAGTCCGGCTAGTCGCTCGCGTC
TCTGGCCCTGGAACCACTGCAACAAGTCTGCAAGCTTGCAAGAGCAAATTGATGGCGCCCGTCATGTCCCCTCCGACCCCG
TCCCGTCCCGCCTCTGCTGCGCCTACGCCTATCTGACACCGCCCGCCGTCAGCCTGCCGTGCGCCGAGGCCGAACCACCATC
CAGCACCACCCCTCCTCCTCCCGCCCTGCTGCTTGCCTTTGACCTGCCTATCATCAGCTCGCCCGCCCCGTCCCCGCCGCCGC
GATTCCATTCCATATGCTCATTCCCTCCACCACCACCACCACTACCGTCCCCTCCTTTTATTCCATCCACCACCACCACGTCCG
CCTCGCCACCACCACAGCCACCACACCACAACCA
>SsLRR-RLK288
GCAGGAAGAGTTCTCTCCGGCAGCTTGCTTTTTCATACTACAAATTTGCGGCGCCCGCAGGAAGGTCACCTTCAAATCGGCA
CCATCGATCAAGATCCTGCTCAGTCACCTCCAAATCGGCGCCGATCAAGGTCCTGTTCACATGCTGCCTGCTGGTGCTGGAG
GAGCACTGCAGTGGAGCAGAGATAGCCTGCTGGTCTGTGTTGCTTAGCTTCAAATCAGTCATTCGGCTCAAAGACGATCTCC
CTTTAACTCGCTGCGATCAGCGCATCAATCATGCACCGTCCGGCCGTCCATGCTGCGTACGCCCTTGTGGCCGGAGGGAGCC
GGAGCCATGCGATGAGGTCCATCGCTTTGCCGAACTGGAGCCGCCGGACCGGAGGGAGTAGGGACGGCGGGGCCGCTGCT
GCATGCAAAGCGGTCAAGCGCGGCCTTTTGTTTTGCAGCCGGCGCGCCCCGGCACCAGCAGCGCCAGCGGCCGCTTTATTT
CTCTCACCTCATCTCACCTCCCCTTTGCTTTCTTTTGCTGGCTGGCCCCTGTCCCCAGTGTCCCGTGTCCCAGCCAGCCAGCC
GGAGCCCGCCGCCTTTGAACGCCGCTTTAGCCGGGCGGCCTGCCGTGTGGGTTACCAGATCACGCATCGCCTTGGCCTTGGC
CTGCAATTGCACCTGCCTCCCACCTCGTCGTGGATGGGCATTGGCAGGGTGACATGGAACCTGTCCCTTCCTGGCTCGCCTT
CCTATCCTGCCCACTCAGATCACTCGCCTTTAGCATCTAACTCTCTTTCCCTGTGACTGAACACAAGAATGGAGTATCATTTTT
TTTAGTTTTGGGTTTCAGAGTTATTCGTTGCTTCACCTAACAAGTAACCAATCTAGAGGATATCTGCAAGGTGTGGTTCGAGA
ACTCTGGTTTCATGGTGACTGCGGCGGGCCGTAGCTCCTAGCAAAGCGACAATCCAGGAGGTGCAGATCTCCCCGATCCATT
TGTTTTGTGTGCTCTACACAATCATTACCGAAACTCATAGGGCCGGCCGGCCGGCTCTTGTACGTTGATGGAGACCGGAGTG
CATTCCTCTTTTGCTGTCGGACGCGACAATGCCATCATCATAATTCAAACAAACCTTTCCTCCAAATACCTCTTAAGTCATAAC
CAATAGCACAAAAGATTTGAACCAACAACCAAAATACAAATACCAAAGGGACCATGCATGCATGTCCTTGGAGAATTTCAA
AGCTAGAGAGAAACTCCCTAGCACACACAGTGCATGCCACTTGCCACACCCATCCCCATCTCTTTTGCCATGGCTGGCTGCT
TCCTCGCCTACAAAAAGCCTTCAACAGTTGCCTTCCCTCTCCTCCCAAAGAAAAGAAAAGCAAAAGGAAGGAGATGAGAG
AATCACACTAGCACCAGTGAAACCAGAGAGCAGAGCAGAGCAATGCACTAGCTAGATAGCAGGGTACTACTGCACTGCGCT
GCTGCTCTATAGTGAGTCTAGACACCCTTCACCCACACGCCATATATATCTCAAAAGACAAACCCCCTCCATGGTCATGCCAC
AGCGAACGAACAAACGAACATATTCCCCTCGATCGCCCCTGGCAGACACTATCATCTTCCACCTCCCAGCTTCACTCTCAAG
CCTCCACTCCATCTTCTTGTGAAGCTTCTCAAGATTCTCGCATGCCTTTACCCCTCCTCCACTTCTCTCTGCCCAGCTTACTAA
ACCACCTCCAATGCCGGCGCCAATGGCAGCAGCTCAGTTCCTCTAGCTAGTCTCCTGAACAGCAATTCACAAACCCGGAGA
CAAAGAAACAATCGACGCACCCCGATCGATGAGGCCCTCGCCACCTCTGCTCCTGCTCGCGCTCCTGCATTTCGCGTTCCTG
TCCACCAATGCCGCCGCCGCCGCCGATGGCAATGGGACCGGGACCGGCGACCTGCGCGGCGACACGATGGCGCTGCTTGCG
CTGAAGGCGGCGCTGGGCTGCCGCCCGGTGCG
>SsLRR-RLK134
ACATCTGCCCAATAAGCATCTGACTTCTTGCTATTTCCATCTACTGGGTCCTTTGAATTATGCAACCAAGCACTGGCCTACAG
AAACAACAAAAAAAAAATAGAATGAAAAAAGACAATCGTTAATCTTCAAGTGTGCATGAAGATAATTGTTTCAACGTACCA
ATCGTACGTCTTCAGGGACAGTCCAATTCAAACGATGTTGAGTTCTAGAATCCTCATCGACATCAATTGTTTCATCTTCTTCTG
CATCTAAATCATCCACTATATCATCAGTTTGCTATGCTGGAGAGGGAGTTCTTGTAGCACCCAGGTCAATTGGTGGTACTGAA
GACTGACTCATTCCAGCACCAACAAAGTGAAAATTTTCATTCCCATTAGGATGGTTAGCAAATGGGTTTGACTGCAGAAGAT
TTGAGAATCCTCCTTGTGGGTATAACCTGCAACCATATGAATTTCCATGCTAAAATTAATTATATCTTCACCAAAATAGCACAA
CATGCCATACAACACTATTTACTAAAATCCACAGAATGCCGATAAACTTCAGTGGTCACAAAAGTAATTGCAGTAGCTTCTCT
GTCAAATTAACACAAAGGCATTCTAATTTCAAGACATTAAGTTTCGGATGTCAAAAAGGAAAAAGGAAGAATATCTTGCAGA
TCTCATTAGACTTAGTAGATAACCAATCTATAATCCACATATCCACTTCTCTGAAAACTTACCCTTCACTAGGAGATGACGTTG
GCCACCATCCACCAACAGGAGAAGCACCAATTCGACCAACACCAGCATCTTGAGGAACACCGGTGGCTCCAGCTTGAGCA
CGGGTTCCAGCAGGTCCGGCGACCGGACCACAGGTCCCAGCAGCACGGACGGATGGAGGATGGGTTCTAGGGGCGCCTTT
GCATCGCCGTCCATCAGTCTTTGTTAATCGATTCATTAGTGAGATTGGGGGATCTCTTTGGTCAGAGTGGATGGGGGAGGAG
ATGGAATCAAAGGGAGCTATGGATGCACCTGGATATGAACCTCGATGGGAGATGGGCAGCATAGGGAGGGAGATTAATAGGT
GGGCTGGAGGGGACAGGGCCTGAAAGAGGCACGCTTCCTGGAAATTTTGGCGGCGACGACGGCTTCACCTCCGCGAGTGC
GCGCGGGAGATTGAGGAGTGCGGGAGATGGCGCGCGCGTAGGAGAGAGGCGCGCGGGAGAAGAGCGCACGCGCGGGAGA
AGACCGCTTGGTGGTGCGCGAGCGGAAGAGGGGCGGCGGCGGCGTACGGGATCCATAGACTCGACTCTGCCCTCACCCAA
CGCGAGTCTCGTGGAGTCTTCCGGCCTCGAAGGGAACGACGACTCGACGTCTTATTTAGACGTTGTTTCTTTGTTTTTCCCC
CTCTTTCCTCATTTAATGGCTTGCCACGTCAGCAAAATACCTACGTGGCAGTGTAATTAATACGGATAGACTCCACGGTGGAG
TATGCACTGTGAGTGCCCTTAACAAACTTTTGTTTCATCGTTTGTAAACTTGCTAACAAACAACAGCATTGCTGGAACACAG
TATAGCATCAAGCAACAGCACTGGATCCCGGATCCTGGTTGTAAACACGTATCAACCCAGAAAGCAACTTTTCATAAACAAA
ATCCAAATCAATACACACTTGTCCCATCCAAAAAAGTAAAGAAAGCCATACAAAAAACAAACCAAATCACCAAACAAGAGA
CAAGTACTCCTACTCAAAAGATCAATTTTTTCTGTTAAAAAAGAAAGAAAAGACAAGTTTCTCAAAAGATCTTCCCCTCTAG
AAAATCCAACAGGAATCACATGCTTTTTTATAGCCGCACTAGAGGTGTGTCAGTGTGTGCAGTGTGCTGTTTCATCCCAGAA
CGACCAGCCAGCCGAGCCTATCGAAAAGCTCTCTCCTTTTCTCGCGGGCCGCGCGGCCTCACTCCTTTGTCTCCACTCTCCC
GGCGGAGACGGAGCCTCGATCGAACCCAGTGCCAA
>SsLRR-RLK118
ATCGAAGGAGTACAGATCAGCAGGGTCGATGCTGCCATCTTCATCCATGGCAGCAAAGGATAGCAACGAATAGTGGGTAGC
AGAGAATAGTGGGCAGCAGATAAGAAAGGGAAGCAGTAGCAGAATTGCCATGCCTATGCTATATATAGCCACAATTGCTGTG
GGCAGCAAAGGAGTTCACACTTGCATGCAAAAAAAACTAGTGGAAGGAGTTCACACTTGCATGCAAAAAAATTAGTGGAA
GGAGTTCACACTTGCATGCAAAAAACAAGTGGAAGGAGTTCACACTTGTATTAAAATACCACTGATCACAGGAATTAAAATA
ACACTGATCACATGTATTAAAGTACCACTGATCACAGGAACTAAAATAGCACTGATCACATGAATTAAAATAGCACTGTCATA
TGAAATAAAAGGATTTCAGTTACCTTATAGAAATTACTTCCATGGTCACATTTGTTTGCACACACTTGCTACTGCATAATCAAT
TCTAGAAAATTACAAGACCATCAGTTACCAATTCCAAGCAATTAAATAAGCAATTGAAGGGTTGACAATGTCGTACCTTAGG
AAATAAAGCAAGTCTCAGGCTCTCCAGTGTCATGTTTCTCTCAGCCAAAGCATCTGCACTAAGCTGATCAGTAGGTGCAAGC
AACAGCTGTGTGTAAGTATCTAGCATTTTACACTTAGTTTGTTCATGAGCTGCTTGATGATTAAGCCTAGCTGCTTCAATCTTC
TTATCAGCCAAGCTTTGCTGAATTTCAGTCATCTTCTCTCTGTCTTCCCTATTCTTGGTGCTTGCTTCAATGAACTTCTCAAATT
TTGCACTAAAATCAACCAAAGCATCAGATTAGTTCTTTTTGCTATTGAGGCCTTTTTTTTTGCTTTCTTATGTCCCATAGGTCG
CTTTTCTCCATCTAATTCTAAATTCACCAGCTGAGCTGGATTAGCCTTTGCACTCTTTTCCTTTTCCTTTTCCTTCTTTAACCTA
GCAAGATATGATCACCACTTCTGCTGACCACGTACTACCTTCCACATATGCAGCAGATGGAAGGCCTTACCATTATTTGGCC

GGCCCACATCTCTAATGCTTTGTCAGTTAACTGGTCATCACTCATCCCAATTTGCCACACTCTACCGGCATTAACCCAAACTC
CATGAAAATCAGTTAATGGTTTCTTAGAACGATCCCAACGTATTTTCAGTTGGTTCCGGTACCTCTTACGGCTAGTTTCTGTGT
TCATGTTGTATTCTTTAGTAACATCTGCCCAATAAGCATCTGACTTCTTGCTATTTCCATCTACTGGGTCCTTTGAATTATGCAA
CCAAGCACTGGCCTACAGAAACAACAAAAAAAAAAACAGAATGAAAAAAGACAATCGTTAATCTTCAAGTGTGCATGAAGA
TAATTGTTTCAACGTACCAATCGTACGTCTTCAGGGACCGTCCAATTCAAACGATGTTGAGTTCTAGAATCCTCATCGACATC
AATTGTTTCATCTTCTTCTGCATCTAAATCATCCACTATATCATCAGTTTGCTATGCTGGAGAGGGAGTTCTTGTAGCACCCAG
GTCAATTGGTGGTACTGAAGACTGACTCATTCCAGCACCAACAAAGTGAAAATTTTCATTCCCATTAGGATGGTTAGCAAAT
GGGTTTGACTGCAGAAGATTTGAGAATCCTCCTTGTGGGTATAACCTGCAACCATATGAATTTCCATGCTAAAATTAATTATAT
CTTCACCAAAATAGCACAGCATGCCATACAACACTATTTACTAAAATCCACAGAATGCCGATAAACTTCAGTGGTCACAAAA
GTAATTGCAGTAGCTTCTCTGTCAAATTAACACAAAGGCATTCTAATTTCAAGACATTAAGTTTCGGATGTCAAAAAGGAAA
AAGGAAGAATATCTTGCAGATCTCATTAGACTTAGTAGATAACCAATCTATAATCCACAGATCCACTTCTCTGAAAACTTACCC
TTCACTAGGA
>SsLRR-RLK96
GCATACGTCAAAAACTCATGTGATGGAGAAAGAGTGAAAAAACTTAGAACTTAGAATTTGGGACTCTACCATCGTCACTCG
ATCCCCGAGCAATCATCCGTCTGCATTTTTTTTTGACAACATCCGTCTTCATATTGAAAAGAGATTGAAAACCAATCACAATA
GACTACCGAGAGACTTGATCAGAAACTTAGAGTGCATTCGACACCAAAAAATGTTTCAGATTCTTAATGGAATATTTGTCAA
ACGGTTTTAGGGAGAATAACTTGTAATCAATTTTTATTTTTGTACTAATAACTCTTAAAATATAGTTTCTCCTGATTCTAGTTCT
ACTTTTAAGAATCTAACGAGCTAGCAAAACGGTTCCAGAAGGTGAGTCTAATCATCGGAGAAATGTTTATTAAAGGAATTTG
AATCCAAATTTTTTCTTCAAGAATAGAACCGTGCCAAACACACCAAGTATTCCACTATGTCGTTTCATCAGTCGTTAACCCAC
AAATGATCGAGAGCTGGCACTCTGAATTAAACTAGCGAAGATAGTGCATGTCAACTAGTCTCTCTCTCCCATACTATTTATGG
CTTTTCCTCGAGAATTAAAGAGGAGTGAAAAATGTGTGGTGCATAGGAATTAAGTATAGAGATGTTTTGAAAAAAGAGAAA
ACACAATTAAAAATGAGAATGACAAATAATTTGCGACAACGCTACAAGGCTAGAATAACTTTCATTACAGAACAGGAAAGTA
CCTCTTGTTTAGTTGAAGAGGCTATTTGGATGCCGGTCGCCACTCGCACAGCTACGGTGCGATGGTGCTATACTTGTGCTAGG
GAAAAAAAAATTGGTCCCATGGACTGCAAGGAGAATAACAAAGATTTTGCTATGTTATTCTCATGGCTGCCTTGTTCTAAGGT
AGTGTTTAGATGGTGTAAAAGTTGGTGTAAAGTTCCTGTAGCACTTTTCGTTGTTATTTGGTAAAATTTATCCAATCATGGACT
AACTAGGCTCAAAAGATTCGTCTCGACATTTACAGACAAACTGTGCAATTAGTTATCTTTTTTACCTATATTTAATGCTCTATG
CATGTGTCTAAAGATTCGATGTGACGGAGAATCTTGTAAAATTTTTAGAAGTTTGAAGGGATCTAAACACTGCCTAACCAAG
CAGTGAGACTAGAAATGCGTGGCCATAACCTAGGATATGTGCAAAAACATAGTACTACCTTCCGTTAACTATTAGACTTAAGG
TTGGGACAACGAAATCAAGAAGGAAACAAGCTGTTTCTTCTTTTGACTGAAACATCCAACAAAGTTAATTGAAGGTTGTAA
TAAAGCCCTATCTAGATGCTTTTTAGGGCCTGTTTAGAATAAACTTTTTTGTAGAAATCATGCAGGAGGTTCATAGAAAAAAA
ACGTGAGAAGTGAATTTTTTTCCACATTCCAAACGGGCTCTTATTCTCTTGAAGCTATTAACAAACTTTTGTTTCATTGTTTGT
AAACTTGCTAACAAACAACAGCACTGCTGGAACACAGGTATGGCATCAAGCAACAGCACTGGATCCCGGATCCTGGTTGTA
AACACGTATCAACCCAGAAAGCAACTTTTCATAAACAAAATCCAAATCAACTAACAATACACACTTGTCCCATCCAAAAAAG
TAAAGAAAGCCATACAAAAAACCAACCAAATCACCAAACAAGAGACAAGTACTCCTACTCAAAAGAACAATTTTTTCCTGT
TAAAAAAGAAAGAAAAGACAAGTTTCTCAAAAGATCACTTTATCTTCCCCTCCAGAAAATCCAACAGGAATCACATGCTTTT
TTATAGCCGCCCTAGAGGCCTACTGAGTAGGTGTGTCAGTGTGTGCAGTGTGCAATTTCATCCCAGAACGACCAGCCAGCCG
AGCCTATCGAAAAGCTCTCTCCTTTTCTCGCGGGCCGCGCGGCCTCACTCCCTTGTCTCCACTCTCCCGGCGGAGACGGAGC
CTCGATCGAACCCAGTGCCAA
>SsLRR-RLK5
CCCTTTTATAGGGTAAGAGGAGGCCGGGATGCCGGGGGCCTTGAGATGAAGTTTTGTCGATCCCTGTGGATGTGGATGTGGC
TGTAGCAACGTGCCTCAGGAATGGCTGGCCATCCCCGGACATTGTGGTGACGTCGGGGACGAGACGGTGAAAAGACCCTG
AGTCCTGTGCCATGTCGGCCTTTCTTCCCTGCTGACCGGTGATGGTCGTCGAGGCCGAGTCGAGGCTGGGTTGAGGTCGGAT
GGATAAGGCCGAGTCCCCGAGATCGTGGGGTACGGGCCTTATCGTATCCTCCCTCGAGGGTGATCAGTGGTAGAACAGTGCG
CGGTAGGGTGCGTACAGTGACGAGTTATCACCTCCCATCCCGGGATGGGCAGTAAAAAGTGAGATATGACACCGGTATTGTT
TCCACGTTGATCACAGCTGTAGCCATGACGGTAGGGGGTAGTAGGTAGTTGCATTAAATGTCCTGACTCTTGACTCGGAGTA
GGCAGGTAAGATGGGTCGAGCGATTCGACCCGGGGTCGTACGAGTCGGAGTTTACCGGTAGTCGAGATACCTTGTCGAGCG
AGCTTCGTACGAATCGTGACGCGGTCAAGGCGTCCCGCCGAGGGTGCCTCGTACGAGTTGGAGTCGGAGCGCGGTCGAGA
CGCTCCGTCGAGCCAGCCTCGTGCGAGGCGGAACGTAGTCGAGGTGCATCGTCTACTGTCCTTTGGTTTTGCTGTGTGGGTG
CATTAGTGCTTCGTTCGTTCTAAACGCTGATTGTGGGTACCCCTATACTATTGGTACCGACAGTAATGTTATATAGTTTGATAAC
ATTTCTATGATAAGTCTAGTAAGACATGTTTGTTTTCCCTTTAGTATATTACAATATCAGGAATTCAAAATGAAAGAAATAGGC
AACTTTTTAGAATACTAGGAGTGATGAACTATTTTTTCATCACTTGTCCATTGTGATGCTAGTGAGCTAGTCATATAAGAAACT
AAGAACTATTGTTTGTCCTTCGCTCATATTTCGTGGGATGTAATGCTTGATTTTTCTTATATGTGTAAATATGTACACTTGCTATA
ATATTTATTTTAACATGTAATATATATATATATATATATATATAAATAGTTCATACATACAAATCTTTAGTCCATTTAACTATGTTCAAA
CACTATAAAAATTGTTAAACTCCAAAAAATATGTGAGTACCTATTTATCGTTTATCATTTAAGATAACACCATTTGTTACATGCC
TCAACATATGGTCTCCATAATAACCCAAGAATCCTTCACCTTTTTACATTCTTGCTTCTCGGTGCAAGATACATTTTAGAATTCT
CACCTAAATTATATCCCATATGTTGTGAATATATTAAATTTTACGTATCCTAATAGATAGATAAAAAACTAACCAATGAAATTATA
CGAGGGAGTATTTACAAACCTATGTGTAATGGTGCCCTTTACTCTCTTTCGTTCGTGTTAGATCCCCTTTAAAATGCAGAAATT
TTACATGAATGCTATAGAAATTTTACAGTAATCACCTCATTTTCACATAAAAACTCAAGAAAAACAAAAAAATCTCACATTTC
AAAGAGAGCAATATTGGTTGACTCAAGAGTTACAAAATCAGTAAGAAGAATAATTTCCAAAAATCATTTGAATATTTAAATGT
TGGATCCGGCACATTTCGCGTCCGCAGGCTCCTTCTAACAACGTGTCCGTTCACCGAGCACTGTATCTGATCCGATACATAGG
CTGCTGATGGCTGATGTTATTTTCCTGATAAGTTTTTCTTTTTATAAGAGACGGCTGATGTATACCGCAAAACCTTGAGGCCA
TGCAAATAAGGAGCCAAAGGAGCACAATGCAAATCTAAAAAATTCCAGGCTGACATACAAAACAACAACGAAAAGAAAAT
GAACGGAAAACAACGCACTCCCTATAACCCACTCTGGCACTCTGTCCGCCTGCCTCGGACACCCACTCCACTCACCCCACC
CATCCA
>SsLRR-RLK37
CCTTTTATAGGGTAAGAGGAGGCCAGGATGCCGGGGGCCTTGAGATGAAGTTTTGTCGATCCCTGTGGATGTGGCTGTAGCA
ACGTGCCTCAGGAATGGCTGGCCATCCCCGGACATTGTGGTGACGTCGGGGACGAGACGGTGAAAAGACCCTGAGTCCTAT
GCCATGTCGGCCTTTCTTCCCTGCTGACCGGTGATGGTCGTCGAGGCCGAGTCGAGGCTGGGTCGAGGTCGGATAGATAAG
GCCGAGTCCCCGAGATCGTGGGGTACGGGCCTTGTCGTATCCTCCCTCGAGGGTGATCAGTGGTAGAACAGTGCGCGGTAG
GGTGCGTACAGTGACGAGTTATCACCTCCCATCCCGGGATGGGCAGTAAAAAGTGAGATATGACACCGGTATTGTTTCCACG
TTGATCACAGCTGTAGCCATGACGGTAGGGGGTAGTAGGTAGTCGCATTAAATGTCCTGACTCTTGACTCGGAGTAGGCGGG
TAAGATGGGTCGAGCGATTCGACCCGGGGTCGTACGAGTCGGAGTTTGCCGGTAGTCGAGATACCTTGTCGAGCGAGCTTC
GTACGAATCGCGACGCGGTCAAGGCGTCCCGCCGAGGGTGCCTCGTACGAGTTGGAGTCGGAGCGCGGTCGAGACGCTCC
GTCGAGCCAGCCTCGTGCGAGGCGGAACGTAGTCGAGGTGCATCGTCTACTGTCCTTTGGTTTTGCTGTGTGGGTGCATTAG
TGCTTCGTTCGTTCTAAACACTGATTGTGGGTACCCCTATACTATTGGTACCGACAGTAATGTTATATAGTTTGATAACATTTCT
ATGATAAGTCTAGTAAGACATGTTTGTTTTCCCTTTAGTATATTACAATATCAGGAATTCAAAATGAAAGAAATAGGCAACTTT
TTTTTTTGAGGGAGAAATAGGCAACTTTTTAGAATACTAGGAGTGATGAACTATTTTTTCATCACTTGTCCATTGTGATGCTAG
TGAGCTAGTCATATAAGAAACTAAGAACTATTGTTTGTCCTTCGCTCATATTTCGTGGGATGTAATGCTTGATTTTTCTTATATG
TGTAAATATGTACACTTGCTATAATATTTATTTTAACATGTAATATATATATATAAATAGTTCATACATACAAATCTTTAGTCCATTTA
ACTATGTTCAAACACTATAAAAATTGTTAAACTCCAAAAAATATGTGAGTACCTATTTATCGTTTATCATTTAAGATAACACCAT

TTGTTACATGCCTCGACATATGGTCTCCATAATAACCCAAGAATCCTTCACCTTTTTACATTCTTGCTTCTCGGTGCAAGATAC
ATTTTAGAATTCTCACCTAAATTATATCCCATATGTTGTGAATATATTAAATTTTACGTATCCTAATAGATAGATAAAAAACTAAC
CAATGAAATTATACGAGGGAGTATTTACAAACCTATGTGTAATGGTGCCCTTTACTCTCTTTCGTTCGTGTTAGATCCCCTTTA
AAATGCAGAAATTTTACATGAATGCTATAGAAATTTTACAGTAATCACCTCATTTTCACATAAAAACTCAAGAAAAACAAAAA
AATCTCACATTTCAAAGAGAGCAATATTGGTTGACTCAAGAGTTACAAAATCAGTAAGAAGAATAATTTCCAGAAATCATTT
GAATATTTAAATGTTGGATCCGGCACATTTCGCGTCCGCAGGCTCCTTCTAACAACGTGTCCGTTCACCGAGCACTGTATCTG
ATCCGATACATAGGCTGCTGATGGCTGATGTTATTTTCCTGATAAGTTTTTTCTTTTTATAAGAGACGGCTGATGTATACCGCAA
AACCTTGAGGCCATGCAAATAAGGAGCCAAAGGAGCACAATGCAAATCTAAAAAATTCCAGGCTGACATACAAAACAACAA
CGAAAAGAAAACGAACGGAAAACAACGCACTCCCTATAACCCACTCTGGCACTCTGTCCGCCTGCCTCGGACACCCACTCC
ACTCACACC
>SsLRR-RLK51
GCCAGCACGCCCCACGCCTGCGCCACAGGCCACTGGGCACACCCGCCGGGACACCCCCTGGGACGCGTCGAGGATGCCTG
TCGTGGCGTCGAGAAGATCGCCAGCGTCTTGAACAGCATCTGCACCCTGCCCAGCGCGCTAGGCGGCCTCCGGCCTCGGAG
AAGCGCTGATGGGCCGCAGCCGGCCGCTGGGTGGCACCGACGGCGTGGCGGAGGCATAGATCCCTAGCCACGGACGTCCT
CGGAATCGCGAGTCGCAGAGGGAGAGGAGCTGACGATGTTTGATTTGTTTTTCTTTTTTGATGGAAAAAAGACAAGCTACTT
GCAACAAGAGGAATCGAACCGTGGTCGTGGACTTCCCGGCGTGGGCGTGCGGCGGGGAAGCGGGCATGAAGGTCGCCTGG
GCGTGGTGAGTTTGAAGGGGGTGGGCGTGAGTTTGAAGCGCATCAAACTTTGATCGACAGCCATCGGATCGTGTTTAGGTG
TTGGTTTAGCCCTTGATTTTAACGAAGACGATTTTCTGTGTGCAATTTTTTGGGTGCACGAAGGTTAGAGCCCTCAGCGGGG
GACGTTTTCTTTGGTGGATCTAAAATAATTTAAGATTTCGATTATAGTAGAGATTTAATTAAATTTATAAAAAAATATTATTGTA
ACATTTATGAATTCAAATAAACATACTTGAAAATATATTTTGTAATTAATCTATTTTTTTATTTGTATCATAAATATTAGTACTTTT
GTATATTTAGTTAATTAAATTGTTTGATTCCTCGAAACACAATAATTGCCTTTTTTAGATGGAGAAAGTAGGTAATGTTATATAG
TTTGATAACATTTCTATGATAAGTCTAGTAAGACATGTTTGTTTTCCCTTTAGTATATTACAATATCAGGAATTCAAAATGAAAG
AAATTGGCAACTTTTTAGAATACTAGGAGTGATGAACTATTTTTTTATCACTTGTCAATTGTGATGCTAGTGAGCTAGTCATAT
AAGAAACTAAGAACTATTGTTTGTCACTCGCTCATATTTCGTGGGATGTAATGCTTGATTTTTCTTATATGCGTAAATATGTACA
CTTGCTATAATATTTATTTTAACATGTAATATATATAAATAGTTCATACATACAAATCTTTAGTCCATTTAACTATGTTCAAACACT
ATCAAAATTGTTAAACTCCAAAAAATATGTGACTAACTATTTATCGTTTATCATTTAAGATAACAACATTTGTTACATGCCTCGA
CATATGGTCTCCATAATAACCCAAGAATCCTTCACCTTTTTACATTCTTGCTTCTCAGTGCAAGATACATTTTAGAATTCTCACC
TAAATTATATCCCATATGTTGTGAATATATTAAATTTTGCGTATCCTAATAGATAGATAAAAAACTAACCAATGAAATTATAGGA
GGGAGTATTTACAAACCTATGTGTAATGGTGCCCTTTACTCTCTTTCGTTCGTGTTAGATCCCCTTTAAAATGCAGAAATTTTA
CATGAATGCTATAGAAATTTTACAGTAATCACCTCATTTTCACATAAAAACTCAAGAAAAACAAAAAAAATCTCACATTTCAA
AGAGAGCAATATTGGTTGATTCAAGAGTTACAAAATCAGTAAGAAGAATAATTTCCAAAAATCATTTGAATATTTAAATGTTG
GATCCGGCACATTTCGCGTCCGCAGGCTCCTTCTAACAACGTGTCCGTTCACTGAGCACTGTAGCTGATCCGATACATAGGCT
GCTGATGGCTAATGCTATTTTCCTGATAAGTTTTTTCTTTTTATAAGAGACGGCTGATGTATACCGCAAAACCTTGAGGCCATG
GAAATAAGGAGGCAAAGGAGCACAATGCAAATCTAAAAAATTCCAGGCTGACATACAAAACAACAACGAAAAGAAAACGA
ACGGAAAACAACGCACTCCCTATAACCCACTCTGGCACTCTGTCCGCCTGCCTCGGACACCCACTCCACTCACCCCACCCAT
CC
>SsLRR-RLK183
GCCAAGATCCATTCAGGGTGTTGTTTTTTTTCATTTCTATGATGTTGTGACATATGGTTTTTAATGTTGTATTGACTTGCTCGAC
ATGTTATCCTGCATAAGTAGATTTTTCTATGTTTCACGGAGTTGGAGATGGATATCTAGATGTATTTTTGATGTCGCGACAACTG
TAGCTAGATGTACCAACAACAAGTTGTTTTTCGTTGCAATGTTTGGTATGTGTTATCCTATGTTGCAGTAGCCTATGTTTTTGAT
GGTGCAACAAGCGACTTAGTAACTTGTTTCAAACATTCACTATCATGTTGTAGCATGTTTTCTCTAGTTGTTTTCTATGATGAG
TTGAGATGTTGAAATAATTGTGCTTTATAGTTGTAGCCATGCATATATACGTCTCTCTTCCTCCTCTACTCTGCCATGTTTGGGC
GGTGCAACGTGAGGGTGGTGTAAGTTGGTGCGCGGTTGCGCTCCAACAAGCGTGGTTGCTCCACCATGAGCGGGCAAGAC
AACATACCACAGGAATAGAAAAGGAGCTGGCACAACATCCGACTATCTGAGGGCTAGCTATTTTGTTTATGGATAGACAAGG
TGGGAATCTTCTCTCCGGAGTCAAGAGTCATATATTCATAGTGTTTACCATAGTAAATGGGCCATGAAATTGTTGCATTGCAAA
TTATGACTGTGCTTAGAAATTGGTTGACTCTATTGCTGGATACATCTAGGCCATTGTCAAAGTGGCGTCCGTGATGCTGACAT
GGCATTCGGGCGCATCAGCTATGTCTATCTTGCCATCGTCATTGAACCTCATATTTACCGATGGTAGACATCTTAGATCGAATC
TGGTAAGAGGGTGTTTGGTTCCCCTTGTTTTTTAACACGTATCACATCGAATATTTGGACACATGCATGAAGTATTAAACATAG
GCTAATTACGAAACTAATTGCACAGATTGAGATTAATTTACGAGACGAATCTATTAAGCCTAATTAGTCCATTATTAGTATCCG
AACACCTTATGGTCAAACCCATGGTCAACACCTGTTAAAGTTGAACAGGGCGAACCAAACAAGCCCTAAAGCTCATCACAT
ACCTATGATTACGGCTCAACTAATATCAACCTAAGGTACTTAGTCCAAGAGGTTGTCCGGAGGGTTGGTGCCTGGTCAGTTG
ATTCGTATGAATTAGGCTATCACAATGTTAATGGCACGTTATATTAGTCGTACTATGGTTACATGCAACCTCAACTACAAGACC
CTCTTGAGAGAAAAAAAAACCAACGTCTATTTTTGTGTTTTGAGTGTCTTACATTGACAAAAACCCTCTTTAGCGAAACAAAC
AGTGCCCAAATTTACAGTTGATGTTATTAACATTTATATCTCGAACAAAATTTACCTGGAAGCGAGCAAGAAAAATTTACTAG
TCCAGTACTGTACTCGTACTAAGCCTTGTCACCGACAGACACGAGTCCGAAGCAAGCGTTGAACGGAGGTACAGGTGTGGT
TTGACCAAAGAGGACCCCACTGGCAGTGGCAGAGACACCCGACAGGATTTGGCAGCGAGAATGTGAAAACAAGACCGAC
ACGCAGGTCAAAGTCGTCGTCTCGTTTTTTTTACCACCTGATTGTCTGCAGCCAACATCATAATTACGGAGTACTAGTAATTA
CGGAGTAATTAACGTACTACAGTGCTCTCACCCGCTCCTCTGCTCTGTGCTCTGTGCTGTGTTCTGGCTCTGCCTCTGCTACT
GCCCTCCAGCTCTCACCCGCACCCGCCCACAGGCCACTTGTGTCGGGGCCCCTCCTCCTCCCTGTTGCTTTCTCTGCAGTGC
GGGGTAGCTTCGCTTCCGCTTCTCCTCCCCGAGCCACTGGCCATCGCCATCGCCAGTACGCCACCACCATTCCATTCCCATTC
TCTTCCCCTTCTTCGCCCGTGCCCATGCCGACGCCTGCTCTGCCCCCACAAGCACAAGCACACACCTTCTGAGCCACCAACG
CCATCGACCA
>SsLRR-RLK155
GTAGTCTCAAAATAGGTGACTTCTTAAATAGAGAAGTAGGAGGTGTATTTTAGGTTGTATTGTTGGAGATGCTCGGTACTTAG
TCCAAGGAGGTTGTCCGGAGGGTTGGTGCTTGGTCAATCGATTCGTATGAATTAGGCTATCACAATGTTAATGGCACGTTATA
TTAGTCATACTACAGTTACATGCAACCTCAACTACAAGCCCCTCTATGAAAAAAAAACGTCTATATTTTGTGCTTTGAGTGCC
TTACATCGGCAAAACCCTCTTTGGCGAAACAAACGGTTGATGTCATTAACATTTATATCTCGAACAAAATTTACCTGGAAGCG
AGGCCTTATTTAAATTCTAATTTTTTTCACTCTCTCTCCATCACATCGAATCTTTGGACACATGCATGGAGCAGTAAATGTTGA
TAAAAAAATAACTAATTGCACAGTTTGATTGTAAATCACGAGACGAACCTTTCGAACCTAATTAATCCATAATTATACACTAAT
TACCAAATACAAACGAAAGTGCTACAGTTGCAAAGATTCAAAAAAAAAATTGTAATCTAAACATTGCCCGAGTAAGAAAAATT
ACTACTCTAGTACTGTACTCGTACTAGCATTGTCACCGACAGACAGGAGTCCGAAGCAAGCGTTGAACGAGGTAGAGGAGT
GGTTTGACCAAAAAGGACCCCACTGGCAGAGACACCCGACAGGATTTGGCTGCGAGAATGTGAAAACAAGACCGACACGC
AGGTCAAGGTCGTCGTCTCGTTTTTTTTTACCACCTGATTGTCTGCAGCCAACATCATAATTACGGAGTAATTAATTAACGTAC
TACAGTGCTCTCACTCGCTCCTCTGCTCTGTGCTCTGTGCTCTGTTCTGCCTCTGCCTCCCTTGTAGCCGAAGCGGCGAGTGA
CACTGTGCCCAGGGTTAAAAAACCGCTCGGTTTACCGCGGTAAACCACGGTTACCGCCCTAACCGGGCCGGACCGGTTCTG
GTCCGGTCCGGTTAGGTATTCCCCAAATTCAAATTTAAAATTTGAAAAATTGCTGAAAAAATTCTTAAAAATAGTTCATGATT
TATAGAAGCCAATGGTGTAAAAAATTTTCAAATTCGAGTTCGTTTAGTATTTATTTATACGTATAGAAGTTTGAAAAAAAAAA
GAAAAAAAATCCCGGTCCCGGCCCGTTAAAGCCCCCAAATTCAAATTTAAAATTTGAAAAAAAACAAAAAATGCTGAAAAA
ATTCCTAAAAATAGTTCATGATTTATAGAAGCCAATGGTGTAAAAATTTTTCAAATTCGAGTTCGTTTAGTATTTATTTATACGT
ATAGAAGTTTGAAAAAAAAGAAAAAAAATCCCGGTCCCGGCCCGTTAAGGCCCCCAAATTCAAATTTAAAATTTGAAAAAA

ACAAAAAATGCTGAAAAAATTCCTAAAAATAGTTCATGATTTATAGAAGCCAATGGTGTAAAAAATTTTCAAATTCGAGTTCG
TTTAGTATTTATTTATACGTATAGAAGTTTGAAAAAAAAAAGAAAAAAGAAAATTCTAAACTCAAACCGAGCGGTAAACCGC
TCAAACCGGGCGGTTACCGCTTCAAGCCGGCCGGTAAACCGGAGACAAACCGAACCGAAGGAGCGTTTGAATTCAAATTC
AAATTCGACCGGTTACCGGCCGGTTACCGATAAACCGGTCCGATTTACCGTTACCGGACCCCGACGGTTTGGGGTGACCCGT
CGGTAAAAAAAAAAAAAAAAAACCTTACTGTGCCTGCTACTGCCCTCCAGCTCTCACGCCACTTGTGTTGGGTCGGGGTCCC
TCCTCCTCCTCACTGTTGCTTTCTCTGCAGTGCTGGGGTAGCTTCTCCTCCCCCCATCTGGCCATCGCCATCGCCACCACCATT
CCATTCCCATTCTCTTCCCCTTCTTCGCCCGTGCCCATGCCGACGCCTGCTCTGCCCCCACAAGCACAAGCACACACCTTCTG
AGCCACCAACGCCATCGACCA
>SsLRR-RLK196
GCTCCTCGCCAAGATCCATTCAGGGTGTTGTTTTTTTTTTTCATTTCTATGATGTTGTGACATATGGTTTTTAATGTTGTATTGA
CTTGCTCGACATGTTATCCTGCATAAGTAGATTTTTCTATGTTTCACGGAGTTGGAGATGGATATCTAGATGTATTTTTGATGTC
GCGACAACTGTAGCTAGATGTACCAACAAGTTGTTTTTCGTTGCAATGTTTGGTATGTGTTATCCTACGTTGCAGTAGCCTATG
TTTTTGATGGTGCAACAAGCGACTTAGTAACTTGTTTCAAACATTCACTATCATGTTGTAGCATGTTTTCTCTAGTTGTTTTCT
AGGATGAGTTGAGATGTTGAAATAATTGTGCTTTATAGTTGTAGCCATGCATATATACGTCTCTCTTCCTCCTCTACTCTGCCAT
GTTTGGGCGGTGCAACGTGAGGGTGGTGTAAGTTGGTGCGCGGTTGCGCTCCAACAAGCGTGGTTGCTCCGCCATGAGCGG
GCAAGACAACATACCACAGGAATAGAAAGGAGCTGGCACAACATCTGACTATCTGAGGGCTAGCTATTTTGTTTATGGATAG
ACAAGGTGGGAATCTTCTCTCCGGAGTCAAGAGTCATATATTCATACTGTTTACCATAGTAAATGGGCCATGAAATTGTTGCAT
TGCAAATTATGACTGTGCTTAGAAATAGGTTGACTCTATTGCTGGATACATCTAGGCCATTGTCAAAGTGGCGTCCGTGATGC
TGACATGGCATTCGGGCGCATCAGCTATGTCTATCTTGCCATCGTCATTGAACCTCATATTTACCGATGGTAGACATCTTAGAT
CGAATCTAGTAAGAGGGTGTTTGGTTCCTCTTGTTTTTTTAACACCTATCACATCGAATGTTTAGACACATACATGAAGTATTA
AACATAGGCTAATTACGAAACTAATTGCACAGATTAAGATTAATTTACGAGACGAATCTATTAAGCCTAATTAGTCCATTATTA
CTATCCGAACACCTTATGGTCAAACCCATGGTCAACACCTGTTAAAATTTAACAGGACGAACCAAACAAGCCATAAAGCTCA
TCACATACCTATGATTACGGCTCAACTAATATCAACCTAAGGTACTCAGTCCAAGGAGGTTGTCCGGAGGGTTGGTGCCTGGT
CAGTTGATTCGTATGAATTAGGCTATCACAATGTTAATGGCACGTTATATTAGTCGTACTATGGTTACATGCAACCTCAACTAC
AAGACCCTCTTGAGAAAAAAAAAAACCAACGTCTATTTTTGTGCTTTGAGTGCCTTACATTGACAAAACCCTCTTTAGCGAAA
CAAACAGTGCCCAAATTTACAGTTGATGTTATTAACATTTATATCTCGAACAAAATTTACCTGGAAGCGAGCAAGAAAAATTT
ACTAGTCCAGTACTGTACTCGTACTAAGCCTTGTCACCGACAGACACGAGTCCGAAGCAAGCGTTGAACGGAGGTACAGGT
GTGGTTTGACCAAAGAGGACCCCACTGGCAGTGGCAGAGACAGCCGACAGGATTTGGCAGCGAGAATGTGAAAACAAGA
CCGACACGCAGGTCAAAGTCGTCGTCTCGTCCGATGCCGACGACTGGTTTTTTTTTTTACCACCTGATTAGTAAACTCCCGTC
CCCAGCCAACATCATAATTACGGAGTAATTAATTAACGTACTACAGTGCTCTCACTCGCTCCTCTGCTCTGTGCTCTGTGCTCT
GTTCTGCCTTGTAGCCGAAGCGGCGAGTGACACTGTGCCTGCTACTGCCCTCCAGCTCTCACCCGCCCACAGGCCACTTGTG
TTGGGTCTGTTGCTTTCTCTGCAGTGCTGGGGTAGCTTCTCCTCCCCCCATCTGGCCATCGCCATCGCCACCACCATTCCATTC
CCATTCTCTTCCCCTTCTTCGCCCGTGCCCATGCCGACGCCTGCTCTGCCCCCACAAGCACAAGCACACACCTTCTGAGCCA
CCAACC
>SsLRR-RLK342
TTACAAGTTTGAAAGCATCTCAAACTTATAGGATTTTAGGACGCTTTTTGTATATTGGATAAAGAACATGCACATATGCATGAA
AAAAACTATTACATCTTGTTTGGATGTTGGTATTGAGCTTGGAATTAGGGATTTAGAATTGCATATCTTCAATACCATGTGTTTG
GATGCTTTGGAATCCGATCTTCAAATTTGACTCACTACACGTTATTTTCGCGGCACCAAACAACATAGTACCAATACAGAGCA
CACAAGCTCGGGTATTGGCCGGAATCGTCACCTGCCACCTAGGCATGCAAGAGGCGGACCCCACCAACGGGCGAAGGAGG
CGAAGGCATGGAGGGACAGAGCTTGTTGGTGCGTGGAGCGGGCGGATCCAGTGCGTGGAGGAGGAGAGCTTGCCGAAGG
CGAAGAGTAGAGCTCATCACGGCGTGTGGAGCAAGCGGATCTAGGAGTACTTCCCATATGTTGACGCGCCTATGTCGACGAT
GATCTTGAGAAACATATATAGTTTTAATTTATGCTTATTTAAAATTTTCTATAGTAGTATATAATTTGTAATTTAGATATAGATCTG
TAGGGATAATTTAGTTTACGTTTAATGACAATAGAAGAAGTTAATTTAAATACAAATTTAAGGGGTATCCCACGTAATGGAATA
TAATATGATACTCTCTCCATCCTAAATTATAAGTCATTTCAAGAATCTCGTAGAGTCAAAACATCTCAAGTTTGATCAAAATTAT
AGAAAAAAAATTACAAAGATTTATGACATCAAATAGATATAGTATAAAAATATAATTAATAAAGAACCTAATGATACTTAGTTG
ATATTATAAATTTTATTATCTTATCATATAAATTTGGTCAGACTTGAGATATTTTGACTCTCCAAGATTCTTAAAATGACTTATAAT
TAAGGATGGCAATCGGGCGGGTTTAGACCCCAGACCCACAGGTTTTAGACCCGACGGGGGCGGATCTTGGGTTCAGGAACC
CGAACCGGGTTGGGTGCAGGGGCAGATTTTGTTATCCCCTGCGGGTGCCCACGGGGCCTCGAAAGTATGGCCCAGCTACCTT
GTCCACAGTTCCCATCTGACTCGTCTGCTCCGTGCCTTCTCTTTCCCCCGTAGGCCGTAGTCCCATCCGCTCCGCAGAGCCGC
GTCTCGTCCGTGCCTCCACCTTCTCCCTTCTGCGACGGCGCGAGACAGATCCACGGTGGCTTCCACGAGACCGCGACAGCG
CGAGGCAGATCCACGACAGCTCCTCCCTTCTACCTTGGCACTCATCTCCCGCTCGACCATAGAGCCGTGCCTCCTTCTCCCTC
CCCCATCTCTTGATTGGCTTTGTTCTTTTTTTTTCTCCTGGCTGCTCCCCTTCGCACGTCGCACATGGCTCACTTGTGCTCCTA
GGAAGGCGACGAACTGACGCTGGGGAGGCAAGAACCCCTTGCGCAGCAGTGTATTAGGCAGCAACACACAAGGCAGCAG
CGCATCAGACAGTGGCGGATGAATTCGTGAGTTCTTGTCAATTTTAGCTCGGGTTTCGGGACCTGTCGGGTTCGGGGTGGGT
GGGAAATTTCACCCGTGGAGGAGTTCGGGGCCAGGGCGGGTCTGATTGTCGGGTTTCGGGTGCGGGTGCGTGGATGCTCCA
CCCGGCCCCGACCCGCTCTGTTGTCATCCTTACTTATAATTTGGGATGGAGTATACAATAGTAGAGGTGGATAATCGATACACG
CATTGAGTAACTTTATATTATTTTAAAAAATAACAGAGTTAGAAGGTTTCTTAGAAATAGAAGAATAGATGCGATAGCTAATAG
TTTGGAGTCTAATCTAACAATTTAATAATTAGTAAAGTAATGATTATGCAAAACATACTGCTTCCCGTATCGCGTGCTTGCAAC
GTGCCGAGCCGAACTGCCGATGTGCTGAGTCATAGGGGAGCCGAACTGCTTCCCATATCCATATCGCGTGCTTAAAACCCCA
CACCCCTTGA
>SsLRR-RLK312
CACCAGCGGGCGGAGGAGGCGAAGCCTGGGCATGGAGGGAAAGAGCTTGTTGGTGCATGGAGCAGGCGGATCCAGTGCGT
GGAGGAGAGCTTGCCGAAGGCGAAGAGTAGAGCTCATCACGGCATGTGGAGCAAGCGGATCTAGAGTGCTTCCCATATGTT
GACGCGCCTATGTCCACGGTGATCTTGAGAAACATACTCCCTCCGTCCTGAAATATAGTATGTTCTGTCATTTGAGAGGTGTC
CTCTTGAGGGAAAAAAAAATAAAATTTACTTAAATAATGTTGTACAAGAGACAAAGTAATTGAGTGCTTACCATATTTTATATAA
GGAACTTAGATTCCACCTTTTAATCATGCGGAATACGATCAAATTTCAATAGATTAGAAAGTTACGTGAGGGGTACATGCGTC
TATTATCCGCTCTCTTATTTTGTCTGATAATAGCCACAATGCATTGTATTCCAGGACGGAGGGAGTATATAGTTTTAATTTATGCT
TATTTAAAATTTTCTATAGTAGTATATAATTGGTAATTTAGATATAGATCTGTAGGGATAATTTAGTTTACGTTTAATGACAATAG
AAGAAGTTAATTTAAATACAAATTTAAGGGGTATCCCACGTAATGGAATACGATATGATACTCCCTCCGTCCCAAATTATAAGT
CATTCCAAGAATCTCGTAGAGTCAAAGCATCTCAAGTTTGACCAAAATTATAGAAAAAAAATTACAAAGGTTTATGGCATCAA
ATAGATATAGTATGAAAATATAATTAATGAAGAATCTAATGATACTTAGTTGGTATTATAAATGTTATTATATTATCATATAAATTT
GGTCAAACTTGAGATACTTTGACTCTCCAAGATTCTTAAAATGACTTATAATTAAGGATGGCAATCGGGCGGGTTTAGACCTC
AGACCCGCAGGTTTTAGACCCGACGGGGGCGGATCTTAGGTTCAGGGACCCGAACCGGGTAGGGTGCGGGGGTAGGTTTTG
TTATCCCCCGTGGGTGCCCATGGGGCCTCGAAAGTATGGCCCAGCTACCATGTCCGCAGTTCCCATCTGACTCGTCCGCTCCG
TGCCTTCTCTGTCCCCCGAAGGCCGTAGTCTCGTCCACTCCGCAGAGCTGCGTCTCGTCCGTGCCTCCACCTTCTCCCTTCCG
CGATGGCGCGAGGCAGATCCATGGTGGCTTCCACGAGACCGCGACGGCGCGAGGCAGATCCACGACAGCTCCTCCCTCCTA
CCTTGGCGCTCATCTCCTGCTCGGCCGTAGAATCGCGCCTCCTTCTCCCTCCCCCATCTCTTGATTGTCTTTGTTCTTTTTTTCT
CCTGGCTGCTCCCCTTCGCACGTCGCACATGGCTCACTTGTGCTCCCAGGAAGGCGACGAACTGACGCTGGGGAGGCAAGA
ACCCCTTGCGCAGCAGCGTATTGGGCAGCAGCACACAAGGCAGCATCGCATCAGACAGTGGCGGATGAATTCGTGAGTTCT
TGTCAATTTTAGCTTGGGTTTCAGGGACCTGTCGGGTTCGGGGGCGGATGAGAAATTTCACCCGTGGAGGAGTTTGGAGCC

GGGGCTGGTCTGATTGTCGGGTTTCGGGTGCATGGATGCTCCACACACACCTCAATCCGTACTTATAATTTGGGACGGAGATA
GTATATAACAGTAGAGGTGGATAATCGATACACGCATTGAGTAACTTTATATTATTTAAAAAATAACATAGTTAGAAGGTTTC
TTAGAAATAGAAGAATAGATACGATAGCTAATAGTTTGGAGTCTAATCTAACAATTTAATAATTAGTAAAGTAATGATTATGCA
AAACATACATCTGCTTCCCGTATCGCGTGCTTGCAACGTGCCGAGCCGAACTGCCGATGTGCTGAGTCATAGGGGAGCCGAA
CTGCTTCCCATATCCATATCGCGTGCTTAAAACCCCACACCCACTCGCTCCACATCGCCCGCCGCTCCACTCTCCCTCCACAC
CTCCCCCAT
>SsLRR-RLK325
CGTTGGGCGCGGTCTCTACTAGAGTTCTCATCCGATATGCTTTTGTGGGATGTGACCATGAACTGGCGATGTAATTGAACTAC
TGAAGTTATGTTTAAACTATGTTGCTTCCGCTATTCAAACTTGGTTGTAATATTTTATTTCGAACTCCGATGGATGTAAGCTGTA
AGCTACTTGTGAACTGTTGTAACAGATGAGATGTTGTGTTGAATCATATACGATCTTGGCTTGTTGTAAGTTGTTTGAAATCTT
TCATGATTTCACGGACTACTGGGGTTATGGGAGCACAAGTACAGGGATTTGATCACTCCGGTGATTGTTTTTTACTTACGTTC
TTATAATTTAGTCGATTCTGTCACAGCGGACCCCACCAGCGGGCAGAGGAGGCAAAGCCTAGAAGTTTTAATTAGGCTATTC
ACCCCCCTCTAGCCTATTTGGACCTTTCAGCGAAGAAGAACCACGGTGGCTTCAACGAGACCACGATGGCGCGAGGCAGAT
CCACGACAGCTCCTCCCTTCTACCTTGGCGCTCATCTCCCGCTCGACCATAAAGCCGTGCCTCCTTCTCCCTCCCCCATCTCT
TGATTGGCTTTGTTCTTTTTTTCTCCTGGCTGCTCCCCTTCGCACGTCGCACATGGCTCACTTGTGCTCCCAGGAAGGCAACG
AACTGACGCTGGGGAGGCAAGAACCCCTTGCGCAGCAGCGTATTAGGCAGCAACACACAAGGCAGCAGCGCATCAGACAG
TGGCAGATGAATTTGTGAGTTCTTGTCAATTTTAGCTCAGGTTTCAGGGACCTGTCGGGTTCGGGGGCGGGTGGGAAATTTC
ACCCGTGGAGGAGTTCGGGGCCAGGGCGGGTCTGATTGTCGGGTTTCGAGTGCGGGTGCGTTGATGCTCCACCCGGCCCCG
ACCCGCTCCGTTGCCATCTTTACTTATAATTTGGGATGGAAGGAGTATACACCCAATAGTAGAGATGGATAATCGATACACAC
ATTGAGTAACTTTATATTATTTAAAAAAATAACAGAGTTAGAAGGTTTCTTAGAAATGGAAGAATAGATGCGATAGCTAATAGT
TTGGAGTCTAATCTAACAATTTAATAATTAGTAAAGTAATGATTTTTTTGGCAACTAATTAGTAAAGTAATGATTATGCAAAAC
ATACATCTGCTTCCCATATCGCACGCTTGCAACGTGCCAAACTGCCGATGTGCTGAGCCATAGGGGAGCCGGACTGCTTCCC
ATATCCATATCGCGTTAAGGCTACCTGCAGCGGCATCCTGCAAACGCCCTGCAATATATGATAGCGGACCACGCGAGCCGCAG
CGGAGTACTGGATGCTGTCTGCTATCCATGCGTCAACGGTAAATATAGCGTTGTACCGTAGTGTTGTAGCGTACTGCCCGCCC
ACTCTACTGCTTGCATGTGCGTGGAGAGAGAAAAAAAAAGGGAAGAAGAAAAAAAAAAAGAGTATGGCATGTAGGCCCCGCG
TGCTAGTAGTTGATATAGAGTATGAAATATAGAATATGATGGGTGCGGAAGTACTGGATATAGAGGAGAGAATATAGATGACC
AGGATGCACTATTCCTTTTAGTGGATGAATTTAGGGTGTGTTTAGTTTCCCATTTCACCTAAAAAATTTTGAGTTTTGGCCCAT
CATTTTGCAAAATGGAACTAAACACCATGCAAAATTTTTGGCAAAAAGGTATCCATTCTTCATTTTGGAAAAAAAAAGCCAAA
AAGCTCTCACCCACCCCATGAAAAATTTAGCATTTTGGATAGAACTAAACACACCCATAAAAATTTTAGCATTTTGTATTTCAT
TATTCTAAAGTAATTGGTATTTTATATTTTGTATTCCATGAGAAACTAGACACAGCCTCAGAGTATAACGAGTGCAGAAAGCCT
AAAACCCCACACCCCCTTGATGACAGTTAAGCGGAAGGCCCCCACTCCTTCCACATCGCCCGCCGCTCCACTCTCCCTCCAG
TCCTCCACACCTCCC
>SsLRR-RLK334
GAGGAGAGCTTGCCGAAGGCGAAGAGTAGAGCTCATCACGACGTGTGGAGCAAGCGGATCTAGGAGTACTTCCCATATGTT
GACGCGCCTATGTCGACGATGATCTTGAGAAACATATATAGTTTTAATTTATGCTTATTTAAAATTTTCTATAGTAGAATATAATT
GGTAATTTAGATATAGATATGTAGGGATAATTTAGTTTACATTAAATGACAATAGAAGAAGTTAATTTAAATACAAATTTAAGGG
GTATCCCACGTAATGGAATATAATATGATACTCCCTCCATCCCAAATTATAAGTCATTTCAAGAATCTCGTAGAGTCAAAACAT
CTCAAGTTTGATCAAAATTATAGAAAAAAAAAATTACAAAGATTTATGACATCAAATAGATATAGTATGAAAATATAATTAATGA
AGAACCTAATGATACTTAGTTGGTATTATAAATGTTATTATCTTATCATATACATTTGGTTAGACTTGAGATACTTTGACTCTCCA
AGATTCTTAAAATGACTTATAATTAAGGATGACAATCAGGCGGGTTTAGACCCCAGACCCGCAGGTTTTAGACCCGACGGGG
GCGAGTCTTGGGTTCAGGAACCCGAACCGGGTTGGGTGTGGGGGCAGGTTTTGTTATCCCCTGTGGGTGCCCACGGGGCCT
CGAAAGTATGACCCAGCTACCTTGTCCGCAGTTCCCATCTGGCTCGTCTGCTCCGTGCCTTCTCTTTCCCCCGTAGGCCGTAG
TCCCGTCCGCTCCGCATAGCCGCGTCTCGTCCGTGCCTCCACCTTCTCCCTTCTGCGACGGCGCGAGGCAGATCCACGGTGG
CTTCCACGAGACCGCGACAACGCGAGGCAGATCCACGACAGCTCCTCCCTTCTACCTTGGCGCTCATCTCCCGCTCGACCAT
AGAGCCGTGCCTCCTTCTCCCTCCCCCATCTCTTGATTGGCTTTGTTCTTTTTTTTCTCCTGGCTGCTCCCCTTCGCACGTCGC
ACATGGCTCACTTGTGCTCCTAGTAAGGCGACGAACTGAGGCTGGGGAGACAAAAACCCCTTGCGCAGCAGTGTATTAGAT
AGCAGCACACAAGATAGCAGCGCATCAGACAGCGCCGGATGAATTCGTGAGTTCTTGTCAATTTTAGCTCGGGTTTCAAGA
AACCTGCCGGGTTTTAGGGACCCGTCGGGTTTGGGGGCAGGTGGGAAATTTCACCCGTGGAGGAGTTCGGGGCCGGGGCA
GGTCTGATTGTCGGGTTTCGGGCCAGCCCCGACCCACCCCGTTGCCATCCTTACTTATAATTTGGGATGGAGGGAGTATATAA
TACTAGAGATAGATAATTGATACACGCATTGAGTAACTTTATATTATTTTAAAAAATAACAGAGTTAGAAGGTTTCTTAGAAAT
AGAAGAATAGATGCGAGCATCTCCAAGAGTTTGCCAAATCAATTTGGCATCCATTTATTTTTGGTAAAACAGAAAAAATTATG
CTCTAATAATTTGGCATCCAACTTGGCAAAATTTAGTAACTTGACAAATCTCCCCCCTCCACGCGTATATTTGCGTGCTAAGCG
CGAGCGCGCATCCGGCTTCACGTGCAAATTTAAGCGGGGCTCTTCGTTTCTTAGCGGGGCTCTTCGTTTCTTAACGGGGCTC
TTCATTCGCTTAGCGGGCCTCTTCTTATTTACCAAGTCCAAATGGCAAACTCTTGGAGAGAGTCTATTTTTTCACTTACCATA
TATTTTAGCTAATTGCCAAATCACGATATTTGTCAAGTGAATTTTGGCAAACTCTCGGAGATGCTCTAGTTTGGAGTCTAATCT
AACAATTTAATAATTAGTAAAGTAATGATTATGCAAAACATACATCTGCTTCCCGTATCGCGTGCTTGCAACGTGCCGAGCCG
AACTGCCGAACTGCCGATGTGCTGAGTCATAGGGGAGCCGAACTGCTTCCCATATCCATATCGCGTGCTTAAAACCCCACAC
CCCCTTGAT
>SsLRR-RLK67
CAAGGAAGGGAAGAAGAGGCCGGGGCAGTGACAAACGGCTAACAGATCATTTTTGTTTGAAGGCGGGGGAAAGCTGCAGT
TTGCATTTGCAGCCAGGAAGAGCCTTTTACACCTGGGCTGGACACCAACGCCGGCCGGCTGGCTGGCTTCTCTGTCACTCTC
TCTCTCTCTATCCTCTGGCTAGAGGCCGCTTCGGTTCTCGTGCACTACCGAACCAACCCGGGCTAGAACTTGTTTTCCGGGGT
GCTGCTACGGCCACCCCTAGAGTCTGCAAGGGATAGCGAGCAGGGTGAGGGCCTGAGGAGCCTTCCTACCATGCTTCCCGC
ATTTCTCAAAACTGATCTCTTGACTTTGTGGTGACTCCCTGTGACCGGCTAAGGCCCCACAGGGTTGAGCACACCAAAGGAT
GTTTCTTAGCAAAACTGGCCACTGGGTTTTTTTTATCTGGCCATAACAATATAAAGATCAATTTATTCACATTTTCTCTTTGTAC
AATCATGCTAATGGACATTTAATTACGGTAAATGACATGCTACGGAGAATGCGCCAGCTAGGATTTTTAATTTCGGTATTGCAA
AAATTTTGGCCACAACCGAAATTGCGGAATTTCGCAAAATTCGGCCGAAATTTCACCAAATTTTTTAGAAAACTAGCACATA
AAGTATGATTTTTCTGAAAAAATAGATTTAAACACATATTAGTATGAATTATTACTAACATCTATTGAATAGAAGAATATGCAAT
ATAAACAATAGAAAATGTGAGTCTAGAATTATATAGCACATGCATTAGTGTATTACAAAATTTCAGATTTTTCTTTCGGCCACC
ACAAAAAATCACGAAATTCAGCCAAAATTAATTTTGAACCCTGGTGCCAGCCATTTTGCCATGGTTTGAATTTTCCTTACTTT
TGACGGCCATATTATCGGTTACTTGAATCGTGTCATCAACCTACTATAAATACAAGCATATCCAAATGCTTGGTCCACTATGAA
CGTCACTTGTCGCTACTTTCATAAGTCCACTTGTAACGTTTTCGATGCCGACAAACGGCTATTTTGACCCAACAAGGATGGGC
GACGGTTAATTACCAACGACTACATTGACCCAACAAGGATGAAATGCGATTAATTACCCAGTGACAGTTGTGTCGCGCCGAT
TTTACTTGAGAAAAATGTATCCCTCCCGAATCCCAATTGCTCGGCACACCATTCATTAGTGATACATCTACCCACTCAGCGTG
AGCCATCTTGGTAGAATTAGCCCAATAGATGCGTTTGATGACATGTTTCTTACTTAGGGCTTTTTTTGAGTTAGTCAGGGCCTA
TCTGGCTGGGTGTACTCCCTCCGTATCGGATTTATAAGTCGTTTTGGATGAGGTTTGAGTCAAACATTGAGAATATAAATCATT
AATAACTTTTAAGTTGTTGAGTTTGCAAATATAAAAATTATATGAATAGATTTATCTTGAAAAATACTTTCATAAAAATATACAT
ATATCACTTTTTCATAAATATTTTTTATAAAAATAAGAGGTCAAAGTTGTGTTTTGGAGACCGTGTCGCTGTCCTAAACGACTT
CTAAACCGATACGGAGCGAGTACCTCCGTTGGTTACCCACCGACAGCTGTGTCGTGCTAATTTACTTGTGAAAAATGTATCCC
TCCTGAATCCCAATTGCTCGGCACACCATTTCATTAGTGATACATCTACCCACTCAGCGTGAGCCATCTAGGTAGAATTAGCCT

GCGTTTGATGACATGTTTCTTACTTAGGGTCTTTTTTTTTTTTGAGTTAGTCAGGGCCTATCTGGCTGGGTGTACCTCCGTTGG
GTTCCCTACCAAAATCACCCTGTGATTTACTTCTCTTCCATCTATAAGAGGAAACCATGGAGAGCGAGCCTTCGAATTCTGTG
ACGGTGTGACCACAAGTCCAAAACATAACTCCCTCCTCAAAACCTCCCCACGCTCTCCACTCGCCCACCACCGCCACCGCC
AGCCA
>SsLRR-RLK90
TTACGGGTTTGCCTACGAAATATCGTCAGCGTTGTAGTATGGCAACTAAATCCGGAGAGTGGTGTTTTCACACTAGCTACTCA
GATTTAGGTGAAGTTCATCCGCGATCTTTGCATCTCTAGTTAAAGCGGGTTTAGGAACTTGTGCGTCTGCAGGAAAGCCGTA
GAAACGCATCCGGCTGCATCACATCACATGAACCGATGGCTGCAAAGTAGTGGTGGCACTTGCCGTTTGGCACTCCGGCCG
GCCGGCCGATGGCGTGCGGAGGCATGCACGCCCCCGCCGGCCACGCCGAGCACGCACCGCGCCTGCCCGCCCGCCCGCCA
GCTTCTCGGCGTCCGTTCCCGGGCCAGGCGGTGCGCGCGTTCTTCCGGCCCCTCGGATCCTCACGCGCTACACGCTAATCCC
TAGCCTGGCTCGCAGCGTACCGCGCGCGACGCCGTCCAAAATCCGGCGGCCGGGAGGTCCTCCTCGCCCCTCACGTTGCCT
GCCAGCCGATCGATGGCGTGGCACGCCCCGTTCCAAAACTTTGCAACTCGGCCCCGCCCGCAGAGGGTCACCCGCGTCGCG
CGCCGCCGAAGCCTGGCTGCAGGCTGCACGCGCCGTGCCCGGCCCAACCGCTCGCTTTGTCCCAGGCTCCCAGCCCTCGCC
GCGCCATGCTCGACCACGCGCGCAGTGCAGCAGCCGCCAGCCAAAAACGGGCGGCGCCCACCGATTTCCAGCCCGCCGCC
AGCACGGCTCTCCCGGAAGAAGCTGAAACGCTTCAGTTGCACACCGGCTGGACGGCAGAGAGTAGCGCGCTGGCAGCCCA
GTGCAGTTGCCGTCGGAAGGAAGGAAGAGGCCGGGGCAGGGCAGTGACAGATTGGCTTTGTTTGAAGGCGGGCAAAAGC
AGCAGTTTGCATTTGCCACCGGGAAGAAGCCAATGCCAAGTCATTAAAGTTTCACTCCAGTAGCCTCGTCCATCGGGACCAT
GCCACGCGAGAGAGCCATTCACATCTGGGCTGGACACCAACGGCCGGCCGGCTGGCTCTGGCTGACTGGCTTCACTGTCAC
ACATTCCACTGGCTAGACGCCGCTTCGGTTCTCGTGCACTGCCGAACCGCCCCAGGTGCTGCAGCTACGGCTACCCCTGGA
GTGATAGAGAGCAGCAGGGTGAGGGGCCTTCCTACGAGGCCACCATGCCTCCGGCATTTCTCAAAACTGATCTCTTGTGGG
GACTCCATGTGTGATCGGCTAAGGCCACACAGGATTAAGCACACTAAAGGATGTTCCTTGGTAAAACCAGCCATTGGGTTTA
TTTATCTAGCCGTAACAATATAAGGATCAATTTACTCACATTTTCTCTTCGTTAAAACATGGTAAATGGACTTTAAGTATGGCA
AATGACATGCTACAGAGAATGTGCCGGCCATTTTGCCGCCATTTGAAATTTTCTTACCTTTGGGTTTCAGTAGCCAGATCATC
GATTACTTGAATCGTGCTGTCAACCTATTATAAATACGGGCATATCCAAATGTTTGGTCCATTGCTAACGTCACTTGTCGCTAG
TTTCATAAGTCCACCTGTAACATTTTCGATGCCGACAAACGGCTATTTTGACCCAACAAGGATGGGAGACGGTTAATTACCA
AAGACTATATTAACCCAACAAGAATGGAAGGTGATTAATTACCCAGCGACAAAATGTATCCCTCCCGAATTCCAATTGCTCGG
CACACCGCACCATTTCCATTAGTGATACATCTACCCACTCAGCGTGAACCACCTCGGTAGAATTACCTCGATAGATGCCTTTG
GGTGACATGTTTCTTACTCAGGGCCTATCTAGGTGTACCTCCATTGGGTTCCCCTAGCAAAATCACCCTCTGCACTTCTCTTCC
CTGTATAAGAGTAAACCATGGAGCCTTCCAATTCTGTGACCAAAACGCAACTCCCTCCTCAAAACCGGGCACTCGGCAACT
CCACGCTCTCTCCACTCGCCCACCACCGCCACCGCCAGC
>SsLRR-RLK109
AGATGAAACTCATTTCTTCTCTCTCTTCTTAAATACACTGCCATATCATTAAAAGTGCATATGTGGCAACCCATTTAATGCAAA
TGAAACTCACATGAAACTTCCACTAAGACTGGCCTAAGGGATAGCGAGCAGGGTGAGGGCCTGAGGGGCCTTTCTACCATG
CTTCCCGCATTTCTCAAAACTGATCTCTCGGCTCTCGTGGCCTTGTGGTACTGGTGACCCCCTGTGACCGGCTAAGGCCACA
CAGGATTGAGCACACCAAAGGATGTTTCTTAGCAAAAACCGGTTACTGGGTTTATTTATCTGGCCATAACAATATAGAGATCA
ATTTATTCACATTTTCTCTTTGTAAAATCATGCTGATGGTAATGGACCTTTAATTATGGTAAATGACATGCTACGGAGAATGTGC
CAGCTAGGGTTTTTACTTTCGGTATTGCAAAAATTTTGGCCACCACCGAAATTACCGAATGTCGCGAAATTTGGCCGAAATTT
CACTGGATTTTTTTAGAAAACTAGCACATAAAGTATGATTTTTCTGAAAAAAATAGATCTAAACACATATTAGTATGAATTATT
ACTAACATCTATTGAATAGAAGAATATGCAATATAAACAATACAAAATGTGAGTCTAGAATTATATAGCACATGCATTAGTGTAT
TACAAAATTTCGGATCTTTCTTTCGGCCACCACAGAAATTTGCGAAATTCGGCCGAAATTAATTTTGAACCTTGGCGCCAACC
ATTTTGCTGTGGTTTGAATTTTCCTTACTTTCGACGGCCATATCATCGGTTACTTGAATCGTGTCATCAACCTACTATAAATACA
GGCATATCCAAATGCTTGGTCCACTGCGAACGTCACTTGTCGCTACTTTCATAAGTGCACTGGTAACGTTTTCGATGCCGACA
AGGATGGGAGACGGTTAATTACCAACGACTACATTGACCAAACAAGGATGGAATGTGATTAATTACCCAGCGACAGCTGTGT
CACGCCGATTTACTTGTGAAAAATGTATCCCTCCTGAATCCCAATTGCTCAGCACACCATTTCATTAGTGATACATCTACCCAC
TCAGCGTGAGCCATCTTGATAGAATTAGCTCAATAGATGCGTTTGATGACATGTTTCTTATTCAGGGCCTTTTTTTTTAGTTAG
TCAGGGCCTATCTGGCTGGGTGTACCTCCGTTGGGTTCCCCTACCAAAATCACCCTATGATTTACTTCTCTTCCATCTATAAGA
GGAAACCATGGAGAGCGAGCCTTCGAATTCTGTGACGGTGTAACCAAAAAGTCCAATATAAGGATCAATTTACTCACATTTT
CTCTTTGTTAAAACATGGTAAATGGATTTTAAGTATGGCAAATGACATGCTACAAAGAATGTGCCAGCCATTTTGCCGCGATT
TGAAATTTTCTTACCTTTGGGTTTTGGCAGCCAGATCATCGATTACTTGAATCGTGCCGTCAACCTACTATAAATACGGGCATA
TCCAAATGCTTGGTCCATTGCTAACGTCACTTGTCACTACTTTCATAAGTCCGCCTGTAACGTTTTCGATGCCGACAAACGGC
TATTTTGACCCAACAAGGATGGGAGACGGTTAATTACCAAAGACTATATTAACCCAACAAGAATGGATGGTGATTAATTACCC
AGCGACAAAATGTATCCCTCCCAAATTTCAATTGCTCGGCACACCGCACCATTTCCATTAGTGATACATCTACCCACTCAGCG
TGAACCACCTCGGTAGAATTACCTCGATAGATGCCTTTGGGTGACATGTTTCTTACTCAAGGCCTATCTAGGTGTACCTCCATT
GGGTTCCCCTAGCAAAATCACCCTCTGCACTTCTCTTCCCTGTATAAGAGTAAACCATGGAGCCTTCCAATTCTGTGACCAAA
ACGCAACTCCCTCCTCAAAACCGGGCACTCGGCCACTCCACGCTCTCTCCCCTCGCCCACCACCGCCACCGCCAGCCATGG
AT
>SsLRR-RLK110
TCGCCGCGCCATGCTCGACCATGCGCACGCGGTCCGTGGACCAAAAACAGGCGGTGGTGCCCACCGCCAGCACCGCTCTCC
CGGAAGAAACGCTTCAGTTGCACACCGGCTGGACGCCAGAGAGTAGCGCGCTGCCAGCCCAGTGCAGTGCGTGAGGCGTT
GCCGTCGGAACCAAGGAAGGGAGGAAGAGGCCGGGGCAGTGACAAACGGCTAACAGATCATTTTTGTTTGAAGGCGGGGG
AAAGCAGCAGTTTGCATTTGCAGCCAGGAAGAAGCCAATGCCATGCCACTGCCACGCTAGAGAGCCTTTTATACCTGGGCT
GGACACCAACGCCGGCCGACTGGCTGGCTTCTCTGTCACTCTCTCTCTCTATCCTTTGGCTAGAGGCCGCTTCGGTTCTCGT
GCACTGCCGAACCAACCCAGGCTGAACTCGTTTCCTGGGTGCTGCTACGGCTACCCCTAGAGTCTGCAAGGGATAGCGAGC
AGGGTGAGGGCCTGAGGAGCAGCCTTCCTACCATGCTTCCCGCATTTCTCAAAACTGATCTCTTGACTCTTGTGGCCTTGTG
GTGACTCCTGTGACCGGCTAAGGCCACACAGGGTTGAGCATTTCTTAGAAAAACTGGCCACTGGGTTTATTTATCTGGACAT
AACAATATAAAGATCAATTTATTCACATTTTCTCTTTGTAAAACCATGCTAATGGTAATGGACCTTTAATTACGGTAAATGACA
AACTACGAAGAAAGCGCCAGCCAAGATTTCTAATTTTGGTACTGCAAAAATTTCGGCCACCACCGAAATTACCGAATTTCAC
GAAATTCGGCCGATATTTCACCGATTTTTTTAAAAAAAAACTAGCACATAAAGAATTCTTTTTCTGAAAAAATAAATCTAAA
CACATATTAGTATGATTTATGACTCCACATCTATTAAATAGAAGAATATGAAATATAAACAATAGAAAATGTGAGTCTAGAATTA
TATAGCACATGCATTAGTGTATTACAAAATTTCAGATCTTTCTTTCGGCCACCACAGAAATTCGCGAAATTCGGCCGAAATTA
ATTTTGAACCCTGGTGTCAGGCATTTTGCCGTGGTTTGAATTTTCCTTACTTTCGACGGCCATATCATCAGTTACTTGAATCGT
GTCATCAACCTACTATAAATACAGGCATATCCAAATGCTTGGTCCACTGCGAACGTCACTTGTCGCTACTTTCATAAGTGCAC
TGGTAACGTTTTCGATGCCGACAAGGATGGGAGACGGTTAATTACCAACGACTACATTGACCAAACAAGGATGGAATGTGAT
TAATTAGGTCAGTCTCAGTGGAGAGTTTCATTTCATTGTTTCCAAGACTGCCATGTCATAGAGAATGAAATGAAACTCGGATA
AAACACTCACTCTCAATGGAAAGTTTCACTCCATAGTTTCATAGACATTTAATTCTATGACTCATAGAGAGTTGGTAACTGTG
CCAAGAGAGTTTCATCCCCATGAAACTCAATTCTTCTCTCTCTTCATTAAAACGTTGACATGTCATCAAAAATGCATATGTGG
CAGGCTATTAAATGCAAATGAAACTCTAGTGAAACTCACACTGAGACTGGCCTTACCCAGCAACAGCTGTGTCACGCCAATT
TACTTGTGAAAAAATGTATCCCTCCCGAATCCCAATTGCTCGGCACACCATTTCATTAGTGATACATCTACCCACTCAGCGTG
AGCCATCTAGGTAGAATTAGCTCAATAGATGCGTTTGATGACATGTTTCTTACTTAGGGCCTTTTTTTAGTTAGTCAGGGCCTA
TCTGGCTGGGTGTACCTCCGTTGGGTTCCCTACCAAAATCACCCTGTGATTTACTTCTCTTCCATCTATAAGAGGAAACCATG

GAGAGCGAGCCTTCGAATTCTGTGACGGCGTGACCAAAAGTCCAAAACATAACTCCCTCCTCAAAACCTCCCCACGCTCTC
CACTCGCCCACCACAGCCAGCCA
>SsLRR-RLK132
CCGGCAGAGGGTCACCCGCCGCGTCGCGCGCCGCCGAAGCCTGGCTGCAGCATGCACGCGCCGTGCCCGGCCCAACCGCT
CGGTTTGTCCAAGTCCTCGCCGCGCCATGCTCGACCATGCGCACGCGGTCCGTGGAGCGCAGCCGCCAGCCAAAAACAGGC
GGTGCCCACCGGTTTCCAGCCCACTGCCAGCACCGCTCTCCCGGAAGAAACGCTTCAGTTGCACACCGGCTGGACGCCAG
AGAGTAGCGCGCTGCCAGCCCAGTGCAGTGCGTGAGGCGTTGCCGTCGGAACGAAGGAGGGGAGGAAGAGACCGGGGCA
GTGACAAACGGCTAACAGATCATTTTTGTTTGAAGGCGGGGAAAGCAGCAGTTTGCATTTGCTGCCGAGAAGAAGCCGTTT
ATTTTTAACCAGCCAGTAATATTTTTCTCTCACAATAAATTAGTCAACAGGACTTTCAGTCGCTTATTTTCAGCCAAACGAACA
GACTTGATTTTCTCCTTCTCTCCCGTAGCCTCGTCCATGGGGACCATGCCACTGCCACGCTAGAGAGCCTTGCACCTGGGCT
GGACACCAACGCCGGCCGGCTGGCTGGCTCTGGCTGACCGGCTTCTCTGTCTCTCTCTATCCTCTAGCTAGAGGCCGCTTCG
GTTCTCGTGCACTGCCGAACCAACCCGGGCTAGAACTCGTTTCCTGGGTGCTGCTACGGCCACCCCTAGAGTCTGCTAGGG
ATAGCGAGCAGGGTGAGGGCCTGAGGAGCCTTCCTACCATGCTTCACGCATTTCTCAAAACTGATCTCTTGACTCTTGTGGC
CTTGTGGTGACTCCCTGTGACCGGCTAAGGCCACACAGGGTTGAGCACACCAAAGGATGTTTCTTAGCAAAAACCGGTTAC
TGGGTTTATTTATCTGGCTATAATAATATAAAGATCAATATATTCACATTTTCTCTTTGTAAAACCATGCTAATGGTAATGGACCT
TTAATTACGGTAAATGACATACTACGAAGAAAGCGCCAGCCAGGATTTCTAATTTTGGTATTGCAAAAATTTCGGCCACCACC
GAAATTACCGAATTTCACGAAATTCGGCCGATATTTCACCTATTTTTTTAAAAAAACTAGCACATAAAGTATTCTTTTTCTGAA
AAAAAATAAATCTAAACACATATTAGTATGATTTATGACTCCACATCTATTAAATAGAAGAATATGCAATATAAACAATAGAAA
ATGTGAGTTTAGAATTATATAGCACATGCATTAGTGTATTACAAAATTTCAGATCTTTCTTTCGGCCACCACAGAAATTCGTGA
AATTCGGCCGAAATTAATTTTCAACCCTGGTGCCAGGCATTTTGCCGTGGTTTGAATTTTCCTTACTTTCGACGGCCATATCAT
CGGTTACTTGAATCGTGTCATCAACCTACTATAAATACAAGCATATCCAAATGCTTGGTCCACTGCGAACATCACTTGTCGCTA
CTTTCATAAGTGCACTGGTAACGTTTTCGATGCCGACAAACGGCTATTTTGACCCAACAAGGATGGGAGACGGTTAATTATC
AACGACTACATTGACCAAACAAGGATGGAATGCGATTAATTACCCAGCGACAACTGTGTCGCGCCGATTTACTTGTGAAAAA
TGTATCCCTCCCAAATCCCAATTGCTCGGCACACCATTTCATTAGTGATACATCTACCCACTCAGCGTGAGCCATCTAGGTAGA
ATTAGCCCAATAGATGCGTTTGATGACATGTTTCTTACTTAGGGTCTTTTTTTTTTAGTTAGTCAGGGCCTATCTGGCTGGGTG
TACCTCCGTTGGGTTCCCTACCAAAATCACCCTATGATTTACTTCTCTTCCATCTATAAGAGGAAACCATGGAGAGCGAGCCT
TCGAATTCTGTGACGGTGTGACCAAAAAGTCCAAAACATAACTCCCTCCTCAAAACCTCCCCACGCTCTCCACTCGCCCACC
ACCGCCAGCGCCAGCCATGGA
>SsLRR-RLK120
TTGCTAGTCATATATCAGTCCAAGCAAGGTCCAAGTGATGACCAAGAACAACCCAATATATATGCAGGCTTGTGGAGCGTTGC
TTCTTCATTCTCACCCCATCTTTTGACCGCACCACCATCTAGCTCATCATCACACAACGATGACCGCTCAGCAGCTAGTGCCA
AACGACTTTTACTCACACCCCACTCCAGAGTACACTGTTCATCTTCCAGTCAAGCCGATAAATGTATCATGCTGCATGCTCTT
GCTCGTTTCATCCTCCACCTTTGCTTCCATGCACTTCCAGTCATTAGTGGCACACGTCCAACCTGTGGTCGCATGGATGGAGT
TTGGAACACAGGGTCCATGGAATTTTGTTCCTACAGTAAATGTGACCCTAATTTTGATGGAGATTTTGCCGGATAGCGAAGTT
TCACTGTCCAATAGCCCGGATTGTGACAGTAGAGGACAAAAACAAACATAAGTGTGTGCAATGGCTAAGGTCATGTTTGTAT
CGGTTTCGGATTCTTAAAAACAAGTTTTTGCCTCTAGTTTCTAAAATCTGGATTCTGAGTAAAAACTGGTGGGGTGTTTGGAT
CCCCCAGTTTTAATAATCTGACTTCATTTATTGCTCACTAAGTCATAAGAAACTGACAAAAGTTAGGTATCAACAACTTCTTG
GTTTTTAAGAATCTGGCAAAAACTAACAGCAAAAACTGGTCTGTTTGGATCCCACCCAGTTTCCAAAAGCCAGATTCTTAAA
AACTGGCAAAAAACTGGAGGAATCCAAACCGGACTTAAAACACCCGTTGATAGGTATATATTCCCCTCTGTCCTAAAACATA
ATATATTTAATTATTCAAAATATGTTCTAAATTATAAGAAACTCTAGGTGAACTAACCCTCAACTCAGTTTAGAAATGCAGCATT
TAATTCATAACTACTATTAATTGATGCCTATATATCAGCAATCCAATATATAGTAATTTATCTCTTAATGATTAAAGAAGGCCACA
TGCTTCATTTGCTTTACCCCGTAATATCTATATTTATATCTATATCTATGTCTGATATTAAAGAGTGAATGGTTTCTTTAGACTTTT
CTTACTTTCCAAAATGCCCATGTGTCCTCATGTCTTGTTTGTGACCCGAACTTACGAATCATGTTTATACGAATTAGAACAACT
CACGTTTAGTGATGATATAGAATGTCCAACATGTATTGAGTTCCATTGCAGCACTTGAACAAGTTCGCTTGTATATATCCTAAA
ATTCTTTGGATACCAGAGTACATTAATTTATTCCCTTTGAACTTGCGCTCACTTTGTATATTCCTACATATTGCACCAGATTATTA
AGCAAAAGCACACCTTGATCACACTTTTGTCGGGTAGCATGCTTGTTTTTTTTGTCGGACATGAGGGCGAAGCTTCTCTTGT
CAAAAGCTCGGGTTGCGAGGGTAGAGGAGGAAAAAAACATGAGTGTTAGATATGGCTAAAACACTTGACCGCCTCGCCCTC
TTTATACAATATGTTTAAGCATTATAAAAATTTATCCCAAATTTTAAACAAATTCTAGGTAAGTTAACCTTCAATTTTTTTTTTAA
AAAGACAACATTCGCGTTGGCTCATAAATATGGTAATCTATACAATGTTTGTGGATGGAGTGAGTATCCTTCCAAAACATGTG
CACTTTGTATTACTCCTACATGTGGCACCAGACTAAGCAAAGCACATGTTGATTACATTTTGTAGAGTATCATATACTTCCTTTT
CAGCAGTGGCTTGCTCTCCCCACTAATTAATTAACTCTATATAATCCAAACCCTTGCTGCCTACTTACTCCTAATCCGGTCTATC
ATTAACACTTTGCCTAGTAGCTGCATCTGCAGACTGAGGGCGGGCCCCCTACTGAGGACGTCGAGTACTGCCACCTTAAAAC
CAGTCAAACATCCACACGCTGACACCGAGGAACCAACGAACCACGCGTCATCCTTCGCTCCCCGACGCCCGGCGCC
>SsLRR-RLK141
CCCAGATTCTGACTTAGGTAGTAATATTGTCTTCCAACACCTTCGTGAGACTCACAGACTTCAACTAGGCCACTGAATGGTTT
GGGCTTCTCGAACTCTTTGAACTTTTGAATTATGGCTTTATAATGGACTTGTGATGGTACTTGTGGTTTGTATGGACTAAAAGA
TTTTGAATTATGGCATGTAATTGTGGTTTGTATGGACTAAAGGACTTTGAATCATGGCATGTAATTGTGGTTCGTACGGATTAT
GGCTACTTGTGATGGTACTTGTGAATTATGACTATATAATGGCTCTTGTGGTTAGATTTGAAATTGAATTTGAATTATATATTATA
TCATGTGGTCAGATTTGAATTTAAATTATATTTTTTTCCTGAAAAACCCACTGTAGGGGCGGTTGAATCATCAACCCCCCTTAC
AAATGCCATTTGTAAGGGCGGTTGGATCACGAACCCCTCTTACAAATGGCATTTGTAAGGGCGGTTGGATCACGAACCCCTC
TTACAAATTCCATTTATAAGAGGGGTTCGTGATCCAACCGCCCTTACAAATGTCATTTGTAAGGGCGGTTTGCCCAACCGCCC
TTACAGTGGGCTGATTTTTAAAAGCTCCCTGGTAAGAGGGGTTGGGTTACCCGCCCTTAAAAACCCCTGAGACCCGCCCTTA
AAAACCCTGGCTGTAGTAGTGGTTTTTAAGAATCTGACAAAAACTAACAGCAAAAACTGGCCTATTTGGATCCCACCCAGTT
TCCAAAAGCCAAATTCTTAAAAACTGGCAAAAAACTGGAGGAATCCAAACCGGACTTAAAACACCTGTTGGTAGGTATATAT
TCCCCTCTGTCTAAAACATAATATATTTAATTATTCAAAATATGTTCTAAATTATAAGAAACTCTAGATGAACTAACCCTCAACT
CAGTTTAGAAATGCAGCATTTAATTCATAACTACTATTAATTGATGCCTATATATCAGCAATCCAACATATTGTAATTTATCTCCT
AATGATTAAAGAAGGCCACATGCTTCATTTGCTTTACCTCGTAATATCTATATCTATATCTATATCTATGTCTGATATTAAAGAGT
GAATGGTTTCTTTAGACTTTTTCTTACTTTCCAAAATGCCCATGCGTCCTAATGTCTTGTTTATACGAATTAGAACAACTCACG
TTTAGCGATGATACAGAATGTCCAACATGTATTGAGTTCCATTGCAGCACTTAACAAGTTCGCTTGTATATATCCTAAAATTCT
TTGGATACCAGAGTACATTAATTTATTCCCTCTGAACTTGCGCTCACTTTGTATATTCCTACATATTGCACCAGATTATTAAGCA
AAAGCACACCTTGATCACACTTTTGTCGGGTAGCATGCTTTTTTTTTTGTCGGACATGAGGGCGAAGCTTCTCTTGTCAATAAC
TCGCGGGTTGCGAGGGTAGAGGAGGAAATAAAACATGAGTGTTAGATATGGCTAAAACACTTGACCGCCTCGCCCTCTTTAT
ACAATATGTTTAAGCATTATAAAAATTTATCCCAAATTTTAAACAAATTCTAGGTAAGTTAACCTTCAATTTTTTTTTAAAAAAA
GACAATATTCGCGTTGGCTCATAAATATGGTAATCTGTACAATGTTTCTGGATGGAGTGAGTATCCTTCCAAAACATGTGCACT
TTGTATTACTCCTACATGTGGCACCAGACTAAGCAAAGCACATGTTGATTACATTTTGTAGAGTATCATATACTTCCTTTTCAG
CAGTGGCTTGCTCTCCCCACTAATTAACCCTATACTATAATCCAAACCCTTGCTGCCTACTTACTCCTAATCCGGTCTATCATTA
ACACTTTGCCTAGTAGCTGCATCTGCAGACTGAGGGCGGGCCCCCTACTGAGGACGTCGAGTACTGCCACCTTAAAACCAG
TCAAACATCCACACACTGACACCGAGGAACCAACGAACCACGCGTCATCCTTCGCTCCCCGACGCCCGGCGCCA
>SsLRR-RLK99
GGATAATCAGCCCTGCTCTTTTCCATATATATGTGGCGAACCGTACGCTAATATGCACTATTTTTTACAACGATTGGCCTGACGT

GGTTCTAAACCTAATAATGCTTCTGGCCAATCAGGAGAGGCGAGAAACACATGATGATGAACAAGGGATTTTTTTTTCTAAA
AAACAATGGACTTGTCATTGTCTCCTTTTTCATGTCCACCACGGTGAGTACATTTTATTTTTATCTAATGGCAAGTGCATGGCA
CCTTTTGAGTGAGCATCTATGTATCACCACCTTCCCGTCACAACTCCTTATCCACCATGGATTCCTCCACATGGACCCACCAC
CACCATCTCCACAAAAATTATATTGTTTTCATATATTCATTCCCTTTTTTGAGCGAGAAATTCAGAAAAGAAACTAAATAAATT
TGACTTTTCCTATATATATTCATGCTGTTCTTTCTAGGACAAATTTTTTCAGAAGTTTTATTCGGTGCTATCCAAACGGCGCTTT
TGGAAAGAGGAAAACATTGGAGCACACGTGTGCATTCCATTCCTGTGGCATTTCATTTTTTGAAGGAATTGTTCTCTTGATGT
GTTAAATAGCAGTAAAGAATTTGTGCCGAATGCCTACAGCTGCTCTAGCAAGTAGCACTAATGGTGAAGTGCCTAAATTCTAT
TTGAAAGTTCTTAATTATTCCAGGTTGAGACTGAATAATGTTGACTGATGGAGTCTTCAGCCAAATCCAAGCTCAAAGCTTGC
ATAACAATTATCAGCAGGCGTACCGCTACGCTTTTTACTCCATTAATCGAATATATAATATAGTACAATAAACTAAAGTTCTAAT
TCGGTTACTAAACTACTAAGCACTGCTGGCTGAACAGGAAAAAGATGCAGATTGGCGTATGCACACATTGCAAACAAACTA
CTGGCAAATAAAGCATGGCATCATTCAGGCCACTACTGAAGAAGCATCAGCAGCTGAGCGTGCAGTAGCGCTCACTTGTTGT
TCTTTTTTTAATTGCTGCTACTTTCTGGTGCAGCCTAATACACTACGCTCCTCAAATAACTCACTGCTTGTCAGGGCTATAAAG
AATTGATTTTTAGGAGATGGAAGCGGCTTTACATTTGTCAGCATGCTGCTGATCCTCCTCAAGTGTGAGCAATGATTGGGATT
GGCATCCACTAGAGGCAGGCTTTGTCCTTGTTCCCCATGGAGATTGCAGGATTAGCAGCTGCACTGTGTTTGCTGCTTGCAA
GTTGATCTGCAGTTCAGCTGGACTGTGCTTTATGCCTTCAGTAGTGTTTTCAGTTAACACTTAACAGCAGGGGGGGCGTGAGT
CTGTGACTGATTGACTGTGAGTGATGCCTCTCCACCACAACACAGTGCTCTCCATTTCAGGTTTCAGCAGGAAATTAATATGC
TGGATGCACAGATAATATACTAGCATGATGGTGTCGGTAATCTATCATTGACCACCGCTTCATGTCAACTCTTAACAGAAACA
CTGATTTACTATGTCAACTCTGAACAGAAAGCTGACGACTGACATGGCTTGCCGGCCAGTACATGTCAATCTCTTGACAGCA
ATTGGTGAACTAATATGGTTTACTGATCATATCTAAAAAAAGCATGGTTCACTGATCACTTGCCTGGAAGTGTGAAACGCATT
TGGGTTCAGTTAGTACTCGGCCGGGCCGGGTTGGACTGGGTCGAGCCGGGCGGGCCTGTGCCAGCTGTGGGCTGAAGAGT
GGGCAGGCCCATATGAGGCCCCTGTCTCTCTCAGCTCTTGATTCTGAACGGCTAGTGGACATGCTGGGCTTGACTGGGCCAG
ACCTGACCAACTGACTGACTGCATGTCAATTTTTGACCAGCAGCACTGCACAAGGAAAATTCCTGGACAACACAAGAGACA
GAGAGAGCTAAAGAGACCACAAGAAGAGGCTGGAGAATGTTGCTCACTGGAGTGGACTGCACTGAGGTACCAATCAGTTT
TGTTGCGATTGGCAA
>SsLRR-RLK121
TGGATTCCTCCACATGGACCCACCACCACCATCTCCACAAAAATTATATTGTTTTCATATATTCATTCCCTTTTTTGAGCGAGA
AATTCAGAAAAGAAACTAAATAAATTTGACTTTTCCTATATATATTCATGCTGTTCTTTCTAGGACAAATTTTTTCAGAAGTTTT
ATTTGGTGCTATCCAAACGGCGCTTTTGGAAAGAGGAAAACATTGGAGCACACGTGTGCATTCCATTCCTGTGGCATTTCATT
TTTTGAAGGAATTGTTCTCTTGATGTGTTAAATAGCAGTAAAGAATTTGTGCCGAATGCCTACAGCTGCTCTAGCAAGTAGCA
CTAATGGTGAAGTGCCTAAATTCTATTTGAAAGTTCTTAATTATTCCAGGTTGAGACTGAATAATGTTGACTGATGGAGTCTTC
AGCCAAATCCAAGCTCAAAGCTTGCATAACAATTATCAGCAGGCGTACCACTACGCTTTTTACTCCATTAATCGAATATATAAT
ATAGTACAATAAACTAAAGTTCTAATTCGGTTACTAAACTACTAAGCACTGCTGGCTAAACAGGAAAAAGATGCAGATTGGC
GTATGCACACATTGCAAACAAACTACTGGCAAATAAAGCATGGCATCATTCAGGCCACTGCTGAAGAAGCATCAGCAGCTG
AGCGTGCAGTAGCGCTCACTTGTTGTTCTTTTTTTTAATTGCTGCTACTTTCTGGTGCAGCCTAATACACTACGCTCCTCAAAT
AACTCACTGCTTGTGAGGGCTATAAAGAATTGATTTTTAGGAGATGGAAGCGGCTTTACATTTGTCAGCATGCTGCTGATCCT
CCTCAAGTGTGAGCAATGATTGGGATTGGCATCCACTAGAGGCAGGCTTTGTCCTTGTTCCCCATGGAGATTGCAGGATTAG
CAGCTGCACTGTGTTTGCTGCTTGCAAGTTGATCTGCAGTTCAGCTGGACTGTGCTTTATGCCTTCAGTAGTGTTTTCAGTTA
ACACTTAACAGCAGGGGGGGCGTGAGTCTGTGACTGATTGACTGTGAGTGATGCCTCTCCACCACAGCACAGTGCTCTCCATT
TCAGGTTTCAGCAGGAAATTAATATGCTGGATGCACAGATAATATACTAGCATGATGGTGTCGGTAATCTATCATTGACCACCG
CTTCATGTCAACTCTTAACAGAAACACTGATTTACTATGTCAACTCTGAACAGAAAGCTGACGACTGACATGGCTTGCCGGC
CGGTACATGTCAATCTCTTGACAGCAATTGGTGAACTAATATGGTTTACTGATCATATCTAAAAAAAGCATGGTTCACTGATCA
CTTGCCTGGAAGTGTGAAACGCATTTGGGTTCAGTTAGTACTCGGCCAGGCCGGGTTGGACTGGGTCGAGCCGGGCGGGCC
TGTGCCAGCTGTGGGCTGAAGAGTGGGCAGGCCCATATGAGGCCCCTGTCTCTCTCAGCTCTTGATTCTGAACGGCTAGTGG
ACATGCTGGGCTTGACTGGGCCAGACCTGACCAACTGACTGACTGCATGTCAATTTTTGATCAGCAGCACTGCACAAGGAA
AATTCCTGGACAACACAAGAGACAGAGAGAGCTAGAGAGACCACAAGAAGAGGCTGGAGAATGTTGCTCACTGGAGTGG
ACTGCACTGAGGTACCAATCAGTTTTGTTGTGATTGGCAATGAGCAGAGCAGCTGGGAGCTGGGGCAGGGCATATATCCCCC
CAACAGCAAAAGCCATGCATCTCTCCCTCCTCCGTCTCCTTTATATCATCACCATCACCATTCAGCAATTCCAATTCCCTCCAC
AGATTCCCACTCCACTTATATACTCCCTCCCTTCCTGATAGCTCCTCCTCACCATTCCAATCCCATTGCAACAAAGCTCCTTCC
AACAACAGCGGCACAAGCTTGCAGGCACACGTACGATCGAGCCAGCACAAGAAAAGCTTCTTCGTTCTTCTATCTCCTACC
TGCTGGTCAGCTGGTG
>SsLRR-RLK142
ATTCCTCCACATGGACCCACCACCACCATCTCCACAAAAATTATATTGTTTTCATATATTCATTCCCTTTTTTGAGCGAGAAATT
CAGAAAAGAAACTAAATAAATTTGACTTTTCCTATATATATTCATGCTGTTCTTTCTAGGACAAATTTTTTCAGAAGTTTTATTC
GGTGCTATCCAAACGGCGCTTTTGGAAAGAGGAAAACATTGGAGCACACGTGTGCATTCCATTCCTGTGGCATTTCATTTTTT
GAAGGAATTGTTCTCTTGATGTGTTAAATAGCAGTAAAGAATTTGTGCCGAATGCCTACAGCTGCTCTAGCAAGTAGCACTA
ATGGTGAAGTGCCTAAATTCTATTTGAAAGTTCTTAATTATTCCAGGTTGAGACTGAATAATGTTGACTGATGGAGTCTTCAG
CCAAATCCAAGCTCAAAGCTTGCATAACAATTATCAGCAGGCGTACCGCTATGCTTTTTACTCCATTAATCGAATATATAATATA
GTACAATAAACTAAAGTTCTAATTCGGTTACTAAACTACTAAGCACTGCTGGCTGAACAGGAAAAAGATGCAGATTGGCGTA
TGCACACATTGCAAACAAACTACTGGCAAATAAAGCATGGCATCATTCAGGCCACTACTGAAGAAGCATCAGCAGCTGAGC
GTGCAGTAGCGCTCACTTGTTGTTCTTTTTTTAATTGCTGCTACTTTCTGGTGCAGCCTAATACACTACGCTCCTCAAATAACT
CACTGCTTGTCAGGGCTATAAAGAATTGATTTTTAGGAGATGGAAGCGGCTTTACATTTGTCAGCATGCTGCTGATCCTCCTC
AAGTGTGAGCAATGATTGGGATTGGCATCCACTAGAGGCAGGCTTTGTCCTTGTTCCCCATGGAGATTGCAGGATTAGCAGC
TGCACTGTGTTTGCTGCTTGCAAGTTGATCTGCAGTTCAGCTGGACTGTGCTTTATGCCTTCAGTAGTGTTTTCAGTTAACAC
TTAACAGCAGGGGGGGCGTGAGTCTGTGACTGATTGACTGTGAGTGATGCCTCTCCACCACAACACAGTGCTCTCCATTTCAG
GTTTCAGCAGGAAATTAATATGCTGGATGCACAGATAATATACTAGCATGATGGTGTCGGTAATCTATCATTGACCACCGCTTC
ATGTCAACTCTTAACAGAAACACTGATTTACTATGTCAACTCTGAACAGAAAGCTGACGACTGACATGGCTTGCCGGCCAGT
ACATGTCAATCTCTTGACAGCAATTGGTGAACTAATATGGTTTACTGATCATATCTAAAAAAAGCATGGTTCACTGATCACTTG
CCTGGAAGTGTGAAACGCATTTGGGTTCAGTTAGTACTCGGCCGGGCCGGGCTGGACTGAGTCGAGCCGGGCAGGCCTGTG
TCAGCTGTGGGCTGAAGAGTGGGCAGGCCCATATGAGGCCCCTGTCTCTCTCAGCTCTTGATTCTGAACGGCTAGTGGACAT
GCTGGGCTTGACTGGGCCAGACCTGACCAACTGACTGACTGCATGTCAATTTTTGACCAGCAGCACTGCACAAGGAAAATT
CCTGGACAACACAAGAGACAGAGAGAGCTAAAGAGACCACAAGAAGAGGCTGGAGAATGTTGCTCACTGGAGTGGACTG
CACTGAGGTACCAATCAGTTTTGTTTCGATTGGCAATGAGCAGAGCAGCTGGGAGCTGGGGCAGGGCATATATCCCCACAAC
AGCAAAAGCCATGCATCTCTCCCTCCTCTGTCTCCTTTATATCATCAACATCACCATTCAGCAATTCCAATTCCCTCCACAGAT
TCCCACTCCACTTATATACTCCCCTCCCTTCCTGATAGCTCCTCCTCACCATTCCAATCCCATTTGCAACAAAGCTCCTTTCAA
CAACAGCGGCACAAGCTTGCAGGCACACGTACGATCGAGCCAGCACAAGAAAAGCTTCTTCGTTCTTCTATCTCCTACCTG
GTGGTCAGCTGGTGCT
>SsLRR-RLK158
GTTCGTAGCCTGCTCCTGTGAATGGAACTTAGCGGTCGCTCCTACCACTTCACGGGGCTCATGTTCAGCACAGAAAAGAGCA
GCACATCACAATCCTCGATCCAATCAGACATGTAGATACAAATCAGTATTCTCTGGTCGTGTACAAAAGTACTACCACTGTAG
AGTAGACAGTACGTATTCTAACAAGTTCAATCACTTATCTGCTTGTCTGAATTCTTTAGGTGCCATGTGTGCACCAAGAGGA

AGAGCTTATCTGACAGATCGTTCTGCAGCGGAGTGCTCATGTGGCCAAGAGAAGTTTCGATGGCCTTGGCCTACATACTGGG
TCCGGTCCAGCAGCTTTCGGTTTGTTCGTTTGCTACGTCGAGGTCTTGTTTAGATTGAAAAGAGAGTGAAATTTGGCACGGT
AGCATTTTCGTTTGTATTTGATAATTATTGTTCAATCATGGACTAACTAGGCTCAAAAGATTCGTCTCGTAATTTATAATCAAAC
TGTGTAATTAGTTATTTTTTTATCTACATTTATTGTTCTATGCATGTGTCCCTAAATTCAATGTGACGAAGAGAGTGAAAAAACT
TATAATCTAAACATGGCCCAAGTAGGCACTCACGGGGCTGAATCAGCAGATCTTTGACTCCAATATCAGCTGGAATTTTTGTT
CCTACGTTCTTGTCCTTATCTGTCAGATCCGATGATTGATCCAAATTTTCTTGCAACCTCTGGATATAGGCCCTGTTCGGCAGG
CTGTTTTTTTGGCTTATAAGCCGGCTGTGGCTGATTTGCTGTGAGAGAAAAACACTGTTCCACGGCTGATAAGCCGTGCTGAT
AAGCTCAGCCGAACAGGGTGATAGTTTCTGGCATGTCGTTAACACCCGATCGACACATGGACTTGATGTTCGAACCCTGAAG
TTCCTCGGCAGAATTGAAACGAGACGTCTATCCGACTTGCTTCGTACTGCAGTCGATCGGATCATGCGGTCGGACGTATAGC
ACCACTCAGAATTAAGTGGAGTTAACTCTAATCTAAATCTTCTACATGTATAAGCATGGTTCCTTAGTCCTTGTGAATCTTACT
ATCGTGGGCATCAGGGATTCAGGGCATGCATGTTTGGATGTTCAGATTTTGTTGACATGAGCTACGGGAGAAAACCACACAC
GACACCCAAATCGCTAGATGCTTCCATGGATCGGCATCCAAGTTGGGGGTAACACAGGTGGTCTGAAACGAACAACGAACC
GCCTCAGGCTCGCGGAATATGATGGCGAGCACGAATATTTATGTATGGCACGTAAAAAACTGCTCCTGCAAACCAAAGATGC
GATCCTGCGGTCGCATGGATGTTGTCGGGATTCGGCTCTGGAAGATTGGATGAGGCAGACGACACGACCTACGAGACAACC
AAGATCCTCCGATCCGTCGTGGCGTCGGTGTACCTTCTTCATCCAGAATAAGTGGACGCGTCGAAAATATCACACAGCTAGG
CAGAGGCAGGATTGAAAAGTACCTCTCGGCACGCCCACACCGACGTCGCCGCACCCCAGGCGTGCGCTGCGTACCGTACCG
CAATAGTACTGTACGCCGGCGAAAAATGAGGGGCGAGACCGAGAGAAAACCAGCGGAATCGAATGAATCTCCGCGTTCCG
GTGGTACTGGTACCCGCCGCGCGCGCGATCCCGCTTCCGCGGGCGTGCGGGTACCGGCGCACGGCCGAGGACCGAGGCCA
AGCACGGGCGTCGGCGTCGCTTTCTGGTGCGGGGTCAAGTGGCGGGCGCCGTCCACAGAATGCAAGAGATTCCGGCGCGC
TCGTCGCGTGCGTAGGAGTACGTTTTTTTCCCCCTCCGTCTCGTCCAATCCGCCTTGTTGCCTTGCGCCCCCGCCCGCCTCTG
GGGCGGGACAAGAGGCTCCTGCGCCACGCTGCGCACCACTACTTGATCCACTGGTCGCCCCGCCCGCTCCCTCCCTCCACC
TCTATAAATCTACCCGTCCTCGCCGCC

>SsLRR-RLK295

CTGCACCTAAAATTAATATATGTGCTATTTTTGGAATCATGTCACCAAAGAAATGCCTCAATAGGGAGGAAACCTGTATATTTT
TTTTCAAAAAAAAAATCAAACCGGACAGAAGAGCTGTCATTATATATATTAATAGGAAGTAAAGAAGAAGAGGAAGTAATTAA
CCTGCTGTGTGGTTGCATGATATGATTGATGACTCCAGATGATTTTGTGGCATGTGTCGTTCTAACAGTCCAATATCTCACTCT
CAGATAGGGCATATAGGAACCTGTTGGAATTATGACTAGTTGACTATGCTGCCAAAACTATATTAGTTCAGGAAACTCTTATTT
GTGGGAATTTTAGTAAAATGAAATGTAAAGAAATAGTAAATTTGATAGGTGAGGTGCAAACTCTTGCCAAATCTGTACTCTCT
GAGAAATTTTGAGTTATTCATGAAAAGTGAATACGTACTTTTCATTATTTTTAGTGAAATGATGGTTTACTTTCTTGATTTAGCC
TTCTAAGTACACCTACAATTTAGTATTTATTTGACATGATAAGTAAGAATCAATCTCTAGATACTGAATGAGGATTCCAGCATG
CATTATATTTGAAAATCACGGTCATACCACAAAAAAAAACTGCAGACAAGTTGTCACGTTTTATGCAGCAATGATAGAGTATA
TGATACTCCGGAGAATTAGTCAGTGGAGGTAGAACCCAAAAACATTTATTTAGCAAGATCAAAAGCTACCACCGATAATTATT
CTTTACCGTTTATCATTTTAGAATTCAGTCATGGGACAAAACATGTTAGGTCAATTTCTTCTTTTCTTGGTCATTCCGCCAAGA
ACTGATACCAAAATTGGATCAACTCCTCCCCCAATAACACTCTCTCTTTTACACAGGTCCCTCATAACGAGTCGCTTCTAGGT
TTCACTAGTTATCCAGCTAAGCACCATGATTTTGGGCCATTTTAGATGTTAGACACTGATGGTTCACTAAAATAGTAAGTGCCA
CCAAAGTATGCTCCCACTGAAATTGCTTTGACCATCTTCTTTTTATCCTTGTGTGTGGATTCCATCATCTGATCCCATTATACAT
CTCTGGTGAGTGCTCTGCCTTTTCACCCCCGGTTAACTTGGTGGAGGTGAATGTGTGGTGTGGCTTCCATGAGGCAATCTATT
GCATTTCCTGGTGTTTGTGGTGAAATGAAGGCAACAGAACAGGGTAGAGGAAGTAGAAGGTGGTAGCTGGTAGGTCATCGT
CTCTCAACATGTTTGCATAGGAAACCAACTTGGGGCCTTTGGGGTGTCCAAATCAGCTACCAATTTGTACACATAGTTGTGGC
CACCTGTCATGTGGGGCTGCCGTAGGCGCATGTGTCCAGCGCTGGTGGCTGAGTCAAGCTTGCTGTCCTGAATCTGAGGAGT
GCATGGTGACCACGTCTAGCGCATGGTGGCTGAGTCCAGCCGGAGGTTTAGTTAGTGAATGGTTAGCAGGGTTAGCAGTGA
ATGGCTAGCAGTGAGTGAAGGAGCGGTTATCAGTGAGTGAAAGAGCTTAGTCATTGCAATAGCTAGCCGTGAGGCTATTTAA
GCCAATTGGCATCTCTGGATGGGGAAGCAATGAAGAACAATTAGTCTTAATCTCATCTCCTTCAACAACAGGGCGCTCAGAG
GTAAAACCTTGCTCCAACCCTATGGATCGAGGCTATGAGCTGCATAGCCTAGCCTCGATCCAGCTACCCTAGGGCCCAACGC
CCATGACACCACCTATTTTTGAGGCTTACATTAGCCTAGTCCAAAAAAGATGAATAGAAGTGTTGAAGTTTTTTTTGTCACT
TCAACGCATCTAATACAGTATCACCAAGAAATTGGATGGTTAATGCATTATAACCCTACTTTATGTCTTGTTTTCCATTTTCAGG
TGCTCTGAATCTGTCAAGACATTCAGGACAGTACAAGAACCCCATGTGATCCATTTCTTCAGAGAATCTGCCAACACCATCT
GCTTGC

>SsLRR-RLK286

CTCAACAGGGAGGAAACCTGTATATTTTTTTTCAAAAAAAAAAAAAATCAAACCGGGCAGAAGAGCTGTCATTATATATATT
AATAGGAAGTAAAGAAGAAGAGGAAGTAATTAACCTGCTGTGTGGTTGCATGATATGAACTGATTTGATGACTCCAGATGAT
TTTCTGGCATGTGTCGTTCTAACAGTCCAATATCTCACTCTCAGATAGGGCATATAGGAACCTGTTGGAATTATGACTAGTTGA
CTATGCTGCCAAAACTATATTAGTTCAGGAAACTCTTATTTGTGGGAATTTTAGTAAAATGAAATGTAAAGAAATAGTAAATTT
GATAGGTGAGGTGCAAACTCTTCCCAAATCTGTACTCTCTGAGAAATTTTGAGTTATTCATGAAAAGTGAATACGTACTTTTC
ATTATTTTTAGTGAAATGATGGTTTACTTTCTTGGTTTAGCCTTCTAAGTATACCTACAATTTAGTATTTATTTGACATGATAAGT
AAGAATCAATCTCTAGATACTGAATGAGGATTCCAGCATGCATTATATTTGAAAACCACGGTCATACCACAAAAAAAGCTGC
AGACAAGTTGTCACGTTTTATGCAGCAATGATAGAGTACATGATACTCTGGAGAATTAGTCAGTGGAGGTAGAACCCAAAAA
TATTTCTTTAGCAAGATCAAAAGCTACCACCGATAATTATTCTTTACCGTTTATCATTTTAGAATTCAGTCATGGGACAAAACA
TGTTAGGTCAATTTCTTCTTTTCTTGGCCATTCTGCCAAGAACTGATACCAAAATTGGATCAACTCCTTCCCCAATAACACTCT
CTCTTTTACACAGATCCCTCATAACGTGTCGCTTCTAGGTTTCACTAGTTATCCAGCTAAGCACCATGATTTTGGGCCATTTTA
GATGTTAGACACTGATGGTTCACTAAAATAGTAAGTGCCACTAGAGTATGCTCCCACTGAAATTGCTTTGACCATCTTCTTTTT
ATCCTTGTGTGTGGATTCCATCATCTGATCCCATTATACATCTCTGGTGAGTGCTCTGCCTTTTCGCCCCCGGTTAACTTGGTG
GAGGTGAATGTGTGATGTGTGGTGTGTGGTGTGGTGTGGCTTCCATGAGGCAATCTATTGCATTTCCTGGTGTTTGTGGTGAA
ATGAAGGCAACAGAACAGGGTAGAGGAAGTGGAAGGTGGTAGCTGGCAGGTCATCGTCTCTCAACATGTTTGCATAGGAAA
CCAACTTGGGGCCTTTGGGGTGTCCAAATCAGCTACCAATTTGTACACATAGTTGTGGCCACCTGTCATGTGGGGCTGCCGT
AGGCGCATGTGTCCAGCGCTGGTGGCTGAGTCAAGCTTGCTGTCCTGAATCTGAGGAGTGCATGGTGACCACGTCCAGCGC
ATGGTGGCTGAGTCCAGCCGGAGGTTTAGTTAGTGAATGGTTAGCAGGGTTAGCAGTGAATGGCTAGCAGTGAGTGAAGGG
CGGTTAGCAGTGAGTGAAAGAGCTTAGTCATTGCAATAGCTAGCCGTGAGGCTATTTAAGCCAATTGGCATCTCTGATGGGG
GAAGCAATGAAGAACAATTAGTCTTAATCTCATCCCCTTCAACAACAGGGCGCTAAGAGGTAAAAACAACAGGGCGCTGAG
AGGTAAAACCTTGCCCCAACCCTATGGATCGAGGCTATGAGCCGCATAGCCTAGACTCGATCCAGCAACCCTAGGGCCCAAC
GCCCATGGCACCACCTATGCAGTTTTTGAGGCTTACATTAGCCTAGTCCAAAAAAGATGAATAGAAGTGTTGAAGTTTTTTTT
TTGTCACTTCAACGCATCTAATACAGTATCACCAAGAAATTGGATGGTTAATGCATTATAACCCTACTTTATGTCTTGTTTTCCA
TTTTCAGGTGCTCTGAATCTGTCAAGACATTCAGGACAGTACAAGAACCCCATGTGATCCATTTCTTCAGAGAATCTGCCAA
CACCATCTGCTT

>SsLRR-RLK287

GCCTCAACAGGGAGGAAACCTGTATATTTTTTTTCAAAAAAAAAAATCAAACCGGGCAGAAGAGCTGTCATTATATATATTAAT
AGGAAGTAAAGAAGAAGAGGAAGTAATTAACCTGCTGTGTGGTTGCATGATATGAACTGATTTGATGACTCCAGATGATTTT
CTGGCATGTGTCGTTCTAACAGTCCAATATCTCACTCTCAGATAGGGCATATAGGAACCTGTTGGAATTATGACTAGTTGACTA
TGCTGCCAAAACTATATTAGTTCAGGAAACTCTTATTTGTGGGAATTTTAGTAAAATGAAATGTAAAGAAATAGTAAATTTGAT
AGGTGAGGTGCAAACTCTTCCCAAATCTGTACTCTCTGAGAAATTTTGAGTTATTCATGAAAAGTGAATACGTACTTTTCATT

ATTTTTAGTGAAATGATGGTTTACTTTCTTGATTTAGCCTTCTAAGTACACCTACAATTTAGTATTTATTTGACATGATAAGTAA
GAATCAATCTCTAGATACTGAATGAGGATTCCAGCATGCATTATATTTGAAAACCACGGTCATACCACAAAAAAAGCTGCAG
ACAAGTTGTCACGTTTTATGCAGCAATGATAGAGTACATGATACTCTGGAGAATTAGTCAGTGGAGGTAGAACCCAAAAATA
TTTCTTTAGCAAGATCAAAAGCTACCACCGATAATTATTCTTTACCGTTTATCATTTTAGAATTCAGTCATGGGACAAAACATG
TTAGGTCAATTTCTTCTTTTCTTGGCCATTCTGCCAAGAACTGATACCAAAATTGGATCAACTCCTTCCCCAATAACACTCTCT
CTTTTACACAGATCCCTCATAACGTGTCGCTTCTAGGTTTCACTAGTTATCCAGCTAAGCACCATGATTTTGGGCCATTTTAGA
TGTTAGACACTGATGGTTCACTAAAATAGTAAGTGCCACCAAAGTATGCTCCCACTGAAATTGCTTTGACCATCTTCTTTTTAT
CCTTGTGTGTGGATTCCATCATCTGATCCCATTATACATCTCTGGTGAGTGCTCTGCCTTTTCGCCCCCGGTTAACTTGGTGGA
GGTGAATGTGTGATGTGTGGTGTGTGTGGTGTGGCTTCCATGAGGCAATCTATTGCATTTCCTGGTGTTTGTGGTGAAATGAA
GGCAACAGAACAGGGTAGAGGAAGTGGAAGGTGGTAGCTGGCAGGTCATCGTCTCTCAACATGTTTGCATAGGAAACCAA
CTTGGGGCCTTTGGGGTGTCCAAATCAGCTACCAATTTGTACACATAGTTGTGGCCACCTGTCATGTGGGGCTGCCGTAGGC
GCATGTGTCCAGCGCTGGTGGCTGAGTCAAGCTTGCTGTCCTGAATCTGAGGAGTGCATGGTGACCACGTCCAGCGCATGG
TGGCTGAGTCCAGCCGGAGGTTTAGTTAGTGAATGGTTAGCAGGGTTAGCAGTGAATGGCTAGCAGTGAGTGAAGGGGCGG
TTAGCAGTGAGTGAAAGAGCTTAGTCATTGCAATAGCTAGCCGTGAGGCTATTTAAGCCAATTAGCATCTCTAGATGGGGGA
AGCAATGAAGAATAATTAGTCTTAATCTCATCCCCTTCAACAACAGGGCGCTAAGAGGTAAAAACAACAGGGCGCTGAGAG
GTAAAACCTTGCCCCAACCCTACGGATCGAGGCTATGAGCCGCATAGCCTAGACTCGATCCAGCAACCCTAGGGCCCAACGC
CCATGGCACCACCTATGCAGTTTTTGAGGCTTACATTAGCCTAGTCCAAAAAAGATGAATAGAAGTGTTGAAGTTTTTTTTTG
TCACTTCAACGCATCTAATACAGTATCACCAAGAAATTGGATGGTTAATGCATTATAACCCTACTTTATGTCTTGTTTTCCATTT
TCAGGTGCTCTGAATCTGTCAAGACATTCAGGACAGTACAAGAACCCCATGTGATCCATTTCTTCAGAGAATCTGCCAACAC
CATCTGCTTGCCA
>SsLRR-RLK303
TCTATTTATTTCCTTTTTTCCTTTGCGGGCTTTTAAATTAGTTGAAATAATCTGATCTGATTGAACAAATATACTGTACATGATCA
TTGAATGAAGATAATAGCTAATGTGTCTGCACCTAAAATTAATATATGTGCTATTTTTGGAATCATGTCACCAAAGAAATGCCT
CAATAGGGAGGAAACCTGTATATTTTTTTCAAAAAAAAAATCAAACCGGGCAGAAGAGCTGTCATTATATATATTAATAGGA
AGTAAAGAAGAAGAGGAAGTAATTAACCTGCTGTGTGGTTGCATGATATGAACTGATTTGATGACTCCAGATGATTTTCTGG
CATGTGTCGTTCTAACAGTCCAATATCTCACTCTCAGATAGGGCATATACGAACCTGTTGGAATTATGACTAGTTGACTATGCT
GCCAAAACTATATTAGTTCAGGAAACTCTTATTTGTGGGAATTTTAGTAAAATGAAATGTAAAGGAATAGTAAATTTGATAGG
TGAGGTGCAAACTCTTCCCAAATCTGTACTCTCTGAGAAATTTTGAGTTATTCATGAAAAGTGAATACGTACTTTTCATTATTT
TTAGTGAAATGATGGTTTACTTACTTGATTTAGCCTTCTAAGTACACCTACAATTTAGTATTTATTTGACATGATAAGTAAGAAT
CAATCTCTAGATACTGGATGAGGATTCCAGCATGCATTATATTTGAAAACCACGGTCATACCACAAAAAAAGCTGCAGACAA
GTTGTCACGTTTTATGCAGCAATGATAGAGTACATGATACTCTGGAGAATTAGTCAGTGGAGGTAGAACCCAAAAACATTTAT
TTAGCAAGATCAAAAGCTACCACCGATAATTATTCTTTACCGTTTATCATTTTAGAATTCAGTCATGGGACAAAACATGTTAGG
TCAATTTCTTCTTTTCTTGGCCATTCTGCCGAGAACTGATACCAAAATTGGATCAACTCCTCCCCCAATAACACTCTCTCTTTT
ACACAGATCCCTCATAACGTGTCGCTTCTAGGTTTCACTAGTTATCCAGCTAAGCACCATGATTTTGGGCCATTTTAGATGTTA
GACACTGATGGTTCACTAAAATAGTAAGTGCCACCAAAGTATGCTCCCACTGAAATTGCTTTGACCATCTTCTTTTTATCCTT
GTGCGTGGATTCCATCATCTGATCCCATTATACATCTCTGGTGAGTGCTCTGCCTTTTCGCCCCCAGTTAACTTGGTGGAGGTG
AATGTGCGGTGTGTGGTGTGGTGTGGCTTCCATGAGGCAATCTATTGCATTTCCTGGTGTTTGTGGTGAAATGAAGGCAACA
GAACAGGGTAGAGGAAGTAGAAGGTGGTAGCTGGCAGGTCATCGTCTCTCAACATGTTTGCATAGGAAACCAACTTGGGGC
CTTTGGGGTGTCCAAATCAGCTACCAATTTGTACACATAGTTGTGGCCACCTGTCATGTGGGGCTGCCGTAGGCGCATGTGTC
CAGCGCTGGTGGCTGAGTCAAGCTTGCTGTCCTGAATCTGAGGAGTGCATGGTGACCACGTCCAGCGCATGGTGGCTGAGT
CCAGCCGGAGGTTTAGTTAGTGAATGGTTAGCAGGGTTAGCAGTGAATGGCTAGCAGTGAGTGAAGGAGCGGTTAGCAGTG
AGTGAAAGAGCTTAGTCATTGCAATATCTAGCCGTGAGGCTATTTAAGCCAATTGGCATCTCTGGATGGGGGAAGCAATGAA
GAACAATTAGTCTTAATCTCATCCCCTTCAACAACAGGGCGATGAGAGGTAAAACCTTGCCCCATCCTTATGGATCGAGGCTA
TGAGCCGCATAGCCTAGACTCGATCCAGCTACCCTAGGGCCCAACGCCCATGGCACCACCTATGCAGTTTTTGAGGCTTACG
TTAGCCTAGTCCAAAAAAGATGAATAGAAGTGTTGAAGCTTTTTTTTGGTCACTTCAACGCATCTAATACAGTATCACCAAGA
AATTGGA
>SsLRR-RLK401
CCAGGGGGAGGAGAAATCATTATCAGAGGAAGCCAGAAAAGTTAGGGTTTAGGAAGTTCTCTCCCGCAGAGAATTAGAAGT
AGCTACTCCATTGTTCTTCAAGTCTTATAGGTTTGATTAGATAGAATTAGAGAAGTAGAGCATTCAAGCTCTGGATTTCGGATC
TTCATCAATAAAGATTGGTATTATTTCATATCATTCTCTACTACTTTATTCTAATTGCATTATGTCTCTGTTTACTATGTTCTTAGT
TTACTCTAGTTCTATAATTGATATAGTTATGATTGATAATGAGTTTATGTATGAGTTTGCAAAGCGCTTAGCTCTTACCTCGAGG
GAGTTAAGTGGTAGATCATATGTGGGCGTGGTGCTTAGATATTATTTACCTGCAAATGTATCCTATTGGCCGGGTTGTGTGGTA
GTTCGCGATAGTGACAGCTTCGTTGATTCTTATATAGTCCACCCTCCGTTGATAGGACAGGCAGAATTTGTATTGCGGAGTAA
GTCTTACTATGTTCTGATTTACTTTAGCAATGTTCCTTATACATAAATAAAGAGTCTTTTATGCTATATATGATCTTGTAGAATGT
TTAGAGTAGATTGTGACTTAGTAGATAGTAGATACTTAGAATCCATTCTCTAGCTAATCCGACGTCACCTTATATCAATATGGAG
TAGTCTATTTCTAATCGCTGTGTTATTTACCCATGAGCTTATAATTCATTATCTTTATTTATGGCTTACCCCCTGCCAAAGCAAGT
GACTGTGTGACGAGTTTCTCATTAGTAATCATGTTTAGCAAAGTTGATCTCTAGTCTATGTCATTCTACACCTGCTTTCACCGT
TCTCTTAAGCAAAATATAAATAACGATACCCGGAATACTTTTCCTGGTGAAATGCTACAATGAGGTATTTTATCTGTGCGTTTG
CGGATAGAATAGATTATCTTCTAAAGAGCCCTTTACATTTATAAATACCTATGTACACTCTGGCACCATGCTGGGGATGACAAC
CTAGTATTTAAGTGGTGTTAGTAAGTGTCAACACCGTCCATTTAAGGGACCACCTCTAAAAATTTATTCTTTGTGTCACAAAT
GATTTATAGAGATGATAAGATAAAACTTATGCTTCTGAAAAATAAAAGTCACCGTCTATTAAATCATGTTTTATAATAGTGTAA
ATTGAGAGTGTTTTGATATGCATATGCATTATCATCGTATGAGGAACTGATTGAGTACTTATTGAGGCGATGTTTAGAATGCCG
AAATCTTGTCTTATTCCTACAATTTTCTATGTGTAATTAAATTGGTTTACGAAATACTCCCTCCGTCCAGGAAAACATGCAATT
CTAGCACAGTATCATATTTAGTATCTTAACTTTGACCAATTATAGATAGAAAAATATTAATATTTATGATATCAAATAAATATCAT
TAGTTTAATTATGAAATATATTTTCATAATAAATTGATTTGGAGCCATAAATATGAATACTATTCACTATAAACTTGGTCAAATGT
GAACTACTTTTTCTGGTACGCAACCCATAGTTACATGTTTTTCCGGACGGAGGAAGTACATATACTGTTCCCGTAAAATTCCTT
GGCTGCAAACGTGCCCAAAGTGGCCAAGTACGATTCAAAATGTCTACGTGACTTGCATAGTTACCAATGTTGGTGCACCGAC
AGTAATCAACAATGTCTGAAGTCCGAAGGAACGAAAAGAAAGAGCAACGGGACTATGGAGTCACTGAGATTTGTTTATTTC
TACTCGATTATTACAGGATTAATTCTTTTATTAAACACTAACCGAGGTGGGAGCTATGGGCTATGGCTATGGGAAATTGGGAAT
TCGATTCCGATTACTCTGAGTGCGCCACTATTGTGCATCACTGGACCACCGAACAGTAGACGGAGACCGTTGACGCTGGCCA
ACGACTCGCCGACCCCTTTCACACGTAGTATAACAGCCGCGCACGCACTCGCACTGTCGCACAACA
>SsLRR-RLK400
GCAGGATCATAATTCTTGGGATGATCACCTGCTCCGAAGGTGCATGCCGACCGAACCCTACCGACGCATACCTCAAATACATG
GGATCTATCTTTTATAAGCACATTTGAAAGACTAGACTCGATTAATAGATAGTGAGATTAATCAAGTTATTATAGACGCGATGG
GCAAGTTATCCGCTACCAAAAGAATAATGTTGTTAAATCTTTTAATAAACCCAAAAATGCACGTGTACCATGCCTAAATCTAA
CCAGCTGAGTTATGCTCAGTTCACAACAACTAAATCATATATATGATAAATATATACTCCCTCCGTATCGGATTTAGAAGTCGTT
TAGGACAGTGACACGGTCTCCAAAACACAACTTTGACCTCTTGTCTTTATAAAAAATATTTATGGAAAAGTGATATATGTATA
CTTTTATGAAAGTATTTTTCAAGACAAATCTATTCATATAATTTTTACATTTGCAAACTCAACAACTTAAAAGTTATTGATGATT
TATATTCCCAATGTTTGACCCAAACTTTGTCCAAAACGACTTCTAAATATGATACGGAGGGAGTATGTCATTTATCAAAGAAC
AATAAACTATTGTCAATGGATGACCTTAATTAACAACTGATTAATCATTGCTGATAGTTCCACATATGTTCTGTGAGGCACAAA

ATGTTCTATTTTGGAAAACATGGAGTTAAATAGTTCCACCAAAGACGTGGACACTTCTTGTTTCTTCTTTTTCCTTTTAAAAAT
GCCTCAAGATAAGGTGATGACATGAGGAGCTGTTCCACTGCTTACGCATGCATCCGATGGGATGTACTCTCTTCAGGTTAGTA
AAAGAAGTCGTTTTAGACAACGATATGGTCTACAAAGCATAACTTTGACTTTATTTTTCTGTAAAAATATTTGTTTAAAAGTG
ATATATGTATACTTTTATGAAAGTATATTTCAAGACAAATTTATTCATATGGTTTTCATATTTCCAAACTCAATAATTTAAAAGTT
ATTCATGATTTATATTTCCAATGTTTGACCCAAATTTTATCCAAAACAACTTCTTTTACCAACATGGAGGGAGTACACTTTTAC
CAACATGGAGGGAGTACATAGCATACGAAGAGCATGTTTACATGGCAACAAGGATGTCACAAAGACACAAACTCACAATAA
CTTTAGGAAAATCCATCTAGCTCCAGCATGTGGAAATCCTCCTCCCTGTTTGGATTCGAGCTAAAATGTTGTATGCTGCAGCC
TTTTTACAGTACTAACCAAAGTACTACATAGCATACGAAGAGCATGTTGTATACCGTAGCGTACTGTAGTGTTACTGTAGCATT
TTGTTTGTATTTGGTAATAATTATCCAACCGTTGACTAATTAGGCTCAAAACGTTCGTCTCACAAAGTACAACCAAACTGTGC
AATTATTTTTTTATTTCATCTACATTTAGTACTCCATGCATGTATCGCAAGTTTAATTTGACGGAAAATTTTCTTTTTGTATAGTG
CATTTTTTAGGAGTTTTGGAGAAACTAAACAATGCGCCCAAAAAGAAAGAGCAACTGGACTACTGAACTATGGAGTCATTG
AGATTAGTTTATTTGTACTCAGTTATTACAGGGTTATTTTTGTGAAACACTAACCGAGGTAGGAGCTATGTCTATGGGGAACTG
GATTCTACAAAACAGACAGACGGAACAGACTTGATTACCCTGAGTGCGCCACTAACTATGCATCGCTGGGCCACCGAAAAG
TAGCCGGAGACCGTTGACGCTGACCAACGACTCGCACACCCTTTTCACACGTACTAGTATAATAGCCGCGCAGGCACTCGCA
CACCAGCCGTCACTCCCGTGGCCTCACTCCCATAGTCCCATTTCTTCCCCGTCCTCTACTCCTCATCTCAGCTGCTCTGCTCG
GCAGCTCAGCTCAGCTCACCTCACCTCCTCACATCACAGCACAGCCGAGGAAGCTTCCAAGAAGGAAAAAAAAAAAGAAC
AA
>SsLRR-RLK434
ATATGGATTGTCGGGTACCAGCAAAACAGGGTACCCCATTTTCATCGCAAAACCACCCACCAAGACAAATATGACGGCTCAC
GACCCATCACAGCCAGCCCAGACCTTACTCCACTCAAAACCAAAGCCAAAGGACAAGCGCCCGAGTCCCCAACGCTCCTCT
GCTTCGTCCGACCCCTCTCTGTCTTGTTCGACCTCCATGTCGTCATCTAGGCGTCCGAGCCCTGTGACATTATCAAGGCGCTC
GACCCCTTTGCACAGCTACTGCTTCGCTCGACCCCCCTACTGTAACACACTATCACGGGCACGCACGGGCCCCTACCGTTTC
CTGCTAAGGAACAGGAGCCCGCCGCGTCGTGCAGGATTGCCTGGGAACTTGTAACACCTGATTCATGATCCGGTCAAGGCT
TCCACGGTTCTTTCTCGCCCCCCACGGCAGTAATAGGGAGAAGCTACGCTACGCCGAACCCTACCATGTCCACCTCAACGCG
ACAGGGAGCAACGGCCGTCCAGGCCCCCCACCTTTTCGCCGGCGCAGCTTCAGGGCCACGAAATCTTCTTCATTATGACAC
CGTGTCAGGGTATGGCCTCGCCGCATCAACTGTGATACATCTACAGCCATGGCATGCAACCACCATGACGACGACTGTGCGA
ATGTACGAACTGAGAGGAAAGCTAGGAATAGGGAGCACCTTAGGGTCTTTTCTTTCTATTTTCGTACATCCAGATACTGTAAC
CCGACTTCTCCCTTTGCATACTATAAAAGGGGAGGGAGAGACCCTCGACAAGTGACGTTGGACAACGCCCAACACCGGCAA
TGTACTAAGCACACACTCAAACACTCGAATACACCCACATACAGAGACCTGAGAGCTATCCCTCTCTCGACCAGCTTGTATC
CCCCCTACTACAAGCACTTCGGTGCAAAGCAACACGAGTAGCAGCGAACACTGGAAGTAGGGACGTTCAGCCCGAACCAG
TATAAATCTTGTGTCCGCCTTGCACATCATCGGAGGCTCCGACGCGCAGCATCATTCACTTGTCTCGTCTCTAGACACCCTCT
GTTCTAGAACACCAACACGGATGTTGCATATGTTGTAAGTGGATTTTATCTGGATACTGCATATGTTTTCATACATATGATGCAA
GTGTTTTATCTAGATGTTGTGTATGTTTATAATAGTTTCAAGTGTTTTCTGGTGTTTTTGCAAATGTATCATACGCATGTTTCAA
ATGTTTTATTTGTTTTTAGACGAATATTGCAATTGTTGCATGTGGATGTTTTAAAACTAGATTGAGCGTTGCATCTCCTCCTCGC
CTTTCTGCTGTCTCACTTCAGTGTTAGACATGGAAAGGCTAAAGTGGTCCCCCACTGGCACGGGACGGGCCCCACGTGCTTC
CGGGCAAGTGCAGCAGGTGAGACGTGGACGAGCACGCCCAAAATTCATTGGCTGCAAACGTGCCCAAAGCTGCCGAGTAT
GATTGAAAATGTCACGTGACTTGCATAGTTACCAATGCCTGTGTACCCATAGTCATCAACACTATCTGAAGTCCGAAGGAAC
GAAAAGAAAGAGCAACTGCACTAGTACTCAACTATGGAGTCATTGAGATTAGTTTATTTGTACTCAGTTATTATAGGGTTATTT
TTTTATGAAACACTAACCGAGGTAGGAGCTATGACTATGGGAACTGGATTCTAACAGAAGAGACAGACAGAACAAACTTGA
TTAGCATCCATCACCCTGAGTGCGCCACTAATTATGCATCCCTGGGCCACCTAGCCGGAGACCGTTGACGCTGACCAACGAC
TCGCCACCCTTTTCACACGTATAACAGCCACGCACGCGCTCGCACACCAGCCGTCACTCCCGTGGCCTCACTCCCGTCTTCC
CCGTCCTCTACTCATCTCAGCTGCTCTGCTCGGCAGCTCACCTCACCTCACCTCCTCACATCACAGCACAGCCGAGGAAGCT
TCCAAGAAGGAAAAAAAAAAGAACAAAA
>SsLRR-RLK199
TTGTAATAAGGTTTAAAATCATGGATTAATTAGAAATCATATCCACTAAATACAATATAAATAGTTTCAAAAGCCAAACTGATA
ATTCAAATATATTCATATAGAGCACTAGAGACTTACAATGTTATCTTGCTGATGAGTTTATTCGACGCGTTCGAAGGGACATAA
ATGTCTCAATTATGTCATCTTCATCAACTTCAAAGAAAATATCTCGCTCAATGAAAGTGACGAGACAATCATCCAGAAGACTA
TCTCCCATCTTGTTTCGGGTTTTTGTTTTCACTAAAACCAATGCAGAAAATACCCTTTCAACACTTGCTGTTGCCACAGGTAG
AAGCAGCACCAATTTGAGAAGCTCATAAACCATATCATACACTTTATGTCTCTTCGTTTGAACAAGCTTAACTGAGAGATCAA
CAATATTTTCTAGACCTTTGAATCTATCATCCTGCTTGATGTCATCAATATAATTATCTAGCTGCAATTCAAGTCTGCGCAAATT
ATTTTTTGAGAAGTCCTTTGGGTAGAACTCAGCTAGTCTGCGTAGCTTCTGCGCATCAAAAGACTCAAACGATTTAGAAGGA
TTGAATGCCACCATACAAGACAACAACTCCATATTGATCTCATCAAACCGATTATTAAGTTCTTGACTTATTTGATCAATGACA
CCAATATATACTTCTCTTCTGAAGTGATCATCATTGGTTTGCTTTCGAGCACGAGCTTTCCTTGTTGATTTTCCATAAGGAACA
TAAACACCATCCATAGCAGGAACATCAACACCATGTTTATCACAAAATGAGGTGACCTTCTCAAGAAAAGTATTCCAACCAT
CAGACCTCAATTGTTGCATTCTTTTCTTTGCCACATTAACAAGTGATATTGCATTGAGAATATCTTGCTCCCTTCTTTGTAGAC
ACTCAGATCACTATGAGTCTATGAGCTTCTTCGAAGACTAGGTCGGCAAATCCCTGAGATTAACGAAGTCACCACAGGCATC
TCATTGTCAATGGAAACGTCGCCTACCACTCTATGGATGCATAAGGCCATTAAATCCTTAGAAAATTTGTTCTCACAAAGAGT
CAGAACCTAGGACCTGAGTCTACTACTAAGACTCTTGGTTGTTCTTGTTGCAACATGTTGATTTTTTTGTTCTCTTTCCCTTAT
CAACTTATAAGACTAGGGAAAGTGCTAATCATAGCCGTTCACTGGACAAAATAGCAGCGGATGAACCCGTGACTTTTCCTTT
TCAAAAAAATATTTTCATAACTTTATGTTTTTGGAAAATTACAAAATTACATTCATATTTTTTGTGTATAACATTTTACGTAATAA
ACCTTATCCATAATTTCTTAGCAAGCTCATAACTTTTGTGTATATATAACATATTGTGAAACAAAACTTATTACCACATTTTTTAT
CATAGCTTTTTCAATACGTTTTTACGTAATTTTATACACAGTTTATCATGACAACTTTTCTGCATGATTTTATAACTCAATATTGT
TCATGACTATTAGCGAACAAATTTATCATAATAATTTTTTAACATAATTTTTGTATAATAAATTGTACGAATAACATAGCTTATAC
ACATGTTTATATTGTCTAACAATTTTTTGACATAACTTGTTCCAAACAAACTTCTAAATCTTAAAAAATATTCCTTGTATATAAA
AAGCAGAATTGATAGGTTAATATGAAAAATTTAAAATGAAATGAAAGAAAAGAAAAAAGTGGAAACTACATAGAGGCAGAG
AGCGCCGGAGGGGGGGAAGAAAAAGGAAAAAAAAAATGAACGATCGCCGTACTGGATTTGGGTACGGAAGTAGACTTGACC
GATGACCCAGCCCAGGTCATTAATCCAGACGGAGCTAACACGGCAAGGCAAAGCTACACATACCATCCACGGTCTACTGCA
CATCACATTCGTCTGTCAACATTGAAGTCTCAAGTACAACGACACACTGCTACACGCTTCTCCAGTTCATTCACAGTGGC
>SsLRR-RLK227
GTGCTACGGGAAACTCATTGTAGGTAGTACAATTTGGCATCGTCTAGCGCCTTGTACGTAGCAGATGGAGCTTGGTCGTCTAC
ATGGCGGCCTTGGGCCTAGACTCGAAACTGGATCTGCATCCGAGGTTCCAGACGTATCGTGGTTGTCCTCAGCGGCGTATGC
GGGAGCACAGGTCCTGGCAGCCGAGCGTCATTTTGAAAACAACTATAAAAAGTGAGTTTTTTTAGGAGTTCTTCTTCTATAA
TAACCGTTAGATCTTTCCAATTTCAAATCTAAAAGGAATTCTAATGCAAATAGAGAAGGATGTATCTATAATATGCACCTTTCA
CGGAAGCTTTGGCGTGGGGAGTTCACGTGTGACTTGAGTTTGGAAATCCTATGCATCTGACATATTTGGATTAATGGCATGCA
TGTACGTAACAAAGGAGAAAAGTATTGGAGTCCGAATTCAATTATGGTGCATCTCATGTTTGCATATGAGCCTGATTGGCCAA
ACAGAAAAAGGAGCGTGAACAGTTGAAGGTGAATTCTATTATGTTTGGACTTTTTTTTCTCAATAGGAATTCAGTTTTCGTTG
CTGTTTGTATCCTTCTTTCCATCTTTTTTTCGGCATGTTTTTTCCAGAGTCCCAAATCAAGAGTCCCAAATCGAGGACTCATGA
ACCAATCCGATTCTTTTCTTTATGTATCACGCTAGGAGTCTTACATCTTACGATCTTGGATGATGGATGACTCATATAGCATGAC
GTCTAGATCGAACAGTTATTATAGAGAGAGAGCCCCTAAAAAAACTCGCTCTTTTATAGTATGATAATGATGATTGGAGTGCT
AGAACAACTTCTTCGATAGCGTGGGTTCCATCGTTTATCTGTCACAATAGATCCAATTGCAAGATATTTACCACCTATTTTGC

CTATTTTCCCTCATATTTTTCAAAATAAGAAAACATATAAATATCTCCTGTATCCATGCAAAATAGTAAGGCGATGGCAATGTCT
CGGTTGTCATTTTGTTGATTTTTTTAGTTATACAGTACAAGACAGACGCTTACAATGCACACAAACTCACATCTAGACACACG
TACGCAAACTCTATCACTGTGAGCTTTTTCGAAGACTAGGTCAGCAAATCCCTGAGATTAACGAAGTCACCACAGACATCTC
GTTGTCAACGGAAACGCCGCCTACCACTCACTGGATGCATAAGGCCATTAAATCCTTAGAAAATTTGTTCTCACAAAGAGTC
GAACCTAGGACCTGAGTCTACTACTAAGACTCTTGGTTGTTCTTGTTGCAACATGTTGATTTTTTGTTCTCTTTCTCTTATCAA
CTTATAAGACTAGGGAAAGCGCTAATCATAGCCGTTCACTGGACAAAATAGCAGCGGATGAACCCGTGACTTTTCCTTTTCA
AAAAAAATATTTTCATAACTTTATGTTTTTGAAAAATTACAAAATTACATTCATATTTTTTGTGTATAACATTTTACGTAATAAAC
CTTATCCATCATTTCTTAGCAAGCTCATAACTTTTGTGTATATATAACATATTGTGAAACAAAACTTATTACCACATTTTTTATCA
TAGCTTTTTCAATACGTTTTTACGTAATTTTATACACAGTTTATCATGACAACTTTTCTGCATGATTTTATAACTCAATTTTGTTC
ATAACTATTAGCGAACAAATTTATCATAATAATTTTTTTACATAATTTCTGTATAATAAATTGTATGAATAACATAGCTTATACAC
ATGTTTATATTGTCTAACAATTTTTTGACATAACTTGTTCCAAACAAACTTCTAAATCTTAAAAAATATTCCTTGTATATAAAAG
CAAAATTGATAGGTTAATATGAAAAAATTAAAATGAAATGAAAGAAAAGAAAAAAGTGGAAACTACATAGAGGCAGAGAGC
GCCGGAGGGGGGGAGAAAAAGGAAAAAAAAAAGAACGATCGCCGTACTGGATTTGCGAACAGAAGTAGACTTGACC
>SsLRR-RLK6
GAAGCGAAGAAAGATGAAATGGAATCGATAATACCAATAATATTTTGGGATCTAGAATAAATTCCTAAGGAGCCAAAACAGT
AGGCCGTAATGGGTCTACAAGACAAAATGTGACTCCAAAGGAAATATGTAAAGATGTGGAGCGCGACTTGAATCAAAAGTT
TTGCGCGAAGCGAATGAATAGATTACAATGAGTGCTAGTGGCACATCATGATCTGATTACATCAGATGAAAGTAAAGACAAC
ATTTCCCAATGGGATTTGTATGAAAATAATTACGTGGCACAACCCAGAGGTTTTGTCATGAAAAGAAAATGTAAGGGATGCC
ACCTGTAGAAATCTCTTATGGACTGGAGCAAATCTCTAGACAGTGGCATTTAAAGTTGAAGAGTCAATTAGAAATTTTGGGTT
AAAGAGAATGAGAGGACAATTGCGTTTGTACAAAGTTATAGAAGGGAAATTCATTTCCTAATCCTATGTGGATGACACCCTA
ATTGTTAGTAGTGATGTCAACCTACTGCATGAGAAGAAGTTCTTGTCCTCAAGGTTGAATAAGAAAATTCTCGGTGAAATGTA
TCTCGTTAAAAGAATTGAAATTCACCGAGATAGAAGAAAAGGGGTATTATGACTATCGCATAGGCATGCTAAGAAAAGTTTC
TAAAGAAATAAAGTACCTATGCGGGTAAACCTATGCCTGTTCTAAAGGTCAGGGTAATAGTTTTGAGAACTTTGATATTCCAA
AAGTAATGTAAGATTGTTATAGATGGACGTCCTATATACTTCAGCTGTTAAAAGCTTAATGAGTACAAAGATAAGACCTTGCC
CTGACACAGTTTACATATCCGGGTTGTTTTGGCAAAAGTCCAGTCCAGATATAGATCACTGGAATGGAGTTAAAAATGTCGG
CCTCATGCTAAAAAGAAAGTACAAGTACTCTCAAGTAGTTGTGGGTACAAATGTTAAATTTTGGCGAGATGTTTAGTGAAAC
CCACATAGTAGCTAACACTCGCAGTTGGAGTTTTATTGTGGAAAAGCTCCAAAAGAAACAGTTCTAGTATCATCAGTGATGT
AAAGTTATAATATAGCATGATATGAGGCTGAGGGACAGGCGAAATGGTTAAGTAACTTGTACCCGGAATTGATAATGGTTGTC
AACAGCAATGACCATTTAAGTAGTTTGCTCCTATAACAACGAGTCAAGTGTGCTGCCAAACACATTGACATTAAAGTTATACG
TTGTAAAGGAGAAAGTCCGGAATCATACTAAAAGTATTGAACGTAAAGGTAACAAGCAAGTGCTTGCGGATCCGTTTACTAA
AGGCTTACCACCCAGTGTGTTCAGAGAACACACAGTCGACATGGGTTTATGGTATAGCCTATGATTTCCGGACAATAAAGGG
CCCAAGGTTAAAGAATCTGTTTCAGATCAGAGATGTGTATTGTAGCTGTTGAATCTATCGGCGACTGACCGTGACGATGAGG
CATGCTCTATACACTGATTTGTGATGGAACAAATAAAAGTAAAAGAAATTAAAGTCAAAGTTTAAAGTTAAAGTAAGTTGAG
ATCAAGGGGGAGAATGTTGGAATTGATCTCCCACCGATTAGGCCCAACGGCCTAATTGGGCCTTGACTCGTGCCCTGATCGG
GGGCGCTCAACCCATTAGCGGTTGGTGGGCCCCGTCGCACAGTGCCATATAAGAAGAGGTGGGAGCCACGGGCTCAGTGCG
AGAGGTTTAACGCCACAGTACACCCACCGTCTAACCCTAGCCGATCCCGAGGGTGGCACTGCCAGCGGCGGGATGTCCCAC
CGGCGATGCCAGTGCATCGCTTCTCGCCACCTCCACCGACTTCACGACGGACTTCACTTAGGACGGCGGTCAGTAACGTCTC
CCGTTCCCATCCTCCTCTCTCTCTCTCTTTCATTAAGACGGTTTATCCCAAATGTAAATAGAACTGCCCGCTAGATCTACCACT
GGACGATCGTAAAAATTCAACAAAGCA
>SsLRR-RLK414
GATGAATACATATGTTATGTTTAAGCAATTATGAAAAAAAACATTCCCTTAACCATTGATATAGGAATAAATATTTTCATCAAAA
AATATAGGAATAATATTAACAAATTATTTAAAGTTATATTAATGCAATGCCATATAATTTATATAATTTGTTACTTTAGATAAATTG
TATCTTTTTAACAGATCTGTGTAGTATATCTATATTGATTGTATAATTGTAGGAAGTCTATTTTATAATAAAGTAATGTTGATACA
GATATAATGCTTTGATAAGAAAAATCCAAATAACTTGGGAAGTGGGGGGAGGTTGGTGAGAGCAATTGGTGGCAATACCTTT
TATGGTCTTTGTGATTTGAGTAAAAAGTGATTATAAAGCCATAATATTTGTATCCTCTTCTTGTTAGCACGGTTACCCTATTCTA
GACCATACTCCAAGCTCTATCATCGATCTTGAGCGATGGGTACATGTTTAATGACCATTTATGTCATCTTAGTATTTAAGAATTC
TGTTTATACGATCTAAAGTCCTTATTTATAATAAACATAAAATGAGAGTTAATAAAAGATACGATAAGCATAAGAGTTCCAACT
TAACATGTAGATTAAAAAACTTATGGCAACTATAAGATCAAAATTTGAAAATAGAAATATATGTTCATAAAGATATGGGTTGTA
ACAAGAGCCACAATAATTTATGTTAGAACTTAATGCATATTGAAAATATACTGACGTGTCATACTAAGAGAAAAAAGAATGAA
TAGGGATGAAAAAACATATAGATCAATAATTAATAAGTTTACCACAACTAGATATAAATGATATAGTATCTATCTACCTATTATGT
TAGAAAATAAACAAAGAGGAAAAACATATAAATTAATATAAATAATTGTAGTTGGTATTAAACACCAATATCTTATGTTTATGT
CTTGCAAGATTTAGCATGGATCAAGAACTTAATGCCTATTGAAAATATACTGAATAGAAAGAGAAAAACAACTATGGATAGG
GATGAAAAAATGTGGATCAATAATTGATAAGTTTAATAGATATAAATTATATAGTATCTACATGTCTACTAGATAAATGCCCGTG
CGTTGCAACGGGTACAATAATATGAACATGATAATTATAGTTCGCACATATGTTGTACCGTAGCCAAACAAAACCATTTTTTAA
ATATAAACACAGAGAAAGAAACATGGAAATTATTACACGTAGTTTTAATTAGTGCTAGGTATACAATCATCAACATCTAATGTT
TATGTATTCTACTGTATAAGATTTTAGTCCGGATCGAGAAGCAGTAATAATTGAGGTCGTGGAAACATGCTATCACACATTAGT
GCTAGATAACAAAATTGTCCATCGGAGTATCTTTCACTAATCAGTGTCGGCAAAGACTTTGACTGTGAGTCGTTGGTCATTGT
CTTATTGCCGCATGCGCAGTGCGCACTGTCCGCTGGCCTCAGACTAGTCGCAGTGGGTAGCTTAAGCGATACACTAAAATTTT
AGAGTATGAATATAGAGTAAAAACTGCTACACTGGATACTCTAAAAAATATTCTATCTTTTCTCTCACTCTATATTTAGAGTAAT
ACAGTGTTTACTCTATAGTGGGCAGTGATGGACCCCACTAGCTAAACAAAGGAAAAAAATGCTATAACTCACAAACAAAAA
ATCTAAATTAAATTTCGATTGCACCATTAAATTCGTCTCAATAAATCCTTTAAAACTAGATCACACATGGATATATTTAGATAAA
ATAATAAGTGGGTCCTATAGTGGATAGAATAGATAGAGGATGATATAGAGGATGGATGGTGCAGGAGAATTAAGTATAAATAA
GAGAATCTTCCTGACTAGGACGTATAGAGGATGAATATAGAGGTCCATAAGTGCGGATAGTCTCATGCCCTCTTGTTATAAGA
ATCTTCCTAGTAAGTTCACTCACCGTATAGTCTGAACGTCAAGAACACACACTTCATGGTGACAAAA
>SsLRR-RLK200
GCCGTTGGCAATCGCCGGGTTCCCGAAGCTCAAGTCGTTGGTCCTCGACACTAATAGCTTCAATGGGAGCTACCCGGGTGCC
GCCATCGGCAACCTCACGCAGCTCGAGACGCTAACGCTGGCTAGTAACCCCTTCGCGCCAGGCCCCATCCCTGACGAGTTT
GGCAAGTTGAAGAAGCTGAAGATGCTGTGGATGTCGGGGATGAACCTGACTGGTGGCATCCCTGACAAACTGTCGTCGCTC
ACTGAGCTGACGCTGTTGGCTTTGTCCGACAATAATCTTGACGGTGAAATCCCGGGGTGGATTTGGAAGCTTCAGAAGCTTG
AGATCCTGTACCTTTTTTCCAACAGCTTCGCCGGTGCAATTGGGCCAAATATCACCGCCGTGAGCCTACAAGAGATCGACCT
TTCCGCGAACTGGCTCACCGGGCACATACCCGAGAGCATCGGCAACTTGAAGAATTTGTGGTTGCTCTACCTGTACTTCAAC
AACCTCACCGGGCCGATCCCGTCGAGCGTCGGGCGGCTCCGGAACGTCGAGGACATCCGGCTGTTCAACAACAGGCTCTCC
GGTCCGATTCCGGAGAGCATTGGCAACTTGAAGAACTTGGGGTTGCTCTACTTGTACTTCAATAACCTCACCGGGCCGATTC
CGTCGAGCCTCGGGCTGCTCCGGAACCTCGAGGACATCCGGCTGTTCAACAACAGGCTCTCCGGCCCCCTCCCTTAACAAA
CCATATCATTGTACTAGCAATTTTTATTCATAATGTTATGTGTCACCATATGTAAACCAAGTAAATAAACAAAAAAAGATTCATA
TGTTTGGATAAAGATTTATTTTAATAGTTTATCAATCATGTTCCCTTCAACAAACCATATCATTGTACTAGCAATTTTTATTTTAA
ATTATATTAACACTCCATGCTATAAATTTATAAGTTGTTATTTTTATGTTTAATAATTTTGACTAAAACATTATGTAATTTTACGCA
CTCTACATATATTTGGATAATAAAACTAGTACCATATTCTTCCGTATAGTAAATTTAGCATCTCGAGTACTGTGATGGATGCTAA
ACCACCGTAGTATGAAAAAGTTTAACCTGGGACCAATCTTTCTTTAATATAAAATGAGAAAAGTAAAAAATTTGAATTAAATA
TACAATAAGCACTAGAGTTTCAAACACTTAGATACGAAAGTTGAAAAAAATAGGGATTCATGATGTTATAAACGAGGTGTAA

CAAGAGTTTGTATTACTTAACCTTAGGTTTCAACTAGGGTGTAACAAGAGTTTATATTACTTAATCTTAGGTTTCAACTAAATC
ATATCGCATTAGAAAAAAAGATGAAACCTAGATATTCGAAATACAGGTCGAGCCTCCATGGTAATCCTCACCAGAAGCACTA
GAAGAAAAAGATAATTCTTAGAACACTAATAAAAAACACAAAATTGTGGTCCTCAATTATGTATACTATAATCACAATTGATA
ATAATAGCAATTGGACATATTATCTTTTCTCGATAATTACCCATTTGAGTTTATGAAAACAAAATAAAATAGCCCATGAATATGT
ATCTAAACTACACCACTTATGTTGTTATGAAACATAATCTAAAGTCTACATTTACCTAAATTTGCATCTAATTACTTACCTTCCA
TTATGATAGATAATCTATGTAATCCCTTAAATTTGCATTGAAATTGCCACTTCTTTCAATTTTTTTAAAAATACTTTAAGATTTCC
TAAAATATATATCAAAGTAATTTATGGTAGAACTTAATGCCTATAGAAAATATACTGAACACAAAGAGAAAAAGGACAATGGA
TAGGGATGAAAAAATGTAGATCAATAAATCGATAAGTTTATATGTGGTATAAATTATATAGTATCTATATCTCTATTATGTTGGAA
AATAAAAAGAGAGAATGAAACATGGAAATTTTATAAGTAATTTGAATTAGTACTTGGTATTAATCATCAGCATCTAATGCTTAT
>SsLRR-RLK228
GACGGTGAAATCCCGGGGTGGATTTGGAAGCTTCAGAAGCTTGAGATCCTGTACCTTTTTTCCAACAGCTTCGCCGGTGCAA
TTGGGCCAAATATCACCGCCGTGAGCCTACAAGAGATCGACCTTTCCGCGAACTGGCTCACCGGGCACATACCCGAGAGCA
TCGGCAACTTGAAGAATTTGTGGTTGCTCTACCTGTACTTCAACAACCTCACCGGGCCGATCCCGTCGAGCGTCGGGCGGCT
CCGGAACGTCGAGGACATCCGGCTGTTCAACAACAGGCTCTCCGGTCCGATTCCGGAGAGCATTGGCAACTTGAAGAACTT
GGGGTTGCTCTACTTTGTACTTCAATAACCTCACCGGGCCGATTCCGTCGAGCCTCGGGCTGCTCCGGAACCTCGAGGACAT
CCGGCTGTTCAACAACAGGCTCTCCGGCCCCCTCCCTTAACAAACCATATCATTGTACTAGCAATTTTTATTCATAATGTTATG
TGTCACCATATGTAAACCAAGTAAATAAACAAAAAAAGATTCATATGTTTGGATAAAGATTTATTTTAATAGTTTATCAATCAT
GTTCCCTTCAACAAACCATATCATTGTACTAGCAATTTTTATTTTAAATTATATTAACACTCCATGCTATAAATTTATAAGTTGTT
ATTTTTATGTTTATTAATTTTGACTAAAACATTATGTAATTTTACGCACTCTACATATATTTGGATAATAAAACTAGTACCATATTC
TTCCGTATAGTAAATTTAGCATCTCGAGTACTGTGATGGATGCTAAACCACCGTAGTATGAAAAAGTTTAACCTGGGACCAAT
CTTTCTTTAATATAAAATGAAAAAAGTAAAAAAATTTGAATAAAATATACAATAAGCACTAGAGTTTCAAACACTTAGATACG
AAAGTTGAAAAAAATAGGGATTCATGATGTTATAAACGAGGTGTAACAAGAGTTTGTATTACTTAACCTTAGGTTTCAACTAG
GGTGTAACAAGAGTTTATATTACTTAATCTTAGGTTTCAACTAAATCATATCGCATTAGAAAAAAAGATGAAACCTAGATATTC
GAAATACAGGTCGAGCCTCCATGGTAATCCTCACCAGAAGCACTAGAAGAAAAAGATAATTCTTAGAACACTAATAAAAAA
CACAAAATTGTGGTCCTCAATTATGTATACTATAATCACAATTGATAATAATAGCAATTGGACATATTATCTTTTCTCGATAATTA
CCCATTTGAGTTTATGAAAACAAAATAAAATAGCCCATGAATATGTATCTAAACTACACCACTTATGTTGTTATGAAACATAAT
CTAAAGTCTACATTTACCTAAATTTGCATCTAATTACTTACCTTCCATTATGATAGATAATCTATGTAATCCCTTAAATTTGCATT
GAAATTGCCACTTCTTTCAATTTTTTTAAAAATACTTTAAGATTTCCTAAAATATATATCAAAGTAATTTATGGTAGAACTTAAT
GCCTATAGAAAATATACTGAACACAAAGAGAAAAAGGACAATGGATAGGGATGAAAAAATGTAGATCAATAAATCGATAAGT
TTATATGTGGTATAAATTATATAGTATCTATATCTCTATTATGTTGGAAAATAAAAAGAGAGAATGAAACATGGAAATTTTATAA
GTAATTTGAATTAGTACTTGGTATTAATCATCAGCATCTAATGCTTATGTCTTCTAGGATTTTAGTCCGGATCGAGAAGCAGTAA
TAATTAGGGTCGTGGAAACCTGCTATCACACACTAGTGCTAGATAACAAAATTGTCCATCGGTATATCTTTCACTAATCAGTGT
GCGCAAAGACTTTGACTGTGCAGTCGTTGGTCGTTGTCTTATTGCCGCATGCGCAGTGCGCACTACCCGCTGGCCTCTTCTTA
TATGAACCTTCCTAGTAAGTTCACTCACCATAGTCTGAACTGAACGTCAAGAACACAACACTTCATGGTGACAAAAA
>SsLRR-RLK249
TAGACTTTGATCGTAATCACCGATAATAGCCATTAGATTTATTATGTTTTACAAAAAAAAAAAACCCACCTCTATCATAATAAAA
AGAATACATAAAGTAACCATCTAACTTTGCATCTAAATTAGAAAAATAACCTCCTGGATTTGAATCTAAATTATCCATAAATGT
TATGTGTCACCATGTAGAAAAATAACCTCTATCCTAGATTAGAAAAATAACCTCCTGGATATAAGTATAATGCTATGTGTCACT
ATGTAGACCAAGTAAACAAATAAAGATTCACACATTTGGATAAATATCTATTTAATAATTTATCAAGCGTGTTCCCTTAACAAA
CCATGTCATTGTACGGACAATTTGTATTTTAAATTACATTAATACTCTATGATAACAAATTTGAGAGTTGTTATTTTAAGTTCATT
TATTTTGAACAAAACAATTTGTAATTTTAAGCACTCTATATTTTGATAAAAAAATTACTCCCATATAATTCTCCGATATAGCAAAT
TAAGCATCTTGAGTGTCGCTGTACATGCTAAACCACCGTACTAACAAAGAATTTAAACTTGGACCAATCTTTTTTAAGAAAAT
AAAATTAATTTTTTAAATAAAATATACAATAATCACTAGAGTTTCAACTTAACACATGGACACAAAAAGTTATAAAAACGTGT
AAGATCAAAAGTTAAAAGCTAAAATATAGATTCAAGATGTTACAAAATGGGATGTAACAAGAGTTTCTAAATAGAGGTTCGC
AATAGAGCCTCCACGGTAATCGTTATCAGAATCATTAGAAAAAGATAATAATTATTAAAAACTCTGACAAAAATCACAAAGTC
GTGGCCATCAATTCAGCATACTCCAATCGCAGTTGATAACAATAGCAATCAGACATTTATGTTTTCTAGATAATTACCCACTTG
TGAATCTCCAACTAAATTATACACTTATGCCATTATGAAAAGTATTCTAAAGTCTAAAATTAGCCTAAATTTGCACCTAATTACC
TATCGTCCATTACGATATATAATTTACATGACACCTTAAATTTTCATCTAAATTGTCACTTTTGTCATTATTAAAAATACCAAAGT
ATCCCTTCAAATCTATATCAAAGTGTTATGAAAAATGATTCATAACCACAAATTATACATATATATACATTTCTAAACTATCCTAT
TAATATATTTAAAATGATTAAGTAAACTTACATGTTGTATCATGATGCATGCATGAGCAATGAATATATATATGCTATGCTTTCTG
CAATTACGAAAGATGTTCCCTTAGCCGTTGATATATAGGAATAGTATTAACAAATTATTTAAAATTATGTTAATATCATGACATTT
AATTTATAGAAATTGTTACTTTAGATAAAATTGTATTTTTTAACTGAATTGTGTAGTATATCTATATTGATTGTATAATTGTAGGA
ACTCTATTTTTATAATAATAAAGTAAGATTGCTTATGACTTGCAAGATTTAGCACGGACCGAGAAGTAGTAATAATGGAATCAA
AGGGTCATGGCTATCCACAAACTATAGTGCTAGATAACAAATTGTCCGTCAGAGTATCTTTCACGAATCAGTGTGGGCGAAG
ACTTTGACTGTGAGTCGTTGGTCATTGTCTTATTGCCGCATGCGCAGTGCCCACTGCCCACTGCCCACTGTCCTCTGGTTATA
AGAATCTTCCTACTAAGGCCTGGTTTAGATTGAAAGTTTTTTCAACTCGATGAATAGTACTATTTTCGTCTTATTTGACAAATAT
TGTCCAATTGTGGACCAACTAGACTCAAAAGATTCATCTCGTGATTTCCAACTAAACTGTGTAATTAGTTATTTTTTTACCTAC
ATTTAATACTCCATGCAAACGACTAAAAATTGATGTGATGGAGAGAAAGTGAAAAAACTTTGGAATTTTGAGGAAAACTAAA
CACGGCCTAAGTTCACTCATCATATAGTCTGAACGTGAAGAACACACACCTCATGGTGACAAA
>SsLRR-RLK8
TATCGTCATATAGATCAAGGACACATGCATGCATGAGCGATGAATATATATGTTATGTTTAAGCAATTATGAAAAAAAACATTC
CCTTAACCATTGATATAGGAATAAATATTTTCATCAAAAAATATAGGAATAATATTAACAAATTATTTAAAGTTATATTAATGCAA
TGCCATATAATTTATAAATTTGTTACTTTAGATAAACTGTATCTTTTTAACAGATCTGTGTAGTATATCTATATTGATTGTATAATT
GTAGGAAGTCTATTTTATAATAAAGTAATGTTGATACAGATATAATGCTTTGATAAGAAAATCCAAATAACTTGGGAAGTGGGG
GAGGTTGGTGAGAGCAATAGGTGGTAATACCTTTTATGGTCTTTATTATTTGAGTAAAAAGTGATTATAAAGCCATAATATTTG
TATCCTCTTCTTGTTAGCACGGTTACCCTATTCTAGACCATACTCCAAGCTCTATCATCGATCTTGAGCGTTAATAAAAGATACG
ATCTAAAATTCTTTTTATACGATCTAAAATCCTTATTTATAATAAACATAAAATGAGAGTTAATAAAAGATACGATAAGCATAAG
AGTTCCAACTTAACATGTAGATTAAAAAACTTATGGCAACTATAAGATCAAAATTTGAAAATAGAAATATATGTTCATAAAGAT
ATGGGTTGTAACAAGAGCCACAATAATTTATGTTAGAACTTAATGCATATTGAAAATATACTGACGTGTCATACAAGAGAAAA
AAGAATGAATAGGGATGAAAAAACATATAGATCAATAATTAATAAGTTTACCACAACTAGATATAGATGATATAGTATCTATCTA
CCTATTATGTTAGAAAATAAACAAAGAGGAAAAACATATAAATTAATATAAATAATTGTAGTTGGTATTAAACACCAATATCTT
ATGTTTATGTCTTGCAAGATTTAGCATGGATCAAGAACTTAATGCCTATTGAAAATATACTGAATAGAAAGAGAAAAAAACTA
TGGATAGGGATGAAAAAATGTGGATCAATAATTGATAAGTTTAATAGATATAAATTATATAGTATCTATATGTCTACTAGATAAAT
GCCCGTGCGTTGCAACGGGTACAATAATAAGAACATGATAATTATAGTTCGCACATATGTTGTACCGTAGCCAAACAAAACCA
TTTTTTTAAATATAAACACAGAGAAAGAAACATGGAAATTATTACACGTAGTTTTAATTAGTGCTAGGTATACAATCATCAACA
TCTAATGTTTATGTATTCTACTGTATAAGATTTTAGTCCGGATCGAGAAGCAGTAATAATTGAGGTCGTGGAAACATGCTATCA
CACATTAGTGCTAGATAACAAAATTGTCCATCGGAGTATCTTTCACTAATCAGTGTCGGCAAAGACTTGAGTGTGAGTCGTTG
GTCATTGTCTTATTGCCGCATGCGCAGTGCGCACTCCGCTGGCCTCAGACAGTCGCAGTGGGTACTTAAGCGATACTCTAAA
ATTTAGAGTATGAATATAGAGTAAAAACTGCTACACTGGATACTCTAAAAATACTCTATCTTTTCTCTCACTCTATATTTAGAG
TAATACAGTGTTTACTCTATAGTGGGCAGTGGCGGACCCCACTAGCTAAACAAAGGAAAAAATGCTATAACTCACAAACAAA

AAATCCAAATTAAATTTTGATTGCACCATTAAATTCGTCCAATAAATCTTCAAAACTAGATCACACATGGATATATTTAGATAA
AATGATAAGTGGGTCCCATAGTGGATAGAATAGATAGAGGATGATATAGAGGATGGATGGATGCGGAGAATTGAGTATAGATG
AGAGAATCTTACTGACTAGGACGTATAAGATGAATATAGAGGTCCATGAATGTGGATAGTCTCATGCCCTCTTGTTATAAGAAT
CTTCCTAGTAAGTTCACTCACCATATAGTCTGAACGTCAAGAACACACACTTCATGGTGACAAAAA
>SsLRR-RLK7
ATGTTATGTTTAAGCAATTATGAAGAAAAAAACATTCCCTTAACCATTGATATAGGAATAAATATTTTCATCAAAAAATATAGG
AATAATATTAACAAATTATTTAAAGTTATATTAATGCAATGCCATATAATTTATATAATTTGTTACTTTAGATAAATTGTATCTTTTT
AACAGATCTGTGTAGTATATCTATATTGATTGTATAATTGTAGGAAGTCTATTTTATAATAAAGTAATGTTGATACAGATATAATG
CTTTGATAAGAAAAATCCAAATAACTTGGGAAGTGGGGGGAGGTTGGTGAGAGCAATTGGTGGCAATACCTTTTATGGTCTT
TGTGATTTGAGTAAAAAGTGATTATAAAGCCATAATATTTGTATCCTCTTCTTGTTAGCACGGTTACCCTATTCTAGACCATACT
CCAAGCTCTATCATCGATCTTGAGCGATGGGTACATGTTTAATGACCATTTATGTCATCTTAGTATTTAAGAATTCTGTTTATAC
GATCTAAAGTCCTTATTTATAATAAACATAAAATGAGAGTTAATAAAAGATACGATAAGCATAAGAGTTCCAACTTAACATGTA
GATTAAAAAACTTATGGCAACTATAAGATCAAAATTTGAAAATAGAAATATATGTTCATAAAGATATGGGTTGTAACAAGAGC
CACAATAATTTATGTTAGAACTTAATGCATATTGAAAATATACTGACGTGTCATACTAAGAGAAAAAAGAATGAATAGGGATG
AAAAAACATATAGATCAATAATTAATAAGTTTACCACAACTAGATATAAATGATATAGTATCTATCTACCTATTATGTTAGAAAAT
AAACAAAGAGGAAAAACATATAAATTAATATAAATAATTGTAGTTGGTATTAAACACCAATATCTTATGTTTATGTCTTGCAAG
ATTTAGCATGGATCAAGAACTTAATGCCTATTGAAAATATACTGAATAGAAAGAGAAAAACAACTATGGATAGGGATGAAAA
AATGTGGATCAATAATTGATAAGTTTAATAGATATAAATTATATAGTATCTACATGTCTACTAGATAAATGCCCGTGCGTTGCAA
CGGGTACAATAATATGAACATGATAATTATAGTTCGCACATATGTTGTACCGTAGCCAAACAAAACCATTTTTTAAAATATAAA
CACAGAGAAAGAAACATGGAAATTATTACACGTAGTTTTAATTAGTGCTAGGTATACAATCATCAACATCTAATGTTTATGTAT
TCTACTGTATAAGATTTAGTCCGGATCGAGAAGCAGTAATAATTGAGGTCGTGGAAACATGCTATCACACATTAGTGCTAGA
TAACAAAATTGTCCATCGGAGTATCTTTCACTAATCAGTGTCGGCAAAGACTTTGACTGTGAGTCGTTGGTCATTGTCTTATT
GCCGCATGCGCAGTGCGCACTGTCCGCTGGCCTCAGACTAGTCGCAGTGGGTAAGCTTAAGCGATACACTAAAATTTTAGAG
TATGAATATAGAGTAAAAACTGCTACACTGGATACTCTAAAATAATATTCTATCTTTTCTCTCACTCTATATTTAGAGTAATACA
GTGTTTACTCTATAGTGGGCAGTGATGGACCCCACTAGCTAAACAAAGGAAAAAAAAATGCTATAACTCACAAACAAAAAA
TCTAAATTAAATTTCGATTGCACCATTAAATTCGTCTCAATAAATCCTTTAAAACTAGATCACACATGGATATATTTAGATAAAA
TAATAAGTGGGTCCTATAGTGGATAGAATAGATAGAGGATGATATAGAGGATGGATGGGTGCAGGAGAATTAAGTATAAATAA
GAGAATCTTCCTGACTAGGACGTATAGAGGATGAATATAGAGGTCCATAAGTGCGGATAGTCTCATGCCCTCTTGTTATAAGA
ATCTTCCTAGTAAGTTCACTCACCGTATAGTCTGAACGTCAAGAACACACACTTCATGGTGACAAAAATG
>SsLRR-RLK203
CTTAGTACTAACTTTTTTCCCAAAATATGTACATATTATGAAAATCTATCGATTTTACTATTACTAACTAGAGATTCTAAAGGAG
TTTTCATATTAACTCTCATAAGACACGTTAGGGAAAAGAAAAAACTTATAAACTCTCGTAATAAAAAAAGAATGACCTTTAAT
TTGGTAGACTTTGATCGTAATCACCGATAATAGCCATTAGATTTATTATGTTTTACAAAAAAAATACCCACCTCTATCATAATAA
AAAGAATACATAAAGTAACCATCTAACTTTGCATCTAAATTAGAAAAATAACCTCCTGGATTTGAATCTAAATTATCCATAAAT
GTTATGTGTCACCATGTAGAAAAATAACCTCTATCCTAGATTAGAAAAATAACCTCCTGGATATAAGTATAATGCTATGTGTCA
CTATGTAGACCAAGTAAACAAATAAAGATTCACACATTTGGATAAATATCTATTTAATAATTTATCAAGCGTGTTCCCTTAACA
AACCATGTCATTGTACGGACAATTTGTATTTTAAATTACATTAATACTCTATGATAACAAATTTGAGAGTTGTTATTTTAAGTTC
ATTTATTTTGAACAAAACAATTTGTAATTTTAAGCACTCTATATTTTGATAAAAAAATTACTCCCATATAATTCTCCGATATAGCA
AATTAAGCATCTTGAGTGTCGCTGTACATGCTAAACCACCGTACTAACAAAGAATTTAAACTTGGACCAATCTTTTTTAAGAA
AATAAAATTAATTTTTTAAATAAAATATACAATAATCACTAGAGTTTCAACTTAACACATGGACACAAAAAGTTATAAAAACGT
GTAAGATCAAAAGTTAAAAGCTAAAATATAGATTCAAGATGTTACAAAATGGGATGTAACAAGAGTTTGTAAATAGAGGTTC
GCAATAGAGCCTCCACGGTAATCGTCATCATAATCATTAGAAAAAGATACTAATTATTAAAAACTCTGACAAAAATCACAAAG
TTGTGGCCATCAATTCAGCATACTCCAATCGCAGTTGATAACAATAGCAATCAGACATTTATGTTTTCTAGATAATTACCCACT
TGTGAATCTCCAACTAAATTATACACTTATGCCATTATGAAAAGTATTCTAAAATCTAAAATTAGCCTAAATTTGCACCTAATTA
CCTATCGTCCATTACGATATATAATTTACATGACCCCTTAAATTTTCATCTAAATTGTCACTTTTGTCATTATTAAAAATACCAAA
GTATCCCTTCAAATCTATATCAAAGTGTTATGAAAAATGATTCATAACCACAAATTATACATATATATACATTTCTAAACTATCCT
ATTAATATATTTAAAATGATTAAGTAAACTTACATGTTGTATCATGATGCATGCATGAGCAATGAATATATATATATGCTATGCTTT
CTGCAATTACGAAAGATGTTCCCTTAGCCGTTGATATATAGGAATAGTATTAACAAATTATTTAAAATTATGTTAATATCATGAC
ATTTAATTTATAGAAATTGTTACTTTAGATAAAATTGTATTTTTTAACTGAATTGTGTAGTATATCTATATTGATTGTATAATTGTA
GGAACTCTATTTTTATAATAATAAAGTAAGATTGCTTATGACTTGCAAGATTTAGCACGGACCGAGAAGTAGTAATAATGGAAT
CAAAGGGTCATGGCTATCCACAAACTATAGTGCTAGATAACAAATTGTCCGTCAGAGTATCTTTCACGATCAGGTGGGCGAA
GACTTTGACTGTGAGTCGTTGGTCATTGTCTTATTGCCGCATGCGCAGTGCCCACTGCCCACTGCCCACTGTCCTCTGGTTAT
AAGAATCTTCCTACTAAGTTCACTCATCATATAGTCTGAACGTGAAGAACACACACCTCATGGTGACAAAAATGGCTCACTC
CTATCTTCTACTCCTCTTCCTACTACTGTTCATCACCTTCAGCTCCAGCTTGCAACAATCCA
>SsLRR-RLK372
CTTAGTACTAACTTTTTTCCCAAAATATGTACATATTATGAAAATCTATCGATTTTACTATTACTAACTAGAGATTCTAAAGGAG
TTTTCATATTAACTCTCATAAGACACGTTAGGGAAAAGAAAAAACTTATAAACTCTCGTAATAAAAAAAGAATGACCTTTAAT
TTGGTAGACTTTGATCGTAATCACCGATAATAGCCATTAGATTTATTATGTTTTACAAAAAAAATACCCACCTCTATCATAATAA
AAAGAATACATAAAGTAACCATCTAACTTTGCATCTAAATTAGAAAAATAACCTCCTGGATTTGAATCTAAATTATCCATAAAT
GTTATGTGTCACCATGTAGAAAAATAACCTCTATCCTAGATTAGAAAAATAACCTCCTGGATATAAGTATAATGCTATGTGTCA
CTATGTAGACCAAGTAAACAAATAAAGATTCACACATTTGGATAAATATCTATTTAATAATTTATCAAGCGTGTTCCCTTAACA
AACCATGTCATTGTACGGACAATTTGTATTTTAAATTACATTAATACTCTATGATAACAAATTTGAGAGTTGTTATTTTAAGTTC
ATTTATTTTGAACAAAACAATTTGTAATTTTAAGCACTCTATATTTTGATAAAAAAATTACTCCCATATAATTCTCCGATATAGCA
AATTAAGCATCTTGAGTGTCGCTGTACATGCTAAACCACCGTACTAACAAAGAATTTAAACTTGGACCAATCTTTTTTAAGAA
AATAAAATTAATTTTTTAAATAAAATATACAATAATCACTAGAGTTTCAACTTAACACATGGACACAAAAAGTTATAAAAACGT
GTAAGATCAAAAGTTAAAAGCTAAAATATAGATTCAAGATGTTACAAAATGGGATGTAACAAGAGTTTGTAAATAGAGGTTC
GCAATAGAGCCTCCACGGTAATCGTCATCATAATCATTAGAAAAAGATACTAATTATTAAAAACTCTGACAAAAATCACAAAG
TTGTGGCCATCAATTCAGCATACTCCAATCGCAGTTGATAACAATAGCAATCAGACATTTATGTTTTCTAGATAATTACCCACT
TGTGAATCTCCAACTAAATTATACACTTATGCCATTATGAAAAGTATTCTAAAATCTAAAATTAGCCTAAATTTGCACCTAATTA
CCTATCGTCCATTACGATATATAATTTACATGACCCCTTAAATTTTCATCTAAATTGTCACTTTTGTCATTATTAAAAATACCAAA
GTATCCCTTCAAATCTATATCAAAGTGTTATGAAAAATGATTCATAACCACAAATTATACATATATATACATTTCTAAACTATCCT
ATTAATATATTTAAAATGATTAAGTAAACTTACATGTTGTATCATGATGCATGCATGAGCAATGAATATATATATATGCTATGCTTT
CTGCAATTACGAAAGATGTTCCCTTAGCCGTTGATATATAGGAATAGTATTAACAAATTATTTAAAATTATGTTAATATCATGAC
ATTTAATTTATAGAAATTGTTACTTTAGATAAAATTGTATTTTTTAACTGAATTGTGTAGTATATCTATATTGATTGTATAATTGTA
GGAACTCTATTTTTATAATAATAAAGTAAGATTGCTTATGACTTGCAAGATTTAGCACGGACCGAGAAGTAGTAATAATGGAAT
CAAAGGGTCATGGCTATCCACAAACTATAGTGCTAGATAACAAATTGTCCGTCAGAGTATCTTTCACGATCAGGTGGGCGAA
GACTTTGACTGTGAGTCGTTGGTCATTGTCTTATTGCCGCATGCGCAGTGCCCACTGCCCACTGCCCACTGTCCTCTGGTTAT
AAGAATCTTCCTACTAAGTTCACTCATCATATAGTCTGAACGTGAAGAACACACACCTCATGGTGACAAAAATGGCTCACTC
CTATCTTCTACTCCTCTTCCTACTACTGTTCATCACCTTCAGCTCCAGCTTGCAACAATCCA
>SsLRR-RLK374

ATTTACAGGATCCCACTGCAGACAGCCTTACAACGACCAAACGCACCCAGAATGGATGAGATCACAGATCCTCTATCGGGGC
AACGCGTTATGGCCCGTGGGCGCCGCGAGGGGCAAACCAGATCAACAGCAGATCATTTAATTCTAAAAAAAAAAAGATCAT
TTTTTCTAAAACAAGAAGAAGACGAGAAGAAGATCAACAGCAGATCATACGGTTTGGACTTCGCCGCCGCCAGTGCCAACA
CACCAGTGCCGGGTGCCCGATATAGCCCTCTATGCAGCGCCACCGCCCAGGATGGTCGCCTGCTGAACCAAGGTAAGATCCG
GTGGGATCGATCCCCGTTGCTGGCCAGATGGGACATGGGAGGGTGAAGCTTCCCGGTGCCGTGCTGTGCCTGTACGTGCTC
GCCGTCTCTACCTCAGGTAAGCGAAAGCGACGACTTGATGCATATACAAGTCGGTCTACGTATAGGCAACATTCATTAGACG
ATCTTGCATAGCCACTAGTCAGTACTCTGTAGTTTGTAGACCGGATTTAGCTCTCTGTCACTCTCACACATGAGGAATAGATG
ATGCAGTAGGTCAGTCAGTCAACGTGGACCCAACCCATTACCCAAGTGAGGGGCCGAAGGTGACCATCAAGAAATTATTGC
TTCAACTTTGACAATGATATTTTGAGTGTTTAGTTAGGTATAAAACAAAAGTTTTTTTTTCTCGATCACGCAAAAGGTTTACGC
GTCTTTGTACGAAGAAAGATAAATTATTCCATATTGAGTACGAAGGCTGAACAAGTGCAAAGGTAATGTGCTACTGCAAAAG
GAGGAACTGCATATATGCTTGATGTAAAAAAAGAGGAAGGCGGGTGCGGTGGCAACAGCAATGCTCATAAAAGATGGCGTG
AAGGTTTCGGTTGAAGTCTAGTCTAGGCTTTTAGCAAATGTATCTTTGTTTCAGCTAATGTATTTTGCATGTACAGAGTAACTG
ATGCCTTACCAATGATTACAGACTTGCGCGCTCAGATCCTGTATTGTGGGCTGTATCGGGGCGCCGCGAGTGGCAAACCAGA
TCAACAGCAGATCATTTTTCTCGATCTCTCAAAAAAAAAATCATTTTTCTCAAAAAAAAAGAAAAAAAAAGAGAGAAAGATC
AACAGCAGATCACACAGCTCATAGGGTTTGGACTTCGCCGCCGCCATTACCAACACACCAGTGCCGGGTGCCCGATATGGCC
CTCTATGCAGCGCCCACCGCCCACCGCCCAGGATGGCCGCCTGCTGAACCAAGGTAAGATCCGGTGGGATCGATCCCCGTTC
CCAGCCAGATGGCCTGGGAGGGTGAAGCTTCCCGGTGCCGTGCTGTGCCTGTGCGTGCTCGCCGTCTACCTCAGGTAAGCG
AAAGCGACGACCCGATGCATATGCATATACAAATACAAGTCGGTCTACGTAAAGGAAGCATTCATTAGACGATCGATCTTGTA
TAGCCAAGTGTCAGTACTCCGTAGAACGGATTTAGCTCTGTGTGGCTCTCGCTGTCTCACACATCAGGAACAGACGATGCAG
TAGGTCAGTCAGTCAACCCCATTTACCCAAGTGAGGGGGCGGCGGAGACCATCAAGTTGACTGACGATGATATTTTGATTAT
TTAGTTAGGTATAAAATAGAAGTTGCATGGTTAGATTTGACATCAAAGGTATTTTAAGAATATTGTATTTTAGAATTCAATGTGT
TACAAGGTACATGTTTTTTTTGAGGAATTACAAGGTACATGTTATTAGAACAGCAGAGCAACAATAAGAATTGACAGCCGTA
GCGCGTACCGCAAGGAAGCCATTGCTGAGCAAACCAACTGACAATGATACCCAAAACCTGGAGAGAATAAAGTATCCTCTT
TTTTTCTTCCCTTGCTCGAGCCGGTGAAGATCAGCAGGAGCCGCGGGAGGAAAAGAGGCGAAGCTAGCAGCAAGTGGAGG
CTGAGACACAAGAAGGTTCAATCAGATGGCCAT
>SsLRR-RLK380
ATTTGGCTGTTCTTCCTTTTCACTCCCATCCTCTATCGCCTGTGCCCATTCGTTGGGAGGCCAAGGCGGATGGCACTGCGCCG
GTGACGCCAAGTTCTTTCTTCGAGTCCTACATCGCGATGAAGATGCTCGCTAATTTTCCACCGCCGTGACTTCAATTTCATCT
TCCTAACGAGCGGGACTTCAATTTCACCACCAACATCCCCATCCGAATTTTGCTGGCTTCTCTCGACATCTCAAATCTTGCTT
GCTTCTCTTCGACGTCTTCCTGAACTTGCCTTGCAGCCTGTCAAGCTTGTCTCGTATGGCCGCAAACTCTTCAGCAGCTGTGC
GCAGATCTCTACTGACGAGCGTGCGCGAGCTCCACGGCGGGCCTCGCCTACCCGCGGCGAGCGTGCCCCTTGCAGCTATCA
CCGGTGCCATCGGCCGCCGAGCAGAAAGCAGCAGAGCACACGCACCGGTCTAGGGACTTTCCCTGCATGTACCACTCGACC
GCGCTGCCTCGCAACGCGCAGGCGCTGGCCACCGGCGCCGTCGTCCGCCGAGCTGAAAGCAGCAGAGCGTGCACCGGAGG
GGGTCTCGCACCGCGCAGGTGCTGGCCACCGGCTCCGGCGGCTGCCGAGCAGAAAGCAGCAGAGCGCGAACCGGAGGGG
GAGATGCAGACTACGATCGTGAAGGTCAGGGCCTCGGTGGATTTGCGGCGCGCGCGAAGCTTGGCGCGGCGGCAGCACAC
AGGATCCGGAGGAGGGCCATGGAAACCGGTGGTTTCTCCCCAACGCGATTTCTATCGTTTCTTCATGTGTTGATCCGCGTGA
AGACGTCGTTCCTTGAGGAGGAAACGGTTTCTTCTTTTTTACCCTCTCTCTTCATTAATTTCTTTGCCACGTCAGCAAATTGCT
GAGTTATCAATGTAATTAAGAGAGATAGAAACTACCGTAAATGTTCCATTGGGACTGCCCTTACCAATAATTACAGACTTGCG
CGCTCAGATCCTGTATTGTGGGCTGTATCGGGGCGCCGCGAGTGGCAAACCAGATCAACAGCAGATCATTTTTCTCGATCTC
CTCCACAAAAAAAAAAATCATTTTTCTCAAAAAAAAAGAGAGAAAGATCAACAGCAGATCACACAGCTCATAGGGTTTGGA
CTTCGCCGCCGCCATTACCAACACACCAGTGCCGGGTGCCCGATATGGCCCTCTATGCAGCGCCCACCGCCCAGGATGGCCG
CCTGCTGAACCAAGGTAAGATCCGGTGGGATCGATCCCCGTTTCCAGCCAGATGGCCTGGGAGGGTGAAGCTTCCCGGTGC
CGTGCTGTGCCTGTGCGTGCTCGCCGTCTACCTCAGTCAGGTAAGCGAAAGCGACGACCCGATGCATATGCATATATACAAAT
ACAAGTCGGTCTACGTATAGGAAGCATTCATTAGACGATCGATCTTGTATAGCCAAGTGTCAGTACTCCGTAGAACGGATTTA
GCTCTGTGTGGCTCTCGCTGTCTCACACATCAGGAACAGACGATGCAGTAGGTCAGTCAGTCAACCCCATTTACCCAAGTGA
GGGGGCGGCGGAGACCATCAAGTTGACTGACGATGATATTTTGATTATTTAATTAGGTATCAAAAAGAAGTTGCATGGTTAGA
TTTGACATCAAAGGTATTTTAAGAATATTGTATTTTAGAATTCAATGTGTTACAAGGTACATGTTTTTTTTGAGGAATTACAAG
GTACATGTTATTAGAACAGCAGAGCAACAATAAGAATTGACAGCCATAGCGCGTACCGCACTCAATTCTCTTTCAAGCGCCC
TGAAGAACAAAACCCAAGGAAGCCATTGCTGAGCAAACCAACTGACAATGATACCCAAAACCTGGAGAGAATAAAGTATC
CTCTTTTTTTCTTCCCTTGCTCGAGCCGGTGAAGATCAGCAGGAGCCGCGGGAGGAAAAGAGGCGAAGCTAGCAGCAAGTG
GAGGCTGAGACACAAGAAGGTTCAATCAGATGGC
>SsLRR-RLK364
AGAAAATGCAACTGTAGCAATTTCGTTGTTATTTGACAAATATTATTCAATCATGGACTAACTAGGTTTAAAAGATTCGTCTCG
TGAATTTCGTCTAAACTATGTAATTAGTTTTATTTTTTATCTATATTTAATGGTTCATGCATATGTCAAAGATTCGATGTGACGGA
GCAAAATTTTTTACAAACTAAACAAGGTCTGAGGCAAAACCATGCCAGGAACTATTCCGGCTACTGAGAGCTTATACAAATT
AAATAGCAATTCCGGCTGGGAACTGTTCCTGGCACTCATTCCTGATAAAACAAACGGGGCCAGTCCTAAAGTAAACATGGCC
ATAGTGTCAACTAGAAGATGAGCAGAAGAAGTGAAGAAGGATTGTGTACCGATTTTTGTTTCTTCTAGAATCCTGGAAATAA
AACGTCACTCTTTCTTCTAGAATCCGCACAAGAGCTTTACAAATAATACTGTAATCACTGAAGCTCTAGAAGCGTACACTGAT
TGAGGTTTTTACTGAGTATAATAATATATGTATACTGGGAATCAGGCAATCAGCAATCGTATATGTATAACTTGCAGCAGCCCTT
CTTTACAATAACTTGCAGCTGCAGTCGTTGCACCCATGACACAGCTACAGTGGACAACAGTGGCAAGGTAGCCGCCGCTGC
TGGAAAAACTTGCAGCTGAACAGTTACGTAGATTTGTATTAAAAAAAAGTAATCTCAATGCGTCCTTGATTCCTTGTTCTAAA
TTCTCGATCAGAATGTTTTGATCCACCAGGACCGCCACGAAGAACATCCAGCTGGCCAAGAGCCCAAGCCCCAAGGGCACA
CTCGTCTCGTGTCTAAGAGCCTGTTTAGTTATAAAAAAAGAACCAAAAAATGTACAGTCGAATCTTACAGCACATGCATGGA
GTATTAAACGTAGTAATTACACAGTTTAGTTATAAATCACGAGACAAATCTTTTGAGTCTAATTAGTCCATAATTGGACACAAA
TTATCAAATAAAACGAAAAGTGCTACAGTAACCGAACCCAAACATTTTTATGAACTAAACAAGCCCTAAGAAAGGCCACGA
AGAGCCGGAGTAGGTGATCGATTGCGATGCTTTGTTATGATTATGAAGCACGTGTGGTATGCAGTATACACAGTTGAAGTGTC
AAACAAGCAAACAGGGGCCACTACAACTTATCTTTGACTTTCCCGTTTGACTCGGTTTTGGCCGGACCCGCCGGGGAGACT
GACATGCCTGGACTGCGTAACAGGCAGGCGGCAGTTCGTGTCGTGGCTAGCTGCCTCGGGCATTGTTTAGTCGGTGAAATGT
TAGAATTTTTAACTGTAGCATTTTTATTTGTATTTGATAATTAGTATTTAATTATAGACTAATTAGGTTTTTAAAAATTTATCTCAT
GATTTATAACTAAATTGTATAATTAGTTGTTTTTTATTTATATTTAACGCTTCATGCATACGTGCAAAAGTTGATGTGACGAAAA
AAGTGAAAAAAATCCAACTTTAGTCTTGAATCTAAACAACGCCTCGTTTTGTTCATGACCTCCAGCTGAGTTGCTGACGCAG
CGACGCTGCGCCAAGCTCTAGCTCTAGTGGGTCCAGCAGTCAGTGGACGCAAGGAAGGCACTCACTTTTTGTTACTTAGGC
TCTGTTTAGTTCTTAAAAAAAAGACCCTAAAAAATTTTGCACAGTACCGTCATATCGAATTTTTGGACGTATGTATAAAGCATTA
AATGTAGGTAAAAAATAACTAATTGCATAGTTTGTCTGTAAATGTCGAGACGAATCTTTTGAGCGTAGTTAGCCCATGGTTGG
ATAAATTTTGCCAAATAACAACGAAAATGTTACAAGAATCTTTTCCAAAAAATTTTGAGATCTAAACAAGGCCTTAAGAAGA
CTCCAATCTAAACATACTTACACTGTGGGATAGAAGTTGAAGAAGATTTGATTTGACAATTAAAGGTTAGGGTTCAGGTTACC
CGGAGCAGCGA
>SsLRR-RLK384
CAGGCTGCTGTTCTACTCCAGAACCAGAAGACATTACGGAGTCATGACAGCTCCATCTATAAATTGTCCTTCTGAGACTTTAG
GTGAAAGCCATAGGGCATGTGTTGCTTCGCGGCGTGCTCATCGGCACGTCAATCCAGCCACCATACACTGAAAATTGACAGC

TTAGGGCTCGTTCGTTTGAGGCAAAACCATGCCAGGAACTATTCCGGCTACTGAGAGCTTATACAAATTAAATAGCAATTCCG
GCTGGGAACTGTTCCTGGCACTCATTCCTGATAAAACGAACGGGGCCTTAGGGGGCTACTTGGTCCAGACAAATCTGCCAGT
CCTAAAGTAAACATGGCCATAGTGTCAACTAGAAGATGAGCAGAAGAACTGCAGAAGGATTGTGTACCGATTTTTGTTTCTT
CTAGAAGCCTGGAAATAAAACGTCACTCTTTCTTCTAGAATCCGCACAAGAGCTTTACAAATAATACTGTAATCACTGAAGCT
CTAGAAGCGTACACTGATTGAGGTTTTAACTGAGTATAATAATGTATGTATACTGGGAATCAGGCAATCAGCAATCGTATAACT
TGCAGCAGCCCTTACAATAACTTGCAGCTGCAGTCGTTGCACCCATGACACAGCTACAGTGGACAACAGTGGCAAGGTAGC
CGCCGCTGCTGGAAAAACTAGCAGCCGAACAGTTACGTAGACTTGTCTCAATGCGTCCTTGATTCTTTGTTCTAAGACCTTG
TTTAGAGTCTCTCCATCACATTAATCTTTGTACGTATGCATGAAGTATTAAATATAAATAAAAATAATTAATTGTACAGTTTAGT
TATAAATCACGAGACGAATCTTTTAAACATAGTTAGTCTATAATTAGATATTAATTGTCAAATATAAACGAAAGTGCTACAGTT
AAAAAAATTCAAACTTTCCACGATCTAAACCAGCCCTAAATTCTCGATCAGAATGTTTTGATCCACCAGGACCGCCACGAAG
AACATCCAGCTGGCCAAGAGCCCAAGGGCACACTCGTCTCGTGTCTAAGAAAGGCCACGAAGAACATCCTGCTCTGGCCG
GAGTAGGTGATCGATTGCGATGCTTTGTTATGATTATGAAGCACGTGTGGTATGCAGTATACACAGTTGAAGTGTCAAACAAG
CAAACAGGGGCCACTACAACTTATCTTTGACTTTCCCGTTTGACTCGGTTTTGGCCGGACCCGCCGGGGAGACTGACATGCC
TGGACTGCGTATCAGCCTAACAGGCAGGCGGCAGTTCGTGTCGTGGCTAGCTGCCTCGGGCATTGTTTAGTCGGTGAAATGT
TAGAATTTTTTAACTTTAGCACTTTTGTTTGTATTTGATAATTAATAATTAGTATTTAATTATAGACTAATTAGATTTAAAAAATTC
ATCTCATAATTTATAATTAAACTGTATAATTAGTTATTTTATCTATATTTAACGCTTCATGCATGCGTGTAAAAGTTGATATGATAG
AGAGAGTGAAAAAATTCCAACTTTAGTCTTGAATCTAAACAAGGCCCCATTTTGTTCATGACCTCCAGCTAAGTAGCTGACG
TAGCGACGCTGCGCCAAGCTCTAGCTCTAGTGGGTCCAGCAGTCAGTGGACGCAACGAAGGCACTCACTTTTTGTTACTTA
GGCCCTGTTTAGTTCTTAAAAAAAGACCTAAAAATTTTGCACAGTACCGTCACATCGAATTTTTGGACGTATGCATGGAGCAT
TAAATATAGGTTAAAAATAACTAATTGCACAGTTTATCTGTAAATGTCGAGACGAATCTTTTGAGCGTAGTTAGTCCATGGTTG
GATAAATTTTGCCAAATAACAACGAAAATGCTACAGGAATTTTTTCCAAAAAATTTTGAGATCTAAACAAGGCCTTAAGAAG
ACTCCAATCTAAACATACTTACACTGTGGGATAGAAGTTGAAGAAGATTTGATTTGACAATTAAAGGTTAGGGTTCAGGTTAC
CCGGAGCGGCG
>SsLRR-RLK352
TTAGGGTTCAGGTTACCCAGAGCAGTGATGCTGCACGGGTGCTTTTCCTACCCTGTTTCATCACCGATAGTCGTCTGGAGAAT
TTTGGCGTGGCTGGTTGCAAGGCATCCTTCAGCACGCGGTGCTCCAGCACCTTCACCGGTAGAAGTTGTTAACAATGTGTTC
TGATGGATCTTCCAGGCGCTTGCTCTTGCGGTAGATCCAAGGTTTAGCGTCCTTTGTGAGTTTAGAAGGTGGATCTTGGTCTG
ATCTCTACAATTGTTTCTGGCGGACGAAGCTCAAGCAAGGACGTCAAGAACATTCCGATGTCTTTGGGTGTTGTGTGTTGTA
ATTCTTCAATTTTGCCCAAGTGTTTTCTACATGACTAGAGACGCACTGTGCTACAGTTCTTAATATAATTTCTCCTCTTGAAAA
AATGGGTATGCCACGCTCTTCCCCTACCTAAAAAATCACATTTTCTTATAGAGTTATATACTTAATCATGCTCAAATATGATTAA
TCTATCGACTTTATAAGTTACCATTGATGACCCCTTAACATTCATACGAAACTCTGCTACTGCTCAATCTTTTGTTTTGCGTATG
CATACCACACTTGTTCTTTGTCACGCTTCTTCTTTGTTGATAAAATAGAGGCCATTAATAAAGCACTCATAGTATGCCATTTTTT
AGGTTTTGGTTCATCTCCATCTTCTATTTTTAGCAATGGTGCTTCTTCCCGCTTCAAGCAACTGAACTCCTTTGCCTCCACTGC
AATGGTTCAAGGTGTGCTTGGTTGCTAGCCGGAGCACACCACCCTCACCGCCTAAGGTGCGGCACTGATTTTGGCCACCAC
ACTCGCCGCACTCAAAATCAACTACGAAAAGCTGTGGCCACCGTAATTATTTCACCAACCAAGCACGTAACGTTTTCTATAG
CACCACCCAAGCTTTGTCATGGCATCCTATGGTGAGGAACCAAGCACGCCCTCAGTCTACTAGCGAGATATTACTGTGTCTG
CCACTACCGTGATTGCCATTTTTGGCCCCACCCTAGCACTGCCTAGCGACCTCTACCTCCCAACGGCTAGTGTCACAATCGTT
TTGCTTTGCCTCTTTGGTGTCTGCCACCACCAGCACTAGATTCTAGCAACTCCTAACCCTAGCGCCAAATCCTAATTTCTCAC
TAGAGCCGGGGAAGGGGAATGGCTGAAAGGGGTGGTCACTGCCATGAATGGCTAAGTAGATGGAGTAAATAAAAATTCAAA
ATGTTTGTACCTAAGAAACAAATGATTGTGAAAATACTCCCTCCATTCCAGTTATAAGTTGTTTCAACAATACCATGTATCTTG
ACATATTGTACATATAGTAAAACTAAGAACCTAAAAATGCTAAAACAACCTATAATTTGATAGAGGAAGTAGGATTTTTCATGA
AATTCACGCGCTTGCACCGTTCAGTGATACTAATAGCAAAATGATGAATACAAGATCTCTTTGGATTGACAGCTACCTGATCA
TTTCATTTTCAGGCCATCCCTTTTTGTCGCAAGCGGTCGATCTCACCCCGCTGATCGCTGCAGGGGAATAGTTTGGCCATTTT
TCGTGCAGCCTAGAGAAGAGCCGCCGCAGCACCATTCCAGTCCAAAGAACCAGAACAGATTGTGTGCTATTTTCTTCGAGA
TGGCCACAAAATCAACGAAGTCTATCTAACAATTTCAATCCTTGCCACTAAGCCACGTAGCTGTAAGTTTGGTCCGTAGTTAG
CAGCCACATCAAACAACTTCACCTTGAACATATCCTCTCTTCTTCCTTTTTATTAAGCAAACAAACATATTCCTTTCAAACAA
AGCCAAAGTTTTGACCCGTCTAAAACAACGTGCAACCGCCACTATCCAATGACACAAGCGACTCTGAAGCTTCCAAGCATC
CGTCGCCCAACAGACCCGCCCAAATGAGCCAATAATTAACGGGACATCGCAAAAGGCCGGGCGTCACCATCCCCGACCCGT
TCCCACTGACCA
>SsLRR-RLK186
TAGTTTGGATGTTGGGATTTTAATTTTCGTATTCTCTAAGAAAAAGTTATTTGGACAATTTTTTGGAAAAAATCTATAATTTTTT
ATAATAAAGTAATAGACTCCTGACGTGTCATGTCAACTAAAATCAATTTCATCTTAGGAAGAAATTCGGGCGGTTTTAAAGGT
TAGATGAGATAAAAAAAAGAACATGTTTTTTGGTTGGATGAAATCACTTTCATCTTAGGAACAAATTCGGTCGGTTTTGAAG
GTTGGATGAGATAAAAAAAGGAACCTATTTTTTTTGTTGGATGAGGTAAATCGAACAGTGAAAGTTGAATTAGGTTAAATGG
ACTTTTTCTGTTAAATTTTTTTAGTGGATTTTTTCTTTTTCTTTTCCTTTTTTTTGAGCAGACAGTGGATTTTTTTCTGTTTATAT
TTTTTAGCAAATTTTTTCTGTTTATATGAAAACAGACCCTGAGTCTCCGTCGCTCAGGCCCACAGCCCACACTAATTGCGTGT
TTGACGGAATACTATTATGCTGGGCTGACCAGTCTCCGGAGACCATCAGAATGAAGATTTGGAAAGGGGCCACACGATGACA
CGACCCCCAAATTTCGTTGTTTATAATAAATGGTGCAGTAAGGAACATAATTGATTCAATGGAGAGTCGTTGCGTTGTTTATAA
GACTGCCATGTCATATGGAATAAAATAAAACTTGGATGAAACACTTATTTTCAATGTAAAGTTTTATCCTATGATATGATTTTAT
AGATATTTAATTCTAAGAATCGTATAGAGTTGATAATTATGCAAGAGAGTTTCATCTCCATGAAACTTATTTCTTCTCTCTCTGT
TAAAATAGTGTCATCTTATGATTTCATAGATATTTAATTCTAAGACTCAGAGAGACTTGATAATTGTGCAAGAGAGTTTCATCG
CCGTGAAACTTATTTCTTCTCTCTCCATTAAAATAGTGTCATGTCACAAATATATATATTTTGCATGCTATTAAATGCAAATGAAT
CTCTCACCAAAGCTCGAAACTGGCGACCGCAAAGACTTCCGGAATCCGGACTTCCGGAGCACTTCCCATCTGAGGTCGGTC
TTTCAACCGTCCACCGTATGAATCACTCCGAACGGACCACAAAGCCACGTAAATGGAAGTCATTTTTTGAAAGCTCCAACAA
AACAAACGGGACTTTTGTGCCGTCAACGGTAGACTTGTGTTAAACAGGGGAGTCCAGTACCTCTGCACTTTACCGAAGCAC
CTCCACTTCCCGCGTTCACCGCGGCGCGTGCACCACCGACGCCACGGCACGAAAACAAACCCGCCGAGGCATGGAGGCAC
CATGCCCGTACCTCCCCGGCCTGCTGCTCGCGCTGCTTTCCTGCTGCTGCTGCCTCCTGCCCCGCGCGGCCCCGCAGTCCTC
CCAGCCGGCGGACGAGGCGCAGCTGCTTCTCCAGATCAAGCGCGCGTGGGGCGACCCGCCCGTGCTCGCCGGATGGAACG
CCTCGGCCGCGGGCGCACACTGCGCGTGGCCATACGCAAGGATTTGGGATTCGGCCCGAATGGGCCGAATTTCGGTGAAAT
TCGCCCATTTCGGTAGGGCCCGAGAGAAATGTAACCCGGCCAAAAAAATTCGGCCCAGTTTGAATTTTTAGCCCAGCCCAAG
ATCCACCATAGCCCACCAATACCACCTCTAGTATAAAAATGAGAACCCCACAGTAGAAACCCTAGGTTTTTCATTTCTCTCCC
CTCTTCCTGGCTGTGCGCGGCGGCTGGAGAAAGCAGAGTGCGGACCTCTTGTTCTCGCTGCATGGTCGTGCCTCCCTGCCG
CCGGTCACGCCTTGGCCGCTCCTCCCCACCTCCGGTCACGTCCCTGCTGCTCGTCCCCATCTCCGGTCGTGTCCCTGCTGCTC
CATCCCCAAAGGTGCTCCCTCTGTGAGTTATTTTGCCTTTAACTGTGATAAAGTTCATCTGTAATGCGTGATAATCTGCATCTC
CTAGGTAGATTCC
>SsLRR-RLK146
ACTCAGGGTTTTATCCCCACTTGTTGCAGGTTGTGGTGTATCTTGGCGACTGCAAGAGTTGGTTGGCTCCTGCCTTGTGGCG
GAGTGTGATCACGGGCATGATCCTTCTGAGTTATCTTCTCTCTAATGCTTTTGTTGGAAGTCGACCTTGGTACTGGCATGTATT
TAAAATCAAAGTAATGTTAGTTTCAAATTACTTTGCTTCCGCTACTTTTCAACTTAAGTTGTAATAACTTTATTGAACTCCGAT
GCATATTTAATGACTTGTGAAATGGTTGTAACATGTGACTTGTTATGTTGAATCACTACGATCTTGGCTTGTATGTTGGTTGGT

TTGAAATCCTGTGTGATTTCATTGGACTACCGGAGTTATATGGGCTCAAGTGCGAGAATTTGATCACTTCGGTGATTGTTTTTG
TACTTGTGCTCTTATAATTTGGACGGTTCTGTTACATGGTGCCCAGGCCACGCGTCCAGGCGGTCGCGGCGACGGCATGGCT
TCGGGCCTGAGGCCCTCACCTTCGGACGACGAGCGACGGGGCTAGGTCGGCGTGGTGGAGGACGCGGCGGCGGAGCTGTG
GACGCGGTGGCTTGGCTGGGGAAGCATCGGCGGCGACGCTACGTGGTCGCGACGGTGAAGGCGACGGCGCTGCTCGGCTG
CAGACGCGAGGGTGAGCGAAGGAGGCGATGAGGCGAATATTGTGTAGTCCGATTTGGATTTGTAATTCTGTTCGGGGTAGGA
GACGTGAGTGAGGATGAATACGATACCTCATCACGTAACCTGGATACATGGCTGGCAGACAGAAAATTAGAGGGCGGAAAC
AAATTTGAGTGCAGAAATTTTCTCTTTTAAATATTAGGTATAGATATAGATATCTAGATATTTAGTCATGAAAAATAAATATGAAT
TTTATTATAATTGAATTACGGGTGGTACATCATCATGATGAATAGTTATATTTTTAATTGACGTATTAAATTATATCGTTATTGCTA
TTTTTGTATGATTTTTCAATCACACATCAATCACAATCATAAGCACGTGCGTGTCGCACGTGCGATATAAATATATTTATACTTT
CCCCCAATCAATAAATCCGCATGAGTCAGCAAATTGAAAGGATTAACAGGTCGTTCATGGTTCTACTGTCCAAGAGACCAGG
AGCTGTCTCAGTTGATTCCTTCCGGCCGATTTGCCTTCAGAACTGCAGTGTCCAGATTCTATCCAAAAGTTTAACCTGCAGAT
TACAGAAAGAGATTGGGAATTTAATTGACCTGAAGCATACAGGATTTCTGCAGGGCCGGTCCATCTCTGAATCTTTTGTATAT
GCAGCTGAGATTGTTCAAACCTGATATAAGAGGAAGATTCCAACGATTGTGCTTAAGCTGGATTTTGCAAAAGCATTCGACA
CTGTCAACTGGCTCGGTTCGGACAGTATTCTAGCTGCTCGAGGCTTCAACTCCAAATGGAGAAGATGGATGCGGTGTGTTCT
CAACTCCTCGAGATCTGCTGTTCTTATTAATGGAGTCCCAGGGCCATGGATTTCGTGCAAACGTGGATTAAGGCAGGGCGAC
CCCATCTAGCCATACCTTTTTCTTCTATACTAGTTGTTGACACCCTTCAAATTTCTAAAAATTTTACATTGTCACATCAAATCTT
TAGACGCATGCATGAAACATTAAATATAAGTAAAAAAATAACTAATTGCACAGTTTGGTTGTAAATCACGAGACGAATCTTTC
GAACCTAATTAGTCCATGATTGGACATTAATTGTCAAATACAAACAAAAGTATTACTGTGCTTCCAAAAAATTTACATGTGCA
ATTAAACACAGCTACAGGTGCAACTAAACGCAGCCTAAAAGACTTTCAAGCACCATCAGACATCCAATTGACCAGGCGTCA
GTTGCTGTGACCCTGCAGTACGCGGATGATACCTTAATTGTCCTATAATAGGTGAGGCTAGTGGAGTCTCTAAGCTCAAGGAA
GTACGGGCAT
>SsLRR-RLK187
AGTACTACTTTCGTCTTATTTGGAAAATATTATCCAATCGTAGATCAACTAGGCTCAAAAGATTTATCTCGTGATTTTCAACTA
AACTGTGTAATTAGTTATTTTTTTTACCTATATTTACTACTTTATGTATGAGTCCAAAGATTGATGTGATGAAGAGAGAGTGAA
AAAATTTTAACTTTTGGGTGCATCTAAACAAGAGCTTAATGGCGTAGCAGTAGCTTACATCCCTTCATAGGATTGTGCTGCTAT
ATTTAGTAGTGTTGGCATTATTCACCAAGGGGATGCTATAATAATTTTCACTAGAGAAGAGGGCGACTCGGGCGGCCCCTCC
CCCACGCCGCCACCGCCTGGCTGCCGCCAGAGCTCGACGCCGGAGGCCGGCAGAGAGCAAGGACGGCGGCGGCTGGGCT
TTTTCTTCTCGTCCCTCCCTCTCTCCCTCCCTCCCTCTCTCTCCCAGAGGAGCTCCCGTGCAGACTGTCGGCGGCCGGTTCCC
AGCAGCGGCGAGCGCGGATCCGATCCCATTCAGGCTTCATCATCTGTATCAAGCATCTTCACATTTGTGTCCCAGAAAAGTGT
TCCATTTCACCCGTATGTCCCCTCCTTGAGTTTTGTGGATTTTTTAATGGATTGACTAACTTGTGTAGGCCAATTGATGACTCT
GGAAGACCTAGCCCAGATTATAGTCCAACATCTCCAAATTTCAGGTAAACTGACACGGCATCAGGAATACATTACCTGCCTGT
CAGGCATCATTAATCACATATTTTTCATTTAACATTGTGCACAGCCCTAGCAGAAGTGACTCCCCCACTGCGGCTAACTACTC
CCCATCCTCCACTGGCCAAGCCATTGACAAGGACGGCGAGATCAGCCAGTGAAAATACGAGAAAAGATTGATGATGGCTGC
AGAGGAAGAAATGAATCCATTGCAATATTCCATTTCTTTCTGCTTTTGTACAGTTGAGCGCATGATTTAGACGAATGGCTGGT
TCTGAACTCTTGTAGCAAATGTAATGATCAAACGGTGTTTTGCAGGAATTCCAAATTTTAATGGCTAAACTGTGTCTGTAAGT
GATTTGAAGTAAATATGGCCTTGTGGAAAGCGTCTGAAATTCATTGGGAAATATCTGTTACATATATATGCCTTTTTCTGCTTTC
ATTGTATATTTATTTAGGTACCATAATGAGAAAAATAAAATCACCGCATAACGCCACAACTTCCTGACGATAATCAGAGTTCAT
ATGTTTCGAAGAGCTTCACAAGCAGAGAATTGCTGAATTGAATGTCAGAAAATTCTATTAGGTCAGTACCAATTTCAGATGA
GTTATTTATATTTGTTATTATGGTATATTATCCTAGAATACAACATGTGTTCATTATTTCCCTCCATTATGCATAGAAGTTTCAAC
AGTTTGCATCTTTGCACTCTGCAGGAGTCCTCTAACTGATGGATCAATATGGGCACCTGGCAGTAAGGGAAACAAATACCCC
ATCTCACAATTGAACAGATATTCACGCTTATATCCTGCATTGCCCCAATTTGTATTCGGAAGATCATAATTTGGTAACACCTTG
AAAATGGATGTGAGCTTAGAAATAGGGCAAATACTTTTGAAGCCTAAATGTAAAATTAGGACTGTTTCAACTGTATCATGTGC
TAAATTGAGTTGTAACATATGAATGGACTCTTGCTTTGTAACTGAATGCCAGAGCTCCTGGCTTTCCATTTTAACCTTTTCTTT
CATGTGTTAACGACTCTTGGCGTTGTGTCCAAAATTCAACCCTTGTTGTTGGGCTACAATTACATGCAATGTTGATATGACATT
TCAGTTTACATATGTTGTTGGGTTGAATGCGAGGATCCTGGAACTCTAAAAGAGAAGGTTAGTGGTCTACATATGCATATCTG
CAGCAAAATGATGCAGACTCAGAATGTCGAACTGTGAATTTGCATGAGCAAATAAATATGCATGTCTAAACATATGCAGGATG
GA
>SsLRR-RLK423
GTTTTGATGTGATCGAACGTTGTTCACCAATAATGACTGGTGACCTGTGCGCCGCTGTGCTACTATGCTACTGATGGAAAGTG
TATTATGCAATAGCTCTACTAGTTCAATGCCAAACATCTCGCTCCGGCCTAAAAAGAGCAGAAACGGGCCCATTTTATGAAGA
GTCTGACGGGTGGGTCCGCCGTGGGAGAACGGACGAGAGAGTAGTGATCGCATGGCGCGGCGCCGGCTGCTAACCTAGGT
ATGCATGCTCGAGCCGAATAATATTATCCCGGACACGATCCCACACCACAAGCATCCTGTTTTGCCTTGTCATTAGTGCGGTTT
TCACGTGTTTGTCCCTGAGATATTCATTCACCATTTCCATCTTCTCTCCCTCCGTAACAGCTGGTAAATCCGTAGTAGCTGTATT
TTCCGTGATGCCGTAGCAAGATATGCTAGTCACACGAGAGTCGCCGCTATACTCCCTCCTTCCTGATTCTAACCATTCTTGGA
CGGAATAAGAGAGAGAGTGCAACTAATCTATTGAAATCCGATTCTATTTCATATGATTAAAGGATGGACTCTTAGCTTCCTTGTAC
AAAATATGACAAGTTTTCTTGTTCTCTGGATGCACTATATCTCGAGACGGAGAGAGTAGTAGACTATAGGGCTGTTTGGTAGA
ACTCTTCGCAGCGGATTCTTCTTAACAATCATTTCTCTTCAGCTACAATTGAACACAGATTCTAGTGGGAATAGACATAAATC
GTTTATTCATTTACGCTAGAGCTCGAAGCTAATGAGCTTCTCTTTCTGATTCTTGAGCTTCACTTTCGATGGTGTGATTTTGGT
GGGGAAGCAGGATGAGGAGCCGATAAGAATTGATTCTAGTGGTGATTGAACGGATAAAGGTGGATATGAGTGGAGAGTGGG
TTTTTAGCTCTTACCTAGGGGTATAAATGAAGAAGTAATTCTCATAAGCTCTTCACCATAATCCAGTTATATTTTAAGCATTTGT
CAGAGATCCACATAATCCACTCCGAAGAAGTTAGGAACATAGCTCTGTCAAAACAAGCCTAAACAACATGTTCCTTACTGCT
CTCTATTGAATATTCACCAAAATTGCTCTAAAATCATTTCTCATCCATTTTCCACTGTGTGTCCCAACCTCAATCGCTCTACCA
AAATTCCTCTAAGATCGGTAGTAGCAGTCGGCACTTGCAATTTACATGTCTTTTCGTGTTGAAACGGATGCACCAGATAATGC
AGCACTCCAGCCTTTTTCCTCCTGTTTTCGGTTACGAAAAGTGGCTTTCTAGGCCACAAGAAAAAGCGAAAATCACTCAACT
CACGAGGAAATGCAAACGCCTAGTATGGTGGAGTGAAGAAATTAGTTAGGGTGTAAATGATGCACAGATGAATCCAAGCAA
CAAGTGACCAAAACTGCAATTAAACAAGTAAAGCTCACTCAGCAACAGTGACACTTCCAGCCAGCCCAGCCGGACCTACG
TTGAGAGGACCTGTCATAGCCACCCATTAAATACTGCTCCATCTCCCAAGAACCGCAGATAAGACCAAAACGCCCGGGGTTT
AACGCAGCCCAAATCAAGGGTCCCAGCAACCACTTTGTACTAGTACTCCTACTACTCCCTCCATAATCCATCTCCTTGCTGGC
CACGCGGCGTAGCGTAACTCAAGTGAGGAAGAGGGAGGAGACGGAGCTGGAGAGAAACGCTCCATATGCTTGTTGGGTGC
GGCACAAGCAACGGCAGCTGCTGAAAGCGTGCGACCGAGCGAACCTCGCCACTGCCCCTCACGCGCCGCGCCCCCACATG
CCTCGGCGACCATCCCGTCCTGCTCCTCCTTGCTCTTCCCAGTAGCAAGCGACCGAACGCGCCGGGGGCGCATGCCGTGAC
CTCGCCGTGCTGGGCGGCCGCGCTCTCGGTTGGAGGAAAAAAGTCGTCGCCTTTTCCGGTTCCCGCGGTGGGTTCTTGATC
GAGGCGGTTGCCGATCGATAATA
>SsLRR-RLK432
GATGGCCGTACTATCAAGAGGGGCAACTTGTTTGTATATCGGTCATCTAGTTCCACTTGAGCGATCTAACCTTGCATACGTGTT
AGGATCGAGTGGCGAGGCAAGTTGAGTGCCTAACTCCTTCGAGAAAATGATTGTGAAAATACTAACACACATGCACAAGGT
GGTGATCTCTTGTTGGTGTTGGCACATTTGCGAAGGAGGTGGAGTTTGAAGGGTAAAGAGGGGTTTGGGGTTTCTCTCTCCC
TCTCGCCGAGCTTGCGAGGTGGGATTCGGCGCTTTCGTGAAAATGAAACGCTTATTTTTCACTACGCCGGAGGTGAAGAAAT
AGAGGCGAATGGAGGTCACTGGGGTGACCGGACGCCGGACCAGACGCGGTCTCTGCACGACCGGCGCGTCCGGTCAGTTC
AACGCAGCAGTCAGGTGCGCGGGCGCTAGGGTTTGCAGCCGGCGCGTCCGGTCCCTCGCGGAACCGGCGCGTCCGATCAG

ACACCCATTGGTGCAGTGCGTGACAAAGAGCTGATTGGACGCGCAGCACATCCGGTCACGATGGGAGCTCTCTGGACCGCG
ATCGGACGCTGGCACTGTCGCGTCCGGTCATTGCGTGGAGCGTGTCCGGTTATGTCGTCGCGCGCGTGGGTGCTGTGCGCAC
CTGTGCGTCCGGTCGGTTGAGCGGCGCGTCCGGTCGTCGCGTTTGACCATTGGCGCGCAGTGACCGTTGAGATCGGGCGTT
CCATGTTGAATGTGGAGGAACACGTGGACGGTGCATGGTGACCGGACGCGGTGCAGCGCGTCCGGTCAGTTCGACCGGCG
CATCCGGTCGTCTCGAAATTTGCCCAGTGAAGGGGTAACGGCTCTATTTGACCGTGGATGTAAGGTCCAAATGGCTAGAGGG
TGGTGAATAGCCTATTTAAAAATCTACAAGCTTCACTAAACAACCACGTTAGTATAAAAGATGGCTAAGCTAATTCTAGCACT
AGCTCAACTACACTATGCAAGCCACCTATACAATACTAGTGAAGCAAGGCTATGATCTCTAATACACACCAACAAGACTAAGT
CACTAAGCTACTTACTCATATGTGATAAAAGCTACACTAAGCAAACTACACTAGGACTAGTACAAGAAAGAAAAGAGCAAGT
GACTTGATTCTTATACCAACGATGTAGAGAAGTAATGCACTCAAAGCATCATGGAGNNNNNNNNNNNNNNNNNNNNNNN
NNNNNNNNNNNNNNNNNNNNNNNNNNNNNNNNNNNNNNNNNNNNNNNNNNNNNNNNNNNNNNNNNNNNNNN
GTTTAGTTGGAAATCACGAGATGAATCTTTTGAGTCTAGTTGGTCCACGGTTGGATAATATTTGTCAAATAAGACGAAAGTGG
TACTATTCATCGGGTTCACTTTTTTTTGCAATCTAAACAAGCCCTAAACAAGTAAAGCTCACTCAGCAACAGCGACACTTCC
AGCCAGCCCAGCCGGACCTACGTTGAGAGGACCTGTCATAGCCACCCATTAAATACTGCTCCATCTCCCAAGAACCGCAGAT
AAGACCAAAACGCCCGGGGTTTAACGCAGCCCAAATCAAGGGTTTAAGCAACCACTTTGTACTAGTACTCCTACTACTCCCT
CCATAATCCATCTCCTTGCTGGCCAGGCGGCGTAGCGTAACTCAAGTGAGGAAGAGGGAGGAGACGGAGCTGGAGAGAAA
CGCTCCATATGCTTGTTGGGTGCGGCACAAGCAACGGCAGCTGCTGAAAGCGTGCGACCGAGCGAACCTCGCCACTGCCCC
TCACGCGCCGCACCCCCACATGCCTCGGCGACCATCCCGTCCTGCTCCTCCTTGCTCTTCCCAGTAGCAAGCGACCGAACGC
GCCGGGGGCGCATGCCGTGACCTCGCCGTGCCGGGCGGCCGCGCTCTCGGTTGGAGGAAAAAAGTCGTCGCCTTTTCCGGT
TCCCGCGGTGGGTTCTTGATCGAGGCGGTTGTCGATCGATAATAATGG
>SsLRR-RLK397
ACCGTCCTTCTGTCGAATCGGTCGAAGACGCAGGAGTCACCGAGGATGAGCTTGACACCCTCCTCGTTCCAGGCCTCCAGC
GGGACATGCTCCATGACGATCTTCACATGGTAGCACCAGGTGTGCTCAGCACCCCCCCCCAGCCGAAAGCCACGGCCGGAT
GGGGAAAATGGAGCCGCCAATCTCGATGTATCCCTGATACAGGGCTCTGTCCTTCTCCGGCGGAGATCCAAACTCGGCCACG
AACTCTGAAGCGCTGTACTTGGTGACGCGGACGGGGCGAGACAGATGCAGCTGCTTGGCCAGGGTATGTGCCACCTCTGGC
GCATTGGTGCGGTATCCCTCCGGCGGCGACGAGATGACAGCAGCGTGCGCCCGTAGCTTGTTTGCCTCACGGAACATGGTCT
CCGTGGACATGATGTTGATGCAGCGGCGCTCGGGGCGCTGATGGGCACGTCTAGGAACGTACGCCATGGCGGGCTTGGCGT
CGGCGGAATGGGCGCGATGCGGGAGCGGAGGGAAGACGATGCGGCTGTGGATGCTTTCGGGTGGGAAGGTGAGGCGGGC
GCGGAGTTGCTCGGATATGAGGCGAGGGCAGCGAGCTGGGCAAGAGCGGGAGAGGTGCCCCACTCGACGGCAGGAGATGC
AACGAACTGGGTCACGGCACTTGGCGACTAGGTGGTCGGAGGCGAGGCACCGAAAACATCTCCCGGCGAAGCGCTTCTTA
AAGGCAGCCAGGGCCGCGGGGGAAGCTCCTCTGGGCGCCGGCTTGGGCGCACCGCTGGGCACCGGCTTGTCCAGAAGGG
GTACGGCAGGGGCACGCTTCCGGCGTCGTGCCTTCGCGTCTTGCCACTTTGGTGAAAGCGGCTCCTCCTGCGCCGCATTAAC
GCCGGAGCACTCGCCCATCTCCCCTCTTGAATCAGAGAAGCCGTAAAGGCCCTTCCCGCAGCGGGTGGCCGGCGTCCAGGA
GCCGAGAGCCAAGGCCTTGCCAGATCCATCTGCAGAAATTGCATTTAGATCCACGCGCTTCCAAGCCGTCTTCAGGGGTCGC
CGTGGTGACTCTGCCGCGCGGGCAGGCTGGACCAGCACGGATTTGAGCCTGGTCGGCGCTGCACCTTCAGCACGCGTAGGC
TCTTGCTTCTTCAGCACGGGGGGCGGCCGGATCGAGGCATCCTCCGACGCCGCGACGGCAGATCCGCCGGGTGCGGCCTCA
GATCCGCCAGTGGCCTCAACCGGTGCTAGCTCCGGAAGGGGGGCGTTGGGGGCCGGGGAGCTGGCTCGTAGCATCAGATTC
CCTGCATCTTCGCCACCTTGAGCTTTGGCGCTGGGAGGCGAGGCGACGTAAATGTGGACAGGGCTCTCGTCGCCCCAGTCC
ACGAGTTCTTCGTCGCCCGCGCCGACCAGGGGCTCCAGATCTTGCACAGACGAAGCAGGCGGGGAGCTGAGTGGAGGATC
TGCTGCAGGGGAGGTGGGCCGAGCTAGGGTGGACGGCGGCGCCCGGAGGGACGCCGGCGGTGTGGGGGGTGGGGGCTCT
CGCTCACCAAGGCTCTCCATCCGTCCTATAGGCCAATACTCAATTCTCCCGAACAAAAAGCCCGAACCACTTTGTACTAGTAC
TCCTACTACTCCCTCCATAATCCATCTCCTTGCTGGCCAGGCGGCGTAGCGTAACTCAAGTGAGGAAGAGGGAGGAGACGG
AGCTGGAGAGAAACGCTCCATATGCTTGTTGGGTGCGGCACAAGCAACGGCAGCTGCTGAAAGCGTGCGACCGAGCGAAC
CTCGCCACTGCCCCTCACGCGCCGCACCCCCACATGCCTCGGCGACCATCCCGTCCTGCTCCTCCTTGCTCTTCCCAGTAGC
AAGCAACCGAACGCGCCGGGGGGCGCATGCCGTGACCTCGCCGTGCCGGGCGGCCGCGCTCTCGGTTGGAGGAAAAAAGT
CGTCGCCTTTTCCGGTTCCCGCGGTGGGTTCTTGATCGAGGCGGTTGTCGATCGATAATA
>SsLRR-RLK411
TTCTGCACTGTTTCCAAAATCTGAATGTGTTGGAAACAGTGACGTGAAATCCCATTGAGACTGGCCTTATCCCGGACACGAT
CCCACACCACAAGCCTCCTGTTTTGCCTTGTCATTAGTGCGGTTTCACGTGTTTGTCCCTGTGATCTTCATTCACCATTTCCAT
CATCTCTCCTTCCGTAACAGCTGGTAAATCCGTAGTAGCTGTAGTTTCCTTGCTGCTGTAGCAAGATATATACTAGTCACACGA
GAGTCGCCGCTATAGGGACTATTTGGTTCGCCCTTATAAACTTTAACAGATGTTGATCATAGGGTTGACTATAGGGTATTCGGA
TATTAATAATAGGCTAATTATGTTTAATAGATTTGTCTCGTAAATTAATCTTAATCTGTGCAATTAGTTTCGTAATTAGTCTACGT
TTAACACTCTATGCATGTGTTCAAACATTCGATGTGATAGCTGTTAAAAAAACAAGGGGAACCAAACACCCCCCGTACTCCT
TCCATCCTGATTCTAACCATTCTTTGACAGAACAAGAGAGAGTGCAACTAATCTATTGAAATCCGATTCTATTTTACATGATTA
AAAGATGGACTCTTAGCTTCCTTATACAAAATATGATAAGTTTTATTGTTCTCCGAATGCAATATATCTCAAGACAGAGAGGGC
AGACTATAGGACCGTTTGGTAGAACTCCTCGCAACGGATTCTTCTTAACAATCATTTTTCTCTTCGGCTGCAATTGAACACAG
ACTCTAGTGGGAATAGACATAAATCGTTTATTCATTTAAACTAGAGCTCGAAGCTAAGGAGCTTCTCTTTCTGATTCTTGAGC
TTCACTTTTGATGGAGGATAAGGAGCTGATAAGAATTGATTCTAGTGGTGATTGAATGGATAAATGTGGATATGAGTGGAAAG
TGGGTATTTAGCTCCTACCTCGTGGTATAAATGAAAAAGTAATTCTCGTGAGCTCTTTACCATAATCCAGTTGCATTTTAAGCA
TTTGTTAGAGATCCACATGATCCACTCCGAAAAAGTTAGGAACATAGCTCTGTCAAAACAAGCCTAAACAACATGTTCCTTA
CTGCTCTCTATTGAATATTCACCAAAATTGCTCTAAAATCATTTCTCATCCATTTTCCACTGTGTGTCGCAACCTCAATCGCTCT
ACCAAAATTGCTCTAAGATCGGTAGTAGCAGTCGTCACTTGCAATTTCCATGTCTTTTCGTTGTTGAAACGGATGCACCAGAT
AATGCAGCACTCCAGCCTTTTTACTCCTGTTTTTCGGTTACGAAAAGTGGCTTTCTAGCCACAAGAAAAAGCGAAAATCACT
CAACTCACGAGGAAATGCAAACGCCTAGTATGGTGGAGTGAAGAAATTAGTTAGGGTGTAAATGATGCACAGATGAATCCA
AGCAACAAGTGACCAAAACTGCAATTAAACAAGTAAAGCTCACTCAGCAACAGTGACACTTCCAGCCAGCCCAGCCGGAC
CTACGTTGAGAGGACCTGTCATAGCCACTCATTAAATACTGCTCCATCTCCCAAGAACCGCAGATAAGACCAAAACGCCCGG
GGTTTAACGCAGCCCAAATCAAGGGGTTAAGCAACCACTTTGTACTAGTACTCCCTCCATAATCCATCTCCGTGCTGGCCAG
GCGGCGTAGCGTAACTCAAGTGAGGAAGAGGGAGGAGACGGAGCTGGAGAGAAACGCTCCATATGCTTGTTGGGTGCGGC
ACAAGCAACGGCAGCTGCTGAAAGCGTGCGACCGAGCGAACCTCGCCACTGCCCCTCACGCGCCGCACCCCCACATGCCT
CGGCGACCATCCCGTCCTGCTCCTCCTTGCTCTTCCCAGTAGCAAGCGACCGAACGCGCCGGGGGCGCATGCCGTGACCTC
GCCGTGCCGGGCGGCCGCGCTCTCGGTTGGAGGAAAAAAGTCGTCGCCTTTTCCGGTTCCCGCGGTGGGTTCTTGATCGAG
GCGGTTGTCGATCGATAA
>SsLRR-RLK338
CCCTAACATGTTTGCCGTCGCTCATGTGATCGCATTTTTAACTTAATAAGGTTTCTATTTTTCATGCACTTGTGGTCTGTTTGTT
GGATGACATTGACTTAGTACATACTACTACTTTTTTTTTTTTTGGAAAAACTAGCAGGAGCACTGCTATATCATATAAGACGGAA
GCAAAACCAGTTACAACAATTTGGTTCTTACATTATTAAGACCACCCAAGCTCACAAACAGGGAGATAAGAAAGAGGATACT
CATGGTGGTAACTCTAATTTCCCCAAAGCTTTACTGAAGAGACCCATCTCTTCCTTGATCAGCCCCAGTACTTGGGCCGGCGT
TCTTGTTGTCCCCTCGAAAGGTCTCCGGTTGCGCTCTTTCCAGATATTCCAAGTCGAGTACATCAAGATGGCACATACTACTA
CTAACGCAAAGCTAATTTGAGATAATAAACTACAACGTATAGCAAAAAAGGAACCTTGGTTTTTTCAGTTTAGGTGACAAAC
GTATAAATTCAACTAGAACATATAGCAAAACGTTATCTTATTAACTCAGTAGTTCTAAGTTCTTTTTTTTTGGAAGTAAAATGG
CAACTAGAACATAAAAGTTTCAGATTTTCACTAGCACCTAATCCTTTTTCTGTTACTTTCCAGCGGGGTTGCCACCCTTCTGC

ACGGTTAAGTTGGTAGTAATATTTTATTTTATCTTTCTATAGGTATTATACTAACCTACACTCCTTTACTGCATGTGCCCCTCCCC
TAAGATTCCTGTCTTATTCTACCTCGCTCATTTTTCTTTAACCCGGACACTCCTCTGATGCCTATGATTGAGTTCACAGACAGT
AATAAGAGTTTGCCGGTCCTTGTATTTTATTGGACTCCTTTCGGTTTCTCTGGAAAGTTAGGCATGTGATGCGAACATGCATG
CTCTCTGAACTTGGAAATTAGTACCACACAGAAGGAGAAGGTGCAGAAACAGTAAATTGAGCATATGAATTTTTTTATTAATT
TGCATATGGTTGATGTACCACTACCACCCTGTCTTGCAGTGTTTTAGTTCTCATCCATGTGAATTACTAATAGAGAGAAGCTTA
GTGTGTGTGTGGCTAGTGGCTAGACTGCTAGACCTCCCAAAAGTTTAATCCTCTGTCCATGTGCAAGAGCACAATTCTCTTGT
CGGATGATTGCAGTAATACTACTCAGTAGTTGAATTATATGACAGAAACACTTAAATTTTTGAGTATAACATGCTGCATGAGAT
TTTCTTAACACAGATAGGAACATTGCCTCAACGCAATATACGCACAGTACAACTACCTGTGGCAGACTGCTACCGTAAGTTTG
TGCAGATCTGAGACAAGTGCAGGGAAAGATGAACGCAGGTTAGATCTAGAGGGATTGATTTTATACAGGAAATGTTAGCCA
GATCATCGGTGTCAGAGAGGTTGCATTACCAAGATCACAGAGAGCATGTTCACCTGCAACACTACTGCATGATTTTTCTCTCA
TTGCAGTTGGCACTTATGTATCTTCTTTTCCATCGTCAGAAAGTAATACGCCGGCACAACAGTGAAGTTCAAGAAAAACATG
CATTGAGGTGTAGATTTGATAAAGCTTGAGGGATGACGATCCAGCTTTGACTTTGATAAAAAAAAAAAAGAAGTGTGATTTGG
TGTGGATATCATTTCTACTGCTGTCACAGTGCAACAAATTTATATTCTTTTTAAAGTTTTTTTTTCACAGAAGCAGCTCTGAA
TCACTGCAGATGGACAGACCATCAGAAACATTTAGAAGGTAAACACCCAGAGCCCAGAGGAGAGCATGTGAATTAAGTAGT
AAACAAACACCAGCAGCAGCCGCTGTCAACTTCTGAGTTCTGACCACCCTGCATTAGTGCAGTACAAAAGAACAAACTTTC
ACAGGCGCTAGAAACACTATACTTAACAAAGGATAAAGTTCAGAATTTCAGATTACCCAGGGAATTACGATTAGCATTTCCCA
GAACCCA
>SsLRR-RLK320
TAATATTTTATTTTATCTTTCTATAGGTATTATACTAACCTACACTCCTTTACTGCATGTGCCCCTCCCCTAAGATTCCTATCTTAT
TCTACCTCACTTGCTCATTTTTCTTTAACCCGGACACTCCTCTGATGCCTATGATTGAGTTCACAGACAGTAATAAGAGTTTGC
CGGTCCTTGTATTTTATTGGACTCCTTTCGGTTTCTCTGGAAAGTTAGGCATGTGATGCGAACATGCATGCTCTCTGAACTTTG
AAATTAGTACCATACAGAAGGAGAAGGTGCAGAAACAGTAAATTGAGAATATGAATTTTTCTATGAATTTGCGTATGGTTGAT
GTACCACTACCACCCTGTCTTGCAGTGTTTTAGTTCTCATCCATGTGAATTACTAATAGAGAGAAGCTTTGTGTGGCTAGTGG
CTAGACTGCTAGACCTCCCAAAAGTTTAATCCTCTGTCCATGTGCAAGAGCACAATTCTCTTGTCGGATGATTACAGTAATAC
TACTCAGTAGTAGAATTATATGACAGAAACACTTAAATTTTTGTAGTATAACATGCTGCATGAGATTTTCTTAACACAGTTAGG
AACATTGCCTCAACGCAATATATGCACTGTACAACTACCTGTGGCAGACTGCTACCGTAAGTTTGTGCAGATCTGAGACAAG
TGCAGGCAAAGATGAACGCAGGTTAGATCTAGAGGGATTGATTTTATACAGGAAATGTTAGCCAGATCATCGGTGTCAGAGA
GGTGGTATTACCAAGATCACAGAGAGCATGTTCACCTGCAACACTACTGCATGATTTTTCTCTCATTGCAGTTGACATTTATGT
ATCTTCTTTTCCATCGTCGGAAAGTAATACGCCGGCACAACAGTGAAGTTCAAGAAAAACATGCATTGAGGTGTAGATTTGA
TAAAGCTTGAGGGATGATGATCCAGCTTTGACTTCGATAAAAAAAAAAGAAGTGTGATTTGGTGTGGATATCATTTCTACTGCT
CTCACAGTGCAACAAATTTATATTCTTTTTAAAGTTTTTTTTTCACAGAAGCAGCTCTGAGTCACTGCAGATGGACAGACCAT
CAGAAACATTTAGAAGGTAAACACCCAGAGCCCAGAGGAGAGCATGTGAATTAAGTAGTAAACAAACACCAGCAGCAGCC
GCTGTTAACTTCTGAGTTCTGACCACCCTGTATCAGTGCAGTACAAAAGAACAAACTTTCACAGGCGCTAGAAACACTGTAC
TTAACAAAGGATAAAGTTCAGAATTTCAGATTACCCAGGGAATTACGATTAGCATTTCCTAGAACCCGAGGAGGAAGAAATG
TTCTCACTCACCTCCTCAACAGAGCACTGGGCACAGCAGCAAGCAATTAACAATTCAGGATCACCCAATGGCAATAATGAGT
ACATCAACTAGGGATAAACATATCCAAATGATGGCATCAGCTGGGCTCCATACATGCCATCTTCCTTCCATCTTCTTTCTTCCC
CCACACTTCAATTGAAAATTGCCAATATTCCTTTCTGATCTGAAACTTCAAGAACCAAATTTTGGACATGTATTCCAAGTAGA
AGTAGAAGAACCACTCACTTACACACATCACTGTATCTAGGAAATGCGAGACCATTTGCGCTATCGTCGCCGTTAAAACGGC
TTCAAGATTTGTGCCAGAGTGAAACCACACTCGCTGATTTTGTGTGTCAGCAACCAACCACAAATCTCAGCGGTGTGTGCA
GTAGGATCTATGGCTAAAGAGTAGAGAGTAGAGAGTGGAGATAGTACAAGTCACTGTACCTAATCAAATCTGTTGTTTATCTA
AAAGAATATCCTGCAAAAGCTAAGCTGTTGTTTCATTATTTCATTTGGATCCAGATCTCTAAACCCTTTGCGTGCTATAAACAA
ATCCTGGGGTCTCCCGCTCTACAATCCCAAGGCAACGAGGAGGTGCCAGTACCACCACTCACCCCGCGGCCTCGGCCACTC
TCTGCTCCACCA
>SsLRR-RLK307
GCACGGTTAAGTTGCTAGTAATATTTTATTTTTCTATCTATAGGTATTATACTAACCTACACTCCTTTACTGCATGTGCCCCTCCC
CTAAGATTCTTATCTTATTCTACCTCGCTCATTTTTCTTTAACCCGGACAGAGGACACTCCTCTGATGCCTATGATTGAGTTCA
CAGACAGTAATAAGAGTTTACCGGTCCTTGTATTCTATTGGGCTCCTTTCGGTTTCTCTGGAAAGTTAGGCATGTGATGCGAA
CATGCATGCTCTCTGAACTTTGGAAAATAGTACCACACAGAAGGTGCAGAAACAGTAAATTGAGCATATGAAATGAATTTGC
ATATGGTTTATGTACCACCCTGTCTTGCATTTTTTTAGTTCTCATTCATGTGAATTACTACTAGAGAGAAGCTTAGTGTTGTGTG
GCTAGTGGCTAGACTATAGACCTCCCAAAAGTTTAATCCTCTGTCCAAGATCACAATTCTCTTGTCGGATGATTACAGTAATAC
TACTCGAATTATATGACAGAAACACTTAAAATTTTGTAGTATAACATGCTGAATGAAATTATCTTATCACAGATAGGAACACTG
CCTCAAGGCAATATATGTAGTTATGTACAGTACAACTACCTATGGCAAACTGCTACCGTAAGTTTGTGCAGATCTGAGACAAG
TGCAGGGAAAGATGAACGCAGGTTAGATCTAAGAGGGATTGATTTTATACAGGAAATGTTAGCCAGATCATCGGTGTCAGAG
AGGTTGCATTACCAAGACCACAGAGAGCATGTTCACCTGCAACACTACCGCATGATTTTTCTCTCATTGCAGTTGGCACTTAT
GTATCTTCTTTTCCATCGTCAGAAAGTAATACGCCGGCACAACAGTGAAGTTCAAGAAAAACATGCATTGAGGTGTAGATTT
GATAAAGCTTGAGGGATGATGATCCAGCTTTGACTTCGATAAAAAAAAAAAGTGTGATTTGGTGTGGATATCATTTCTACTGCT
CTCACAGTGCAACAAATTTATATTCTTTTTATTTATTTTTTTTCACAGAAGCAGCTCTGAATGACTGCAGATGGACAGACCATC
AGAAACATTTAGAAGGTAAACACCCAGAGCCCAGAGGAGAGCATGTGAATTAAGTAGTAAACAAACACCAGCAGCAGCCG
CTGTTAACTTCTGAGTTCTGACCACCCTGCATCAGTGCAGTACAAAAGAACAAACTTTCACAGGCGCTAGAAACACTGTACT
TAACAAAGGATAAAGTTCAGAATTTCAGATTACCCAGGGAATTACGATTAGCATTTCCCAGAACCCGAGGAGGAAGAAATGT
TCTCACTCACCTCCTCAACAAAGCACTGGGCACAGCAGCAAGCAATTAACAATTCAGGATCACCCAATGGCAATAATGAGTA
CATCAACTAGGGATAAACATATCCAAATGATGGCATCAGCTGGTCTCCATACATGCCATCTTCCTTCCATCTTCTTTCTTCCCC
CACACTTCAATTGAAAATTGCCAATATACCTTTCTGATCTGAAACTTCAAGAACCAAATTTTGGACATGTATTCCAAGTAGAA
GTAGAAGAACCACTCACTCAAACACATCACTGTATCTAGGAAATGCGAGACCATTTGCGCTATCGTCGCCGTTAAAACGGCT
TCAAGATTTGTGCCAGAGTGAAACCACACTCGCTGATTTTGTGTGTTAGCAACCAACCACAAATCTCAACGGTGTGTGCAGT
AGGATCTATGGCTAAAGACTAGAGAGTAGAGAGTGGAGATAGTACAAGTCACTGTACCTAATCAAATCTGTTGTTTATCTAAA
AGAATATCCTGCAAAAGCTAAGCTGTTGTTTCATTATTTCATTTGGATCCAGATCTCTAAACCCTCTGTGTGCTATAAACAAAT
CCTGGGGTCTCCCGCTCTACAATCCCAAGGCAACGAGGAGGTGCCAGTGCCACCACTCACCCCGCGGCCTCGGCCACTCTC
TGCTCCACCA
>SsLRR-RLK127
AATATTGGTATTACTACTAAAGAGAGTTGTTTTTACATAGTTGACTTGAAATCCAGAATTTCACTGATACATGCTAACAAGTCA
TAAGTAACATGACTAGTTCTAACAAAACAGGCAAAATAATTGGAGCCTCTTTGGTTGAAATTCAAATTTTGGAACTGGAGAG
CCATTCATCCCCAGTAAGATTCGGAGTGAACATTTCATCTGAATTGAATAAAAAAAAAAAGGTGGCGGATAATTGATCTGAAA
AAATGGAAACACAATGTACGAACAATAATGAAATAATTGTATCCACCAAGCAGTGGGCAAACATCCATTTCTCTTTTCAAGG
ATTGTGCATTGCCATTCGTGCTTTTAAAAGTAGTATAAGTTTGATGTGGAAATGTTGTTTTAGTTACCAAAATTAAAGGGATCT
ACCAGCCATGGTGATGATGCTAAGCTAAGATAGGTCCATGGATTTAACTATGAACCATAGGGTGGTTATCTTAGTATCTTATTC
CTCATTGTCCCTTCCACTCAAAGACATGAACATGTTCTCCAAAATTTTTATTTCTATCACCGCTCATCCAATCTTTTTCAGTTAC
TTTCCAGCGATTTGCCACCTATTCACATGGGTAAGTTGCTGAAAGTTTTTTTATGCCTTTGCATTGTACTAACCTTCACCCCCT
TCTCTTACCGCCATGTGCACCCTAAGATTCCTCTCTTATTCCCACTCTTACCCCTCTTGTTTAACTAGCAGGCCTCTCTCTGAT
GCCCATGACGAGTTCACACTTCAAAGGAAGCAATTAAGTTGCCAATTCTAGTATCATGGTGATCTCCTTTTTGCAGAAACTTG

ATCATGTGACTGAACATTCTGGGGCTCACACAGATTTACCCGTTACTGAGCTGCAGAAGACTAATTTAGGATCACTCATTGAT
GCGCACAAACTGAGAAGTACATACAAATTACAATGCCTGAAAAGAGGATGTAATAGTATTTTTGCAATTATTAGTAGCAAGTC
CAGAAAAAGATTAAGGTCTCAGGGACTGTAAGTATTGCAGTTGCAGGCAATGATGAATGCAGGTAGTGGTAAAGCAAATGTG
AACATTAAGACTTCAAATGTTGAATTAGCATCGAAAGATGCAATTTTTTACAGCTGTAGTAGACTTGCAGCAAGGAGGATAC
CTGCAGAGAAGTAGTTACTTGTAAAGAGGTAGTACCTGTAAAGCAGCACTGTCACAGGCTCACAGCAGGGTAACCGTAAAG
CAGCACTATCACAGCAGGCCTTCAGAAAACGTTTTAGAAGCTTACATATGCCGAAAGCCAGCAGCAACAGCCACTGTCAAC
TCCTGTACCAACGGTACAAAGGTTCAGGGATTAAGAATAACTGACAGTGCATGCAAAATGCTCTTCCATGGAACATTGGATA
AAAGTCCGATTACCGAGAGAATTAGCATTAATCTGTTTTCGCAGAACACGAGGAAGAGATGTTTTCACCTCCAAAGACAGCA
GCAGCAATTAACAATTCAGGCTGCAATGGCAGTAATAAGTACACCAACTGGCGATAAACATACGCAGATCATGACACCAACC
ACACCGCCTTCCTCTCATCTTCCCCGTCACTAGCACAAGTGCACAACTGCATCCAGTATCCATTTTCCTGATTAAATACTAAAT
CCAGTACAATTATTTTAAGTGTTCAGGCCAAGATCCAATCAAGCCTGCTCTTTAGTAGCAGCAGCACACTGTCACACACAAC
ACTGCAACAATCAATCGAAAATCTGTTCTTTTGTTTGGATTTTTTTGGAGGGGGCAAATTGCAGCAATTGTTCGGTGCTCATT
TGGATCCAGATCTCCTGTGCTTTCCCTGTATAAACAATTCATTGGGTCTCCCCCTCTACAATCCCAATGCTTGCGAGAGGCGC
CACCTCACAAAGCCAGCCATCCACCGCATTGCCGCCCCCCTCGCCTCTCCTCACGTCACCCGCCCGCCTCAGCCTCGCCACC
GAGGCGCGCCGCCAA
>SsLRR-RLK87
CAAAACAGGCAAAATAATTGGAGCCTCTTTGGTTGAAATTCAAATTTTGGAACTGGAGAGCCATTCATCCCCAGTAAGATTC
GGAGTGAACATTTCATCTGAATTGAATAAAAAAAAAGGTGGTGGATAATTGATCTGAAAAAATGGAAACACAATGTACGAA
CAATAATGAAATAATTGTATCCACCAAGCAGTGGGCAAACATCCATTTCTCTTTTCAAGGATTGTGCATTGCCATTCGGGCTTT
TAAAAGTAGTATAAGTTTGATGTGGAAATGTTGTTTTAGTTACCAAAATTAAAGGGATCTACCAGCCATGGTGATGATGCTAA
GCTAAGATAGGTCCATGGATTAACTATGAACCATAGGGTGGTTATCTTAGTATCTTATTCCTCATTGTCCCTTCCACTCAAAG
ACATGAACATGTTCTCCAAAAATTTATTTTTATCACCGCTCATCCAATCTTTTTCAGTTACTTTCCAGCGATTTGCCACCTATTC
ACATGGGTAAGTTGCTGAAAGTTTTTTTATGCCTTTGCATTGTACTAACCTTCACCCCCTTCTCTTACCGCCATGTGCACCCTA
AGATTCCTCTCTTATTCCCACTCTTACCCCTCTTGTTTAACTAGCAGGCCTCTCTCTGATGCCCATGACGAGTTCACACTTCAA
AGGAAGCAATTAAGTTGCCAATTCTAGTATCATGGTGATCTCCTTTTTTGCAGAAACTTGATCGTGTGACTGAACATTCTGGG
GCTCACACAGATTTACCCGTTACTGAGCTGCAGAAGACTAATTTAGGATCACTCATTGATGCGCACAAACTGAGAAGTACAT
ACAAATTACAATGCCTGAAAAGAGGATGTAATAGTATTTTTGCAATTATTAGTAGCAAGTCCAGAAAAAGATTAAGGTCTCAGG
GACTGTAAGTATTGCAGTTGCAGGCAATGATGAATGCAGGTAGTGGTAAAGCAAATGTGAACATTAAGGCAGTGTTTACTTC
CTTCAAAATTCCAAAAAATTTTACAAGATTTCCCGTCACATCGAATCTTTGGACGCATGCATGGAGTATTAAATGTAGGTAAAA
AAAATAACTAATTGCACAGTTTGTCTGTAAATGTCGAGACGAATCTTTTGAACCTAGTTAGTCCATGATTGGATAAATTTTACC
AAATAACAACGAAAAGTGCTACAGGAACTTTACACCCACTTTTACACCATCTAAACACTACCTAAGACTTCAAATGTTGAAT
TAGCATCGAAAGATGCGATTTTTTACAGCTGTAGTAGACTTGCAGCAAGGAGGATACCTGCAGAGAAGTAGTTACTTGTAAA
GAGGTAGTACCTGTAAAGCAGCACTGTCACAGGCTCACAGCAGGGTAACCGTAAAGCAGCACTATCACAGCAGGCCTTCAG
AAAACGTTTTAGAAGCTTACATATGCCGAAAGCCAGCAGCAACAGCCACTGTCAACTCCTGTACCAACGGTACAAAGGTTC
AGGGATTAAGAATAACTGACAGTGCATGCAAAATGCTCTTCCATGGAACATTGGATAAAAGTCCGATTACCGAGAGAATTAG
CACTAATCTGTTTTCACAGAACACGAGGAAGAGATGTTTTCACCTCCAAAGACAGCAGCAGCAATTAACAATTCAGGCTGC
AATGGCAGTAATAAGTACACCAACTGGCGATAAACATATGCAGATCATGACACCAACCACACCGCCTTCCTCTCATCTTCCCC
GTCACTAGCACAAGTGCACAACTGCATCCAGTATCCATTTTCCTGATTAAATACTAAATCCAGTACAATTATTTTAAGTGTTCA
GGCCAAGATCCAATCAAGCCTGCTCTTTAGTAGCAGCAGCACACTGTCACACACAACACTGCAACAATCAATCGAAAATCT
GTTCTTTTGTTTGGATTTTTTTGGAGGGGGCAAATTGCAGCAATTGCTCGGTGCTCATTTGGATCCAGATCTCCTGTGCTTTCC
CTGTATAAACAATTC
>SsLRR-RLK128
AGATGTACTAGCATTTTTTTAACTGAAGGAACATGATGCTTAGGATCAGATCTTGGCTTCAGCCAGACCTGAGAAATGTCATA
CTGTCAGAGAACTAACGGAAGAATTAGCTGTCAAAGAACTGTGCCTCAGTACTTTAAGAAGGTACAGGGTTATCCTAATTAA
CCCGGGACAAAGTCTGCTTCAAACTTAACCATGTGCTCCCTGCCTCCGTCTTGTCCACTACCCGGCCCCTTGATGGGATCTG
AATCCAAATCAACCCCATATTCCCTTGATGTTTACCACCACCCCATAATGCTCCAACATTATCAACCATGTGTGGTACCTAACA
TGTCTCCGGGCTTGTCCATCATCACCCATGTGGCCACATTCTAACCTCGAGTTAAGCGCGAATGACGGTTGATTGGCATGACT
TGTTCATTTGTTTAGTTTAATATTCGTATTGCTACTAAAGAGAGTTGTTTTTACATAGTTGACTTGAAATCCAGAATTTCACTGA
TACATGCTAACAAGTCACAAGTAATATGACTAGTTCTAACAAAACAGGCAAAATAGTTGGAGCCTCTTCAGTTGAAATCCAA
ATTTTACAACTGGAGAGCGTTCATCCCCAGTAAGATTCGGAGTGAACATTTCATCTGAATTGAATAAAAAAAGGTGGTGGAT
AATTGATCTGAAAAAATGGAAACACAAATGTACGAACAATAATGAAATAATTGTATCCACCAAGCAGTGGGCAAACATGCAT
TTCTCTTTTGAAGGATTGTGCATTGCCATTCCGGCTTTTAAAAGTAAGTAAGCTTGATGTGGAAATGTTATTTTAGTTATCAAA
ATTAAAGGGATCTACCAGCCATGGTGATGATGCTAAGCTAAGATAGATCCATGGAATTAACTATGAACCATAGGGTGGTTATCT
TAGTATCTTATTCCTCATTGTCCCTTCCACTCAAAGACATGAACATGTTCTCTCTCCAAAAATTTTATTTTTATCACCGCTCATC
CAATCTTTTTCAGTTACTTTCCAGCGATTTGCCACCTATTCACATGGGTAAGTTGCTGAAAGTTTTTTTATGCCTTTGCATTGTA
CTAACCTTCACCCCCTTCTCTTACCGCCATGTGCACCCCAAGATTCCTCTCTTATTCCCACTGTCTTACCCCTTTTGTTTAACC
AGCAGGCCTCTCTCTGATGCCCATGATGAATTCACACTCCACCGGAAGCAATTAAGTTGCCAGTTCTAGTATCATGGCGATCT
CCTTTTTGCAGAAACTTGATCATGTGAGTGAACATTCTGGGTCTCACACAGATTTACCCGTTACTGAGCTGCAAAAAGACTA
ATTTGAGATCACTCATGGATGCGCATAAACTGAGAAGGGCATACAAATTACAATGCCTGAAAAGAGGATGTAACAGCAGAA
ATAGTATTTTTGCAATTGTTAGTAGCAAGTCCTGTAAAGATTAAGGTCTCTGGGACTGTAAGTATTGCAGCTGCAGGCAAAGA
TAGATGCAGGTAATGGTAAAGCAAATGTGAACATTAAGACTTGAAATGTTGAATTAGCATCGAAAGATGCATTTTTTACAGCT
TTAGTAGACTTGCAGCGAAGAAGGATACCTGCAGAGCAGTAGTTACCTGTAAAGAGGTACCCGGAAAGCAGCACTGTCACA
GGCTCACAGCAGGGTAACCGTAAAGCAGCACTGTCGCAGCAGGCCGTCAGAAAACGTTTTAGAAGCTTACGTACGCCTAAA
GCCAGCAGCAACAGCCACTGTCAACTCTTGTACCAACGGTACAAAGGTTCAGGGATTAAGAATAACTGACAGTGCATGCAA
AATGCTCTTCCATGGAACATTGGATAAAAGTCCGATTACCGAGAGAATTAGCACTAATCTGTTTTCACAGAACACGAGGAAG
AGATGTTTTCACCTCCAAAGACAGCAGCAGCAATTAACAATTCAGGCTGCAATGGCAGTAATAAGTACACCAACTGGCGATA
AACATATGCAGATC
>SsLRR-RLK180
CTCATCTAACCAAGGACATGTGCCGCCCTCCATCAATGGGTGTAGATCTGATCCTTTTGTTTAACATCCGGAGCAGAACTGTA
ACAGTGAGAAAGTGCTGTGATTGTTAATGGTTACTGCATGAAGGAGCTGTTATGCGTGCCGCGTGTTACTACCACTTCACCC
ACTTCAGCTGTGATGCGCCCTTTTACTTTGTTTTCTTTACCCCTTTTCACCTTTCGGTTTCAACTCTTGAGATTCTTGCTTCGG
CTGCTTGCCATCCTGTAGTGCCTGTAATCACAAGTTCATATGTGGGGTGGTTTAAACAAAGGTGATTTTGCAGAAATGTAAGT
GCTTTTGGAACTGAGCATACGAATTAAAGGAGGTAGATGGAGGATTGTTTGCTATGATTCTTTTTTTTTTCTTTTGCTTTGTAT
CCCTCTGTTTCAAATTGTAAGTTGTTGTGGCCATTTAGAATGTCCTCATAAAAAGACATAGGACATGTTTAGTATAAGGGCTA
ATTATTAGCTAGCTAAAAATTAGCCTTAGCTCATCTAAATAGAAGAGCTAATTTTCTATCTAGTCTTTAACTAGTTGTTGGGTAA
CTAGTTAGCTGGCCAACTCTAGCTAATTCAGTCTAATTTTTAGCCCAAATTAGTAACCAGCCCTAATGATTTAAACATGCCTGT
ACCGATTAAAGTAAAAAATGGGCTACAAACTACGGCCGAAAGACATAAAATTGTAGGCAGATGGAGGGAGAAGTTACTTGC
CACGTGTTGGTGGCCATGTTAGGCTATCCGCACTCATGGTCCTCTATATTCATCCTCTATATATCCTAGTCAGCAAGATTCTTTC
CTCTATACTTAATTCTTCCGCACCCATCCATCCTCTATATCATCCTCTATCCACTCTATCTACTGTGGGACTCACTTGTCACTTTA
TCTAAATATATCCATGAGTGATCTAGTTTTGAAGGATTTATTGAGACAAATTTAATGGTGCAATCGAAATTTAATTTGGATTTTT

GGTTTATGAGTTATAACATTTTTTTCCTTGGTTTAGCTAGTAGGGCCTGCCACTGCGCATTATAGAGTAAACACTGTACTACTC
TAAATATAGAGTGAGAGAAAGAATAGAGTATCGTTTTAGAGTATCCAGTGCAGCAGTTTTTACTCTATATTCATACTCTAAAGC
TTTAGAGTATCGTTTAGAGTACGCCACTGTGGACAGTCTTATTGTAGGCGAGCTGTTACGTTCCCAAGATATTCTTTGCCCACT
GCCGCGCCTTTGCTGTTGCACTTGCCATGCAGATGCTGCGGCAGCGCGGCTTGCATGGTGAAAGTCTCTGCGCTGCCAACTG
CAAGAATTTGTTTGTTTTCTTCAGCAGAAAGGGTAACAAACCAACCAACCTGTTTCAGGCATAATTGGTGAAGCCAAAATTG
CACTGTAGGCTGTAGTATCATACTAGCATCATCCATGGAAATGGAACAGCAGCAGCTTCACAGCCTTTTCATGACGGTGGAG
GAGTTGCCTCAACTGAAACCTGACTGACTGATGCGATGCGATGCAACGGCTTCAGTGAAATGTGATTCACCATCTTTCCCTG
AGGGCCAAAGCTGATGCGGCTGCTACAATAGCCTCTCACCTCCTTTTCCATCTCAGGATCTCTCCTTCACTTCATTCAAGCAT
TTTGAAATCAAATTCGTTGTCGTTGATGACTCCTCTCTGAAATTTGAAACCAAAGAAAGCCGCTAATCATGTATCACCACCAT
TCAGCACAGGTAGTATATACTACTGTAGCATTGCTAATTGACAATGCATTTGCAACTAGTAGGGTCCAGGATGATGCCGGCAT
AACATAATTGATTGATGTAAACAAGTGCAGGGAGGGACAGGGGTGCGGAGCGTCCTGACGGCCTGCCTGCCTGACCCTGAC
CCAACCGGTGGCCTCGGATCCGGAGATTCTGCAGACACACATGAGTGCCTGCCACTGCCACCCACACCGTACCCTCTCTTCT
CCC
>SsLRR-RLK165
CGGTGGCTGCCGGCACCGGGGGAGGTCCCCGGCAGGGAGAGGTAGCGGGCGGCGCCGTCGAGCCCGCCATGGGCGCTGG
AGGTGGCCGACGGGGCTGGGGAAGCCACCGGCGGGCTGGGGTTCGAGTTGCCCAAAGAACTCCGCCCATGGATTGGTAGA
TCAGGAGCTGGGGAACTCGGATCTGAGGTTGGAATGGAGGGAGGGGGGCTGAGGAGGGAGATCGACGCGGGAGCAAGTG
GAGCGGCGCCGCCGTCGCCTGGAAGGGGAAACGGGTCGGGGGGCTCCAGCGGCTCCATCCTAATGTAAGTGGTTTTGGAA
CTGAGCATACGAATTAAAGGAGGTAGATGAAGGATTGTTTGCTATGATTCTTTTTTTTCTTTTGCTATCTACCCCTCCGTTCTA
AATTGTAAGTTATTCTGACCATTGAGAATGTCACCATAAAAAGGCTCAGGATATGTTTGATATAAGGGCTAATTAGTAGCTAGC
TAAAAATTAGACATAGCTCGTCTAAACAGGAGGGCTAAATTCCTACCCTAGTCTTTAACTAGTTGTTATGGAACGAGTTAGCT
AACTTGGGTTAATTTTTAGCCCTAATTAGTAACCAGGCCTAGTGATTTAAACATGCTCTTACGGGTTAAAGTCAAAAATGGTC
TCAAACTACGGCCGAAAGACATAAAATTGTAGGCAGATGGAGGGAGAAGCTACTTGCCACGTGTTGGTGGCCATGTTATTGT
AGGCGAGCTGTTACGTTCCCAAGATATTCTTTGCCCACTGCCCCCGCCTTTGCTGTTGCTGCTGCACTTGCCATGCAGATGCT
GCGGCAGCGCGGCGTGCATGGTGAGAAATCTTTGCGCTGCCAACTGCAAGAATTTGTATGTTTTCTTCAGCAGAAAGGGTA
ACAAACCAACCAACCAACCTGTTTCAGGCATAATTGGTGAAGCCAAAATTGCACTGTACTAGGCTGTAGCATTCTAGCATCA
TCCATGGAACAGCAGCAGCTTCACAGCCTTTTCGTGACGGTGGAGGAGTTGCCTCAACTGAAACCTGACTGATGCGATGCG
ATGCGATGATGCAACAGCTTGAATGAATGATTCACTATCTTTCCCCGAGGGCCAAAGCTGTTGCGGCTGCTAAAATAGCCTCT
CACCTCCTTTCTTTTCCATCTCAGGATCTCTCCTTAATTCAAGCATTTTGAAATCAAATTCGTAGTCGTTGGTGACTAATTTGA
GAGTCCCAGATATGCAATTTTGTCGAAATTTGAAACCAAAGAAATCCGCTAATCCTGTATCACCATCCAGCACAGGAATATAA
TACTGTAGCATTGCTAATTGACAGTGCATTTACAGCTAGTAGGGTCCTGGATGATGCCGGCATAACATAATTGATTGATGTAAA
CAAGTGCAGGGAAAGACAGGGTGCGGAGCGTCCTGACGGCCTGTCTGCCTGAGCCTGACCCAACCGGTGGCCTCGGATCC
GGAGATTCTGCAGACACATGAGTGCCTGCCACTGCCACCCACACCCTCTCCTCATGTACTTTGCAATGTCAGCAGCTCAGTT
AAGCCTGAACCTGAACCCCGCTTCTGCTTGCTGCCACCTAATCTGCCATTTCTCGCTGGTTTCCTGCTCCATCTGACCATCCC
TATCGTTCTCGTTGTCATCGGCATATGCCTCTGCCTCTTCTTGTCCCTCTACTAAACCTGTTCTCAGTGAGGCTTGGCTTAAAC
CTCGCCACCAATCCACCTACTCCGGGCTTTGCAAAGTTGCAGAGCCACTTTCTTGCGCTGCTGCTGCTGCTGCTTCCGGCAC
AAGCAACGACACAGATTTGCAAGTGACACCAGCTGCAGCTGATCCTACAGCATCACCTCACCTCATCCCCTTCATCGGGTTC
ATCGCAGTTTGGTCGCCTTGATGGATATGCTTTGGGAGTGAAGTACAGCGTCAAGTGTAGCGCTTGCTGCCTGGCGACTTGG
CAGGCGGTTGGAGAGCTGCAGATCGAGGGG
>SsLRR-RLK387
ATATATATATATATATAATATATATATATATATAGGGGTATAGAAAAAGAACAGAAAGAAAAGGAAAGGGAAAAAAACGTGCAGTG
TCGACACGCGACCCAGATCGGTCCAGCCCAAGCGGCACGGCCTTCGTAAACACAAAAACAAAAACAAATTGCGTGTCGTC
TTTATGAAATAAATTTAATTTAATTTAATTTAATTATTATTATCTGATGAATAAAGCTTGCCGATTGGCTATGTCAAAAAAAAAA
ATTAATGGCTTGCCTCCCTCCGCAAGCTTAAGCCTCCGCTTCCGCCCCCCAACAGCTCCGATCGGCTCATCCGCCGCCCGCA
GTCGGCTCGTGTCGCCGACTCGCCGCGATGAACGCGTCGCAGTTCATGGACAAGCAGATCCAGGGCCTCGCCGCCTCCGCC
GCCGCCGCCGCCGCCGCGTCCTCGCCGCCGGCATCCGACGGCGGCGGGCTAGTCGATCTGATGAGCCCCGACCCG
CAGGAGGAGGCGGTCAGCCACGGCCCGGGCACCGCTCCCTCCACGGCGGCGCAAACGGCACCGGCAACGGCGCCGACGA
GGTGTTGCCNNNNNNNNNNNNNNNNNNNNNNNNNNNNNNNNNNNNNNNNNNNNNNNNNNNNNNNNNNNNNNNNNNNN
NNNNNNNNNNNNNNNNNNNNNNNNNNNNNNNNNNNNNNNNNNACTTCTGCCATGTGTTACCACTGGGTCACCAGCTGTGATGTCTCCTTT
TATTCTTCTTTTAAACTTTCAACTTTGGAGATTCTTACTATCACAGTTGTTCAATTGTCACCCTGCAATGGTTGTAATTACAGTG
CAGGGCATACTGGTTGTTTACCCCATGACTTTAAGCAATAGCCATATTTGATATGGTACTAAACATGCATTTTTAAGTATTCCCA
AGTATTCTGAAATGATCAACAAAGTGGACTGAGAAAAATTGTTTTCACATGCATTCAAATTTTCAAACAAAATTATAAAGTAT
GTTCTAACATGAATGAAAATCACCTGTATCTGCCTCCTTGATCAGCTGAAATATGTTCTCTACTGTTTCAGCAAATTTTACTGA
CTGTATTCTGAATTATAACTTCTAAAGCTTGCGGATATGCTCAAGGTATGTTGCATGGAATGAATGACAAAACTTCGATGACCC
TGATGGATGTATCCTTTTATTTCATCTCCAATTATTCCAAGTTTCAGCTCTCGTCATTCTTCTGTTCTTTCAATTGTGACCCTGC
AAAGCTCAAAACTGCAACCAAGTCCTTGCTTGCTGTCAAAAAATGCTGCTCTAACTGCATGTTAATTTATCTTATCCCCCGTG
ACACACAGGCTTTCCATGAAGTTACTTAACTGTCTGCCTGATCTGATGCCGCCGGTGTGGTGTGGTGTGGTGTGGCTCTGAA
GAGGTGAGGCACACTATCTTTTCCTCTGATCATCAAAGCTGTTGCAGCCATTAAAAAACTCCCCTGCAAAGCAAATTAGTCG
TTGGTGTGATCTCTCCCTACCCAAGTTTATTTCAAAACCTAACCTTCCAATCCTAACAGTAACCTAGCACTTAGCACCAGTAA
TTACACTGCACTAAGAGTCCACAAAGATGGACAGAGGCGTATGCAGTGTGCAGCACACTGACCTTGGATCCGGAGATTCTG
CAGGCACAAGCTGCACAGCCTCCATTGCTCCATGACCTCATCAAACTCTAAACTCTTTCGCCTCCTCCTACCCTCTCAAACAT
AGCCTATTTAGACCCCACTCCACTTTCTTGTTCTTACTGCTATCTTTGTCCTCCCTCTTCCCCTGCAACTACTACGAGGCGAGC
ATATAAAAAACCTTGCACCATCTCCCATGGCCTCTCTTTCTCTCTCTTCGTCTTCTTCCTCTGTTTGTACTAGGCTTGAGACAT
AAGCAAGACCAAGATTTGGGGGCAATTAGAAGTAGTACGTTGCAATTCTACAGAATTATCGTGTTCTTGGACATATACTTACA
AATTCAGTACAAGTGTTAGT
>SsLRR-RLK356
TCGGTGAGTGTTGTTCCTCAGTCTAGTCAGTCAGAGTTTGTAAGTAGTAACTCATTGATCTCATGGACTACAGATTGTTCTAC
TTCAACTTTAGTCTCTGTATATGTATGCTATCAGAGTTCACGGCACTTATCTCTGTACATCTATTCTTTTTTACTGTAAACACCG
AGCAGGAGTTTGTACCATAACCTTTTCAGGTAAAACAACCAGCACTGCAACAGAAGTTCTACTTCCAATGTAAATCCCCTGC
AAGTTCCATTATCTTCTACCGGATCGGGATCAGTAACGCTGCAGTGCTTACTCAGAACAGCTCATTTACCAATGGGAATTTGG
AGAGGCAGGAAGGTAGATCTAGATGCGTGTGCCTGCAAAATTTCCTGCCCTTTTGCCAGTATATATATTTGATCTGCTGGATG
GCGGCTTCCTCTGCATTAACACTCAAGTACAAGGGGGCAGTAATTAACAAAGTATTTGACAGTGGAAAGGCAAGATTAGATC
TAACCCAGGGCATGTGACAGCAACTCCATTGAGGGGTTTGGATCCCATCCTGACTGTTTAATCCGCCAAGGAACAGGCTCTG
GCATTGGGGTTTTTATTATTGTTACTGCATGGAATGAACTGGTTCTTATTCACTTCTGCCATGTGTTACCACTGGGTCACCAGC
TGTGATGTCTCCTTTTATTCTTCTTTTAAACTTTCAACTTTGGAGATTCTTACTATCACAGTTGTTCAATTGTCACCCTGCAATG
GTTGTAATTACAGTGCAGGACATACTGGTTGTTTACCCCATGACTTTAAGCAATAGCCATATTTGATATGGTACTAAACATGCA
TTTTTCAGTATTCCCAAGTATTCTGAAATGACTGAGAAAAAATTGTTTTCACATGCATTCAAATTTTCAAACAAAATTATAAAGT
ATGTTCTAACATGAATGAAAATCACCTGTATCTGCCTCCTTGATCAACTGAAATATGTTCTCTACTGTTTCAGCAAATTTTACT
GACTGTATTCTGAATTATAACTTCTAAAGCTTGCGGATATGCTCAAGGTATGTTGCATGGAATGAATGACAAAACTTCGATGA
CCCTGATGGATGTATCCTTTTATTTCATCTCCAATTATTCCAAGTTTCAGCTCTCGTCATTCTTCTGTTCTTTCAATTGTGACCCT

GCAAAGCTCAAAACTGCAACCAAGTCCTTGCTTGCTGTCAAAAAATGCTGCTCTAACTGCATGTTAATTTATCTTATCCCCCG
TGACACACAGGCTTTCCATGAAGTTACTTAACTGTCTGCCGGATCTGATGCCGCCGGTGTGGTGTGGTGTGGTGTGGCTCTG
AAGAGGTGAGGCACACTATCTTTTCCTCTGATCATCAAAGCTGTTGCAGCCATTAAAAAACTCCCCTGCAAAGCAAATTAGT
CGTTGGTGTGATCTCTCCCTACCCAAGTTTATTTCAAAACCTAACCTTCCAATCCTAACAGTAACCTAGCACTTAGCACCAGT
AATTACACTGCACTAAGAGTCCACAAAGATGGACAGAGGCGTATGCAGTGTGCAGCACACTGACCTTGGATCCGGAGATTC
TGCAGGCACAAGCTGCACAGCCTCCATTGCTCCATGACCTCATCAAACTCTAAACTCTTTCACCTCCTCCTACCCTCTCAAAC
ATAGCCTATTTAGACCCCAGTCCACTTTCTTGTTCTTACTGCTATCTTTGTCCTCCCTCTTCCCCTGCAACTACTACGAGGCGA
GCATATAAAAAACCTTGCACCATCTCCCATGGCCTCTCTTTCTCTCTCTTCGTCTTCTTCCTCTGTTTGTACTAGACTTGAGAC
ATAAGCAAGACCAAGATTTGGGGGCAATTAGAAGTAGTACATTGCAATTCTACAGAATTATCGTGTTCTTGGACATATACTTA
CAAATTCAGTACAAGTGTTAGTATGAGTCTTGGTGTTATGATGAGTAGGAGAAGCAAATGGCCGGCGGTGGCGACAGCGCC
GCTC
>SsLRR-RLK388
CAGTAGTAATAATTTCTAGCGTGCTGTTCTTTGTCAGGTTCATCCATCAATCGGTGAGTGTTGTTCCTCAGTCTAGTCAGTCAG
AGTTTGTAAGTAGTAACTCATTGATCTCATGGACTACAGATTGTTCTACTTCGACTTTAGTCTCTGTATATGTATGCTATCAGAG
TTCACGGCACTTATCTGTGTACATCTATTCTTTTTTACTGTAAACACCGAGCAGGAGTTTGTACCATAACCTTTTCAGGTAAAA
CAACCAGCACTGCAACAGAAGTTCTACTTCCAATGTAAATCCCCTGCAAGTTCCATTATCTTCTACCGGATCGGGATCAGTAA
CGCTGCAGTGCTTACTCAGAACAGCTCATTTACCAATGGGAATTTGGAGAGGCAGGAAGGTAGATCTAGATGCGTGTGCCTG
CAAAATTTCCTGCCCTTTTGCCAGTATATATATTTGATCTGCTGGATGGCGGCTTCCTCTGCATTAACACTCAAGTACAAGGGG
GCAGTAATTAACAAAGTATTTGACAGTGGAAAGGCAAGATTAGATCTAACCCAGGGCATGTGACAGCAACTCCATTGAGGG
GTTTGGATCCAATCCTGACTGTTTAATCCGCCAAGGAACAGACTCTGGCATTGGGGTTTATATTATTGTTACTGCATGGAATGA
ACTGGTTCTTATTCACTTCTGCCATGTGTTACCACTGGGTCACCAGCTGTGATGTCTCCTTTTATTCTTCTTTTAAACTTTCAA
CTTTGGAGATTCTTACTATCACAGTTGTTCAATTGTCACCCTGCAATGGTTGTAATTACAGTGCAGGGCATACTGGTTGTTTAC
CCCATGACTTTAAGCAATAGCCATATTTGATATGGTACTAAACATGCATTTTTAAGTATTCCCAAGTATTCTGAAATGATCAACA
AAGTGGACTGAGAAAAATTGTTTTCACATGCATTCAAATTTTCAAACAAAATTATAAAGTATGTTCTAACATGAATGAAAATC
ACCTGTATCTGCCTCCTTGATCAGCTGAAATATGTTCTCTACTGTTTCAGCAAATTTTACTGACTGTATTCTGAATTATAACTTC
TAAAGCTTGCGGATATGCTCAAGGTATGTTGCATGGAATGAATGACAAAACTTCGATGACCCTGATGGATGTATCCTTTTATTT
CATCTCCAATTATTCCAAGTTTCAGCTCTCGTCATTCTTCTGTTCTTTCATTTGTGACCCTGCAAAGCTCAAAACTGCAACCA
AGTCCTTGCTTGCTGTCAAAAAATGCTGCTCTAACTGCATGTTAATTTATCTTATCCCCCGTGACACACAGGCTTTCCATGAA
GTTACTTAACTGTCTGCCTGATCTGATGCCGCCGGTGTGGTGTGGTGTGGTGTGGCTCTGAAGAGGTGAGGCACACTATCTT
TTCCTCTGATCATCAAAGCTGTTGCAGCCATTAAAAAACTCCCCTGCAAAGCAAATTAGTCGTTGGTGTGATCTCTCCCTACC
CAAGTTTATTTCAAAACCTAACCTTCCAATCCTAACAGTAACCTAGCACTTAGCACCAGTAATTACACTGCACTAAGAGTCCA
CAAAGATGGACAGAGGCGTATGCAGTGTGCAGCACACTGACCTTGGATCCGGAGATTCTGCAGGCACAAGCTGCACAGCCT
CCATTGCTCCATGACCTCATCAAACTCTAAACTCTTTCGCCTCCTCCTACCCTCTCAAACATAGCCTATTTAGACCCCACTCCA
CTTTCTTGTTCTTACTGCTATCTTTGTCCTCCCTCTTCCCCTGCAACTACTACGAGGCGAGCATATAAAAAACCTTGCACCATC
TCCCATGGCCTCTCTTTCTCTCTCTTCGTCTTCTTCCTCTGTTTGTACTAGGCTTGAGACATAAGCAAGACCAAGATTTGGGG
GCAATTAGAAGTAGTACGTTGCAATTCTACAGAATTATCGTGTTCTTGGACATATACTTACAAATTCAGTACAAGTGTTAGT
>SsLRR-RLK193
AAGACTAGTAGAAGTGCTAGGCATATGTTACCCCCAGTCAGCCGTCACTAAGAAGAATGGAGCCTGAAGTTTAGTCACCGTT
CCGCGATACTCTGATGTAAGTATTAATATAGCTATGTATACGTTATATAATAACATTGTTTCTGATACTTTTTCATTCTGGCATATG
TGTGGACTTCCTGGGCACACATATGATTAGCCTGGTTTTGTTCTTTAGAAAATCGGGTGTGACAGAGTGGTATCAGAGCGGT
GTTGACTGTAGGACGTATGCCAAGTAACACTGGACGTATTATAAAACTTATTTACTGTAAAACTATTTTGTCATCCCTTGACT
CATTGATTTTGACTATTTCCTCTTCCTTGTCTTGTTGTATCCCTTCCTTGATAGCTTCCTTTGAGCTATTATTTCTTACTCCCTTG
GTTTTTGTTTTATGTTGTTTTTATTAAAAGTTTTCTGATCCACTGGTTCAAAGATTCGATGGCCTTTCATCATCCATAATGTACC
TTTAGGATGACCACCATTGTTGAAGAAGCACGTGTAGTACCGATGATGTGATGTGTGCTTGTTTTGCTTGATGTGTTGCTTGT
GTGTTGCCTTGTTCTCGTGTTTCAAAGAGTGTCTCGTAAGGTATAAGTCTTGAGGAGTAGCTCATCCATGTTAAATGTGGGGT
AGGTTTTACGTAATACTCCGCATGCTGATTTCTATAGCAGTCTGCAATGTTTCTGTTATATCTCAAGATATGGACATGAGAAATT
TATGAAATTTTACTGGGAATAACTAGACTTCTGTATCTTTCCAAACCCACTGGACTTACCTTTTTATCTATTTTGAGCTAAGAG
ATANNNNNNNNNNNNNNNNNNNNNNNNNNNNNNNNNNNNNNNNNNNNNNNNNNNNNNNNNNNNNNNNN
NNNNNNNNNNNNNNNNNNNNNNNNNNTTCAGGCATAATTGGTGAGCCAAAATTGCACTGTACTAGGCTGTAGCATTCTAGC
ATCATCCATGAACAGCAAGCAGCTTCACAGCCTTTTCATGACGGTGGAGGAGTTGCCTCAACTGAAACCTGACTGATGATGC
GATGCGATATGCAACAGCTTGAAATGTGATTCACTATCTTTCCCGAGGGCCAAAGCTGATGCGGCTGCTAAAATAGCCTCTCA
CCTCCTTTTCCATCTCAGGATCTCTCCTTAATTCAAGCATTTTGAATCAAATTCGTAGTCGTGGTGACTAATTTGAGAGTCCCA
GATATGCAATTTTGTCGAAATTTGAAACCAAAGAAATCCGCTAATCCTGTATCACCATCCAGCACAGTATATAATACTGTAGCA
TTGCTAATTGACAGTGCATTTGCTAGTAGGGTCCAGGATGATGCCGGCATAACATAATTGATTGATGTAAACAAGTGCAGGGA
GGGACAGGGTGCGGAGCGTCCTGACGGCCTGCCTGCCTGAGCCTGACCCAACCGGTGGCCTCGGATCCGGAGATTCTGCA
GACACATGAGTGCCTGCCACTGCCACCCACACCTCTCCTCATGTACTTGCAATGTCAGCAGCTCAGTTAAGCCTGAACCCCG
CTTCTGCTTGCTGCCACCTAATCTGCCATTTCTCGCTGGTTTCCTGCTCCATCTGACCATCCCTATCGTTCTCGTTGTCATCGG
CATATGCCTCTGCCTCTTCTTGTCCCTCTACTAAACCTGTTTCAGTGAGGCTTGGCTTAAACCTCGCCACCAATCCACCTACTC
CGGGCTTTGCAAAGTTGCAGAGCCACTTTCTTGCGCTGCTGCTGCTGCTGCTTCCGGCACAAGCAACGACACAGATTTGCA
AGTGACACCAGCTGCAGCTGATCCTACAGCATCACCTCACCTCATCCCCTTCATCGGGTTCATCGCAGTTTGGTCGCCTTGAT
GGATATGCTTTGGGAGTGAAGTACAGCGTCAAGTGTAGCGCTTGCTGCCTGGCGACTTGGCAGGCGGTTGGAGAGCTGCAG
ATCGAGGGGG
>SsLRR-RLK367
GTTCCTCAGTCTAGTCAGTCAGAGTTTGTAAGTAGTAACTCATTGATCTCATGGACTACAGATTGTTCTACTTCGACTTTAGTC
TCTGTATATGTATGCTATCAGAGTTCACGGCACTTATCTGTGTACATCTATTCTTTTTTACTGTAAACACCGAGCAGGAGTTTGT
ACCATAACCTTTTCAGGTAAAACAACCAGCACTGCAACAGAAGTTCTACTTCCAATGTAAATCCCCTGCAAGTTCCATTATCT
TCTACCGGATCGGGATCAGTAACGCTGCAGTGCTTACTCAGAACAGCTCATTTACCAATGGGAATTTGGAGAGGCAGGAAG
GTAGATCTAGATGCGTGTGCCTGCAAAATTTCCTGCCCTTTTGCCAGTATATATATTTGATCTGCTGGATGGCGGCTTCCTCTG
CATTAACACTCAAGTACAAGGGGGCAGTAATTAACAAAGTATTTGACAGTGGAAAGGCAAGATTAGATCTAACCCAGGGCAT
GTGACAGCAACTCCATTGAGGGGTTTGGATCCAATCCTGACTGTTTAATCCGCCAAGGAACAGCTCTGGCATTGGGGTTTAT
ATTATTGTTACTGCATGGAATGAACTGGTTCTTATTCACTTCTGCCATGTGTTACCACTGGGTCACCAGCTGTGATGTCTCCTT
TTATTCTTCTTTTAAACTTTCAACTTTGGAGATTCTTACTATCACAGTTGTTCAATTGTCACCCTGCAATGGTTGTAATTACAGT
GCAGGGCATACTGGTTGTTTACCCCATGACTTTAAGCAATAGCCATATTTGATATGGTACTAAACATGCATTTTTAAGTATTCCC
AAGTATTCTGAAATGATCAACAAAGTGGACTGAGAAAAATTGTTTTCACATGCATTCAAATTTTCAAACAAAATTATAAAGTA
TGTTCTAACATGAATGAAAATCACCTGTATCTGCCTCCTTGATCAGCTGAAATATGTTCTCTACTGTTTCAGCAAATTTTACTG
ACTGTATTCTGAATTATAACTTCTAAAGCTTGCGGATATGCTCAAGGTATGTTGCATGGAATGAATGACAAAACTTCGATGAC
CCTGATGGATGTATCCTTTTATTTCATCTCCAATTATTCCAAGTTTCAGCTCTCGTCATTCTTCTGTTCTTTCAATTGTGACCCT
GCAAAGCTCAAAACTGCAACCAAGTCCTTGCTTGCTGTCAAAAAATGCTGCTCTAACTGCATGTTAATTTATCTTATCCCCCG
TGACACACAGGCTTTCCATGAAGTTACTTAACTGTCTGCCTGATCTGATGCCGCCGGTGTGGTGTGGTGTGGTGTGGCTCTG
AAGAGGTGAGGCACACTATCTTTTCCTCTGATCATCAAAGCTGTTGCAGCCATTAAAAAACTCCCCTGCAAAGCAAATTAGT

CGTTGGTGTGATCTCTCCCTACCCAAGTTTATTTCAAAACCTAACCTTCCAATCCTAACAGTAACCTAGCACTTAGCACCAGT
AATTACACTGCACTAAGAGTCCACAAAGATGGACAGAGGCGTATGCAGTGTGCAGCACACTGACCTTGGATCCGGAGATTC
TGCAGGCACAAGCTGCACAGCCTCCATTGCTCCATGACCTCATCAAACTCTAAACTCTTTCGCCTCCTCCTACCCTCTCAAAC
ATAGCCTATTTAGACCCCAGTCCACTTTCTTGTTCTTACTGCTATCTTTGTCCTCCCTCTTCCCCTGCAACTACTACGAGGCGA
GCATATAAAAAACCTTGCACCATCTCCCATGGCCTCTCTTTCTCTCTCTTCGTCTTCTTCCTCTGTTTGTACTAGGCTTGAGAC
ATAAGCAAGACCAAGATTTGGGGGCAATTAGAAGTAGTACGTTGCAATTCTACAGAATTATCGTGTTCTTGGACATATACTTA
CAAATTCAGTACAAGTGTTAGTATGAGTCTTGGTGTTATGATGAGTAGGAGAAGCAAATGGCGGGCGGTGGCGACAGCGCC
GCTC
>SsLRR-RLK302
TTCCCCCTGTTCGGCTGAGCTTATCAGCACGGCTTATCAGCCATGGAATAGTATTTTTCTCTCACAGCAAATCAGCCACAGCC
GGCTTATAAGCAAAAAAAAAAAAAAAAAACAGCCTGCCGAACAGGGCCATTCTTAATCTTGTTTAGGCTAGGCTGAAGATGT
AGCTCGTGGCAGCGGCATATGCATAGCATAGGCATGCAACAGCTGGTTCTGAGCGCGCGCGTGACGTACGCGTGATCCAAG
GAATAACCCATGCCGGCATCTTGACATCTCTATGCTAACATTACACTACTGGCTGATTATTACTGTACTGGGTTAGGGAGGGAC
AGGCTTGCAGGCCGTACTCTGTTTTTTAATTTCAATGCAAGGCCTGTTTGATTCACCTTGCTAAATTTTAACGGGCGTTGACT
ATGGAGTTGACCATAAGGTGTTCAGATACTAATAATGAGCTAATTAAGTTTAATCGATTCGTCTCGTAAATTAATCTCAATTTG
TGCAATTAGTTTCGTAATTAATCTATATTTTACACTCTATACATATGTCCAAACATTAAATGTGATAGGTGTTAAAAAAACAAGG
GGAACCAAAATCAAACAATTGTGCATTCAGAGTGGGAAATGTATTTATGAGTTTTTTTTATATCTAGTAAGTGGTTTCAACTG
CTGGCCGGGTCATCACTCAGAATTAGCAAATTTATCAAAAAATGCTGATTGCGCAACTTCAGGTAGTTTATTATCAACAAGTT
TGCCATTCTAGACCGGCAGTTAATTTGTACCCATTTCTTTTTAAAAAAAAAACCAAGATAGCATAGGGTTTAAGCTAGTGAAAT
AATTGTGGGTATCTACTGCCTTACAATGTTTACCTAAAGTTGTCGTAGTCGTAGTAATTGATGAGAGTTGCCGGATTTGAAAG
GAGGCCTGAGGCTTCTACTATTTTTCCCTCCACGAGCATGCATATTGCATCCACATGCAGAGGCTGGAACGGTGTTCCATTAT
CTAAAAACAAAAAAAGTACATATTGCATACATAGAAAATCTATTACCCTCACATTTGATGGGCCACGAGTGCCTGCCGCCCCA
TGCTTGGCCAGCGCACTTGTCGTGAACTCGTGATAGCATTAGTACGTTGTGGGCCTTGTATAGATCCAAAATATTTCGCGAAA
AAACAGTCCATCACATTGAATCTTTCGACACATGTATGAAGCATTAAATATAGATTAAAAAATAAAACTAATTACACAGTTTAG
ATGAAATAGACGAGACGAATCTTTTAAGCCTAATTAAGTCATGATTGGACACTAATTATCAAATAATAACGAAAAGTGCTACA
GTAGCATTTCCCGAAATATTTCAGGAACTAAACGAGCCCTTGCTTATTTGCATGTACGTACAGAAATTACTCAAATAGTATAAA
GCACAGTAGTGGCTAGCTGAAGGGCATGCACAGCAGCACCAATCTAAACTAATCCCTCCGTCCCCTTAATTAATTTGTCAGT
GTGTGCCCACACTAATTTCTTTCCCGCGCGCTCTCCGATGCGCGCGCACCACCACCAAACAATGGTAGACTACTGCTGCGAG
GGTTTCGAGGAGCCCAATGCTGCGTCGTTGCGTTGATGCAAGCACCCATTCATCGTCCACCTCCTCCTGCTGCTGCCTGCCT
GCAACAACCAAACCAAACCAAGCAAGGAGCAGGACACAAGCAAACACCTTTGAGGGTTTTGAGGAAGACAGAGAGAGAG
AAACAAGAGACTGTGACAGTGTGCGAGCTGAGCTGAGCCTCCTCGTCTCCCTCCTCCTCTCCGCTCCGATCCCCCCGCACTC
GCTCTGCATTGAATCCATTCTCTTCTTCCGTCTCCATTGAGATCATCGCCCAGGGCAGCAAGCAAGCAGCCTTGCTTGGTGCT
TGGTGCCATTGTGCACCGCCCGAGCCGAGCCCCCGAACACCGCGCACTTTTTCATGCGCCTGCCGCCCTTGCCATTGCCGCC
ATCGCCAAGCCCCA
>SsLRR-RLK270
GACTCTGTAAGCAATAAATGAAGCCAGATTCTTAAAAACCGAGGGACCCAAAAGCCCTCACCAGTTTTTTTTTCTAGAATCC
AGATTTTATAGACTAAAGACAAAAACTAGTTTTTAAAAATCTGAAACTCACCCAAACATATCCTTAATCTTGTTTAGGCTAGG
CTGAAGATGTAGCTCGTGGCAGCGGCATATGCATAGCATAGGCATGCAACAGCTGGTTCTGAGCGCGCGCGTGACGTACGCG
TGATCCAAGGAATAACCCATGCCGGCATCTTGACATCTCTATGCTAACATTACACTACTGGCTGATTATTACTGTACTGGGTTA
GGGAGGGACAGGCTTGCAGGCCGTACTCTGTTTTTTAATTTCAATGCAAGGGCTGTTTGTTCACCCTGCTAAACTTTAACAG
GTGTTGACTATGGGGTTGACCATAAGGTGTTCAGATATTAATAATGTGCTAATTAGGTTTAATAAATTCGTCTCGTAAATTAATC
TCAATTTATGCAATTAGTTTCGTAATTAGTCTATATTTTACACTCCATGCATATGTCCAAACATTCAATGTGATAGGTGTTAAAA
AAACAAGAGAAACCAAACACCCCCTAAGGCCAGTCTCAATGGGGTTTCATCAGAGTTTCATAGGCATTAATTATGCTGATGT
GACACCATATTAATGAAGAGAGATGATAAGAGTTTCATGAGAGTAGAGAGAGCTTCATGGAATGAAACTCTTCTGCACTGTT
TCCAAAATCTGGATGTGTTGAAAATAGTAACATGAAATCCCCACTAAGACTGGCCTAAGAGTGCACGTAGGAATCAAACAAT
TGTGCATTCAGAATGGGAAATGTGTTTATGAGTTTTTTTTATATCTAGGAAGTGGTTTCAACTGCTGGCCGGGTCATCATTCAG
AATTAGCAAATTTATCAAAAAAATGCTGATTGCGCAACTTCAGGTAGTTTATTATCAAAAAGTTTGCCATTCTAGACCGGCAG
TTAATTTGTACCCATTTCTTTTTTTTTTAAAAAAAAAAAAGAAAACCAAGATAGCATCGGGTTTAAGCTAGTGAAATAATTGTG
GGTATCTACTGCCTTACAATGTTTTATGTTTACCTAAAGTTGTCGTAGTCGTAGTAATTGATGAGAGTTGCCGGATTTGAAAGG
AGGCCTGAGGCTTCTACTATTTTTCCCTCCACGAGCATGCATATTGCATCCACATGCAGAGGCTGGAACGGTGTTCCATTATC
TAAAAACAAAAAAAAAACATATTGCATACTCCATTACCCTCACATTTGATGGGCCACGAGTGCCTGCCGCCTCATGCTTGGC
CAGCACACTTGTCGTGAACTCGGGATAGCATTACTTTGCAATTTGCTTATTTGCTGGAACGGAGTACGTACAGAAATTACTCA
AATATAAAGCACAGTAGTGGCTAGCTGAAGGGCATGCACAGCAGCACCCATCTAAACTAATCCCTCCGTCCCCTTAATTAATT
TGTCAGTGTGTGCCCACACTAATTTCTTTCCCGCGCGCTCTCCGATGCGCGCGCACCACCACCAAACAATGGTAGACTACTG
CTGCGAGGGTTTCGAGGAGCCCAATGCTGCGTCGTTGCGTTGATGCAAGCACCCATTCATCGTCCACCTCCTGCTGCCGCCT
GCCTGCAACAACCAAACCAAACCAAGCAAGGAGCAGGACCCAAGCAAACACCTTTGAGGGTTTTGAGGAAGAGAGAGAC
TGTGACAGTGTGCGAGCAGAGCTGAGCCTCGTCTCCCTCCTCCTCTCCGCTCCGATCCCCCCGCAGTCGCTCTGCATTGAAT
CCATTCTCTTCTTCCGTCTCCATTGAGATCATCGCCCACGGCCAGCCAGGGCAGCAAGCAAGCAAGCCTTGCTTGGTGCTTG
GTGCTTGGTGCCATTGTGCACCGCCCGAGCCGAGCCCCCGAACACCGCGCACTTTTTCATGCGCCTGCCGCCCTTGCCATTG
CCGCCATCGCCAAGCCC
>SsLRR-RLK280
CAATATGAAATAAAATAGCCATATACAACATGGATTTACATATGGTGCTGCTAAAACATAGGAGGATGAAGCTAGCAACCTTT
CAAACAAATCGACGACGGCGTAGAAGCGTTTGAAGTGGATTGATCGCCGGAGATGAGACAAGCAAGAACAATGGAAGAAC
AAGCAACAAGAACCTGAACAAGGAAGGGCTCGGGCAGAGAGAAAGAAGAGGGAGGTTCAATTTATAGGGGGAGGTTTTA
ATCCCGGTTGGTAACACCAACCGGGACTAAACCCAGTCCTATAGTCCCGGTCGGTGCCACGGACCGGGACAAAACTGTAGT
CCCGGTCCGTGGCACCGACCGGGACTAAAGGCAGGGCTTTAGTCCGGTTGGTGTTACCAACGGGACTAAAGGTCCCTCCCG
ACAGCGCCTGACAACAGAGCCGTTGGCGAGGGACCTTTAGTCCCGGTTGGTAACACCAACCGGAACTAAAAGTCTATTTAG
TCCCGGTCGCCAAAATCGCCGAGACTAAAGACATTTTGGGCGATCAACGAAAGGTCAGTTGTGTAGTAGTGTGGGGATGAA
ACTCTTTTGCACTGTTTCCAAAATCTGGATGTATTGGAAACAGTGACGTGAAATCCCCACTGAGACTGGCCTAAGAGTGCAC
GTAGGAATCAAACAATTGTGCATTCAGAATGGGAAATGTATTTATGAGTTTTTTTTTATATCTAGTAAGTGGTTTCAACTGCTG
ACCGGGTCGTCATTCAGAATTAGCAAATTTATCAAAAAAATGCTGATCGCGCAACTTCAGGTAGTTTATTATCAACAAGTTTGC
CATTCTAGACCGGCAGTTAATTTGTACCCATTTCTTTTAAAAAAAAAACCAAGATAGCATCGGGTTTAAGCTAGTGAAATAATT
GTGGGTATCTACTGCCTTACAATGTTTTATGTTTACCTAAAGTTGTCGTAGTCGTAGTAATTGATGAGAGTTGCCGGATTTGAA
AGGAGGCCTGAGGCTTCTACTATTTTTCCCTCCACGAGCATGCATATTGCATCCACATGCAGAGGCTGGAACGGTGTTCCATT
ATCTAAAAACAAAAAAATACATATTGCATACATAGAAAACTTGTCGTGAACTCGTGATAGCATTACGTTGCAATTTGCTTATTT
GCATGTACTGTACGTACAGAAATTACTTAAATATAAAAGCACAGTAGTGGCTAGCTGAAGGGCATGCACAGCAGCACCAATC
TAAGGCCCTGTTTAGTTCCCAGAAAATTTTGCAAAATTTTTCAGATTCCCCGTCACATCGAATCTTTAGACACATTAAATATAG
ATAAAAAATAAAACTAATTACACAGTTTAGACGAAATGAACGAGACGAATCTTTTAAACCTAATTAAATCATAATTGGACAAT
ATTTGTCAAATAACAACGAAAAGCGCTATAGTGTCATTTTGCCAAAAATTTCGGATCTAAACCAATCCTAAACTAATCCCTCC
GTCCCCTTAATTAATTTGTCAGTGTGTGCCCACACTAATTTCTTTCCCGCGCGCTCTCCGATGCGCGCGCACCACCACCAAAC

AATGGTAGACAAAGAGGGGGAGAGAGAAAGAACAAGTACTCCTGCTACTGCAGCGAGGGTTTCGAGGAGCCCAATTGCTG
CGTCGTTGCGTTGATGCAAGCACCCATTCATCGTCCACCTCCTCCTGCTGCTGCCTGCCTGCAACAACCAAACCAAACCAAG
CAAGGAGCAGGACCCAAGCAAACACCTTTGAGGGTTTTGAGGAAGAGAGAGACTGTGACAGTGTGCGAGCTGAGCTGAG
CCTCCTCGTCTCCCTCCTCCTCTCCGCTCCGATCCCCCCGCACTCGCTCTGCATTGAATCCATTCTCTTCTTCCGTCTCCATTG
AGATCATCGCCCAGGGCCAGCCAGGGCAGCAAGCAAGCAGCCTTGCTTGGTGCTTGGTGCCATTGTGCACCGCCCGAGCCG
AGCCCCCAAACACCGCGCGCGCACTTTTTC
>SsLRR-RLK282
TTCAAACATAAAAAGTGATTGGAGCTAGCAATATGAAATAAAATAGCCATATACAACATGAATTTACATATGGTGCTGCTAAA
ACATAGGAGGATGAAGCTAGCAACCTTTCAAACAAACCGACGACGGCGTAGAAGCGTTTGAAATGGATTGATCGCCGGAGA
TAAGACGAGCAAAAACAATGGAAGAACAGGCAACAAGAACATGAACAAGGAAGGGCTCGGGCAGAGAGGAAGAAGAGG
GAGGTTCAATTTATAGGGGGAGGTTTTAATCCCGGTTGGTAACACTAACCGGGACTAAACCCAGTCCTATAGTCCCGGTCGG
TGCACGGACCGGGACAAAAACTGTAGTCCCGGTCCGTGGCACCGACCGGGACTAAAGGCAGGGCTTTAGTCCAGGTTGGT
GTTACCAACTGGGACTAAAGGTCCCTCCCGACAGCGCCTGACAACAGAGCCGTTGGCGAGGGACCTTTAGTCCCGGTTGGT
AACACCAACCGGAACTAAAAGTCTATTTAGTCCCGGTCGCCAAAATCGCCGAGACTAAAGACATTTTGGGCGATCAACGAA
AGGTCAGTTGTGTAGTAGTGTGGGGATGAAACTCTTTTGCACTGTTTCCAAAATCTGGATGTATTGGAAACAGTGACGTGAA
ATCCCCACTGAGACTGGCCTAAGAGTGCACGTAGGAATCAAACAATTGTGCATTCAGAATGGGAAATGTATTTATGAGTTTTT
TTTATATCTAGTAAGTGGTTTCAACTGCTGACCGGGTCGTCATTCAGAATTAGCAAATTTATCAAAAAATGCTGATCGCGCAA
CTTCAGGTAGTTTATTATCAACAAGTTTGCCATTCTAGACCGGCAGTTAATTTGTACCCATTTCTTTTAAAAAAAAACCAAGAT
AGCATCGGGTTTAAGCTAGTGAAATAATTGTGGGTATCTACTGCCTTACAATGTTTTATGTTTACCTAAAGTTGTCGTAGTCGT
AGTAATTGATGAGAGTTGCCGGATTTGAAAGGAGGCCTGAGGCTTCTACTATTTTTCCCTCCACGAGCATGCATATTGCATCC
ACATGCAGAGGCTGGAACGGTGTTCCATTATCTAAAAACAAAAAAATACATATTGCATACATAGAAAACTTGTCGTGAACTC
GTGATAGCATTACGTTGCAATTTGCTTATTTGCATGTACTGTACGTACAGAAATTACTTAAATATAAAAGCACAGTAGTGGCTA
GCTGAAGGGCATGCACAGCAGCACCAATCTAAGGCCCTGTTTAGTTCCCAGAAAATTTTGCAAAATTTTTCAGATTCCCCGT
CACATCGAATCTTTAGACACATTAAATATAGATAAAAAATAAAACTAATTACACAGTTTAGACGAAATGAACGAGACGAATCT
TTTAAACCTAATTAAATCATAATTGGACAATATTTGTCAAATAACAACGAAAAGCGCTATAGTGTCATTTTGCCAAAAATTTCG
GATCTAAACCAATCCTAAACTAATGTGTGCCCACACTAATTTCTTTCCCGCGCGCTCTCCGATGCGCGCGCACCACCACCAAA
CAATGGTAGACTACTGCTGCGAGGGTTTCGAGGAGCCCAATTGCTGCGTCGTTGCGTTGATGCAAGCACCCATTCATCGTCC
ACCTCCTCCTGCTGCTGCCTGCCTGCAACAACCAAACCAAACCAAGCAAGGAGCAGGACCCAAGCAAACACCTTTGAGGG
TTTTGAGGAAGAGAGAGACTGTGACAGTGTGCGAGCTGAGCTGAGCCTCCTCGTCTCCCTCCTCCTCTCCGCTCCGATCCCC
CCGCACTCGCTCTGCATTGAATCCATTCTCTTCTTCCGTCTCCATTGAGATCATCGCCCAGGGCCAGCCAGGGCAGCAAGCA
AGCAGCCTTGCTTGGTGCTTGGTGCCATTGTGCACCGCCCGAGCCGAGCCCCCGAACACCGCGCACTTTTTCATGCGCCTGC
CGCCCTTGCCATTGCCGCCATCGCCAAGC
>SsLRR-RLK60
AAGCAACGGCTGATTGTAAAAGTTTGCTAGTTTCCAGCTTCTACACTATTCCATATTTCCACACTAGTCGTTTCAGAGTCACA
CAAGTCACAGCCTTTATTTGTTAGGAAGAGCCGCACACAGCTCAGCCCATGATGATCCTTTTCAAGTGCGGCCATCTGATCA
CACTCATTGATACCCTGTTTGCTGGAACTTATCAGCCCGGTTTAGTAGGGATGGTACAATGTTTTTCTCTCACAATAAAATAGC
ATCAGTCGTTTTGTCAGCCGCAGAAACCATCCAGCGAACAGAGTGACGTGTAGTCACACAGAAATTTCAGTGTCAGTGGCA
TGCATGGATTGCCATGTCCAGTTTAATTCGTGGAAAGAAGCTGTTAGGTAAGGCCAGACCCACAGCGAGTGATTCGTCGCGT
CAGAGATGACCCAACAATAAGATCCTGCTGGCAATCTCACATAATTTGAGCTCTATACGGATAACGAAACAGCGACATCTCTT
CAGTCCTTTACCATGTGAAAAAGCCTTGAGCCAGTTCTTTATATGCCAGCATGCAAATGTTAGTAGAATTCTCAATTTGTCATA
AAAAAAGTCATGATCCCTTCACGTACCATCACTTTATCTTTTTTTCCTTTTAGTTTTCATATGCGTGCCACTTTCATATAATAAC
AGAGTTGACACCACCAATGTCACGTAATGTCAAAGGCCTAACTAAATTCATCACAGCACCAAAAAAAAAAGGAAAAACTTG
CAAGCACAGACTAGTACATACCTACTCCCTCAATTTTGCAGGGAGTTGATTATTCCCTTTTCCGAGCACCTCTAATCTAGTGAT
TGGCCTCTTTGTCGTTGAAACTGTCCTAAGTGCTCCTATATTACTAGTACCAGCTGGTAACAGCCTGAGACCCTGTCGACGCA
ACACGGTTTTCCTTTGCAAATAGGAGTACTATATCAGCGTACAGTCGCCGTACATGTAGCGCACTACAGGCAATAAATAAAAC
CGGCCACTAGAAAAACACACGGTTGCCTACTCACCTGATATGATAACTGATATATTTGTATAAGCATGGATCAAGTTGATGAA
ACCACAGGACATTGACAGGCGCTAGAGATGGACCGGCCGGATGAGGAAGCCAGCGGCCAACCAAGACCACGACCCAGCAT
AACATTTTTATTTTTTATTTTTTTTTGGTGGTGGTGTTGGAGATGGACTACCGGATTTGAACCAGATCTATGGAGCAGGCGCT
GAAAATGTATCAGGGTCTCGCCGGAGCTCCGCGGCCGTGGAGGGGCAACCATGCATTGAGAAGAGGAAGAAGACAGTGAA
GGAAAAAAATCGAGTGTTCTCAGGGCCCAAAACACCCGAACGACGCAAACTCATTTTATAAACTTGAAATTACAAAATGAA
CCTGTTGCTACATTGACAAGAAAAGGGAGAAGGACAAGTCTTTAAATCAAATATAATCAGTTTATTAGATCCCTCCTACACCT
GAAATTTATGAGTGTGTTATAAAAGATTCAGATGTTTTAGATGAGCTTCTAACCCAGATTAAAGGTGTTGTAATAATCCGGGAT
AATATTCCTTCACCACACCTGAATTTTTACTTACACCAATTTATATCTAAAAATTACACCAAAATATTCACCGTAAATCTAAAAG
TTAAGTATAAATCAAATATAAAAGTCAAGTATAATAAATCTAATAAAAAAGGCAACTATAAAAATTCACCGTAGATTTAGGATG
ACGAGAGAGAAGACTTTCATTTGTTTGAATCTTATTAGTCTTATTCCCTTCTATGCATGCCATTTAGATATATAGAAAGAAGAA
AGGGGAAATGAAGGAAAGAAAAAATTCTGACGTGGGGGGAAAAGATTTTAATTTCACATGTGTGTCGACCTCTCTAATCCC
ACGCGTTAACTCTTCAGAAGCAATGCAGTGCAACGCCTGATCACATATTCACATGTGTGCTTAGCCGCCTTTGCCTAGATTCT
GGAGAGGAAGAGA
>SsLRR-RLK30
CTGGGGCTGAAAGGGCTCTGGAGAAATAGTAAGCAACGGCTGATTGTAAAAGTTTGCTAGTTTCCAGCTTCTACACTATTCC
ATATTTCCATACTAGTCGTTTCGGAGTCACACAAGTCACAGCCTTTATTTGTTAGGAAGAGCCGCACTAGCTCAGCCCATGAT
GATCCTTTTCAATTGCGGCCATCTGATCACACTCATCGATACCCTATTCGCTAGAACTTATCAACCCAGTTTATCAGCTATGGT
ACAATATTTTTCTCTCACAACAAAACAACATTAGCTGCTTTATCAGCCGCAGAAACCATCCAATGTAATCACACAGAAATTTC
AGTGTCAGTGGCATGCATGGATTGCCATGTCCAGTTTAATTCGTGGAAAGAAGCTTTAGGTAAGGCCAGACCCACAGCGAGT
GATTCGTCGCGTCAGAGATGACCCAACAATAAGATCCTGCTGGCAATCTCACATAATCTGAGCTCTATACGGGTAACGAAAC
AGCGACATCTCTTCAGTCCTTTACCATGTGAAAAAGCCTTGAGCCAGTTCTTTATATGCCAGCATGCAAATGATAGTAGAATT
CTCAATTTGTCATAAAAAAAAAGTCATGATCCCTTCACGTACCATCACTTCATCTTTTTTTCCTTTTCGTTTTCATATGCGTGCCA
CTTTCATATAATAACAGAGTTGACATCAATGTCACGTAATGTCAAAGGCCTAACTAAATTCATCACAGCACAAAAAAAAAATA
AGAAAAAAGAAAAACTTGCAAGCACAGACTAGTACATACCTACTCCCTCAATTTTGCAGGGAGTTGATTATCCCCTTTTCCG
AGCACCTCTAATCTAGTGATTCACCTCTTTGTCGTTGAAACTGTCCTAAGTGCTCTTATATTACTAGTACCAGCTGGTAACAGC
CTGAGACCCTGTCGACGCAACACGGTTTTCCTTTGCAAATAGGAGTACTATATATCAACGTACAGTCGCCGTACATGTAGCGC
ACTGCAGGCAATAAATAAAACTGGCCACTAGAAAAACACACGGTTGCCTACTCACCTGATATGATAACTGATATATTTGTATA
AGCATGGATCAAGTTGATGAAACCACAGGACATTGACAGGCGCTAGAGATGGACCGGCCGGATGAGGAAGCCAGCGGCCA
ACCAAGACCACGACCCAGCATAACTTTTTTTTTTTGTGGTGGTGTTGGAGATGGACTACCGGATTTGAACCAGATCCATGGA
GCAGGCGCTGAAAATCTACCAGGGTCTCGCCGGAGCTCCGCGGCCGTGGAGGGGCAACCACGCATTGAGAAGAGGAAGAA
GACAGTGAATGAAAAAATCAAGTGTTCTCAGGGCCCAAAACACCCGAACGACGCAGACTCATTTTATAAACTTGAAATTAC
AAAATGAACCTGTTGCTACATTGACAAGAAAAGGGAGAAGGACAAGTCTTTAAATCAAATATAATCAGTTTATTAGATCCCT
CCTACACCCGAAATTTATGAGCGTGTTATAAAAGATTCAGATGTTTTAGATGAGCTTCTAACCCAGATTAAAGGTGGTGTAAT
AACCCGGGATAATATTCCTTCACCACACCTGAATTTTTACTTACACCAATTTATATCTAAAAATTACACCAAAATATTCACCGTA
AATCTAAAAGTTAAGTATAAATCAAATATAAAAGTCAAGTATAATAAATCTAATAAAAAAAGGCAACTATAAAAATTCACCGT

AGATTTAGGATGACGAGAGAGAAGACTTTCATTTGAATCTTATTAGTCTTATTCCCTTCTATGCATGCCATTTAGATATATAGAA
AGAAGAAAGGGGAAATGAAGGAAACCAAAAAATTCTGACGTGGGGGAAAAGATTTTCACATGTGTGTCAACCTCTCTAAT
CCCACGCGTCAACTCTTCAGAAGCAATGCAGTGCAACGCCTGATCACATATTCACATGTGTGCTTAGCCGCCTTTGCCTAGAT
TCTGGAGAGGAAGAGA
>SsLRR-RLK13
ATACCCCGCCATGATGGGTGGCGGGTTTGGCGTACCCCGCCACCCATCATGGCGGGGTACGCCCCGGGTGGCGGCCACGTC
ACCCTGCCACGTGGGATGTGTACCCCGCCACCATGCATGGCGGGGTTCAGTGCATTTGCCCCGCCATGCATGGTGGCGGGGC
CAAACAGGTTAGATTTAAAAAAAATTTCAAACAAGGGTTAAATTTAAAATTTGTTTCAAATTTGGGCTAAAAATAAAAAAAA
TTCCACATAATCTGAGCTCTATACGATAACGAAACAGCGACATCTCTTCAGTCCTTTACCATGTGAAAAAGCCTTGAGCCAGT
TCTTTATATGCCAGCATGCAAATGTTAGTAGAATTTTCAATTTGTCATAAAAAAAAAAGTCATGATCCCTTCACGTACCATCACT
TCATCTTTTTTTCCTTTTCGTTTTCATATGCGTGCCACTTTCATATAATAACAGAGTTGACACCAATGTCACGTAATGTCAAAG
GCCTAACTAAATTCATCACAGCACCAAAAAAAAAGGAAAAACTTGCAAGCACAGACTAGTACATACCTACTCCCTCAATTTTG
CAGGGAGTTGATTATCCCCTTTTCCGAGCACCTCTAATCTAGTGATTGACCTCTTTGTCGTTGAAACTGTCCTAAGTGCTCCT
ATATTACTAGTACCAGCTGGTAACAGCCTGAGACCCTGTCGACGCAACACGGTTTTCCTTTGCAAATAGGAGTACTATATCAA
CGTACAGTCGCCGTACATGTAGCGCACTGCAGGCAATAAATAAAACCGGCCACTAGAAAAACACACGGTTGCCTACTCACC
TGATATGATAACTGATATAGGCAGTGTTTACTTCCCTCAAAATTTCAAAAATTTTACAAGATTCCCCGTCACATCGAATCTTTA
GACGCATGCATGGAGTATTAAATGTAGGTTAAAAAAATAACTAATTGCATAGTTTGTCTGTAAATGTCGAGACGAATCTTTTA
ACCCTAGTTACATGATTGGATAAATTTTACCAAATAACAATGAAAAGTGCTACAGGAACTTTACACCCACTTTTACACCATCT
AAACACTACCATATTTGTATAAGCATAGATCAAGTTGATGAAACCACAAGACATTGACAGGCGTTAGAGATGGACCGGCCGG
ATGAGGAAGCCAGCGGCCAACCAAGACCACGACCCAGCATAACATTTTTTATTTTATTTTTTTGGTGGTGGTGTTGGAGATAG
ACTACCGGATTTGAACCAGATCCATGGAGCAGGCGCTGAAAATGTACCAGGGTCTCGCCGGAGCTCCGCGGCCGTGGCCGC
GGCCGTGGAGGGGCAACCACGCATTGAGAAGAGGAAGAAGACAGTGAAGGAAAAAATCGAGTGTTCTCAGGGCCCAAAA
CACCCGAACGACGCAAACTCATTTTATAAACTTGAAATTACAAAATGAACCTGTTGCTACATTGACAAGAAAAGGGAGAAG
GACAAGTCTTTAAATCAAATATAATCAGTTTATTAGATCCCTCCTACACCCGAAATTTATGAGCGTGTTATAAAAGATTCTGAT
GTTTTAGATGAGCTTCTAACCCAGATTAAAGGTGTTGTAATAACCCGGGATAATATTCCTTCACCACACACCAATTTATATCTA
AAAATTACACCAAAATATTCACCGTAAATCTAAAAGTTAAGTATAAATCAAATATAAAAGTCAAGTATAATAAATCTAATAAAA
AAGGCAACTATAAAAATTCACCGTAGATTTAGGATGACTTATTAGTCTTATTCCCTTCTATGCATGCCATTTAGATATATAGAAA
GAAGAAAGGGGAAATGAAGGGAAGAAAAAATTCTGACGTGGGGGGAAAGATTTAATTTTCACATGTGTGTCAACCTCTCTA
ATCCCACGCGTCAACTCTTCAGAAGCAATGCAGTGCAACGCCTGATCACATATTCACATGTGTGCTTAGCCGCCTTTGCCTAG
ATTCTGGAGAGGAAGAGA
>SsLRR-RLK47
GTGTCGTTGAGGGATGCCAGCAGCTGGCCACTGTAGATATTGGGAACAACAGGTTCCATGGTGATATTCCTCCTTGGATTGG
AAGCCGTGCTCCAGCACTGAAAATCCTTAGACTTAGTTCGAACAACTTCACTGGTCAAATTCCTCCGGAGTTATCACAGCTT
TCACAACTTCAGTTGCTAGACTTGTCCCACAATAGATTGACAGGATTGATCCCAGCAAAGTTGGGAAACTTAGAGTCCATGA
AGCATCCGAAAATTAATTCCAGTATAGGATCGCTAGATGGGTCCACCTATCAGGATAGAATTGACATCATCTGGAAGGGACAA
GAGCTGATATTCCAAAGAATACTTGAGCTGATGACAGGCATCGATTTGTCAGGCAATTCACTGTCGCACTGCATCCCTGAAG
AGTTAACCAATCTCCAAGGCCTCCGATTTCTGAACCTGTCAAGAAACAATATGTCATGTACCATTCCAAAAAACATTGGTAGC
CTCAAATATCTTGAGTCCCTTGATCTTTCGTGGAATGAAATTTCAGGGCCCATTCCTCCAGGCATGAGTAGTTTGCAGTCCCT
TAACACATTGAATCTCTCAAACAACCATTTGTCAGGCAAGATACCTACCGGGAATCAACTTCAGACACTCATAGACCCATCG
ATTTATGGAAACAATCCTGACCTCTGTGGACCTCCTTTGAACATTTCTTGCCAAGATCCGTCACAGGCATTTGATGAGGGAAA
TGGCGGAGAATGCCAGGACCAATGGCTGTACTACTGTGTGATCGGTGGAATTGTTTTTGGATTCTGGCTGTGGTATGGGATGC
TCTTTTCCATTGCGAAGTTGAGGTATTCTGTTTTCTTGTTTGTTGATCGCATACAATACAAAATTATGCAAAAGGTGCGGCCTA
TCAATCATTTTCTTTCGAAAGAAAAGAGTGATCCATCTTCCGTACCTATGTTTTCCACAAGTGATACAAGCTGAAAAATGGAG
TAAGAGTAATGATGTCTTCTCTACTATACTTAGCAATCCAATCTTCTCTTTTTTTTTTCCTCCCCTAGTACGGCAGGAGATTTGC
TGCCTTTCTTTGTATGCTTTAAGATGTAATGCTGAGAGTATGCCAGAAGGATTATAGTTAACTGTTATATAGCAATTTCAGTAA
AAAAAACATTATATGTTTAAGGAGATTTGCTGTCTTTCTCTGTATGCCTTTCTGATATGACACGTTTAAGAAGTTCTTACTTTTT
TGTACTTGATTCATTTCTACTACTGTTTTGAAAAGAATATGCATAAGAAATTATTTTCAAGAAGTGGCATAGATATACCCGTCAT
GCATTGAGGGAAAACTTATGCTGGGAGCTGCACTTCCATCGTCAAAAAGATTTAGAGAGAACTAAGAACTTATCATGCACAG
CCAGAAAAGGGTAAGGAAAATAAAGTGACAATAGTTTCTCTTAGCCTGCTGCCTTCTGATATCTATATTCAGTTACATATTTAG
CCCTTCCCGTCAAATGGAGCAATAAATCATAAGTATGGCAATAAAGTAGAACACCTAAGTTGACTACAGACTAATTCTTGCAA
TTGGTGACTTCCATAATAATTTCGTAGTCTATCAACCAGTTCTACCAATAAACTTTCCTAGTTCTTGTCTTTGGTACTAGAACTT
GAGAAACTTGATGCTCTAGAACTGTTGGTTTCTGCACAGGTATTTATTGCTTCAGGTAGTCTGTTAGTTTGCTTACTACTAGG
ATCTGTCGGGATGGTGCAGAAAGACACGCCTGAGTATGCGACGAACATGTACAGTTTCTTCTGCAATGTCTAATATGTCCTGT
AGTTGAGCAGGCAGACATGTTGAACAAACAAACAGTCAAACATAGAACCCTAAACTACCCTGGTCTCTTCCTCTCCCAAAA
TTCTAGCTATCTGATTGCGCCTAGCTGTTATCCAGCTAGTTGGGTGCTGCCAAACGAAGCTAGATCGTTACAGGCTGCCAGCC
GATCC
>SsLRR-RLK31
TGTGTATTAAATATAGACTAAAAAATAATTAATTGTACAGATTGCGACTAATTTACGAGACGAATTTTTTTAAGCCTAATTAGTT
TATGATTTGATACTGTGGTGCTATAGTAAACATGTGCTAATAACGAATTAATTAAGCTTAATAAATTCGTCTCGCGAAATCTATA
ATTTATTTTTTTATTAGTATCAAACACCCCTAACATTGGATGTGACACCTACCTACAAGACTTGGGAGGCCATCATTCCGCATA
AGTACAACAAAGCGCGTGACTGACTGCACGTCCAGAGAAACGACGTAAGATGATGGTTGGGTTAGGTCATTAGGCGTACGT
TAAGGGGTAGTTACGTTTTTTTTTAGAATGGGCCAGCGAATAACACTACTGTCCGCCTATCCGGTTCCTGTGTCAATGGATTTT
TGTTTCTAGTACTTTTCGAACATGATCACATGATGAAGTACTCCGTATGTAAGTTGTAAGACATGCTGGAAACTAGTTTATTTG
TGCTCGTCCTGGCCTGGGCCGGATAAACTAGTAGTAGCTGTAACTACCAAAGTCCCGCAAAGATTTTTATATCTCAAGTCAAA
CGAACGTAAGTTTAACTAAGTTTATATAAATAATAATAACAATAATATCTATGATATTAAATAGATACAATCAGATTCATGCATAT
GTATAATGCAATATATCTTTATAGTAAATTTATTTAGTGTTATATGTGTTGAAATATAATCTTCCCTTCAAAAAAAGCGTTGATAA
TCTACTCTTTAAAGTTAGTTAAACTTAAAATATTTTAACCTAGAACAAAACAAAAACTTCACTCTTTGCTGGACGAGGGTAGC
CAGCTACTAATACAGCCTTAATCGTGCATCCACAAATGTCGTTCACGAATTAACTGCTTGTGCACCTCAAGCAATAGCATAGC
CCTCCCTTTCCTGGGTTGGACTAGGACCGCGACCCTAACGACCACAAATCCGGAATGGGATGACAAATCGAGCTTTACCAA
GGCTTGCCATTTTGTTCCAGGTCAAAAATCACCACCCTGTTCGGCAGGCTGTTTCTGCGGCTTATAAGCCGGCTGTGGCTGTT
TTGCTGTGAGAGAAAAACACTGTTCCACGGCTGATAAGCCGTGCTGATAAGCTCAGCCGAACAGGGTGCACGTAATTGAAC
CATCATACCTTCTCAGCACACCAATATACCTATATACAAGAATTCTAGTGGGAATAAGCTTTGTTATCTAAATGCATAATAAAAA
GGGAGTAGTACAAGTTAGAGTATGTTTTTTCCCCCTCAGACATGTTGGCTCTCTTGGGAATCTATTCAGTTCGGTGTGTTTATC
ACGAGCAATAGTTGGTTTGGTTTTGATGCAATGATGTTAACCAATTCATTTTGTCTGTTCTATGACAAGGGTTGACTCTCCAA
CCATCTGTTCTCTAGTGGTGGAAAATTATAAACTTATTTTGGGAGTTTTTTCACTTGTTACTAGATCTAATTCCTAGCGGCAAG
TGGATTTTGGGGCCATCAAAGTCTTGCATGGCAATGACGTTTGTAGGTGTCCTTTTTATCTTATTGCTCTTTGATTGCAATTTTC
AAATTCTGACAAATAATCGAAGAAAGAAGACAACTATATCAGCTTTTGATGCACTCTATTTTTATGGTCATTTTGTGACAAATT
ATTCATATTTTACAATAGTAATCATTTTTCTTGATAAATATCCGACATAAGACATTCTTTCGAGCGTTTTTATTAAAAAAACATTT
TTTATCGAGAGAATGAGAACCGGAATCGCACGCATACTTGACCGGCCGTAGCACCGCATGTGCTCGCAAGTCAACCCACTG
CATTGACCGCCAGCCAGCCGCGCCCGCGCGCGCCGCGAGCTCAAAGAGTCAAGACCACCACGTGCTCACCGCCACACCGC

ACCCCCGTCCGGCCCTCCCCGCCCGCCTCACCGCACGAACGGGCACGGGCACGCCGCGTTCCGCTCTGCCACCCGGCCA
>SsLRR-RLK61
CCTTTTTCAGGTTTGTGAAGATCAGCCCAGAGGGTAAGGTGCATGTGTATAACGGTGGTGATGGCAAATGGATTGCTAATTCT
GATGTGATCACCCAAGTCATTGAGTAGAAGTACCCAACTCCATCTCTGATCACCACTCAGTGTAATAAGTTCAACTGGTGCTT
GTTTCTAAAGATCCTTGCTATATATTTATCCCTGTAATTTTCATAGGAGTTACCTCCTAAAGGAAAAAAGGAAGTAATGCATTT
CTCAAGTATATACAGATCATAAGTTTCAGTAGGAAGCAGATGTACAATCTGTCATGCAATTAATTTTTATCCAGTATGGTTGAA
CTATGTCCATTTGCCTACAGCTCCAAGCTTGGTCTTTGTTTCCATTGACGGAATCGACGGAAGACATCTGAGCTACCTGGGAG
ACTATGTTGAAGGATGAAATAGTTGCTAGGAATCATCAAAAGTCGTCAAATGTTTAGTGTAGTTTTGACGTGTTCTTGAGACA
TGTGCATTTGTATGTCTGTGATTTGATTCCCTGTTATTCTATCTACTCTGAACTCATCCATCATGCTACAAGAACTAATGCTTCC
TGATGTTAGGAAAGGAAAGCTATCCTAATTGTTCAATTGCAACCTTGTAATATTTCTTTTACATGAAAAAAAATTGGTCTGCA
CTCAGCTTGGTTGAAAAAAAAATTGGTGCATGAGACTAAAAACTATGGAAAAAAAATATCGGCGTTTAGTTGAAAATGGTTGTT
TTCAAGTGCATCCTTAGAATGAAGCCGTGGCGTTAACACGGATACTATACTAGTTATAAGTAATTATATAAGATGATGTTTGAT
CGTTTATTTATTTAACACAAGCAAAAACTGAAACATTCCAGTTTGAGATAAAAACATTCATAAAAGATAGCAATATTAACCAT
GGAATGCCAGGACTGGTAAAAAAAACTCCAGGGGTAAAATGGACTTTTCACATAGGACTTAACACCGTTAGCTACCCAAAC
TGACGGAAGTGTCAAATAGGTAAGAAAAACTCGAGTTGAGGACATATAGGAAAGATTTTCAGTTTCAGTGTCAAATAGAAA
ATGAGCATTATTTTCAGGGCCAAATAGGAAATTTTCTCATAAACTTAGGCCCTGTTCATCCGGCTTATAATCCGTACTTTTCAA
CTTATTTTTTCAGCCAGAACAATATTTTTCTCTCACAATAAATCATCCAACAATGTTTTTTAGCCGGCTTATCAGCACAGCCGA
ACGGGGCCTTATTTTGGGTTTCCTATAGCCGATGTTTTTTTAGTCACTACTAGATCTAATTCATAGCGACGAGTGGTTTTTGAG
GTCACCAAAGTCTTGCAAGACAATGTCCCGTTCGGCTGTGCTGATAAGCCGACTGAAAAACACCGTTGGCTGATTTGTTGTG
AGAGAAAAATACTGTTCCGGCTAAAAAACAAACTGAAAAATACGGATTATAAGCCGGATGAACAGGACCAATGGTGTTTGT
ATTTTTGTCTTGTAGCTTTCATTGCAATTTTCAAATTCTGACAGACTACGTATAATCGAAGAAAGAAGACAACCATATTAGTTT
TTGATATACCCTTATTTTTATGGTCATTTTGTGACCAACTTTTATAATAGTAATCATTTTTCTTGATAAATACCCAAATAAGACAT
TCTTTTGAGTGTTTTTATTGAAAAAAAAAAGCTTGGAACGTTTTTCTTTATCTAGAATGAAAACCGGCATCGCATATGCTTGAC
CAAATGCAAGTGCGCCGCTGCCTCTTTTCCCGTTCACGCCGCTTCCCACCCGCGTGGCCCGTGGCACTGCATGTGCTCGCAA
GTCAACCCACTGCATTGACCGCTTGCCAGCCGCGCCCGCGCGCGCCGCGAGCTCAAAGAGTCAAGACCACCACGTGCTCA
CCGCCTCACCGCACCCCCGTCCGGCCCTCCCCGCCCGCCTCACCGCACGAACGGGTACGGCCAAGCCGCGCTCCGCTCTGC
CACCCGGCCA
>SsLRR-RLK46
CTCCATCGCTACCGCTTGGGAGACCATCATTCCGCATAAGTACAACAAAGTGCACGTCCAGAGGAACGACGTAAGATGATGG
TTGGGTTAGGCCATTAGGCGTACGTTGAGGGGTAGTTACGGTTTTTTTTAAGAATGGGCCAGCGAATAACAGTACTGTCCGC
CTGTCCGGTTCCTGTGTCAATGGATTTTTGTTTCTAGTACTTTTCGAACATGATCACATGATGAAGGACATGCTGGAAACTAG
TTTATTTGTGCTCGTCCTGGCCTGGACCGGATAAATTAGTAGTAGCTGTAACTACCAAAGTCCCGCAAAGATTTTTATATCTCA
AGTCAAACAAACGTAAGTTTAACTAAGTTTATATAAATAATAATAACAATAATATCTATGATATTAAATAGATACAATCAGATTC
ATGCATATGTATAATGCAATATATCTTTATAGTAAATTTATTTAGTGTTATATGTGTTGAAATATAATCTTCCCTTCAAAAAAAAG
CGTTGATAATCTACTCTTTAAAGTTAGTTAAACTTAAAATATTTTAACTTAGAACAAAACAAAAACTTCACTCTTTGCTGGAC
GAGGGTAGCCAGCTAGTAATACAGCCTTAATCGTGCATCCACAAATGTCGTTCACGAATTAACTGCTTGTGCACCTCAAGCA
ATAGCATAGCCCTCCCTTTCCTGGGTTGGACTAGGACCGCGACCCTAAGGCCAGTCTCAGTGGGAGTTTCACTAGAGTTTCA
TTTGCATTTAATAGCCTGCCACATATGCATTTTTGATGATATGTCAACGTTTTAATGAAGAGAGAGAAGAATTGAGTTTCATGG
GGATAAAACTCTCTTGGCACAGTTACCAACTCTCTATGAGTCATATAATTAAATGTCTATGAAACTATGGAGTAAAACTTTTCA
TTGAGAGTGGGTGTTTCATCCGAGTTTCATTTCATTCTATATGACATGGCAGTCTTGAAAACAACGAAATGAAACTCTCCACT
GAAACTGGCCTAACGACCACAAATCCGGAATGGGATGACAAATCGAGCTTTACCAAGGTTTGCCATTTTGTTCCAGGTCAAA
AATCACGTAATTGAACCATCATACCTCAGCACACCAATATACCTATATACAAGAATTCTAGTGGGAATAAGCTTTGTTATCTAA
ATGCATAATAAAAAGGGAGTAGTACAAGTTAGAGTATGTTTTTCCCCCTCAGACATGTTGGCTCTCTTGGGAATCTATTCAGT
TCGGTGTGTTTATCACGACCAATAGTTGGTTTGGTTTTGATGCAATGATGTTAACCAATTCATTTTGTCTGTTCTATGACAAGG
GTTGATTCTCCAACAATCTGTTCTCTAGTGGTGGAAAATTATAAACTTATTTTGGGACTTTTTTTTTCACTTGTCACTAGATCTA
ATTCCTAGCGGCAAGTGGATTTTGGGGCCATCAAAGTCTTGCATGGCAATGACGTTTGTAGGTGTCCTATTCATCTTGTTGCT
CTTTGATTGCAATTTTCAAATTCTGACAAATAATCGAAGAAAGAAGACAACTATATCAGCTTTTGATGTACTCTATTTCTATGG
TCATTTTGTGACAAATTATTCATATTTACAATAGTAATCATTTTTCTTGATAAATACTCCCTCCATTCCAAATTATAAGGCATTC
CAAGAATCTTGGAGAGTCAAAGCATCTTAACTTTGACCAAAATTATAGAGAAAATTACAAAAAATTATAACATCAAATAGGTA
TATTATGAAAATATAATTAATGAAGAATCTAATGATACTTAATTGGTGTCATAAATATTATTATCTTATCATATAAATTTGGTCAAA
CTTGAGATCCTTTGACTCTCCAAGATTCTTGAAATGTCTTATAATTTGGGATGGAGGGAGTATCCGACATAAGACATTCTTTTG
AGCGTTTTTATTAAAAAACATTTTTTTATCGAGAGAATGAGAACCGGAATCGCACGCATACGTGACCAAATGCA
>SsLRR-RLK169
CATTGACTAACGAATGACTGTGGTGGATGCTTGGATGCCGCACCTGCATCTTCTAAAGTTGCTTTCAGATTCCAGTCCAAATC
CACCACCTCGCTGTTACATAGTTTTTTCTAGTGACTTGGTCAGGATTGGGATCCGATGATCGTTATGCAGTACGGACTATGGA
CCACTGAAGTCAAGAACGGCACACGTTTAAAAACTCAAGCACTCGTATAGTTTCTCCCTAAAAGTTTACGCCCTATCCTACG
AATGTTTGTATACGTGCATGAAGTATTAAATATAGACTAAAAAAATAACTAATTATACAGATGGCGACTACTTTACGAGACAAA
TCTTTTAAGCCTAATTAGTCTATGATTTGATAATAAAGTGCTACAGTACACATATGCTAATGATAGATTAATTAGGCTTAATAAAT
TCGTCTCGCGGTTTACTGACGGACTGTAATTTATTTTTTATTAGTATCCGAACACTCCCCTATAACGCCCCGGACACGAATACG
ATGCTGCACGCGTGAGCATGAGCGTGACCTGATCGTGCTCGCCGTTGCCCCGCGAAGCATGGCGTGTCCTGACGGAGTACG
CGTACTACTACTGTGCTGACAAAACGTCCAGCTGCTCTTGGGCCTTGTTTGGCTGTGCTGATAAGCCGGCTGAAAAACACTG
TTGACTGATTTGTTGTGAGAGAAAAATACTATTCCAGCTGAAAAAAACAAACTGAAAAGTACGGATTATAAGCCGAATGAAC
AGAGCCTTTGTGGAAAATATTTTTGTTCCATTTTGGTTCAGAATTTTTTACAATTTGTTCCATTGTGGAAATGAAGCACAGAAT
TGACAATTCTAGTGTTGTGACTGTCGTGGCAAACTGGCAACATCAAGGAAAGTCCATCTACATACCTAGATGGCTAGTGTTT
CTCTGGTTTTGGTTGCCTGGCTATCTAATAGCGCGTTTGGTTCACATCATGAGGTTGTGCGGAATCATCTCATCCAACATCCAA
CCTTGGTGAGACGTTTGGTTTGCATCTCGTCGTCAACTCATCCGTCACGAAACCATTCCTTGTACGATGTTTCCGTAGGCCAA
CCCCAACAAATCTATCGAGTAGAGTCATTTCTAGAAAAATGCCGTACGACTTCATTCCAGACCAACTCAATTCTGTGCGCGTT
TGGATCAATCAGGATTTTTTTACTAGCCATGTGCGCGTTGCTCATTTTTTTCATCTAATTATTCTTGTAACGATAAGTAATGTGC
ATCAGTACACGCATCTTATATAATGTAAAGAGCTTAATTAGATCATCTGATTTATTAGGAAAAATGATGAAAAACTGGCATCAA
AATTATCTGTTTTTTAGGACGGCCTCGCCACCAATTTTGAGCTAAAAACGGCTCAAATAAAACCACACAATTTAATCAGTCAC
CACACCTGCGGACCCGCGGTCAACACAGGAAGGTTGCAAATACAAATACTACGAAATGTAAATAAACGAGAAAAAAATTAA
AGAGCTGTAGTAGTTCGTGTTTGGCCCATCATAGAAATGTTTCATTTGATCCGGCCCACAGTCGATTTCCAAGACCTCAGCCC
AACAGCCCCAAGTACCCCCAAGCCCAACAGGCCTAGGTTTGTTCCCCTCAAAGAAAAAAAAAAAAGGCCAAGGTTTTGAAT
GTACTGTATAATACTCCTCCTTTCGGGGAAGGCGTACTCACGTAAATCTTACTTTGGCTCTACCGCCGGCCGGAGAAGAGAC
GAACACTGTGCGGATCTGTAACCGTAGTGCGTTTCCACTTTCCACTTCCTTCAGTTCATCCACGACAGAGCAATTAATCAATT
GCACAGCTGCGTTCGTCAATCCTCACCCGTCAATCTTCCGTTCCCAAGTCGCAACTGCGTTTCCTGCTCCGCGTCTTCAAAG
AAGGGGAGACCTGAAGGAGAAACGAATCCAGGCTGCTGGCTGCCGGTGCCGGGCTGGACGCTGGTGCATACTGCGTAGGC
AAAGAGCAGGCAAT
>SsLRR-RLK357
TAGCTACTAAAACCACCTCATTCTCCTCTCTTTTTTAGATTACTTCAAAAAAAAGAATCATATAGAAACTTCAAAATCTTCCAA

CATGAAAGTTGTTCAAAATTGAAAAATCTACAACTTTCATTTTTGGATCAACTCCAAATTCCAATTAGTTTTGAAAGTAGGAT
TTTGAATTTGAGTTTAGGGTGATTAAATAATTCATTTACAAACTTGGTATTCAATTAGTGAATAATTACTATTCAAACCACTCAA
TCATTTGTACCATTCCAAAGCACCAATTTTATTTATGAAATTTATTTTGATAATTCCTCCAAAATTTAAATTCAAATTTGAATTCC
AACTCCAACAACATATGCAACATATACATGTTATTACTGCAACATGTGAACCACTACATATGCAACATTTGAATATTACTCATG
AAACATGATATGTTTTTATGCCATGATACTTGATGATGACATGATGAAGGCTTTATTTAGTGCTTAACACCTAGGGTGCTACAC
TTGGGACGTGTGGGTAGAAGTGAAGTCATGCATTGCATTGCATCATTTGCATTGCACTTGGAAGTATATTTATATTTTTACGAA
GCAATTGATGTCTCTATTATTATGCGATTTAAATTTGATGCTTAATCGAACTGTGATTTAAATAACTTGGTTGAAGCAGTTTGCT
TACTGAGGTTTTCTATAAACCTCATACGCTTGTTTCCCTATCTCAGGTACTTGATTTATGAAGATGCTTGGTGGGATGGAAGTA
GCAACATGTTTGCACACGTGTCAATATACACTTCAAACAATCAATTATGTTGTAATGAGTTCTCTTTGATGTTGGAGTTGTATC
TTTGCTTCCTTTCAAAGTTGCTAGTTGCAGCTCAACTTTGTTTATTGTTATGTCATCGCAGAAGACTTAATTTAATACTTAATCT
ACATGACATATTTTTTGCTTCCGCGTGGTCTGTTGTTGTAGTGTAGTGTGGTAATTTCCTAGAGGATTGTTGTGTTTTCTTCTG
AGGTGTTACACAATCATTATCCGGATCCTAGACTTACGACACGTTAGTAAAGCTGTCTCATTGCTAGCTCTAACACACCTGGC
AATAATAGGCCAGCAGGAATGTTGTGTTCTGTAGTAGTGCATCTTTGTTTCACATCCTTTCCCCATGCTGGTTGCAGCACATC
GCCTTACCCAGTGTTGCCATTATATTATTCCCTATAGTTTACGCGGTGCCTGTTAAATTGTCCTCCTCCCAGCACCGGCAATGC
AGGAACGAGGGCAGCAGGCTTCTTGCACCAGGAAAGCAATGGGGAGCGAGAGAAGCTCCTTGAGTGGAAATCTGACGCA
GAATTGTTCCACGTGCAGCTTCCTTTTTCTATACTCCCCCTGCGTTAGTGAGAAGAAGCTACTCTAGTCAAAAGGCCAAAGA
AAAATGGGAACAACCATCATCAAGCAATAATAGCATAGCTACTTTACGTCTTGCACCTGGATAGCAATGGGGAGCGAGAGAT
GCTCCTTGAGTGGAAATCTGACGCAGAATTGTTCCACGTAGGCTTCCTTTTTCTATACTCCCCTGCGTTAGTGAGAAGAAAG
AAGCTAGCATAGCTACTTTACGACTACACATGTCACATGGTCTAACGCACCTACAATTATTATTTGCACTATACTGTGCATCAC
TCGCGCTGCTTCATGTTATGTATCATTACATATGGTACGACTTACTACTGAGTCAATTAGTCAAGGTAACCGTGTATCATGTGGT
TCTCGATCAACTAGTGAATGACGCGTCTGTGATAACGTGTATCATGTGGTTCTCGATCAACTACTGAGTCAATTAGTCAAGTC
AACCGTGTATCATGTGGTCCTCGATCAACCAGTGAATGACGCGTCTGTGATAACGATATCTGACATAGTGACTTACTCACAGT
GAATGACTCGTCCTAAGTGTGTTTATATATCTCCAGAGCAGCAGGGGCAGCCTCATCACCAACACACATCTAGCATAT
>SsLRR-RLK368
GATTGGACAAATTTTGCCAAATAACAACGAAAAGTGCTACAAGAATTTTAGCCAAAAAATTTTGACATCTAAACCGGCCCTG
AGTGGAAATCTGACGCGGAATTGTTCCACGTACGGCTTCCTTTTTCTATACTCCCCTGCGTCACTACTGGAAACAGGCGCGC
CTATAATGCACTCGACAAAGGCTTTGCCGAGTACCGCACTCGGCAAAGCCCACACAACAAAAAAAAAAAGTCGGCAAAGGC
CACTTTGCCAAGTATTTTTTATCCAGCAAAATTCAAAACGAAAAAATCCCTTTAAAAATAGCAAAAAAAAATTTTACCGGGG
AGGCCCCATCGGCCAGCGCCCGCCCGTCTCCGTCGACGTCGCAAGTCGTGGCATTTTTCGCGTAAAATTCGTGCGCTACGCG
GCTGGCGGGATTCGAACACGCGACCTCTCCCTTGCGTGTAACCCCCTGTACCACTGCAACACACTATCACTTGTGTCTAGAT
TCCGTTTTGATTCCCCATATACTATACTAATCCAAGTGTAAATTGATTGTTTGGGGCCCTAAACGAATTCAAATCAAAAGGTTT
CAACTGCAAAGTTTCATAACTTTTTGAGATCTACAATTTTCATATAGGAAGTTTCTCCATCCAAGGTCGTTTACAAATTTGAAT
TTCAAATTTGAGAAATTCAAACATAGTTTTGCATGGCAAGATGATTTCAAATGAAATAGTTGTCAACTATAAAGTTTTATAACT
TTTCGAGATCTACAAAGTTTATTTTGGTTGTTTTTCCATCCGAGGTCTTTTGAAAAATTCGAATTTTAAATTTTTAAAATTTAGA
TGCAGTTTTGCATGACAAAATGATTTCAAATTAAAAAAATTTCAACTACAAAGTTTCATAACTTTTCAATATCTACAAAGTTTA
TTTTGGTTGTTTTTTCATCTGAGGTCTTTCGAAAAATTCGAATTTTAAATTTTTAAAATTCAGATGCAGTTTTGCATGACAAGA
TGATTTCAAATCAAAAAGTTTTCAACTATAAAGTTTCATAACTTTTCAAGATCTACAAAGTTTATTTTGGTTATTTGATCATTTA
TGCATCCGACATGGTGGTTTTAACATTGTTCACAAATCTTATATAAACTACAAGAGAGCTACGTAAGATTTATGAACAAATTTA
CTTTCACTTTGTCATATGAATAAATGACCAAAATAAAAAATGTTCATCTTGATGAGTTATATAACTTTGTAGTTGAAAACTTTT
TCATTAGAATTAATTTACTCCTTCAAAATGTGTTTTGAAATTTATCTTTGCCGAGTGAAAAAAAAAAACATTCTTGGCCGAGTG
TCAAAAAAAACACTCGGCAAAGAAGCTTATTTGCCGAGTGTCCGAGAAAAAACACTCGGCAAATCATTTACCACTCGGCAA
AGTTTGATTCTCCGGTAGTGCGTTAGTGAGAATAAAGAAGCTACTCTAGTCAGAAGGCCAAAGAAAAGTGGGAACAACCAT
CATCAAGGAGTAATAGCATAGAGCCTGTTCGCTTGGGCTAAAAATAAGCGGCTAAAAGTACTGTTGGCTGATTTATTGTGAG
AGAAAAATACTGTTGGCTGGCTGAAAATAAGCGGCTGAAAGCTCAAGCGAACGGGACTTTACGACTACACATGTCATGGTC
TAACGCACCTACAATTATTATTTGCACTATACTGTGCGTCACTCGCGCTGCTTCATGTTATGTATCATTACATATGGTACGACTT
ACTACTGAGTCAATTAGTCAAGGTAACCGTGTATCATGTGGTTCTCGATCAACGACTGAGTCAATTAATCAAGGCAACCGTG
TATCATGTGGTCCTCGATCAACCAGTGAATGACGCGTCTGTGATAACGATATCTGACATAGTGACTTACTCACAATGAATGAC
TCGTCCTAAGTGTGTTTATATATCTCCAGAGCAGCAGGGGCAGCCTCATCACCAACACACATCTAGCATATGCGTCTCCTGGC
CTTCCAGATCA
>SsLRR-RLK92
TATTTTTCAACCACCTCCGTAAATAGATTTTGCGAGGCGGTTTTTTTTTTGTAACCGCCTCGGTTAATAAAAAGTGCCCGTCTC
GGTTAATCCGAGGCATTAACGGAGGCGGTTGAAAGTGCTGCCCGCCTCCGAAAATAATTCCAAAACACCTCGCAAAAGATT
TTTTGTAGTAGTGCATCGTGCACAAGTTCCTACAAACCACGGAAAAAAAATTCAGTGAGGACCATCCCTACCATGACGCCATG
TAGATGTTTGGCTGACGTATGGCCCATGGGCAACTAATTCGCCACGCGTATGTTTGATACTGTGGCCTATATTTCAATTGCACA
GTCCTCACCATGTCTTGGTAGGGATGATCCTCACCGGATTTTTTCCCCAAACGACACCCCCAAGGAACTCTTAGATTGTAATG
ATAATTAATTGAGACTCTCCTTGATCTCCAGCGAGGTGATTGGGCAGCTCAACAAGGTGATGGAGGAACAGAATGATCTTGG
TGATAGCAAATTACGTTGAAATATGATTTCAAAGACCACCCCTCTCTATATATGAGAGGATCACGGTCAATTGAGAAATCTATC
AATCCGATCGATCAAAAACTAATCTACTACGTACCCTACTTTTTAACCCTAAACTTTTCCAATTCCAACCCCCCTTGCTGTCCAG
ACTTCTCCCGAGGAGATTTGCCAGCATTCTAGATGGCTATACGGATCCTAGGACACTCTTGCTATGCTCCTCCTCGACGACAG
GTCTTCCTAGAAAGTGTTCACAGGCTTTTCGGGGTAAAGAACGAGTATCAAAAGTACATAGCCGACTTTTCGATCGGCTTTAT
TATGTTGACCTCACTCTACTCGGGTAATCAAGTCCTAGCTAGTACGTGGCCATATTGCCGAACCATTTTGTTTTTTTTTTAGCTT
CATATTGCATCTCTACTATAATGGACCAATCAAAATAATACTAGGTATAATTTTTTTTAATATTAATATAAGTACACTATTCCAGC
CCTGTCTTATTTTATTTTTGGAATTCATATATATATATATATATATATATATATATATATATATATATATATATATATATATATATATAT
ATATATATATATATATATCTTTTGCTTCTGTCATTCACTGTGAATTTTGCTAATGTGTGGGAACTGATCTTGTGTATTTTGCTTGTG
AAGCCTTCTCAACATAATATTATAAGACAGACGACCTGATACATGGCAATGGCATACTCAGTTTATGGCAGAACAAGAGTCCT
CTGTTCTTTCTCCTGGGTTGGTAATGCAGAGGAAAATCATATCTTCAGTTAATATATTTGAGTCAAAATAGGTATGCGTGATGC
AGACACATTCTGCTGGAGGTGGATAGGATTTTGCGGCCAGTGGGTGCAGTCATACTACGGGACAGTGTTGATGTACTGAACA
TGGTCATATACAATTGTGTCAAAGTTGAACTCTGAAAAAAAATCACCATCTGATTATTTTCATGTACATCCCTGAAATGAAGC
CGTGGCATTAGCACGGGCACGTGACACGTCACAAATTAAACCGAAGGCATTGTTGCACCGGAAGACATTTAATTTGCACTGT
TTAGGTCAAGCACACCCCGGCAAGCACGGTAGATGGAGACCGGCCTGGTGGTGAACGGGTCTTGCAAGTTCATCAGAATCG
CTGTAAGGAAGACTTGAGCTTCCTTGAATTGAAGTCGTTCTAGTGTCGTTTGTGGTACAGCTACACTTTTGTTGTTGTCCGGC
AGAAGACCGGGTTAACCAAATTTGAGGTCAGGCGGCCAGAAGGCGAACTAAACAAGTGCCTTCCGAGAGCAATGCGCAGC
AGTCTTGGTTCAGCGTTGTCATCATCCAATACACAGTGCATGTTCTCCGAACGATCATTTAGCGCTATATATACACACACTCTC
TGGCCAAGCTGTTCGGGCAGTGTGACCATTTCTCCTATATATACCTCGAGCTACCAACAAAATAACAGAGAAAGCC
>SsLRR-RLK11
CTCAAAGCCCTTTTCCTCCTCTGCTGGAGACACGCCGCCGCACTGCATCCATCTGCAGATGAGGAGATGGTTGGAGAGGAAT
GACATGGGAGCAAGTGTGGGACTCTCACGGGTCATGGTTACTTGGATCTGCTCCATGGAAGGGAAAAGGTGGAGGAGACTG
AGCAGGAAGAAGATGGAGATGGCAGGAAGGAAGATGCCGGAGGCGCGCATGTAGATGGGAGCACGGGATCGGAGGAGAT
GAAATGAAGCCTCTCAGTGTGGGTGTCGTTAGTTTCTAACGAGTTAGAAATAGTACTGGCTCGTTTCGTACAATGAAATATTT
TCCTTCTCTCTCCTCATTAGTATGGACCAAAAACTCTATTTTTACTGATGTATCAATTTTATTTAATACATATAAAACTCTCATGA

CATCCCATTGAGACTCGCTAAGCCCCCACTGTCCTTGTTTTCCAGGCAGCTTTGGGAGGATATCAACCTTAGGAACCGTTTTC
AGGTTTGAGGGCAATCAATAGTCTCCAGATACAAGAATATAATGATTCTTACTTTTATTTTTGCGTACTGCAGTCAAAGAGGG
CGGCTTATTGTTTCCCGAGGGAAAGGGTGGCCGGACGTGGGTGAGGTGCTGATTGCGACATTGCAATTTGCGCTCATTTCCC
TGTCTCAGCTCTCAACTCTCACTCTCTCAGTGCTTTGCACGGCAAGCTGCATATAATAACTATATAAGTGCCCATCACGCATCG
GCTCCCAACCAAGCATGTCTTCACACCTGCCCAACTTCTACACACATTCTGAACTCCAGATGACAATGCTATCCAACAAAGT
GCAACCTTCCTCAAGGCCTCTGCTGTGCCTCCACCTATTGATCCTGCCATGCCTATGCCTTCTCTTCTCACAAGCACATGCAC
TGCATCATGGGGACACACCACTCAGATTTCAGAAGATGGCTCTGCTCCACTGGAAATCTACACTTGATAGCTTGCCGCCAAT
GATGAGTTCCTGGCAGGACAACACCGGCCCGTGCAACTGGACTGGCATCACATGTATGTCCATGCGCCACGGTCGCCGCCC
AACCAGCCGGGTTGTGACCAACATCTCCCTGCCAGATGCCGGTATCCATGGCCAGCTTGGTGAGCTCAACTTCTCAGCTCTC
CCTTTCCTCACACACATCGACTTGCATAACAATAGTCTCTATGGTCCAATACCAGCTAATGTCAGTGCATCATCCTCACTTCAG
TATCTAAACTTGCACCACAACCATTTCAGTCAGAGGCGGAGCCACCTATATGGCGAGGTTGGGCGGCCGCCCTACCTCCCCT
CCGGCGAGCACCACAGTTAGGACGCCCATCTCCGGCGGCGGCGCAGGTCCGAGGCTCCGAGATGGCGAGATGGCGACGGC
GACCAGAAGTTGGGGACGATGACTTGACGAGCGAAGTAAAGGGCGTGCGGGCCTCACTGCTTGGCCGTCTAGCTCTGGCC
AACAAGGCCCAAAGGCCACCCATCGCACAGCCCAAAGGCCTTAGGGCAGGCTGCAAACCAGGAGAGGCATCACGCTTGCT
CAGTCTCGGTTTGACGGCGCCTCCGCGGCTTCGTAGTGACGCAGTTGGTATTTTAGCGGCGCTTGATTAATTGTTCATCCTGA
TTACTGAAGCAATTAAATTCTGCTTATGTCTTTTCATGTGAAAGGTAATCGTGTTCCAACTTCTTTTGTACCATTTATTTGCTGA
ATTACATATTAGAATATAATTGCTACATGATAAACCGACATACCTAGTTAGTCCGATATATCGGTAAAGTTCTGTGTGTGTATATA
CAGTGGTCCGCCCTACCTCATTTTCTCGGCTGGCTCCGCCACTGATTTCAGTGGAAATATACCATATGAGATTGGTGGCCTAC
AGAGCCTCAGATTCCTTGAGGTCTCCTTTAACAACCTCACAGGCCCCATCCCTGCATCCTTGTGCAATCTAACATCGTTAACA
CGTCTTGTTATTCACCAAACC
>SsLRR-RLK43
TCGATCTGCCAACTTTGAATTGATATTAAGATATTCTCTGGCTTTACCGTTTCCATGGATACCGAACTTTCAACTAATGGTTAG
AACTGAGAGGATGTATATTGGCTCCATCTCTATGATAGGTCACCTTAGATAGTGACATCATATCAATCTGACTAGAGATCCTTC
TTATTTAAAACCAAGAACTCTGTGTATCCACAATCTTTGAGAAAGTTCTGAAAGTAGAATAATAACATCATTGGACTGTTCAA
ATGAACCGAGATCTAATAACGTTTTCAAAGTTTCAAATGCGAATCAAATAAATCCAGTCAGCTGTAAATAAGATGATGCCCTA
TTTTTTTTTGAAACTAGATGATGCCCTATTTGGTATAGCTTTTCTGAGAAGCTGCACACAGATAGAGAAAAAACATTATCAAA
ATAAGCTGGTCAGACCCAGTTTCTACGTTTTAGTACAAATGAAGTTGTTAGGCGAAGCGTGACAAAAGAAGGCTAAAAAAG
CATACCGTTTGACTTCAGTTTCAGTTTATTTTGCCAATAAAAAACTTTTGTTTCAGATCAACTACGACAATATAATTTATCAAC
CTTAGGAACCGTTTTTTTATGGAACATGAGGTGCCCCCCTAGTTCCCTACTGGAATTCTATTGGAAAAAAGAAGAACACTAG
CACAGTACAAGAGTTACAATCAGGAAACGAAGAAGAAAATAAAGAAAATTAAAGCAATTACACAAGAGTGTCTAGCCATCT
ATTAAACAAGGGCTGGTATCTGGCCTTAGGCTATCTCCAATAGCACAGACCCAAATCGGAGACCCATTCGATAGTTTGGGTG
GTGCACAGTAAAAGGGTCACGGGCCTCTCTCCTCATTCTCTCCAACAGCACACCCAAATCGCCCGTCCCTCTCTCCTCACTC
TCTCTCCCCCGCGCTGCCCATTCCCGTCCCTCTCTCCCTCTCTACCCCGGCGCCGGCGGCAGCACCCCTCCCTCCCGGATCCG
GCGGTTGCGGGCGAGGAGGACCGGCGAGATCCAAGGCAGGACCAGCGGAGCGGACCGACAAGATCCAAGGCGGGCCCCG
CGGTGCGGACCGGCGAGCAGGGGGCGGGGGTGGGGAGCGCGGGCGGATCCGAGCCGCCCTCGTCTTCTCCTCCGGTCACC
GCGGCGGCGGCGGGGGCCGAGCATCAGCAGTAGGCAAGGGTGGGGGCGCAAGCGGATCCGAGCCACCCTCGTCTTCTCCT
CCGGTCACCACAGCAGCGGCGGGGGGCCGAGCAGCAGTAGGCCAGTAGCAGGCGGGGGTGGGGCGCGAGCGGATCTAGG
GCTGCAGCGGCCGCCGCGTTGAGACGGCTCCGGCGACCTCGAGGCGCTTCTCTCACCTGACCGGCTCCAGTCGTCCCCTTC
CTCCTGGTGGTGGGCCCGCCTGCGTGGAGACGCAAAGAGACGGATAGAGGACCTTCATTTGCGTTCCCCGTCGCCTCGTAC
CCAAAATGGGTCACCTGTTTGGCTACTCTGTTAGAGCCTTGTTAATATGAACCAAAAACTCTATTTTTACTGATGTGTCAATTT
TATTTAATACACATAAAACTCCCATAACATCCCATTGAGACTCGCCTAAGCCCCAACTGTCCTTGGGGACACAGATCATTTTCT
AGGCAGCTTTTGGAGTTTTTTTTTGCTATTGATGTCAACCTTAGGAACCGTTTTCAGGTTTGAGGGCAATCAATAGTCTCCAG
ATACAAGAATATAATGATTGTCTCCAATCATTCGATCAAATGGAAGTCGTTCTGCAATAGTTTCTTACCTTTATTTTTGCGTACT
GCAGTCAAAGAGGGCGGCTTATTGTTTCCCGGGGGAAAGGGTGGCCGGACGTGGGTGAGGTGCTGATTGCGACATTGCAAT
TTGCTCTCATTTCCCTGTCTCAGCTCTCAACTCTCACTCTCTCAGTGCTTTGCACGGCAAGCTTCGTATAACTATATAAGTGCC
CATCACGCATCGGCTCCCAACCAAGCA
>SsLRR-RLK27
CTGTGTTGTGATCAAATGTGCCATGTGATTTATGTGGCAGTTCACACTTTAAAAACTAGGTGAATACCCTGCGCGTTGCCGCG
GAAATTGCTGATGATTTAGAATAAATTACTTATACAAAGGGATCAACAATTATAAGTTAATATTAGTGGATGACGTGGCACGCT
GATGTGAACAACTAAATGCATGTTAGTGGATGATGTGTCTTGCTGATATGTAGATCATAATTGCAAATGCTAGTTGGCTATAAA
TGCATCCGATAGTGGACTGATTAGGTGGACAGCTTATATGTTTAGAAAAATAGCTAGTGGTTTAGCTTGATAGAGTTTATAGAT
TACCAGCACTCACATTACGAACTGATCAGCTTCACACTCTAAAAATGCTGTTAGCACTCACGTTACGAACTGATCGGCTTCA
CACTCTAAAAATACAGCATTCACACATGACTGCATATAGCAGTTTCATATAGCGATGAAAACGGACGGAAACGGACGGAAAA
ACCCTATTTCTACTTCCGTTTCTATATTTTTTGGCGGAAACGGGATCGGGTTCGGAAAATGCGGGTTCGAAAACGGAATCGGA
ATATGCGGACATTCGGAAACGAACAAATACGGATGAAGAGCCGGAAAATTAAATTAAGATCACATAATATAATATTCTCAAAG
TATACATATATAAAGTCATGTAATAATATATTTAAAGTTTAATATTCATTTACATGCTATGTACATGAACATAACATGCATATTTGG
TGATTCGTAGTTATATTAACATAACATAAGGACATGTAATTGTTGAACATGTTGAGCATAGATTGAAAACGGGATAAAAACGG
AATAAATACGGGTCTATTCCATATTAAAACGGGATTACTCGGAAACGGACGGAAAACACCATTTTCTACTTCCGCAAATACGA
AAACGGGATCTCGCAAATACGGAAACGGACGGAAAAATATAGAAAACGGAACGGGACGGTACGGGAAAAATTCCGTCCGT
TTTCATCCCTAGTTTCATATTCAGTTTCACATAAAAAAATGCAGCATTCACACATGCACTGCGTAGGCAGTTTCATGTTCAGTTT
CACACCAAAAATTTCAGTTTTCACACCAGGCAACTAGAAGTGAACTGCAGCATCTCAAATGAATTAGCAGCACAAAAATTT
CTCCATGATCAGCAATTAGCATACTAGTCCAACTTAATCCAATTCATACAACAATTGCAATCAATTCTCAAAGCCCTTTTCCTC
CTCTGCTGGAGACGCGCCGCCGCACTGCATCCATCTGCAGATGAGGAGATGGTTGGAGAGGAATGACATGGGAGCAAGTGT
GGGACTCTCACGGGTCATGGTTACTTGGATCTGCTCCATGGAAGGGAAAAGGTGGAGGAGACTGAGCAGGAAGAAGATGG
AGATGGCAGGAAGGAAGATGCCGGAGGTGCGCATGTAGATGGGAGCACGGGATCGGAGGAGATGAAATGAAGCCTCTCAG
TGTGGGTGTCGTTAGTTTCTAACGAGTTAGAAATAGTACTGGCTCGTTTCGTACTCATGAAATATTTTCCTTCTCTCTCCTCAT
CAGTATGGACCAAAAACTCTATTTTTACTGATGTATCAATTTTATTTAATACATATAAAACTCTCATGACATCCCATTGAGACTC
GCTAAGCCCCCACTGTCCTTGGGGACACAGATCATTTTCCAGGCAGCTTTGGGAGGATATCAACCTTAGGAACCGTTTTCTG
GTTTGAGGGCAATCAATAGTCTCCAGATACAAGAATATAATGATTCTTACTTTTATTTTTGCGTACTGCAGTCAAAGAGGGCG
GCTTATTGTTTCCCGAGGGAAAGGGTGGCCGGACGTGGGTGAGGTGCTGATTGCGACATTGCAATTTGCGCTCATTTCCCTG
TCTCAGCTCTCAACTCTCACTCTCTCAGTGCTTTGGACGGCAAGCTGCATATAATAACTATATAAGTGCCCATCACGCATCGG
CTCCCAACCAAGC
>SsLRR-RLK116
TAATGTGGTTTGCTAATGTGAATAATGGGATGTGCTAGTGGAAGTTGACGTGGACACTTTGCATGGTGAGATAATTGCATGTG
CTAGTGGATGCTAAAGAGATAGATTTGCCAGTAGGGTTTGAAAATATAAGATATATAGATATAGATATTGATATAGATATAGATA
TGGTGTGTCTTTATATAGGTTAAGCAACTTTCAGTGTTTAATGATTGTTGCCTTAAATTGCCATATGGAATTAACATGCTTCTAT
TTTGCTCCATAAAAAGTATAAGACTTTTTTTAAGTAAACAATGCTATCGTGAGCCACCATACTAAATTTAGTTCCCCCCTTAAA
AAGAATATAAAGCTTTTGGTGATTATAAAGCATACAATAGACATATAATTATTTAGCAATATGCATCTATACGCTTAGATAAATAT
ATAACAGTTTTCGTGTCCCTGTAAAAGCCACCTTAAAAAGTTTATTCTATCATTCTACCATTTGAGAAAGAGAAAATAGCAAA
ATTTATCTTTGCATCTAGTAATACAAAAAAATGTGTGCATCATAACACTTCGATTATACTAGTGGTAGATGACCGAACAATATC

AGAACGTGGCAAAGTCAATAGTATCGCCATATGCCTCAATGTCATTGAACACATGGTCTTCATGGCTCTGCCCGTTGACGGCT
CTATAGAGTTATGACTTTGGGTGATAGGCTCATTATCTTCTATGGCAATGAGAGATAAGATTTGTGCTCATGTCCCTTTGACGG
TGGCCCACATAACACCATATATAACTCAAAACTTCATCTAATCATATGGGCCTAGAGTCGGTCACAAGACTTGACCCATCGAC
TAACAAATGAGTCCACGACCCAAACAAACGGTTTGGTCCCTTCTCCCTCCCGCGCGCCATTCAAGTTTGCTCGATTCACGAG
AGTCCATAACCTAAATAATCGGTTCAGTCCTTTCTTCCTCATGCGTATCCTAACTTGCTCGATTTATGGCCATATGCCTCACTAT
TACTACAAGCATCGTCTCGTGGCTCTGCACGTTGATACCTCTATAGAGTTATGACTTGGGGTGACAAAGCTCATTACCTTTTAT
GGCAATGAGAGATAAGAGTTGTGCTCATGTCCCTTTGACTGTGGCCAATGTATTGCCAATATGACTCAAAACCTCATCTACCC
GTGGGGATCTAGAGTCAGACACAAGACTTGGCCCATCGACCAACAAATGAGGCCACGGCCCAGATAAACGGTTCGGCCCCT
TCTTCCTCCCGCGTCTATTCAACCTCTCTCTATTCATGGTAAGCCCACGGCCCAAATAAACGGTTATCTCCCTTCTTCCTCCCG
CGTCCATTCAAACTTGCACGAGTCATGACGAGCCCACGGCCCAAATAATCGGTTCGGTCTCTTCTTCCTCCCGCGTCCATCCA
AACTTGCTTGATTCATGACGGCAACCATTAGAAAAAAAAAGCAGAAGAGGAAGAATGGGATATTCATAGCAGAACCAAAGC
ATGCTAAACTTTTTCCTATCCAGTCATCTGTCACTGATCATCAAATTATATTAGATGCCCGCGTATAGTAACGTACTAACATACT
ATCAAAGTATCAAGTATCAACACTTGTCTCTTAGCTCTAACCCTCGAAGCAATGTGCTATCGGTGTACAACAATGGTGTTCAC
CTAGCATCTCTTCTTGGTGGGGTATCCATTGCCATCTCTGAGTAGTACTGTAGTACTAGAATTTTGGACCCAAATGCACCGAG
CACAAGACCGCTTTTCCTCTCTCCCCTCTCCTCCACTTTCTCTCTCTAAACCACGGTCCCTCTCTCTCTCCCAGTCTCCCCCCC
GACGGAGTGCGAGTGGGAGCGAGCGGCGGCAGCAGCCACGGCCCCTGTCGCCGTGTGGTGTGCCGTGCGTGCCCCCAAAA
CCACACAGAGAAAAGCAGGCGCACGCACACGCCAGGCCTGGGCACCACCACTGCCTCGCCTCGCCAAGAATCCCCACCCC
AA
>SsLRR-RLK93
TGACGGCTCTATAGAGTTACGACTTTGGGTGATAGGCTCATTATCTTCTATGGCAATGAGAGATAAGATTTGTGCTCATGTCCC
TTTGACGGTGGCCCACATAACACCATATATAACTCAAAACTTCATCTAATCATATGGGCCTAGAGTCGGTCACAAGACTTGAC
CCATCGACTAACAAATGAGCCCACGACCCAAACATACGGTTTGGTCCCTTCTCCCTCCCGCGCGCCATTCAAGTTTGCTCGA
TTCATGAGAGCCCATAACCTAAATAATCGGCTCAGTCCTTTCTTCCTCCTGCGTATCCTAACTTGCTCGATTTATGGCCATATGC
CTCACTATTACTACAAGCGTCGTTCCGTGGCTCTGCACGTTGATACCTCTATAGAGTTATGACTTGGGGTGACAAAGCTCATT
ACCTTTTATGGCAATGAGAGATAAGAGTTGTGCTCATGCCCTTTGACTGTGGCCAATGTATTGCCAATATGACTCAAAACCTC
ATCTACCTGTGGGGATCTAGAGTCAGACACAAGACTTGGCCCATCGACCAACAAATGAGGCCACGGCCCAGATAAACAGTT
CGGCCCCTTCTTCCTCCCGCGTCTATTCAACCTCTCTCTATTCATGGTAAGCCCATGCCCCAAATAAACGGTTATCTCCCTTCT
TCCTCCCGCGTCCATTCAAACTTGCACGAGTCATGACGAGCCCACGGCCCAAATAATCGGTTCGGTCTCTTCTTCCTCCCGC
GTCCATCCAAACTTGCTCGATTCATGGCGGCAACCATTGGGAAAAAAAAAGCAGAAGAGGAAGAATCGGATATCCATAGCA
GAACCAAAGCATGCTAAACTTTTTCCTATCCAGTCATCTGTCACTGATCATCAAATTATATTAGATGCCCGCGTATAGTAACAT
ACTAACATACTATCAAAGTATCAAGTATCAACACTGGTCTCTTAGCTCTAACCCTCGAAGCAATGTACTATCGGTGTACAACA
ATGGTGTTCACCTAGCATCTCTTCTTGGTGGGGTATCCATTGCCACCTCTGAGTAGTACTGTAGTACTAGAATTTTGGACCCA
AATGCACCGAGCACAAGACCGCCTTCGTATCGTCTGCGCCGCTCCGTCCAACCACCTTTCCGCCTTTCCTCTCTCCCCTCTCC
TCCACTTTCTCTCTCTAAAACACGGTCCCTCTCTCTGATGACATCAAAATTCCAAGTTTTTTCACTCTCTTTCCATCACATCAA
TTTTTAGCCGCTTGCATAGAGTATTAAATGTAGGTAAAAAAAAATAACTAATTACACAGTTTAGTTGGAAATCACGAGATGAAT
CTTTTGAGCCTAGTTGGTCCACGATTGGACAATATTTATCAAATAAGACGAAAATAGTACTATTTATCGGTTTGAAATTTTTTC
AGCATCTAAACGAGGCCCCAGTCTCCCCCCGACGGAGTGCGAGTGGGAGCGAGCGGCGGCAGCAGGCCAGCAGCCACGGC
CCCTGTCGCCGTGTGGTGTGCCGTGCGTGCCCCCAAAACCACACAGAGAAAAGCAGGCGCACGCACACGCCAGGCCTGGG
CACCACCACCACCACTGCCTCGCCTCGCCAAGCATCCCCACTCCAAATGCCTTCCTCCTCCCCCTGCTACCGCCGCAGCGTC
CTCTTCTTGGCCCTCCTCTCGGCGTCCTTTTCGGCGCCGGGGGCGGCAGACGGCGCCGCCGGGGAAGCCGGCGGCGGCGG
GGATGCCGCGGCAATGCAGGCGCTGCGGCGGGCCCTGGCGCCGCCGGGCTGGGGCCCGGGCGCGGACCACTGCGCGTGGC
GCGGCGTCACCTGCTCCCCCGCCGCCGCCGGCGCGGTCACCGCGATCGAGCTGCCGCGCCGGGGGCTCCGGGGGGACTTCT
CCGCCGCCTCTTCTCTCCGCGCGCTGGCGCGCCTCGACCTCTCCGCCAACTCGCTCAGCGGCGCCCTCCCGCCGGCGCTGG
GCGCGCTCGCCCGCCTCGAGTTCC
>SsLRR-RLK136
TGTGCTAGTGGAAGTTGACGTGGACACTTTGCATGGTGAGATAATTGCATGTGCTAGTGGATGCTAAAGAGATAGATTTGCC
AGTAGGGTTTGAAAATATAAGATATATAGATATAGATATAGATATAGATATAGATATAGATATAGACAATGTTAGCCTAAACGTTA
AACGGTGAACAGTCGGTCACCCATCGTTTAGCAAAAAGTCGGTATACACGGCCGTTTAAACGGCCTTTTAAACGGTCGAAA
CGGTTTTGCATGGCTTTACACGGCTAATCGGCCTACACGGTTACCCATCGAGTAGCGTTTACACGCCATGTAAACGGCCTACA
CGGCCGTGTAGGCGAACAGTGGATATAGATATATATATAGATATAGATATGGTGTGTCTTTATATAGGTTAAGCAACTTTCAGTG
TTTAATGATTGTTGCCTTAAATTGCCATATGGAATTAACATGCTTCTATTTTGCTCCATAAAAAGTATAAGACTTTTTTTAAGTA
AACAATGCTATCGTGAACCACCATACTAATTTTAGTTCCCCCCTTAAAAAGAATATAAAGCTTTTGGTGATTATAAAGCATACA
ATAGACATATAATTATTTAGCAATATGCATCTATACACTTAGATAAATATATAACAGTTTTCGTGTCCCTGTAAAAGCCACCTTA
AAAAGTTTATTCTATCATTCTACCATTTGAGAAAGAGAAAATAGCAAAATTTATCTTTGCGTCTAGTAATACAAAAAAAAATATG
TGCATCATAACACTTCGATTATACTGGTGGTAGATGACCGAACAATATCAGAACGTGGCAAAGTCAATAGTATCGCCATATGC
CTCAATGTCATTGAACACATGGTCTTCATGGCTCTGCCCGTTGACGGCTCTATAGAGTTACAACTTTGGGTGATAGGCTCATT
ATCTTCTATGGCAATGAGAAATAAGATTTGTGCTCATGTCCCTTTGACGGTGGCCCACATAACACCATATATAACTCAAAACTT
CATCTAATCATATGGGCCTAGAGTCGGTCACAAGACTTGACCCATCTACTAACAAATGAGCCCATGACCCAAACAAACGGTT
TGGTCCCTTGTCCCTCCCGCGCACCATTCAAGTTTGCTCGATTCACGAGACCCATAACCTAAATAATCGGTTCAGTCCTTTCT
TCCTCCTGCATATCCTAACTTGCTCGAGTTATGGCCATATGCCTCACTATTACTACAAGCATCGTCTCGTGGCTCTGCACGTTG
ATACCTCTATAGAGTTATGACTTGGGGTGACAAAGCTCGTTACCTTTTATGGCAATGAGAGATAAGAGTTGTGCTCATGTCCC
TTTGACTGTGGCCAATGTATTGTCAATATGACTCAAAACCTCATCTACCCGTGGGGATCTAGAGTCAGACACAAGACTTGGC
CCATCGACCAACAAATGAGGCCACGGCCCAGATAAACGGTTCGGCCCCTTCTTCCTCCCGCGTCTATTCAACCTCTCTCTATT
CATGGTAAGCCCACGGTCCAAATAAACGGTTATCTCCCTTCTTCCTCCCGCGTCCATTCAAACTTGCACGAGTCATGACGAG
CCCACGGCCCAAATAATCGGTTTGGTCTCTTCTTCCTCCCGCGTCCATCCAAACTTGCTCGATTCATGGCGGCAACCATTGGA
AAAAAAAGCAGAAGAGGAAGAATGGGATATTCATAGCAGAACCAAAGCATGCTAAACTTTTTCCTATCCAGTCATCTGTCAC
TGATCATCAAATTATATTAGATGCCCGCGTATAGTAACATACTAACATACTATCAAAGTATCAAGTATCAACACTTGTCTCTTAG
CTCTAACCCTAGAAGCAATGTGCTATCGGTGTACAACAATGGTGTTCACCTAGCATCTCTTCTTGGTAGTACTGTAGTACCATA
ATTTTGGACCCAAATGCACCGAGCACAAGACCGCCTTCGTATCCTCTGCGCCGCTGCGTCCAACCACCTTTCCGCCTTTCCT
>SsLRR-RLK391
ACGTTTAGTACTCCATGTATATGTCTAAATATTCGATGTGATGGGTGAAGACAAAAATTTTTGAAAACTAAACCAGGCCTTAC
TTTGACTCGCCTTTCAATTCCTGCATTTGTGTCAGCAAATCTGGTTCACGTTTCCTACCTGGAACCAGTGATCACCAGTTTTA
CTAGCTAGGACAACTGGAAAGTCGCTTTCAGAATTAAGAGGATCCTTGTGTAGCTGAGCCAAGACTCAAGAGCACCAACCT
CTTTGACCCGGCACCTGGAGACTGCAAATGGAAACCCAAGGTCGATTTGCAGTAGCACACAAAAAGAATTTCAATTTGCAC
ATGAAACCCGGGAGAATCTTGGAATTTCAAAATGTTCTTACTTGCTCGTTCTGTTGGTCTAAGAAAACTACTTCTCTTTCATA
AATGGAGAACATCACAAAGCTTTAAAAAAAAAACTCGACCAATCTAAACATGCAGGTGCTACTAAAGCACTTGCAACCCTT
AGGCTACAAGCTCTTTTGCCCATCACAAAGCTTTCATCGCAATGGCTTTACATTTTATGATTTAGTCATCAACTATATGCTATAT
ATATGATTCAATAAAGTTGTCCTTGATTTGTGTACATTGTGGCATAGGAATCTCACAGAAACATTGAGGGGCCAGGATGAATA
TATAAATATAACCATTTTAAAAACTGTCACCTGTATTCTCATTTCACTTAGAACGGCAACCTCCATTCCATGCCCTTCTTCATGT
GCTAGTTTTTTTTCGTTTAGTGTGGAAGTTCAAAGCTCCTTTTTAATGATCTGTTCTCTTTTTGACCGAAGTCAGCAGGGGAA

ACCCCAATCTATTTTTTTTAATTTTCTTTTATTTCAGACCTTTTTAATTCGCTATAACGGAAAGTCGAATCCAGGCCTGCTGGAT
GCTACTGAGGTGCTCTAACCATTAGGCTAGAGGCCACCTAAAAATAGAGTAGCTTTAAAGCTTTGCAACAGACTAAACAAAA
TTTTTAAGTGAATTCCAAAGTTGCACTATCTTTTAAGCAAGACTTTATAGCTGCTTAGTTGCTAGTAGAACGTTAAAGATGCTC
TATTTTTAGCCATAGTTCAAAACTGCCAAATCTTCAGTGGAACTTACAGTTTCTACATTCTTAGCGGAGTTATAAAGTTACTCA
ATCTTTTTAGCAAGAATTAAAGTATGCTATGTTTTCATTAGCAATTAAGTGTGAACATTTTTTTCTAGAATTTGGAAGGTACTCT
AGAAAATACTTCCTCCATCCAAAAAAAAAGAATGCAACTATGAAGTTTGTGCCCGTCAAACTAGTTTAAGTTGACCAAATTTA
TAATAAATAATGTTAACATGTGAGTTTACTATGAAAGTAGTAGTTATTTTTGTATCATGATGTTAATATTTTTATATAATTTTAGCC
AAAGTTTATATTGATTGATTTCTCGAAAAACAAGAATTGTATTCTCCTGTGAACGAAGGAAATACCAAAATAGCTTTGACGAA
AAAATACCAAAATAGCTTTGACGAAACTCCTCCTAAATCTTTATGCTCCCTATAGAGCCCACTGCACAACCTTAAACATTAGT
CACTCATAATTAGCAAAGATAAACCACGCAAAACTCAAAGACGAAGAATACAGCTTTTGCCTGCAAAACACGAGAGCAGAA
AGTGCTGGACCATTTTGCAAAAAACTGCCAAGTTGTCCTCAAATTCTATTCGTTGCGGGCCCCACGGTCCAGCCTTGCTCGA
GGGTTTTCAGGCTTTCCGCTCCCACTAGCGTTTTGGTCGCGCTCACTCGCTCTCACAAGTCACAAGTCACACAGCAGTAGGC
GGTGTTGTACGTCATTGGTCAGTCAACGCTGGACCCAACGCAAACCCAAGAGAAGGGGCACCACCAGAAACACCTCAACA
AAATCGTGAGCAGACTTGGGTGGGTCTCCCTCTCTGAAGCATACTTCTCATTCTCAACAAAACCCATGGAGGAAGCCATGGG
CGAGCACACG
>SsLRR-RLK359
AGTCAGTTTGTTCCTGGAGTGAAGTCTACACCCGGTGTTCGGAGTTGTCTTTCCGCTGTGTTGCTCTTTAAGGTATATTTTATA
CCATTGTGTTATGTATATTAAGCATTTGTCTCTTGATATTACACTTATCTGTGGCTATATGTGAGATTTGATTACTTTATTTCACAT
ATAGTGTATGTCTGGTTTTATCCTTAAAACCGGGTGTGACAACAACCTTAAACATTAGTCACTCATAATTAGCAAAGATAAAC
CACGCAAAACTCAAAGACGAAGAATACAGCTTTTGCCTGCAAAACACGAGAGCAGAAAGTGCTGGACCATTTTGCAAAAA
ACTGCCAAGTTGTCCTCAAATTCTATTCGTTGCGGGCCCCACGGTCCAGCCTTGGGCCTTATTTAGATGCACCCTAAAAGTTA
AAATTTTTTCACTCTCTCCATCACATCAATCTTTGGACCCATGCATGAAGTAGTAAATATAGGTAAAAAAATAACTAATTACAC
GGTTTAGTTGGAAATCACGAGATGAATCTTTTGAGCCTAGTTGGTCCACGATTGGATAATATTTAACAAATAAGACGAAAGTA
GTACTATTCATCGGTTTGAAATTTTTTTCAATCCAAACGAGGGCTTGCTCGAGGTCGACGAAAGTAGTACTATTCATCGGTTT
GAAATTTTTTTCAATCCAAACGAGGGCTTGCTCGAGGTTTTTCAGGCTTTCCGCTCCCACTAGCGTTTTGGTCGCGCTCACTC
ACTCTGGGCATTCGGTTAACCGAAACCGATCGGTTCGGTTCCTCGGTTTTCTGAGAAATTCGGTTACCTGAGAAAGCAGAAC
CGATCGGTTCTTTAGAAAACTCGGAACTGACCAATTCCGGTTTCGGTTTTTCGGTTTCGGTTCCGGTTCTAACCGAAAAGAA
CCGAGTGAAATCAACACAGGCTTTACCGATCCAAGAAAAAAGAAAAAGAAAAGAAAAAGGCAACCGCTGCGCTACTAGCA
GCTCGCCCCATCTCGTGCACAGCCACCGCCGCGCGCCGTCACGCTCCTGGCGGCCGGTTGACCTCGCTCCTGGGTCCTGGC
AGGCTGGCGGCCGGGGACTCCTTGCTCCTGGCGCCCTGCGCTCCGGCAGCTGGCGGCCACGAGTGAGGGCTGGAGGAGAT
CCTCCCACAGGAGAGGGCGAGTGGCCGGTGGAGCTCCACGGTCCACGCCTCCACCCCTGAGAGAGAAAAGGGGTTCGGAC
GCCGGAGCCCAGAGGCCAGAGGAACGAGGAGGAGGAGAGCAAACGTCAGCGTGGTGGCGTGAACAGGGGCGGGACACA
GGAGGCGCCGAGGAGGGATAGCCGTGCGCCGCCAGGGGATGGGAGAGGGTGGGAGAGCTAAGCGGCTGAGAGAGGGATG
AGGGGGATTTTGGTTTGTATATATAGCTAGGGTTGTTATTGGGCCACATCTGTTATTGGGCCTATTTGTTTATTTCGGTGCTTTC
GGTTATTTCGGGTAACCGAGGCTGGGAACCGAATTAACCTTATAAATTTCGGTTATCTGCTGACAGGAACCGAATTAAGAAC
CGAAATTTTCGGTTTCGGTTAGTTCGGTTTCGGTTCCGGTTATTTCGGTTCGGTTTTCGGTTCTCGGTTAATTTTGCCCAGGGC
TAGCGCTCACTCGCTCTCACAAGTCACACAGCAGTAGGCGGTGTTGTACGTCATCGGTCAGTCAACGCTGGACCCAACGCA
AACCCGAGAGAAGGGGCACCACCAGAAACCCCTAAACAAAATCGTGAGCAGACTTACTGTCTCCACCCTCTCTGAAGCAC
ACTTCTCATTCTCAACAAAACCCATGGAGGAAGCCATGGGCGAGCACACGCAGCACTCAAAATCCTAGAGAGCGGAAAAA
GATATCGTCTTTTTCTTCCTTCCCCTGCTACTCGAGCTGGTGGAGGTCGGCTGGGATTGAGGAGGAGGGAAAGAGGCGAGG
CGGTTAATCGAGCGGGAGGCCAACAACTGGCGGGA
>SsLRR-RLK373
TCAAAATGTTCTTACTTGCTCGTTCTGTTGGTCTAAGAAAACTACTTCTCTTTCATAAATGGAGAACATCACAAAGCTTTTAA
AAAAAACTCGACCAATTTAAACATGCAGGTGCTACTAAAGCACTTGCAACCCTTAGGCTACAAGCTCTTTTGCCCATCACAA
AGCTTTCATCGCAATGGCTTTACATTTTATGATTTAGTCATCAACTATGGCTATGTTTAGATGATGGTGTAAAATTTTTGGAAGC
ACATTAAGTCTTTCGTTTTTATTTGGCAATTAGTGTCCAATCATGGGCTAATTAGGTTCGAAAGATTCGTCTCGTGATTTACAA
CCAAACTGTGTAATTAGTTATTTTTTACCTATATTTAATGCTCCATGCATGCGTCCAAAGATTCGATGTGACAGGGAATCTTGTA
AAATTTTTGGAACTTTGAAGCAACTCAACACTGCCTATATGCTATATATGATTCAATAAAGTTGTCCTTGATTTGTGTACATTGT
GGCATAGGAATCTCACAGAAACATTGAGGGGCCGGGATGAATATATAAATATAACCATTTAAAAACTGTCACCTGTATTCTCA
TTTCACTTAGAACGGCAACCTCCATTCCATGCCCTTCTTCATGTGCTAGTTTTTTTTTCGTTTAGTGTGGAAGTTCAAAGCTCC
TTTTTAATGATCTGTTCTCTTTTTGACCGAAGTCAGCAGGGAAACCCCAATCTATTTTTTTAATTTTCTTTTATTTCAGACCTTT
TAAATTCGCTATAACGGAAAGTCGAATCCAGACCTGCTGGATGCTACTGAGGTGCTCTAACCATTAGGCTAGAGGCCACCTA
AAAATAGAGTAGCTTTAAAGCTTTGCAACAAACTAAACAAAATTTTAAGTGAATTCCAAAGTTGCACTATCTTTTAAGCAAG
ACTTTATAGCTGCTTAGTTGCTAGTAGAACGTTAAAGATGCTCTATTTTTAGCCATAGTTCAAAACTGCCAATTCTTCAGTGGA
ACTTACAGTTTCTACATTCTTAGCGGAGTTATAAAGTTACTCAATCTTTTTAGCAAGAATTAAAGTATGCTATGTTTTCATTAGC
AATTAAGTGTGAACATTTTTTTTCTAGAATTTGGAAGGTACTCTAGAAAATACTTCCTCCATCCAAAAAAAGAATGCAACTAT
GAAGTTTGTGCCGGTCAAACTAGTTTAAGTTGACCAAATTTATAATAAATAATGTTAACATGTGAGTTTACTATGAAAGTAGTA
GTTATTTTGTATAATGATGTTAATATTTTTATATAATTTTAGCCAAAGTTTATATTGATTGATTTCTCGAAAAACAAGAATTGTATT
CTCCTATGAACGAAGGAAATACCAAAATAGGTTTGACGAAACTCCTCCTAAATCTTTATGCTCCCTATAGAGCCCACTGCACA
ACCTTAAACATTAGTCACTCATAATTAGCAAAGATAAACCACGCAAAACTCAAAGACGAAGAATACAGCTTTTGCCTGCAAA
ACACGAGAGCAGAAAGCGCTGGACCATTTTGCAAAAAACTGCCAAGTTGTTCTCAAATTCTATTCGTTGCGGGCCCCACGG
TCCAGCCTTGCTCGAGGTTTTTCAGGCTTTCCGCTCCCACTAGCGTTTTGGTCGCGCTCACTCGCTCTCACAAGTCACACAG
CAGTAGGCGGTGTTGTACGTCATCGGTCAGTCAACGCTGGACCCAACGCAAACCCGAGAGAAGGGGCACCACCAGAAACC
CCTCAACAAAATCGTGAGCAGACTTGGGTCTCCACCCTCTCTGAAGCACACTTCTCATTCTCAACAAAACCCATGGAGGAA
GCCATGGGCGAGCACACCCAGCACTCAAAATCCTAGAGAGGGGAAAAAGATCTCGTCTCGTCTTTTTCTTCCTTCCCCTGCT
ACTCGAGCTGGTGGAGGTCGGCTGGGATTGAGGAGGAGGGAAAGAGGCGAGGCGGTTAATCGAGCGGGAGGCCAAGAAC
TGTCGGG
>SsLRR-RLK311
AGTAAATTTGTATCTGCACATCTAGACTCGGATGGTATGTAATGCGGACACAAGGTTTTATACTGGTTCGAGCAAAAATGTCC
CTATGTCCAGTGGATTATGCTGCTGCTTGTGTTGCCTTACACCGAAGTGCTCGTAGTAGGGGTTATAAGCTTGAGAGAGAGA
GAGAGCGGATCCCAAGTCTCTTTGGTGATGTGTGAGTGTGAGTTCTTGATCAGAGAGGTGGATTTCCAGCAGGGGTCTGTTT
GGATCGGGGTGTCCCTCTAGACGTGGCCCCTGTCTCCCCTTTTATAGTTTCCAAGGGGAGGGTTCGGGTTACATTTGTGGTAA
AAAGACGAAATGAGGAAAAGACAAAAGAGAAAAACAAAAAGGTGGCCTCGGGTGCGGCTGTCTTCCTCCTTATTCGGCGT
GAGCAGTTTACCCTGTAGCCAGAGACGTAATGAGCGTCGAGCTTTTGGCCTCTCCGTCCACTGGTGAAATGTGCTGTGGCCA
TGCTTCGTCCTATGTGCTTCTGTACCATCGAGGATGGGCGGCACGTGTGCCCGAATTTGCGTAGAGAGGGAGTTAGGTAGTAT
AATTGTCGTTACCTCTGTTATTGTAGAGGCATGGATGTCGTTGTGGTGGAATGACCGGAGTCATGACAGATCATAACGCGGCT
CCCATGCTCCCGTTCACTGTGCATGGTCATGCACGTCCCATCATTGGGAATGAGTGCCGTGGTGGGGGCCCATGTGGCTCGT
GGCCTTGCCAGAGGGCCTTACACGTCCCTCGGGCGAGACGGAAATATGCCAAGGGGGGCGGGCGCTCCCGTACTTACGTTG
CAGGGGTCGGGCGAGGCGGAGATCATTCCGGGTGCAAGGGTCGGACGCTTTAATGACGTCATAAGGGCTTGGGCGAGGCG
GAAATCAGCCCTAAGCCACGGGCGACTTAACAGCTGACACGCTTTTAATGCCCTTGGTTGAGTATGCCTAATATCGGTACCCG

ACAACTTTGATTTTTTTGGTACATTTAACCTGCTATGTATCTAGATATACGTTATGTCTATATCTAGATATATAGTAAATTCGATGT
ACCAAAAAAAGTTAAAACGGCTTATAATTTGGAATGAAGGGAGTATATTGGTATAGGATAAGCTTGTGTTTAGCTATAAGATG
ACCAGAACGTCGTTTCAAAAAGAAAAAGATTGACCAGAACAGATGGTTCCATACAACATAAAGAGTTGTATCGTTTTCTCTT
ACTCTGATCGTGGGCCCCGCTTTAATGTATATTTGTCGCCACTAGGTGACATATGTATATGCGCCAGAACAGCAAGCTTTGCTC
ATCTAATAATAAATTAATAATTAAATATAAGACGAATTGACCAGTCCAGAACCGTTACAACTTTTGTGATTAAAAAAAGGTTAC
CAACAGGGCTGCTCACTAACTAATACATCCACGTGATAGATTAGAAAACAGTAATAAACTCTTAGAAAAGCATCAAGGGCAG
GCTTCATCCATCGCTAAAACGTACTCCGTACGTCTGACTTCGATAGATAGGATCAGACTACTAGCCCATTGGTTCTTGTTCGCT
GAAATTCCTGGCCTATGGATGCAGAGACGCAGTTCCACTACTAACTAAGACAATTTTCGTGAAATTGCTTGAGTATTTCTTTG
GCACCCAAACGCGCCCGAATCCCGGATTAAAATGCCGCCAGTGTGAAGCGAAATTCAAAAACCCACGTCGTCCTGTGTGAA
CTTGCGCCGCGCAGGCCACACGGCCCACAGTGTGCGAACGCGACGCGAATGGACCGTGGACGCGTCCCCGACCCAACCCG
CAGCCGCACGGATCCCGCCGCGGCCACGCGCCACACGTGTCGACAGGCACAGCACCCTCGCCCCCGGTGTGTACCCGTGTG
AACGGATCCCCAACCGCACTACTGATTGCCGCAACCACCCCCTCCGCTGGACTCGCCCCACAAAAGGCAGGCGGCGCGGGC
GCGGCTGCCTAAAAAACGGCCTCG
>SsLRR-RLK340
CTATACTCTATAGTGGCGGGCCCCACTAGCTAAATAAAGAGAAAAATGCTATAACTCACAAACCAAAAATCCAAATTAAATTT
TGATTGCACCATTAAATTCGTCTCAATAAATCCTTCAAAACTAGATCACACATGGATATATTTAGATAAAGTGACAAGTGGGTC
CCACAGTGGATAGAGTGGATAGATGATGATATAGAGGATGGATGGGTGCGGGAGAATTGAGTATAGAGGAGAGAATCTTACT
GACTAGGACATATAGAAGATGAATATAGAGGTCCATAAGTGCGGATAGTCTTAACTAAGACGATTTTCGTGAAATTGCTTGAG
TATTTCTTTGGCACCCAACGCCCCCAAATCTCCGATTTAAAATGCCGCCAGTCTGAAGCGAAATTCAAAAACCCACGTCGCC
CTGTGTGAACTTGCGCCGCGCAGGCCACACGGCCCACAGTGTGCGAACGCGACGCAAATGGACCGTGGACGCGTCCCCGA
CCCAACCCGCAGCCGCACGGATCCCGCCGCGGCCAGCCGGCCACGCGCCACACGTGTCGACAGGCACAGCACCCTCGCCC
CCGGTATGTACCCGTGTGAACGGATCCCCAACCGCACTACTGATTGCCGCAACCACCACCTCCGCTGTACTCGCCCCACAAA
AGGCAGGCGGCGCGGGCGCGGCTGCCTAAAAAAAGGGCCTCGCTCCTGCCTCCGCCAAACAGTCCACCGCGCTGCCGGTG
CTCTCTCTCTTTCTCGTCCGCCATGGATGCTTCTAGTGCTACTAGCGAAAACTAGAGCAGCCCCTGCCGGCGGCCCCCATTTC
CCCAGGATCCTCTCCTCTCGCCCGCGGCCGCGGGCACCGAGCTGCCGTCTCCGGTAAGCGCCGCTCTCGCTCGCTTCGCCCC
TGTAATCAGTTCCCGATCGAGCAGTTGCCGACTGTAGTTCCTGGTGTGTGCTGCTTGTTGTTTGTTTGAACCCGAGGAACCG
CCGCTCCTTCATTCGTTTCGTTCCTGCTGTCTTGTCTGTTGCATTCGCATCTGCTTGGTAGTTTCTCCGGTTCTTGCTGGCAGC
GCGGGCTCTCGAATTTAGAGAGTGTTGTTCAGGAACGAAGCGAATTCGATGGCTGTTTCAATCGTAGCCCACTGAAATGAGC
TTTCCTGGGTGCCGATTTCCCTGAGGAATCCTTCTGCTGTGCCTAGGAAGCTTCTTAATTATTGTGTCCTGCCGTCGGCGTAG
TAGTGCAGAGGATCTCTCCCTTGCAATTGCAATCGAGGCGCCCATTTTCTTTTTGTTTTTCTAAAGAGAAGAAATTGAGGTGC
TCATCATCAGCCCATGTGGTGGTGCCCAACTGCCCATTGTTTTTAGAAATGGGGAAATGCTTCTGTGTTCGAGATCGGATCCC
ATCCCATCTCGTTGATTAGTATGGCTTATGTTAAACAGTAAGAAAAGAAGCCCGTCATAAACTCAATTTCTGCCCCTTTTAGG
AAAATGCGAGACTCCGTCTTCCTGCTTCATTTGCAGTGGCTTCGGAAACCATCCAGCGTCCTGAGTATAATTAATAATTAAGT
TTAGCATTGGGTTTTGGTTTTGCCATATTTGCATTTTGCTTCTCTCACAGTATGATCATCGAAACTACCGGTAGGTGTGCGTAG
CAGTTTGGGGGGCTCTAGTTAACCAAACCCGTTTATGAGTATATTCTTCTGTCATATACTATATTACTATGTGTTTTTACATGAG
TACGTTCTACTTGGTGTTAGTATGTGTCCCAGGACAGTAGGCGGGGCTTCCTTCAAGATTGATAAATGATAATGTTACAAAAG
GATTTCTACCATCTTTTATTGGGAAAAATAAGTAACTTGTAGTTTCATCAAATTGTTCTACAACGTTGCTTTTAGGGGGGAAG
GTTACCAACGTGCACAATTTTGATGATGACAACCGTTCATACATGCAATCCCCATTTGATTGTGCGTTGAACTACTTGTACATA
GGACAGAAAAAATGATC
>SsLRR-RLK322
ACATCACTAGTAGTCTCTGCTGTGAGATTATTAACCCCATTTCTCTTTTGCCTACGTGGACAATAATTCTGAGTGAAGCCAAC
GACCTTGGAGAAATGGGGCACACTCATACCCTGTGCTAGTATGCTCACGGCGACACGCGCGCACGCTGCTCTGTTGACCAA
CTTTTCAGTAGCTGAGCCGCCGATCACACGTGAACTGTGGGGACTGGGGACTGGAGGGTTCAGACCTCATTTTTCAACCATG
ACTCGAGAAATTCATCAGTGTGCACGGGCACGACTATACGCGTACAGGCAAAAAAACTTGCTTGATGGTGGTCAAGTCATGT
CTTCGACTCATGGAGGAAAAGACAAACGGATATGCTCAGTCGTGTCCGTACGGGCAACAAAAAAAGAACTCAGCCATGTGC
ACGGACCGGGCGCACGCTGCTCCGGACGGCAATGATATGATCACGCGCGTGAAAACCTTACGAGTTGCGACAAAAGCAGC
GACGATCCATACTGTACTAGCGTATGCATACAGATGTTCTTTAGCTCTTACCAACAACTGTCTCTAGTGCCTTAGGTCGGTTGG
CACTTAATAGAGGCTTTGATGGAAAAGAAAAATCTGTCGATAGCCTTTTTAATTAGTCAGATCTTTAAATATACTCCTCCGTTT
CAAATTATAAATCGCTTTGACTTTTTTATTCATCCATTTTACTATGTATCTAGATATATACATATCAAAATAGATATATCAAAAAA
GTCAAAACGACTTATAATTTGTAATGAATAGAGTACTATGCTTTATTTTAGATTTCTTGCTGGCTAAAGATAGAGAGCAGGACT
AGCTCTCTATACTATGCATACGATATTTGAGGATGAAAATATATAAAAATAGTTTATTCAAATAATATAAAAGATGATTTAGAGA
TTATGTTAAGAAATACACTTGAAAATGCTCTAAGCCATCTGATACACTCCATCTGTTCCAAATTATAAGTCACTTTGATTTTTT
GGTACATTTAACTTGCTATGTATCTACATATACGTTATGTCTAAATATATAGCAAAGTGGATGTACCAAAAAAGTGAAAATGGC
TTATAATTTGGAATGAAGGGAGTATAATAGTATAGGATAAGCTTGTGTTTAGTTATAAGATGACCAGAACGTCGTTTCAAAAA
GAAAAAGATTGACCAGAACAGATGGTTCCATACAACATAAAAGAGTTGTATCGTTTTCTCTTACTCTGATTGTGGGCCCCGCT
TTAATGTATATTTGTCTCCGTGAGGTGACATATGTATATGCGCCGGAACAGCAAGCTTTGCTCATCTAATAATAAACTAATAATT
AAATATAAGACGAATTGACCTGTGCAGAACCGTTGCAACTTTTGTGATTAAAAAAAAAGCTTACCAACAGGGCTGCTGACTA
ACTAATACATCCACGTGATAGATTAGAAAACAATAATAAACTCTTAGAAAAGCATCAAGGGCAGGCTTCATCCATCGCTAAAA
CGTACTCCGTAGGTTTGACTTCGGTAGATAGGATCAGACTACTAGCCCATTGGTTCTTGTTCGCTGAAATTCCTGGCCTATGG
ATGCAGAGACGCAGTTCCACTACTAACTAAGACAATTTTCGTGAAATTGCTTGAGTATTTCTTTGGCACCCAACGCCCCCAA
ATCCCCGATTTAAAATGCGGCCAGTCTGAAGCGAAATTCAAAAACCCACGCCGTCCTGTGTGAACTTGCGCCGCGCAGGCC
ACACGGCCGACAGTGTGCGAACGCGACGCAAATGGACCGTGGACGCGTCCCCGACCCAACCCGCAGCCGCACGGATCCCG
CCGCGGCCACGCGCCACACGTGTCGACAGGCACAGCACCCTCGCCCCCGGTGTGTACCCGTGTGAACGGATCCCCAACCGC
CCTACTGATTGCCGCAACCACCCTCCCTCCGCTGGACTCGCTCCACAAAAGGCAGGCGGCGCGGGCGCGGCTGCCTAAAAA
AACGGCCTCGCTCCTG
>SsLRR-RLK306
CTCATTCCATTGTTGGTCCTCAGATGGAGGGCGGCAGCGGAGCGGCGTAGCTACGAGGAGTGGTGCAGGGCACAATGGTCA
CGGCGTAACTAGCGAGTATGCTGTTCGGCGGTGGCGCAAATCGACGATCGATTGGTGTCCTTCGGCTTGTGTTTAGGCTTATC
TTTGAGTCCTCGTGTTGGTTCTTCTTGTTTTTCTTTAAGTGTTGTTGTTGTTGTAATCTCTCCCTCCCTCTTTGTACATCTCTGG
TCTTATTGTATTTGGATTGACGCCATGCCCTTCCACCTCCTGGCTTGCTCGGCATCTATAAAATAAAAATTTAGGTGGAGATAG
TTCATCCCCGATGACCTTTCCAAAAAAAACTCAAATAGGTGACTTGTGTGTGTAACTTTGTGAAGTGAAGGATGTGCCAAAC
GCACACGCGAGCGAAGCCAAGTCATGCACGGGCCCGTAGTGGGCTAGTTTTGCCACTTGGGGCCGATTGATGGTGCTATCAT
ATGACTCGGTCCACCTTCTCTGTTGCTGCTGCTCCCGATCATTCCTGTTCTGTTCTAACACTGGTTCGCACCGGAGTTGGATT
AGTTATTTGAATTAGAACAACACCACACAACGCAAAATAAAAGAATCTCAGATGCGTTAGTTATTTGAGCACATCCAGTGCA
GTCTGCCAGGGGATCATGCATAGAAGAAGCACTTGCAGCACGGTTTCTGTCATCTAGAAAGCATGCATCAAAAAACTTTTCA
GAAAAAGAAAGCCAGAAACAAACAAACTCTCACTCTCACCAATTCTGCTGTAGGCCCGGCATGCCAGTACTGCTAGTAGGC
CCATTTGAGCACTAGCCCTCCTTCACAACCACCCCCACCGCAACACCACCAACATTTGCCTCTGCCACCCTCTACGAGCAGA
GTGCACTTGTCCTAAGTGTCTAGTGCTGAATAACGACGACGGTGATGATTGATGATTGATGAACCAATCAGCACATGTTTTTG
GTCATAGGATAGCTTATTAGCCCTTGTTTAGTTTTAAAAACTTTCTCAAAAAGTGCTACGGTAGCCGTCACATCGAATCTTTGG
ACGCATGCATGAAGCATTAAATATAGGTAAAAAAATAACTAATTGTACAATTTGTCTATAAATGTCGAGATGAATCTTTTGAGC

CTAATTGGTCCATGGTTGGACAAATTTTGTCAAATAACAACGAAAGTGCTACAGGAATTTTTCAAAAAAAATTTGCAATCTA
AACACGACCTTAGTTGCACTGGTACAAACCTTAATCAGCAGGCATGCAGGTCAGCAACTTTGGGCATCCCCACCAGATCTAT
GGTGAGGTGCGGTAGGCTGACAAAGGGTAATGCCCATCCAGACATGATGACCAACCTTTTTTTGGGTTCCATGTAAAAAACA
GTCAGATGACCATCAAGCAAAACTACTGTCCTCCATTAACCTGTTCCTCAGTTTACTGTAGCAACTTTGAGCTTCCGTTTTTT
ATGAATTTATTTATAGTTTAGAAATTAGAATGCATAAAATTATTGAAGTATCACAAACACATGTTCCCCAGTTTACTGTTGCAA
CTAGAGGATCCTGCACGGCTGCACCTAAGCCCACTGCCAAATCTAGTTGCGTCCACAGATCATCAGCGGTCACAAAGGCAG
GGCAAAGATTCTGAGCCCTAGAAAAAAAATCCAACTCGCACAGATGAATTCCTCCTGTGGATATCACCTGCACCCTCACCGG
TTCTTGTTCCTAGCATCTGTGCTTGGAATATTAGCCGAAAGCTCAACCACCGCCAGCAACTTGCTCTCGGATAGCCTGATCAT
CACAGTCAGACTCTAGAAAGTGGATTCAGAAACTGTATTCAATTTAAAAAAAATCCACAGATGGTGCAGCCTGCAAATCTGC
AATGCAGCAACACACAAAATTTAGACCCAACGGTAATTTTAAATTACGCGCGCCATGGTAATCGCTATCAGAGGTCAGAGCT
ATGTATATGCATATGCTTT
>SsLRR-RLK339
GTCAGCCATTGTCGATGTTTCGACGAGTGTCCTCCTCTGGCGTCTCTGCTCCGTCGGCGCTCTCCTTGATGGTGCCAGAAAAT
CTTATGTCGGGTGGTGTTTCCACCGCTGCCACCATTCCCCTTCATGGTTTCCTCCTCGAGGACGGCATTGAATGGTGGTACGT
GGGCCCGGATGTGCCGTAGGTGTCATCATCAGCTTCAAGGGAGGTTGCCGTGTCTGGTGATCTTAGGCTTGTGTGTCCGTCA
CCACTCTTACTCCCCATTCCACTGTTGGTCCTCAGATGGAGGGCGGCAGCGGAGCGGCGTAGCTACGGGGAGTGGTGCATG
GCACAATGGTCACGGTGTAACCGGCGAGTATACTGTTTGGCGGTGGGGCAAATCGATGATCGTTTGGCGTCCTTCGACTTGT
GTTTAGACTTAGTCCTTGTGTTGGTTCTTCTTGTTTGTTGTTGTTGTTGTTGTAATCTCTCCCTCCCTCTTTGTACTTCTCTGGT
TTTATTGTATCTGGTTTGGCGTCATGTCCTTCCACCTCCCGGCTTACTCGGCATCTGTGAAATAAAAATTCAGGTGGGGATAGT
TCATCCCCAGTGACCTTTCAAAAAAAAAATTCAAATAGGTGACTTGTGTGTGTAACTTTGTGAAGTGAAGGATGTGCCAAACG
CACACGCGAGCGAAGCCAAGTCATGCACGGGCCCGTAGTGGGCTAGTTTTGCCACTTGGGGCCGATTGATGGTGCTATCATA
TGACTCGGTCCACCTTCTCTGTTGCTGCTGCTCCCGATCATTCCTGTTCTAACACTGGTTCGCACCGGAGTTGGATTAGTTATT
TGAATTAGAACAACACCACACAACGCAAAATAAAAGAATCTCAGATGCGTTAGTTATTTGAGCACATCCAGTGCAGTCTGCC
AGGGGATCATGCATAGAAGAAGCACTTGCAGCACGGTTTCTGTCATCTAGAAAGCATGCATCAAAAAACTTTTCAGAAAAA
GAAAGCCAGAAACAAACAAACTCTCACTCTCACCAATTCTGCTGTAGGCCCGGCATGCCAGTACTGCTAGTAGGCCCATTTG
AGCACTAGCCCTCCTTCACAACCACCCCCACGGCCCACCGCAACACCACCAACATTTGCCTCTGCCACCCTCTACGAGCAG
AGTGCACTTGTCCTAAGTGTCTAGTGCTGAATGACGACGACGGTGACGATTGATGAACCAATCAGCACATGTTTTTGGTCAT
AGGATAGCTTATTAGTTGCACTGGTACAAACCTTAATCAGCAGGCATGCAGGTCAGCAACTTTGGGCATCCCCACCAGATCT
ATGGTGAGGTGCGGTAGGCTGACACAAAGGGTAATGCCCATACAGACATGATGACCAACCTTTTTTGGGTTCCATGTAAAAA
ACAGTCAGATGACCATCAAGCAAAACTACTGTCCTCCATTAACCTGTTCCTCAGTTTATTGTAGCAACTTTGAGCTTCCGTTT
TTTATGAATTTATTTATAGTTTAGAAATTAGAATGCATAAAATTATTGAAGTATCACAAACACATGTTCCCCAGTTTACTGTTGC
AACTAGAGGATCCTGCACGGCTGCACCTAAGCCCACTGCCAAATCTAGTTGCGTCCACAGATCATCAGCGGTCACAAAGGC
AGGGCAAAGATTCTGAGCCCTAGAAAAAAAATCCAACTCGCACAGATGAATTCCTCCTGTGGATATCACCTGCACCCTCACC
GGTTCTTGTTCCTAGCATCTGTGCTTGGAATATTAGCCGAAAGCTCAACCACCGCCAGCAACTTGCTCTCGGATAGCCTGATC
ATCACAGTCAGACTCTAGAAAGTGGATTCAGAAACTGTATTCAATTTAAAAAAATCCACAGATGGTGCAGCCTGCAAATCTG
CAATGCAGCAACACACAAAATTTAGACCCAACGGTAATTTTAAATTACGCGCGCCATGGTAATCGCTATCAGAGGTCAGAGC
TATGTATATGCATATGCTTTCA
>SsLRR-RLK28
TTGGGCTAGAGAACACTGTAGATGAAGACGATCACTCCATGAGACATACATGGAGACGTACGGGCTATGGTGTGGTGATGAC
TGAGAGTCATGACACGAGAACATGTAGGGAGTACATGGGAGTGTGTGGTCACGACAGCATTCGAGAGGTGCTAGATGTGCA
TGCGTGGTGCAGGAGACCGGGTGGTATTTGTTGGTACTGTGACTTGGTCAGGTAGGCATGCAGACCAGTGCATGTACGTTAG
GACTAGTGTTTGGCTCATGTTGGCTAATGGAGGAGCAGCTTGGGTGGCCGGTGGCGCCATCAGAATTTGACCAGGACTTGG
CAATTGGCAAGGAGAGGCAATCCGTGCTCATGCAAAGCTTTGTTGGTTTTGTGGCGTAGAGCTCGGTCAAGGTGCAGACCA
TGTGAATGGTTGTACGTGAATTTGAGGCTAAAATCAGCATGCGTAGGGACTGCTGTGATAGGCTATAAAAGGAGGACATTGT
GTCTGTATGTAGTGGGGAAAACAGAGAGGAGTGTGACGACAAGAGAAAGAGTTTTTGCTGGCTATTTCAAGGTTCAGTAGT
TTGTTTAGAATTCCTTTGTGTGGATGCTTGTAAGTTGTATCTTGTGAACACATTATCAGAGTAATGAAGTCCAGTTCTCTCATA
TCGATGTGTTGCTAATCTCGTTCTACAGATCAGCTTCTTGATTTACATGTCTAGCTTTTCGTTTTAGATTGATATCTTGTTTGCA
AGTTTAGACATGTCTTTTGTTCGATCTGAACTGTGCCAAGGCACTGTGCTCGTAATCTGAAAAGTCTCATTGCATAAGCTTTA
ATAGATTTTGCATAATCACAAAAATAAAGCTGAACCAATTCTCGTCACTATCAGACACAGTGACATTGTTTATTTTATATCTCA
AGTTCTAGACCTCCGATTGAGATGATTCTAGTTGGGTTCATTCACAAATTTCATATAGTTTGTTGTGTTAAATTTTGAGATCAAT
TGGAGCACATTTGGTGGTCTGAGTTTATTTTTATTAAAGGCACAATGTTCTGTCTAGAATTAAAAAAATGAGGGTTAATTCGG
TTTTGTGTTTAGGGTGAAGTTTTAAATTTAATTTTGGCTTCCGCAATCATTCACTCCCTCTGATTGCATATCCTAGAGTGAATCG
ATCCTATATAAACATGTATCAAAACAAAGAAATATTAGAGGGACTATAATAGATTAGCTTGAAGCAGCAATTTTTATCTTAATAT
CTACCCTAACTTACTTGGGACAAAAGGCTATGTTGTTGTTGTACCTAGATAACAAAACTATACAAATTGTCCGAGGCTTGAAA
GAGTGGATACAGATTATATAGCAGAAATGGTATTTTGCTACGGCATTACATCAAACACTTGGCCAGCATCGTCAGTCATGCGC
CTCCTTGCACATCGGGCTATAGAGAATTCCTAAGCATCCGCAAGACGGACAGCTACCTTGCCTGCCTACGGGCTCGCCGCCG
CTCTGCTCAACGTGGCGACGCCGGACCTGAGTGTGGCCGCGCCAGCACCCAGGTCAGCCGGGGCATGGCTCGCCACGCCG
CCACCTAGGCCGGTCAGAGCGTCGCCTCCAAGACCGCCCGCCGCGCAACCCCGCAAGCAGTGCAGCGTGATAGCTCCAATG
GCACCCACCACGCCGTCTACGCCATCATCTTCCATCCGCACCACCAGACCGGATTCGCCCGCCGCTCATCAAATCACGCAAA
ATTTGCTGTGCGCCGCCACCACCTGTCTCGGTGGCCACGACCGCACACCAACGGCTTCCTTTTCAGCCGCACCGATCCGGCG
GGGCCCATTTCCACTTCCATTCCCTGATGCTGCCGCCACTAGCCCACCCGCCACAACCGCAGCCGCCATGGCCCAGCCATCT
CTGTTTGTTCCCGTTCCTCACTTCAAAGCGCCGCACGCGGCACGCCCAAGAAAACAGCAGCGGCCTCCTCCTCCTCCTCAA
GGCTCAAGGTTCAAGGCAGC
>SsLRR-RLK44
ATGATTTTGGGACGAAATTGCTGATAGCCTCGTTGAGCACTAAAAGGAATGAAATTGTGTGAATAATGAGGTTAGTTGATTCC
GGTTGGTATTGCTGCCTGCCATTTGCTGCAGCAGTGTTGGCTGCTAGCACTGATCTCTTTCAGCACACAAACAAACACAATAT
GCATTCTATCTTCTAGTTTTTGCAATAGGATAAAACTTATGCATCAGGAGGACATCAACCCATGTGAGGAGGACCAGGGCAG
GGGGGGCATAGCCCAGGTTCGCCTCCACTGCCCTCCGCCAGTGGTGCGATCGTTTGAGGGAAAATGAGGTAGGAAAACACT
CCATTCGTTCTAAATTATAAGATTTTTTTTGATTTGTTAGATACATTGTTTTTACTATGTATGTAGATATAGTGTATATCCTTTTTT
TGCGTAAAGATAGAGTGTATATCTAAGTGTATAGTAGAAACTATGTATCTAGACAAATCAAAGCGTGTAAATGTAGAGACGGA
GGGGCATGGCTTAATATTGAAGATGAGGGGGTGAAGTGATAGTTCTTGATATTGGGTCAAATTCTAATTCCAAATTCTTAATCA
TCCAAACAACGGCATTTGGTGCTAGCCAATAACAACTCTATTTGGACTGTGGAGGATGAATATCTATATCCAAACAGGGTTAT
AATAACGTGGGTTTAAATATGAACTTGACAGCTGGTAAAGGTTCAATTTTTTCTATTGCCACTTTTTAAGACCAAGGCTCAGC
CGGCTACGTACTCATGCCATGTCACTTAAAGCCACAATTAGCGAACACTTATTATATAATGAACTGGCTATTTAGATTGGCTAT
AAGGAGTCTTTGAGTGTTTATTGTTTGTATGTGTAAATCATCAGCTAGGATTCAATAAGCTACTTTATGGCCATTTTAAACATA
CAAACATGCTCCTCTGTTCCATCAACACTTATAGGAATGAAAAGAAGCGGATATTTTTTGATATCCGTCTGTCCCGAAAGAGT
TAAAACCCTACTTGGATAATTTTTATATGGATCAGAACATTTAACATTTGTATCTGATTTGTACCTGAATACTTAAAATTTGATTT
CTGATACATATATTATACATATATCAATACCTGCATCCAACACTATTTGTATTTAACTTTGAATCCCGGAAAGAAACAACTAGCC
ATATTTAGTAGACGTAATATCCATTCGTATTCGATCCAGTTTTCATCCCTAAACATGTACCAAAACAAAGAAATATTAGAGGGA
CTATAATAGATTAGCTTGAAGCAGCAATTTTTATCTTAATACCTAGATAACAAAACTATACAAATTGTCCGAGGCTTGAAAGAG

TGGATACAGATTATATAGCAGAAATGGTATTTTGCTACGGCATTACATCAAACACTTGGCCAGCATCATCAGTCATGCGCCTCC
TTGCACATCGGGCTATAGAGAATTCCTAAGCATCCGCGAGACGGACAGCTACCTTGCCTGCCTACGGGCTCGCCGCCGCTCT
GCTCAACGTAGCGACGCCGGACCTGAGTGTGGCCGCGCCAGCACCCAGGTCAGCCGGGGCATGGCTCGCCACGCCGCCAC
CTAGGCCGGTCAGAGCGTCGCCTCCAAGACCGCCCGCCGCGCAACCCCGCAAGCAGTGCAGCGTGATAGCTCCAATGGCAC
CCACCACGCCGTCTACATCATCTTCCATCCGCACCACCAGACCGGATTTGCCCGCCGCTCATCAAATCACGCAAAATTTGCTG
TGCGCCGCCACCTGTCTCGGTGGCCACGACCGCACACCAACGGCTTCCTTTTCAGCCGCACCGATCCGGCGGGGCCCATTT
CCACTTCCATTCCCTGATGCTGCCGCCACTAGCCCACCGGCCACAACCGCAGCCGCCATGGCCCAGCCATCTCTGTCTGTTC
CCGTTCCTCCCTTCAAAGCGCCGCACGCGGCACGCCCAAGAAAACAGCAGCGGCCTCCTCCTCCTCAAGGCTCAAGGTTCA
AGGCAGC
>SsLRR-RLK38
TTTTATTTTATAATTTTTATGAGTGACGAGTGGTTGGGCAGCCTCGGTAGCACCTTGGTGTCCCAGGTTCGAACCCCCACAGG
GCCAATCGCAGGGTGGAGGTAAAAAAAACCCTCTTCTGTCCCACGTGTCCAAAGCACACATGTAAGGACGGGCTCAGTTCA
TATGAGTGATGGTGCTGCATTGTAAGGACAAGGCTAGAGGTTCGAGACTTTTCTCGGTCTACGTGAAAGGCTTTTCTACTTTA
AACACCATGCTCGGGAGCCGGCTTCCTCCTGTAGGCCGAGTCTTTTTATAAGGCCTTGTTTAGCTAGGGAGAAGAAAATTTT
TGGATGTCACATCGGATGTGTCGGAAGGATGTCGGGAGGGGTTTTCGGATACTAATAAAAAAACAAATTACAGAACCTGTCA
GAAAATTGCGAGACGAATCTAATAATATTAATTAATCTGTCATTAGCACATGATGGTTATTGTAGCACTTAAGGCTTTTCATGG
ACTAATTTGGCTTAAAAGATTCGTCTCGCGATTTTCCCAAGAACTGTGCAATTAATTTTTTTTTGTCTATATTTAGTACTCCATG
CATGTATCAAACATGCCCTTTTACTATAGAAGGCAAAAAAAAAAATTTGAAAACTAAACAGGCGAGCGAGGTGTATAGAAATG
AGACAAACCTTTTCACCAAACGAGCTAGGTTATGCAACACCCCACACACCGGATGCTCCCTCTCTTTCACGGGAGGAAGAA
TCTCTCGCTTTCTCGCTCCCAACTGGGTATGTCATTTTCGGTCAGCATGCCATACACGTACATGCGCCCCTGATTAATCAATCG
ATCCATTTAGCTACGAGAAAGAAAGGTGTGGGGAAGGAAAAAGGCAGAGAAAGCGGTGCGAGGAGCAGAAAAACGGGGG
TGATTAGCTGTTCGTCCCGTGTCCCGTTCCTCCTCGCCCACCACCGCACGCGGACAAAACCGCCCTCCTCACGGCCTCGCCA
GTCGCCACACTCCCAGCCCCCTTCGCTCGCGCACACGGCACACCACCAGTCCAATTCCCAGTACACTGCTCTACGCCTAATC
TCTCCAGCCCTTTCGCCCCCTTGCCTTGCCACGCCGCCCACCGCTGCCGCATCATCCGCCCGTTGCCGGCCGACGTCGCCTG
GCCCGCGGCTTCCGCCCCCATTCGCCGCCGAGCCCCGTCCCAGATCGCCGCGGCGCTTCCTGCCAGCGTCGGCGCGGCGCA
GGTATCCCGTCCTCGCGCCGGATCTGCCTCCGTTTCTGCTGATTTGCGCTGCCTTTGGTGTGTGGCCGTGGTAAATGCGAACG
CGTTAGGCTGCCGCCGCCCCCCAAATTGCTGCGCTTAATTCGTGCGTCGGTGGCGTCGCCTGTAGATGAGCCGTGCTTGCAG
ACTGCAGTGGCTTTGACTCTGGATGCATGCTCGTGTCTGGTAGTGCCTACTGTTAGCTGGGGTGTTTTTGCCTGCTTCTTTGG
AAGTTACTAATACTAAAGGAGAGGAGGCATTGGATTGCTGGGAGCTGGAACTAAGTGGCCGTGGATTTAAGCCGGGTAGTG
ACAAGCTCATTAGCCCTCCACGTGTTTCTCTTCGAGTTCGAACTAGAGGCTTTGTGGTTCTCCCCAGTTGTTATCCCCTTTGA
AGGTTTGTGTCTCTGAAGCAATTATCCAGAGGCAAATTAGGAAAGTTTGCATCACATAGAAGAAAGAAAACATTTCTAATTT
CCCACTGTATGACCGTCGATGTGTGCTGCATTTGATCTGCTGTGTGTTTTTTGTGATCATATCATGATTTATTTGCTGAAGCGCT
TTTGGTTATGCTTTTGCCTTGTTAACTTTATTTGTATGACTGTTCCTGCTCCAGGGTTGGAAATCTATTTGACCACTCAGTGGG
TGGCTTCATGTTAAATCCATTTCTGGTTTGAAGATGCTTCATCAATGACATCTGGAAAGTGTAATAGTATGTGCTGATCTTCTA
TGCTGCACCAGCTGCTTATC
