# Supplementary material for: Genome-Wide Identification of LRR-RLK Family in Saccharum and Expression Analysis in Response to Biotic and Abiotic Stress
Source: Curr Issues Mol Biol. 2021 Oct 18;43(3):1632–51. doi: 10.3390/cimb43030116 (PMC8929030; doi:10.3390/cimb43030116)
Supplement: Supplementary file 1 [file cimb-43-00116-s001.zip › Supplementary Material Table S5.docx]

**Table S4.** Statistics of *A. thaliana* and *S. spontaneum* LRR-RLK gene distribution among different subfamilies. For both *A. thaliana* and *S. spontaneum*, the number of *LRR-RLKs* genes belonging to each subgroup were counted respectively. The corresponding percentage in all *LRR-RLKs* genes from specific species was computed and showed in brackets.

| **Subfamily** | **Number (Percentage) of Genes in Each Subfamily** |
| --- | --- |
|  | ***A. thaliana* *S. spontaneum*** |
| I-a  I-b  II  III  IV  V  VI-a  VI-b  VII-a  VII-b  VII-c  VIII  IX  X-a  X-b  X-c  XI-a  XI-b  XII  XIII-a  XIII-b  XIV  XV | 48 (21.33%) 12 (2.75%)  2 (0.89%) 0 (-)  14 (6.22%) 13 (2.97%)  46 (20.44%) 75 (17.16%)  3 (1.33%) 10 (2.29%)  9 (4.00%) 15 (3.43%)  5 (2.22%) 8 (1.83%)  8 (3.56%) 8 (1.83%)  5 (2.22%) 14 (3.20%)  3 (1.33%) 5 (1.14%)  2 (0.89%) 6 (1.37%)  8 (3.56%) 6 (1.37%)  9 (4.00%) 0 (-)  4 (1.78%) 5 (1.14%)  9 (4.00%) 59 (13.50%)  1 (0.44%) 3 (0.69%)  33 (14.67%) 124 (28.38%)  2 (0.89%) 1 (0.23%)  8 (3.56%) 51 (11.67%)  4 (1.78%) 1 (0.23%)  3 (1.33%) 2 (0.46%)  2 (0.89%) 6 (1.37%)  2 (0.89%) 13 (2.97%) |
