# Supplementary figures and images for: Genome-Wide Identification of LRR-RLK Family in Saccharum and Expression Analysis in Response to Biotic and Abiotic Stress
Source: Curr Issues Mol Biol. 2021 Oct 18;43(3):1632–51. doi: 10.3390/cimb43030116 (PMC8929030; doi:10.3390/cimb43030116)

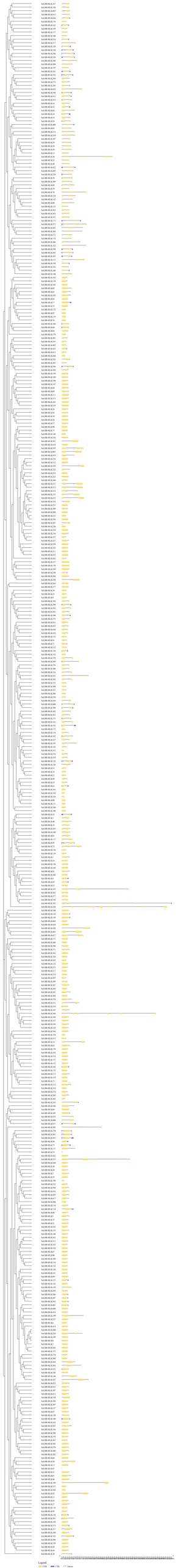

Supplement: Supplementary file 1 [file cimb-43-00116-s001.zip › Supplementary Material Figure S1.pdf]

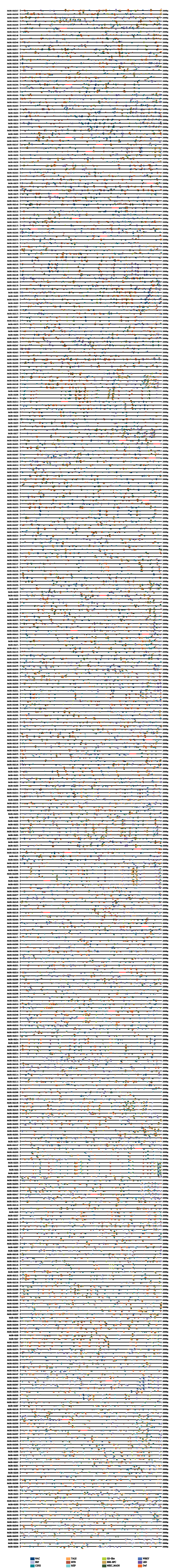

Supplement: Supplementary file 1 [file cimb-43-00116-s001.zip › Supplementary Material Figure S3.pdf]
