# Supplementary material for: Genome-Wide Identification of LRR-RLK Family in Saccharum and Expression Analysis in Response to Biotic and Abiotic Stress
Source: Curr Issues Mol Biol. 2021 Oct 18;43(3):1632–51. doi: 10.3390/cimb43030116 (PMC8929030; doi:10.3390/cimb43030116)
Supplement: Supplementary file 1 [file Supplementary Material Figure S2.pdf › Supplementary Material Figure S2.pdf]

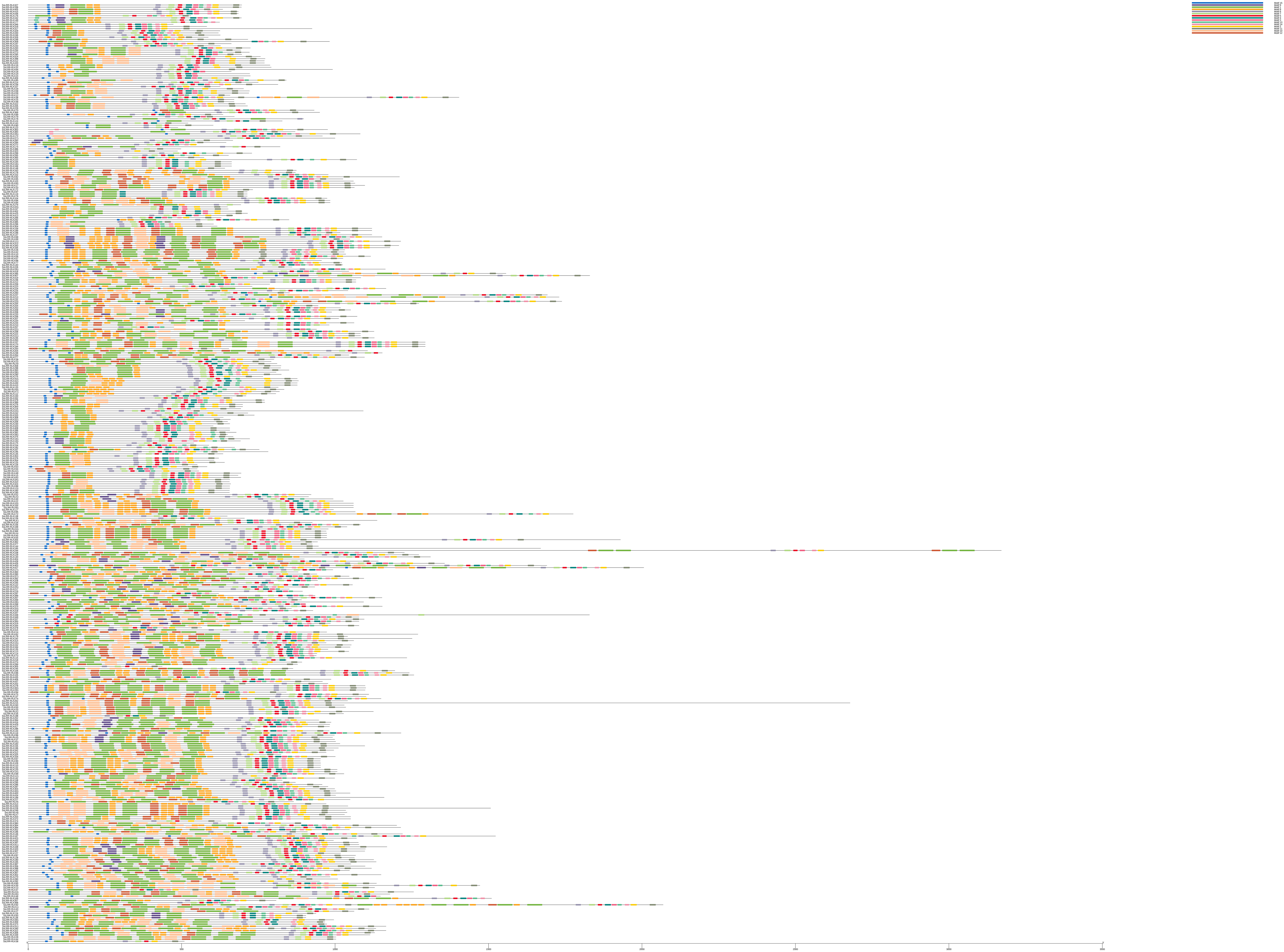

Motif 15
Motif 9
Motif 1
Motif 8
Motif 6
Motif 7
Motif 5
Motif 4
Motif 3
Motif 13
Motif 14
Motif 2
Motif 11
Motif 10
Motif 12
0
500
1000
1500
2000
2500
3000
3500
